# Supplementary material for: Social media usage patterns during natural hazards
Source: PLoS One. 2019 Feb 13;14(2):e0210484. doi: 10.1371/journal.pone.0210484 (PMC6374021; doi:10.1371/journal.pone.0210484)
Supplement: S1 Fig — (PDF) [file pone.0210484.s001.pdf]

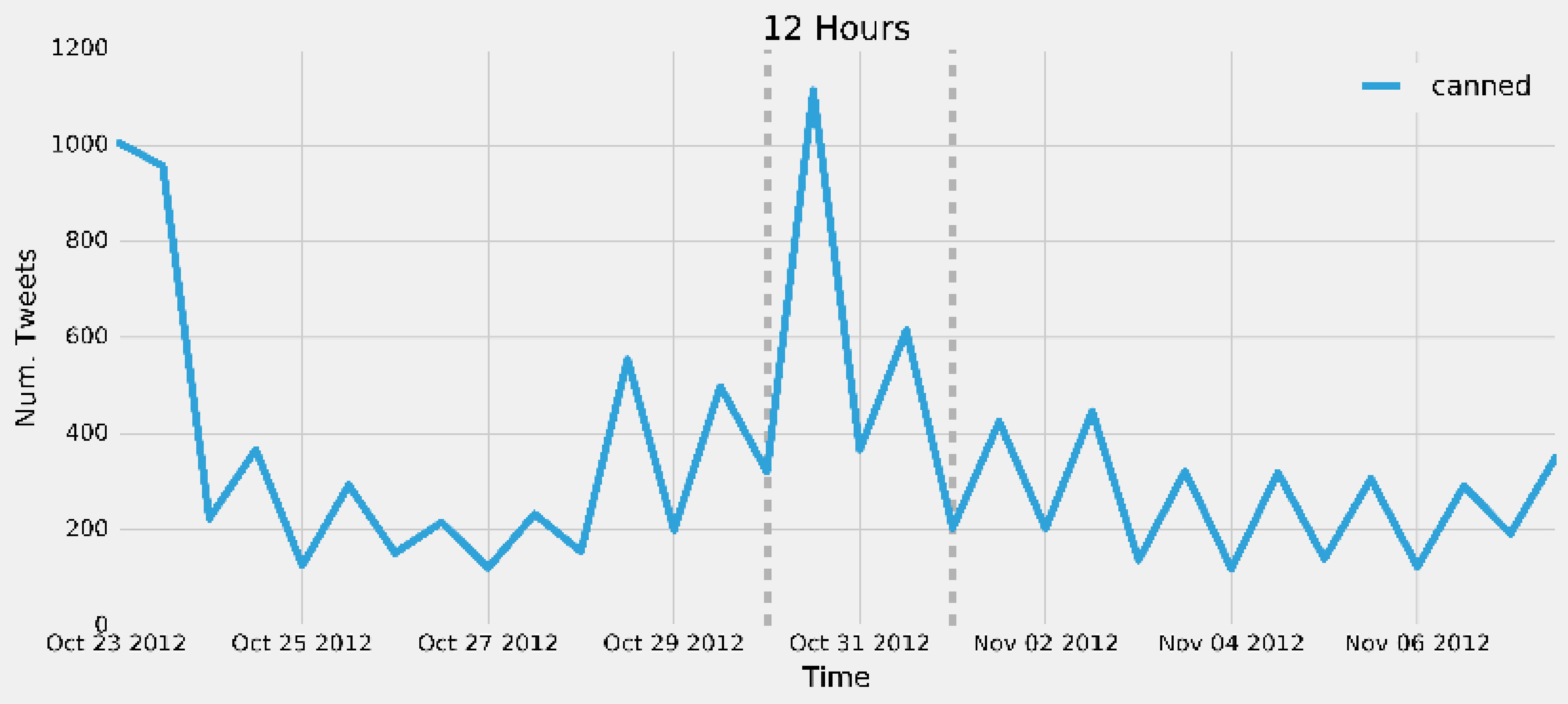
12 Hours
canned
Num. Tweets
0
200
400
600
800
1000
1200
Oct 23 2012
Oct 25 2012
Oct 27 2012
Oct 29 2012
Oct 31 2012
Nov 02 2012
Nov 04 2012
Nov 06 2012
Time

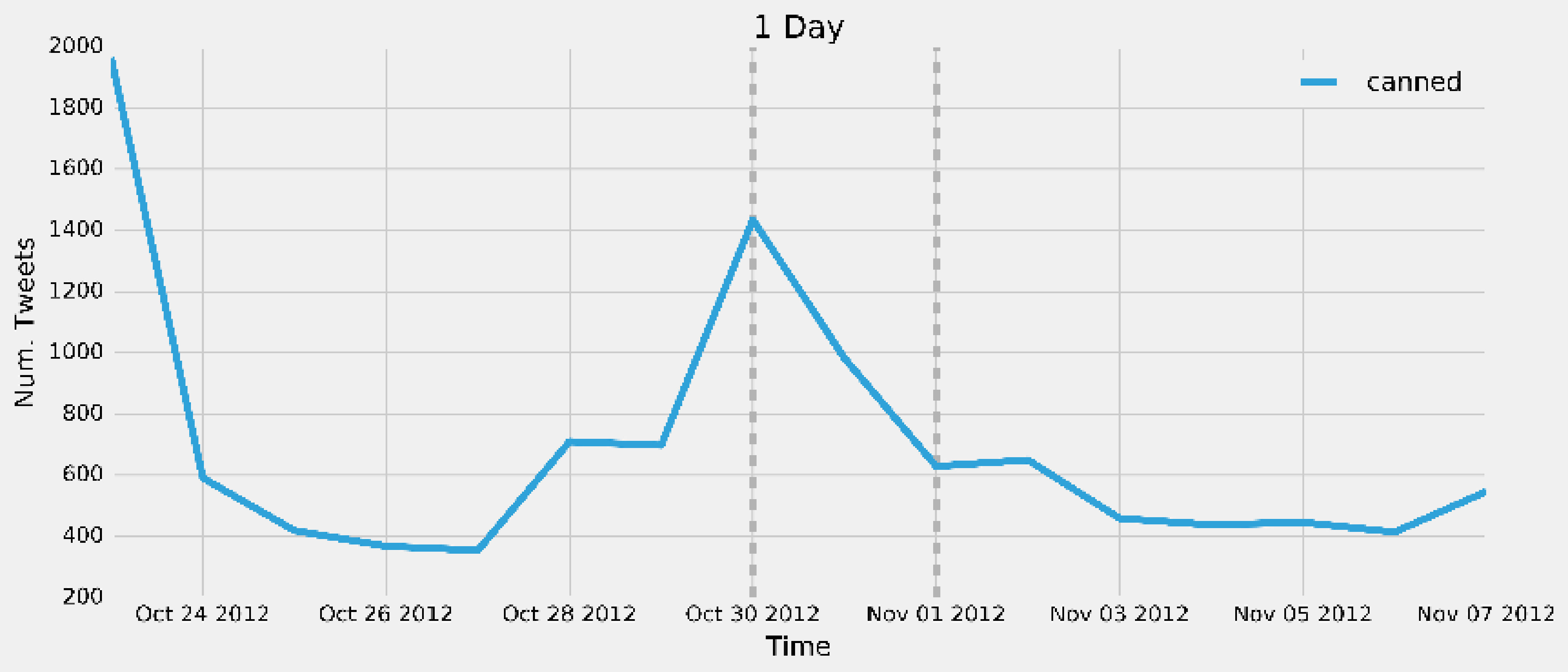
1 Day
canned
Num. Tweets
2000
1800
1600
1400
1200
1000
800
600
400
200
Oct 24 2012
Oct 26 2012
Oct 28 2012
Oct 30 2012
Nov 01 2012
Nov 03 2012
Nov 05 2012
Nov 07 2012
Time

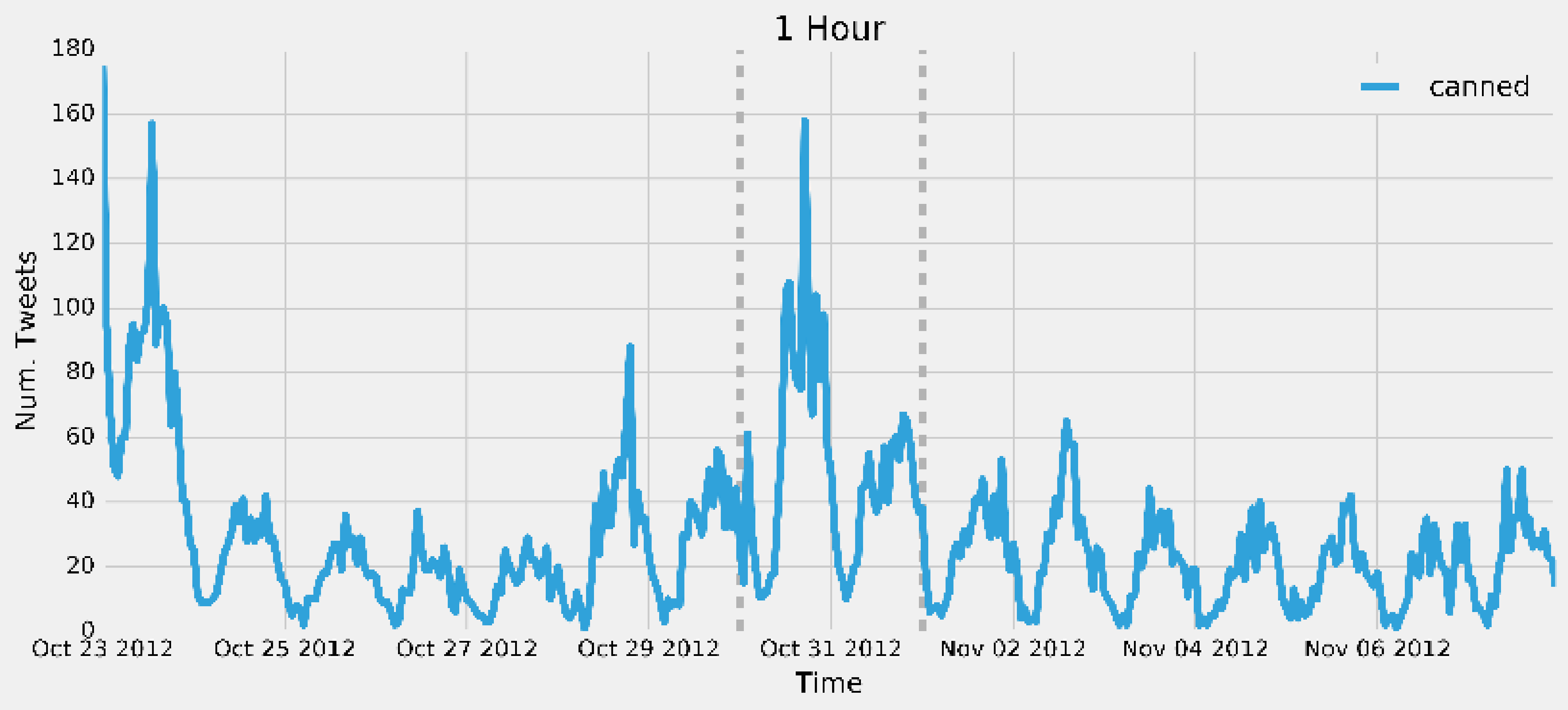
1 Hour
canned
Num. Tweets
180
160
140
120
100
80
60
40
20
0
Oct 23 2012
Oct 25 2012
Oct 27 2012
Oct 29 2012
Oct 31 2012
Nov 02 2012
Nov 04 2012
Nov 06 2012
Time

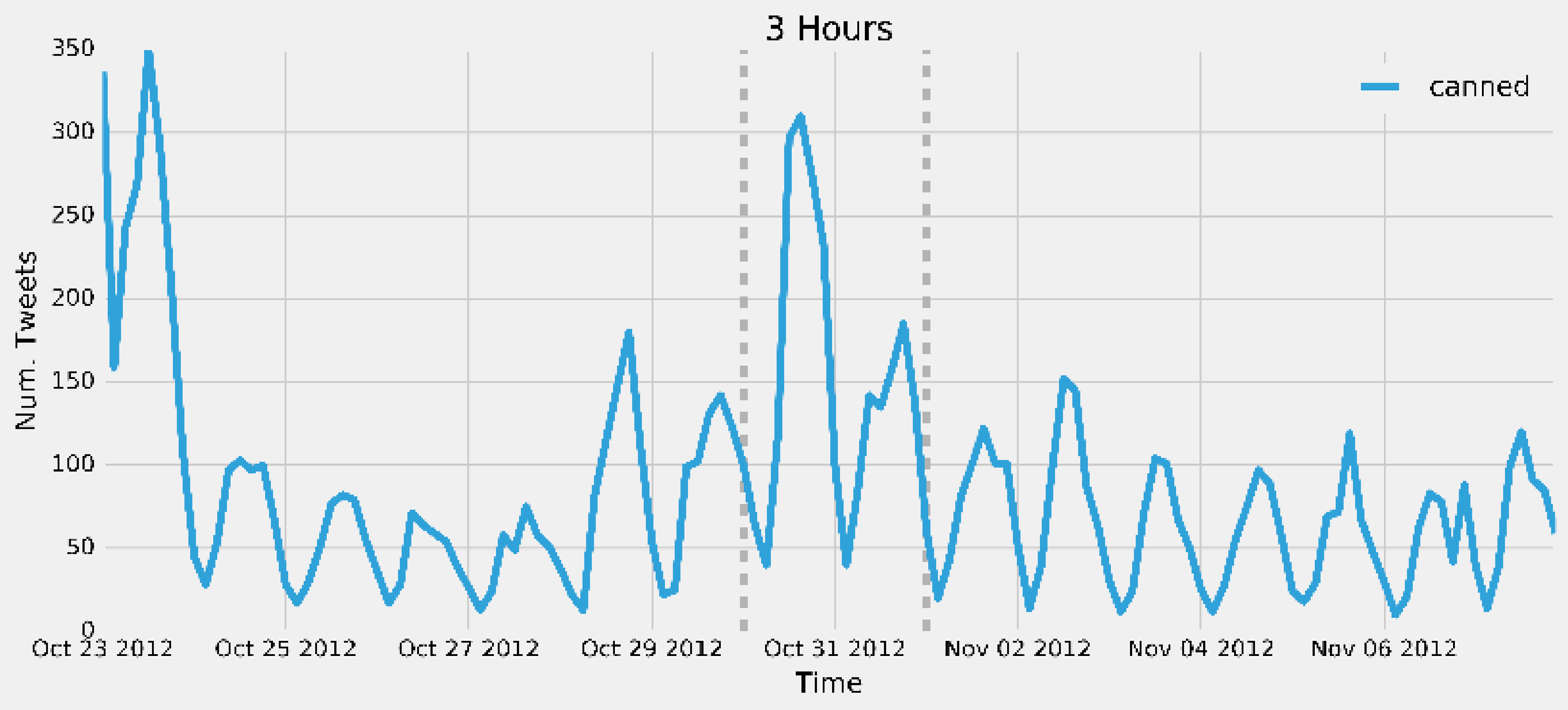

3 Hours
canned
Num. Tweets
0
50
100
150
200
250
300
350
Oct 23 2012
Oct 25 2012
Oct 27 2012
Oct 29 2012
Oct 31 2012
Nov 02 2012
Nov 04 2012
Nov 06 2012
Time

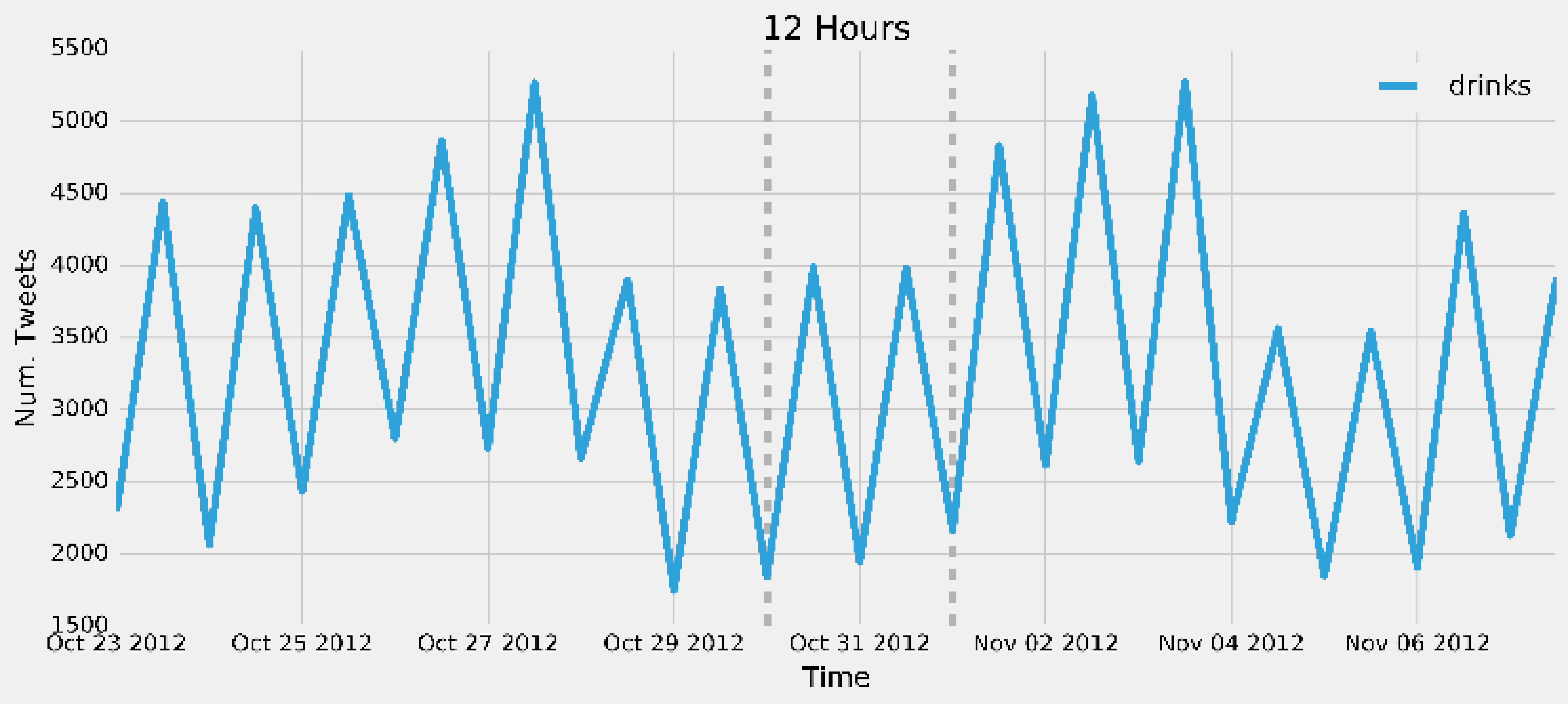

12 Hours
drinks
5500
5000
4500
4000
3500
3000
2500
2000
1500
Num. Tweets
Oct 23 2012
Oct 25 2012
Oct 27 2012
Oct 29 2012
Oct 31 2012
Nov 02 2012
Nov 04 2012
Nov 06 2012
Time

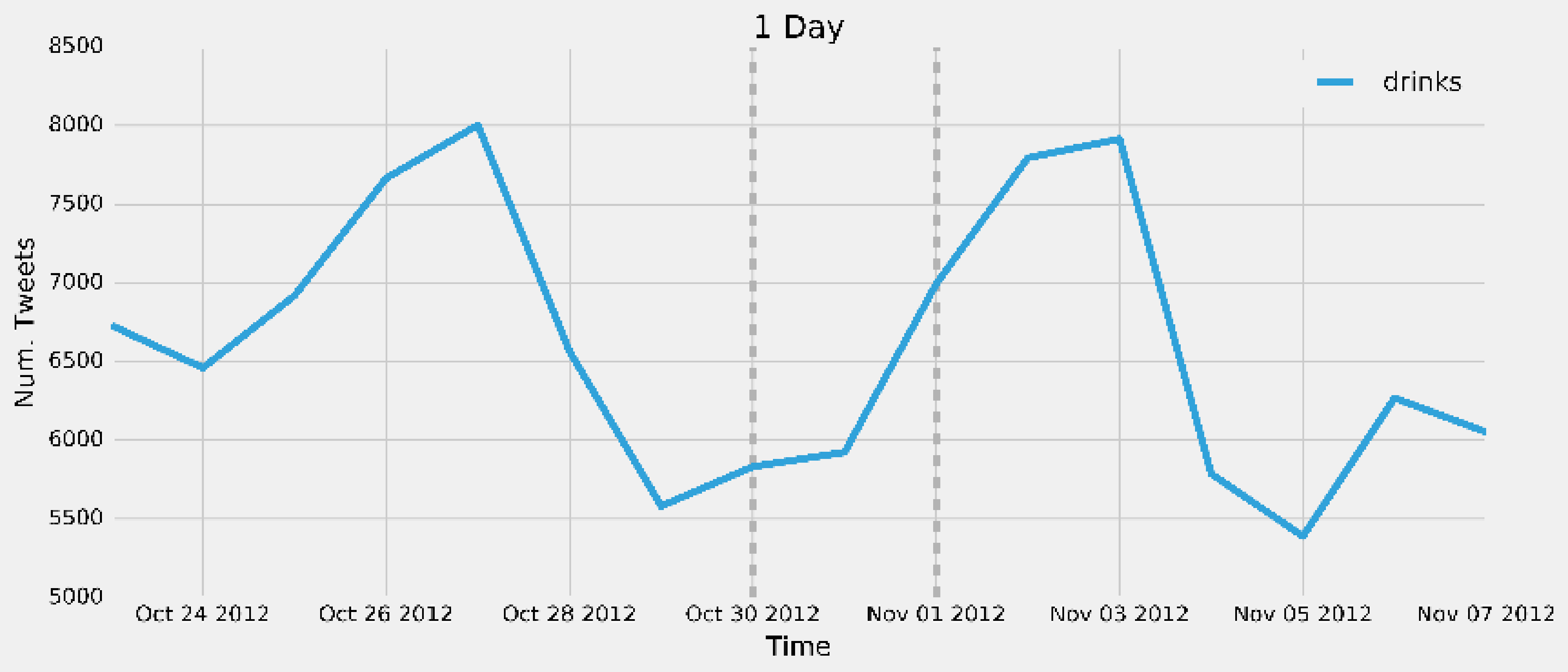
1 Day
drinks
Num. Tweets
8500
8000
7500
7000
6500
6000
5500
5000
Oct 24 2012
Oct 26 2012
Oct 28 2012
Oct 30 2012
Nov 01 2012
Nov 03 2012
Nov 05 2012
Nov 07 2012
Time

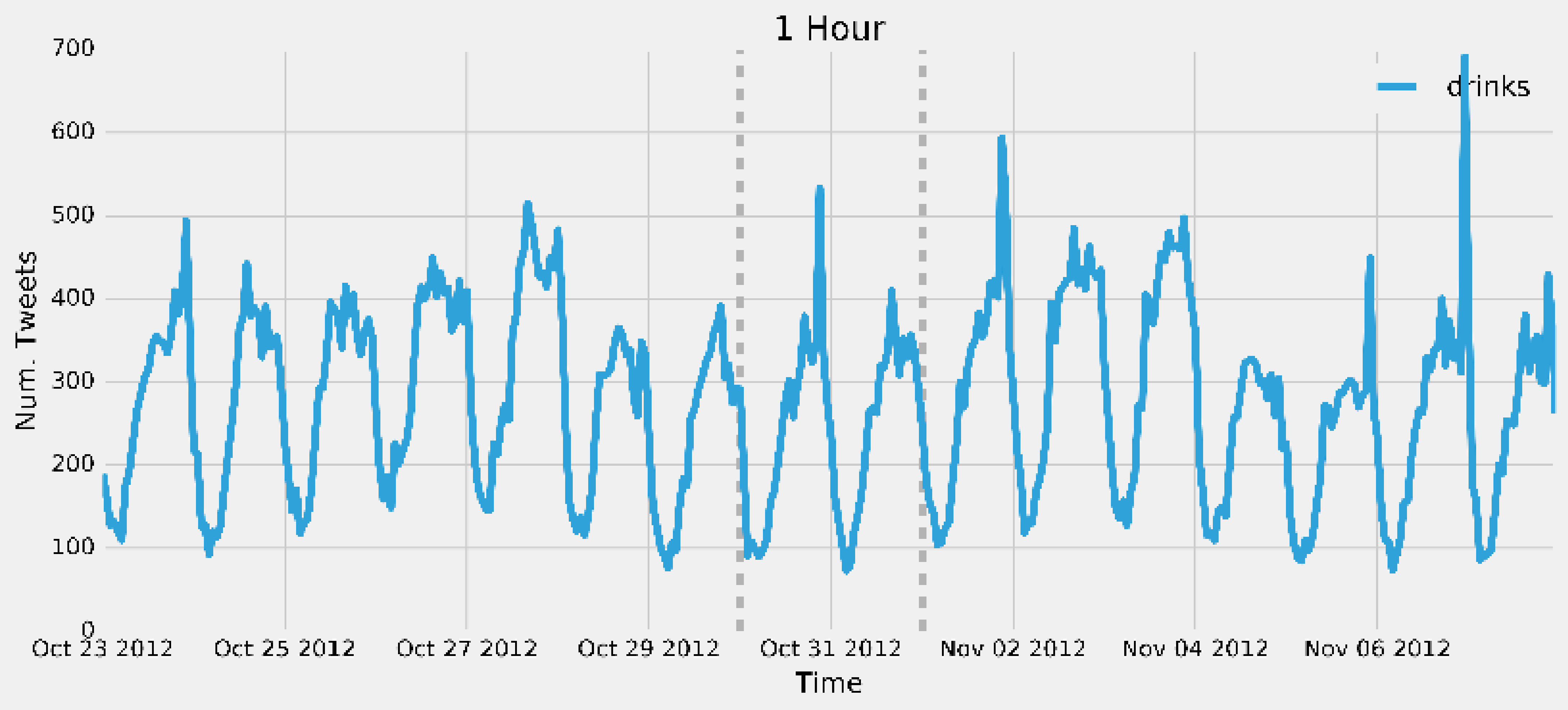
1 Hour
drinks
Num. Tweets
700
600
500
400
300
200
100
0
Oct 23 2012
Oct 25 2012
Oct 27 2012
Oct 29 2012
Oct 31 2012
Nov 02 2012
Nov 04 2012
Nov 06 2012
Time

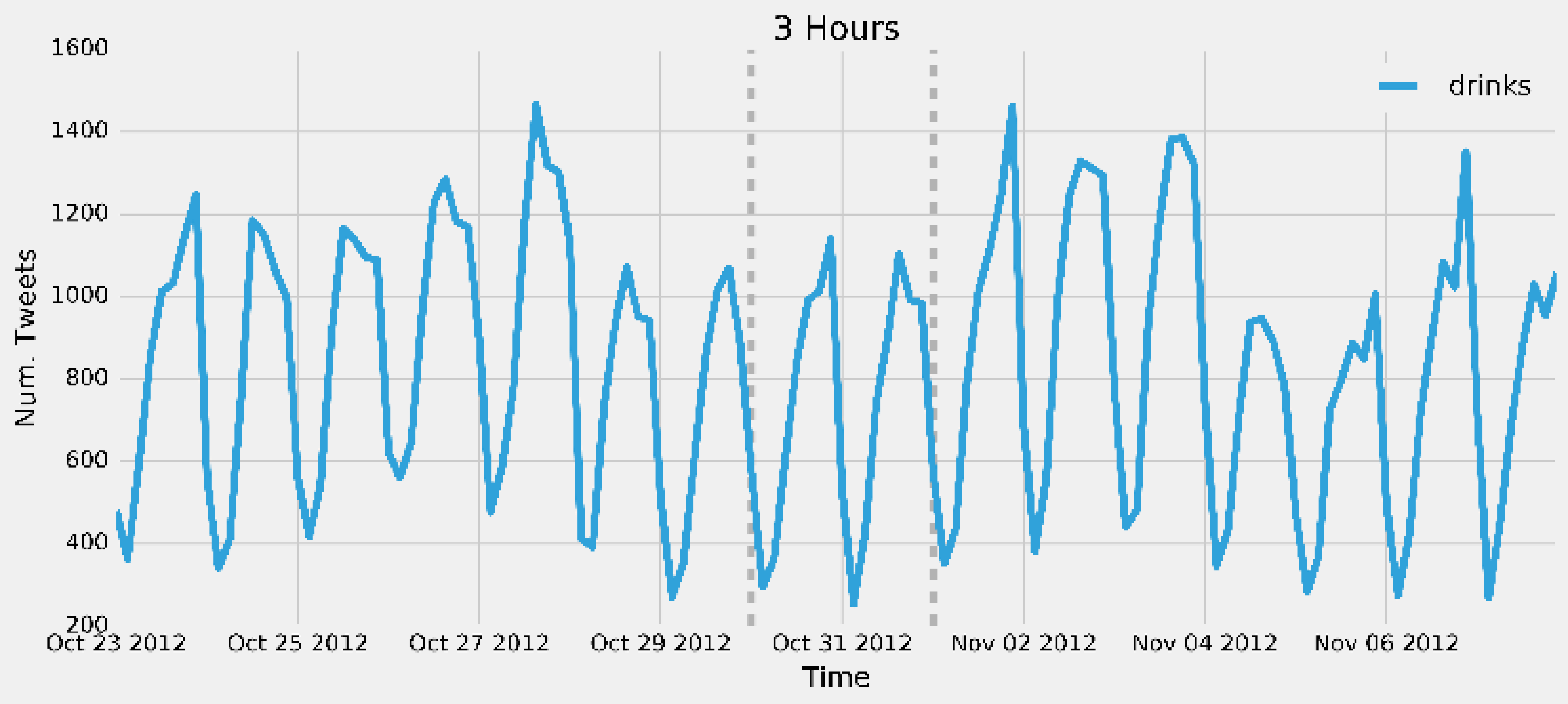
3 Hours
drinks
Num. Tweets
Time
1600
1400
1200
1000
800
600
400
200
Oct 23 2012
Oct 25 2012
Oct 27 2012
Oct 29 2012
Oct 31 2012
Nov 02 2012
Nov 04 2012
Nov 06 2012

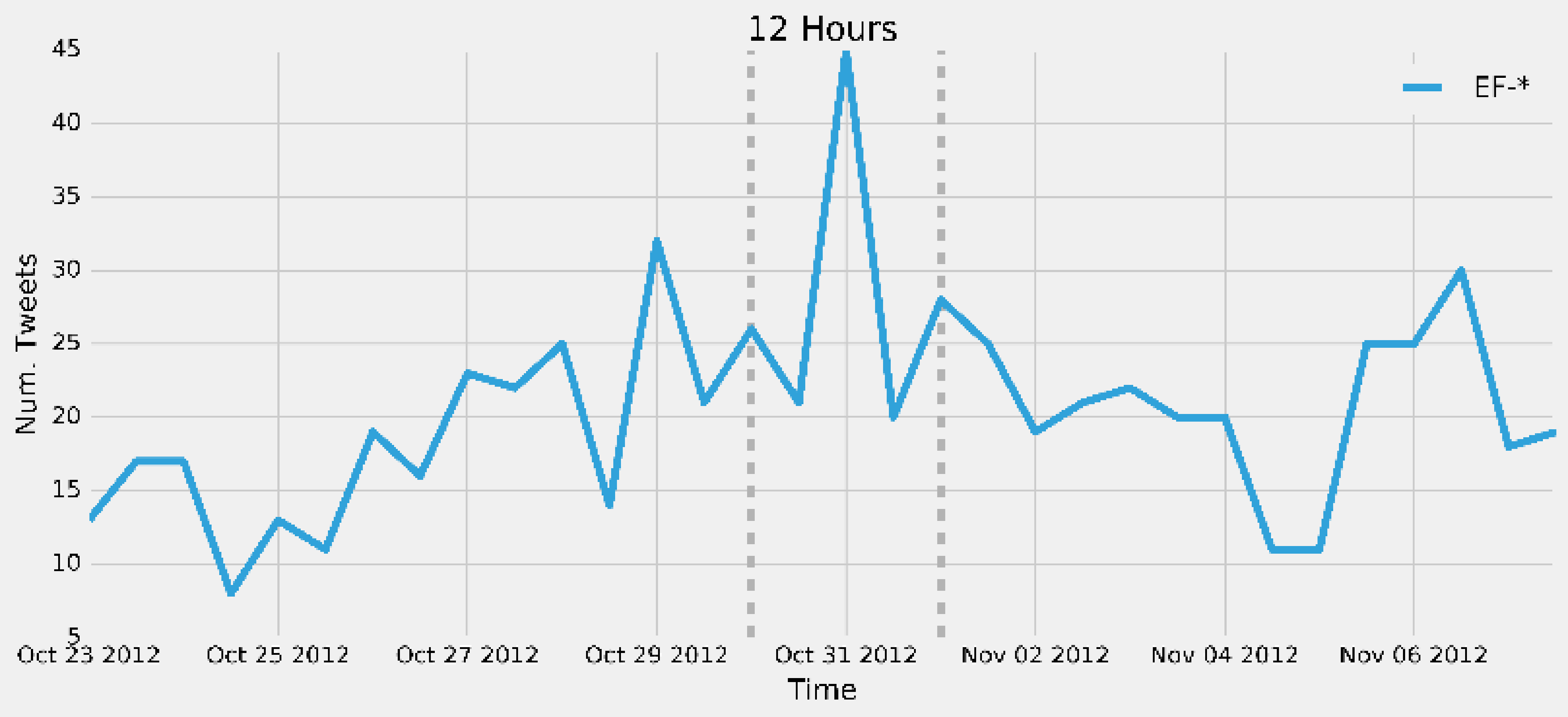

12 Hours
EF-*
Num. Tweets
Time
45
40
35
30
25
20
15
10
5
Oct 23 2012
Oct 25 2012
Oct 27 2012
Oct 29 2012
Oct 31 2012
Nov 02 2012
Nov 04 2012
Nov 06 2012

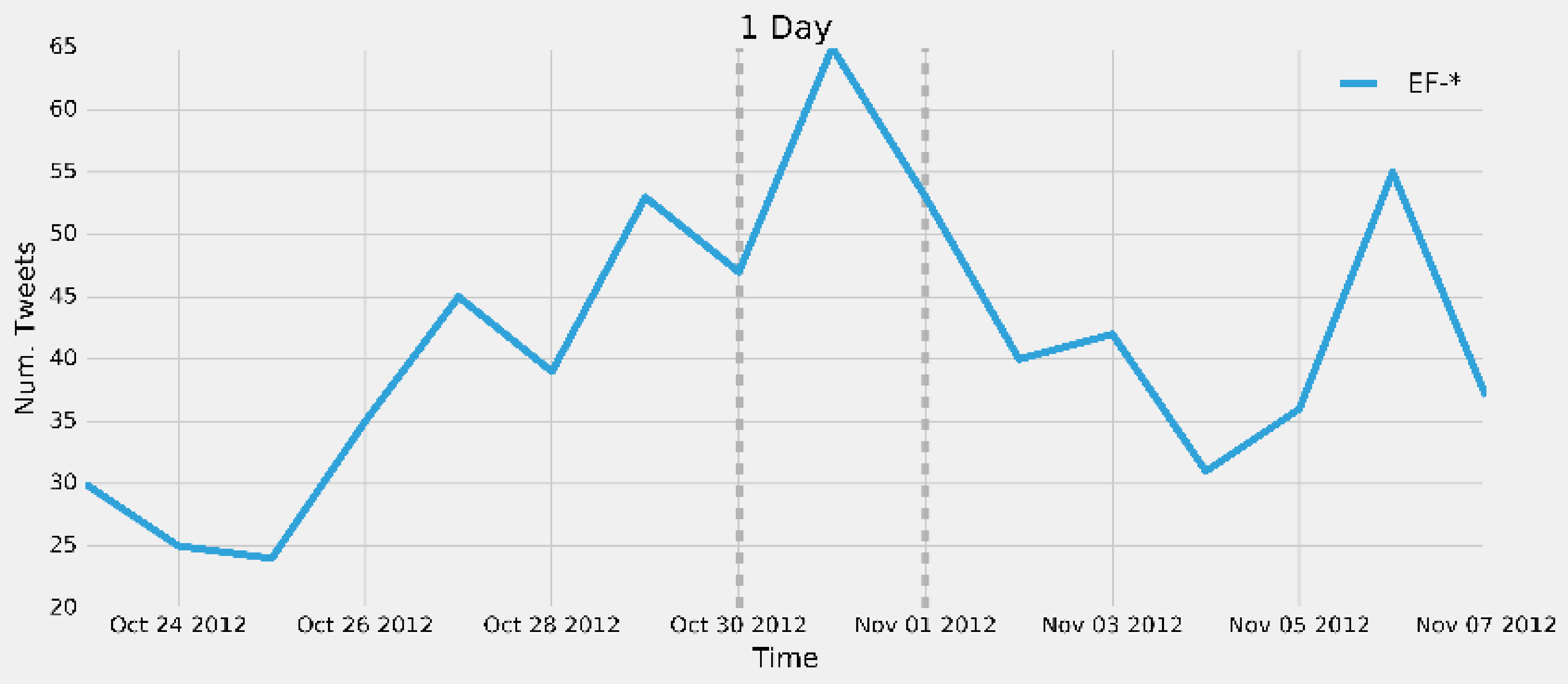

1 Day
EF-*
Num. Tweets
Time
65
60
55
50
45
40
35
30
25
20
Oct 24 2012
Oct 26 2012
Oct 28 2012
Oct 30 2012
Nov 01 2012
Nov 03 2012
Nov 05 2012
Nov 07 2012

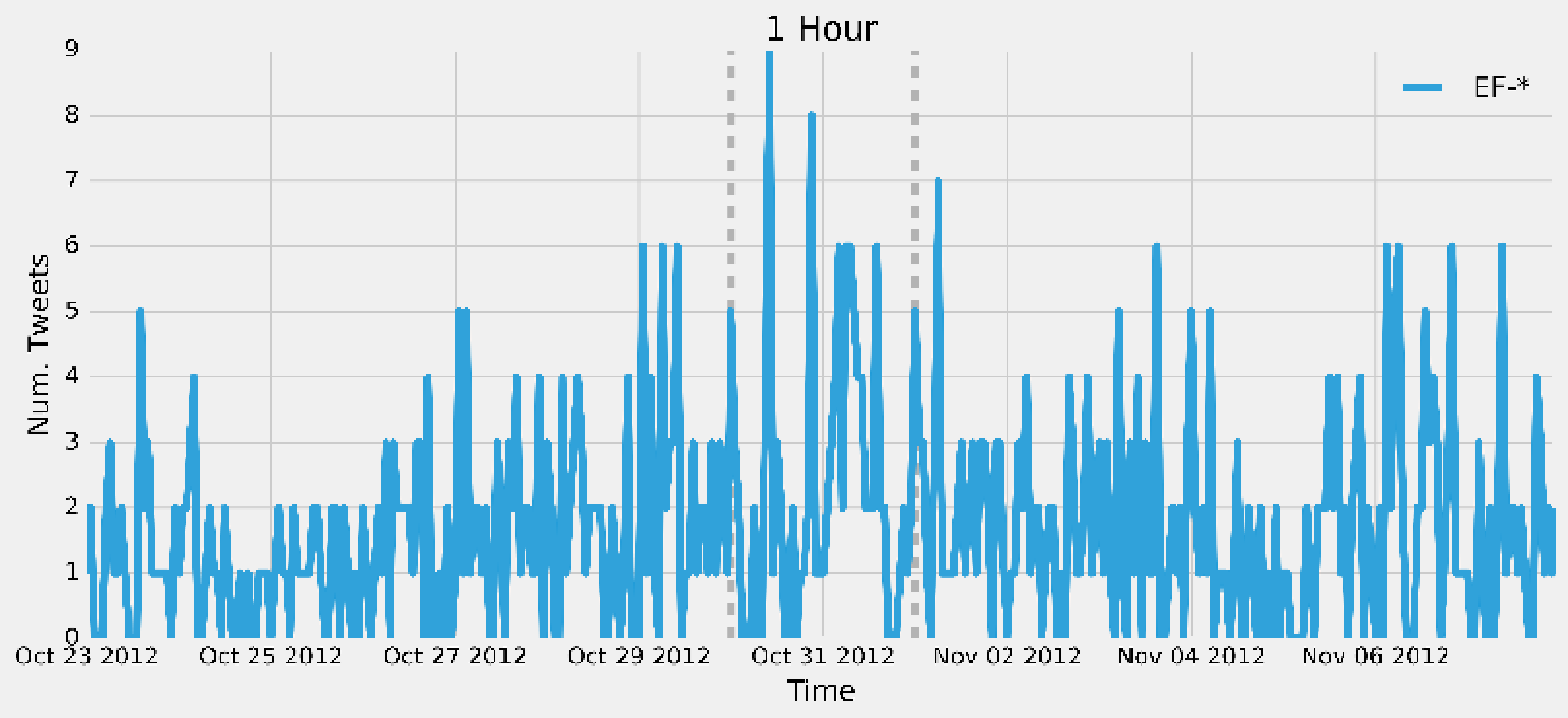

1 Hour
EF-*
Num. Tweets
Time
0
1
2
3
4
5
6
7
8
9
Oct 23 2012
Oct 25 2012
Oct 27 2012
Oct 29 2012
Oct 31 2012
Nov 02 2012
Nov 04 2012
Nov 06 2012

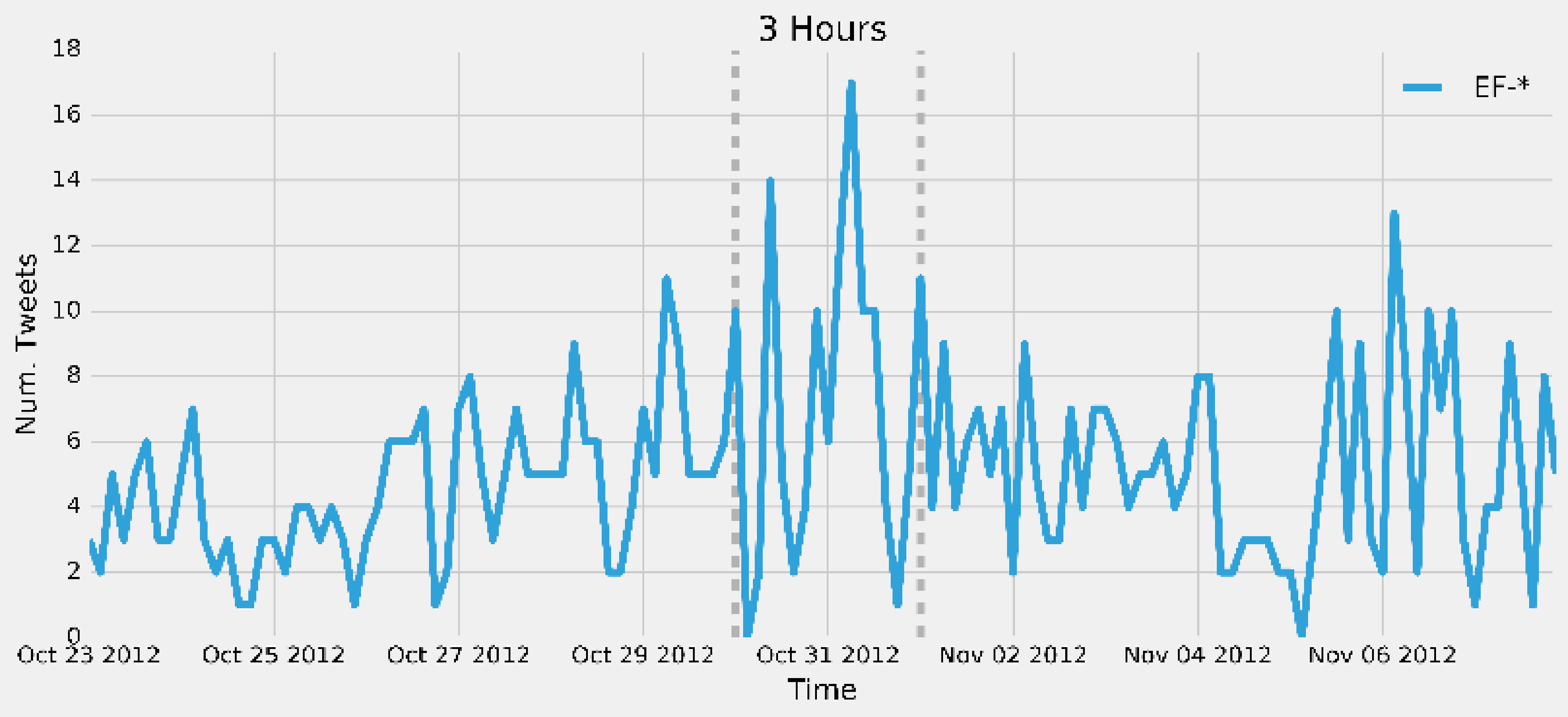
3 Hours
EF-*
Num. Tweets
Time
0
2
4
6
8
10
12
14
16
18
Oct 23 2012
Oct 25 2012
Oct 27 2012
Oct 29 2012
Oct 31 2012
Nov 02 2012
Nov 04 2012
Nov 06 2012

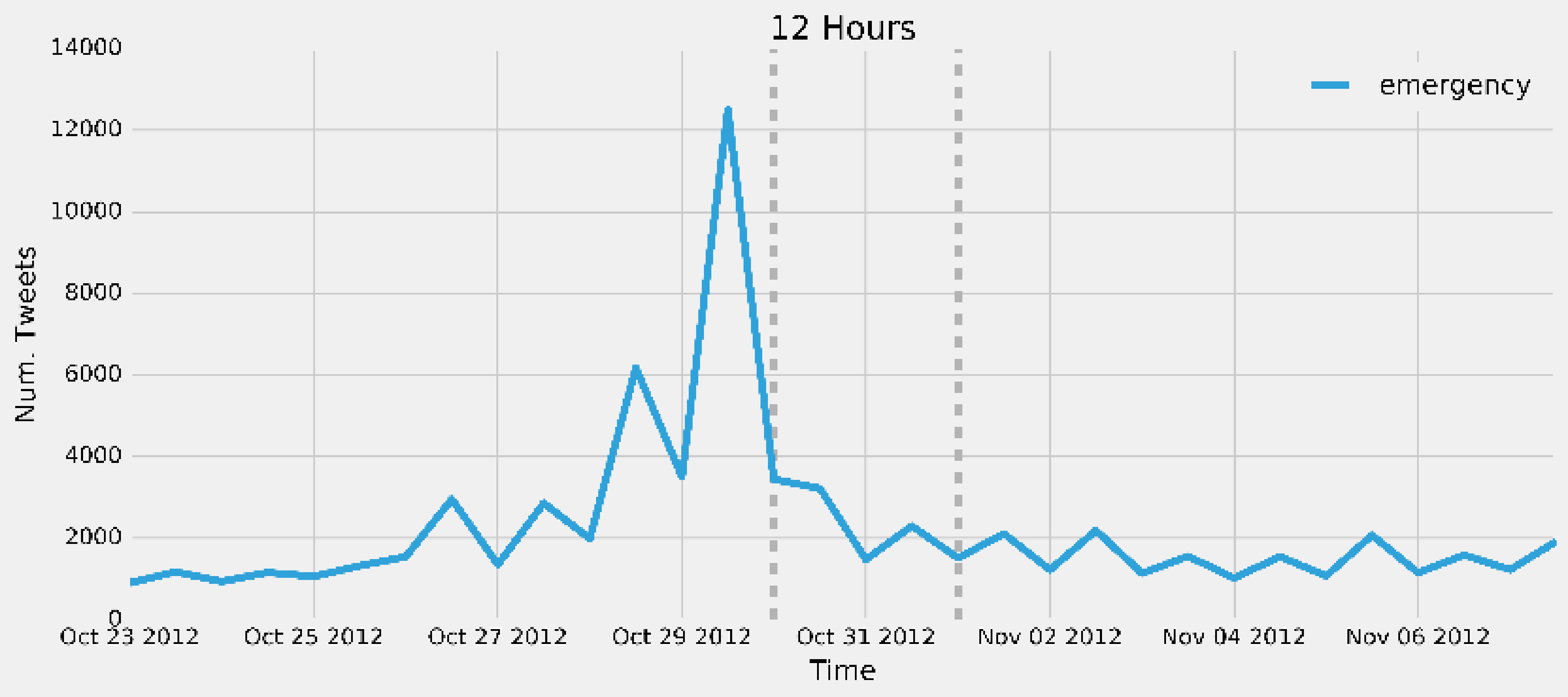
12 Hours
emergency
Num. Tweets
0
2000
4000
6000
8000
10000
12000
14000
Oct 23 2012
Oct 25 2012
Oct 27 2012
Oct 29 2012
Oct 31 2012
Nov 02 2012
Nov 04 2012
Nov 06 2012
Time

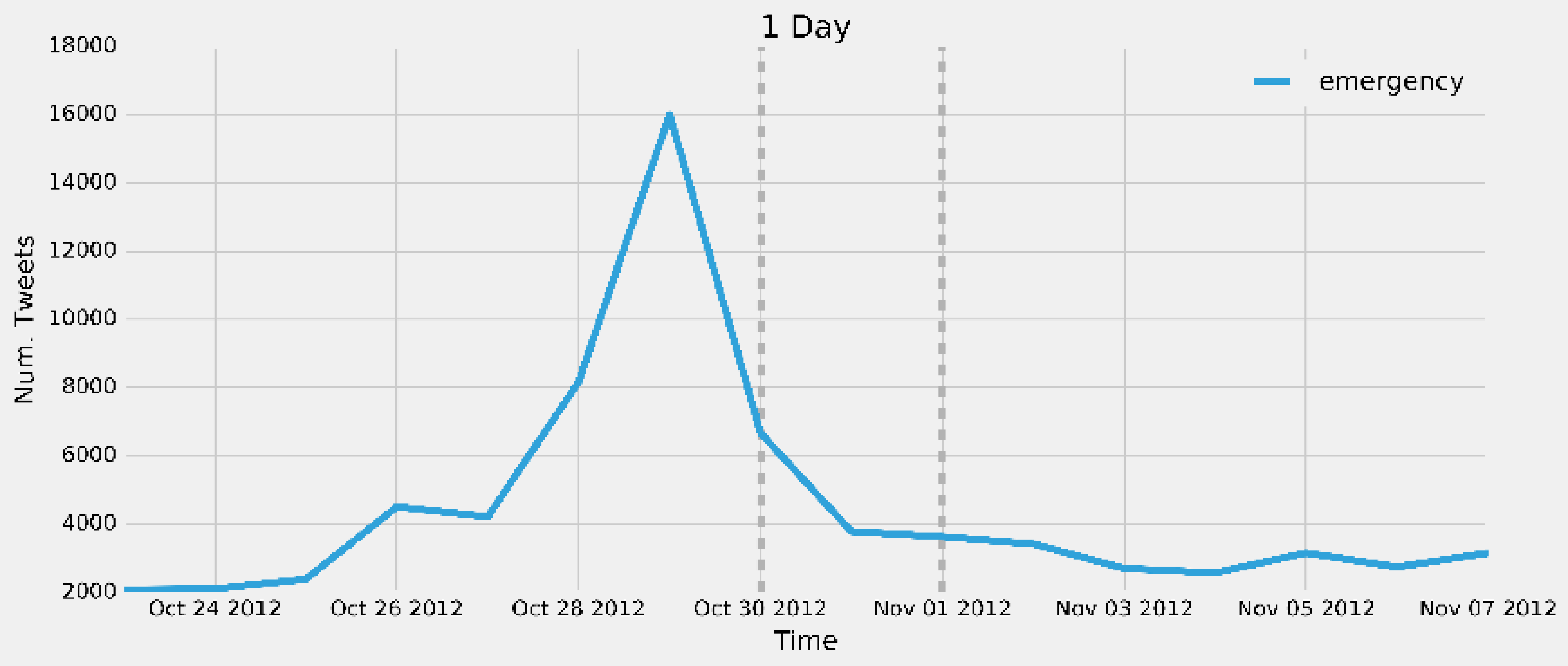

1 Day
emergency
18000
16000
14000
12000
10000
8000
6000
4000
2000
Num. Tweets
Oct 24 2012
Oct 26 2012
Oct 28 2012
Oct 30 2012
Nov 01 2012
Nov 03 2012
Nov 05 2012
Nov 07 2012
Time

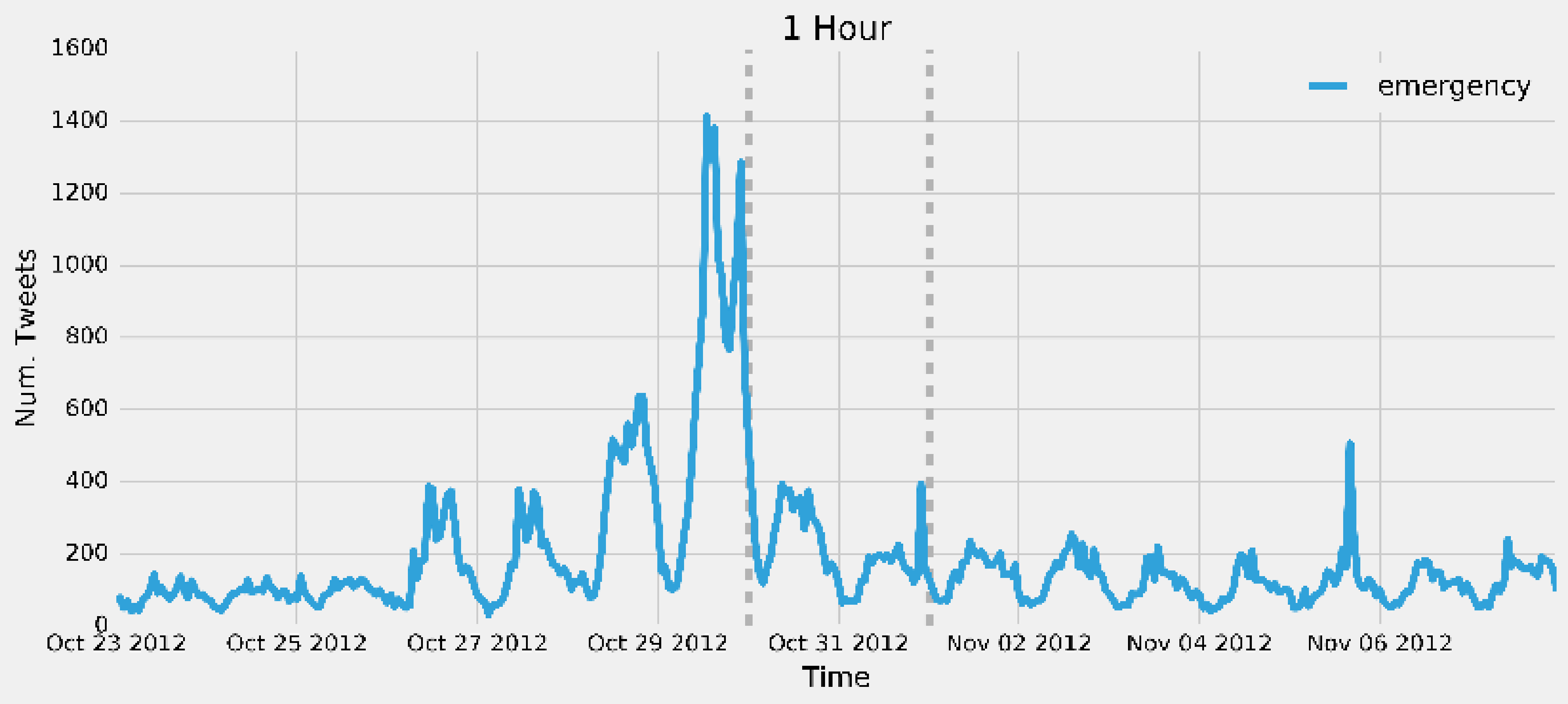

1 Hour
emergency
Num. Tweets
Time
1600
1400
1200
1000
800
600
400
200
0
Oct 23 2012
Oct 25 2012
Oct 27 2012
Oct 29 2012
Oct 31 2012
Nov 02 2012
Nov 04 2012
Nov 06 2012

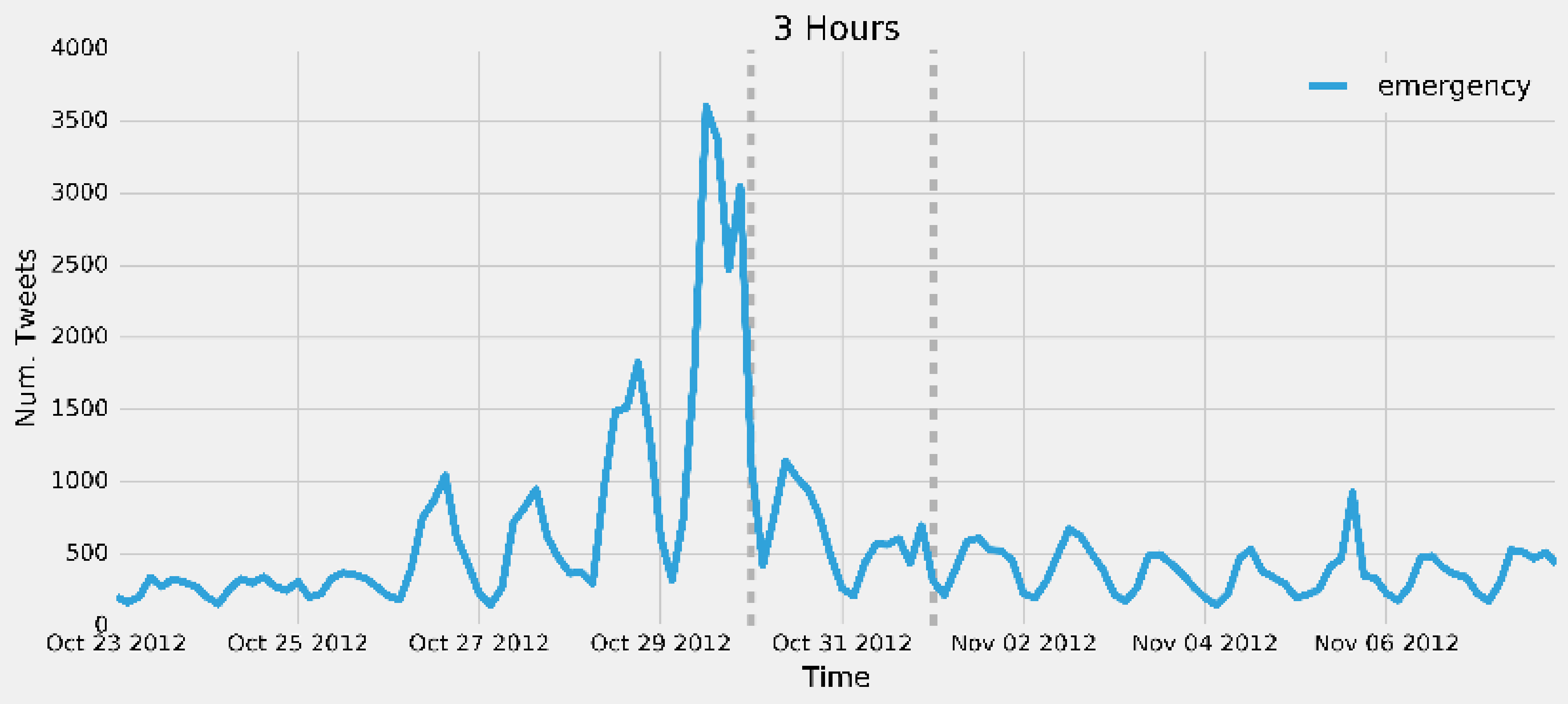
3 Hours
emergency
Num. Tweets
0
500
1000
1500
2000
2500
3000
3500
4000
Oct 23 2012
Oct 25 2012
Oct 27 2012
Oct 29 2012
Oct 31 2012
Nov 02 2012
Nov 04 2012
Nov 06 2012
Time

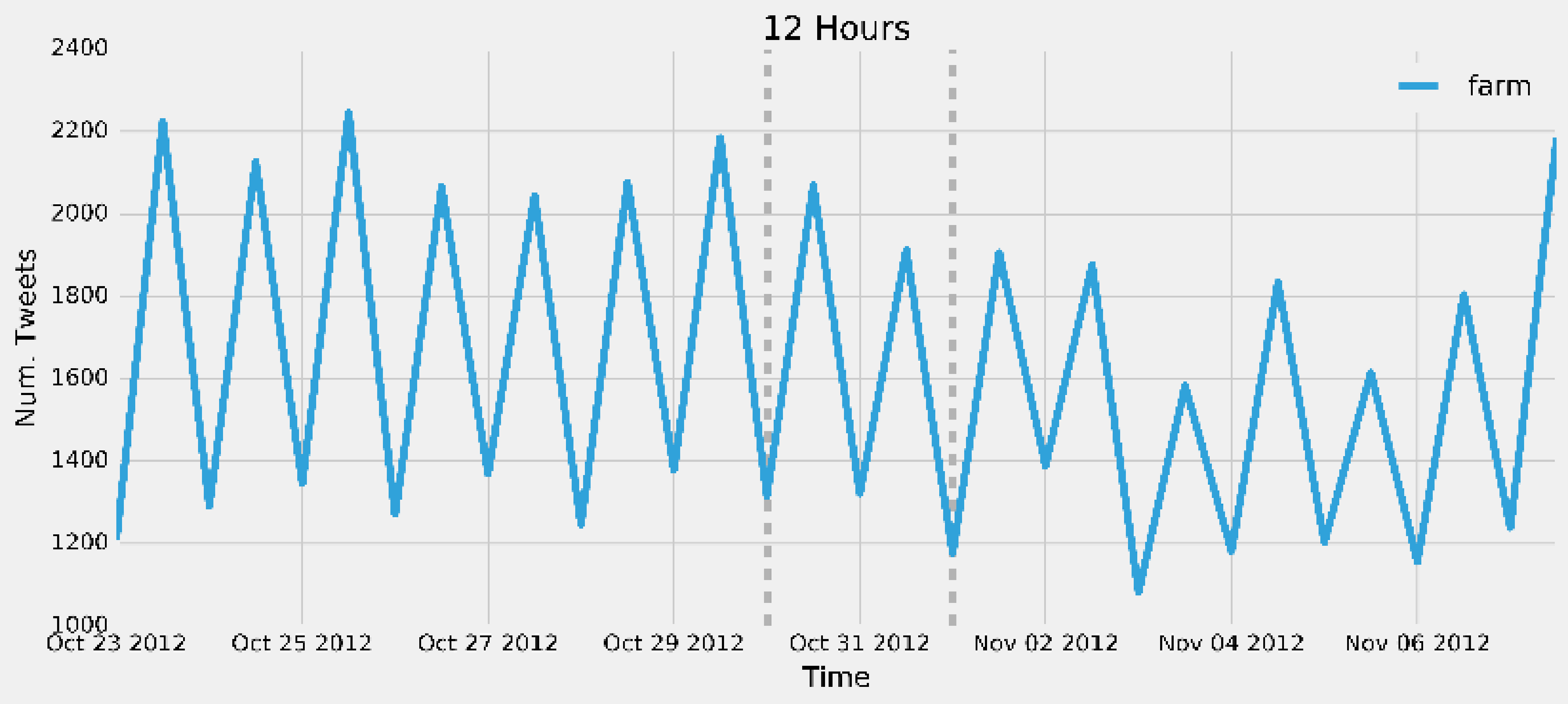

12 Hours
farm
Num. Tweets
Time
2400
2200
2000
1800
1600
1400
1200
1000
Oct 23 2012
Oct 25 2012
Oct 27 2012
Oct 29 2012
Oct 31 2012
Nov 02 2012
Nov 04 2012
Nov 06 2012

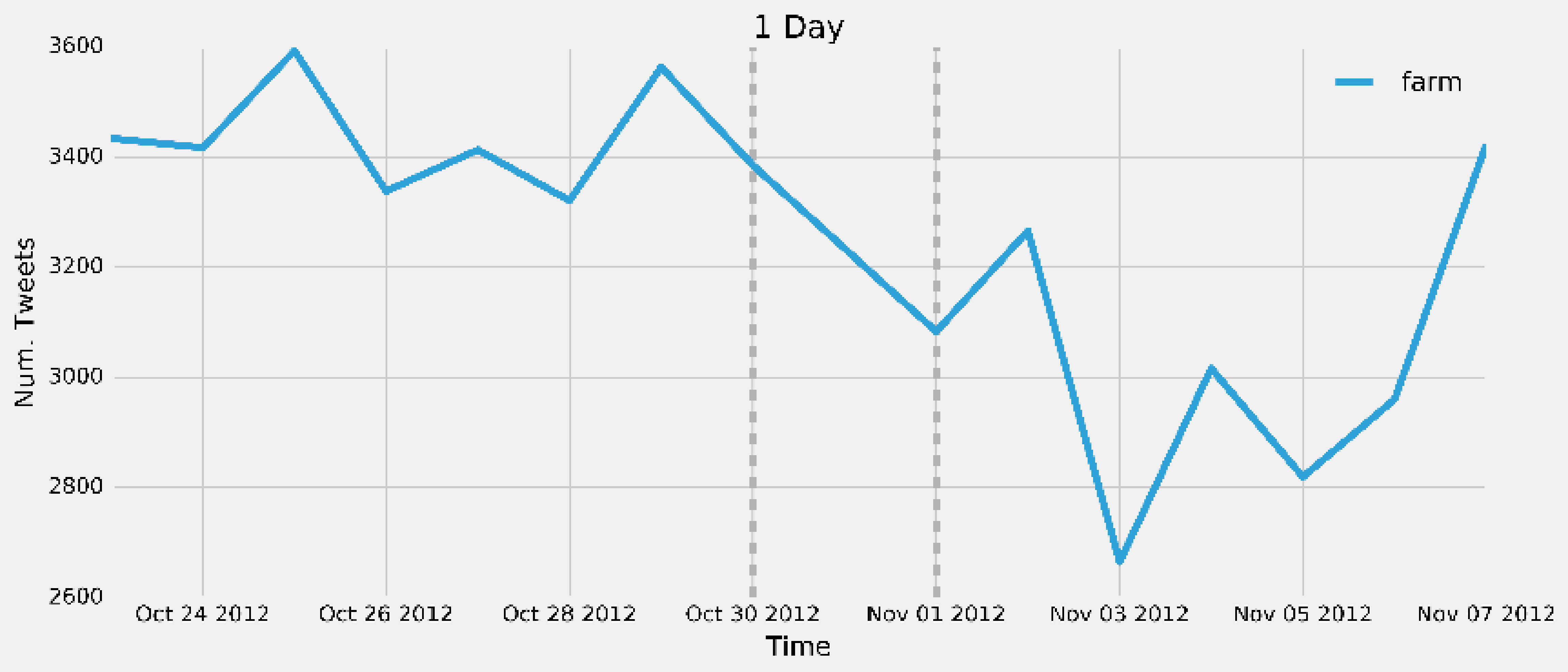
1 Day
farm
Num. Tweets
Time
3600
3400
3200
3000
2800
2600
Oct 24 2012
Oct 26 2012
Oct 28 2012
Oct 30 2012
Nov 01 2012
Nov 03 2012
Nov 05 2012
Nov 07 2012

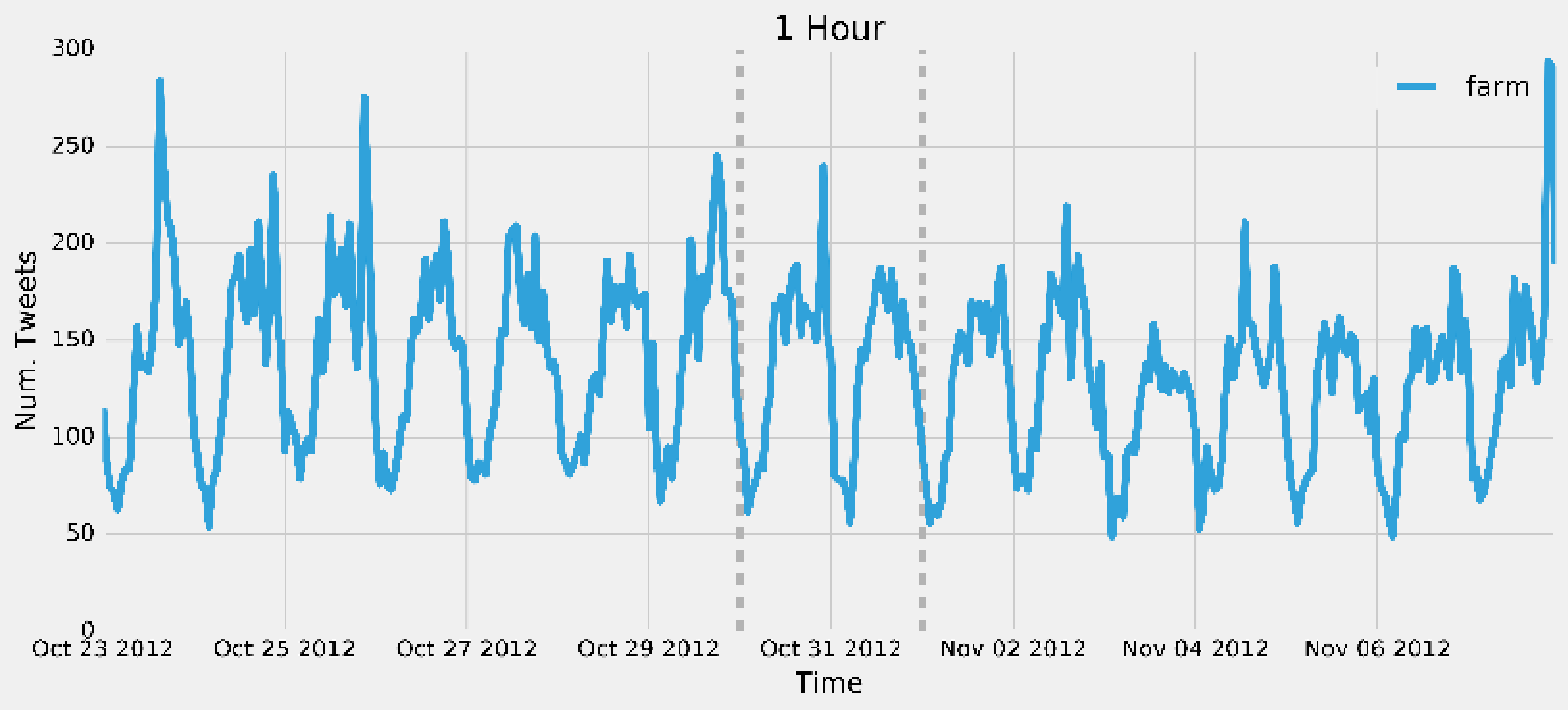
1 Hour
farm
300
250
200
150
100
50
0
Num. Tweets
Oct 23 2012
Oct 25 2012
Oct 27 2012
Oct 29 2012
Oct 31 2012
Nov 02 2012
Nov 04 2012
Nov 06 2012
Time

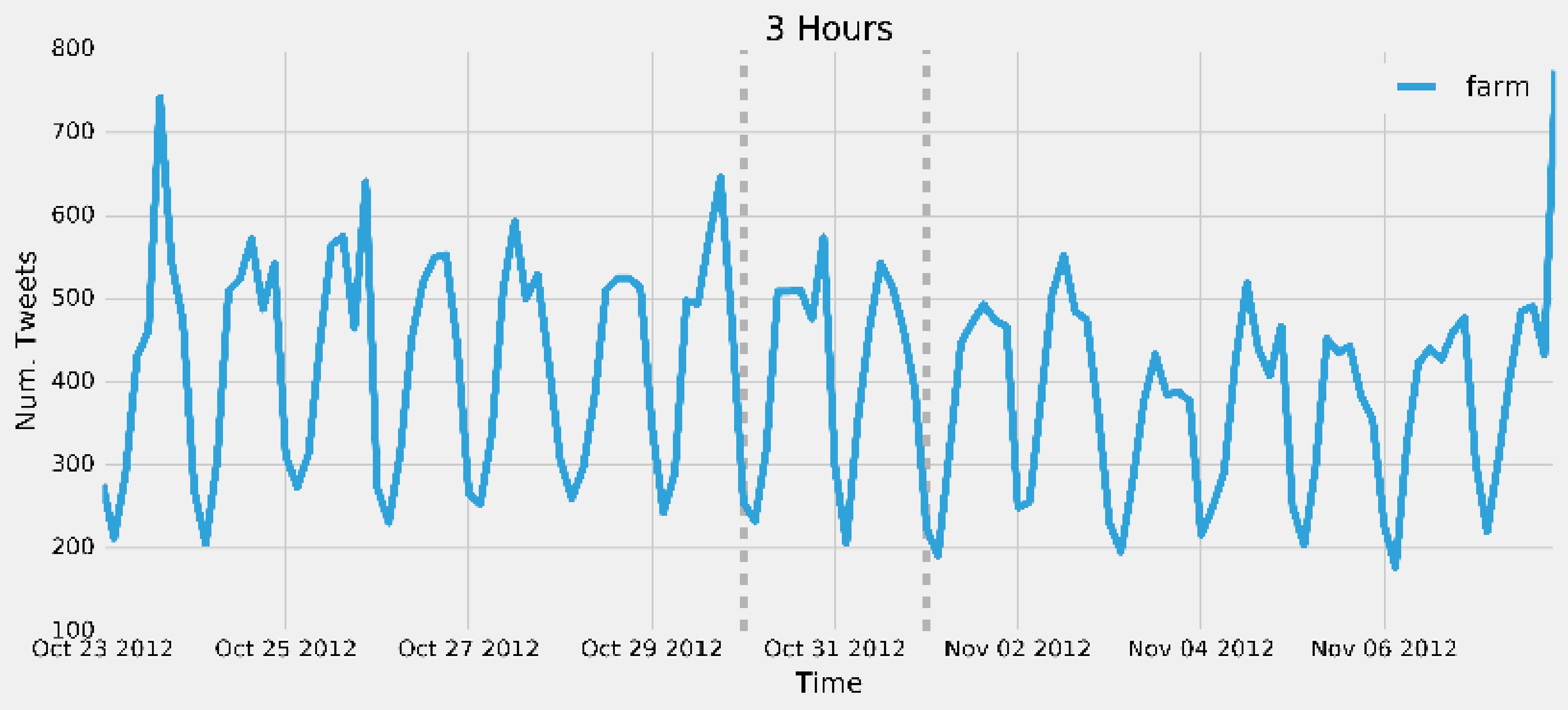
3 Hours
farm
800
700
600
500
400
300
200
100
Num. Tweets
Oct 23 2012
Oct 25 2012
Oct 27 2012
Oct 29 2012
Oct 31 2012
Nov 02 2012
Nov 04 2012
Nov 06 2012
Time

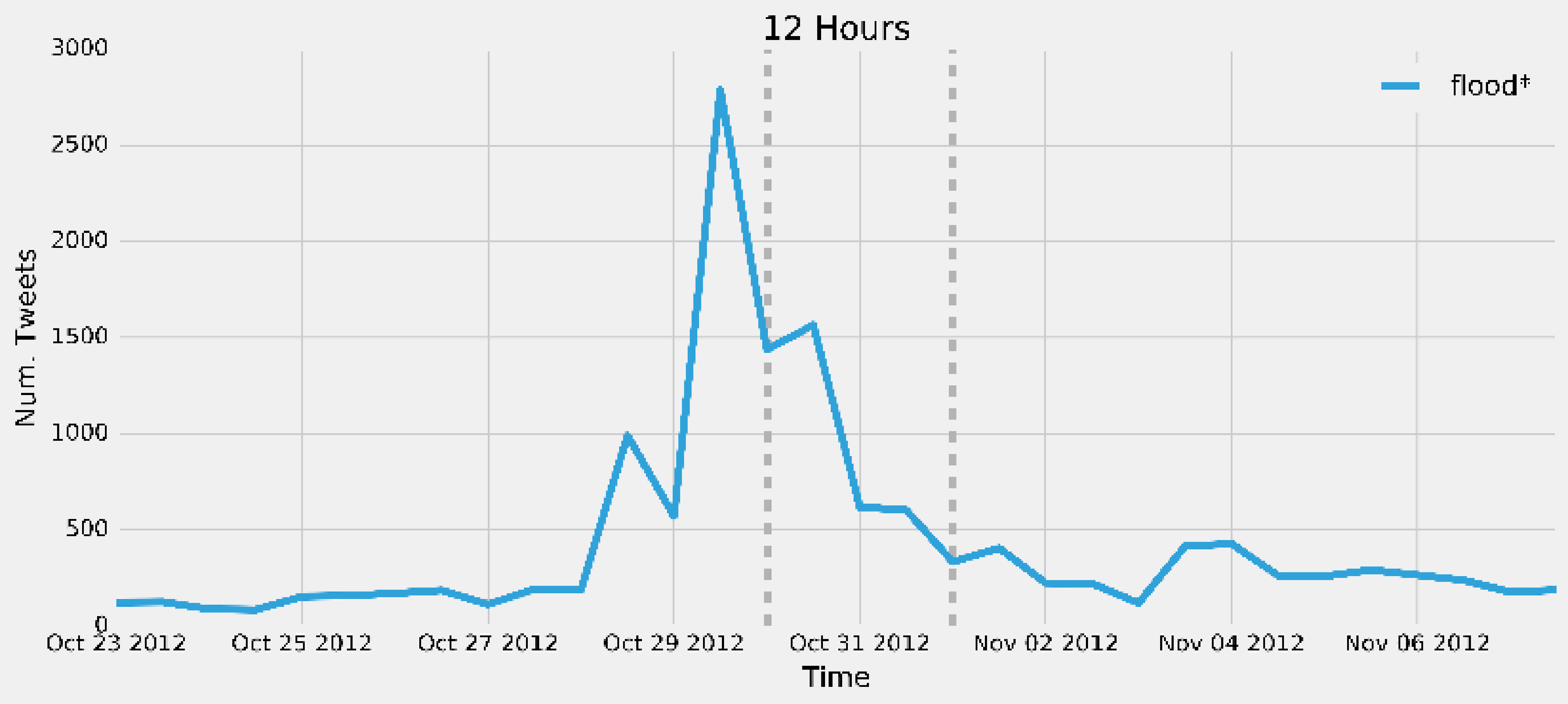
12 Hours
flood‡
Num. Tweets
0
500
1000
1500
2000
2500
3000
Oct 23 2012
Oct 25 2012
Oct 27 2012
Oct 29 2012
Oct 31 2012
Nov 02 2012
Nov 04 2012
Nov 06 2012
Time

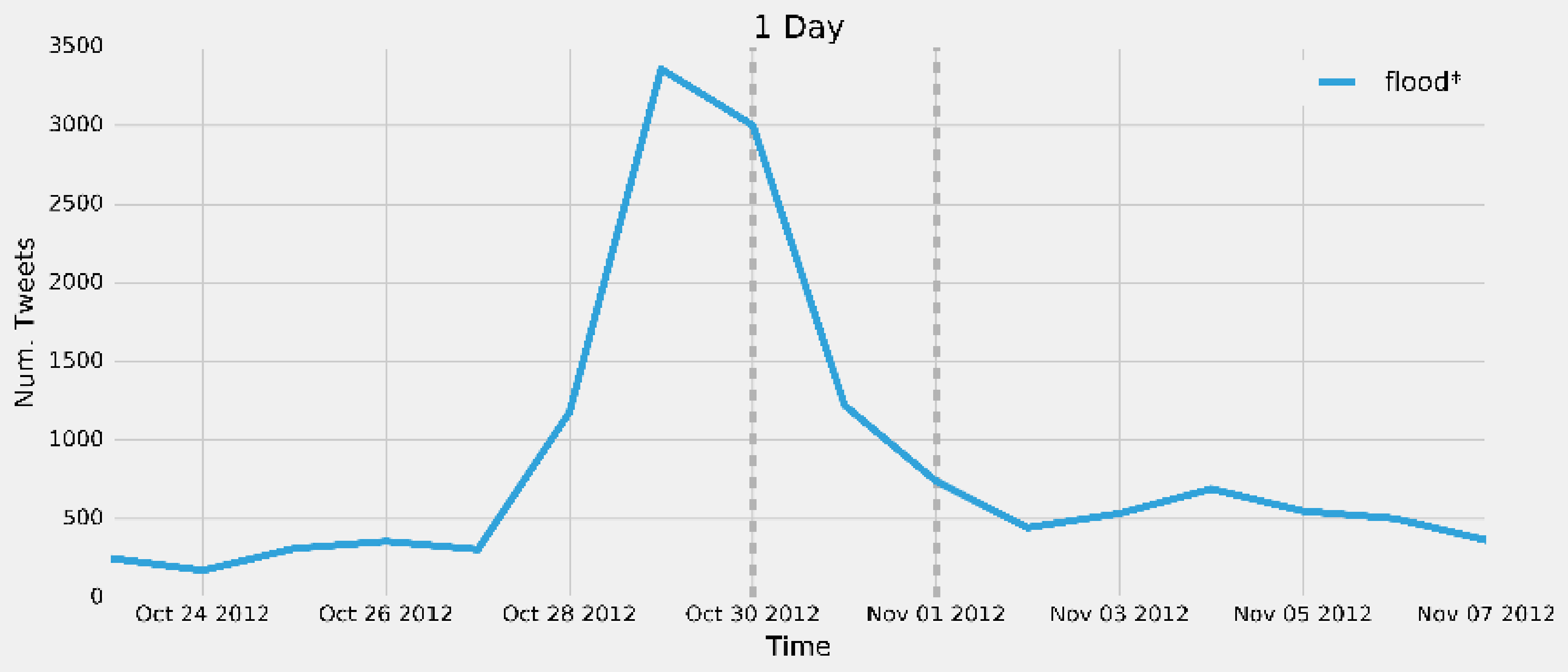

1 Day
flood*
Num. Tweets
0
500
1000
1500
2000
2500
3000
3500
Oct 24 2012
Oct 26 2012
Oct 28 2012
Oct 30 2012
Nov 01 2012
Nov 03 2012
Nov 05 2012
Nov 07 2012
Time

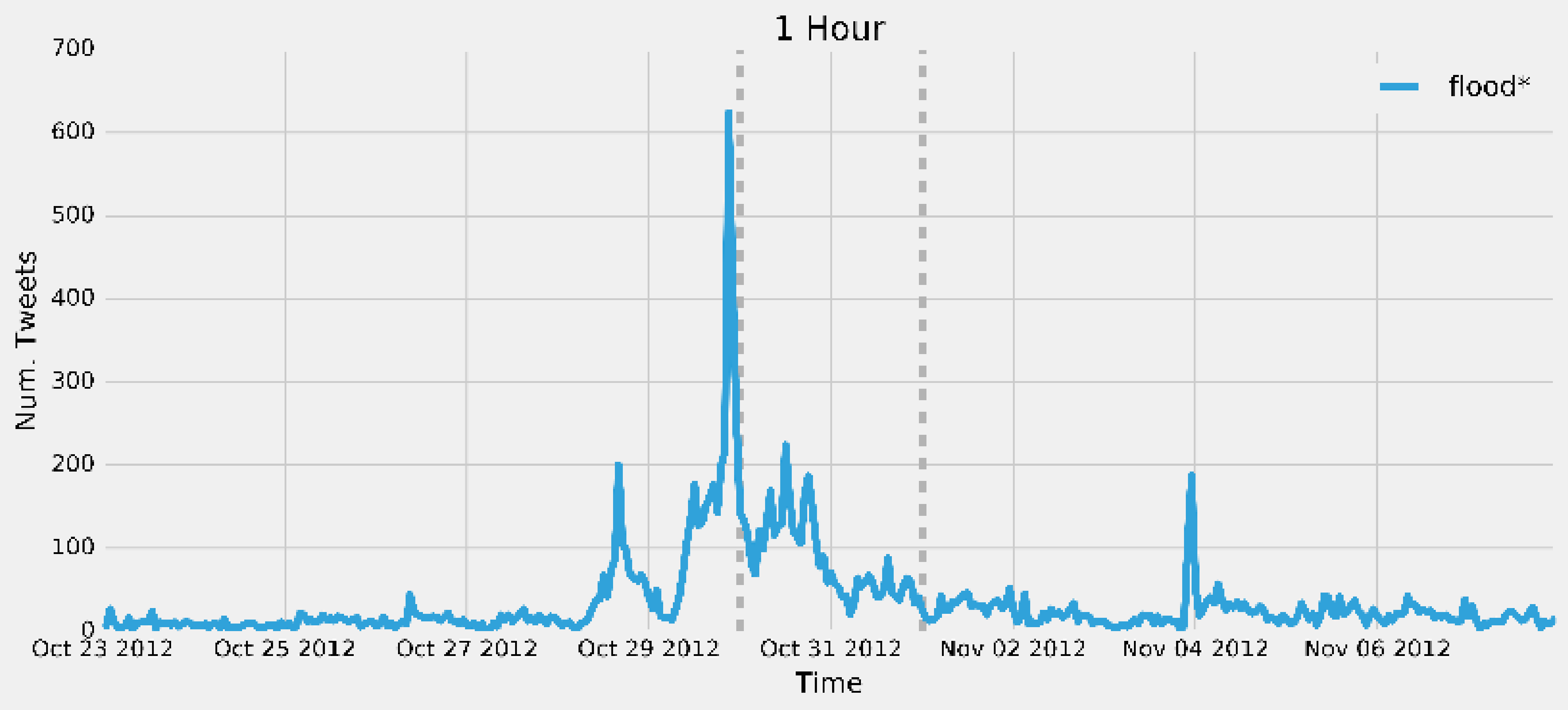

1 Hour
flood*
Num. Tweets
0
100
200
300
400
500
600
700
Oct 23 2012
Oct 25 2012
Oct 27 2012
Oct 29 2012
Oct 31 2012
Nov 02 2012
Nov 04 2012
Nov 06 2012
Time

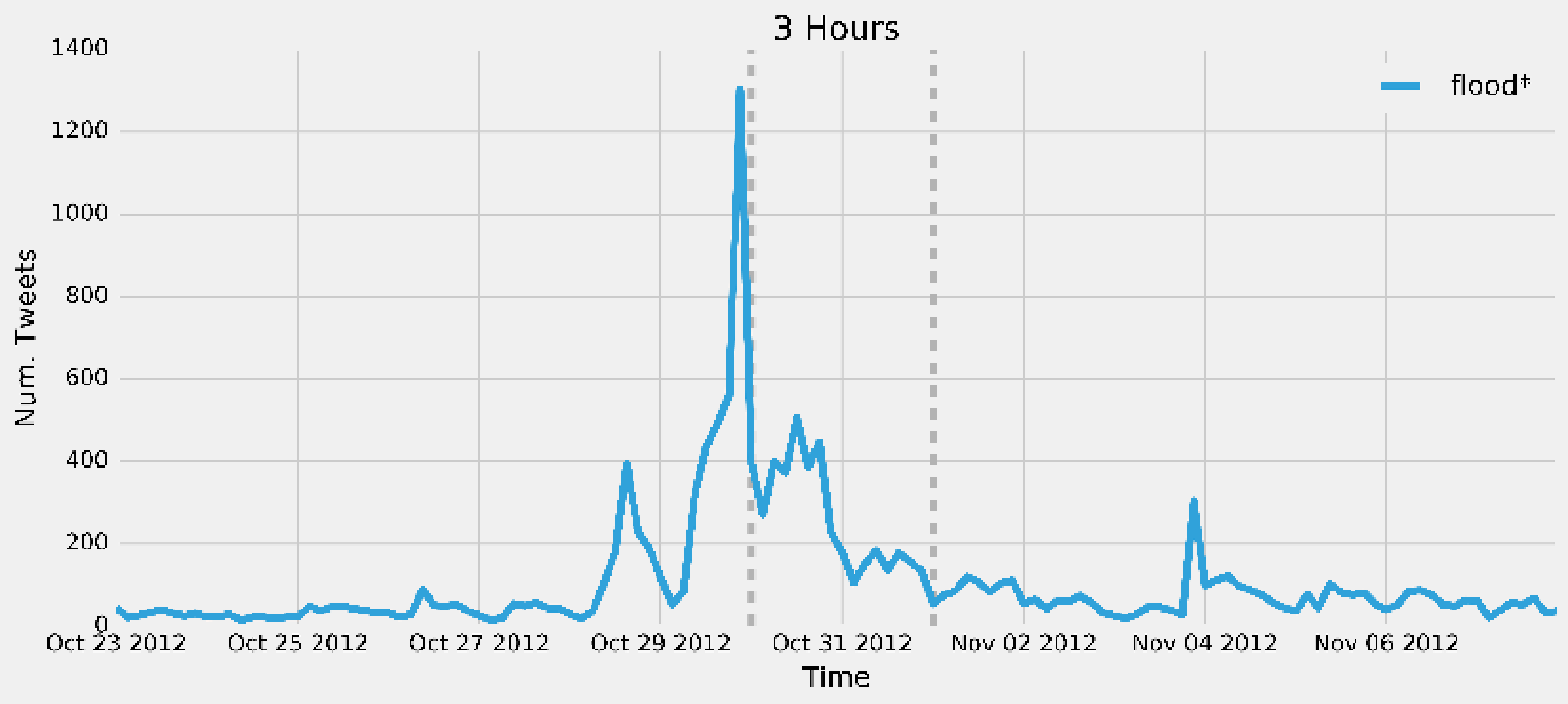

3 Hours
1400
1200
1000
800
600
400
200
0
Num. Tweets
flood‡
Oct 23 2012
Oct 25 2012
Oct 27 2012
Oct 29 2012
Oct 31 2012
Nov 02 2012
Nov 04 2012
Nov 06 2012
Time

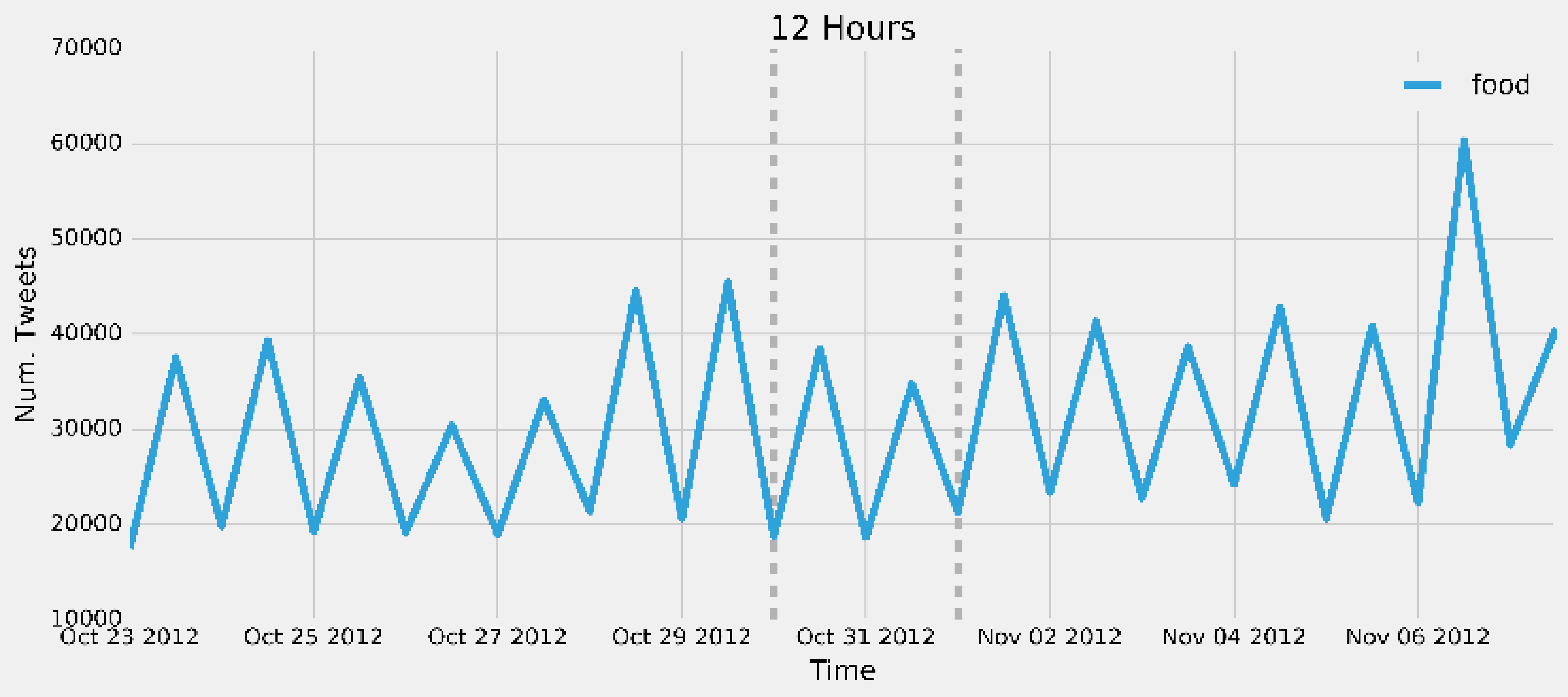
12 Hours
food
Num. Tweets
70000
60000
50000
40000
30000
20000
10000
Oct 23 2012
Oct 25 2012
Oct 27 2012
Oct 29 2012
Oct 31 2012
Nov 02 2012
Nov 04 2012
Nov 06 2012
Time

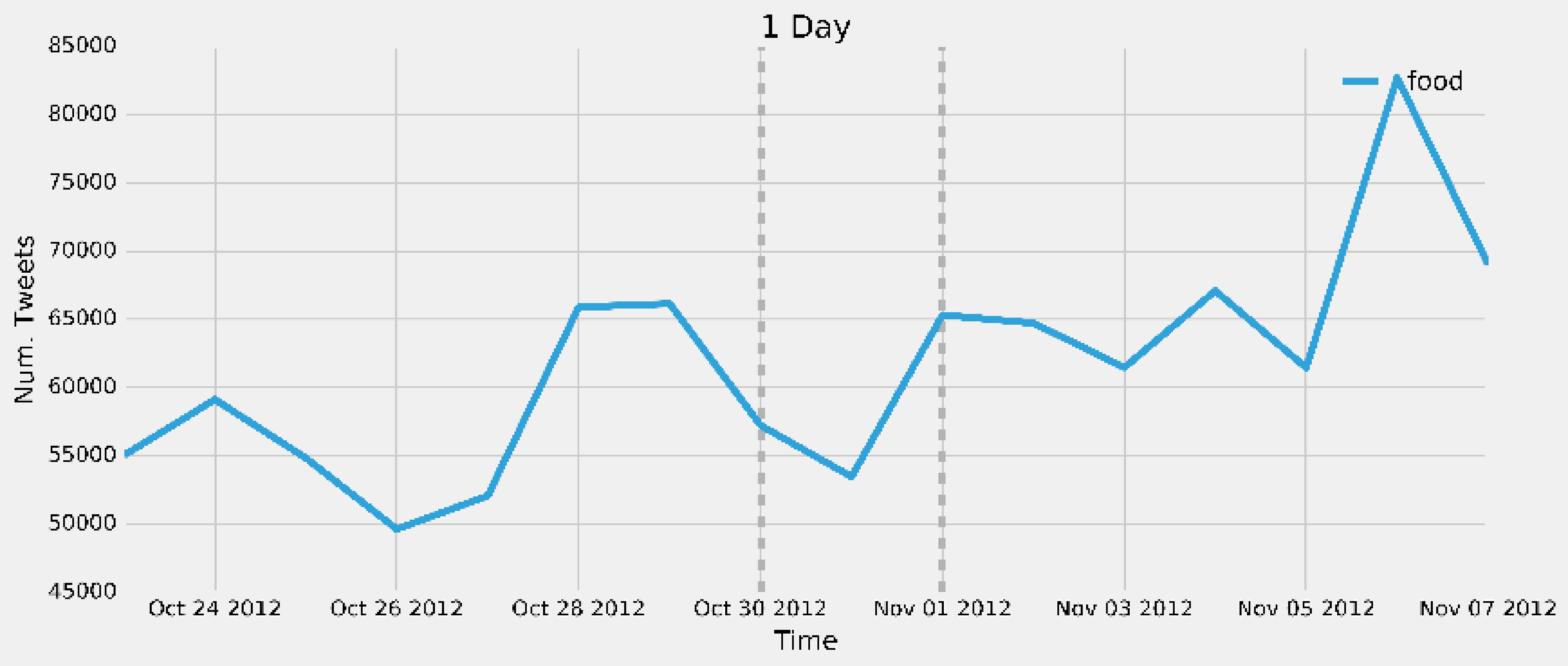
1 Day
food
Num. Tweets
85000
80000
75000
70000
65000
60000
55000
50000
45000
Oct 24 2012
Oct 26 2012
Oct 28 2012
Oct 30 2012
Nov 01 2012
Nov 03 2012
Nov 05 2012
Nov 07 2012
Time

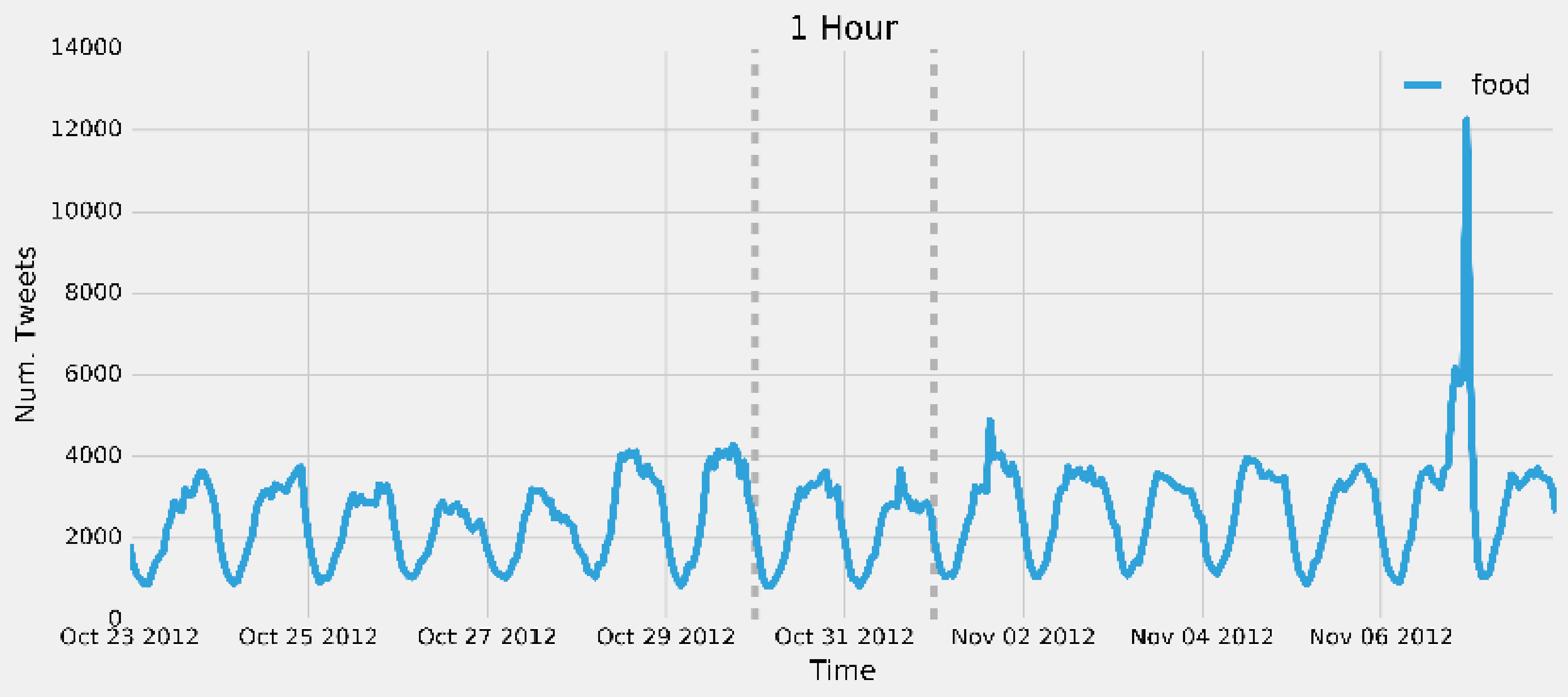
1 Hour
food
Num. Tweets
0
2000
4000
6000
8000
10000
12000
14000
Oct 23 2012
Oct 25 2012
Oct 27 2012
Oct 29 2012
Oct 31 2012
Nov 02 2012
Nov 04 2012
Nov 06 2012
Time

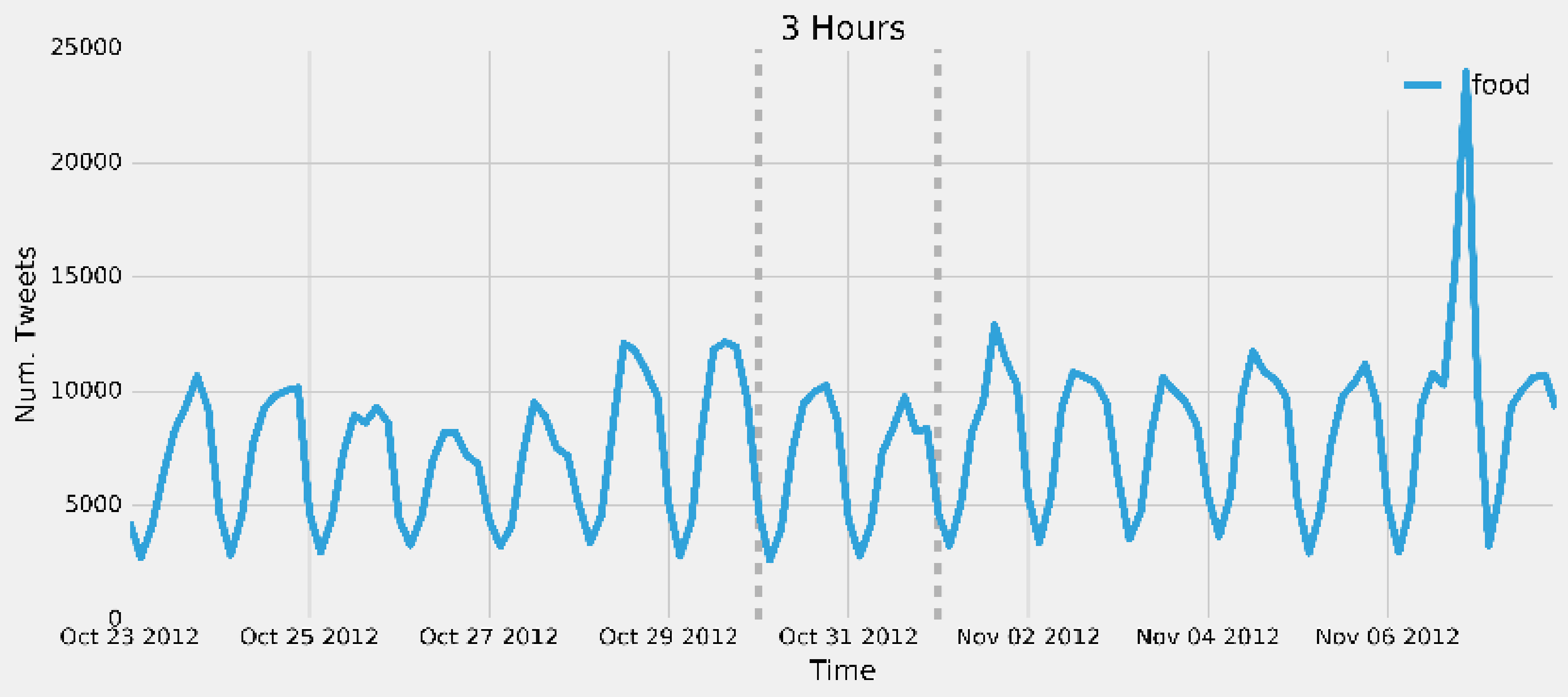

3 Hours
food
Num. Tweets
25000
20000
15000
10000
5000
0
Oct 23 2012
Oct 25 2012
Oct 27 2012
Oct 29 2012
Oct 31 2012
Nov 02 2012
Nov 04 2012
Nov 06 2012
Time

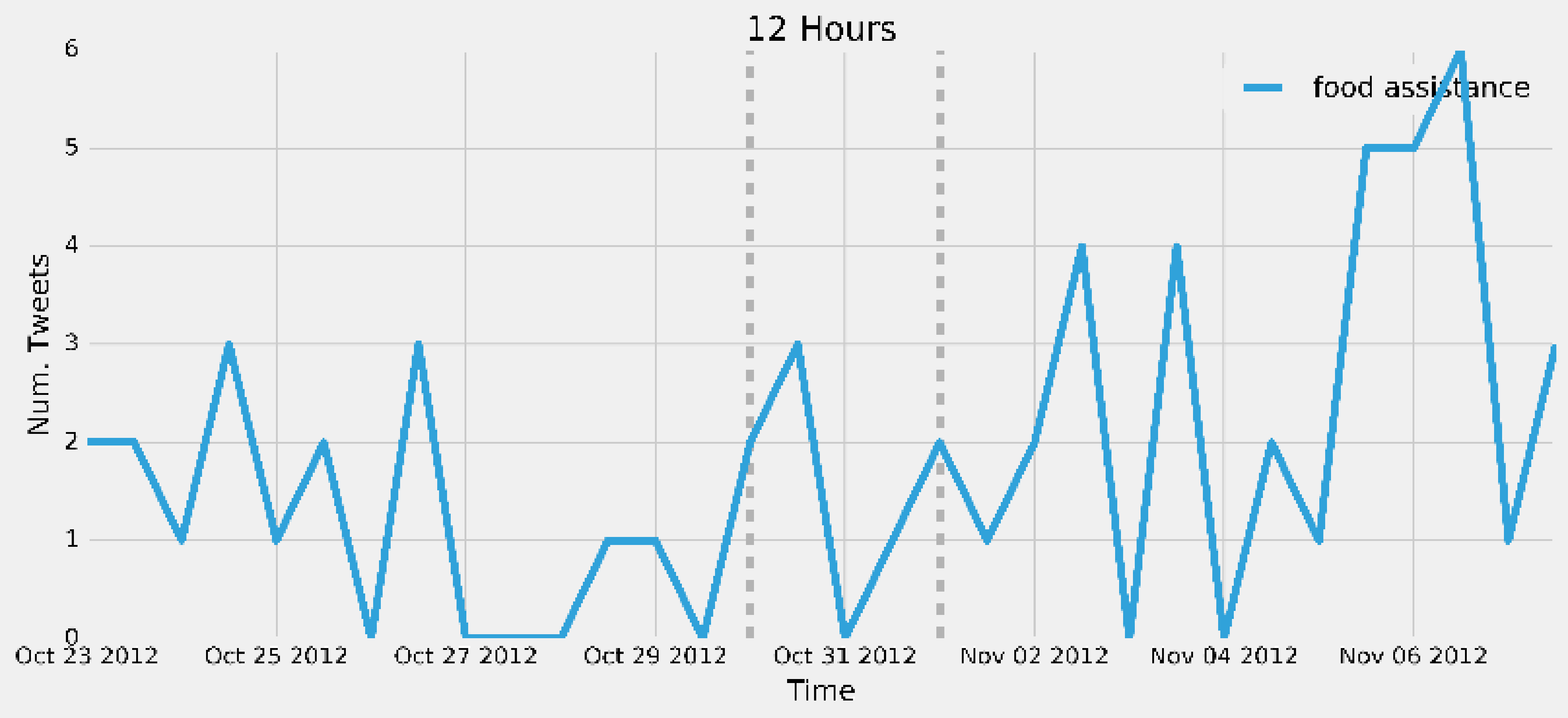

12 Hours
food assistance
Num. Tweets
0
1
2
3
4
5
6
Oct 23 2012
Oct 25 2012
Oct 27 2012
Oct 29 2012
Oct 31 2012
Nov 02 2012
Nov 04 2012
Nov 06 2012
Time

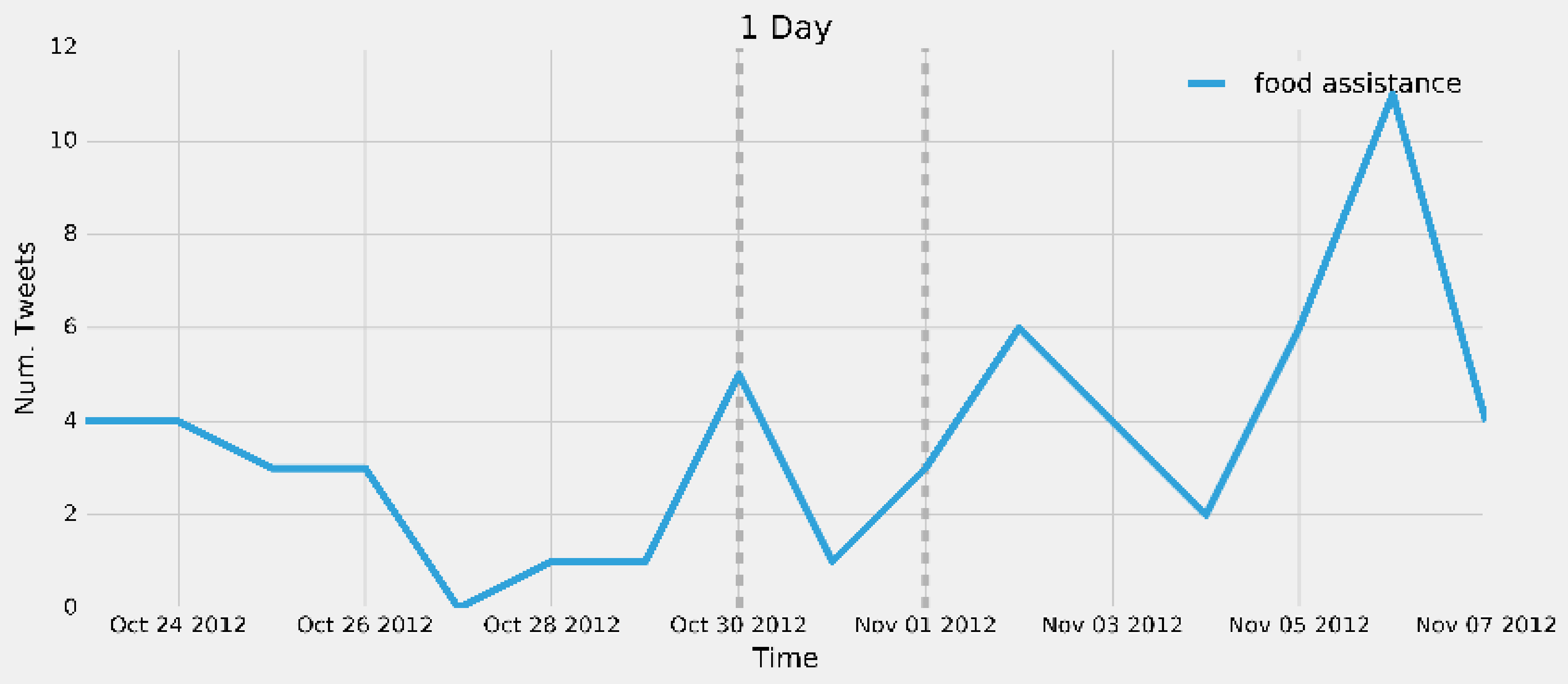

1 Day
food assistance
Num. Tweets
12
10
8
6
4
2
0
Oct 24 2012
Oct 26 2012
Oct 28 2012
Oct 30 2012
Nov 01 2012
Nov 03 2012
Nov 05 2012
Nov 07 2012
Time

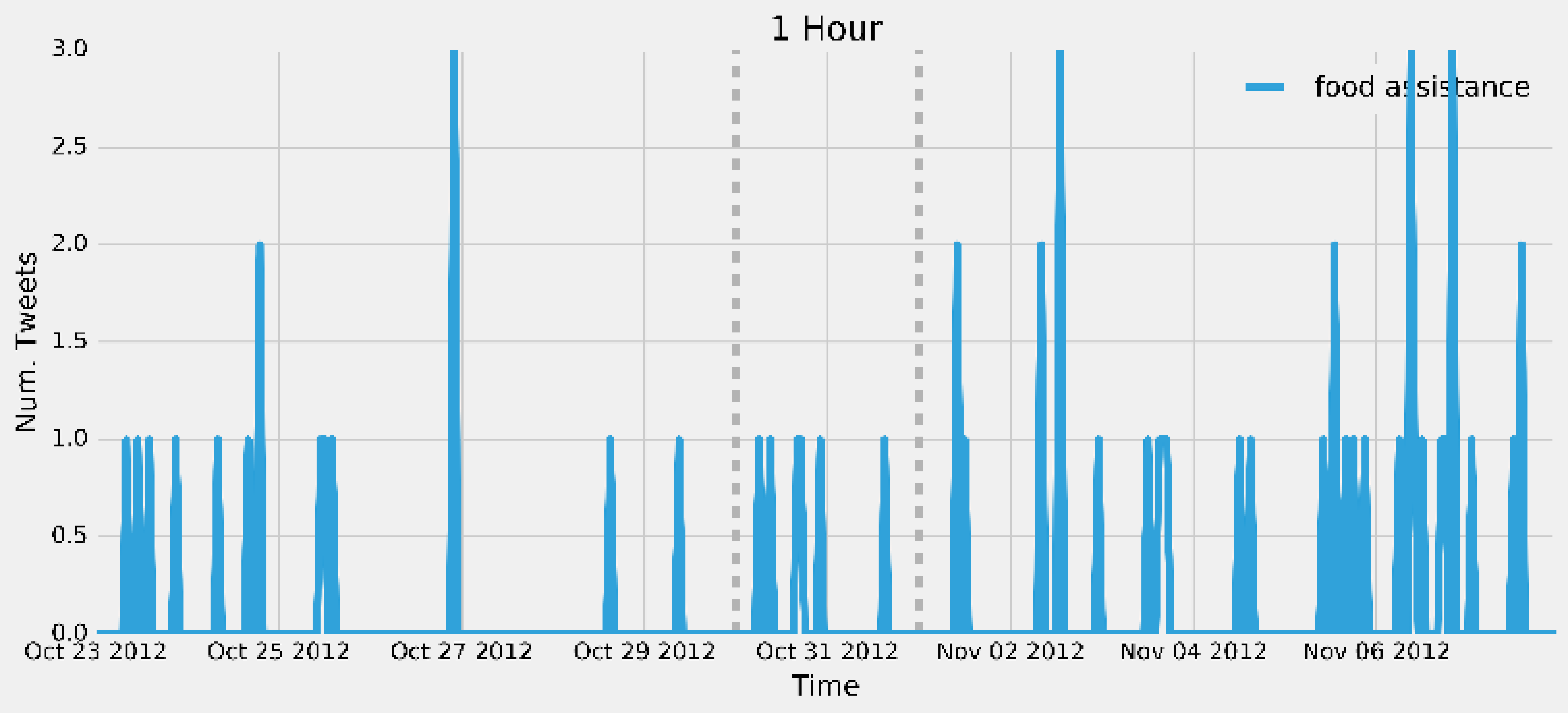
1 Hour
food assistance
Num. Tweets
0.0
0.5
1.0
1.5
2.0
2.5
3.0
Oct 23 2012
Oct 25 2012
Oct 27 2012
Oct 29 2012
Oct 31 2012
Nov 02 2012
Nov 04 2012
Nov 06 2012
Time

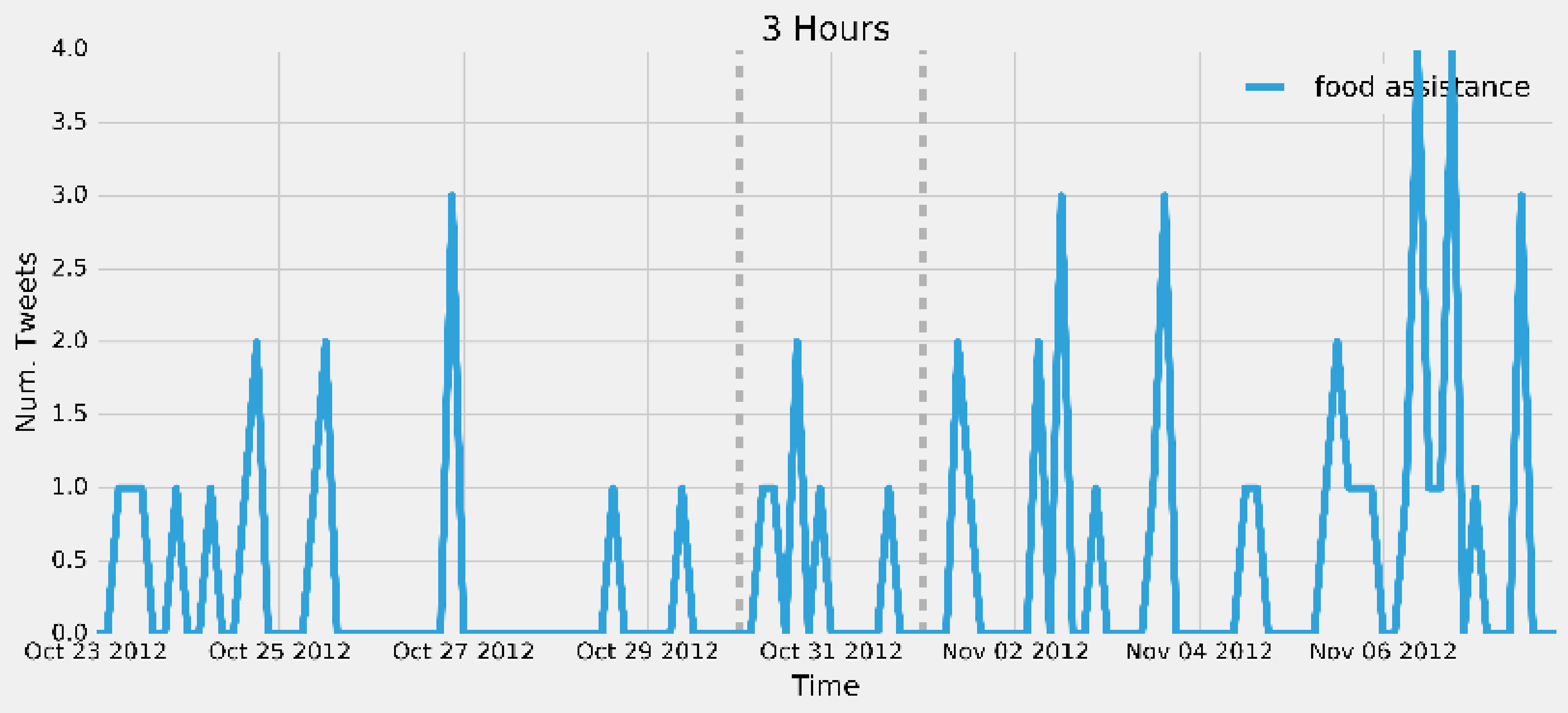
3 Hours
food assistance
Num. Tweets
Time
4.0
3.5
3.0
2.5
2.0
1.5
1.0
0.5
0.0
Oct 23 2012
Oct 25 2012
Oct 27 2012
Oct 29 2012
Oct 31 2012
Nov 02 2012
Nov 04 2012
Nov 06 2012

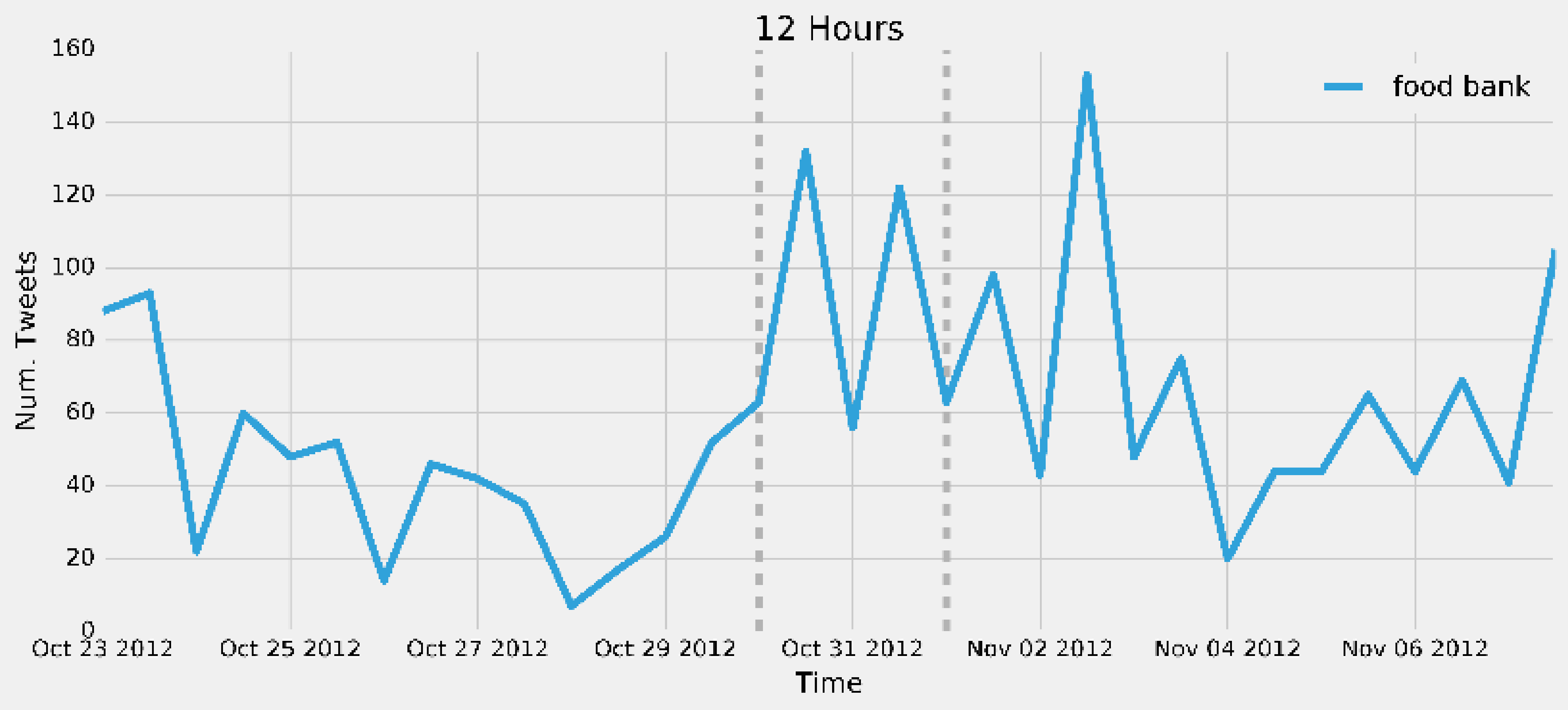

12 Hours
food bank
Num. Tweets
0
20
40
60
80
100
120
140
160
Oct 23 2012
Oct 25 2012
Oct 27 2012
Oct 29 2012
Oct 31 2012
Nov 02 2012
Nov 04 2012
Nov 06 2012
Time

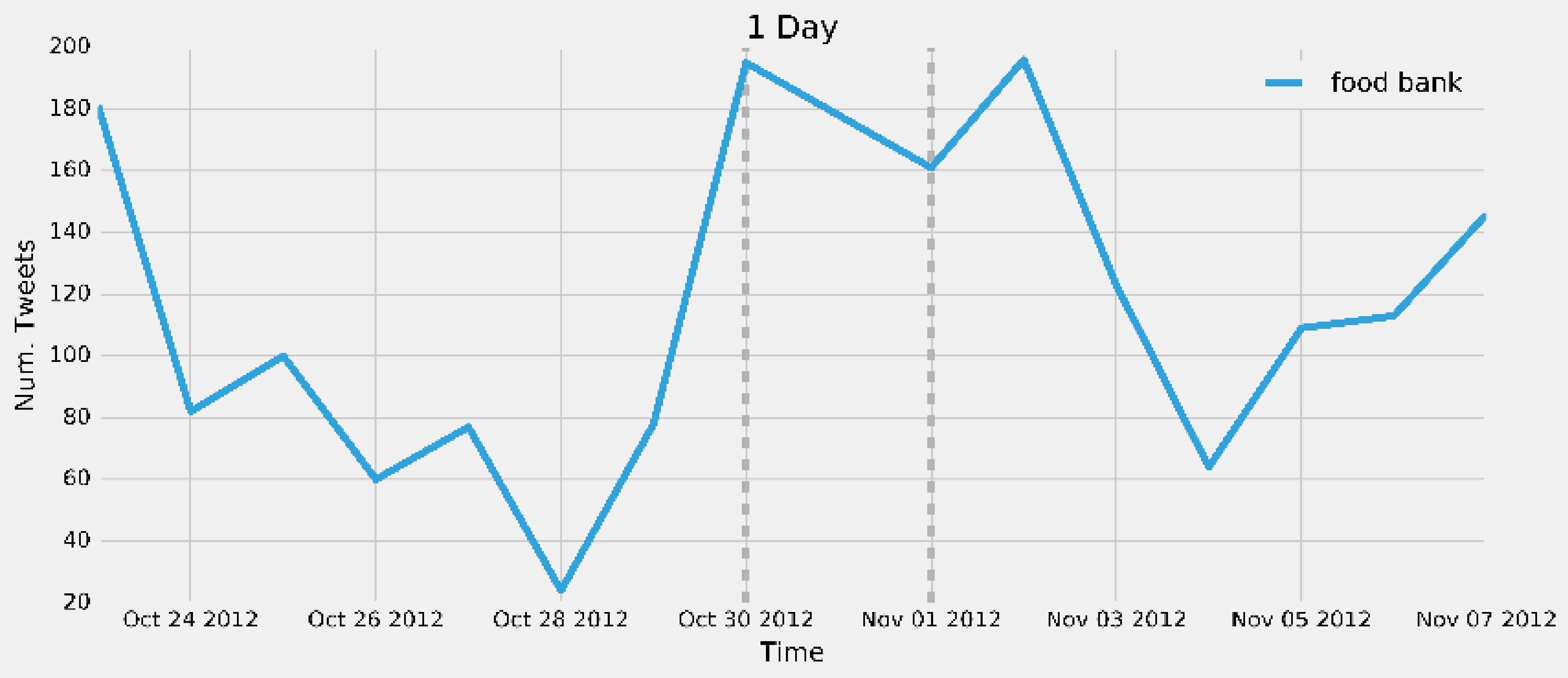
1 Day
food bank
Num. Tweets
200
180
160
140
120
100
80
60
40
20
Oct 24 2012
Oct 26 2012
Oct 28 2012
Oct 30 2012
Nov 01 2012
Nov 03 2012
Nov 05 2012
Nov 07 2012
Time

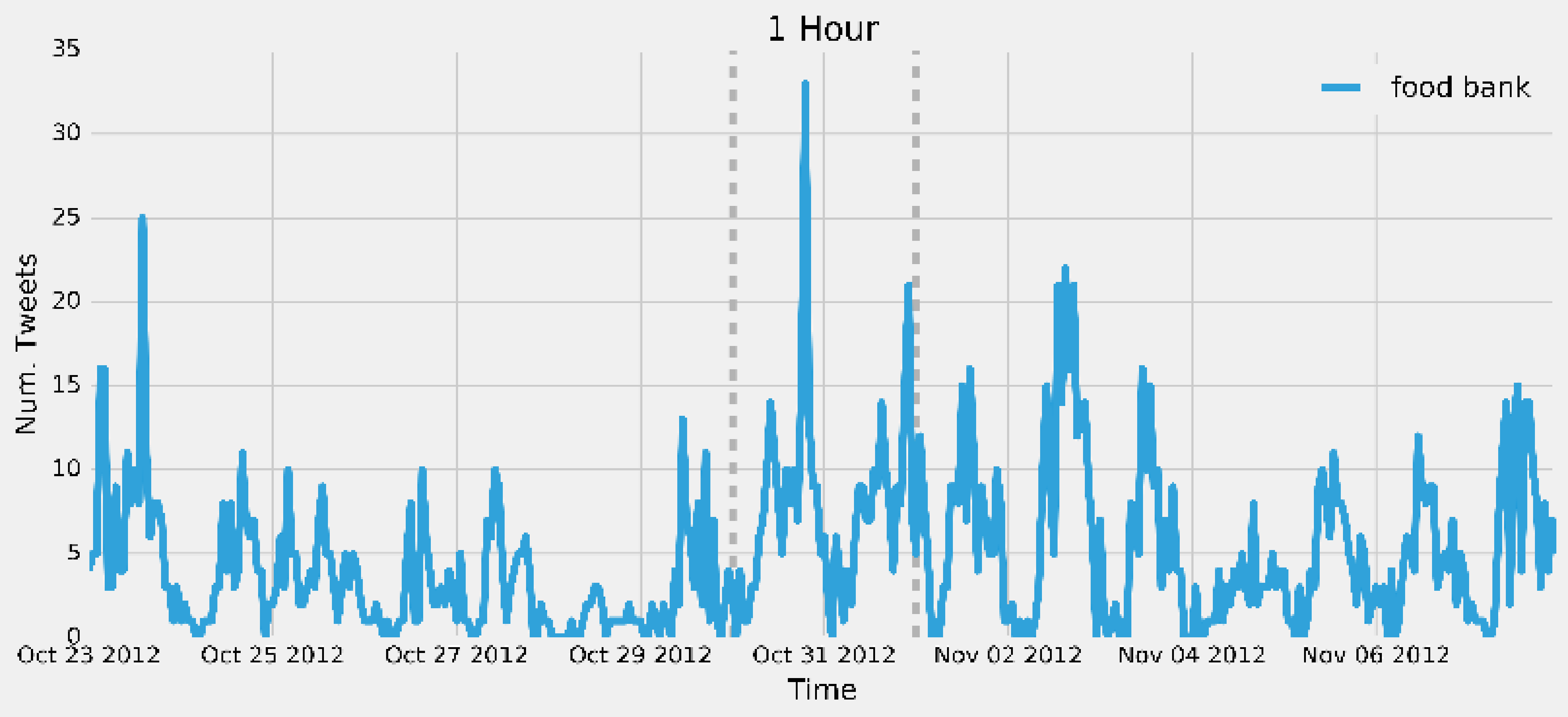
1 Hour
food bank
Num. Tweets
0
5
10
15
20
25
30
35
Oct 23 2012
Oct 25 2012
Oct 27 2012
Oct 29 2012
Oct 31 2012
Nov 02 2012
Nov 04 2012
Nov 06 2012
Time

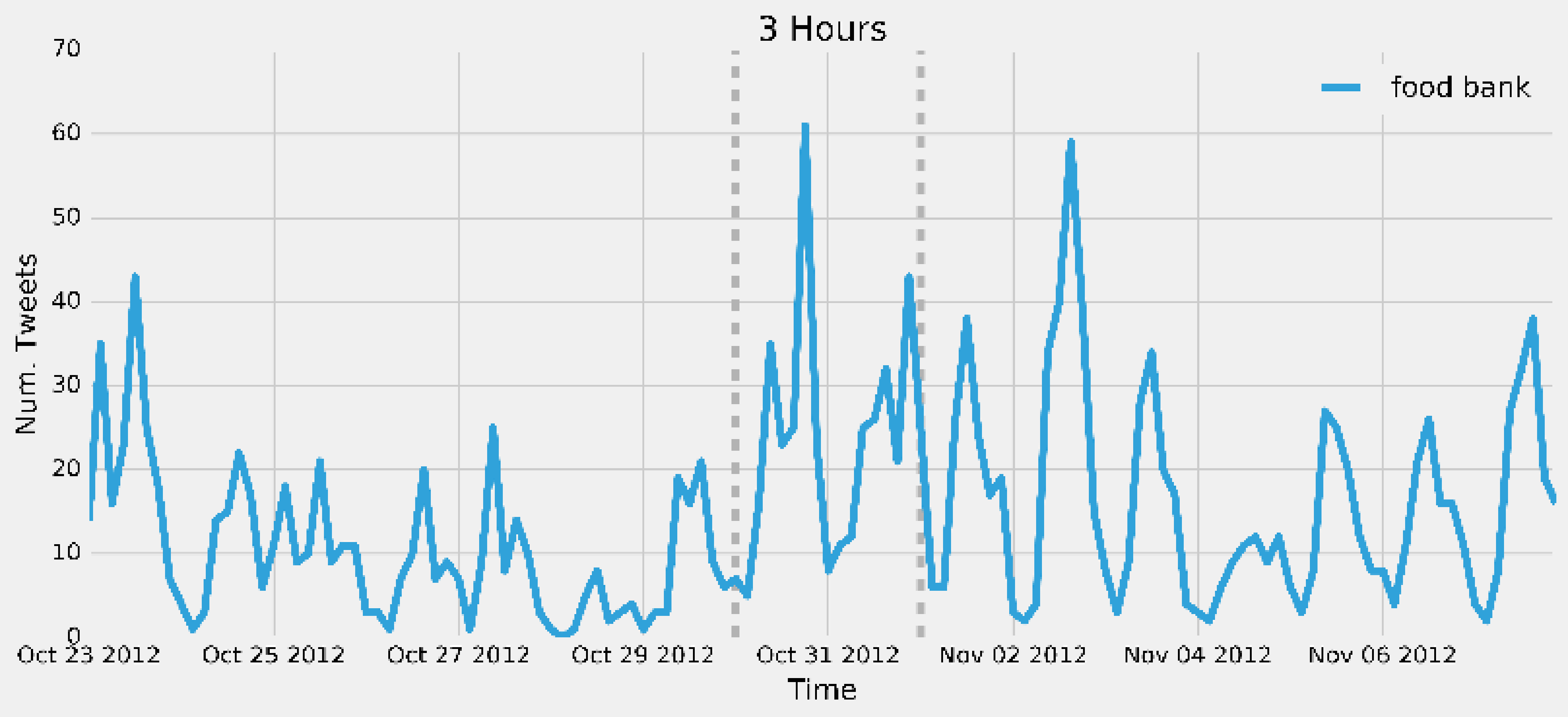

3 Hours
food bank
Num. Tweets
0
10
20
30
40
50
60
70
Oct 23 2012
Oct 25 2012
Oct 27 2012
Oct 29 2012
Oct 31 2012
Nov 02 2012
Nov 04 2012
Nov 06 2012
Time

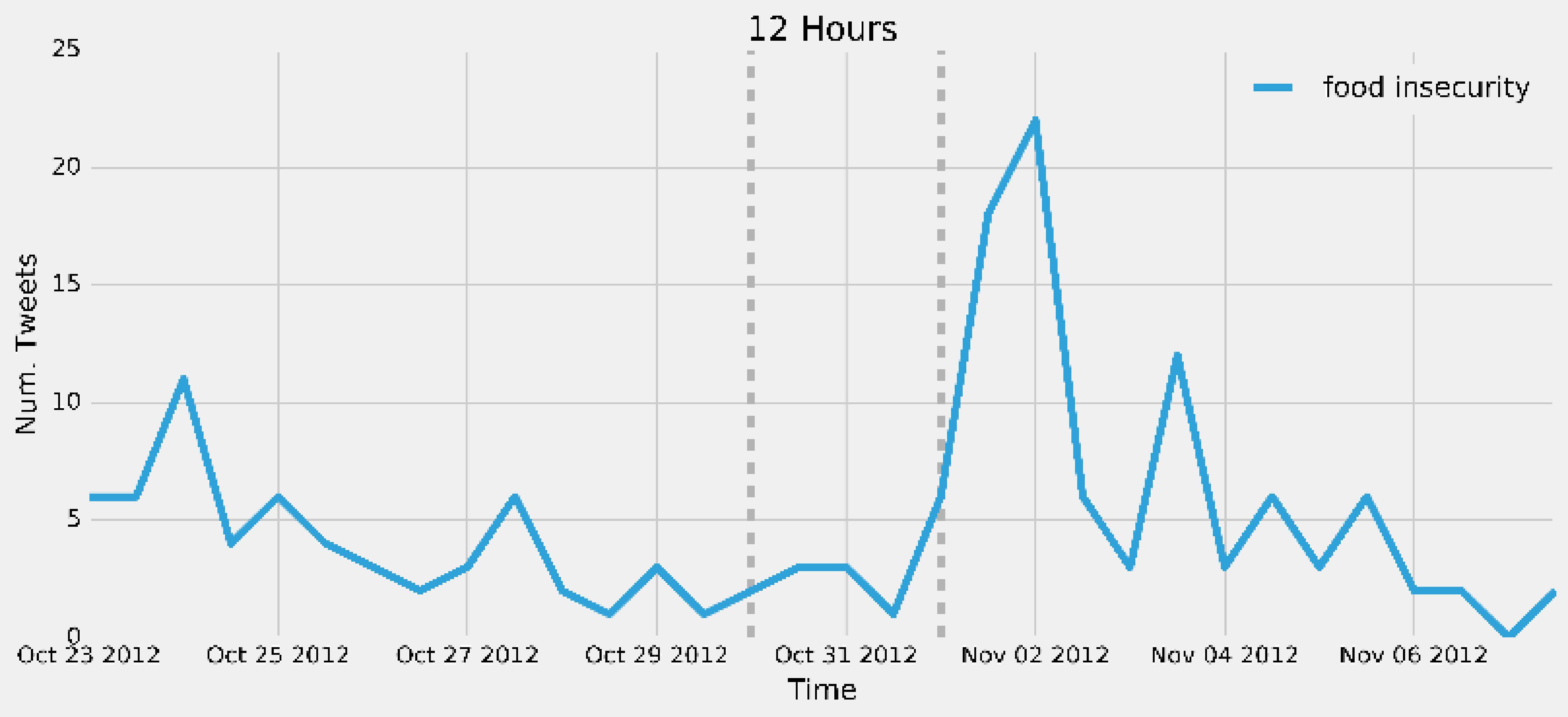
12 Hours
food insecurity
Num. Tweets
0
5
10
15
20
25
Oct 23 2012
Oct 25 2012
Oct 27 2012
Oct 29 2012
Oct 31 2012
Nov 02 2012
Nov 04 2012
Nov 06 2012
Time

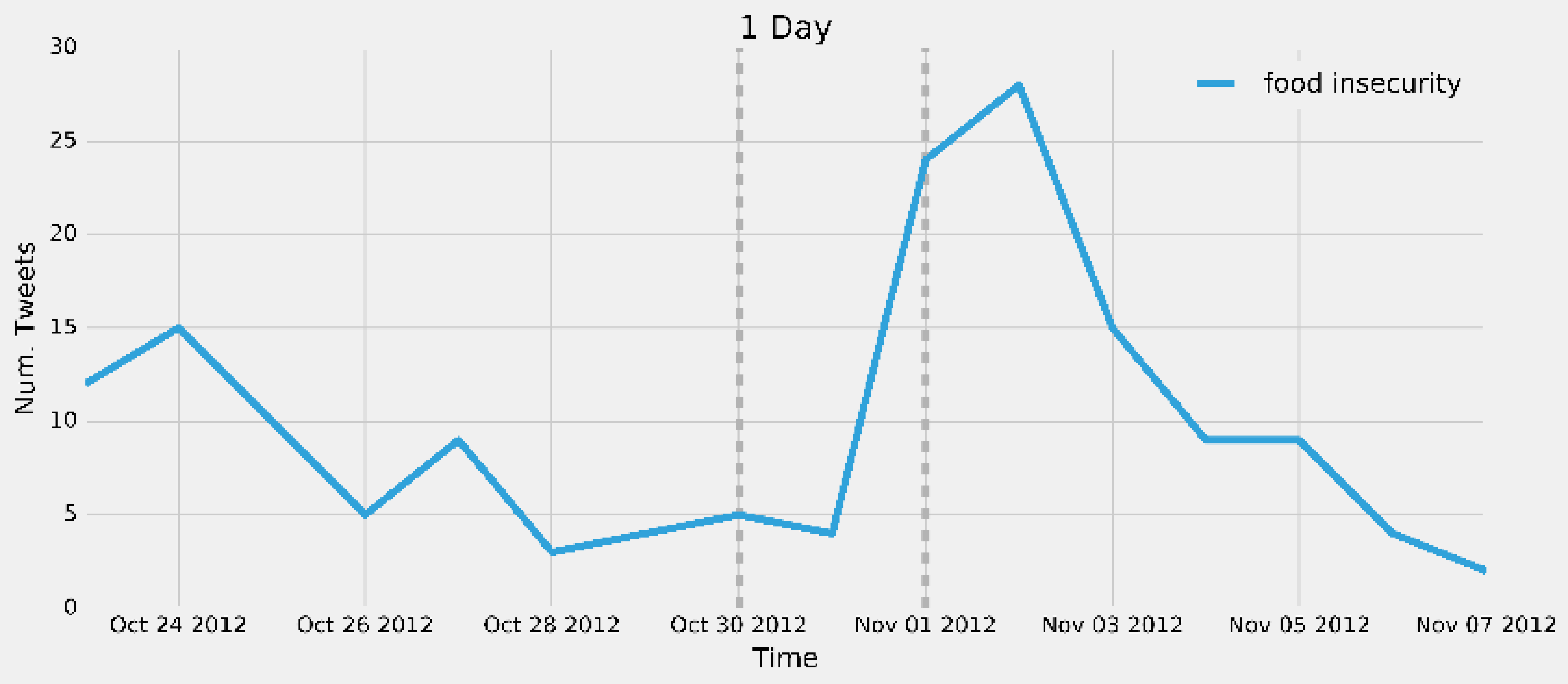

1 Day
food insecurity
Num. Tweets
0
5
10
15
20
25
30
Oct 24 2012
Oct 26 2012
Oct 28 2012
Oct 30 2012
Nov 01 2012
Nov 03 2012
Nov 05 2012
Nov 07 2012
Time

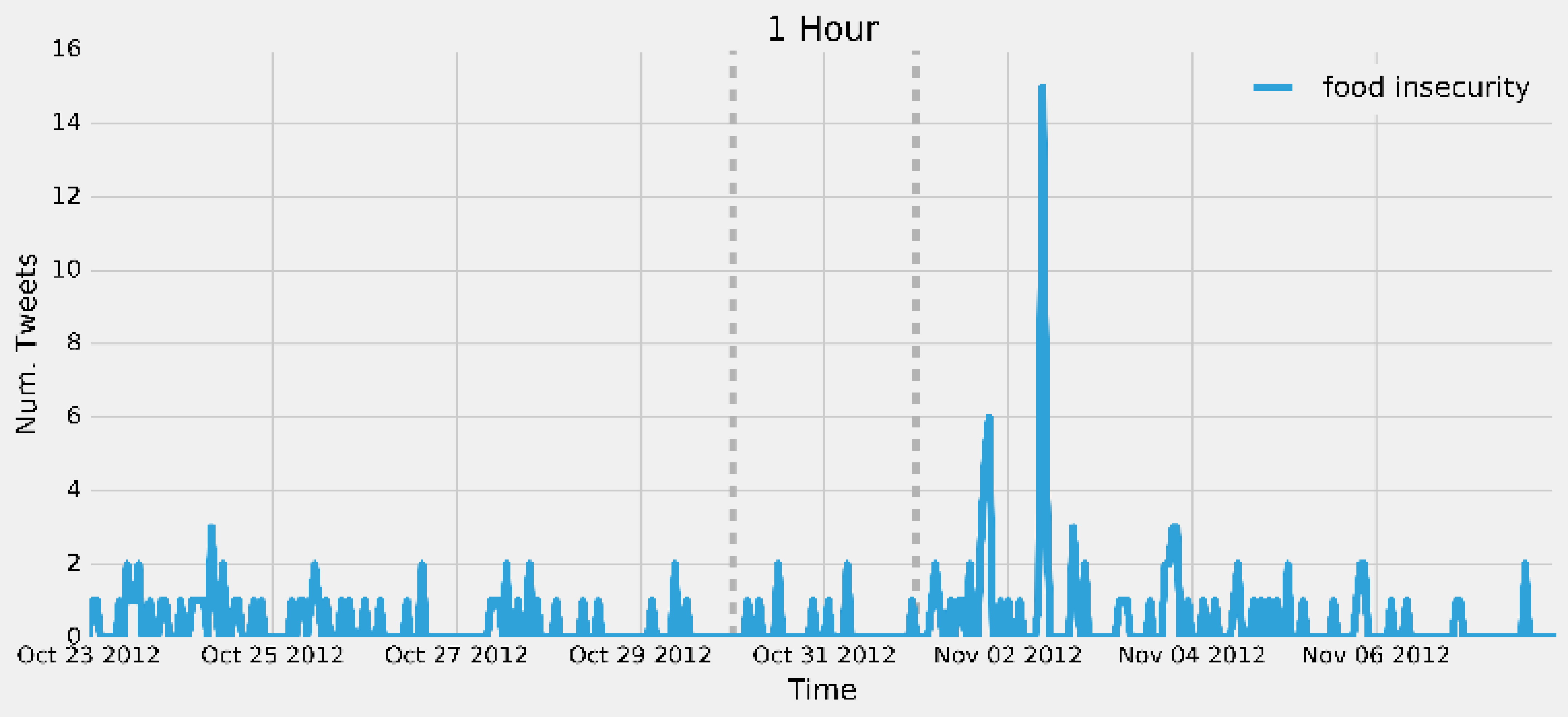
1 Hour
food insecurity
Num. Tweets
0
2
4
6
8
10
12
14
16
Oct 23 2012
Oct 25 2012
Oct 27 2012
Oct 29 2012
Oct 31 2012
Nov 02 2012
Nov 04 2012
Nov 06 2012
Time

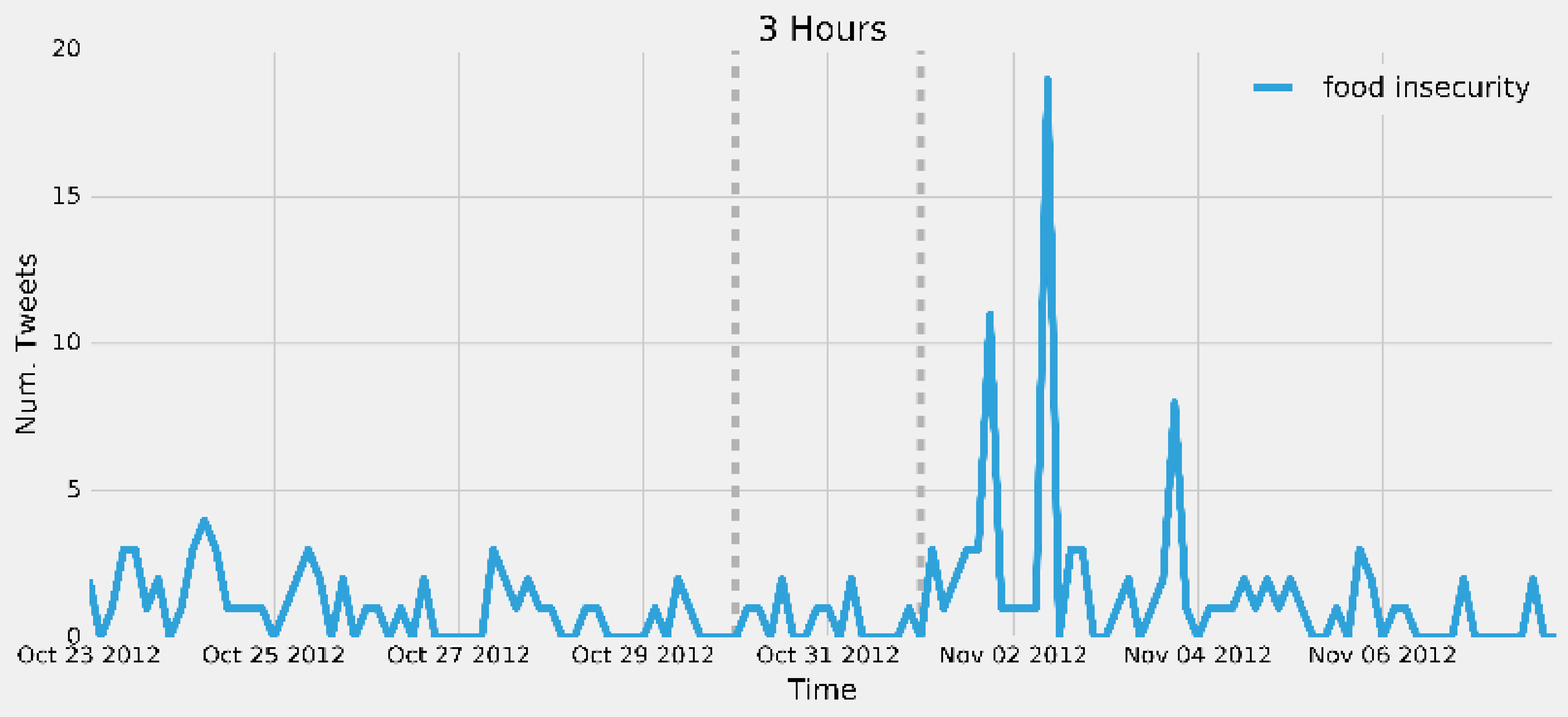
3 Hours
food insecurity
Num. Tweets
20
15
10
5
0
Oct 23 2012
Oct 25 2012
Oct 27 2012
Oct 29 2012
Oct 31 2012
Nov 02 2012
Nov 04 2012
Nov 06 2012
Time

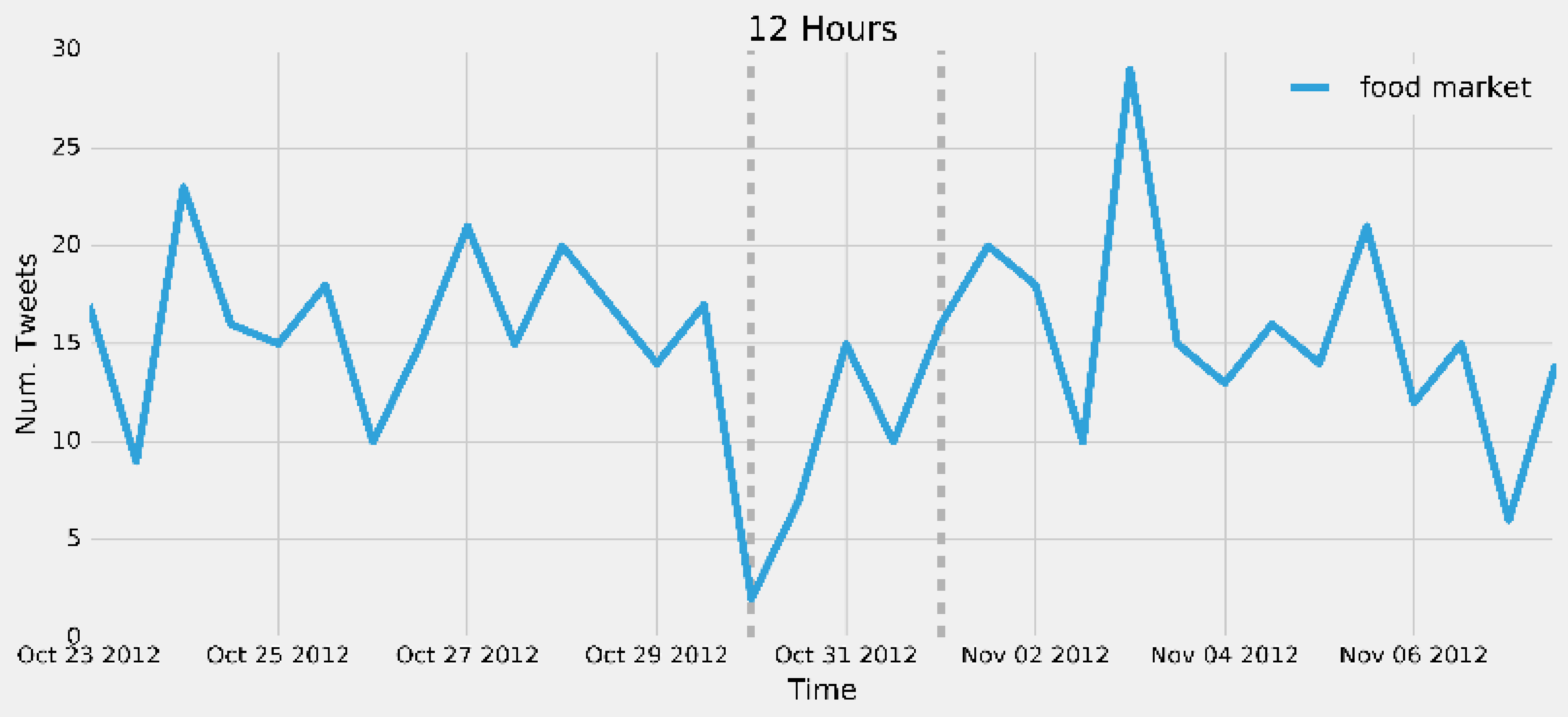

12 Hours
food market
Num. Tweets
Time
0
5
10
15
20
25
30
Oct 23 2012
Oct 25 2012
Oct 27 2012
Oct 29 2012
Oct 31 2012
Nov 02 2012
Nov 04 2012
Nov 06 2012

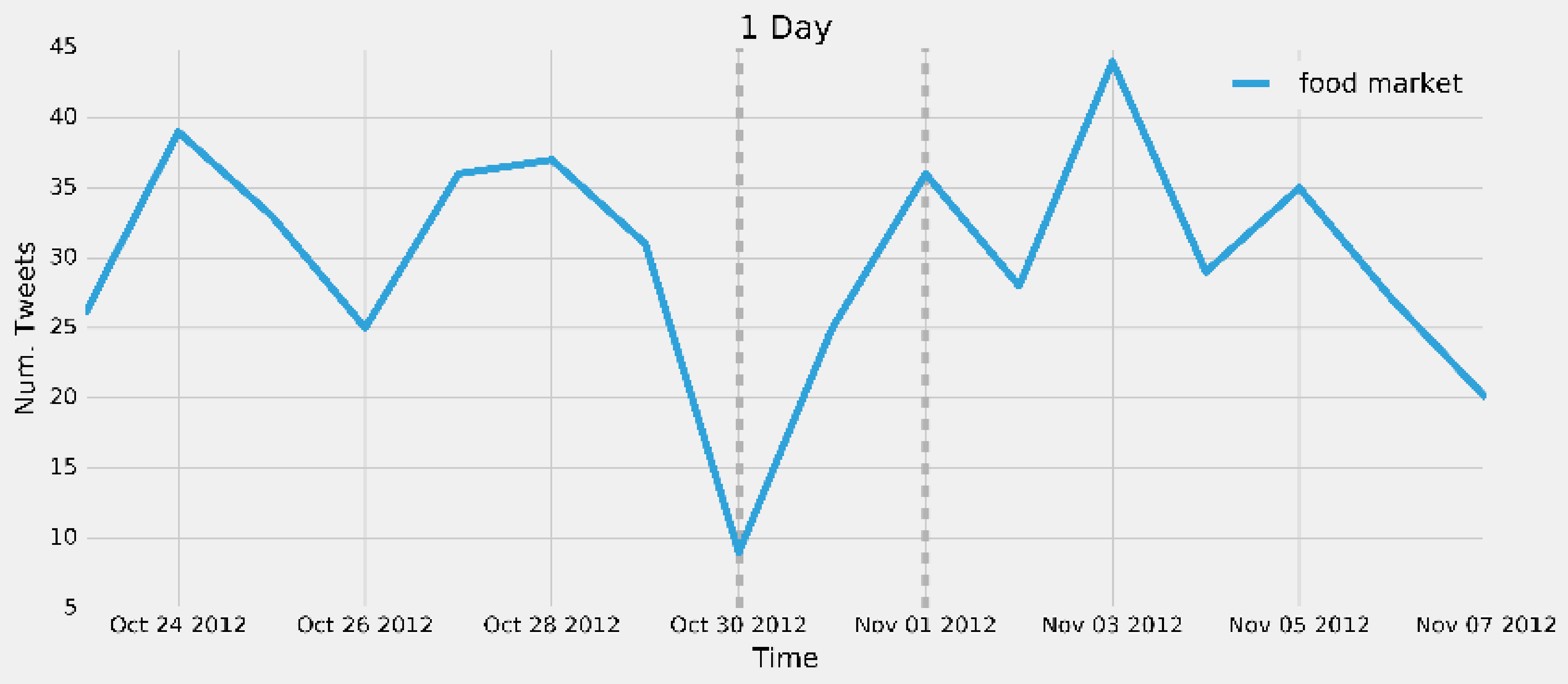

1 Day
food market
Num. Tweets
45
40
35
30
25
20
15
10
5
Oct 24 2012
Oct 26 2012
Oct 28 2012
Oct 30 2012
Nov 01 2012
Nov 03 2012
Nov 05 2012
Nov 07 2012
Time

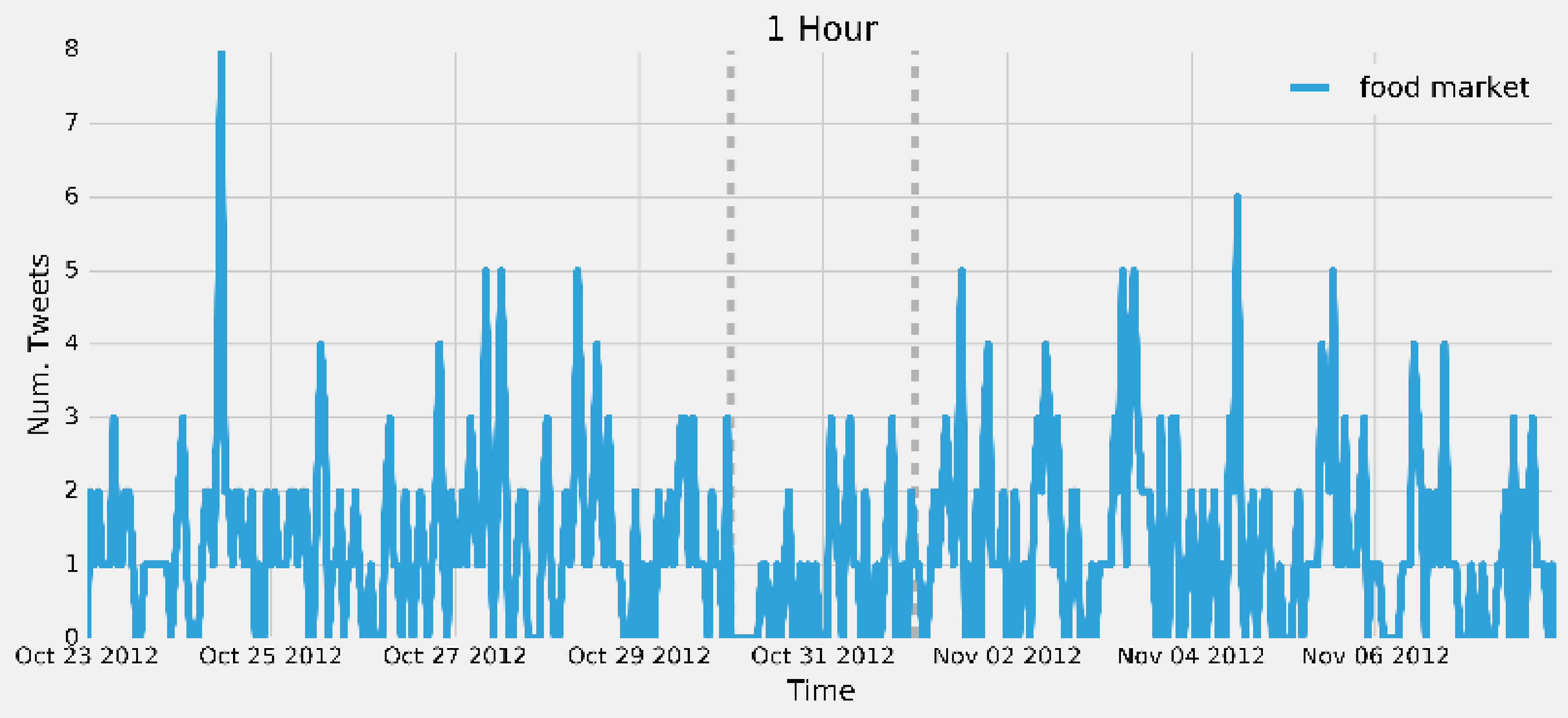
1 Hour
food market
Num. Tweets
0
1
2
3
4
5
6
7
8
Oct 23 2012
Oct 25 2012
Oct 27 2012
Oct 29 2012
Oct 31 2012
Nov 02 2012
Nov 04 2012
Nov 06 2012
Time

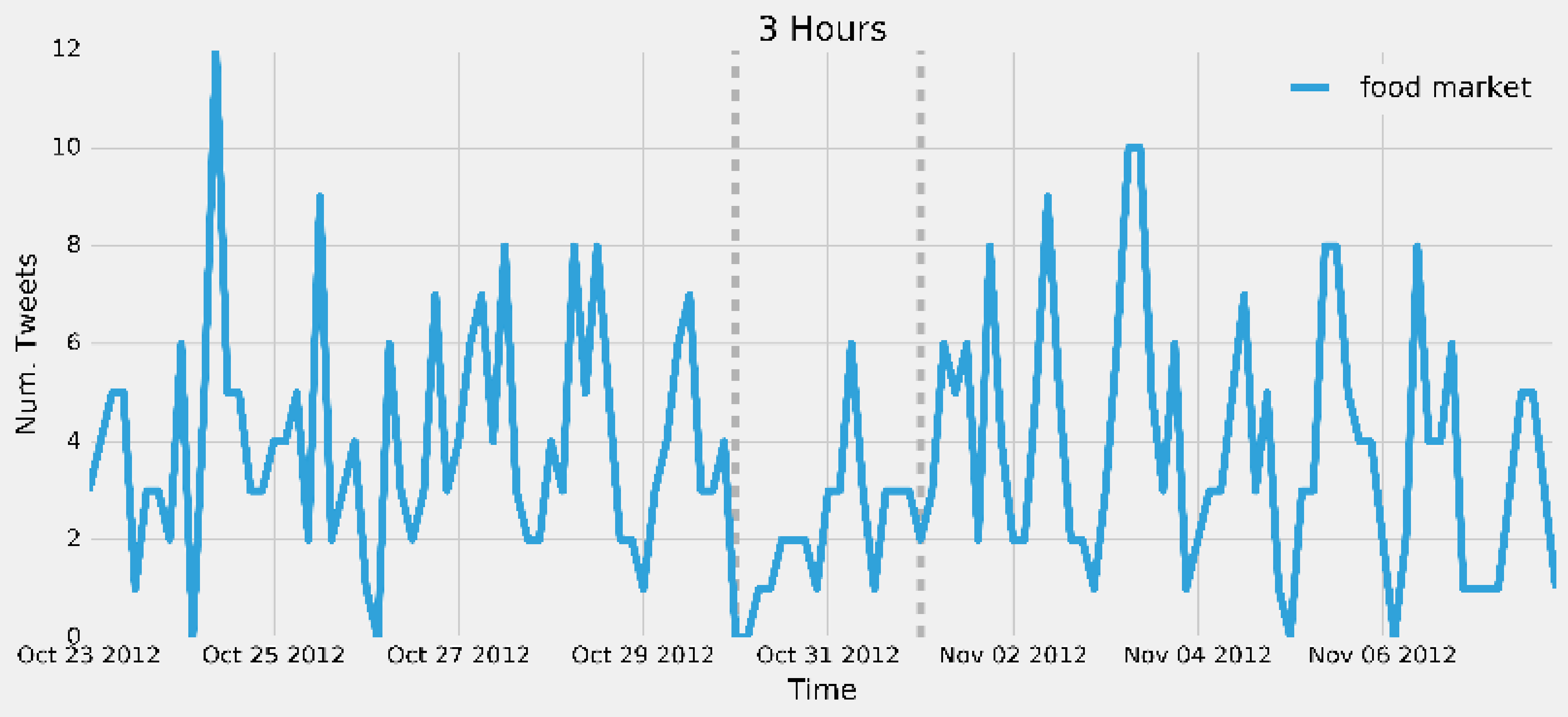
3 Hours
food market
Num. Tweets
0
2
4
6
8
10
12
Oct 23 2012
Oct 25 2012
Oct 27 2012
Oct 29 2012
Oct 31 2012
Nov 02 2012
Nov 04 2012
Nov 06 2012
Time

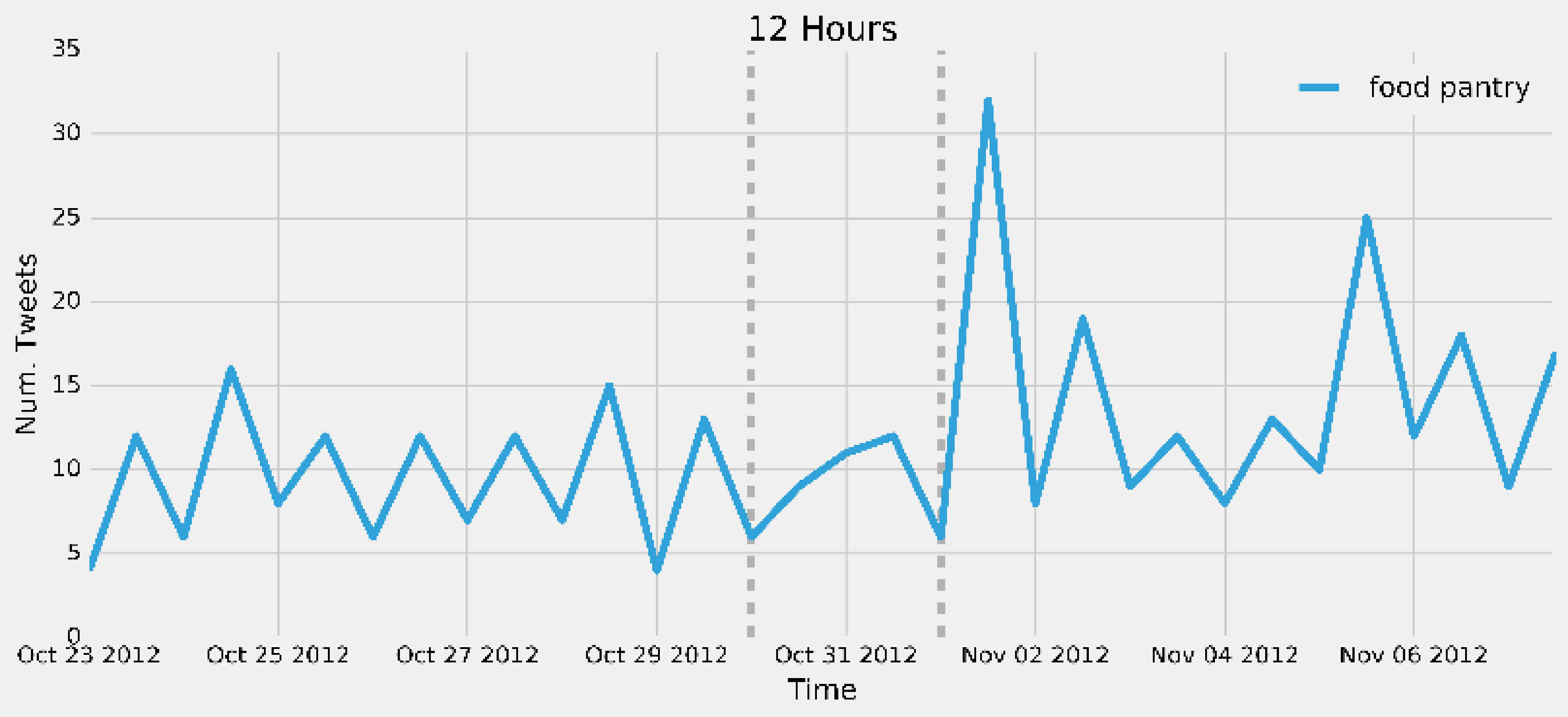
12 Hours
food pantry
Num. Tweets
35
30
25
20
15
10
5
0
Oct 23 2012
Oct 25 2012
Oct 27 2012
Oct 29 2012
Oct 31 2012
Nov 02 2012
Nov 04 2012
Nov 06 2012
Time

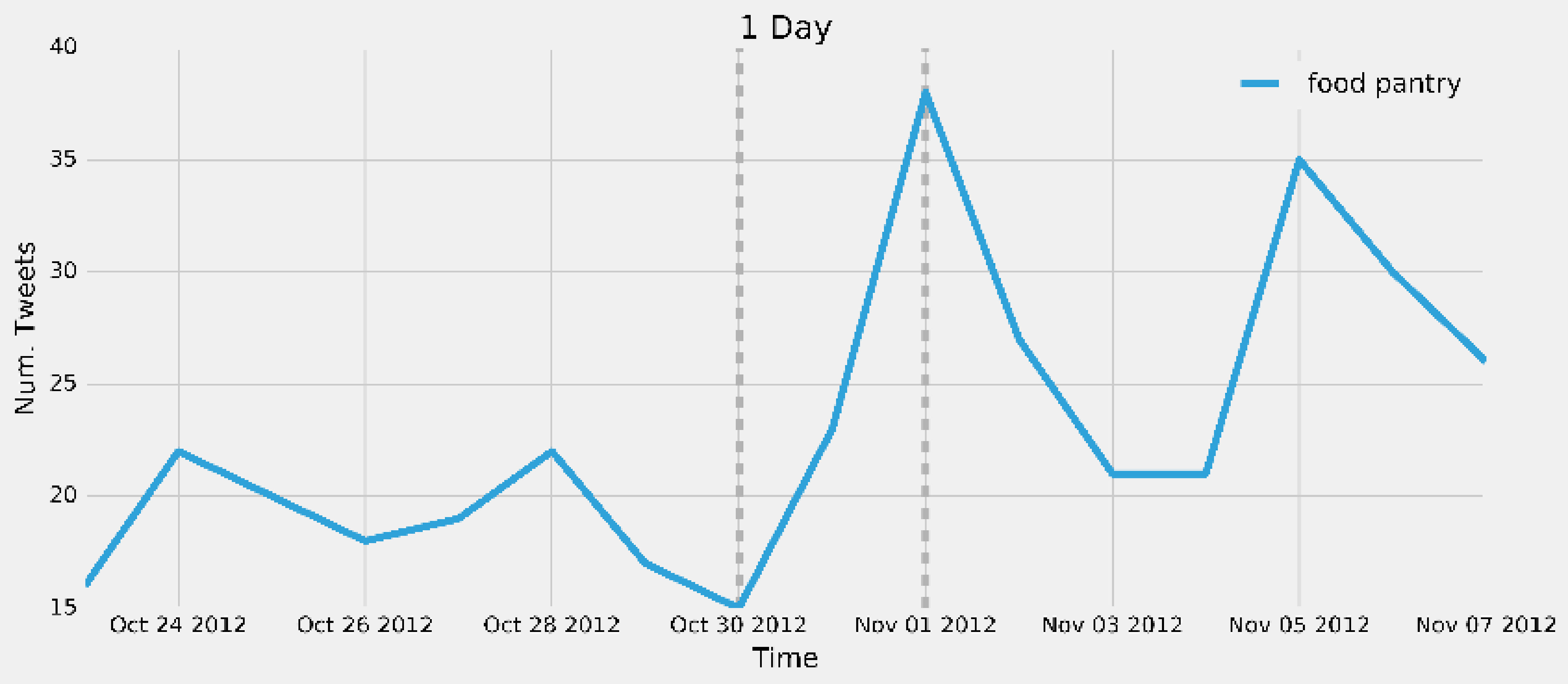

1 Day
food pantry
Num. Tweets
Time
40
35
30
25
20
15
Oct 24 2012
Oct 26 2012
Oct 28 2012
Oct 30 2012
Nov 01 2012
Nov 03 2012
Nov 05 2012
Nov 07 2012

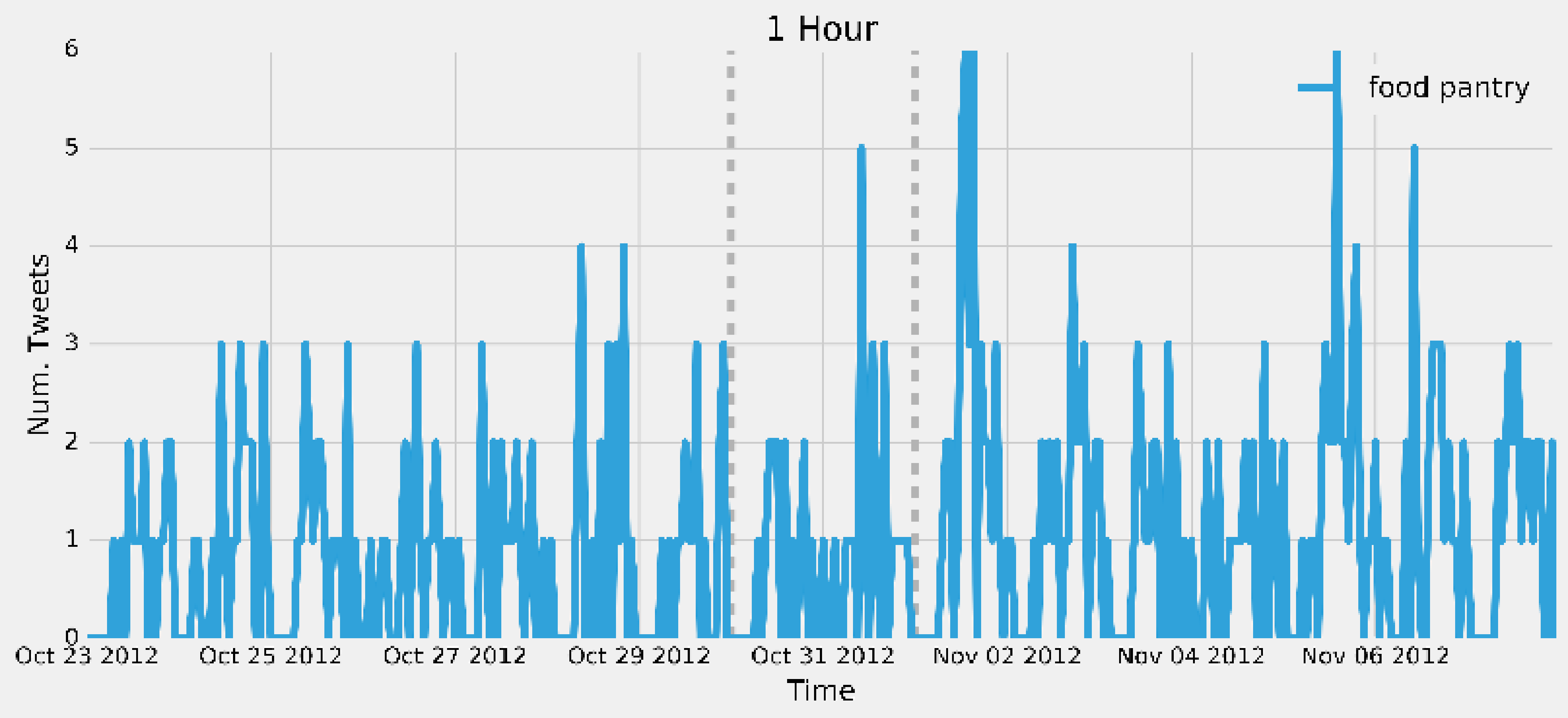

1 Hour
food pantry
Num. Tweets
0
1
2
3
4
5
6
Oct 23 2012
Oct 25 2012
Oct 27 2012
Oct 29 2012
Oct 31 2012
Nov 02 2012
Nov 04 2012
Nov 06 2012
Time

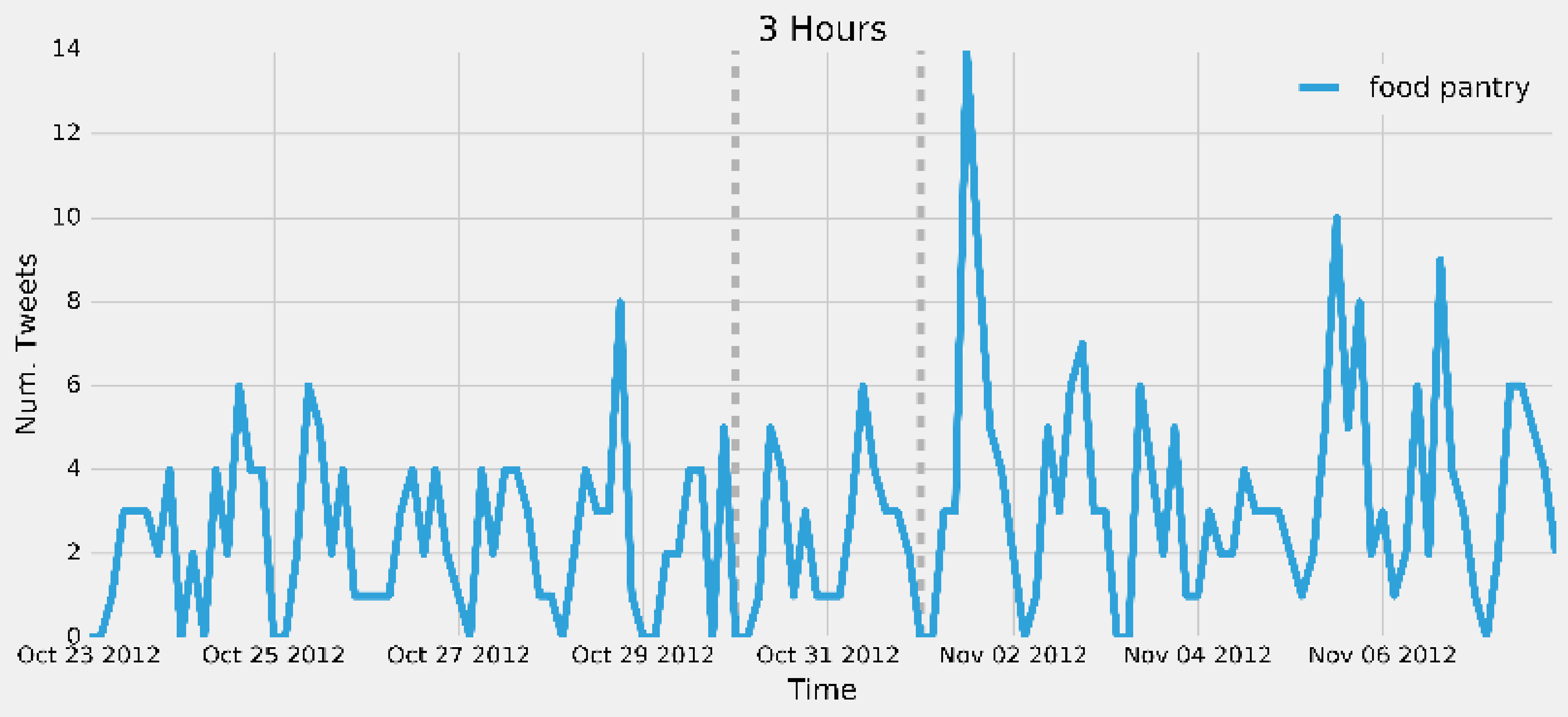
3 Hours
food pantry
Num. Tweets
0
2
4
6
8
10
12
14
Oct 23 2012
Oct 25 2012
Oct 27 2012
Oct 29 2012
Oct 31 2012
Nov 02 2012
Nov 04 2012
Nov 06 2012
Time

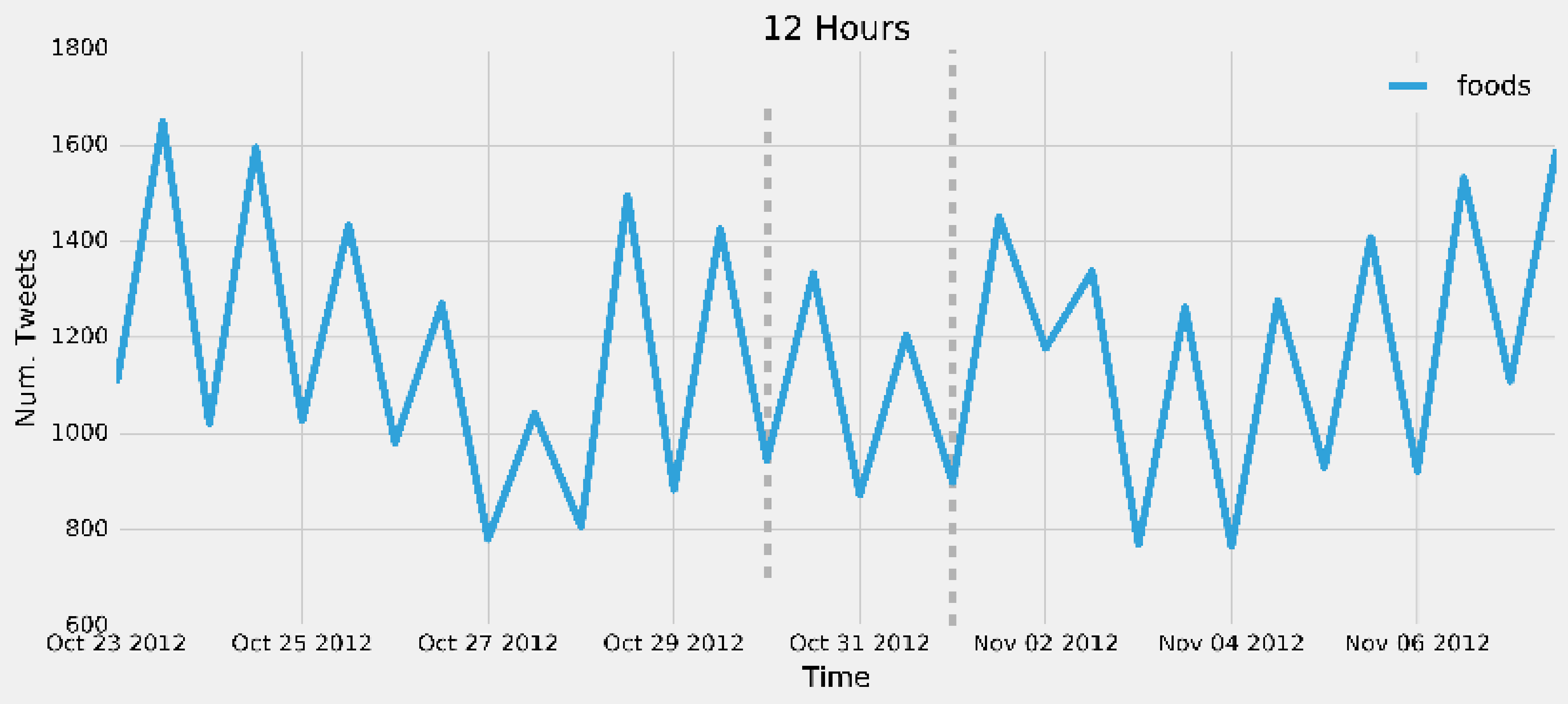
12 Hours
foods
Num. Tweets
1800
1600
1400
1200
1000
800
600
Oct 23 2012
Oct 25 2012
Oct 27 2012
Oct 29 2012
Oct 31 2012
Nov 02 2012
Nov 04 2012
Nov 06 2012
Time

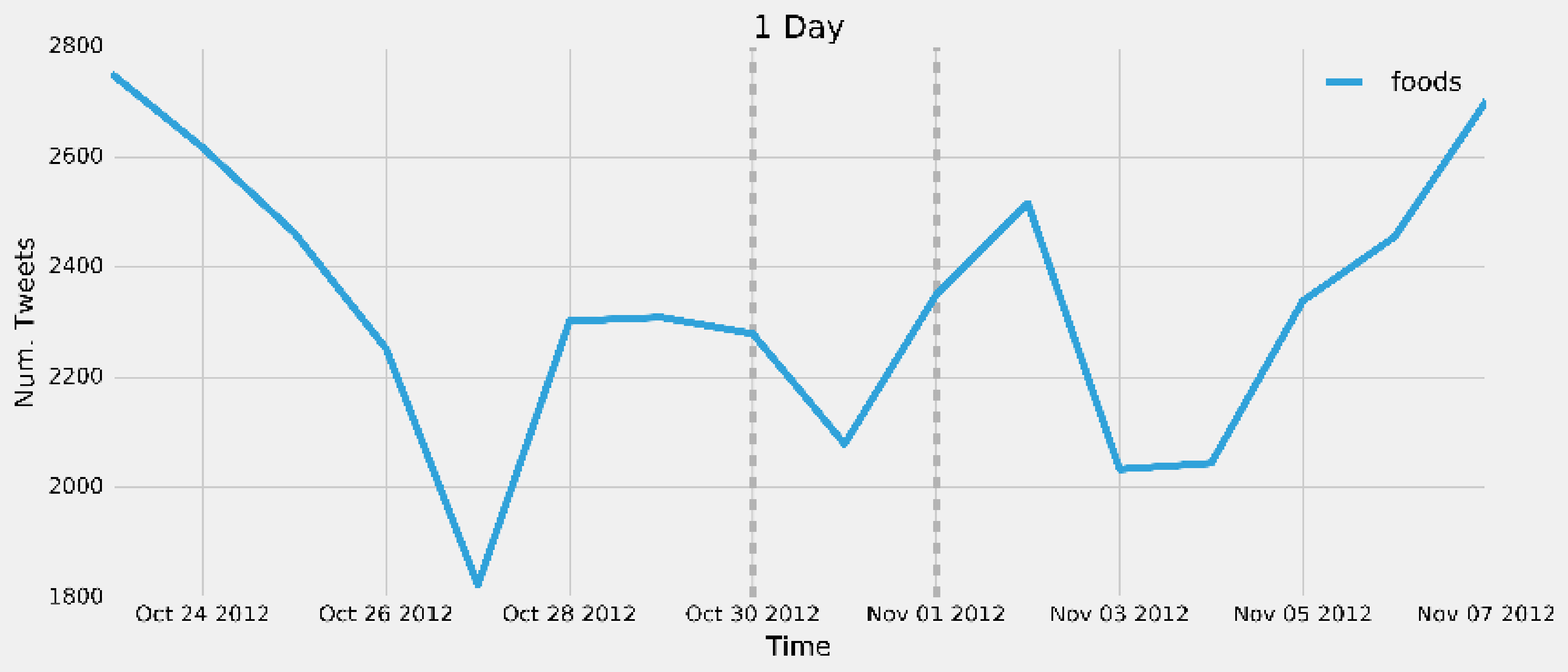
1 Day
Num. Tweets
2800
2600
2400
2200
2000
1800
foods
Oct 24 2012
Oct 26 2012
Oct 28 2012
Oct 30 2012
Nov 01 2012
Nov 03 2012
Nov 05 2012
Nov 07 2012
Time

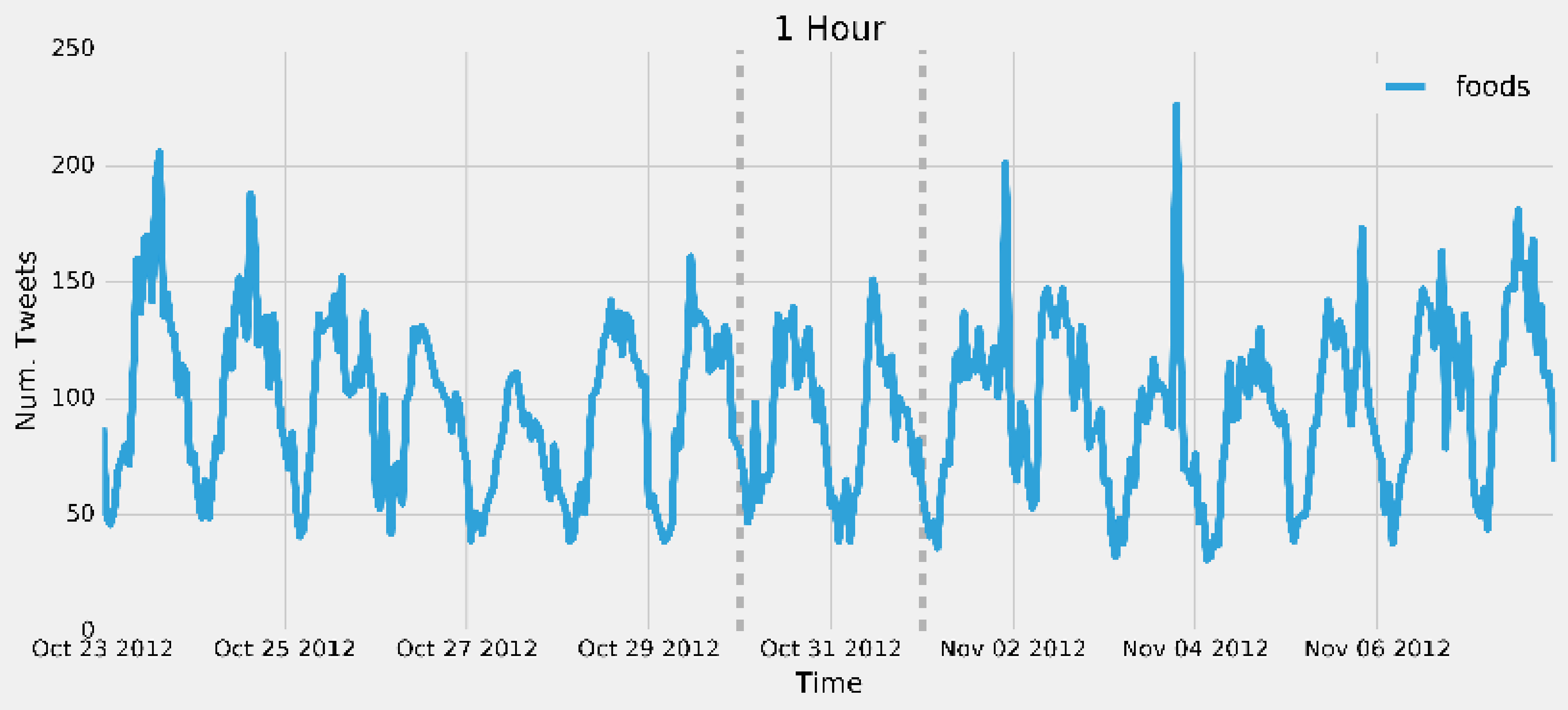

1 Hour
foods
Num. Tweets
Time
250
200
150
100
50
0
Oct 23 2012
Oct 25 2012
Oct 27 2012
Oct 29 2012
Oct 31 2012
Nov 02 2012
Nov 04 2012
Nov 06 2012

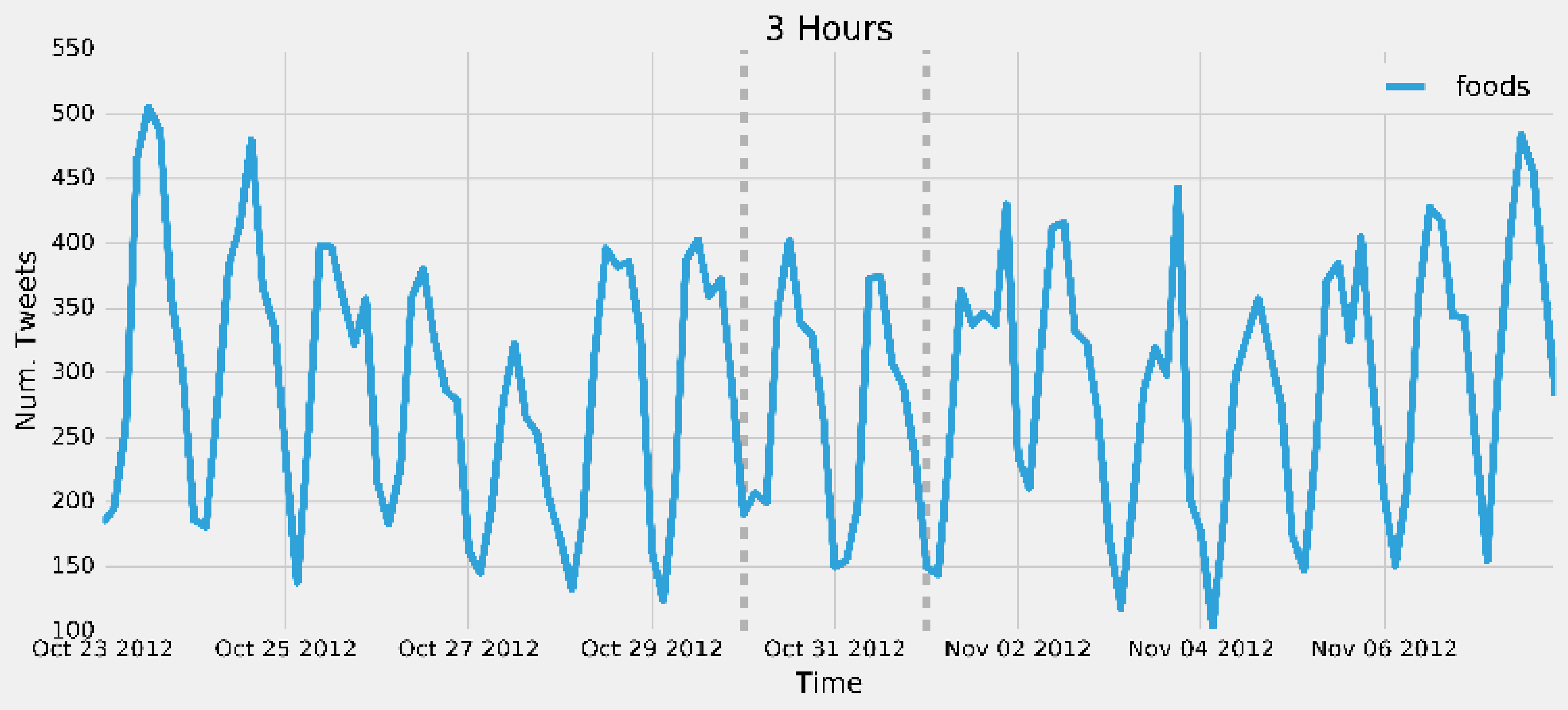
3 Hours
foods
550
500
450
400
350
300
250
200
150
100
Num. Tweets
Oct 23 2012
Oct 25 2012
Oct 27 2012
Oct 29 2012
Oct 31 2012
Nov 02 2012
Nov 04 2012
Nov 06 2012
Time

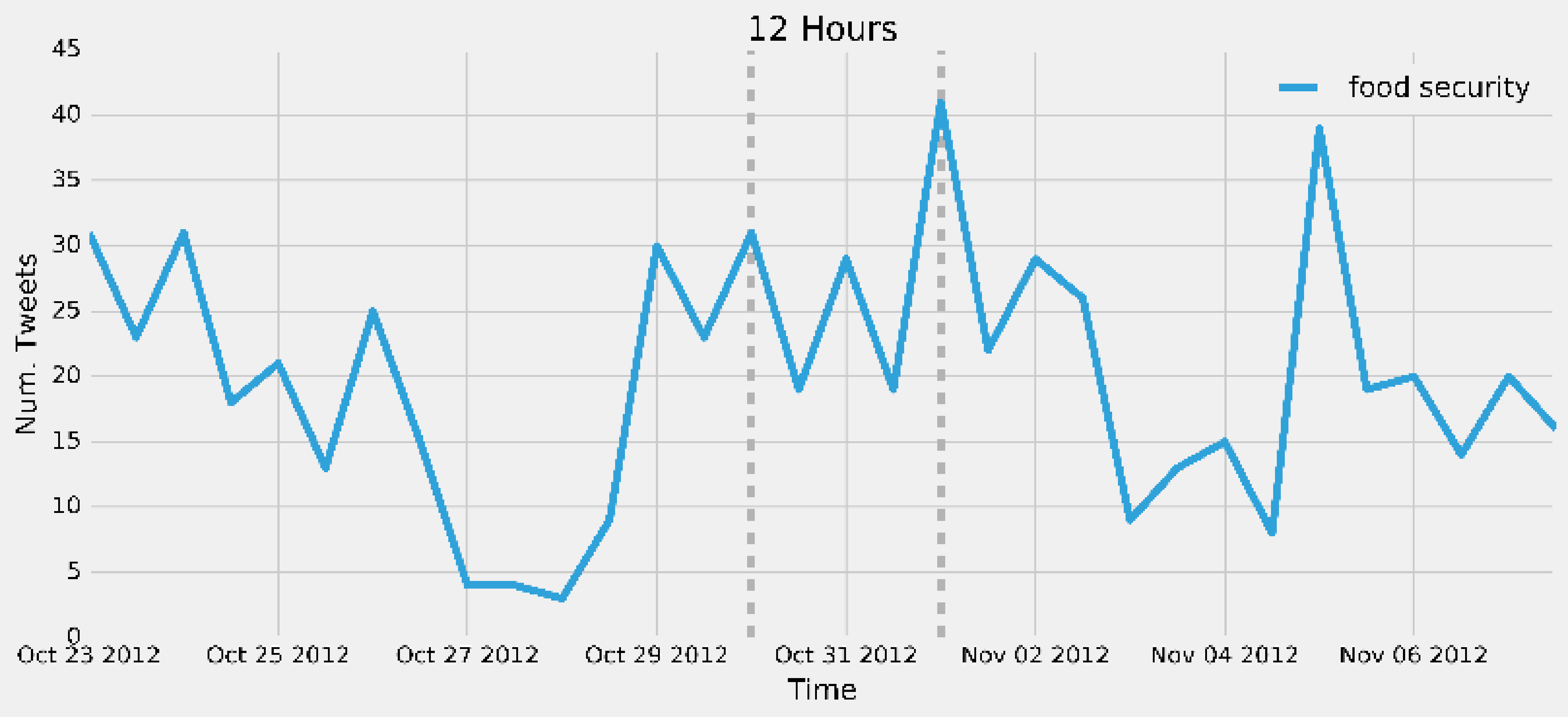
12 Hours
food security
Num. Tweets
0
5
10
15
20
25
30
35
40
45
Oct 23 2012
Oct 25 2012
Oct 27 2012
Oct 29 2012
Oct 31 2012
Nov 02 2012
Nov 04 2012
Nov 06 2012
Time

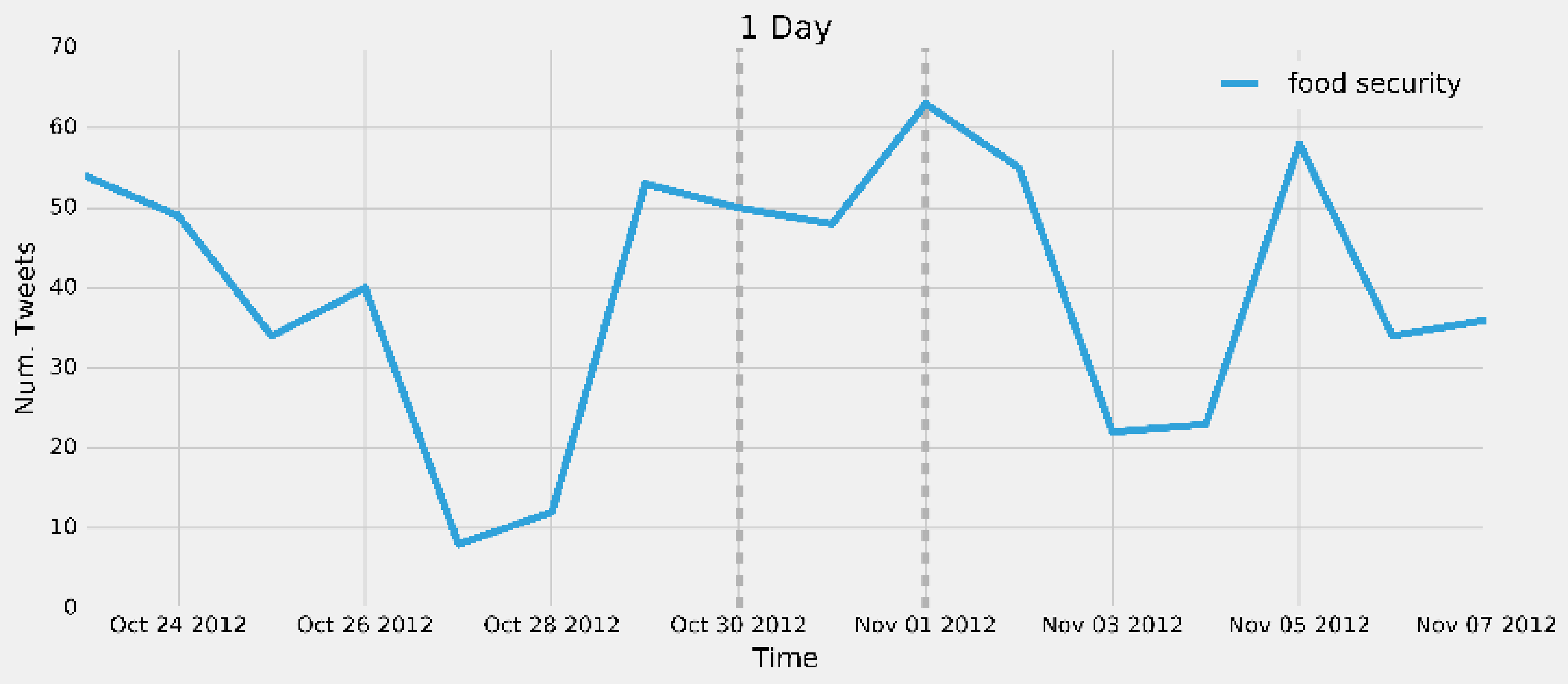
1 Day
food security
Num. Tweets
0
10
20
30
40
50
60
70
Oct 24 2012
Oct 26 2012
Oct 28 2012
Oct 30 2012
Nov 01 2012
Nov 03 2012
Nov 05 2012
Nov 07 2012
Time

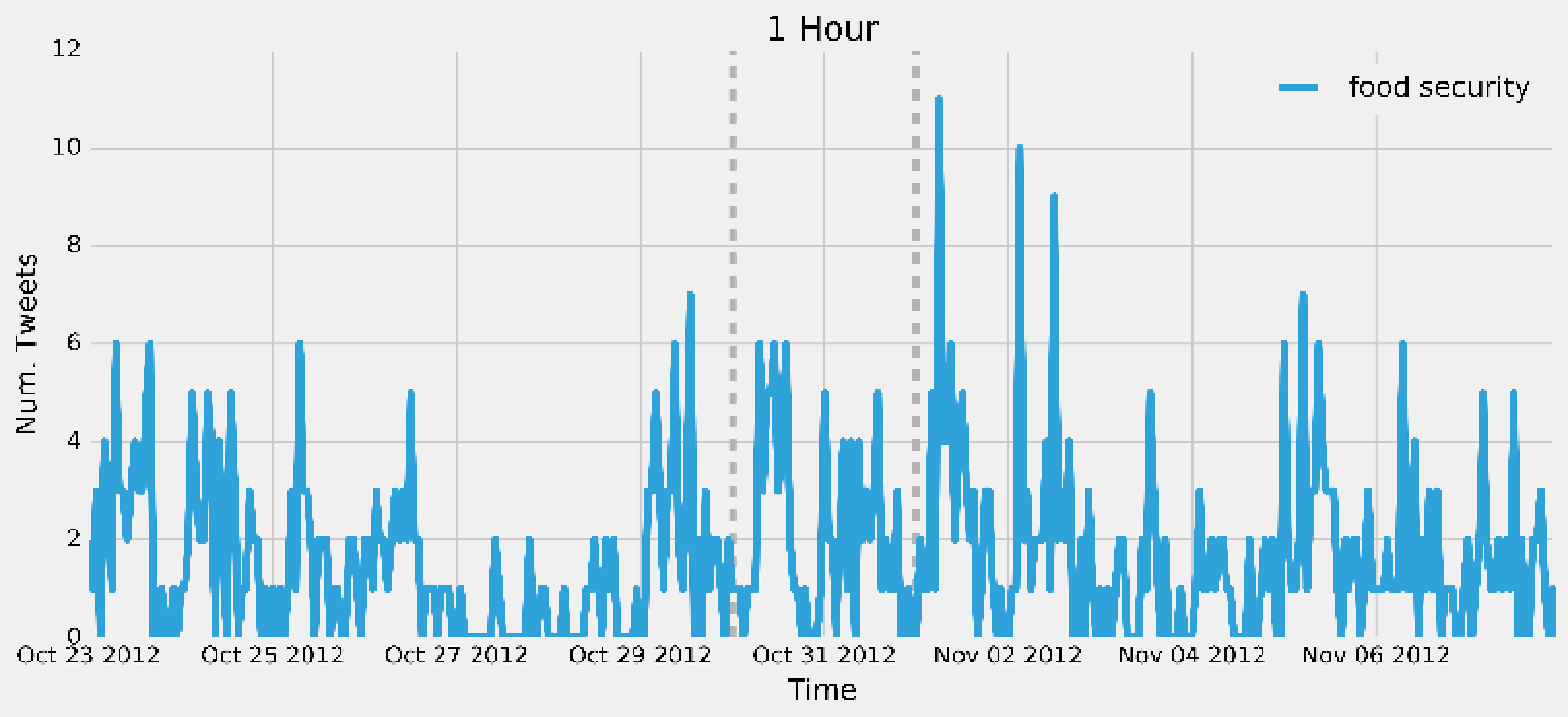
1 Hour
food security
Num. Tweets
0
2
4
6
8
10
12
Oct 23 2012
Oct 25 2012
Oct 27 2012
Oct 29 2012
Oct 31 2012
Nov 02 2012
Nov 04 2012
Nov 06 2012
Time

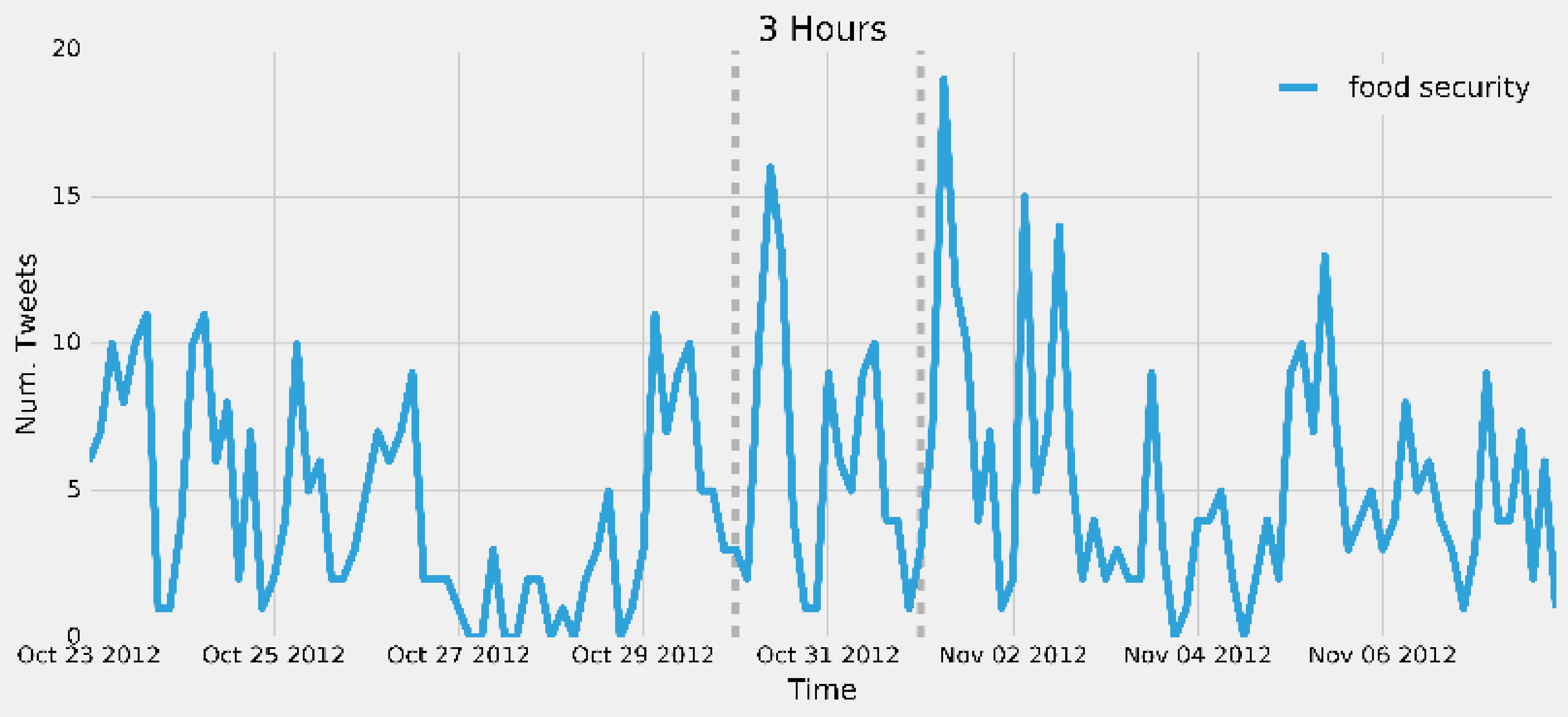
3 Hours
food security
Num. Tweets
Time
0
5
10
15
20
Oct 23 2012
Oct 25 2012
Oct 27 2012
Oct 29 2012
Oct 31 2012
Nov 02 2012
Nov 04 2012
Nov 06 2012

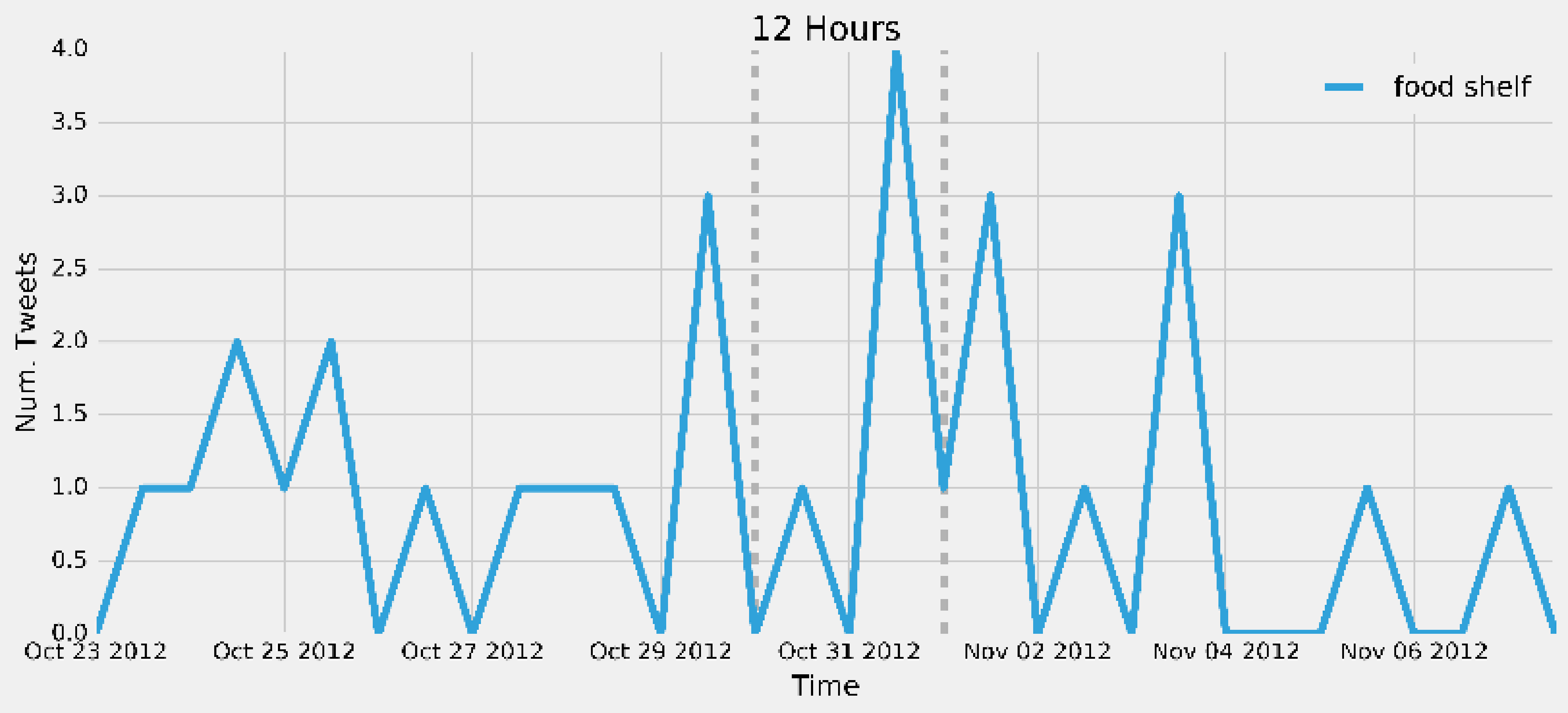

12 Hours
food shelf
Num. Tweets
Time
0.0
0.5
1.0
1.5
2.0
2.5
3.0
3.5
4.0
Oct 23 2012
Oct 25 2012
Oct 27 2012
Oct 29 2012
Oct 31 2012
Nov 02 2012
Nov 04 2012
Nov 06 2012

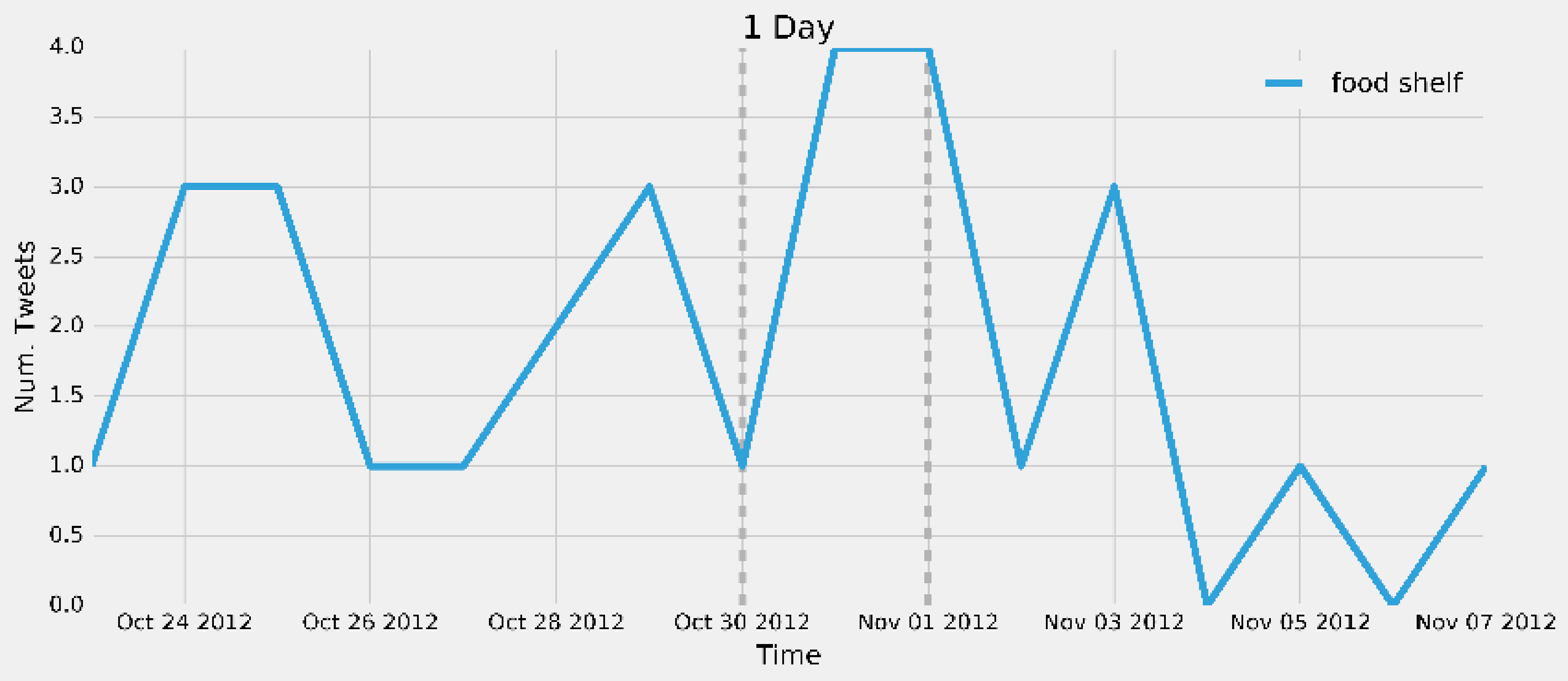
1 Day
food shelf
Num. Tweets
0.0
0.5
1.0
1.5
2.0
2.5
3.0
3.5
4.0
Oct 24 2012
Oct 26 2012
Oct 28 2012
Oct 30 2012
Nov 01 2012
Nov 03 2012
Nov 05 2012
Nov 07 2012
Time

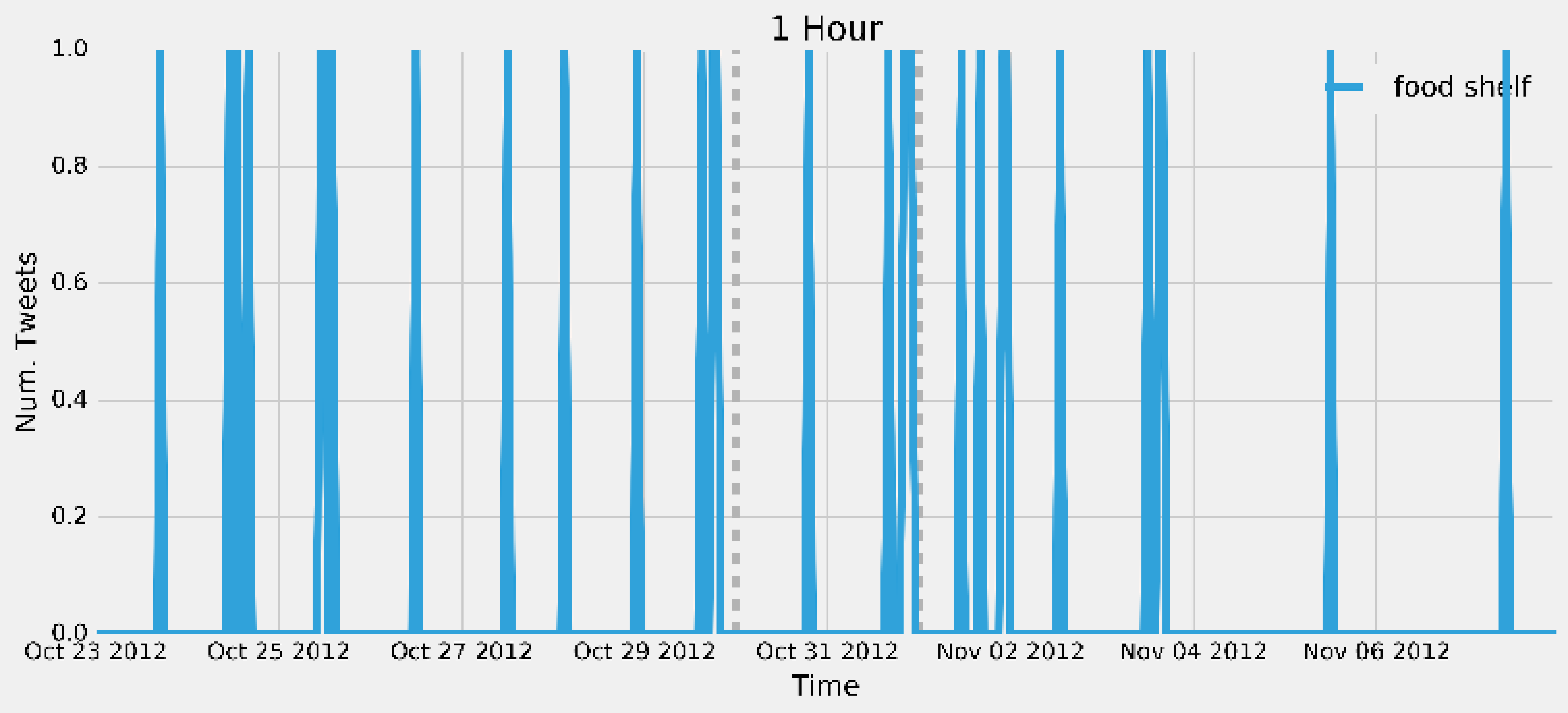
1 Hour
food shelf
Num. Tweets
1.0
0.8
0.6
0.4
0.2
0.0
Oct 23 2012
Oct 25 2012
Oct 27 2012
Oct 29 2012
Oct 31 2012
Nov 02 2012
Nov 04 2012
Nov 06 2012
Time

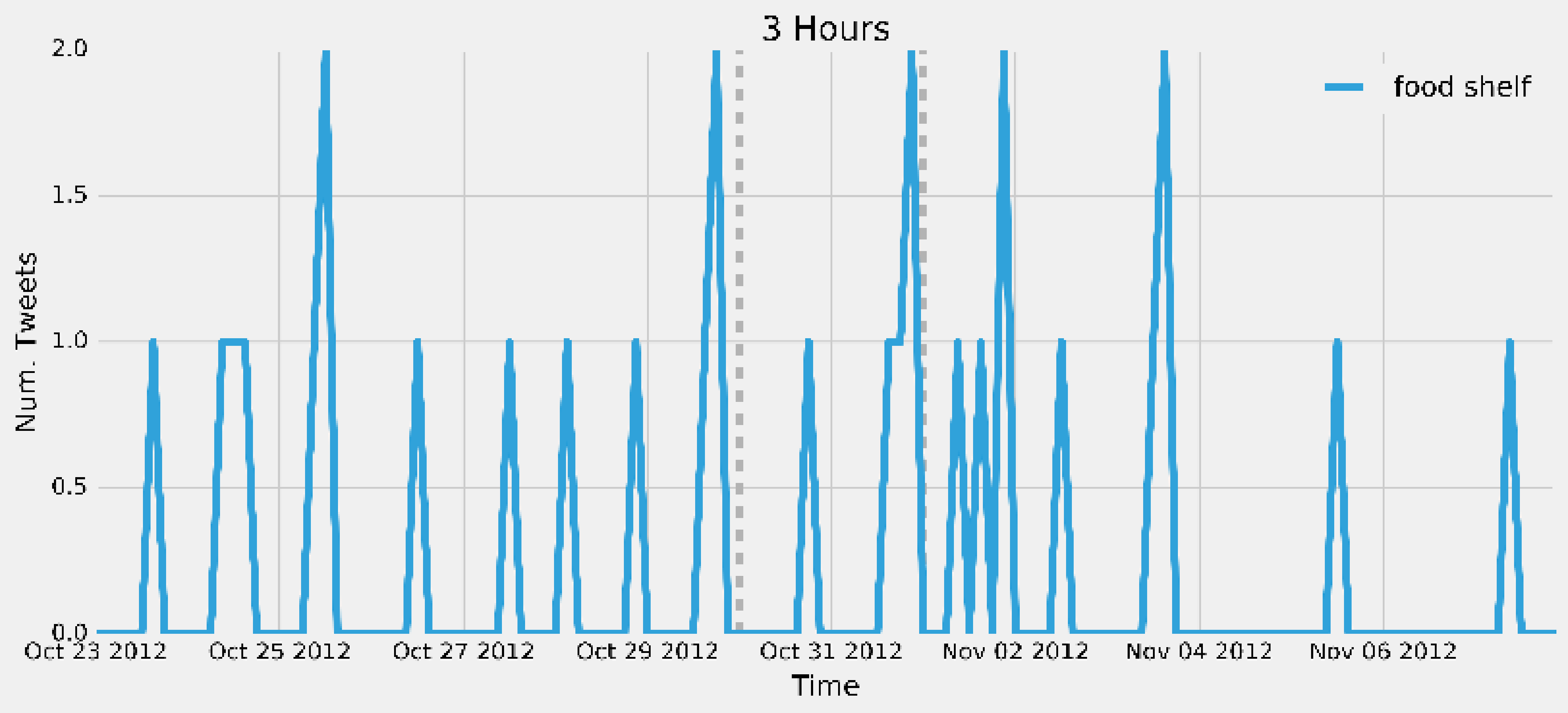

3 Hours
food shelf
Num. Tweets
Time
0.0
0.5
1.0
1.5
2.0
Oct 23 2012
Oct 25 2012
Oct 27 2012
Oct 29 2012
Oct 31 2012
Nov 02 2012
Nov 04 2012
Nov 06 2012

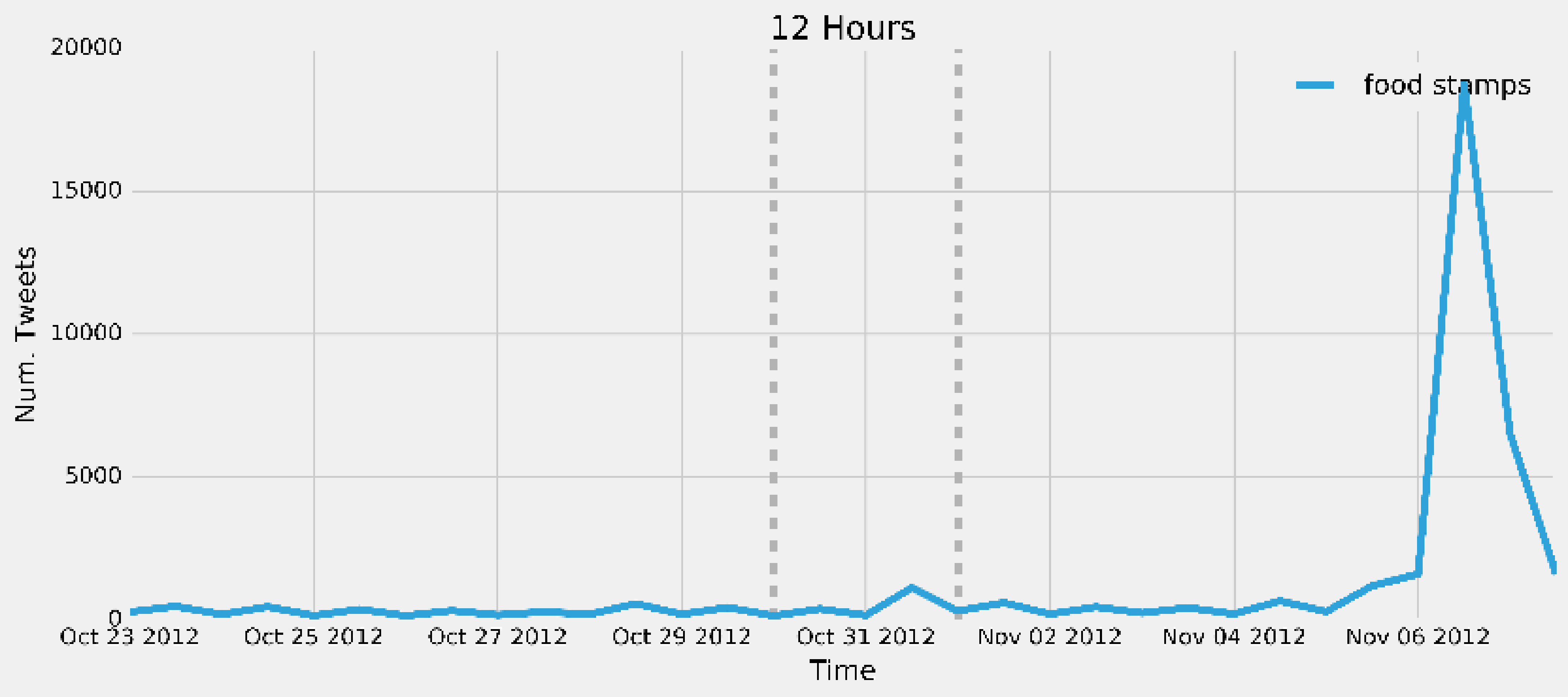
12 Hours
20000
15000
10000
5000
0
Num. Tweets
Oct 23 2012
Oct 25 2012
Oct 27 2012
Oct 29 2012
Oct 31 2012
Nov 02 2012
Nov 04 2012
Nov 06 2012
Time
food stamps

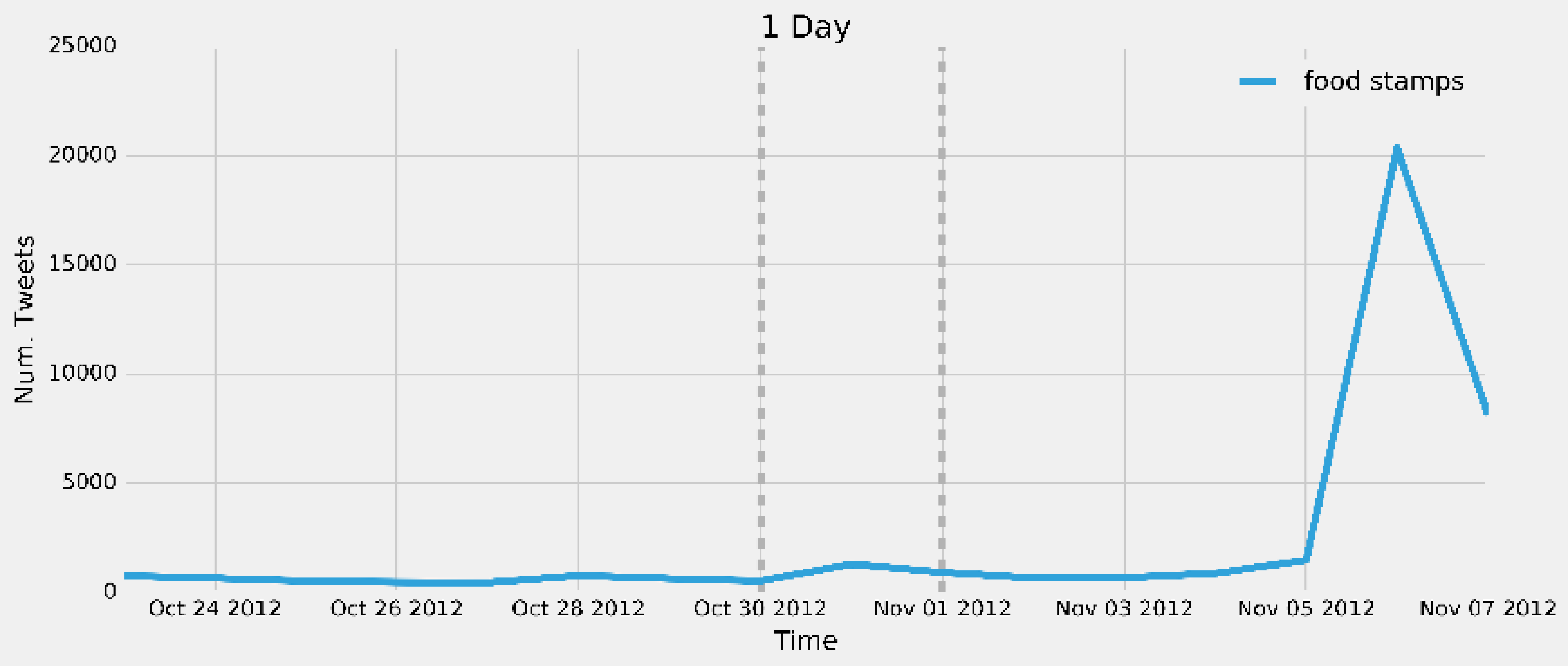
1 Day
food stamps
25000
20000
15000
10000
5000
0
Num. Tweets
Oct 24 2012
Oct 26 2012
Oct 28 2012
Oct 30 2012
Nov 01 2012
Nov 03 2012
Nov 05 2012
Nov 07 2012
Time

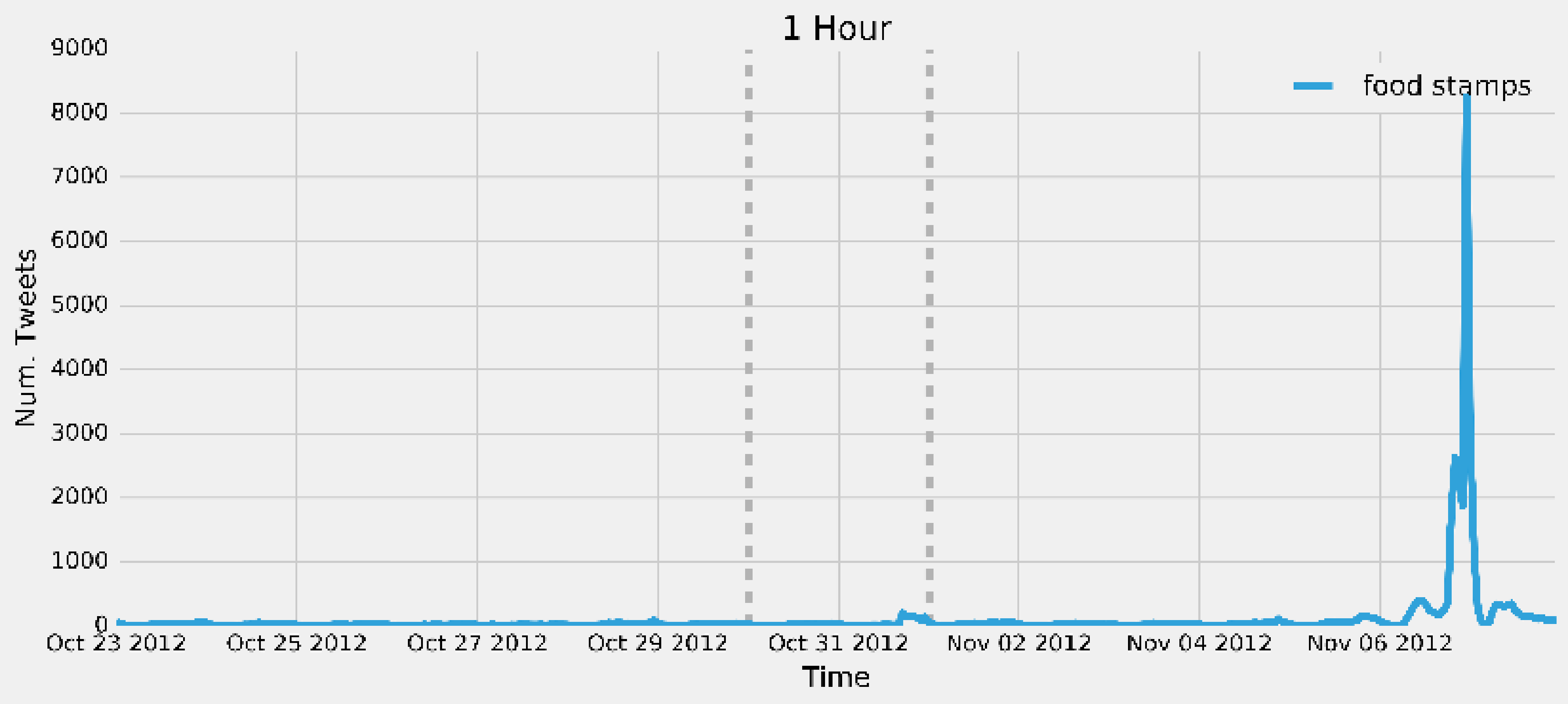

1 Hour
food stamps
Num. Tweets
Time
0
1000
2000
3000
4000
5000
6000
7000
8000
9000
Oct 23 2012
Oct 25 2012
Oct 27 2012
Oct 29 2012
Oct 31 2012
Nov 02 2012
Nov 04 2012
Nov 06 2012

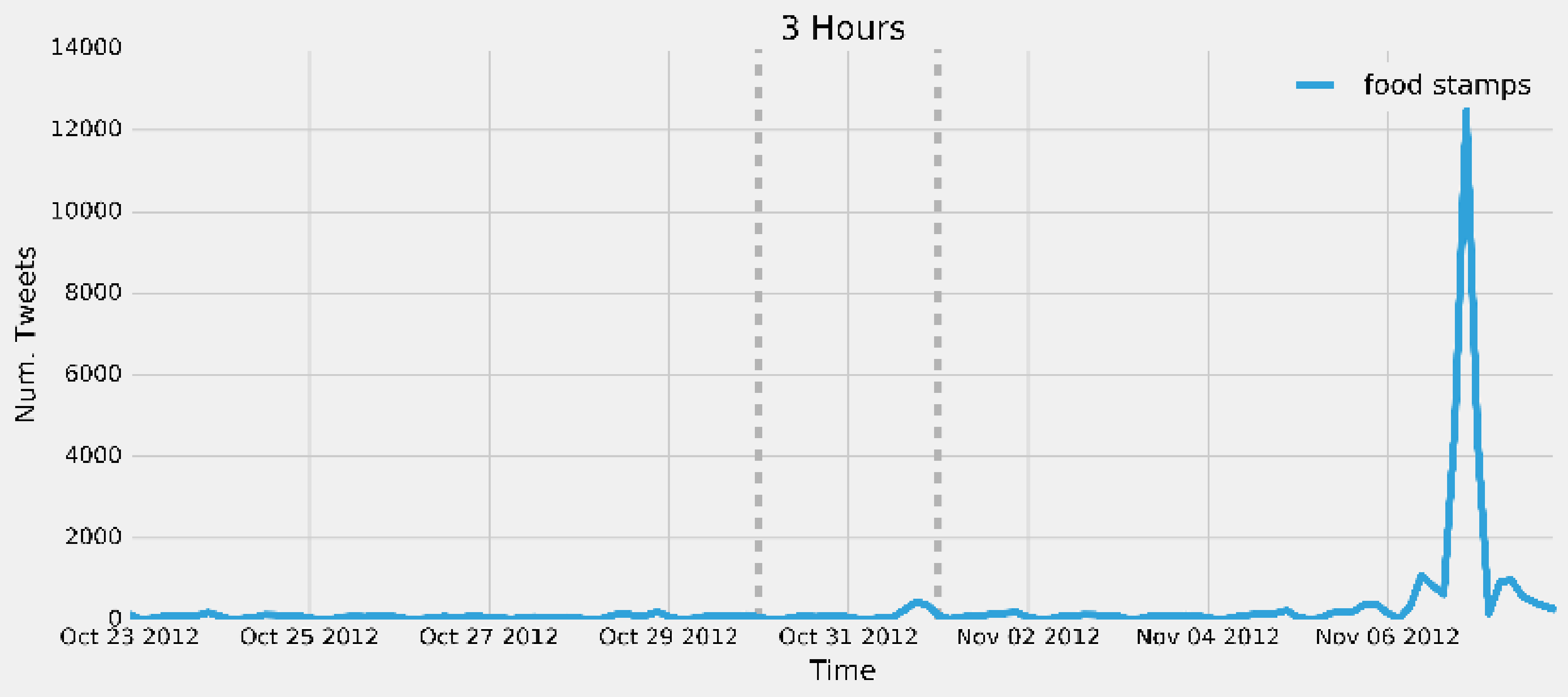

3 Hours
food stamps
Num. Tweets
0
2000
4000
6000
8000
10000
12000
14000
Oct 23 2012
Oct 25 2012
Oct 27 2012
Oct 29 2012
Oct 31 2012
Nov 02 2012
Nov 04 2012
Nov 06 2012
Time

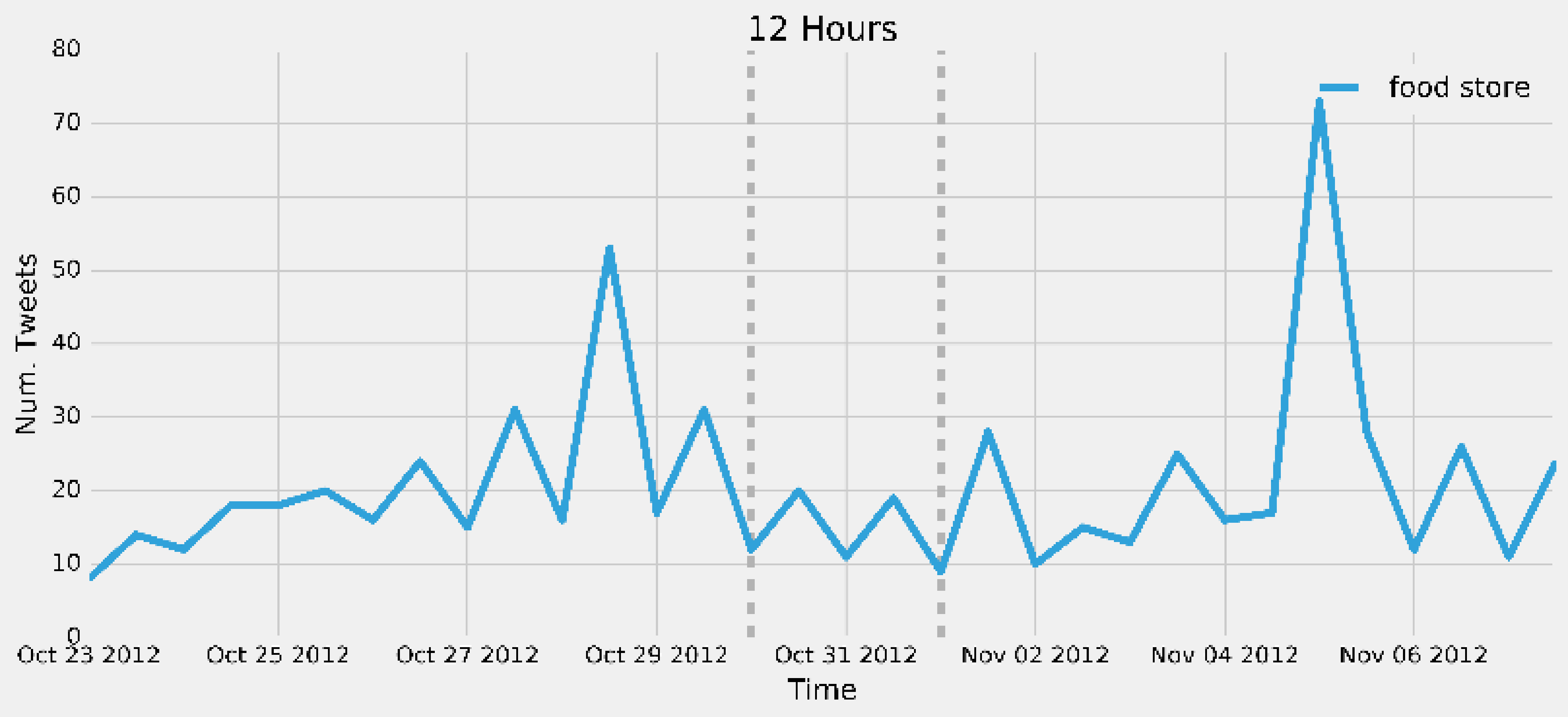
12 Hours
food store
Num. Tweets
Time
0
10
20
30
40
50
60
70
80
Oct 23 2012
Oct 25 2012
Oct 27 2012
Oct 29 2012
Oct 31 2012
Nov 02 2012
Nov 04 2012
Nov 06 2012

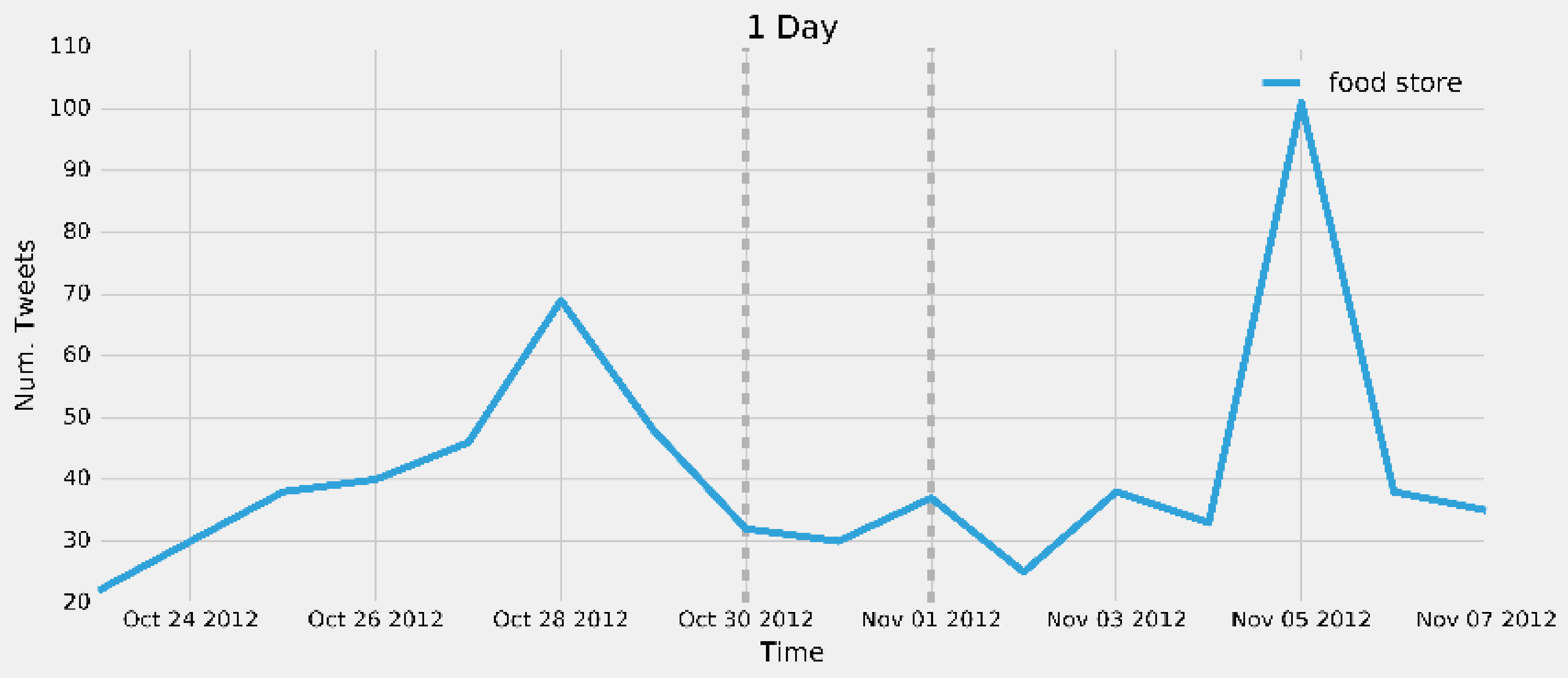

1 Day
food store
Num. Tweets
110
100
90
80
70
60
50
40
30
20
Oct 24 2012
Oct 26 2012
Oct 28 2012
Oct 30 2012
Nov 01 2012
Nov 03 2012
Nov 05 2012
Nov 07 2012
Time

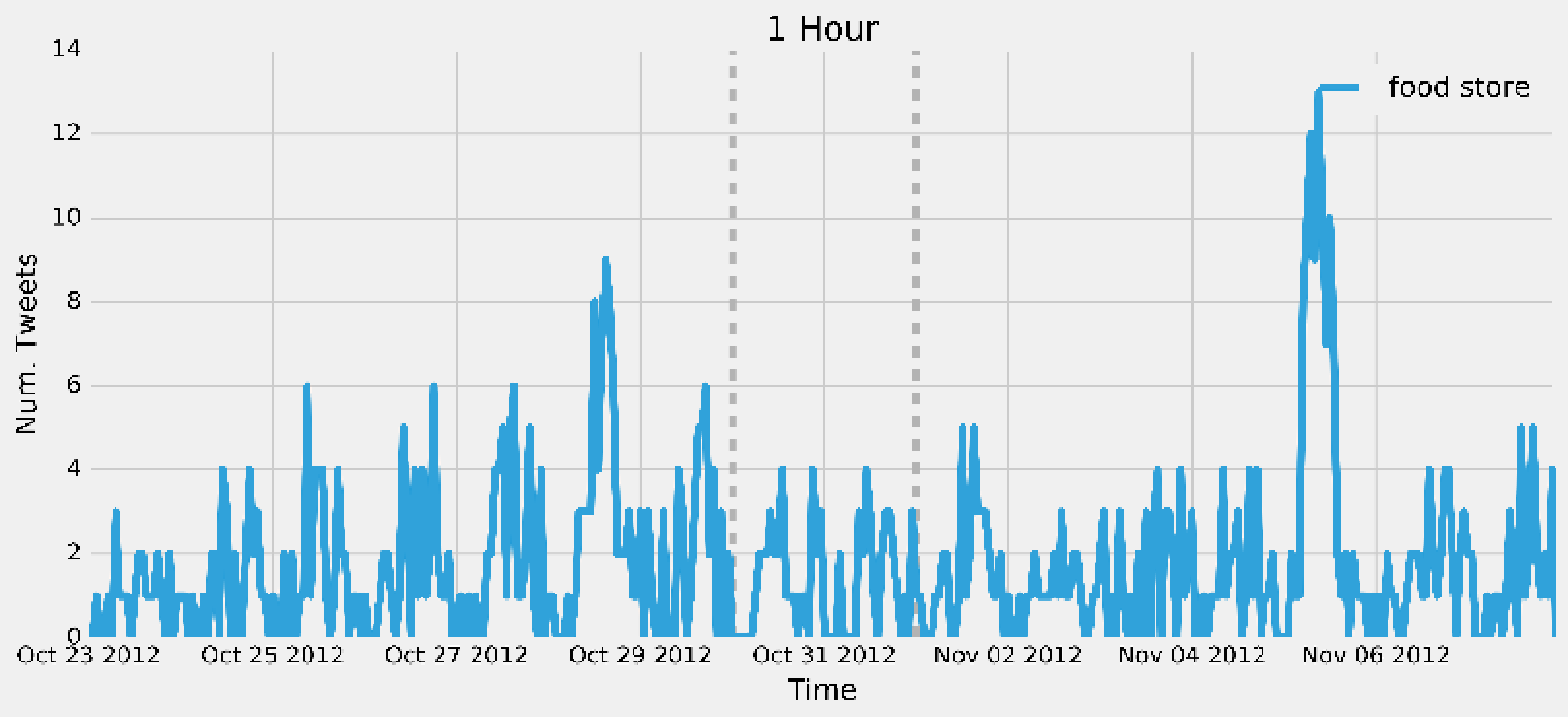
1 Hour
food store
Num. Tweets
Time
0
2
4
6
8
10
12
14
Oct 23 2012
Oct 25 2012
Oct 27 2012
Oct 29 2012
Oct 31 2012
Nov 02 2012
Nov 04 2012
Nov 06 2012

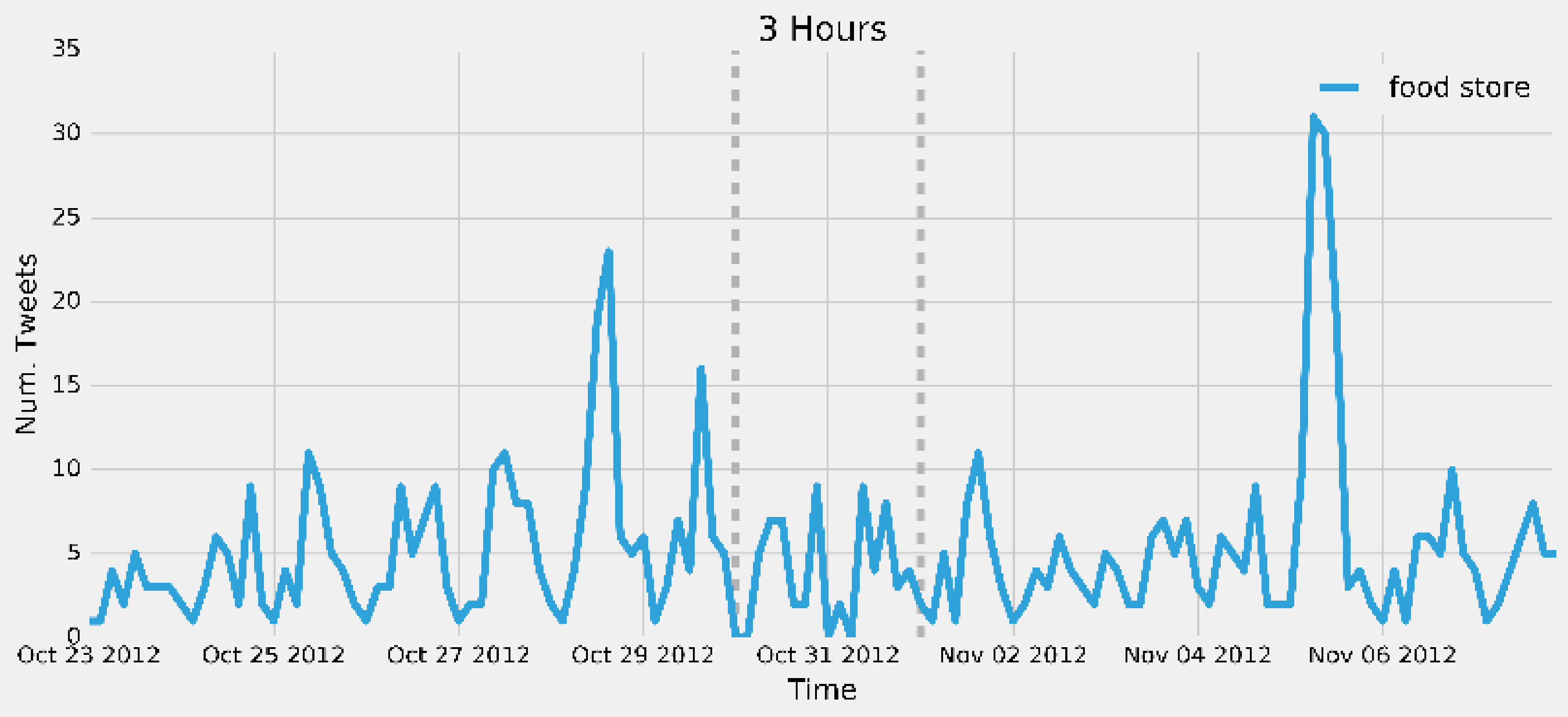
3 Hours
food store
Num. Tweets
Time
0
5
10
15
20
25
30
35
Oct 23 2012
Oct 25 2012
Oct 27 2012
Oct 29 2012
Oct 31 2012
Nov 02 2012
Nov 04 2012
Nov 06 2012

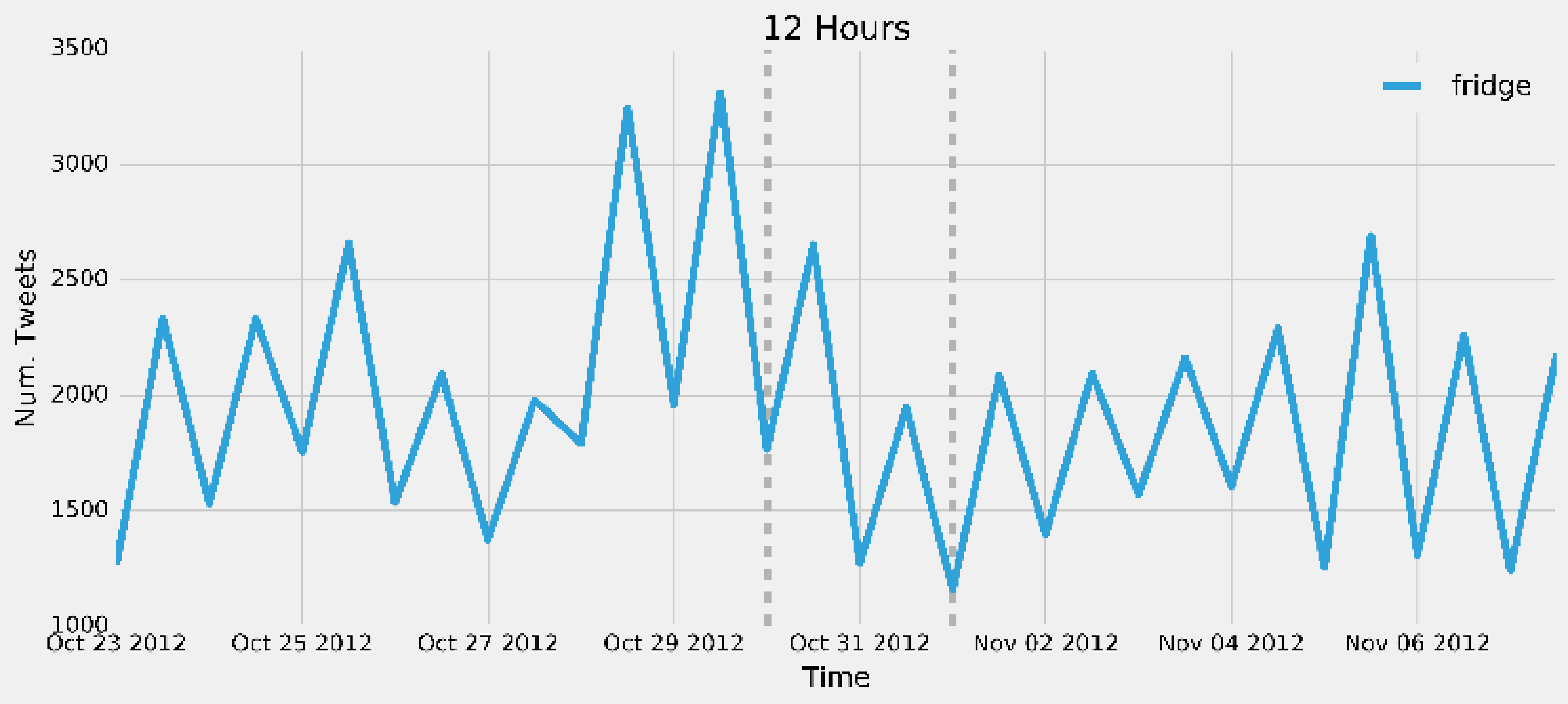
12 Hours
fridge
Num. Tweets
3500
3000
2500
2000
1500
1000
Oct 23 2012
Oct 25 2012
Oct 27 2012
Oct 29 2012
Oct 31 2012
Nov 02 2012
Nov 04 2012
Nov 06 2012
Time

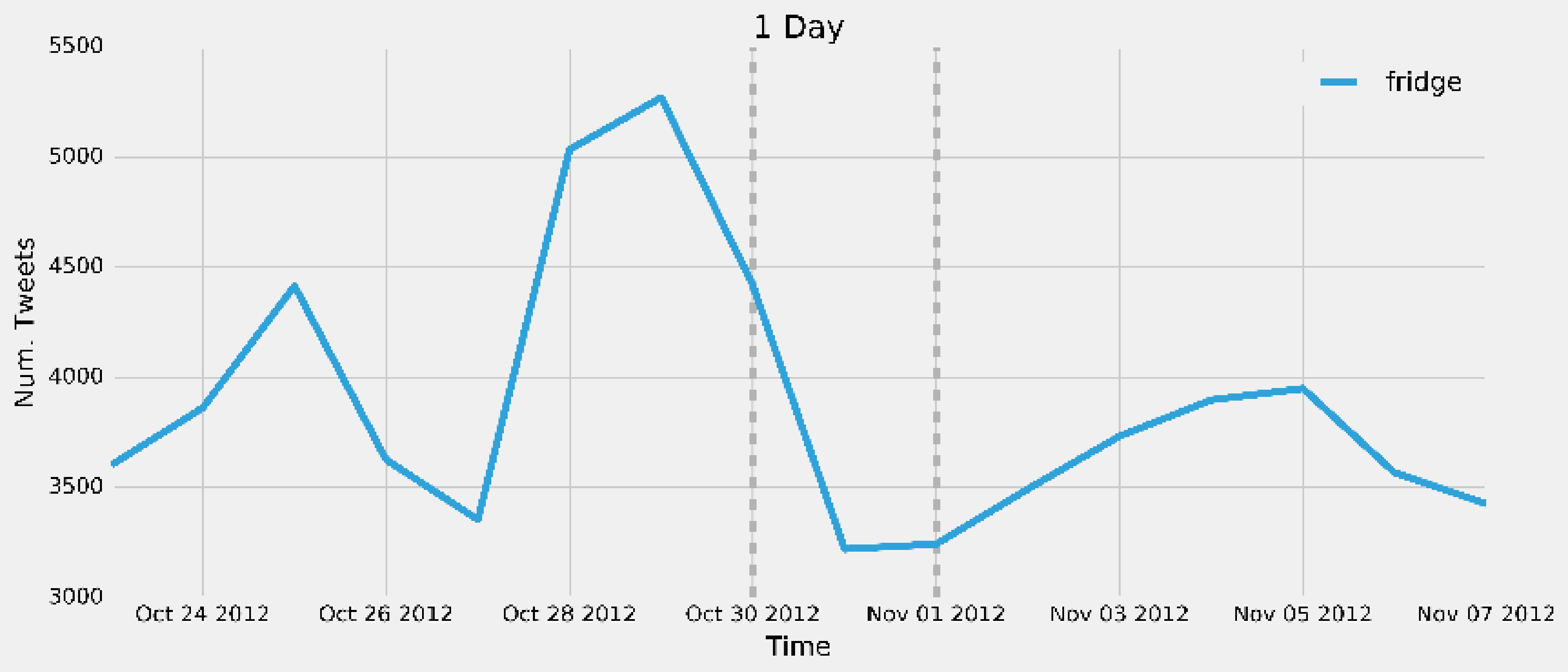
1 Day
fridge
Num. Tweets
5500
5000
4500
4000
3500
3000
Oct 24 2012
Oct 26 2012
Oct 28 2012
Oct 30 2012
Nov 01 2012
Nov 03 2012
Nov 05 2012
Nov 07 2012
Time

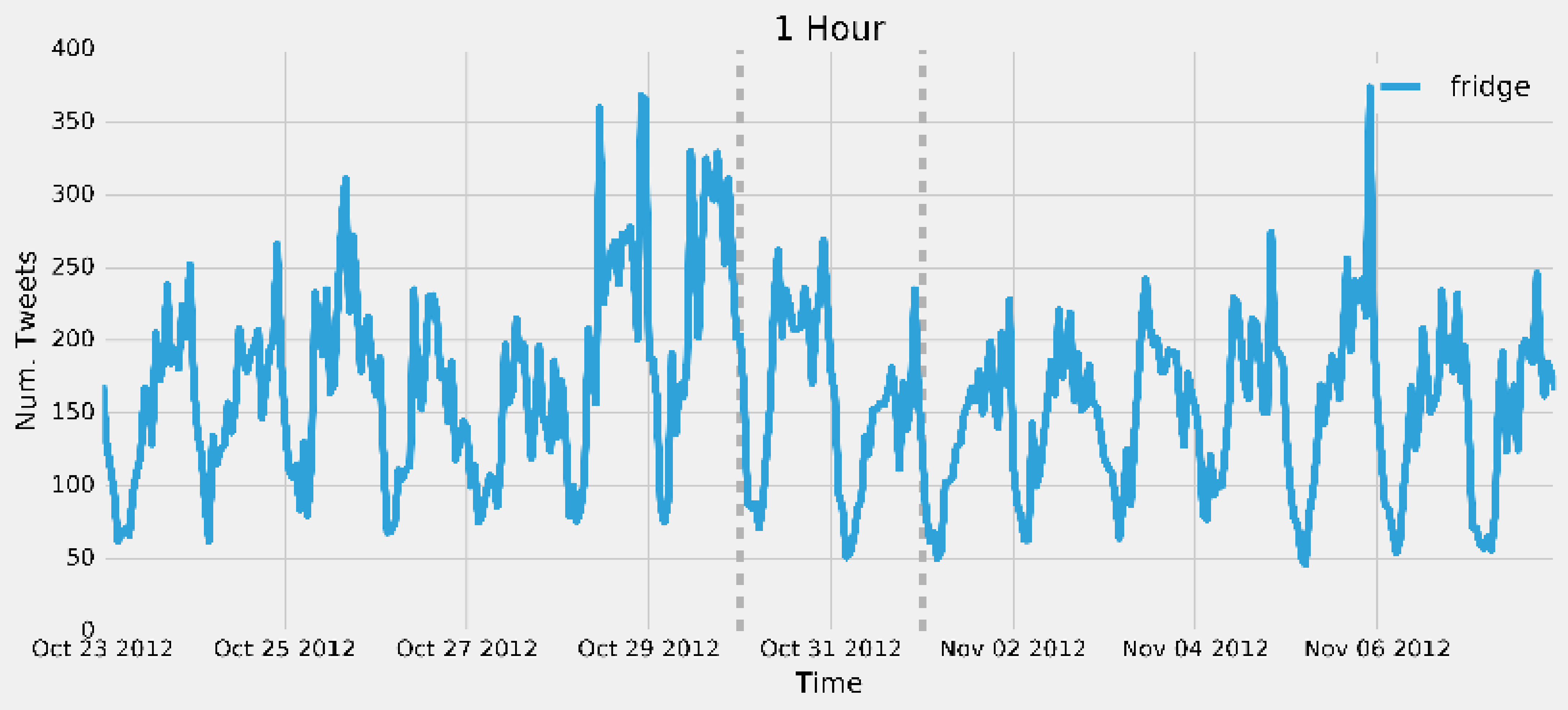
1 Hour
fridge
Num. Tweets
0
50
100
150
200
250
300
350
400
Oct 23 2012
Oct 25 2012
Oct 27 2012
Oct 29 2012
Oct 31 2012
Nov 02 2012
Nov 04 2012
Nov 06 2012
Time

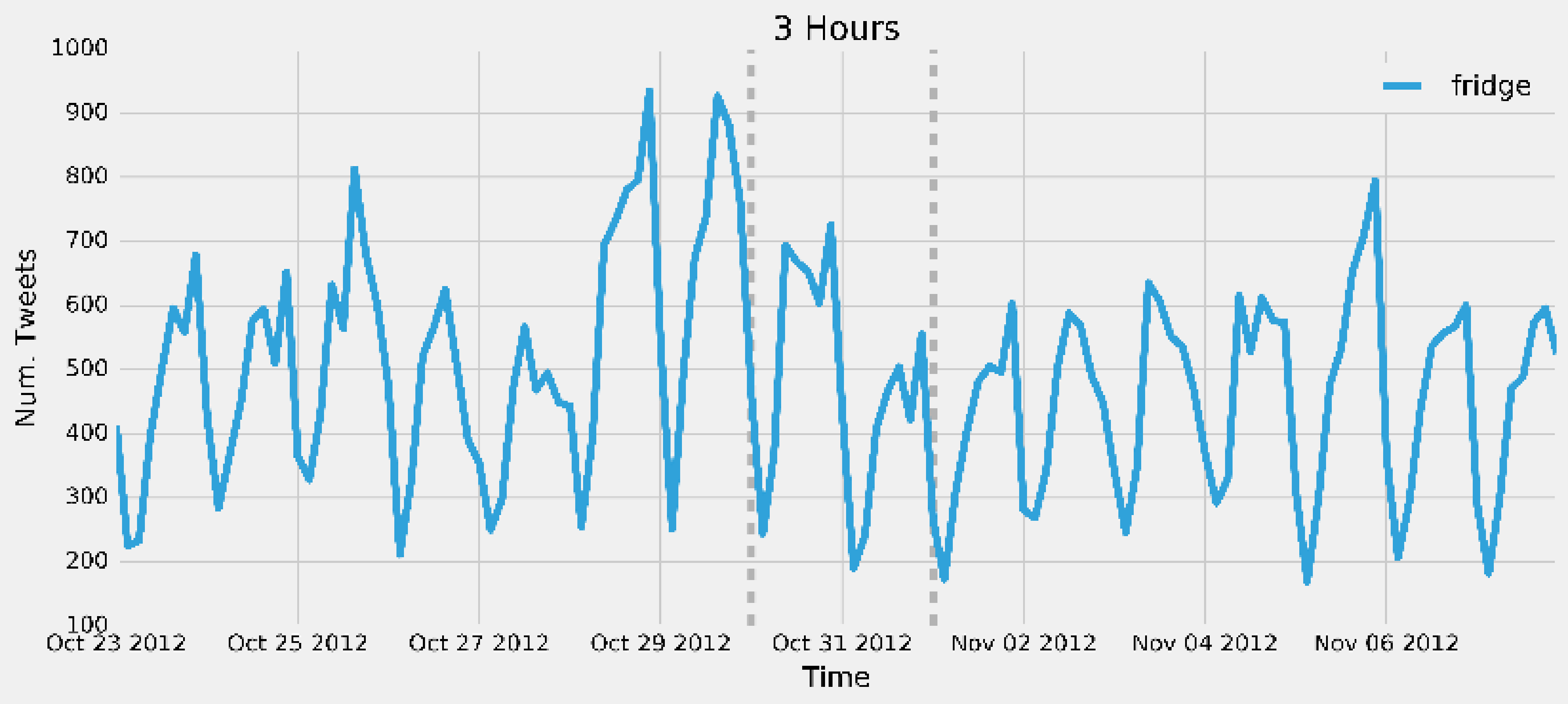
3 Hours
fridge
Num. Tweets
1000
900
800
700
600
500
400
300
200
100
Oct 23 2012
Oct 25 2012
Oct 27 2012
Oct 29 2012
Oct 31 2012
Nov 02 2012
Nov 04 2012
Nov 06 2012
Time

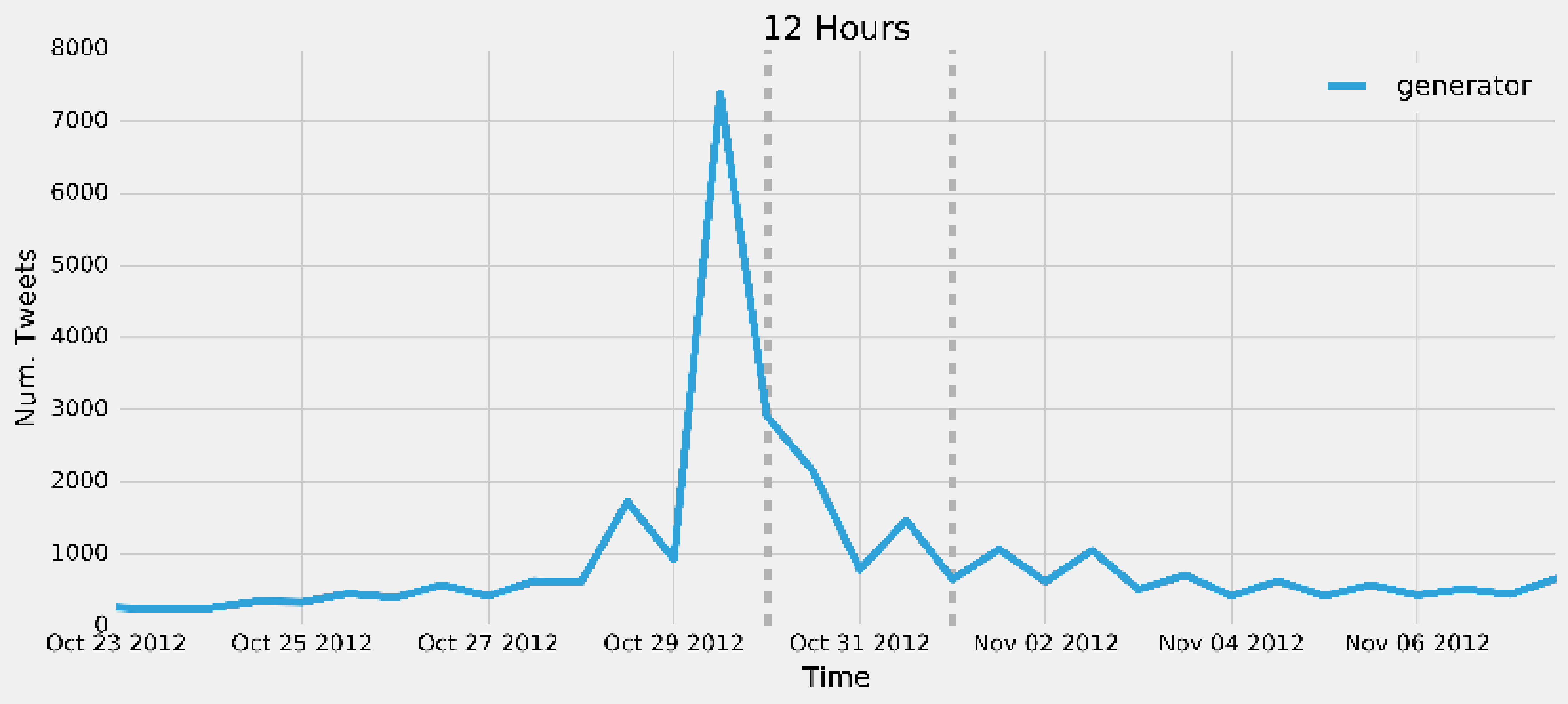
12 Hours
generator
Num. Tweets
0
1000
2000
3000
4000
5000
6000
7000
8000
Oct 23 2012
Oct 25 2012
Oct 27 2012
Oct 29 2012
Oct 31 2012
Nov 02 2012
Nov 04 2012
Nov 06 2012
Time

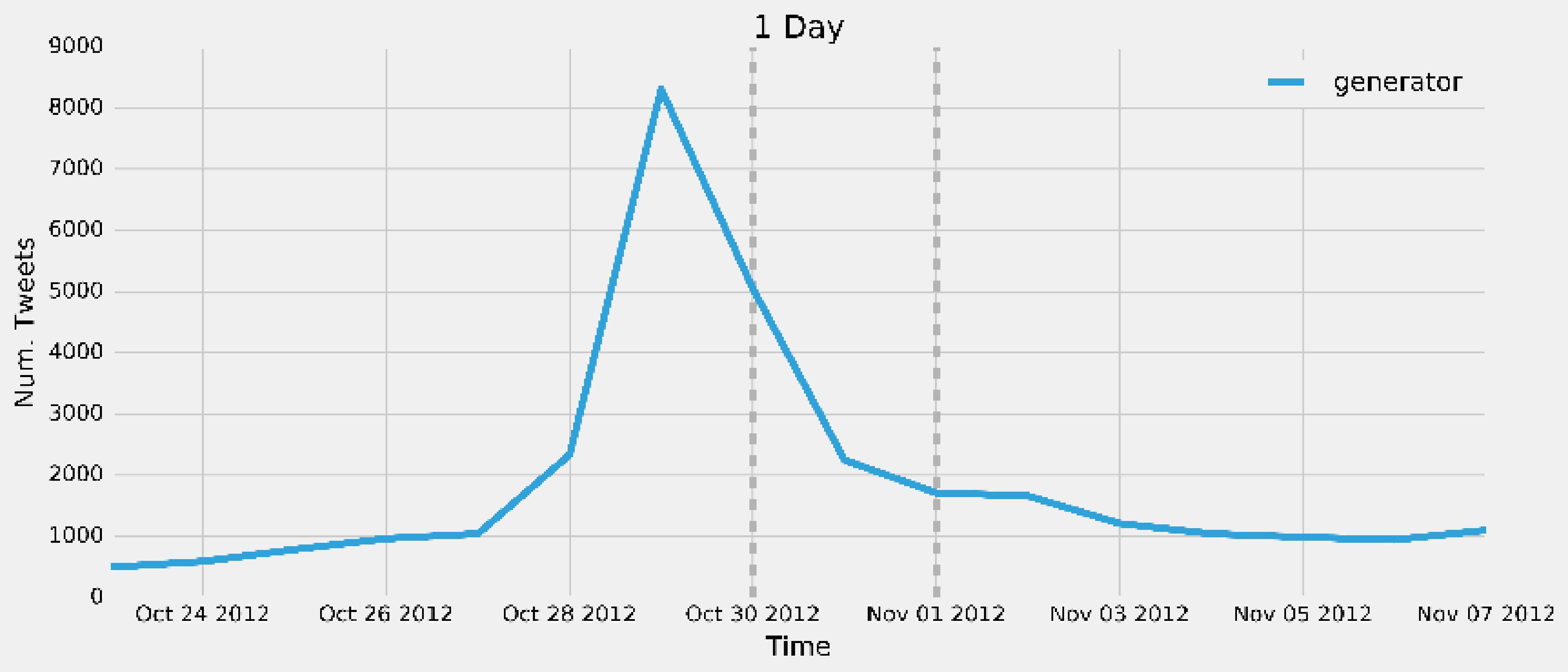

1 Day
9000
8000
7000
6000
5000
4000
3000
2000
1000
0
Num. Tweets
generator
Oct 24 2012
Oct 26 2012
Oct 28 2012
Oct 30 2012
Nov 01 2012
Nov 03 2012
Nov 05 2012
Nov 07 2012
Time

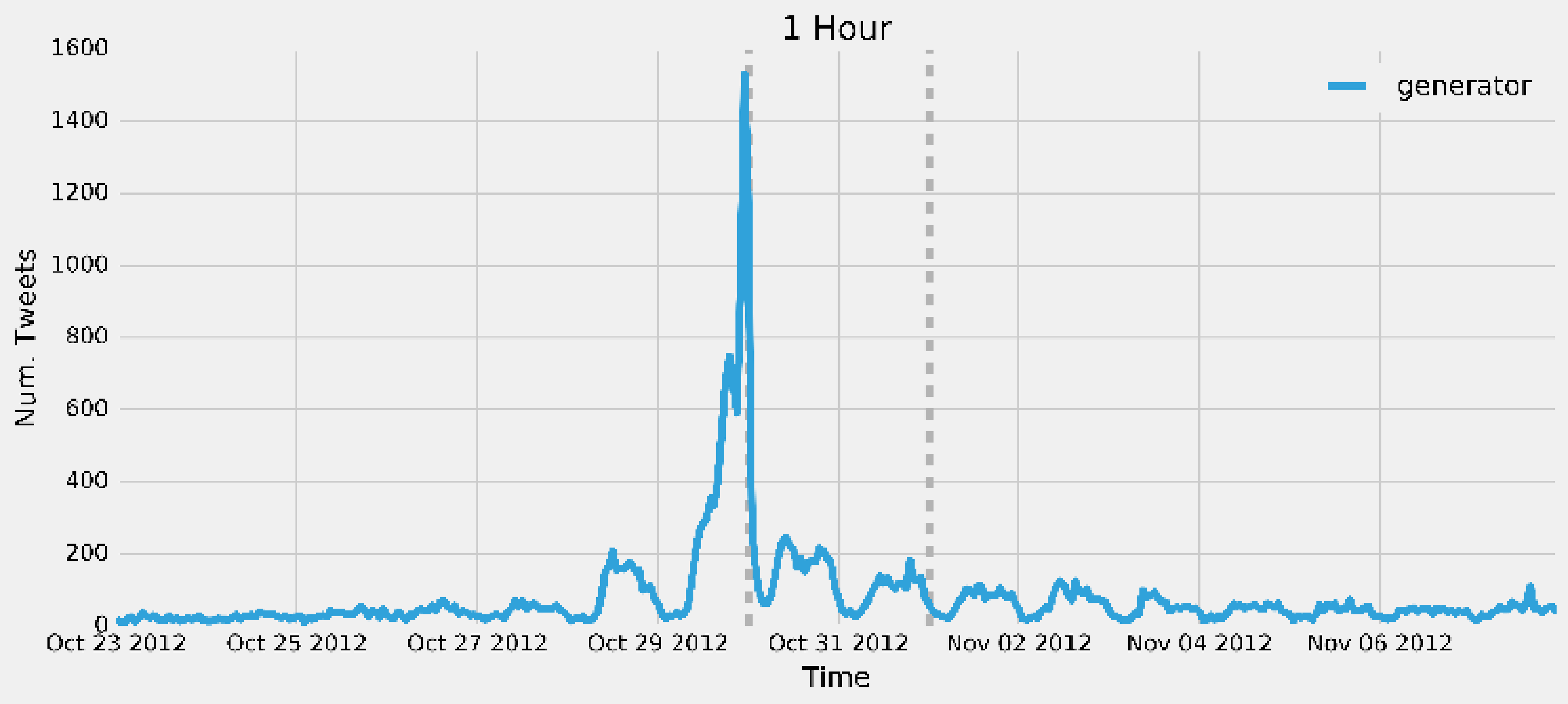

1 Hour
1600
1400
1200
1000
800
600
400
200
0
Num. Tweets
Oct 23 2012
Oct 25 2012
Oct 27 2012
Oct 29 2012
Oct 31 2012
Nov 02 2012
Nov 04 2012
Nov 06 2012
Time
generator

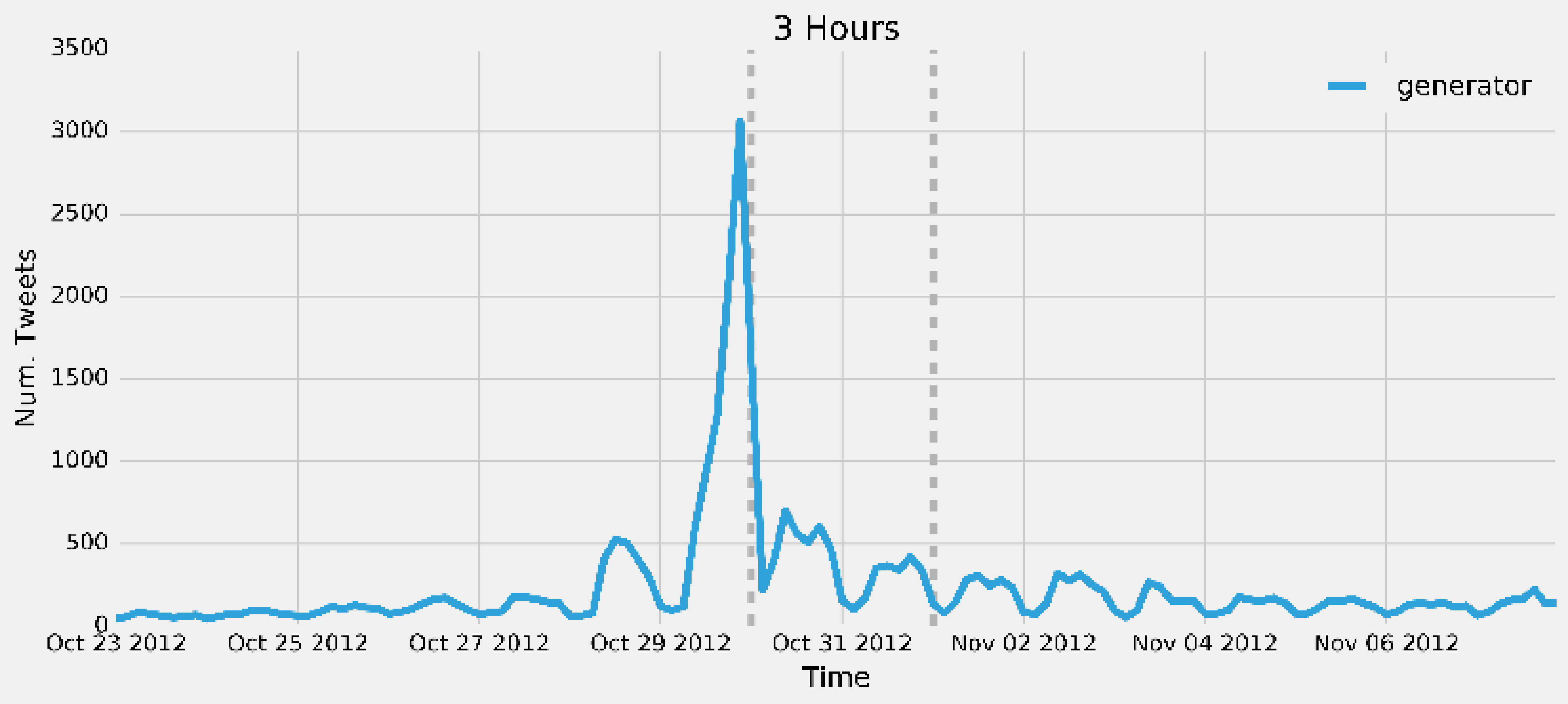

3 Hours
generator
Num. Tweets
3500
3000
2500
2000
1500
1000
500
0
Oct 23 2012
Oct 25 2012
Oct 27 2012
Oct 29 2012
Oct 31 2012
Nov 02 2012
Nov 04 2012
Nov 06 2012
Time

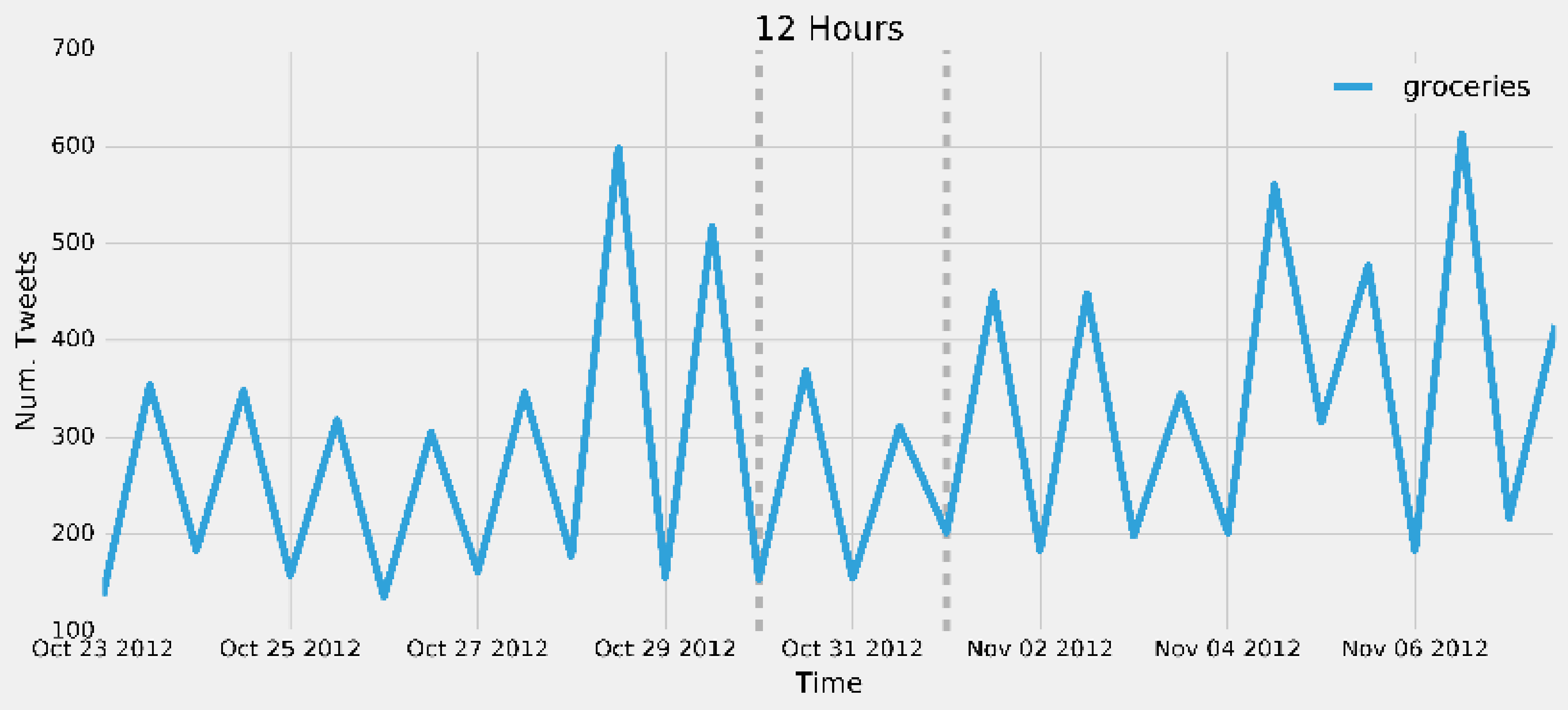

12 Hours
groceries
Num. Tweets
Time
700
600
500
400
300
200
100
Oct 23 2012
Oct 25 2012
Oct 27 2012
Oct 29 2012
Oct 31 2012
Nov 02 2012
Nov 04 2012
Nov 06 2012

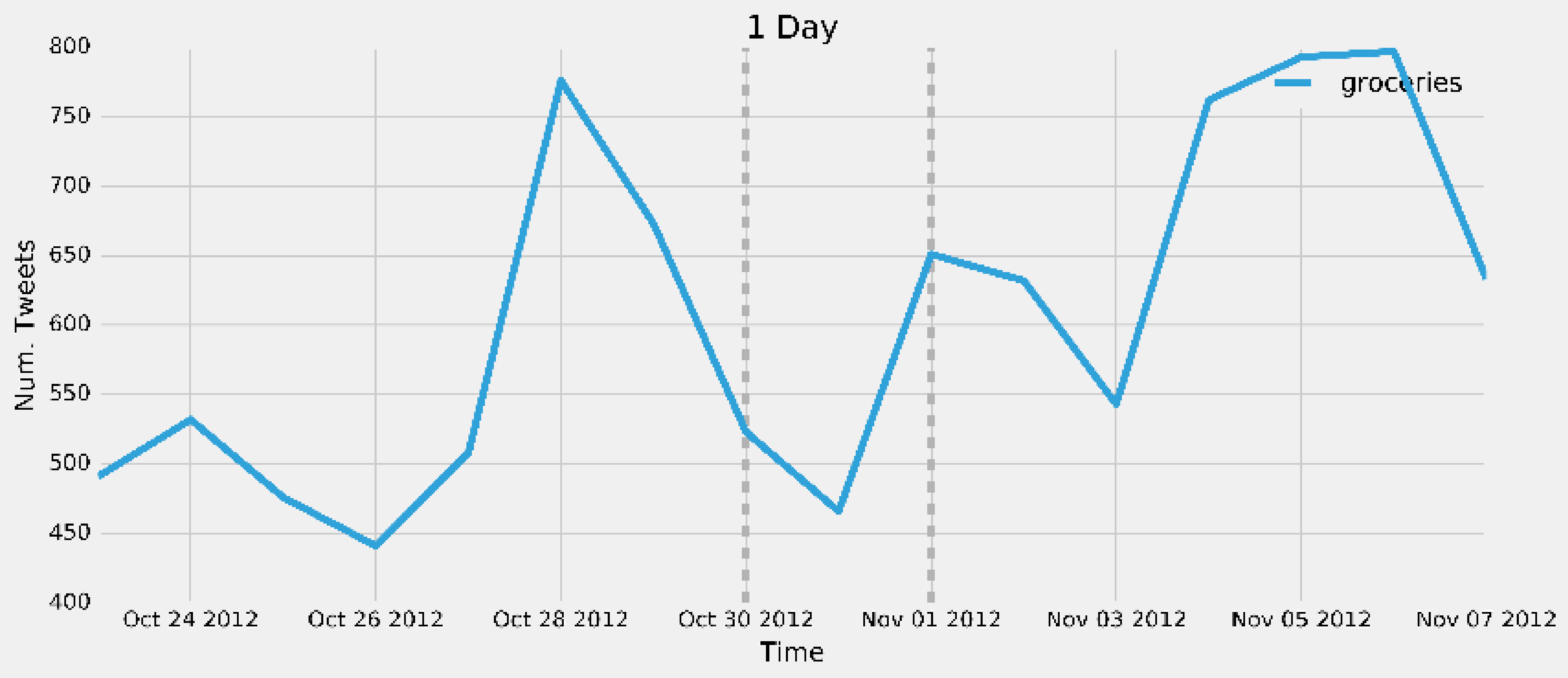
1 Day
groceries
Num. Tweets
Time
800
750
700
650
600
550
500
450
400
Oct 24 2012
Oct 26 2012
Oct 28 2012
Oct 30 2012
Nov 01 2012
Nov 03 2012
Nov 05 2012
Nov 07 2012

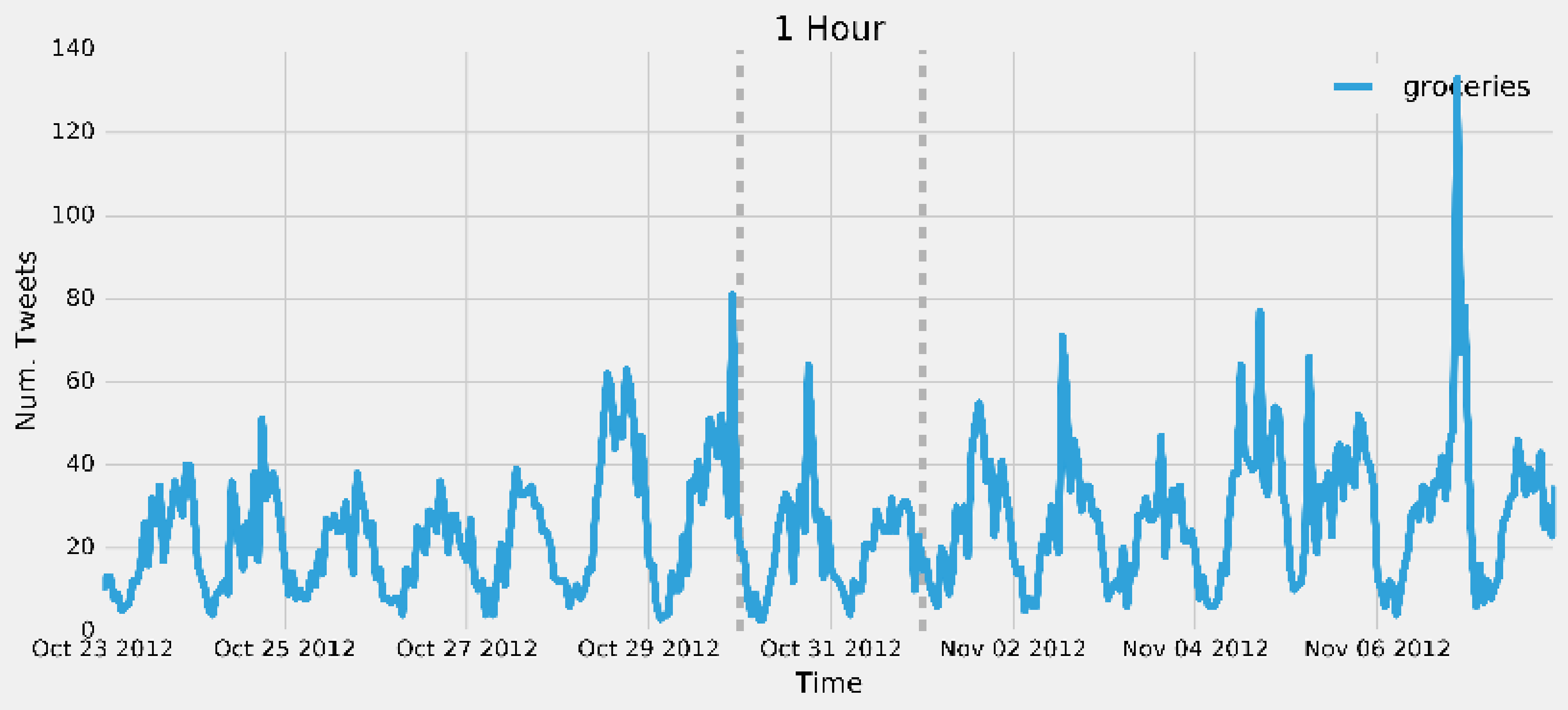

1 Hour
groceries
Num. Tweets
0
20
40
60
80
100
120
140
Oct 23 2012
Oct 25 2012
Oct 27 2012
Oct 29 2012
Oct 31 2012
Nov 02 2012
Nov 04 2012
Nov 06 2012
Time

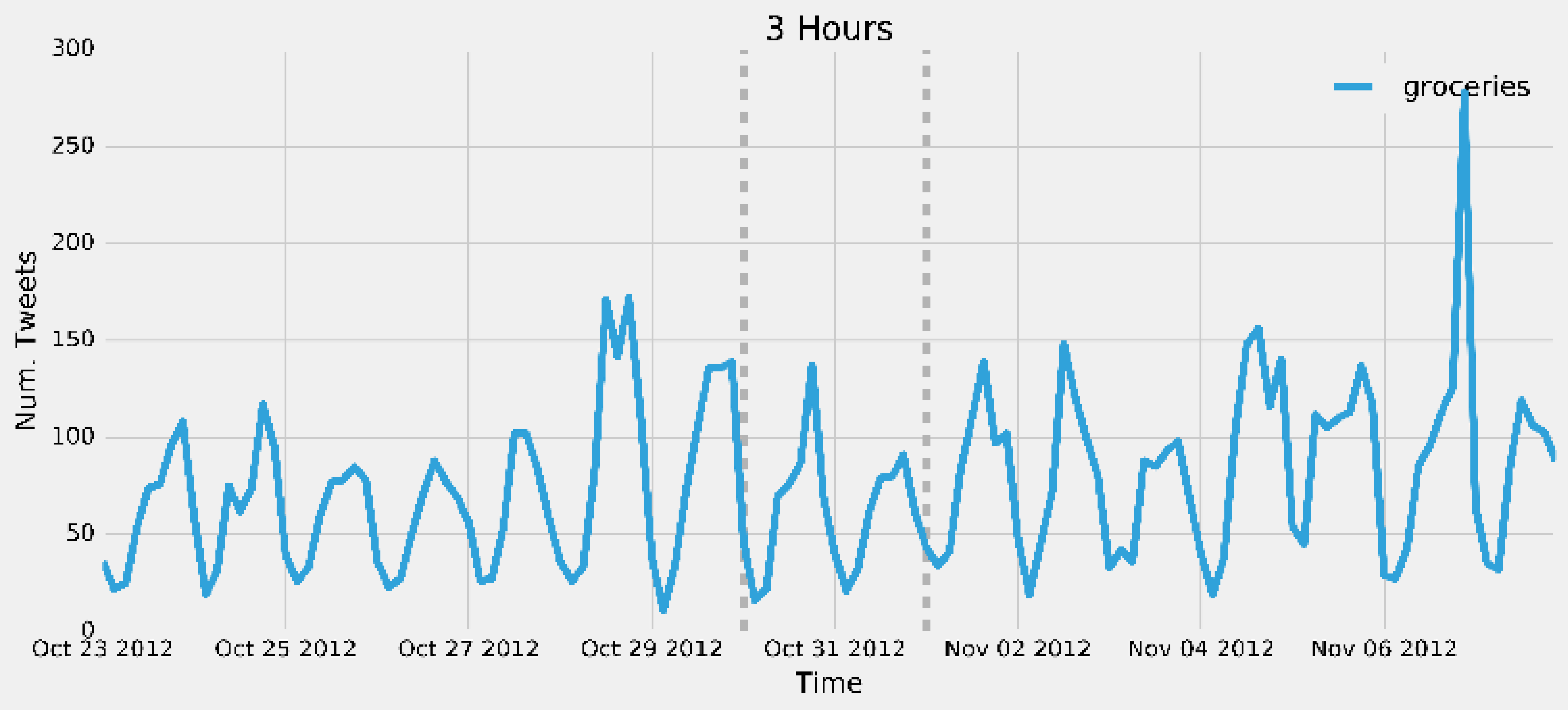
3 Hours
groceries
Num. Tweets
0
50
100
150
200
250
300
Oct 23 2012
Oct 25 2012
Oct 27 2012
Oct 29 2012
Oct 31 2012
Nov 02 2012
Nov 04 2012
Nov 06 2012
Time

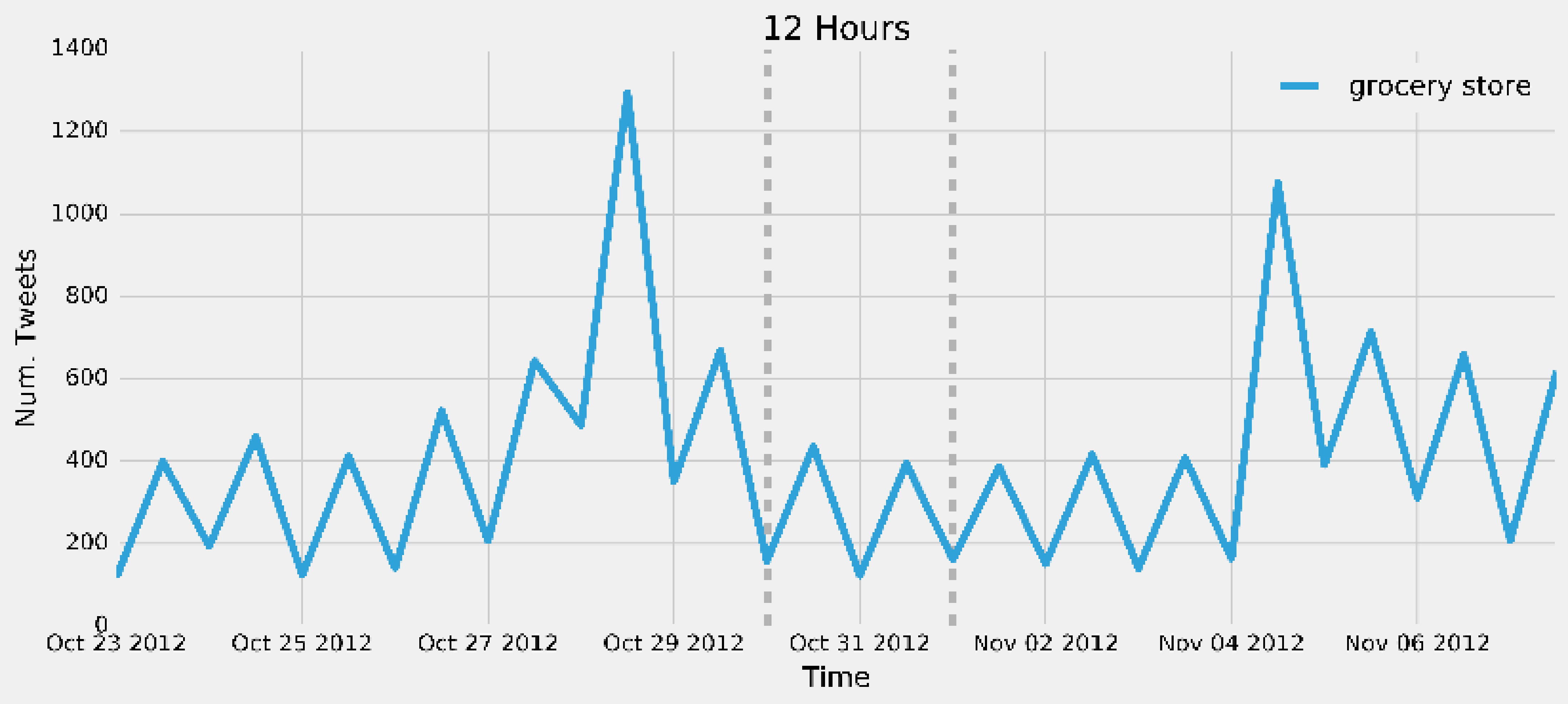

12 Hours
grocery store
Num. Tweets
Time
0
200
400
600
800
1000
1200
1400
Oct 23 2012
Oct 25 2012
Oct 27 2012
Oct 29 2012
Oct 31 2012
Nov 02 2012
Nov 04 2012
Nov 06 2012

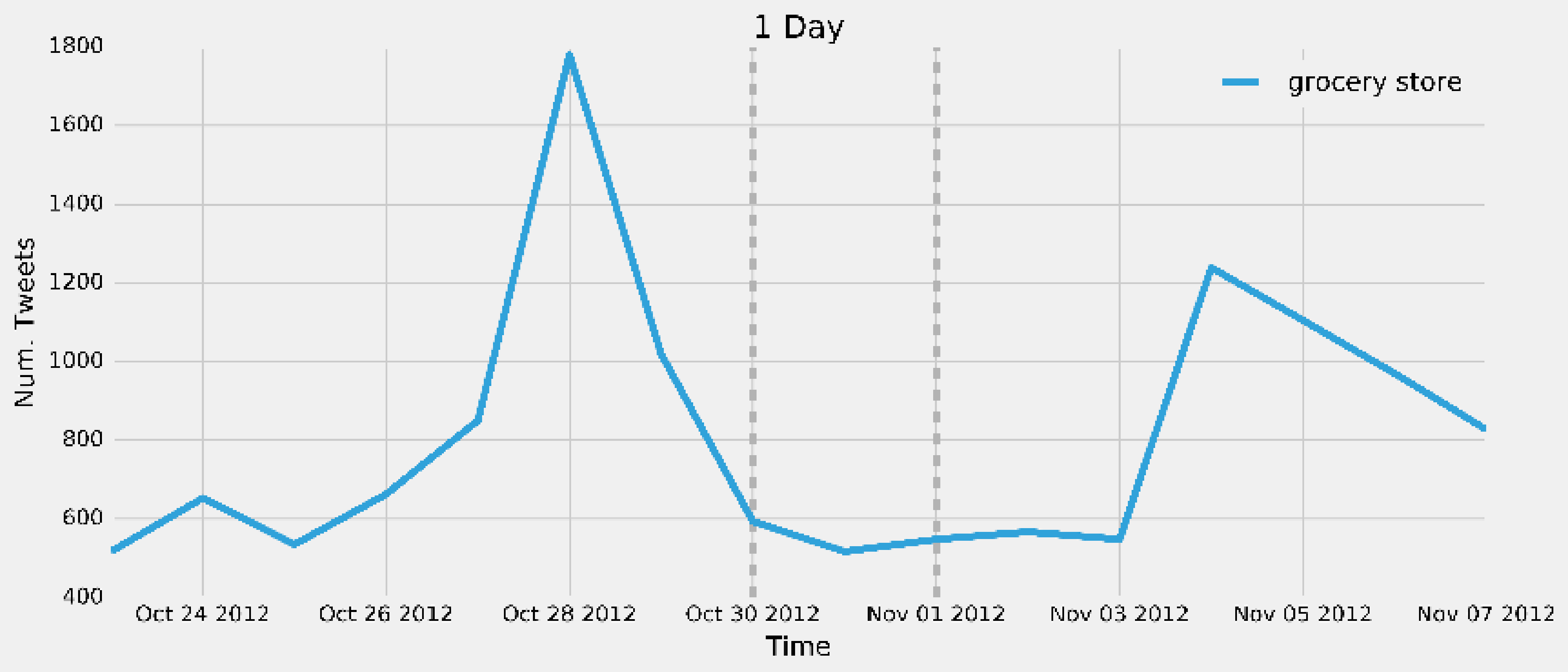

1 Day
grocery store
Num. Tweets
1800
1600
1400
1200
1000
800
600
400
Oct 24 2012
Oct 26 2012
Oct 28 2012
Oct 30 2012
Nov 01 2012
Nov 03 2012
Nov 05 2012
Nov 07 2012
Time

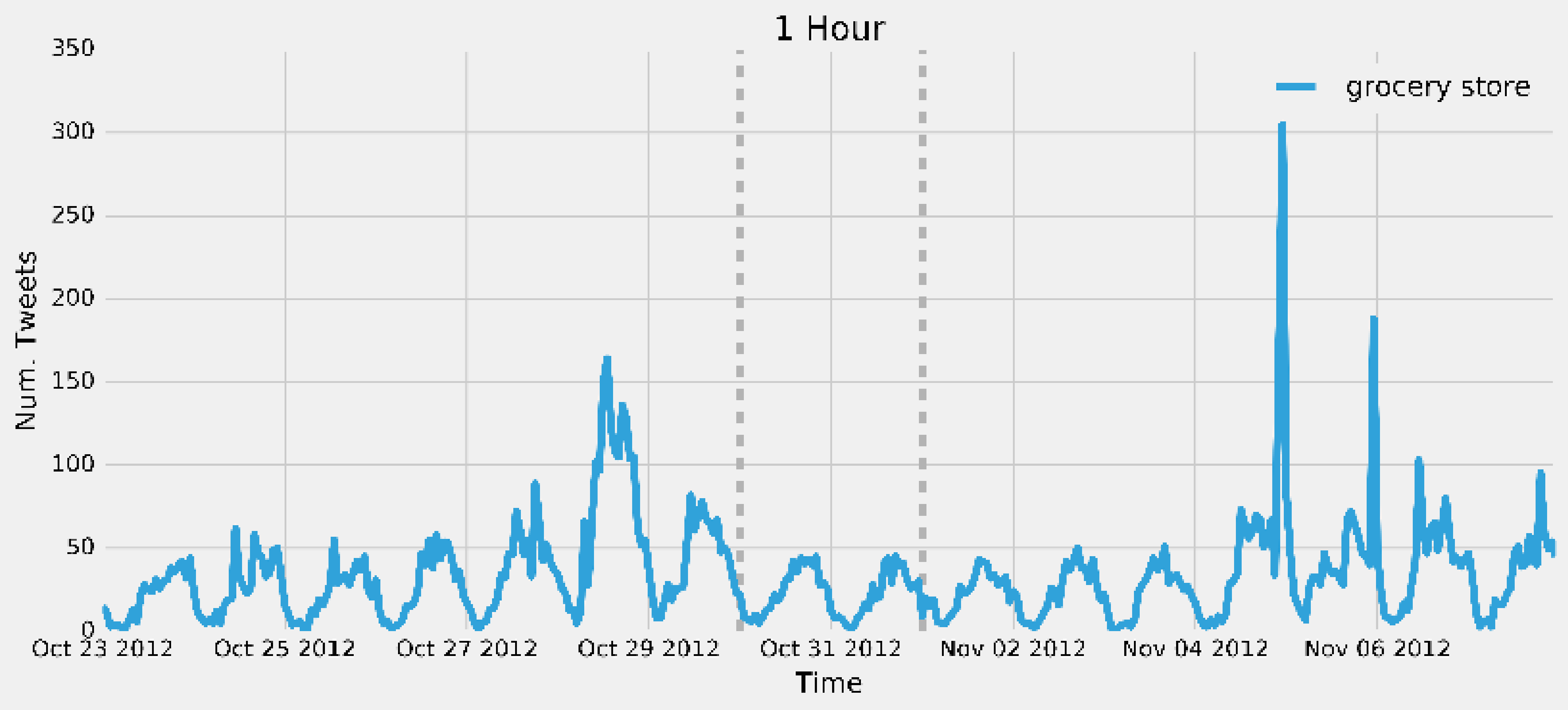

1 Hour
grocery store
Num. Tweets
0
50
100
150
200
250
300
350
Oct 23 2012
Oct 25 2012
Oct 27 2012
Oct 29 2012
Oct 31 2012
Nov 02 2012
Nov 04 2012
Nov 06 2012
Time

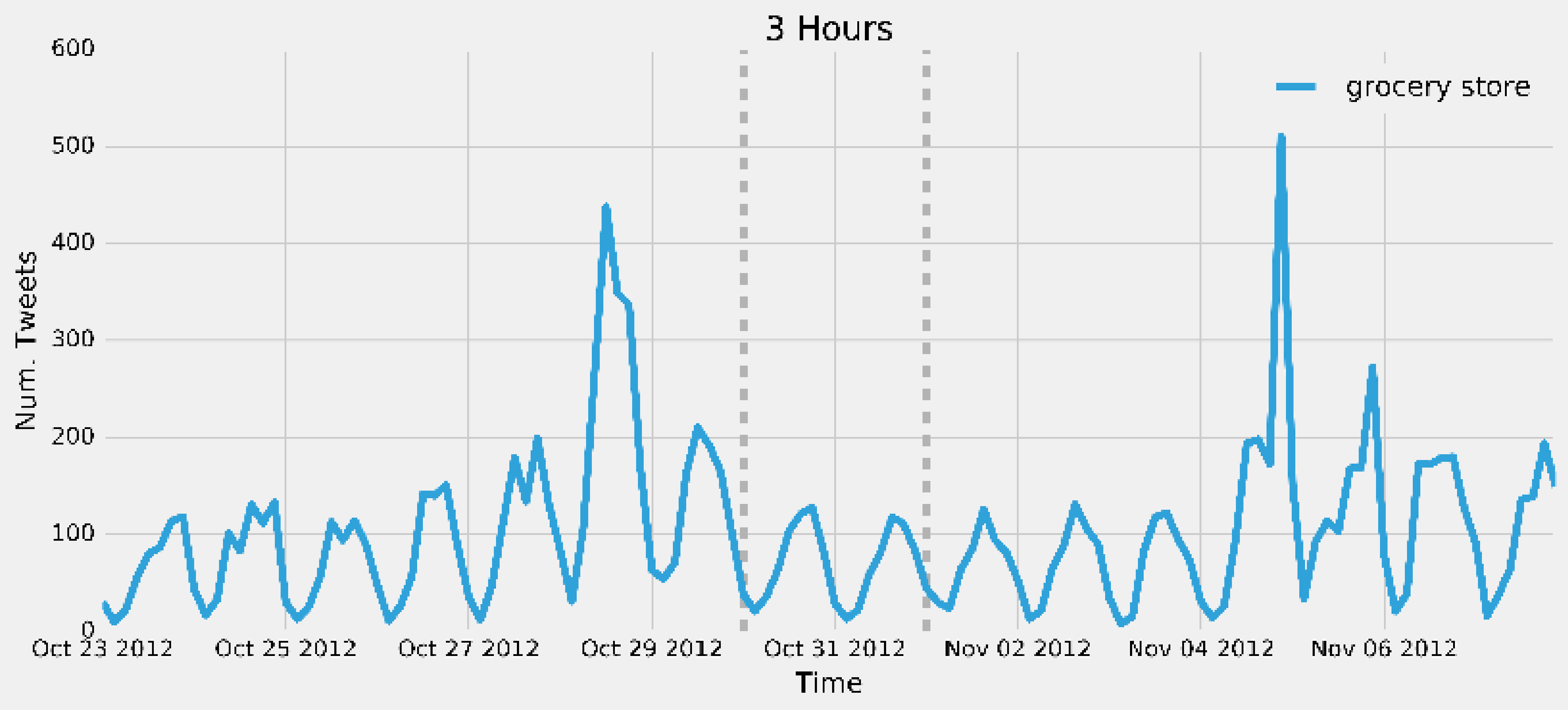
3 Hours
grocery store
Num. Tweets
600
500
400
300
200
100
0
Oct 23 2012
Oct 25 2012
Oct 27 2012
Oct 29 2012
Oct 31 2012
Nov 02 2012
Nov 04 2012
Nov 06 2012
Time

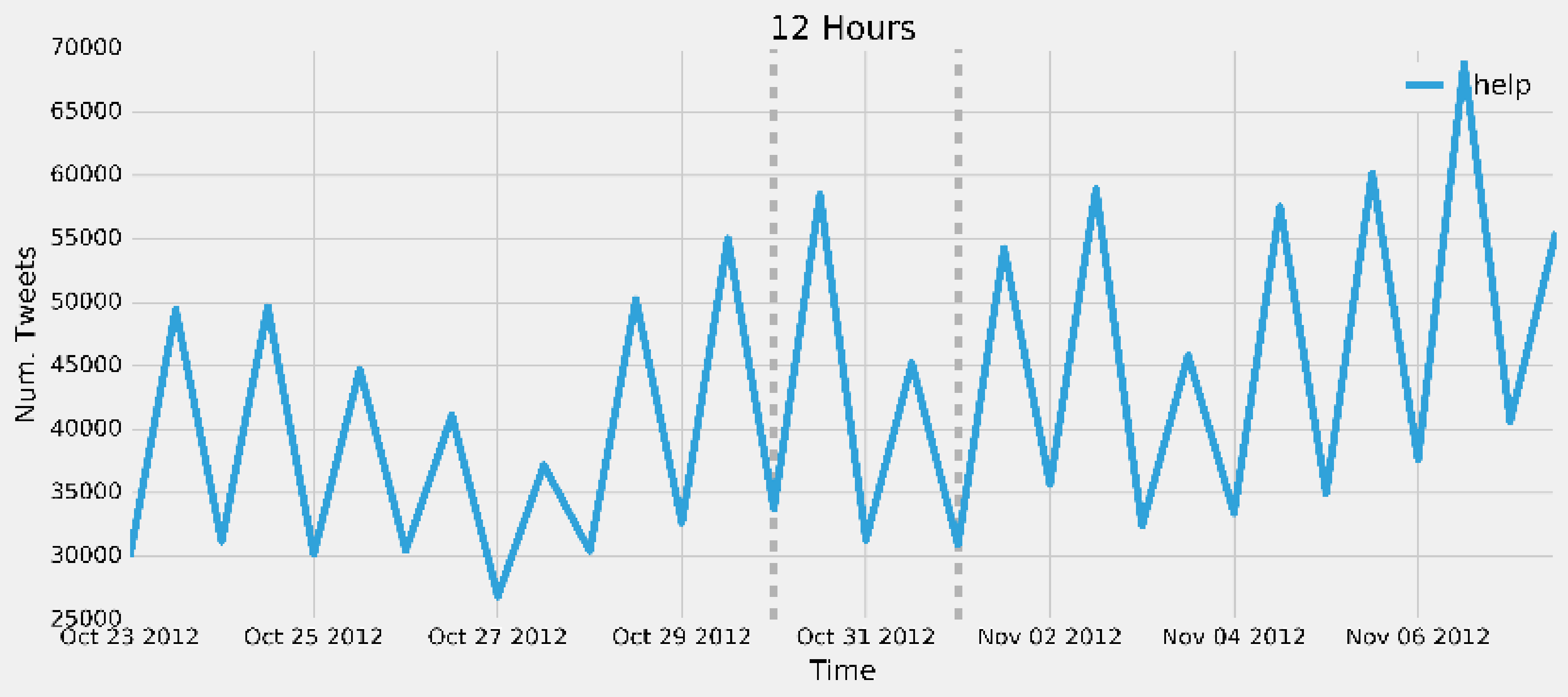
12 Hours
help
Num. Tweets
70000
65000
60000
55000
50000
45000
40000
35000
30000
25000
Oct 23 2012
Oct 25 2012
Oct 27 2012
Oct 29 2012
Oct 31 2012
Nov 02 2012
Nov 04 2012
Nov 06 2012
Time

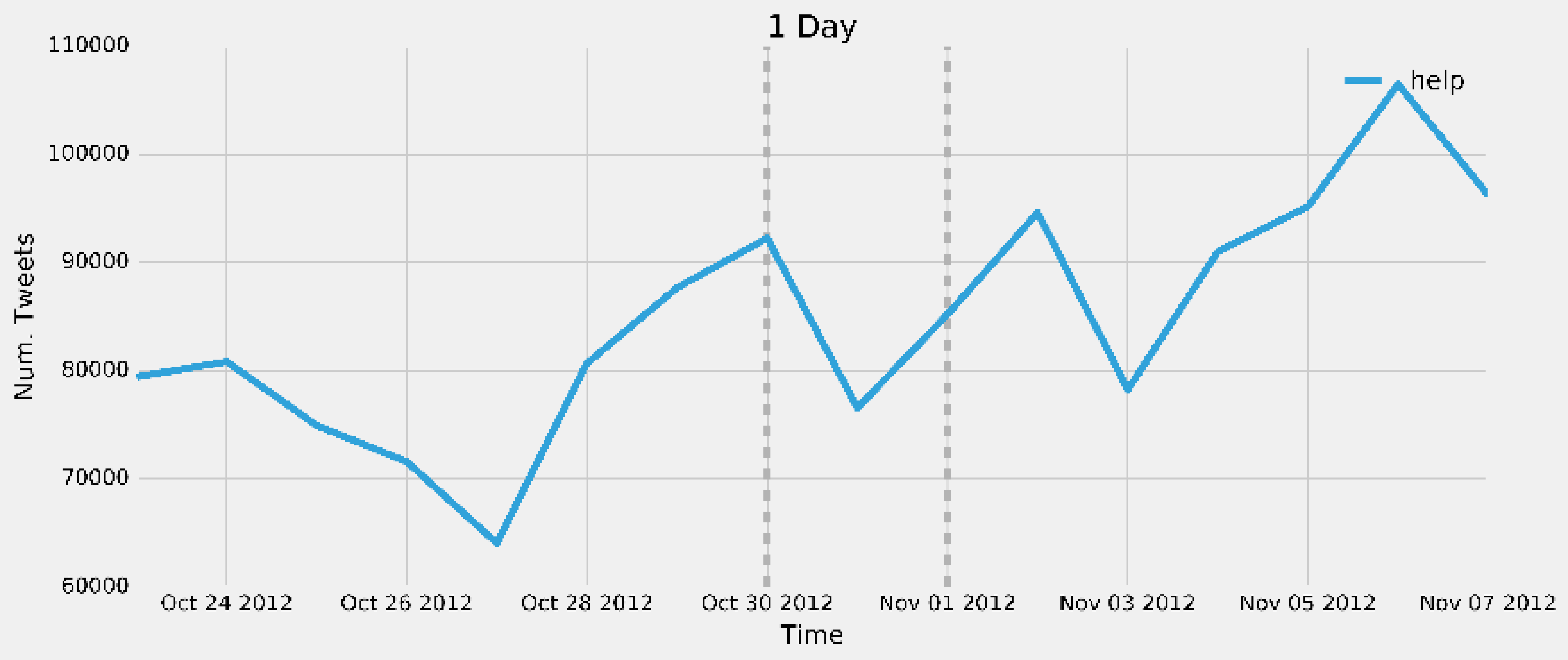

1 Day
help
Num. Tweets
110000
100000
90000
80000
70000
60000
Oct 24 2012
Oct 26 2012
Oct 28 2012
Oct 30 2012
Nov 01 2012
Nov 03 2012
Nov 05 2012
Nov 07 2012
Time

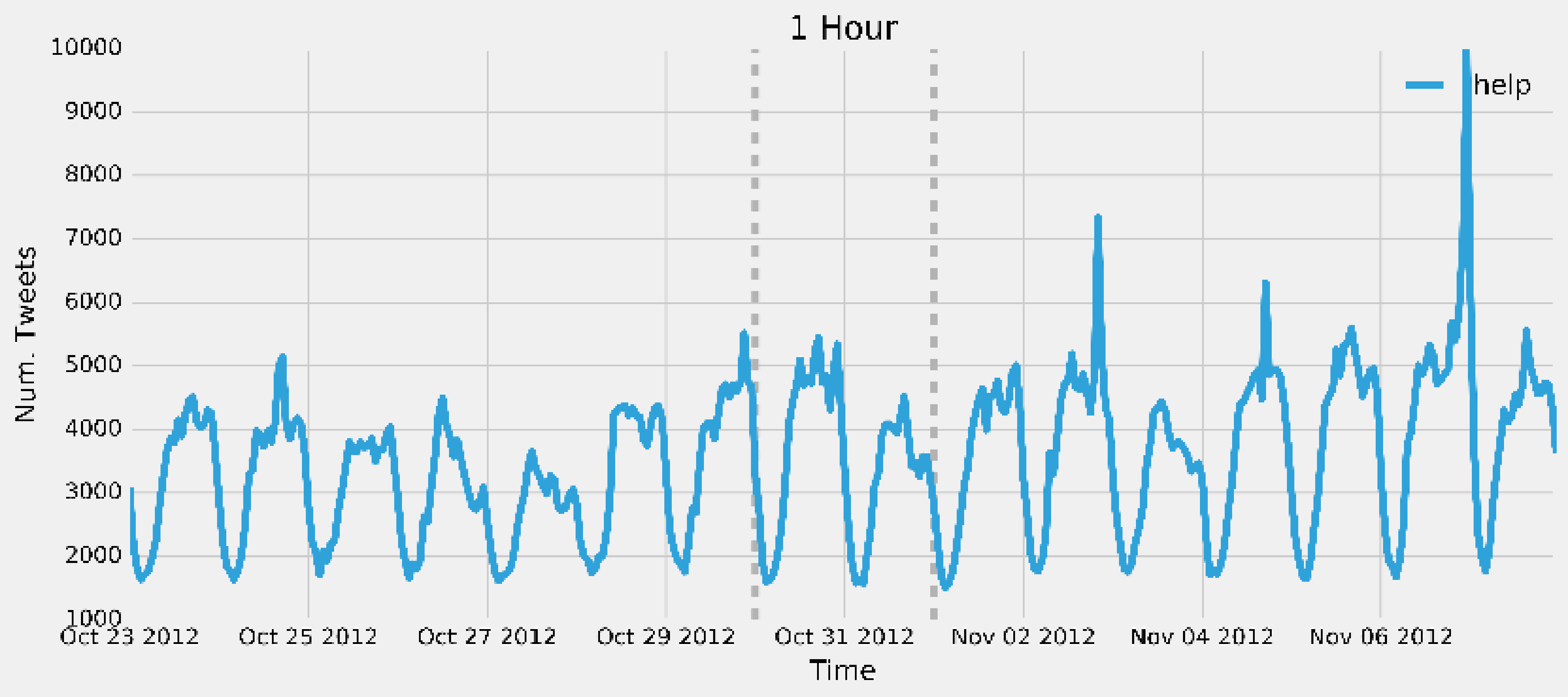

1 Hour
help
Num. Tweets
10000
9000
8000
7000
6000
5000
4000
3000
2000
1000
Oct 23 2012
Oct 25 2012
Oct 27 2012
Oct 29 2012
Oct 31 2012
Nov 02 2012
Nov 04 2012
Nov 06 2012
Time

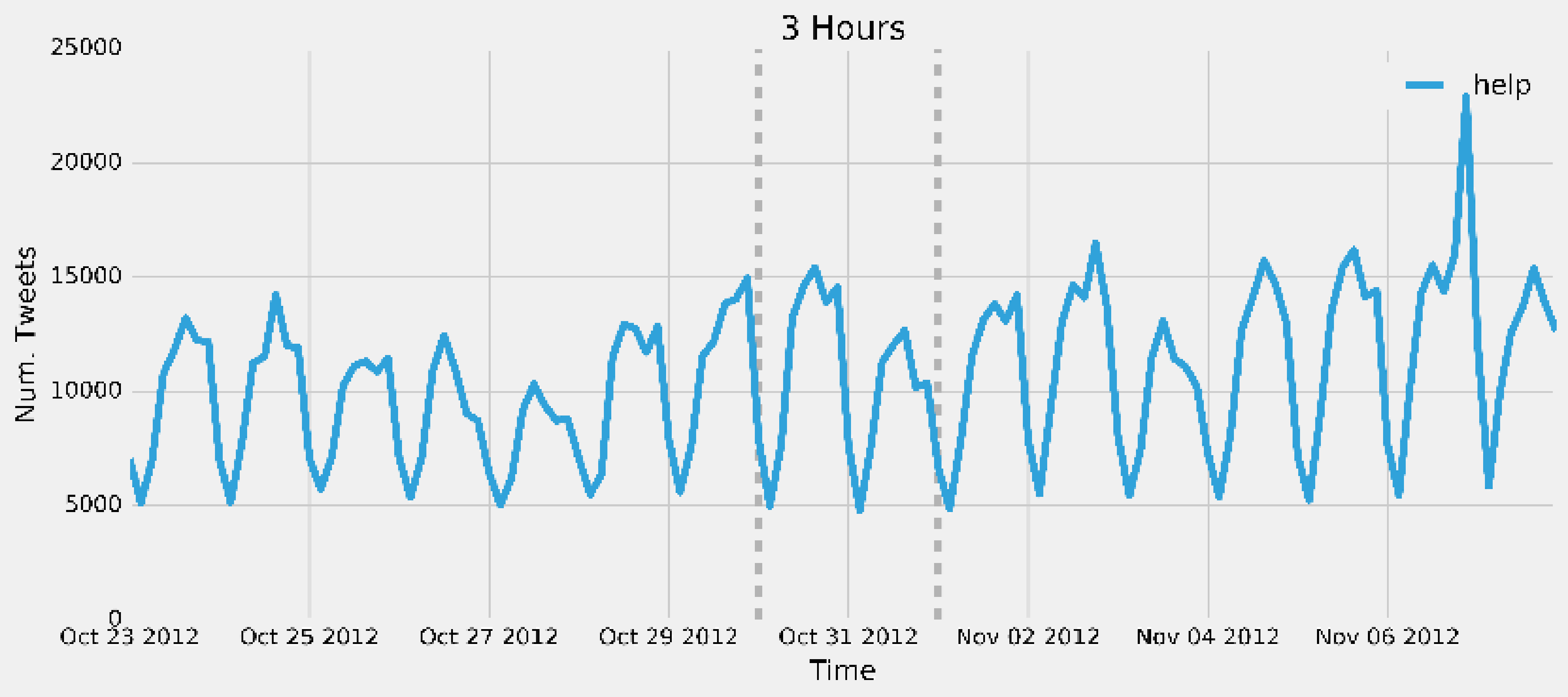

3 Hours
help
25000
20000
15000
10000
5000
0
Num. Tweets
Oct 23 2012
Oct 25 2012
Oct 27 2012
Oct 29 2012
Oct 31 2012
Nov 02 2012
Nov 04 2012
Nov 06 2012
Time

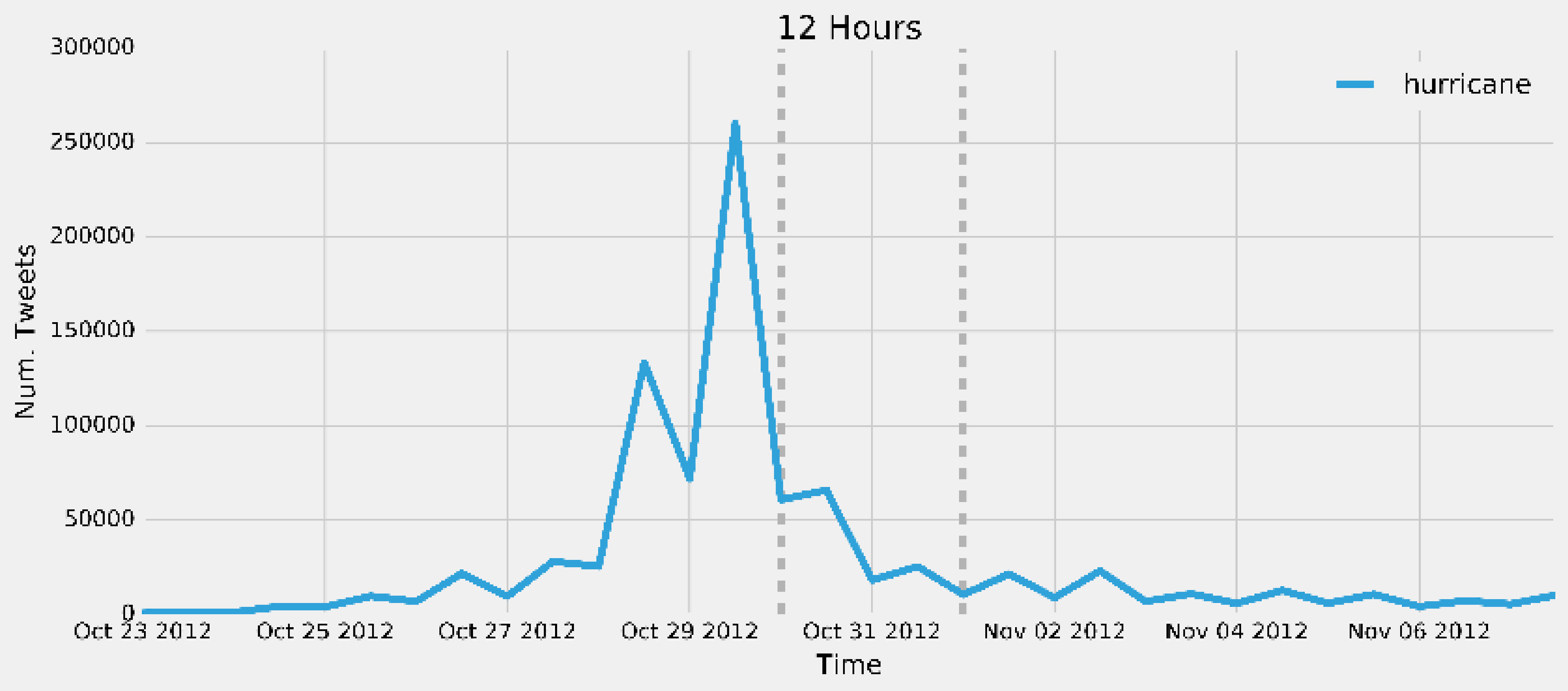
12 Hours
hurricane
300000
250000
200000
150000
100000
50000
0
Num. Tweets
Oct 23 2012
Oct 25 2012
Oct 27 2012
Oct 29 2012
Oct 31 2012
Nov 02 2012
Nov 04 2012
Nov 06 2012
Time

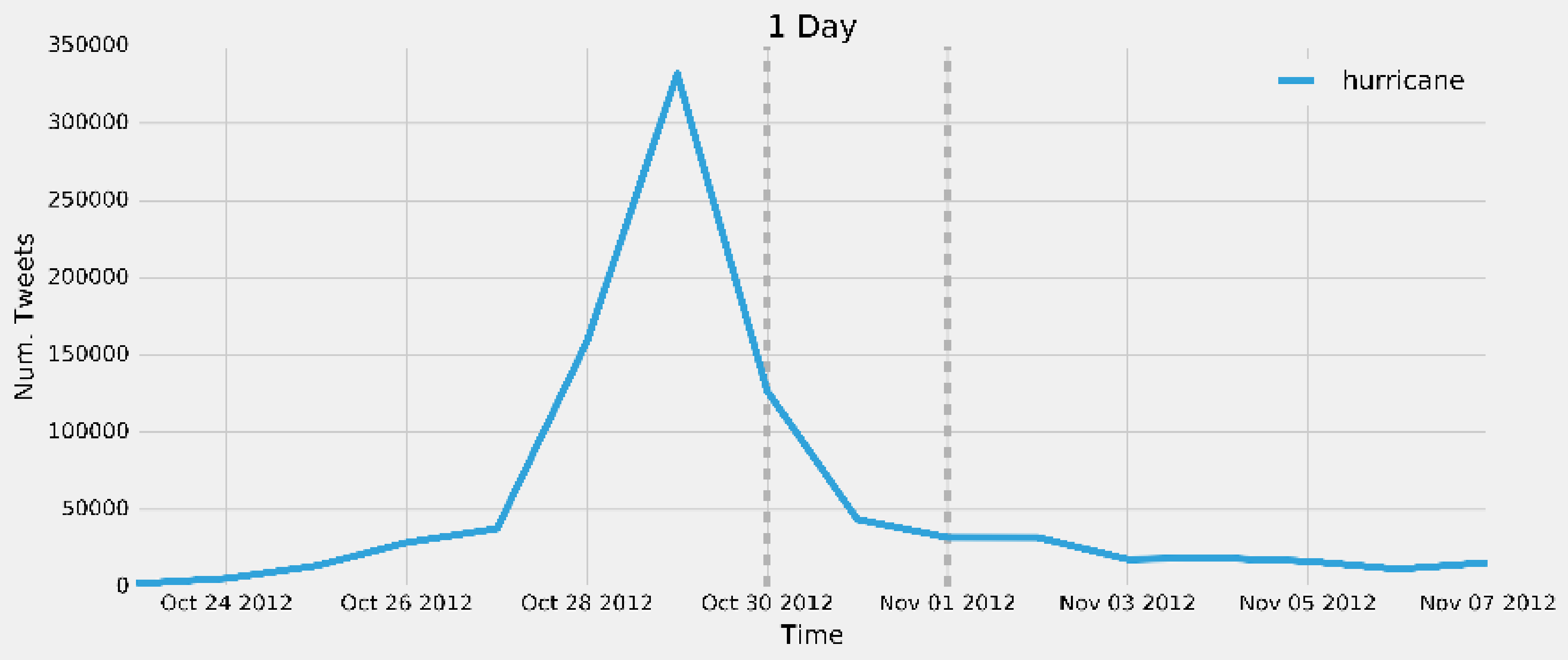
1 Day
hurricane
350000
300000
250000
200000
150000
100000
50000
0
Num. Tweets
Oct 24 2012
Oct 26 2012
Oct 28 2012
Oct 30 2012
Nov 01 2012
Nov 03 2012
Nov 05 2012
Nov 07 2012
Time

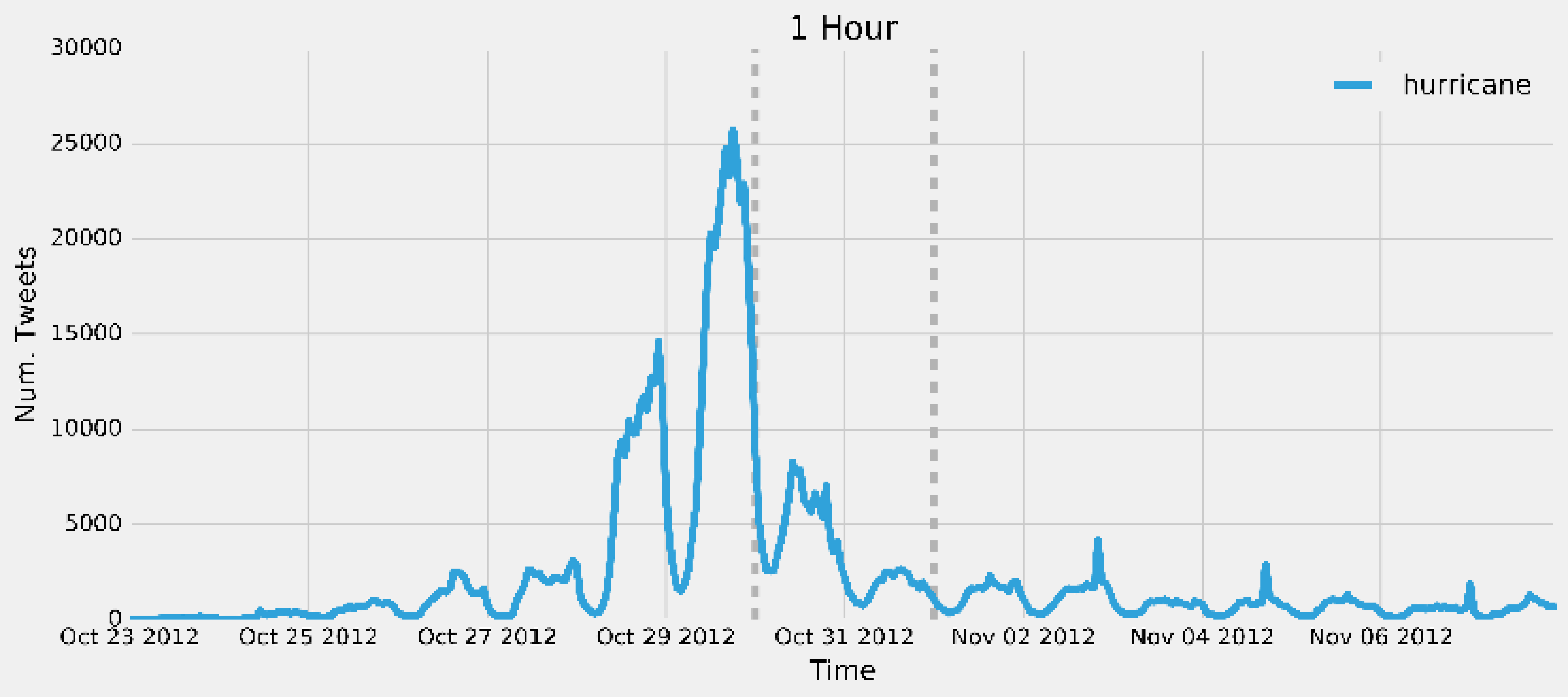

1 Hour
hurricane
30000
25000
20000
15000
10000
5000
0
Num. Tweets
Oct 23 2012
Oct 25 2012
Oct 27 2012
Oct 29 2012
Oct 31 2012
Nov 02 2012
Nov 04 2012
Nov 06 2012
Time

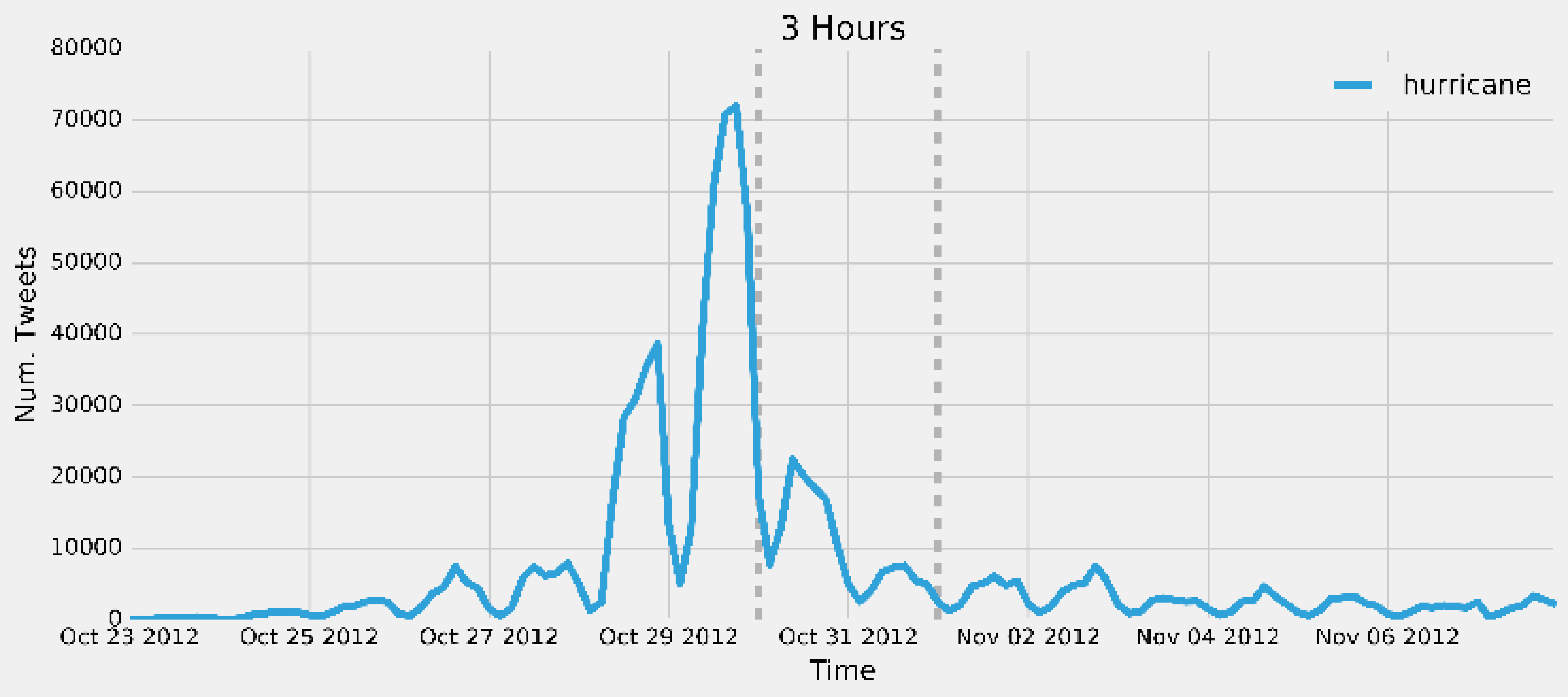
3 Hours
hurricane
Num. Tweets
80000
70000
60000
50000
40000
30000
20000
10000
0
Oct 23 2012
Oct 25 2012
Oct 27 2012
Oct 29 2012
Oct 31 2012
Nov 02 2012
Nov 04 2012
Nov 06 2012
Time

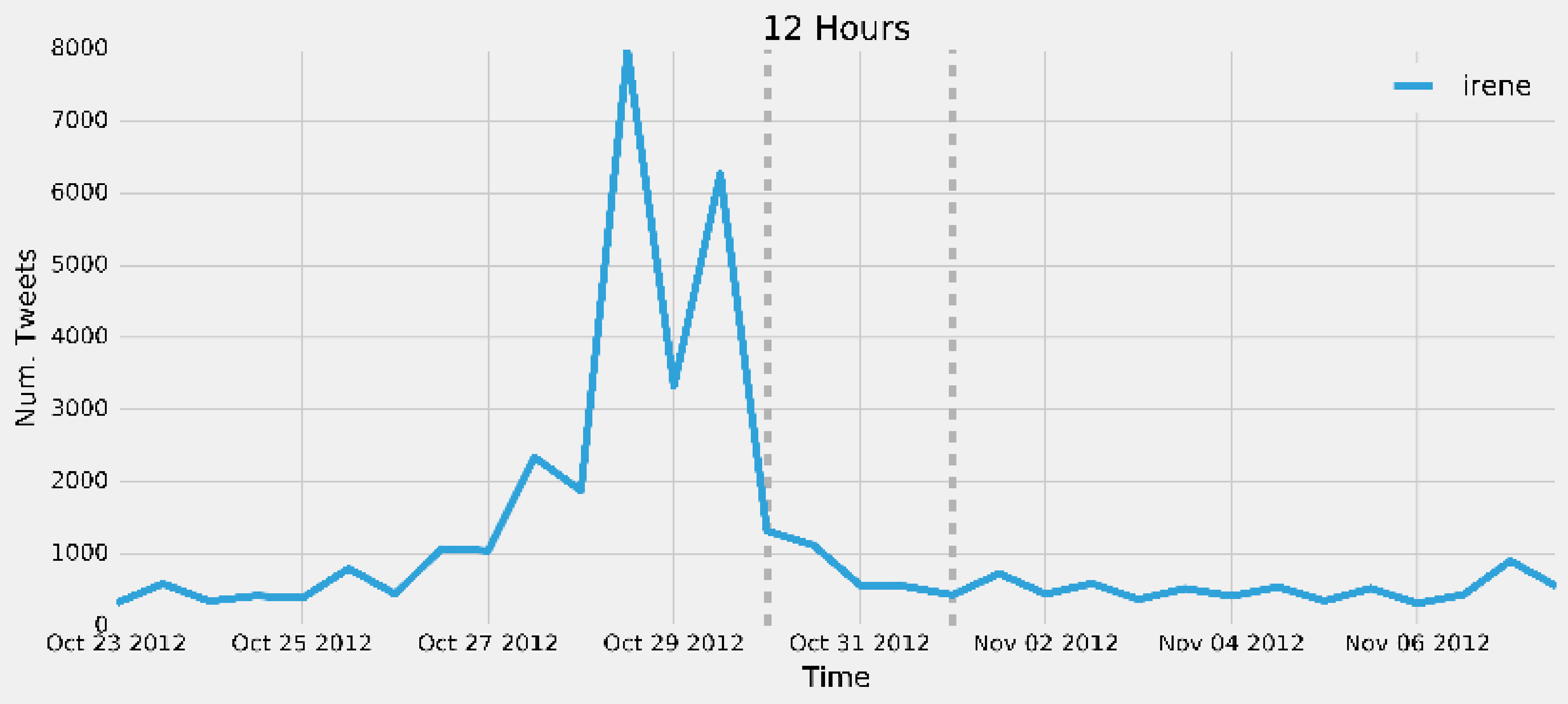
12 Hours
irene
Num. Tweets
8000
7000
6000
5000
4000
3000
2000
1000
0
Oct 23 2012
Oct 25 2012
Oct 27 2012
Oct 29 2012
Oct 31 2012
Nov 02 2012
Nov 04 2012
Nov 06 2012
Time

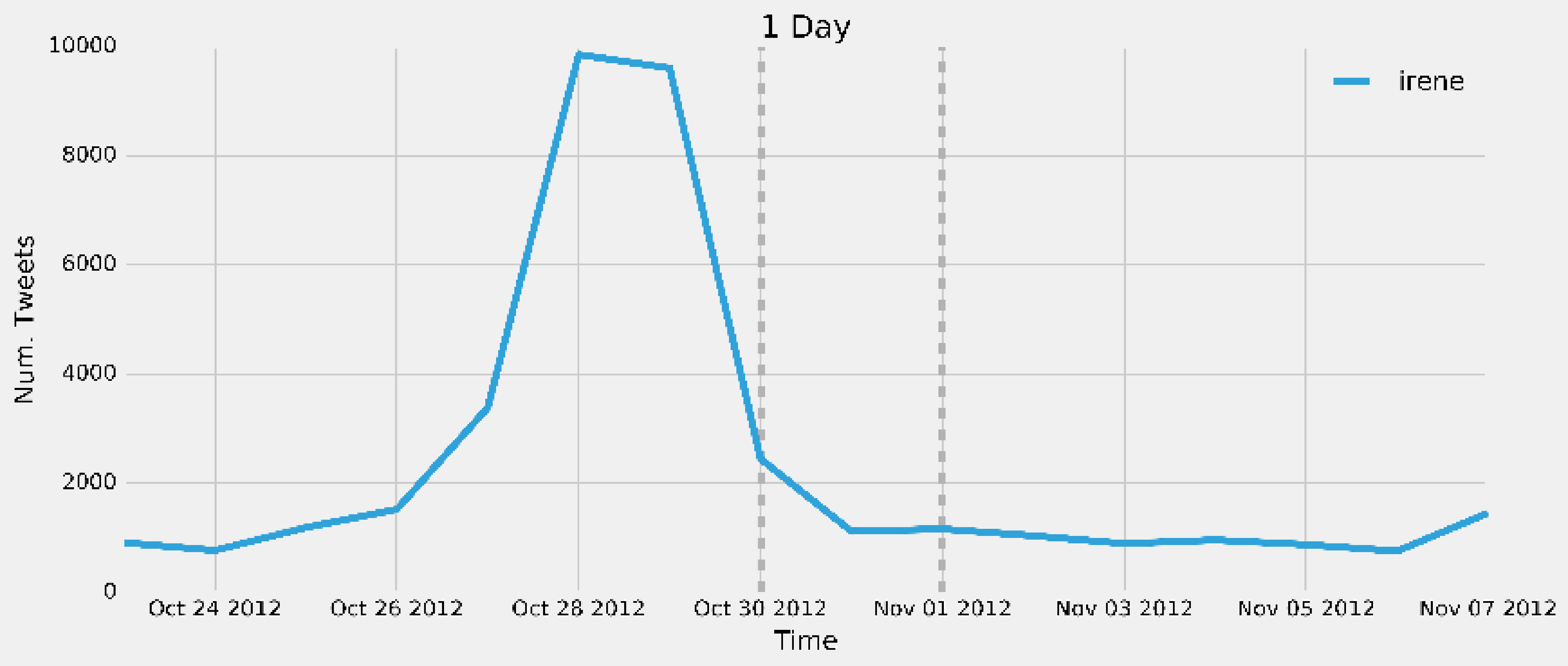
1 Day
irene
Num. Tweets
0
2000
4000
6000
8000
10000
Oct 24 2012
Oct 26 2012
Oct 28 2012
Oct 30 2012
Nov 01 2012
Nov 03 2012
Nov 05 2012
Nov 07 2012
Time

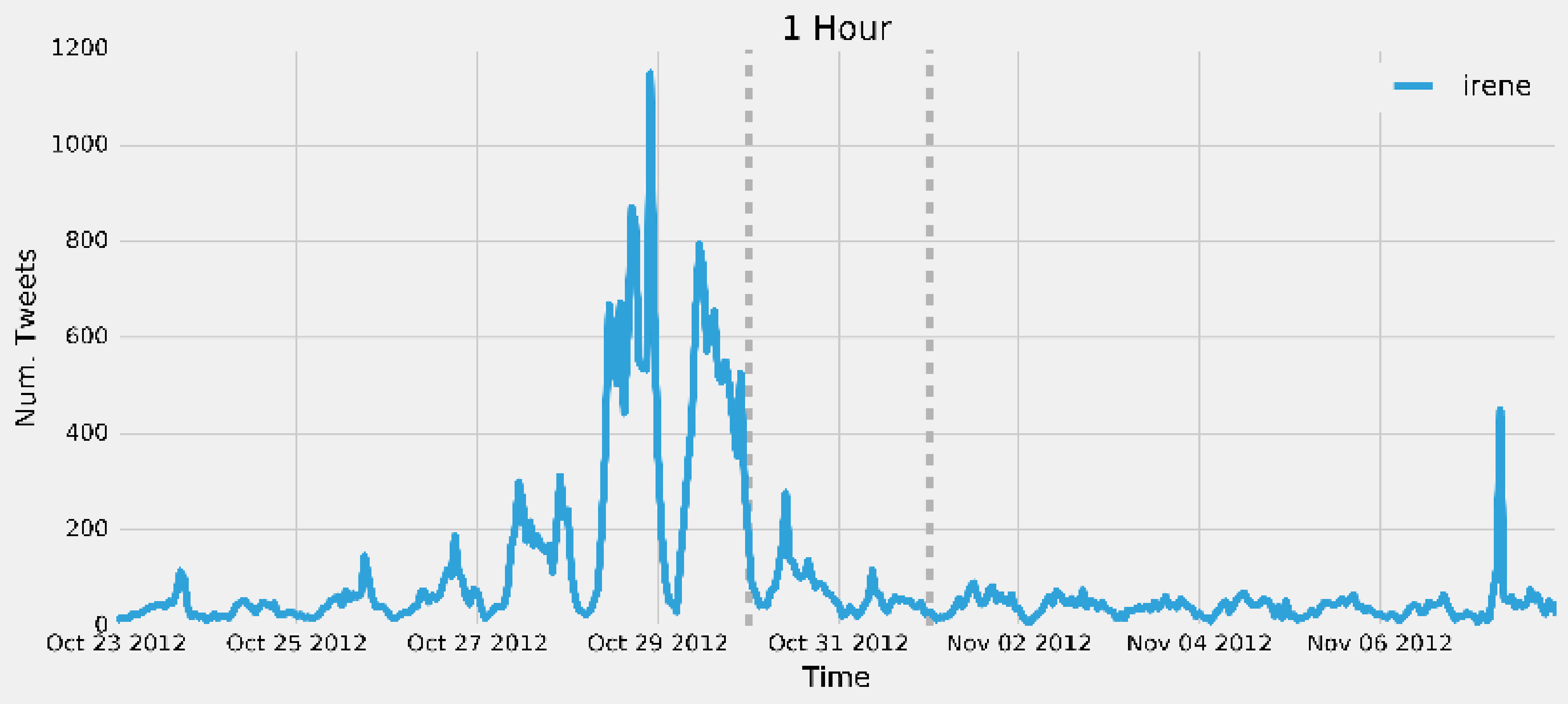

1 Hour
irene
Num. Tweets
0
200
400
600
800
1000
1200
Oct 23 2012
Oct 25 2012
Oct 27 2012
Oct 29 2012
Oct 31 2012
Nov 02 2012
Nov 04 2012
Nov 06 2012
Time

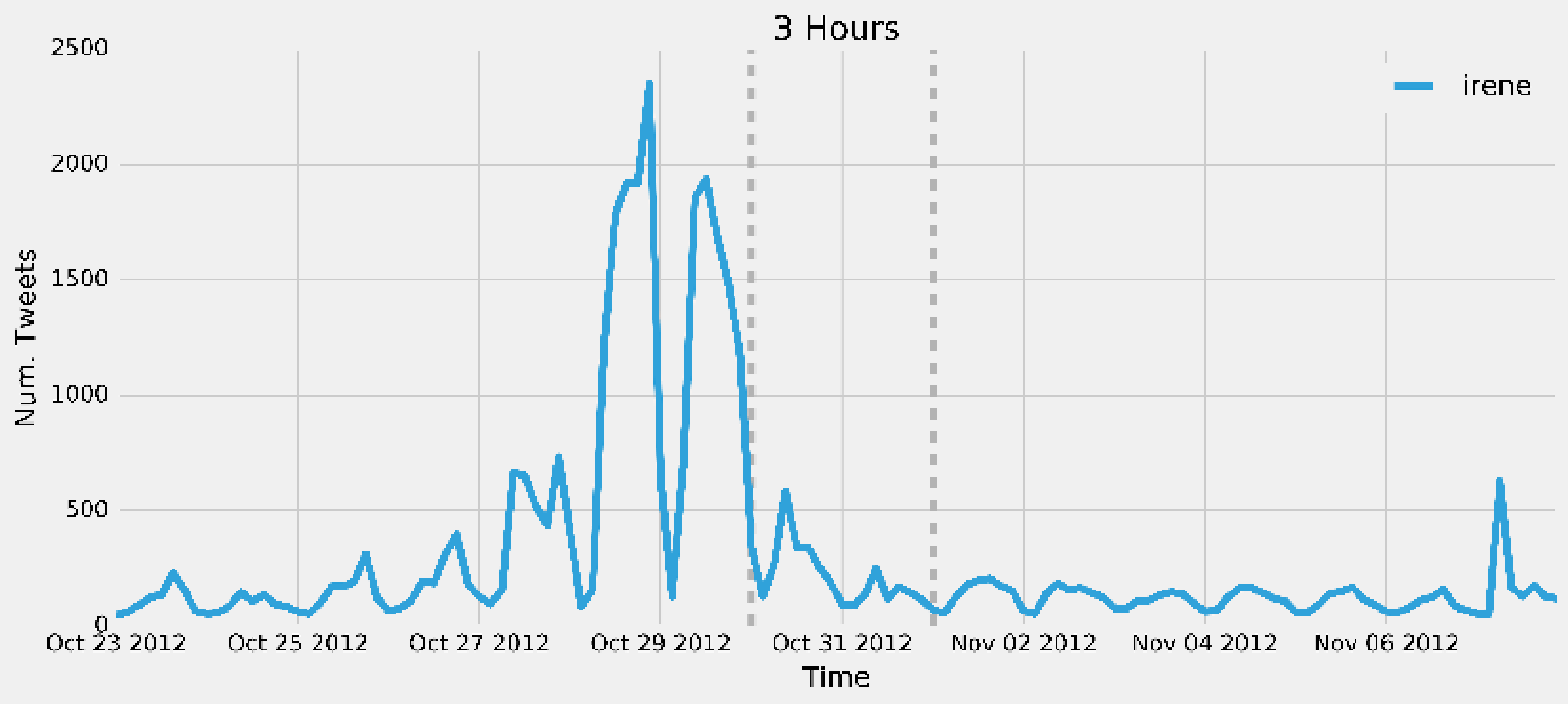
3 Hours
irene
Num. Tweets
2500
2000
1500
1000
500
0
Oct 23 2012
Oct 25 2012
Oct 27 2012
Oct 29 2012
Oct 31 2012
Nov 02 2012
Nov 04 2012
Nov 06 2012
Time

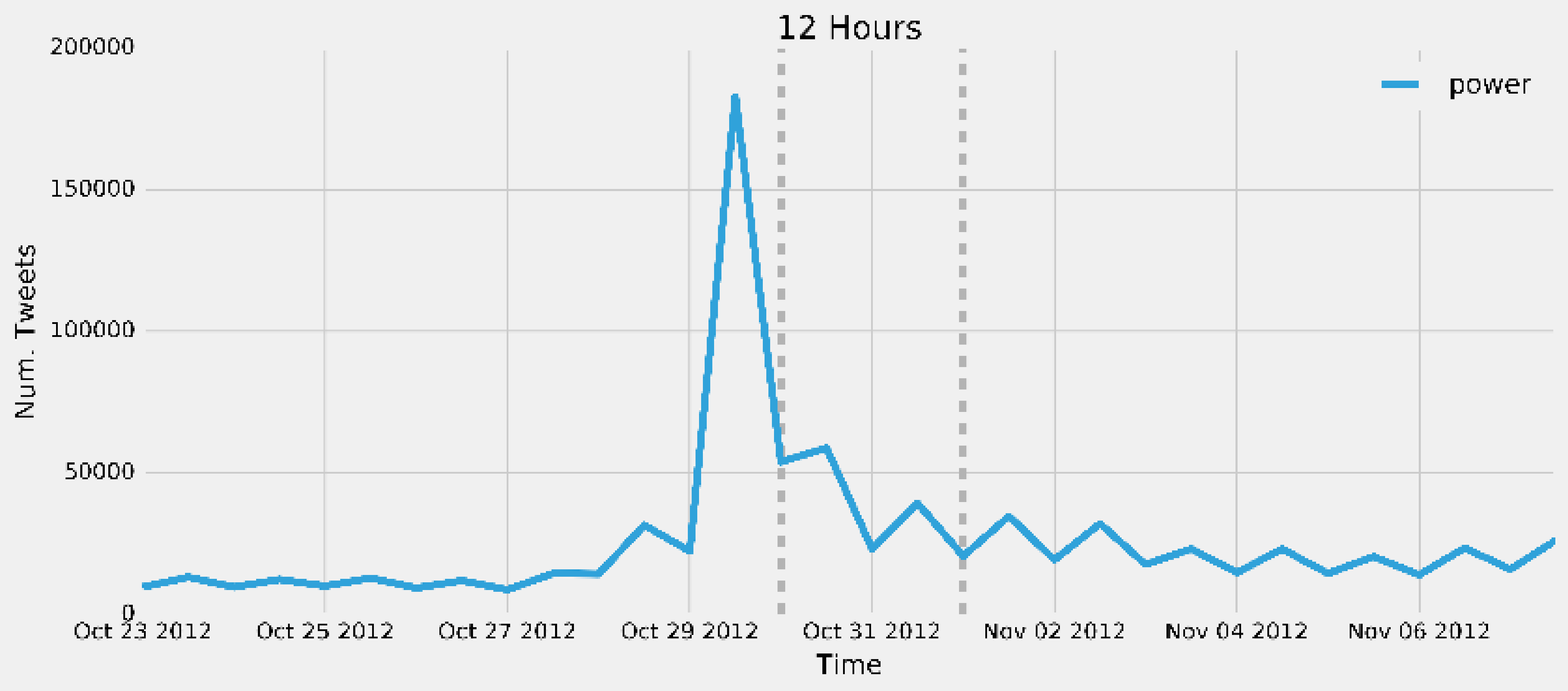

12 Hours
power
Num. Tweets
200000
150000
100000
50000
0
Oct 23 2012
Oct 25 2012
Oct 27 2012
Oct 29 2012
Oct 31 2012
Nov 02 2012
Nov 04 2012
Nov 06 2012
Time

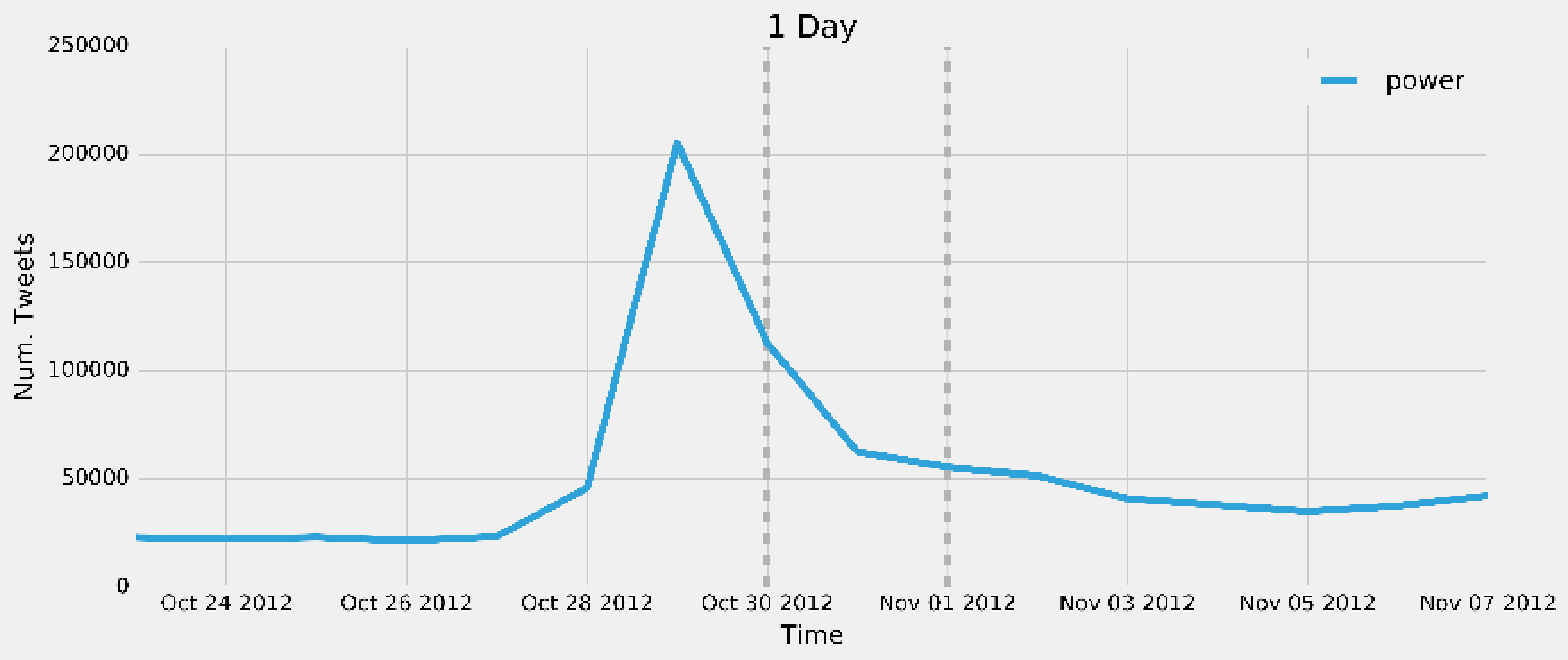
1 Day
power
Num. Tweets
0
50000
100000
150000
200000
250000
Oct 24 2012
Oct 26 2012
Oct 28 2012
Oct 30 2012
Nov 01 2012
Nov 03 2012
Nov 05 2012
Nov 07 2012
Time

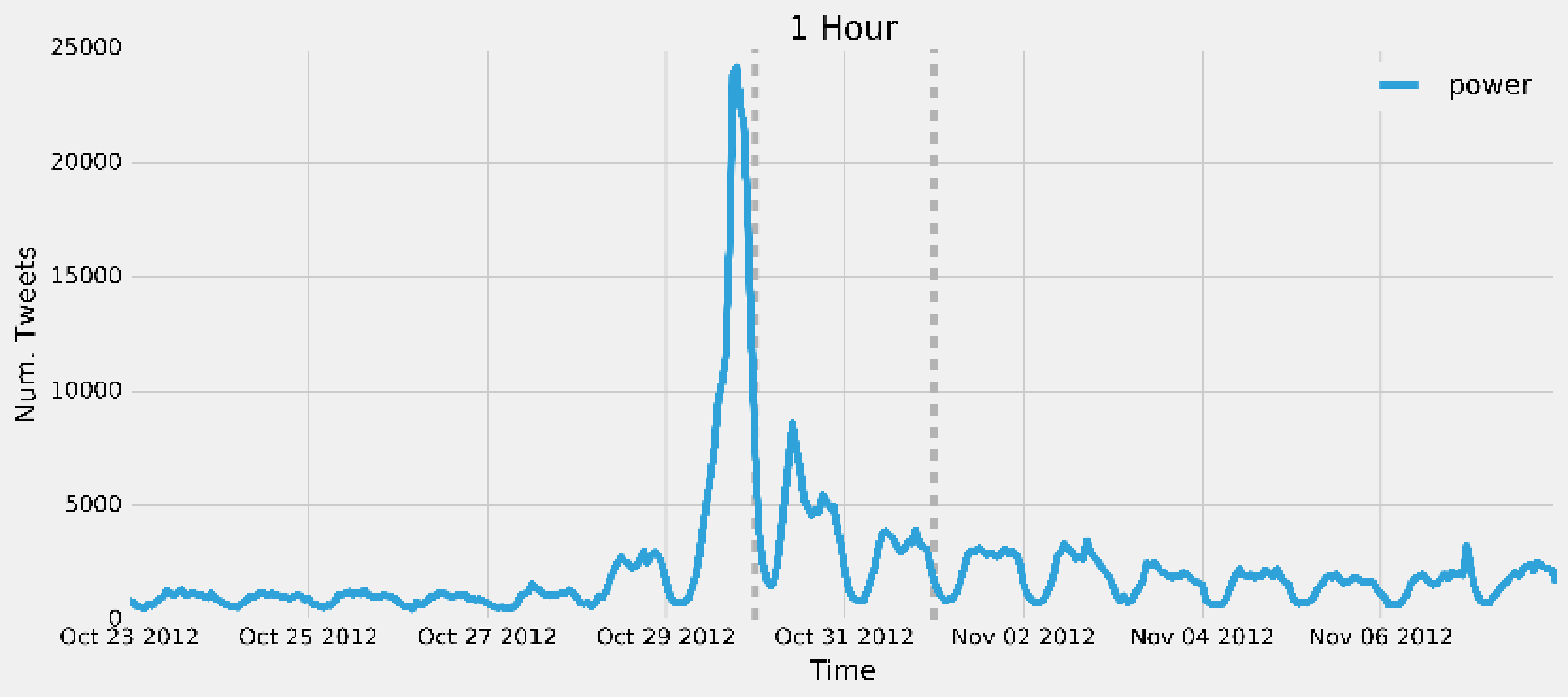
1 Hour
25000
20000
15000
10000
5000
0
Num. Tweets
power
Oct 23 2012
Oct 25 2012
Oct 27 2012
Oct 29 2012
Oct 31 2012
Nov 02 2012
Nov 04 2012
Nov 06 2012
Time

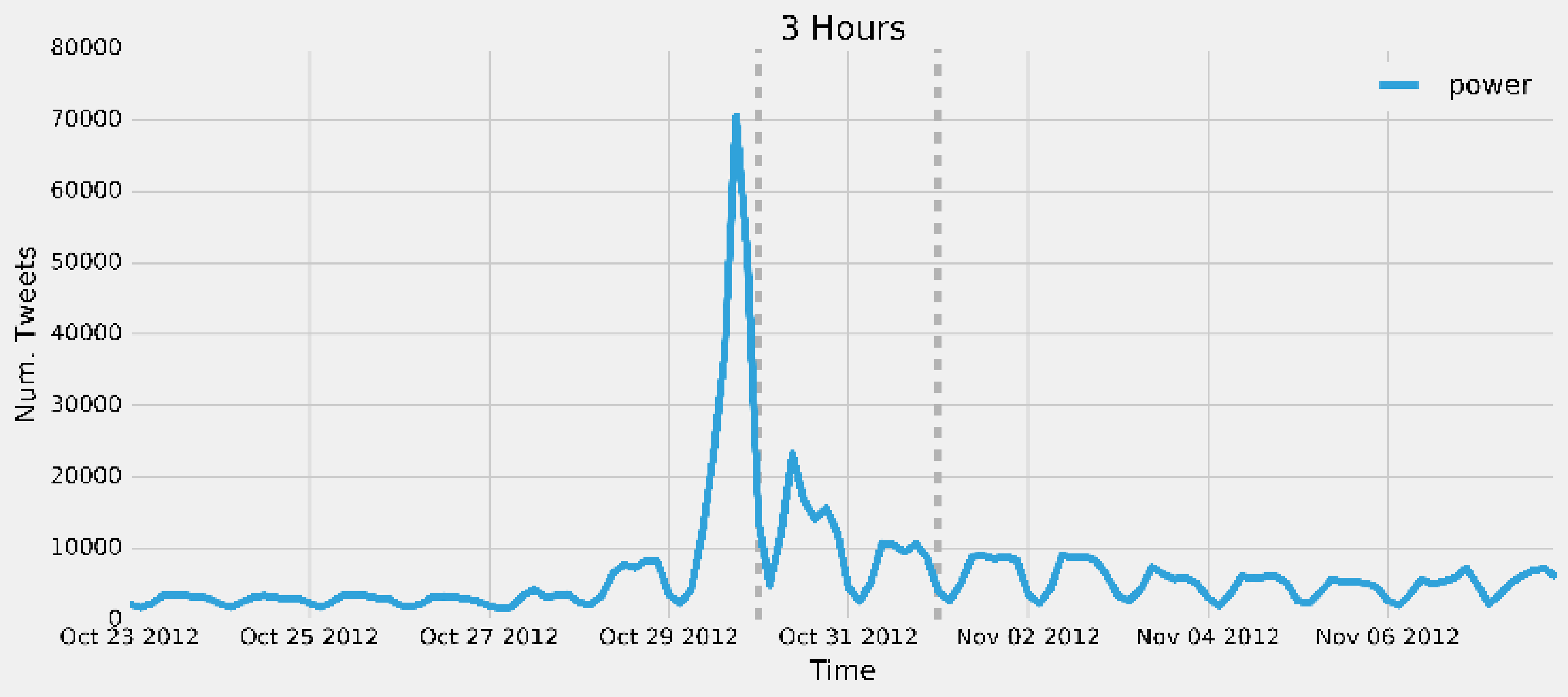

3 Hours
power
Num. Tweets
0
10000
20000
30000
40000
50000
60000
70000
80000
Oct 23 2012
Oct 25 2012
Oct 27 2012
Oct 29 2012
Oct 31 2012
Nov 02 2012
Nov 04 2012
Nov 06 2012
Time

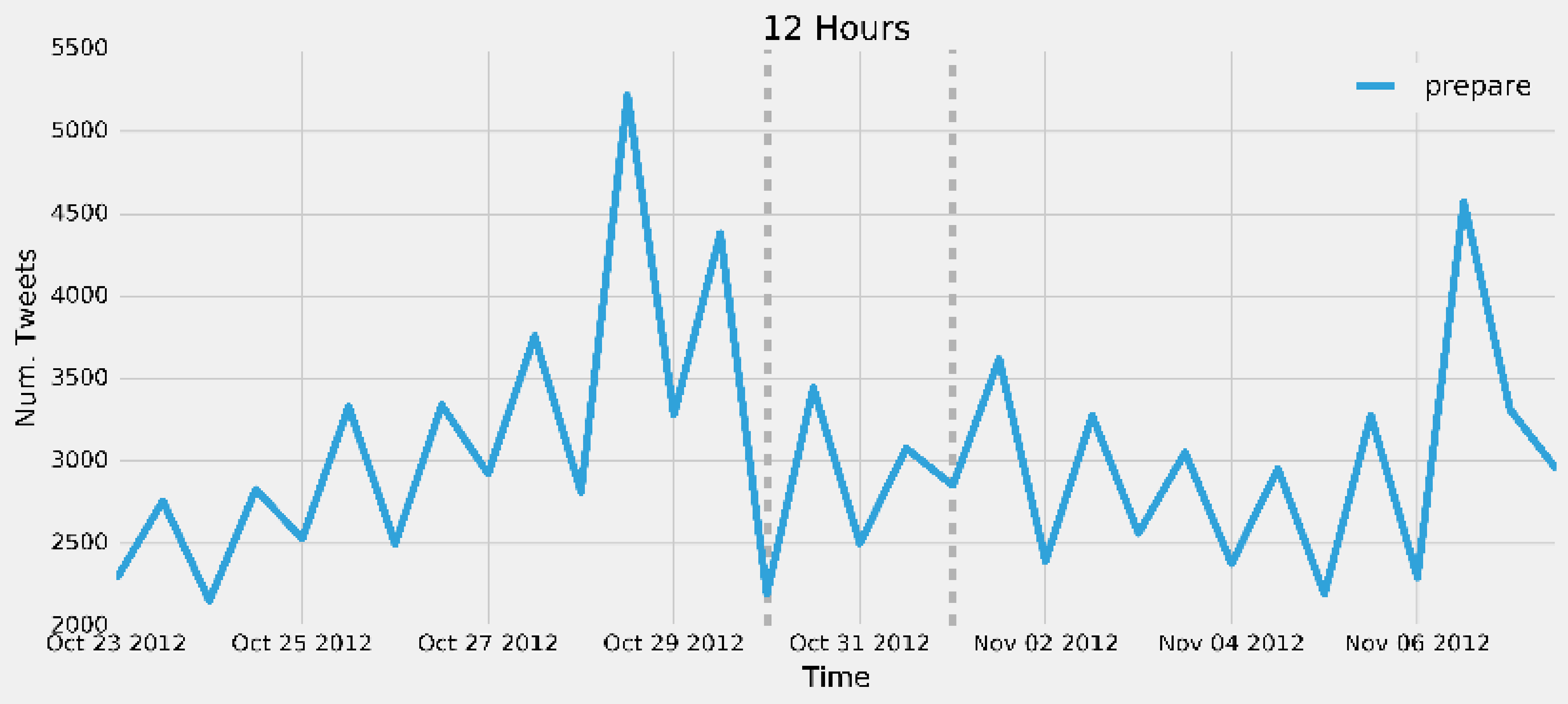
12 Hours
prepare
Num. Tweets
5500
5000
4500
4000
3500
3000
2500
2000
Oct 23 2012
Oct 25 2012
Oct 27 2012
Oct 29 2012
Oct 31 2012
Nov 02 2012
Nov 04 2012
Nov 06 2012
Time

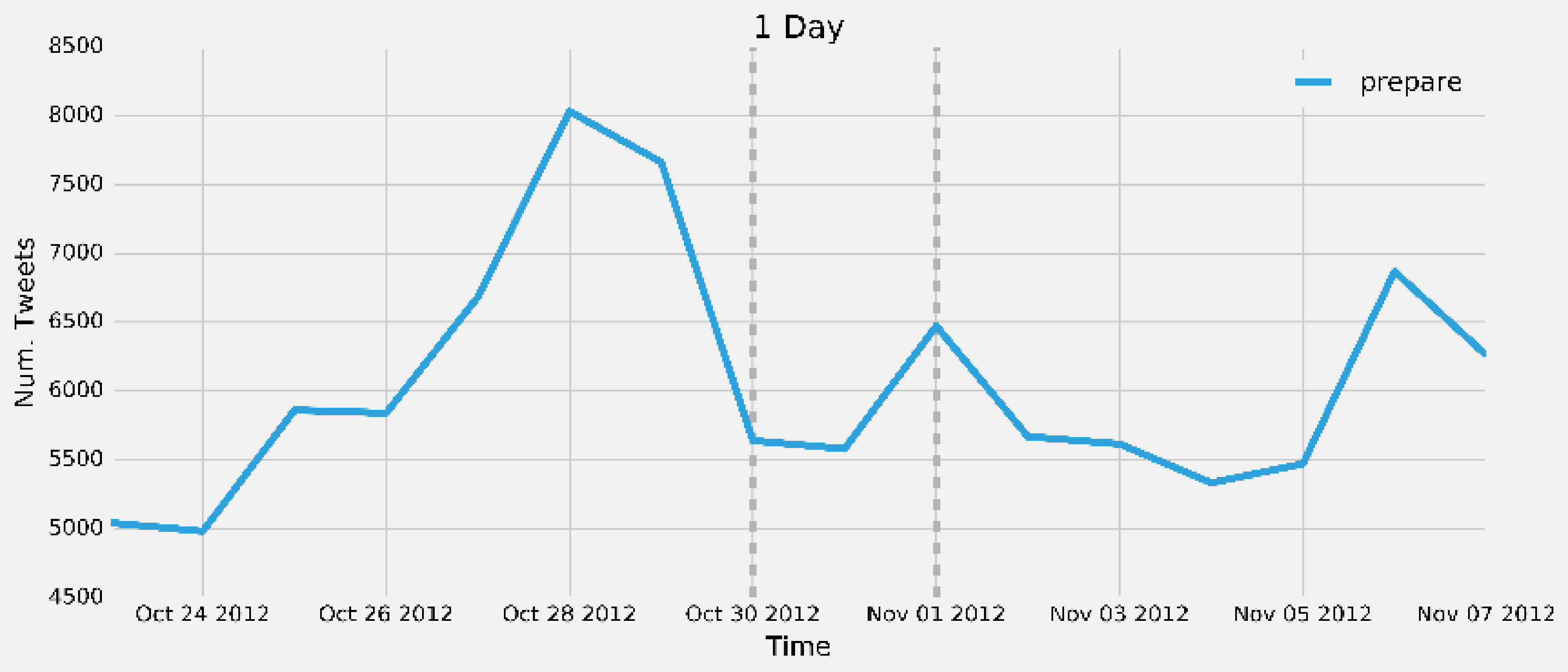

1 Day
prepare
Num. Tweets
8500
8000
7500
7000
6500
6000
5500
5000
4500
Oct 24 2012
Oct 26 2012
Oct 28 2012
Oct 30 2012
Nov 01 2012
Nov 03 2012
Nov 05 2012
Nov 07 2012
Time

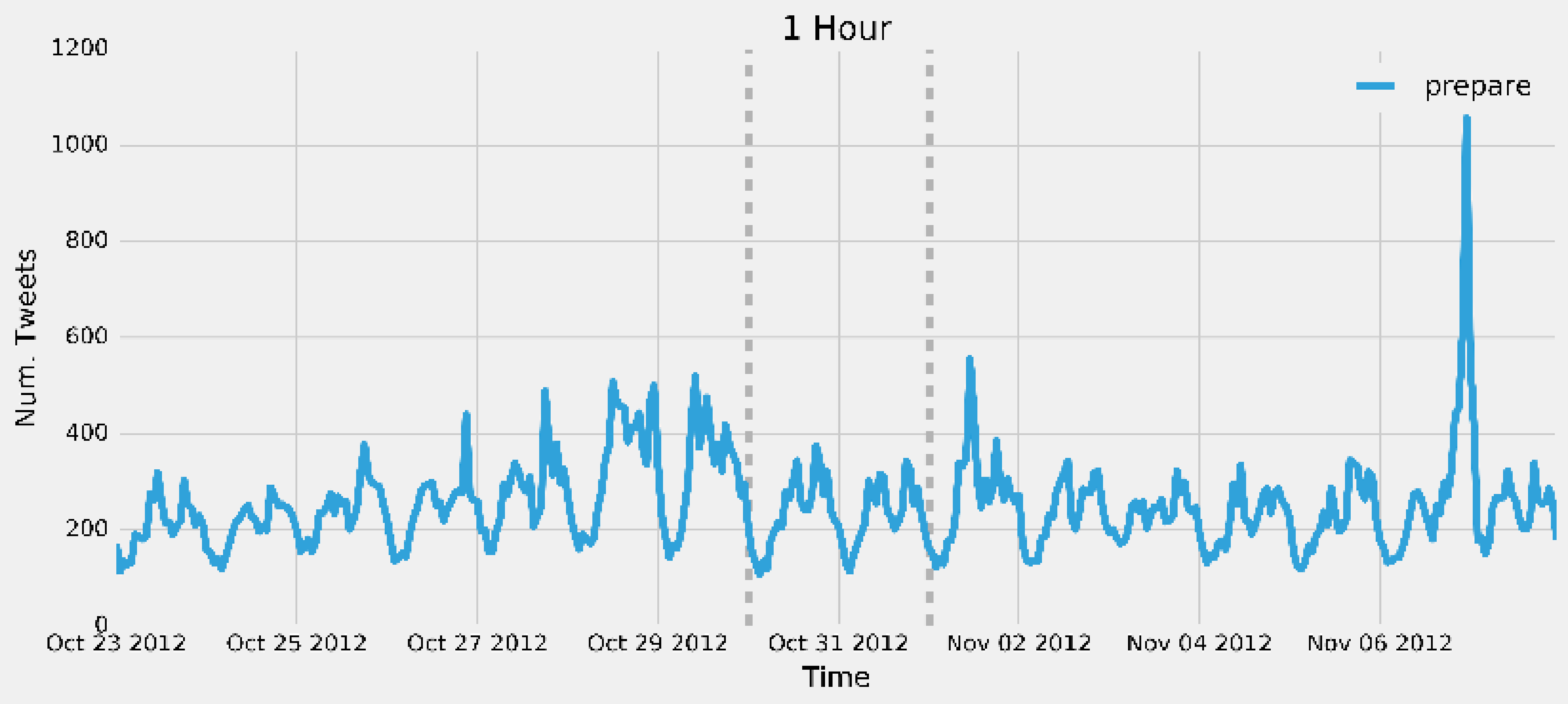
1 Hour
prepare
Num. Tweets
0
200
400
600
800
1000
1200
Oct 23 2012
Oct 25 2012
Oct 27 2012
Oct 29 2012
Oct 31 2012
Nov 02 2012
Nov 04 2012
Nov 06 2012
Time

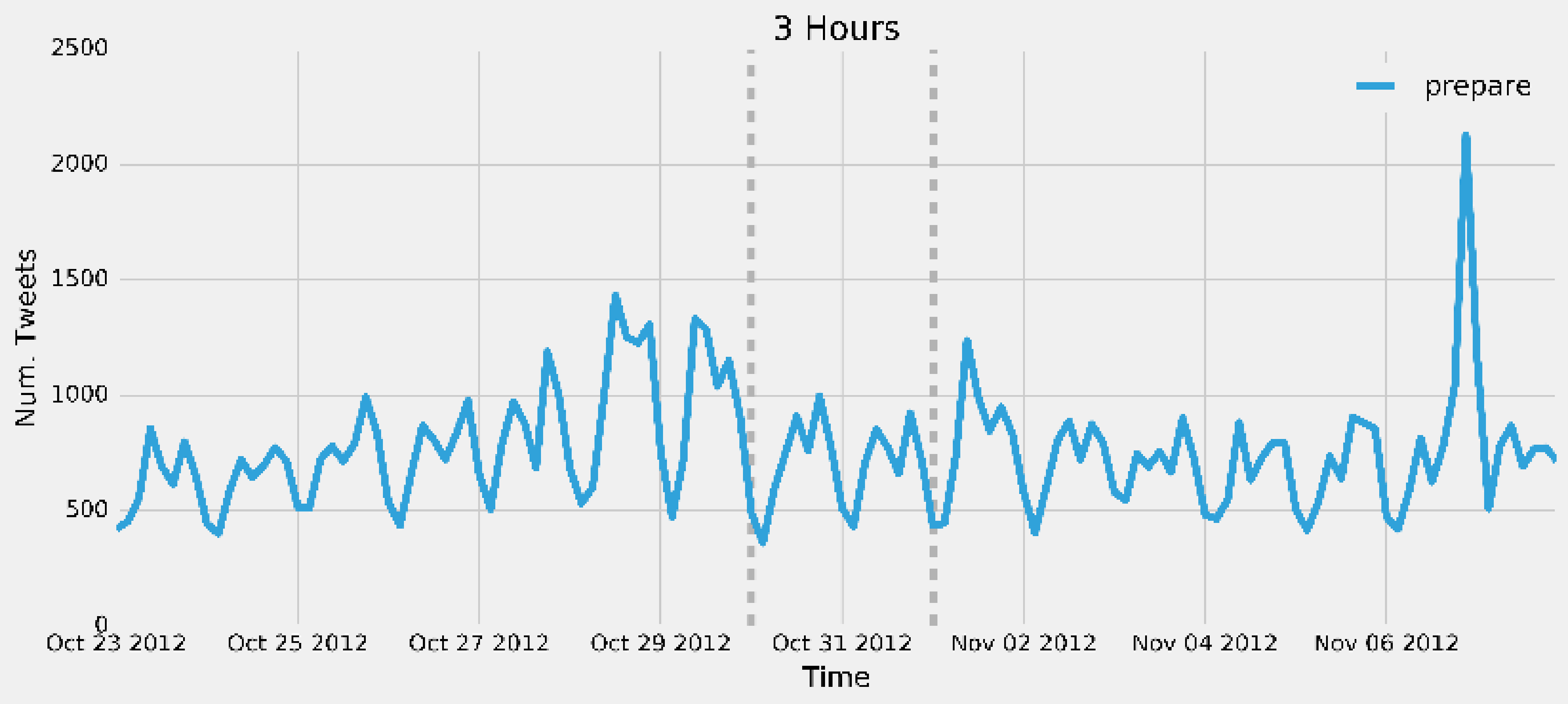
3 Hours
prepare
Num. Tweets
2500
2000
1500
1000
500
0
Oct 23 2012
Oct 25 2012
Oct 27 2012
Oct 29 2012
Oct 31 2012
Nov 02 2012
Nov 04 2012
Nov 06 2012
Time

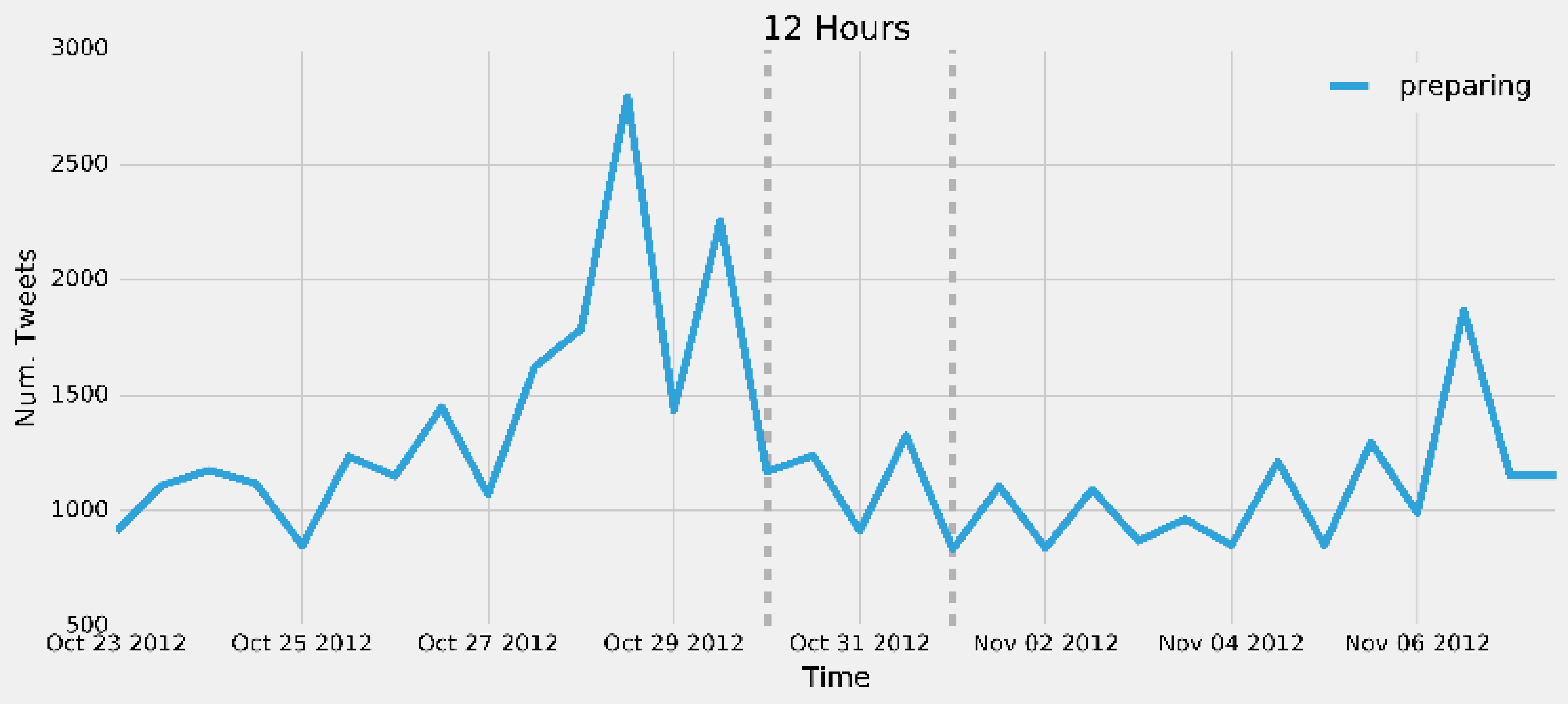

12 Hours
3000
2500
2000
1500
1000
500
Num. Tweets
preparing
Oct 23 2012
Oct 25 2012
Oct 27 2012
Oct 29 2012
Oct 31 2012
Nov 02 2012
Nov 04 2012
Nov 06 2012
Time

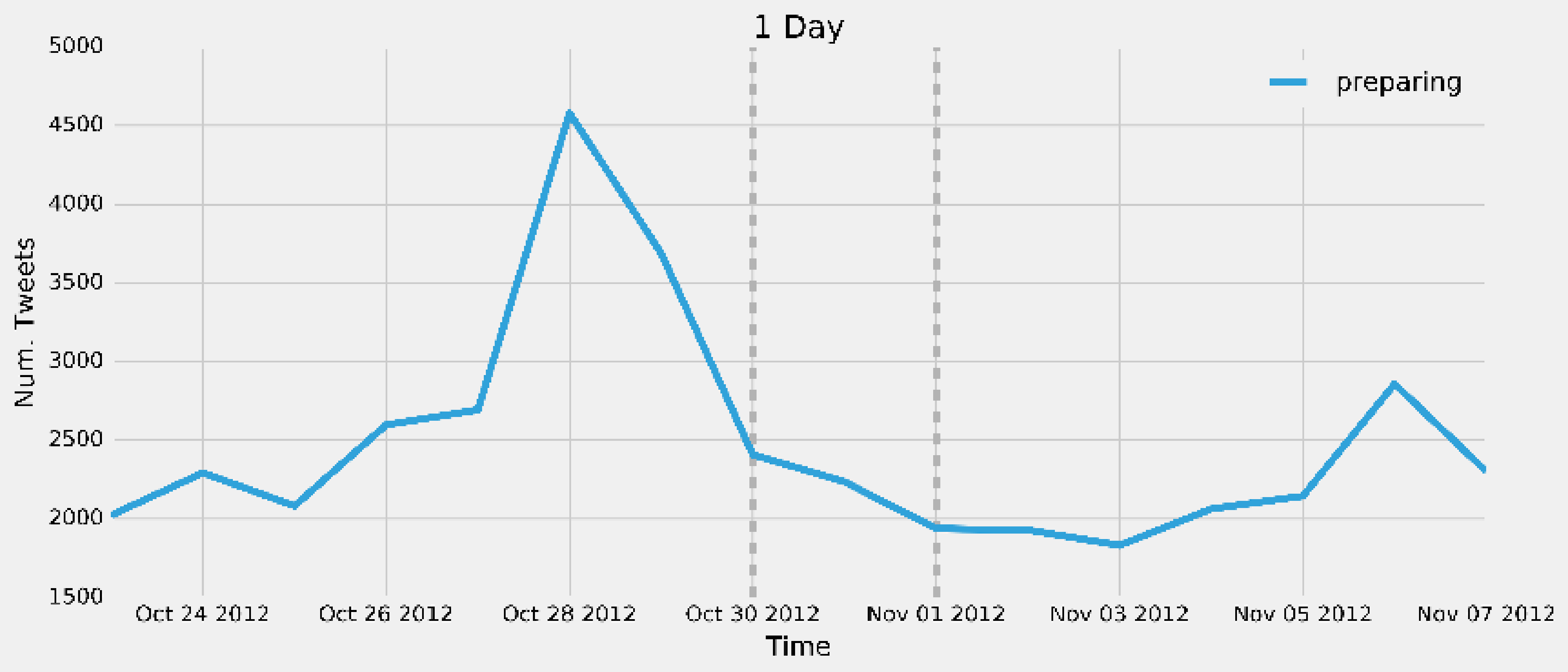
1 Day
preparing
Num. Tweets
5000
4500
4000
3500
3000
2500
2000
1500
Oct 24 2012
Oct 26 2012
Oct 28 2012
Oct 30 2012
Nov 01 2012
Nov 03 2012
Nov 05 2012
Nov 07 2012
Time

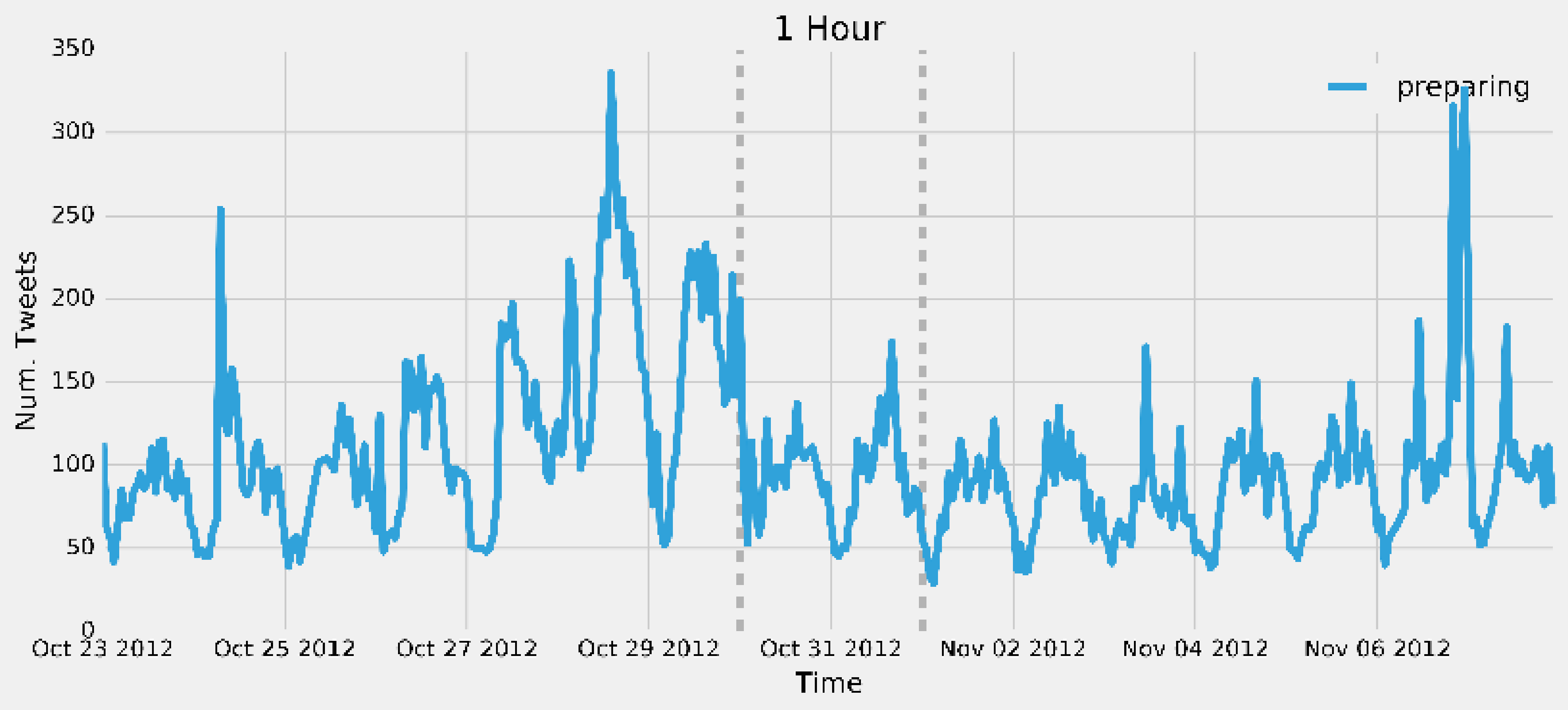
1 Hour
preparing
Num. Tweets
350
300
250
200
150
100
50
0
Oct 23 2012
Oct 25 2012
Oct 27 2012
Oct 29 2012
Oct 31 2012
Nov 02 2012
Nov 04 2012
Nov 06 2012
Time

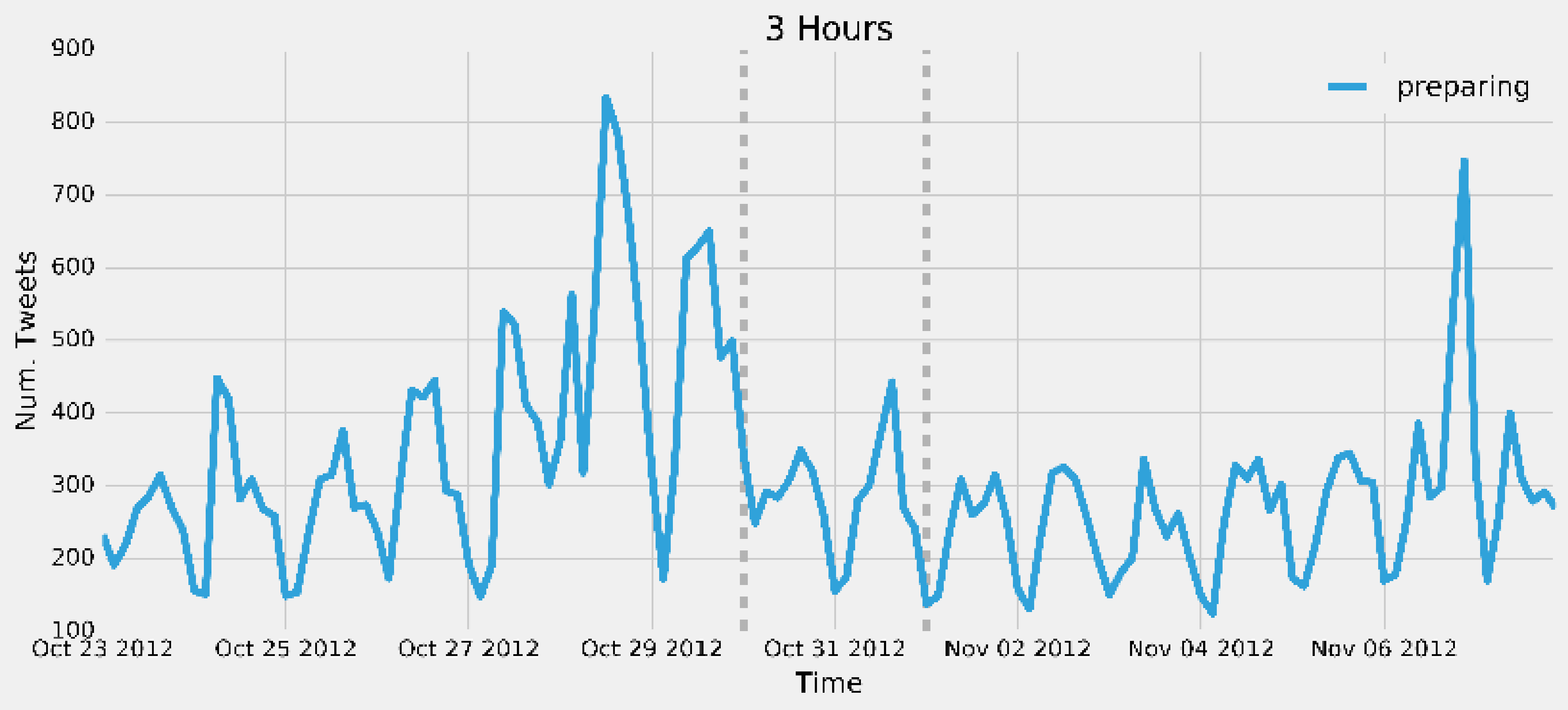

3 Hours
preparing
900
800
700
600
500
400
300
200
100
Num. Tweets
Oct 23 2012
Oct 25 2012
Oct 27 2012
Oct 29 2012
Oct 31 2012
Nov 02 2012
Nov 04 2012
Nov 06 2012
Time

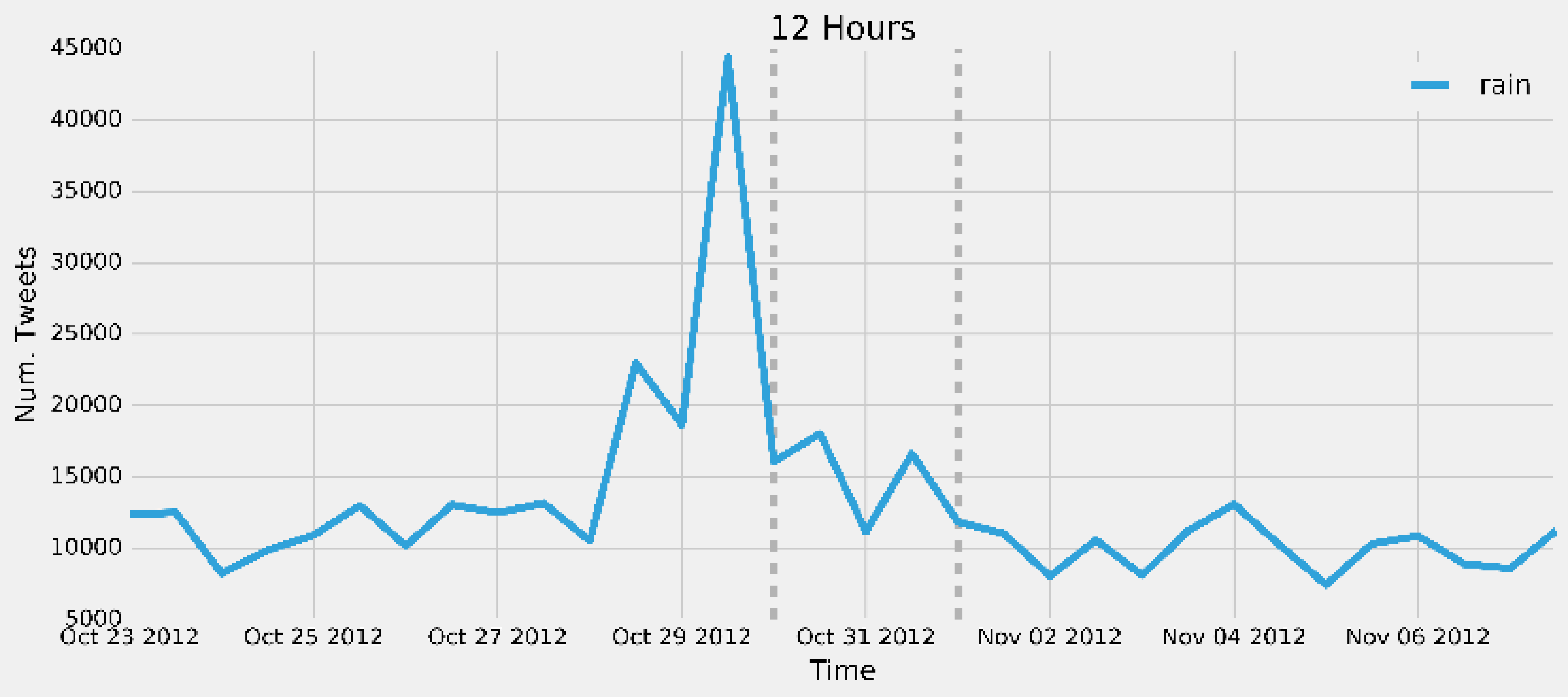
12 Hours
rain
Num. Tweets
45000
40000
35000
30000
25000
20000
15000
10000
5000
Oct 23 2012
Oct 25 2012
Oct 27 2012
Oct 29 2012
Oct 31 2012
Nov 02 2012
Nov 04 2012
Nov 06 2012
Time

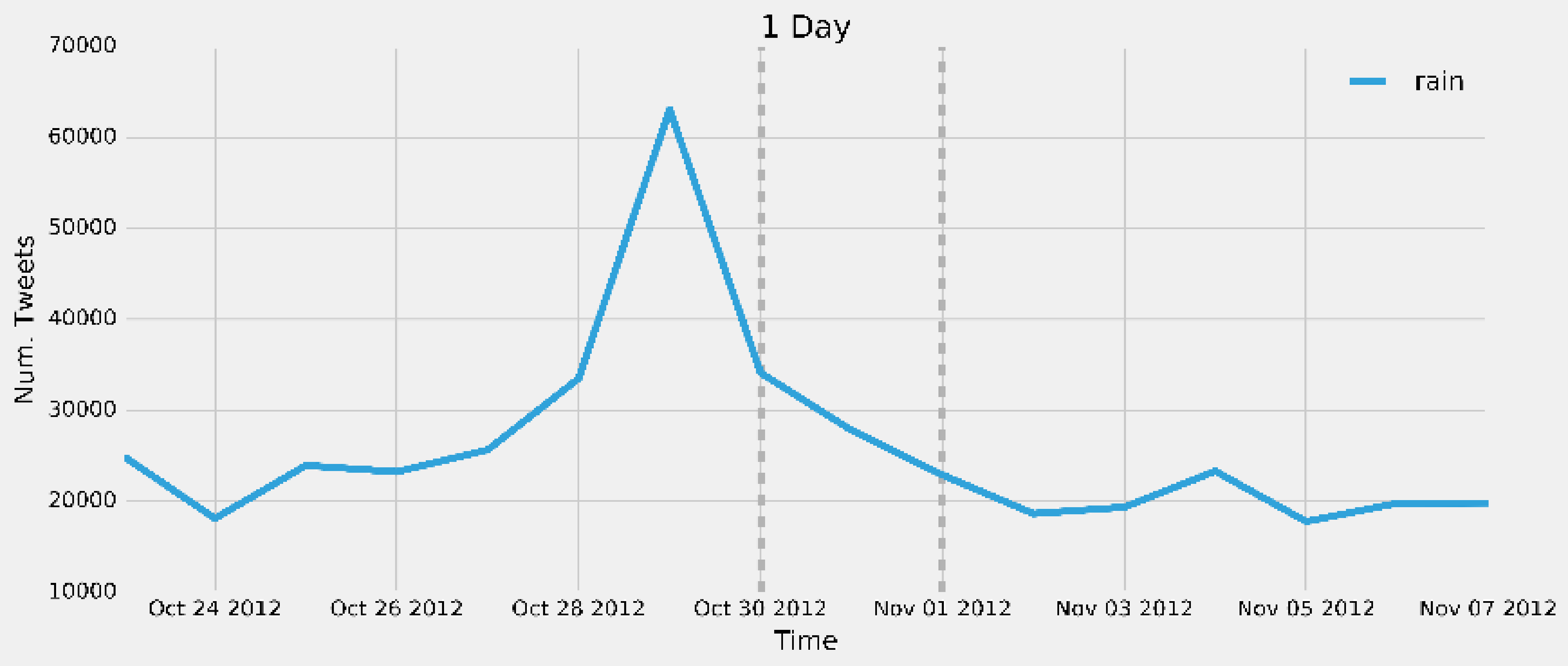
1 Day
rain
Num. Tweets
70000
60000
50000
40000
30000
20000
10000
Oct 24 2012
Oct 26 2012
Oct 28 2012
Oct 30 2012
Nov 01 2012
Nov 03 2012
Nov 05 2012
Nov 07 2012
Time

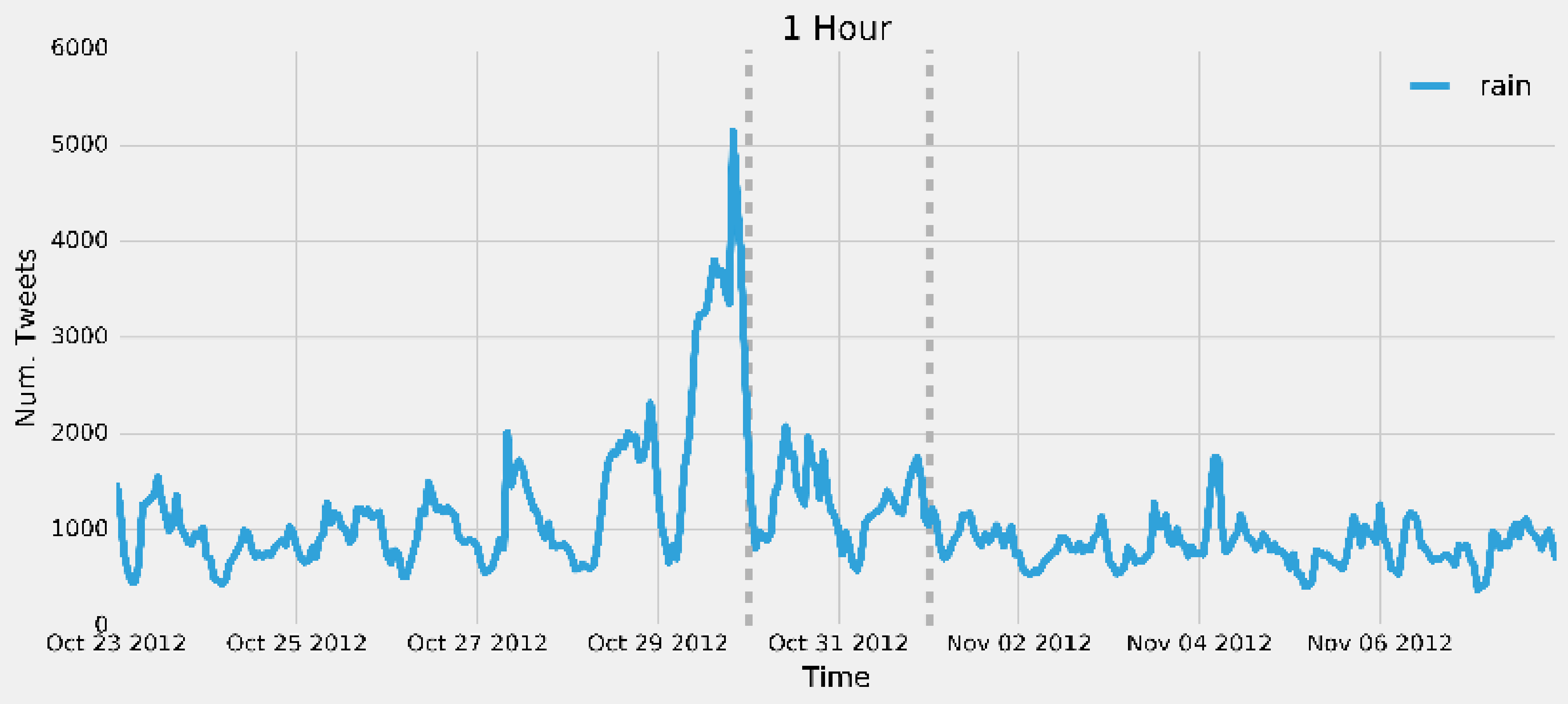

1 Hour
rain
Num. Tweets
Time
6000
5000
4000
3000
2000
1000
0
Oct 23 2012
Oct 25 2012
Oct 27 2012
Oct 29 2012
Oct 31 2012
Nov 02 2012
Nov 04 2012
Nov 06 2012

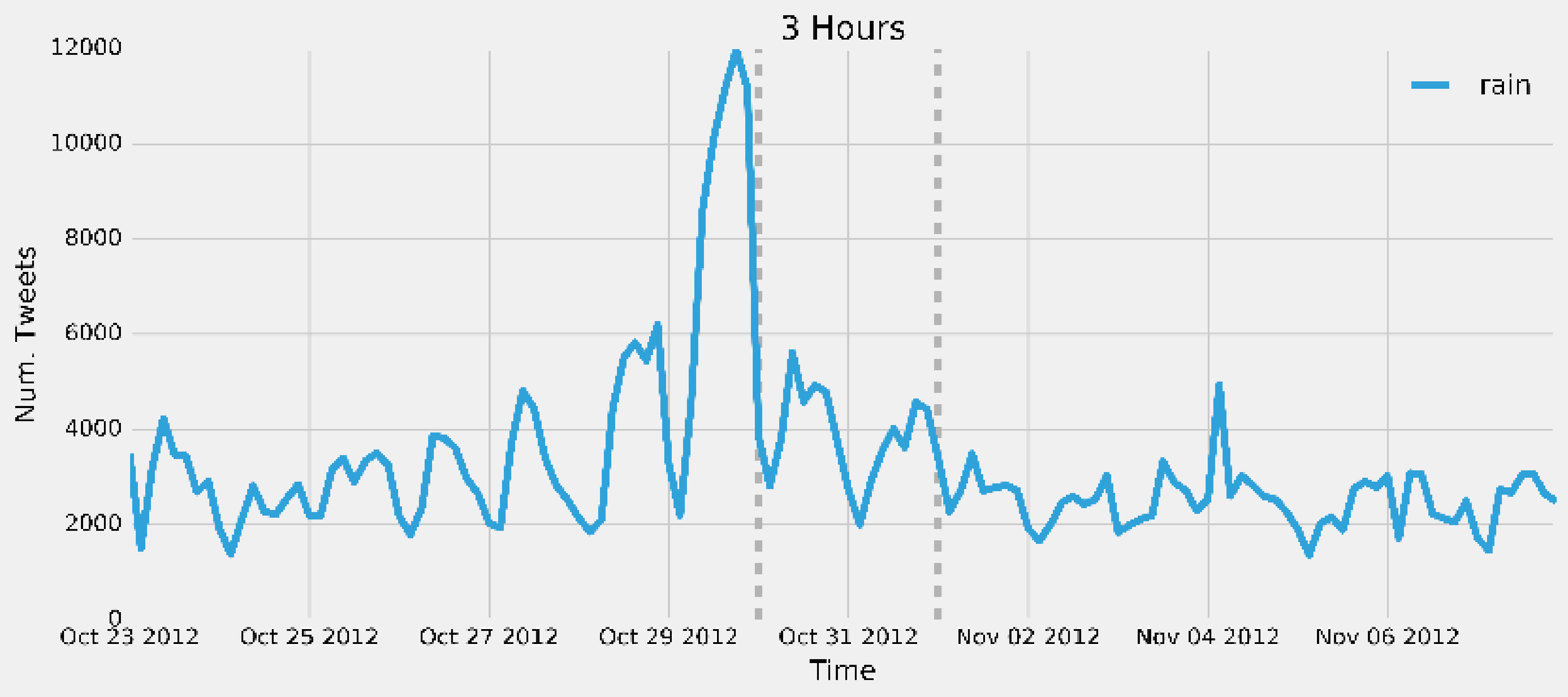

3 Hours
rain
Num. Tweets
Time
12000
10000
8000
6000
4000
2000
0
Oct 23 2012
Oct 25 2012
Oct 27 2012
Oct 29 2012
Oct 31 2012
Nov 02 2012
Nov 04 2012
Nov 06 2012

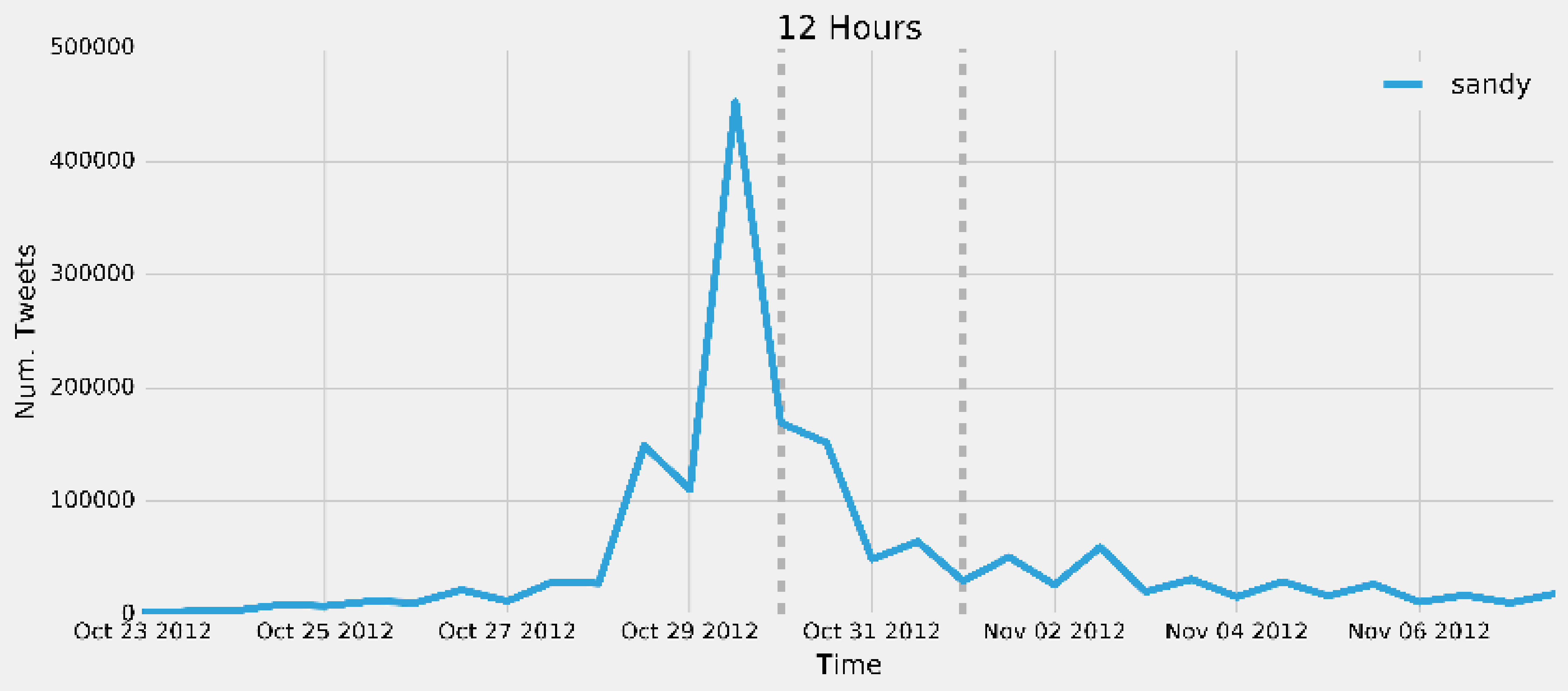
12 Hours
sandy
500000
400000
300000
200000
100000
0
Num. Tweets
Oct 23 2012
Oct 25 2012
Oct 27 2012
Oct 29 2012
Oct 31 2012
Nov 02 2012
Nov 04 2012
Nov 06 2012
Time

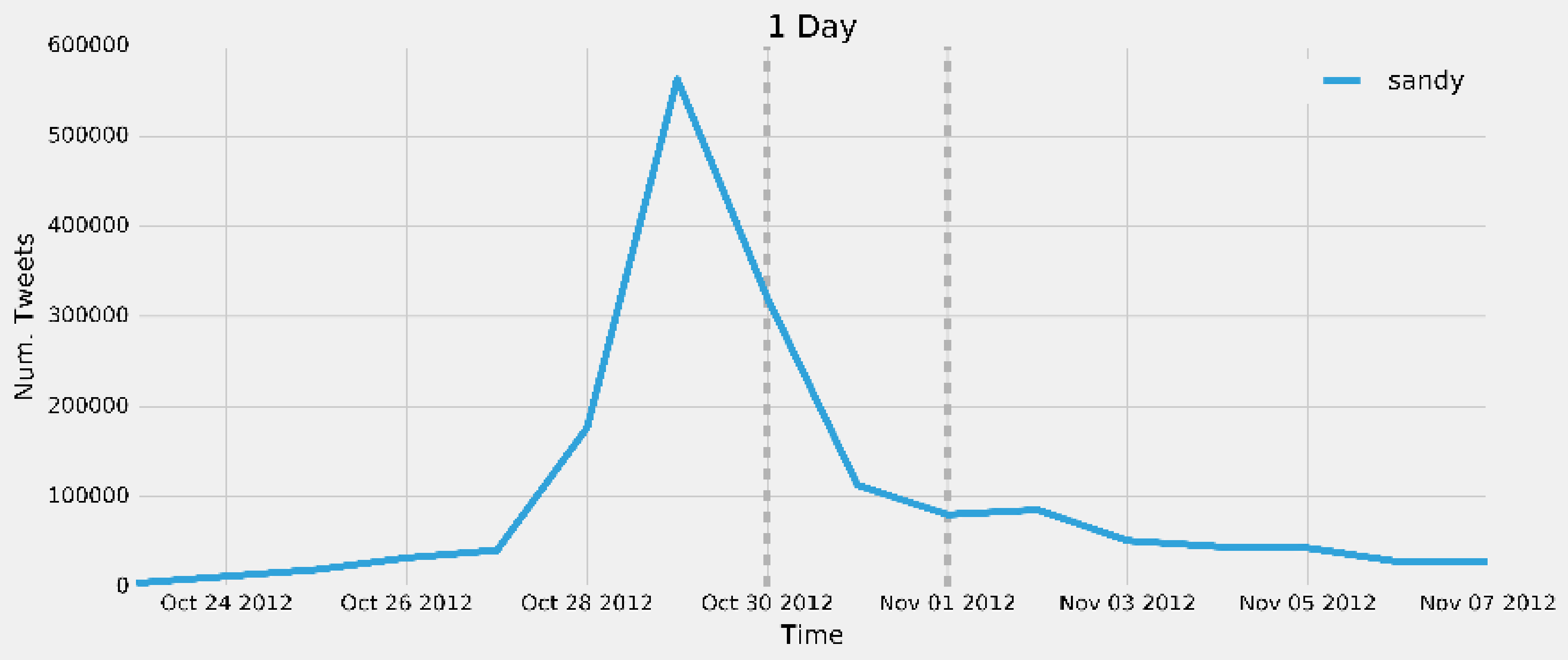
1 Day
600000
500000
400000
300000
200000
100000
0
Num. Tweets
Oct 24 2012
Oct 26 2012
Oct 28 2012
Oct 30 2012
Nov 01 2012
Nov 03 2012
Nov 05 2012
Nov 07 2012
Time
sandy

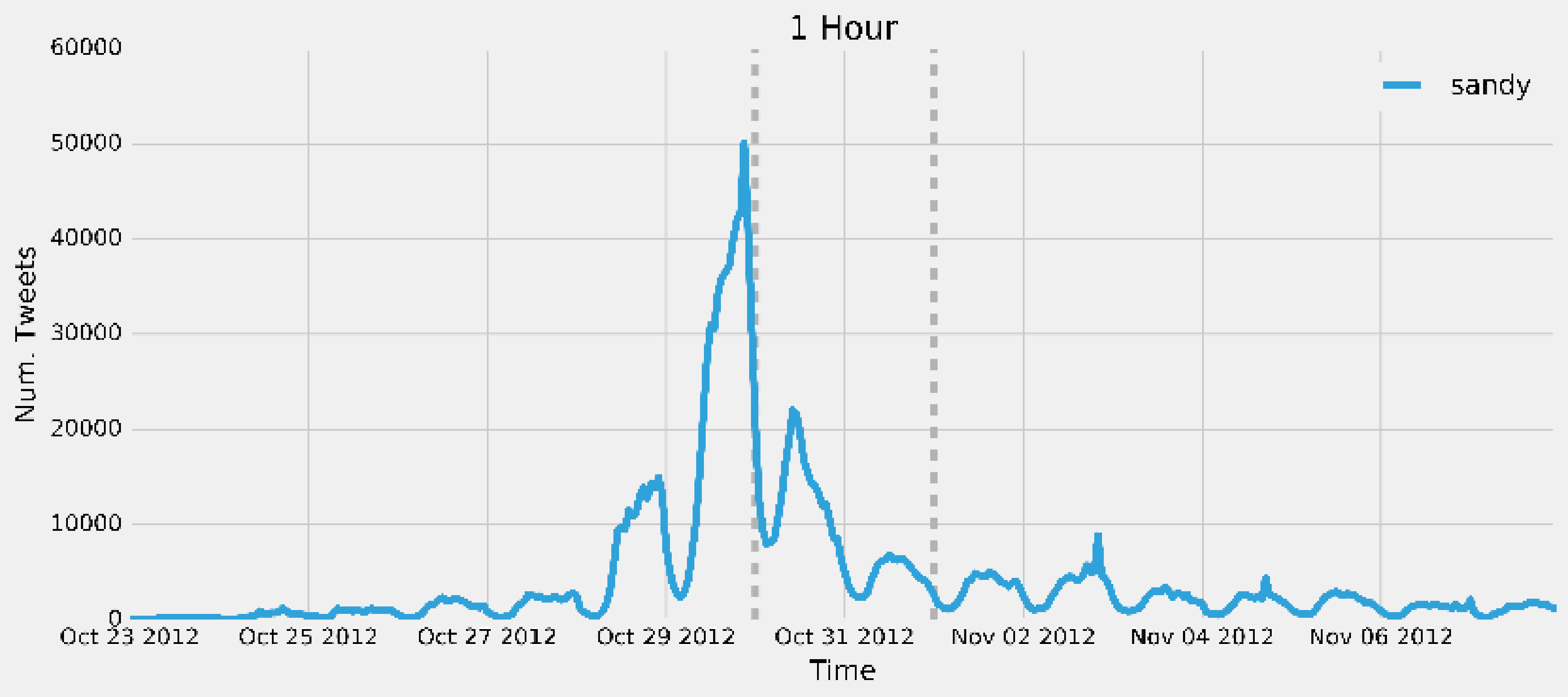
1 Hour
sandy
60000
50000
40000
30000
20000
10000
0
Num. Tweets
Oct 23 2012
Oct 25 2012
Oct 27 2012
Oct 29 2012
Oct 31 2012
Nov 02 2012
Nov 04 2012
Nov 06 2012
Time

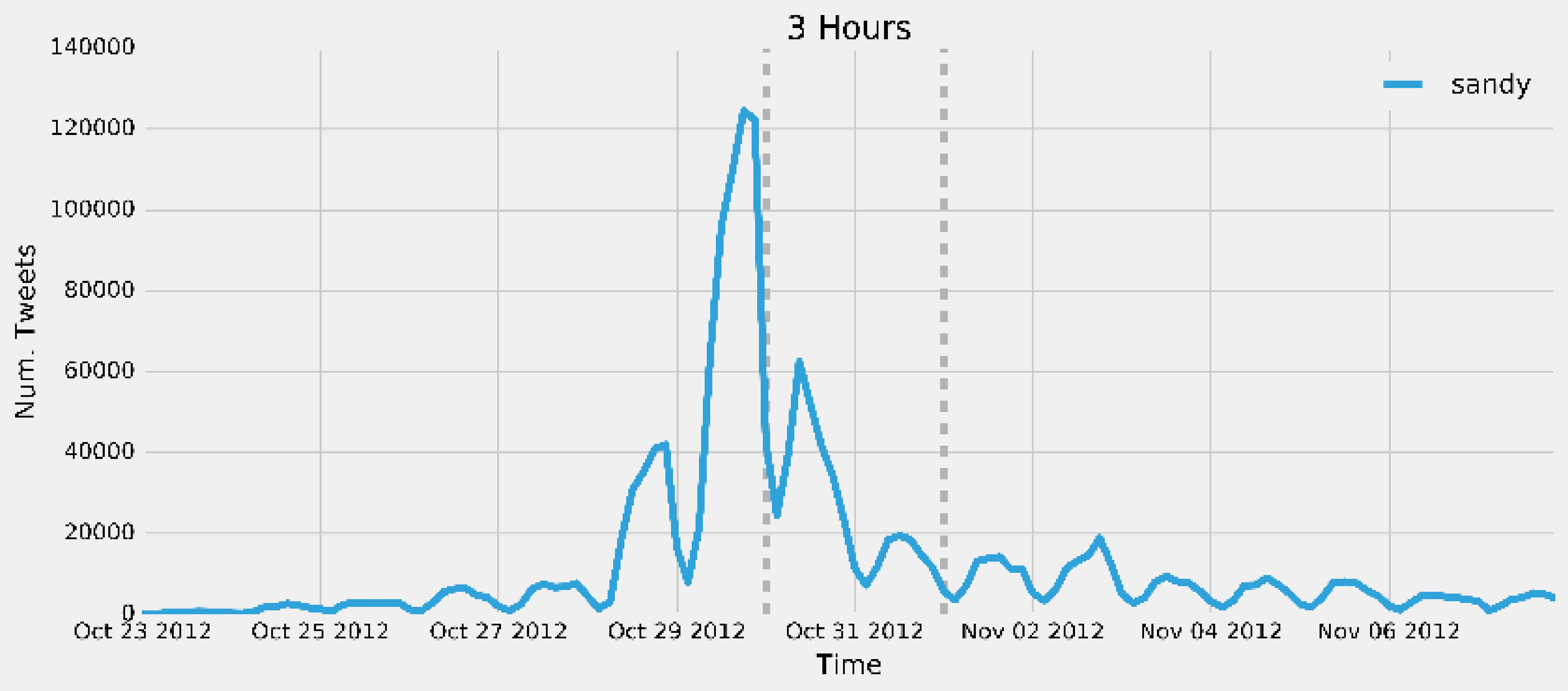

3 Hours
sandy
140000
120000
100000
80000
60000
40000
20000
0
Num. Tweets
Oct 23 2012
Oct 25 2012
Oct 27 2012
Oct 29 2012
Oct 31 2012
Nov 02 2012
Nov 04 2012
Nov 06 2012
Time

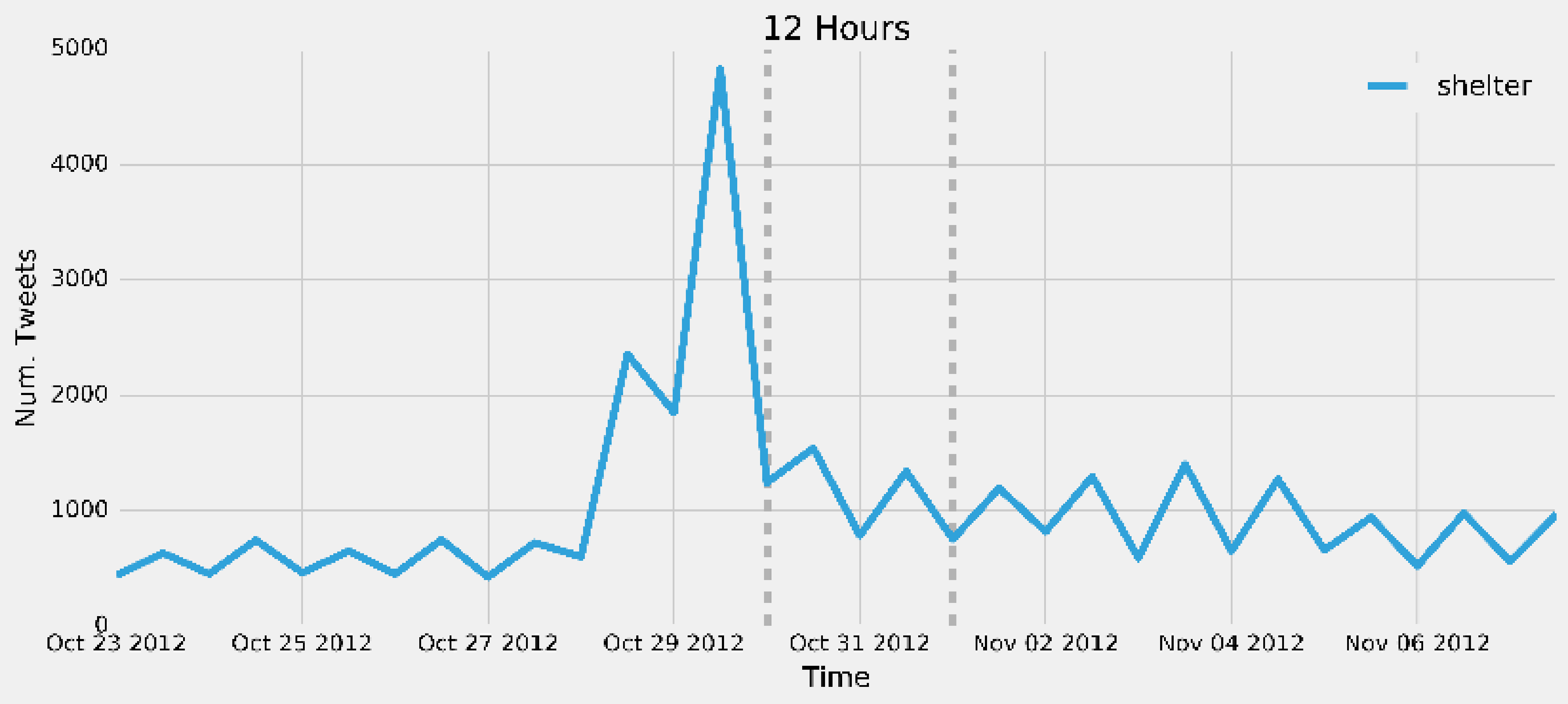
12 Hours
shelter
Num. Tweets
0
1000
2000
3000
4000
5000
Oct 23 2012
Oct 25 2012
Oct 27 2012
Oct 29 2012
Oct 31 2012
Nov 02 2012
Nov 04 2012
Nov 06 2012
Time

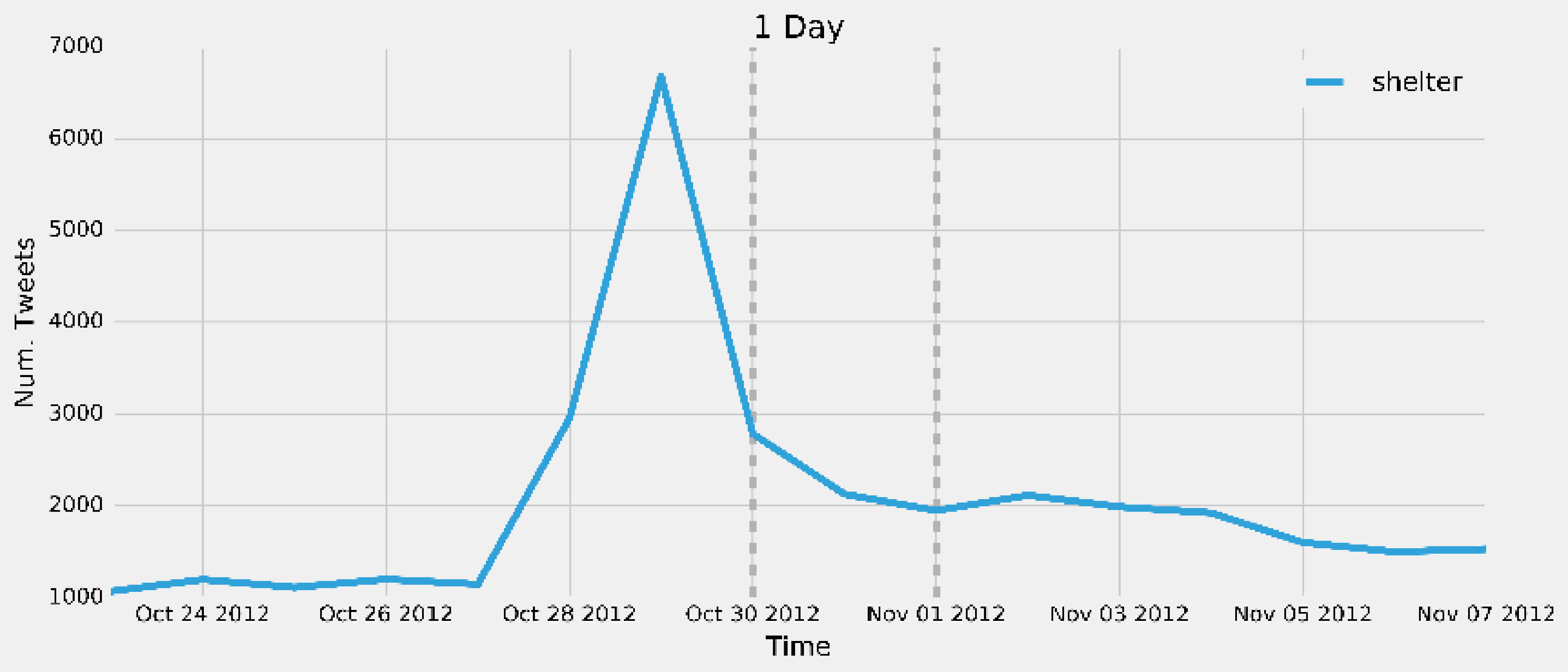

1 Day
shelter
Num. Tweets
7000
6000
5000
4000
3000
2000
1000
Oct 24 2012
Oct 26 2012
Oct 28 2012
Oct 30 2012
Nov 01 2012
Nov 03 2012
Nov 05 2012
Nov 07 2012
Time

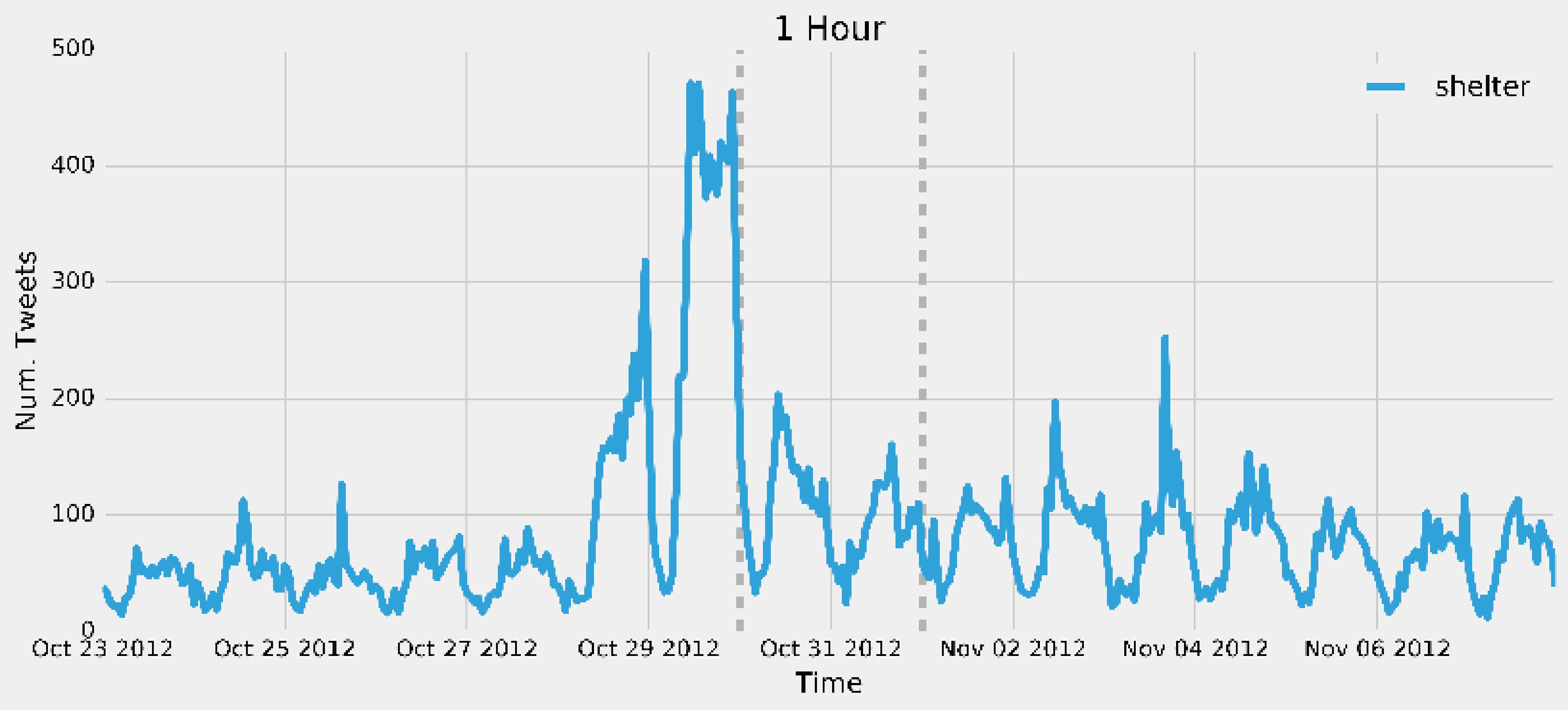
1 Hour
shelter
Num. Tweets
0
100
200
300
400
500
Oct 23 2012
Oct 25 2012
Oct 27 2012
Oct 29 2012
Oct 31 2012
Nov 02 2012
Nov 04 2012
Nov 06 2012
Time

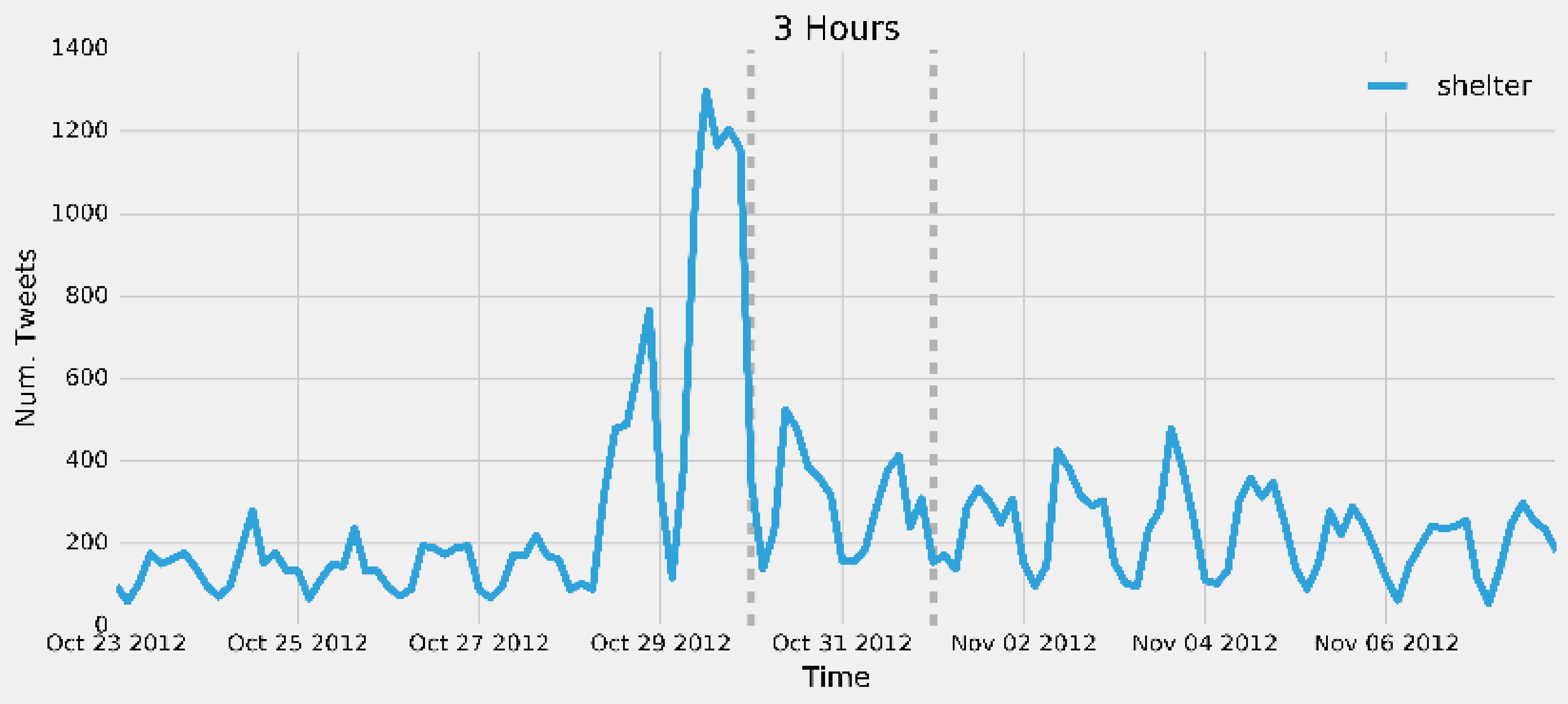
3 Hours
shelter
Num. Tweets
0
200
400
600
800
1000
1200
1400
Oct 23 2012
Oct 25 2012
Oct 27 2012
Oct 29 2012
Oct 31 2012
Nov 02 2012
Nov 04 2012
Nov 06 2012
Time

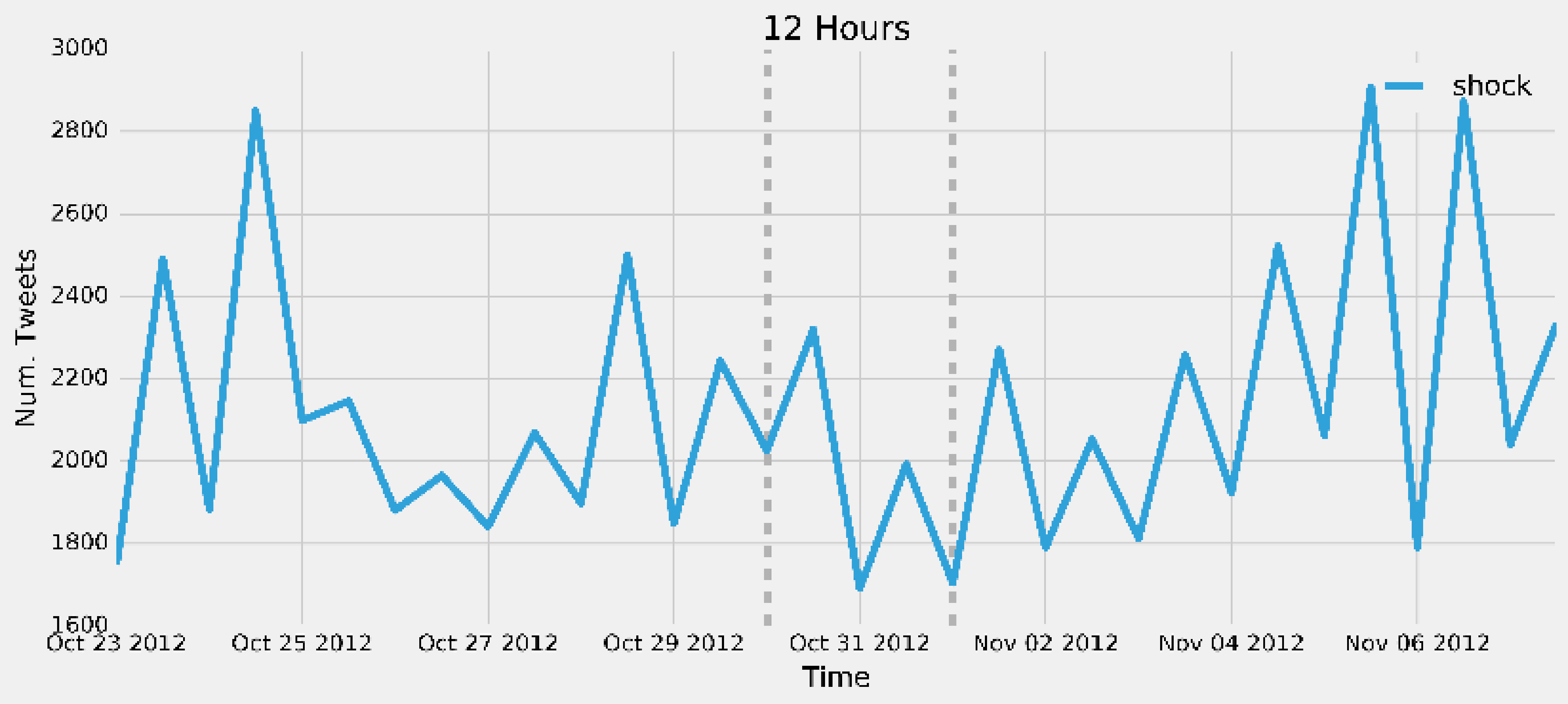
12 Hours
shock
Num. Tweets
3000
2800
2600
2400
2200
2000
1800
1600
Oct 23 2012
Oct 25 2012
Oct 27 2012
Oct 29 2012
Oct 31 2012
Nov 02 2012
Nov 04 2012
Nov 06 2012
Time

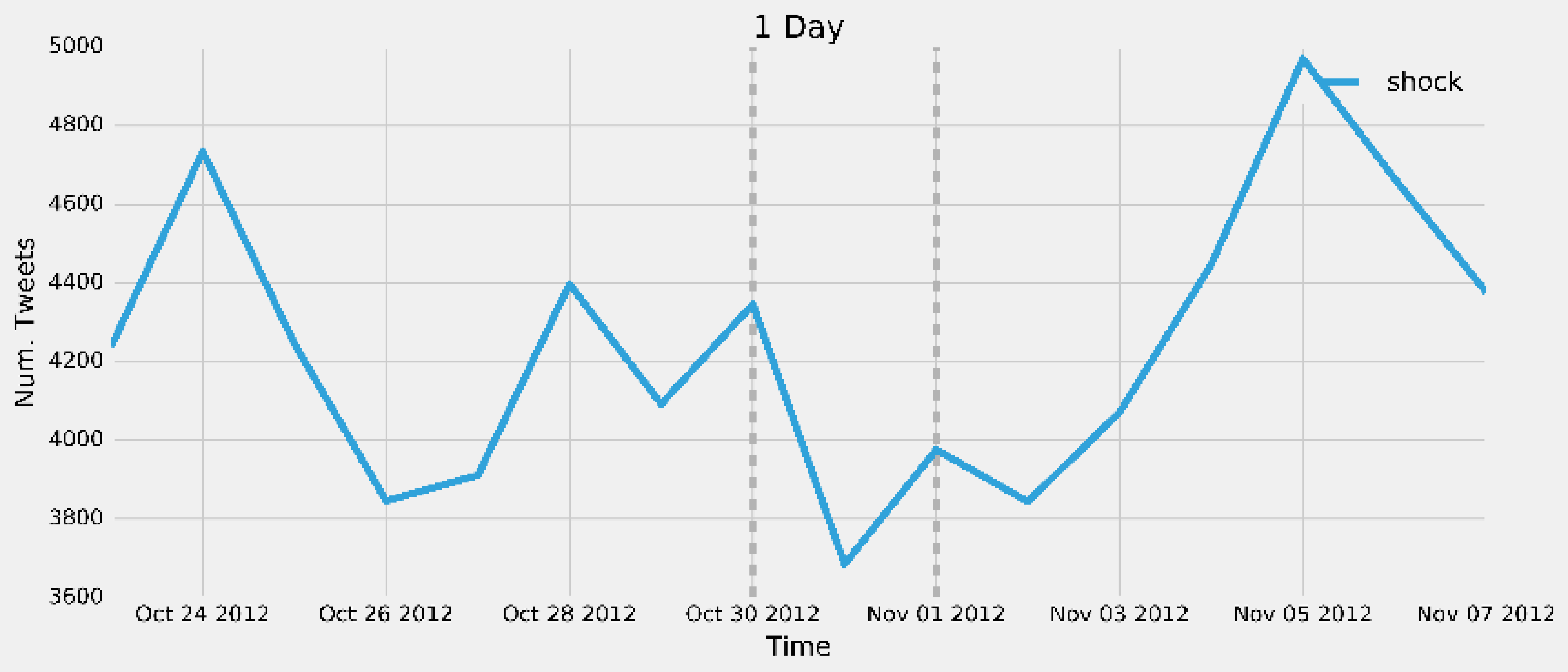

1 Day
shock
5000
4800
4600
4400
4200
4000
3800
3600
Num. Tweets
Oct 24 2012
Oct 26 2012
Oct 28 2012
Oct 30 2012
Nov 01 2012
Nov 03 2012
Nov 05 2012
Nov 07 2012
Time

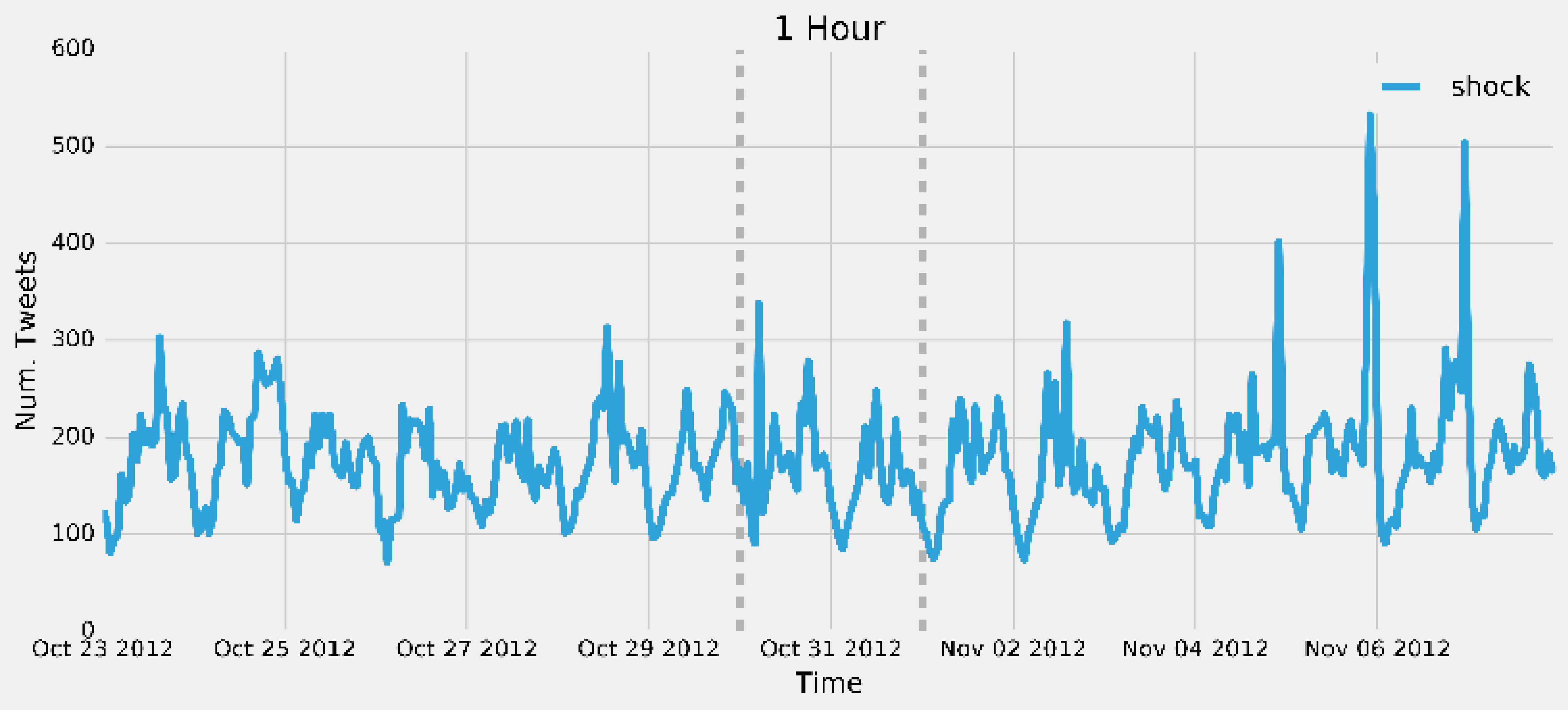

1 Hour
shock
Num. Tweets
0
100
200
300
400
500
600
Oct 23 2012
Oct 25 2012
Oct 27 2012
Oct 29 2012
Oct 31 2012
Nov 02 2012
Nov 04 2012
Nov 06 2012
Time

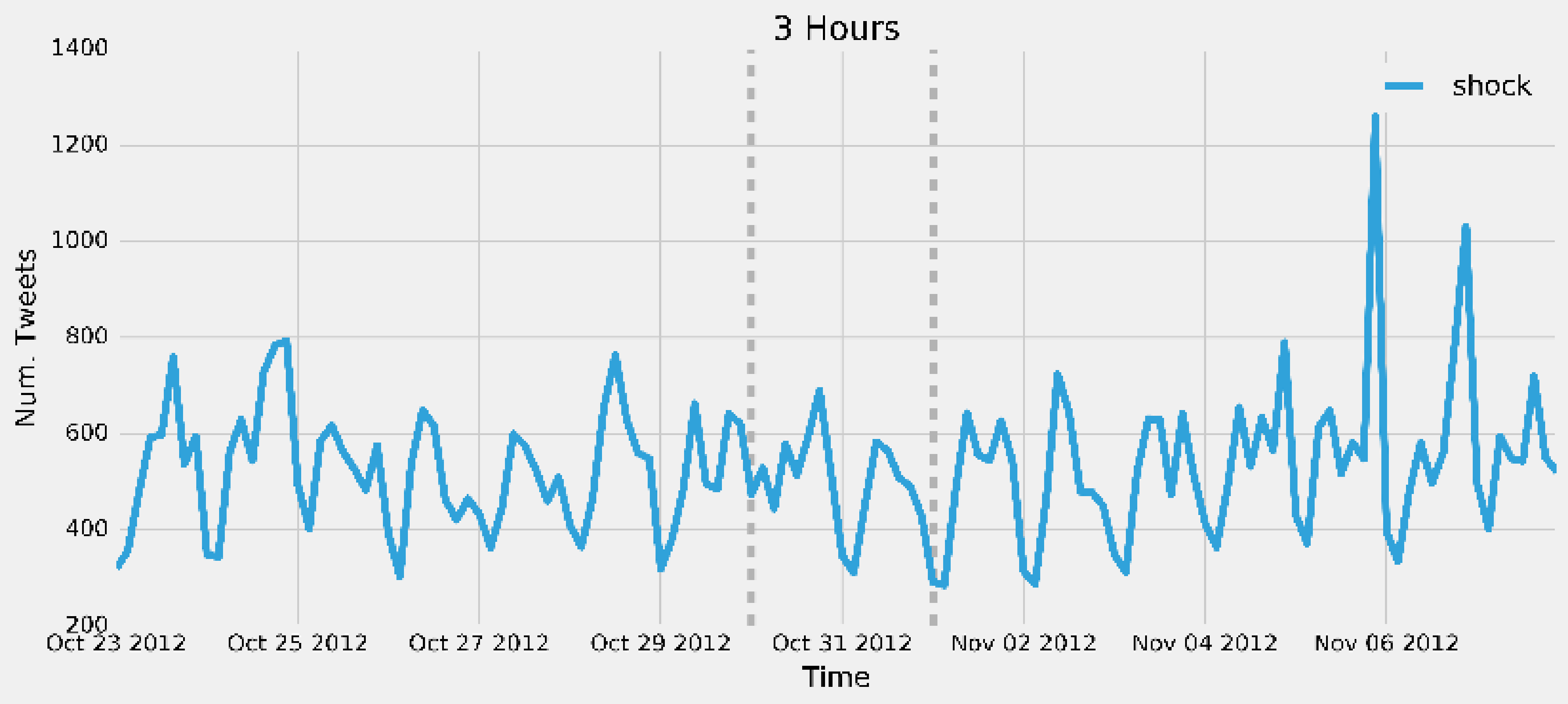
3 Hours
shock
1400
1200
1000
800
600
400
200
Num. Tweets
Oct 23 2012
Oct 25 2012
Oct 27 2012
Oct 29 2012
Oct 31 2012
Nov 02 2012
Nov 04 2012
Nov 06 2012
Time

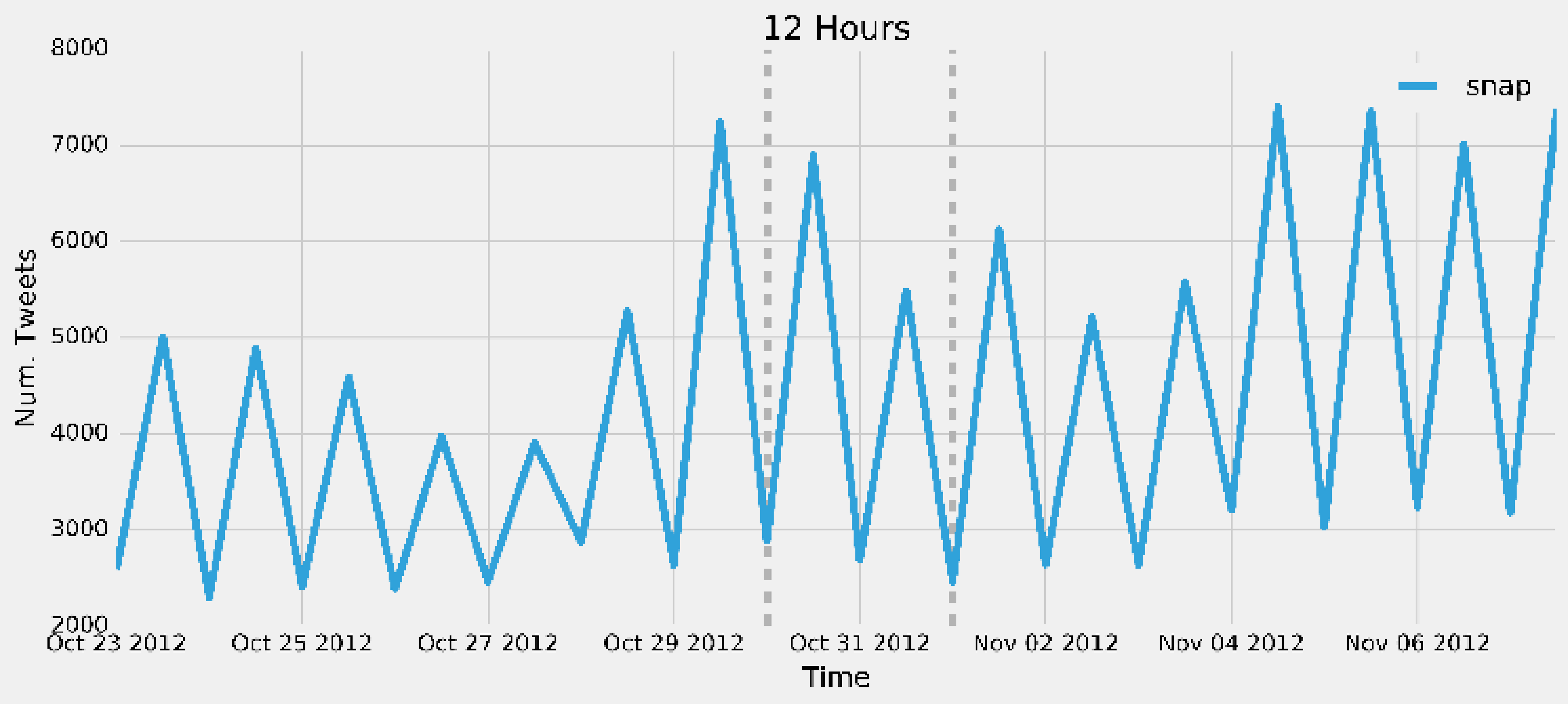
12 Hours
snap
Num. Tweets
8000
7000
6000
5000
4000
3000
2000
Oct 23 2012
Oct 25 2012
Oct 27 2012
Oct 29 2012
Oct 31 2012
Nov 02 2012
Nov 04 2012
Nov 06 2012
Time

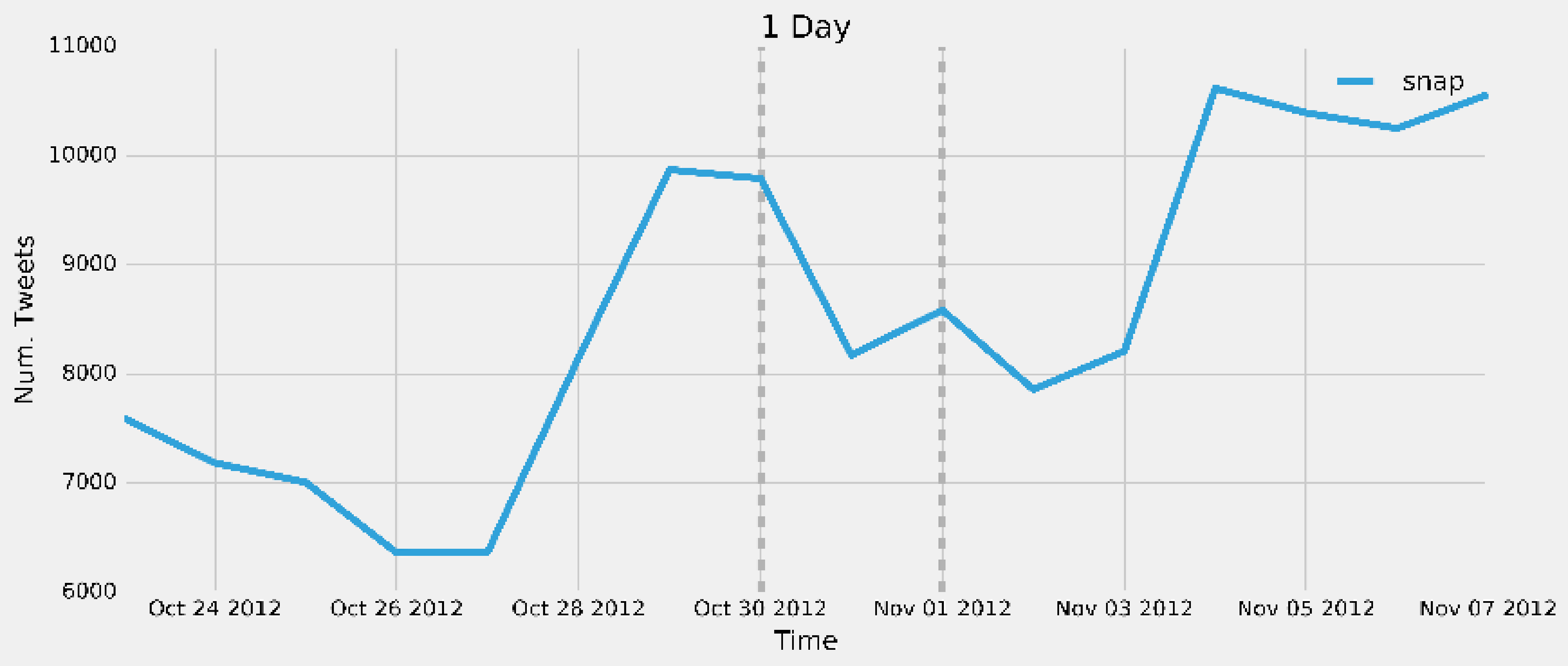

1 Day
Num. Tweets
11000
10000
9000
8000
7000
6000
snap
Oct 24 2012
Oct 26 2012
Oct 28 2012
Oct 30 2012
Nov 01 2012
Nov 03 2012
Nov 05 2012
Nov 07 2012
Time

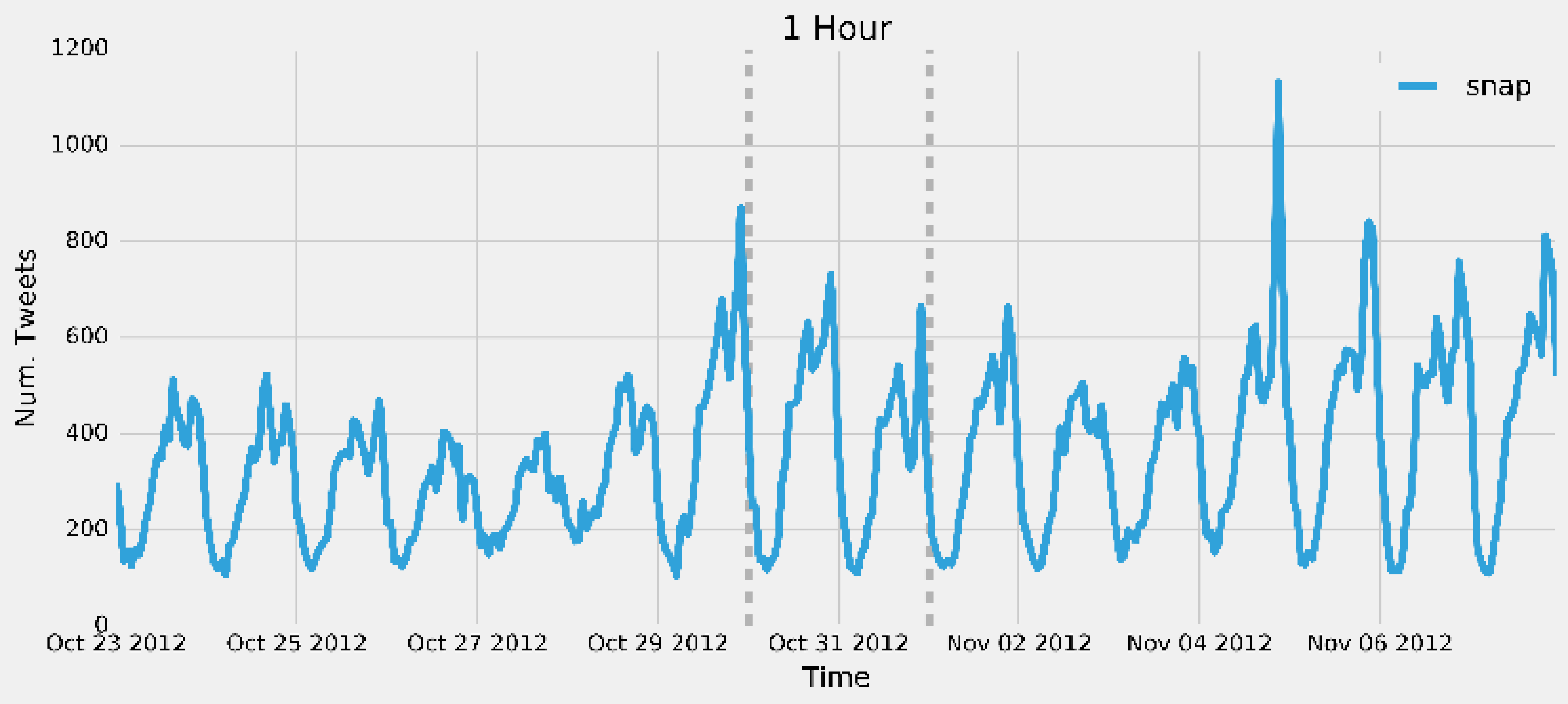

1 Hour
snap
Num. Tweets
0
200
400
600
800
1000
1200
Oct 23 2012
Oct 25 2012
Oct 27 2012
Oct 29 2012
Oct 31 2012
Nov 02 2012
Nov 04 2012
Nov 06 2012
Time

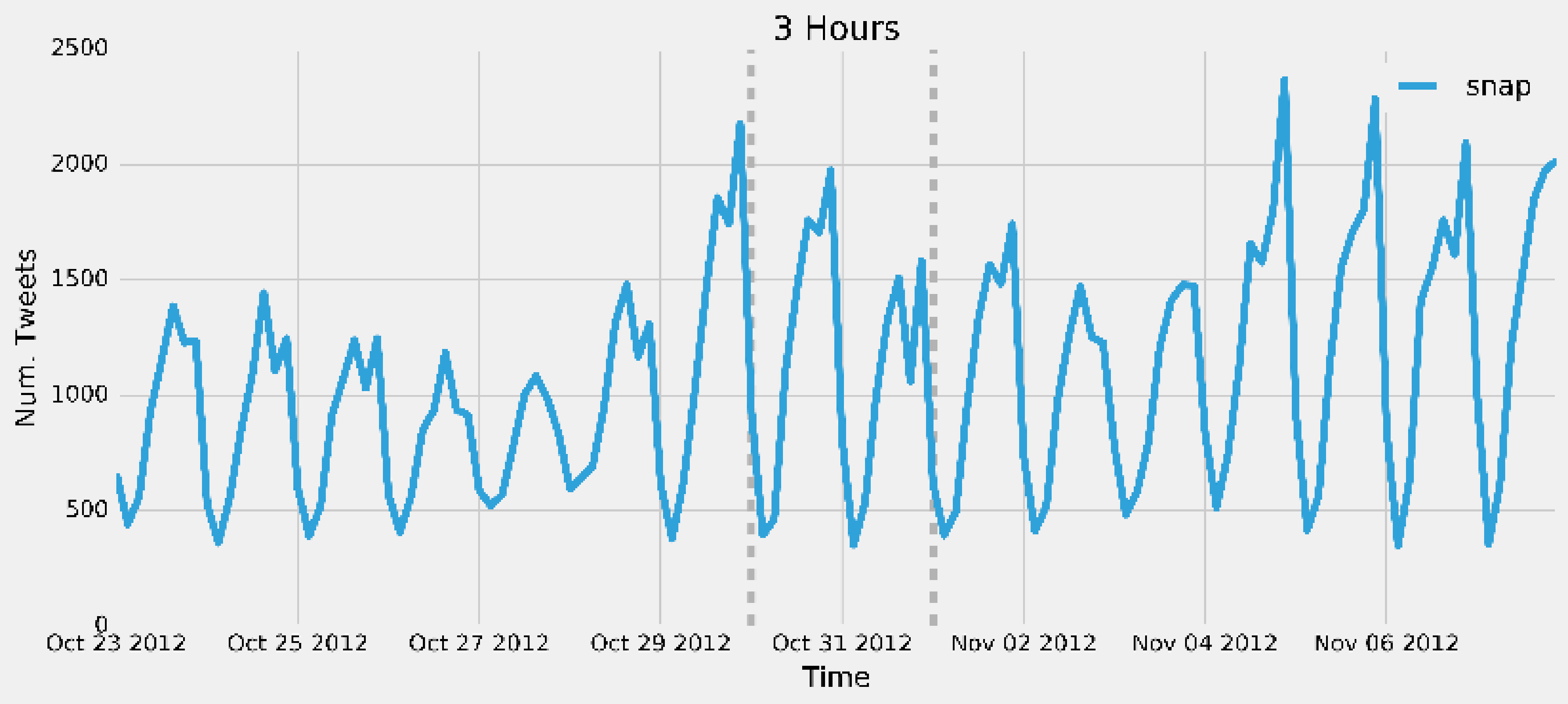

3 Hours
snap
Num. Tweets
2500
2000
1500
1000
500
0
Oct 23 2012
Oct 25 2012
Oct 27 2012
Oct 29 2012
Oct 31 2012
Nov 02 2012
Nov 04 2012
Nov 06 2012
Time

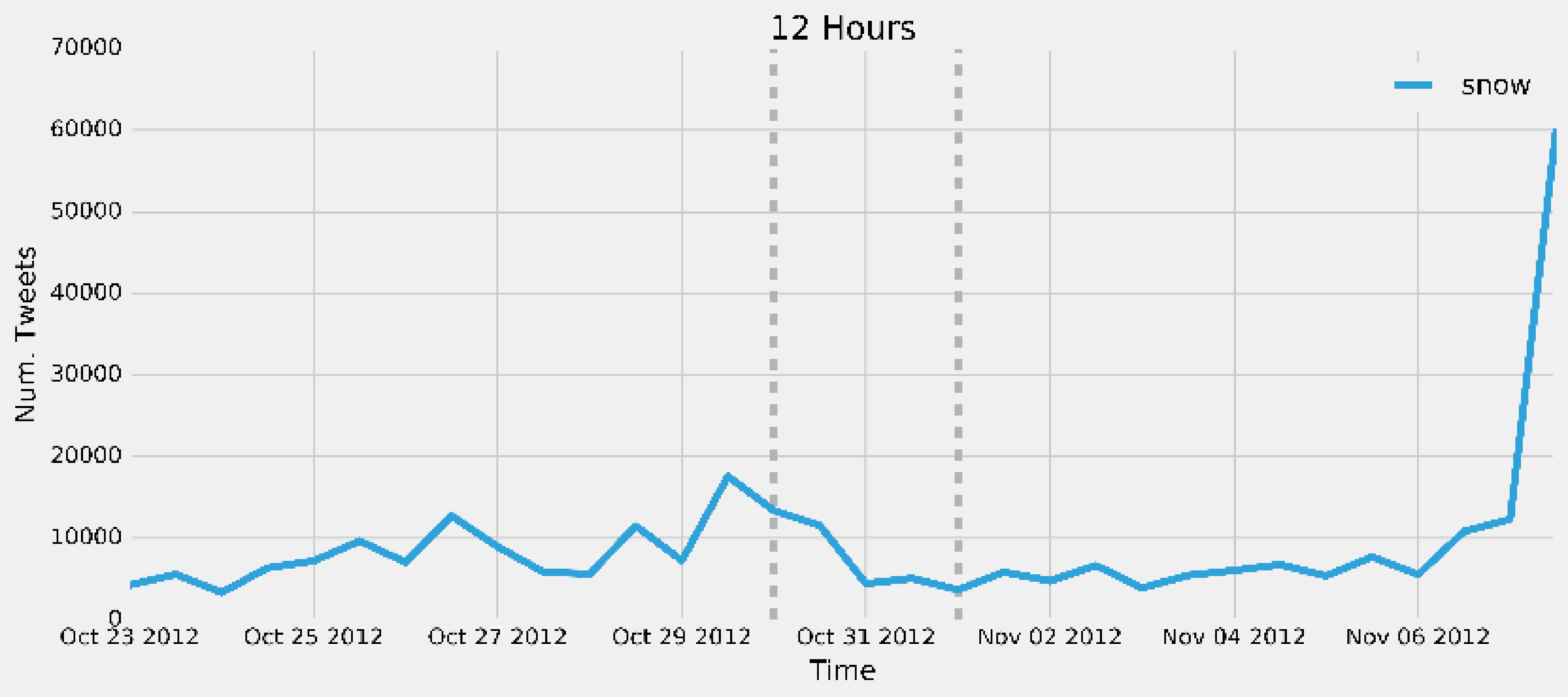
12 Hours
snow
70000
60000
50000
40000
30000
20000
10000
0
Num. Tweets
Oct 23 2012
Oct 25 2012
Oct 27 2012
Oct 29 2012
Oct 31 2012
Nov 02 2012
Nov 04 2012
Nov 06 2012
Time

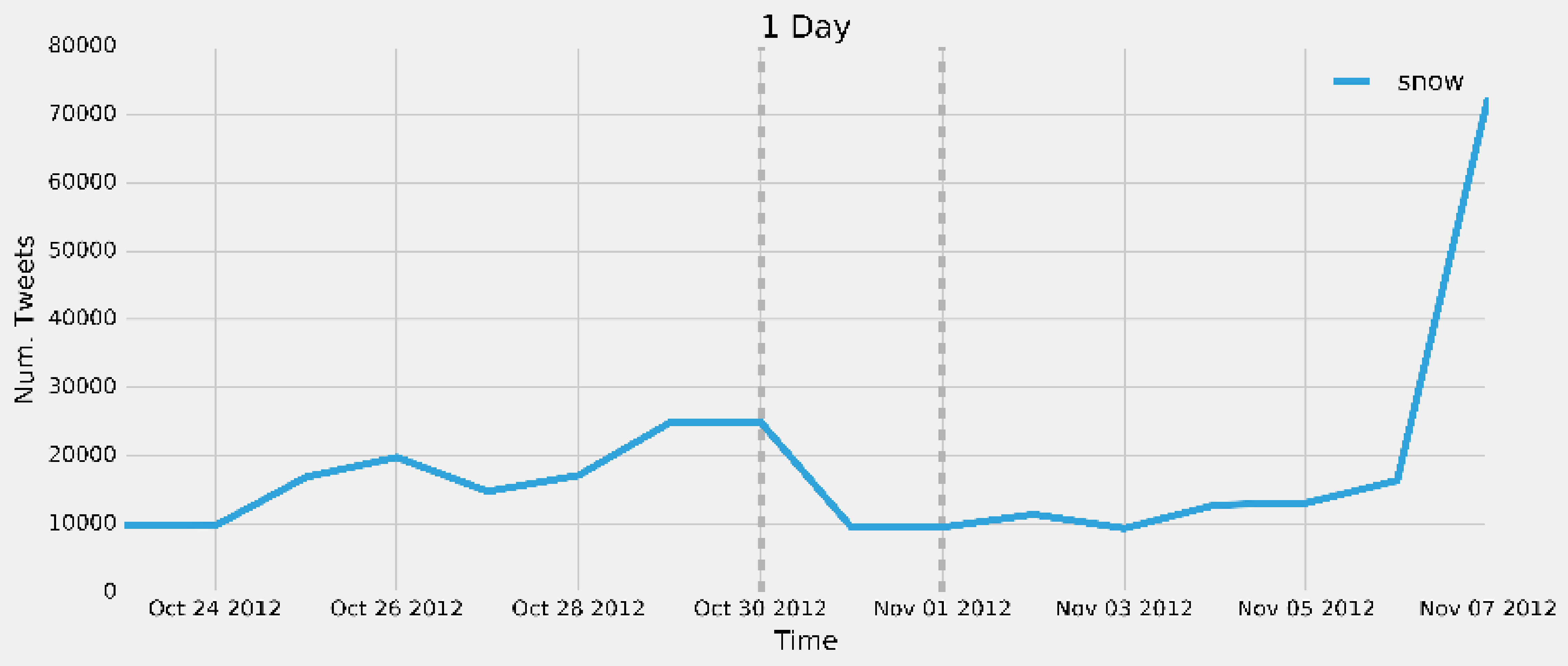
1 Day
snow
Num. Tweets
0
10000
20000
30000
40000
50000
60000
70000
80000
Oct 24 2012
Oct 26 2012
Oct 28 2012
Oct 30 2012
Nov 01 2012
Nov 03 2012
Nov 05 2012
Nov 07 2012
Time

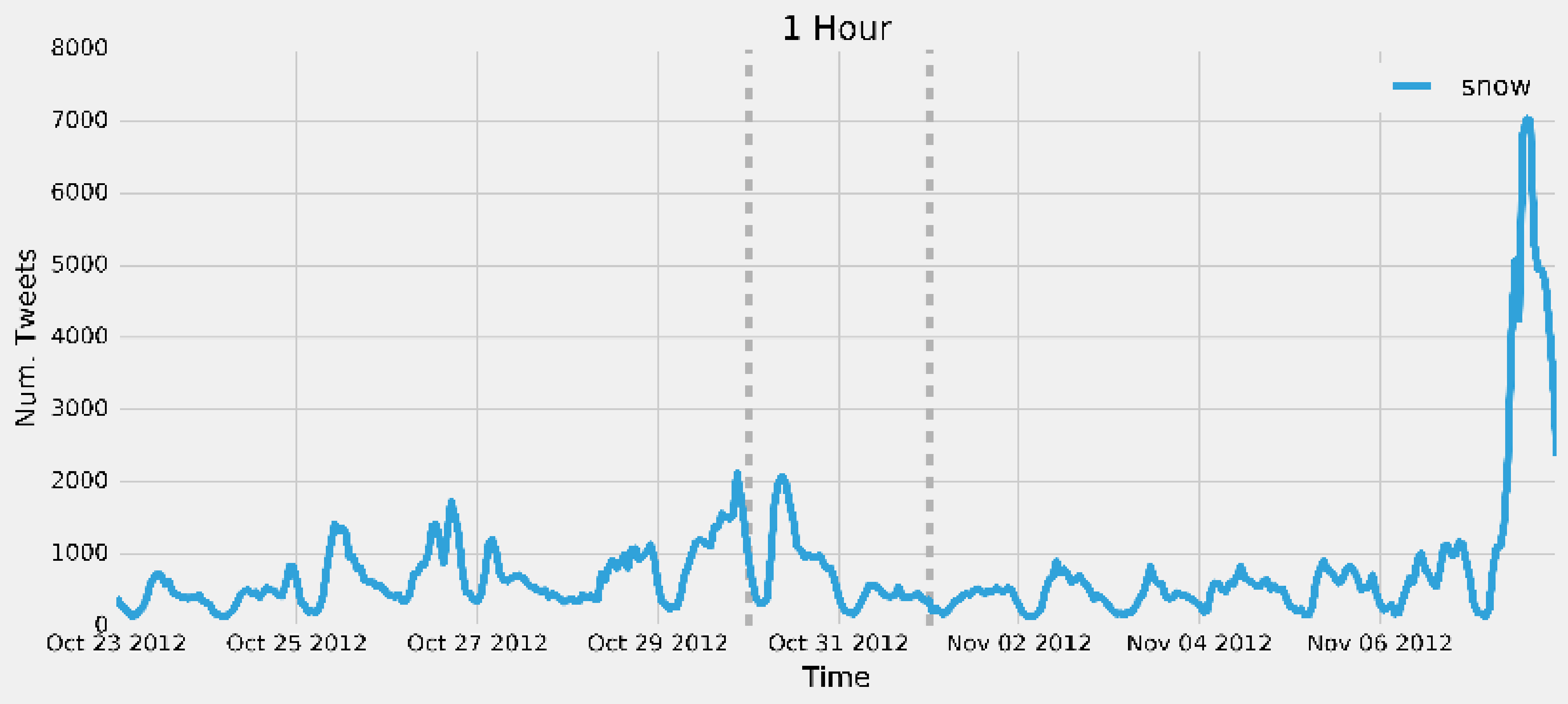
1 Hour
snow
Num. Tweets
0
1000
2000
3000
4000
5000
6000
7000
8000
Oct 23 2012
Oct 25 2012
Oct 27 2012
Oct 29 2012
Oct 31 2012
Nov 02 2012
Nov 04 2012
Nov 06 2012
Time

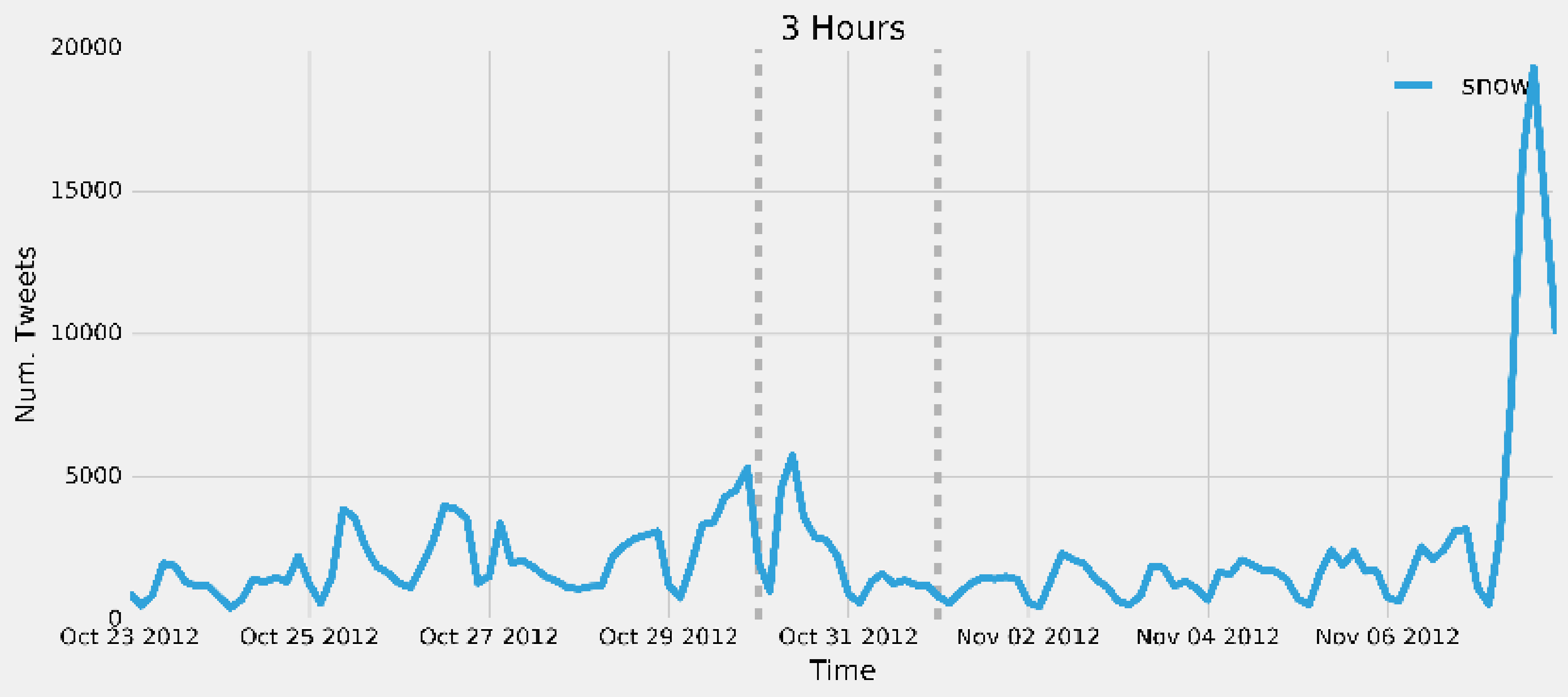

3 Hours
snow
20000
15000
10000
5000
0
Num. Tweets
Oct 23 2012
Oct 25 2012
Oct 27 2012
Oct 29 2012
Oct 31 2012
Nov 02 2012
Nov 04 2012
Nov 06 2012
Time

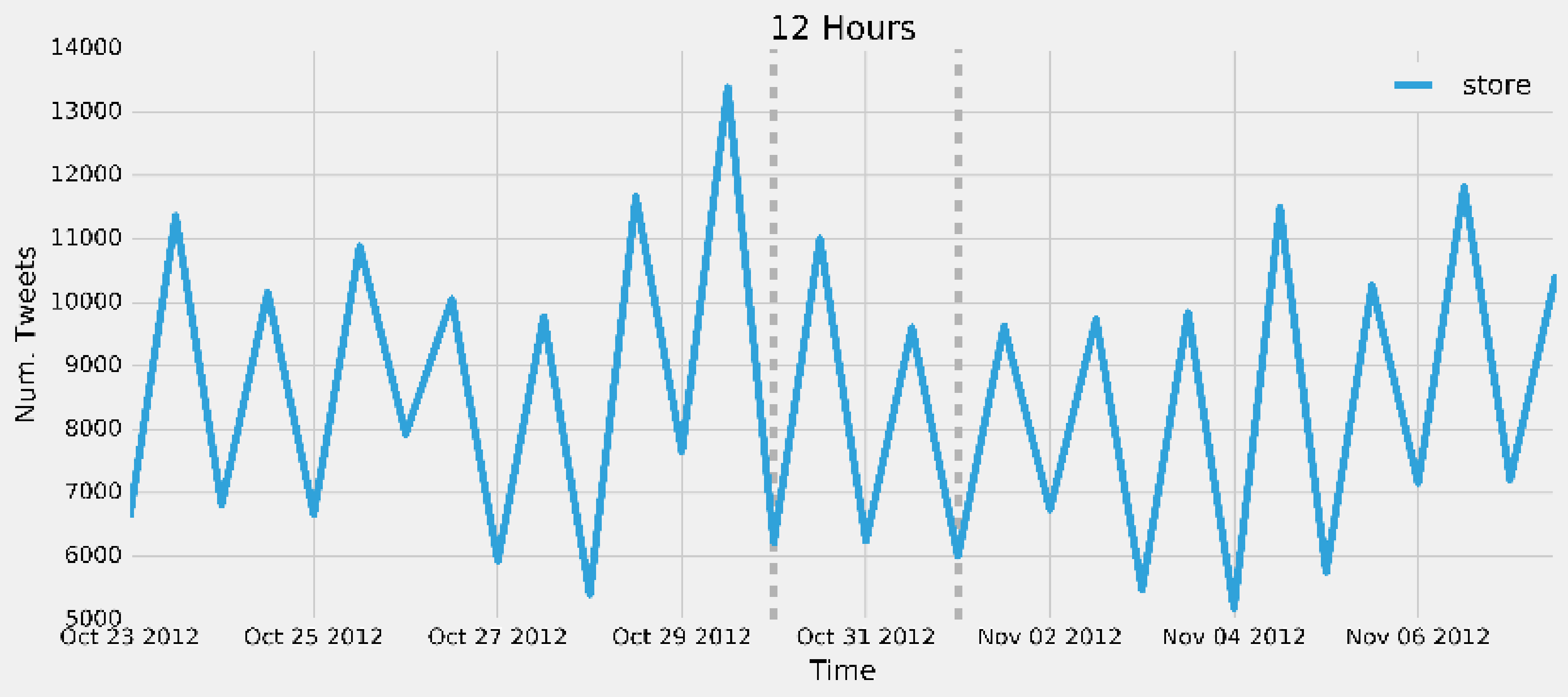

12 Hours
store
Num. Tweets
14000
13000
12000
11000
10000
9000
8000
7000
6000
5000
Oct 23 2012
Oct 25 2012
Oct 27 2012
Oct 29 2012
Oct 31 2012
Nov 02 2012
Nov 04 2012
Nov 06 2012
Time

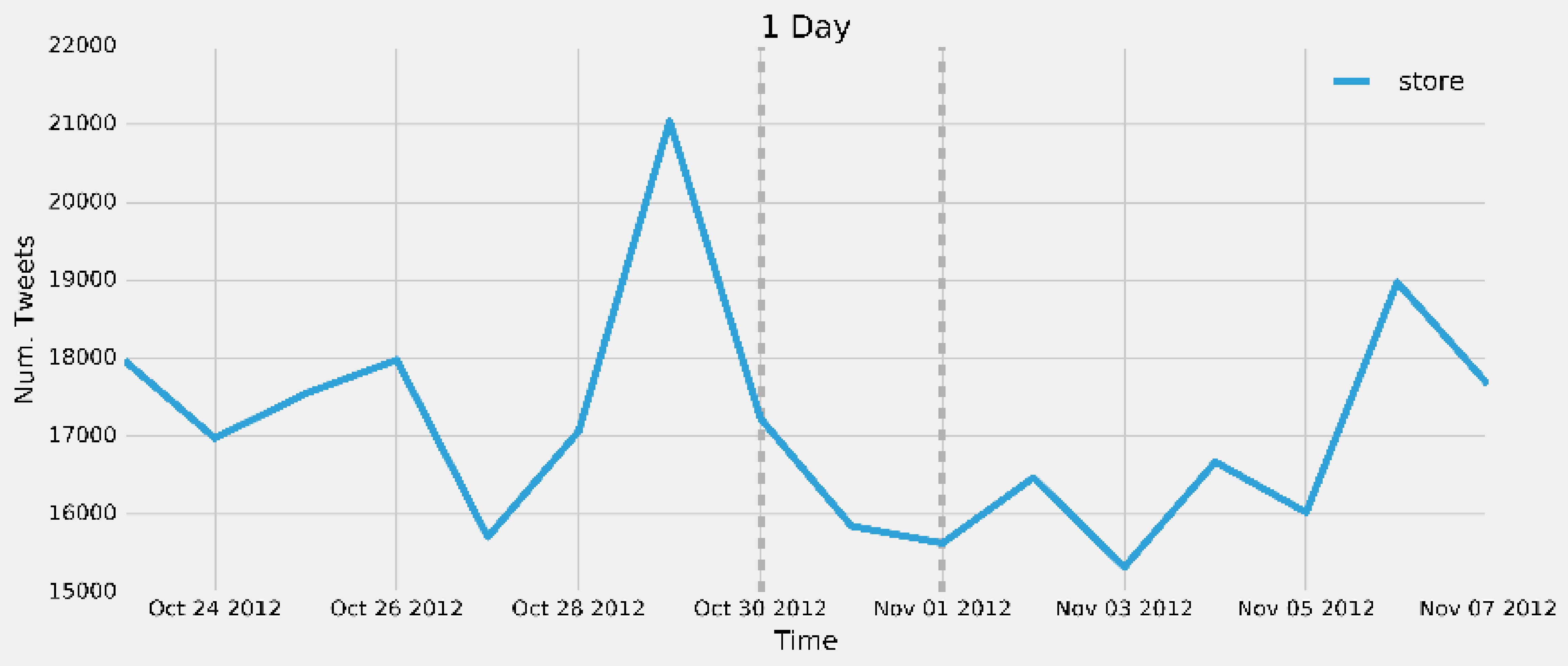
1 Day
store
Num. Tweets
22000
21000
20000
19000
18000
17000
16000
15000
Oct 24 2012
Oct 26 2012
Oct 28 2012
Oct 30 2012
Nov 01 2012
Nov 03 2012
Nov 05 2012
Nov 07 2012
Time

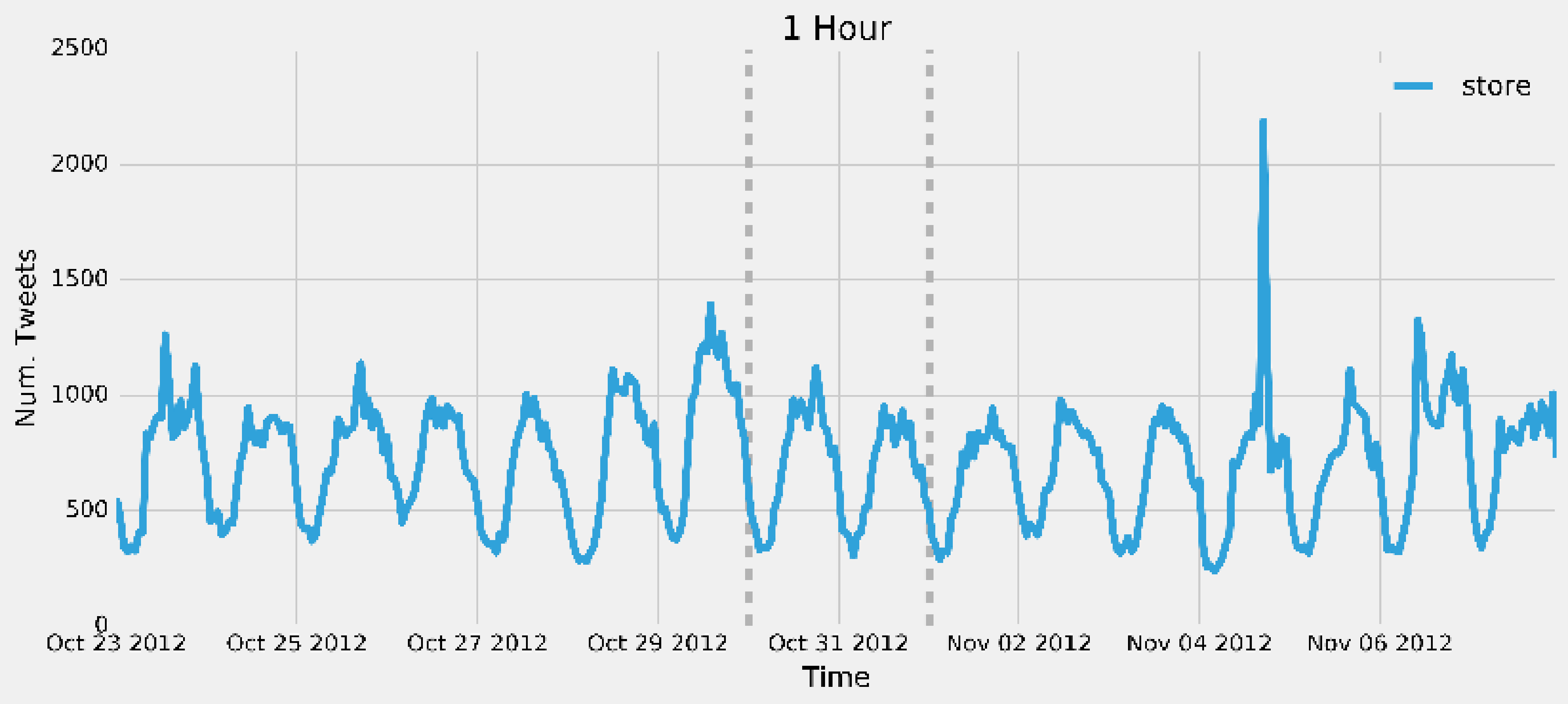

1 Hour
store
Num. Tweets
Time
2500
2000
1500
1000
500
0
Oct 23 2012
Oct 25 2012
Oct 27 2012
Oct 29 2012
Oct 31 2012
Nov 02 2012
Nov 04 2012
Nov 06 2012

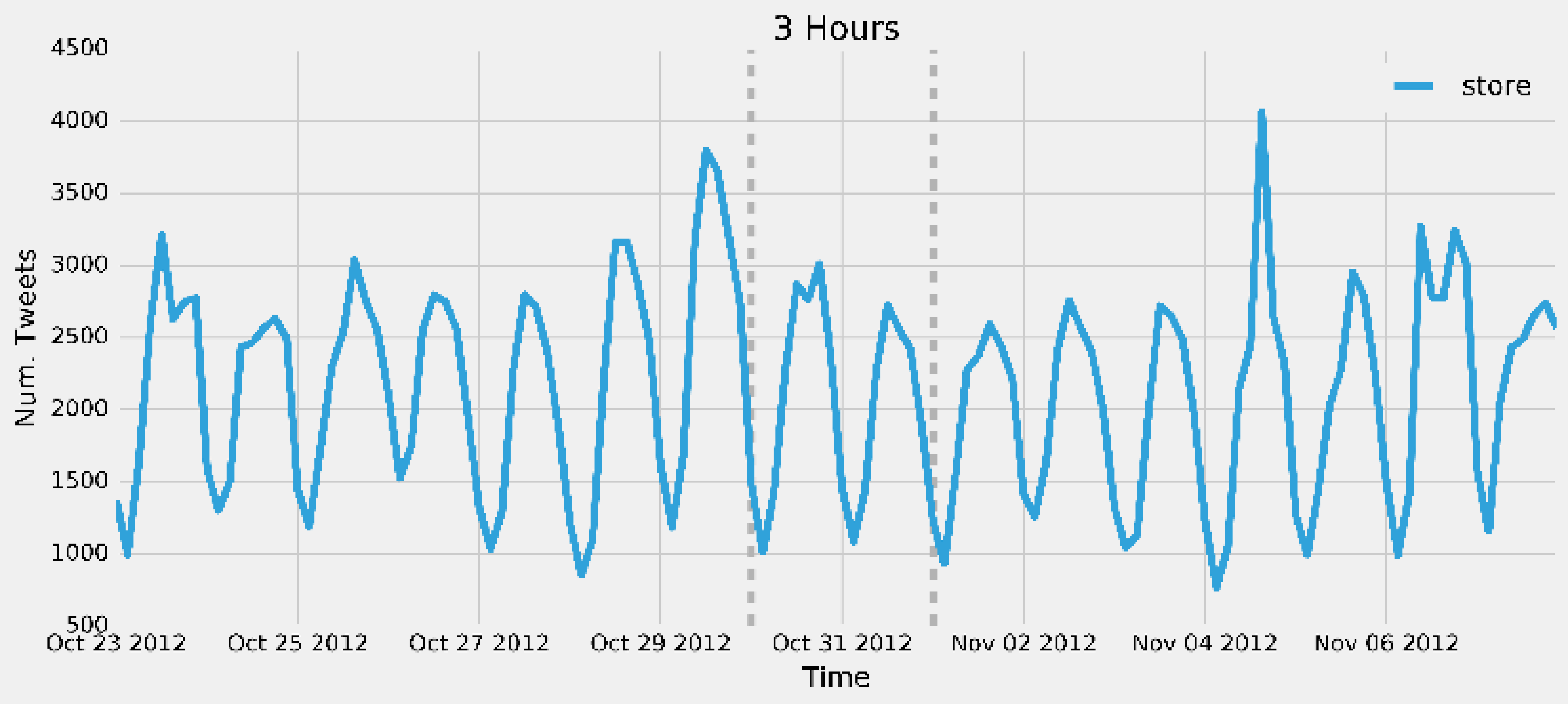
3 Hours
store
Num. Tweets
4500
4000
3500
3000
2500
2000
1500
1000
500
Oct 23 2012
Oct 25 2012
Oct 27 2012
Oct 29 2012
Oct 31 2012
Nov 02 2012
Nov 04 2012
Nov 06 2012
Time

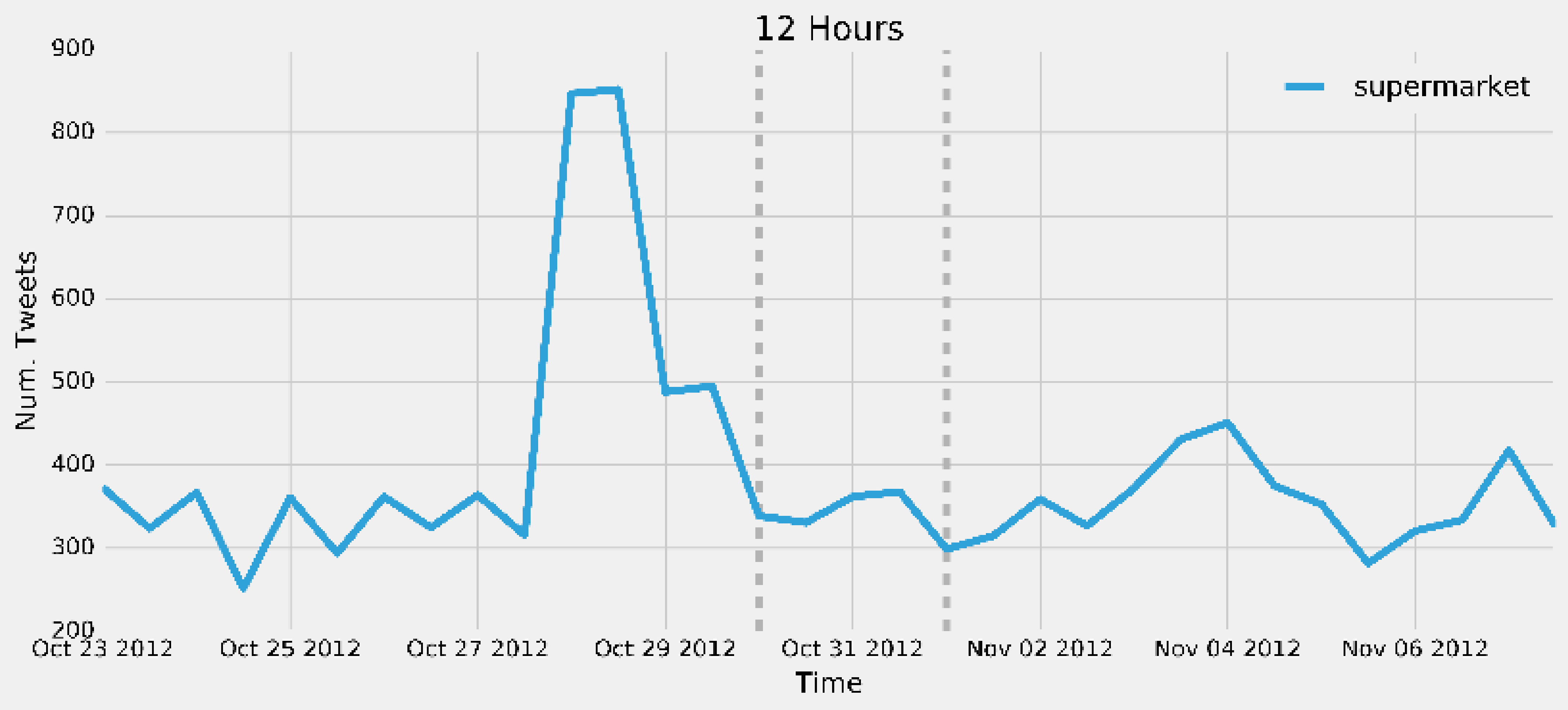

12 Hours
supermarket
Num. Tweets
900
800
700
600
500
400
300
200
Oct 23 2012
Oct 25 2012
Oct 27 2012
Oct 29 2012
Oct 31 2012
Nov 02 2012
Nov 04 2012
Nov 06 2012
Time

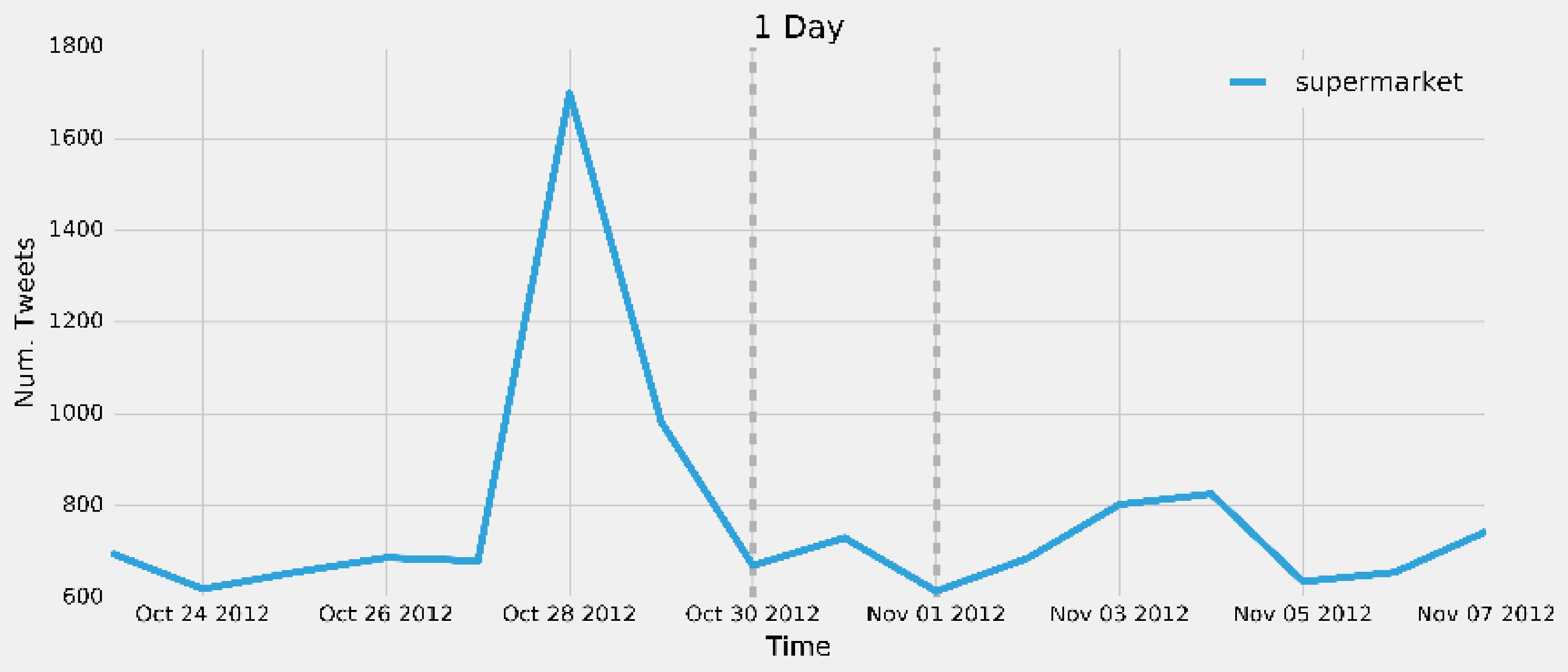

1 Day
supermarket
Num. Tweets
1800
1600
1400
1200
1000
800
600
Oct 24 2012
Oct 26 2012
Oct 28 2012
Oct 30 2012
Nov 01 2012
Nov 03 2012
Nov 05 2012
Nov 07 2012
Time

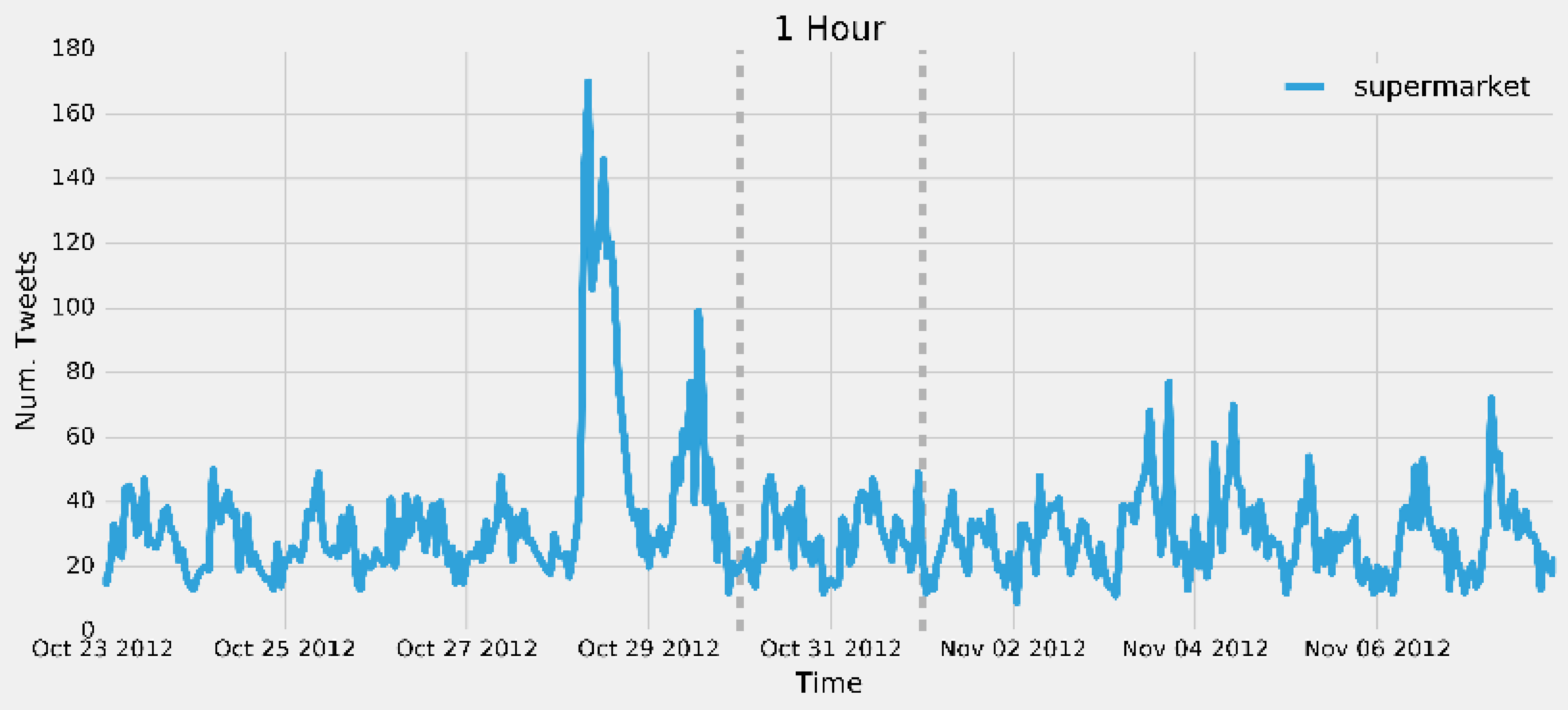
1 Hour
supermarket
Num. Tweets
0
20
40
60
80
100
120
140
160
180
Oct 23 2012
Oct 25 2012
Oct 27 2012
Oct 29 2012
Oct 31 2012
Nov 02 2012
Nov 04 2012
Nov 06 2012
Time

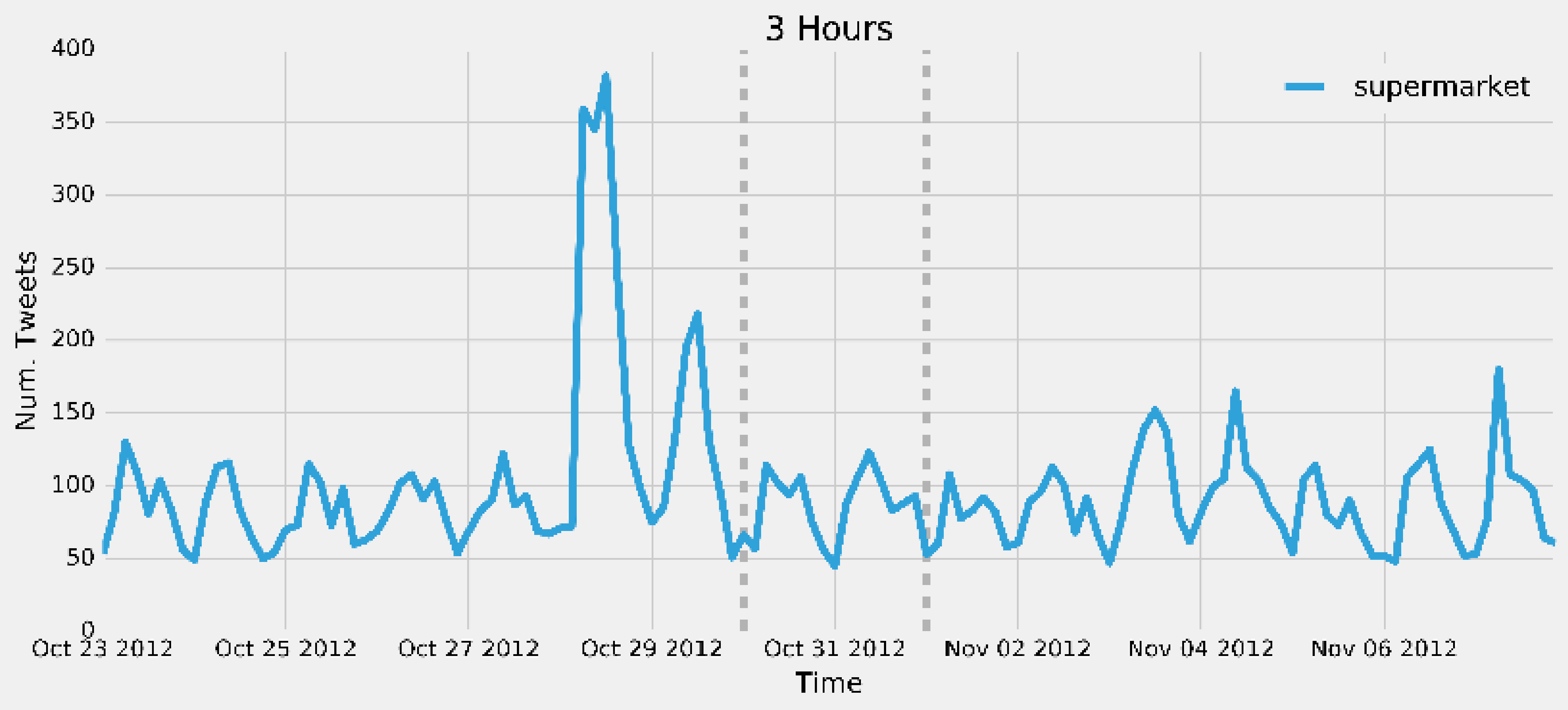
3 Hours
supermarket
Num. Tweets
400
350
300
250
200
150
100
50
0
Oct 23 2012
Oct 25 2012
Oct 27 2012
Oct 29 2012
Oct 31 2012
Nov 02 2012
Nov 04 2012
Nov 06 2012
Time

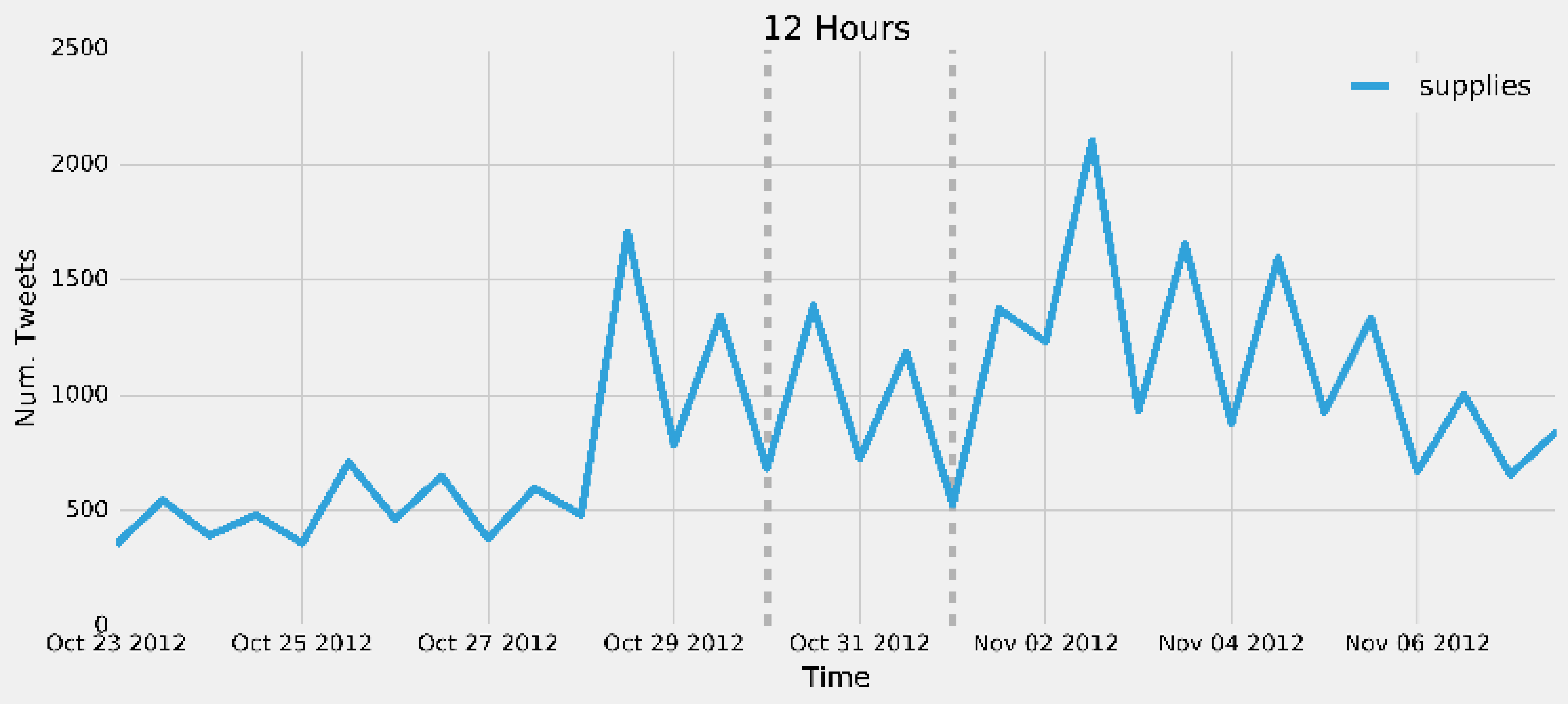
12 Hours
supplies
Num. Tweets
0
500
1000
1500
2000
2500
Oct 23 2012
Oct 25 2012
Oct 27 2012
Oct 29 2012
Oct 31 2012
Nov 02 2012
Nov 04 2012
Nov 06 2012
Time

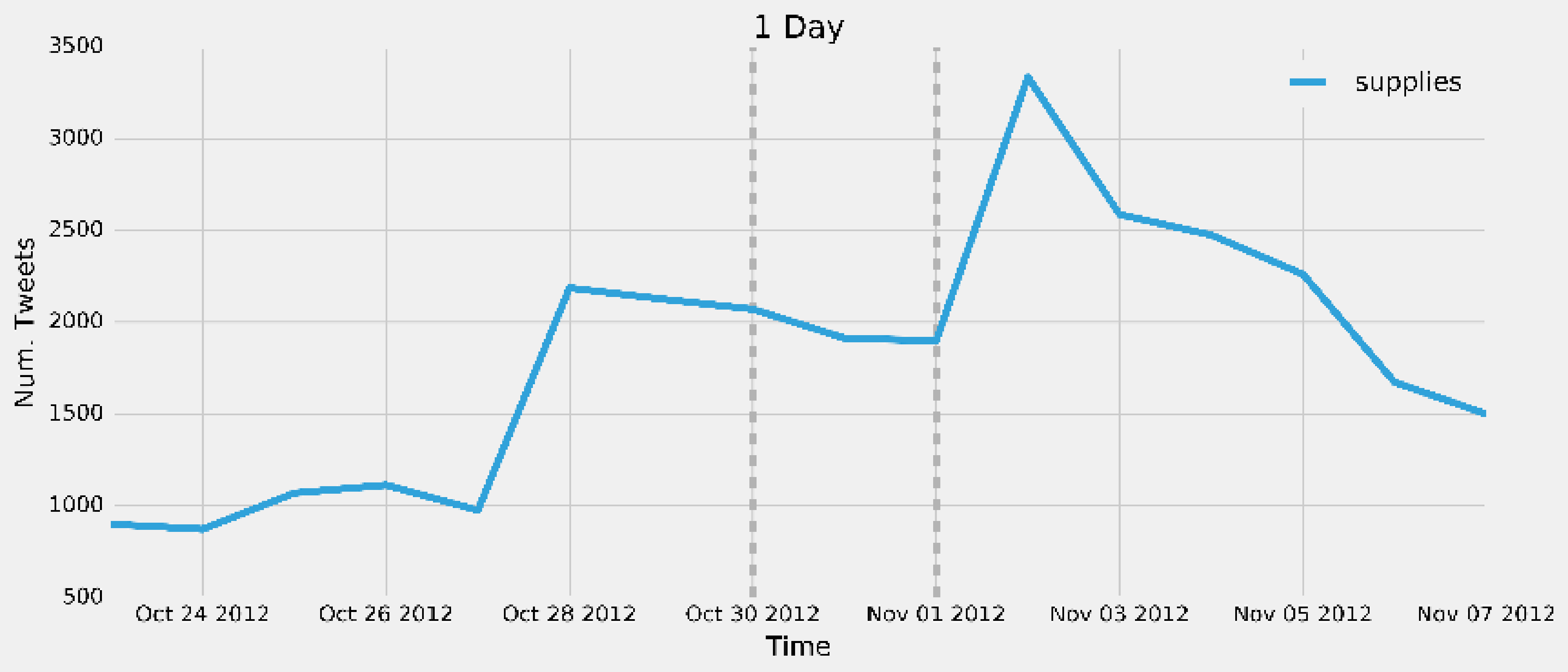
1 Day
supplies
Num. Tweets
3500
3000
2500
2000
1500
1000
500
Oct 24 2012
Oct 26 2012
Oct 28 2012
Oct 30 2012
Nov 01 2012
Nov 03 2012
Nov 05 2012
Nov 07 2012
Time

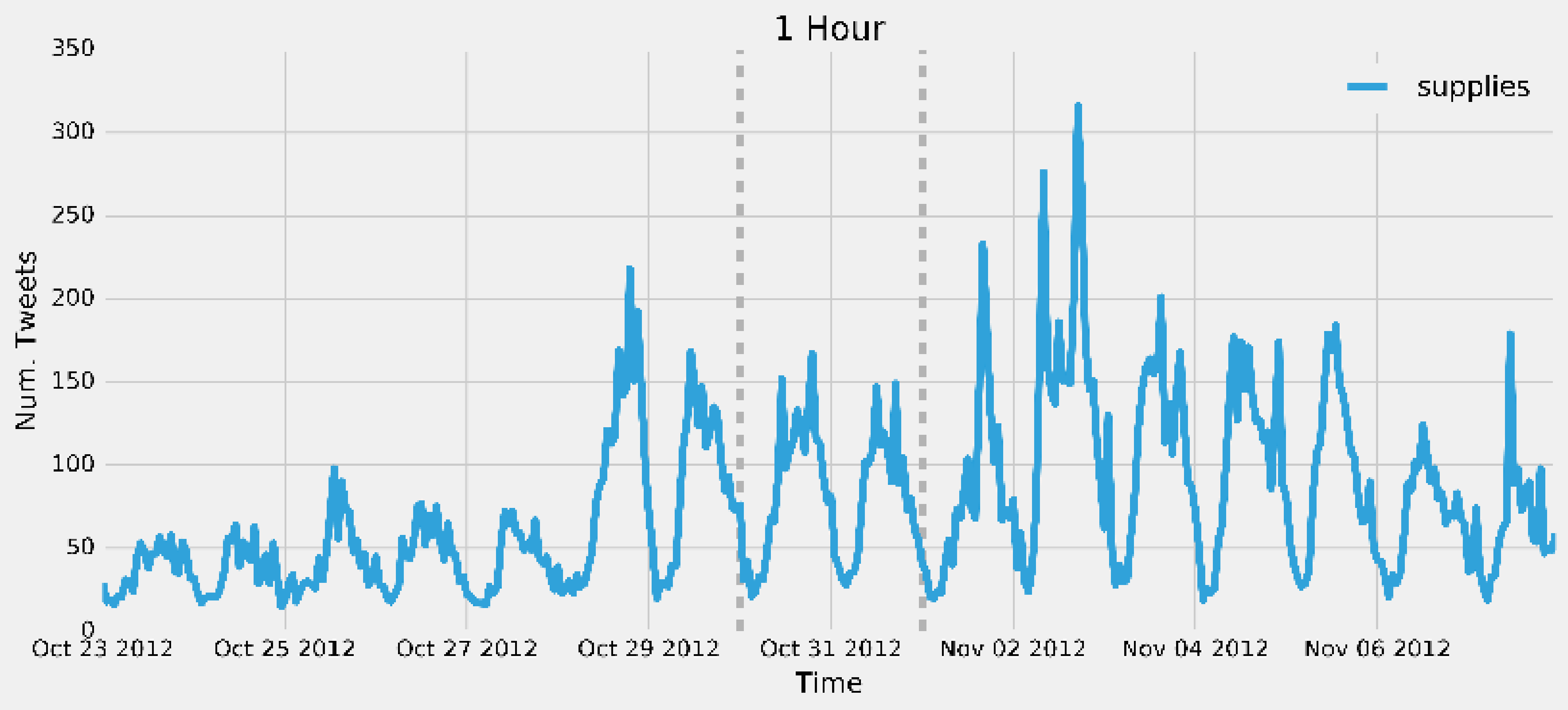
1 Hour
supplies
Num. Tweets
0
50
100
150
200
250
300
350
Oct 23 2012
Oct 25 2012
Oct 27 2012
Oct 29 2012
Oct 31 2012
Nov 02 2012
Nov 04 2012
Nov 06 2012
Time

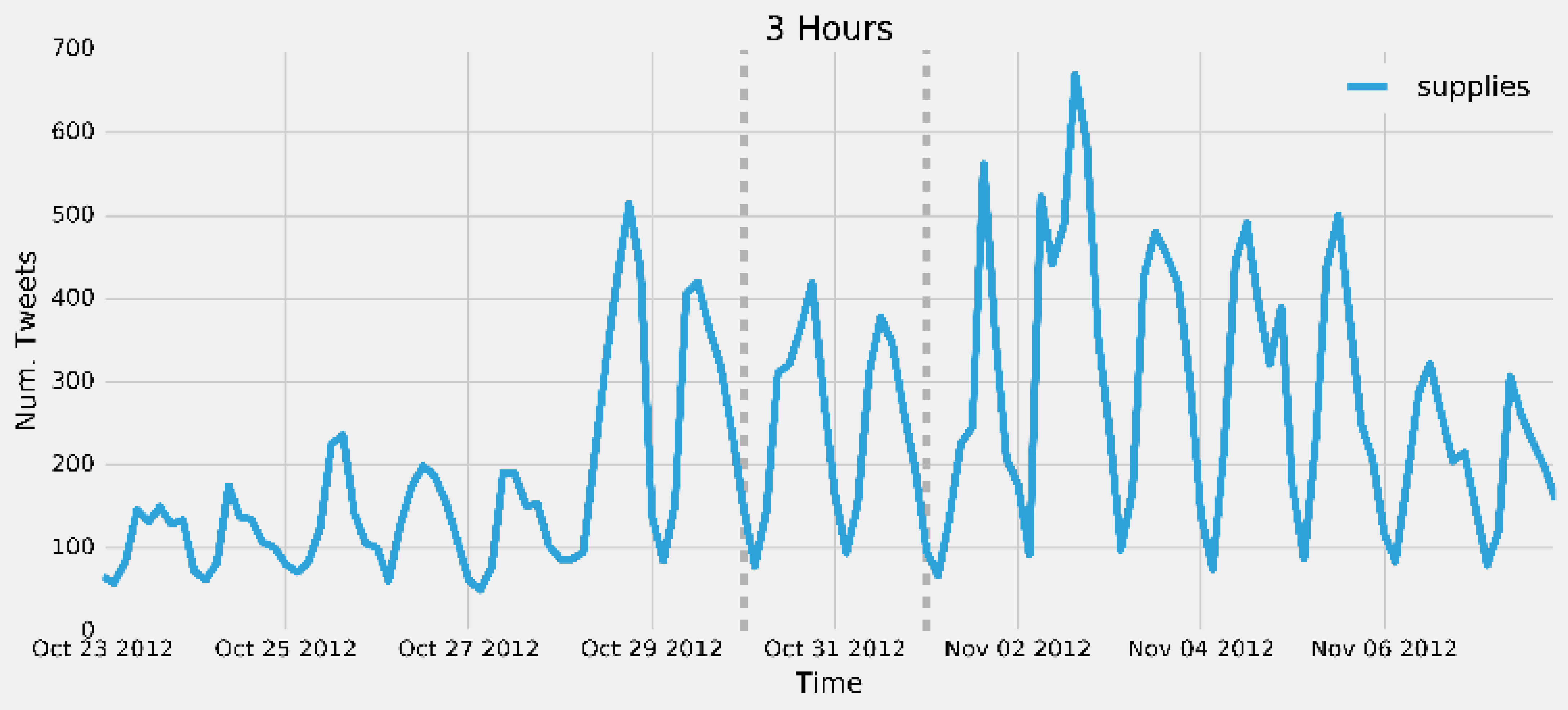
3 Hours
supplies
Num. Tweets
0
100
200
300
400
500
600
700
Oct 23 2012
Oct 25 2012
Oct 27 2012
Oct 29 2012
Oct 31 2012
Nov 02 2012
Nov 04 2012
Nov 06 2012
Time

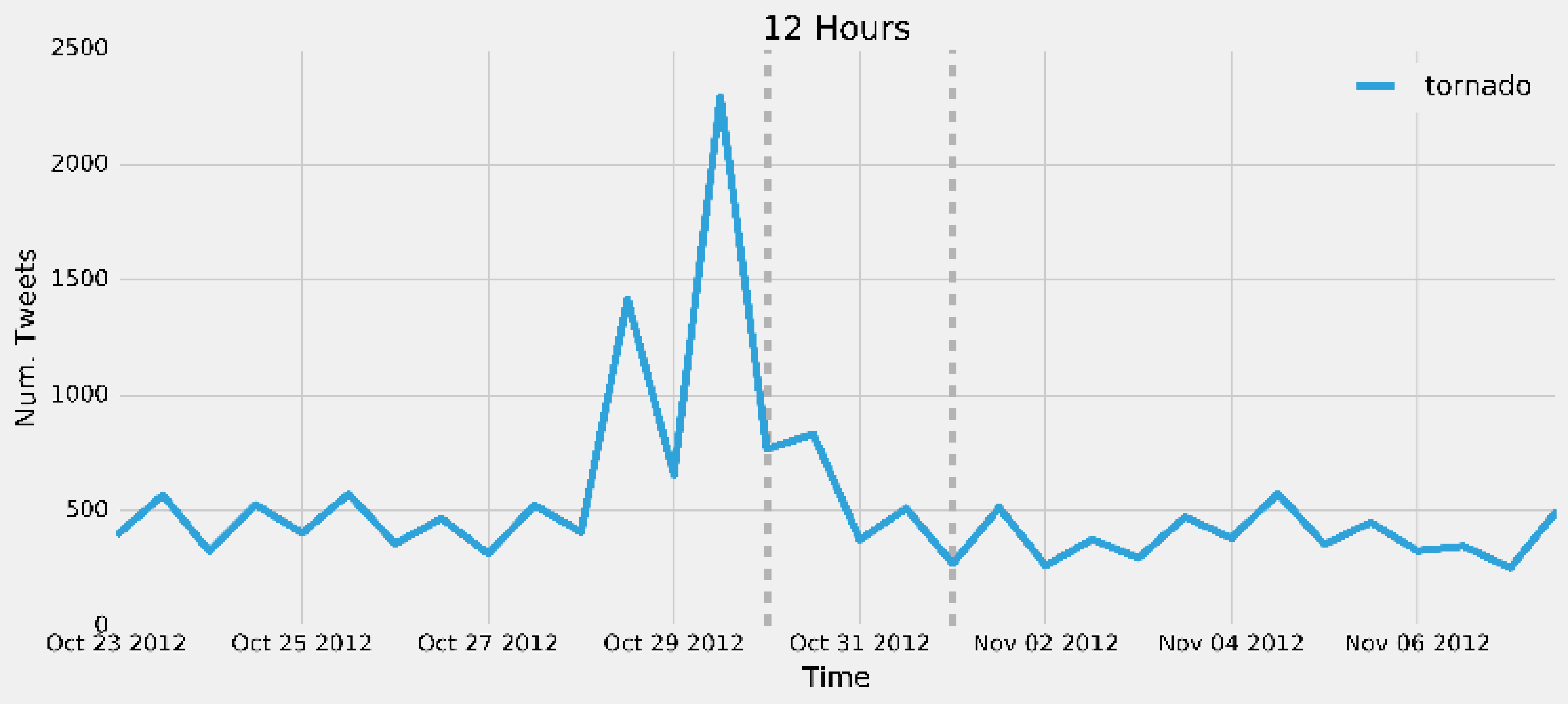
12 Hours
tornado
Num. Tweets
0
500
1000
1500
2000
2500
Oct 23 2012
Oct 25 2012
Oct 27 2012
Oct 29 2012
Oct 31 2012
Nov 02 2012
Nov 04 2012
Nov 06 2012
Time

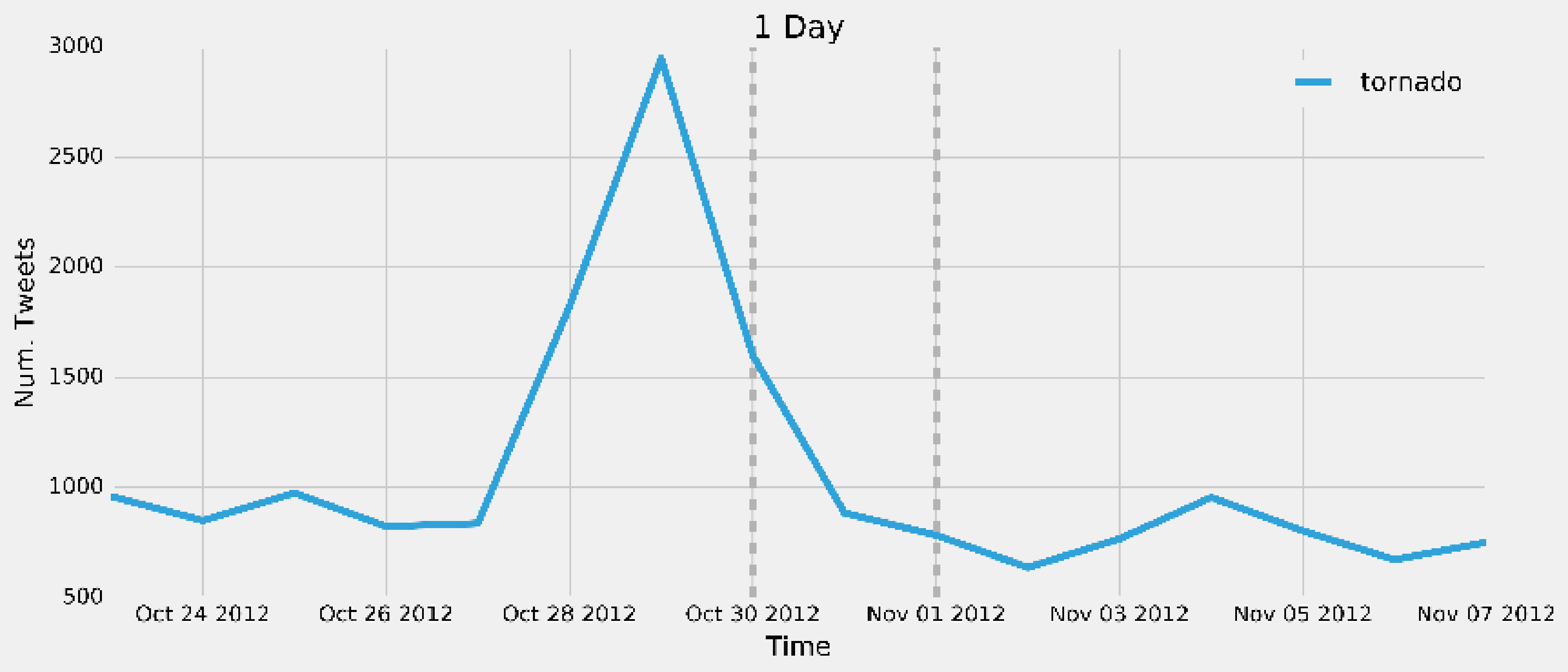
1 Day
tornado
Num. Tweets
3000
2500
2000
1500
1000
500
Oct 24 2012
Oct 26 2012
Oct 28 2012
Oct 30 2012
Nov 01 2012
Nov 03 2012
Nov 05 2012
Nov 07 2012
Time

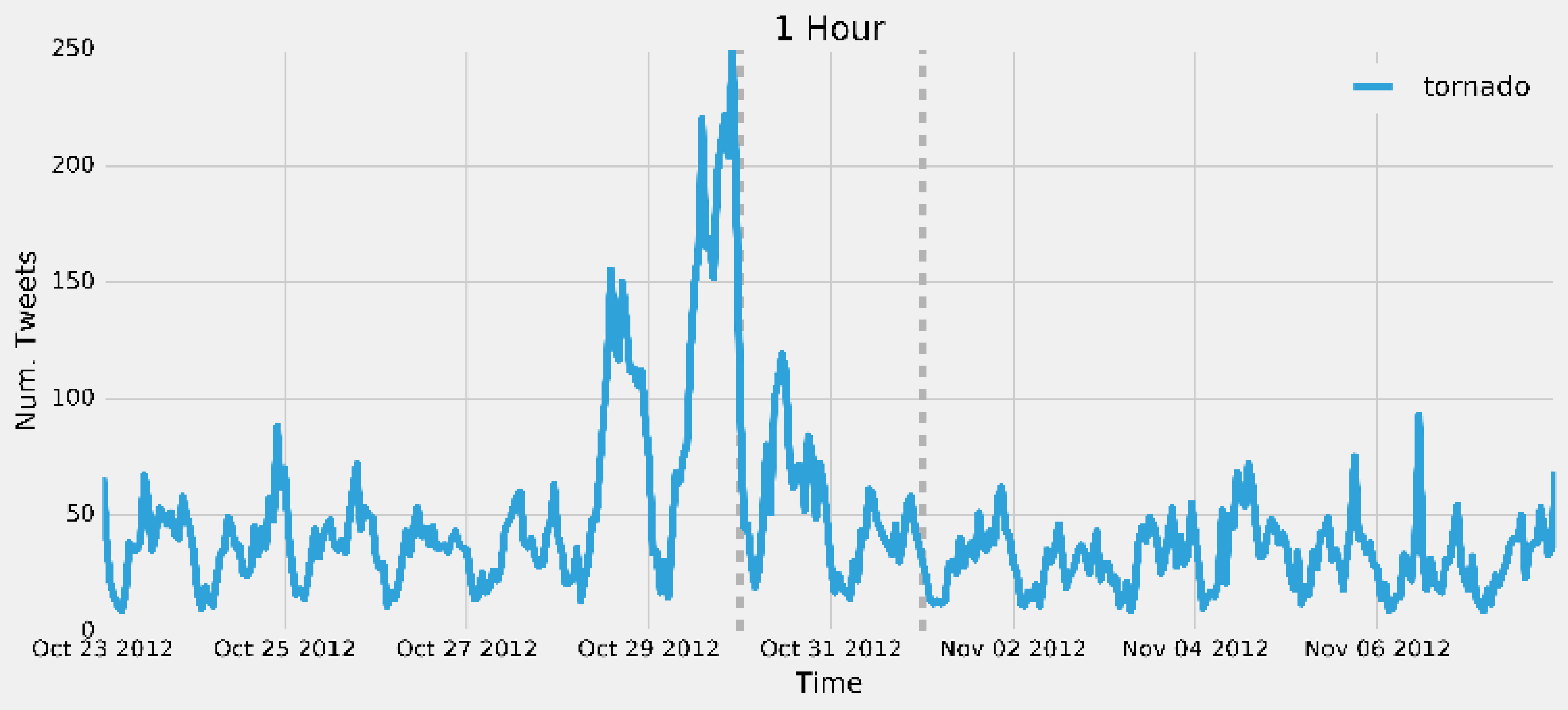

1 Hour
tornado
Num. Tweets
Time
250
200
150
100
50
0
Oct 23 2012
Oct 25 2012
Oct 27 2012
Oct 29 2012
Oct 31 2012
Nov 02 2012
Nov 04 2012
Nov 06 2012

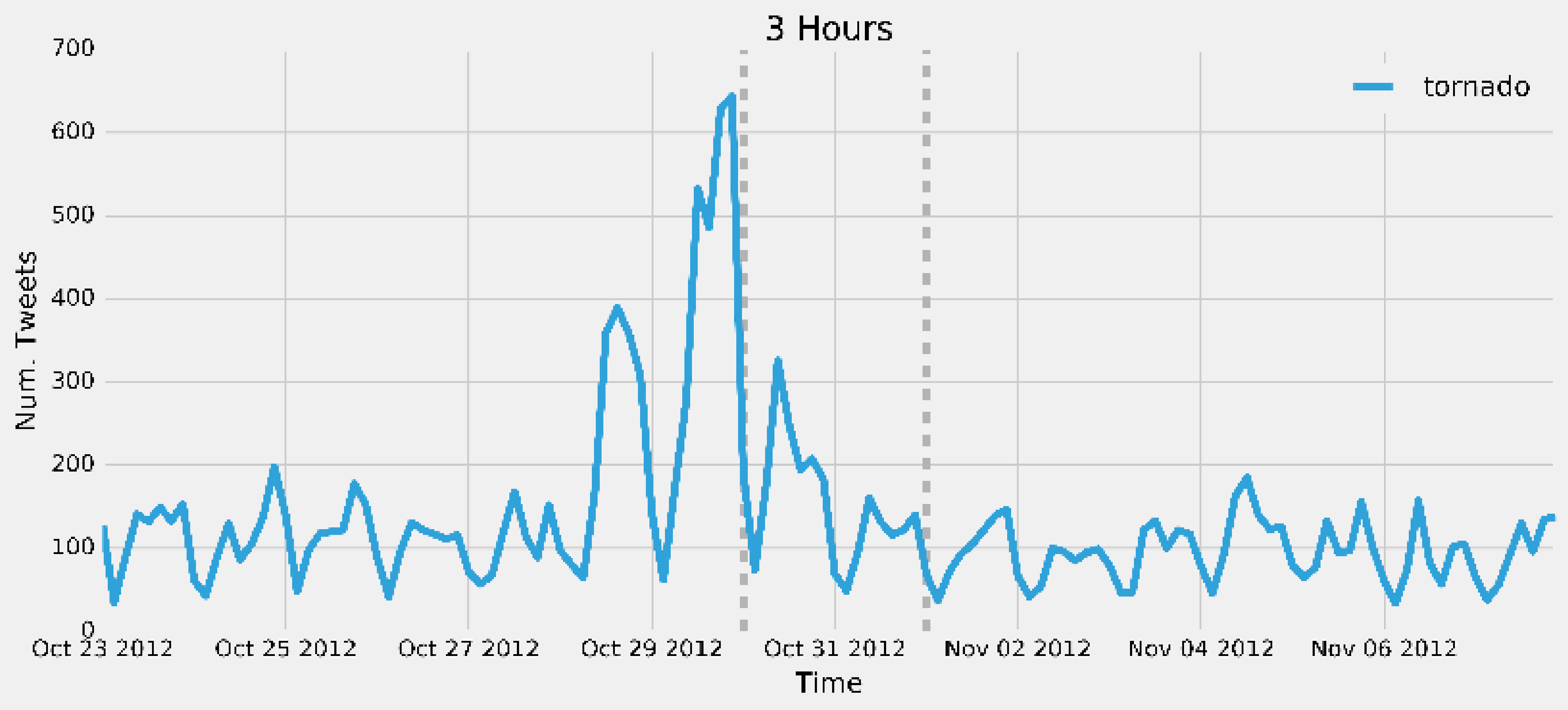
3 Hours
tornado
Num. Tweets
0
100
200
300
400
500
600
700
Oct 23 2012
Oct 25 2012
Oct 27 2012
Oct 29 2012
Oct 31 2012
Nov 02 2012
Nov 04 2012
Nov 06 2012
Time

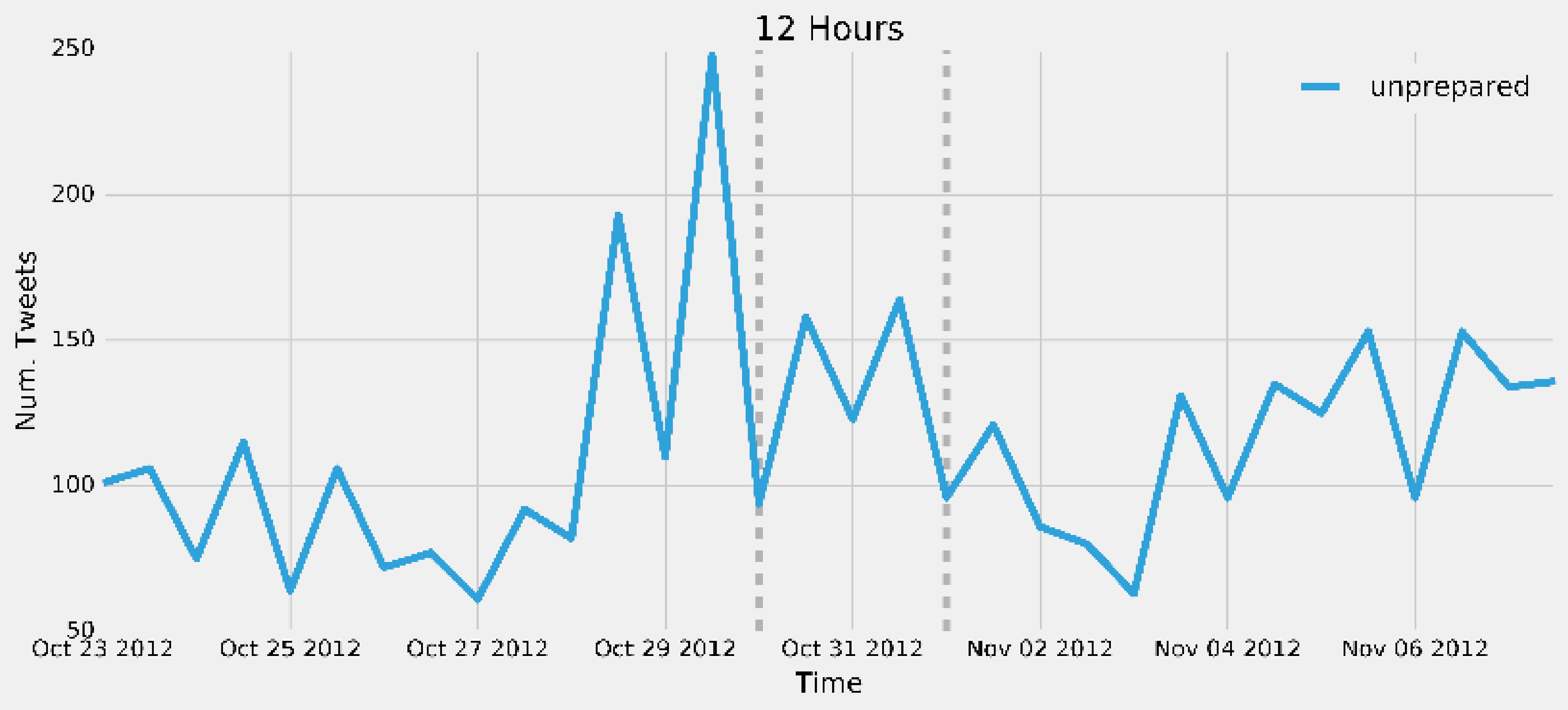
12 Hours
unprepared
250
200
150
100
50
Num. Tweets
Oct 23 2012
Oct 25 2012
Oct 27 2012
Oct 29 2012
Oct 31 2012
Nov 02 2012
Nov 04 2012
Nov 06 2012
Time

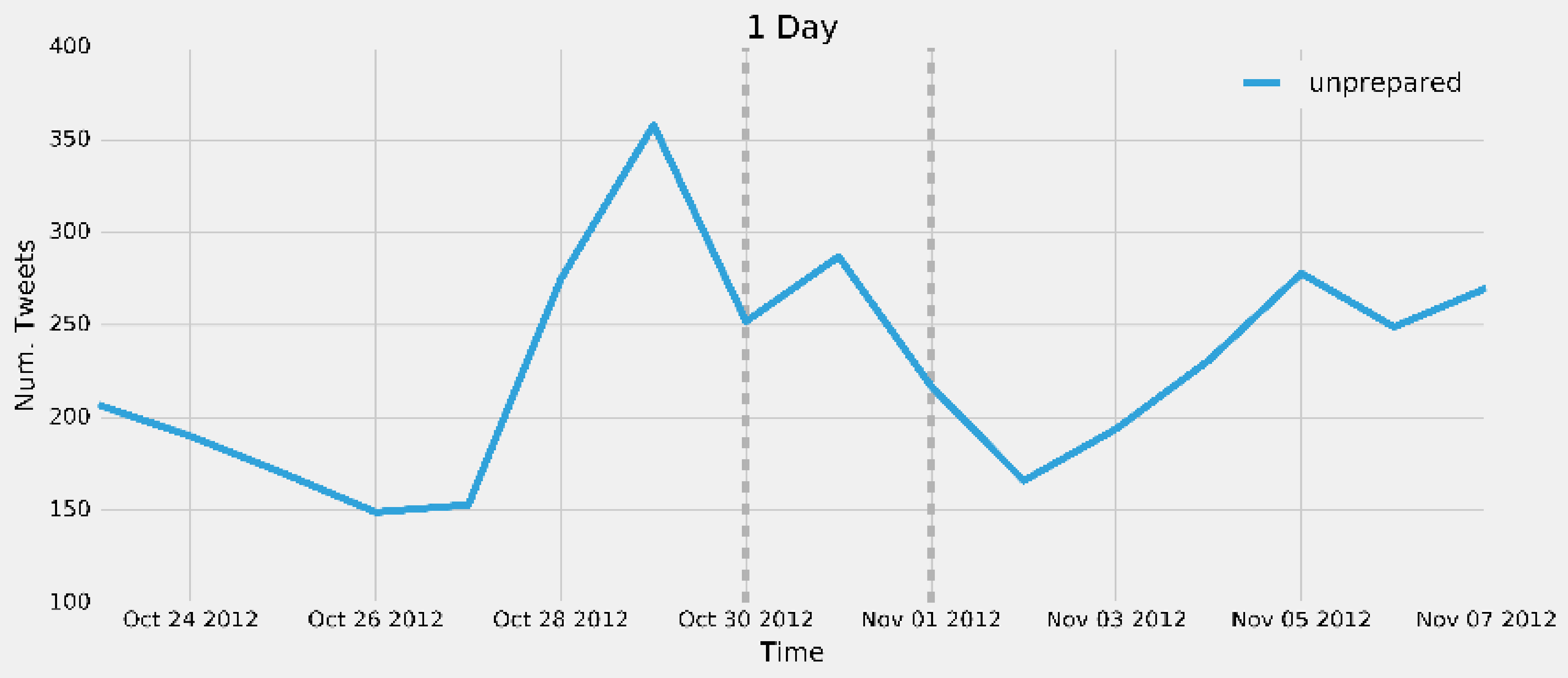
1 Day
unprepared
Num. Tweets
400
350
300
250
200
150
100
Oct 24 2012
Oct 26 2012
Oct 28 2012
Oct 30 2012
Nov 01 2012
Nov 03 2012
Nov 05 2012
Nov 07 2012
Time

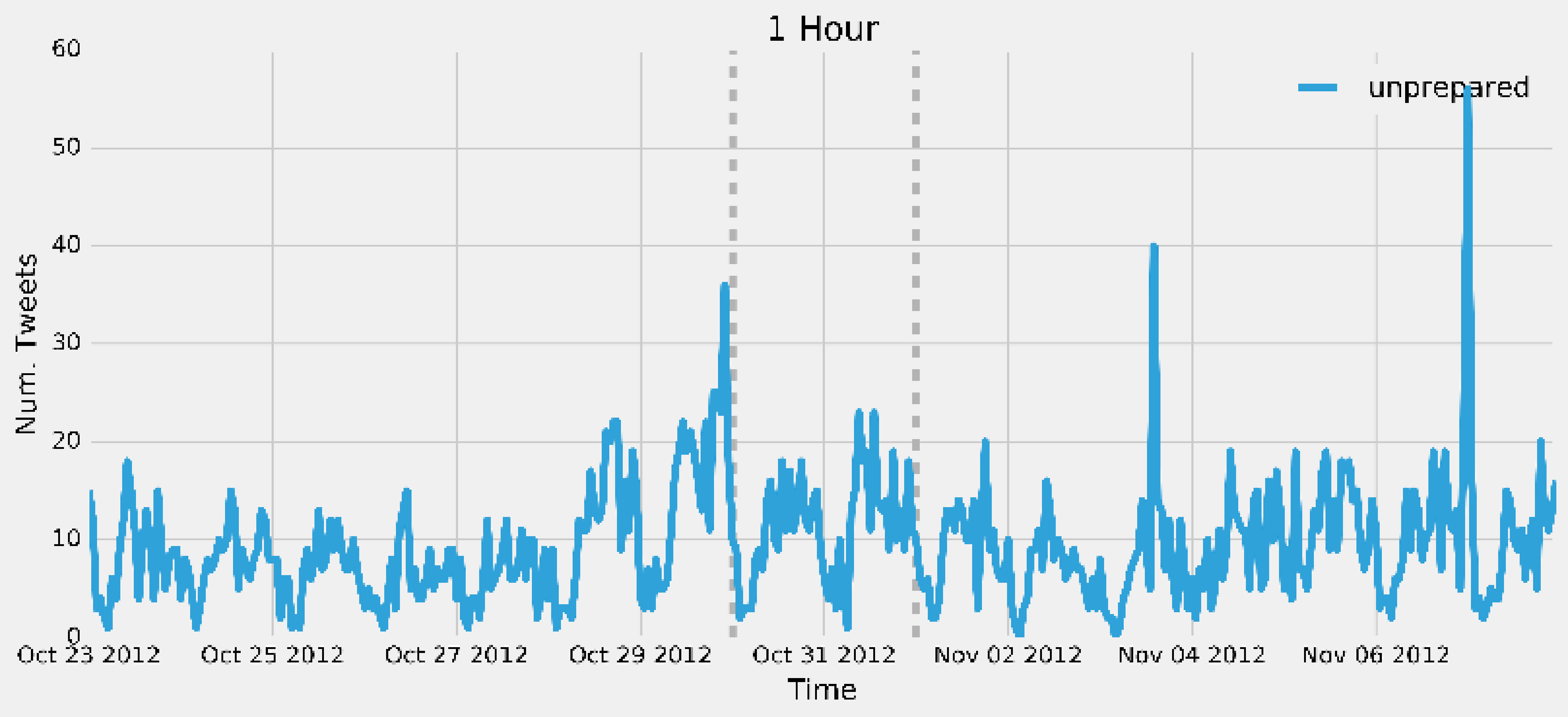
1 Hour
unprepared
Num. Tweets
0
10
20
30
40
50
60
Oct 23 2012
Oct 25 2012
Oct 27 2012
Oct 29 2012
Oct 31 2012
Nov 02 2012
Nov 04 2012
Nov 06 2012
Time

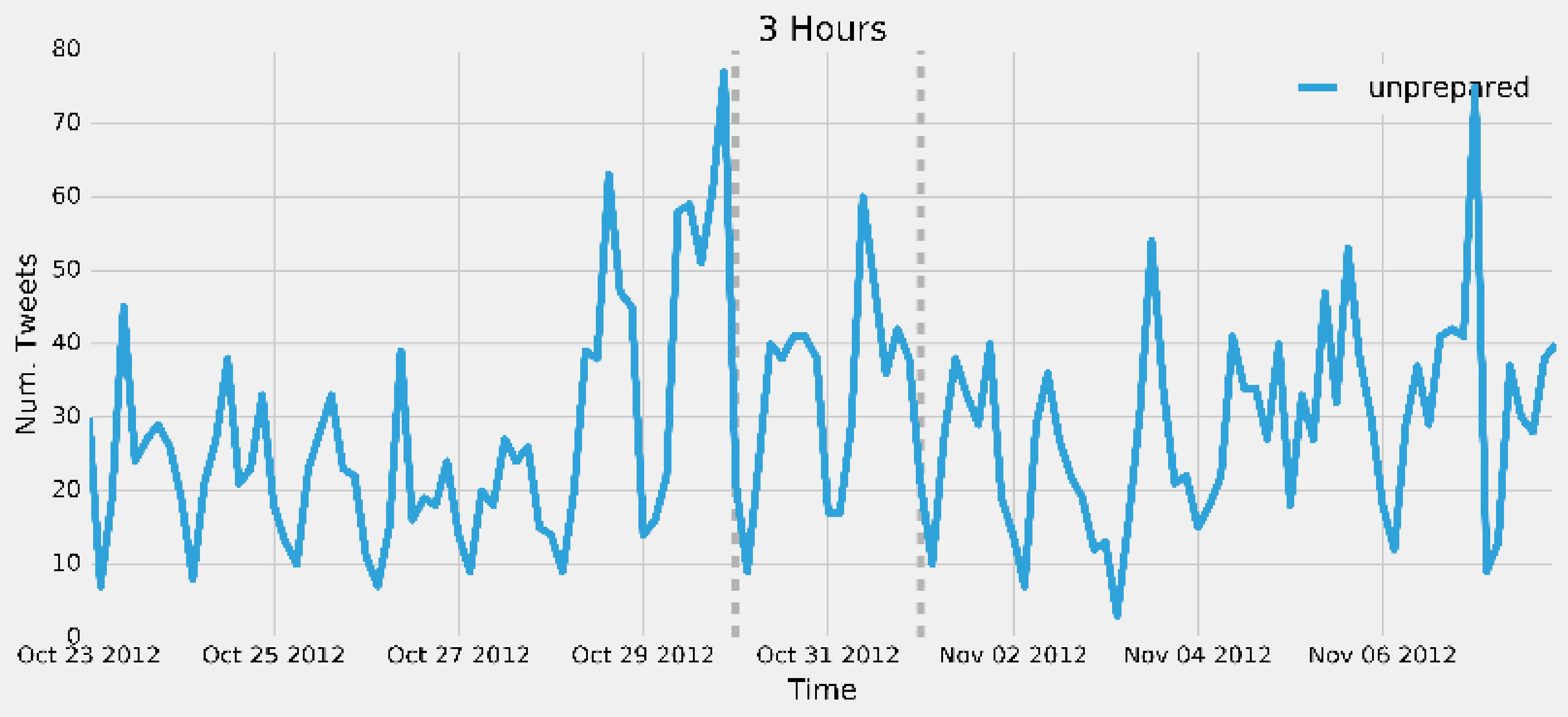
3 Hours
unprepared
Num. Tweets
0
10
20
30
40
50
60
70
80
Oct 23 2012
Oct 25 2012
Oct 27 2012
Oct 29 2012
Oct 31 2012
Nov 02 2012
Nov 04 2012
Nov 06 2012
Time

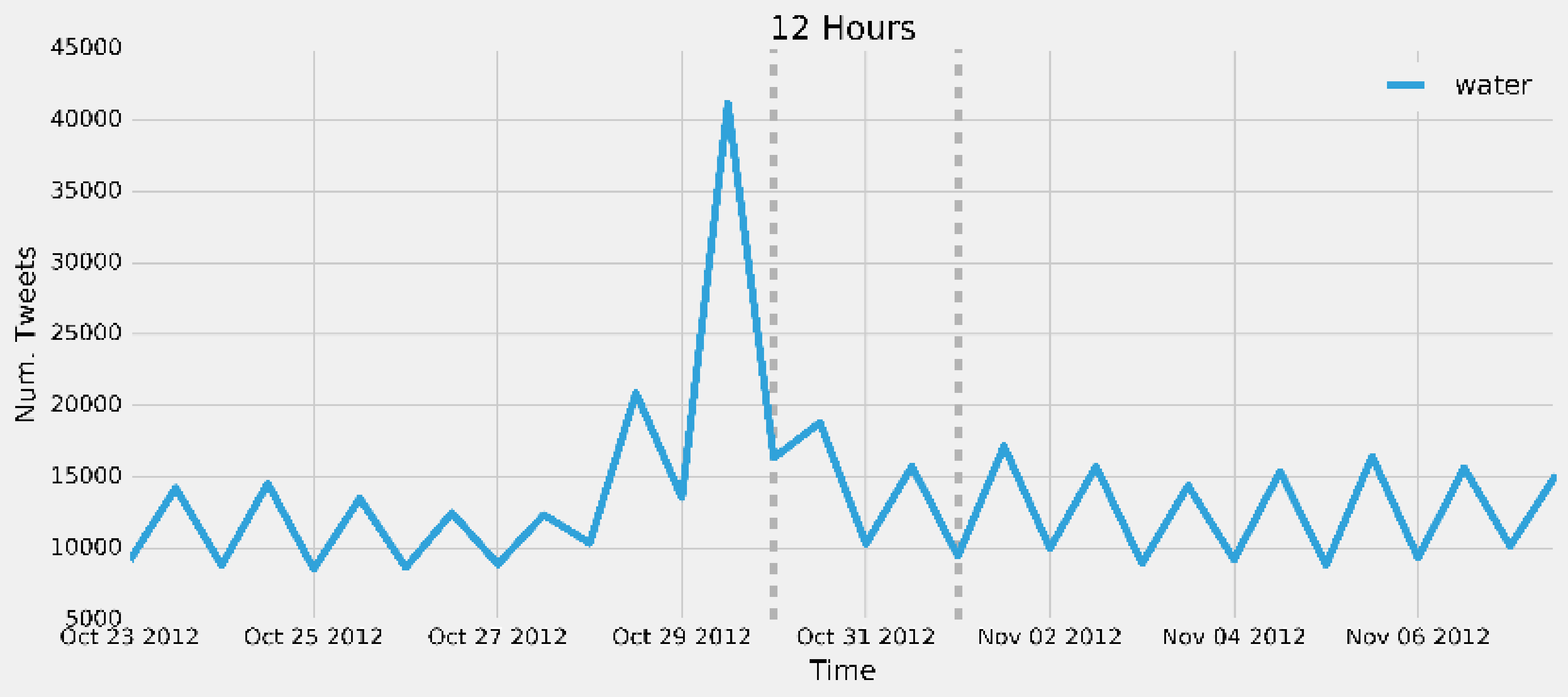
12 Hours
water
Num. Tweets
45000
40000
35000
30000
25000
20000
15000
10000
5000
Oct 23 2012
Oct 25 2012
Oct 27 2012
Oct 29 2012
Oct 31 2012
Nov 02 2012
Nov 04 2012
Nov 06 2012
Time

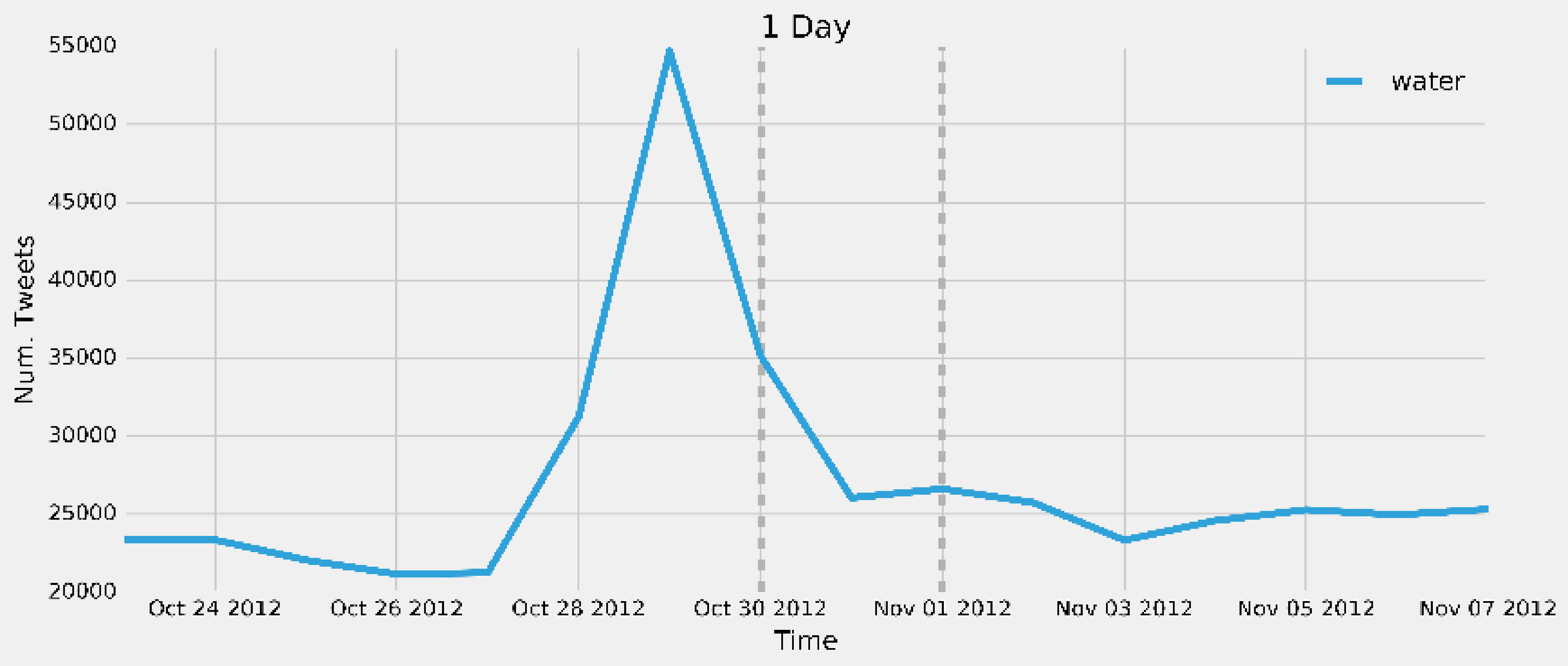
1 Day
water
Num. Tweets
55000
50000
45000
40000
35000
30000
25000
20000
Oct 24 2012
Oct 26 2012
Oct 28 2012
Oct 30 2012
Nov 01 2012
Nov 03 2012
Nov 05 2012
Nov 07 2012
Time

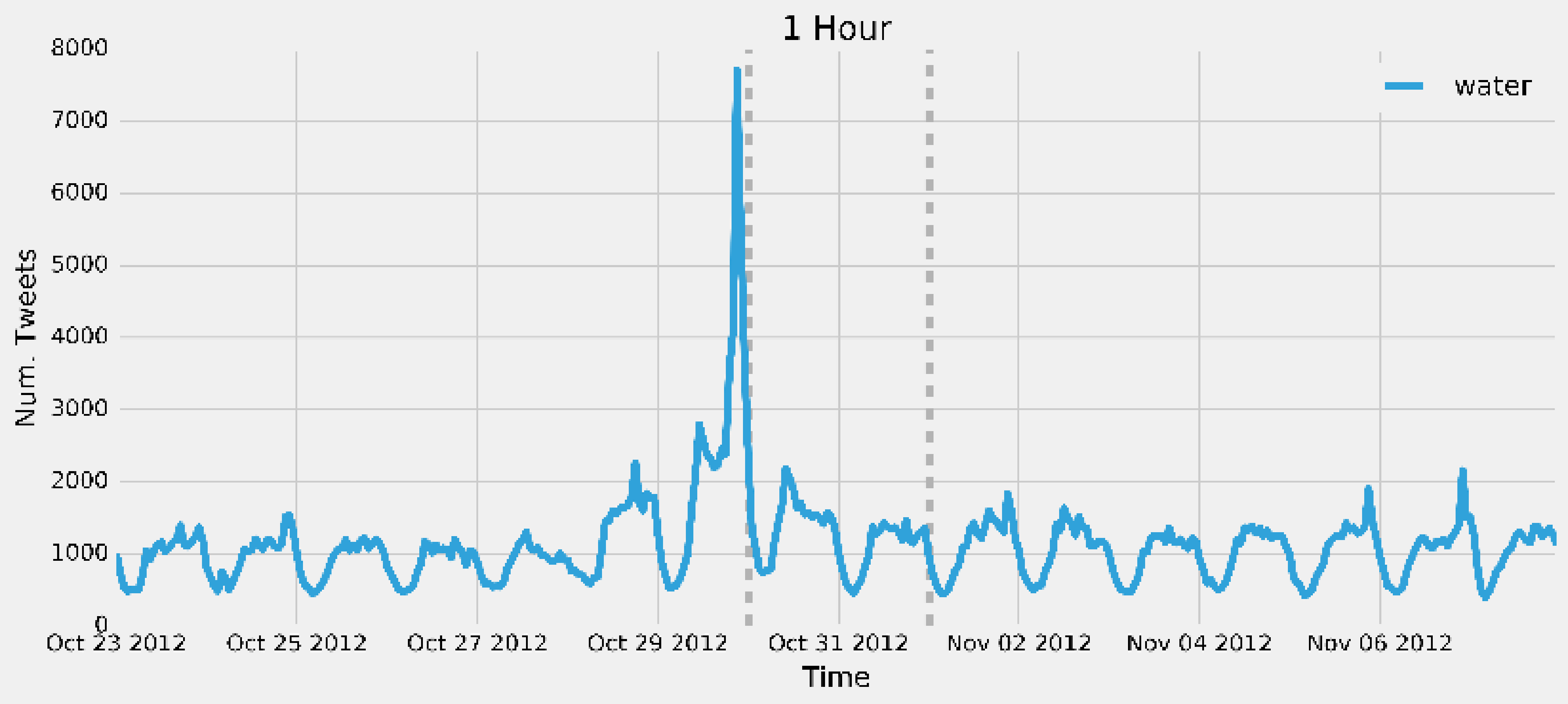
1 Hour
water
Num. Tweets
8000
7000
6000
5000
4000
3000
2000
1000
0
Oct 23 2012
Oct 25 2012
Oct 27 2012
Oct 29 2012
Oct 31 2012
Nov 02 2012
Nov 04 2012
Nov 06 2012
Time

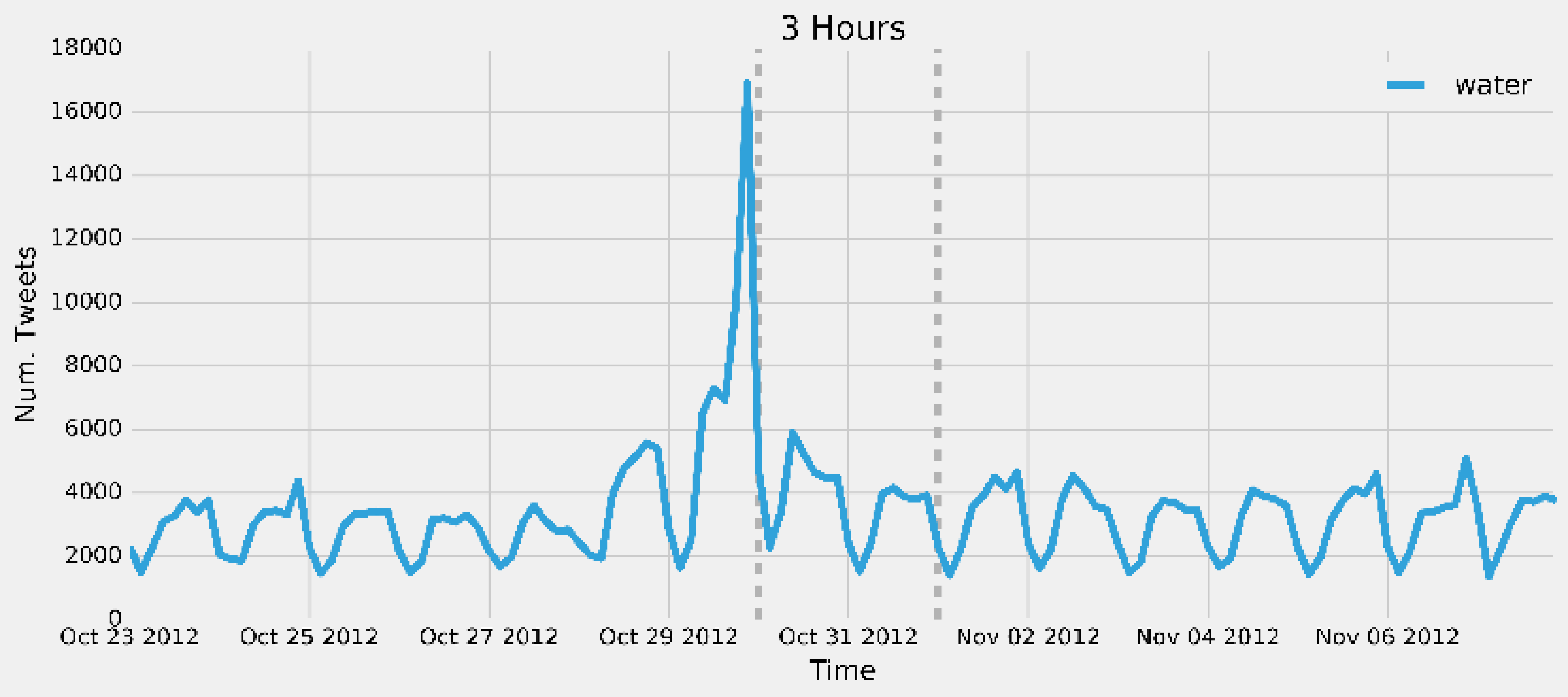
3 Hours
water
Num. Tweets
0
2000
4000
6000
8000
10000
12000
14000
16000
18000
Oct 23 2012
Oct 25 2012
Oct 27 2012
Oct 29 2012
Oct 31 2012
Nov 02 2012
Nov 04 2012
Nov 06 2012
Time

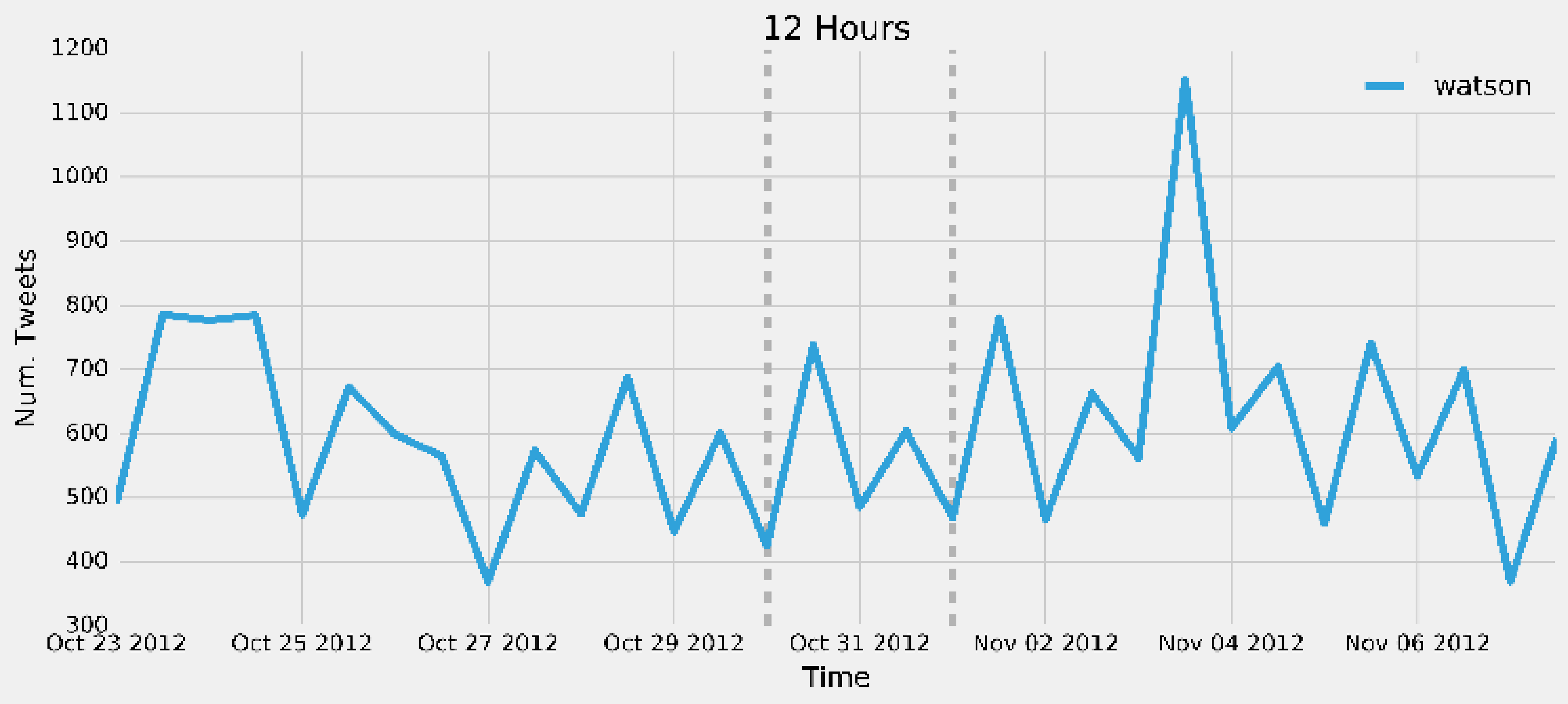
12 Hours
watson
Num. Tweets
1200
1100
1000
900
800
700
600
500
400
300
Oct 23 2012
Oct 25 2012
Oct 27 2012
Oct 29 2012
Oct 31 2012
Nov 02 2012
Nov 04 2012
Nov 06 2012
Time

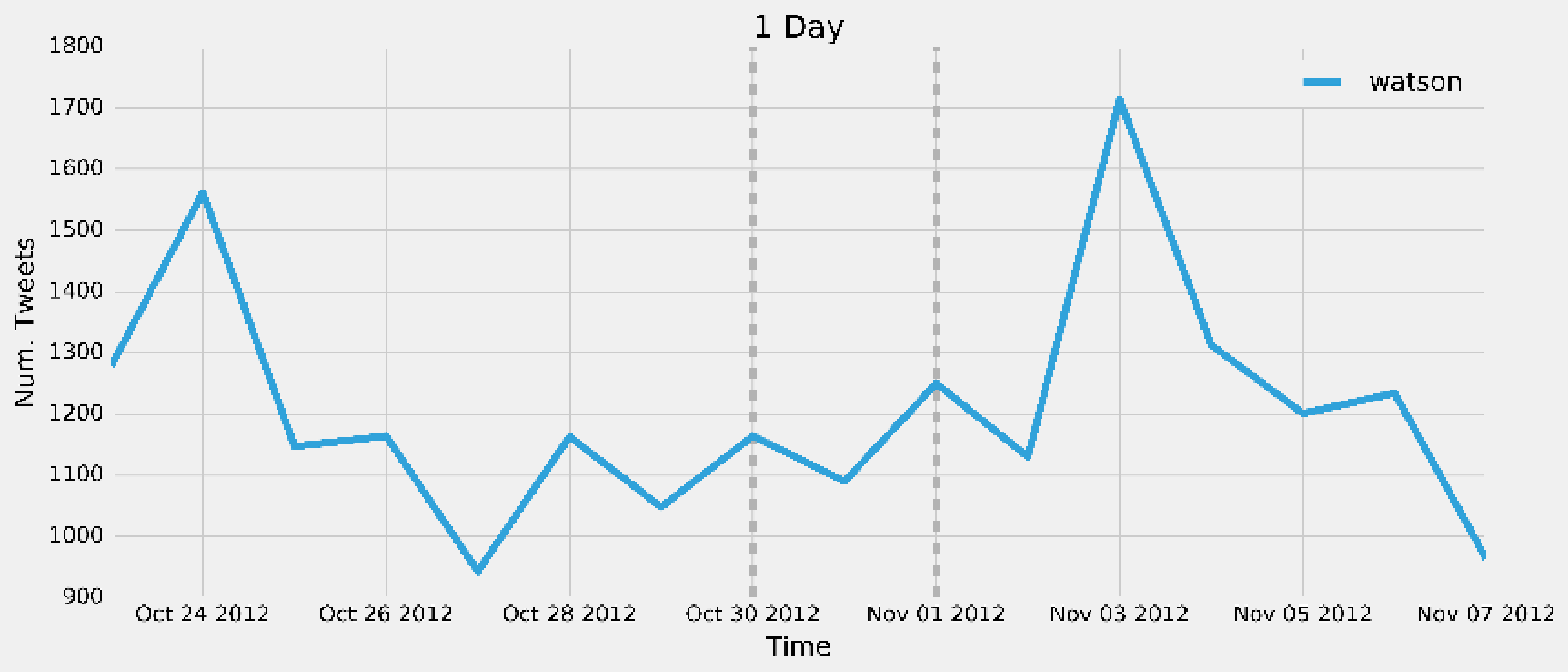

1 Day
watson
Num. Tweets
1800
1700
1600
1500
1400
1300
1200
1100
1000
900
Oct 24 2012
Oct 26 2012
Oct 28 2012
Oct 30 2012
Nov 01 2012
Nov 03 2012
Nov 05 2012
Nov 07 2012
Time

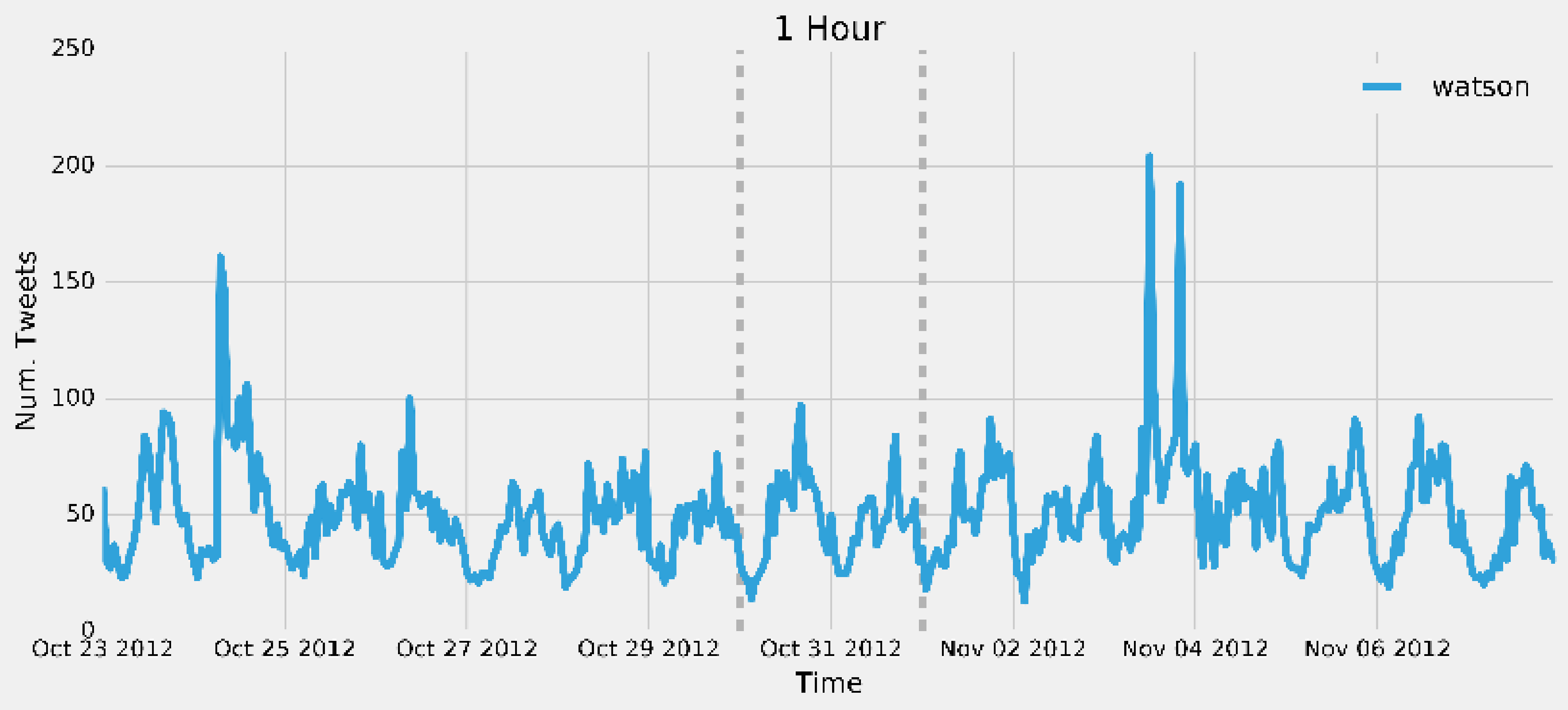
1 Hour
250
200
150
100
50
0
Num. Tweets
watson
Oct 23 2012
Oct 25 2012
Oct 27 2012
Oct 29 2012
Oct 31 2012
Nov 02 2012
Nov 04 2012
Nov 06 2012
Time

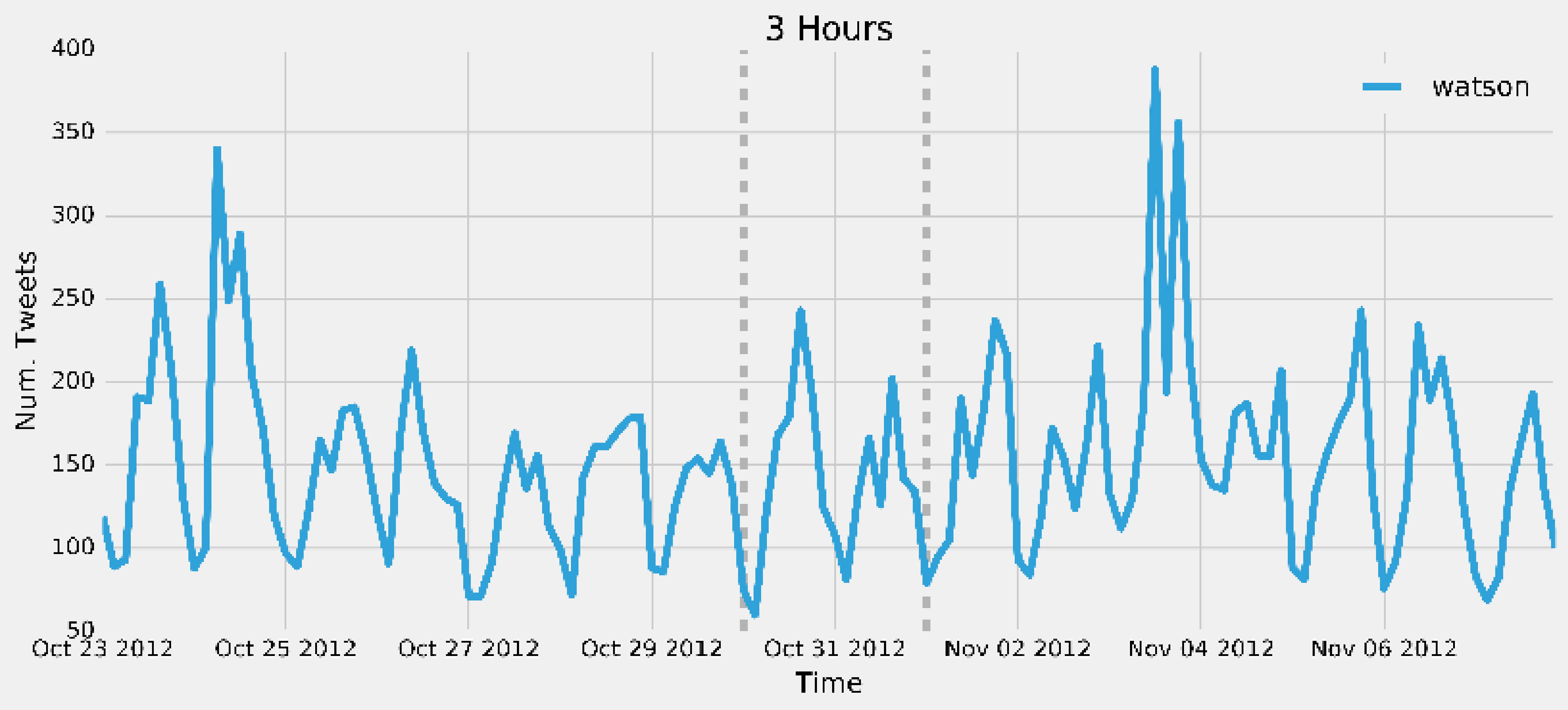
3 Hours
watson
Num. Tweets
400
350
300
250
200
150
100
50
Oct 23 2012
Oct 25 2012
Oct 27 2012
Oct 29 2012
Oct 31 2012
Nov 02 2012
Nov 04 2012
Nov 06 2012
Time

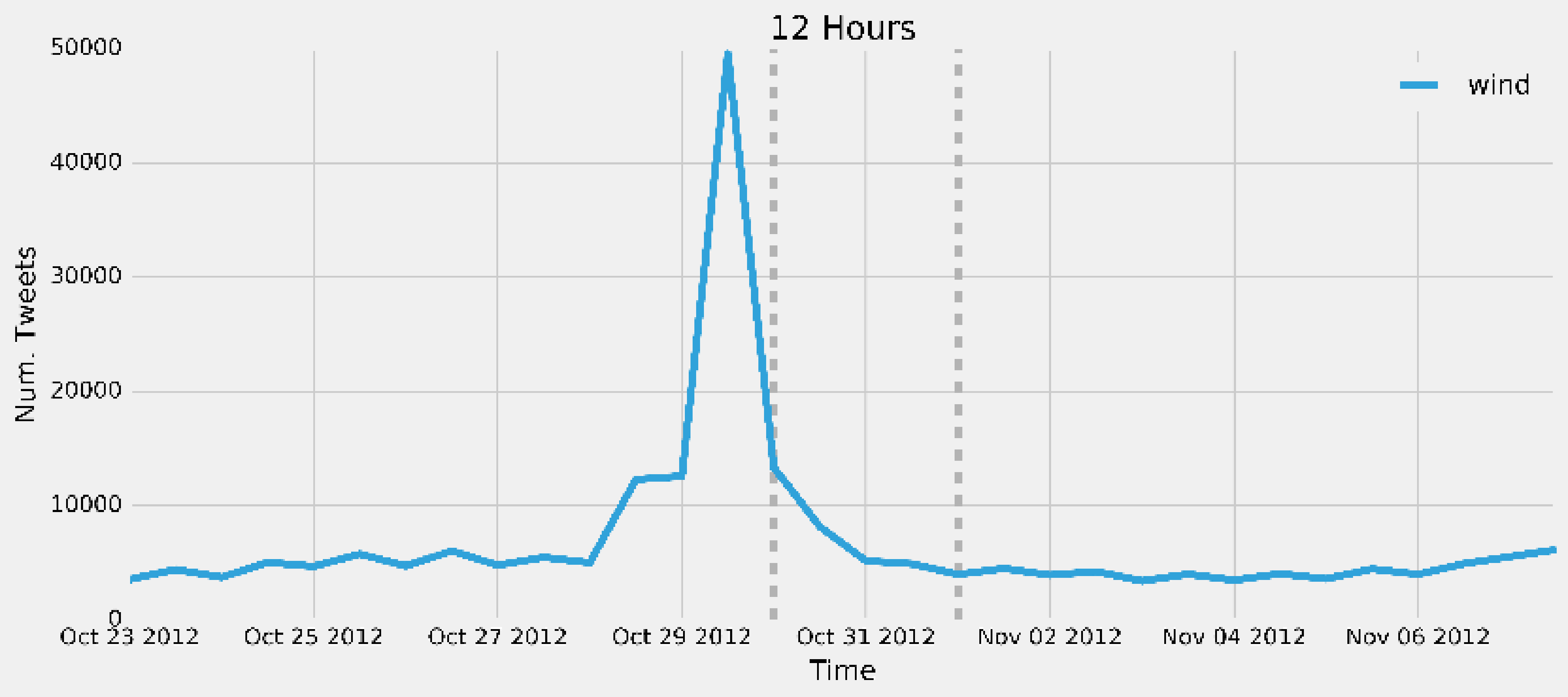
12 Hours
50000
40000
30000
20000
10000
0
Num. Tweets
wind
Oct 23 2012
Oct 25 2012
Oct 27 2012
Oct 29 2012
Oct 31 2012
Nov 02 2012
Nov 04 2012
Nov 06 2012
Time

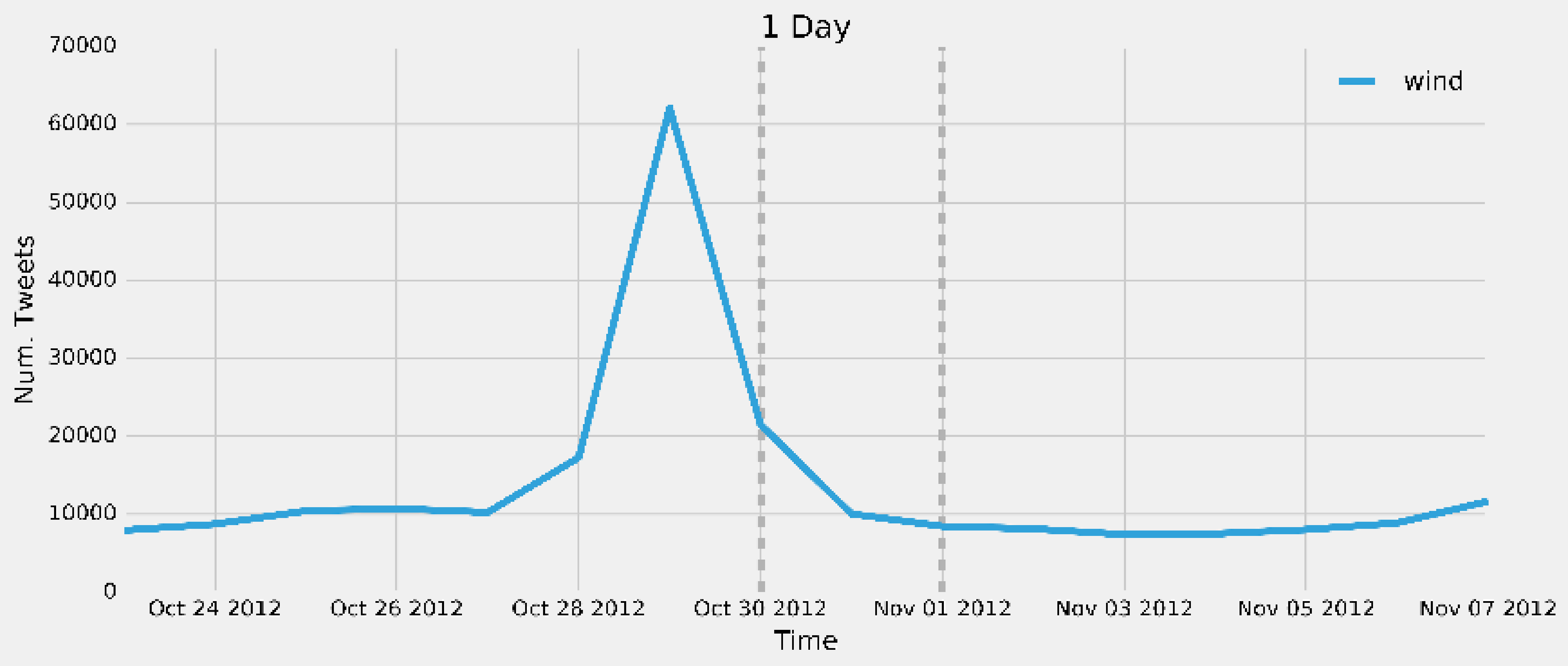
1 Day
70000
60000
50000
40000
30000
20000
10000
0
Num. Tweets
wind
Oct 24 2012
Oct 26 2012
Oct 28 2012
Oct 30 2012
Nov 01 2012
Nov 03 2012
Nov 05 2012
Nov 07 2012
Time

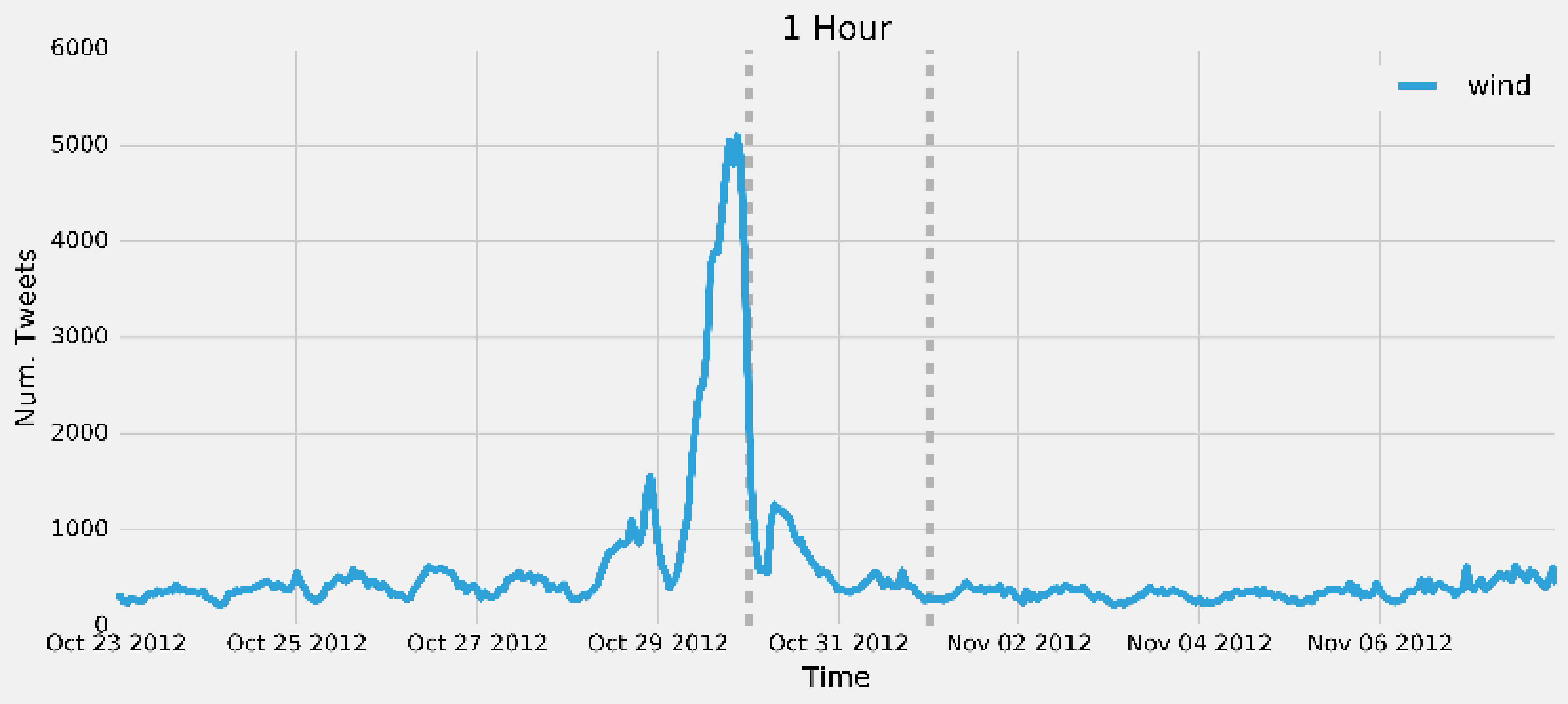
1 Hour
wind
Num. Tweets
0
1000
2000
3000
4000
5000
6000
Oct 23 2012
Oct 25 2012
Oct 27 2012
Oct 29 2012
Oct 31 2012
Nov 02 2012
Nov 04 2012
Nov 06 2012
Time

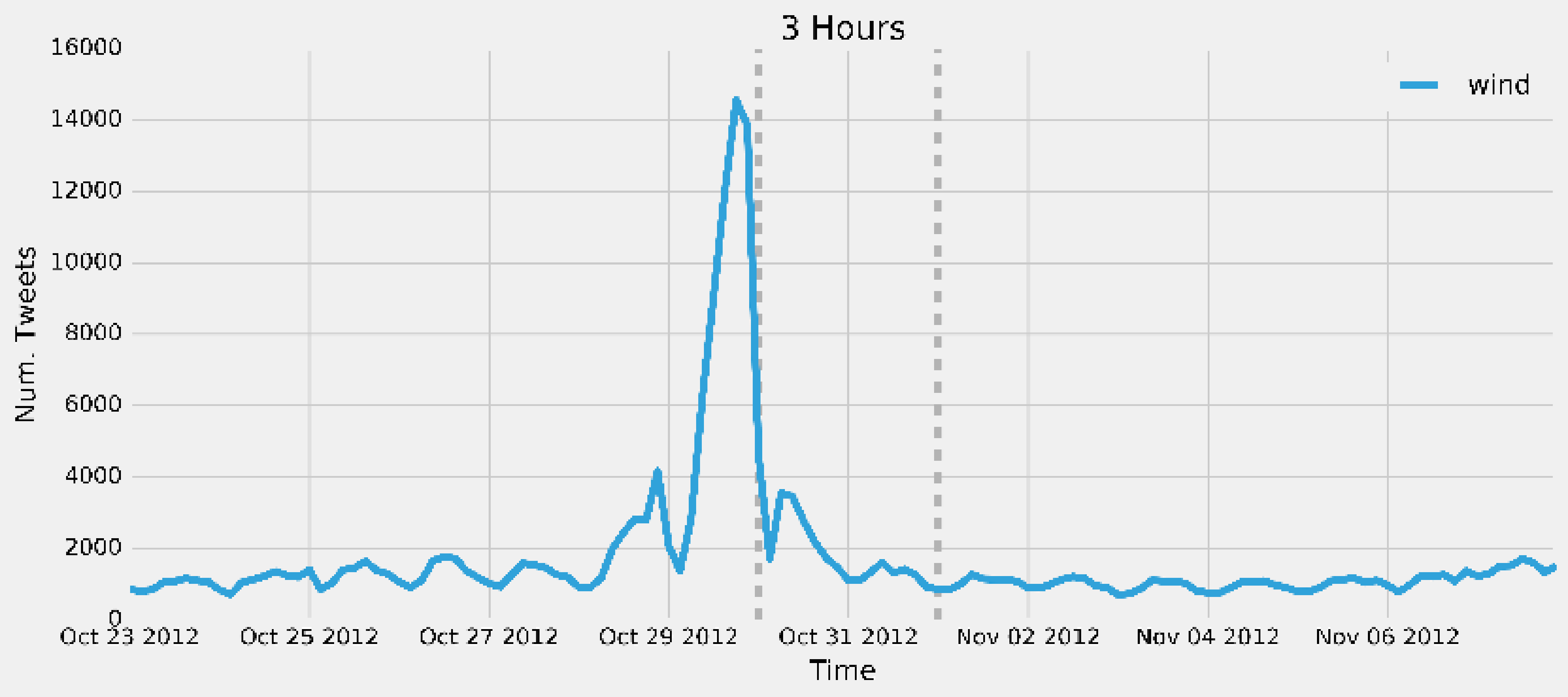
3 Hours
wind
Num. Tweets
16000
14000
12000
10000
8000
6000
4000
2000
0
Oct 23 2012
Oct 25 2012
Oct 27 2012
Oct 29 2012
Oct 31 2012
Nov 02 2012
Nov 04 2012
Nov 06 2012
Time
