# Supplementary material for: Social media usage patterns during natural hazards
Source: PLoS One. 2019 Feb 13;14(2):e0210484. doi: 10.1371/journal.pone.0210484 (PMC6374021; doi:10.1371/journal.pone.0210484)
Supplement: S2 Fig — (PDF) [file pone.0210484.s002.pdf]

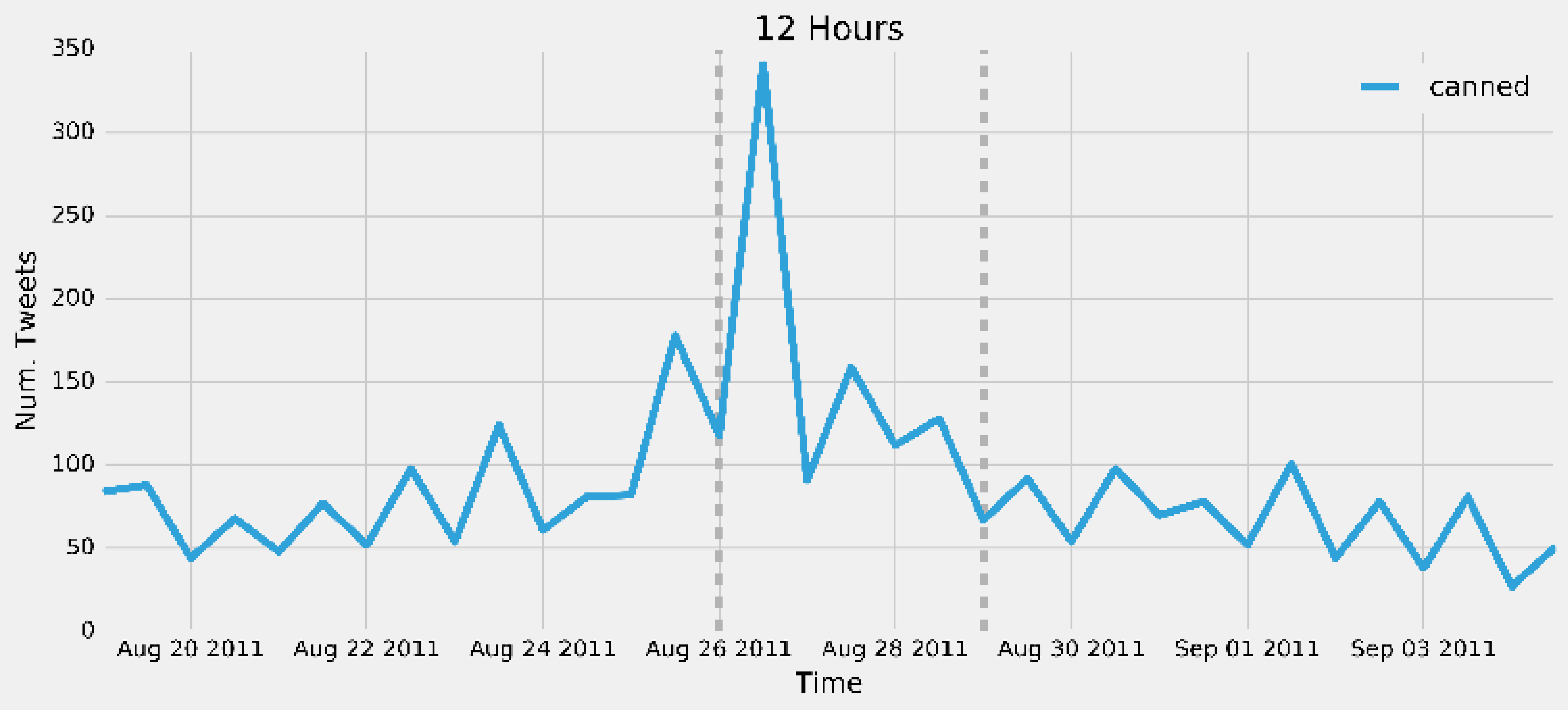

12 Hours
canned
Num. Tweets
0
50
100
150
200
250
300
350
Aug 20 2011
Aug 22 2011
Aug 24 2011
Aug 26 2011
Aug 28 2011
Aug 30 2011
Sep 01 2011
Sep 03 2011
Time

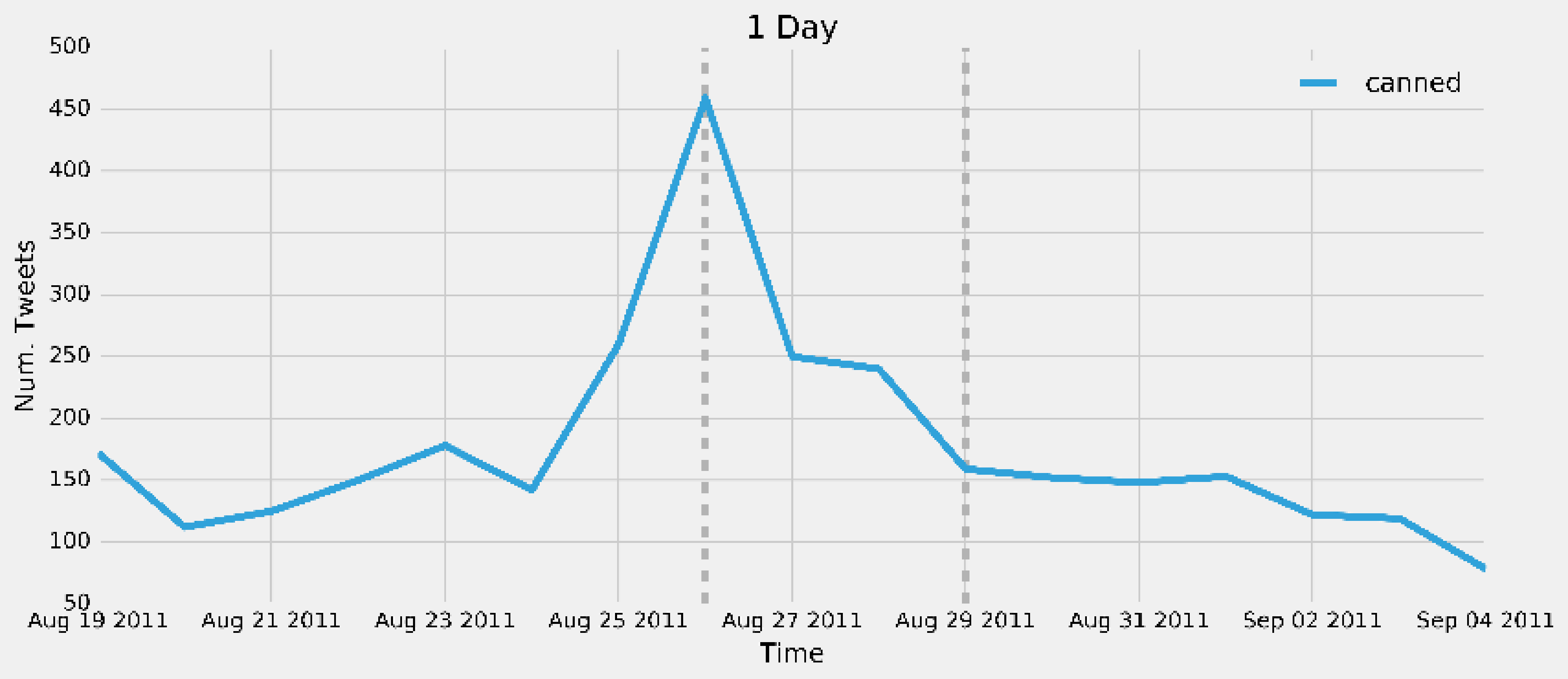
1 Day
canned
Num. Tweets
Time
500
450
400
350
300
250
200
150
100
50
Aug 19 2011
Aug 21 2011
Aug 23 2011
Aug 25 2011
Aug 27 2011
Aug 29 2011
Aug 31 2011
Sep 02 2011
Sep 04 2011

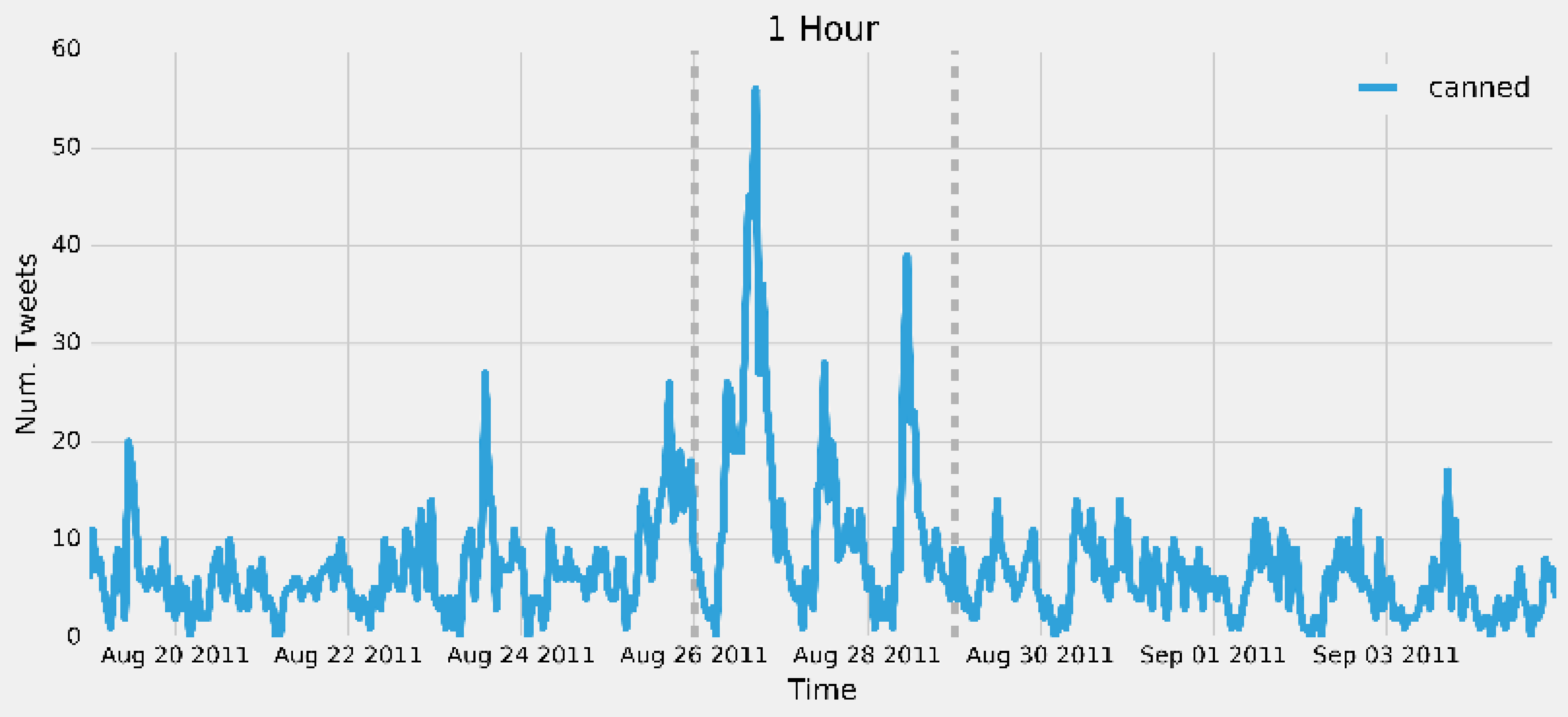
1 Hour
canned
Num. Tweets
Time
0
10
20
30
40
50
60
Aug 20 2011
Aug 22 2011
Aug 24 2011
Aug 26 2011
Aug 28 2011
Aug 30 2011
Sep 01 2011
Sep 03 2011

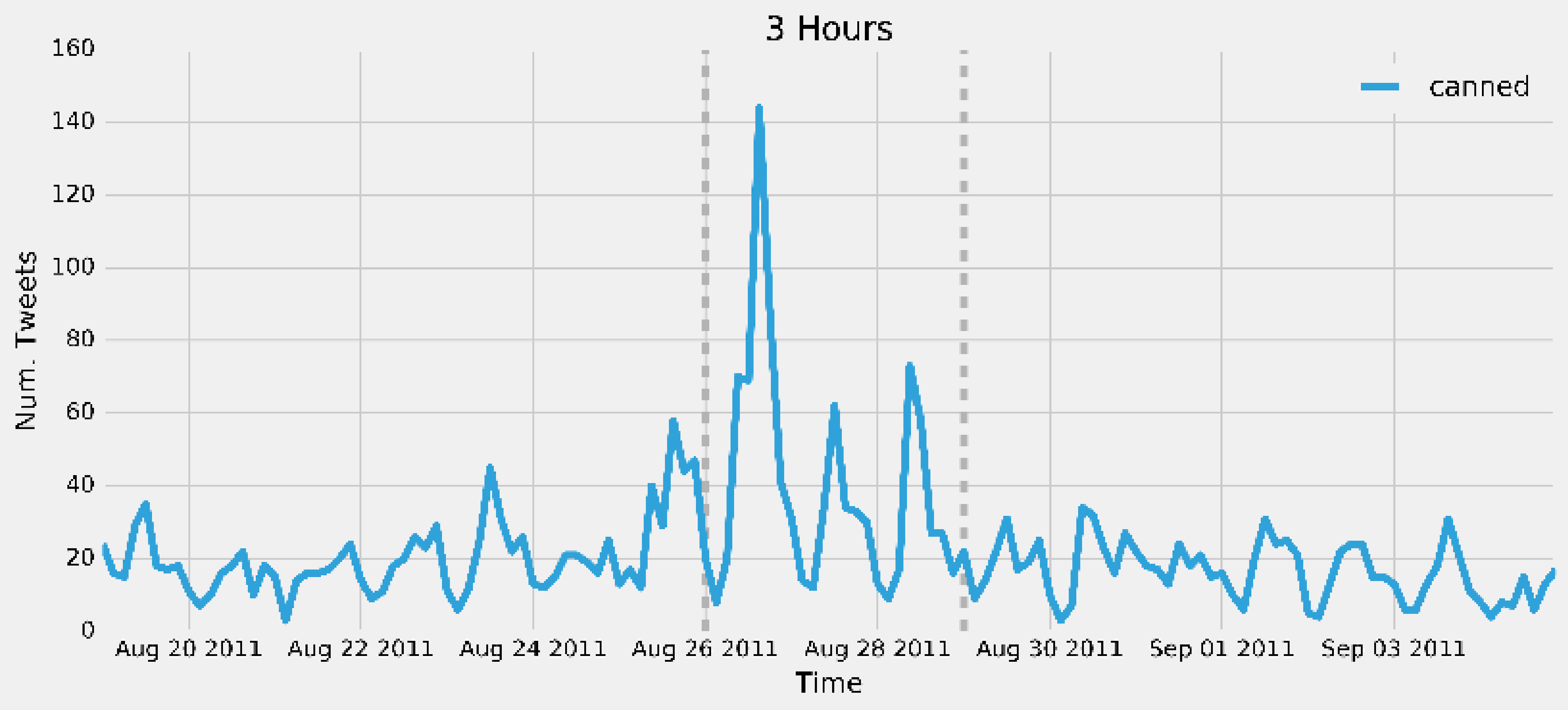
3 Hours
canned
Num. Tweets
Time
0
20
40
60
80
100
120
140
160
Aug 20 2011
Aug 22 2011
Aug 24 2011
Aug 26 2011
Aug 28 2011
Aug 30 2011
Sep 01 2011
Sep 03 2011

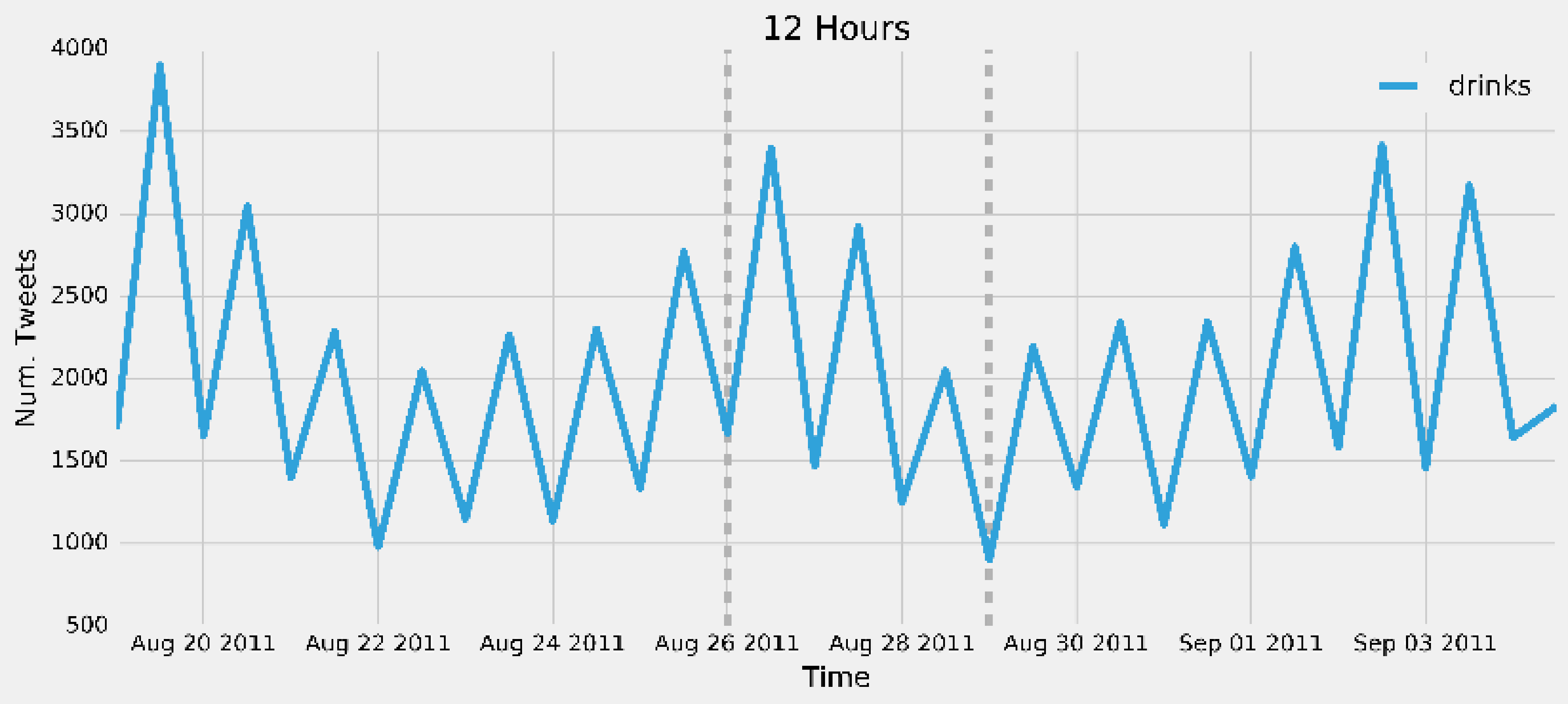

12 Hours
drinks
Num. Tweets
4000
3500
3000
2500
2000
1500
1000
500
Aug 20 2011
Aug 22 2011
Aug 24 2011
Aug 26 2011
Aug 28 2011
Aug 30 2011
Sep 01 2011
Sep 03 2011
Time

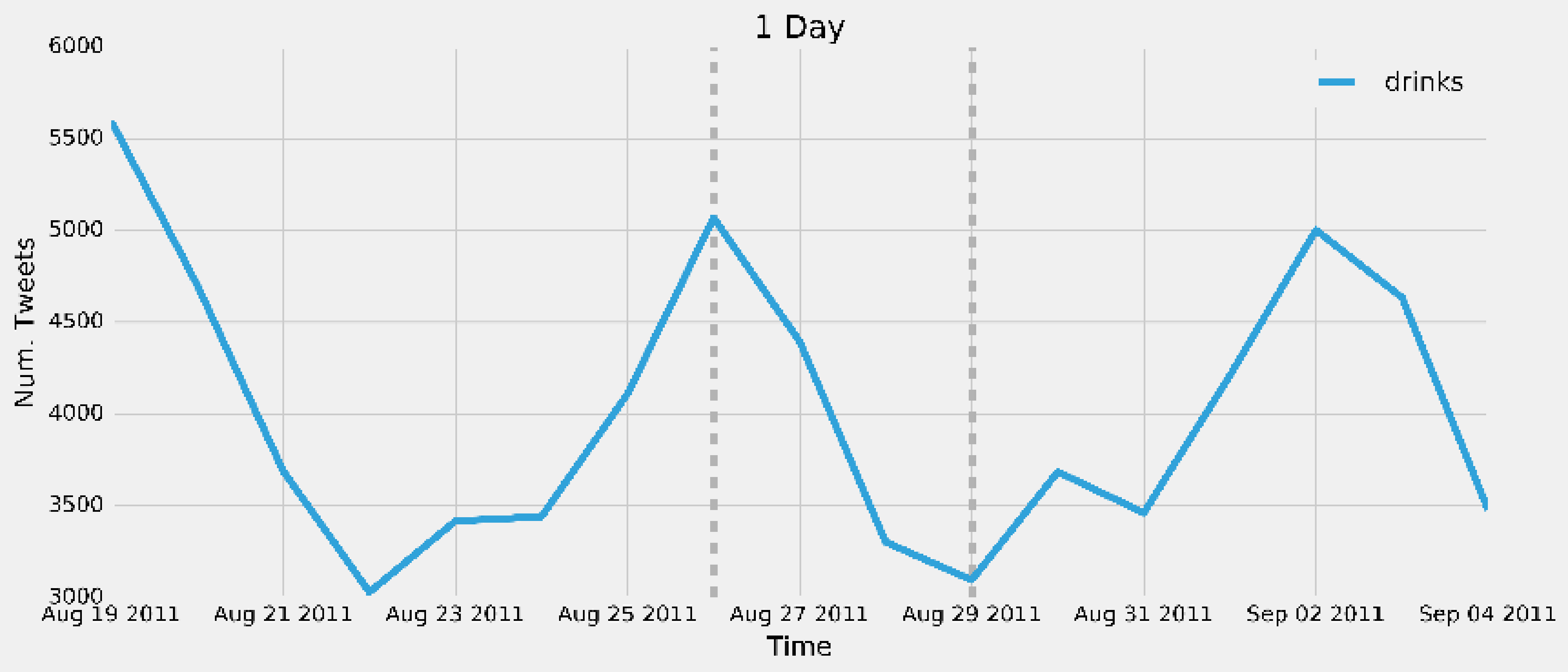
1 Day
drinks
Num. Tweets
Time
6000
5500
5000
4500
4000
3500
3000
Aug 19 2011
Aug 21 2011
Aug 23 2011
Aug 25 2011
Aug 27 2011
Aug 29 2011
Aug 31 2011
Sep 02 2011
Sep 04 2011

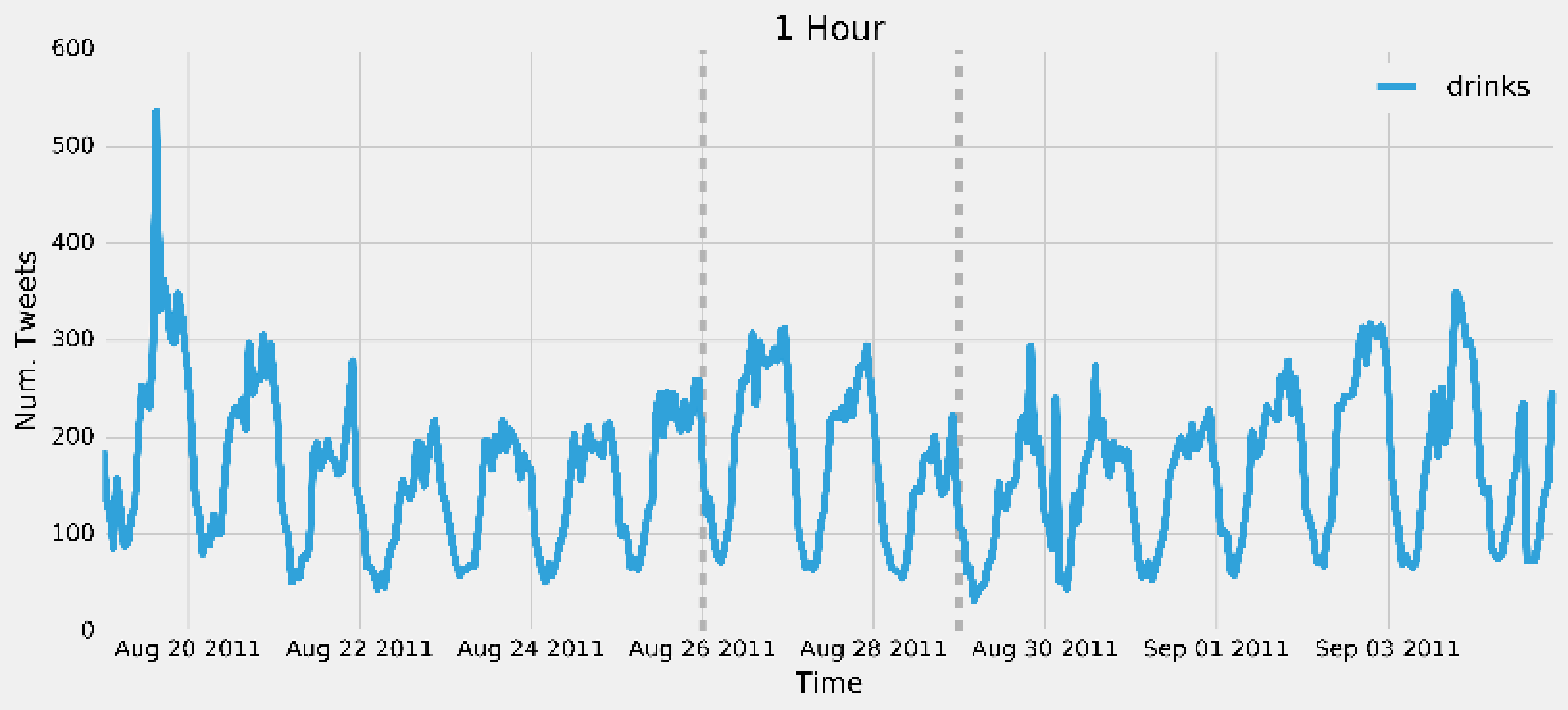

1 Hour
drinks
Num. Tweets
600
500
400
300
200
100
0
Aug 20 2011
Aug 22 2011
Aug 24 2011
Aug 26 2011
Aug 28 2011
Aug 30 2011
Sep 01 2011
Sep 03 2011
Time

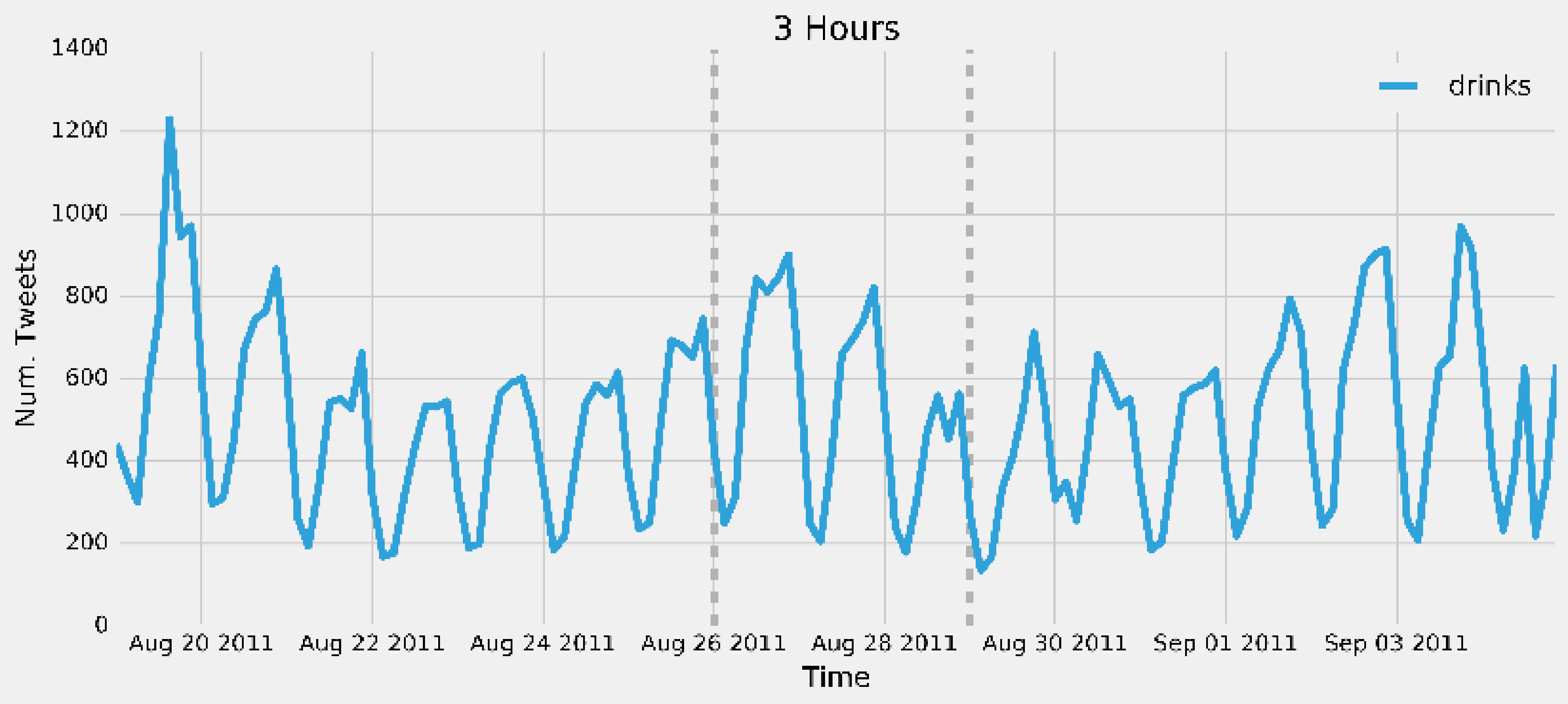

3 Hours
drinks
Num. Tweets
Time
0
200
400
600
800
1000
1200
1400
Aug 20 2011
Aug 22 2011
Aug 24 2011
Aug 26 2011
Aug 28 2011
Aug 30 2011
Sep 01 2011
Sep 03 2011

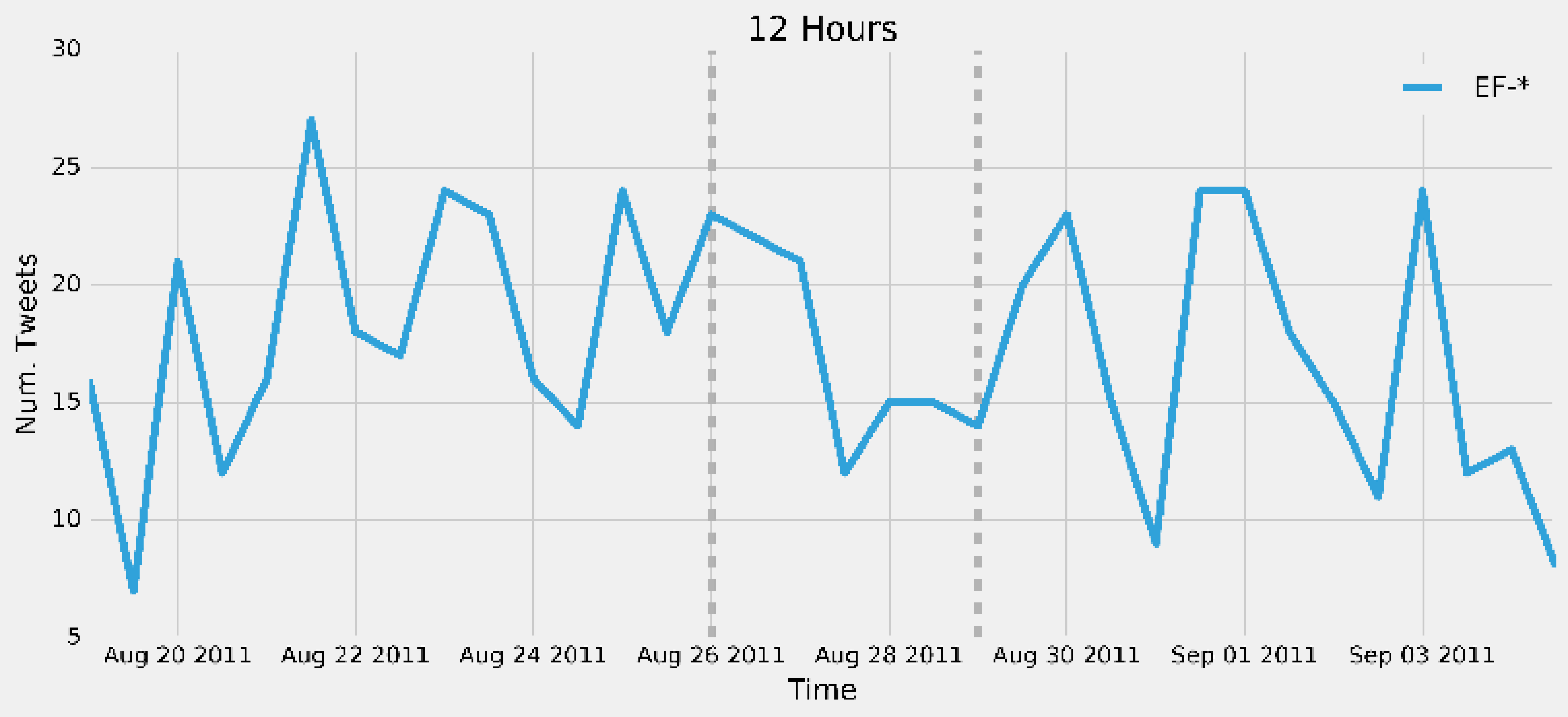
12 Hours
EF-*
Num. Tweets
30
25
20
15
10
5
Aug 20 2011
Aug 22 2011
Aug 24 2011
Aug 26 2011
Aug 28 2011
Aug 30 2011
Sep 01 2011
Sep 03 2011
Time

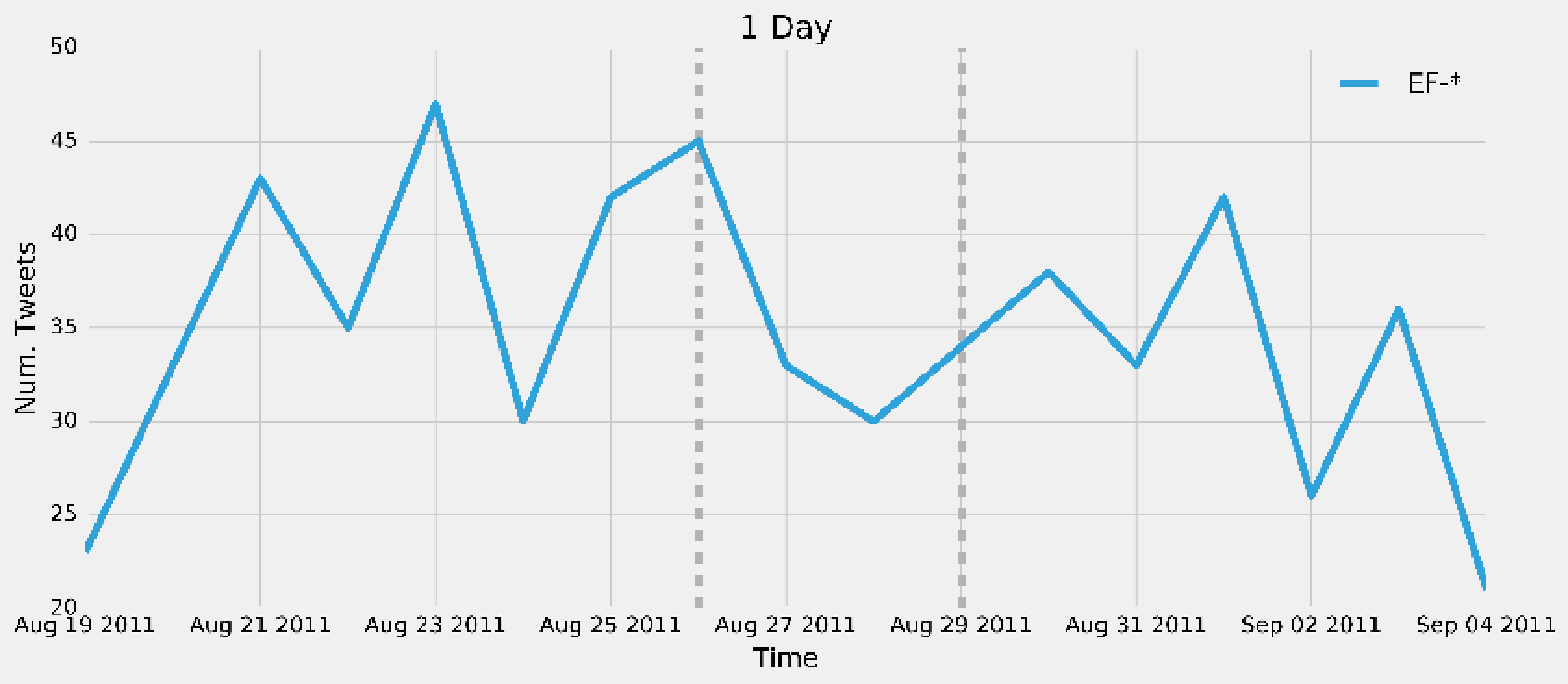

1 Day
EF-*
Num. Tweets
Time
50
45
40
35
30
25
20
Aug 19 2011
Aug 21 2011
Aug 23 2011
Aug 25 2011
Aug 27 2011
Aug 29 2011
Aug 31 2011
Sep 02 2011
Sep 04 2011

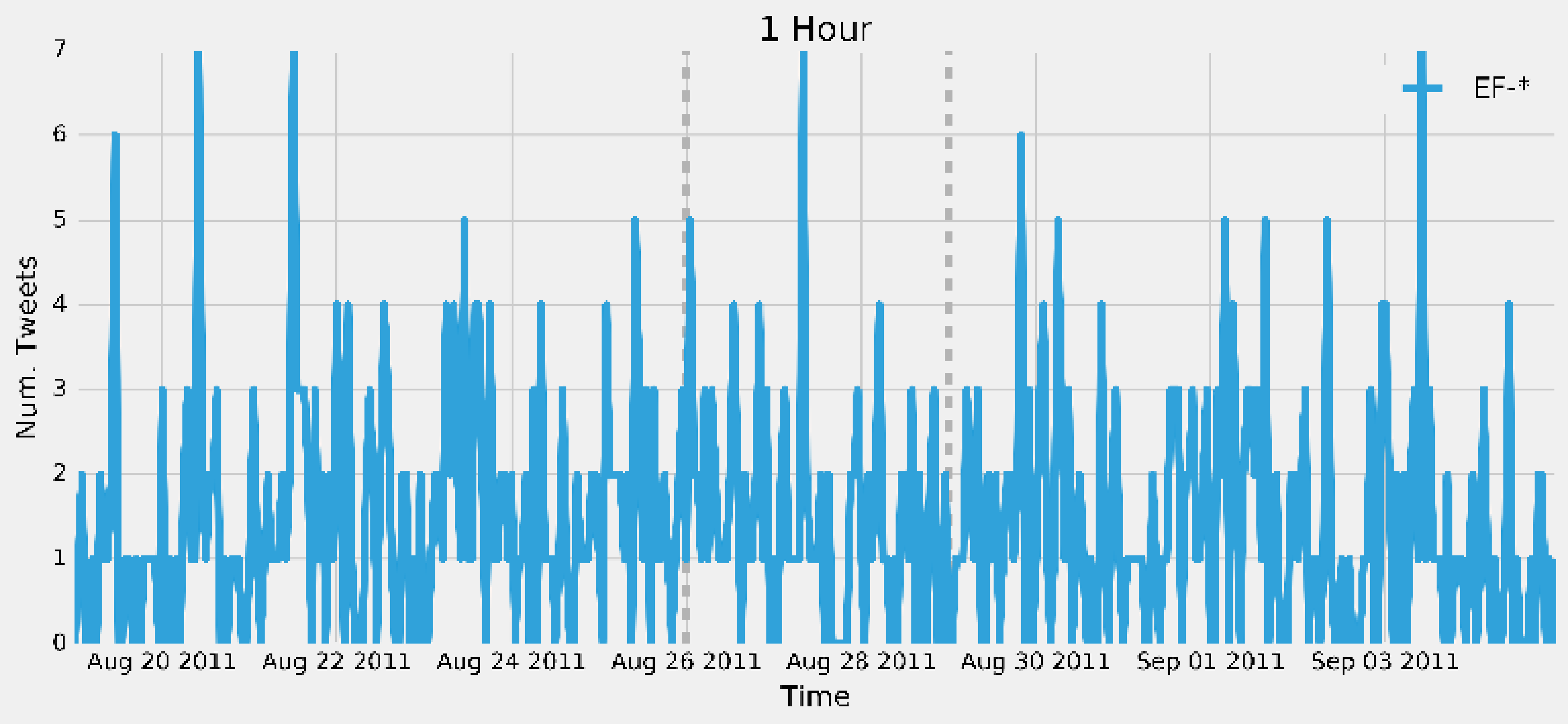

1 Hour
EF-*
Num. Tweets
Time
0
1
2
3
4
5
6
7
Aug 20 2011
Aug 22 2011
Aug 24 2011
Aug 26 2011
Aug 28 2011
Aug 30 2011
Sep 01 2011
Sep 03 2011

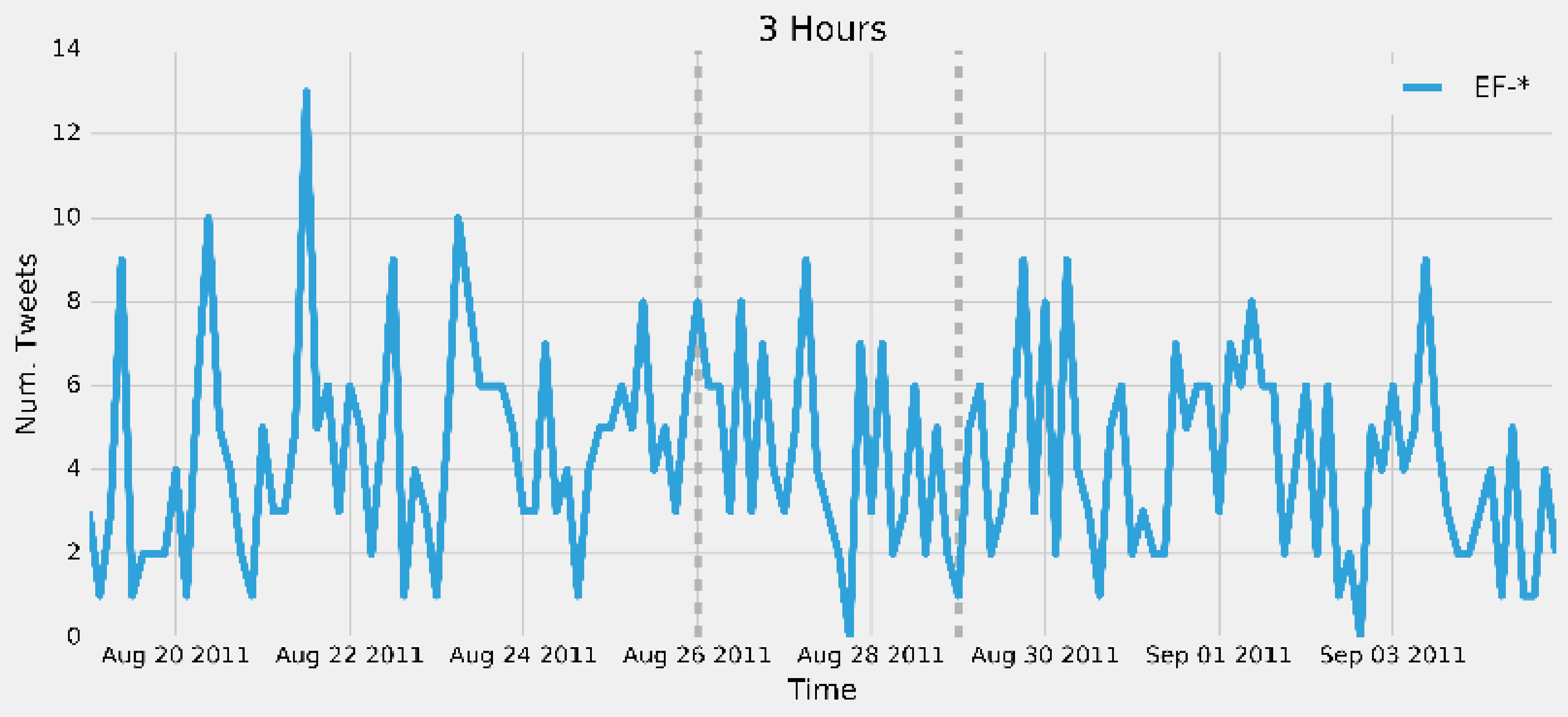
3 Hours
EF-*
Num. Tweets
Time
0
2
4
6
8
10
12
14
Aug 20 2011
Aug 22 2011
Aug 24 2011
Aug 26 2011
Aug 28 2011
Aug 30 2011
Sep 01 2011
Sep 03 2011

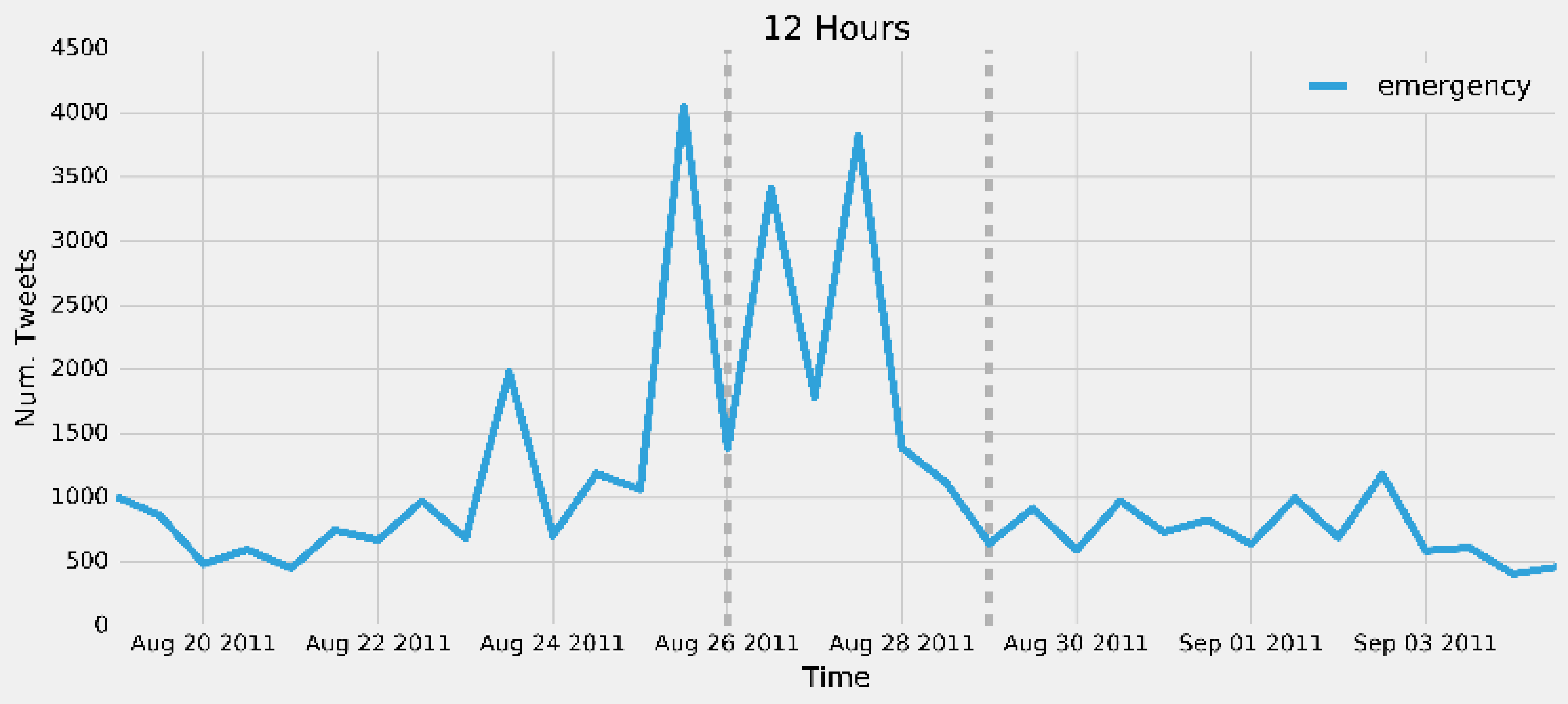
12 Hours
emergency
Num. Tweets
0
500
1000
1500
2000
2500
3000
3500
4000
4500
Aug 20 2011
Aug 22 2011
Aug 24 2011
Aug 26 2011
Aug 28 2011
Aug 30 2011
Sep 01 2011
Sep 03 2011
Time

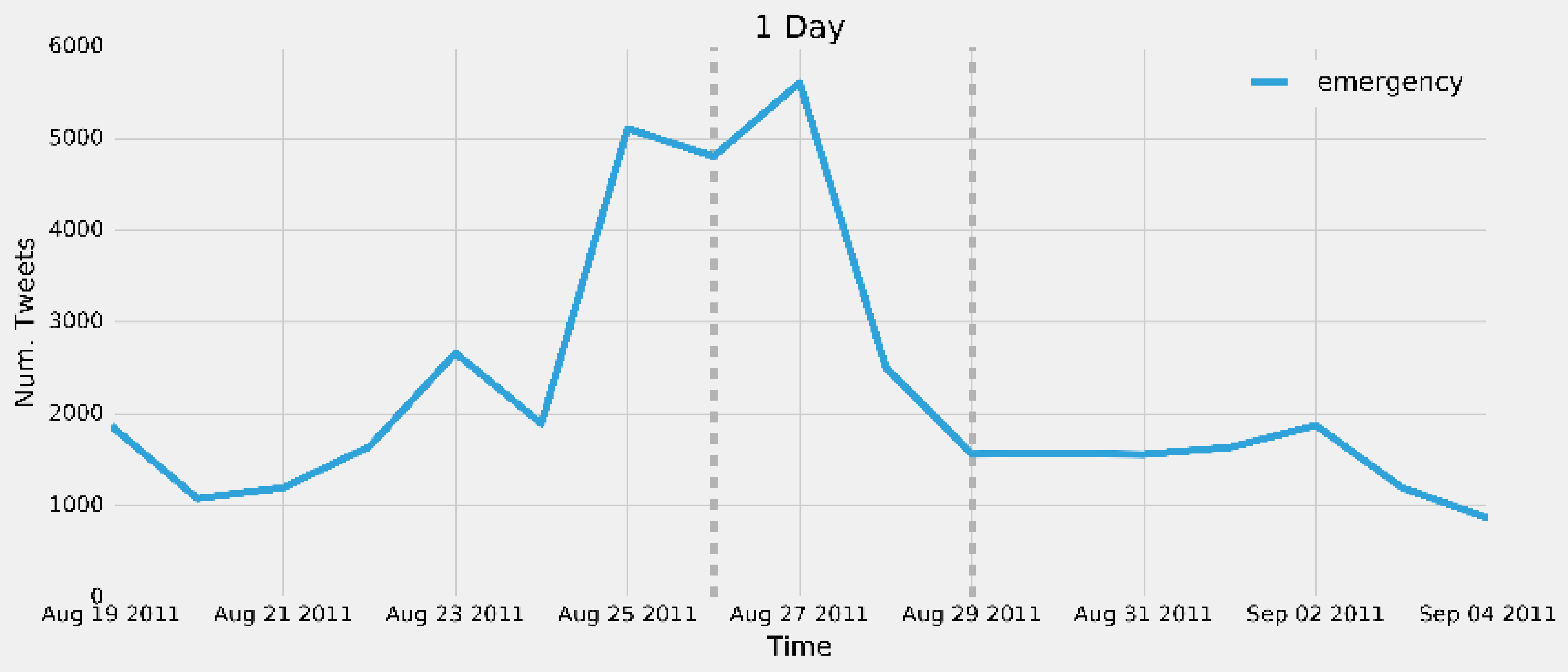
1 Day
emergency
Num. Tweets
0
1000
2000
3000
4000
5000
6000
Aug 19 2011
Aug 21 2011
Aug 23 2011
Aug 25 2011
Aug 27 2011
Aug 29 2011
Aug 31 2011
Sep 02 2011
Sep 04 2011
Time

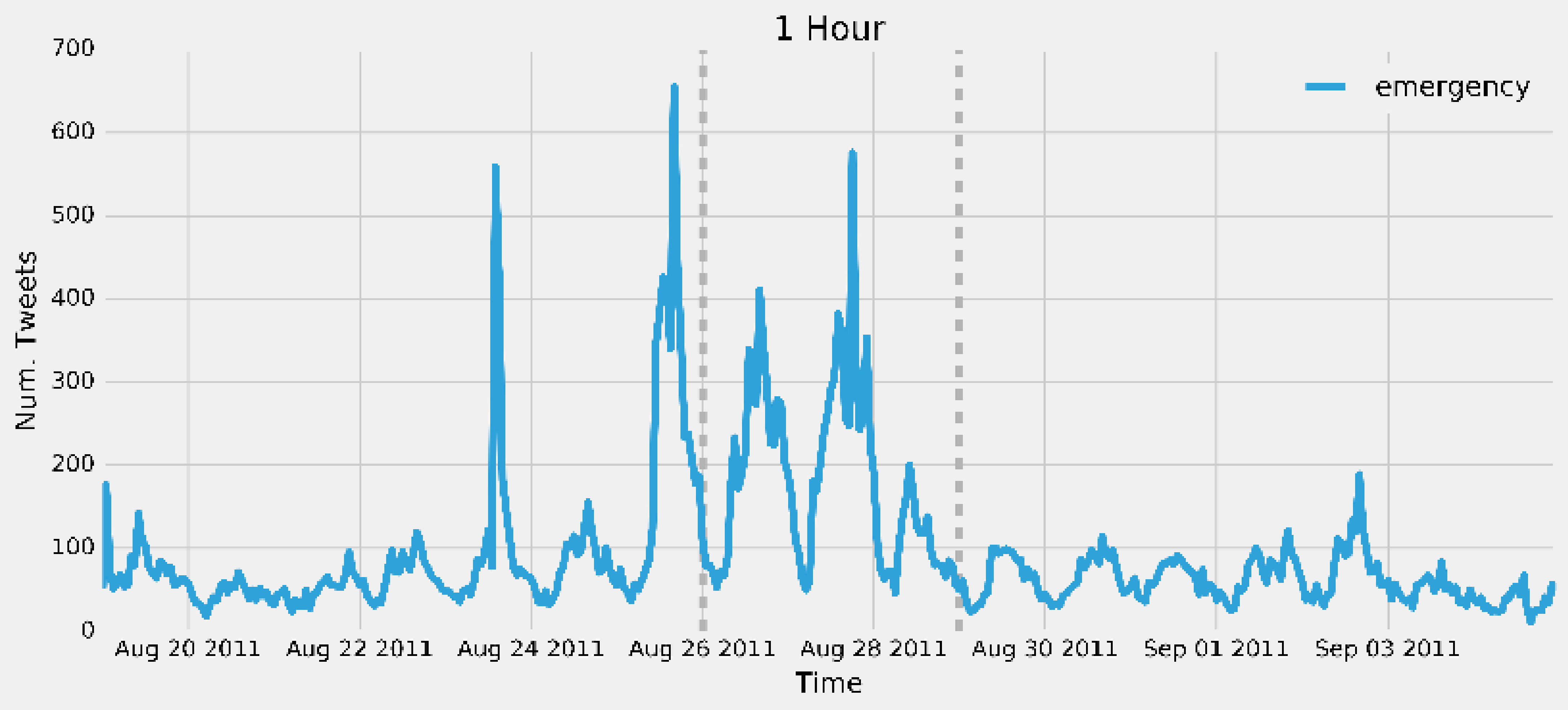

1 Hour
700
600
500
400
300
200
100
0
Num. Tweets
emergency
Aug 20 2011
Aug 22 2011
Aug 24 2011
Aug 26 2011
Aug 28 2011
Aug 30 2011
Sep 01 2011
Sep 03 2011
Time

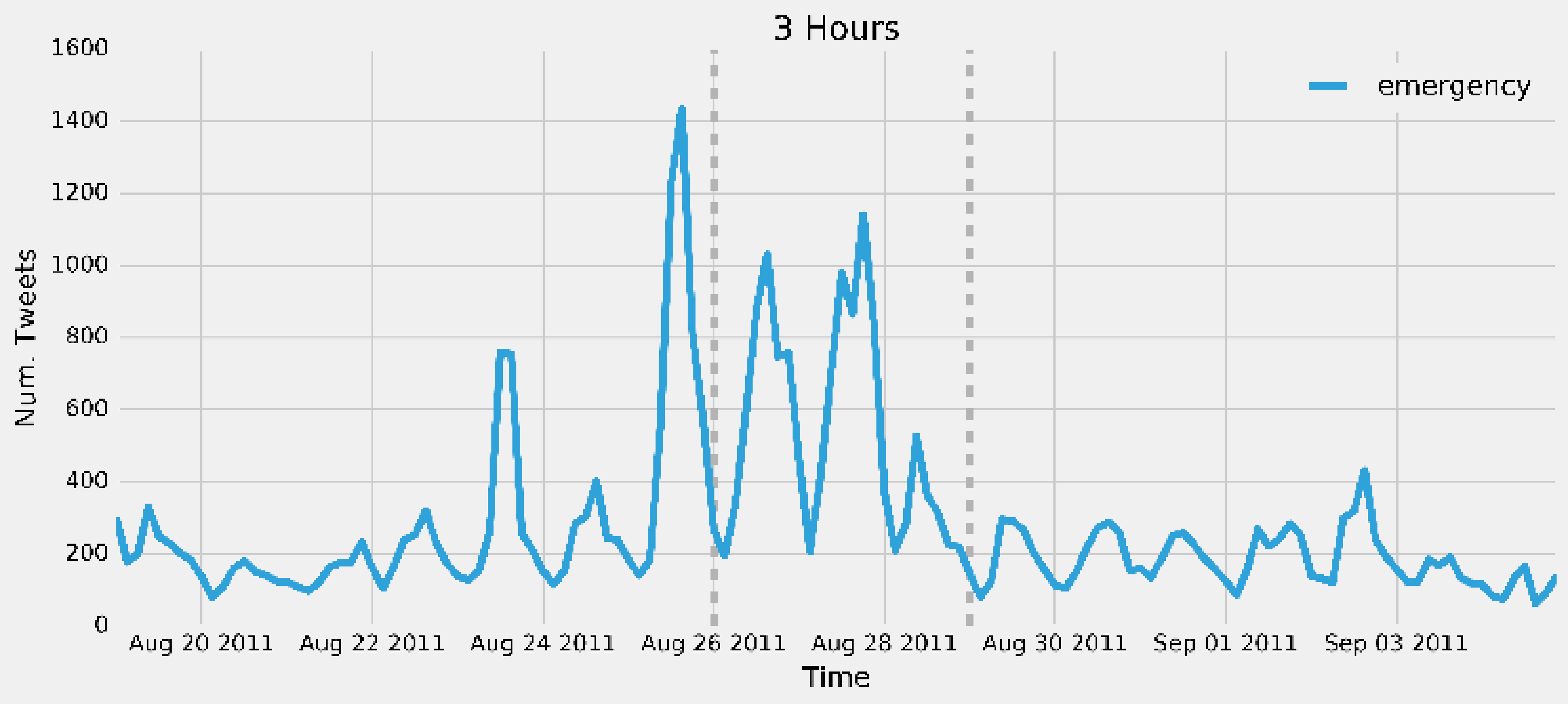

3 Hours
emergency
Num. Tweets
0
200
400
600
800
1000
1200
1400
1600
Aug 20 2011
Aug 22 2011
Aug 24 2011
Aug 26 2011
Aug 28 2011
Aug 30 2011
Sep 01 2011
Sep 03 2011
Time

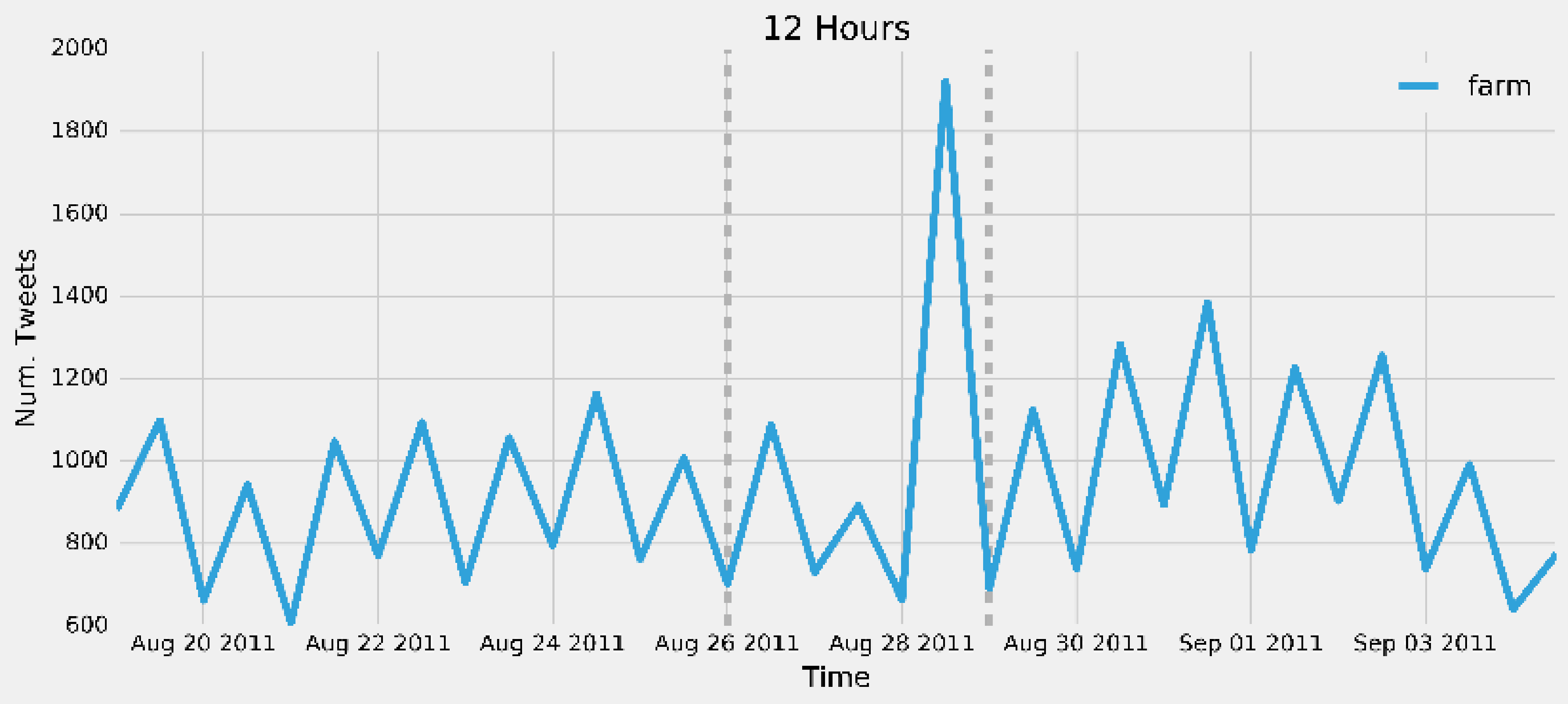

12 Hours
farm
Num. Tweets
Time
2000
1800
1600
1400
1200
1000
800
600
Aug 20 2011
Aug 22 2011
Aug 24 2011
Aug 26 2011
Aug 28 2011
Aug 30 2011
Sep 01 2011
Sep 03 2011

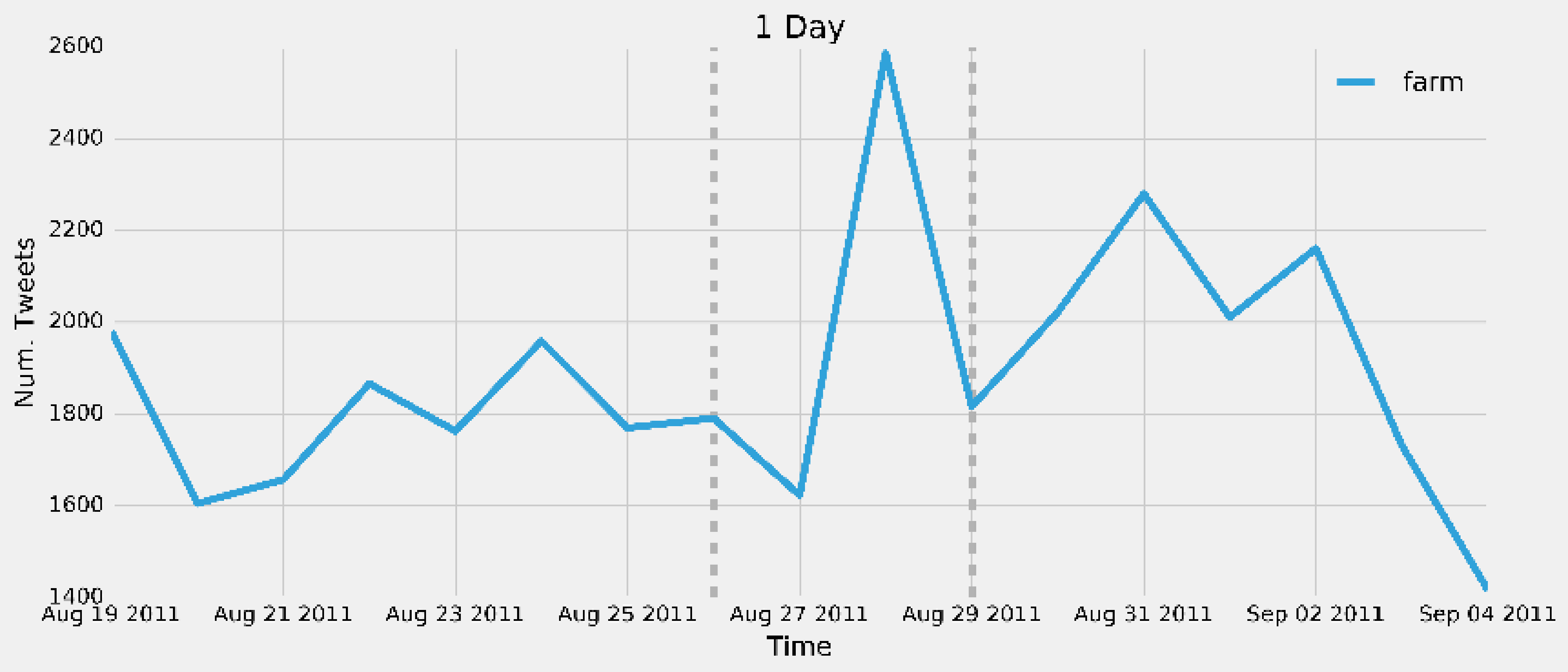

1 Day
farm
Num. Tweets
2600
2400
2200
2000
1800
1600
1400
Aug 19 2011
Aug 21 2011
Aug 23 2011
Aug 25 2011
Aug 27 2011
Aug 29 2011
Aug 31 2011
Sep 02 2011
Sep 04 2011
Time

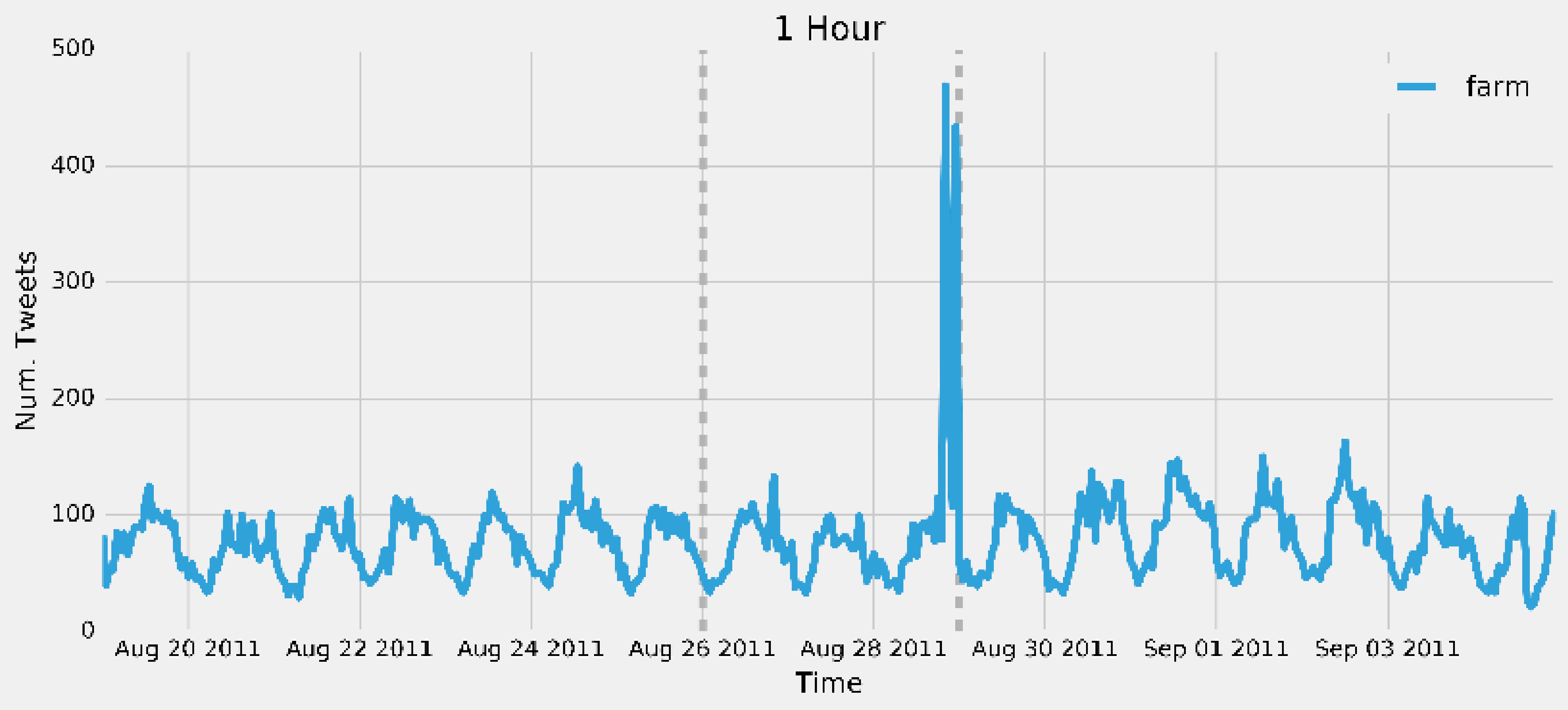

1 Hour
500
400
300
200
100
0
Num. Tweets
farm
Aug 20 2011
Aug 22 2011
Aug 24 2011
Aug 26 2011
Aug 28 2011
Aug 30 2011
Sep 01 2011
Sep 03 2011
Time

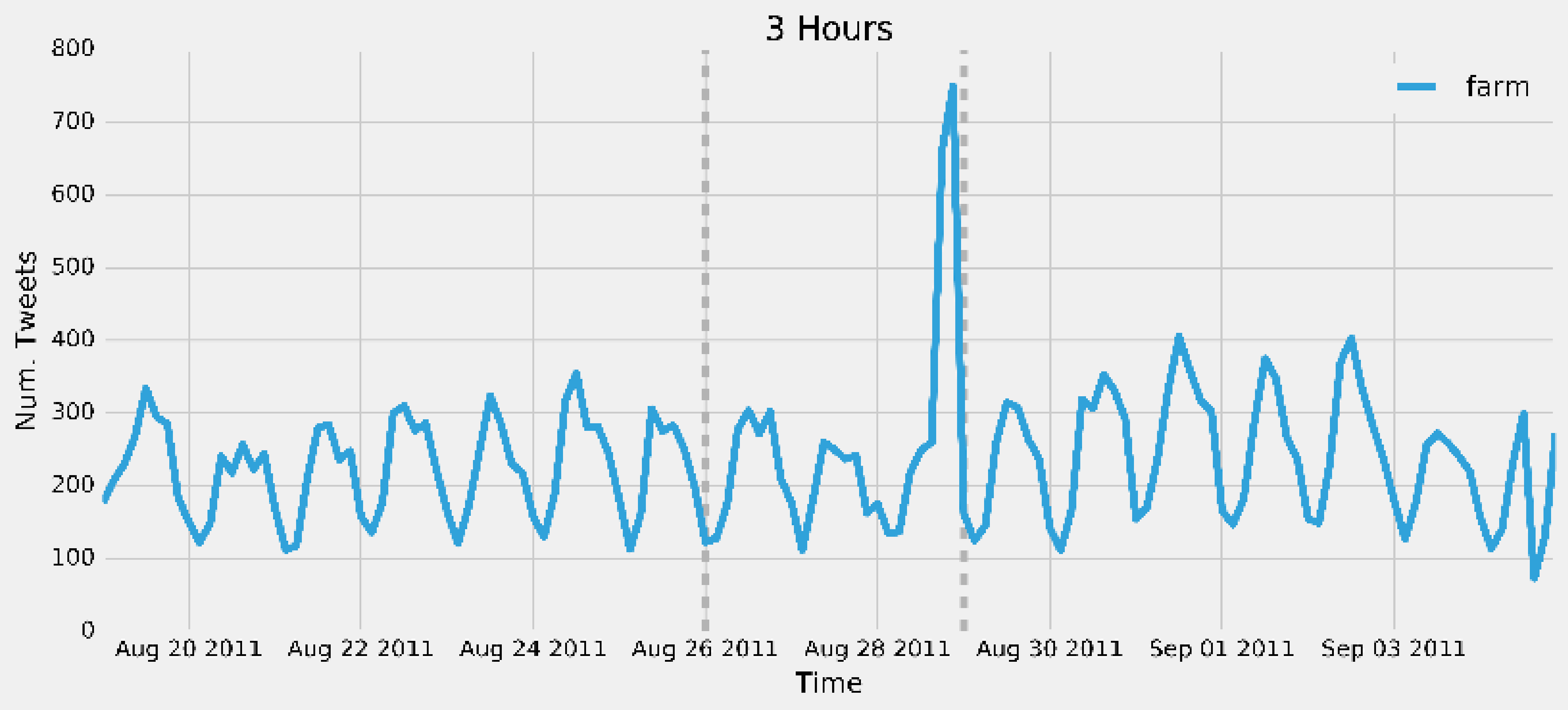

3 Hours
farm
Num. Tweets
Time
0
100
200
300
400
500
600
700
800
Aug 20 2011
Aug 22 2011
Aug 24 2011
Aug 26 2011
Aug 28 2011
Aug 30 2011
Sep 01 2011
Sep 03 2011

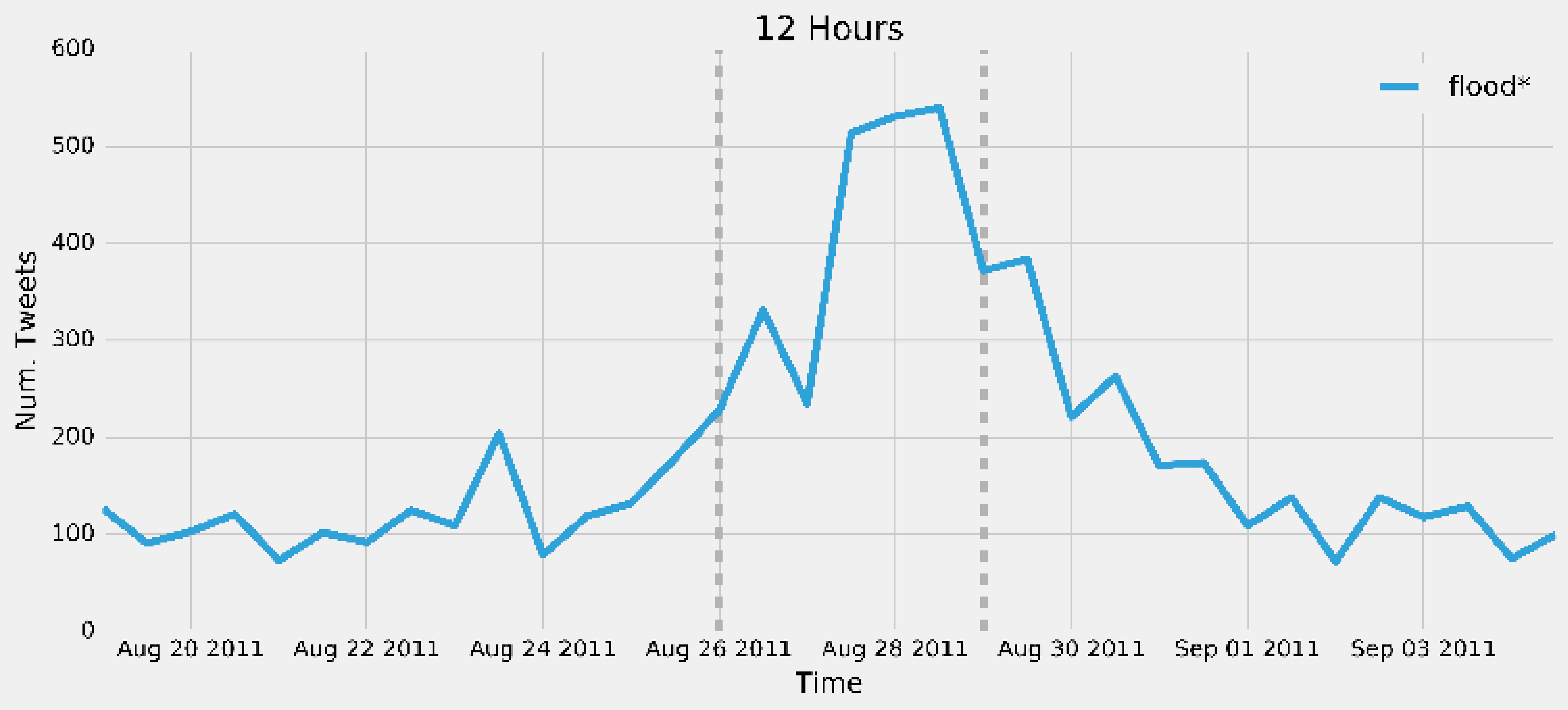
12 Hours
flood*
Num. Tweets
Time
0
100
200
300
400
500
600
Aug 20 2011
Aug 22 2011
Aug 24 2011
Aug 26 2011
Aug 28 2011
Aug 30 2011
Sep 01 2011
Sep 03 2011

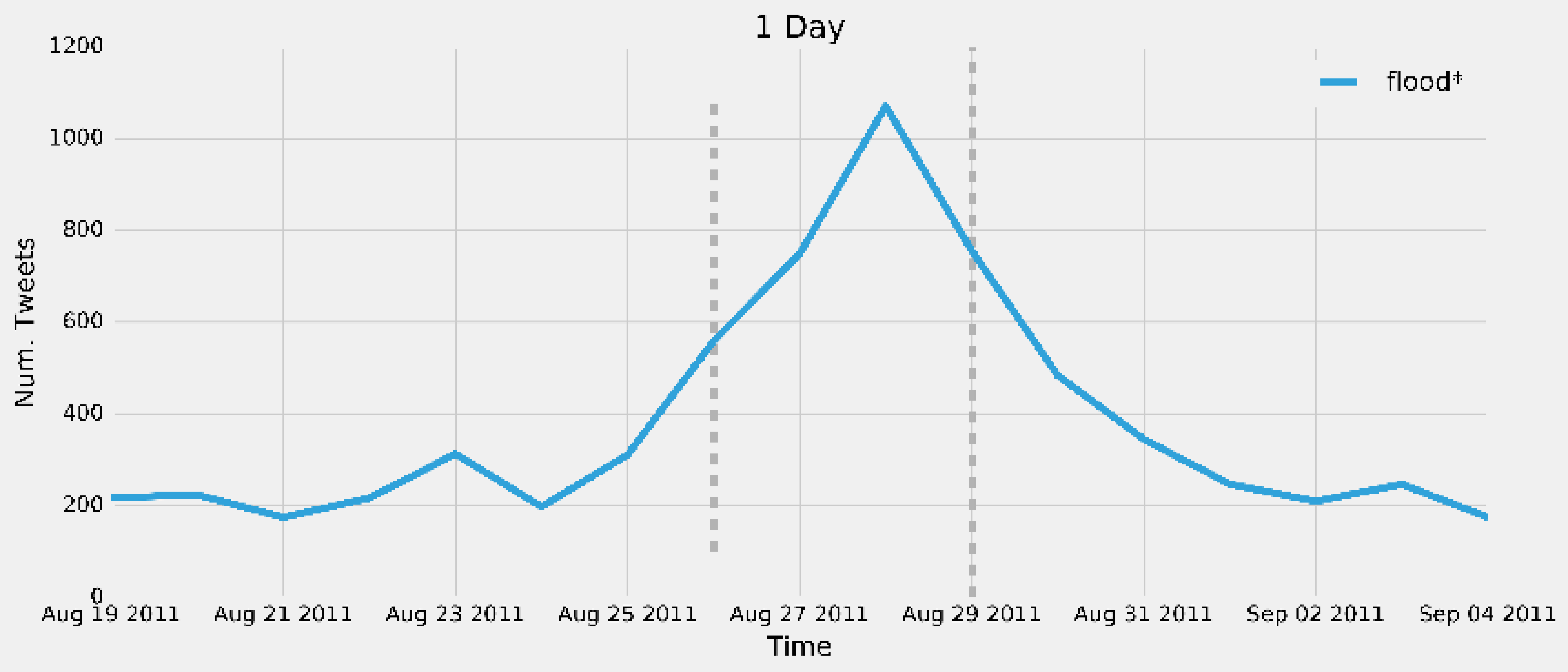
1 Day
flood*
Num. Tweets
0
200
400
600
800
1000
1200
Aug 19 2011
Aug 21 2011
Aug 23 2011
Aug 25 2011
Aug 27 2011
Aug 29 2011
Aug 31 2011
Sep 02 2011
Sep 04 2011
Time

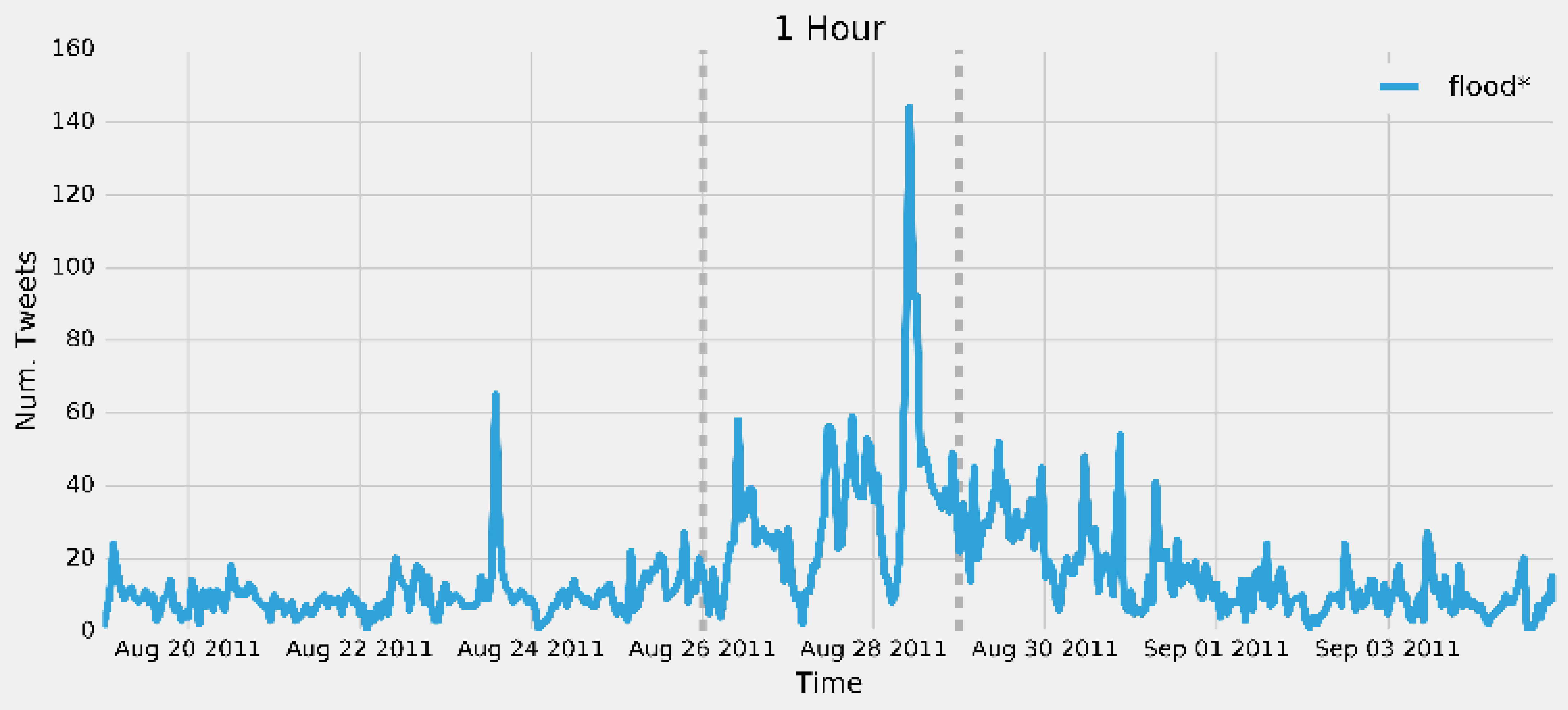
1 Hour
flood*
Num. Tweets
0
20
40
60
80
100
120
140
160
Aug 20 2011
Aug 22 2011
Aug 24 2011
Aug 26 2011
Aug 28 2011
Aug 30 2011
Sep 01 2011
Sep 03 2011
Time

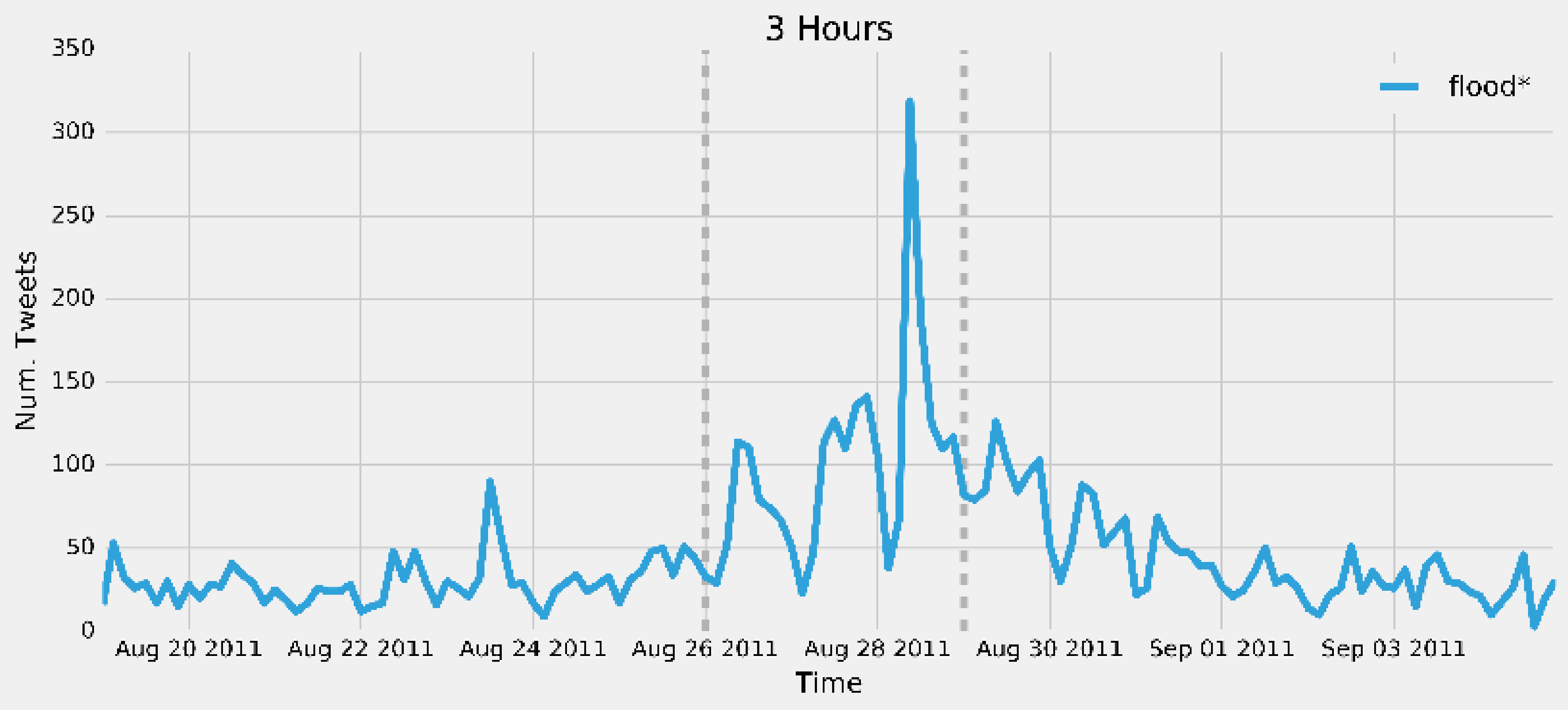
3 Hours
flood*
Num. Tweets
Time
0
50
100
150
200
250
300
350
Aug 20 2011
Aug 22 2011
Aug 24 2011
Aug 26 2011
Aug 28 2011
Aug 30 2011
Sep 01 2011
Sep 03 2011

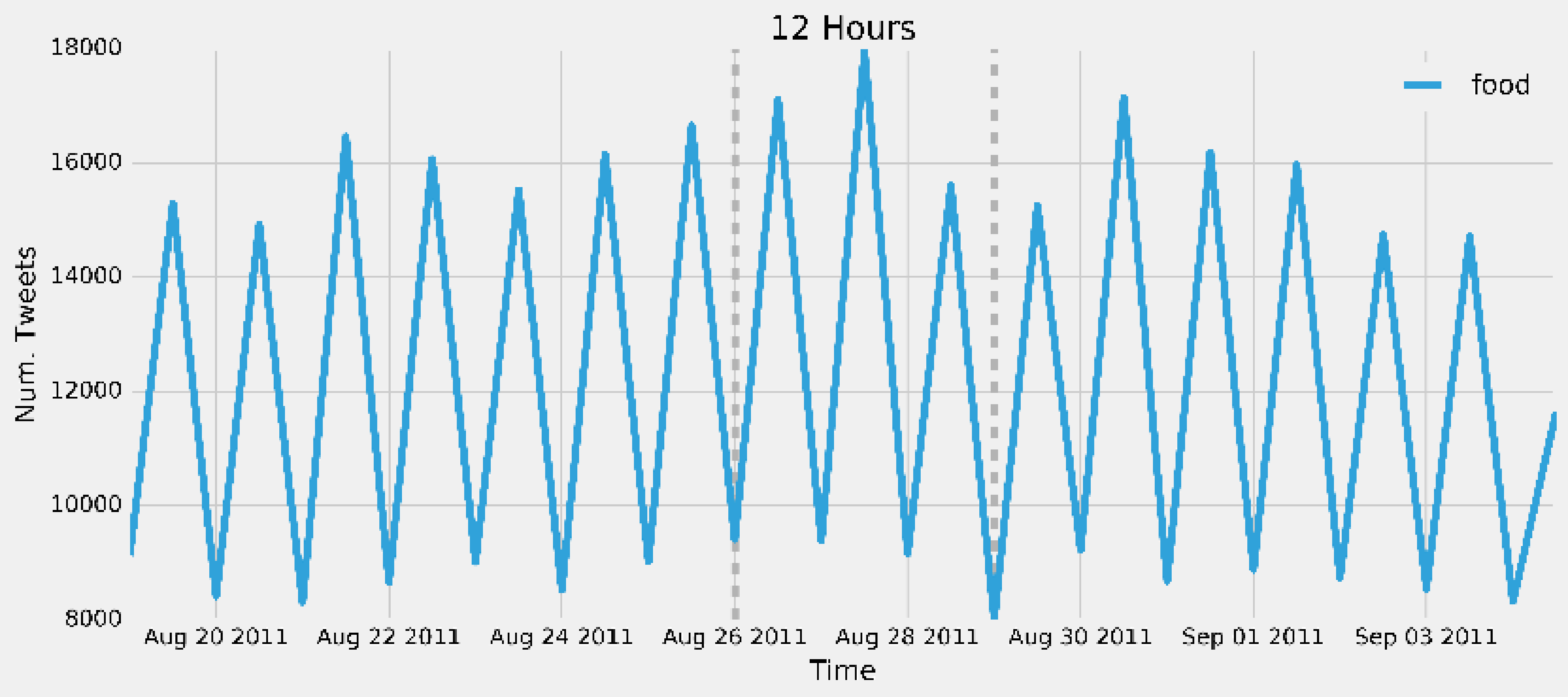
12 Hours
food
Num. Tweets
18000
16000
14000
12000
10000
8000
Aug 20 2011
Aug 22 2011
Aug 24 2011
Aug 26 2011
Aug 28 2011
Aug 30 2011
Sep 01 2011
Sep 03 2011
Time

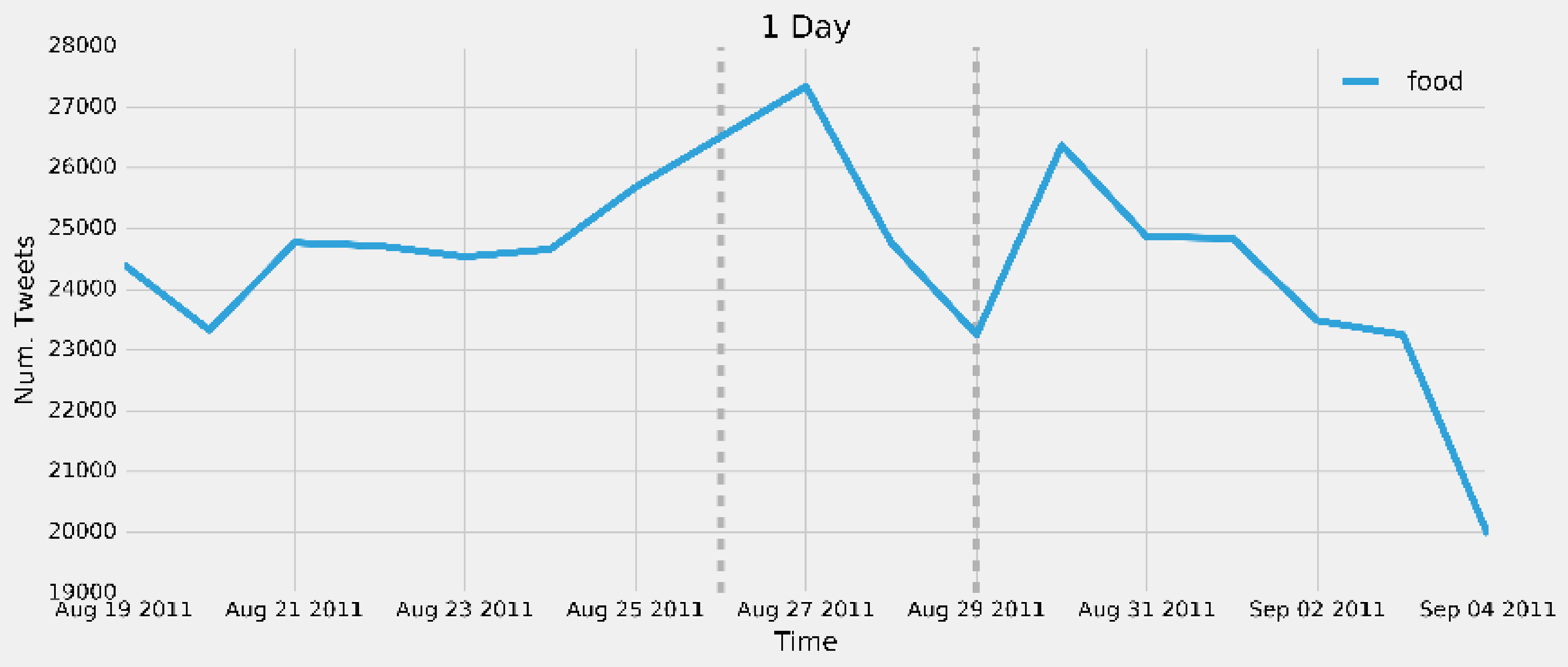
1 Day
food
28000
27000
26000
25000
24000
23000
22000
21000
20000
19000
Num. Tweets
Aug 19 2011
Aug 21 2011
Aug 23 2011
Aug 25 2011
Aug 27 2011
Aug 29 2011
Aug 31 2011
Sep 02 2011
Sep 04 2011
Time

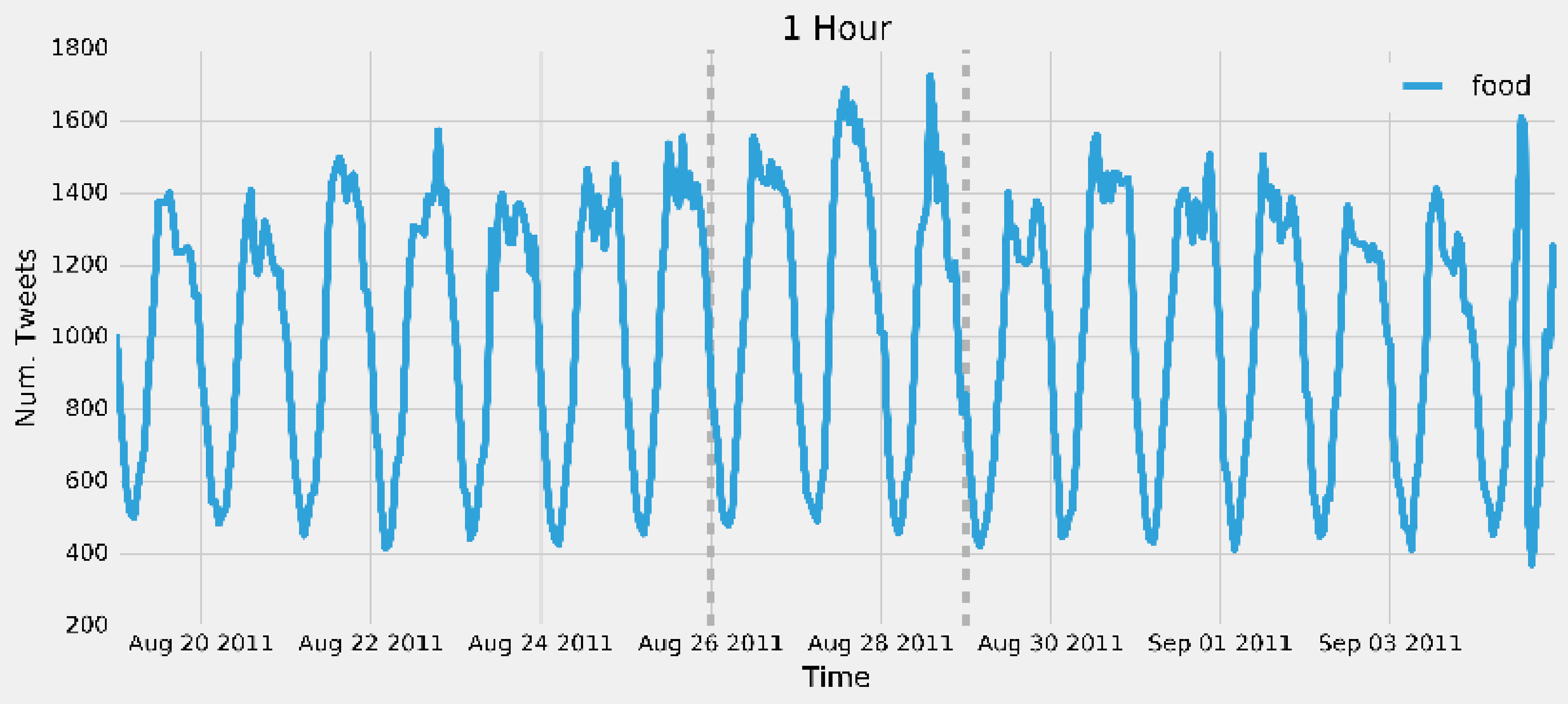

1 Hour
food
Num. Tweets
1800
1600
1400
1200
1000
800
600
400
200
Aug 20 2011
Aug 22 2011
Aug 24 2011
Aug 26 2011
Aug 28 2011
Aug 30 2011
Sep 01 2011
Sep 03 2011
Time

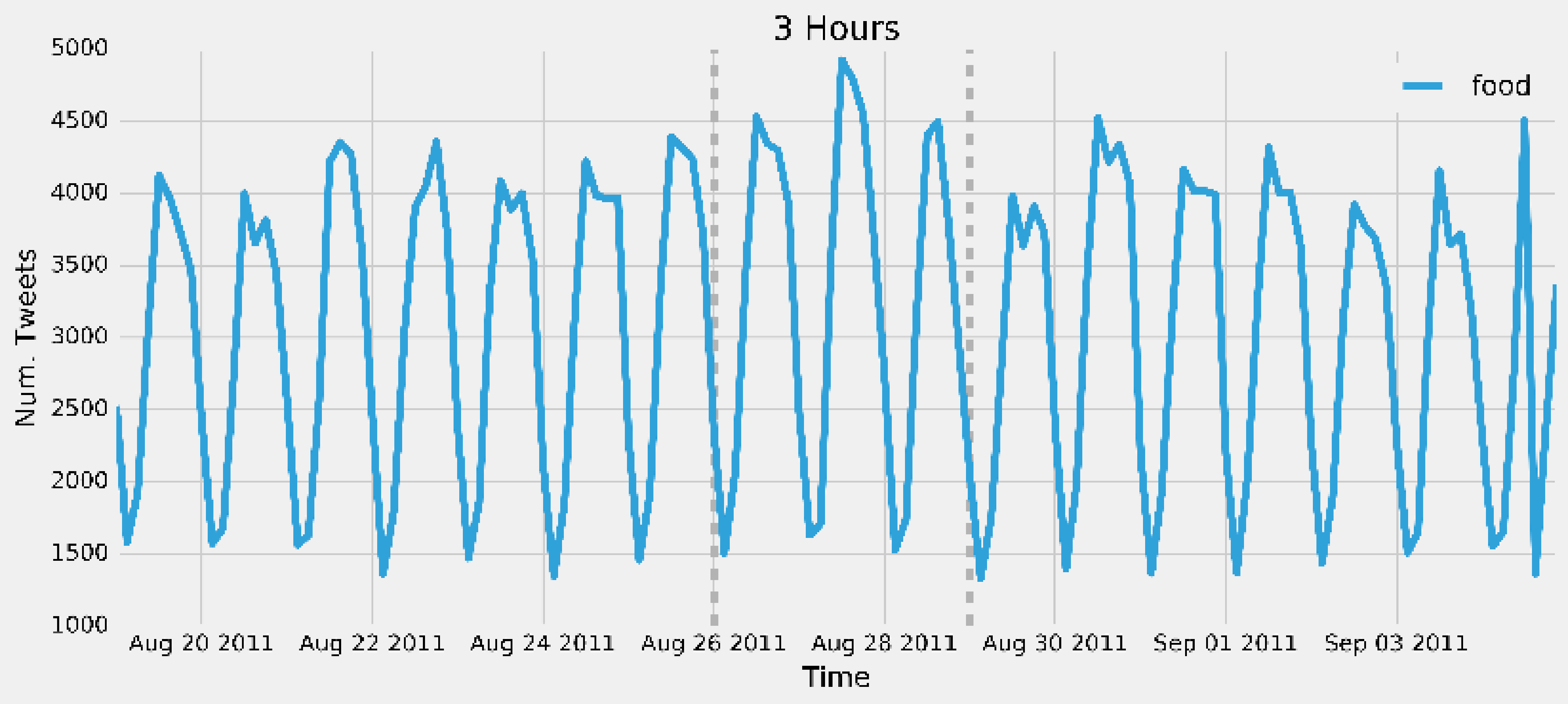
3 Hours
food
Num. Tweets
5000
4500
4000
3500
3000
2500
2000
1500
1000
Aug 20 2011
Aug 22 2011
Aug 24 2011
Aug 26 2011
Aug 28 2011
Aug 30 2011
Sep 01 2011
Sep 03 2011
Time

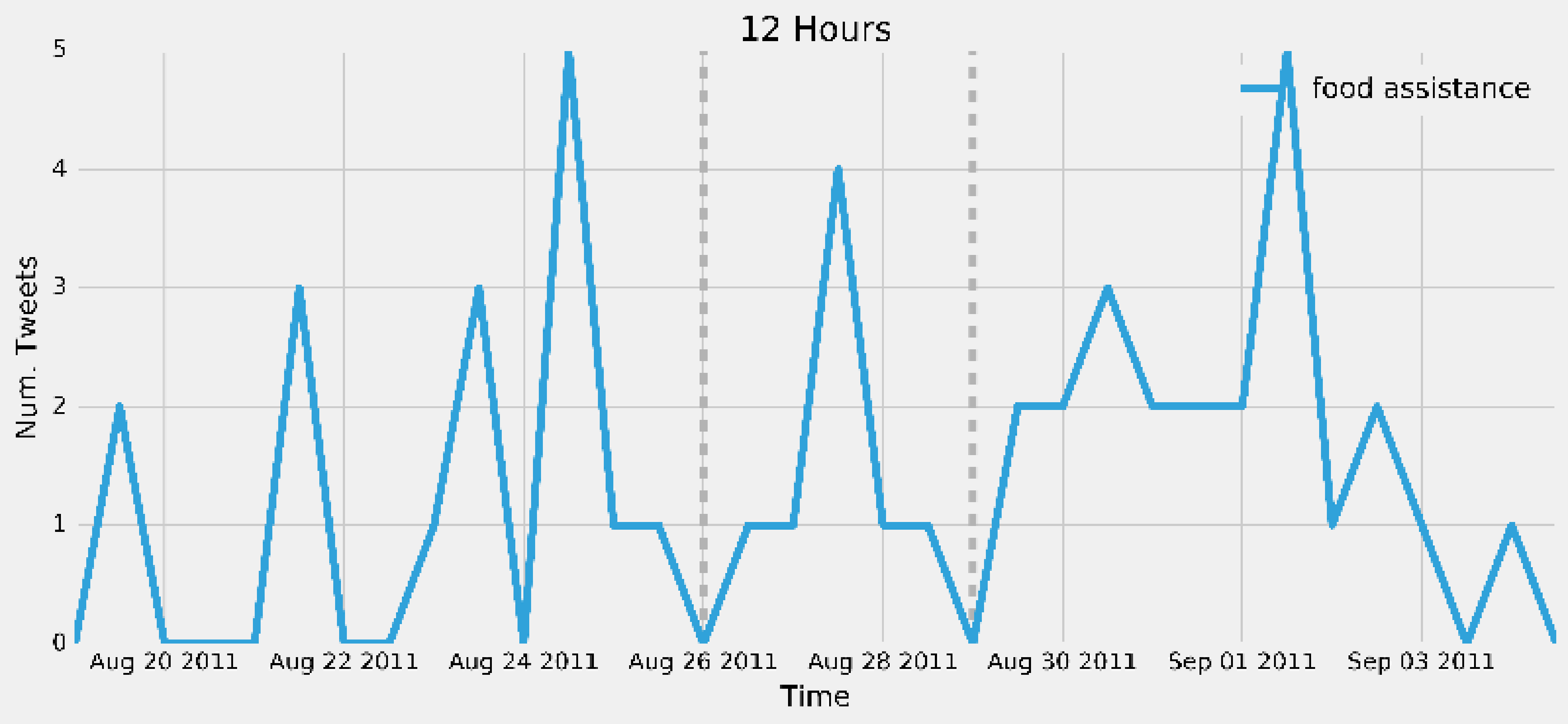

12 Hours
food assistance
Num. Tweets
Time
0
1
2
3
4
5
Aug 20 2011
Aug 22 2011
Aug 24 2011
Aug 26 2011
Aug 28 2011
Aug 30 2011
Sep 01 2011
Sep 03 2011

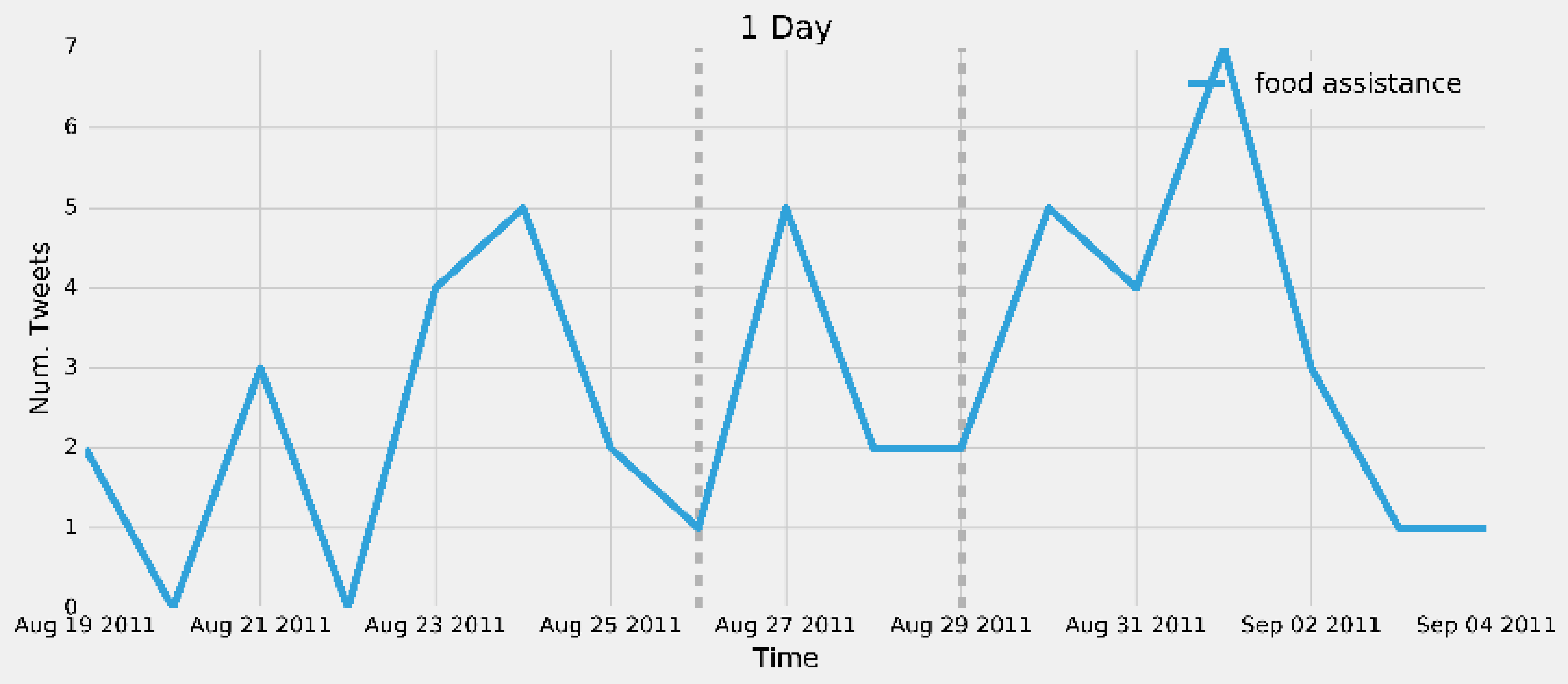
1 Day
food assistance
Num. Tweets
Time
0
1
2
3
4
5
6
7
Aug 19 2011
Aug 21 2011
Aug 23 2011
Aug 25 2011
Aug 27 2011
Aug 29 2011
Aug 31 2011
Sep 02 2011
Sep 04 2011

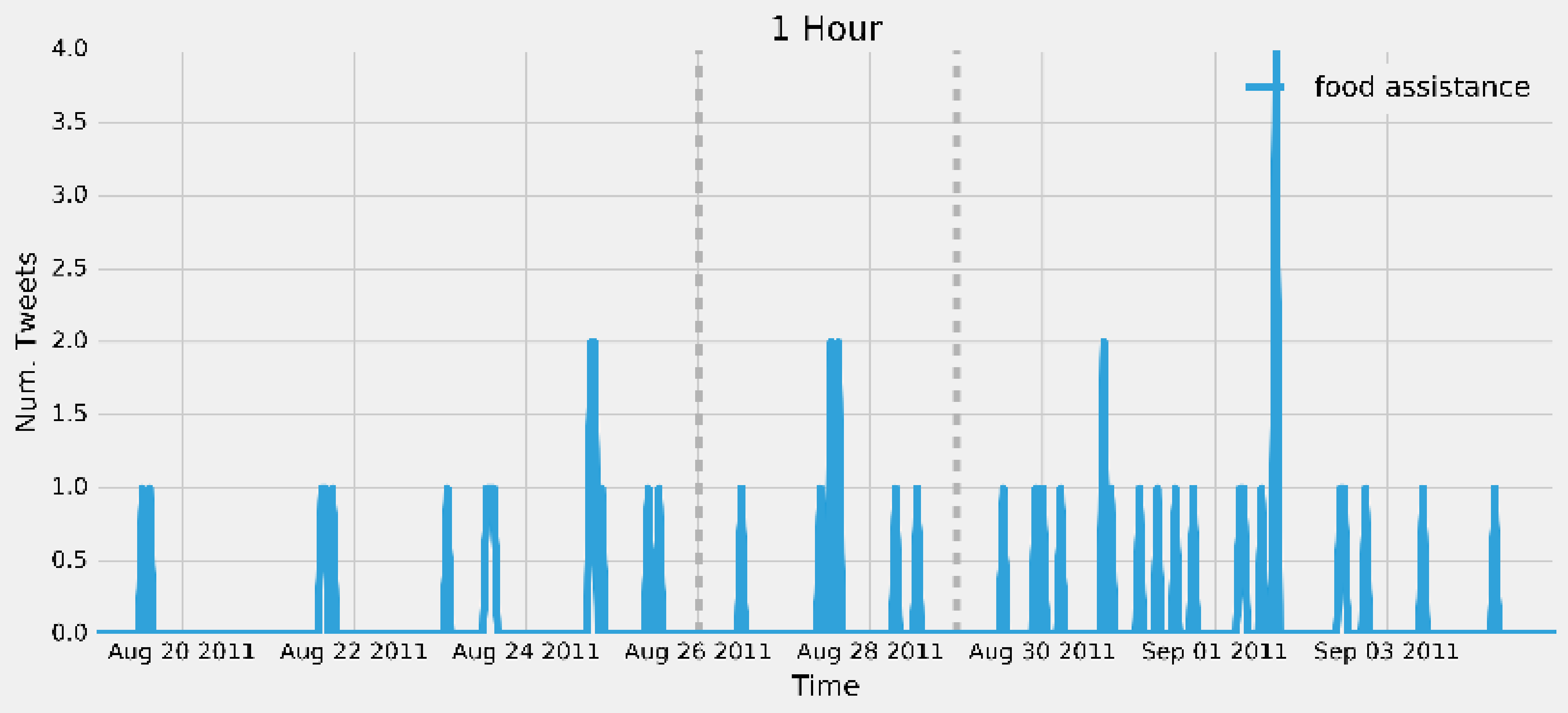
1 Hour
food assistance
Num. Tweets
0.0
0.5
1.0
1.5
2.0
2.5
3.0
3.5
4.0
Aug 20 2011
Aug 22 2011
Aug 24 2011
Aug 26 2011
Aug 28 2011
Aug 30 2011
Sep 01 2011
Sep 03 2011
Time

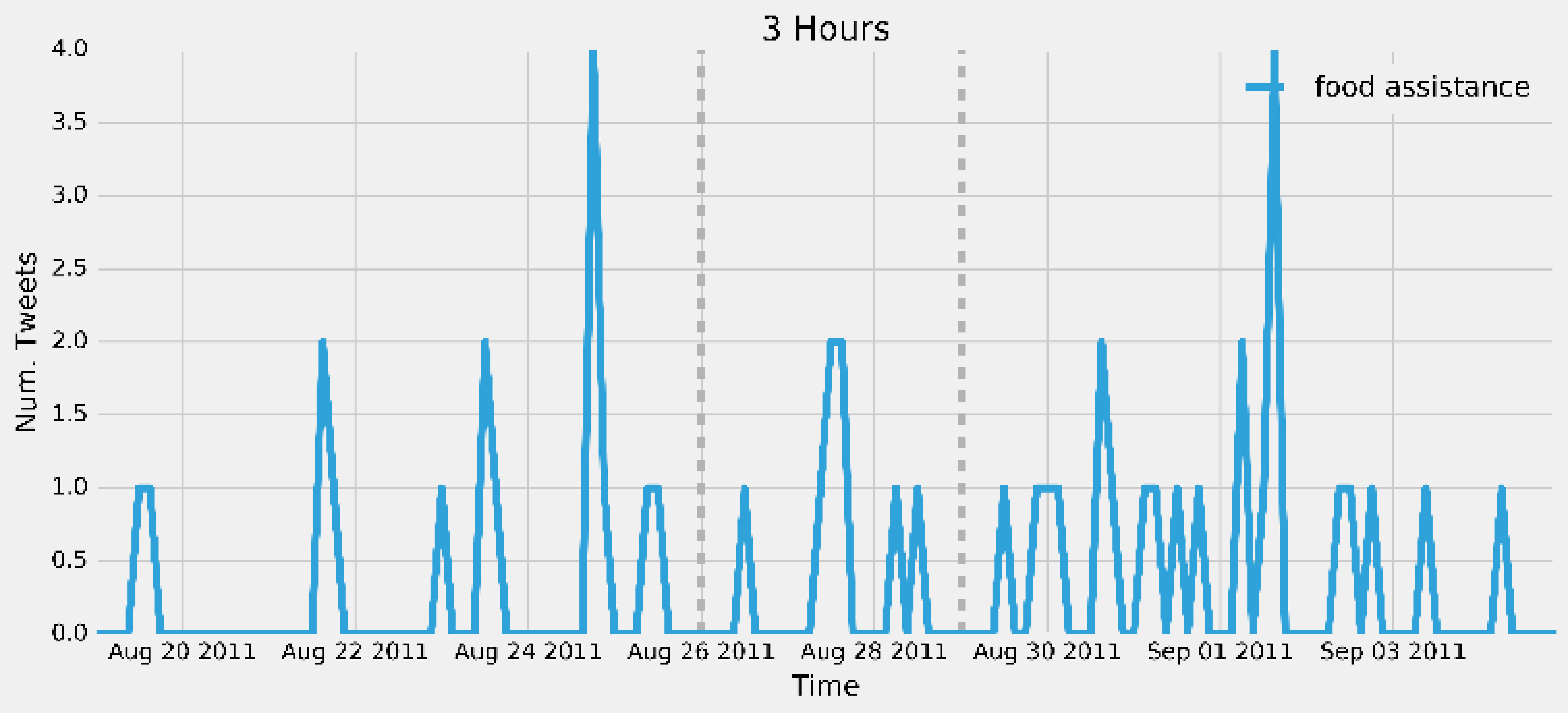
3 Hours
food assistance
Num. Tweets
4.0
3.5
3.0
2.5
2.0
1.5
1.0
0.5
0.0
Aug 20 2011
Aug 22 2011
Aug 24 2011
Aug 26 2011
Aug 28 2011
Aug 30 2011
Sep 01 2011
Sep 03 2011
Time

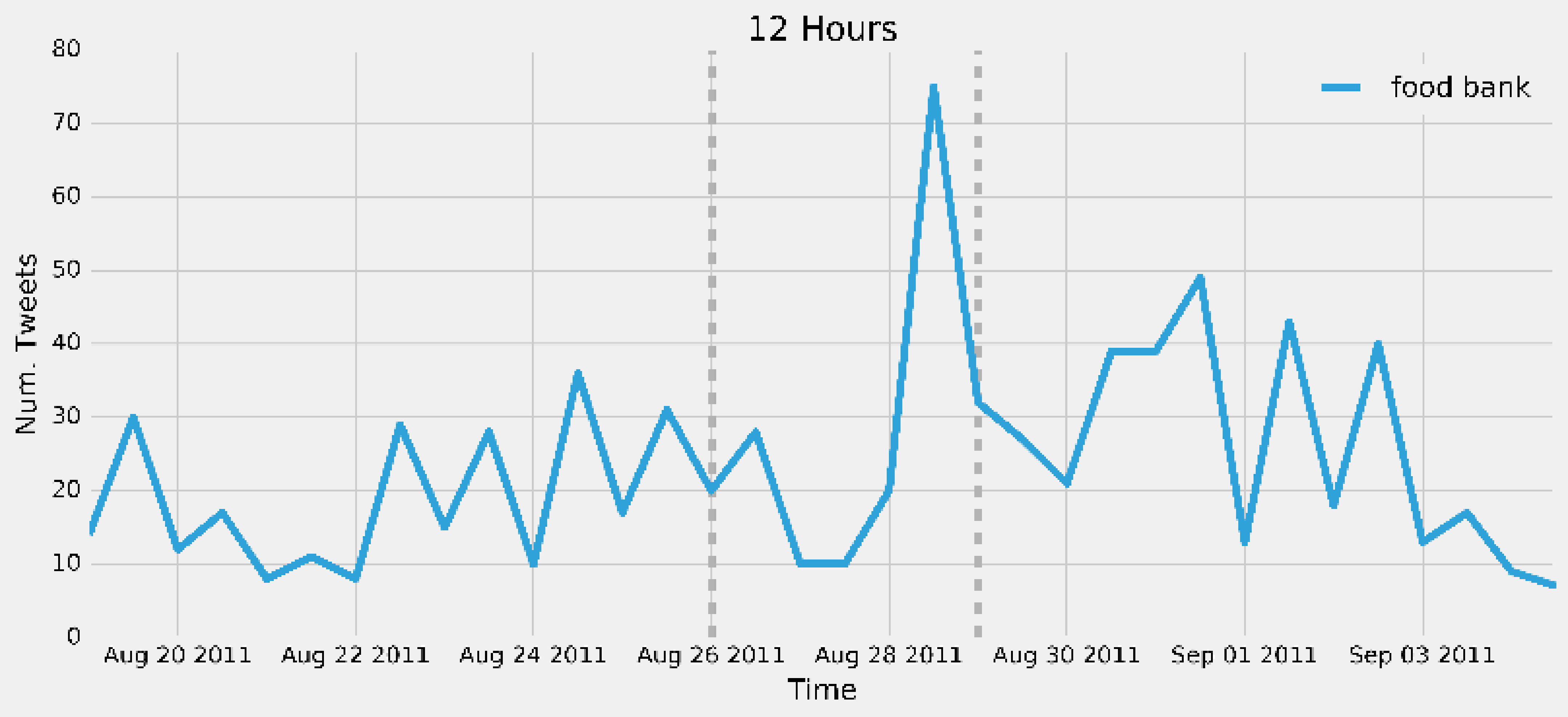

12 Hours
food bank
Num. Tweets
Time
0
10
20
30
40
50
60
70
80
Aug 20 2011
Aug 22 2011
Aug 24 2011
Aug 26 2011
Aug 28 2011
Aug 30 2011
Sep 01 2011
Sep 03 2011

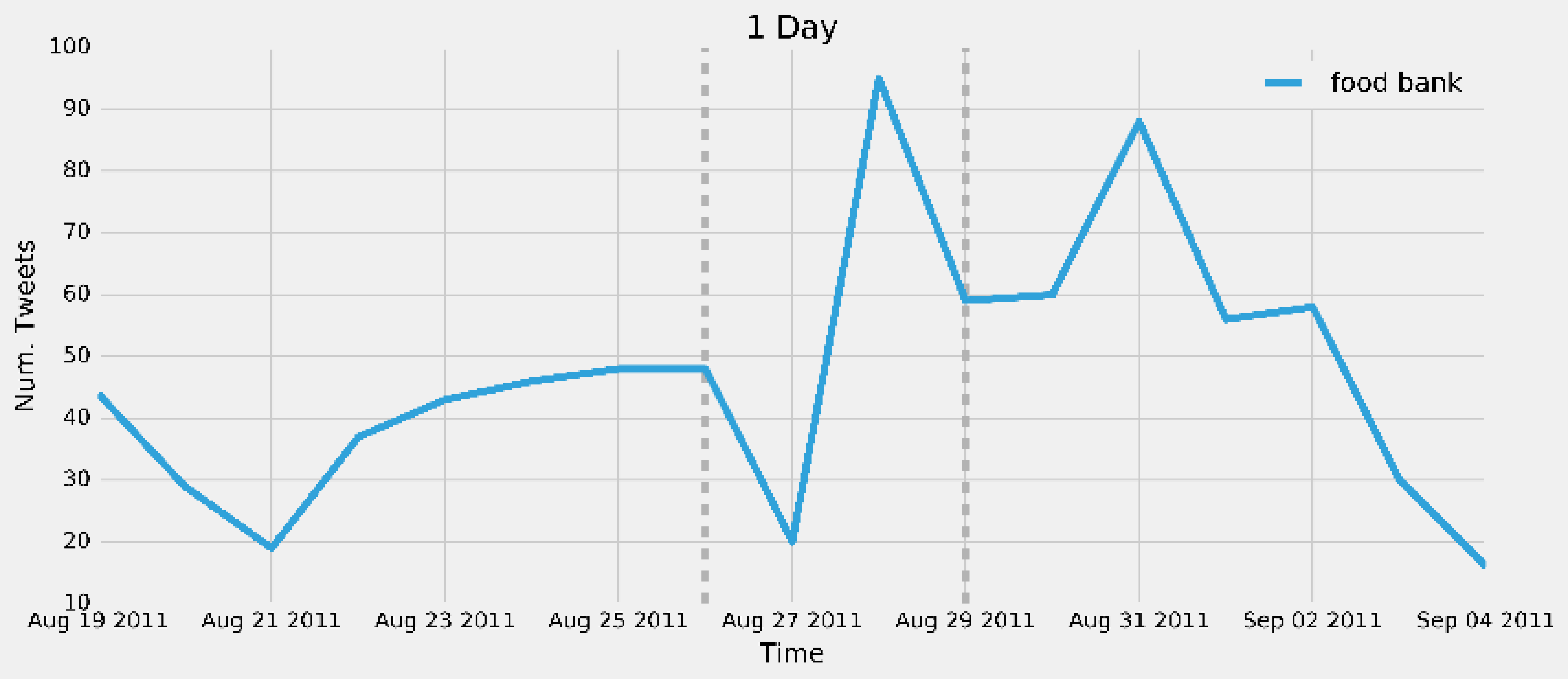

1 Day
food bank
Num. Tweets
100
90
80
70
60
50
40
30
20
10
Aug 19 2011
Aug 21 2011
Aug 23 2011
Aug 25 2011
Aug 27 2011
Aug 29 2011
Aug 31 2011
Sep 02 2011
Sep 04 2011
Time

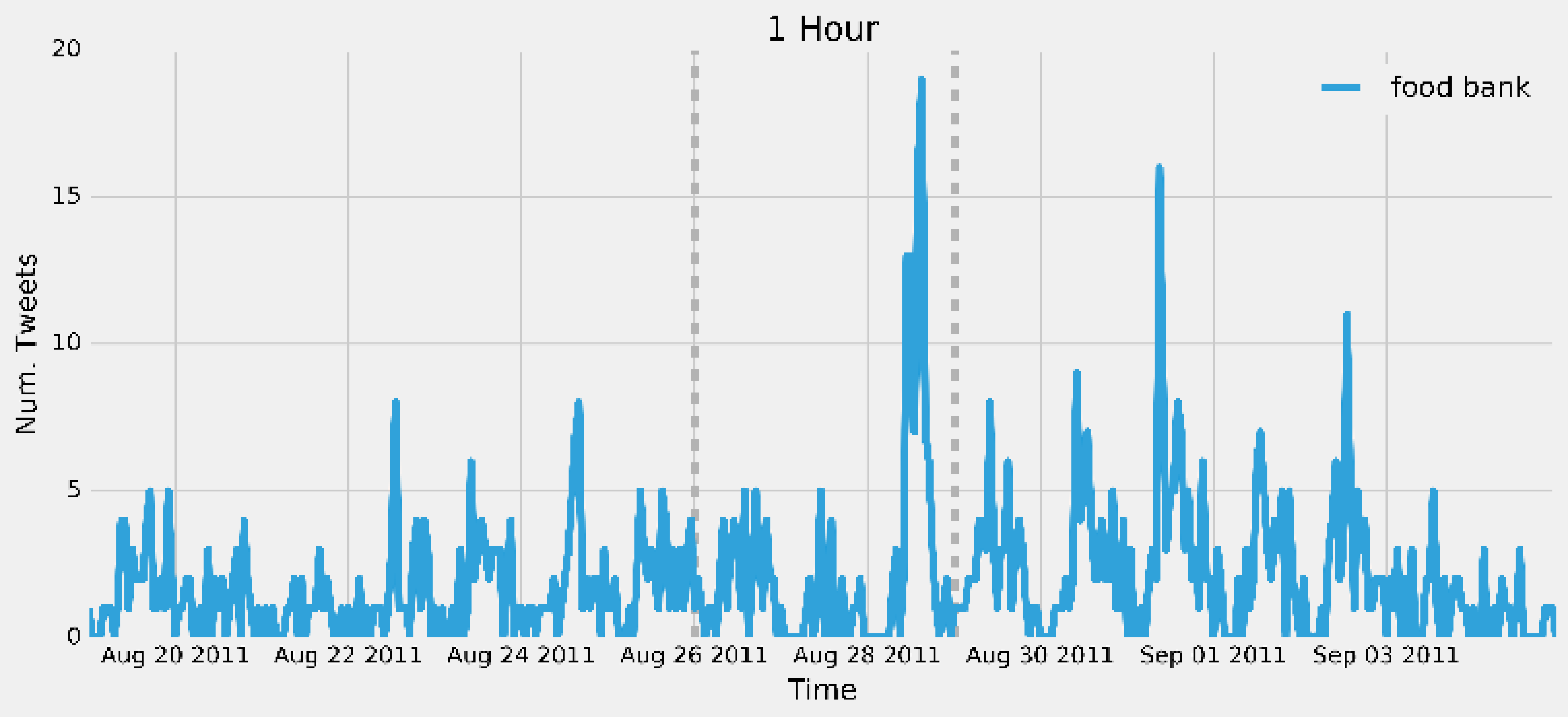
1 Hour
food bank
Num. Tweets
0
5
10
15
20
Aug 20 2011
Aug 22 2011
Aug 24 2011
Aug 26 2011
Aug 28 2011
Aug 30 2011
Sep 01 2011
Sep 03 2011
Time

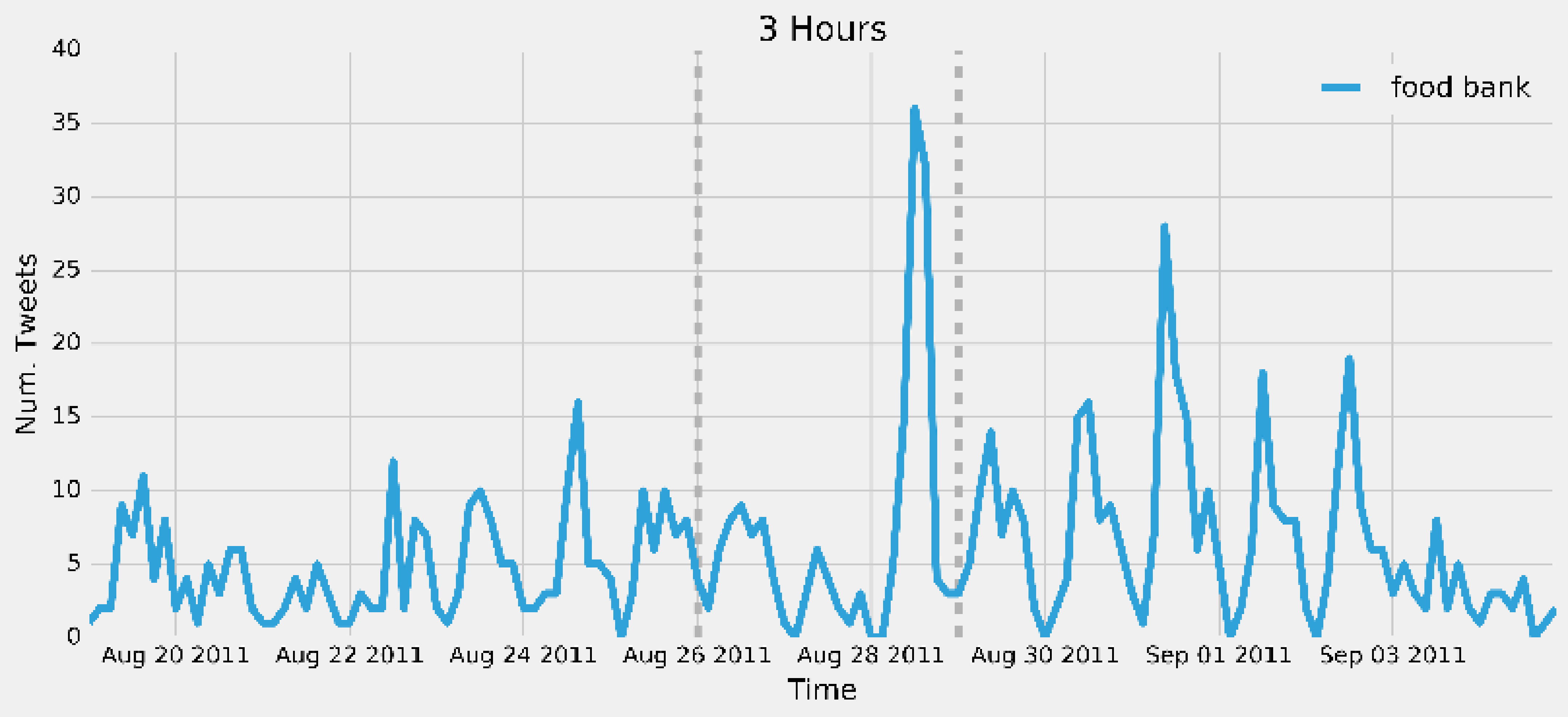

3 Hours
food bank
Num. Tweets
Time
0
5
10
15
20
25
30
35
40
Aug 20 2011
Aug 22 2011
Aug 24 2011
Aug 26 2011
Aug 28 2011
Aug 30 2011
Sep 01 2011
Sep 03 2011

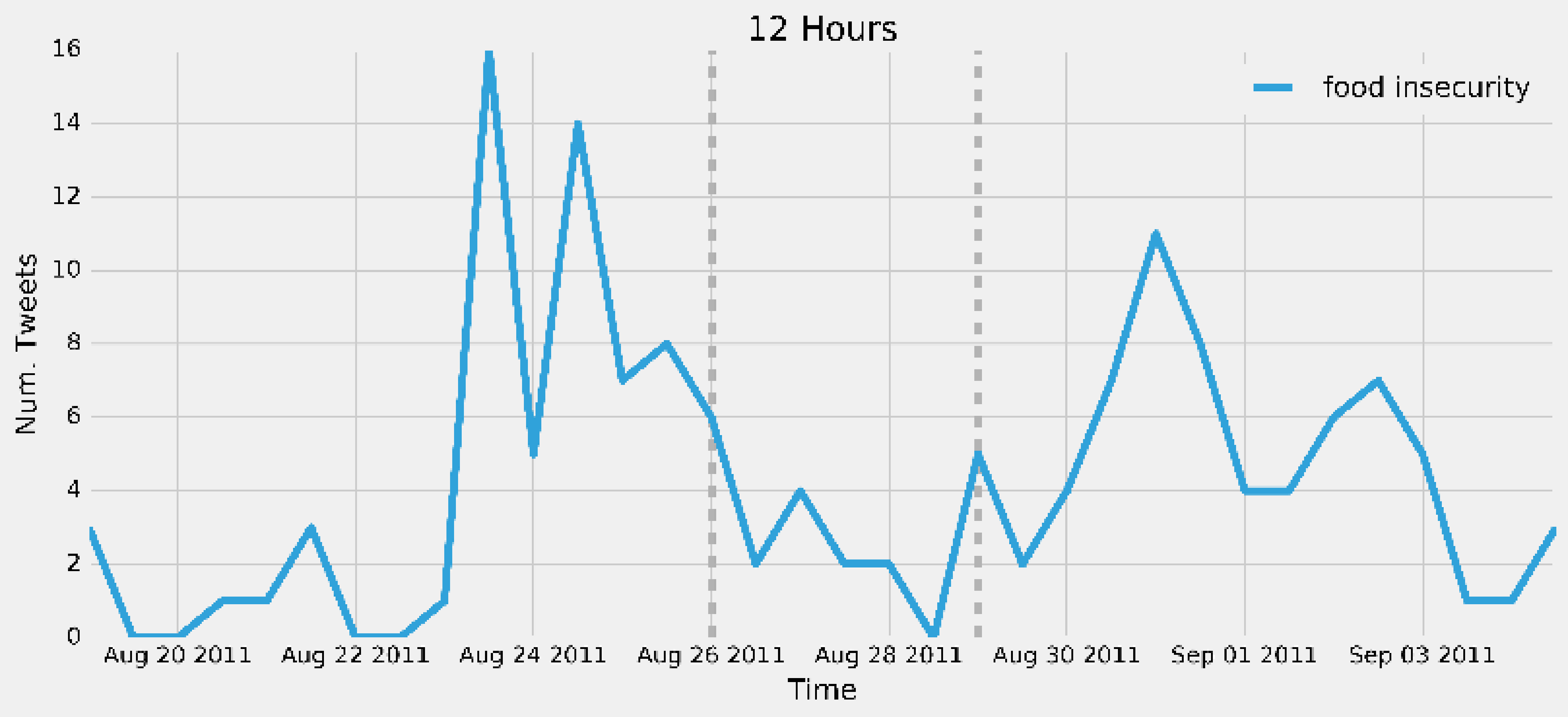
12 Hours
food insecurity
Num. Tweets
0
2
4
6
8
10
12
14
16
Aug 20 2011
Aug 22 2011
Aug 24 2011
Aug 26 2011
Aug 28 2011
Aug 30 2011
Sep 01 2011
Sep 03 2011
Time

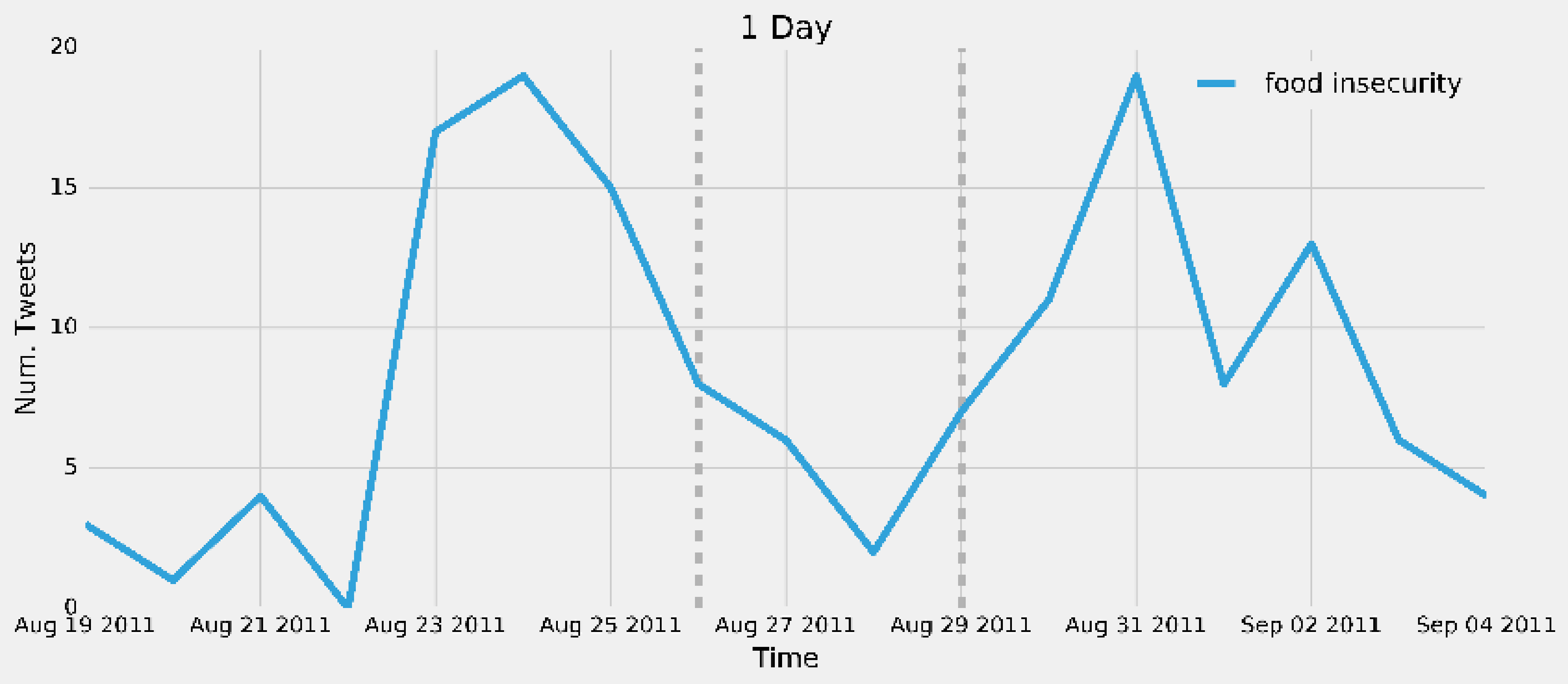
1 Day
food insecurity
Num. Tweets
20
15
10
5
0
Aug 19 2011
Aug 21 2011
Aug 23 2011
Aug 25 2011
Aug 27 2011
Aug 29 2011
Aug 31 2011
Sep 02 2011
Sep 04 2011
Time

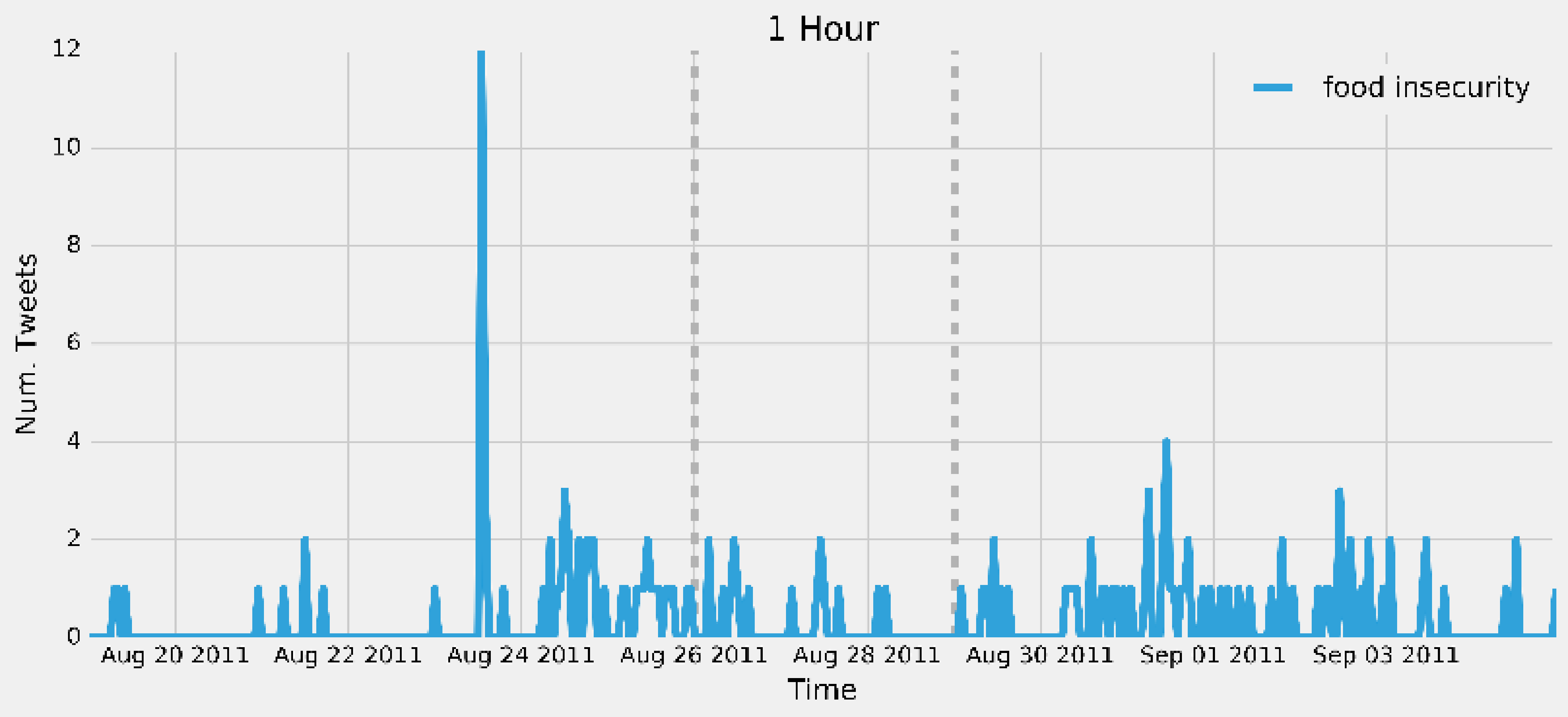
1 Hour
food insecurity
Num. Tweets
Time
0
2
4
6
8
10
12
Aug 20 2011
Aug 22 2011
Aug 24 2011
Aug 26 2011
Aug 28 2011
Aug 30 2011
Sep 01 2011
Sep 03 2011

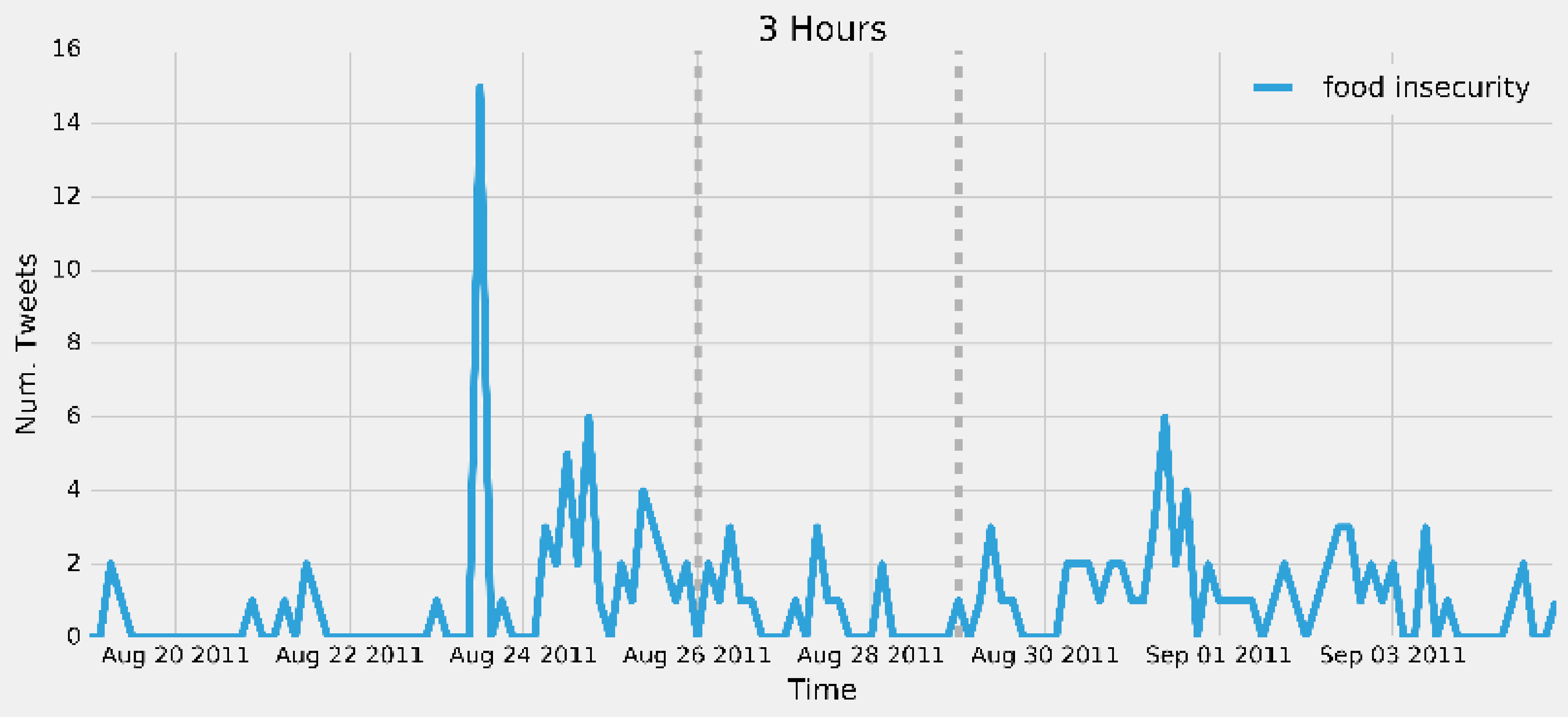

3 Hours
food insecurity
Num. Tweets
0
2
4
6
8
10
12
14
16
Aug 20 2011
Aug 22 2011
Aug 24 2011
Aug 26 2011
Aug 28 2011
Aug 30 2011
Sep 01 2011
Sep 03 2011
Time

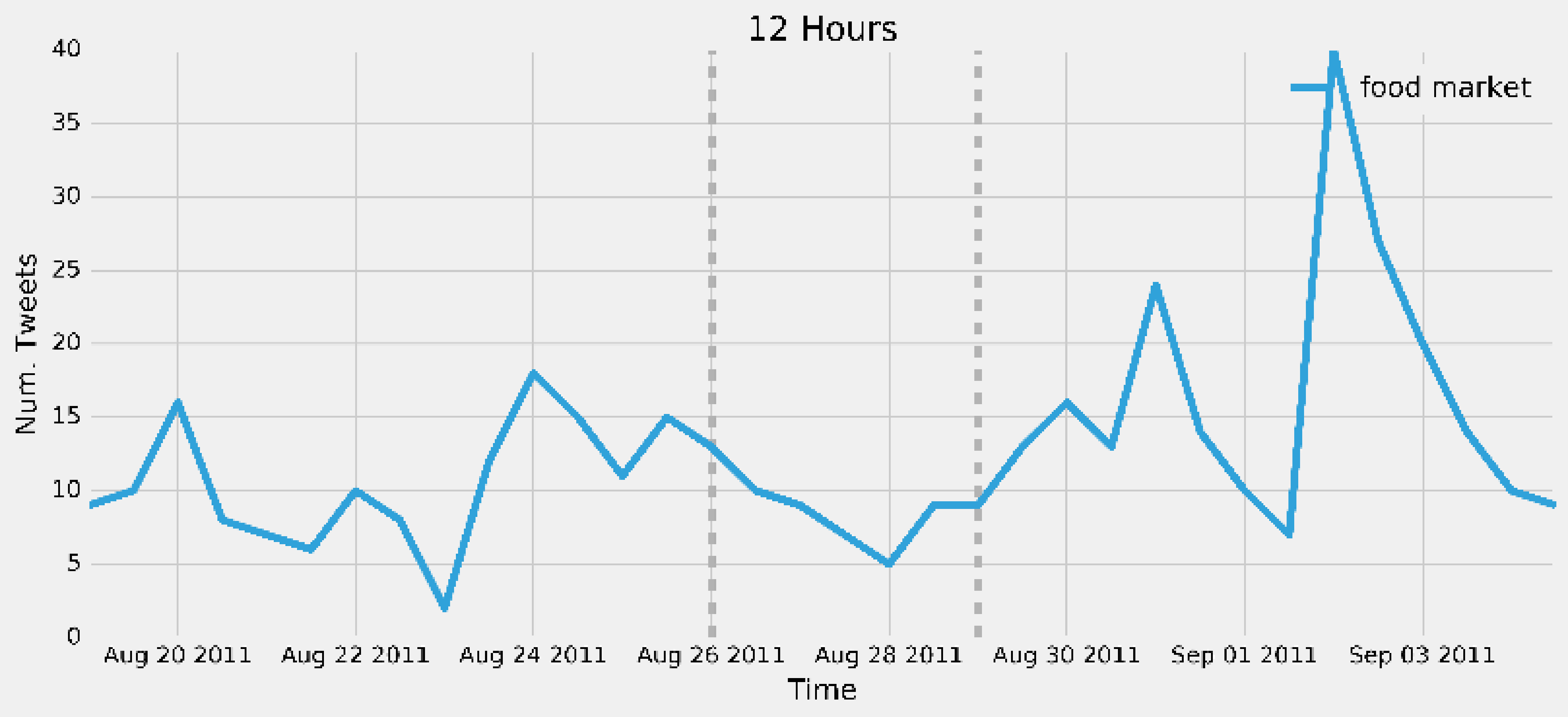
12 Hours
food market
Num. Tweets
Time
0
5
10
15
20
25
30
35
40
Aug 20 2011
Aug 22 2011
Aug 24 2011
Aug 26 2011
Aug 28 2011
Aug 30 2011
Sep 01 2011
Sep 03 2011

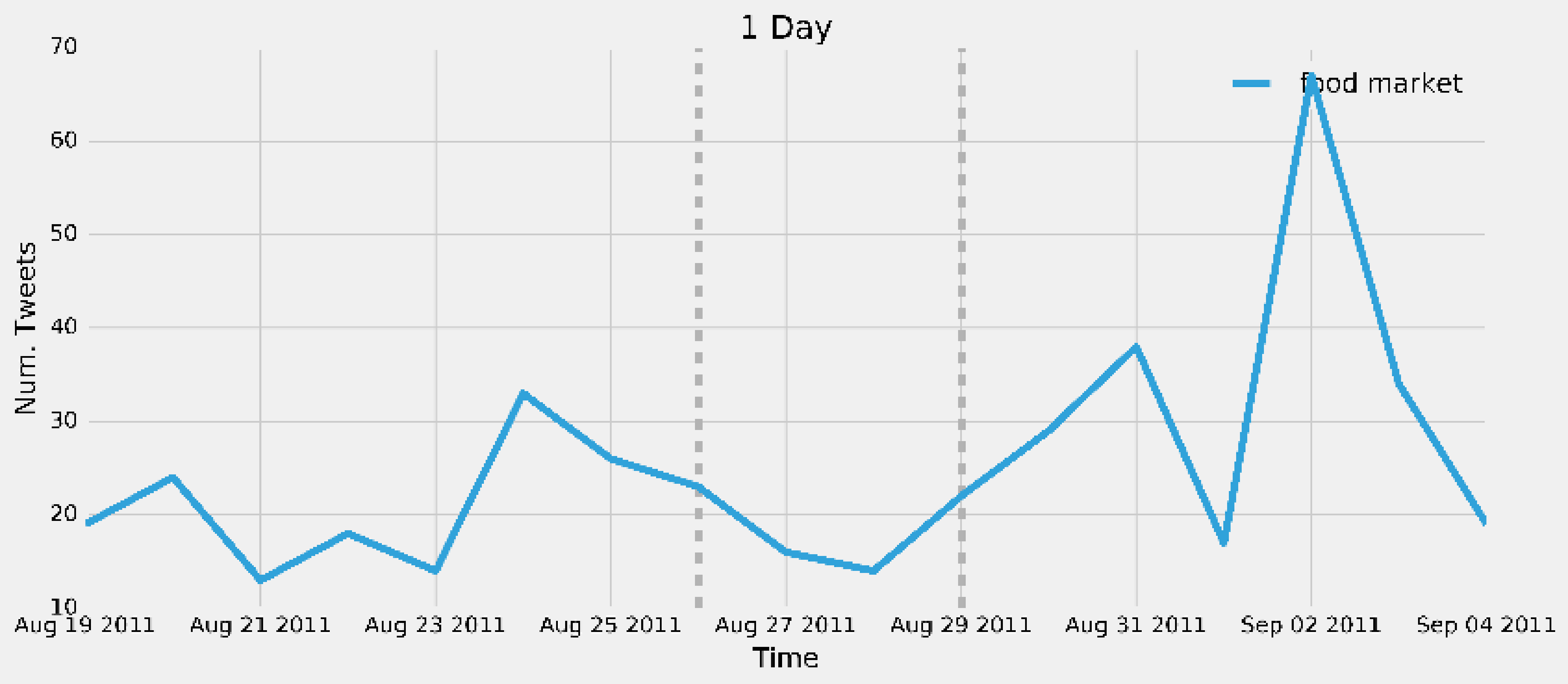

1 Day
food market
Num. Tweets
70
60
50
40
30
20
10
Aug 19 2011
Aug 21 2011
Aug 23 2011
Aug 25 2011
Aug 27 2011
Aug 29 2011
Aug 31 2011
Sep 02 2011
Sep 04 2011
Time

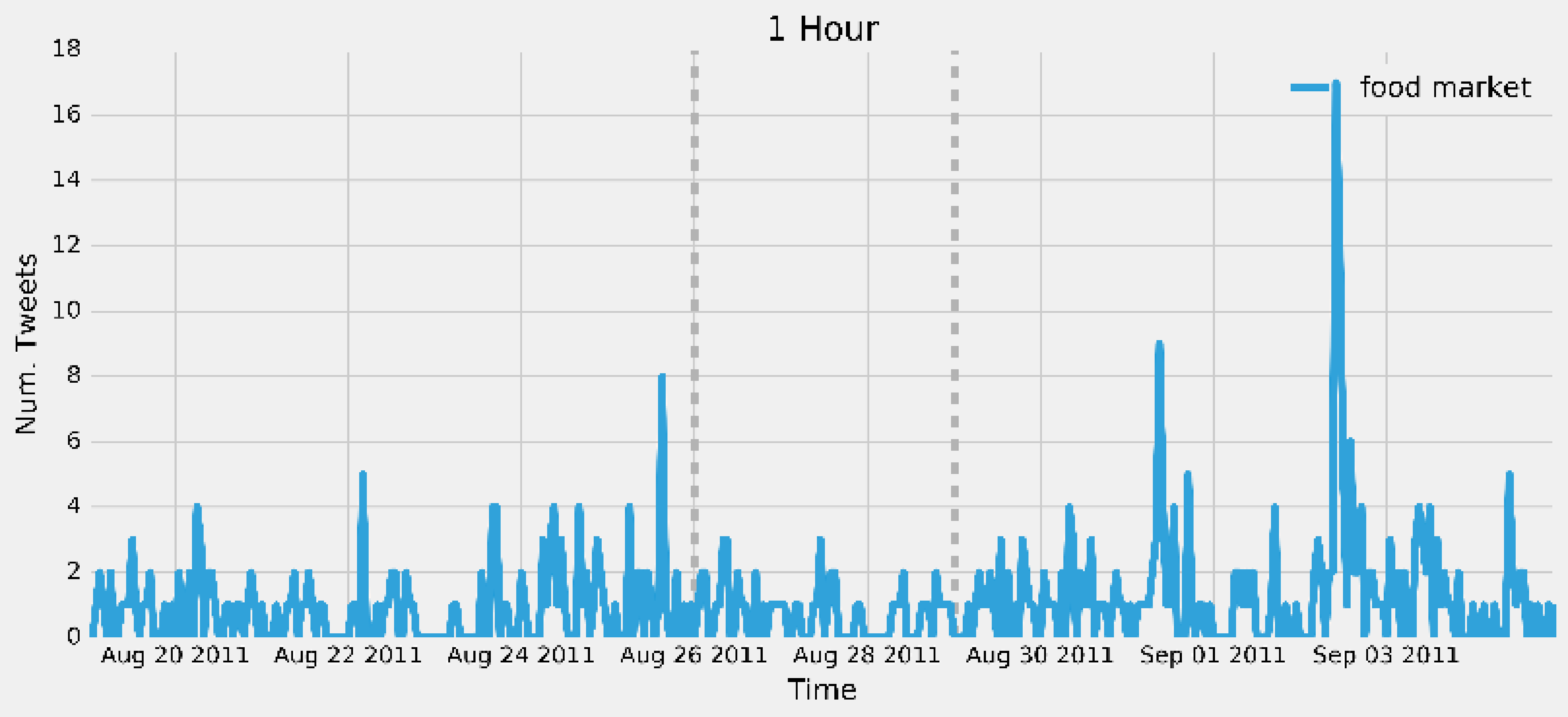

1 Hour
food market
Num. Tweets
Time
0
2
4
6
8
10
12
14
16
18
Aug 20 2011
Aug 22 2011
Aug 24 2011
Aug 26 2011
Aug 28 2011
Aug 30 2011
Sep 01 2011
Sep 03 2011

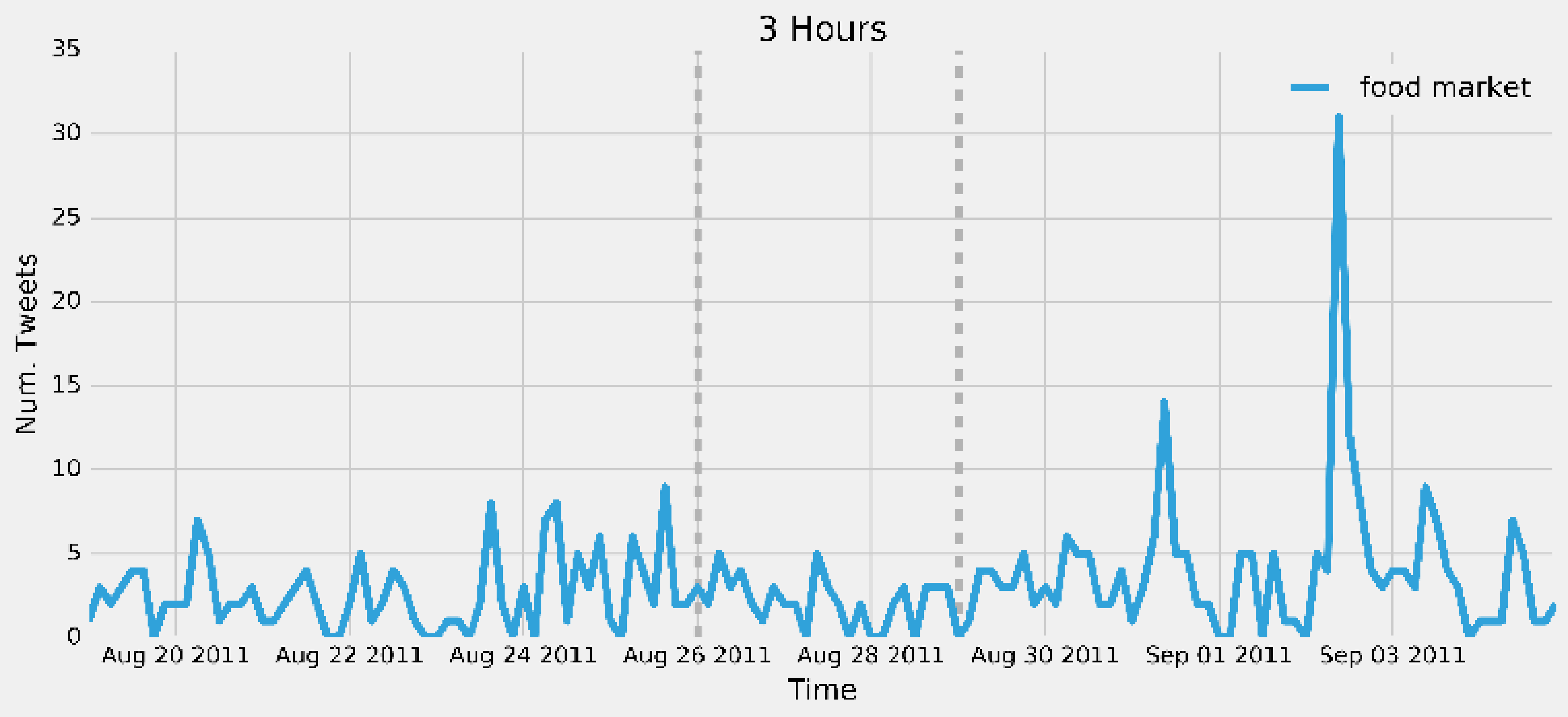

3 Hours
food market
Num. Tweets
0
5
10
15
20
25
30
35
Aug 20 2011
Aug 22 2011
Aug 24 2011
Aug 26 2011
Aug 28 2011
Aug 30 2011
Sep 01 2011
Sep 03 2011
Time

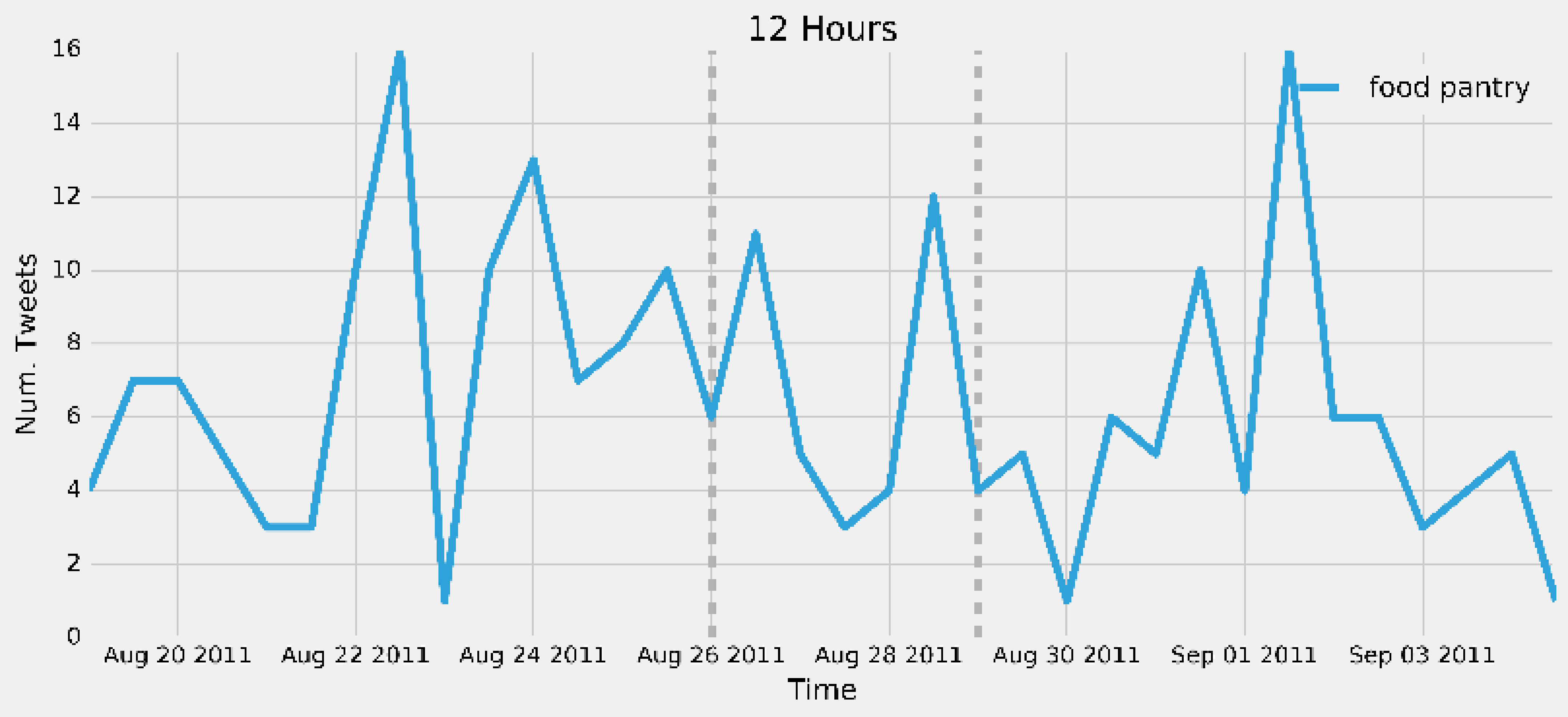
12 Hours
food pantry
Num. Tweets
Time
0
2
4
6
8
10
12
14
16
Aug 20 2011
Aug 22 2011
Aug 24 2011
Aug 26 2011
Aug 28 2011
Aug 30 2011
Sep 01 2011
Sep 03 2011

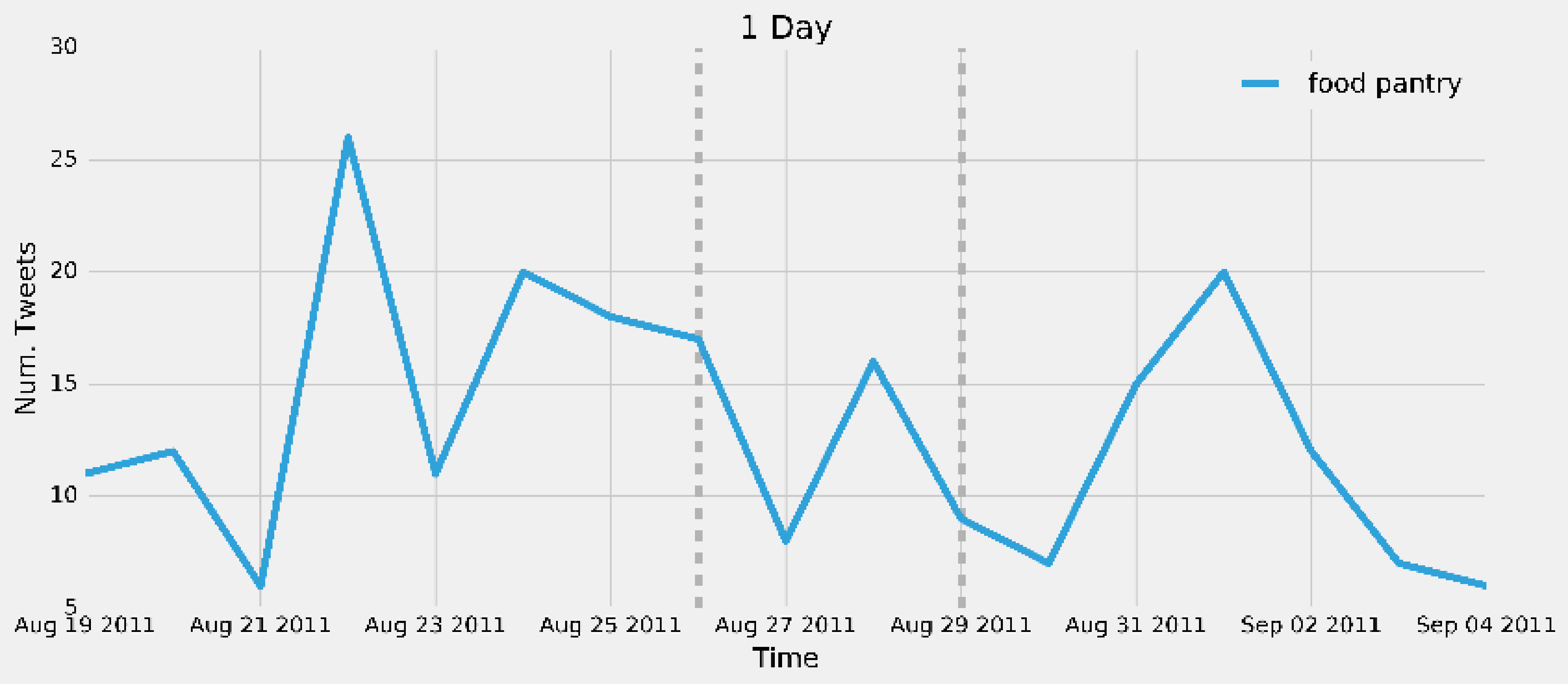
1 Day
food pantry
Num. Tweets
30
25
20
15
10
5
Aug 19 2011
Aug 21 2011
Aug 23 2011
Aug 25 2011
Aug 27 2011
Aug 29 2011
Aug 31 2011
Sep 02 2011
Sep 04 2011
Time

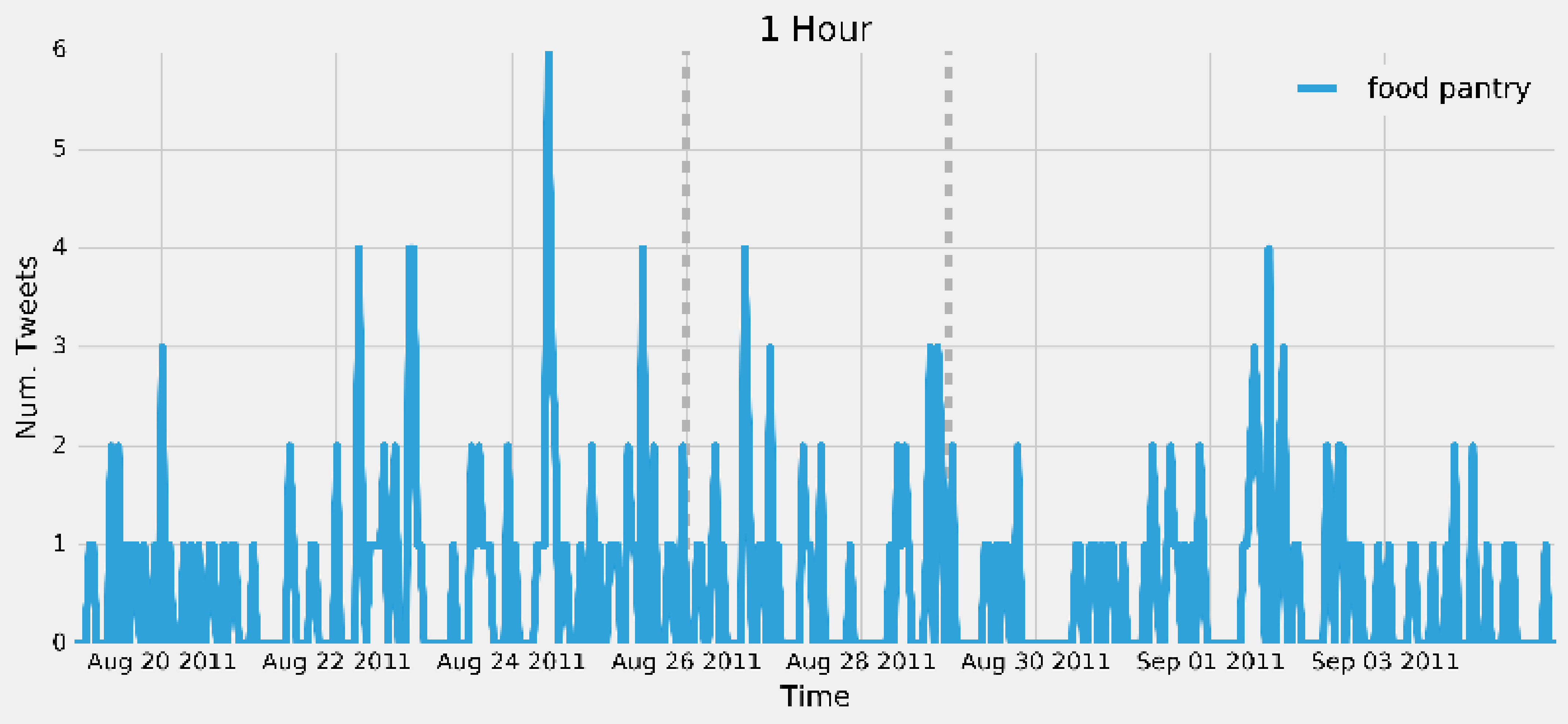
1 Hour
food pantry
Num. Tweets
0
1
2
3
4
5
6
Aug 20 2011
Aug 22 2011
Aug 24 2011
Aug 26 2011
Aug 28 2011
Aug 30 2011
Sep 01 2011
Sep 03 2011
Time

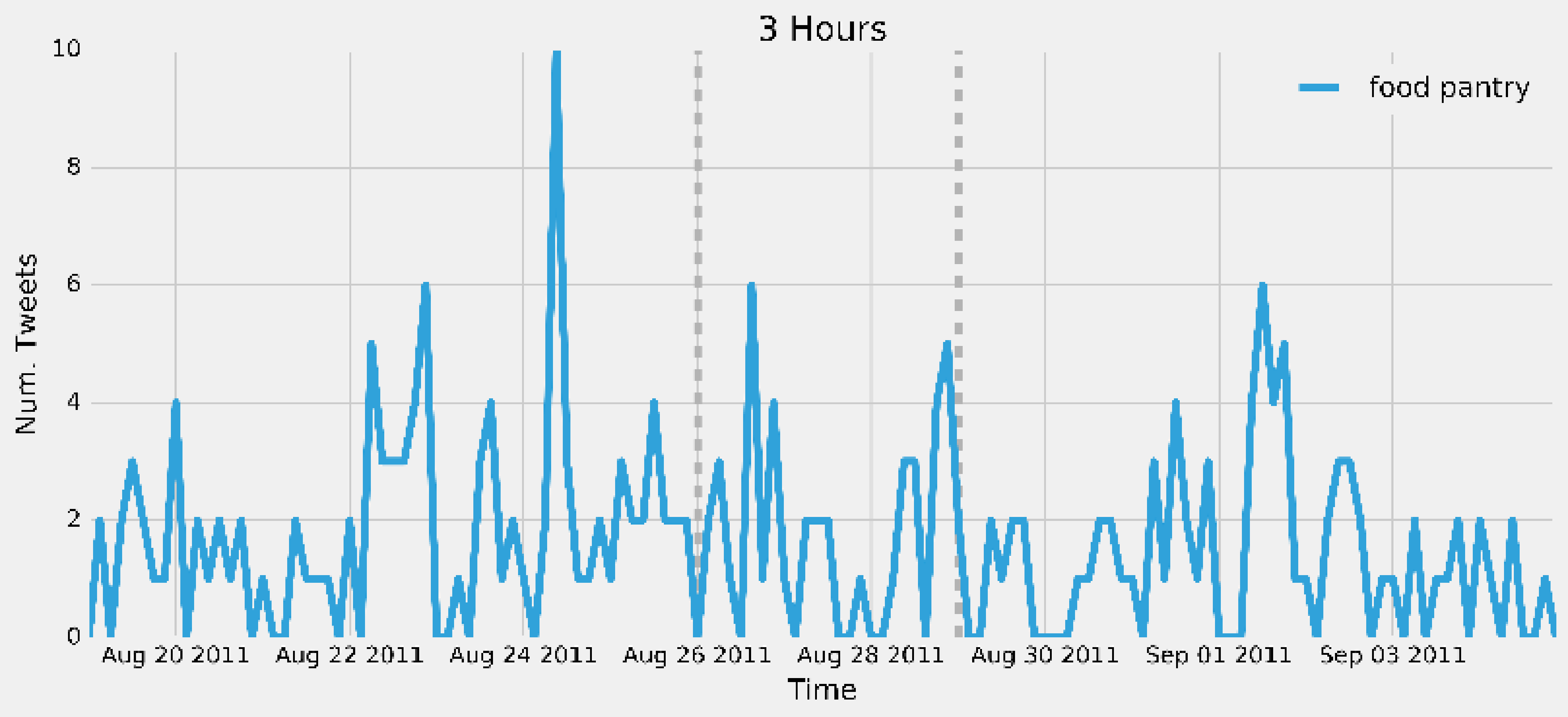
3 Hours
food pantry
Num. Tweets
0
2
4
6
8
10
Aug 20 2011
Aug 22 2011
Aug 24 2011
Aug 26 2011
Aug 28 2011
Aug 30 2011
Sep 01 2011
Sep 03 2011
Time

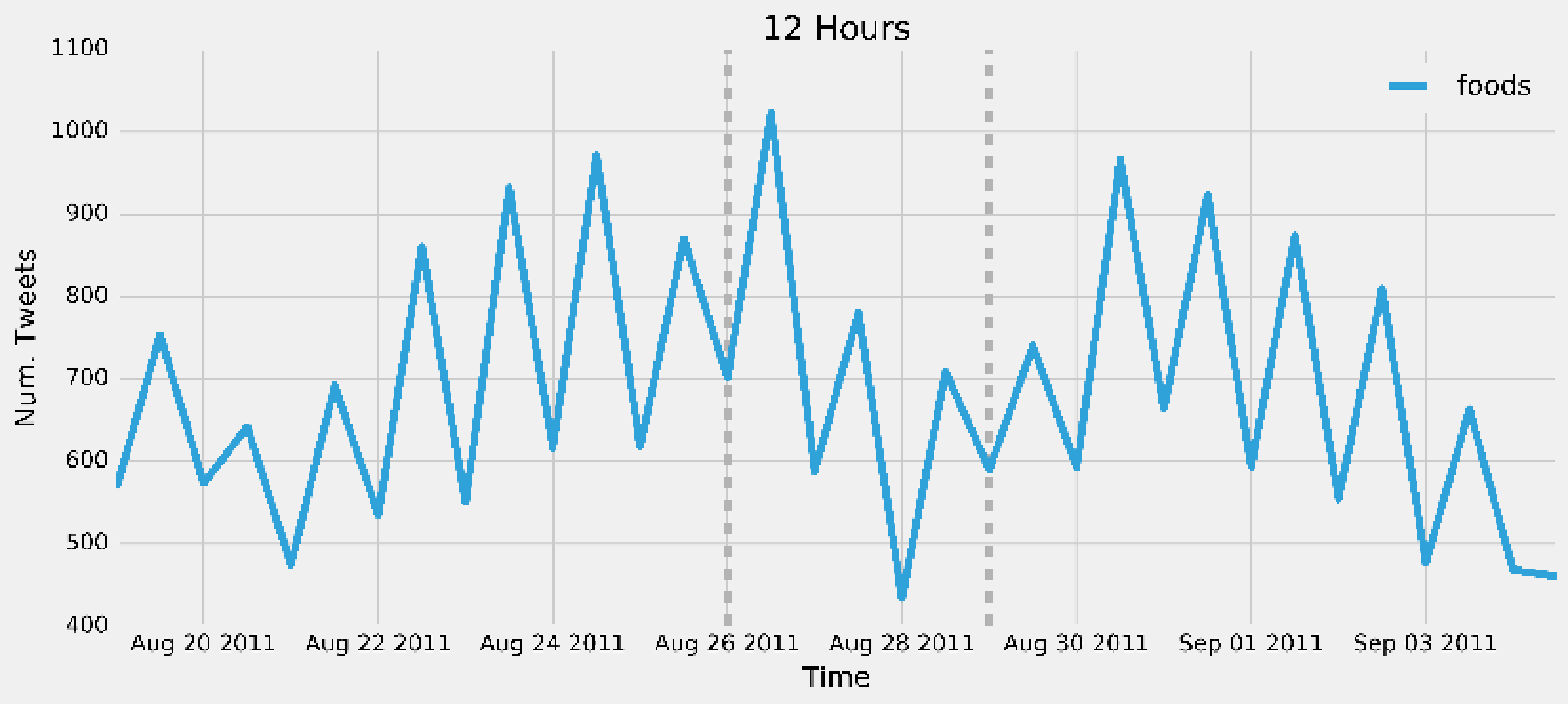

12 Hours
foods
1100
1000
900
800
700
600
500
400
Num. Tweets
Aug 20 2011
Aug 22 2011
Aug 24 2011
Aug 26 2011
Aug 28 2011
Aug 30 2011
Sep 01 2011
Sep 03 2011
Time

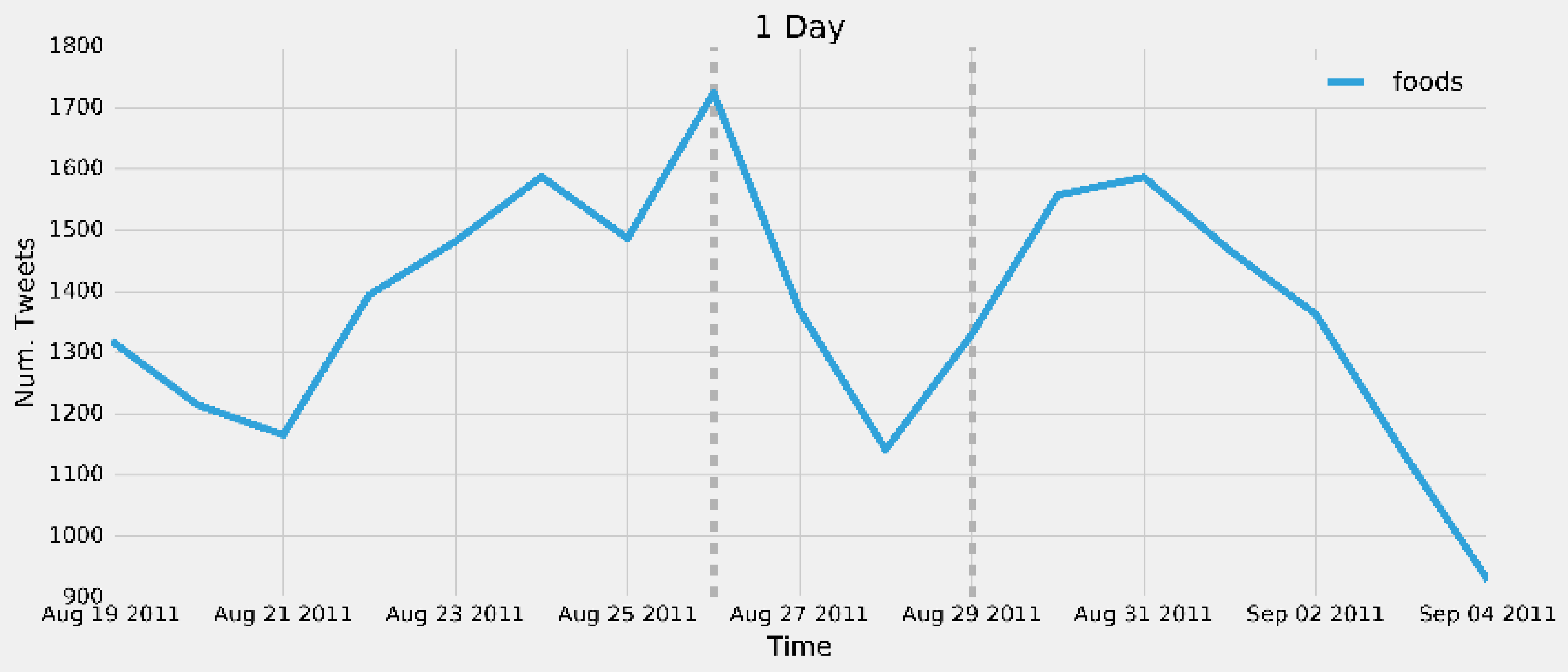
1 Day
foods
Num. Tweets
1800
1700
1600
1500
1400
1300
1200
1100
1000
900
Aug 19 2011
Aug 21 2011
Aug 23 2011
Aug 25 2011
Aug 27 2011
Aug 29 2011
Aug 31 2011
Sep 02 2011
Sep 04 2011
Time

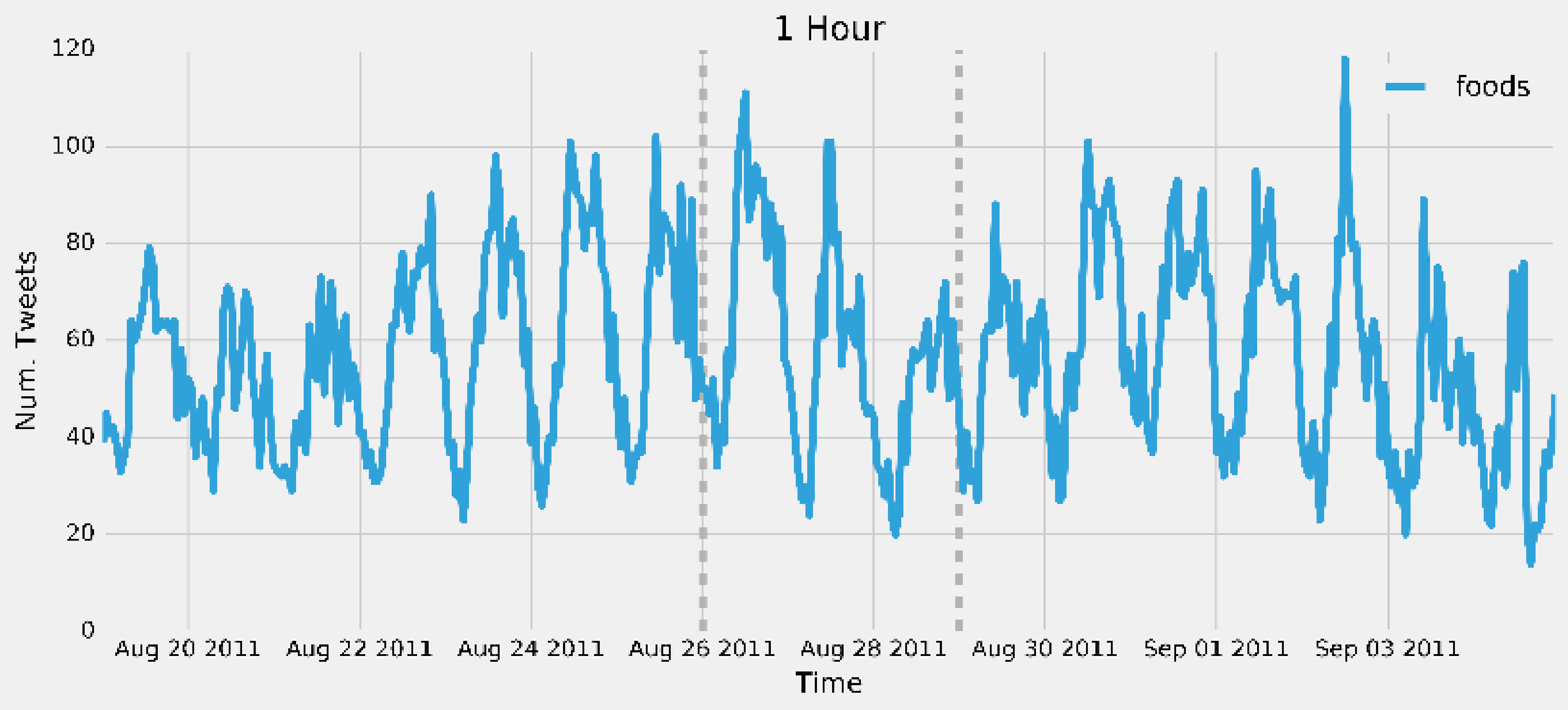
1 Hour
foods
Num. Tweets
0
20
40
60
80
100
120
Aug 20 2011
Aug 22 2011
Aug 24 2011
Aug 26 2011
Aug 28 2011
Aug 30 2011
Sep 01 2011
Sep 03 2011
Time

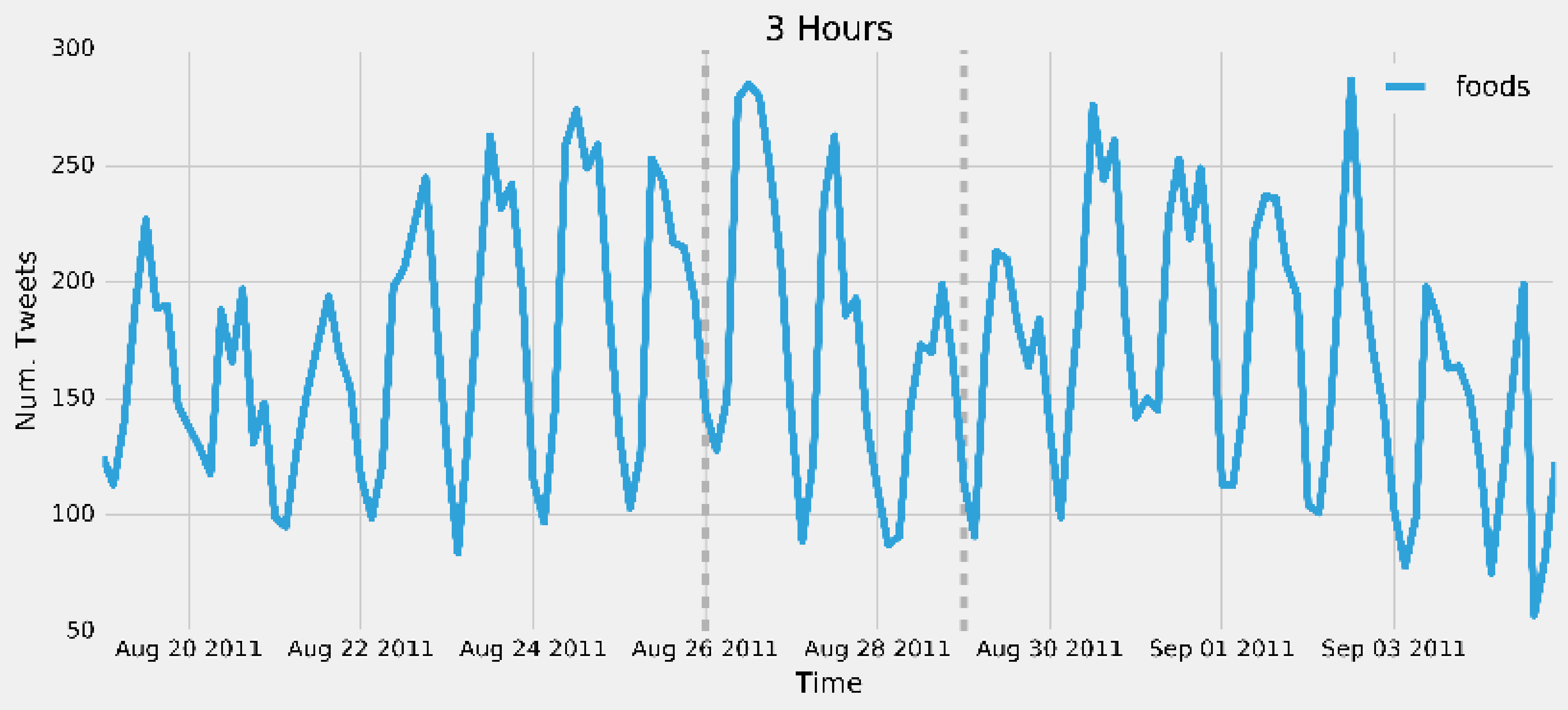

3 Hours
foods
Num. Tweets
300
250
200
150
100
50
Aug 20 2011
Aug 22 2011
Aug 24 2011
Aug 26 2011
Aug 28 2011
Aug 30 2011
Sep 01 2011
Sep 03 2011
Time

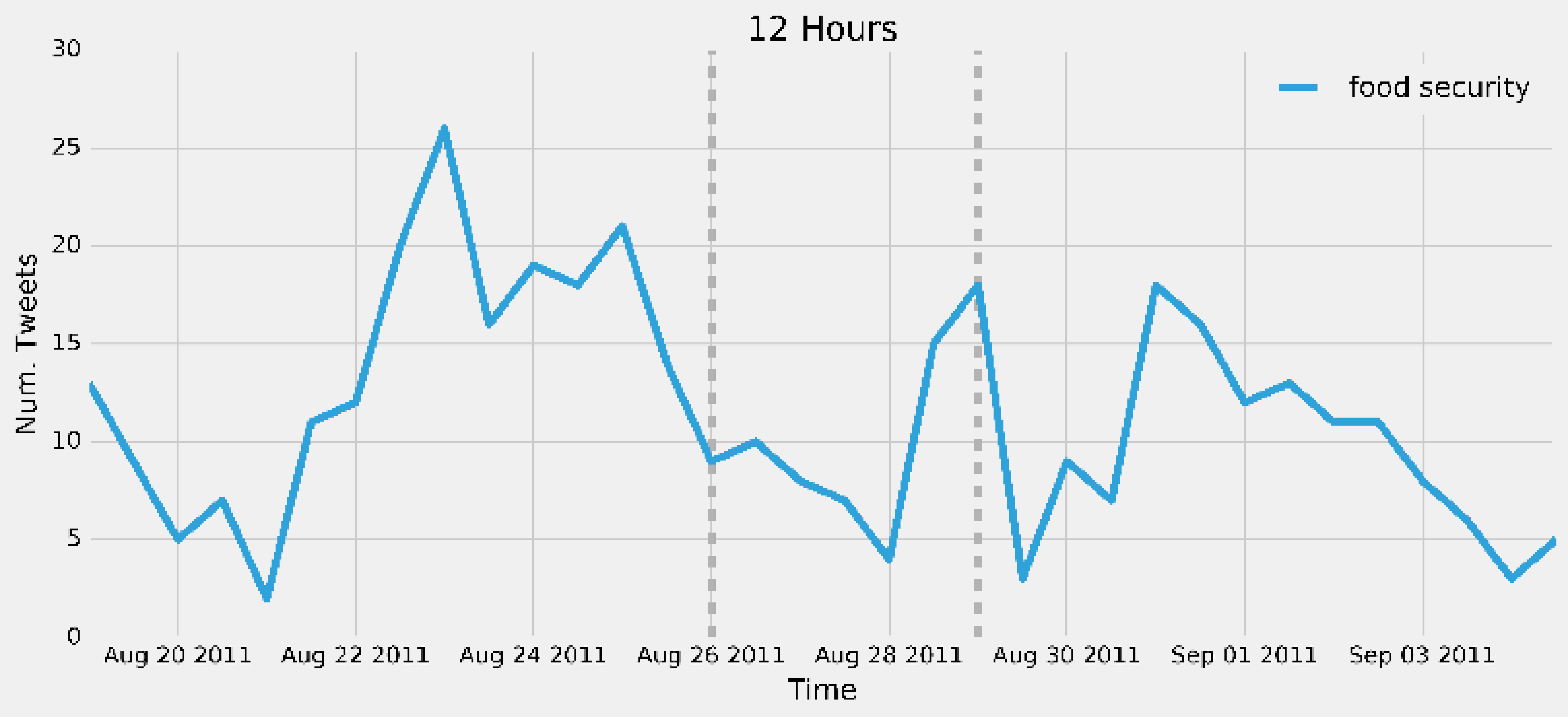

12 Hours
food security
Num. Tweets
Time
0
5
10
15
20
25
30
Aug 20 2011
Aug 22 2011
Aug 24 2011
Aug 26 2011
Aug 28 2011
Aug 30 2011
Sep 01 2011
Sep 03 2011

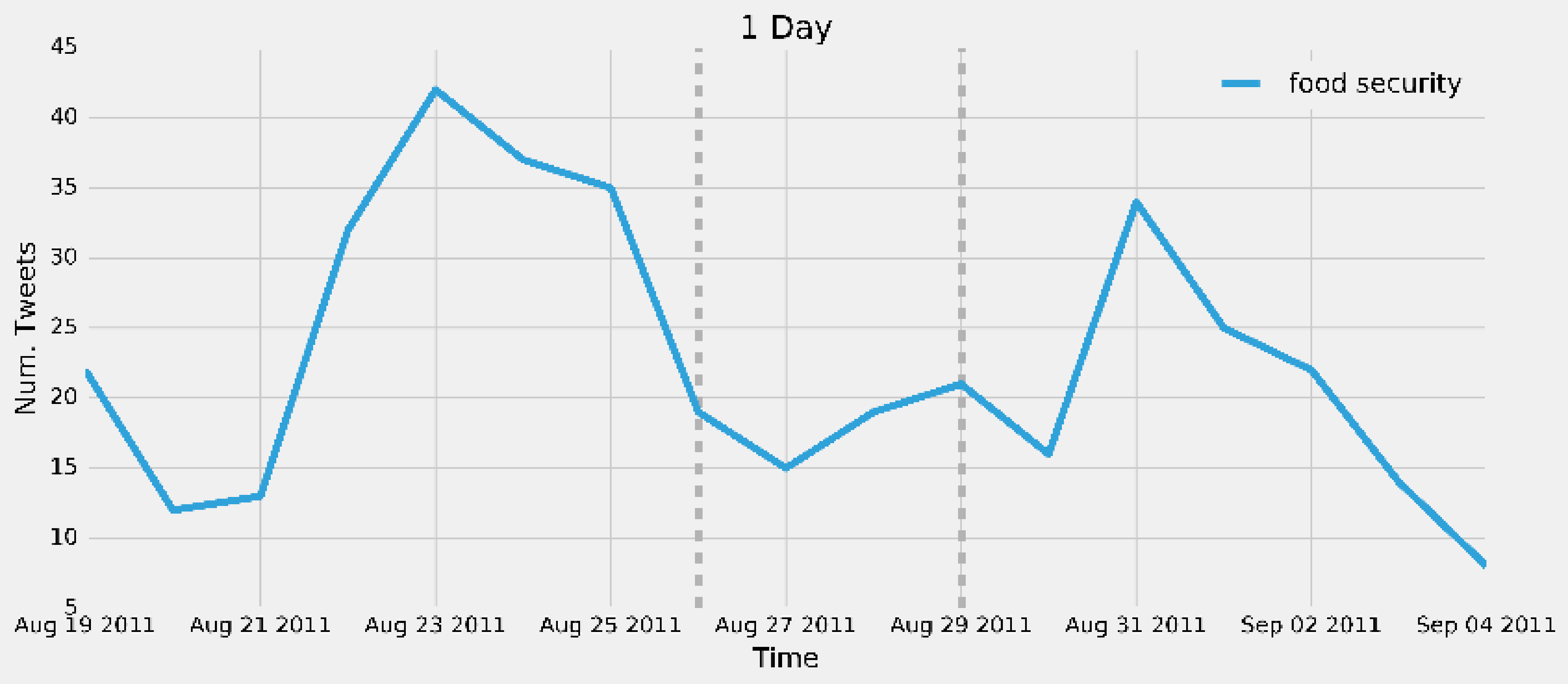
1 Day
food security
Num. Tweets
Time
45
40
35
30
25
20
15
10
5
Aug 19 2011
Aug 21 2011
Aug 23 2011
Aug 25 2011
Aug 27 2011
Aug 29 2011
Aug 31 2011
Sep 02 2011
Sep 04 2011

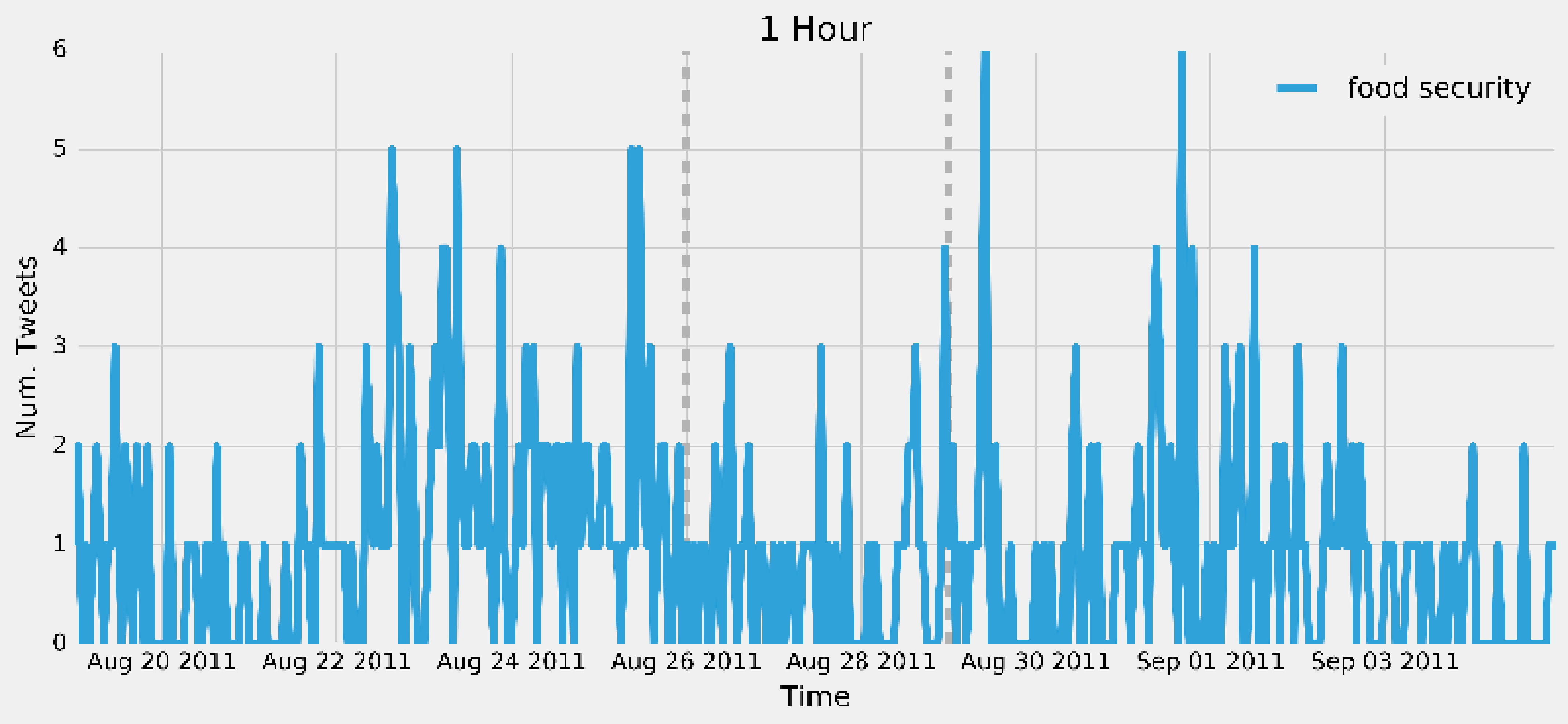
1 Hour
food security
Num. Tweets
0
1
2
3
4
5
6
Aug 20 2011
Aug 22 2011
Aug 24 2011
Aug 26 2011
Aug 28 2011
Aug 30 2011
Sep 01 2011
Sep 03 2011
Time

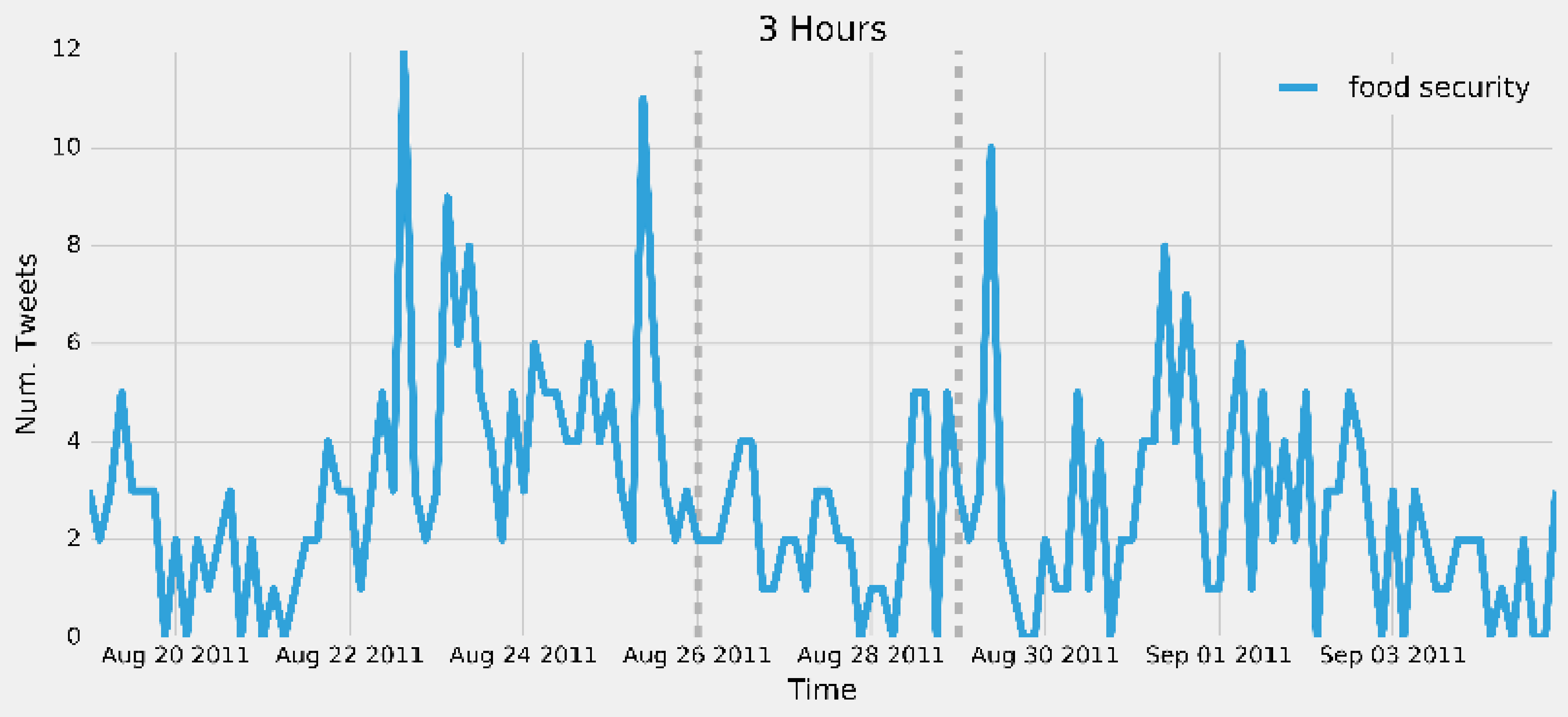

3 Hours
food security
Num. Tweets
0
2
4
6
8
10
12
Aug 20 2011
Aug 22 2011
Aug 24 2011
Aug 26 2011
Aug 28 2011
Aug 30 2011
Sep 01 2011
Sep 03 2011
Time

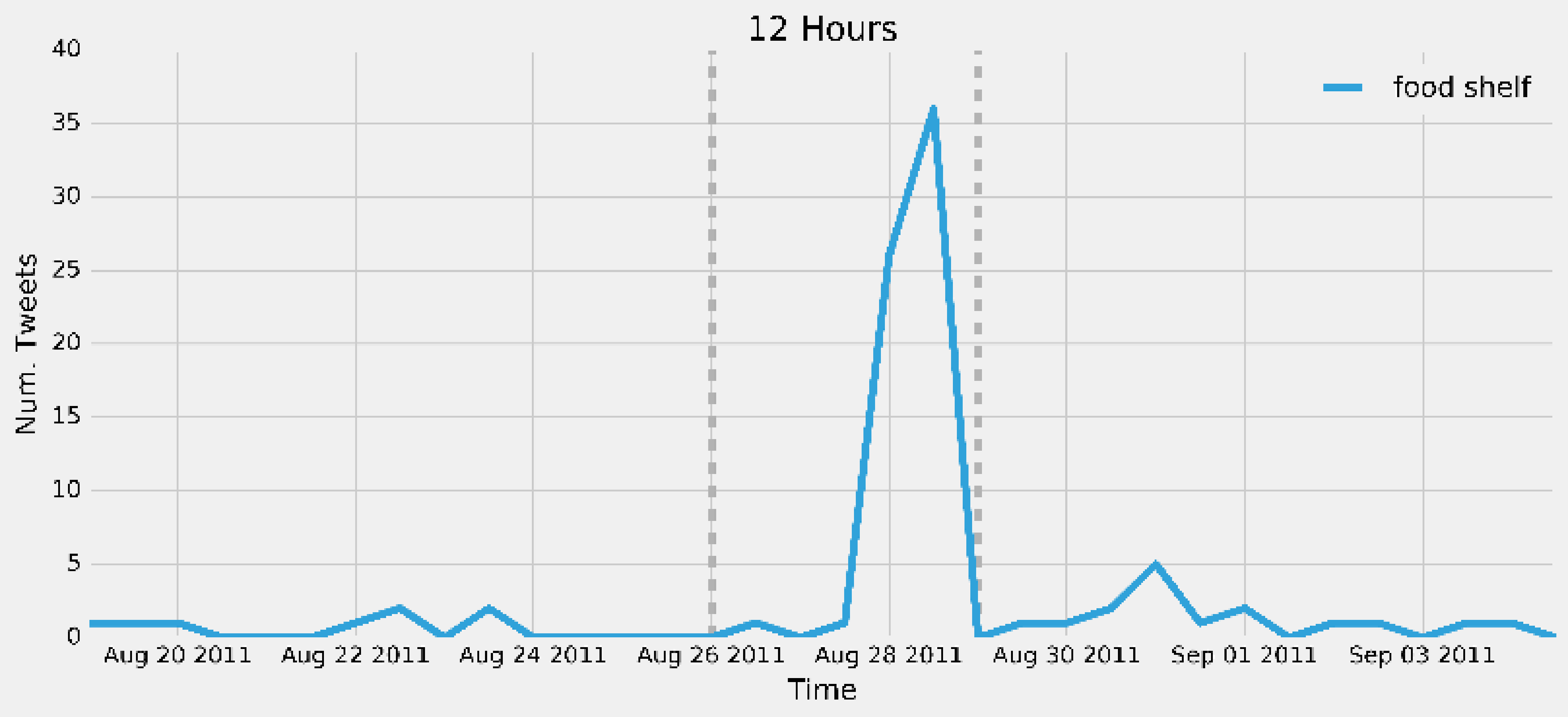

12 Hours
food shelf
Num. Tweets
Time
0
5
10
15
20
25
30
35
40
Aug 20 2011
Aug 22 2011
Aug 24 2011
Aug 26 2011
Aug 28 2011
Aug 30 2011
Sep 01 2011
Sep 03 2011

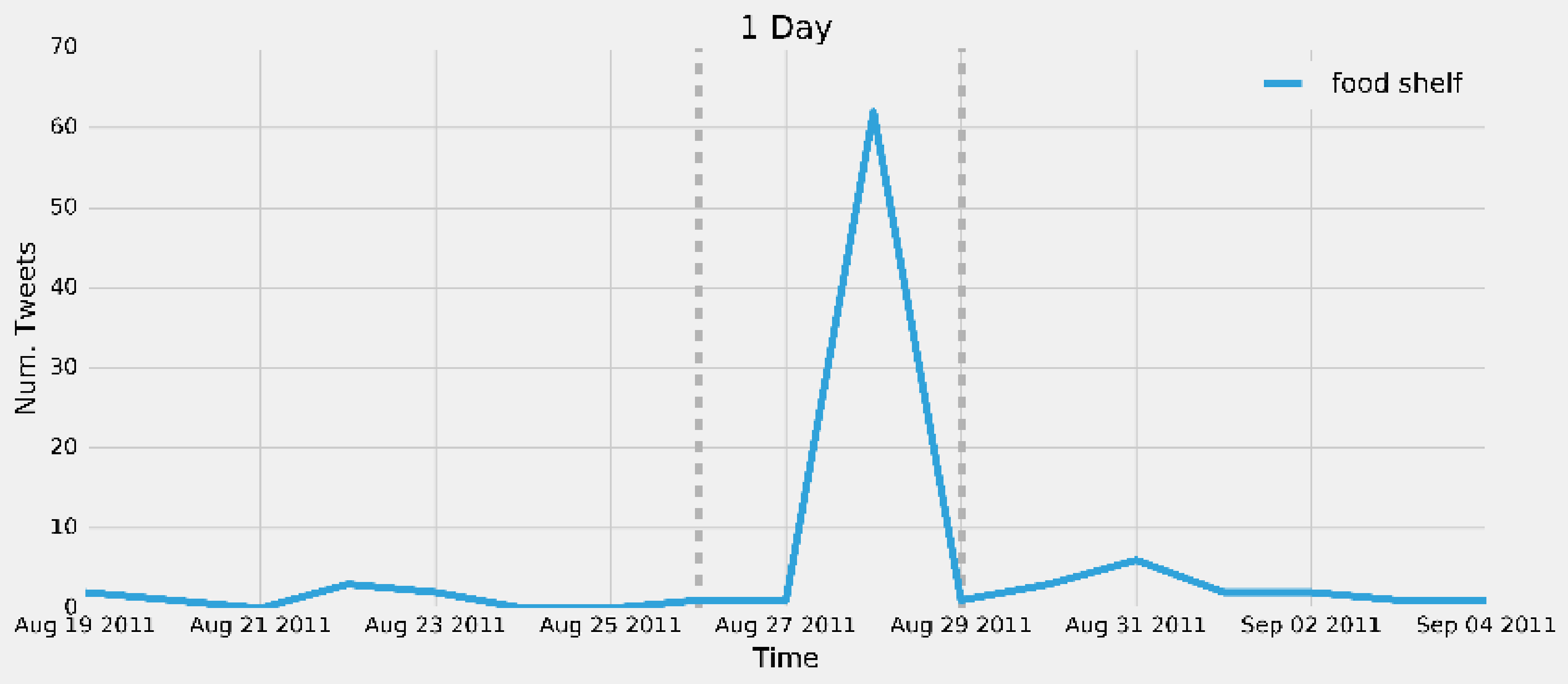

1 Day
food shelf
Num. Tweets
0
10
20
30
40
50
60
70
Aug 19 2011
Aug 21 2011
Aug 23 2011
Aug 25 2011
Aug 27 2011
Aug 29 2011
Aug 31 2011
Sep 02 2011
Sep 04 2011
Time

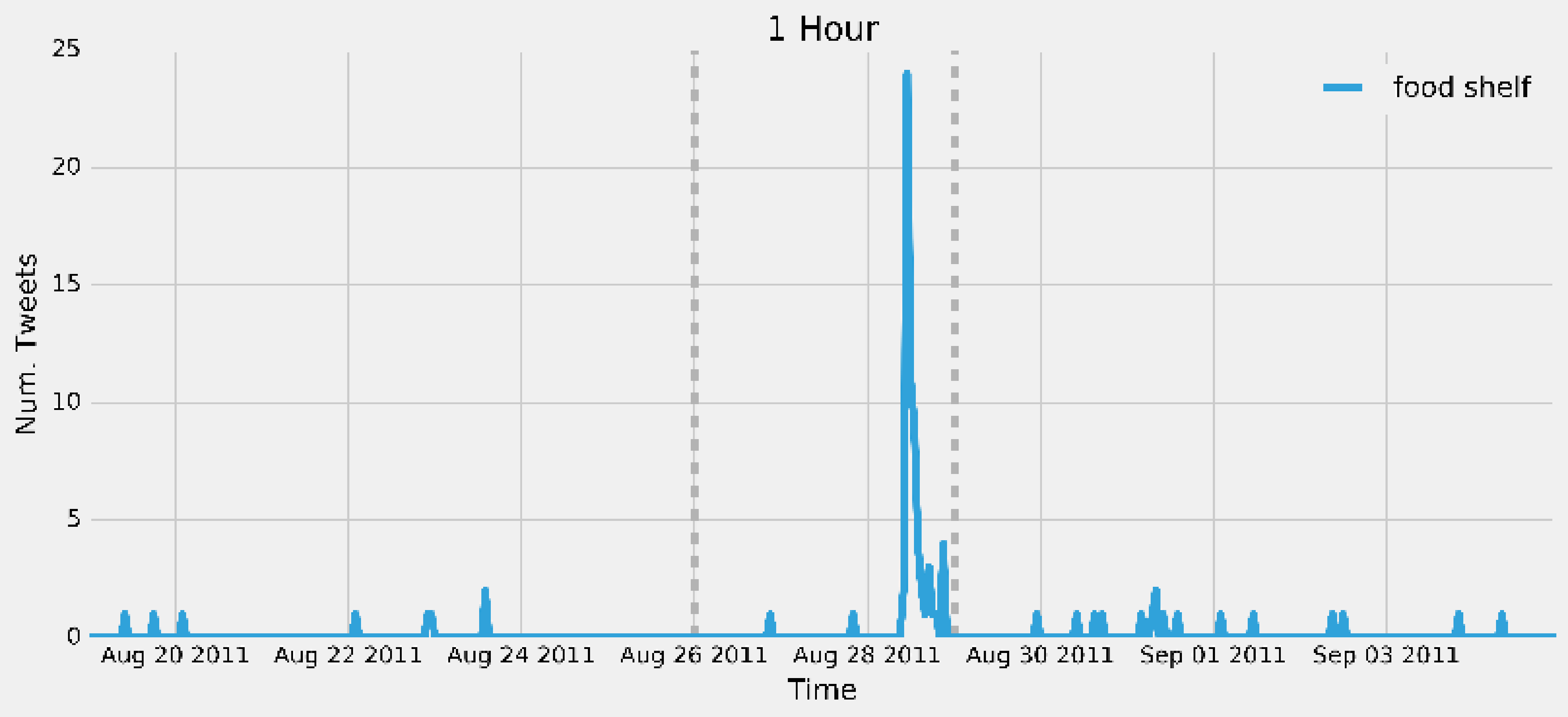

1 Hour
food shelf
Num. Tweets
Time
0
5
10
15
20
25
Aug 20 2011
Aug 22 2011
Aug 24 2011
Aug 26 2011
Aug 28 2011
Aug 30 2011
Sep 01 2011
Sep 03 2011

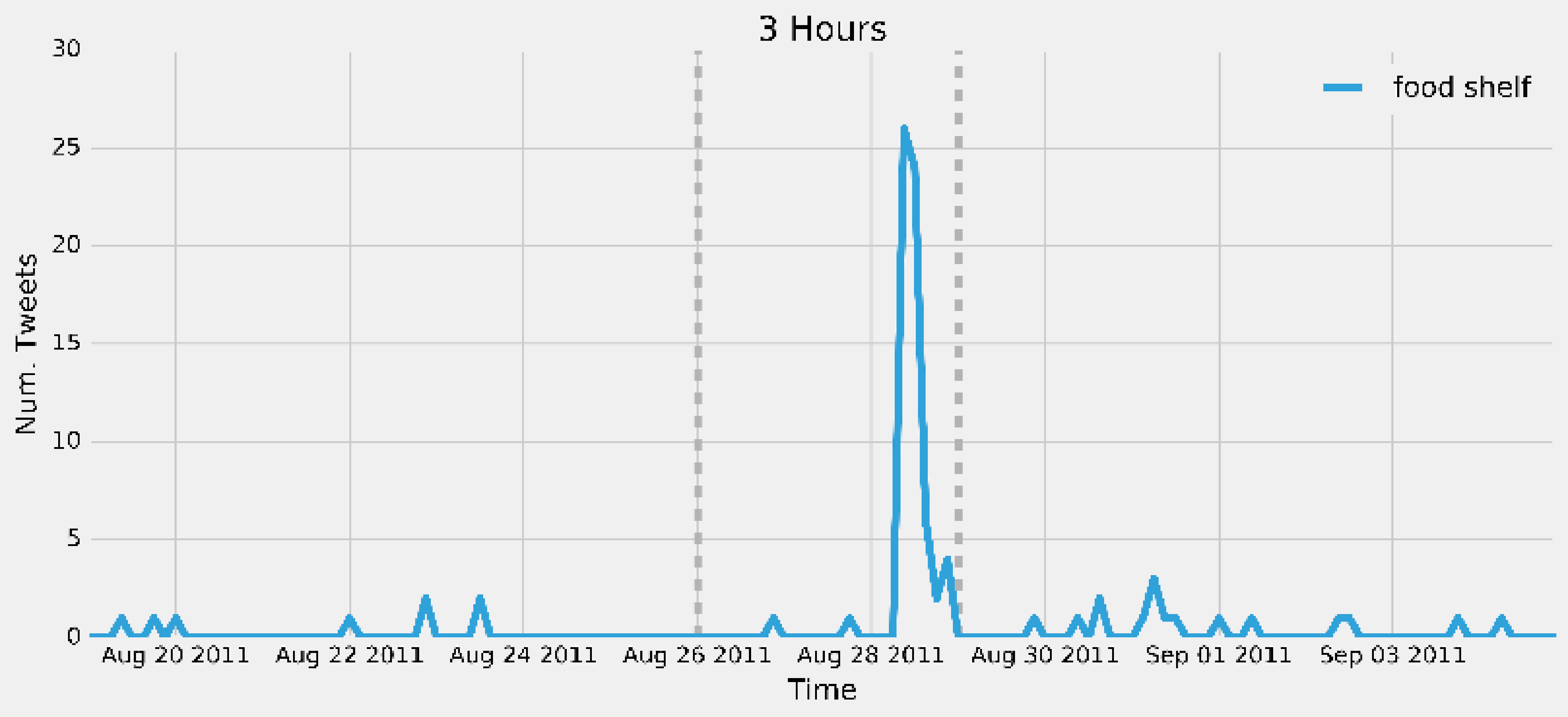
3 Hours
food shelf
Num. Tweets
30
25
20
15
10
5
0
Aug 20 2011
Aug 22 2011
Aug 24 2011
Aug 26 2011
Aug 28 2011
Aug 30 2011
Sep 01 2011
Sep 03 2011
Time

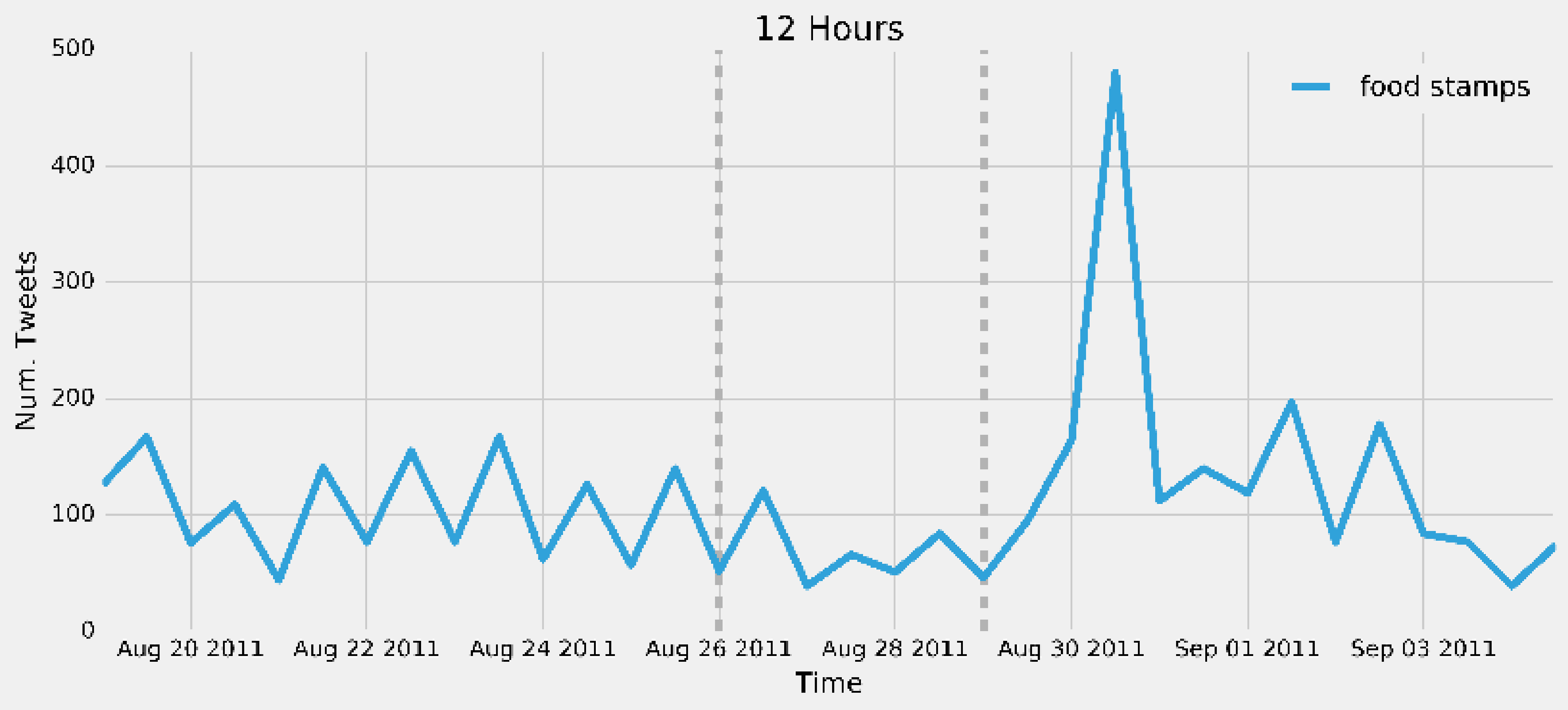
12 Hours
food stamps
Num. Tweets
0
100
200
300
400
500
Aug 20 2011
Aug 22 2011
Aug 24 2011
Aug 26 2011
Aug 28 2011
Aug 30 2011
Sep 01 2011
Sep 03 2011
Time

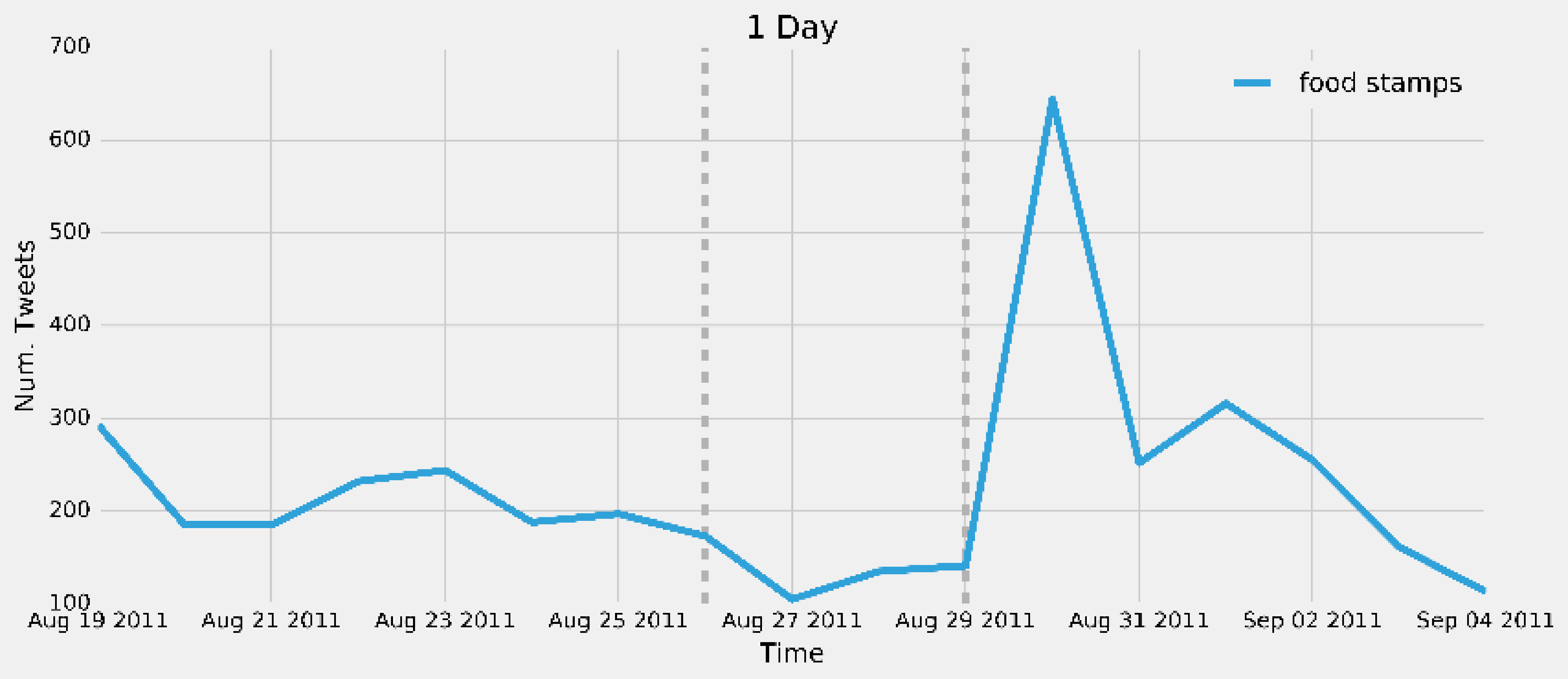

1 Day
food stamps
Num. Tweets
700
600
500
400
300
200
100
Aug 19 2011
Aug 21 2011
Aug 23 2011
Aug 25 2011
Aug 27 2011
Aug 29 2011
Aug 31 2011
Sep 02 2011
Sep 04 2011
Time

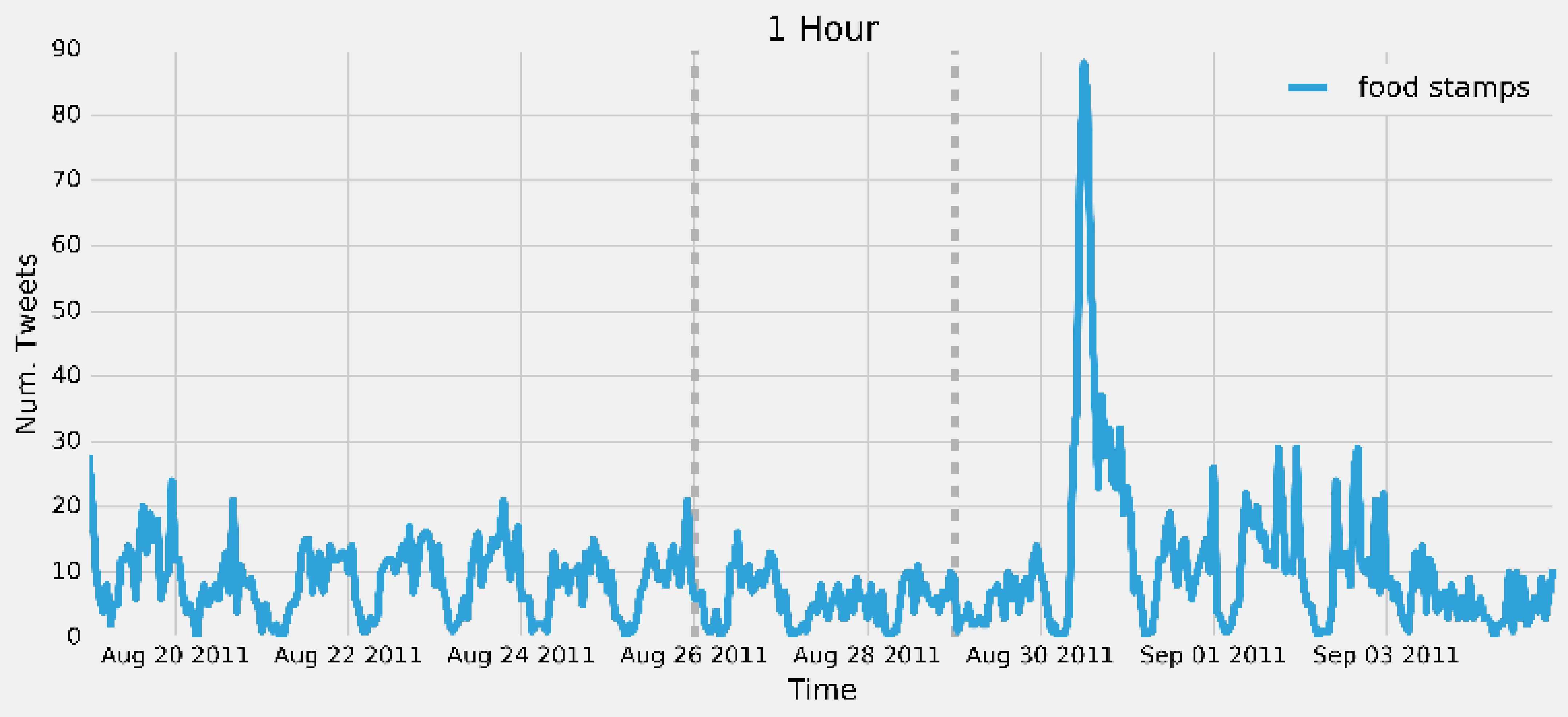

1 Hour
food stamps
Num. Tweets
0
10
20
30
40
50
60
70
80
90
Aug 20 2011
Aug 22 2011
Aug 24 2011
Aug 26 2011
Aug 28 2011
Aug 30 2011
Sep 01 2011
Sep 03 2011
Time

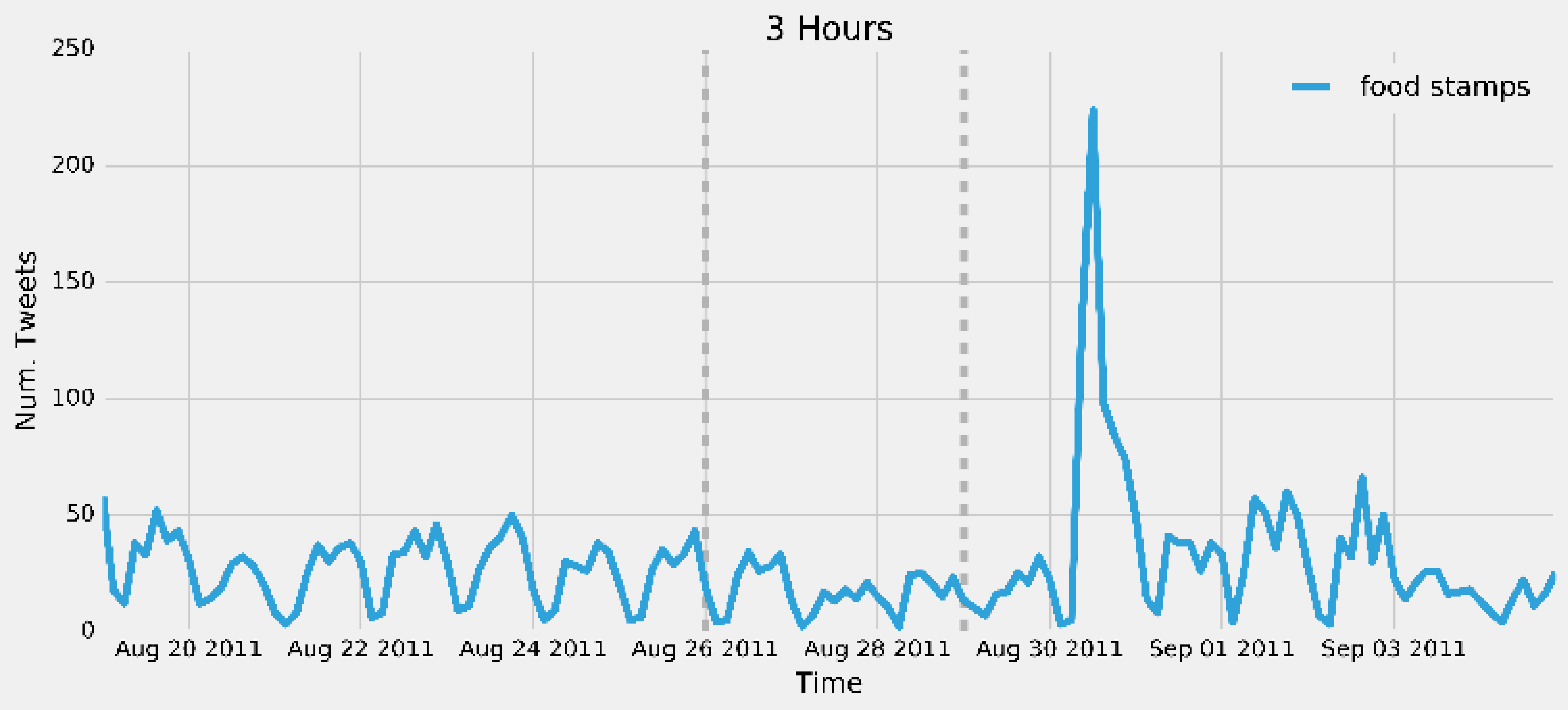

3 Hours
food stamps
Num. Tweets
0
50
100
150
200
250
Aug 20 2011
Aug 22 2011
Aug 24 2011
Aug 26 2011
Aug 28 2011
Aug 30 2011
Sep 01 2011
Sep 03 2011
Time

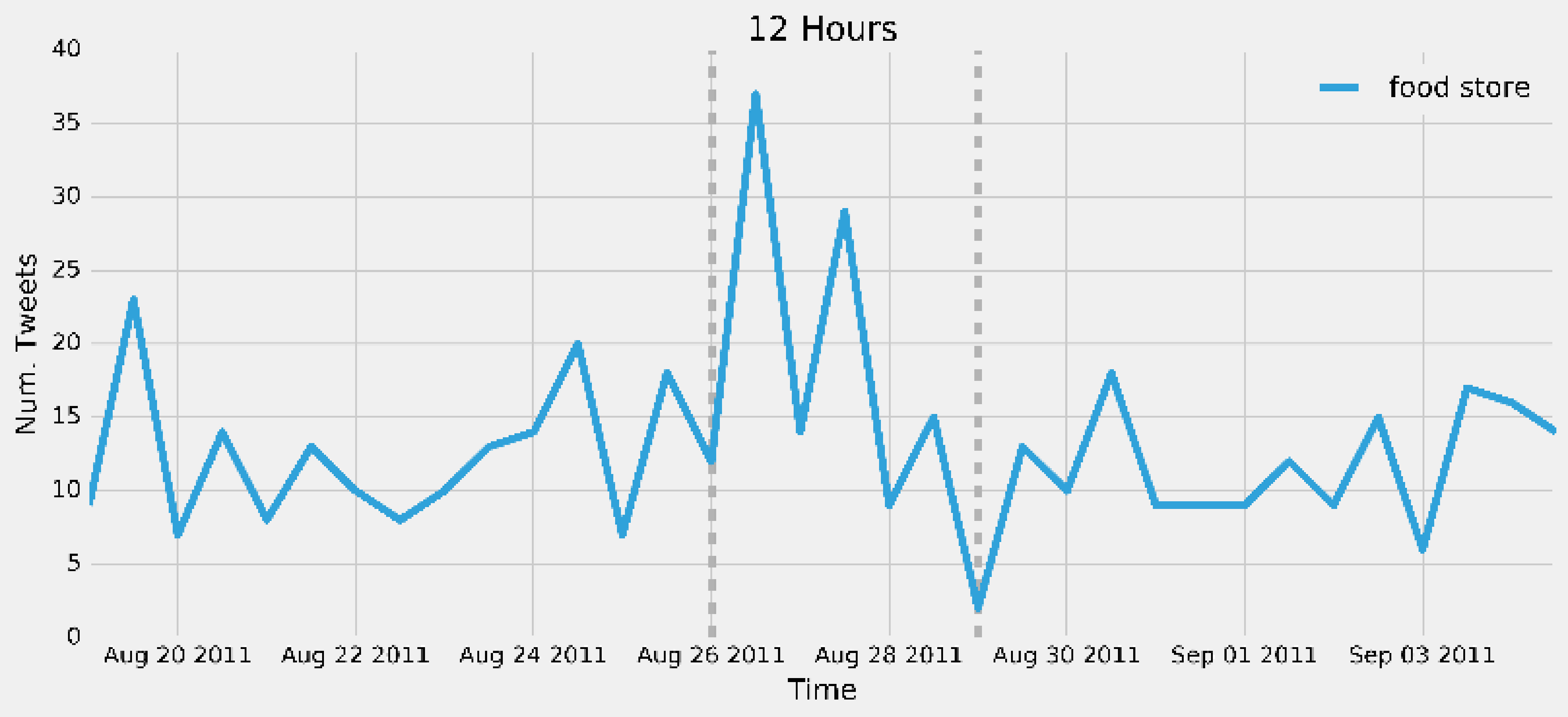

12 Hours
food store
Num. Tweets
0
5
10
15
20
25
30
35
40
Aug 20 2011
Aug 22 2011
Aug 24 2011
Aug 26 2011
Aug 28 2011
Aug 30 2011
Sep 01 2011
Sep 03 2011
Time

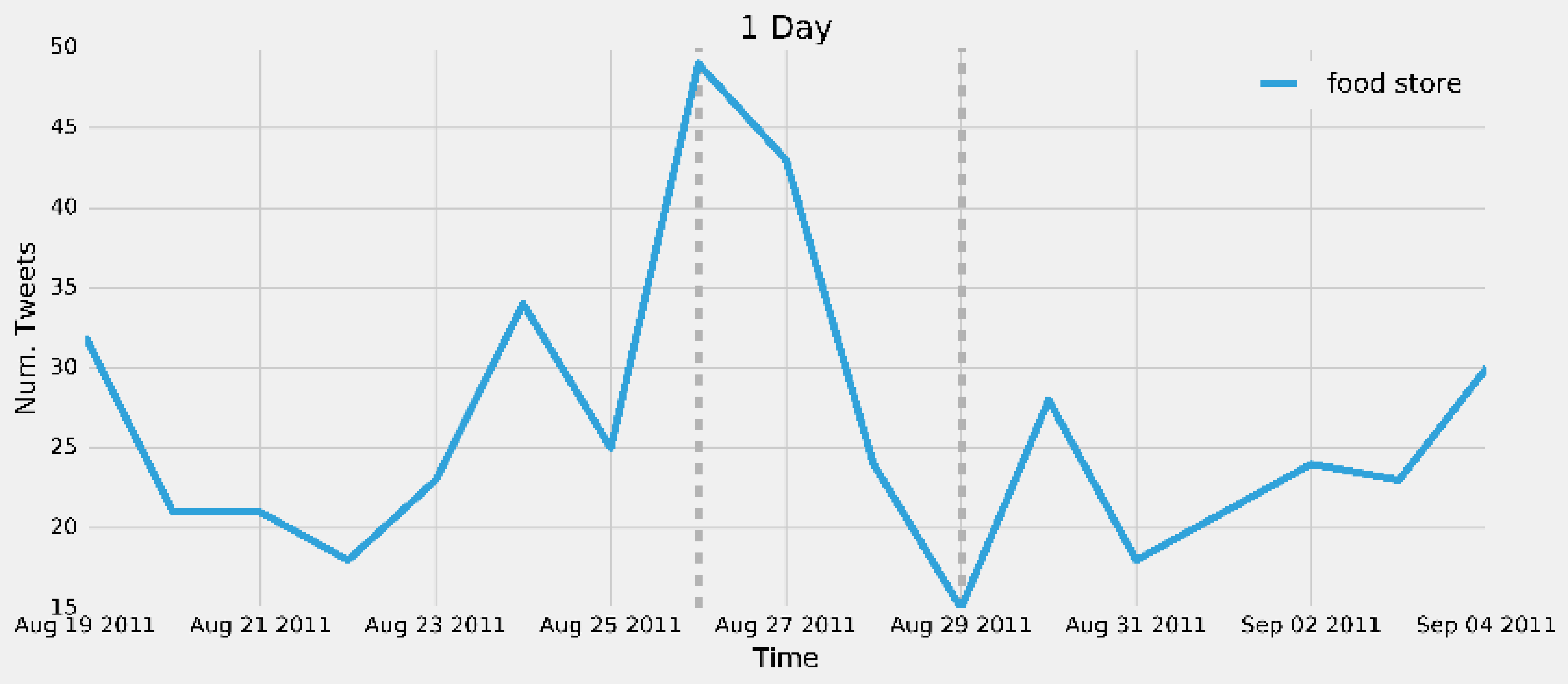
1 Day
food store
Num. Tweets
50
45
40
35
30
25
20
15
Aug 19 2011
Aug 21 2011
Aug 23 2011
Aug 25 2011
Aug 27 2011
Aug 29 2011
Aug 31 2011
Sep 02 2011
Sep 04 2011
Time

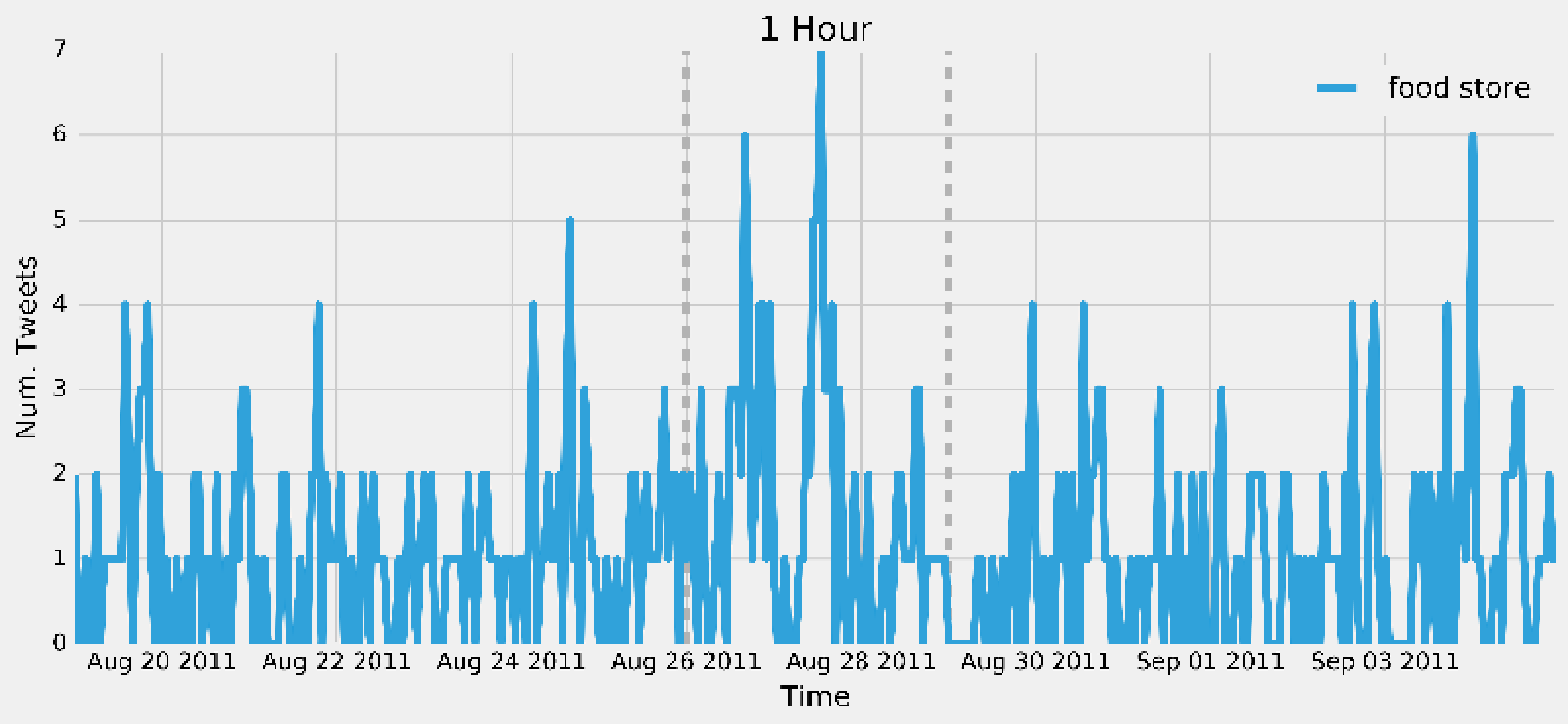
1 Hour
food store
Num. Tweets
0
1
2
3
4
5
6
7
Aug 20 2011
Aug 22 2011
Aug 24 2011
Aug 26 2011
Aug 28 2011
Aug 30 2011
Sep 01 2011
Sep 03 2011
Time

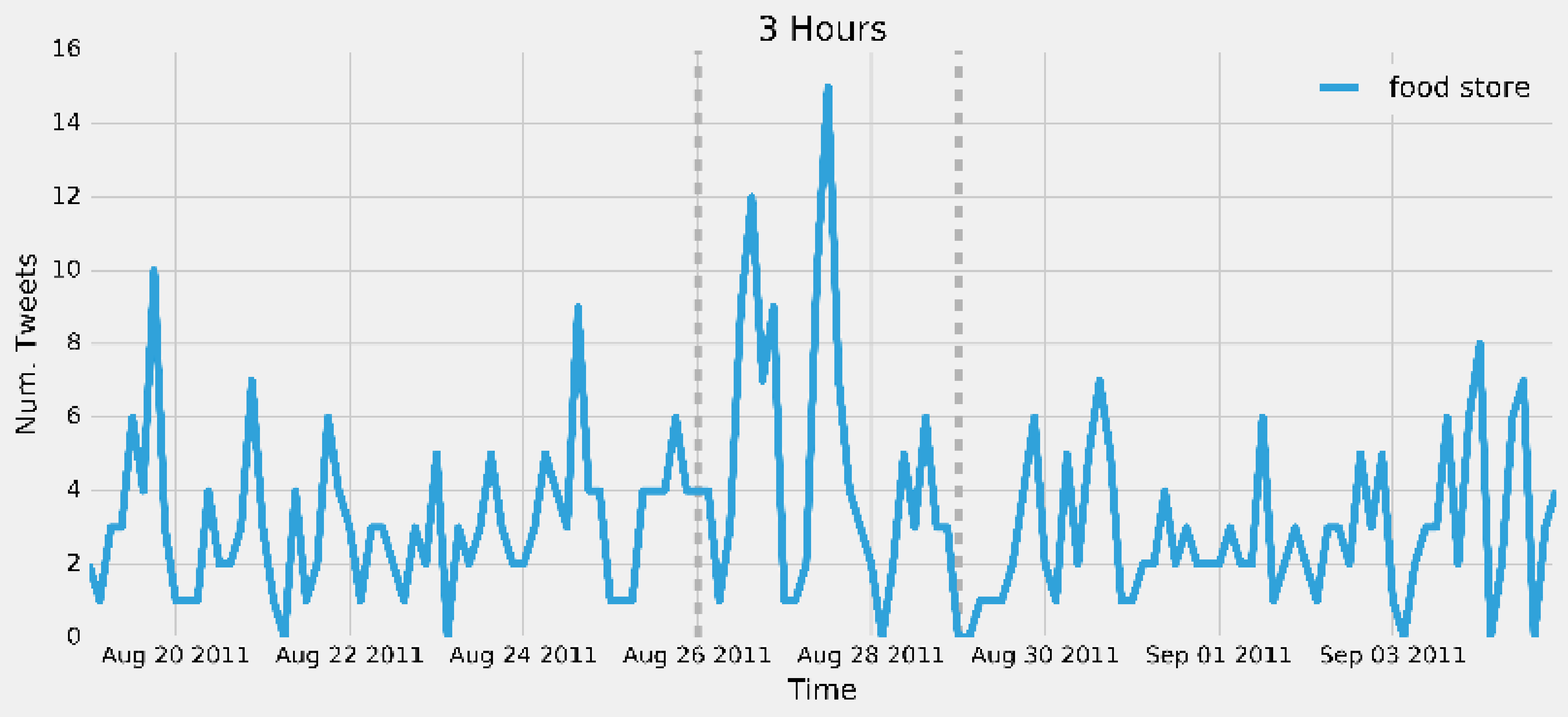
3 Hours
food store
Num. Tweets
Time
0
2
4
6
8
10
12
14
16
Aug 20 2011
Aug 22 2011
Aug 24 2011
Aug 26 2011
Aug 28 2011
Aug 30 2011
Sep 01 2011
Sep 03 2011

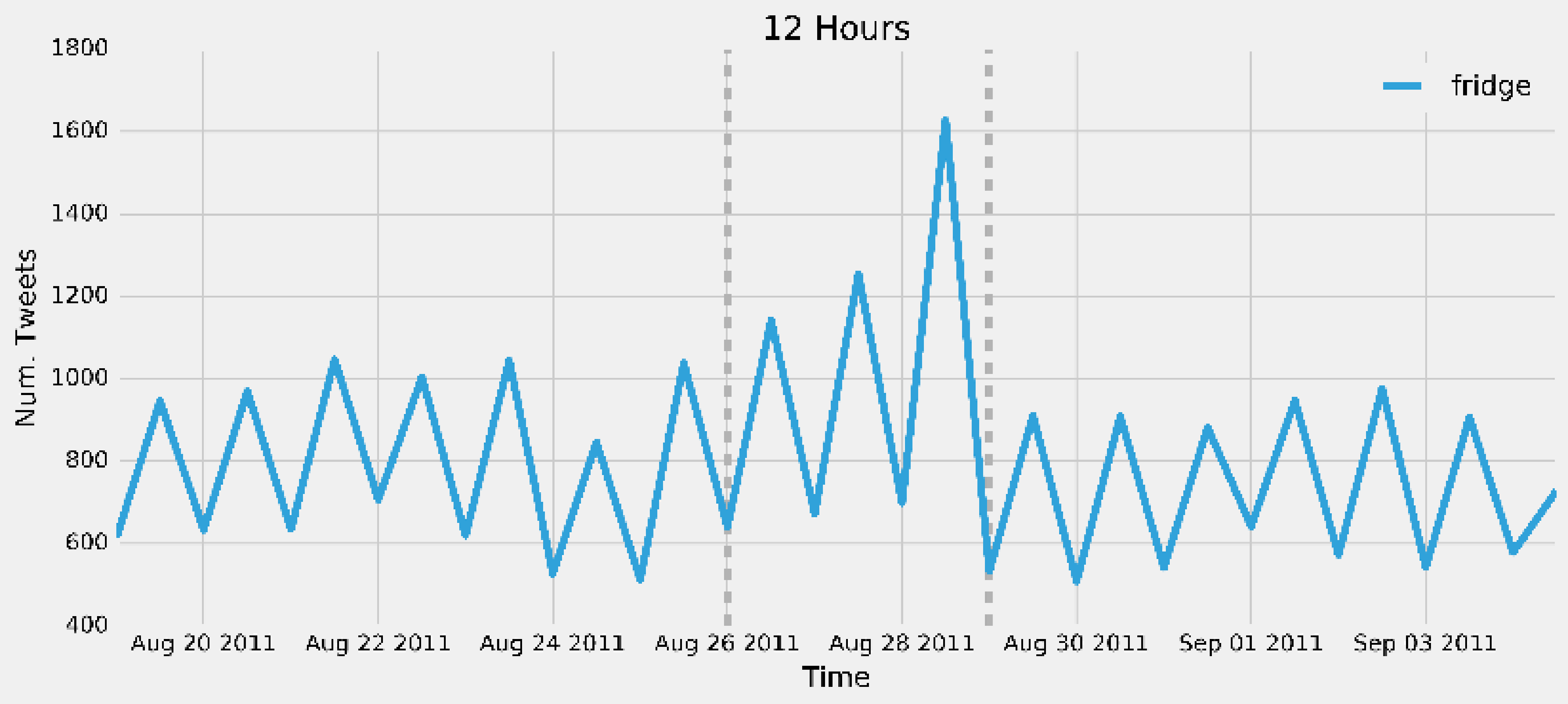
12 Hours
fridge
Num. Tweets
1800
1600
1400
1200
1000
800
600
400
Aug 20 2011
Aug 22 2011
Aug 24 2011
Aug 26 2011
Aug 28 2011
Aug 30 2011
Sep 01 2011
Sep 03 2011
Time

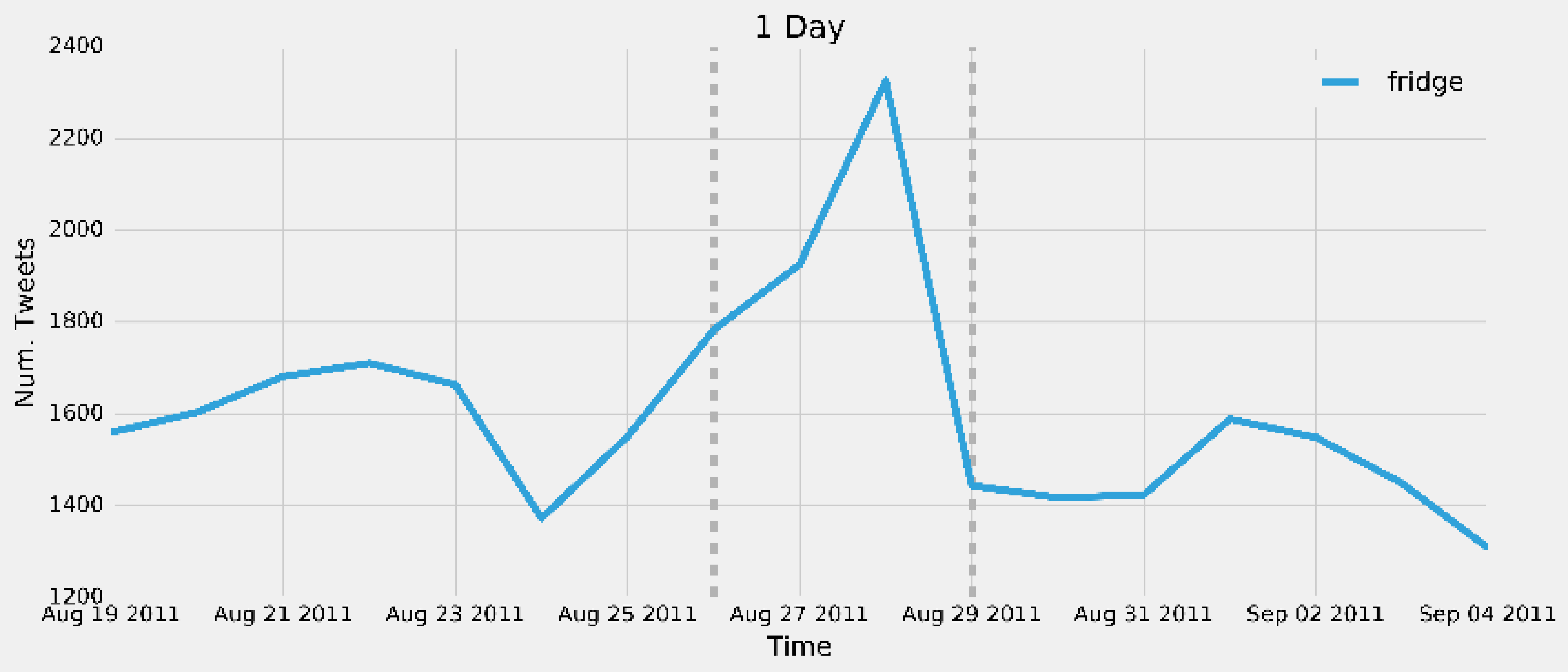
1 Day
fridge
Num. Tweets
Time
2400
2200
2000
1800
1600
1400
1200
Aug 19 2011
Aug 21 2011
Aug 23 2011
Aug 25 2011
Aug 27 2011
Aug 29 2011
Aug 31 2011
Sep 02 2011
Sep 04 2011

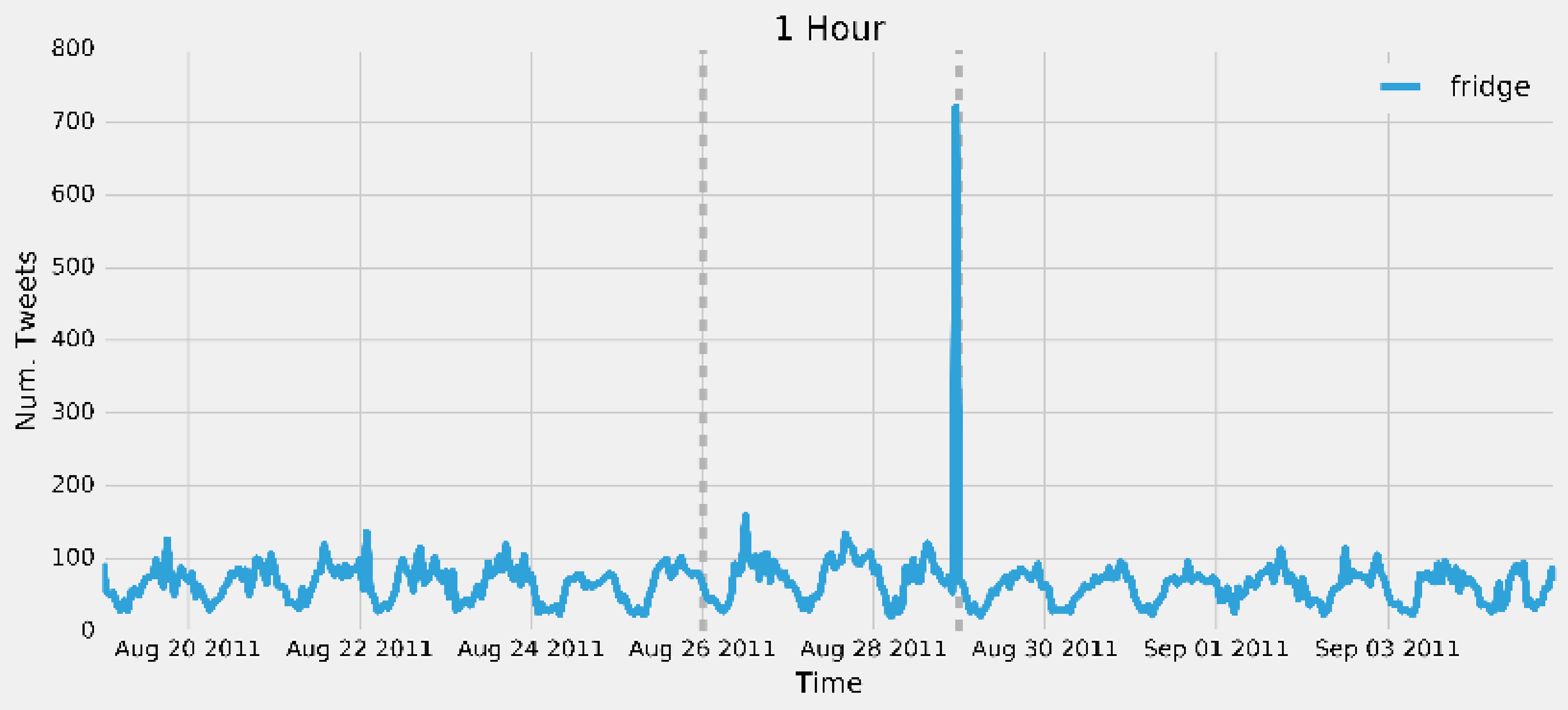
1 Hour
Num. Tweets
Time
fridge
0
100
200
300
400
500
600
700
800
Aug 20 2011
Aug 22 2011
Aug 24 2011
Aug 26 2011
Aug 28 2011
Aug 30 2011
Sep 01 2011
Sep 03 2011

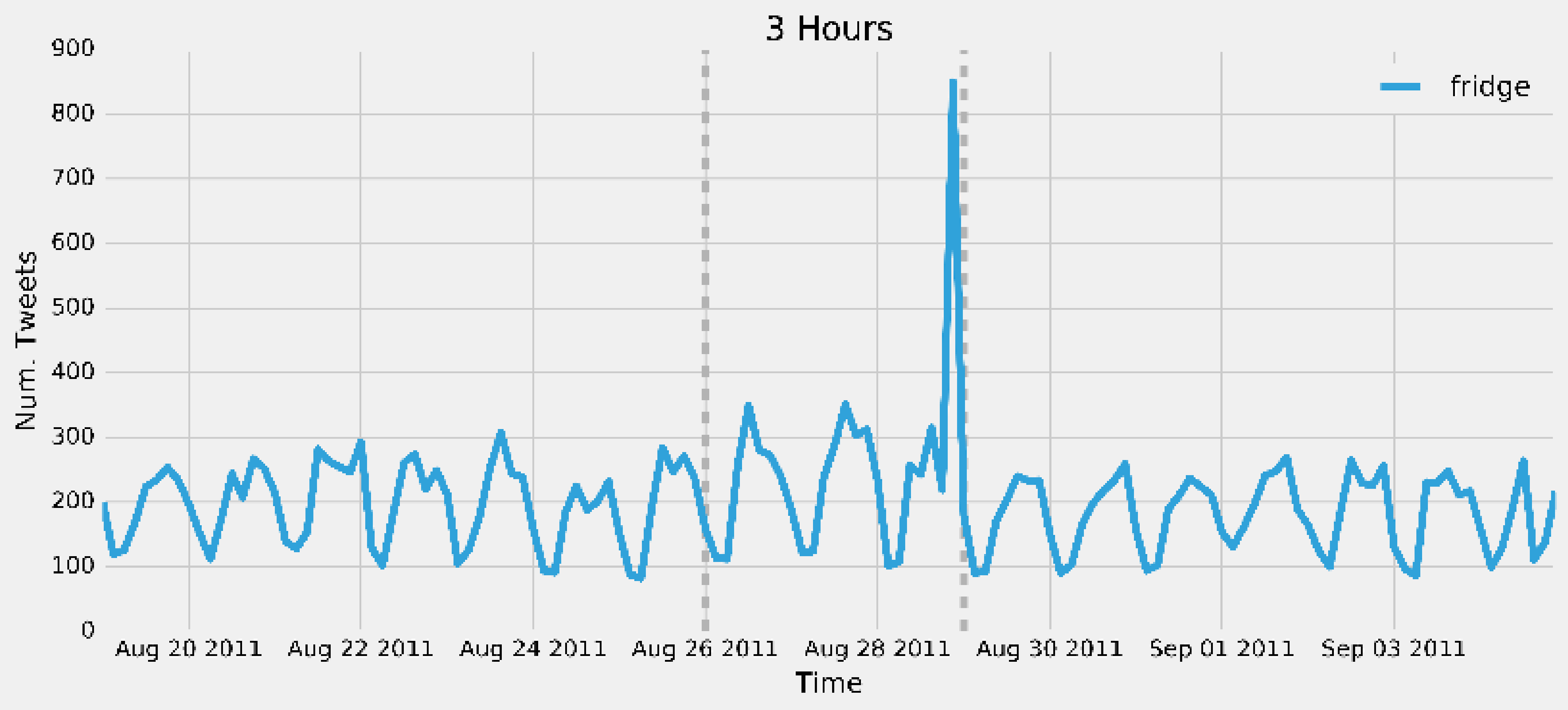
3 Hours
fridge
Num. Tweets
0
100
200
300
400
500
600
700
800
900
Aug 20 2011
Aug 22 2011
Aug 24 2011
Aug 26 2011
Aug 28 2011
Aug 30 2011
Sep 01 2011
Sep 03 2011
Time

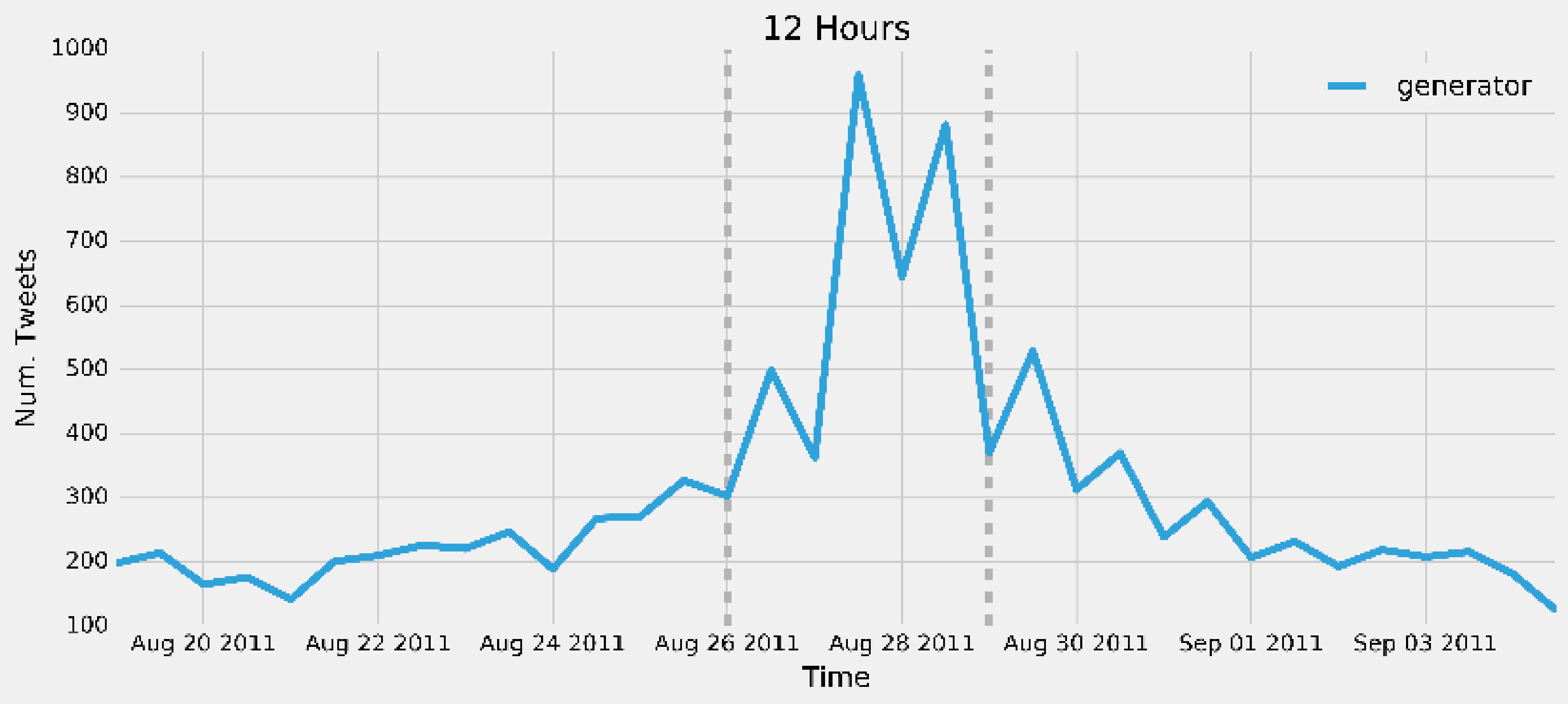
12 Hours
generator
Num. Tweets
1000
900
800
700
600
500
400
300
200
100
Aug 20 2011
Aug 22 2011
Aug 24 2011
Aug 26 2011
Aug 28 2011
Aug 30 2011
Sep 01 2011
Sep 03 2011
Time

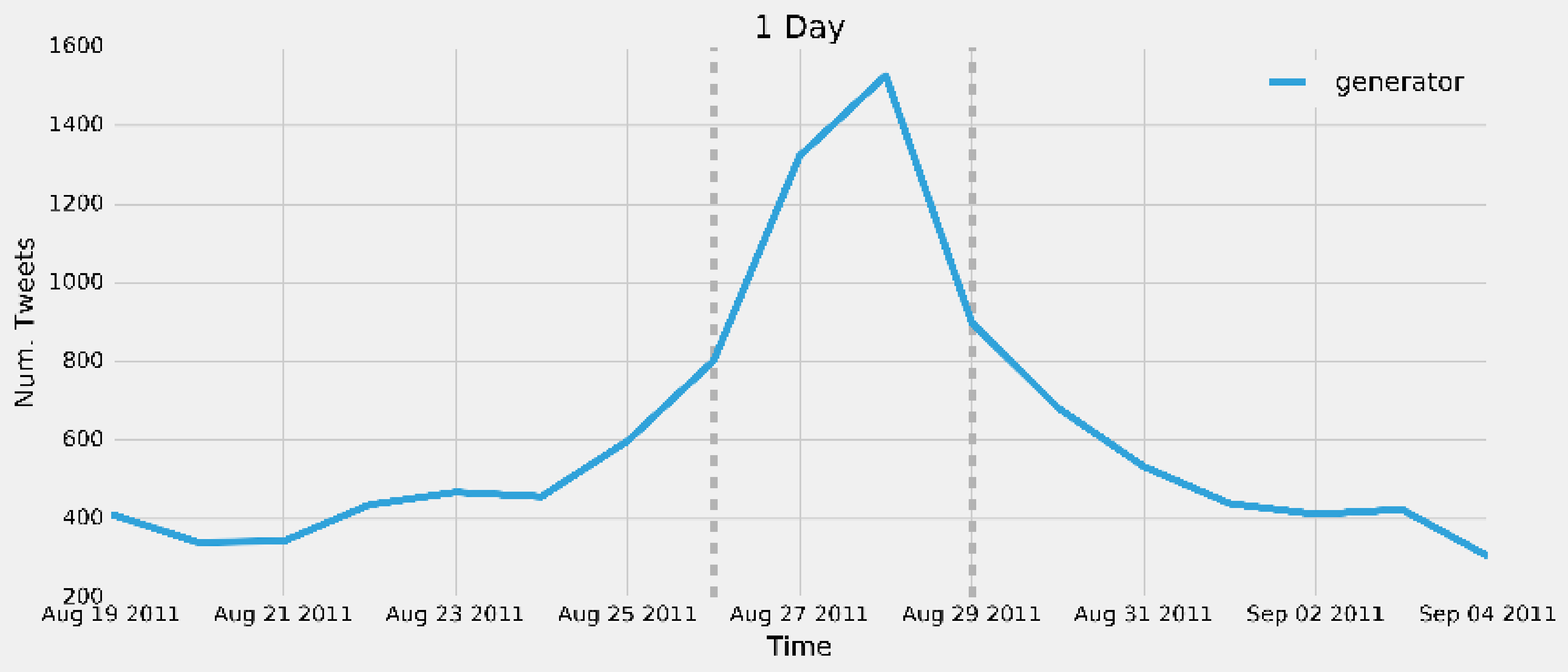

1 Day
generator
1600
1400
1200
1000
800
600
400
200
Num. Tweets
Aug 19 2011
Aug 21 2011
Aug 23 2011
Aug 25 2011
Aug 27 2011
Aug 29 2011
Aug 31 2011
Sep 02 2011
Sep 04 2011
Time

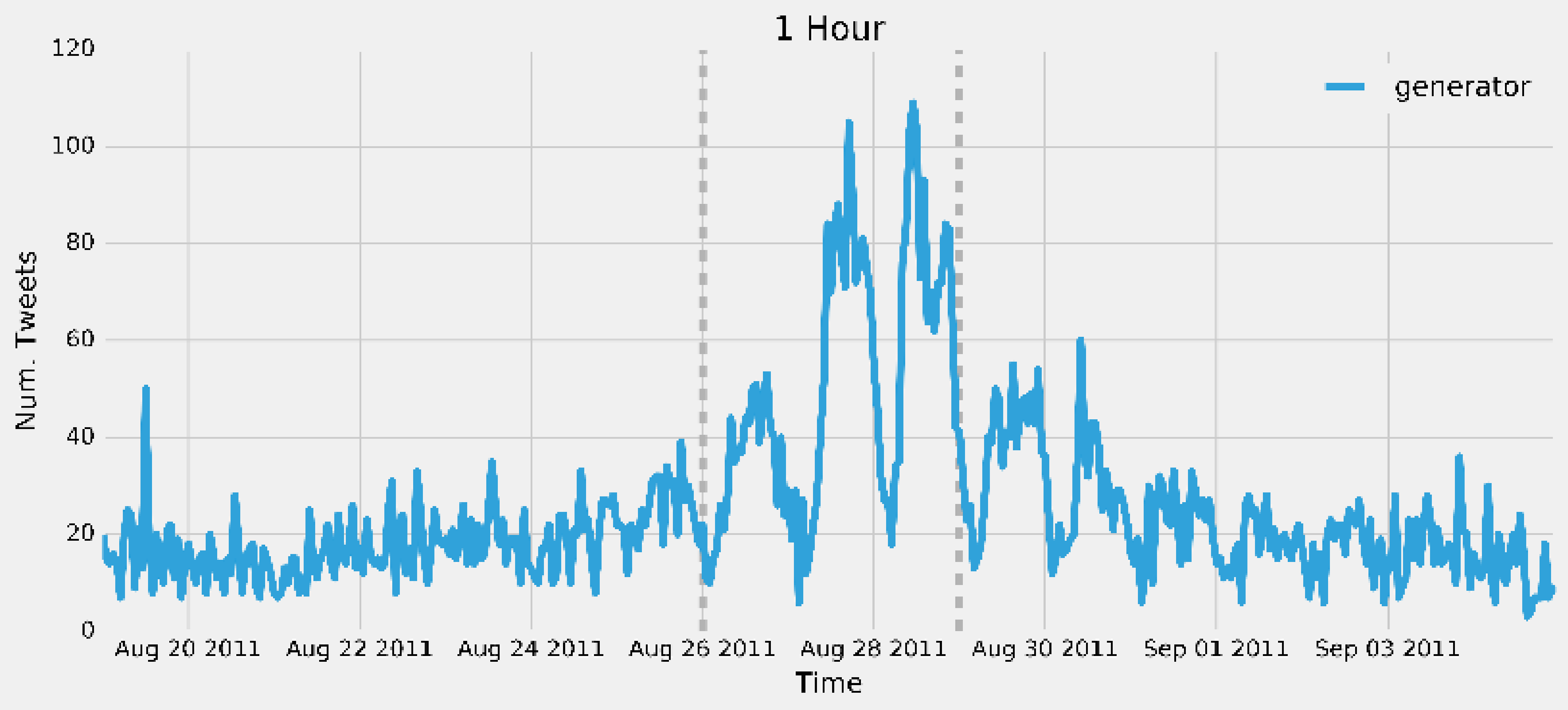

1 Hour
generator
Num. Tweets
0
20
40
60
80
100
120
Aug 20 2011
Aug 22 2011
Aug 24 2011
Aug 26 2011
Aug 28 2011
Aug 30 2011
Sep 01 2011
Sep 03 2011
Time

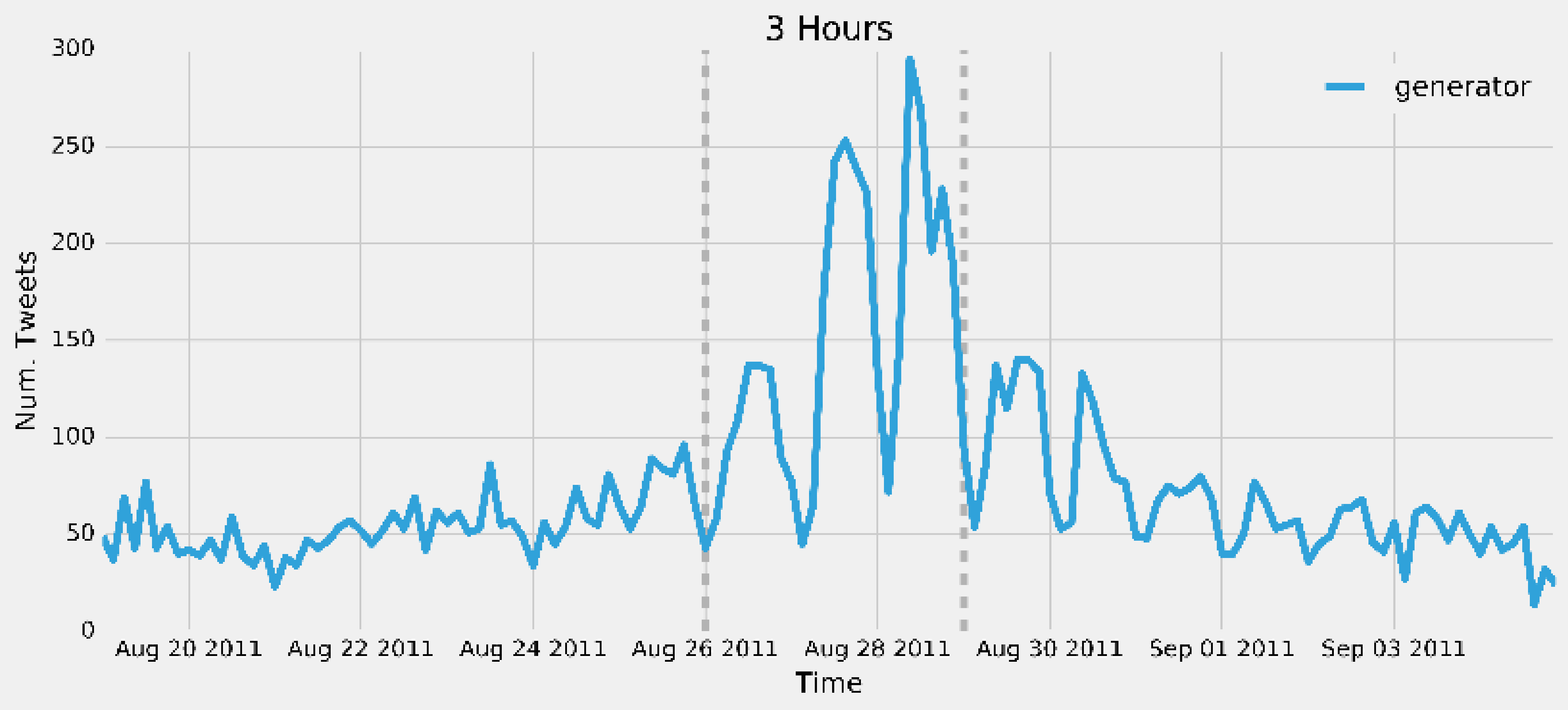
3 Hours
generator
Num. Tweets
300
250
200
150
100
50
0
Aug 20 2011
Aug 22 2011
Aug 24 2011
Aug 26 2011
Aug 28 2011
Aug 30 2011
Sep 01 2011
Sep 03 2011
Time

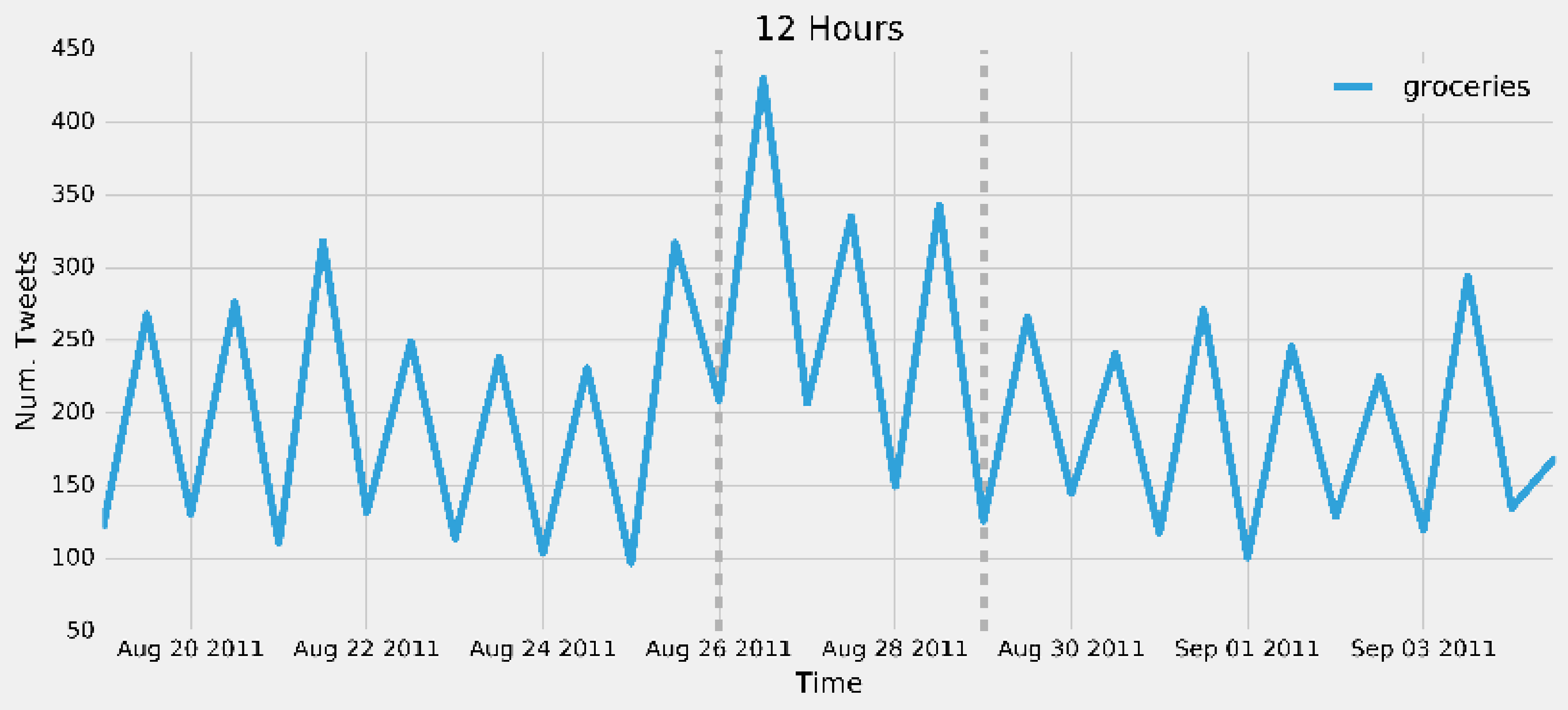
12 Hours
groceries
Num. Tweets
450
400
350
300
250
200
150
100
50
Aug 20 2011
Aug 22 2011
Aug 24 2011
Aug 26 2011
Aug 28 2011
Aug 30 2011
Sep 01 2011
Sep 03 2011
Time

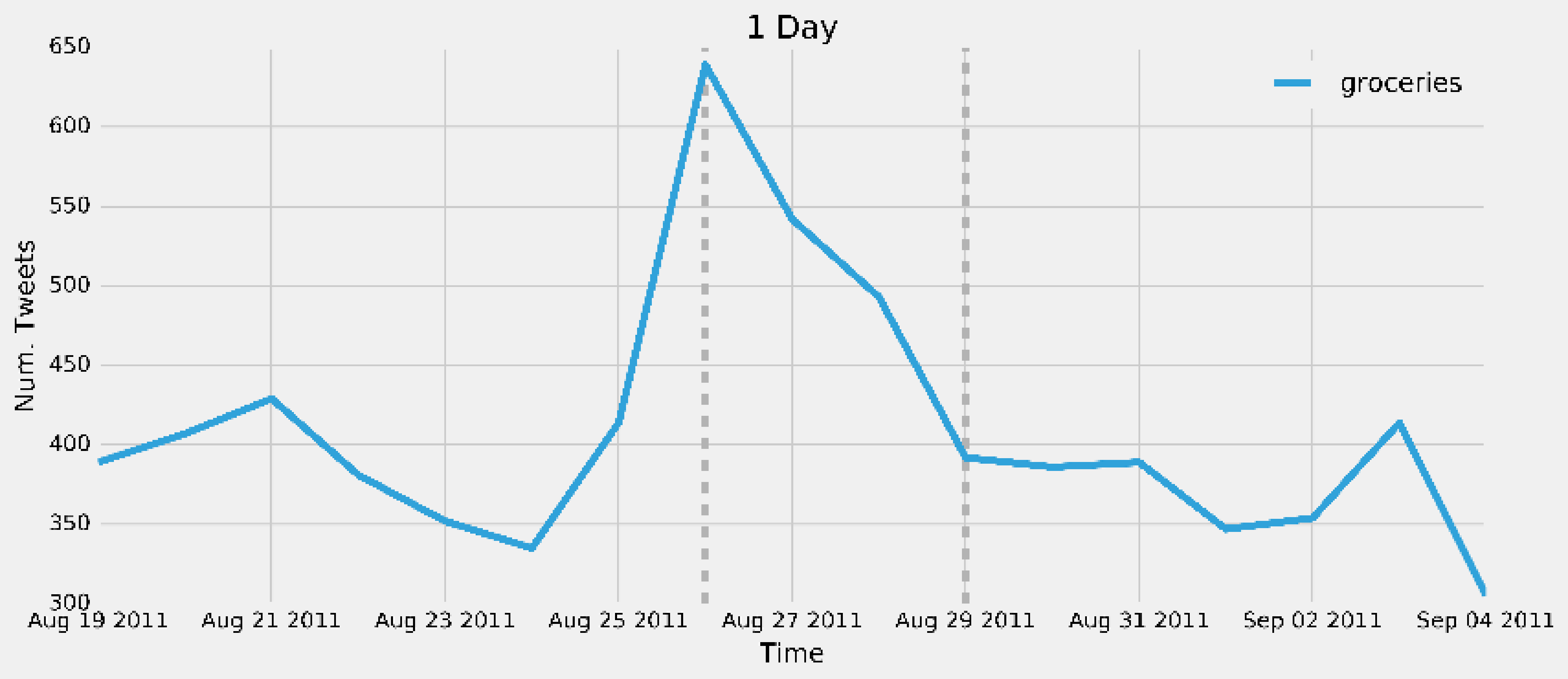
1 Day
groceries
Num. Tweets
650
600
550
500
450
400
350
300
Aug 19 2011
Aug 21 2011
Aug 23 2011
Aug 25 2011
Aug 27 2011
Aug 29 2011
Aug 31 2011
Sep 02 2011
Sep 04 2011
Time

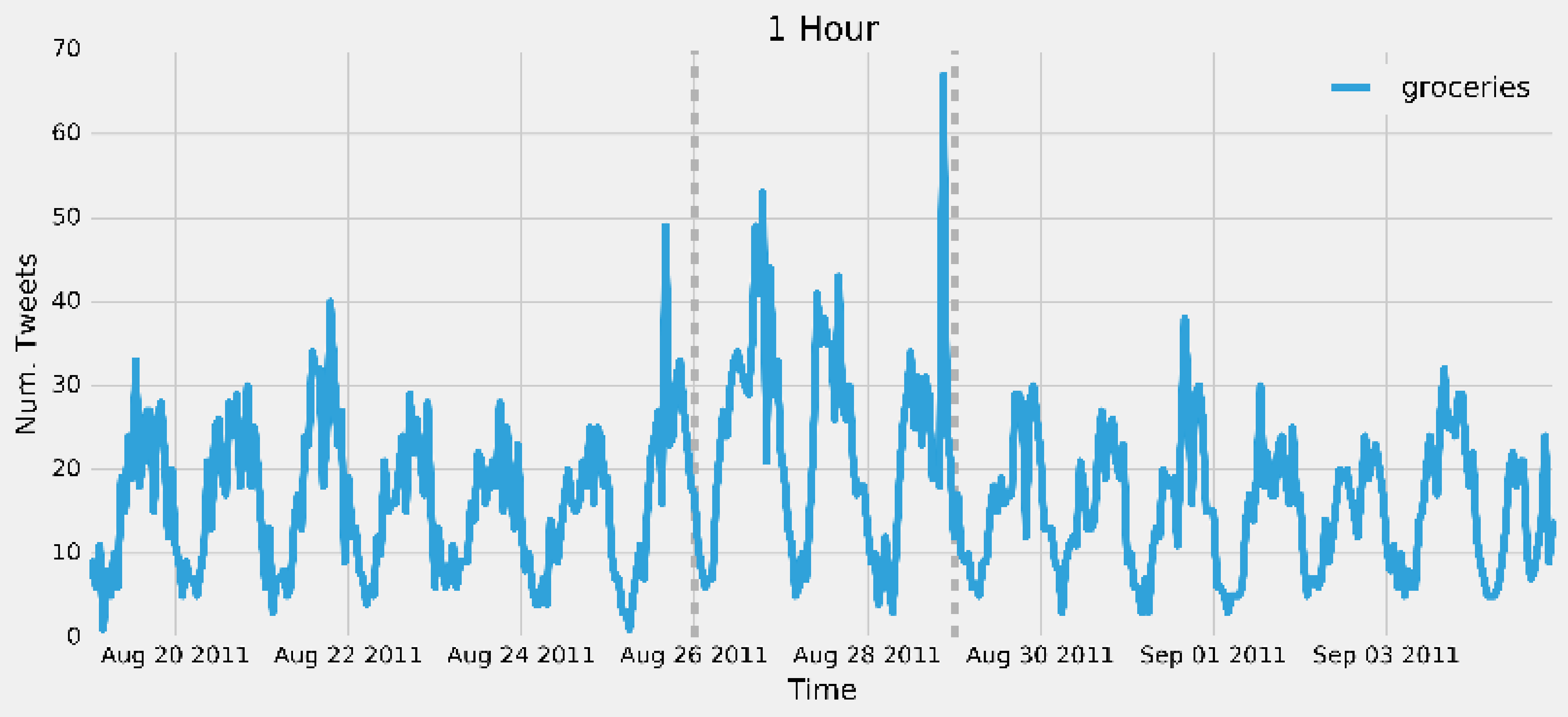

1 Hour
groceries
Num. Tweets
Time
0
10
20
30
40
50
60
70
Aug 20 2011
Aug 22 2011
Aug 24 2011
Aug 26 2011
Aug 28 2011
Aug 30 2011
Sep 01 2011
Sep 03 2011

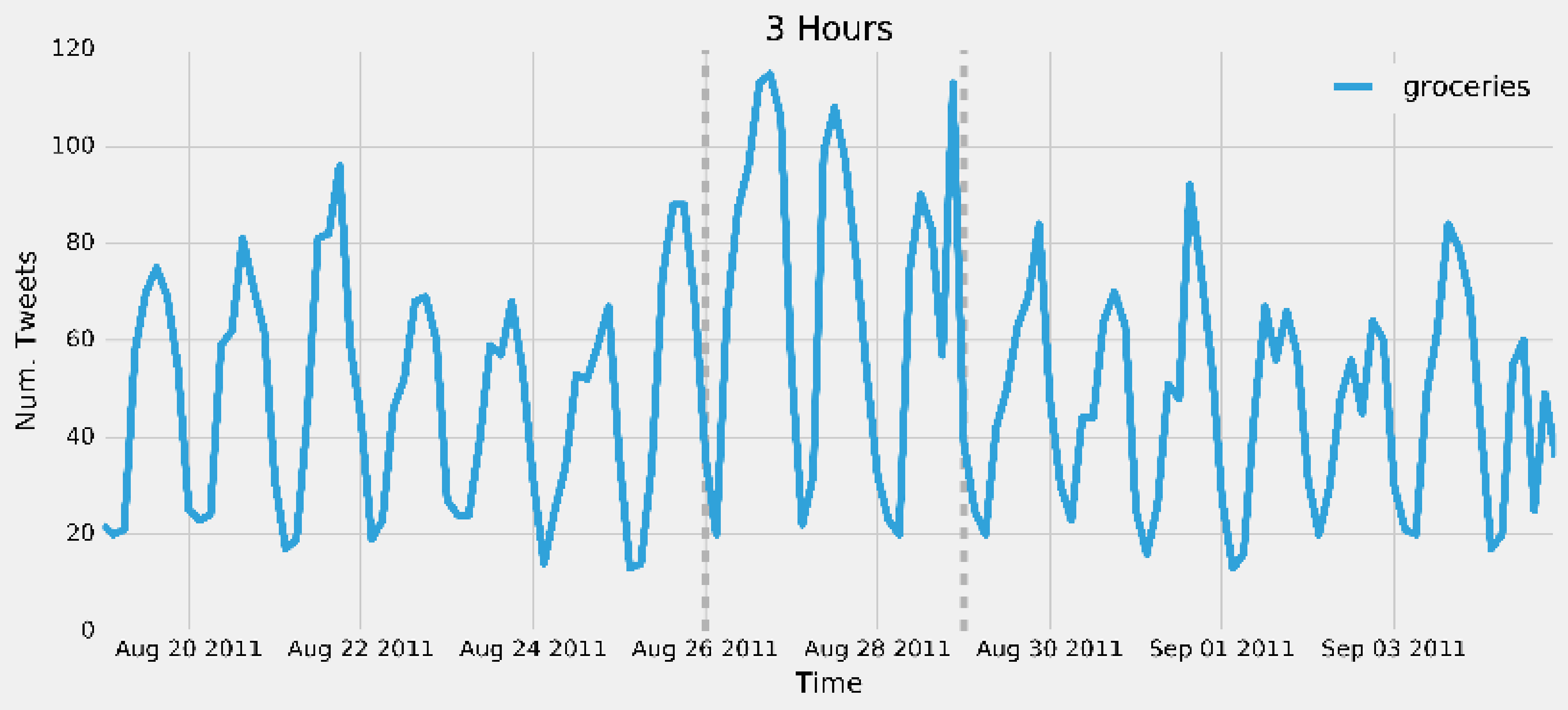
3 Hours
groceries
Num. Tweets
0
20
40
60
80
100
120
Aug 20 2011
Aug 22 2011
Aug 24 2011
Aug 26 2011
Aug 28 2011
Aug 30 2011
Sep 01 2011
Sep 03 2011
Time

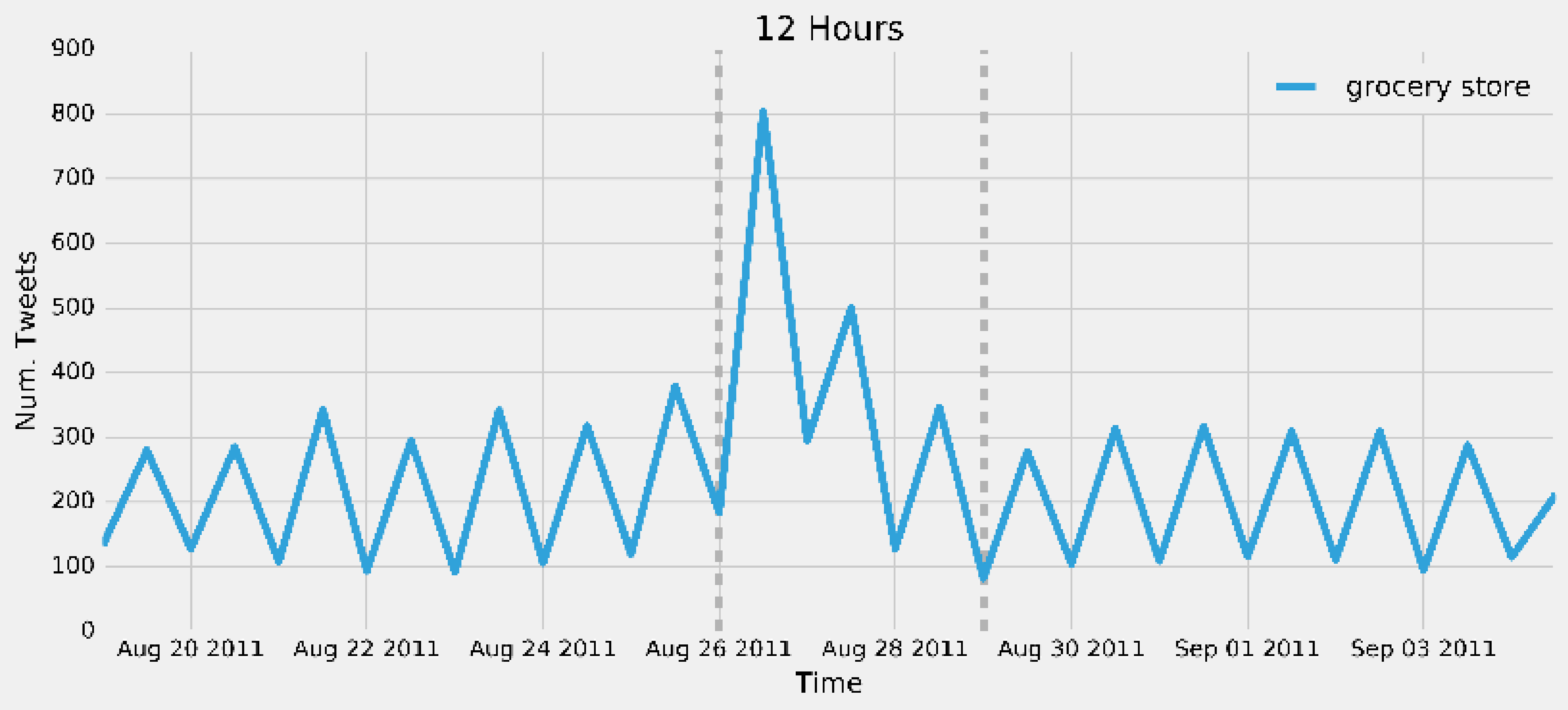
12 Hours
grocery store
Num. Tweets
900
800
700
600
500
400
300
200
100
0
Aug 20 2011
Aug 22 2011
Aug 24 2011
Aug 26 2011
Aug 28 2011
Aug 30 2011
Sep 01 2011
Sep 03 2011
Time

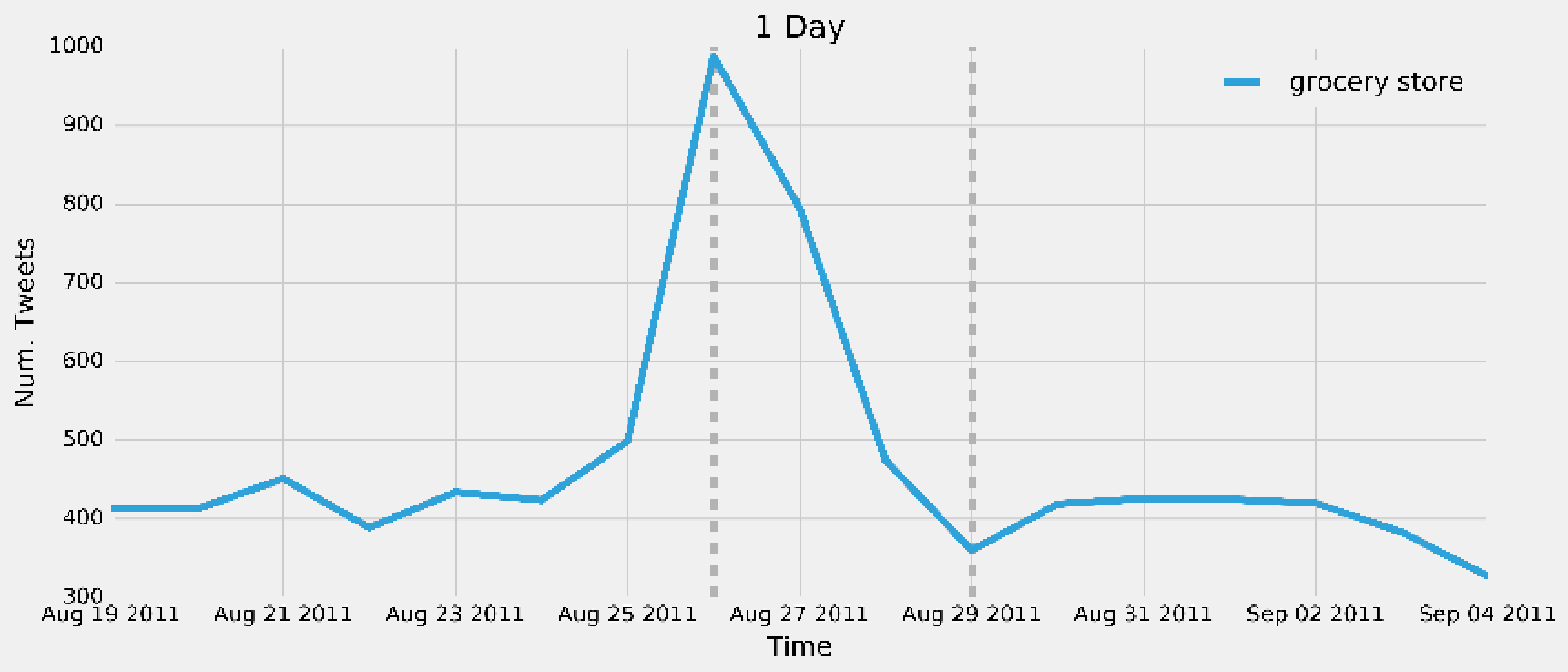

1 Day
grocery store
Num. Tweets
1000
900
800
700
600
500
400
300
Aug 19 2011
Aug 21 2011
Aug 23 2011
Aug 25 2011
Aug 27 2011
Aug 29 2011
Aug 31 2011
Sep 02 2011
Sep 04 2011
Time

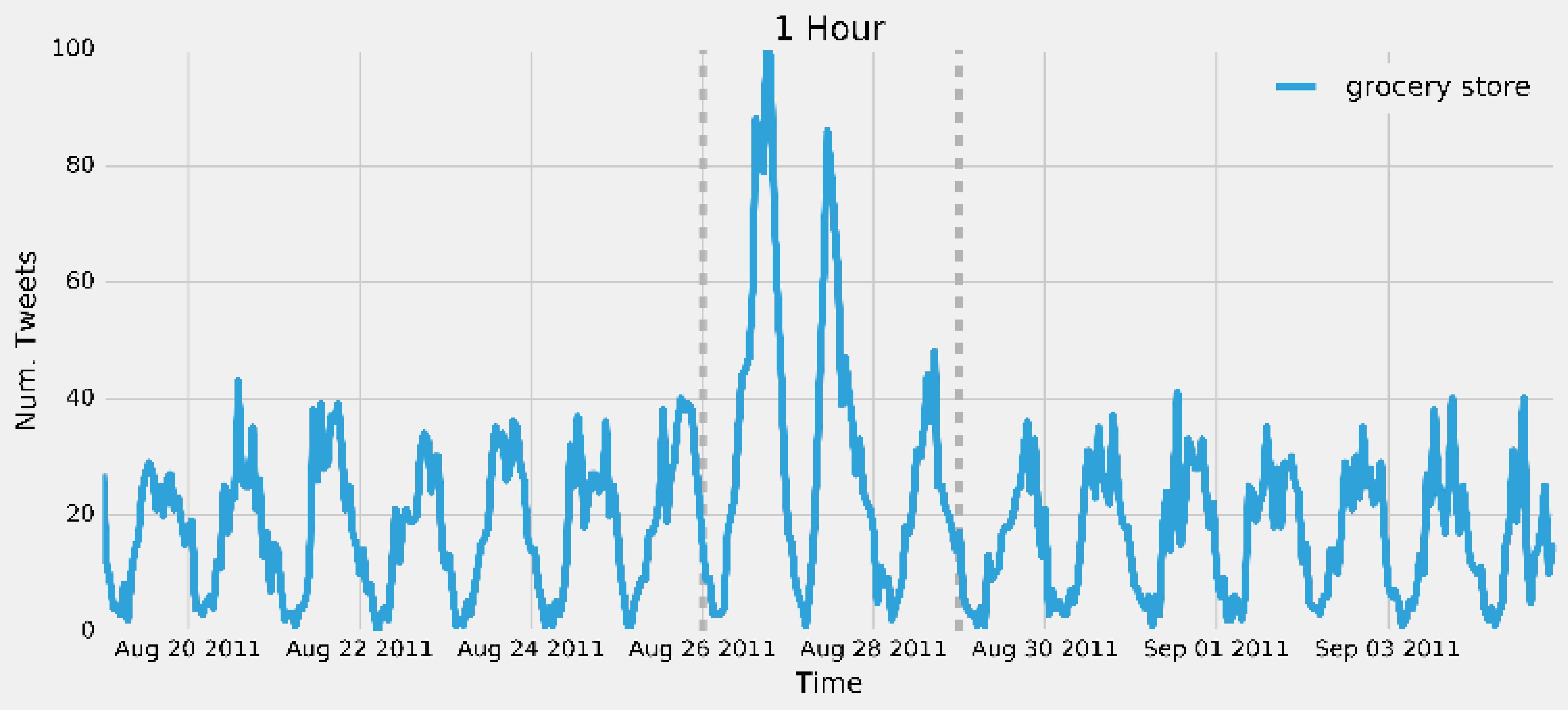

1 Hour
grocery store
Num. Tweets
100
80
60
40
20
0
Aug 20 2011
Aug 22 2011
Aug 24 2011
Aug 26 2011
Aug 28 2011
Aug 30 2011
Sep 01 2011
Sep 03 2011
Time

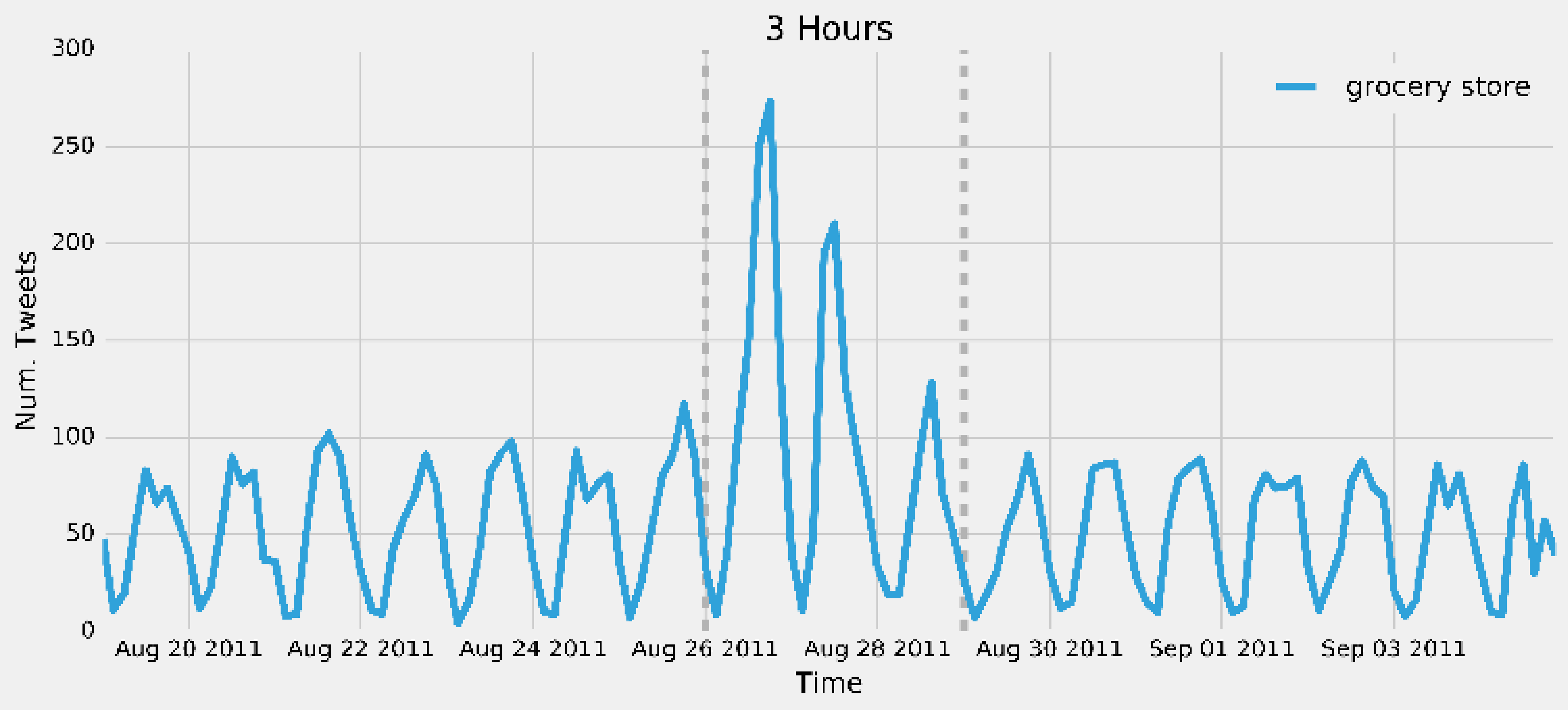
3 Hours
Num. Tweets
300
250
200
150
100
50
0
Aug 20 2011
Aug 22 2011
Aug 24 2011
Aug 26 2011
Aug 28 2011
Aug 30 2011
Sep 01 2011
Sep 03 2011
Time
grocery store

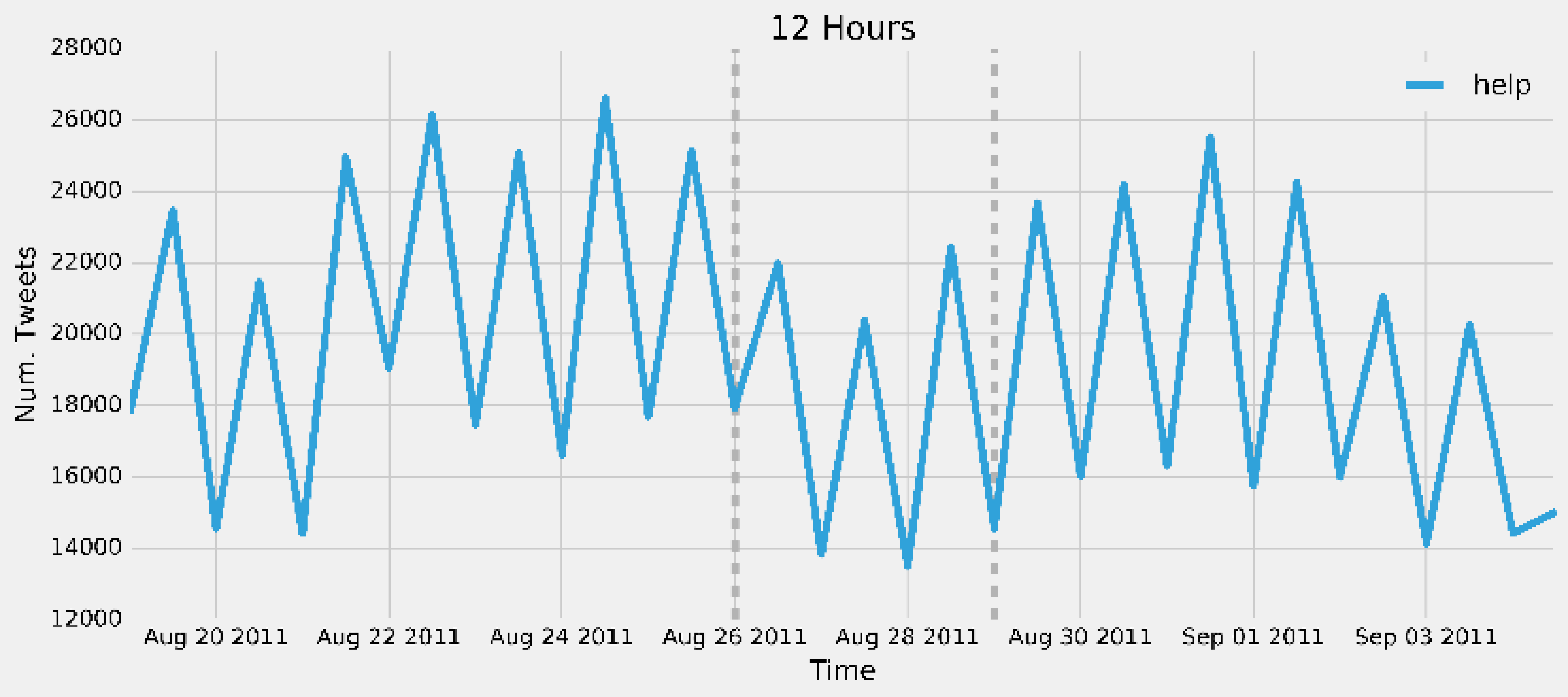
12 Hours
help
Num. Tweets
28000
26000
24000
22000
20000
18000
16000
14000
12000
Aug 20 2011
Aug 22 2011
Aug 24 2011
Aug 26 2011
Aug 28 2011
Aug 30 2011
Sep 01 2011
Sep 03 2011
Time

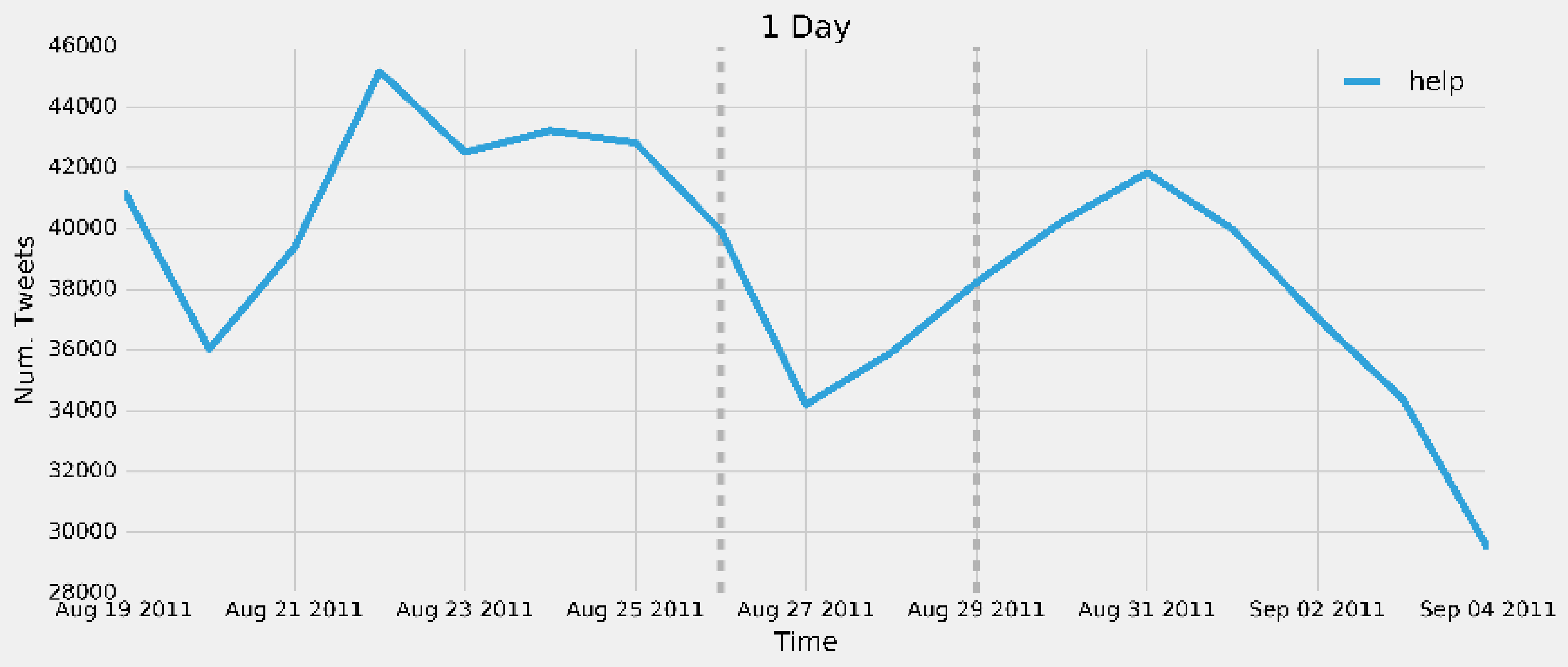

1 Day
help
Num. Tweets
46000
44000
42000
40000
38000
36000
34000
32000
30000
28000
Aug 19 2011
Aug 21 2011
Aug 23 2011
Aug 25 2011
Aug 27 2011
Aug 29 2011
Aug 31 2011
Sep 02 2011
Sep 04 2011
Time

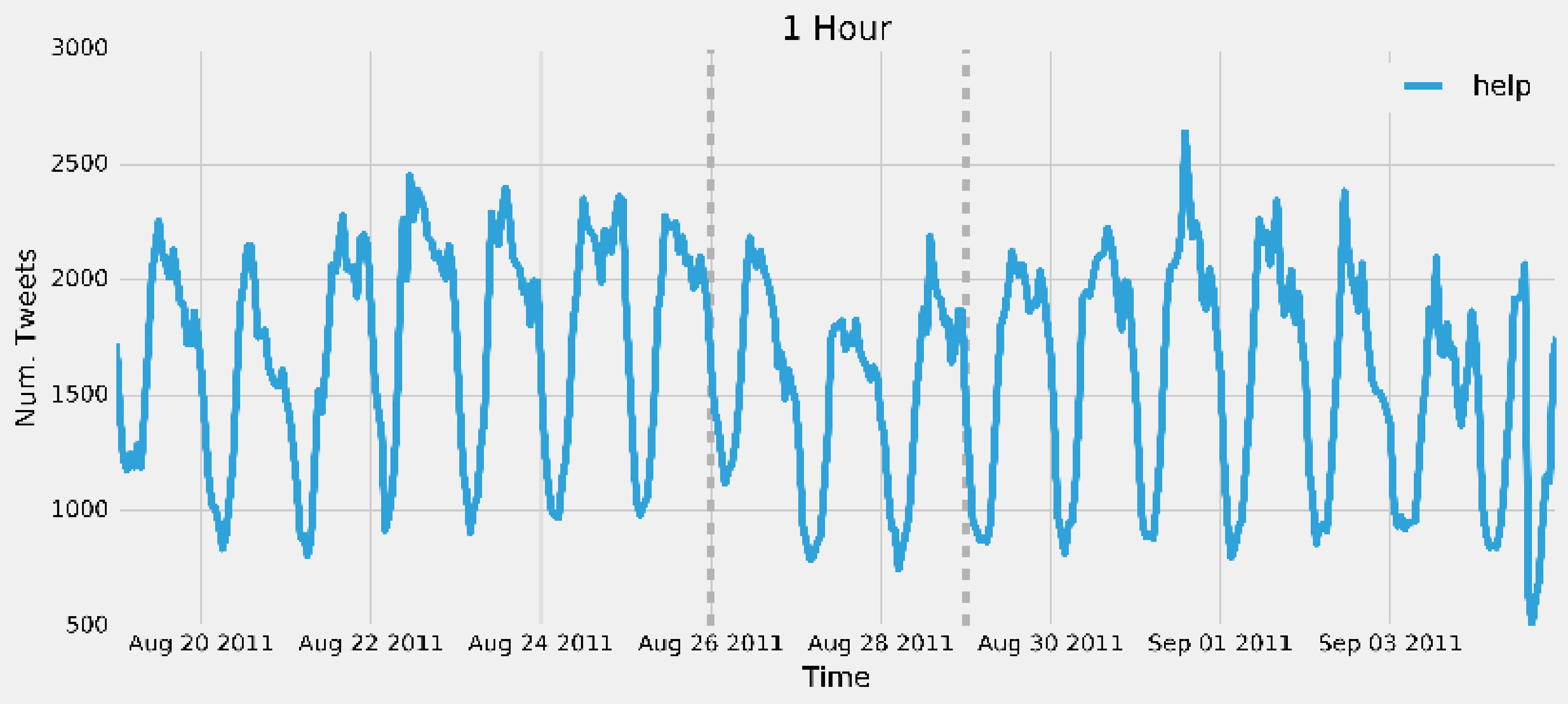
1 Hour
help
3000
2500
2000
1500
1000
500
Num. Tweets
Aug 20 2011
Aug 22 2011
Aug 24 2011
Aug 26 2011
Aug 28 2011
Aug 30 2011
Sep 01 2011
Sep 03 2011
Time

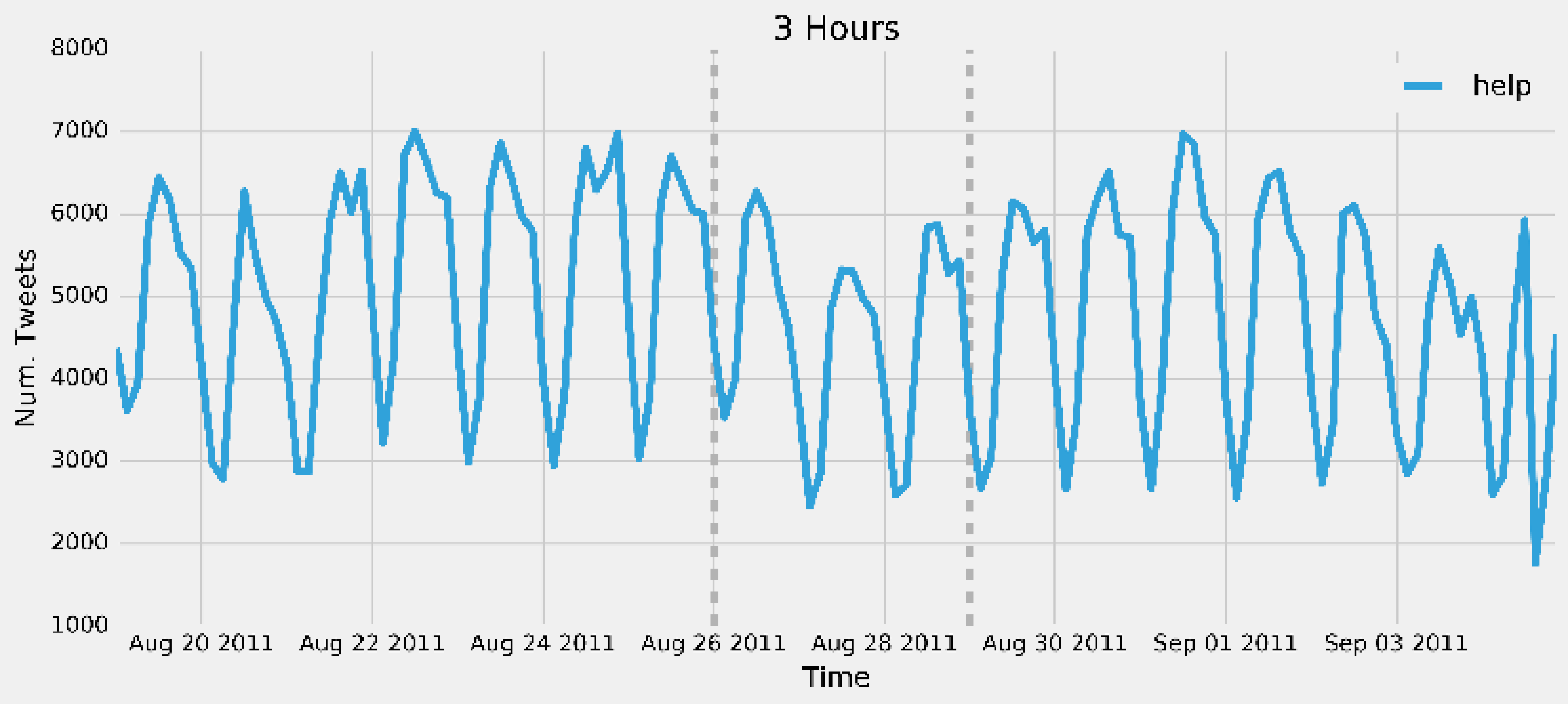

3 Hours
help
8000
7000
6000
5000
4000
3000
2000
1000
Num. Tweets
Aug 20 2011
Aug 22 2011
Aug 24 2011
Aug 26 2011
Aug 28 2011
Aug 30 2011
Sep 01 2011
Sep 03 2011
Time

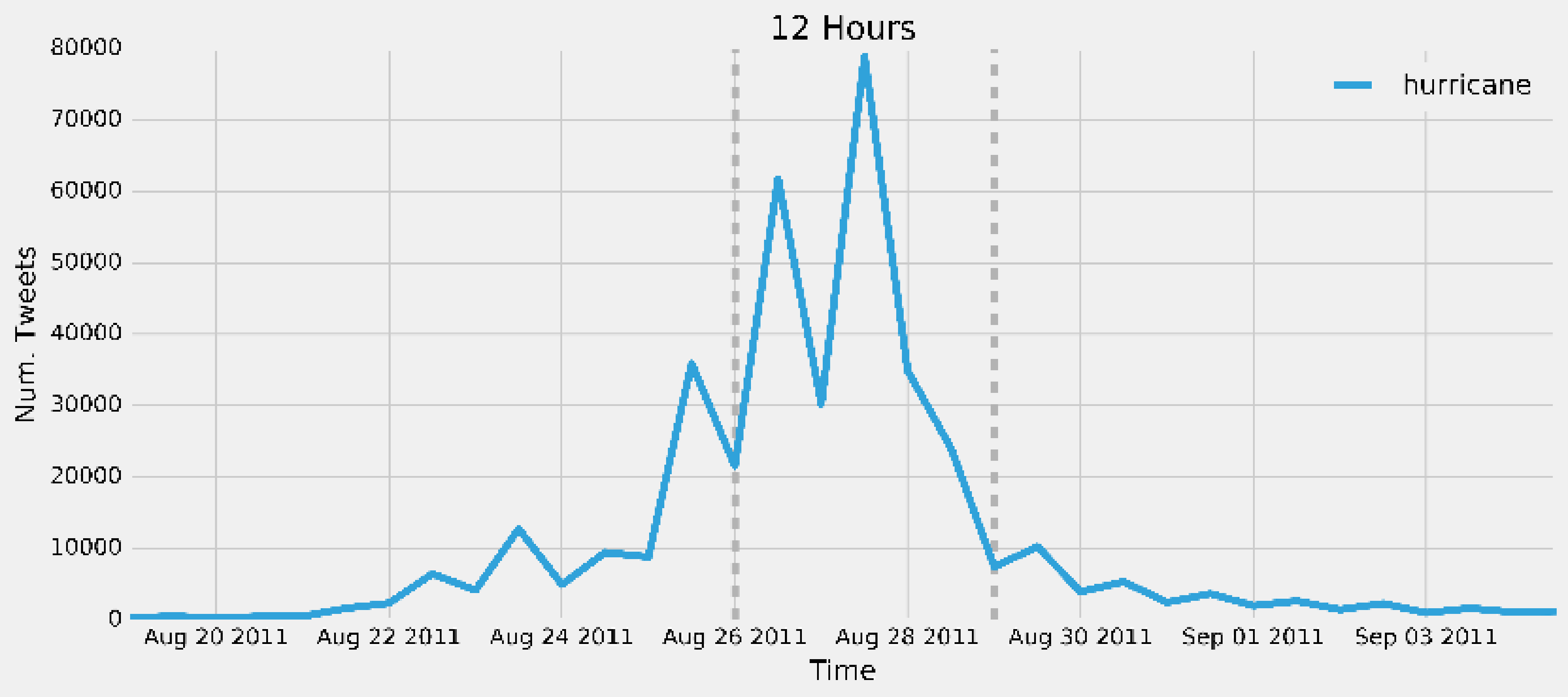

12 Hours
80000
70000
60000
50000
40000
30000
20000
10000
0
Num. Tweets
hurricane
Aug 20 2011
Aug 22 2011
Aug 24 2011
Aug 26 2011
Aug 28 2011
Aug 30 2011
Sep 01 2011
Sep 03 2011
Time

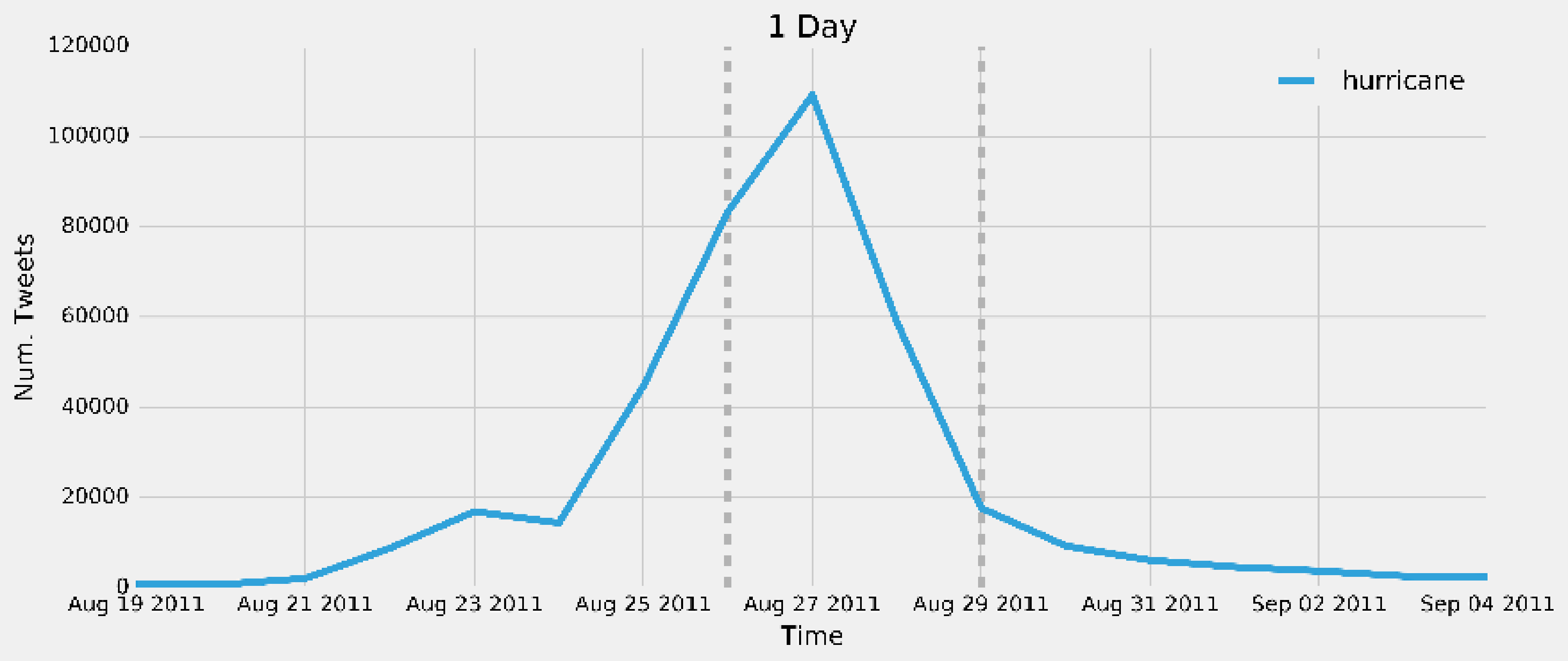
1 Day
hurricane
Num. Tweets
0
20000
40000
60000
80000
100000
120000
Aug 19 2011
Aug 21 2011
Aug 23 2011
Aug 25 2011
Aug 27 2011
Aug 29 2011
Aug 31 2011
Sep 02 2011
Sep 04 2011
Time

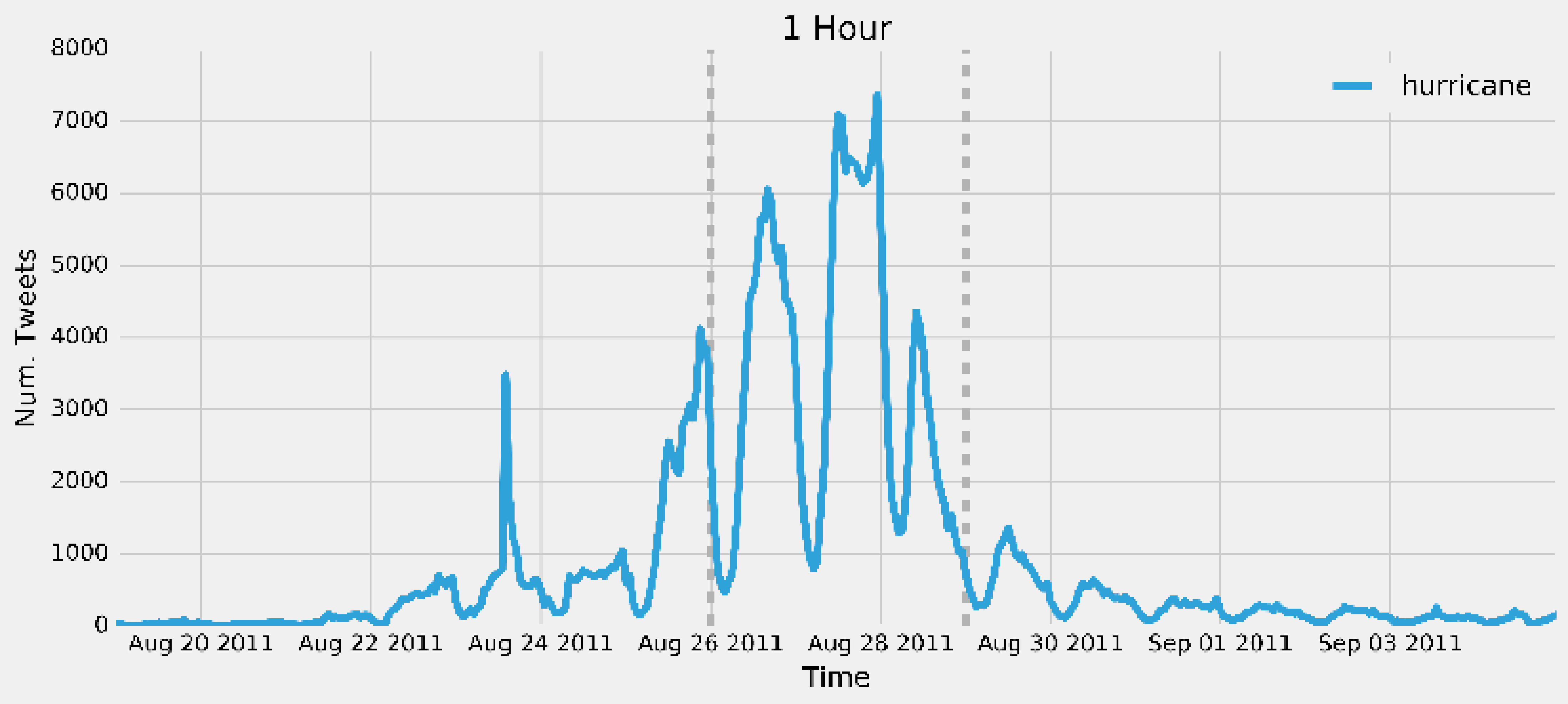
1 Hour
8000
7000
6000
5000
4000
3000
2000
1000
0
Num. Tweets
hurricane
Aug 20 2011
Aug 22 2011
Aug 24 2011
Aug 26 2011
Aug 28 2011
Aug 30 2011
Sep 01 2011
Sep 03 2011
Time

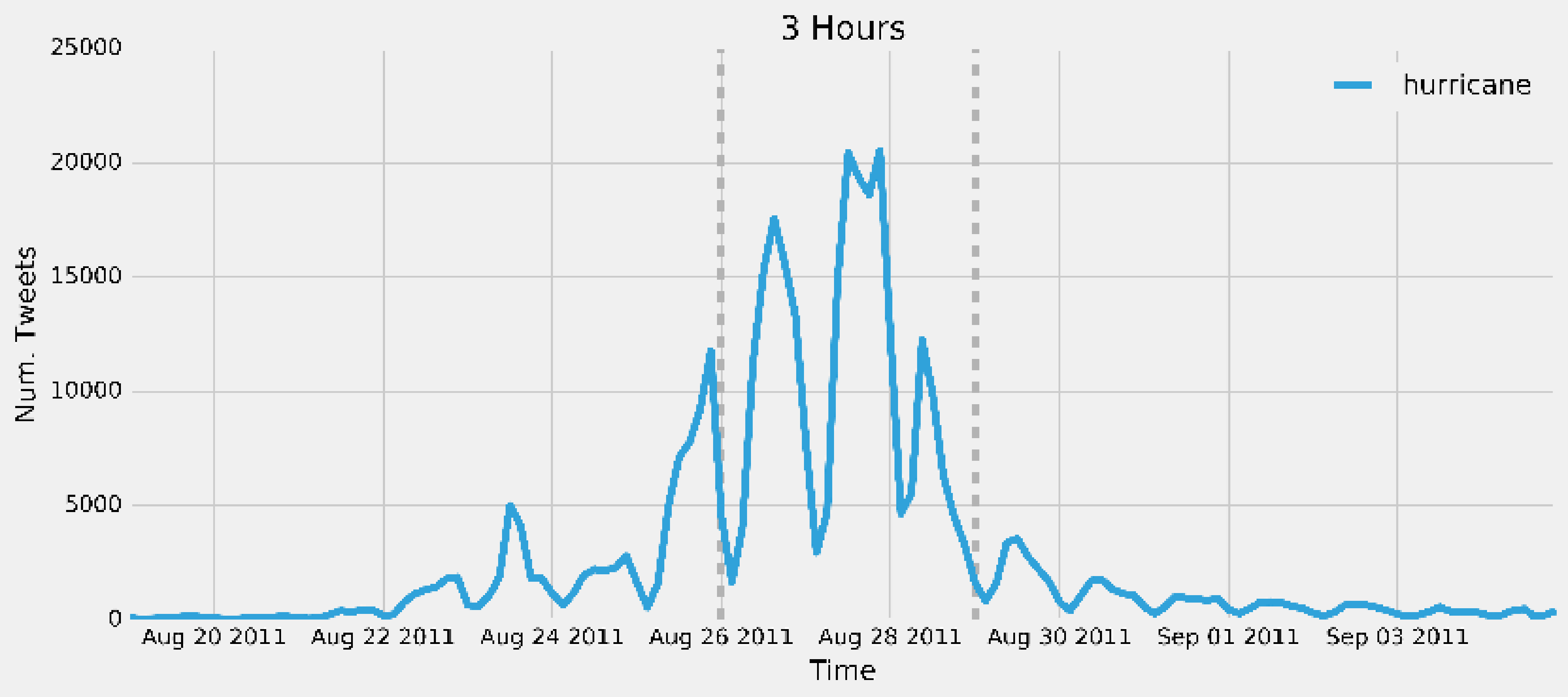

3 Hours
25000
20000
15000
10000
5000
0
Num. Tweets
Aug 20 2011
Aug 22 2011
Aug 24 2011
Aug 26 2011
Aug 28 2011
Aug 30 2011
Sep 01 2011
Sep 03 2011
Time
hurricane

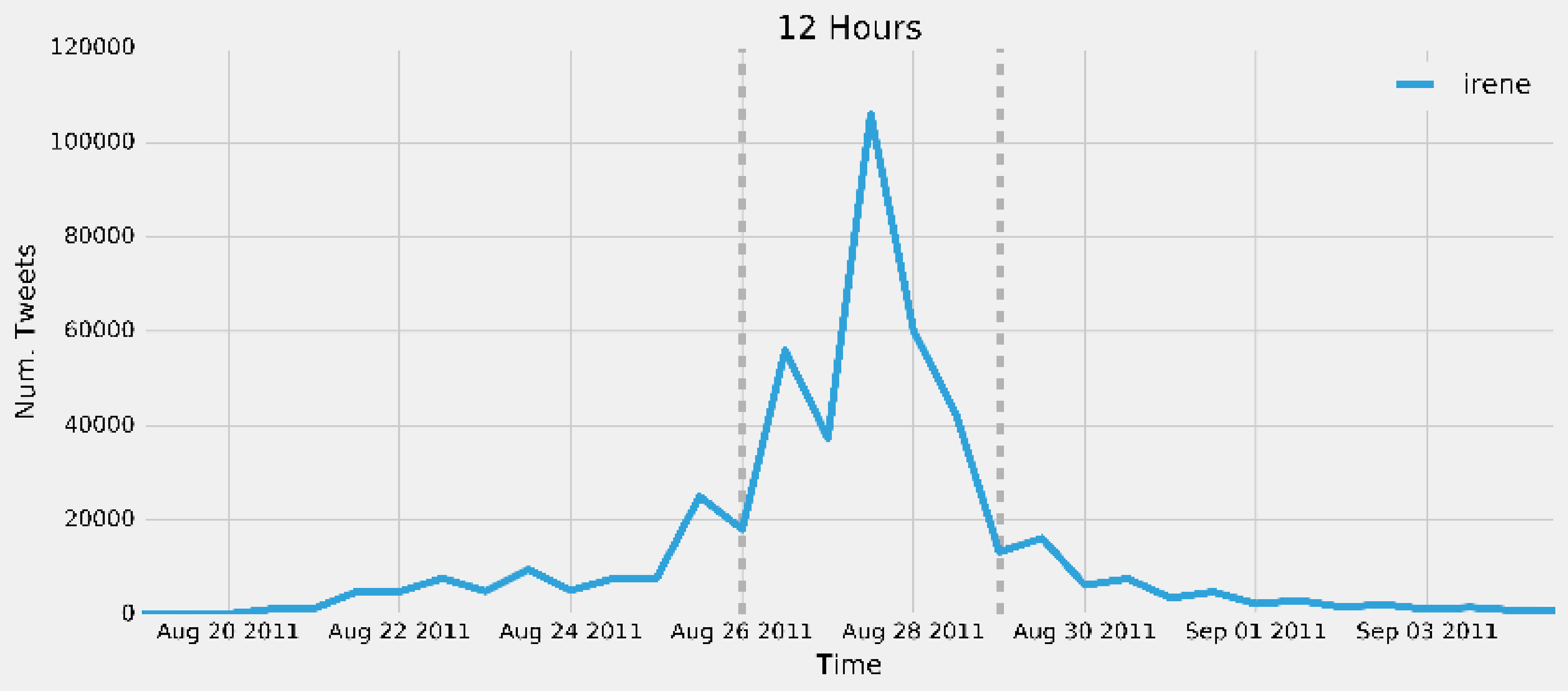
12 Hours
irene
Num. Tweets
0
20000
40000
60000
80000
100000
120000
Aug 20 2011
Aug 22 2011
Aug 24 2011
Aug 26 2011
Aug 28 2011
Aug 30 2011
Sep 01 2011
Sep 03 2011
Time

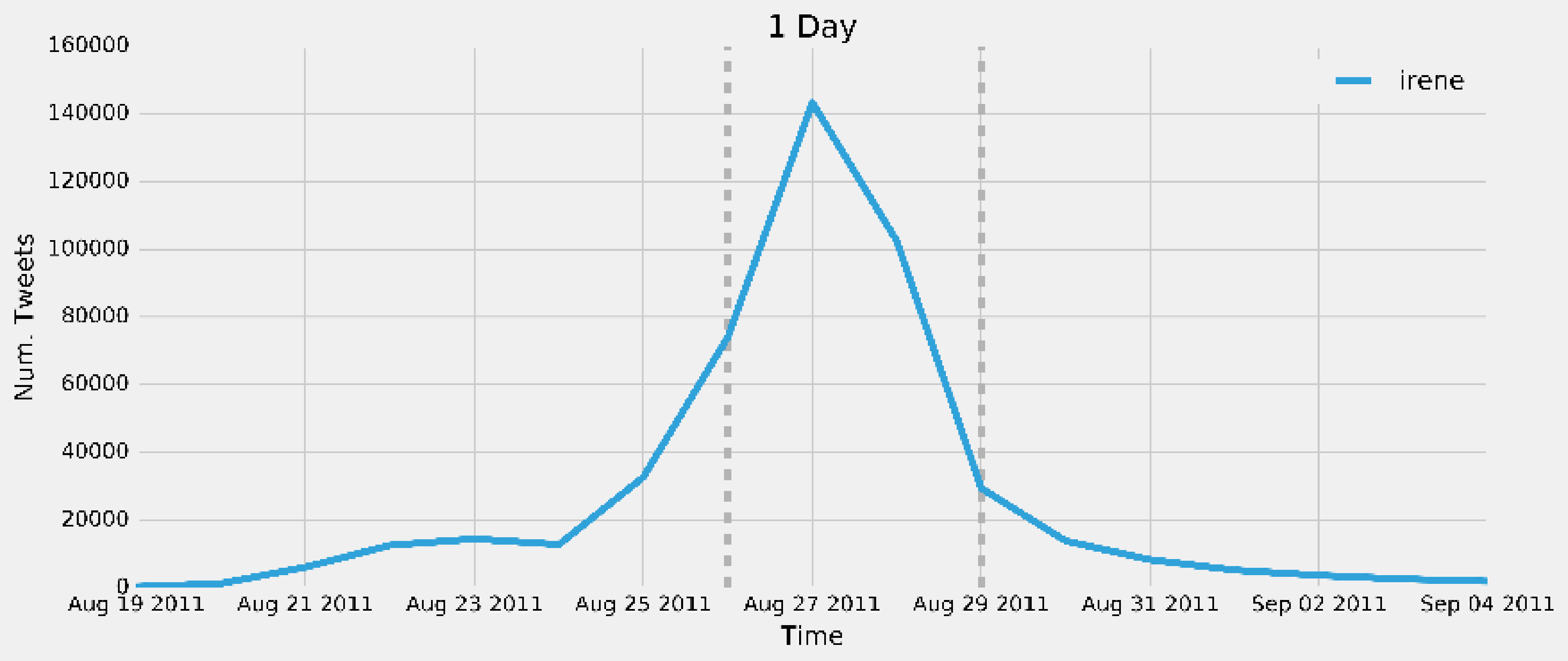
1 Day
irene
Num. Tweets
0
20000
40000
60000
80000
100000
120000
140000
160000
Aug 19 2011
Aug 21 2011
Aug 23 2011
Aug 25 2011
Aug 27 2011
Aug 29 2011
Aug 31 2011
Sep 02 2011
Sep 04 2011
Time

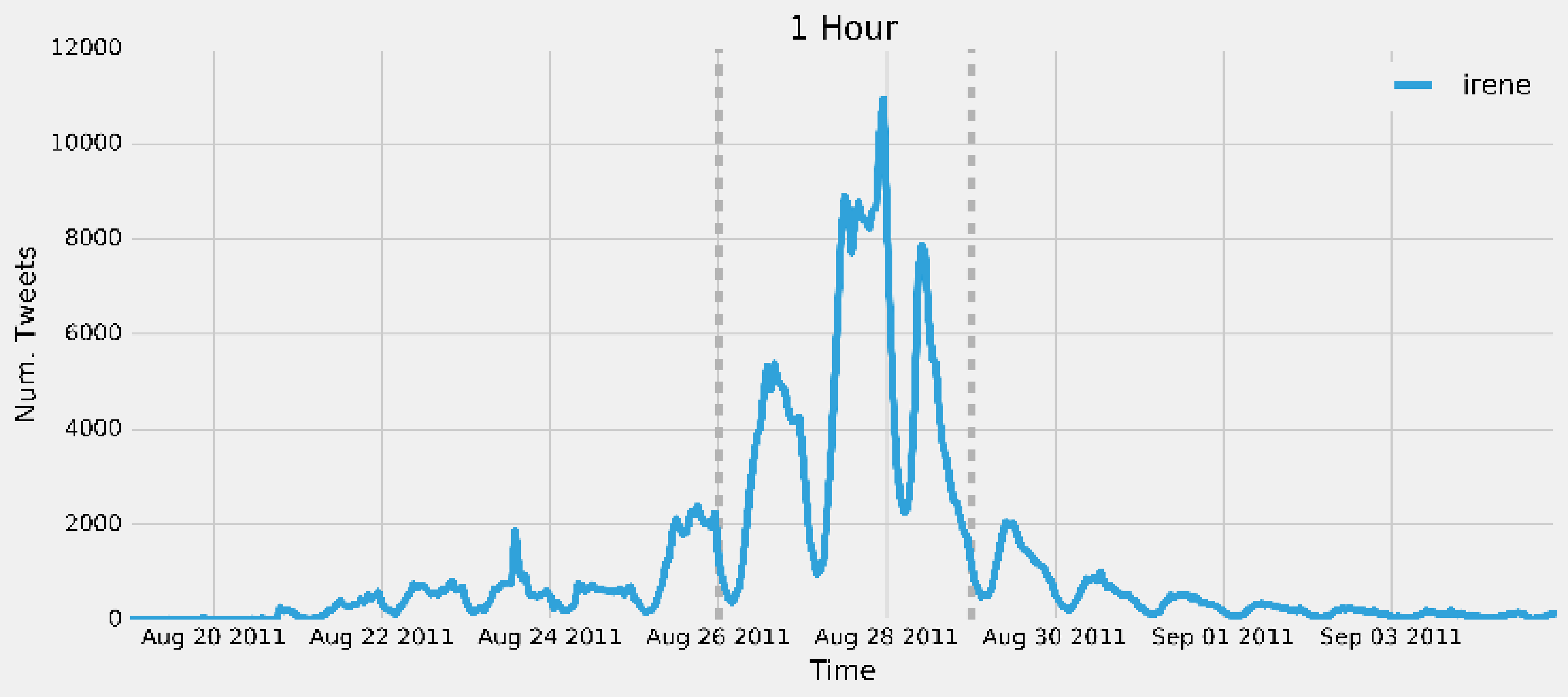

1 Hour
irene
Num. Tweets
12000
10000
8000
6000
4000
2000
0
Aug 20 2011
Aug 22 2011
Aug 24 2011
Aug 26 2011
Aug 28 2011
Aug 30 2011
Sep 01 2011
Sep 03 2011
Time

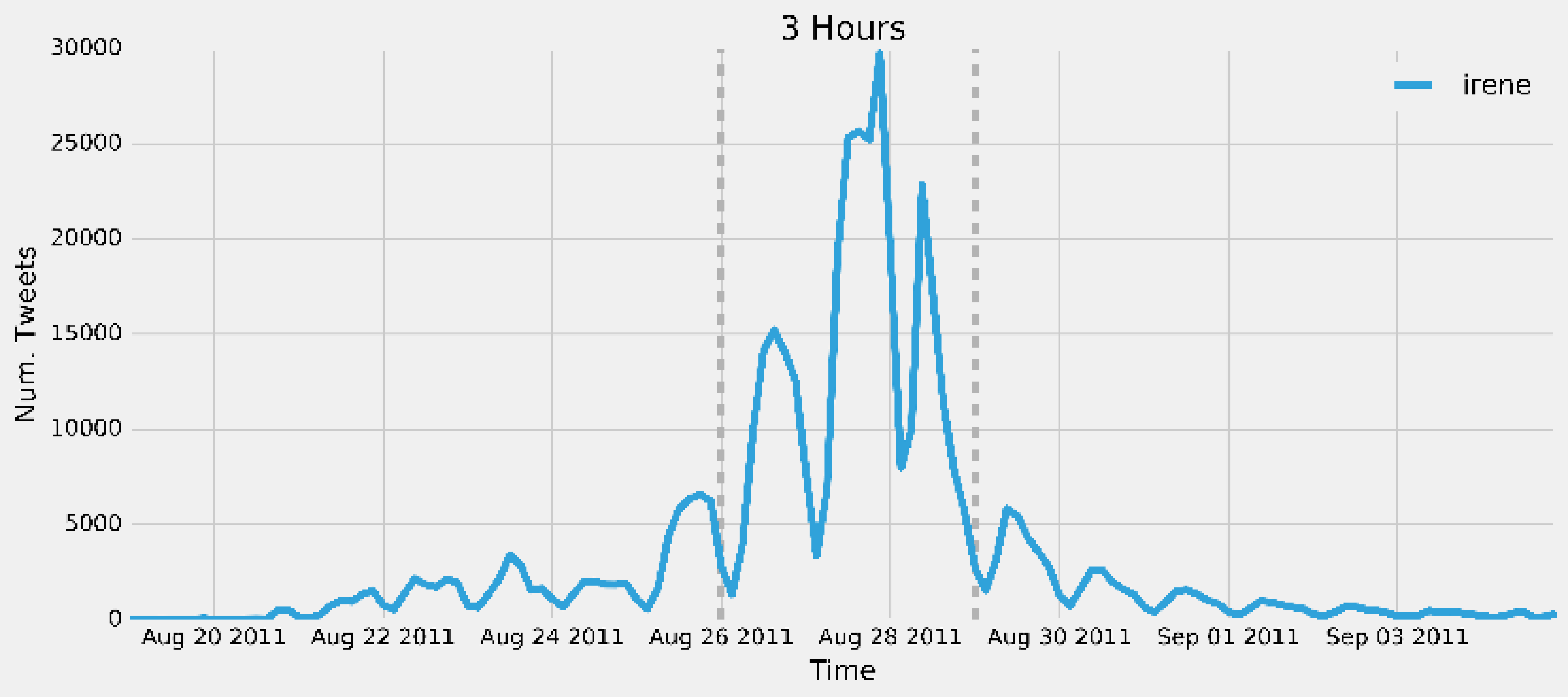

3 Hours
irene
Num. Tweets
0
5000
10000
15000
20000
25000
30000
Aug 20 2011
Aug 22 2011
Aug 24 2011
Aug 26 2011
Aug 28 2011
Aug 30 2011
Sep 01 2011
Sep 03 2011
Time

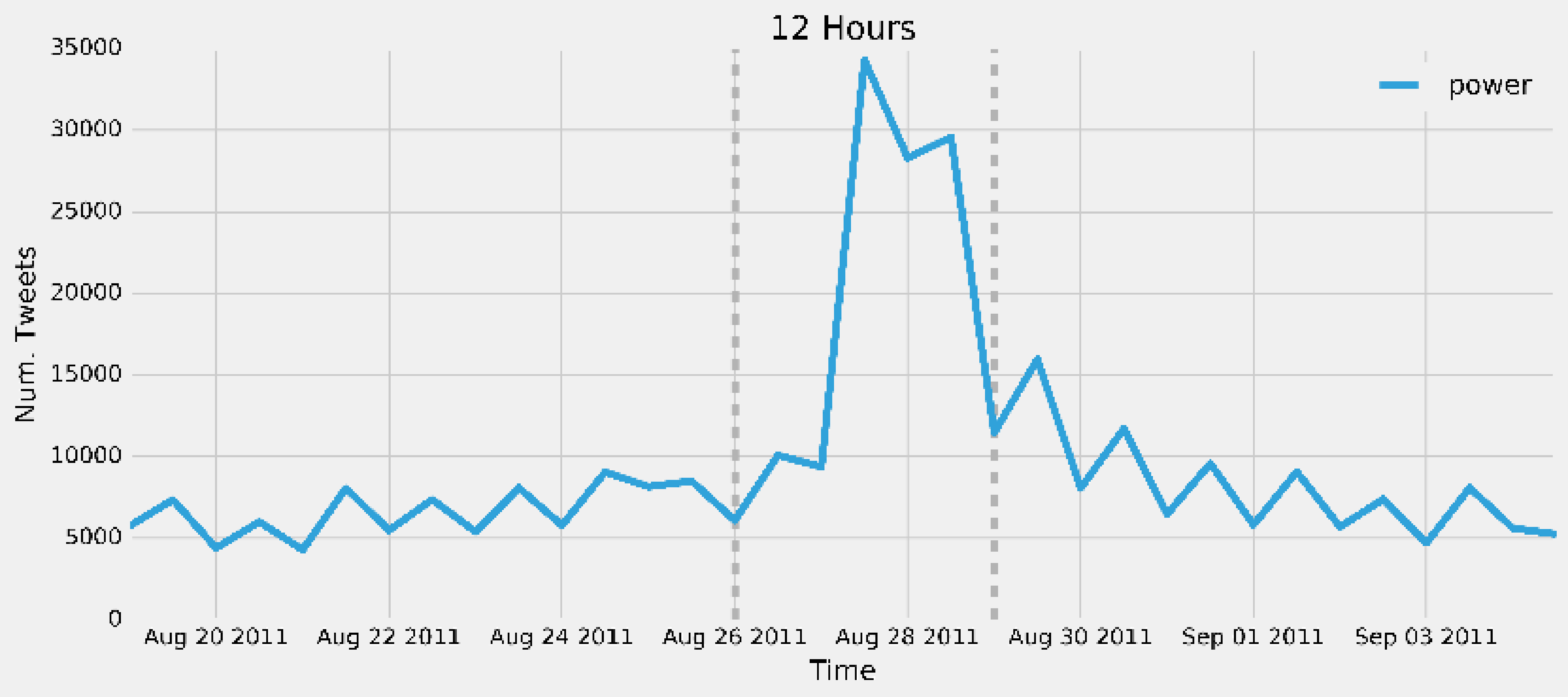

12 Hours
power
35000
30000
25000
20000
15000
10000
5000
0
Num. Tweets
Aug 20 2011
Aug 22 2011
Aug 24 2011
Aug 26 2011
Aug 28 2011
Aug 30 2011
Sep 01 2011
Sep 03 2011
Time

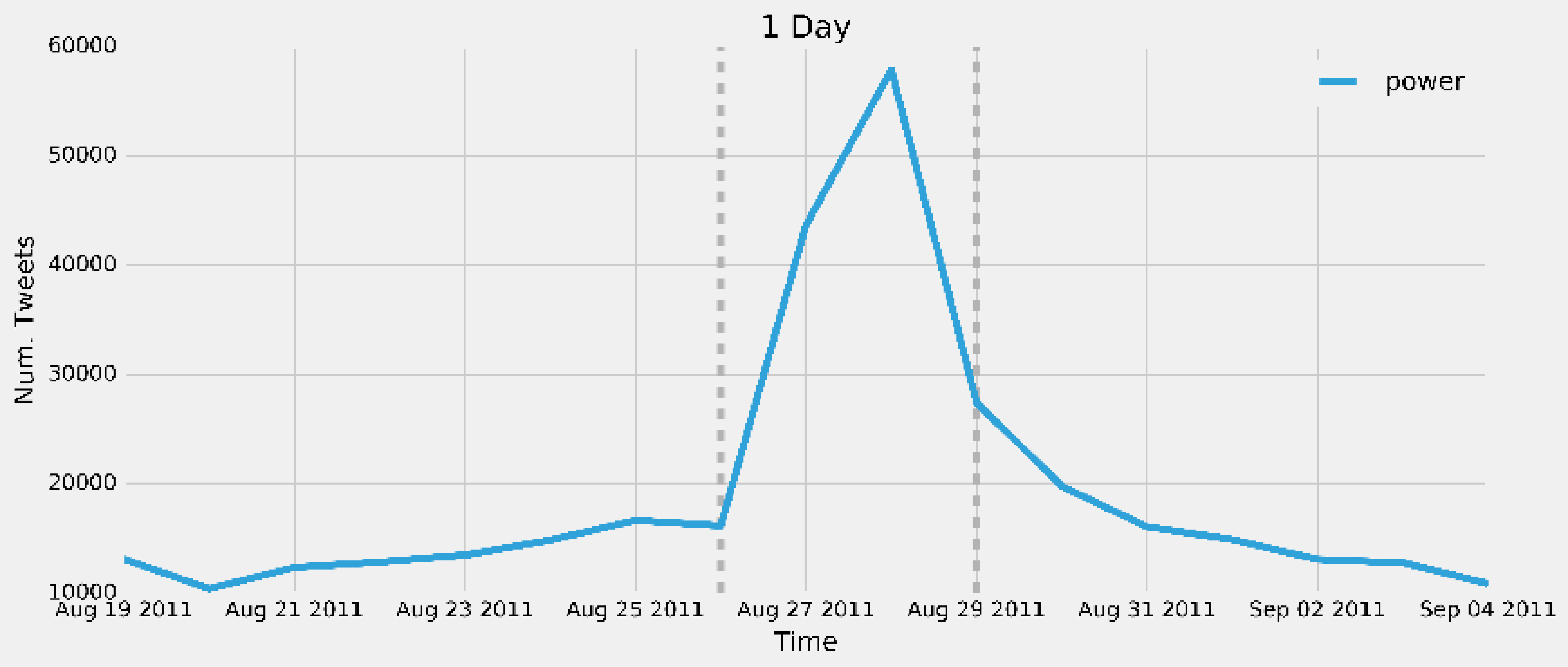

1 Day
power
Num. Tweets
60000
50000
40000
30000
20000
10000
Aug 19 2011
Aug 21 2011
Aug 23 2011
Aug 25 2011
Aug 27 2011
Aug 29 2011
Aug 31 2011
Sep 02 2011
Sep 04 2011
Time

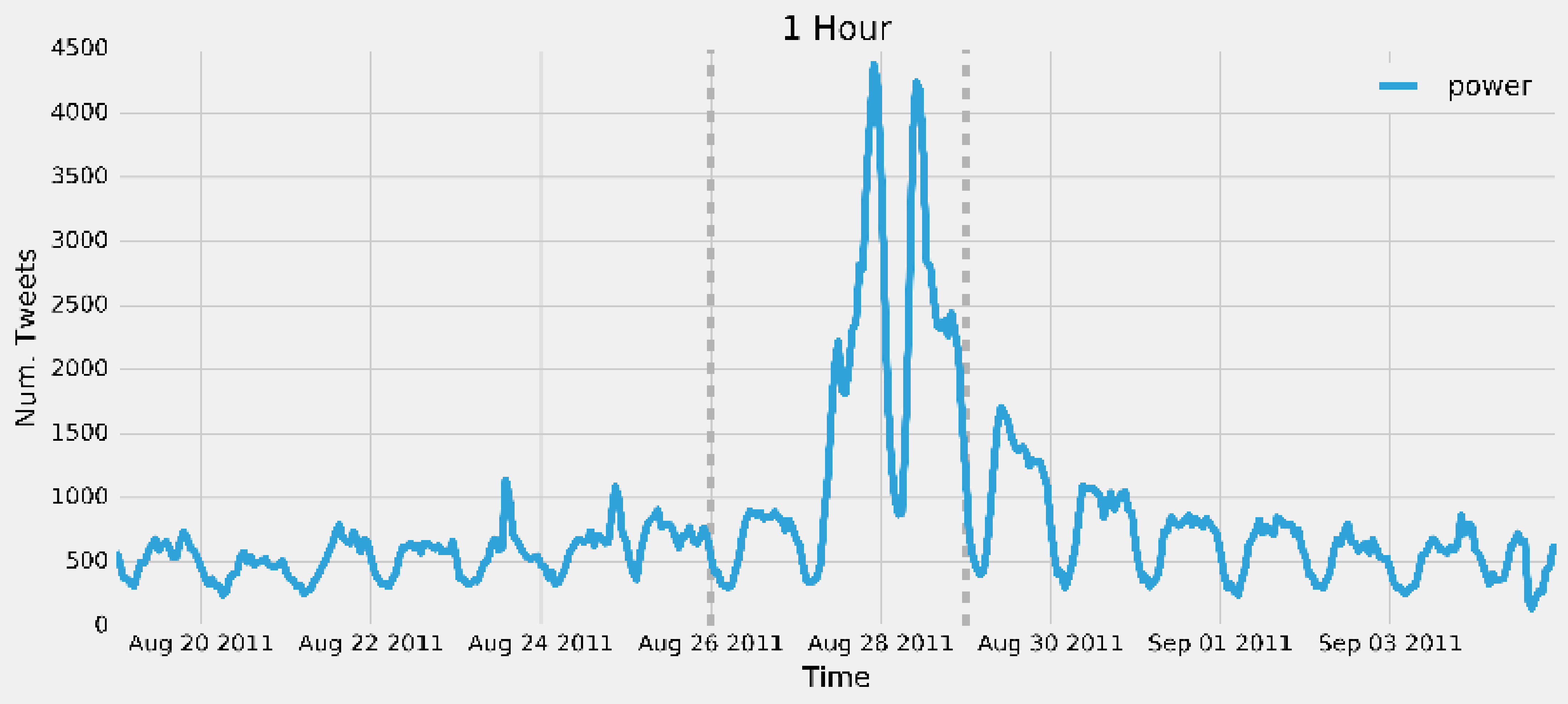

1 Hour
power
Num. Tweets
4500
4000
3500
3000
2500
2000
1500
1000
500
0
Aug 20 2011
Aug 22 2011
Aug 24 2011
Aug 26 2011
Aug 28 2011
Aug 30 2011
Sep 01 2011
Sep 03 2011
Time

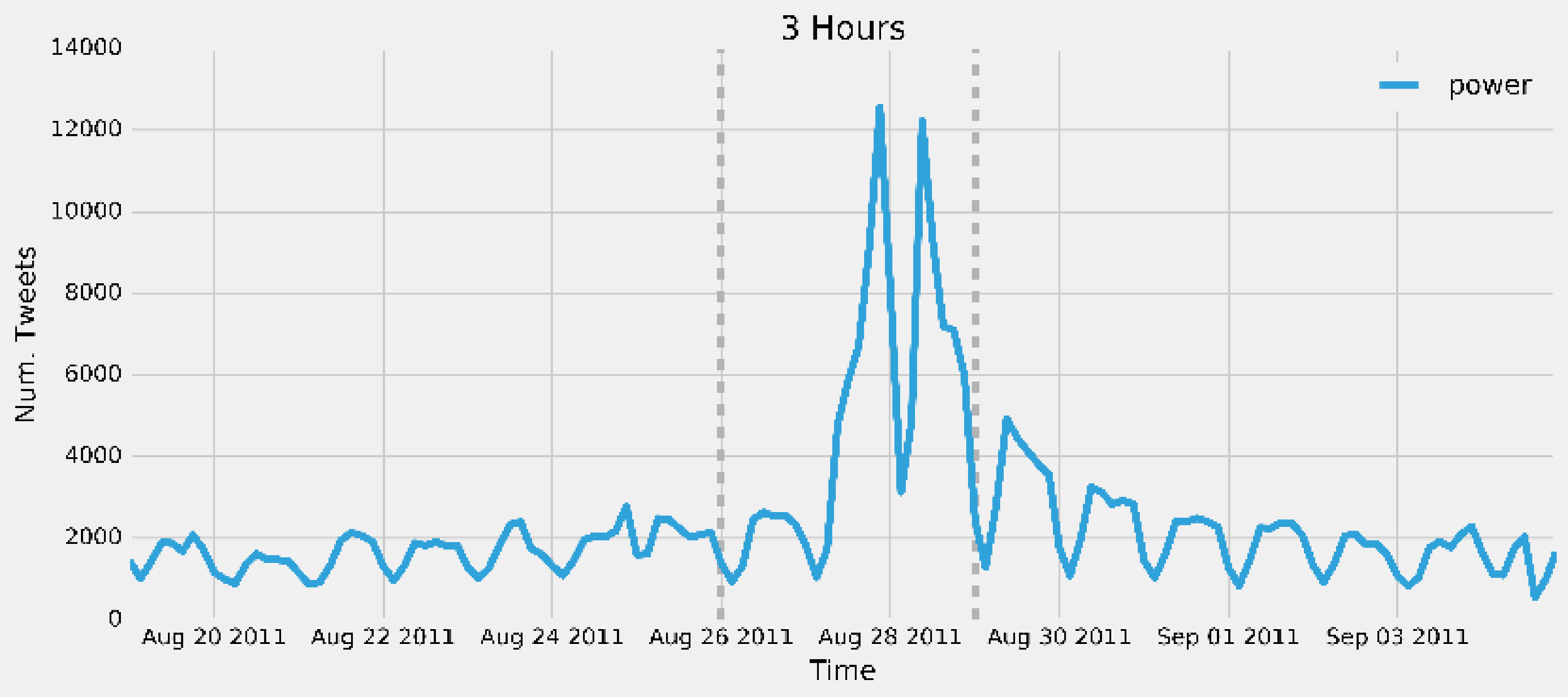
3 Hours
power
Num. Tweets
Time
0
2000
4000
6000
8000
10000
12000
14000
Aug 20 2011
Aug 22 2011
Aug 24 2011
Aug 26 2011
Aug 28 2011
Aug 30 2011
Sep 01 2011
Sep 03 2011

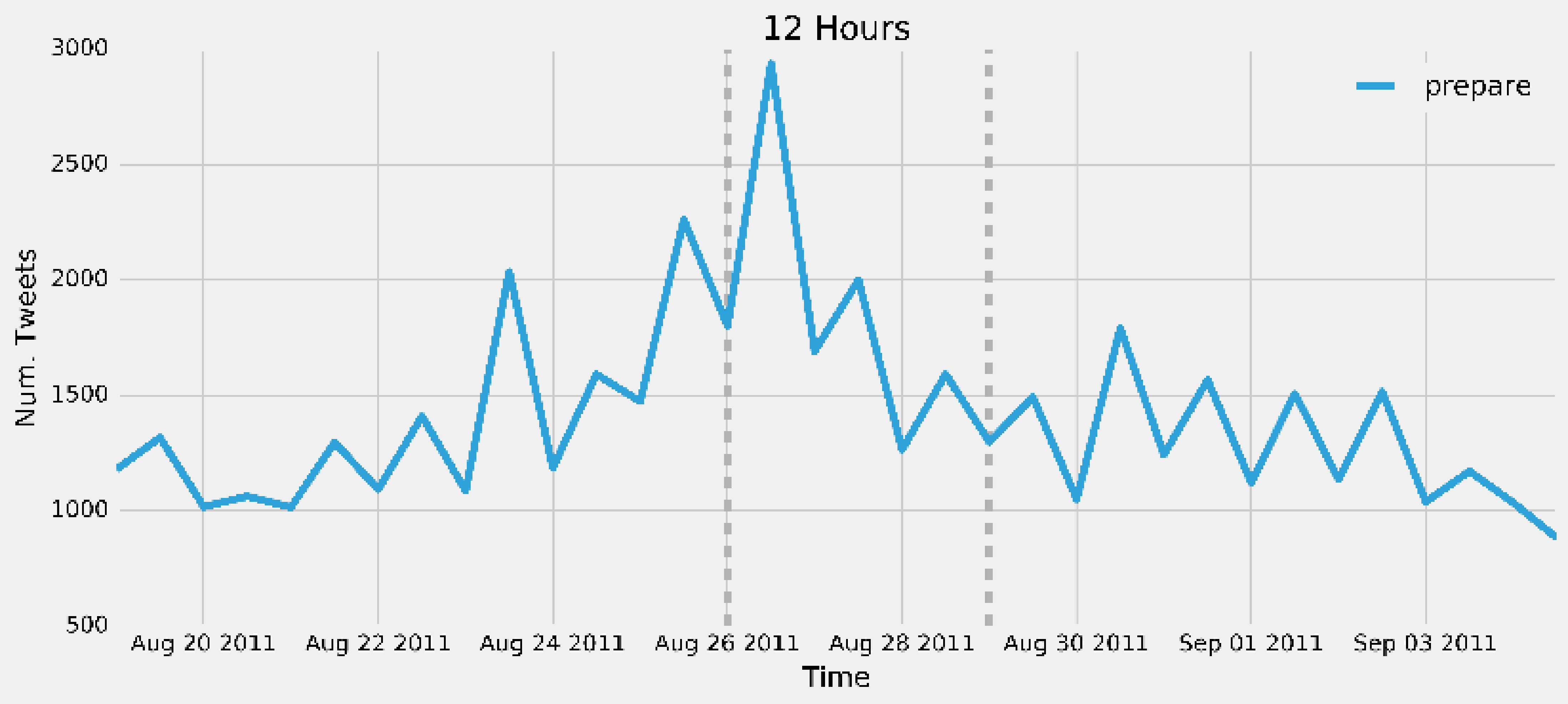

12 Hours
3000
2500
2000
1500
1000
500
Num. Tweets
prepare
Aug 20 2011
Aug 22 2011
Aug 24 2011
Aug 26 2011
Aug 28 2011
Aug 30 2011
Sep 01 2011
Sep 03 2011
Time

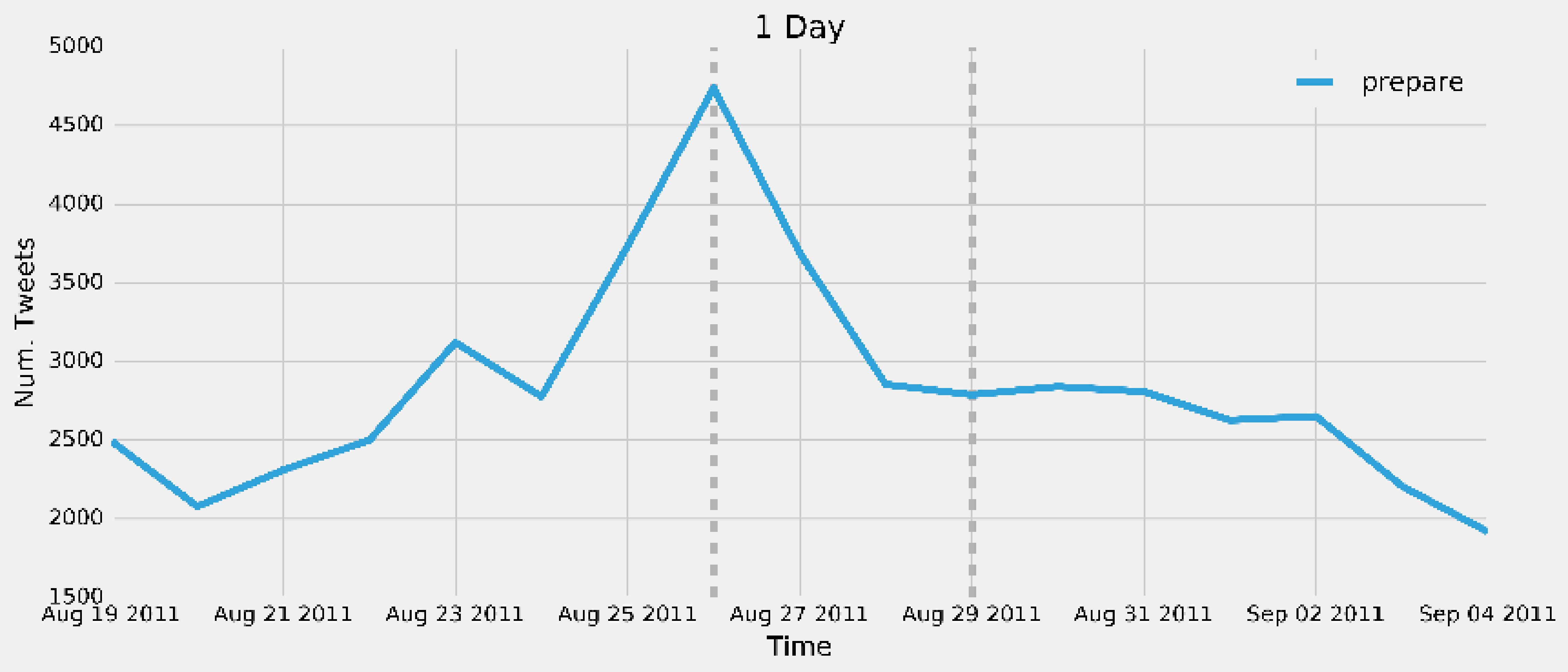
1 Day
prepare
Num. Tweets
5000
4500
4000
3500
3000
2500
2000
1500
Aug 19 2011
Aug 21 2011
Aug 23 2011
Aug 25 2011
Aug 27 2011
Aug 29 2011
Aug 31 2011
Sep 02 2011
Sep 04 2011
Time

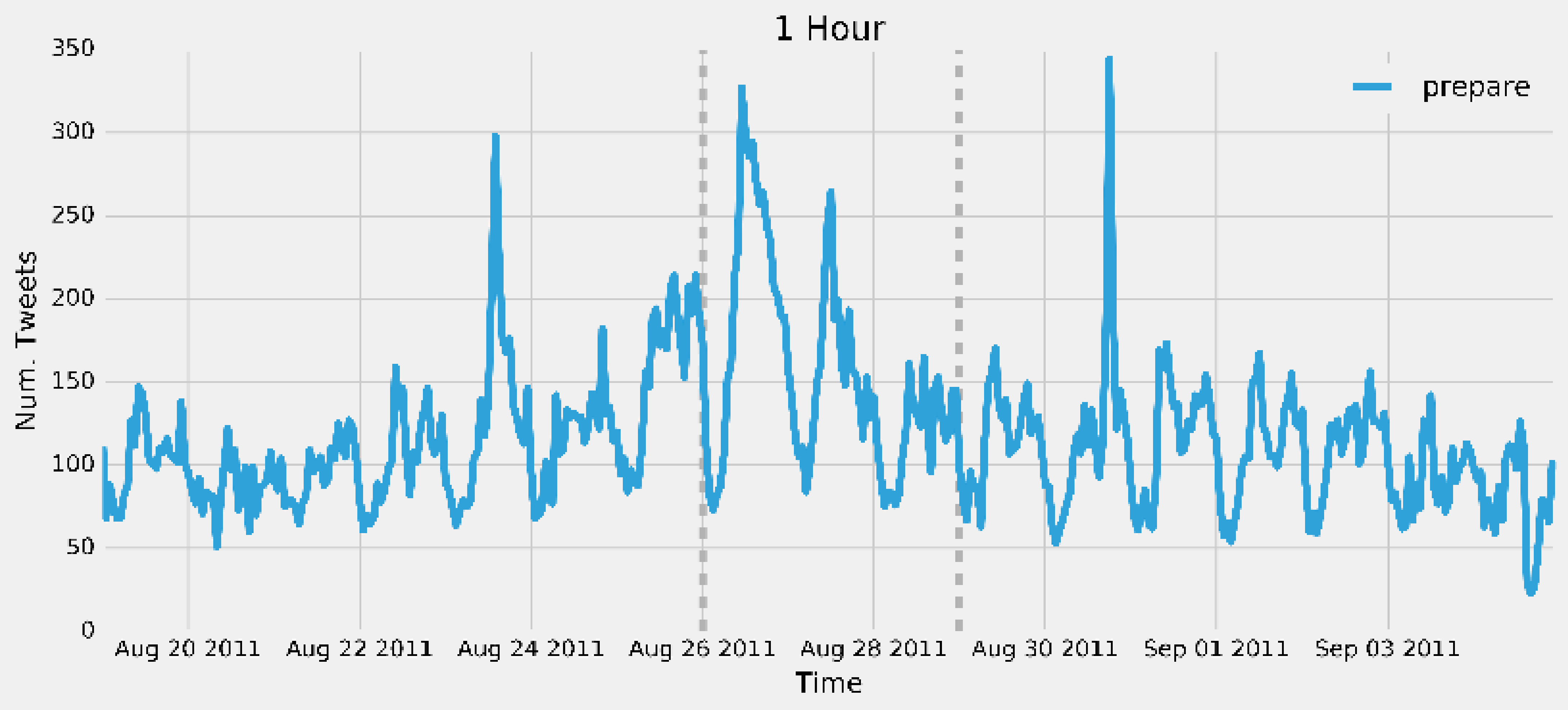
1 Hour
prepare
Num. Tweets
0
50
100
150
200
250
300
350
Aug 20 2011
Aug 22 2011
Aug 24 2011
Aug 26 2011
Aug 28 2011
Aug 30 2011
Sep 01 2011
Sep 03 2011
Time

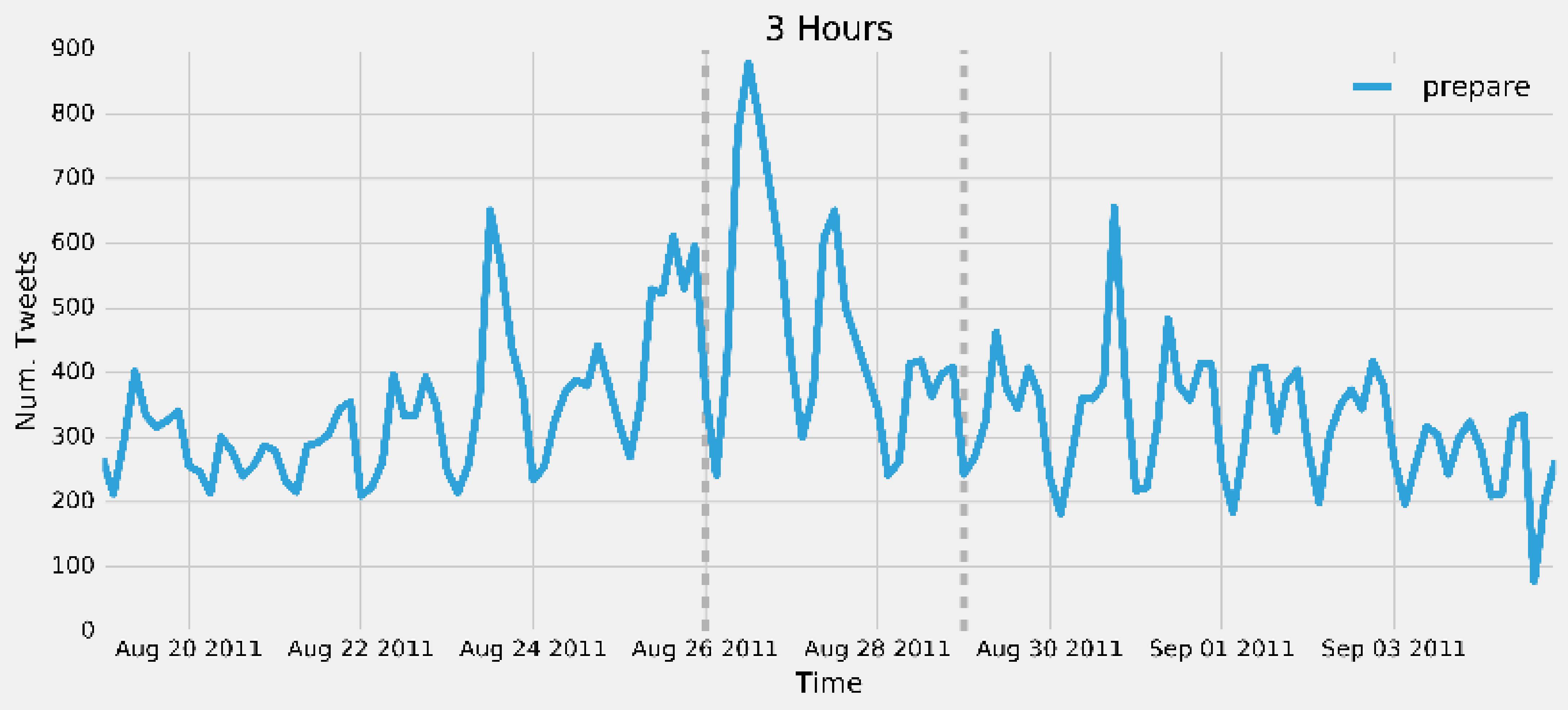
3 Hours
prepare
Num. Tweets
0
100
200
300
400
500
600
700
800
900
Aug 20 2011
Aug 22 2011
Aug 24 2011
Aug 26 2011
Aug 28 2011
Aug 30 2011
Sep 01 2011
Sep 03 2011
Time

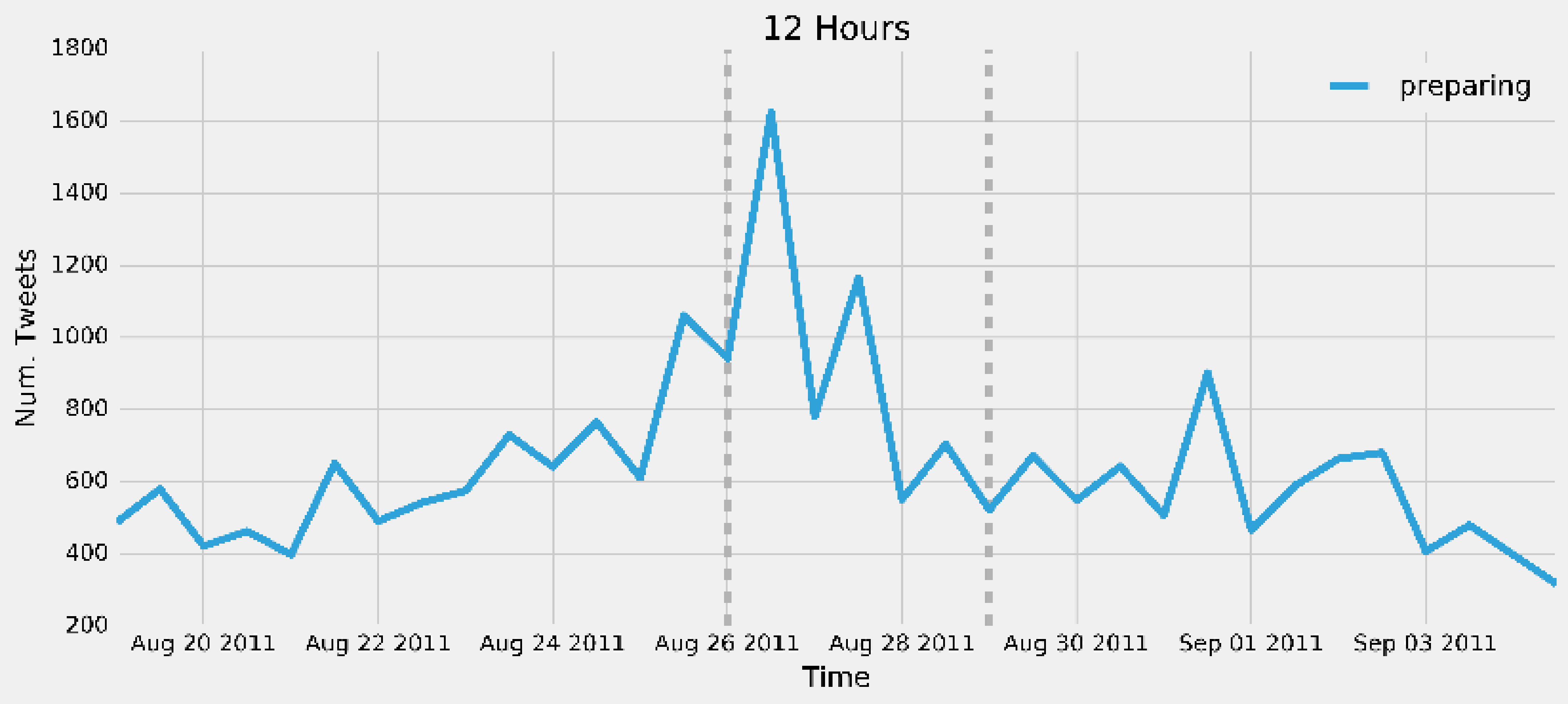

12 Hours
preparing
1800
1600
1400
1200
1000
800
600
400
200
Num. Tweets
Aug 20 2011
Aug 22 2011
Aug 24 2011
Aug 26 2011
Aug 28 2011
Aug 30 2011
Sep 01 2011
Sep 03 2011
Time

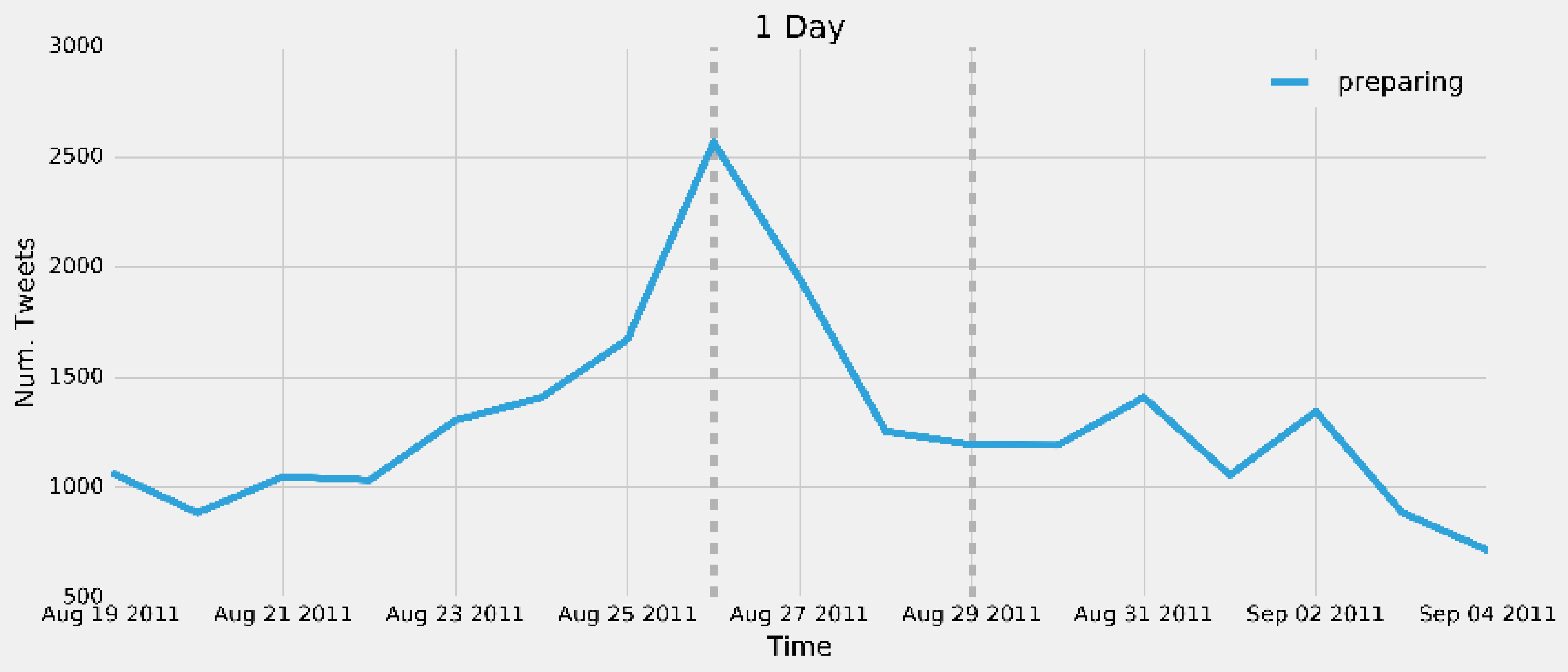

1 Day
preparing
3000
2500
2000
1500
1000
500
Num. Tweets
Aug 19 2011
Aug 21 2011
Aug 23 2011
Aug 25 2011
Aug 27 2011
Aug 29 2011
Aug 31 2011
Sep 02 2011
Sep 04 2011
Time

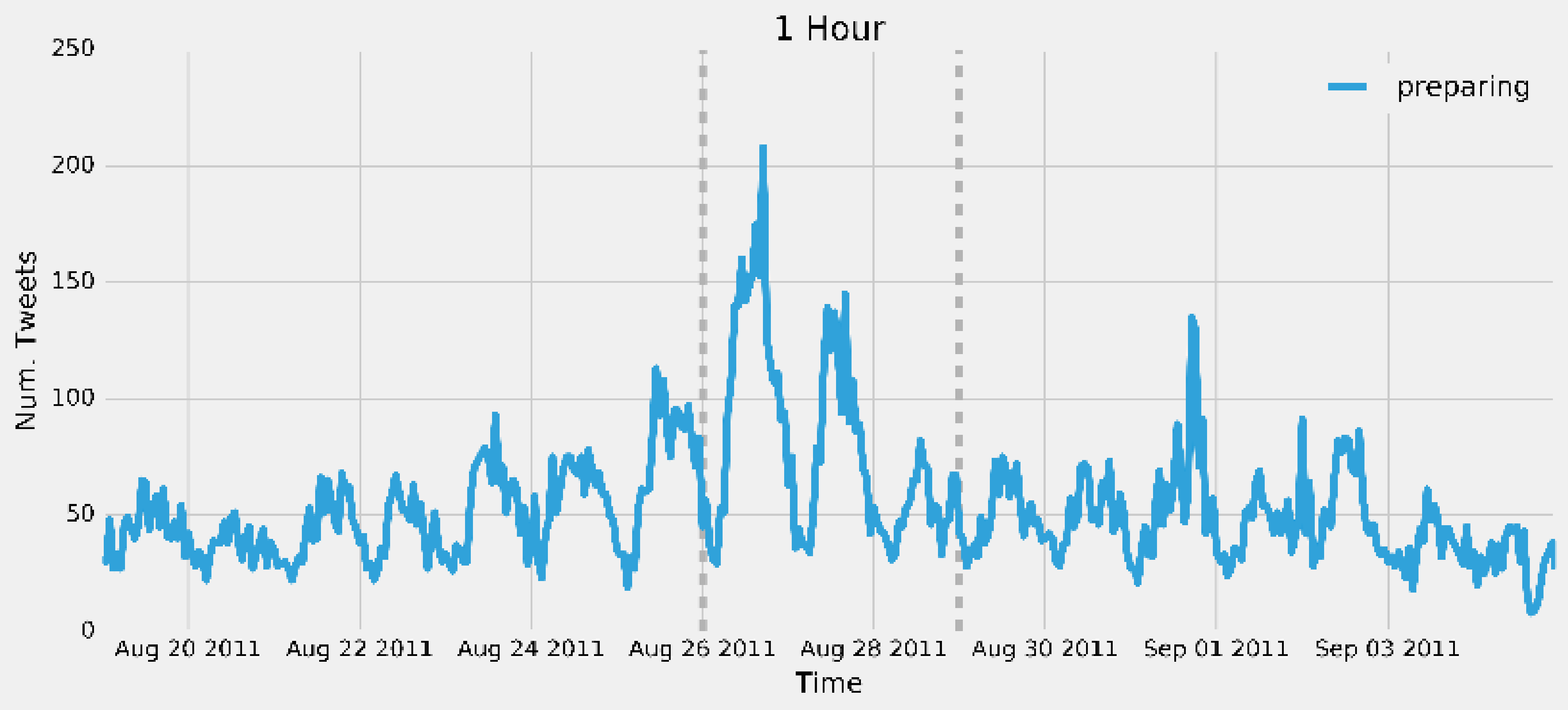
1 Hour
preparing
Num. Tweets
0
50
100
150
200
250
Aug 20 2011
Aug 22 2011
Aug 24 2011
Aug 26 2011
Aug 28 2011
Aug 30 2011
Sep 01 2011
Sep 03 2011
Time

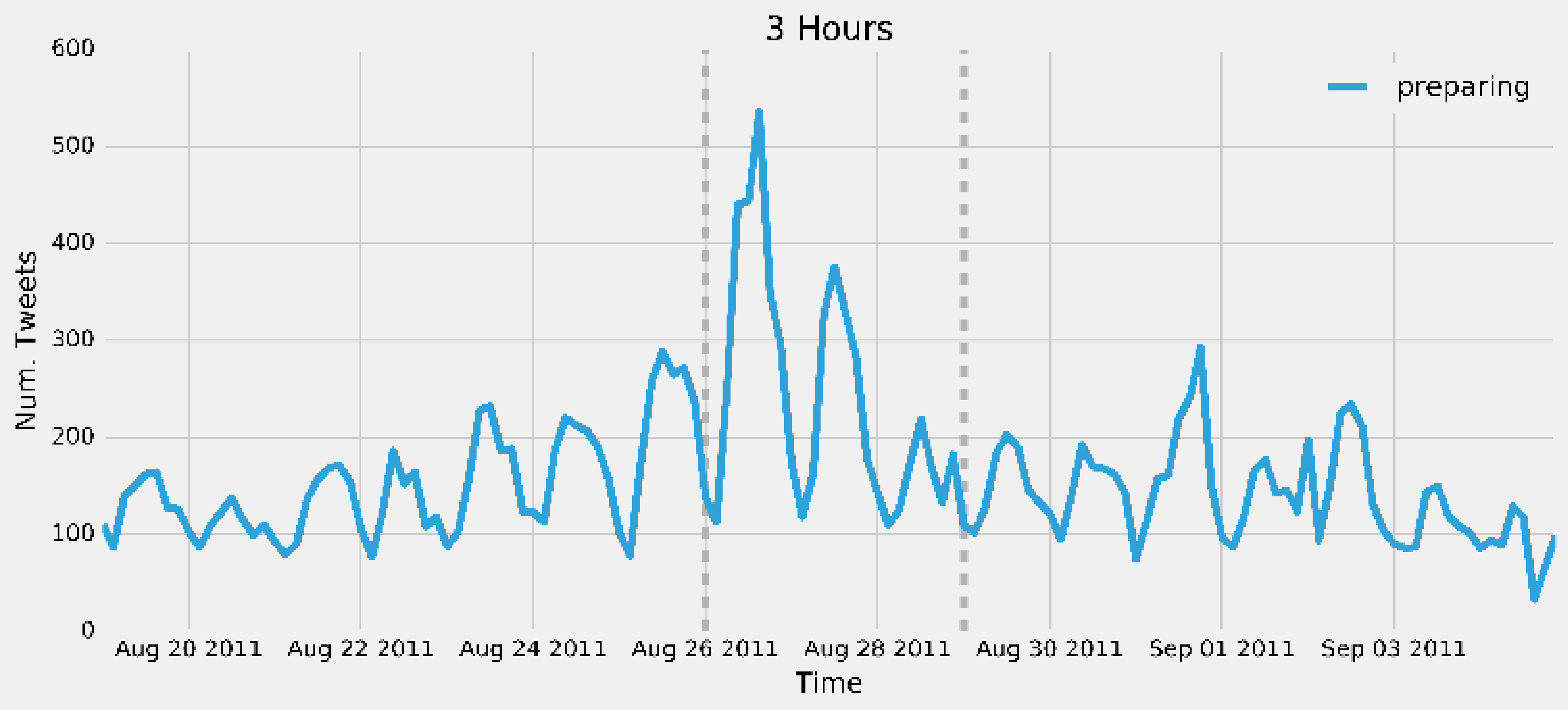

3 Hours
preparing
Num. Tweets
0
100
200
300
400
500
600
Aug 20 2011
Aug 22 2011
Aug 24 2011
Aug 26 2011
Aug 28 2011
Aug 30 2011
Sep 01 2011
Sep 03 2011
Time

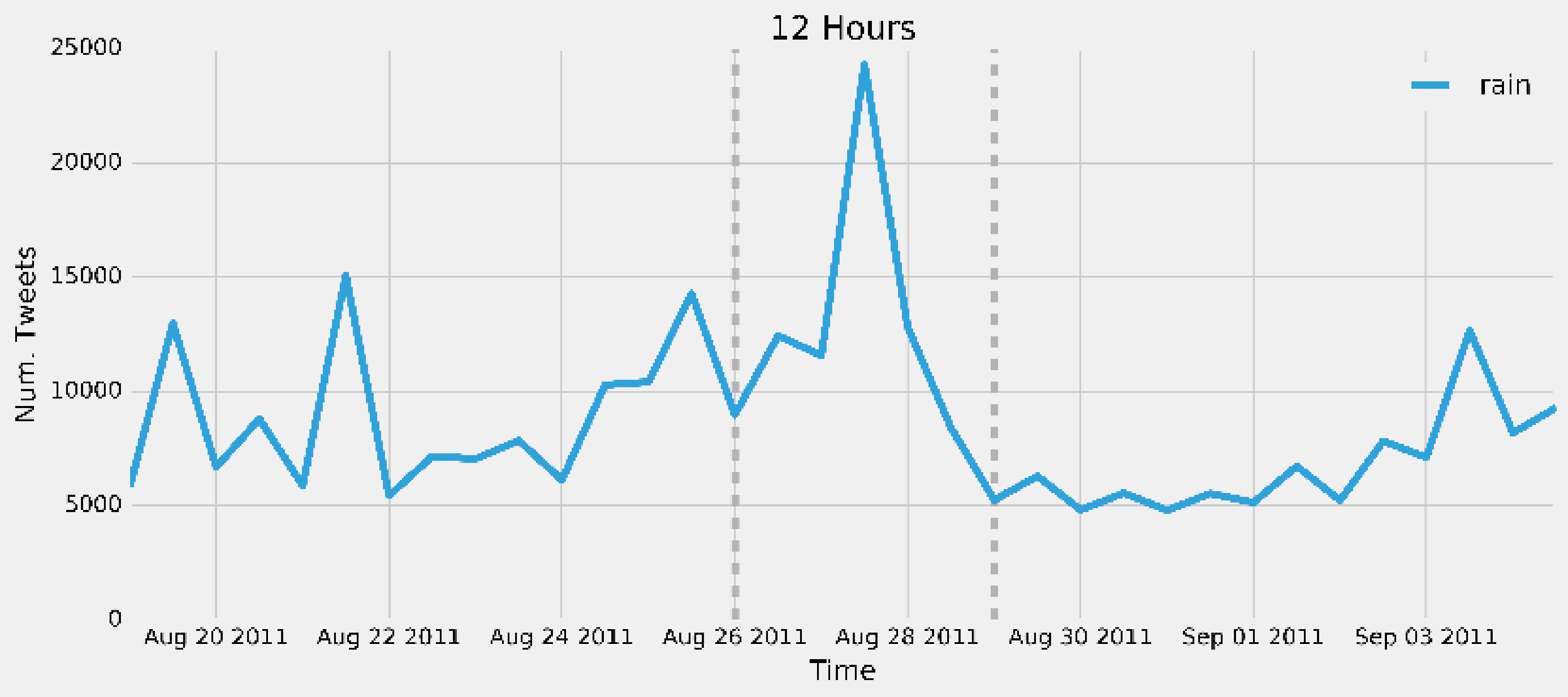

12 Hours
rain
Num. Tweets
0
5000
10000
15000
20000
25000
Aug 20 2011
Aug 22 2011
Aug 24 2011
Aug 26 2011
Aug 28 2011
Aug 30 2011
Sep 01 2011
Sep 03 2011
Time

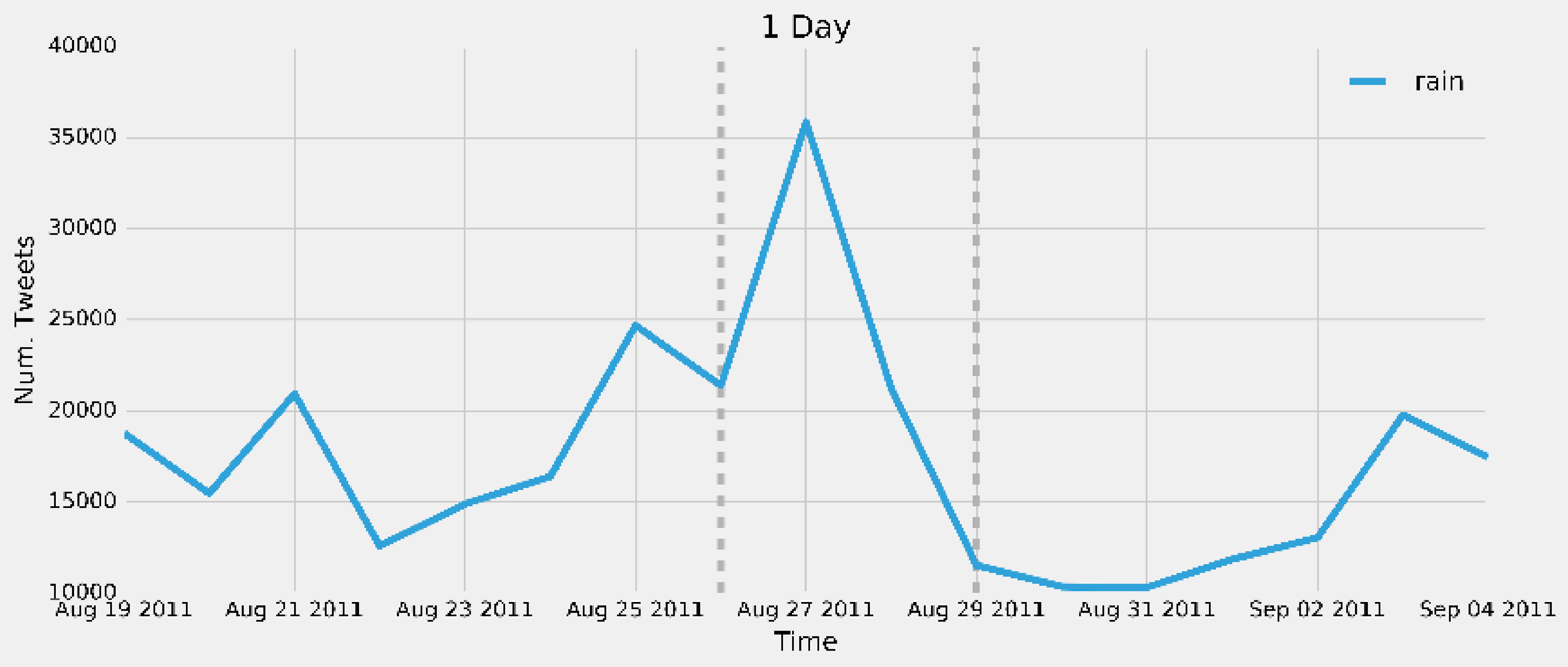
1 Day
rain
Num. Tweets
Time
40000
35000
30000
25000
20000
15000
10000
Aug 19 2011
Aug 21 2011
Aug 23 2011
Aug 25 2011
Aug 27 2011
Aug 29 2011
Aug 31 2011
Sep 02 2011
Sep 04 2011

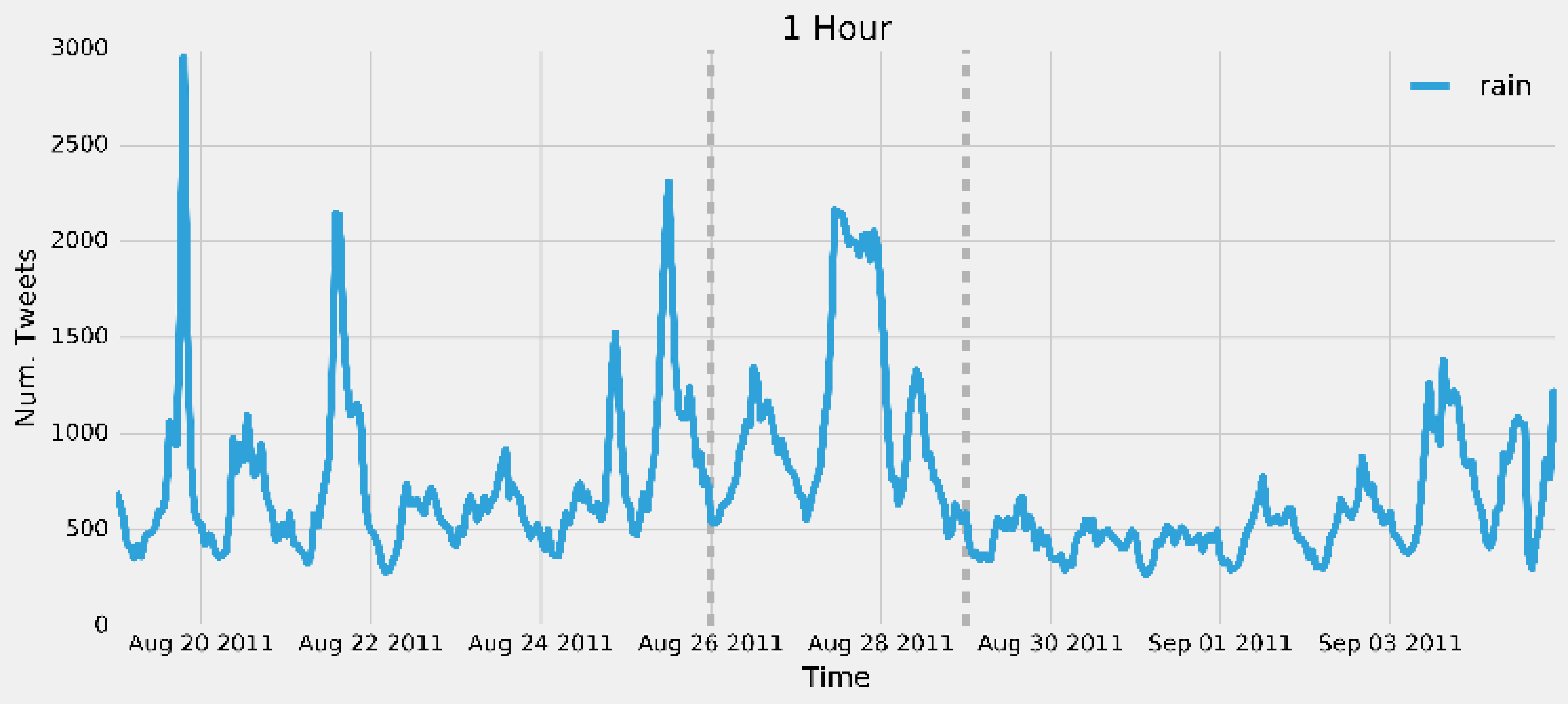

1 Hour
rain
Num. Tweets
3000
2500
2000
1500
1000
500
0
Aug 20 2011
Aug 22 2011
Aug 24 2011
Aug 26 2011
Aug 28 2011
Aug 30 2011
Sep 01 2011
Sep 03 2011
Time

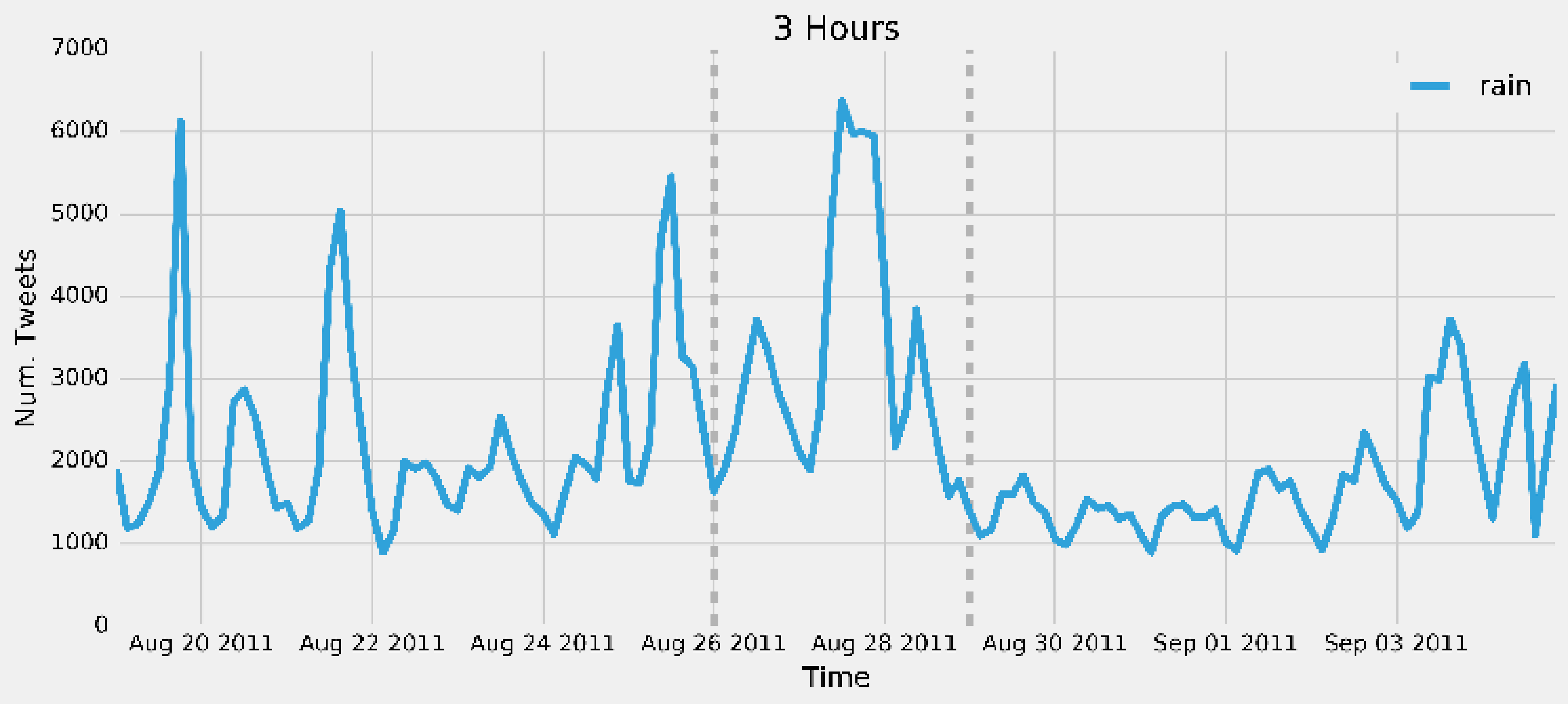
3 Hours
rain
Num. Tweets
0
1000
2000
3000
4000
5000
6000
7000
Aug 20 2011
Aug 22 2011
Aug 24 2011
Aug 26 2011
Aug 28 2011
Aug 30 2011
Sep 01 2011
Sep 03 2011
Time

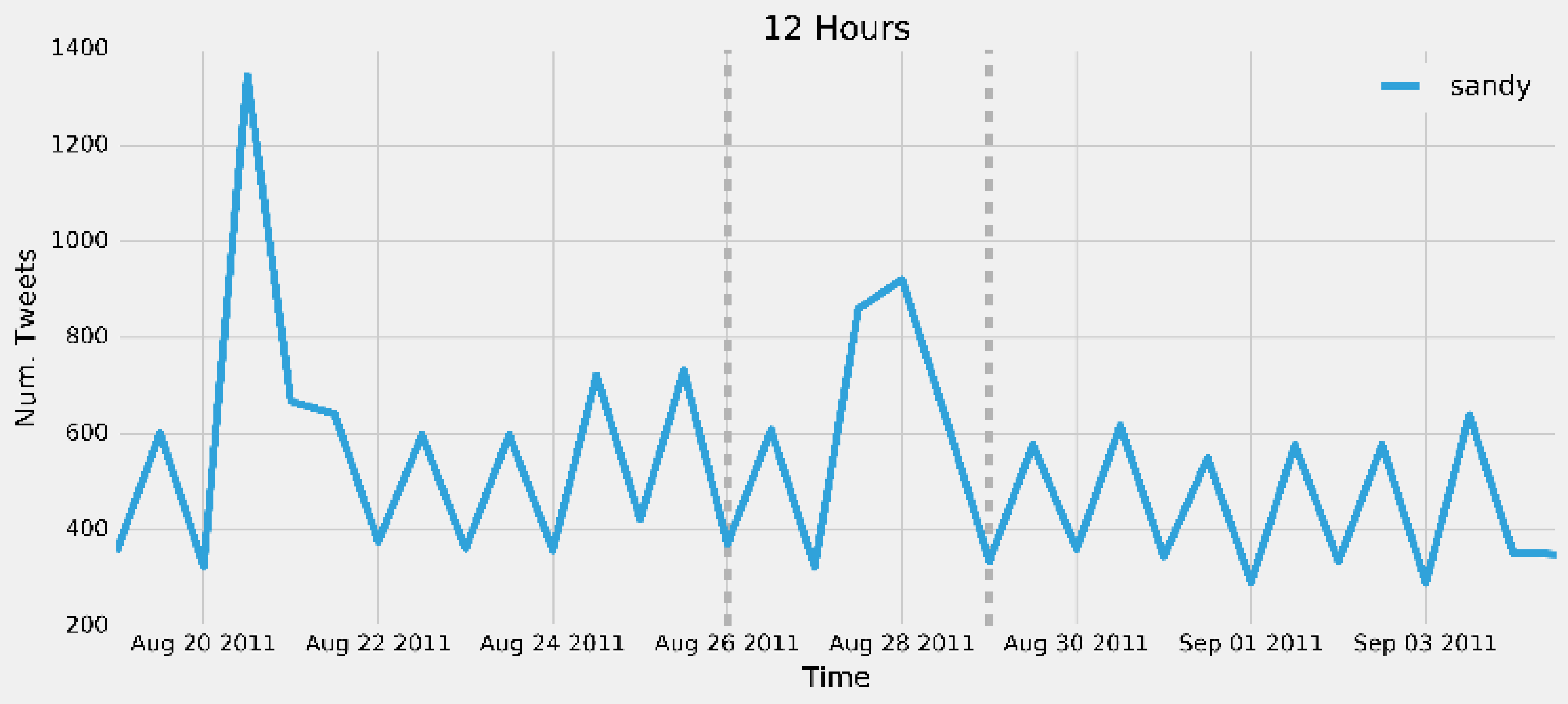

12 Hours
sandy
Num. Tweets
Time
1400
1200
1000
800
600
400
200
Aug 20 2011
Aug 22 2011
Aug 24 2011
Aug 26 2011
Aug 28 2011
Aug 30 2011
Sep 01 2011
Sep 03 2011

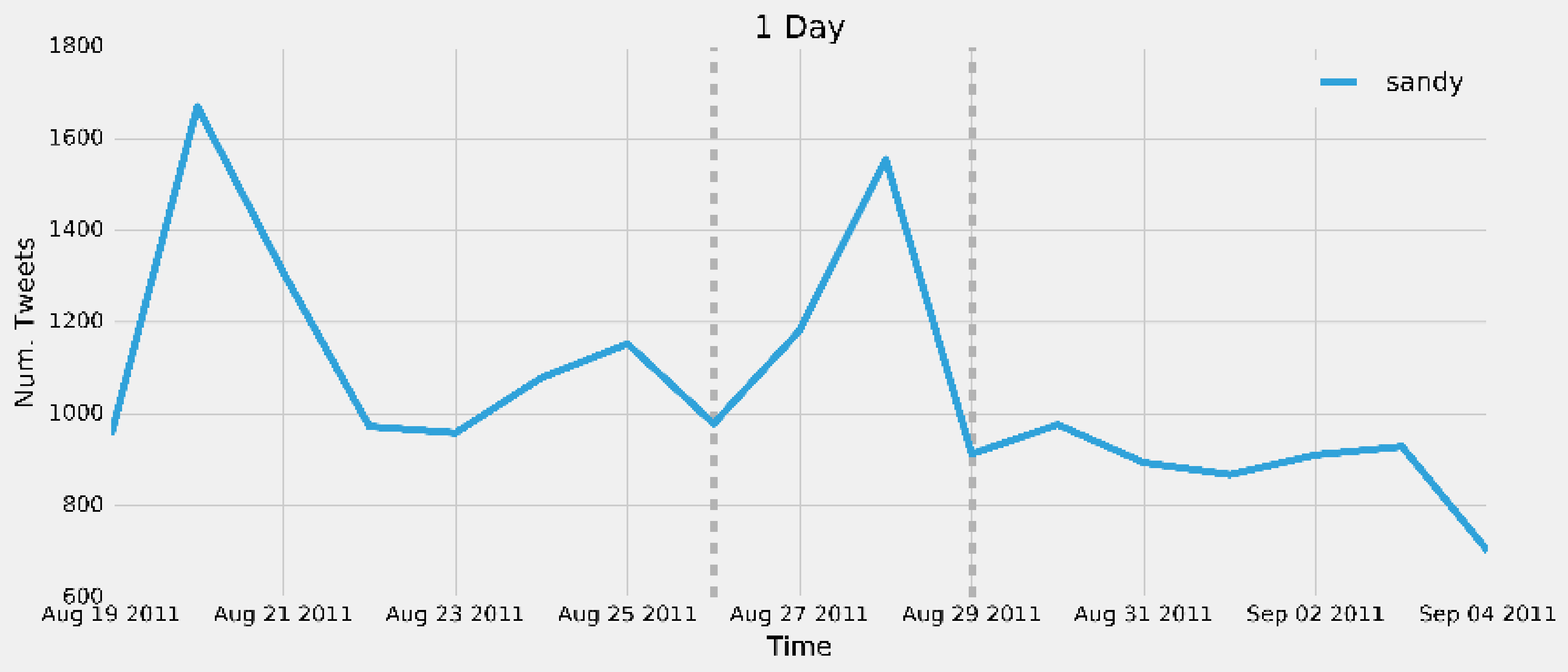
1 Day
sandy
Num. Tweets
1800
1600
1400
1200
1000
800
600
Aug 19 2011
Aug 21 2011
Aug 23 2011
Aug 25 2011
Aug 27 2011
Aug 29 2011
Aug 31 2011
Sep 02 2011
Sep 04 2011
Time

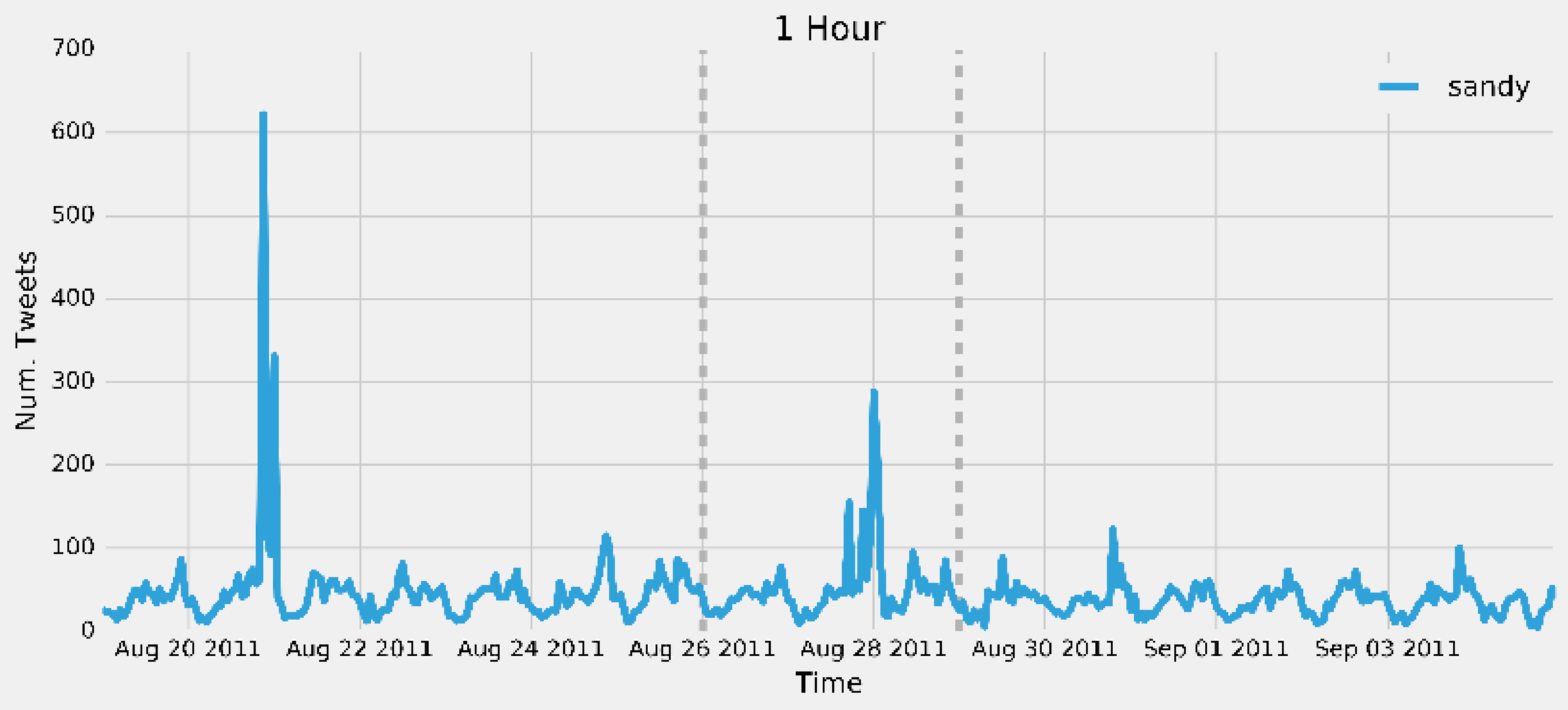

1 Hour
sandy
Num. Tweets
0
100
200
300
400
500
600
700
Aug 20 2011
Aug 22 2011
Aug 24 2011
Aug 26 2011
Aug 28 2011
Aug 30 2011
Sep 01 2011
Sep 03 2011
Time

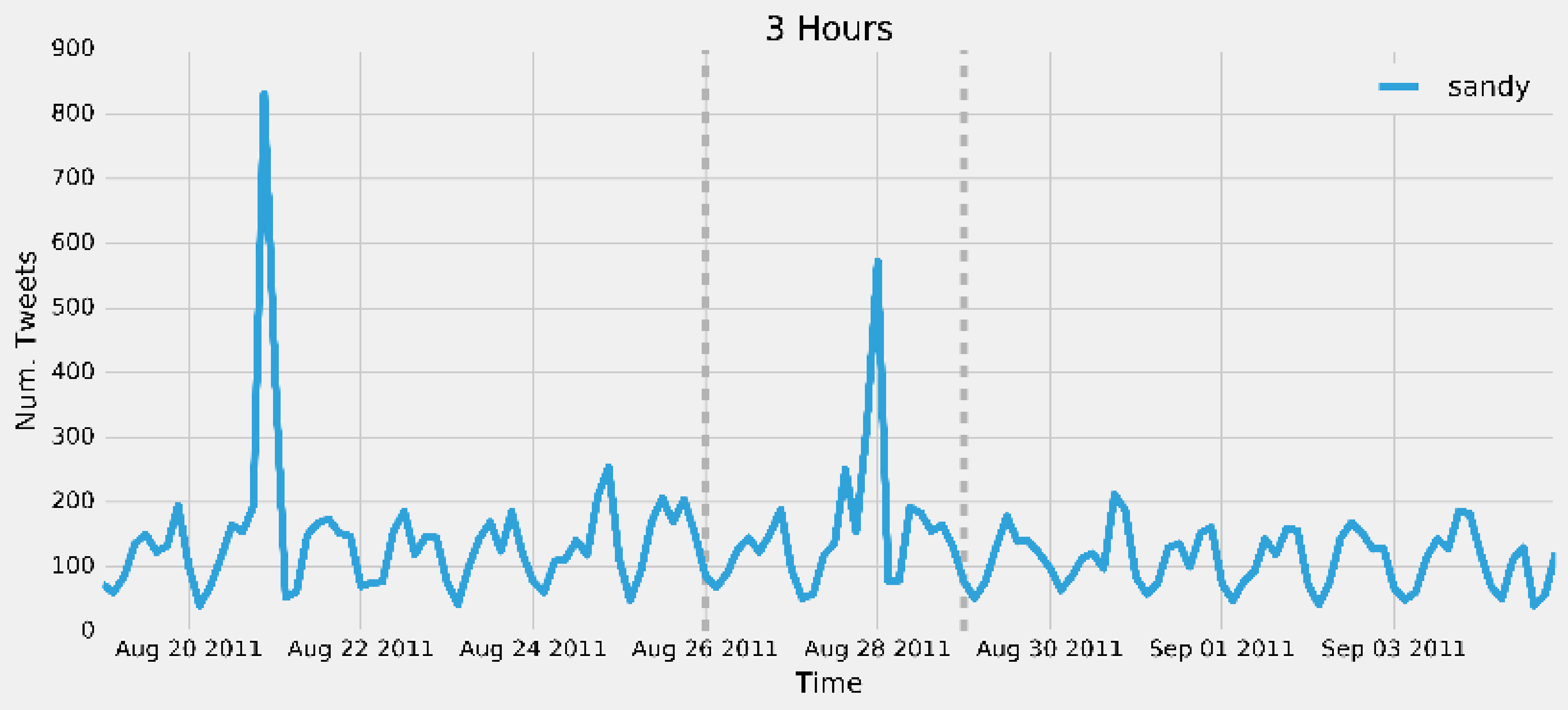

3 Hours
sandy
Num. Tweets
900
800
700
600
500
400
300
200
100
0
Aug 20 2011
Aug 22 2011
Aug 24 2011
Aug 26 2011
Aug 28 2011
Aug 30 2011
Sep 01 2011
Sep 03 2011
Time

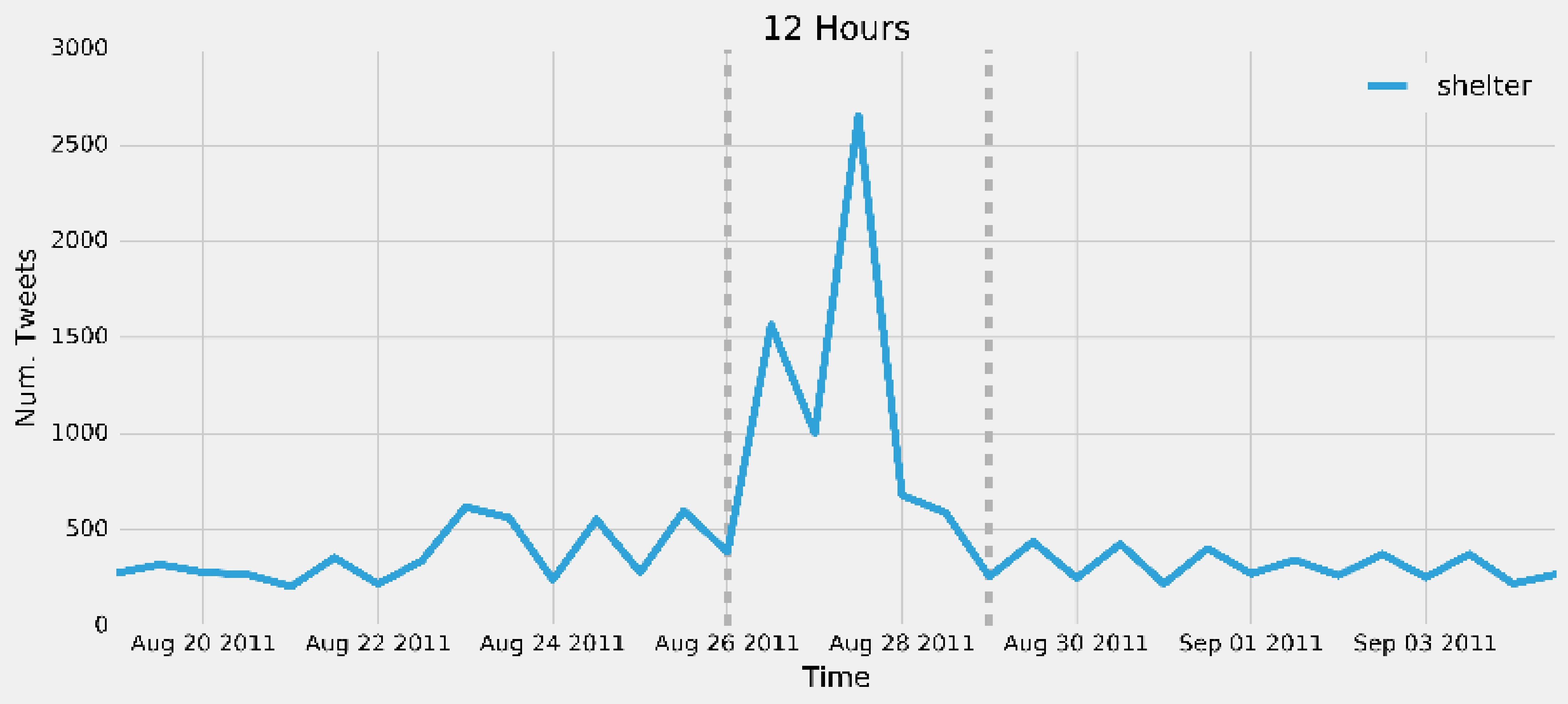
12 Hours
shelter
Num. Tweets
0
500
1000
1500
2000
2500
3000
Aug 20 2011
Aug 22 2011
Aug 24 2011
Aug 26 2011
Aug 28 2011
Aug 30 2011
Sep 01 2011
Sep 03 2011
Time

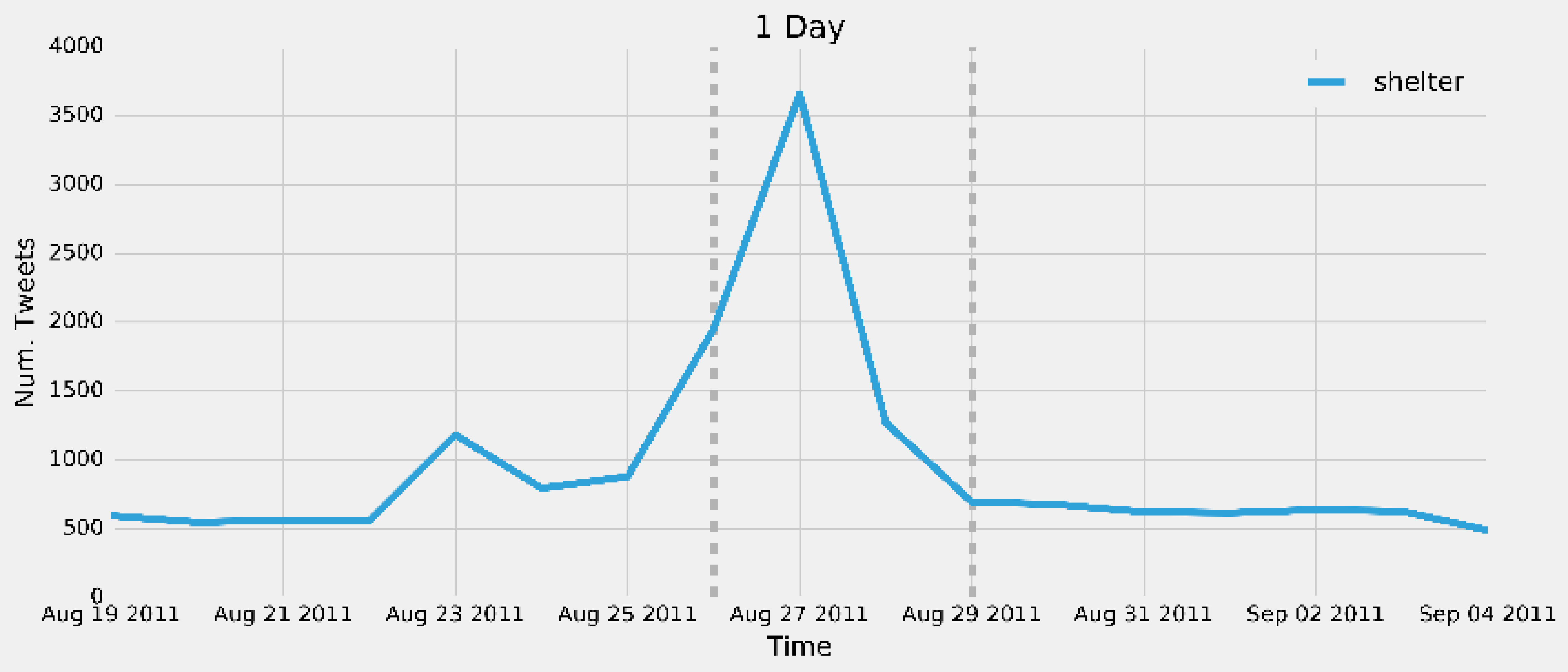

1 Day
shelter
Num. Tweets
4000
3500
3000
2500
2000
1500
1000
500
0
Aug 19 2011
Aug 21 2011
Aug 23 2011
Aug 25 2011
Aug 27 2011
Aug 29 2011
Aug 31 2011
Sep 02 2011
Sep 04 2011
Time

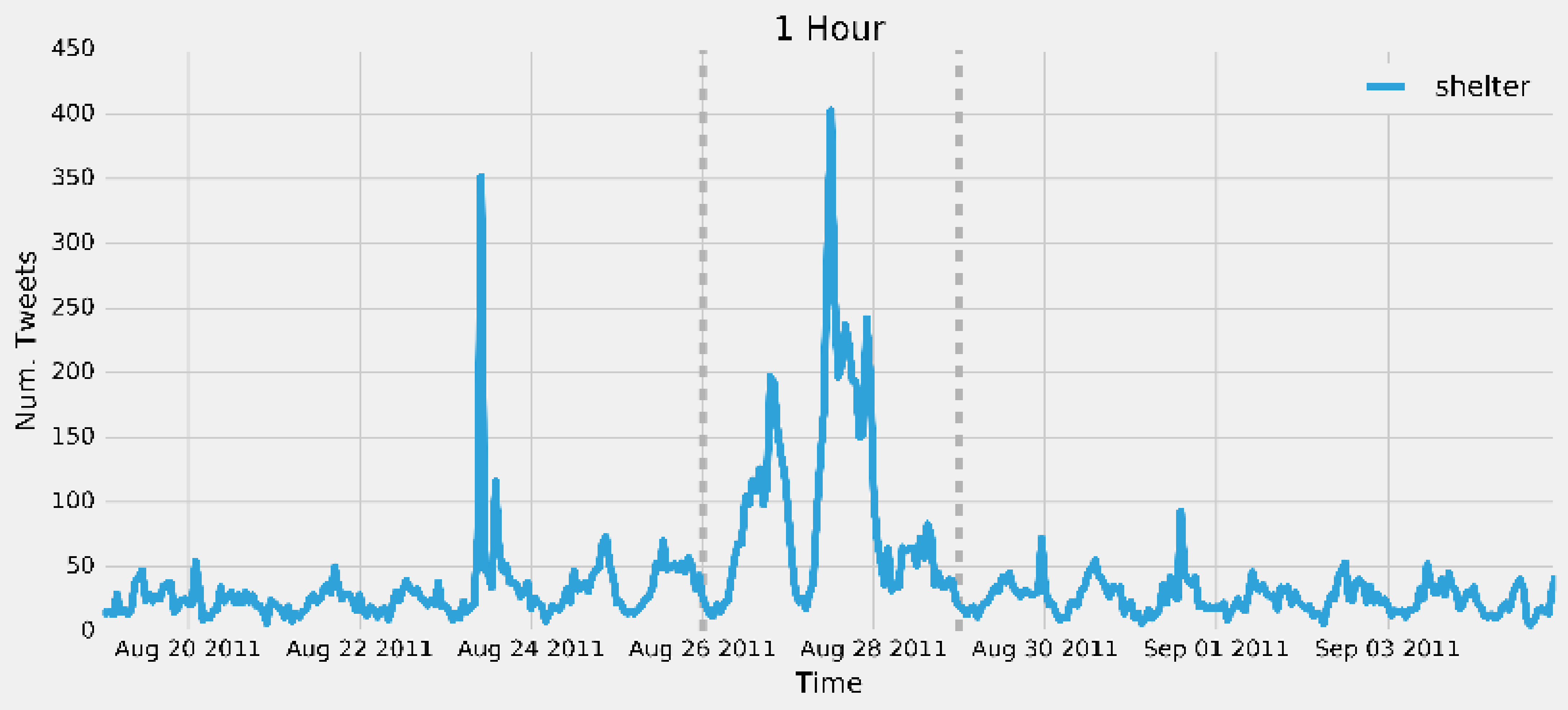

1 Hour
shelter
Num. Tweets
0
50
100
150
200
250
300
350
400
450
Aug 20 2011
Aug 22 2011
Aug 24 2011
Aug 26 2011
Aug 28 2011
Aug 30 2011
Sep 01 2011
Sep 03 2011
Time

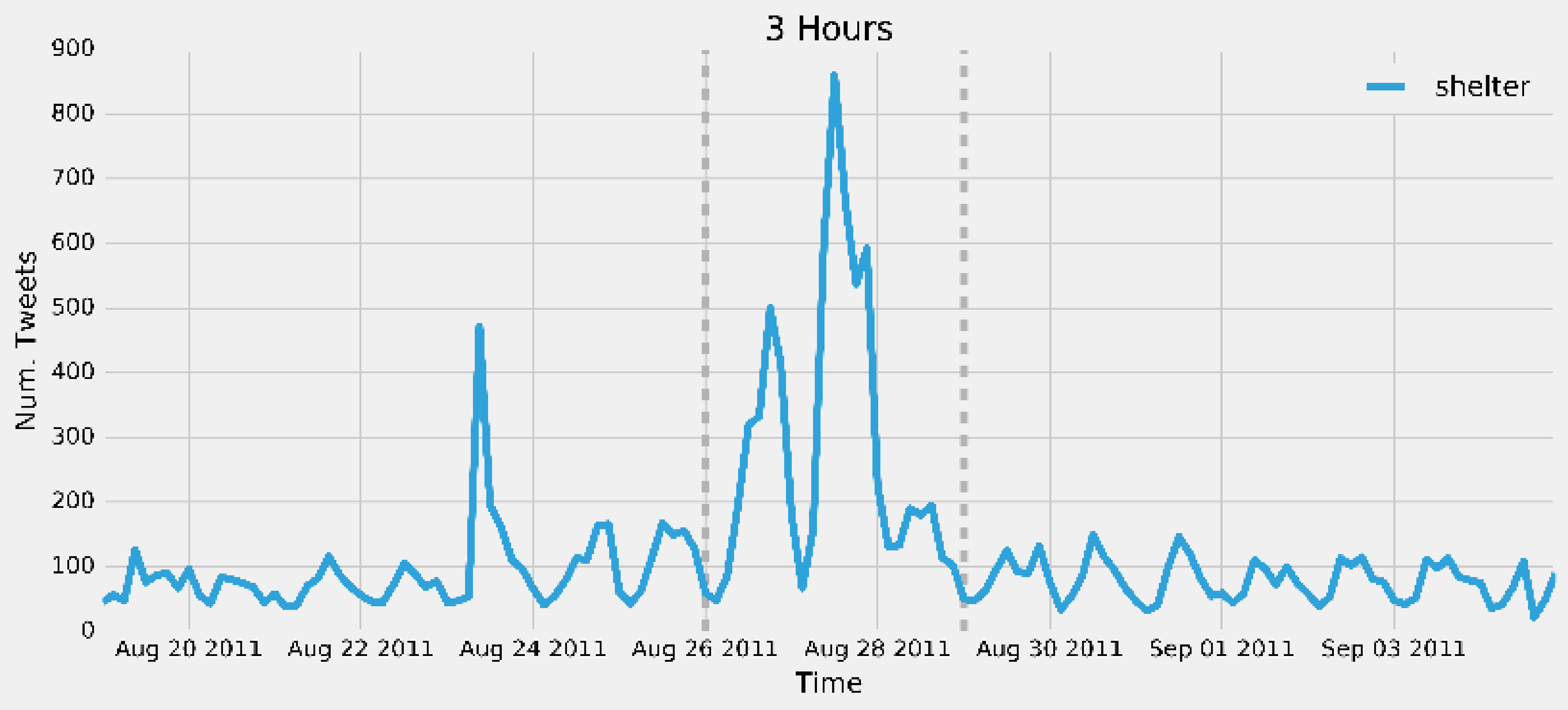
3 Hours
shelter
Num. Tweets
0
100
200
300
400
500
600
700
800
900
Aug 20 2011
Aug 22 2011
Aug 24 2011
Aug 26 2011
Aug 28 2011
Aug 30 2011
Sep 01 2011
Sep 03 2011
Time

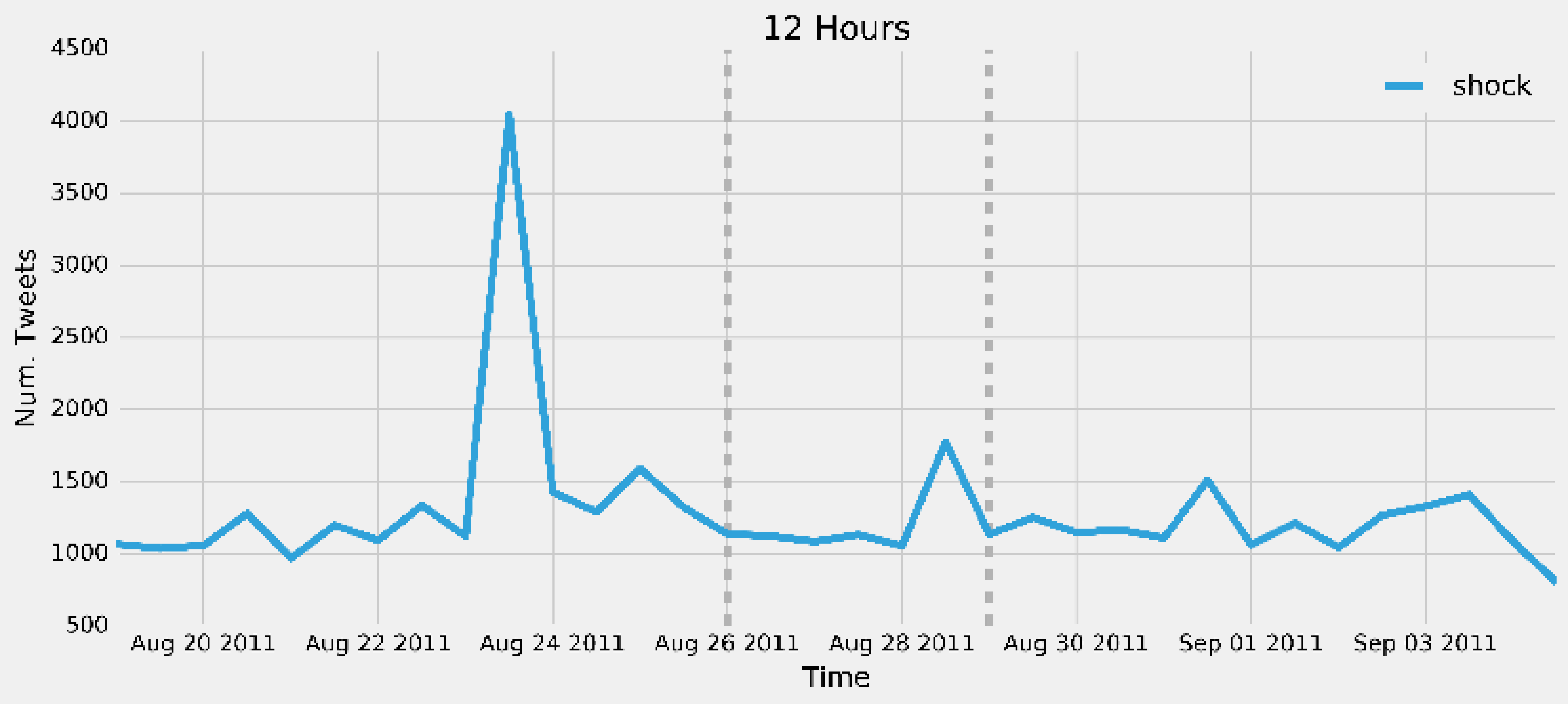

12 Hours
shock
Num. Tweets
4500
4000
3500
3000
2500
2000
1500
1000
500
Aug 20 2011
Aug 22 2011
Aug 24 2011
Aug 26 2011
Aug 28 2011
Aug 30 2011
Sep 01 2011
Sep 03 2011
Time

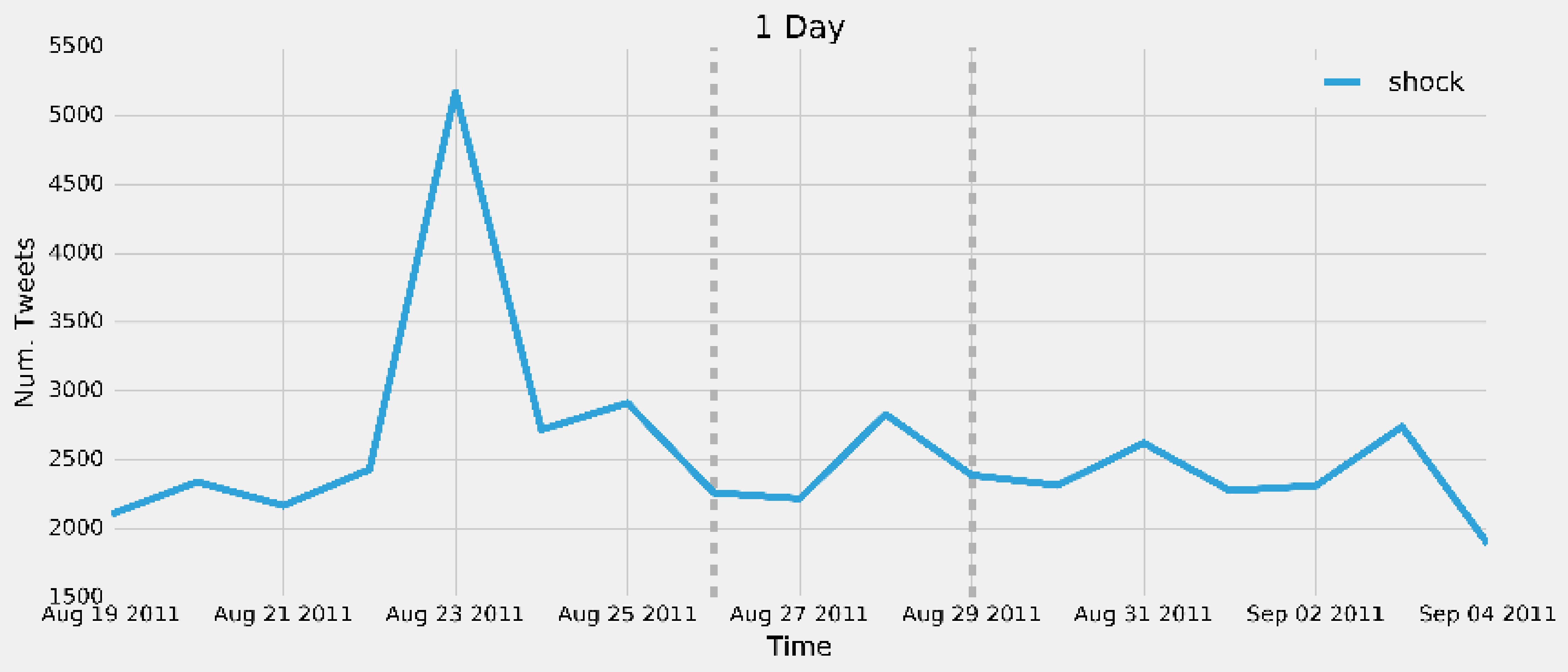
1 Day
shock
Num. Tweets
5500
5000
4500
4000
3500
3000
2500
2000
1500
Aug 19 2011
Aug 21 2011
Aug 23 2011
Aug 25 2011
Aug 27 2011
Aug 29 2011
Aug 31 2011
Sep 02 2011
Sep 04 2011
Time

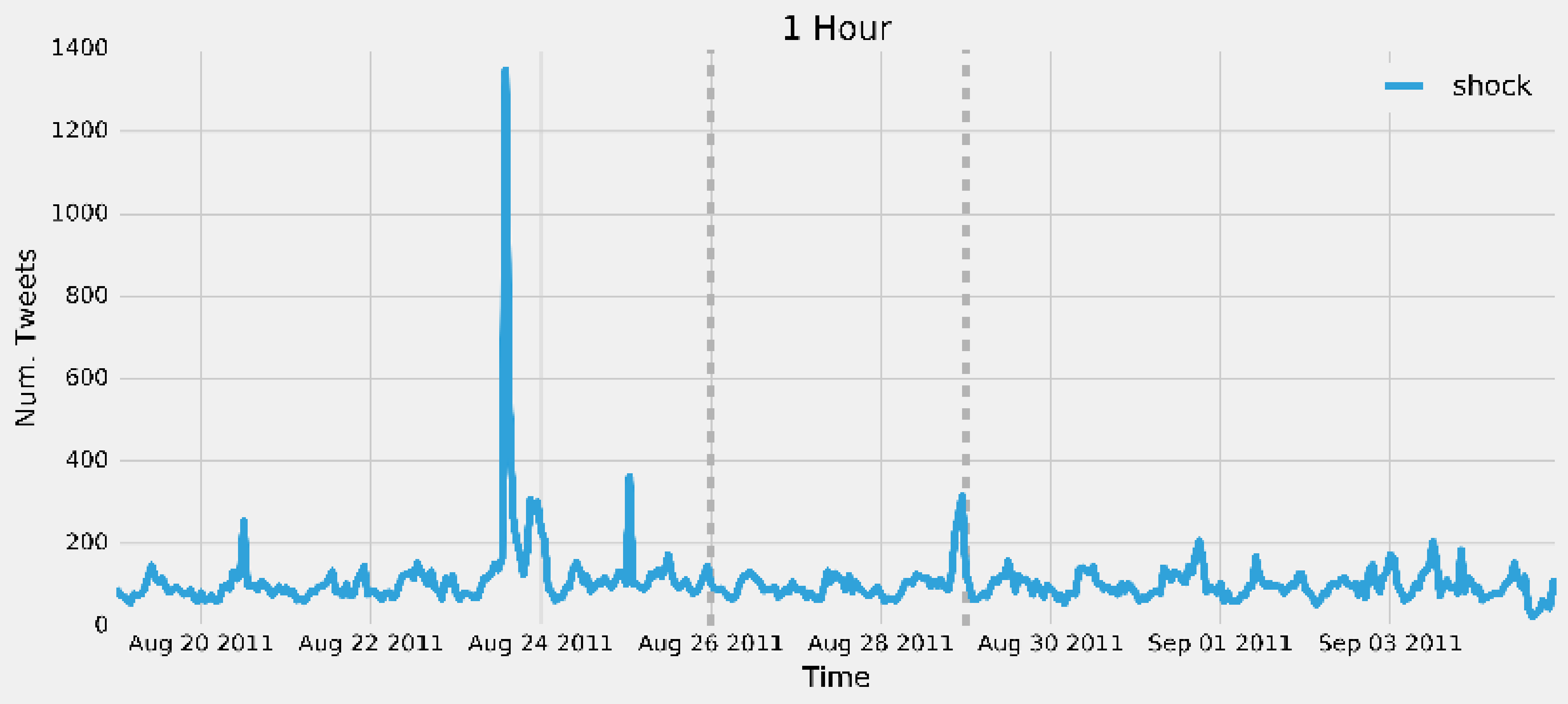
1 Hour
shock
Num. Tweets
0
200
400
600
800
1000
1200
1400
Aug 20 2011
Aug 22 2011
Aug 24 2011
Aug 26 2011
Aug 28 2011
Aug 30 2011
Sep 01 2011
Sep 03 2011
Time

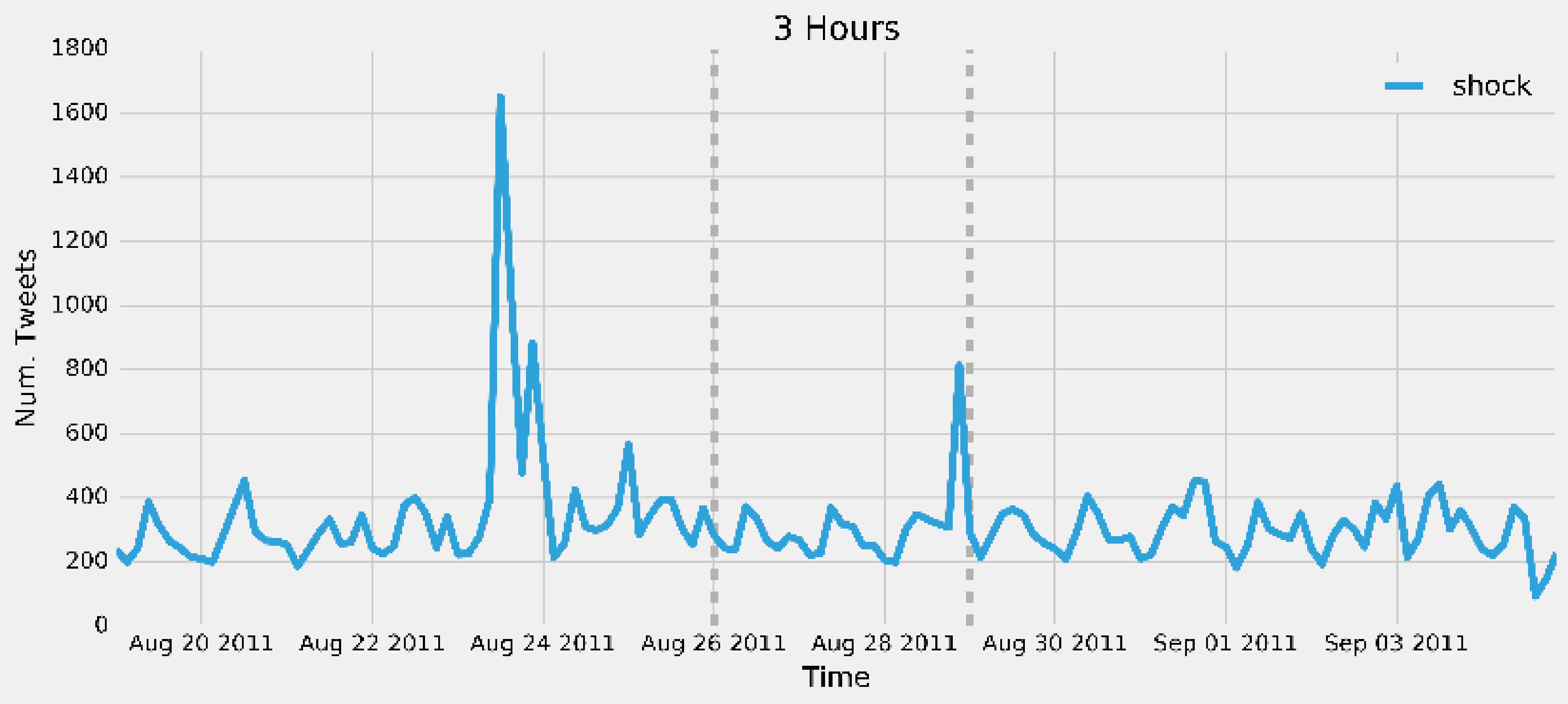

3 Hours
shock
Num. Tweets
Time
0
200
400
600
800
1000
1200
1400
1600
1800
Aug 20 2011
Aug 22 2011
Aug 24 2011
Aug 26 2011
Aug 28 2011
Aug 30 2011
Sep 01 2011
Sep 03 2011

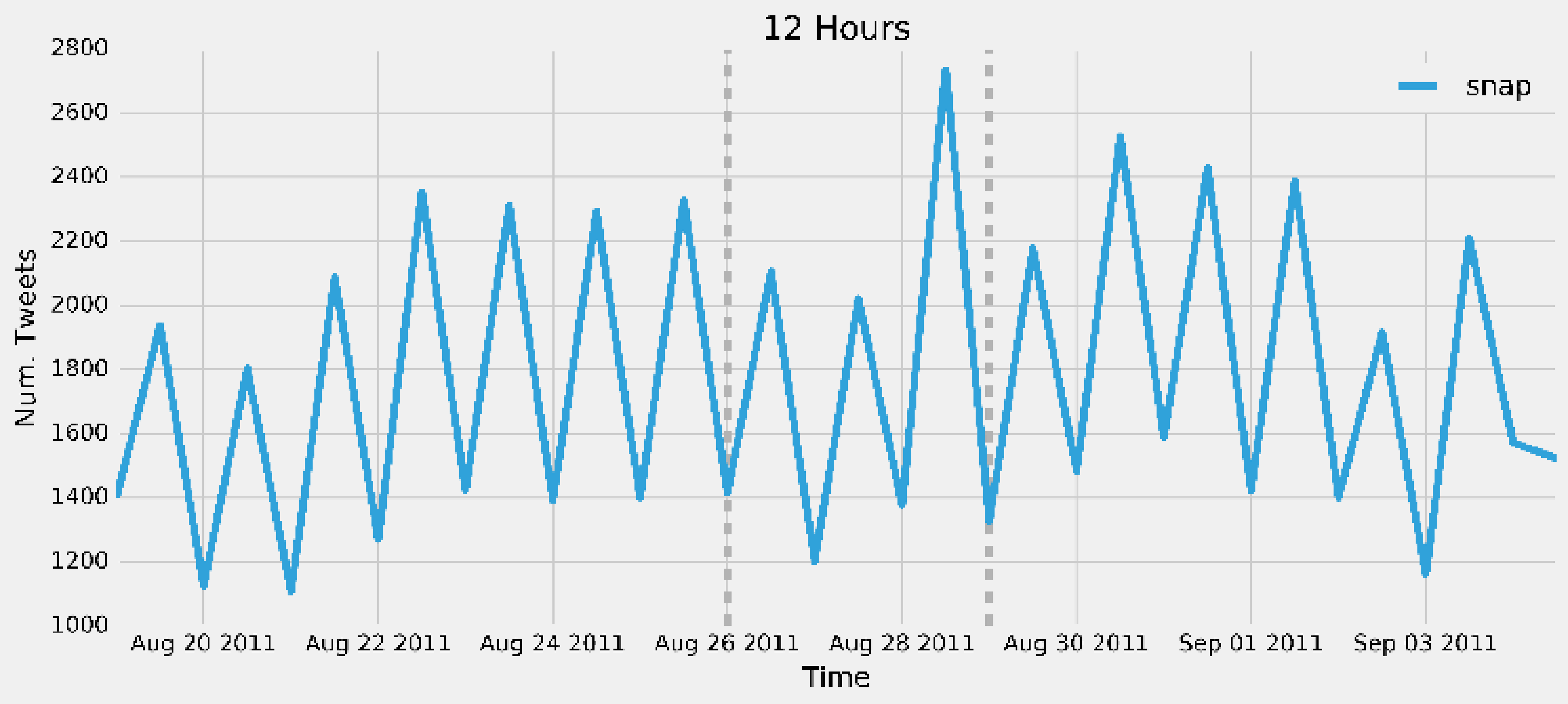

12 Hours
snap
Num. Tweets
Time
2800
2600
2400
2200
2000
1800
1600
1400
1200
1000
Aug 20 2011
Aug 22 2011
Aug 24 2011
Aug 26 2011
Aug 28 2011
Aug 30 2011
Sep 01 2011
Sep 03 2011

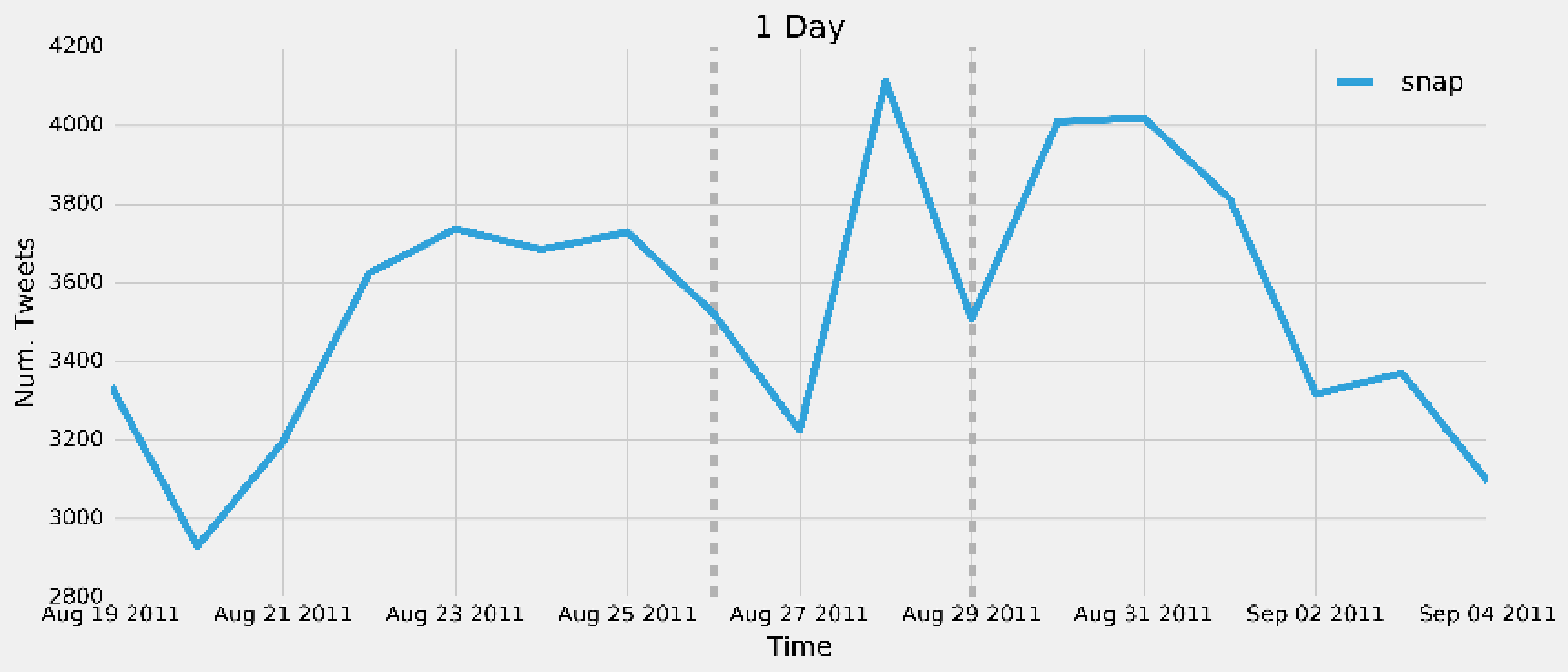

1 Day
snap
Num. Tweets
4200
4000
3800
3600
3400
3200
3000
2800
Aug 19 2011
Aug 21 2011
Aug 23 2011
Aug 25 2011
Aug 27 2011
Aug 29 2011
Aug 31 2011
Sep 02 2011
Sep 04 2011
Time

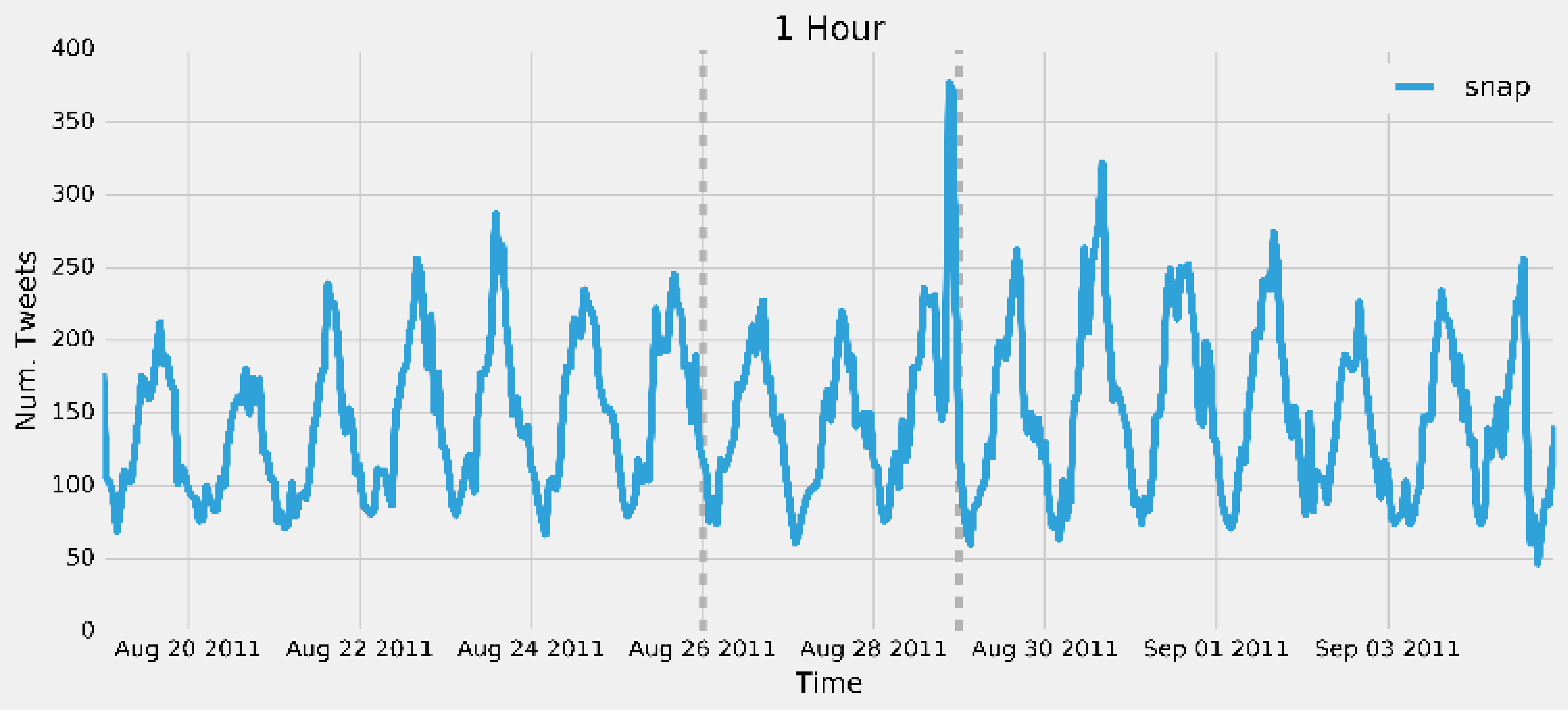

1 Hour
snap
Num. Tweets
400
350
300
250
200
150
100
50
0
Aug 20 2011
Aug 22 2011
Aug 24 2011
Aug 26 2011
Aug 28 2011
Aug 30 2011
Sep 01 2011
Sep 03 2011
Time

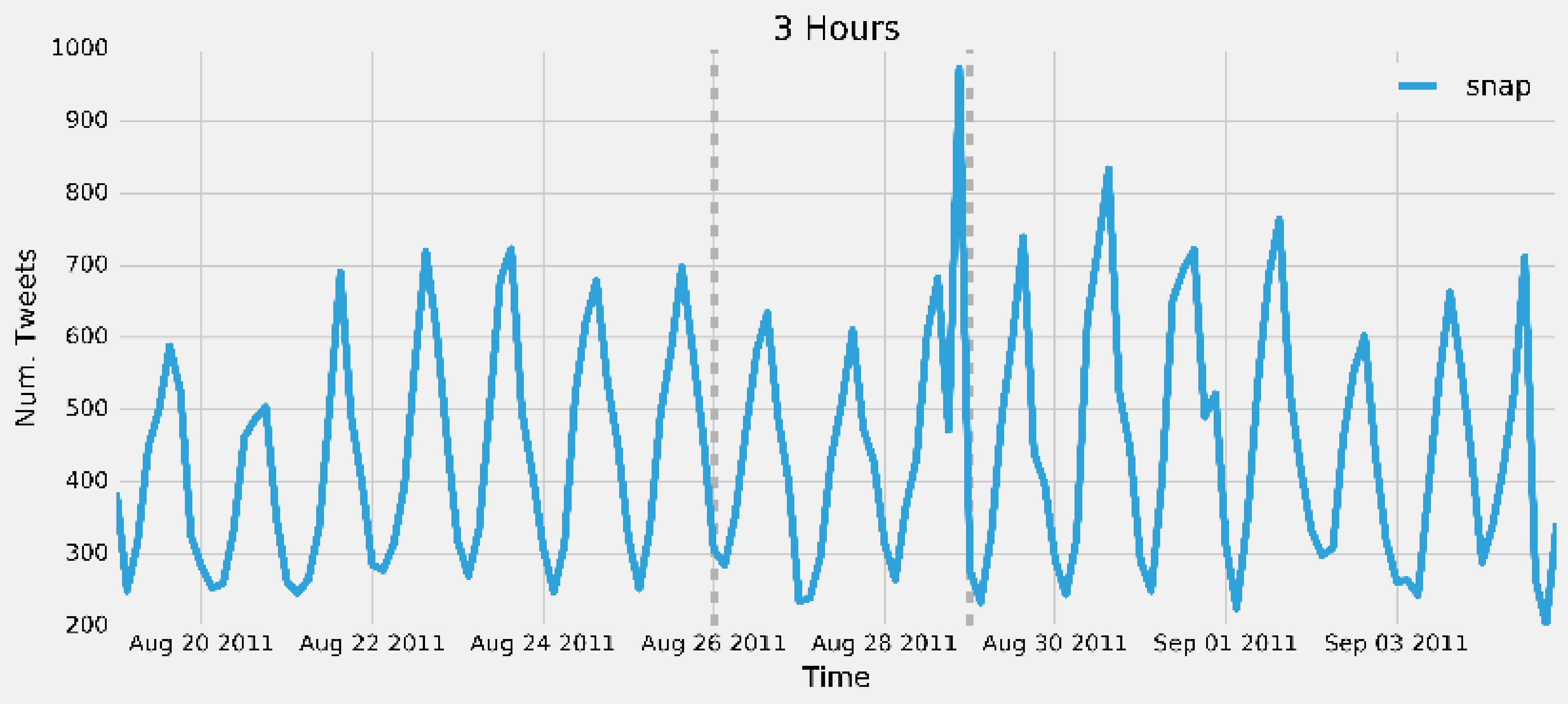

3 Hours
snap
1000
900
800
700
600
500
400
300
200
Num. Tweets
Aug 20 2011
Aug 22 2011
Aug 24 2011
Aug 26 2011
Aug 28 2011
Aug 30 2011
Sep 01 2011
Sep 03 2011
Time

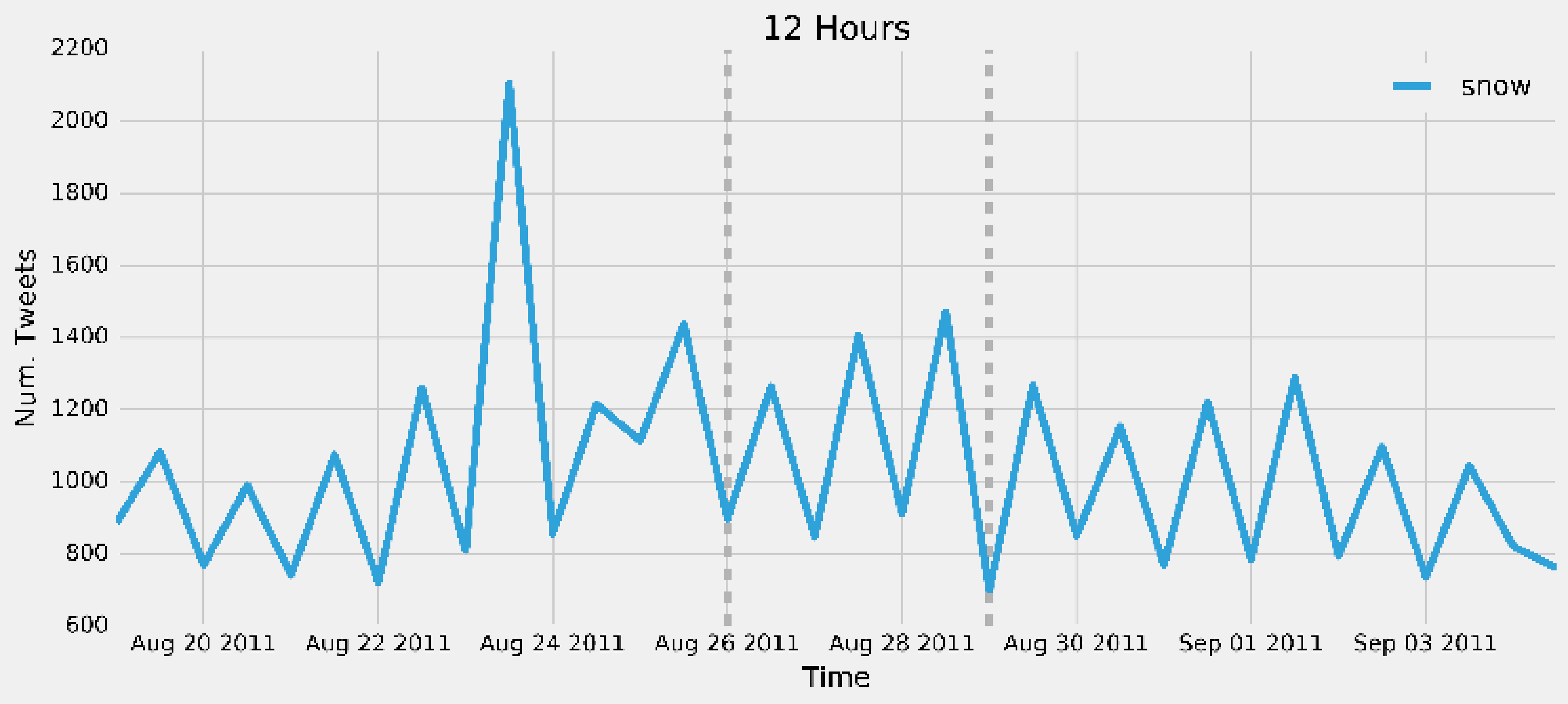
12 Hours
snow
Num. Tweets
2200
2000
1800
1600
1400
1200
1000
800
600
Aug 20 2011
Aug 22 2011
Aug 24 2011
Aug 26 2011
Aug 28 2011
Aug 30 2011
Sep 01 2011
Sep 03 2011
Time

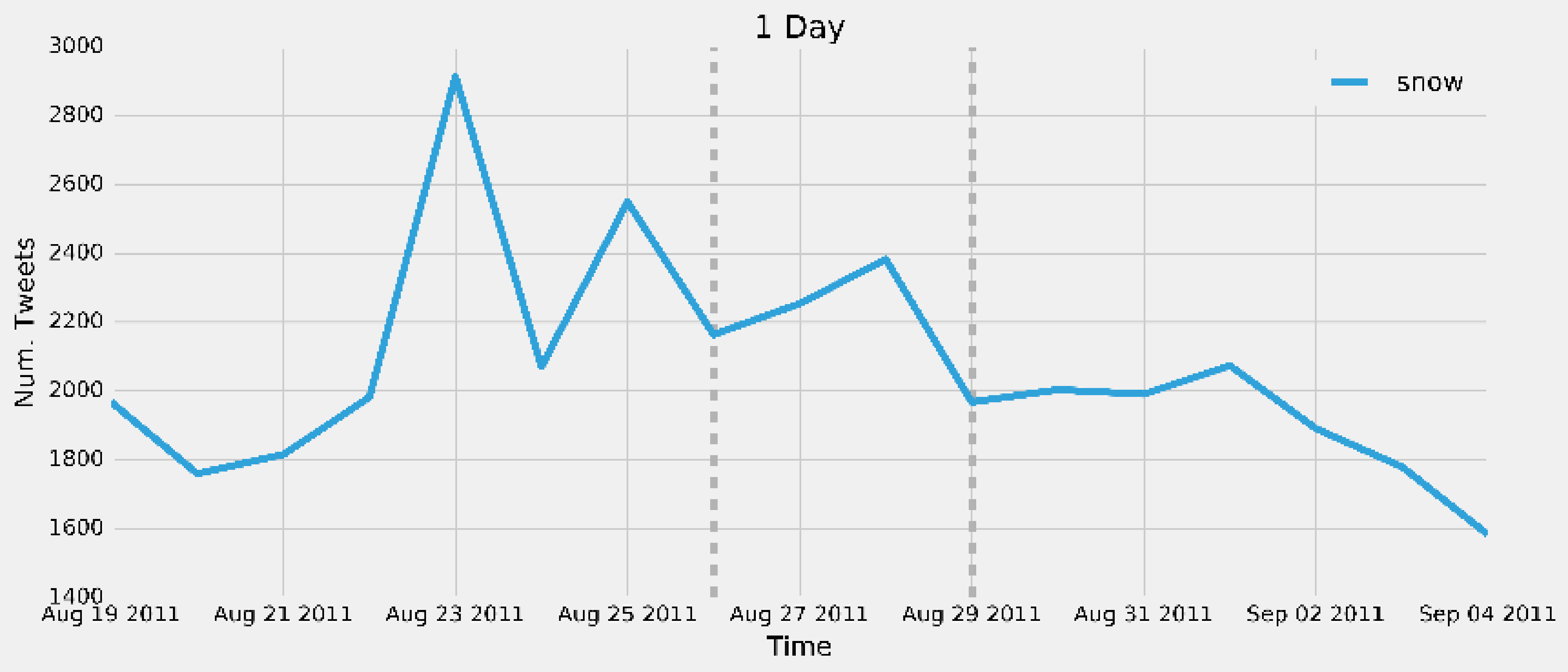

1 Day
snow
3000
2800
2600
2400
2200
2000
1800
1600
1400
Num. Tweets
Aug 19 2011
Aug 21 2011
Aug 23 2011
Aug 25 2011
Aug 27 2011
Aug 29 2011
Aug 31 2011
Sep 02 2011
Sep 04 2011
Time

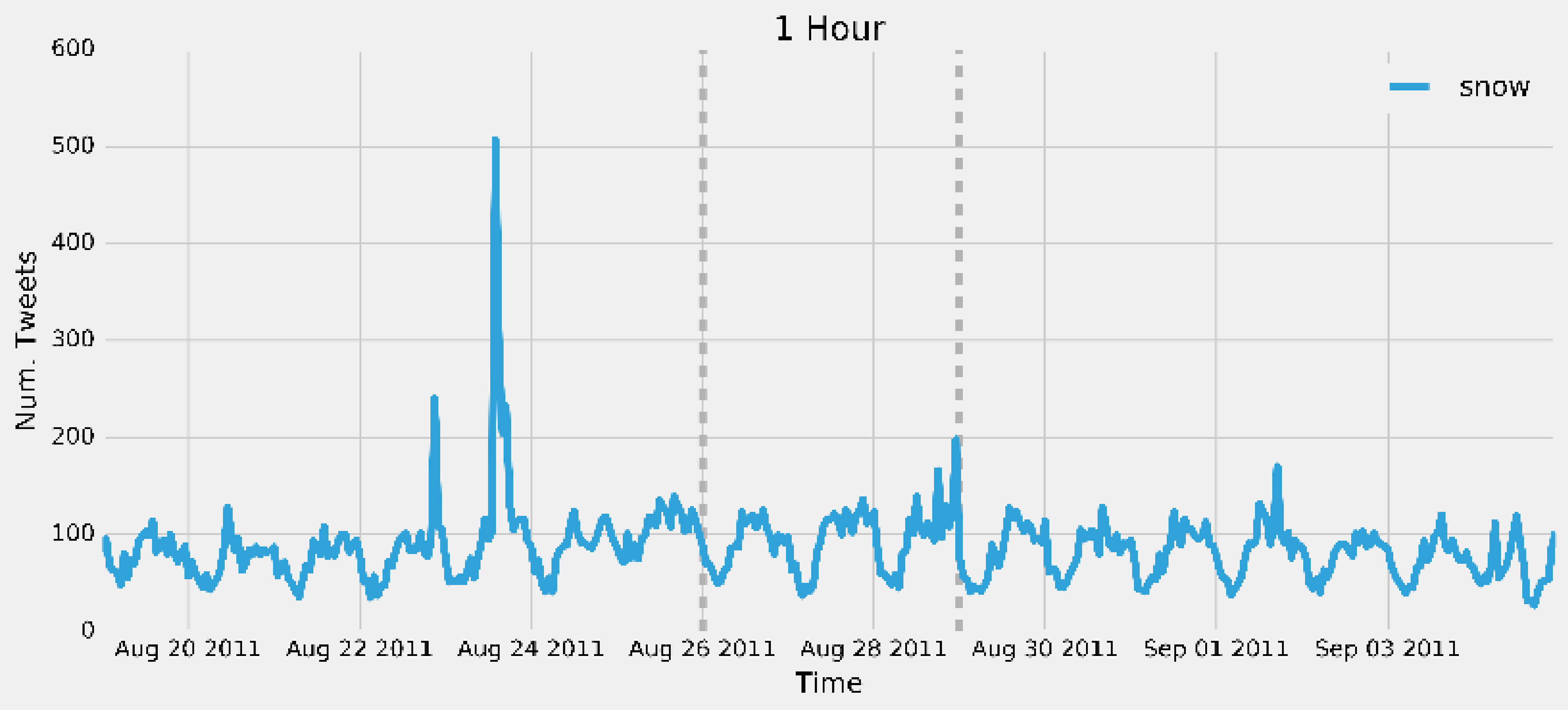
1 Hour
snow
Num. Tweets
0
100
200
300
400
500
600
Aug 20 2011
Aug 22 2011
Aug 24 2011
Aug 26 2011
Aug 28 2011
Aug 30 2011
Sep 01 2011
Sep 03 2011
Time

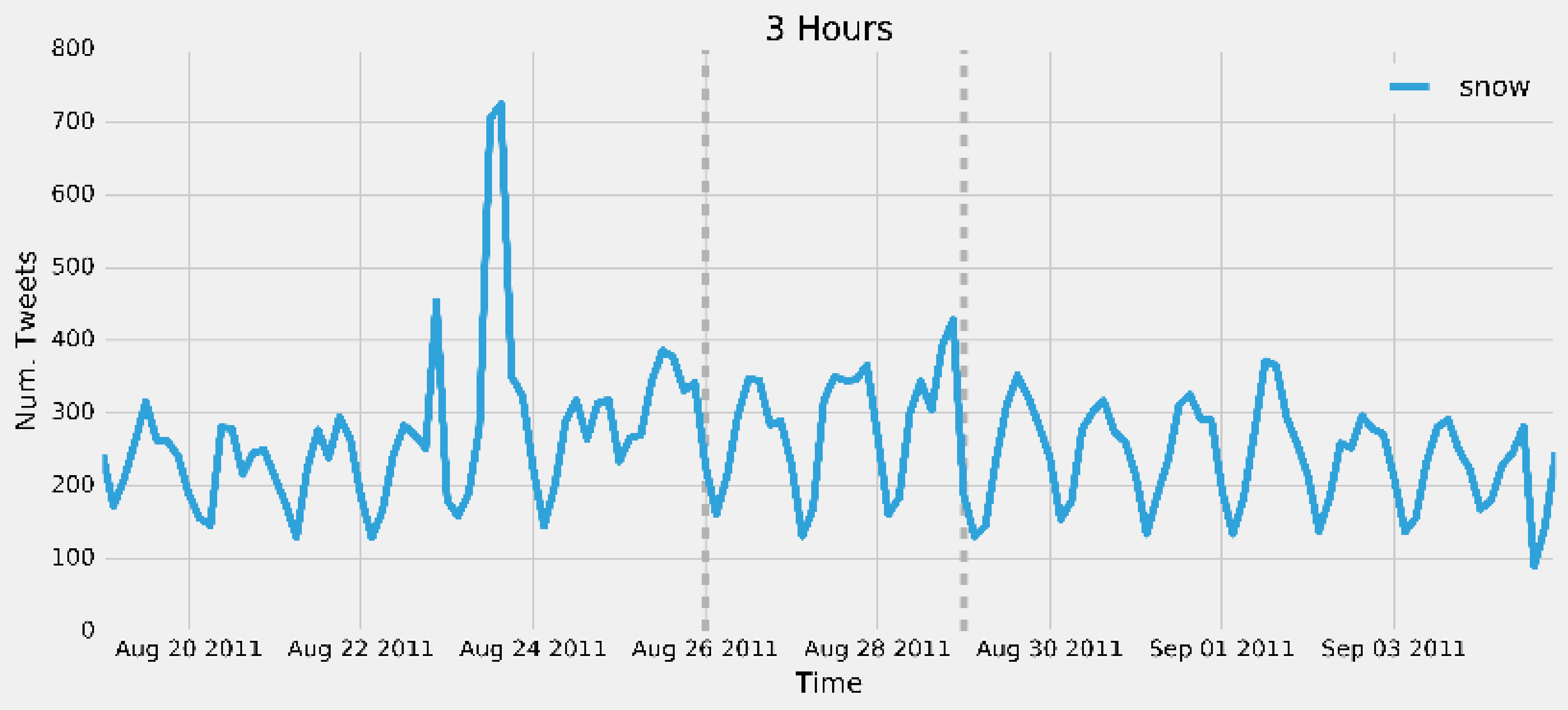

3 Hours
snow
Num. Tweets
0
100
200
300
400
500
600
700
800
Aug 20 2011
Aug 22 2011
Aug 24 2011
Aug 26 2011
Aug 28 2011
Aug 30 2011
Sep 01 2011
Sep 03 2011
Time

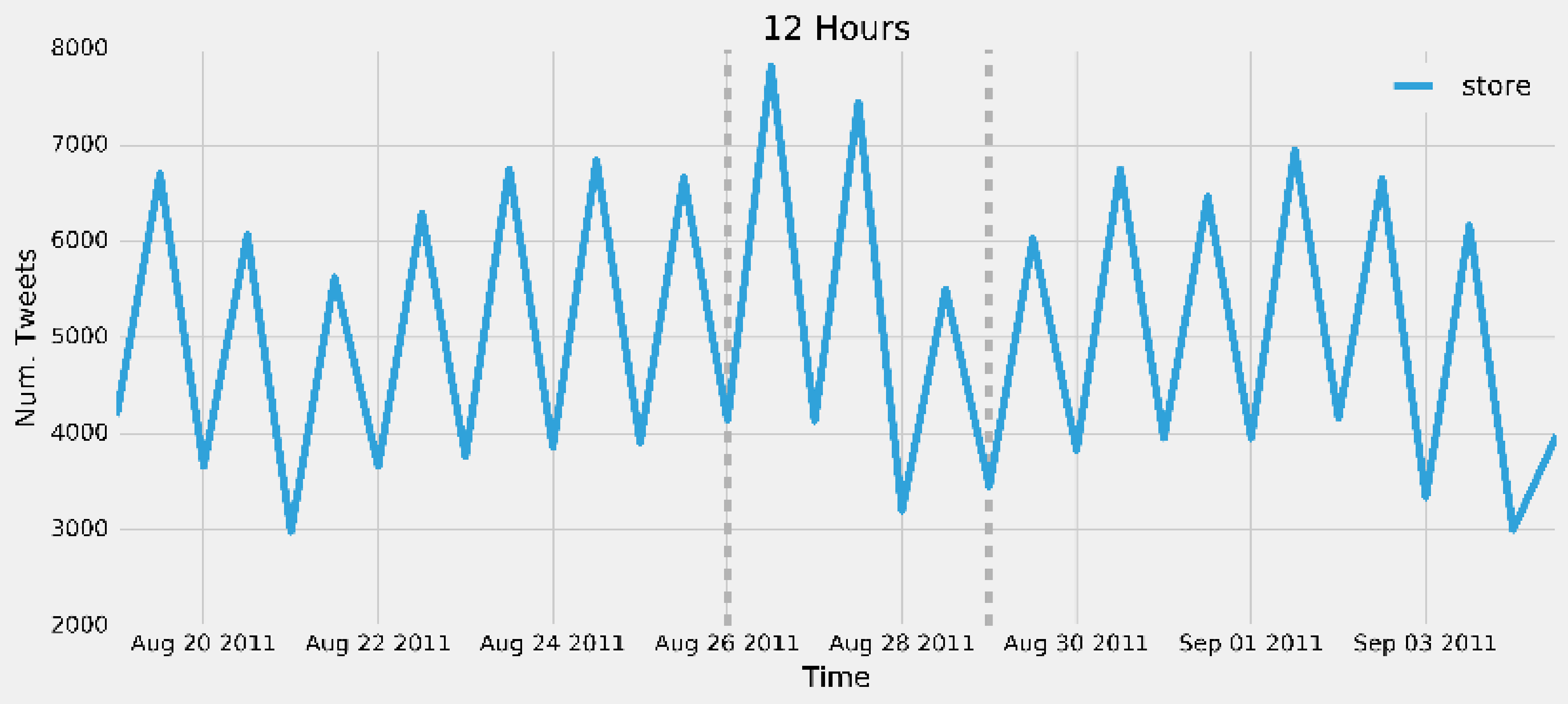

12 Hours
store
Num. Tweets
Time
8000
7000
6000
5000
4000
3000
2000
Aug 20 2011
Aug 22 2011
Aug 24 2011
Aug 26 2011
Aug 28 2011
Aug 30 2011
Sep 01 2011
Sep 03 2011

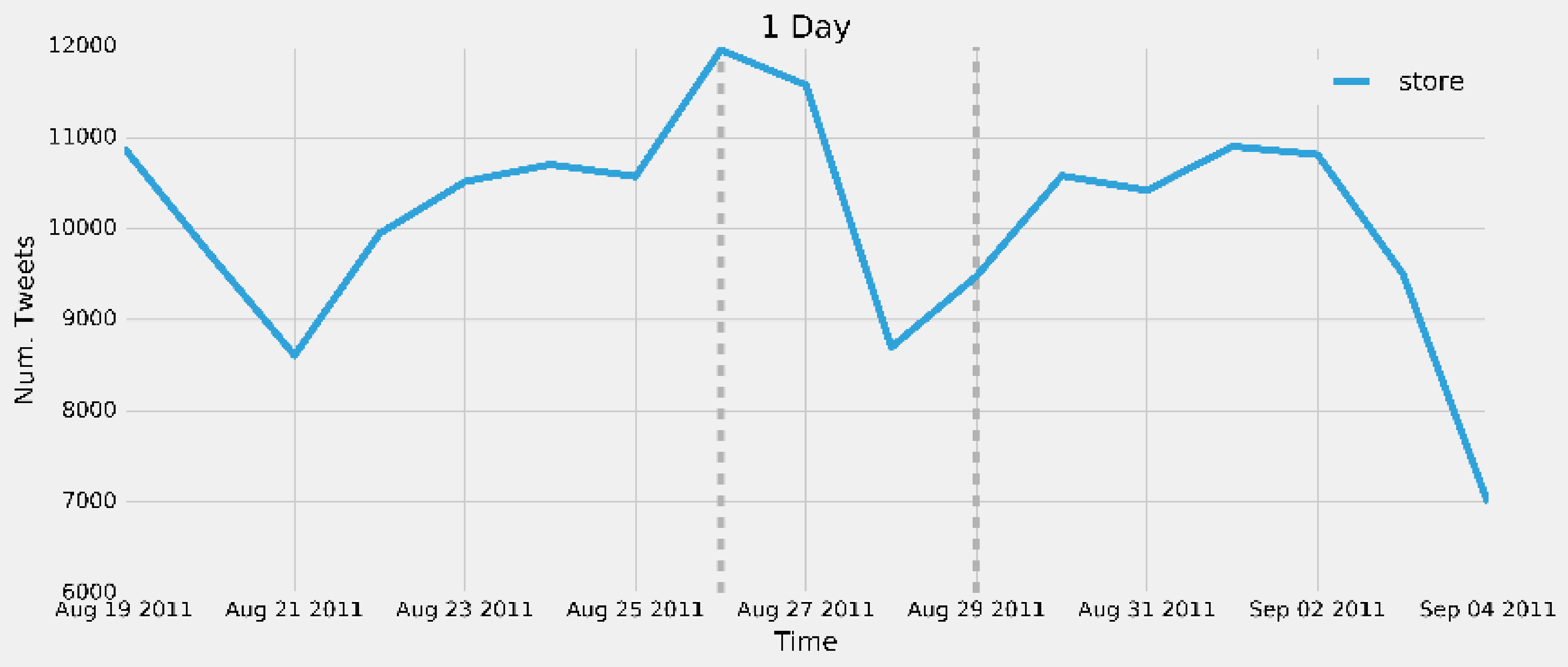
1 Day
store
Num. Tweets
12000
11000
10000
9000
8000
7000
6000
Aug 19 2011
Aug 21 2011
Aug 23 2011
Aug 25 2011
Aug 27 2011
Aug 29 2011
Aug 31 2011
Sep 02 2011
Sep 04 2011
Time

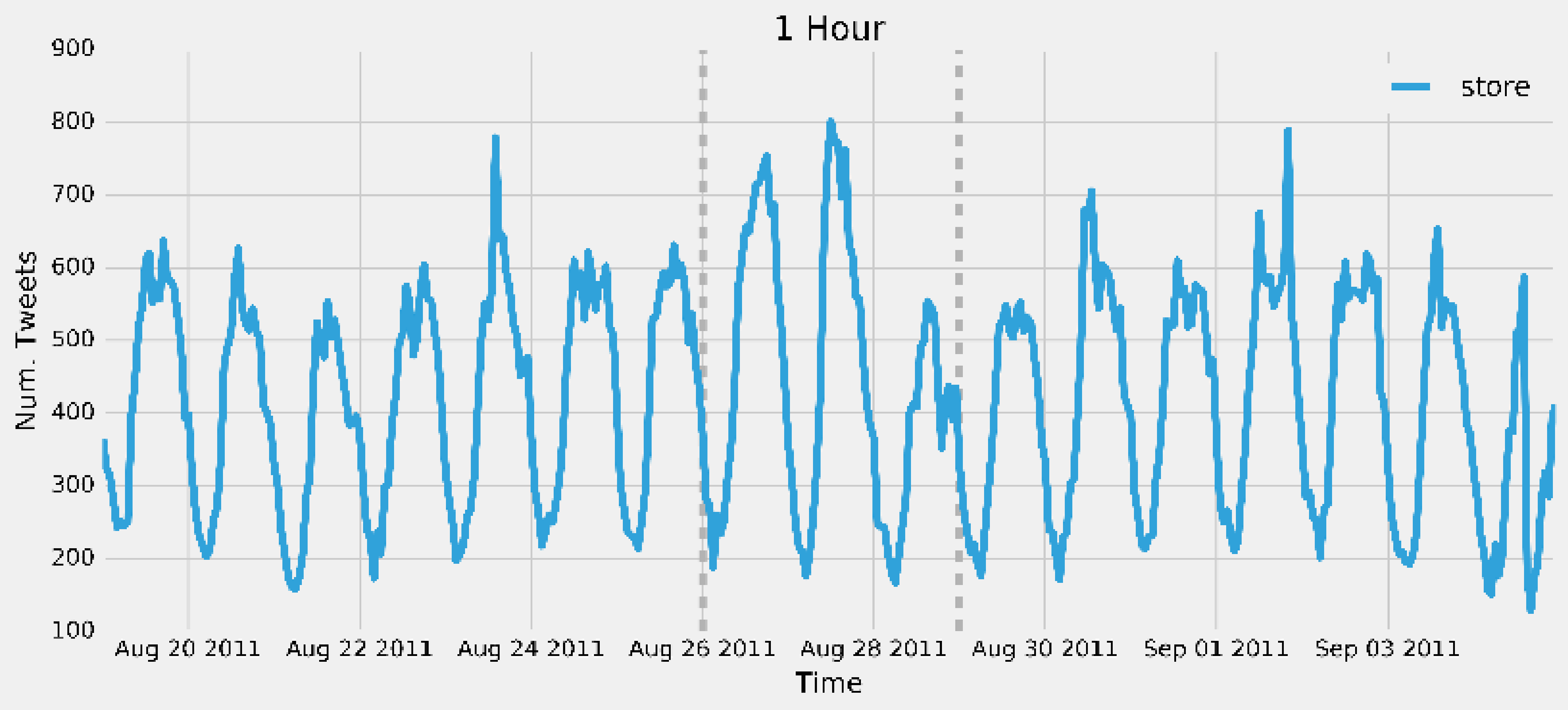
1 Hour
store
900
800
700
600
500
400
300
200
100
Num. Tweets
Aug 20 2011
Aug 22 2011
Aug 24 2011
Aug 26 2011
Aug 28 2011
Aug 30 2011
Sep 01 2011
Sep 03 2011
Time

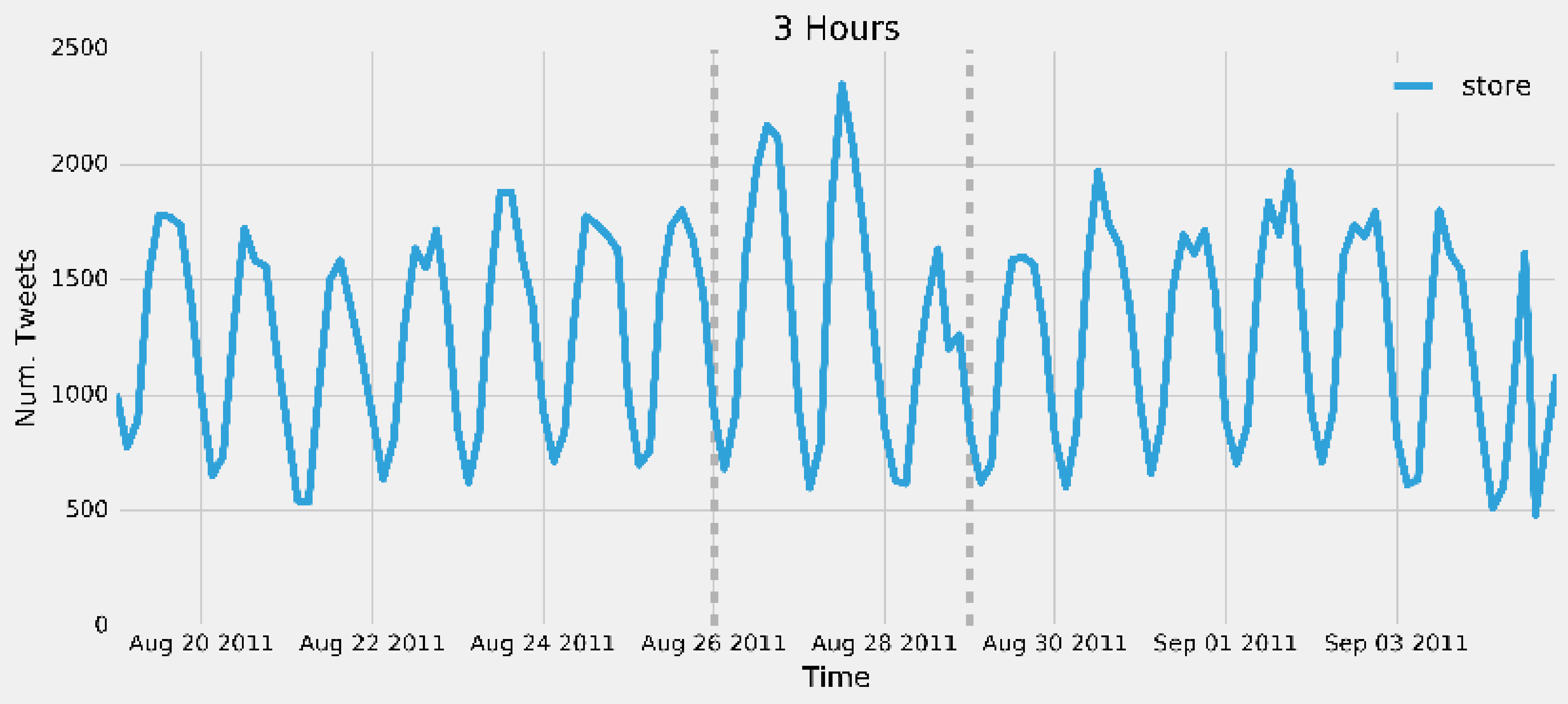
3 Hours
store
Num. Tweets
0
500
1000
1500
2000
2500
Aug 20 2011
Aug 22 2011
Aug 24 2011
Aug 26 2011
Aug 28 2011
Aug 30 2011
Sep 01 2011
Sep 03 2011
Time

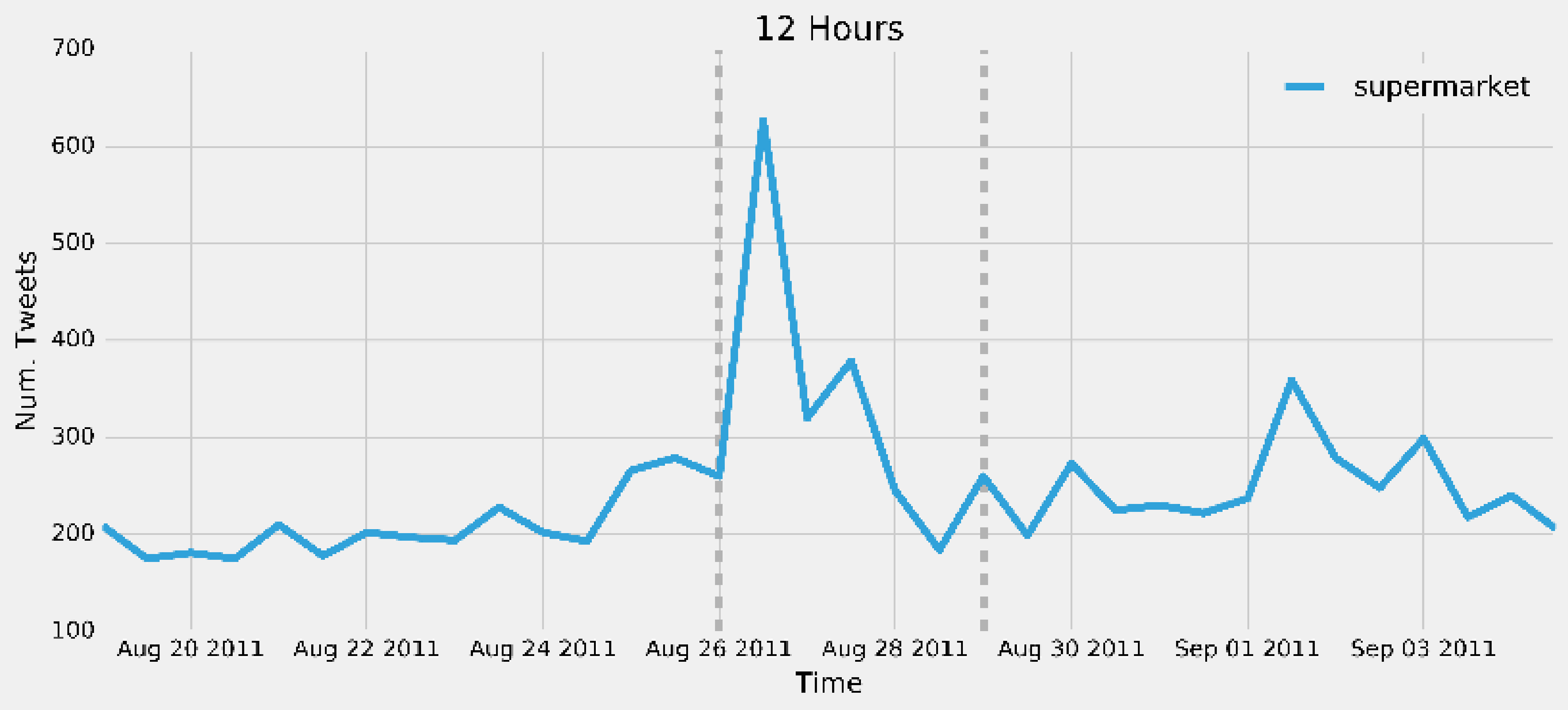
12 Hours
supermarket
Num. Tweets
Time
700
600
500
400
300
200
100
Aug 20 2011
Aug 22 2011
Aug 24 2011
Aug 26 2011
Aug 28 2011
Aug 30 2011
Sep 01 2011
Sep 03 2011

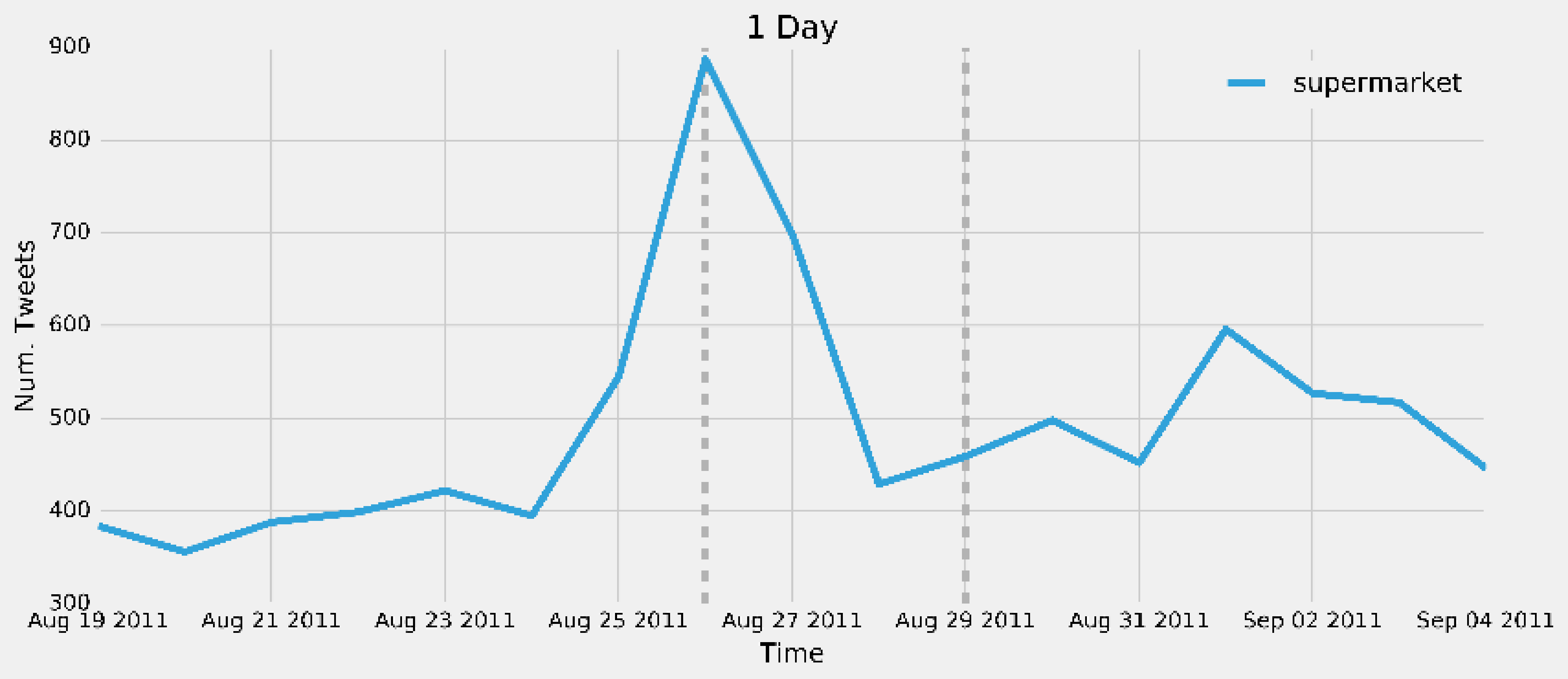

1 Day
supermarket
Num. Tweets
900
800
700
600
500
400
300
Aug 19 2011
Aug 21 2011
Aug 23 2011
Aug 25 2011
Aug 27 2011
Aug 29 2011
Aug 31 2011
Sep 02 2011
Sep 04 2011
Time

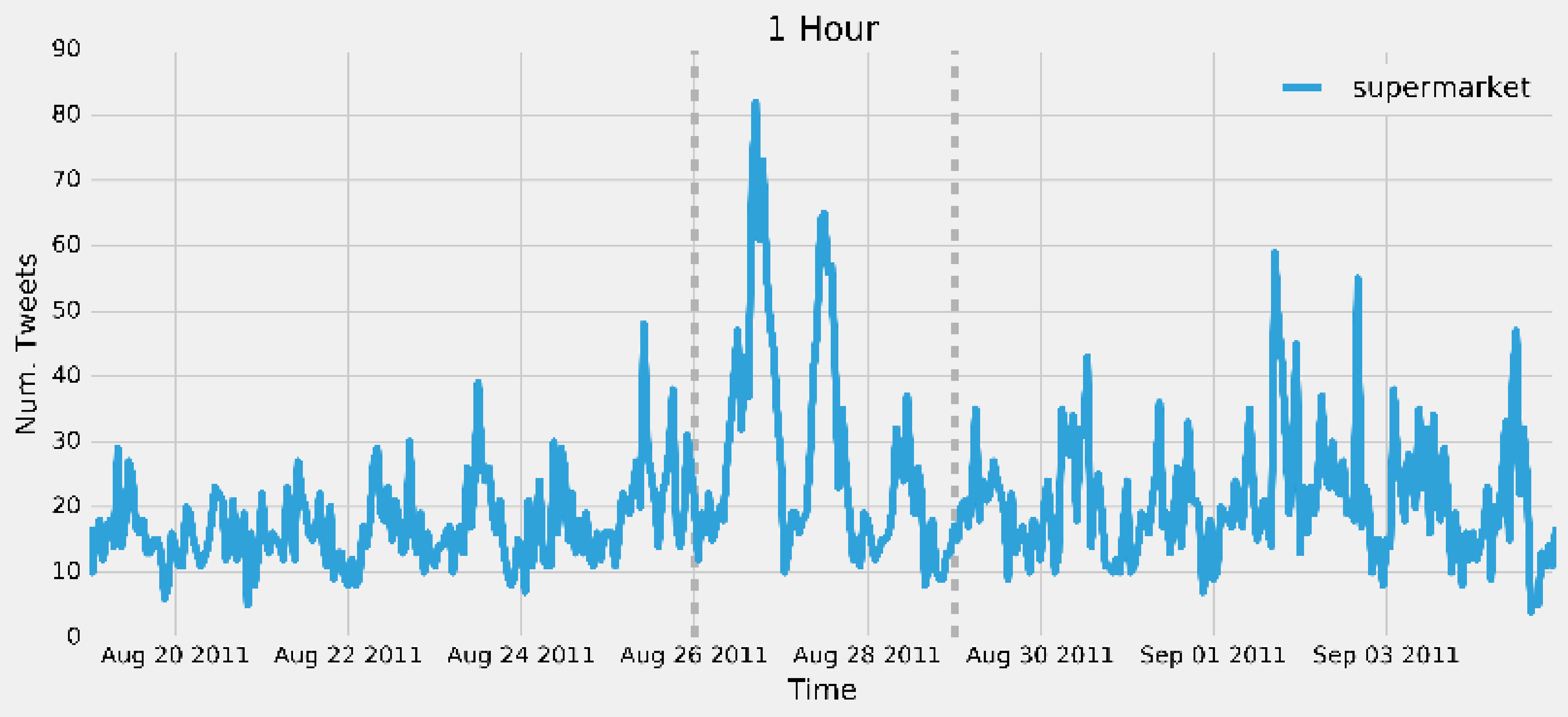
1 Hour
supermarket
Num. Tweets
Time
0
10
20
30
40
50
60
70
80
90
Aug 20 2011
Aug 22 2011
Aug 24 2011
Aug 26 2011
Aug 28 2011
Aug 30 2011
Sep 01 2011
Sep 03 2011

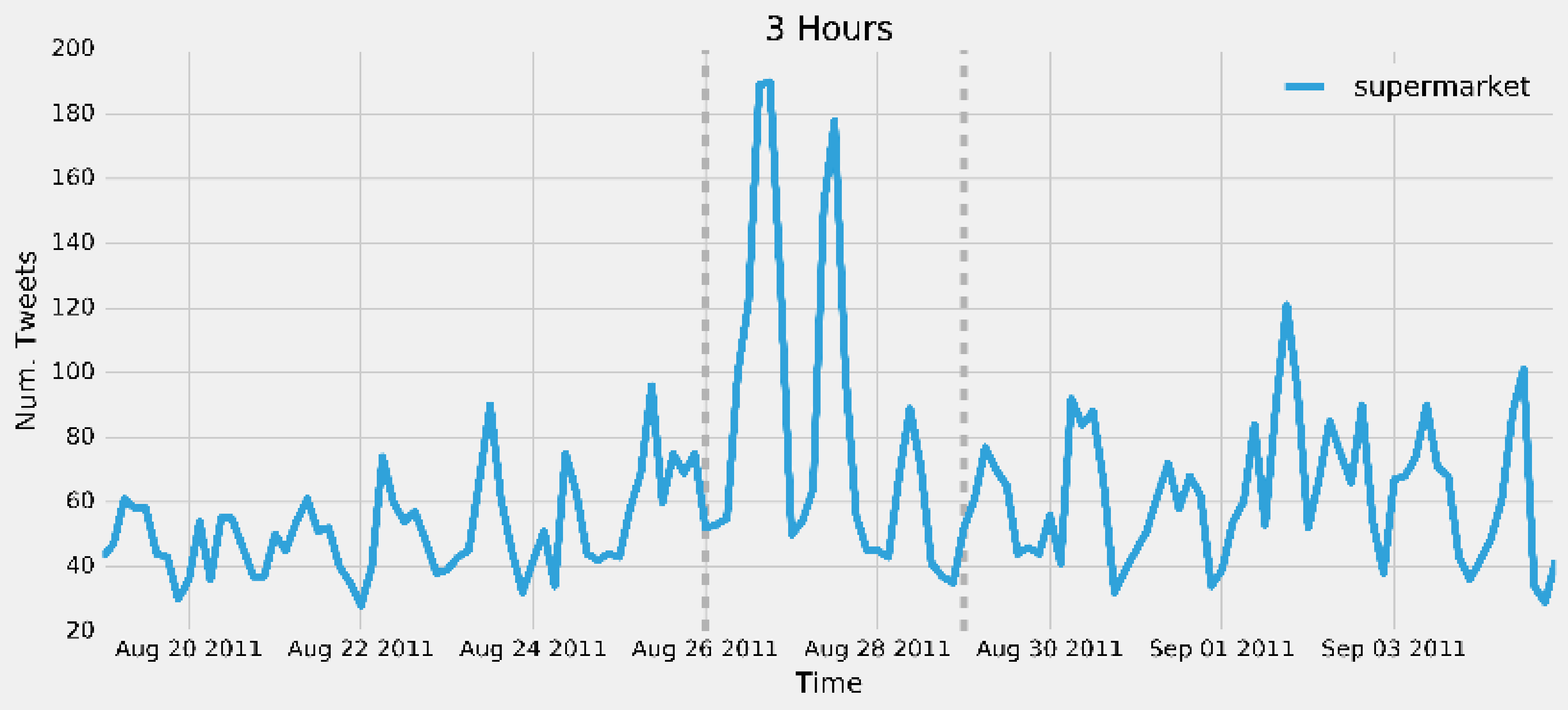
3 Hours
supermarket
Num. Tweets
200
180
160
140
120
100
80
60
40
20
Aug 20 2011
Aug 22 2011
Aug 24 2011
Aug 26 2011
Aug 28 2011
Aug 30 2011
Sep 01 2011
Sep 03 2011
Time

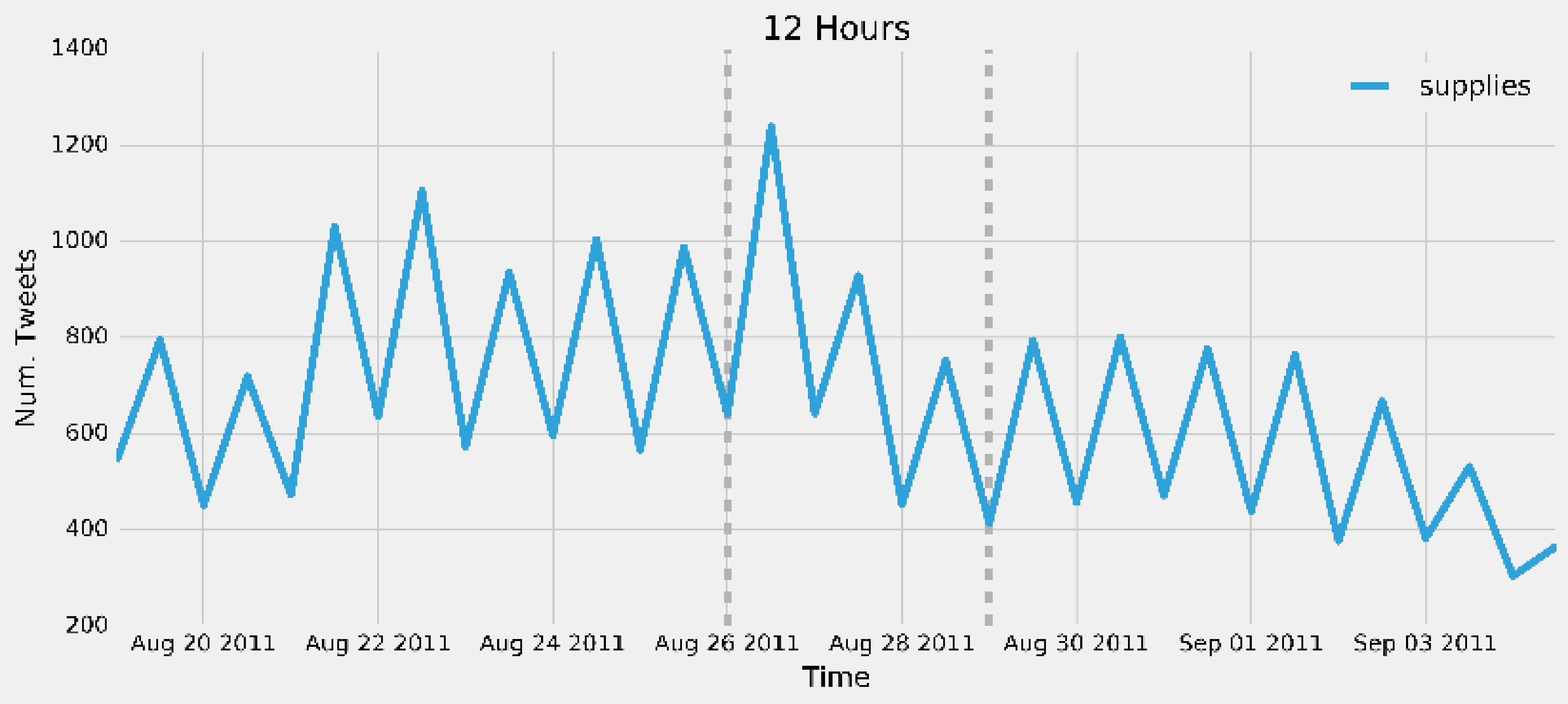
12 Hours
supplies
Num. Tweets
1400
1200
1000
800
600
400
200
Aug 20 2011
Aug 22 2011
Aug 24 2011
Aug 26 2011
Aug 28 2011
Aug 30 2011
Sep 01 2011
Sep 03 2011
Time

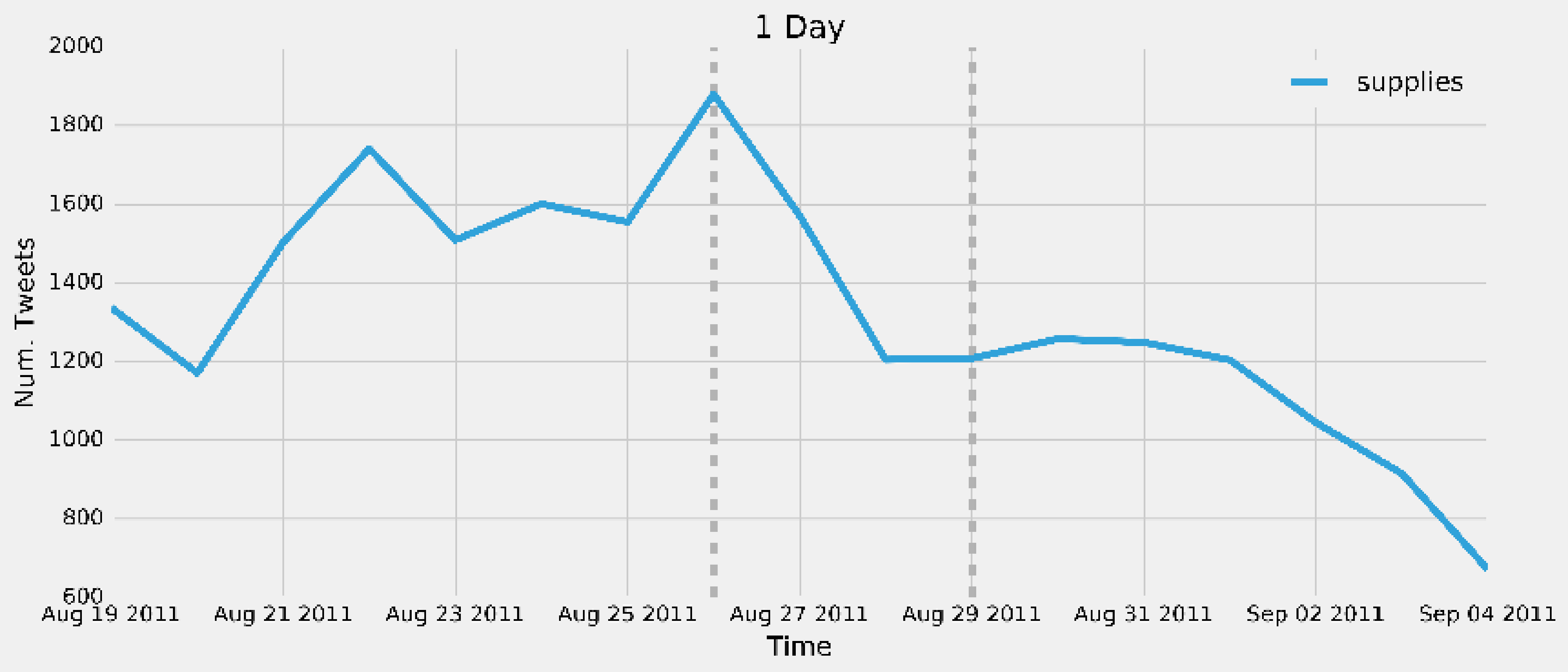
1 Day
supplies
Num. Tweets
Time
2000
1800
1600
1400
1200
1000
800
600
Aug 19 2011
Aug 21 2011
Aug 23 2011
Aug 25 2011
Aug 27 2011
Aug 29 2011
Aug 31 2011
Sep 02 2011
Sep 04 2011

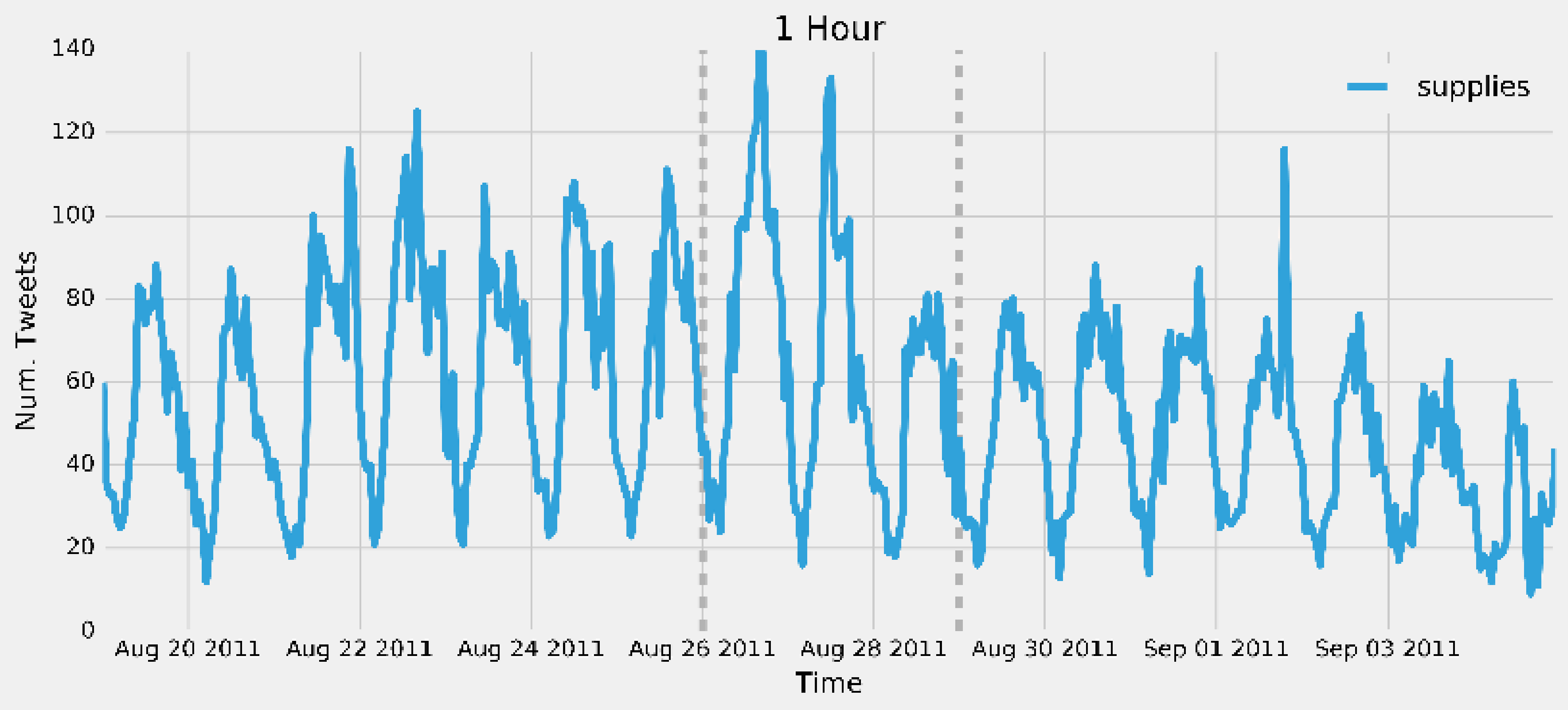
1 Hour
supplies
Num. Tweets
0
20
40
60
80
100
120
140
Aug 20 2011
Aug 22 2011
Aug 24 2011
Aug 26 2011
Aug 28 2011
Aug 30 2011
Sep 01 2011
Sep 03 2011
Time

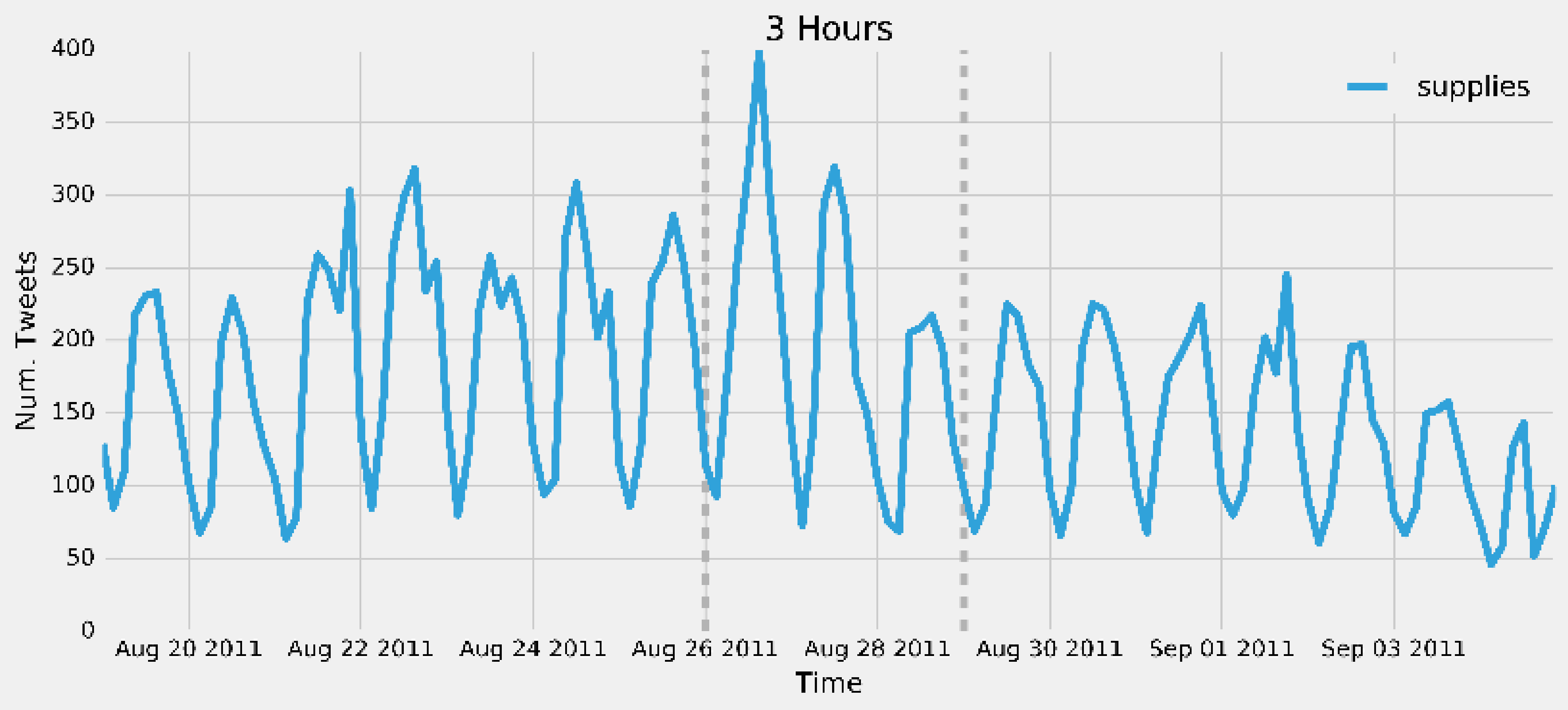
3 Hours
supplies
Num. Tweets
0
50
100
150
200
250
300
350
400
Aug 20 2011
Aug 22 2011
Aug 24 2011
Aug 26 2011
Aug 28 2011
Aug 30 2011
Sep 01 2011
Sep 03 2011
Time

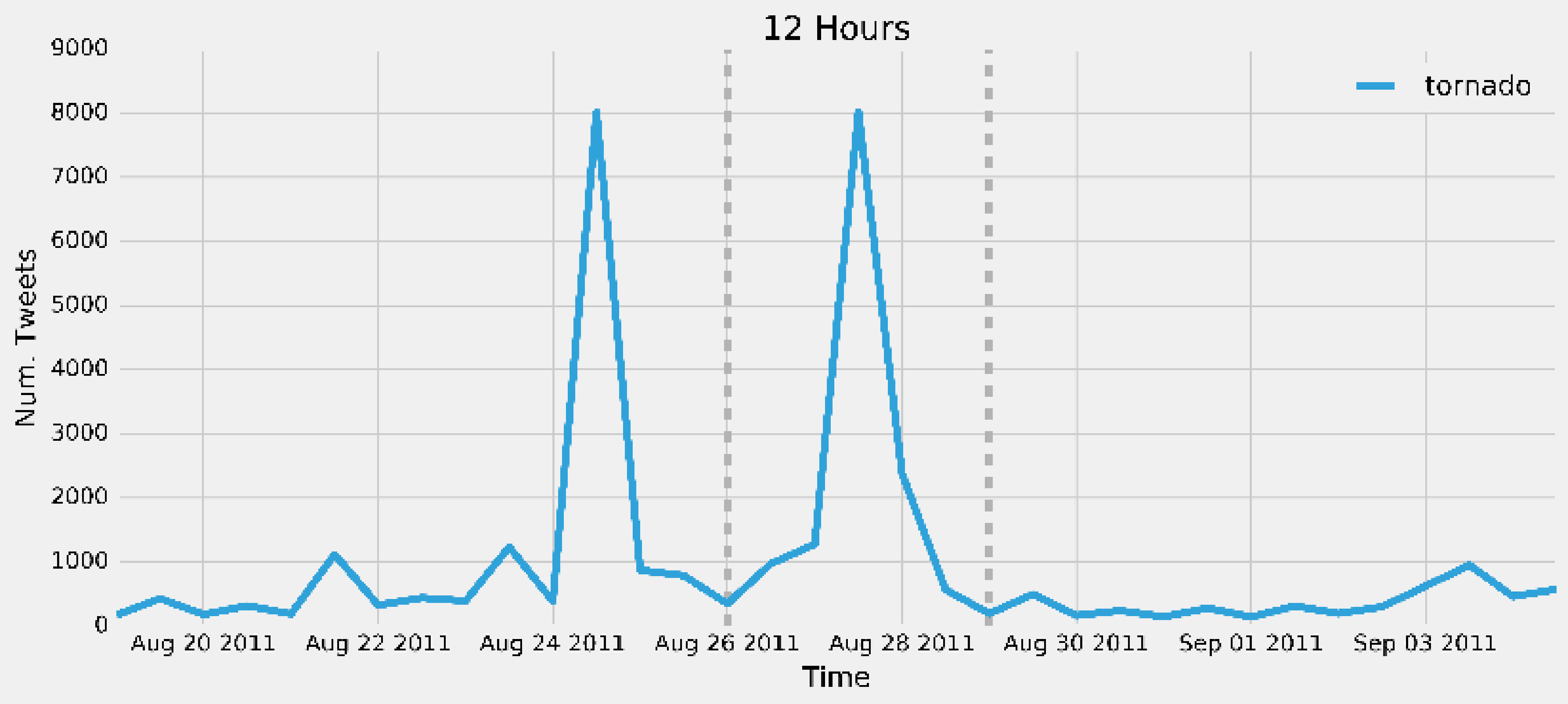

12 Hours
9000
8000
7000
6000
5000
4000
3000
2000
1000
0
Num. Tweets
tornado
Aug 20 2011
Aug 22 2011
Aug 24 2011
Aug 26 2011
Aug 28 2011
Aug 30 2011
Sep 01 2011
Sep 03 2011
Time

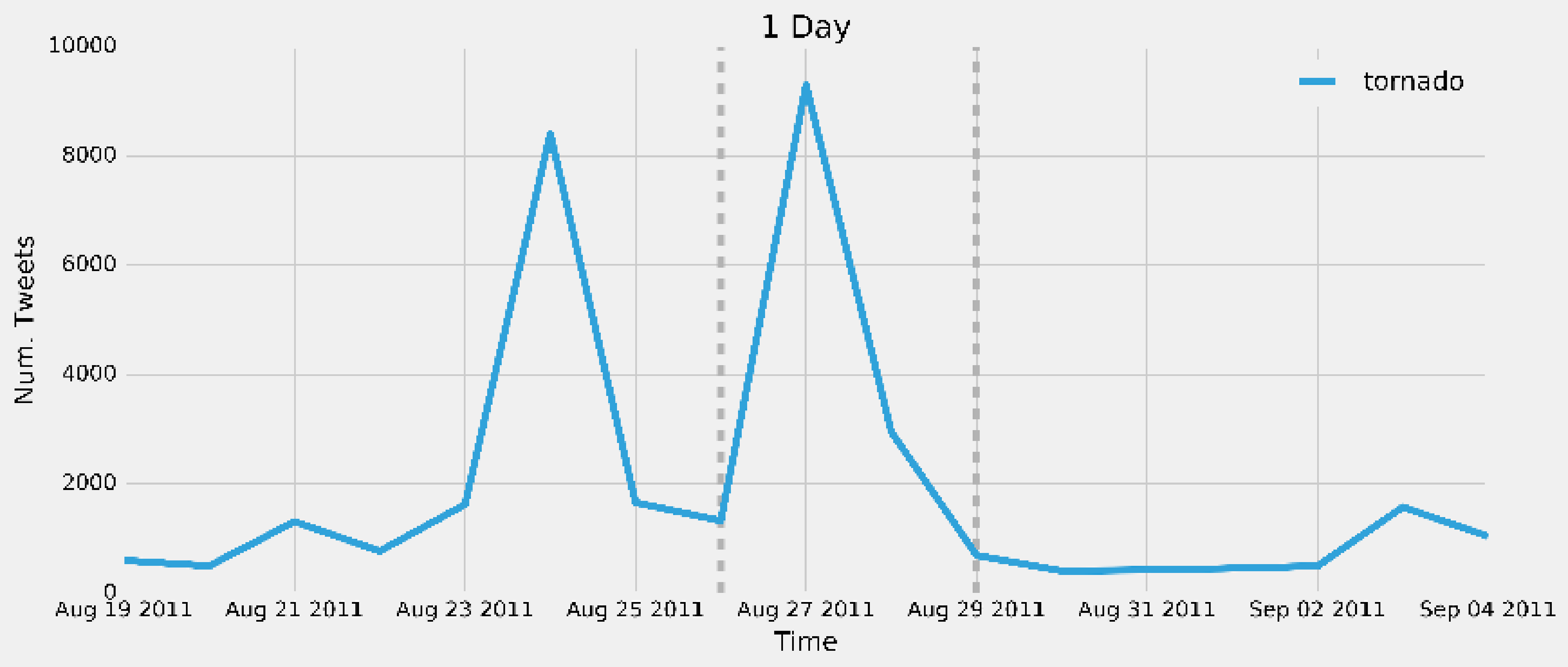

1 Day
tornado
10000
8000
6000
4000
2000
0
Num. Tweets
Aug 19 2011
Aug 21 2011
Aug 23 2011
Aug 25 2011
Aug 27 2011
Aug 29 2011
Aug 31 2011
Sep 02 2011
Sep 04 2011
Time

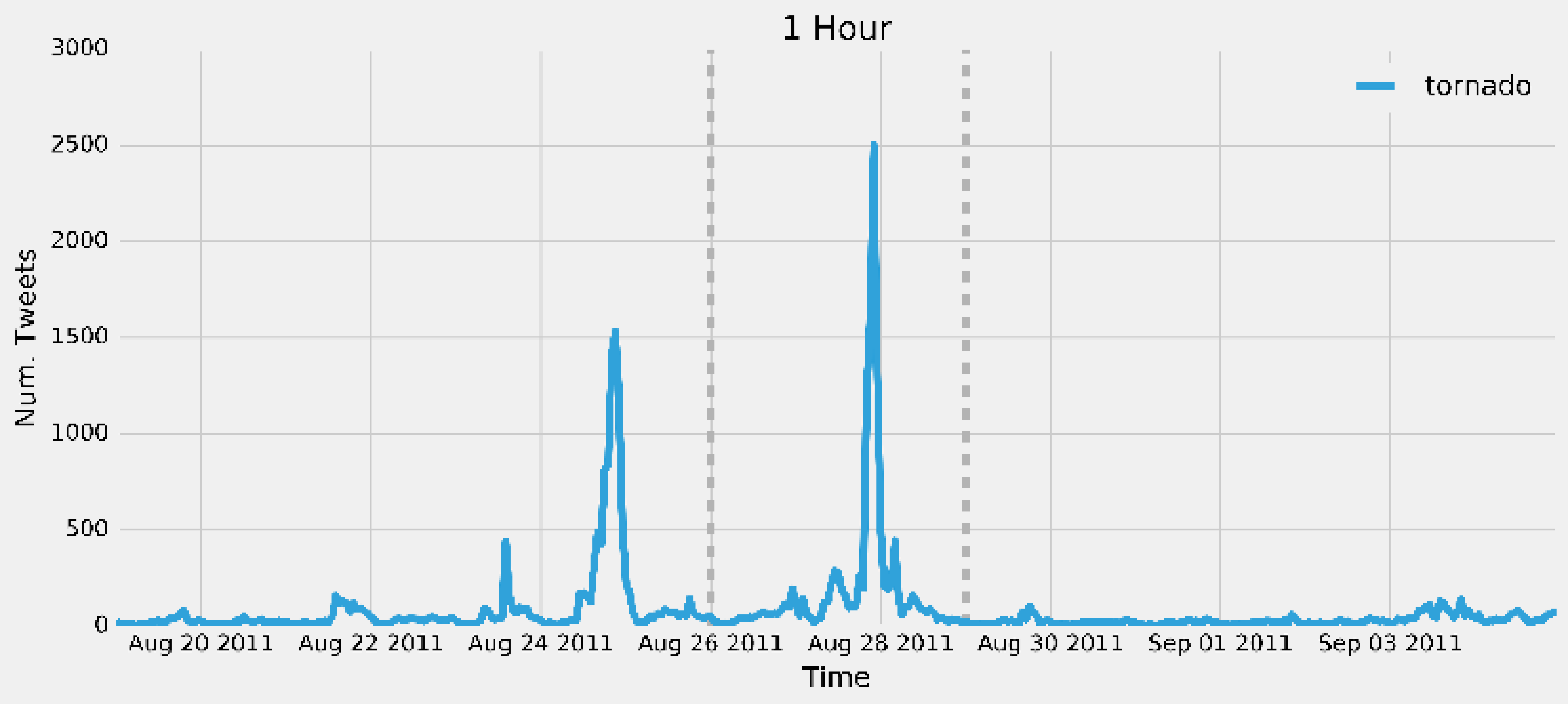
1 Hour
tornado
Num. Tweets
3000
2500
2000
1500
1000
500
0
Aug 20 2011
Aug 22 2011
Aug 24 2011
Aug 26 2011
Aug 28 2011
Aug 30 2011
Sep 01 2011
Sep 03 2011
Time

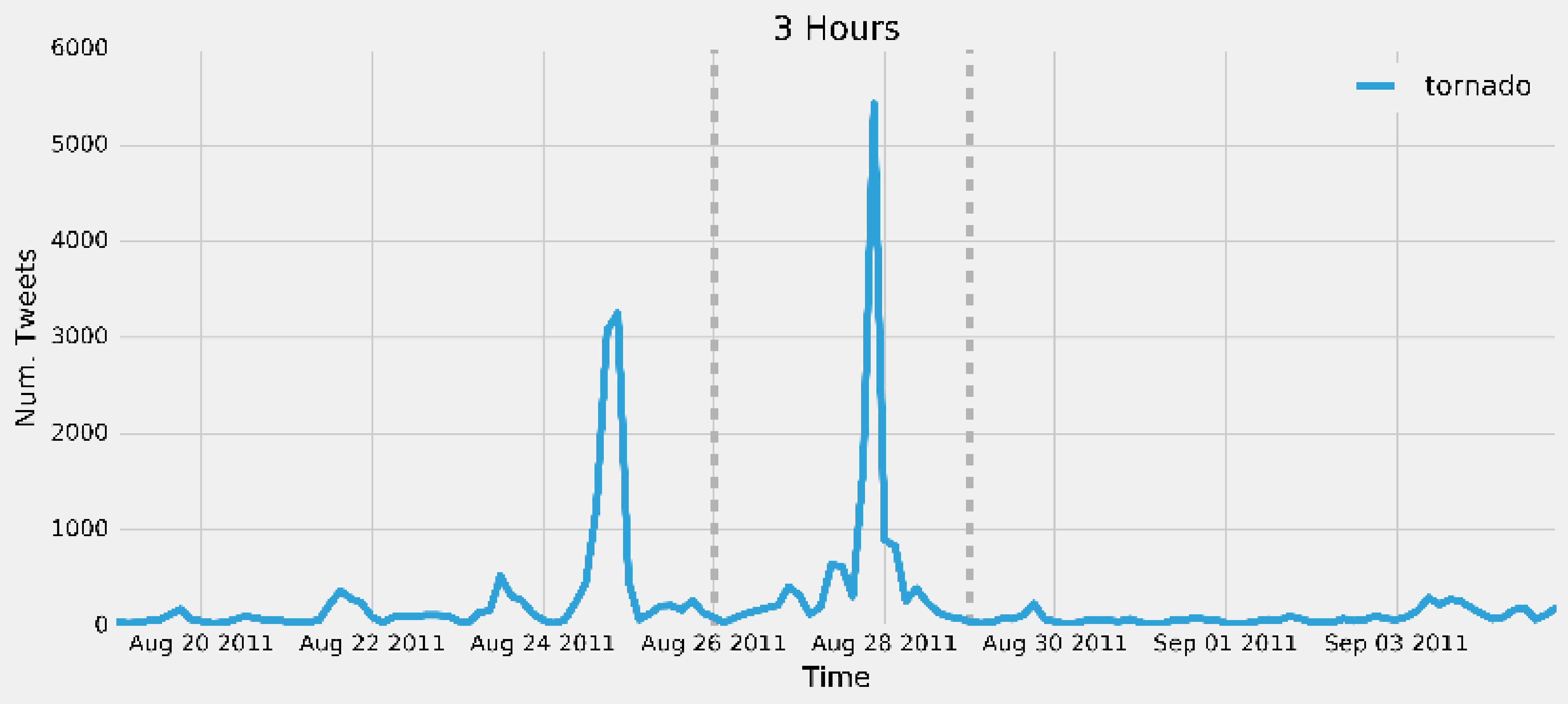

3 Hours
tornado
Num. Tweets
6000
5000
4000
3000
2000
1000
0
Aug 20 2011
Aug 22 2011
Aug 24 2011
Aug 26 2011
Aug 28 2011
Aug 30 2011
Sep 01 2011
Sep 03 2011
Time

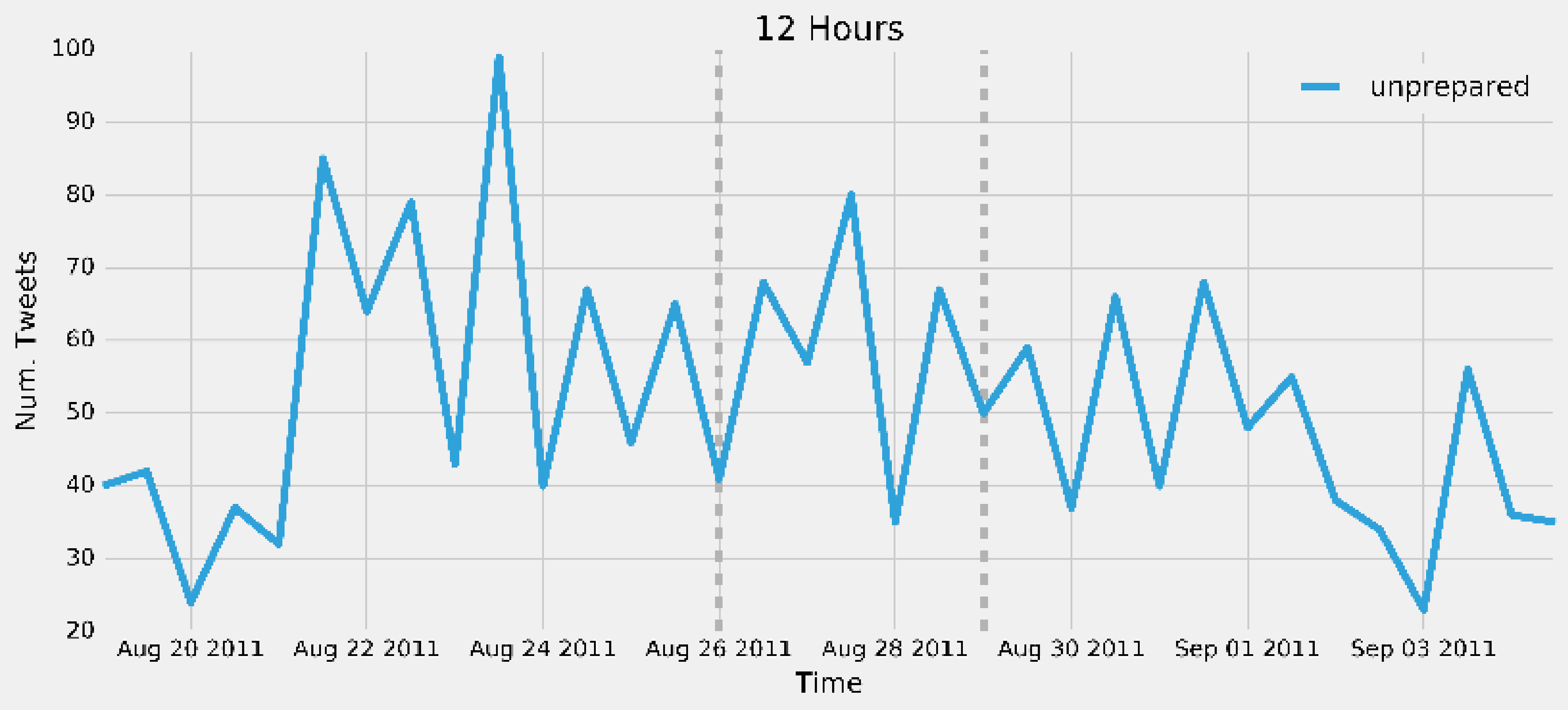
12 Hours
unprepared
Num. Tweets
Time
100
90
80
70
60
50
40
30
20
Aug 20 2011
Aug 22 2011
Aug 24 2011
Aug 26 2011
Aug 28 2011
Aug 30 2011
Sep 01 2011
Sep 03 2011

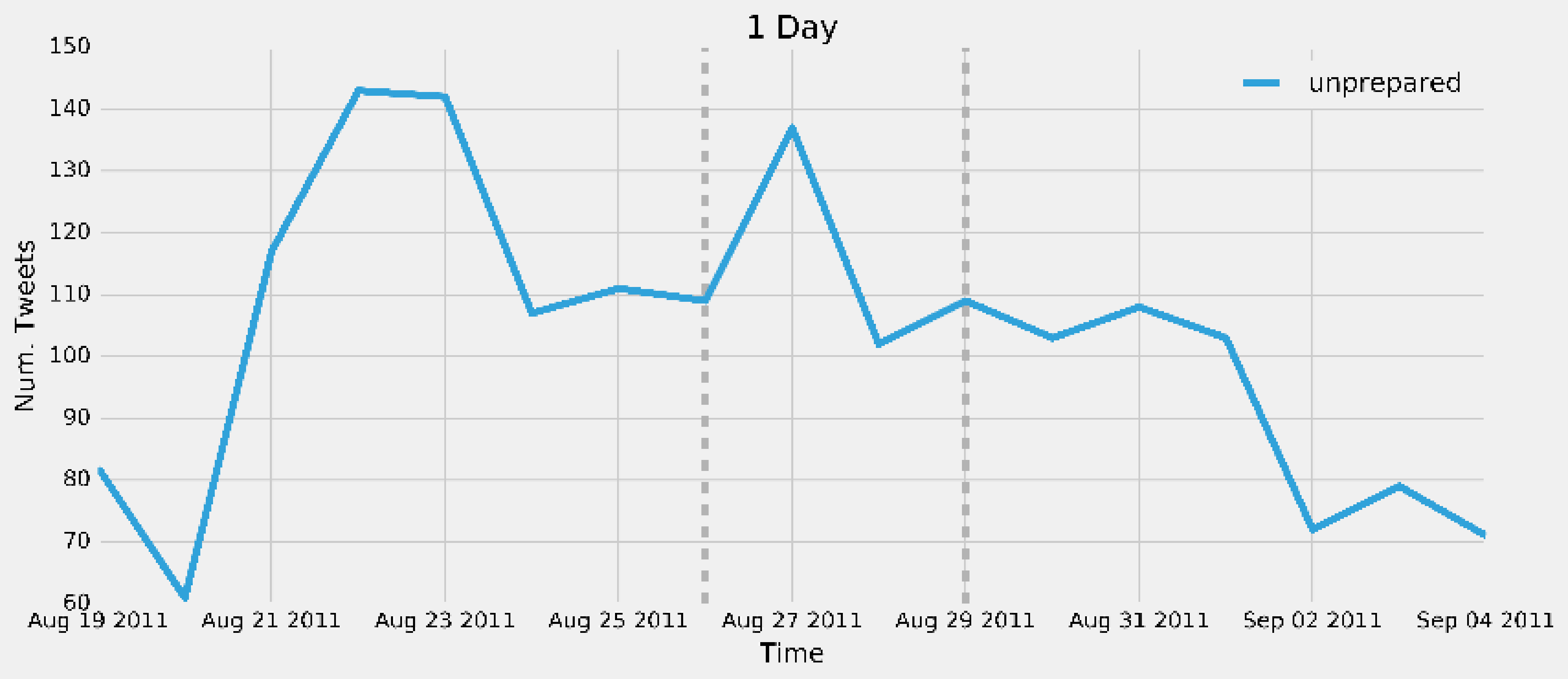

1 Day
unprepared
Num. Tweets
150
140
130
120
110
100
90
80
70
60
Aug 19 2011
Aug 21 2011
Aug 23 2011
Aug 25 2011
Aug 27 2011
Aug 29 2011
Aug 31 2011
Sep 02 2011
Sep 04 2011
Time

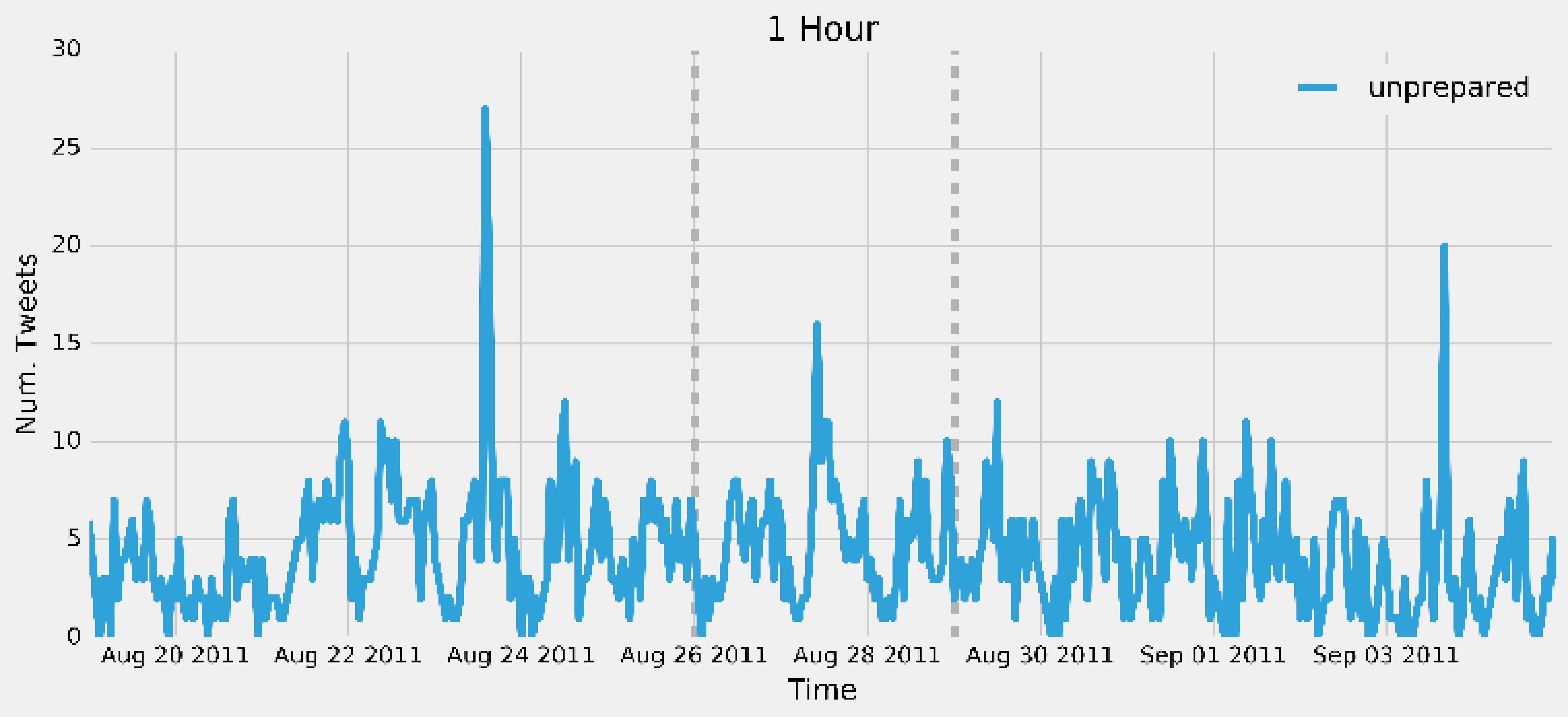
1 Hour
unprepared
Num. Tweets
Time
0
5
10
15
20
25
30
Aug 20 2011
Aug 22 2011
Aug 24 2011
Aug 26 2011
Aug 28 2011
Aug 30 2011
Sep 01 2011
Sep 03 2011

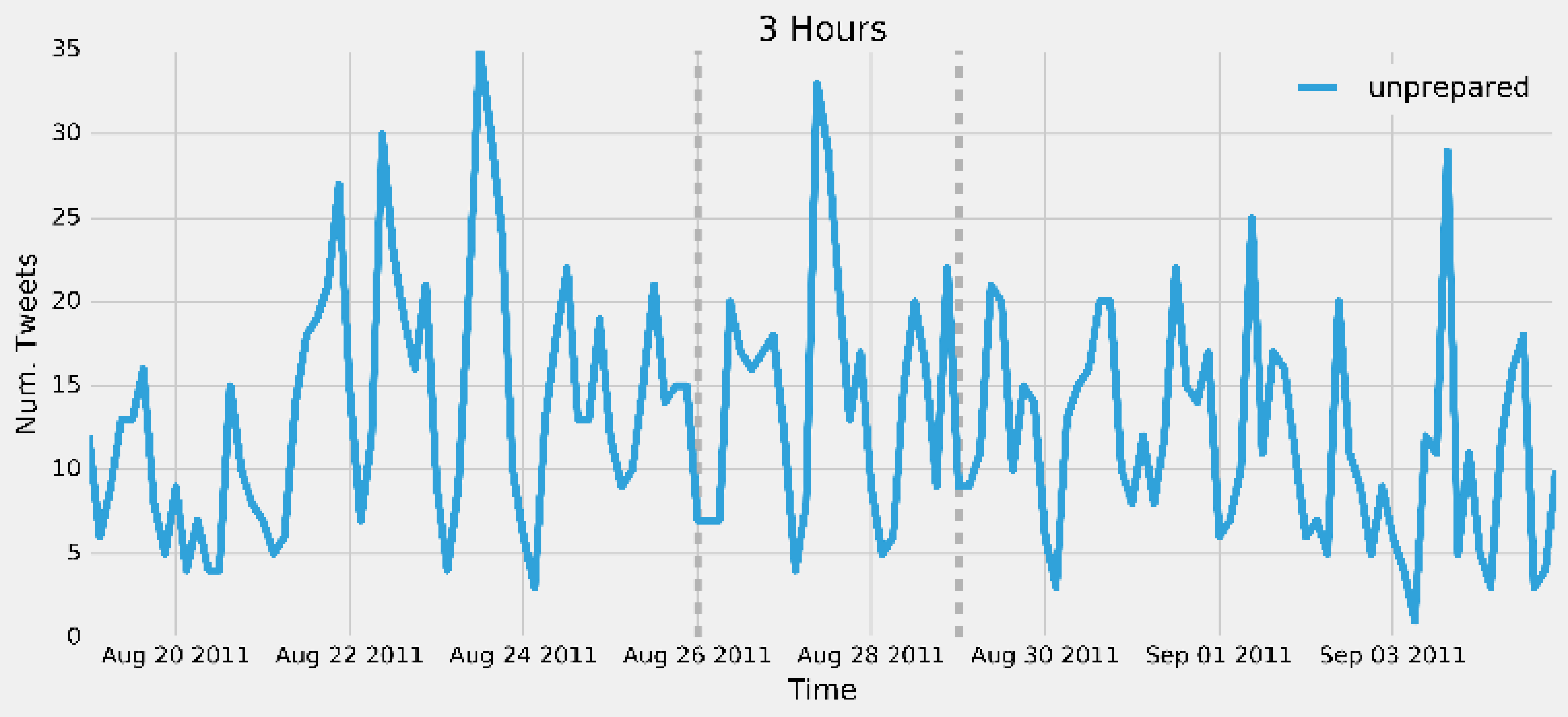
3 Hours
unprepared
Num. Tweets
0
5
10
15
20
25
30
35
Aug 20 2011
Aug 22 2011
Aug 24 2011
Aug 26 2011
Aug 28 2011
Aug 30 2011
Sep 01 2011
Sep 03 2011
Time

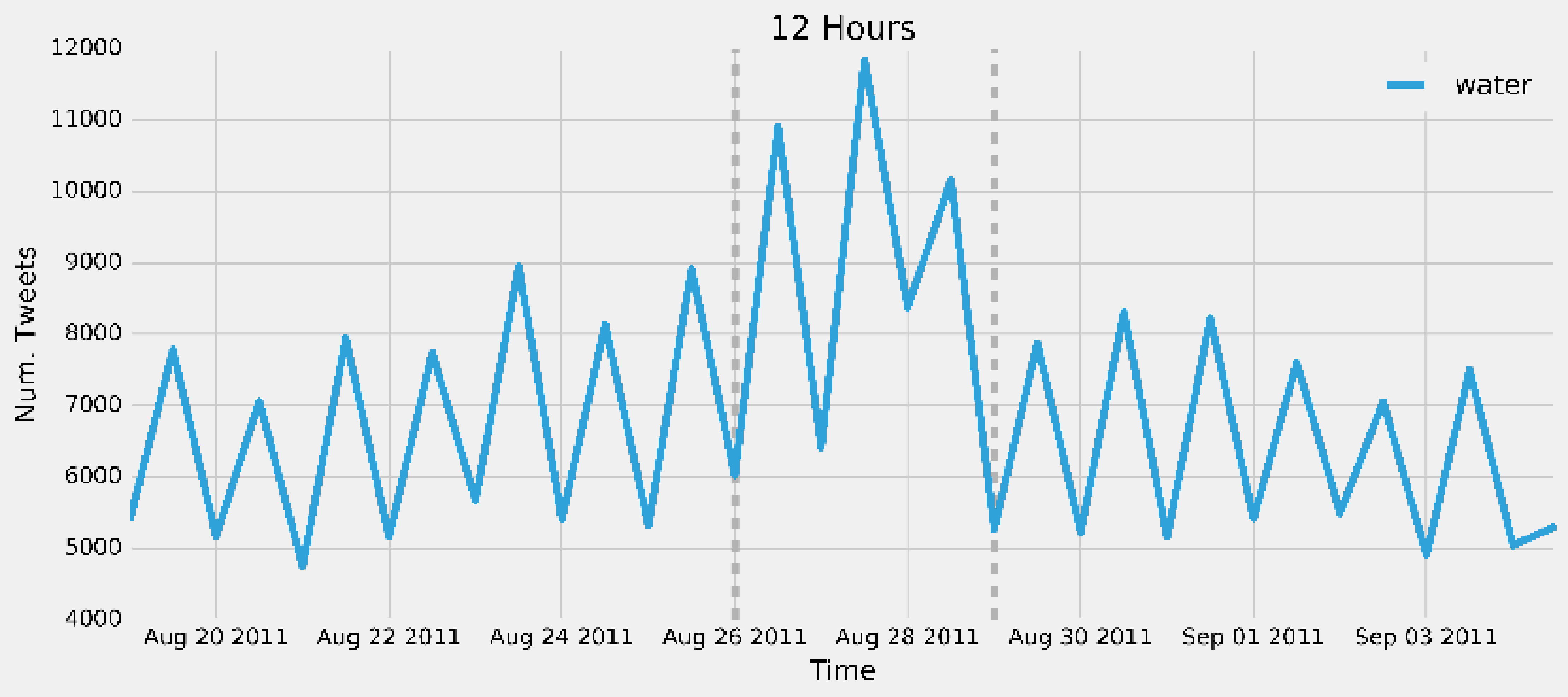

12 Hours
water
Num. Tweets
4000
5000
6000
7000
8000
9000
10000
11000
12000
Aug 20 2011
Aug 22 2011
Aug 24 2011
Aug 26 2011
Aug 28 2011
Aug 30 2011
Sep 01 2011
Sep 03 2011
Time

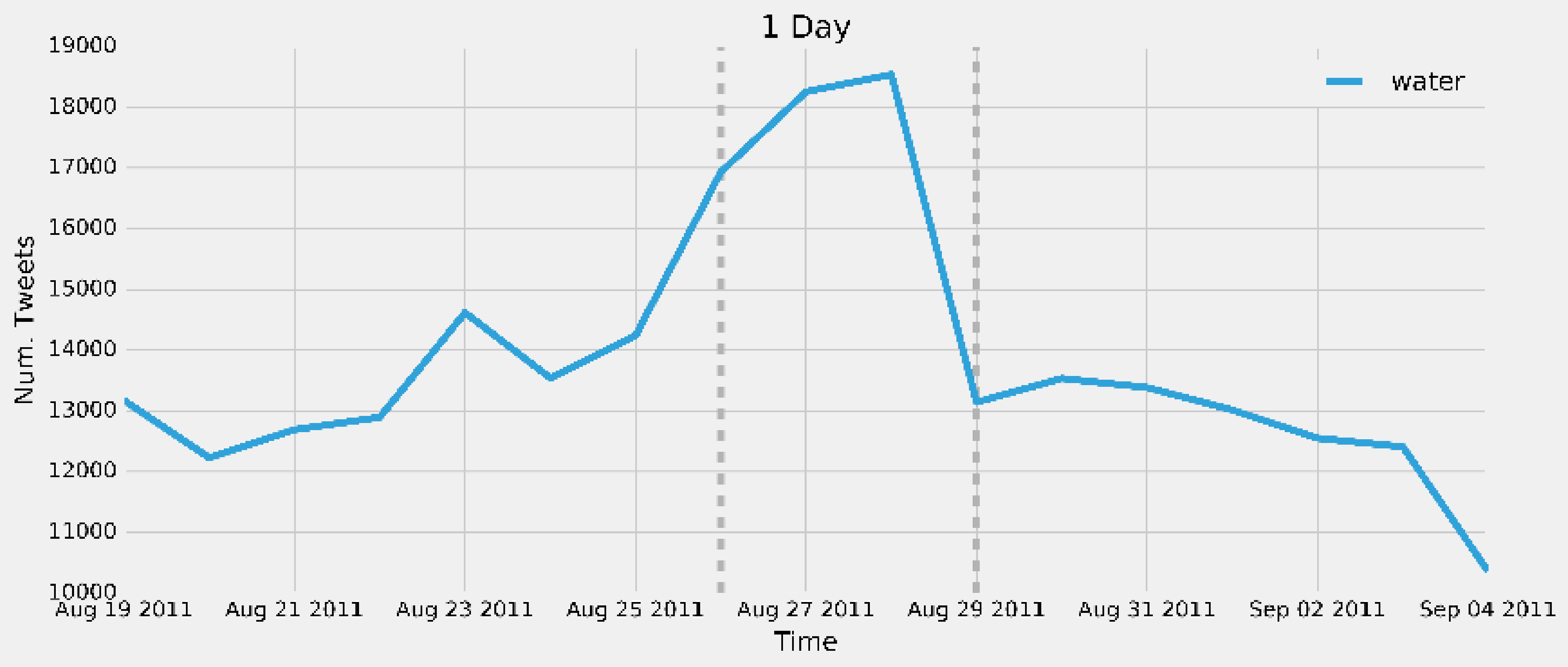
1 Day
water
Num. Tweets
19000
18000
17000
16000
15000
14000
13000
12000
11000
10000
Aug 19 2011
Aug 21 2011
Aug 23 2011
Aug 25 2011
Aug 27 2011
Aug 29 2011
Aug 31 2011
Sep 02 2011
Sep 04 2011
Time

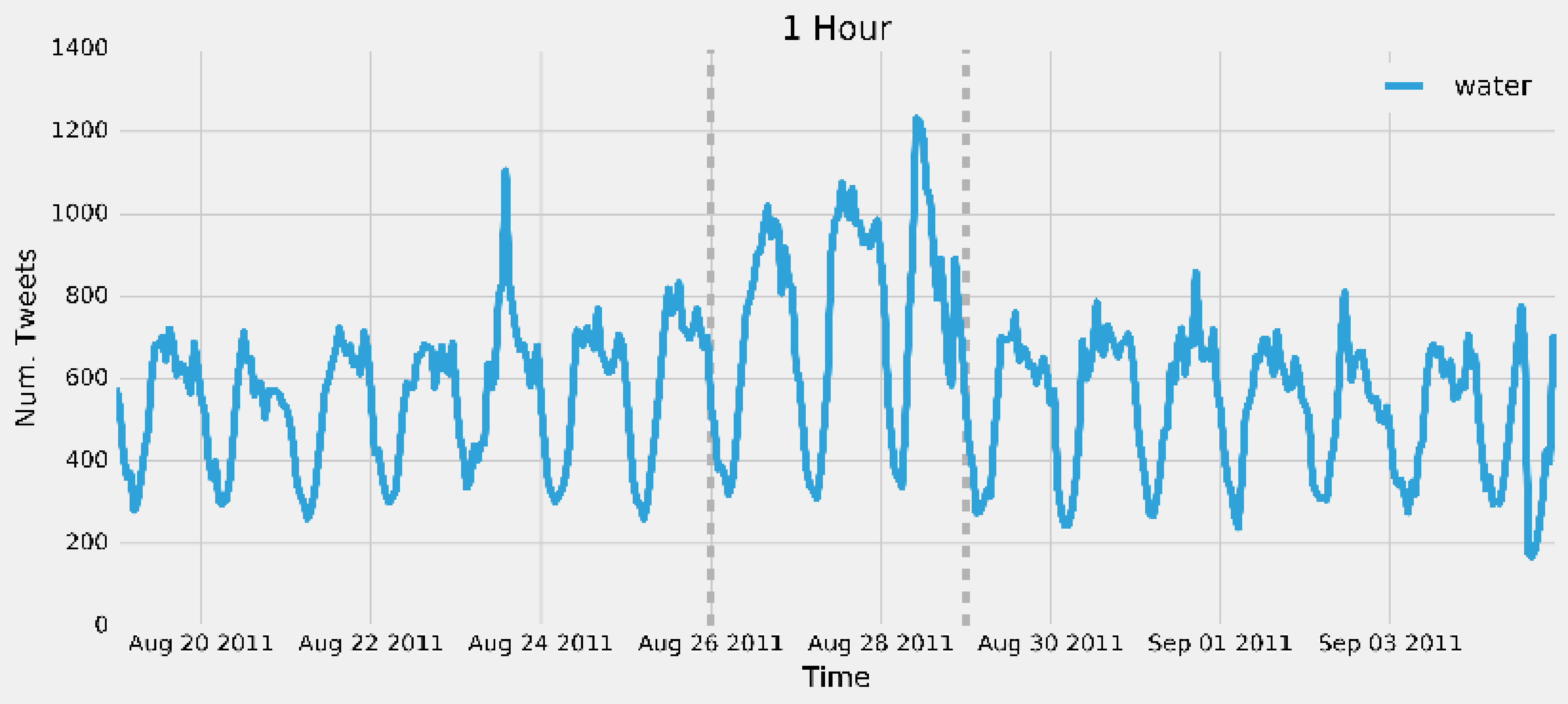
1 Hour
water
Num. Tweets
1400
1200
1000
800
600
400
200
0
Aug 20 2011
Aug 22 2011
Aug 24 2011
Aug 26 2011
Aug 28 2011
Aug 30 2011
Sep 01 2011
Sep 03 2011
Time

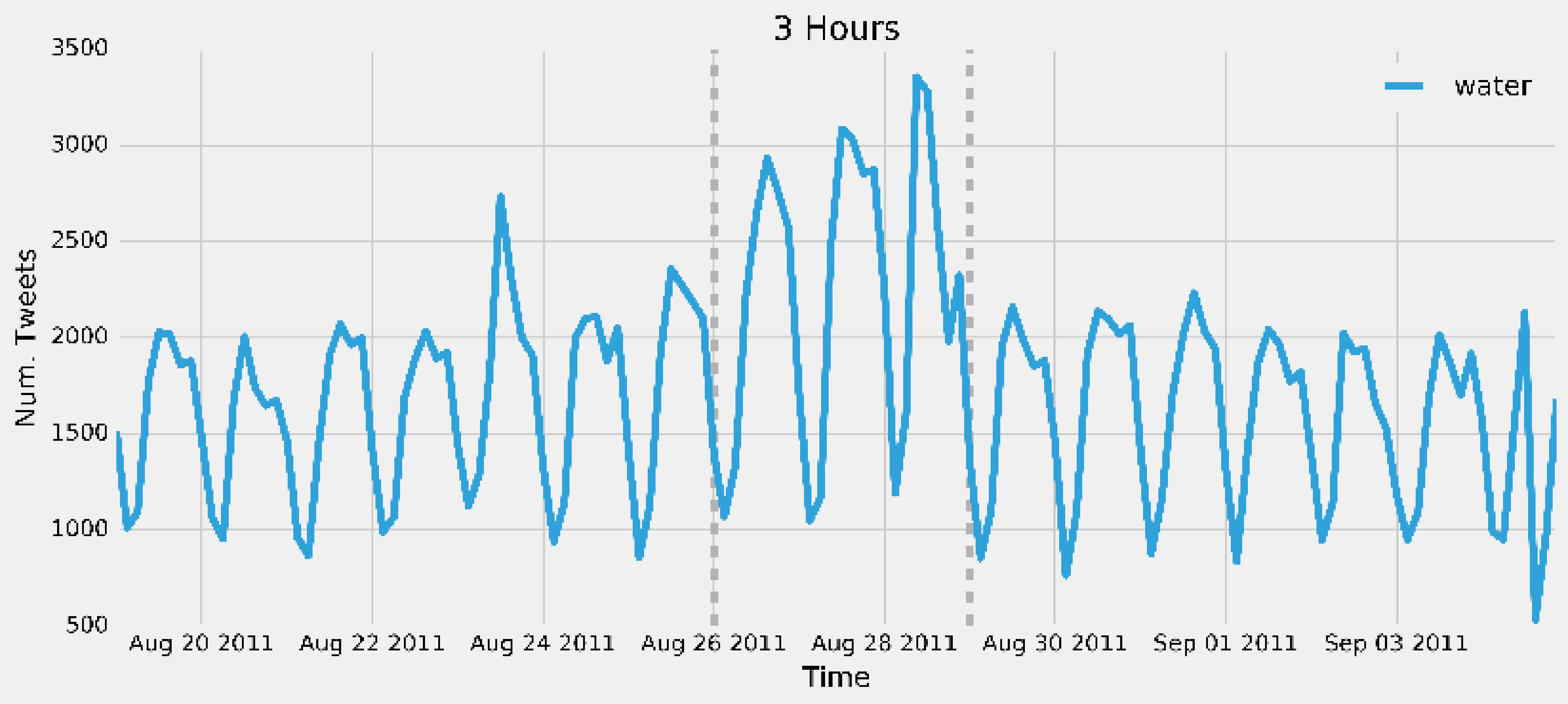
3 Hours
water
Num. Tweets
3500
3000
2500
2000
1500
1000
500
Aug 20 2011
Aug 22 2011
Aug 24 2011
Aug 26 2011
Aug 28 2011
Aug 30 2011
Sep 01 2011
Sep 03 2011
Time

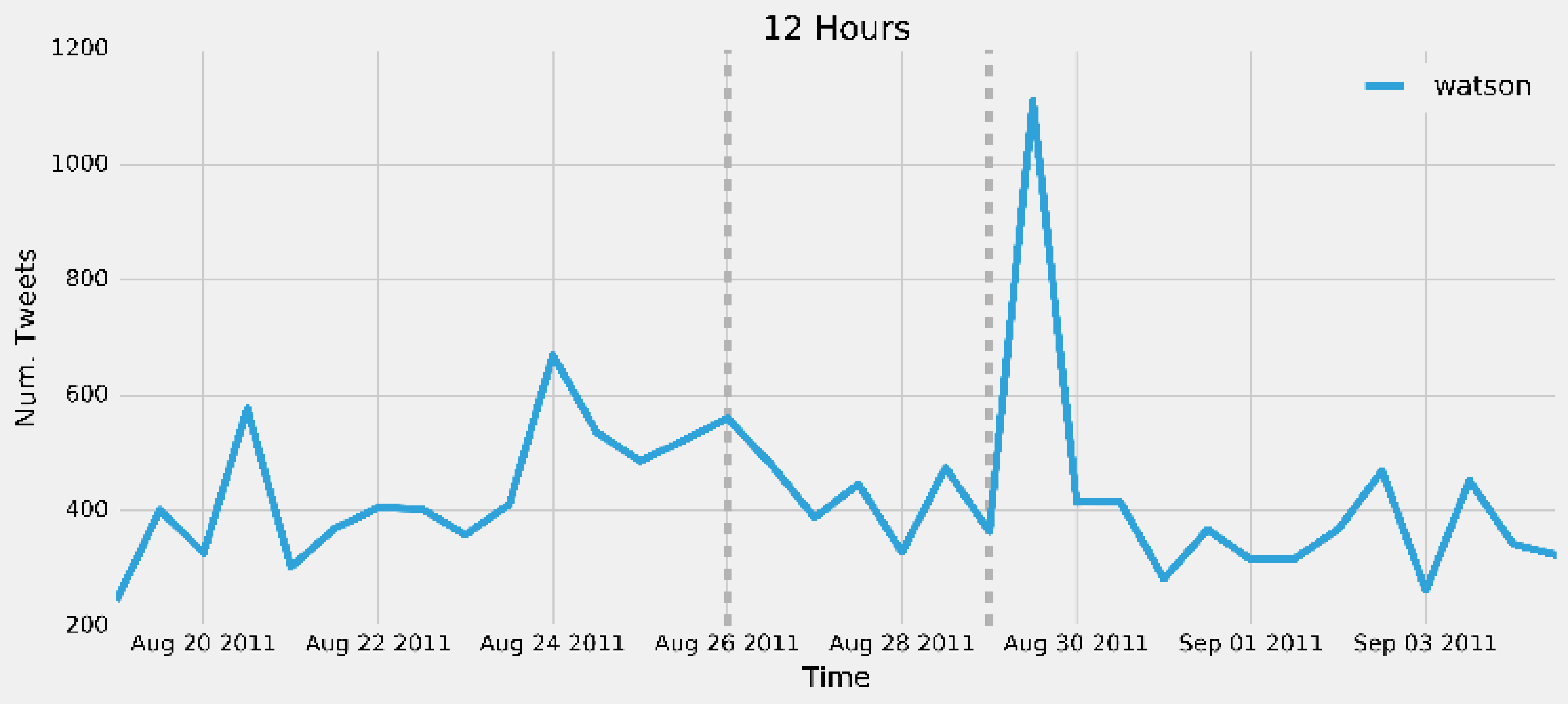
12 Hours
watson
Num. Tweets
Time
1200
1000
800
600
400
200
Aug 20 2011
Aug 22 2011
Aug 24 2011
Aug 26 2011
Aug 28 2011
Aug 30 2011
Sep 01 2011
Sep 03 2011

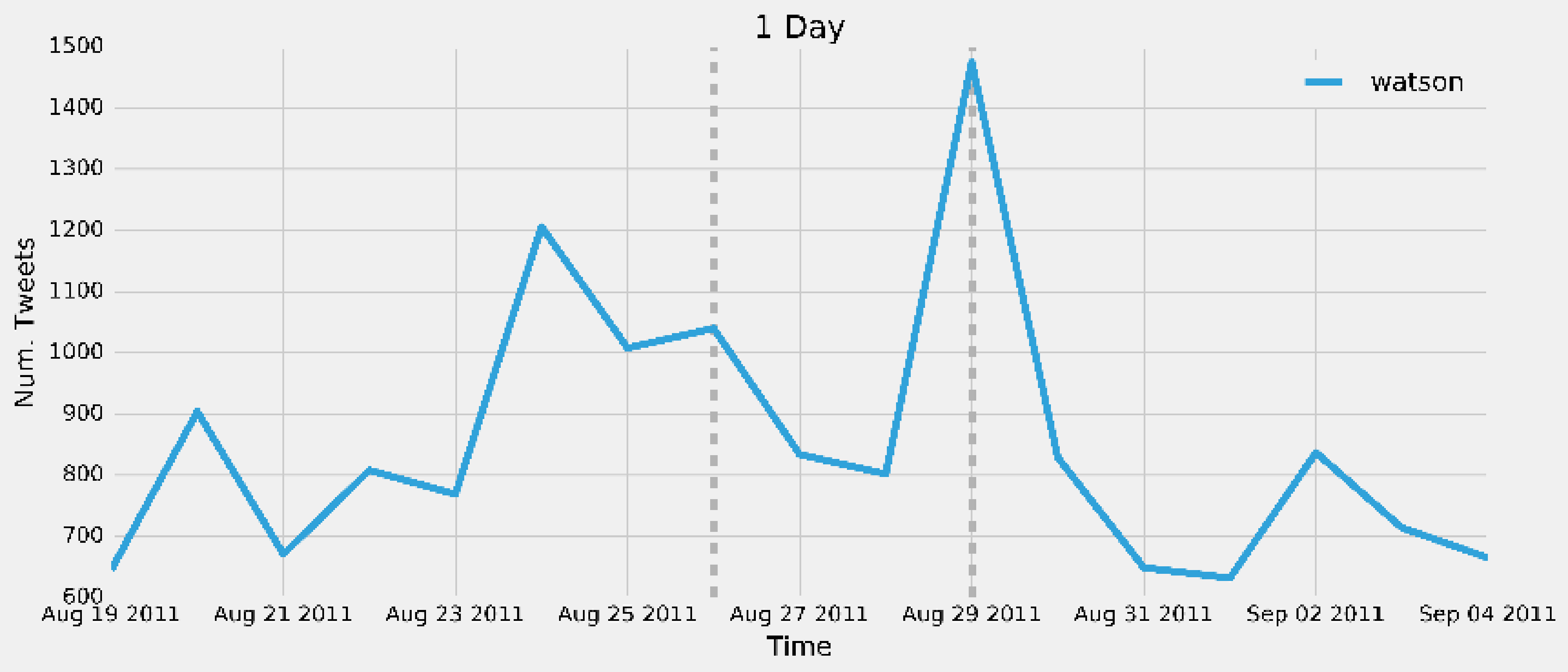

1 Day
watson
1500
1400
1300
1200
1100
1000
900
800
700
600
Num. Tweets
Aug 19 2011
Aug 21 2011
Aug 23 2011
Aug 25 2011
Aug 27 2011
Aug 29 2011
Aug 31 2011
Sep 02 2011
Sep 04 2011
Time

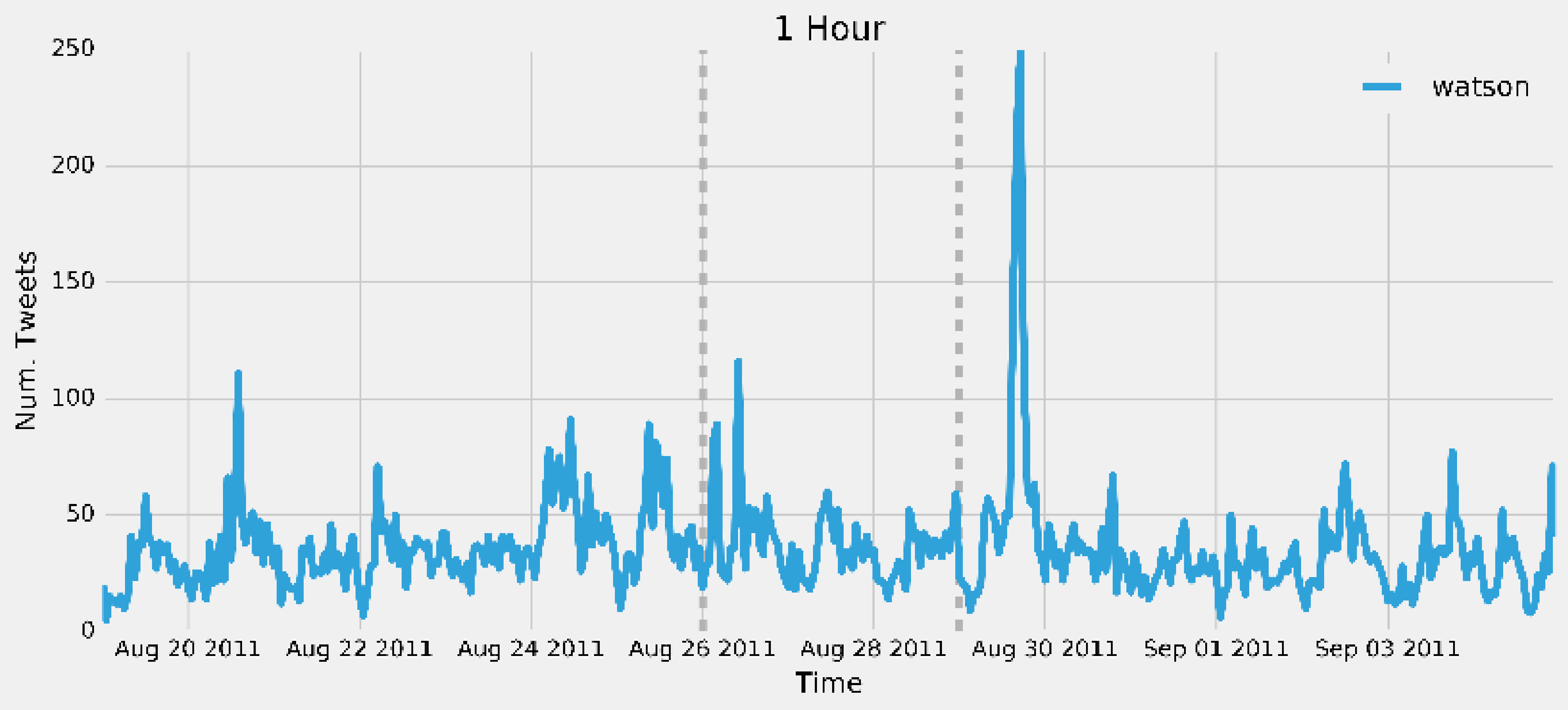

1 Hour
watson
Num. Tweets
0
50
100
150
200
250
Aug 20 2011
Aug 22 2011
Aug 24 2011
Aug 26 2011
Aug 28 2011
Aug 30 2011
Sep 01 2011
Sep 03 2011
Time

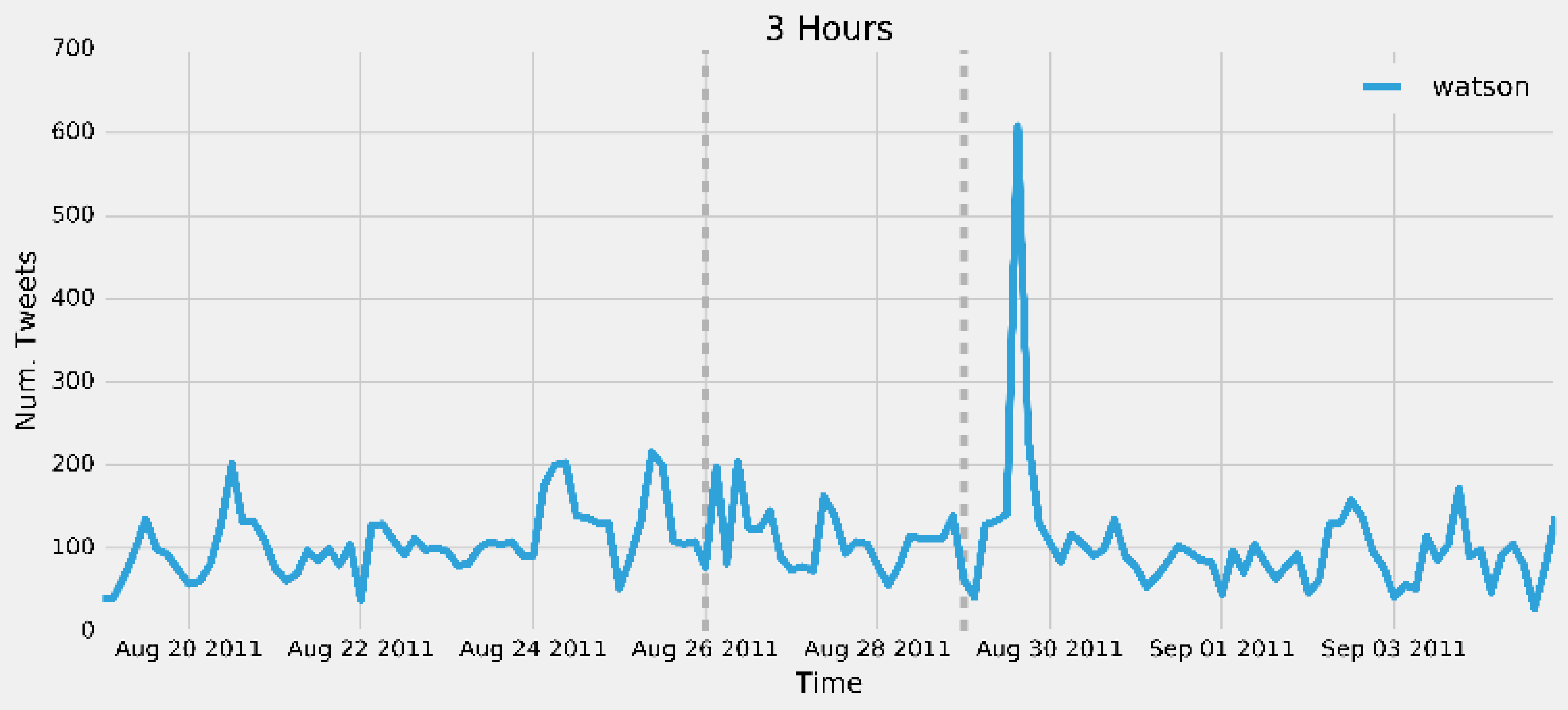
3 Hours
watson
Num. Tweets
0
100
200
300
400
500
600
700
Aug 20 2011
Aug 22 2011
Aug 24 2011
Aug 26 2011
Aug 28 2011
Aug 30 2011
Sep 01 2011
Sep 03 2011
Time

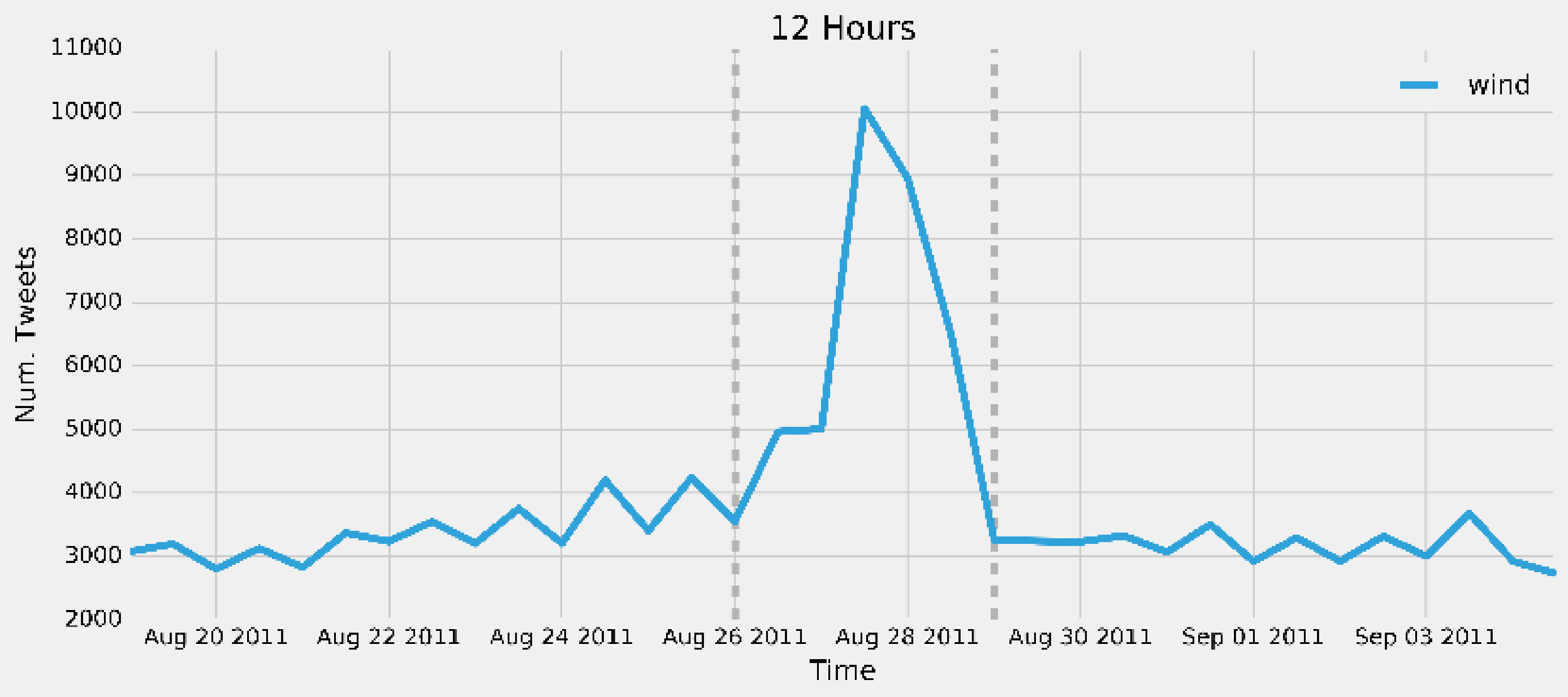
12 Hours
wind
Num. Tweets
Time
11000
10000
9000
8000
7000
6000
5000
4000
3000
2000
Aug 20 2011
Aug 22 2011
Aug 24 2011
Aug 26 2011
Aug 28 2011
Aug 30 2011
Sep 01 2011
Sep 03 2011

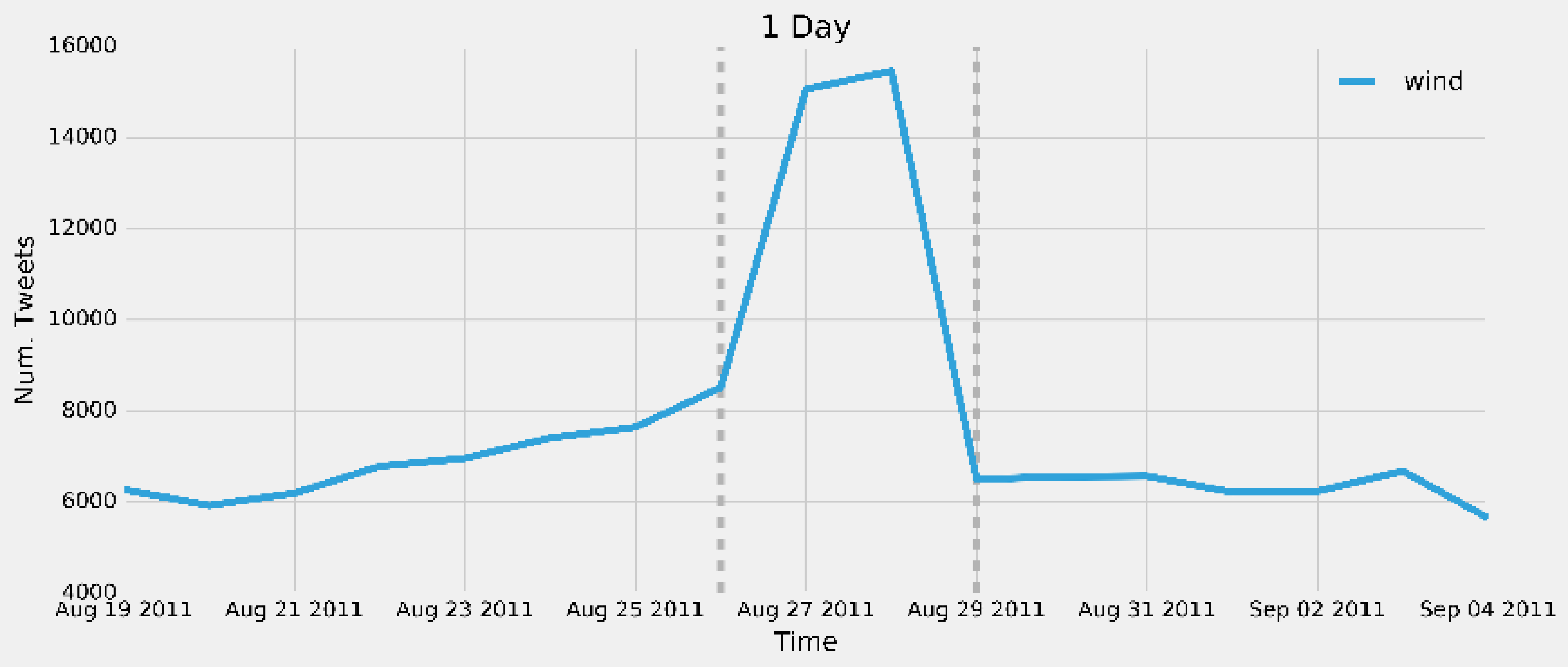
1 Day
wind
Num. Tweets
16000
14000
12000
10000
8000
6000
4000
Aug 19 2011
Aug 21 2011
Aug 23 2011
Aug 25 2011
Aug 27 2011
Aug 29 2011
Aug 31 2011
Sep 02 2011
Sep 04 2011
Time

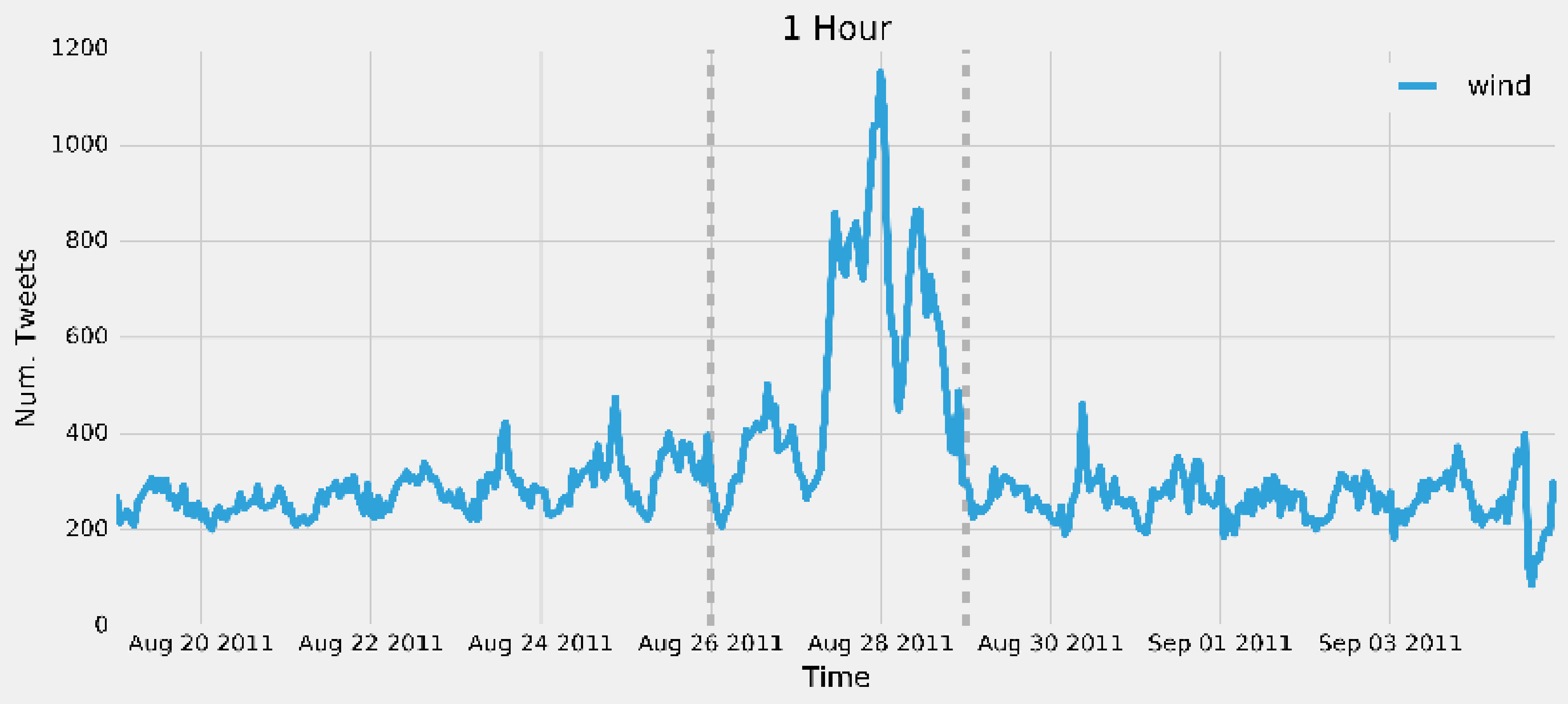

1 Hour
wind
Num. Tweets
1200
1000
800
600
400
200
0
Aug 20 2011
Aug 22 2011
Aug 24 2011
Aug 26 2011
Aug 28 2011
Aug 30 2011
Sep 01 2011
Sep 03 2011
Time

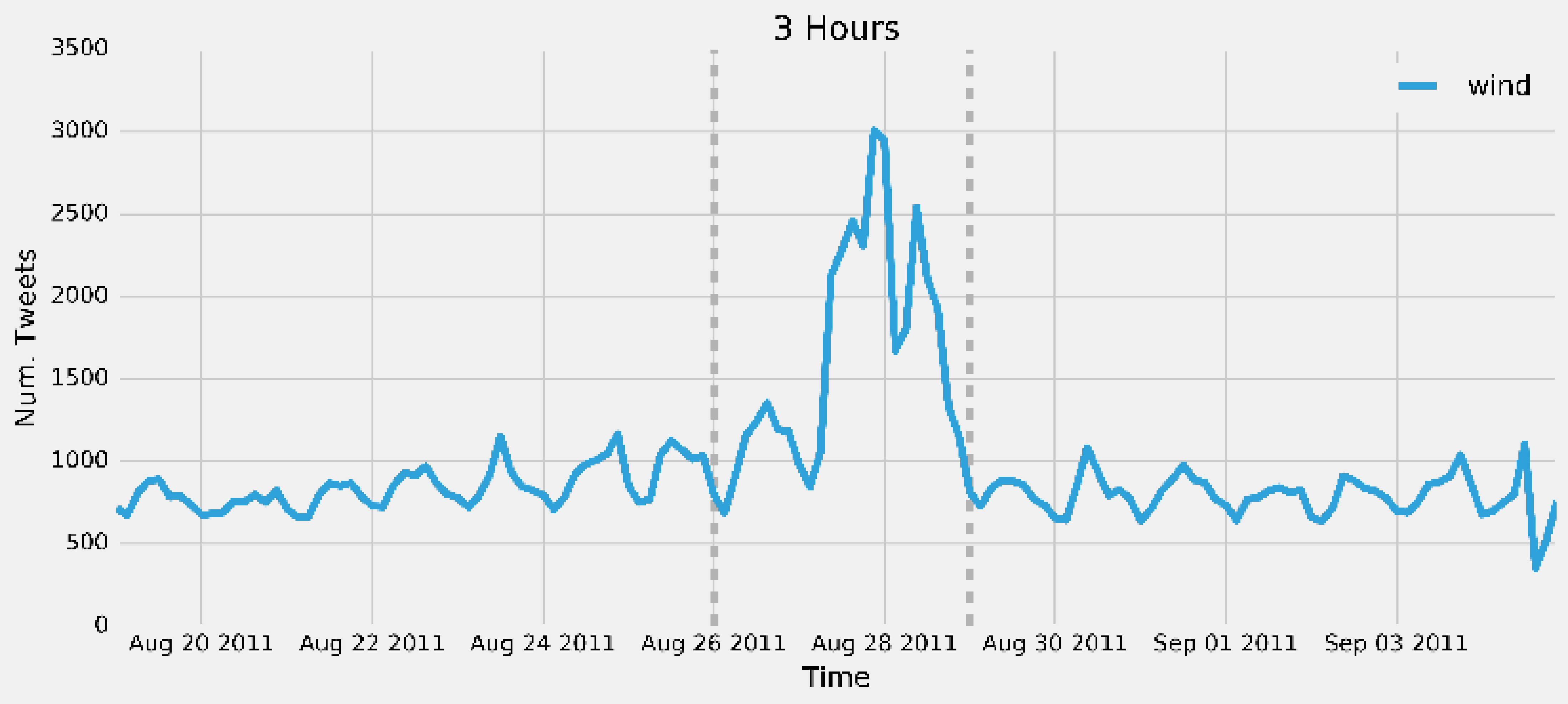
3 Hours
wind
Num. Tweets
Time
0
500
1000
1500
2000
2500
3000
3500
Aug 20 2011
Aug 22 2011
Aug 24 2011
Aug 26 2011
Aug 28 2011
Aug 30 2011
Sep 01 2011
Sep 03 2011
