# Supplementary material for: Social media usage patterns during natural hazards
Source: PLoS One. 2019 Feb 13;14(2):e0210484. doi: 10.1371/journal.pone.0210484 (PMC6374021; doi:10.1371/journal.pone.0210484)
Supplement: S3 Fig — (PDF) [file pone.0210484.s003.pdf]

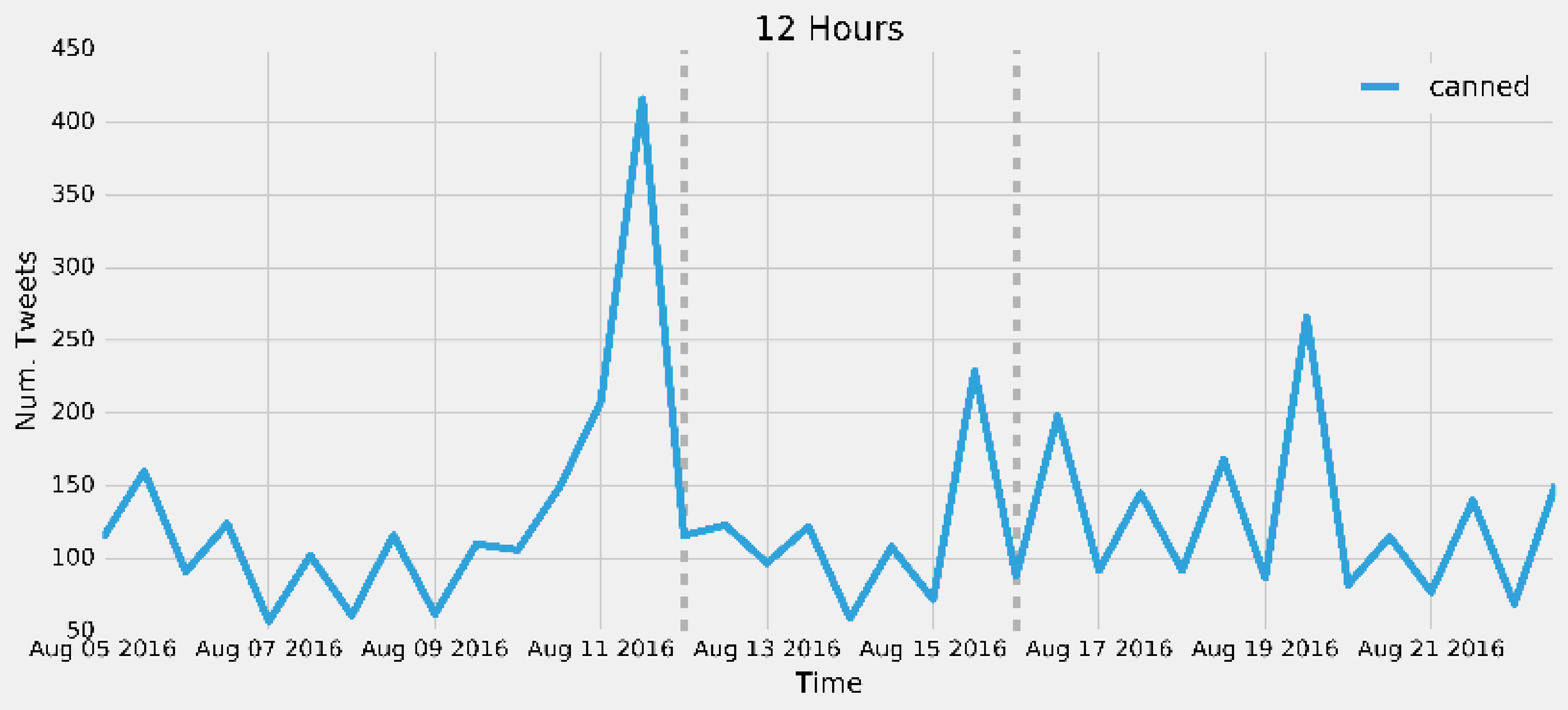
12 Hours
canned
Num. Tweets
450
400
350
300
250
200
150
100
50
Aug 05 2016
Aug 07 2016
Aug 09 2016
Aug 11 2016
Aug 13 2016
Aug 15 2016
Aug 17 2016
Aug 19 2016
Aug 21 2016
Time

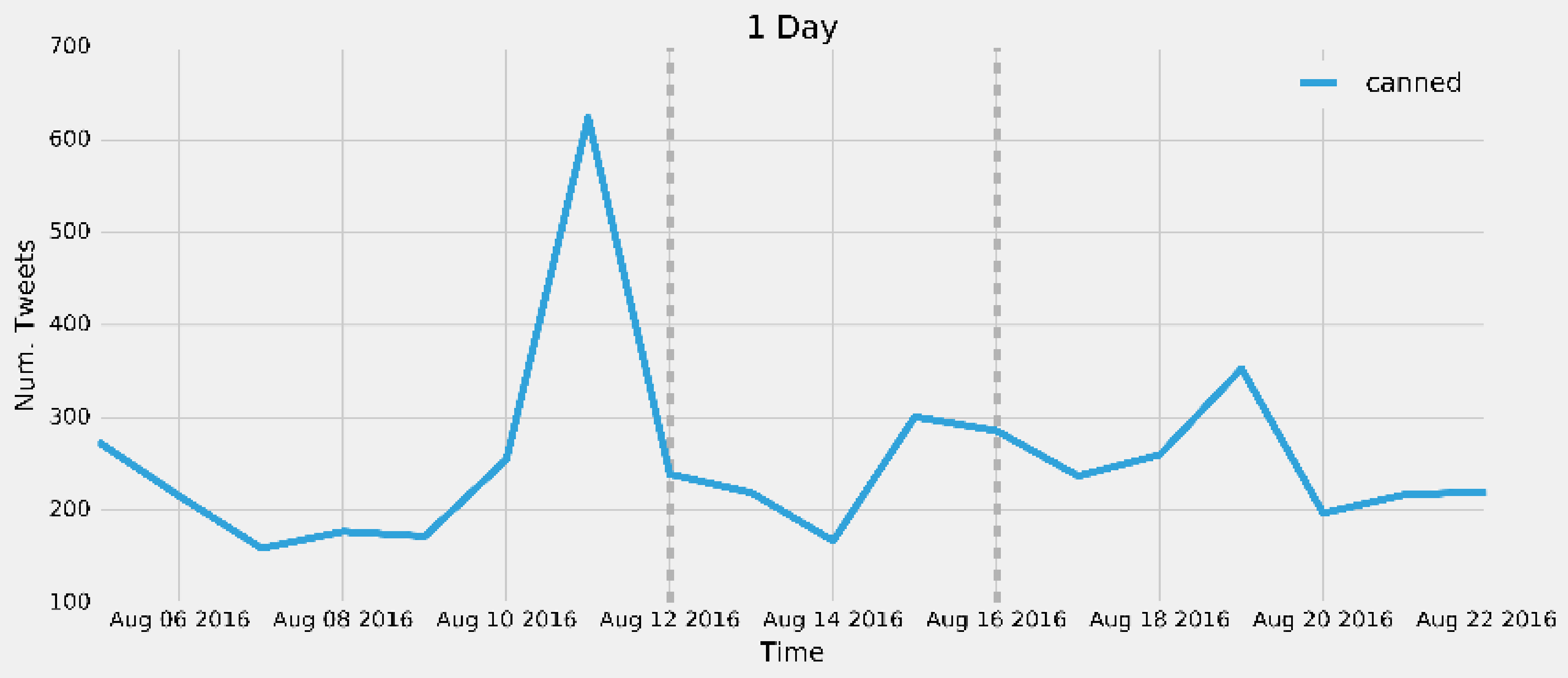
1 Day
canned
Num. Tweets
700
600
500
400
300
200
100
Aug 06 2016
Aug 08 2016
Aug 10 2016
Aug 12 2016
Aug 14 2016
Aug 16 2016
Aug 18 2016
Aug 20 2016
Aug 22 2016
Time

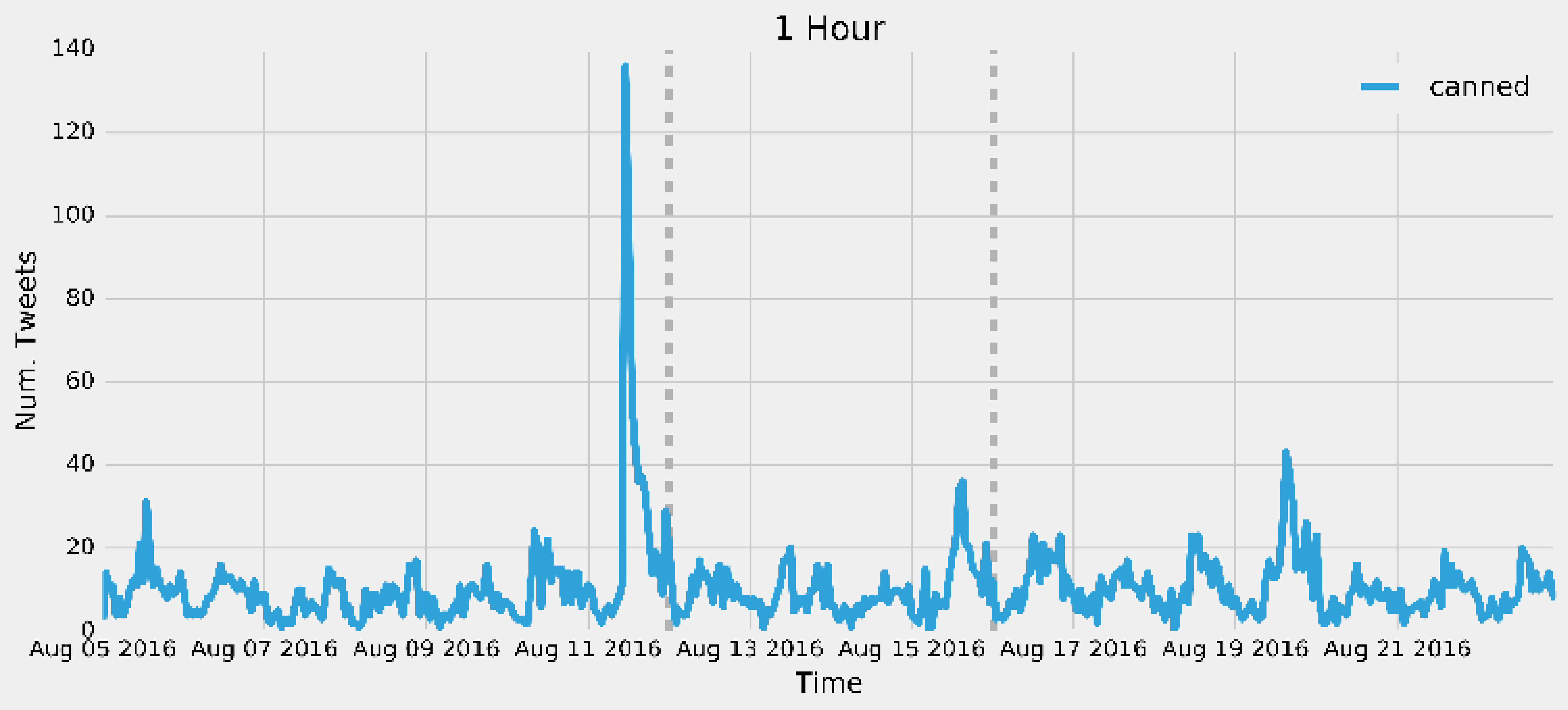
1 Hour
canned
Num. Tweets
0
20
40
60
80
100
120
140
Aug 05 2016
Aug 07 2016
Aug 09 2016
Aug 11 2016
Aug 13 2016
Aug 15 2016
Aug 17 2016
Aug 19 2016
Aug 21 2016
Time

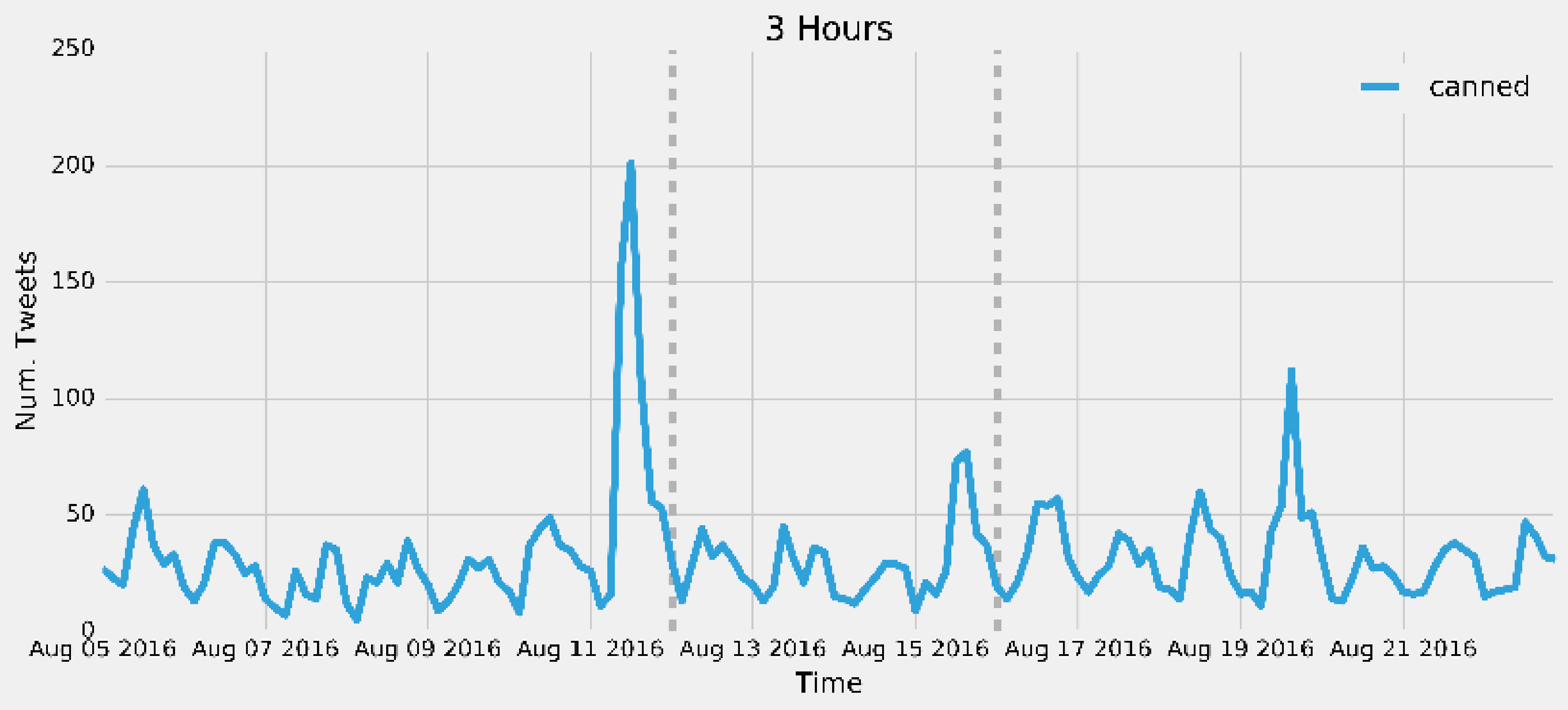

3 Hours
canned
Num. Tweets
0
50
100
150
200
250
Aug 05 2016
Aug 07 2016
Aug 09 2016
Aug 11 2016
Aug 13 2016
Aug 15 2016
Aug 17 2016
Aug 19 2016
Aug 21 2016
Time

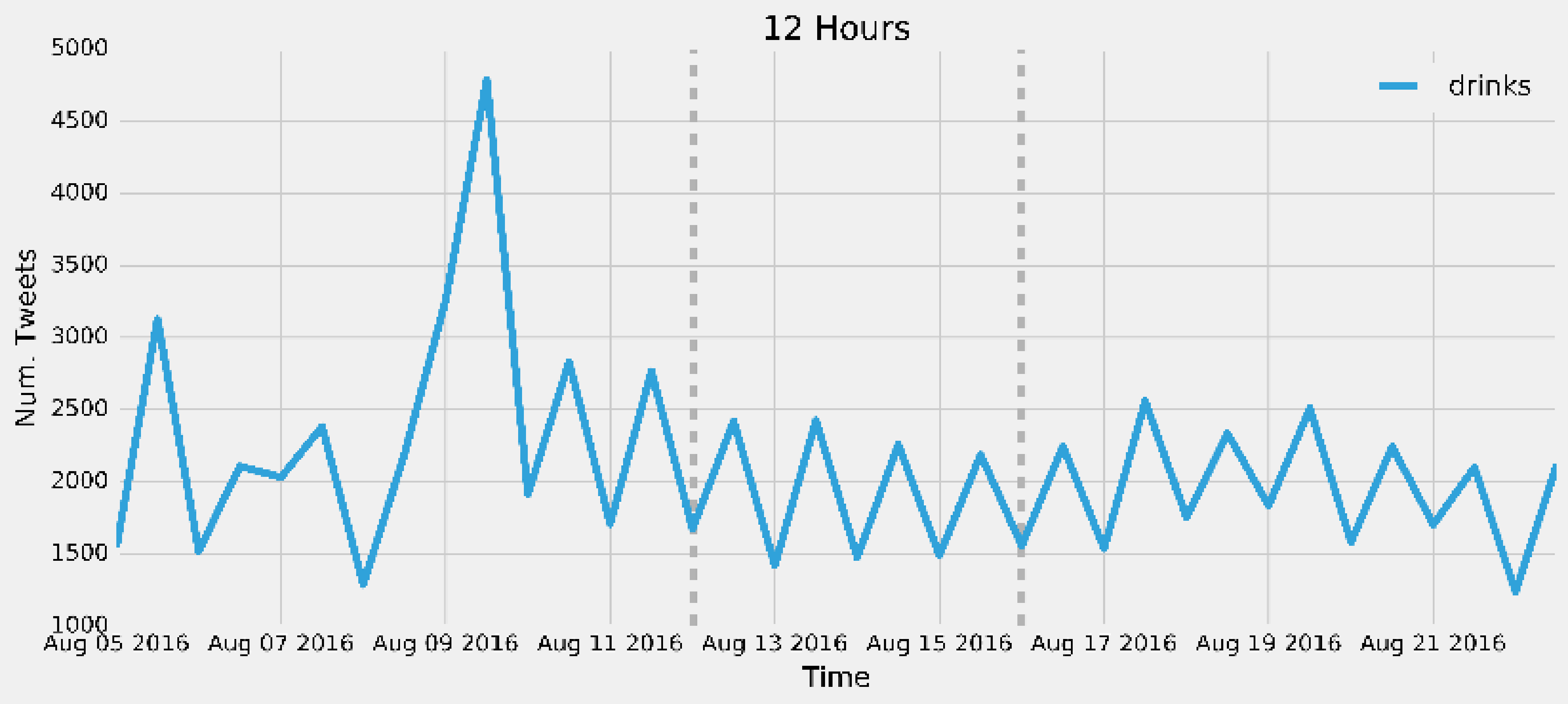

12 Hours
drinks
5000
4500
4000
3500
3000
2500
2000
1500
1000
Num. Tweets
Aug 05 2016
Aug 07 2016
Aug 09 2016
Aug 11 2016
Aug 13 2016
Aug 15 2016
Aug 17 2016
Aug 19 2016
Aug 21 2016
Time

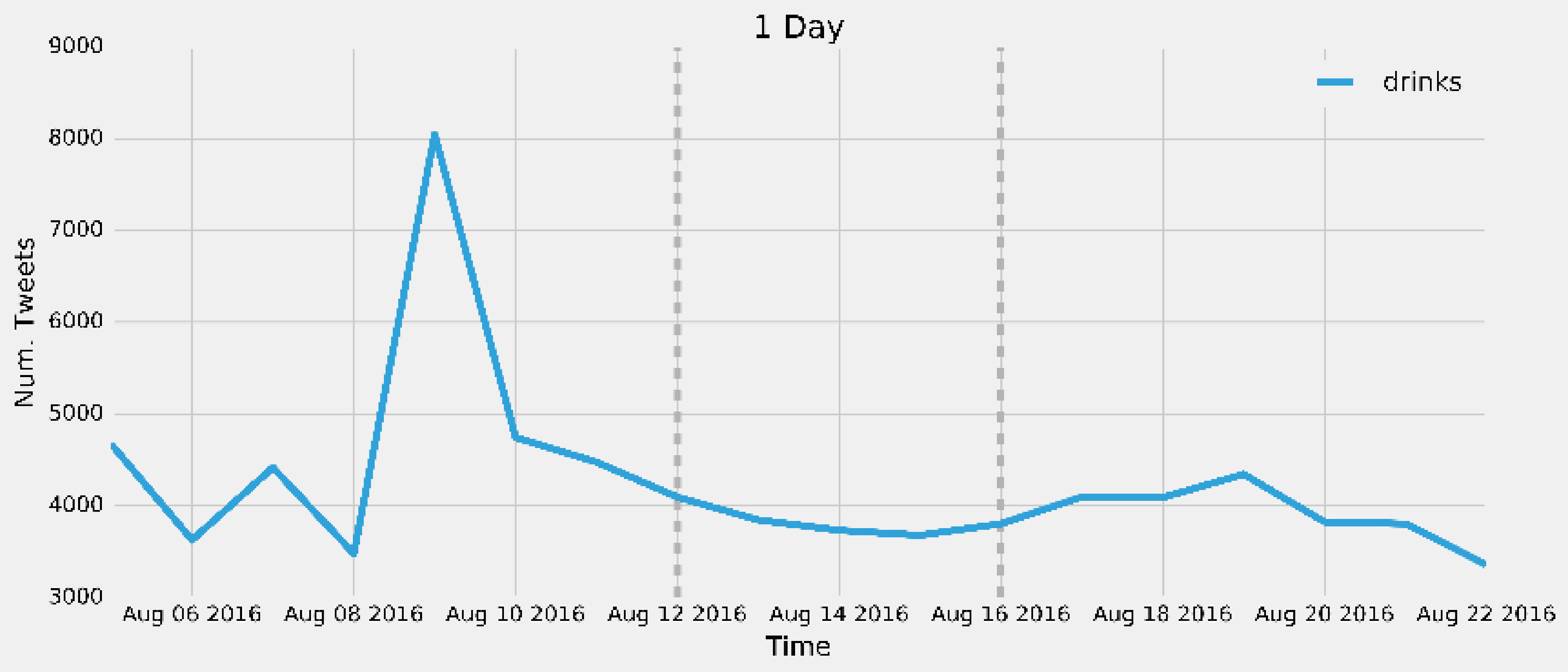
1 Day
9000
8000
7000
6000
5000
4000
3000
Num. Tweets
drinks
Aug 06 2016
Aug 08 2016
Aug 10 2016
Aug 12 2016
Aug 14 2016
Aug 16 2016
Aug 18 2016
Aug 20 2016
Aug 22 2016
Time

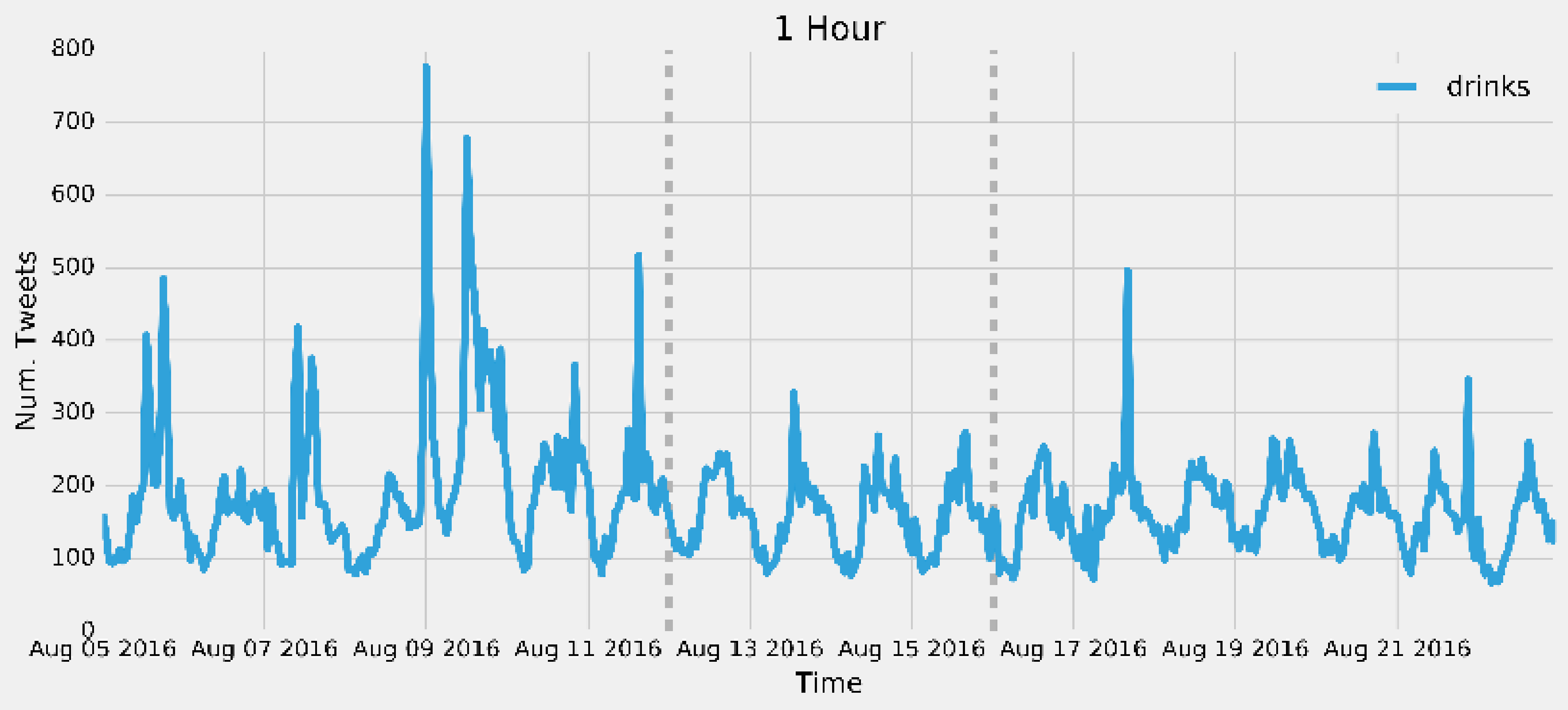

1 Hour
drinks
Num. Tweets
0
100
200
300
400
500
600
700
800
Aug 05 2016
Aug 07 2016
Aug 09 2016
Aug 11 2016
Aug 13 2016
Aug 15 2016
Aug 17 2016
Aug 19 2016
Aug 21 2016
Time

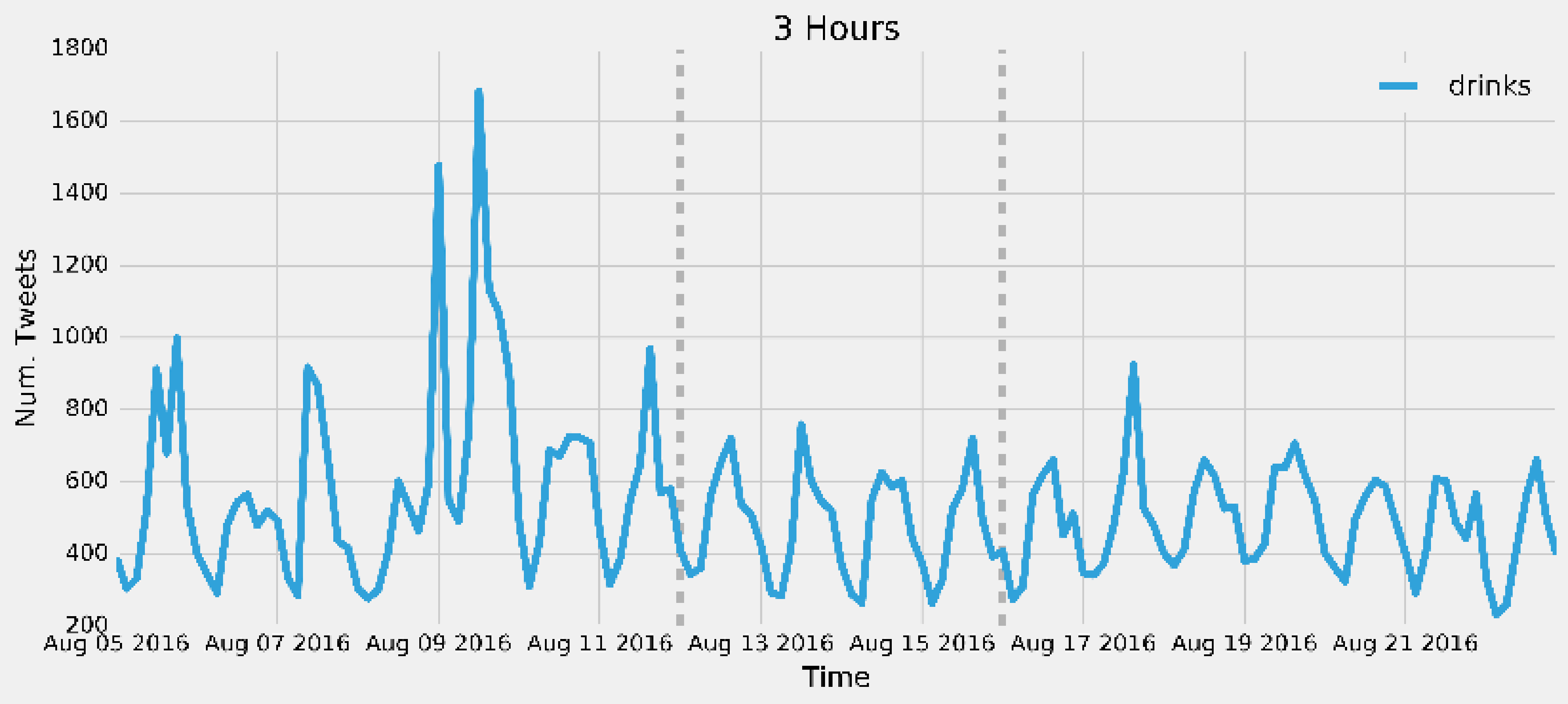

3 Hours
drinks
Num. Tweets
1800
1600
1400
1200
1000
800
600
400
200
Aug 05 2016
Aug 07 2016
Aug 09 2016
Aug 11 2016
Aug 13 2016
Aug 15 2016
Aug 17 2016
Aug 19 2016
Aug 21 2016
Time

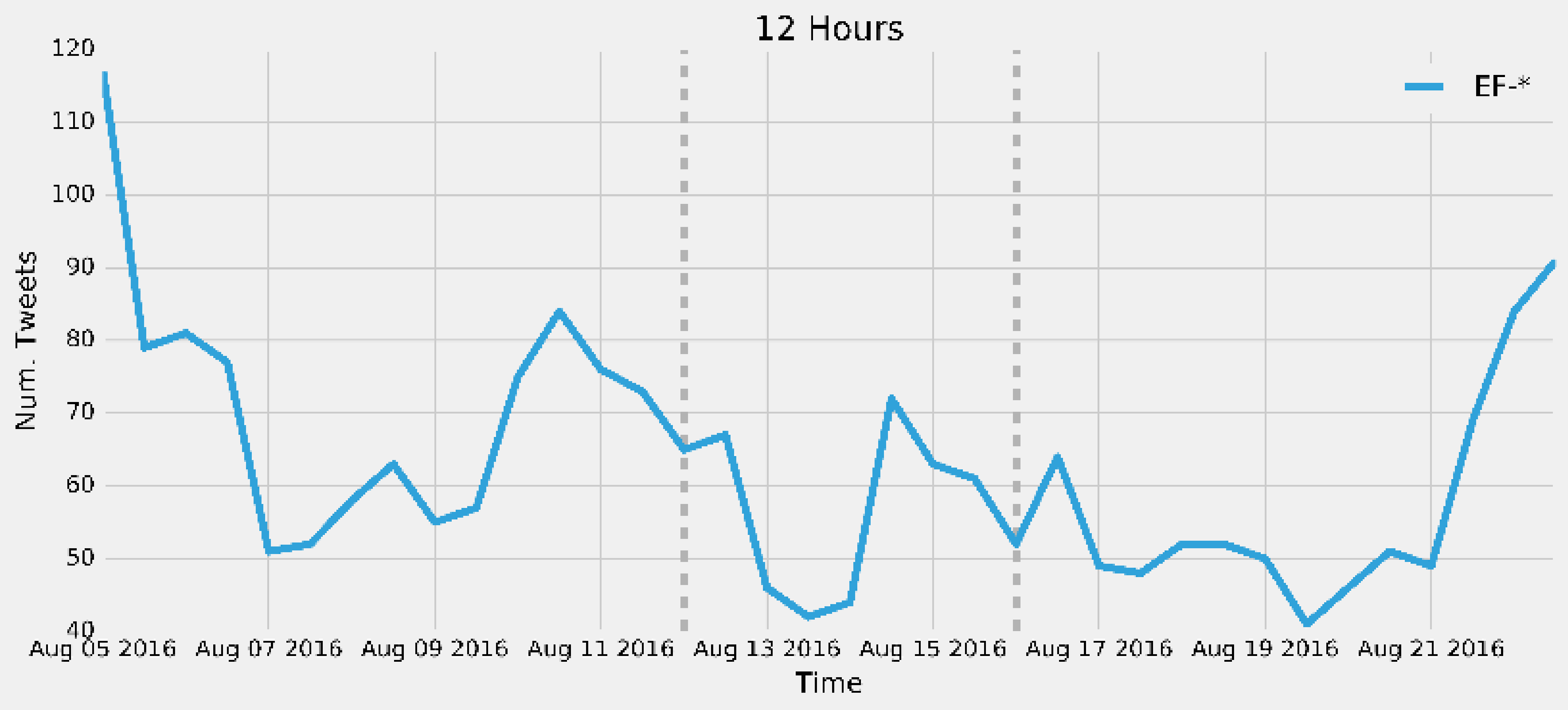

12 Hours
EF-*
Num. Tweets
Time
120
110
100
90
80
70
60
50
40
Aug 05 2016
Aug 07 2016
Aug 09 2016
Aug 11 2016
Aug 13 2016
Aug 15 2016
Aug 17 2016
Aug 19 2016
Aug 21 2016

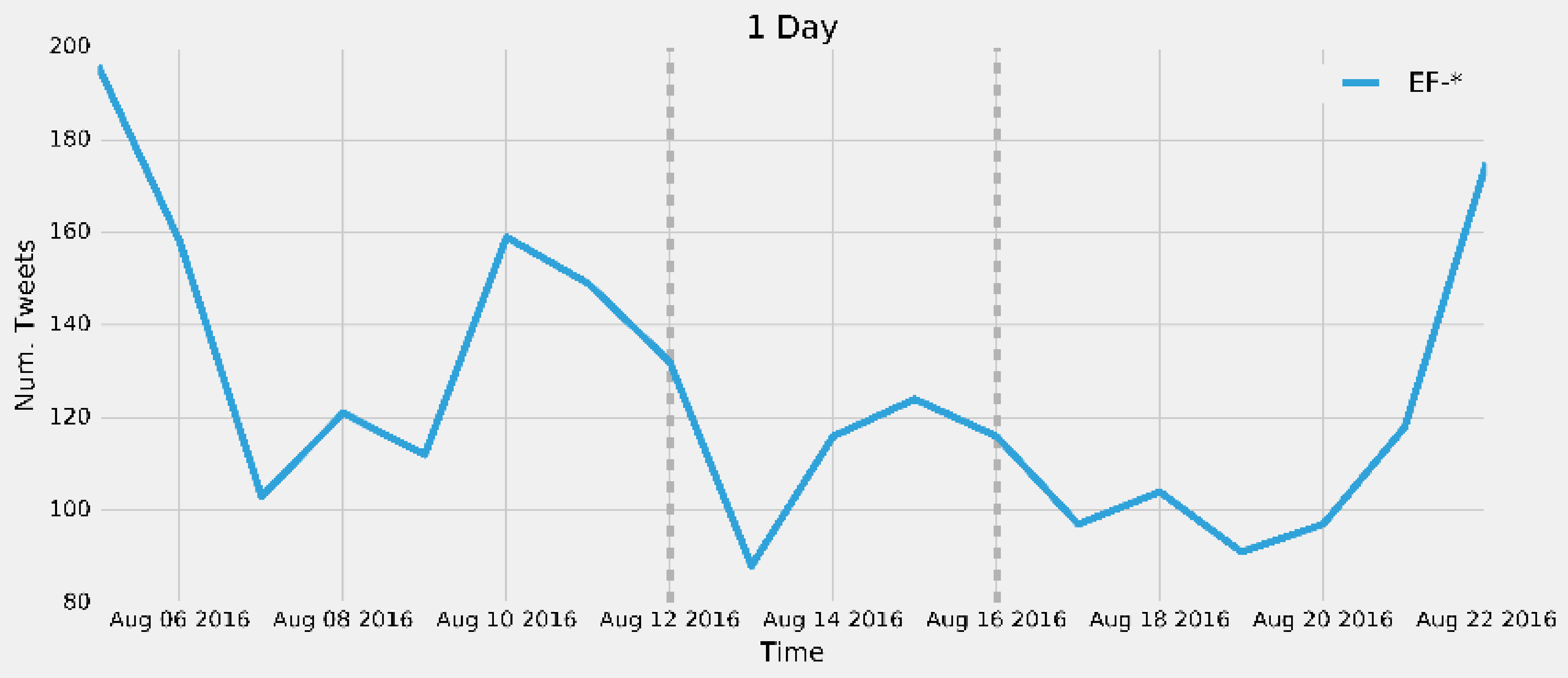

1 Day
200
180
160
140
120
100
80
Num. Tweets
EF-*
Aug 06 2016
Aug 08 2016
Aug 10 2016
Aug 12 2016
Aug 14 2016
Aug 16 2016
Aug 18 2016
Aug 20 2016
Aug 22 2016
Time

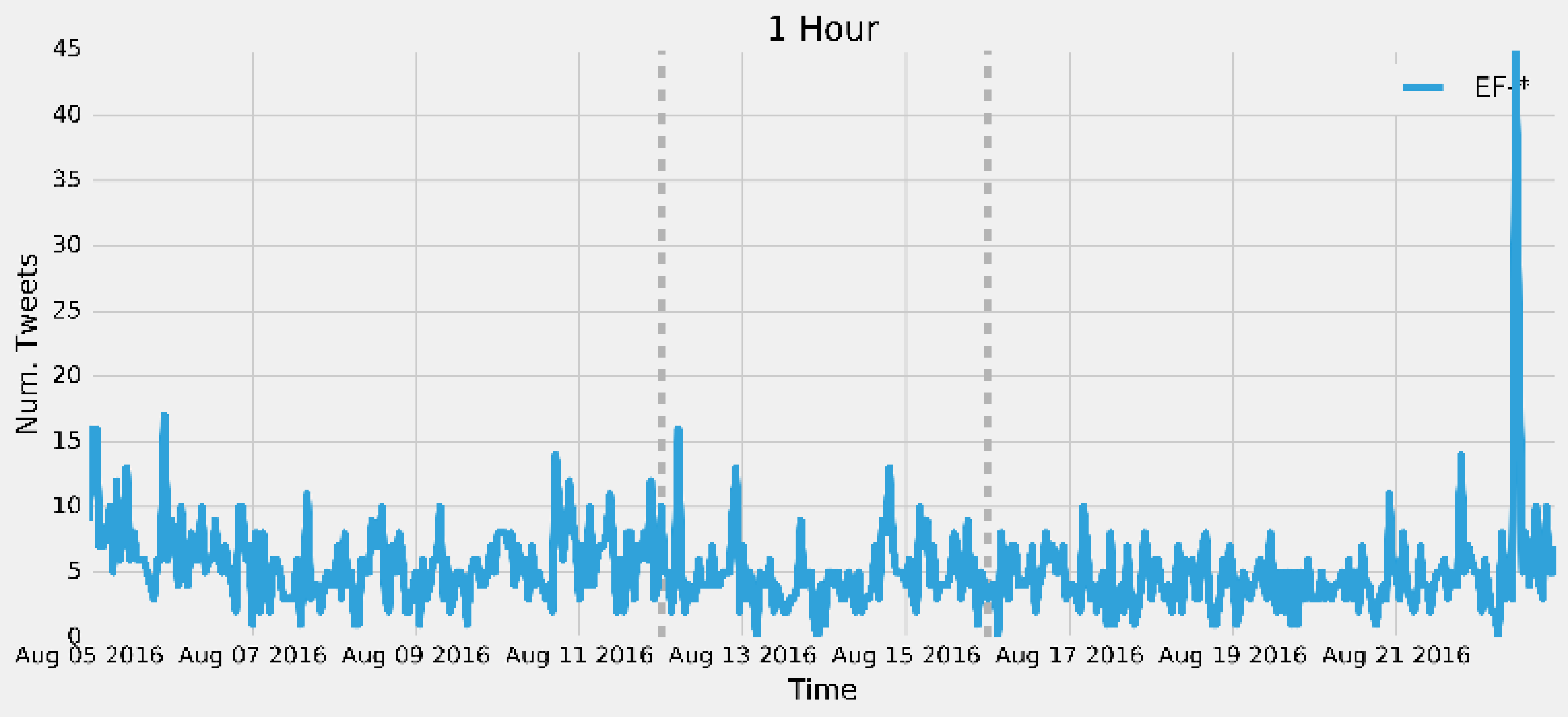
1 Hour
EF-*
Num. Tweets
Time
0
5
10
15
20
25
30
35
40
45
Aug 05 2016
Aug 07 2016
Aug 09 2016
Aug 11 2016
Aug 13 2016
Aug 15 2016
Aug 17 2016
Aug 19 2016
Aug 21 2016

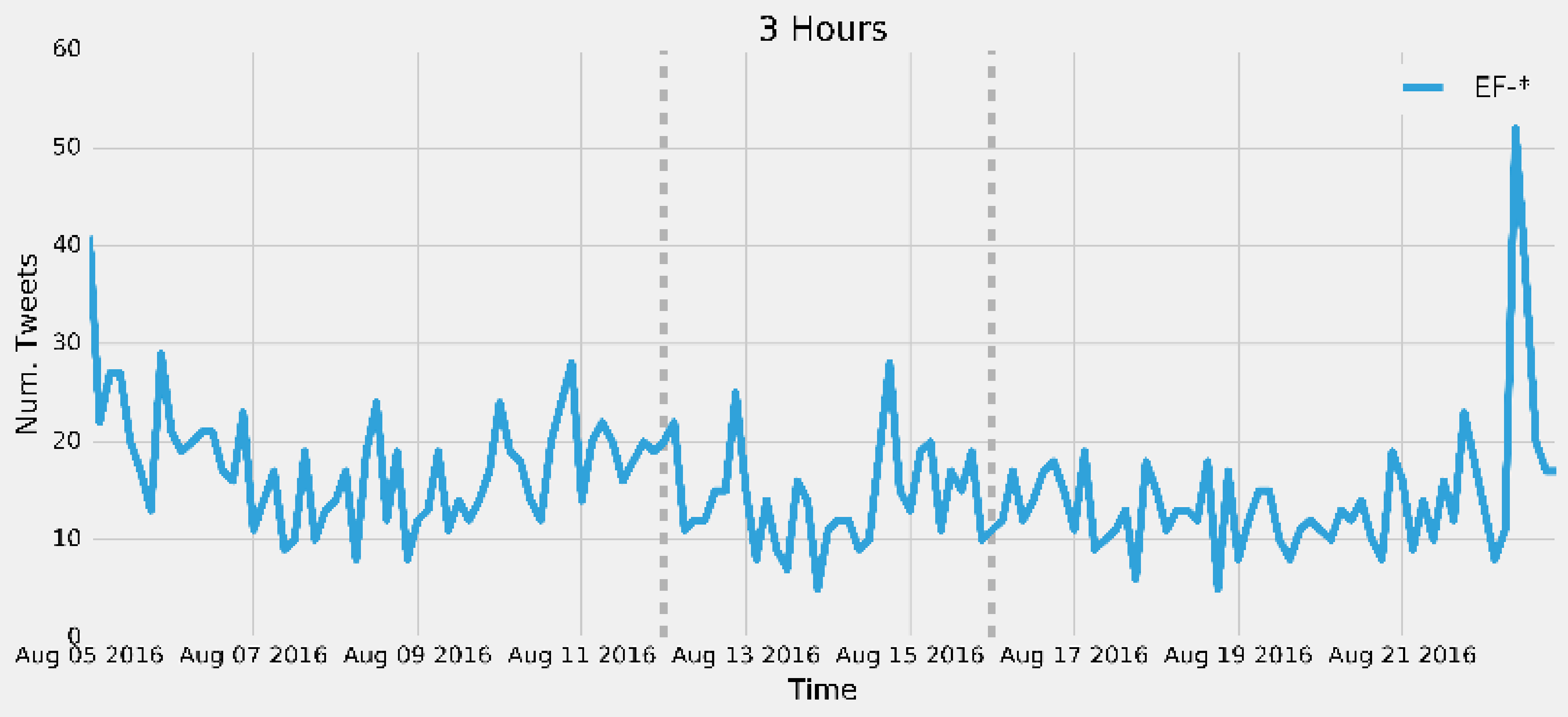
3 Hours
EF-
Num. Tweets
0
10
20
30
40
50
60
Aug 05 2016
Aug 07 2016
Aug 09 2016
Aug 11 2016
Aug 13 2016
Aug 15 2016
Aug 17 2016
Aug 19 2016
Aug 21 2016
Time

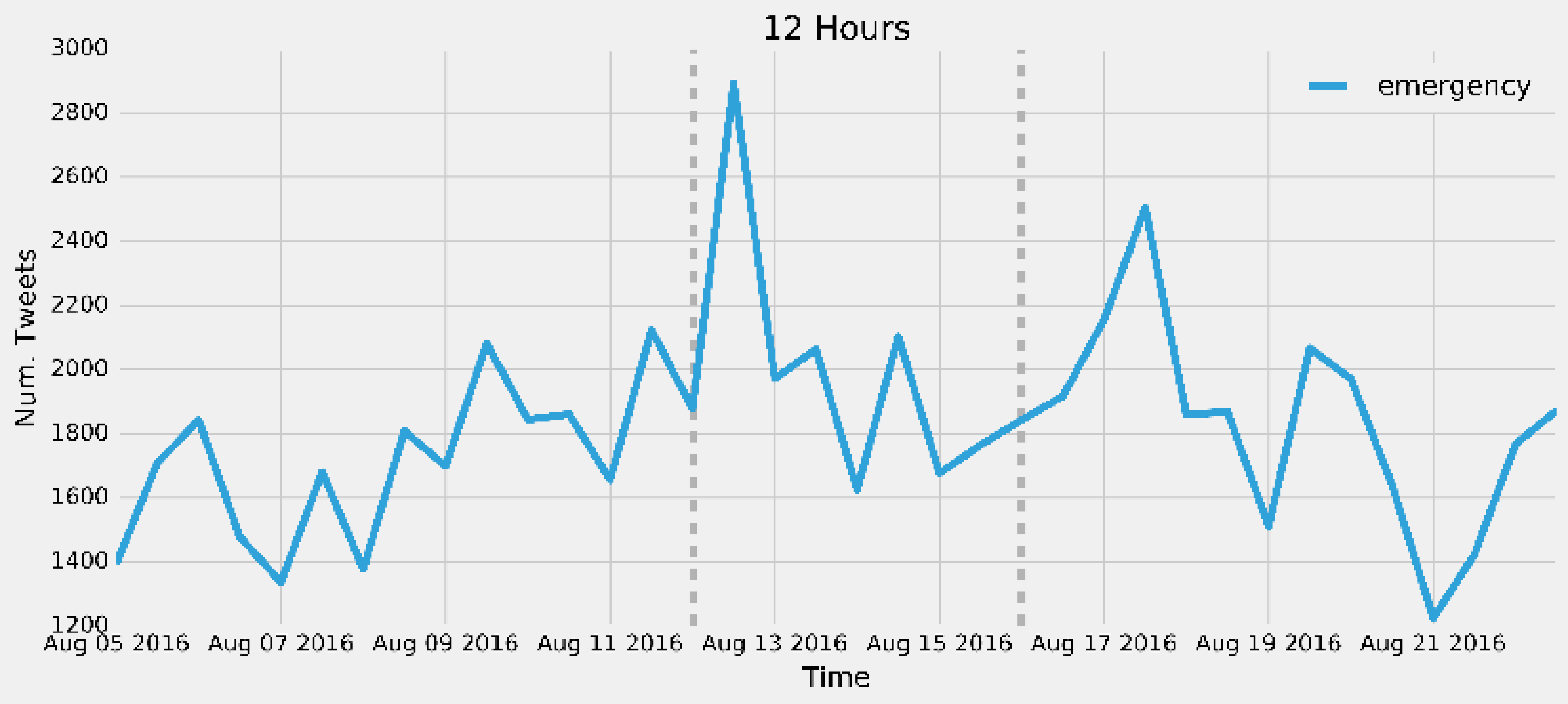

12 Hours
Num. Tweets
3000
2800
2600
2400
2200
2000
1800
1600
1400
1200
Aug 05 2016
Aug 07 2016
Aug 09 2016
Aug 11 2016
Aug 13 2016
Aug 15 2016
Aug 17 2016
Aug 19 2016
Aug 21 2016
Time
emergency

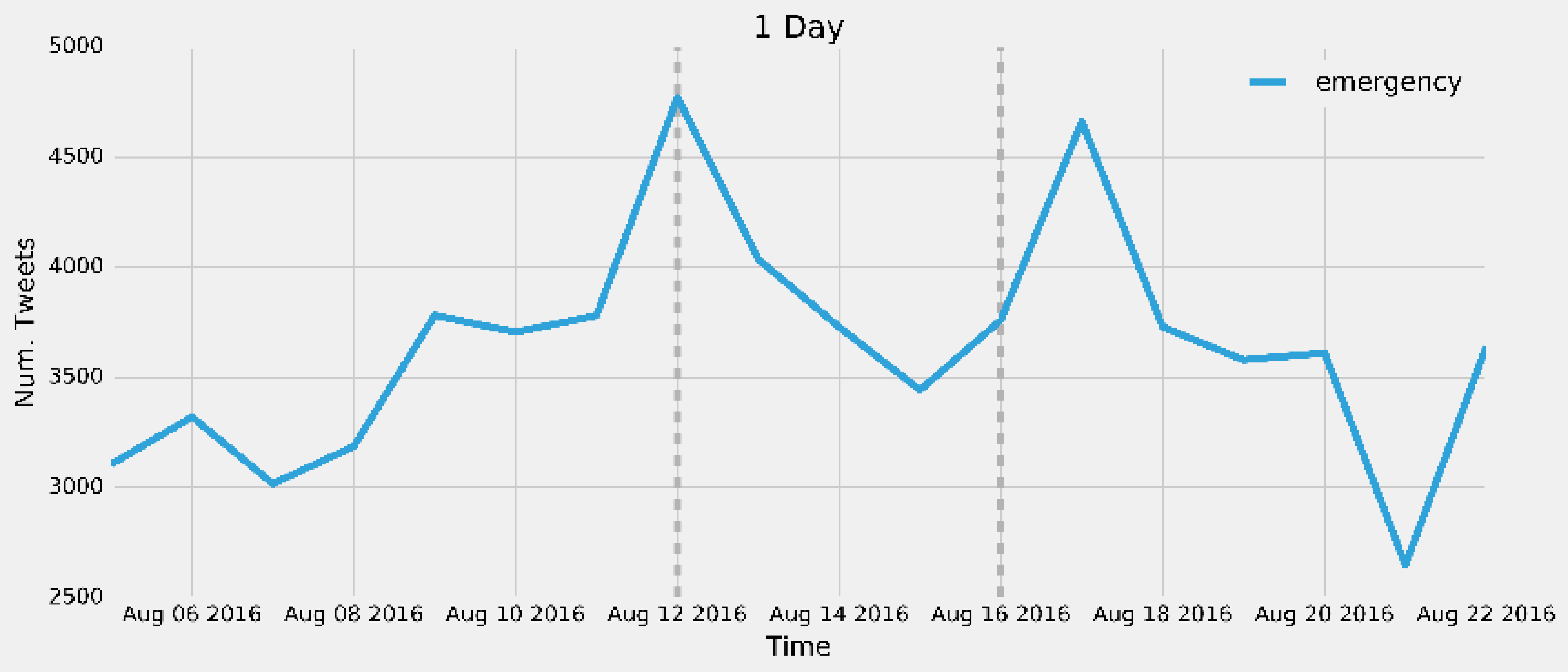

1 Day
emergency
Num. Tweets
5000
4500
4000
3500
3000
2500
Aug 06 2016
Aug 08 2016
Aug 10 2016
Aug 12 2016
Aug 14 2016
Aug 16 2016
Aug 18 2016
Aug 20 2016
Aug 22 2016
Time

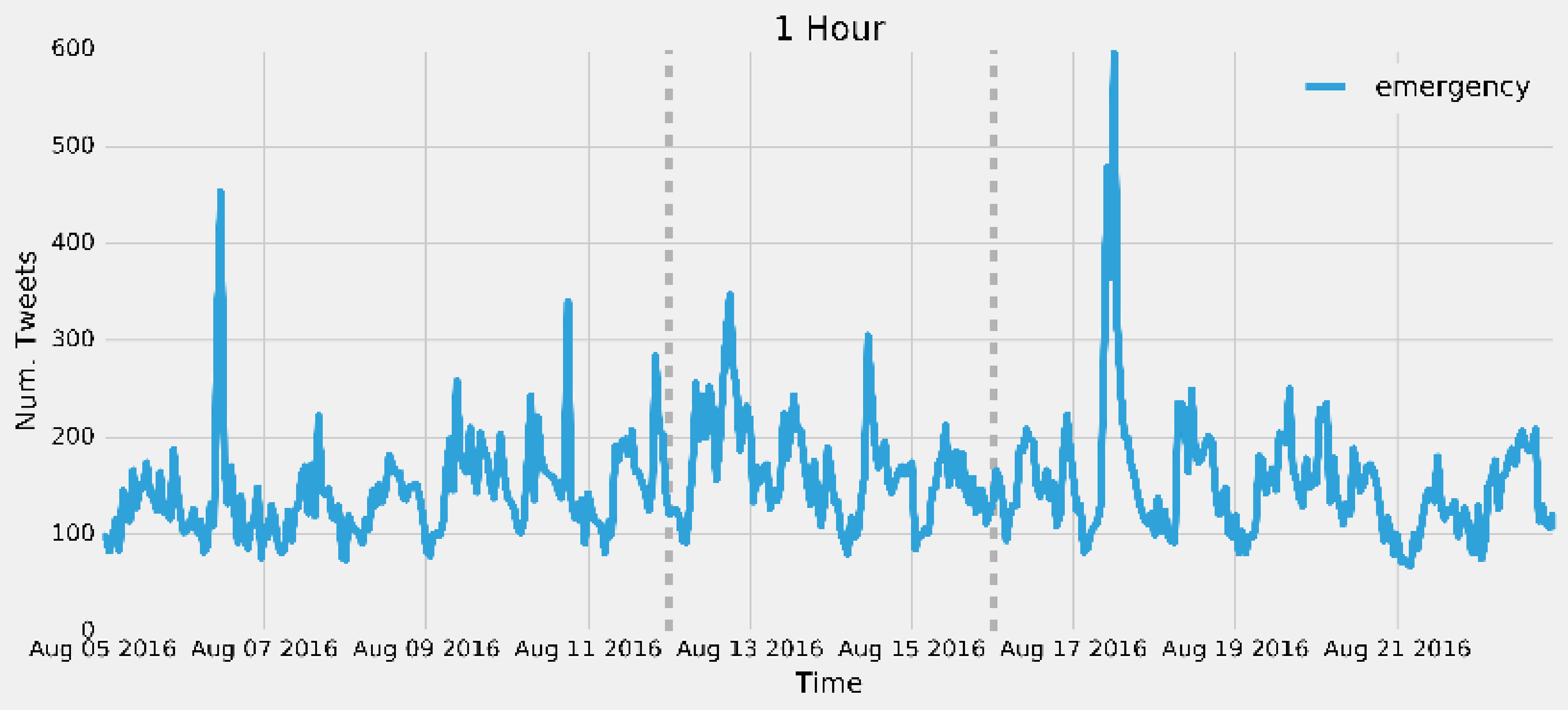

1 Hour
emergency
Num. Tweets
600
500
400
300
200
100
0
Aug 05 2016
Aug 07 2016
Aug 09 2016
Aug 11 2016
Aug 13 2016
Aug 15 2016
Aug 17 2016
Aug 19 2016
Aug 21 2016
Time

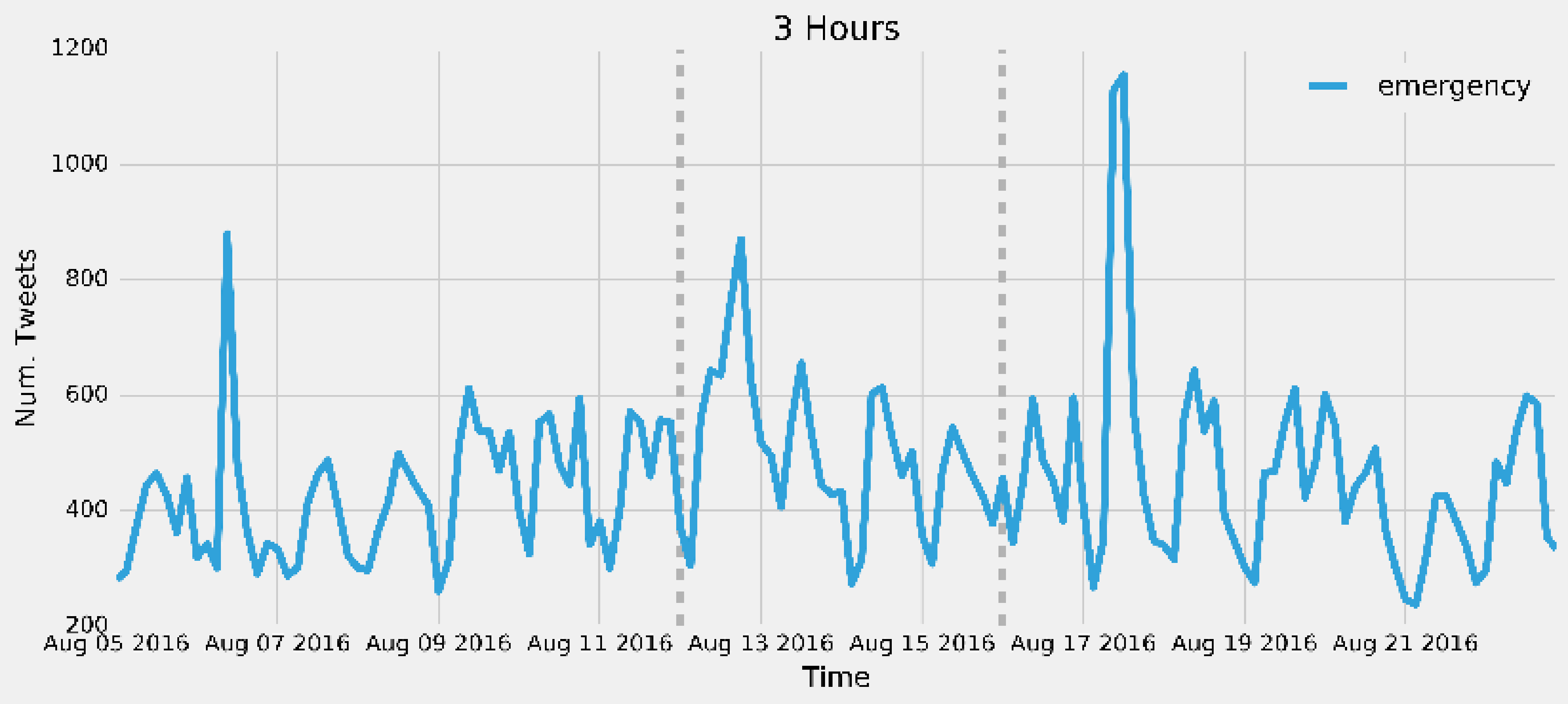

3 Hours
emergency
Num. Tweets
Time
200
400
600
800
1000
1200
Aug 05 2016
Aug 07 2016
Aug 09 2016
Aug 11 2016
Aug 13 2016
Aug 15 2016
Aug 17 2016
Aug 19 2016
Aug 21 2016

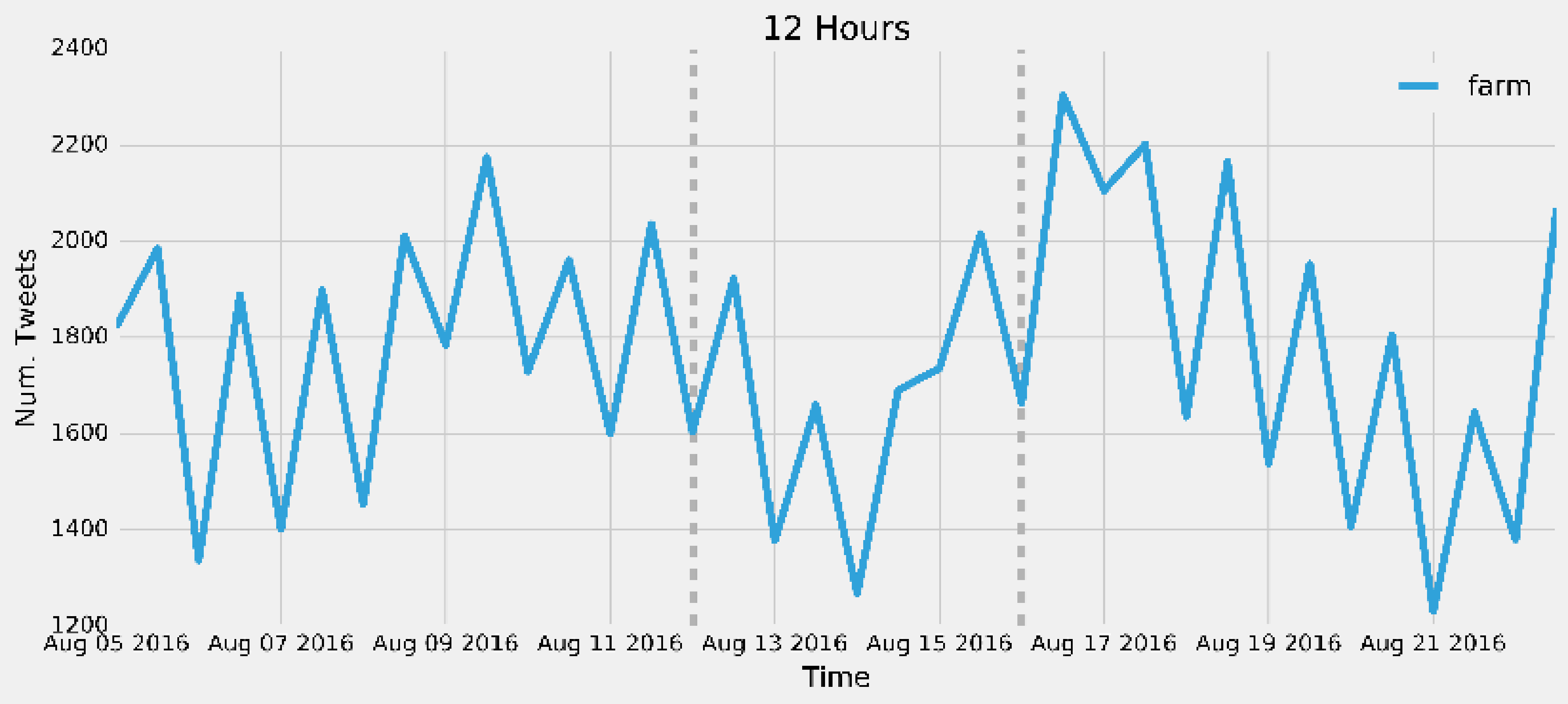
12 Hours
farm
Num. Tweets
Time
2400
2200
2000
1800
1600
1400
1200
Aug 05 2016
Aug 07 2016
Aug 09 2016
Aug 11 2016
Aug 13 2016
Aug 15 2016
Aug 17 2016
Aug 19 2016
Aug 21 2016

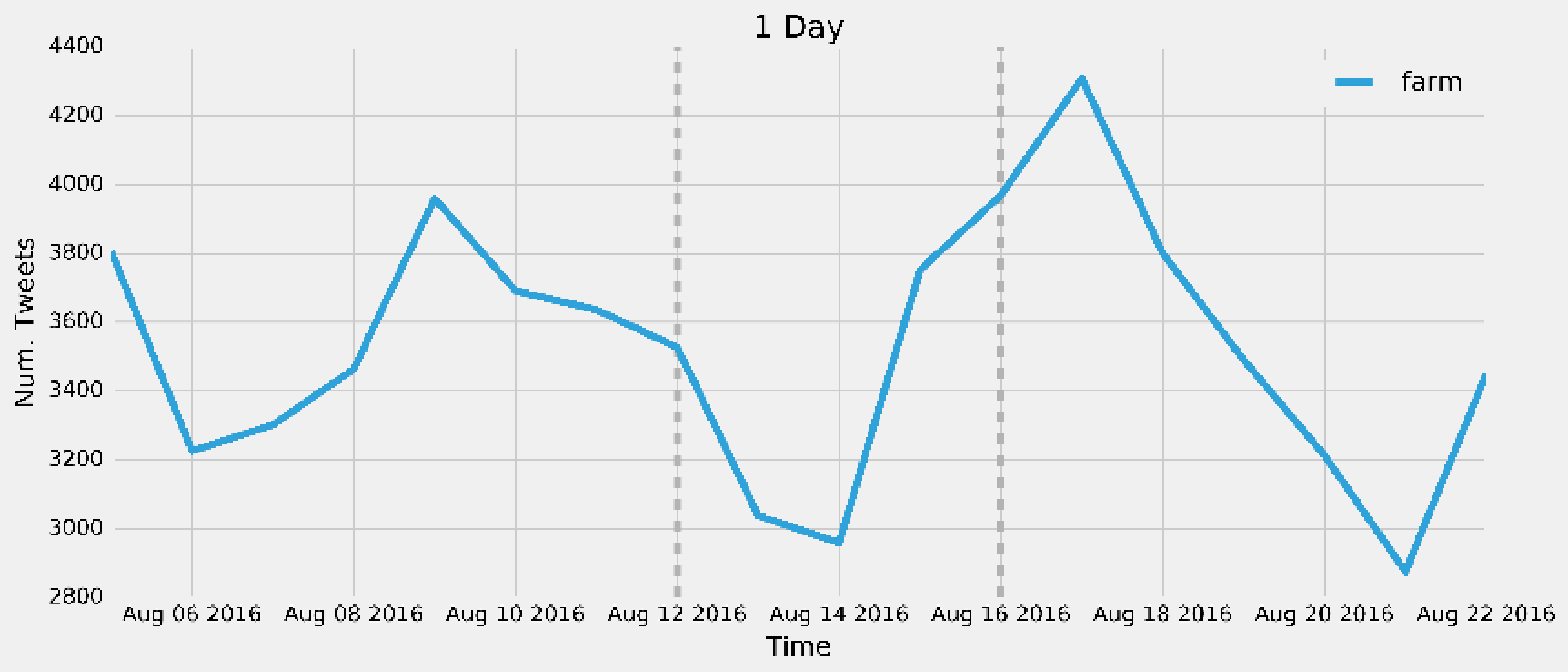
1 Day
Num. Tweets
Time
farm
4400
4200
4000
3800
3600
3400
3200
3000
2800
Aug 06 2016
Aug 08 2016
Aug 10 2016
Aug 12 2016
Aug 14 2016
Aug 16 2016
Aug 18 2016
Aug 20 2016
Aug 22 2016

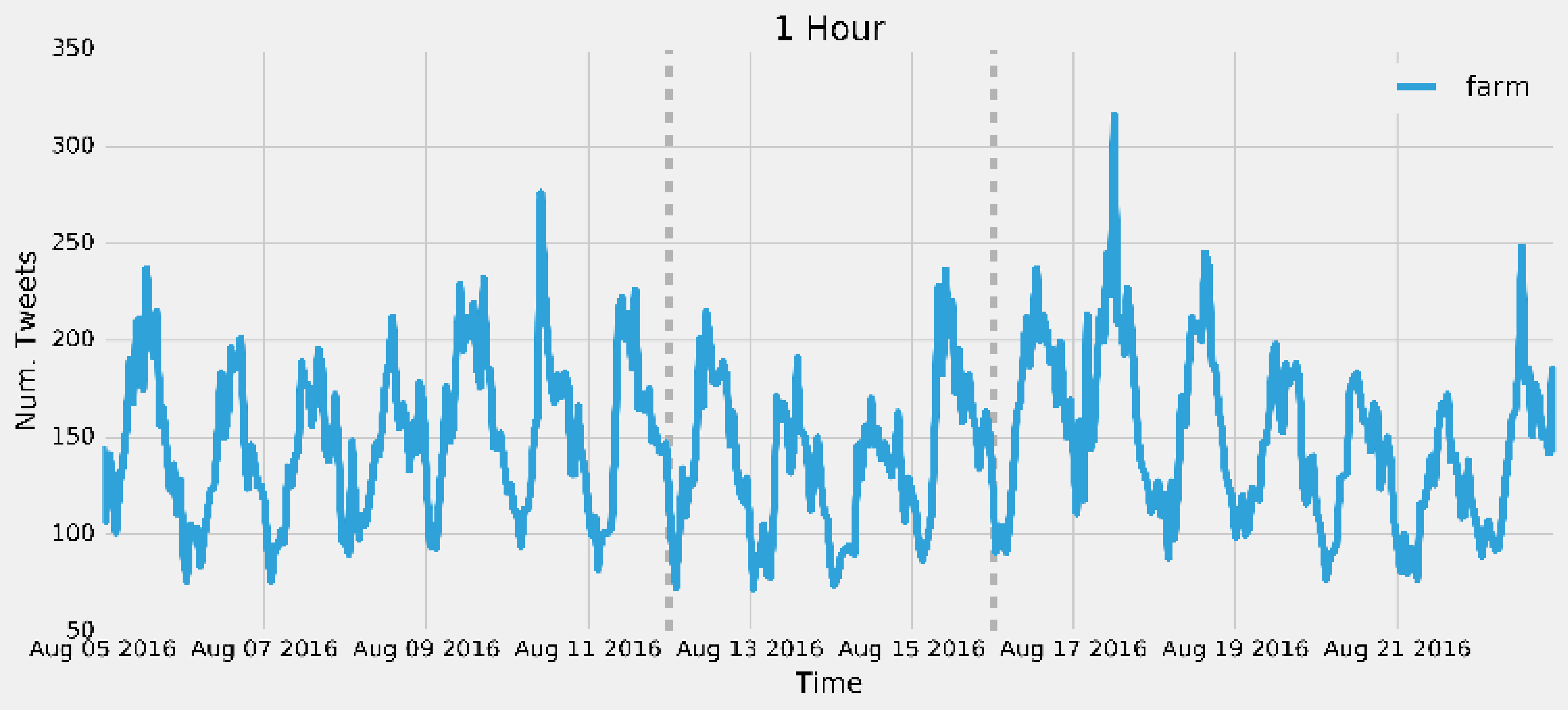
1 Hour
farm
Num. Tweets
350
300
250
200
150
100
50
Aug 05 2016
Aug 07 2016
Aug 09 2016
Aug 11 2016
Aug 13 2016
Aug 15 2016
Aug 17 2016
Aug 19 2016
Aug 21 2016
Time

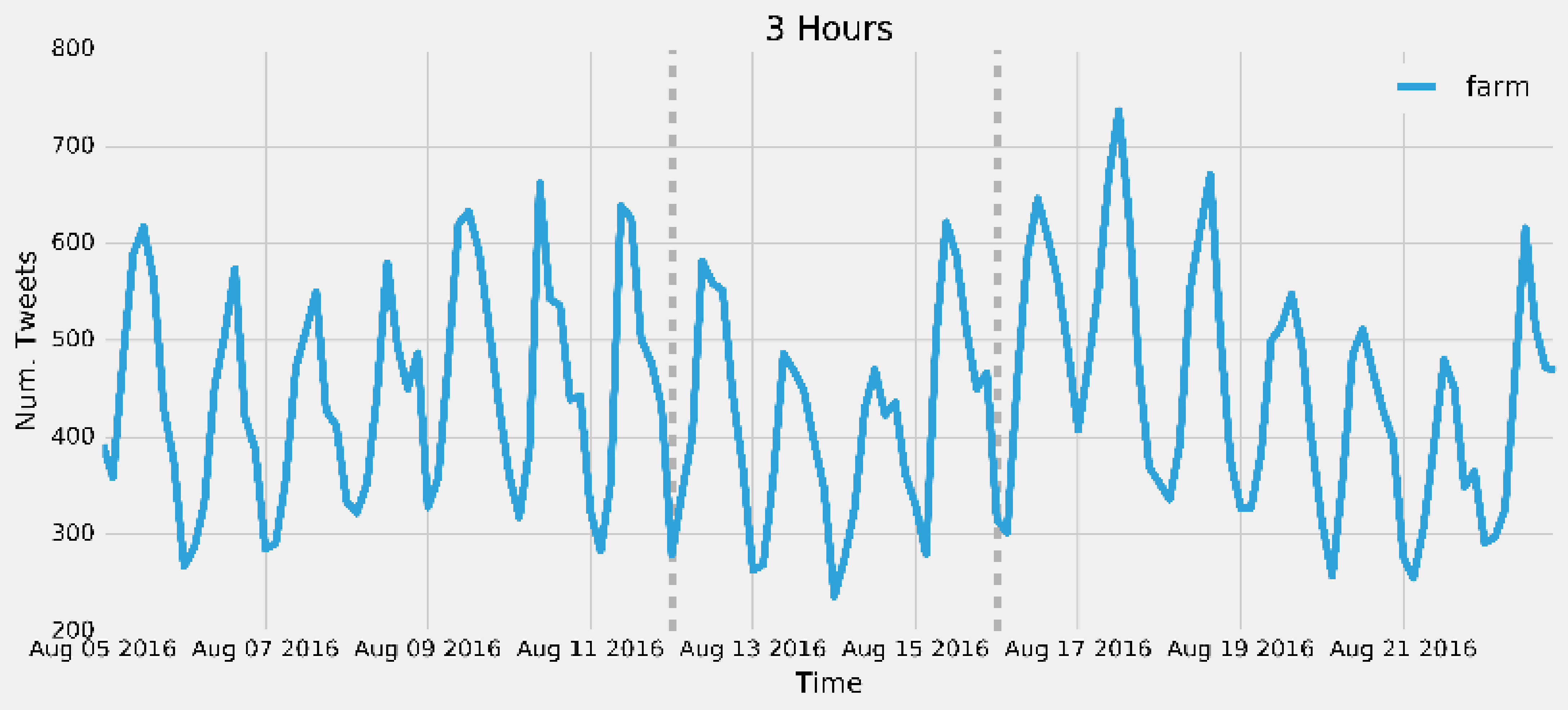

3 Hours
farm
800
700
600
500
400
300
200
Num. Tweets
Aug 05 2016
Aug 07 2016
Aug 09 2016
Aug 11 2016
Aug 13 2016
Aug 15 2016
Aug 17 2016
Aug 19 2016
Aug 21 2016
Time

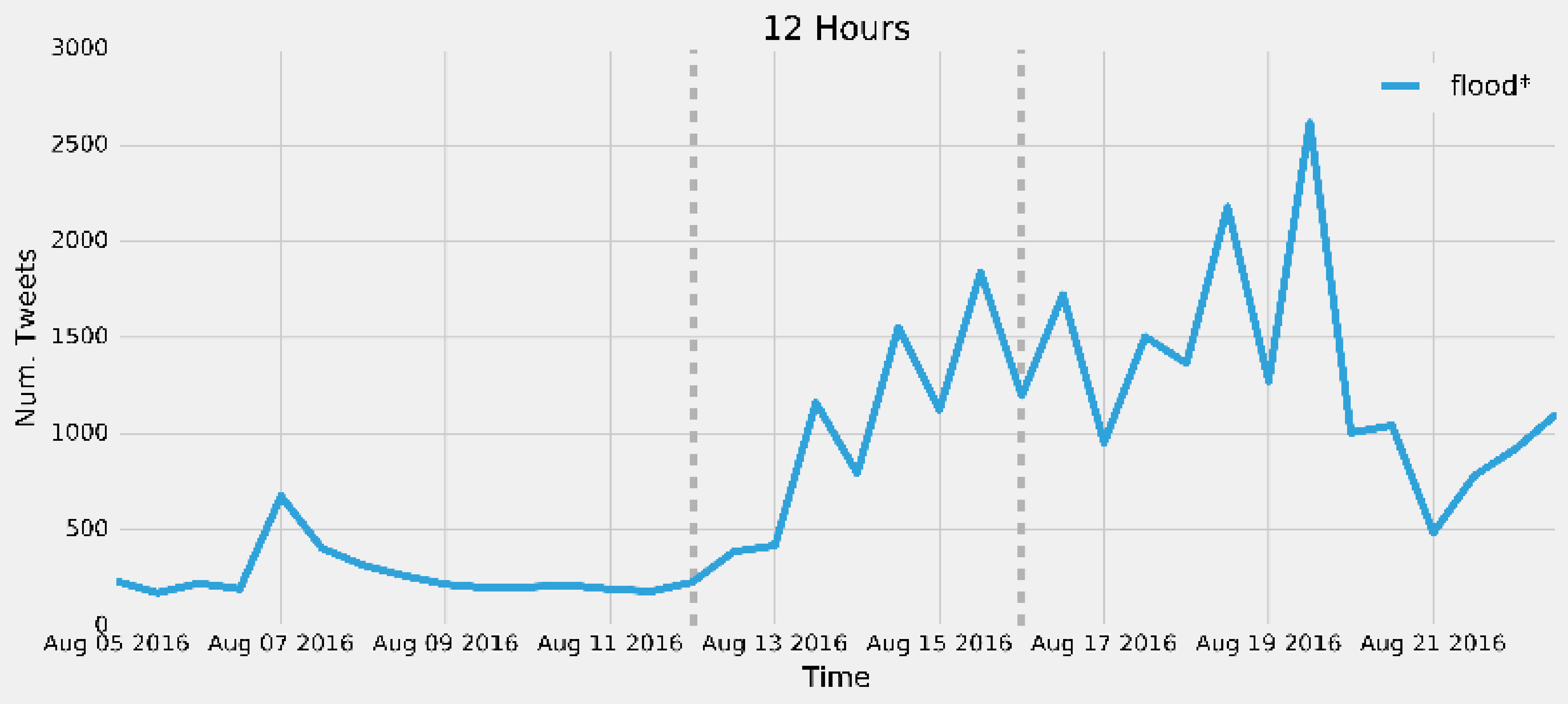

12 Hours
flood‡
Num. Tweets
3000
2500
2000
1500
1000
500
0
Aug 05 2016
Aug 07 2016
Aug 09 2016
Aug 11 2016
Aug 13 2016
Aug 15 2016
Aug 17 2016
Aug 19 2016
Aug 21 2016
Time

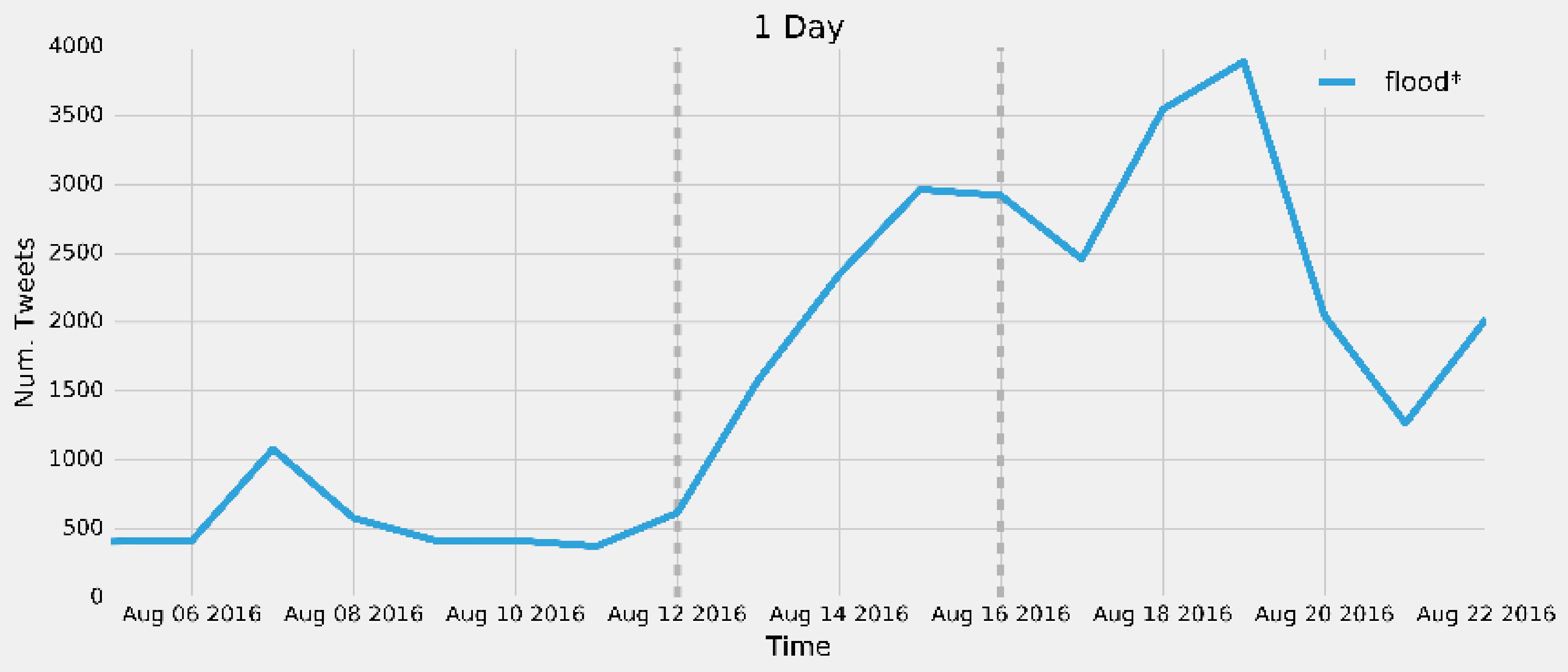
1 Day
4000
3500
3000
2500
2000
1500
1000
500
0
Num. Tweets
flood*
Aug 06 2016
Aug 08 2016
Aug 10 2016
Aug 12 2016
Aug 14 2016
Aug 16 2016
Aug 18 2016
Aug 20 2016
Aug 22 2016
Time

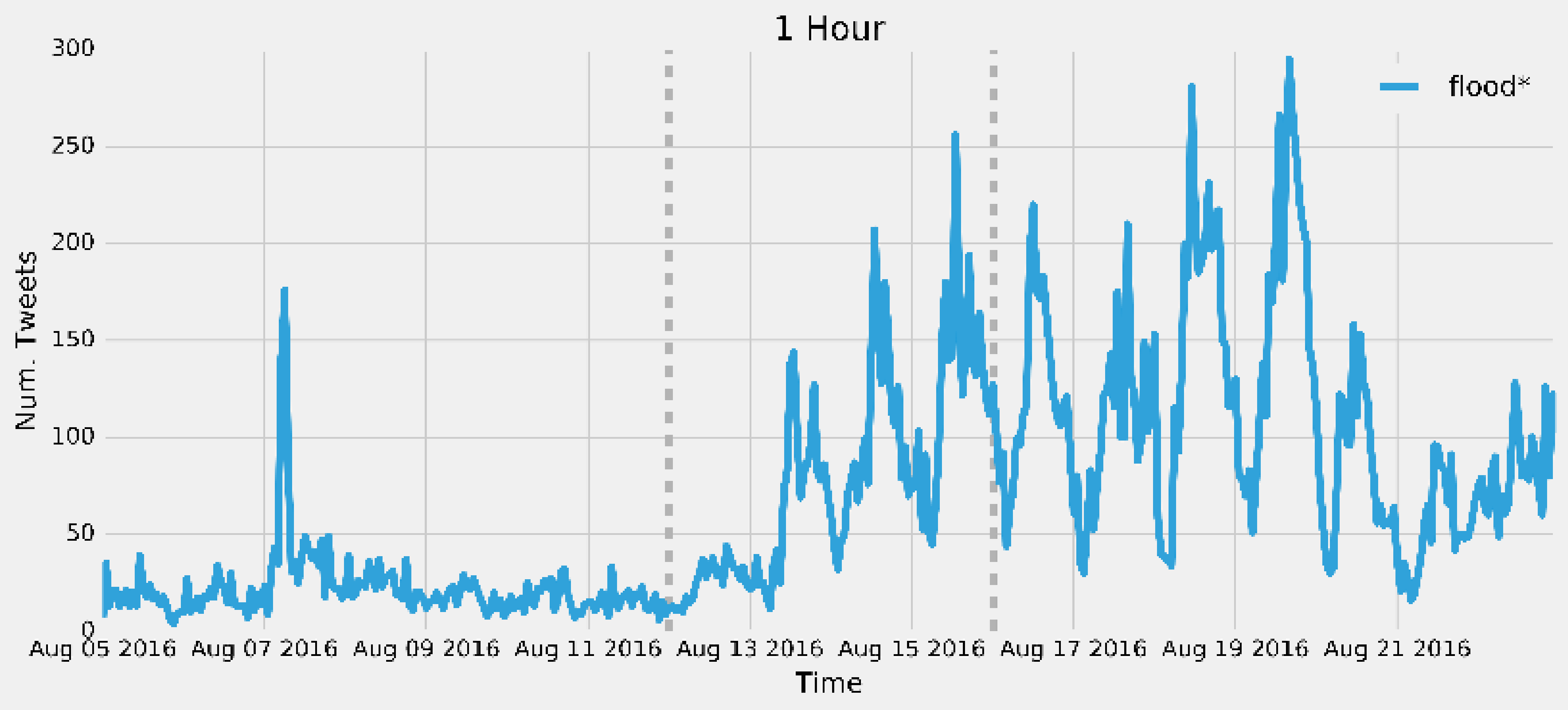

1 Hour
flood*
Num. Tweets
0
50
100
150
200
250
300
Aug 05 2016
Aug 07 2016
Aug 09 2016
Aug 11 2016
Aug 13 2016
Aug 15 2016
Aug 17 2016
Aug 19 2016
Aug 21 2016
Time

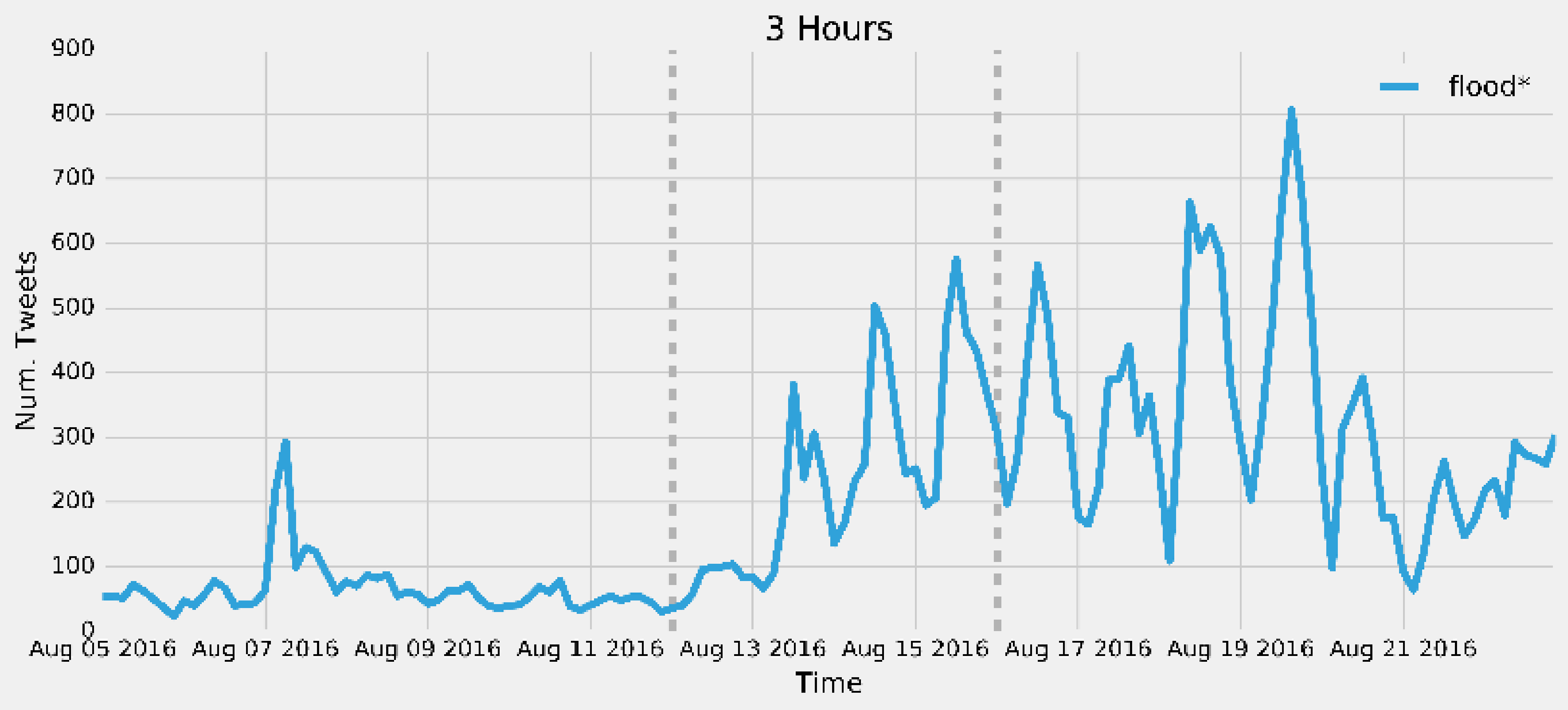
3 Hours
flood*
Num. Tweets
900
800
700
600
500
400
300
200
100
0
Aug 05 2016
Aug 07 2016
Aug 09 2016
Aug 11 2016
Aug 13 2016
Aug 15 2016
Aug 17 2016
Aug 19 2016
Aug 21 2016
Time

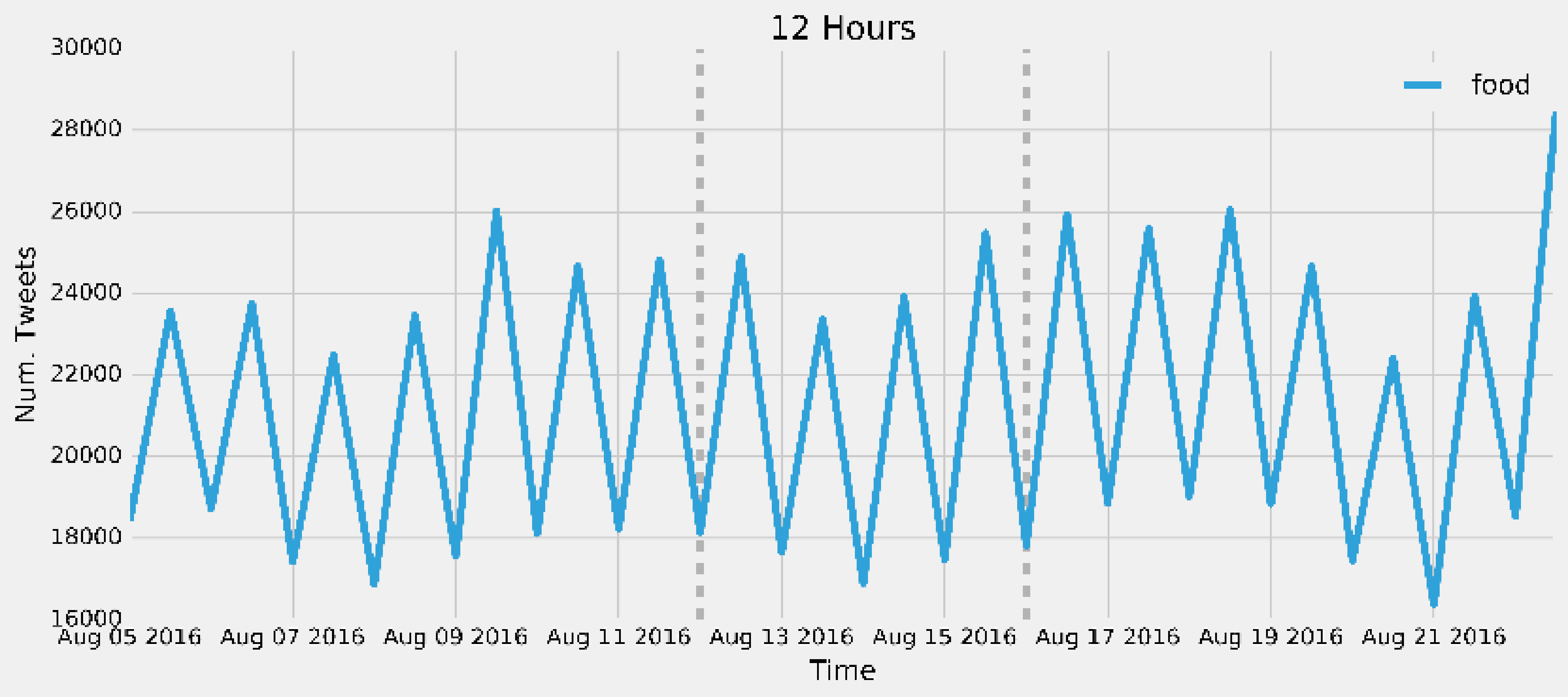

12 Hours
food
Num. Tweets
30000
28000
26000
24000
22000
20000
18000
16000
Aug 05 2016
Aug 07 2016
Aug 09 2016
Aug 11 2016
Aug 13 2016
Aug 15 2016
Aug 17 2016
Aug 19 2016
Aug 21 2016
Time

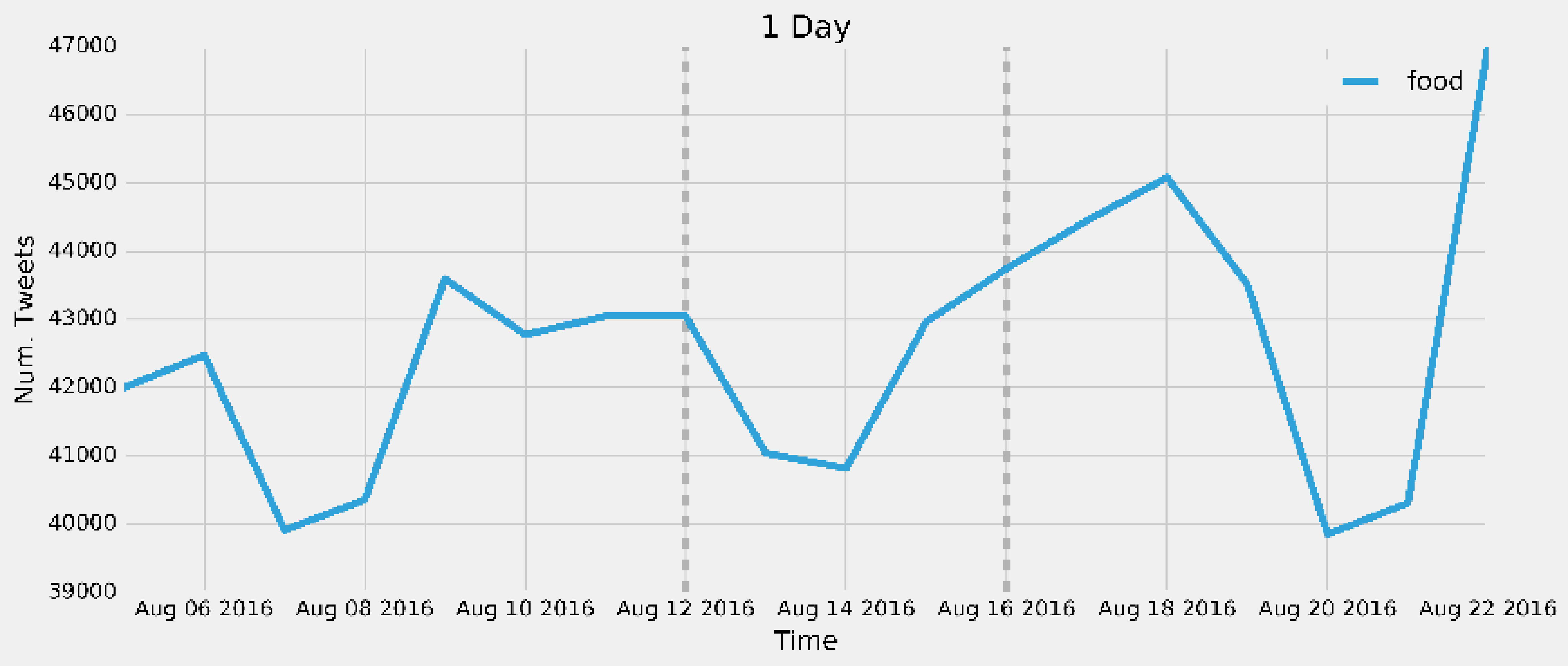
1 Day
food
Num. Tweets
47000
46000
45000
44000
43000
42000
41000
40000
39000
Aug 06 2016
Aug 08 2016
Aug 10 2016
Aug 12 2016
Aug 14 2016
Aug 16 2016
Aug 18 2016
Aug 20 2016
Aug 22 2016
Time

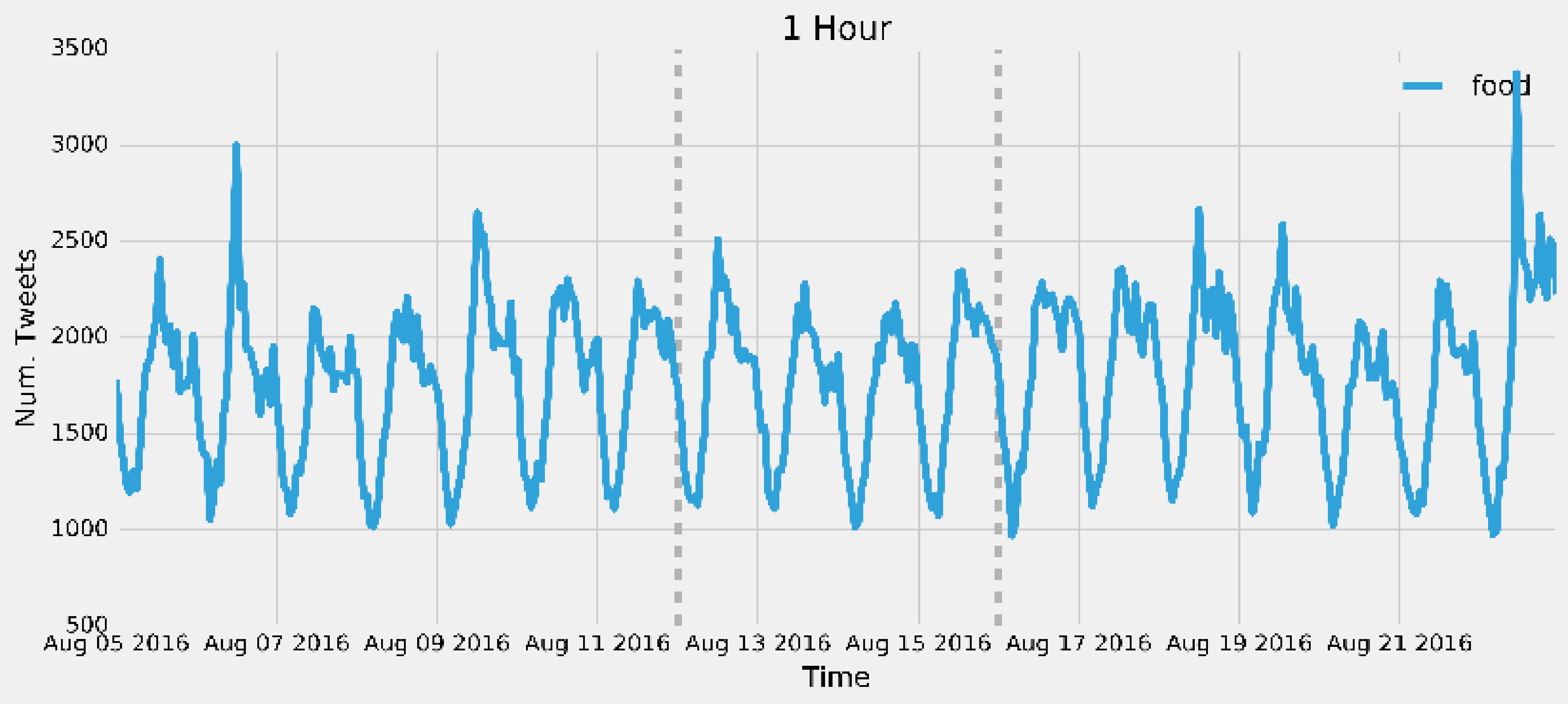
1 Hour
food
Num. Tweets
3500
3000
2500
2000
1500
1000
500
Aug 05 2016
Aug 07 2016
Aug 09 2016
Aug 11 2016
Aug 13 2016
Aug 15 2016
Aug 17 2016
Aug 19 2016
Aug 21 2016
Time

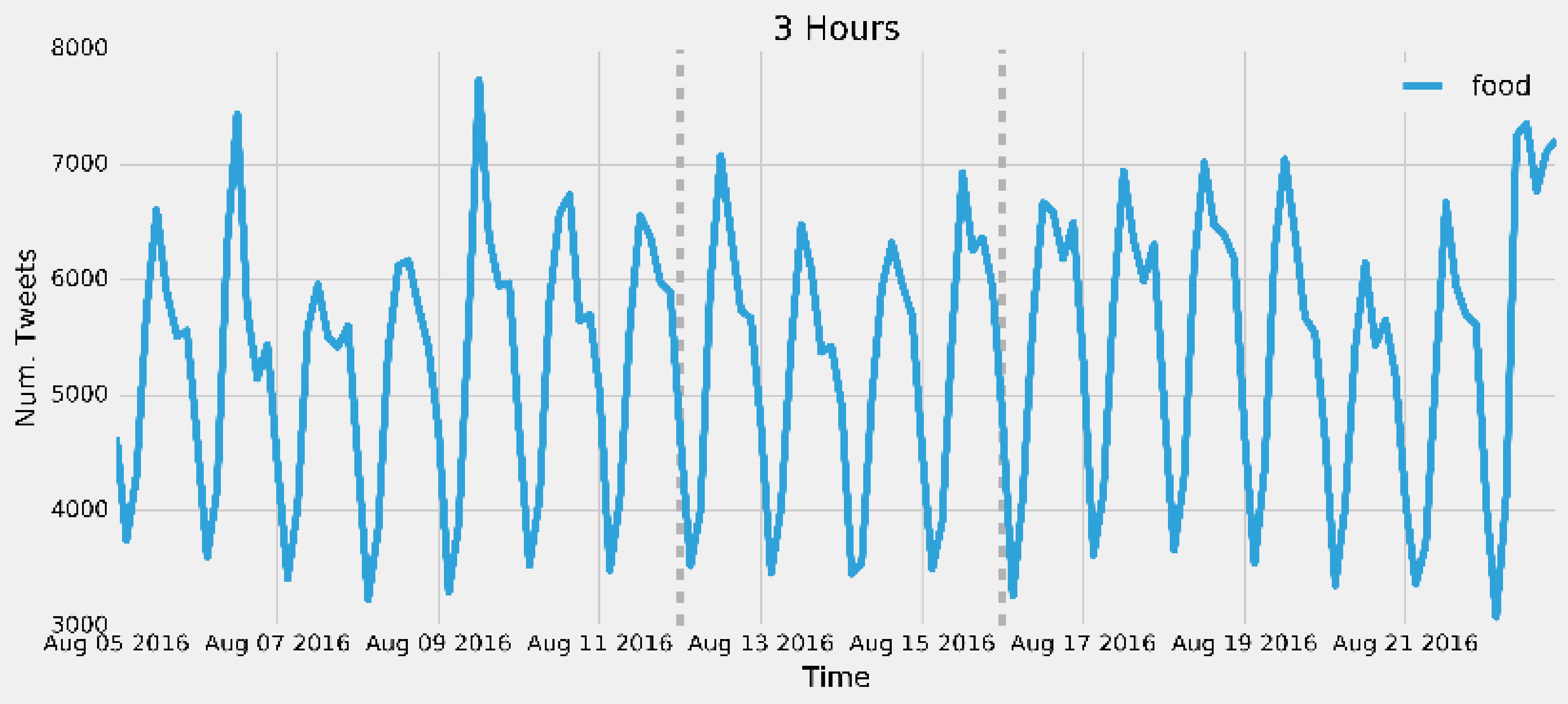

3 Hours
food
Num. Tweets
8000
7000
6000
5000
4000
3000
Aug 05 2016
Aug 07 2016
Aug 09 2016
Aug 11 2016
Aug 13 2016
Aug 15 2016
Aug 17 2016
Aug 19 2016
Aug 21 2016
Time

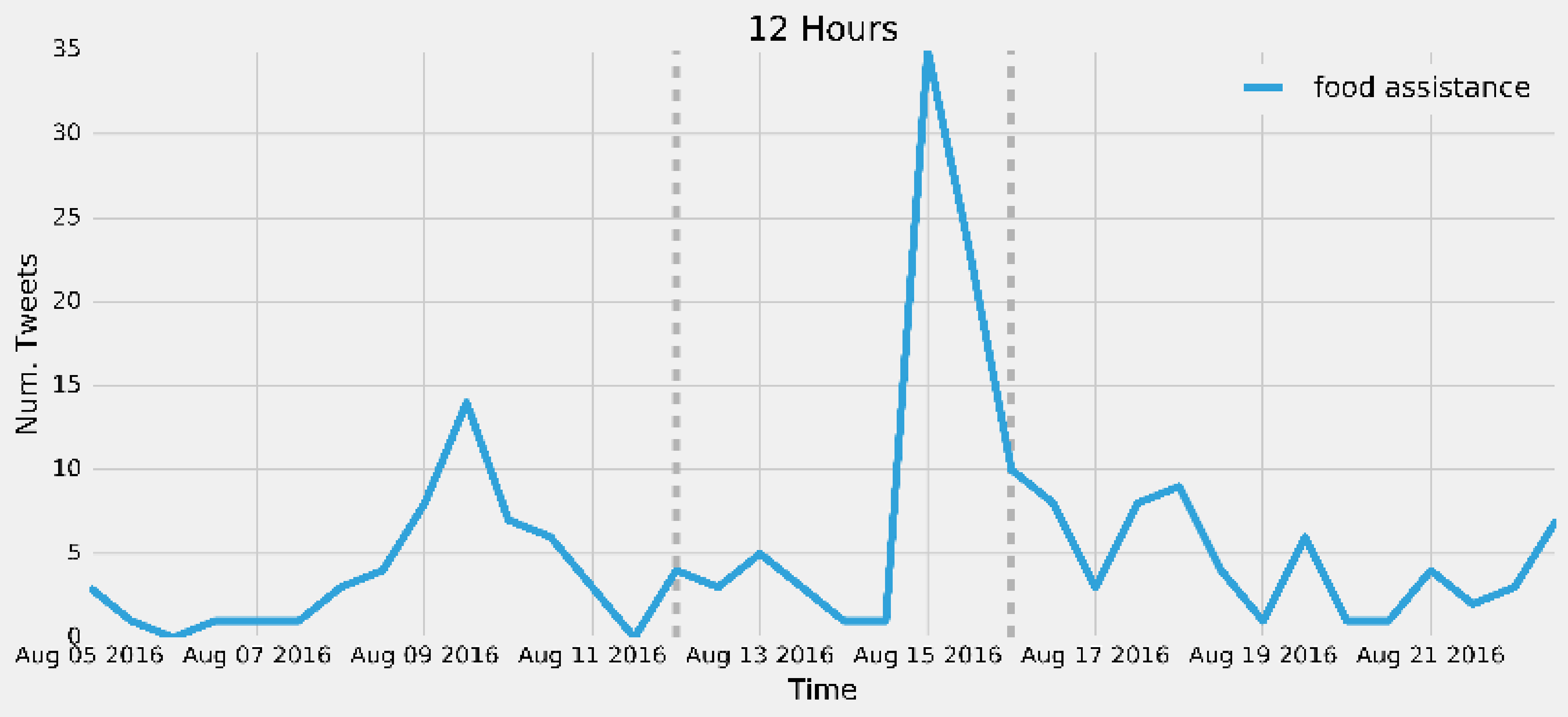

12 Hours
food assistance
Num. Tweets
0
5
10
15
20
25
30
35
Aug 05 2016
Aug 07 2016
Aug 09 2016
Aug 11 2016
Aug 13 2016
Aug 15 2016
Aug 17 2016
Aug 19 2016
Aug 21 2016
Time

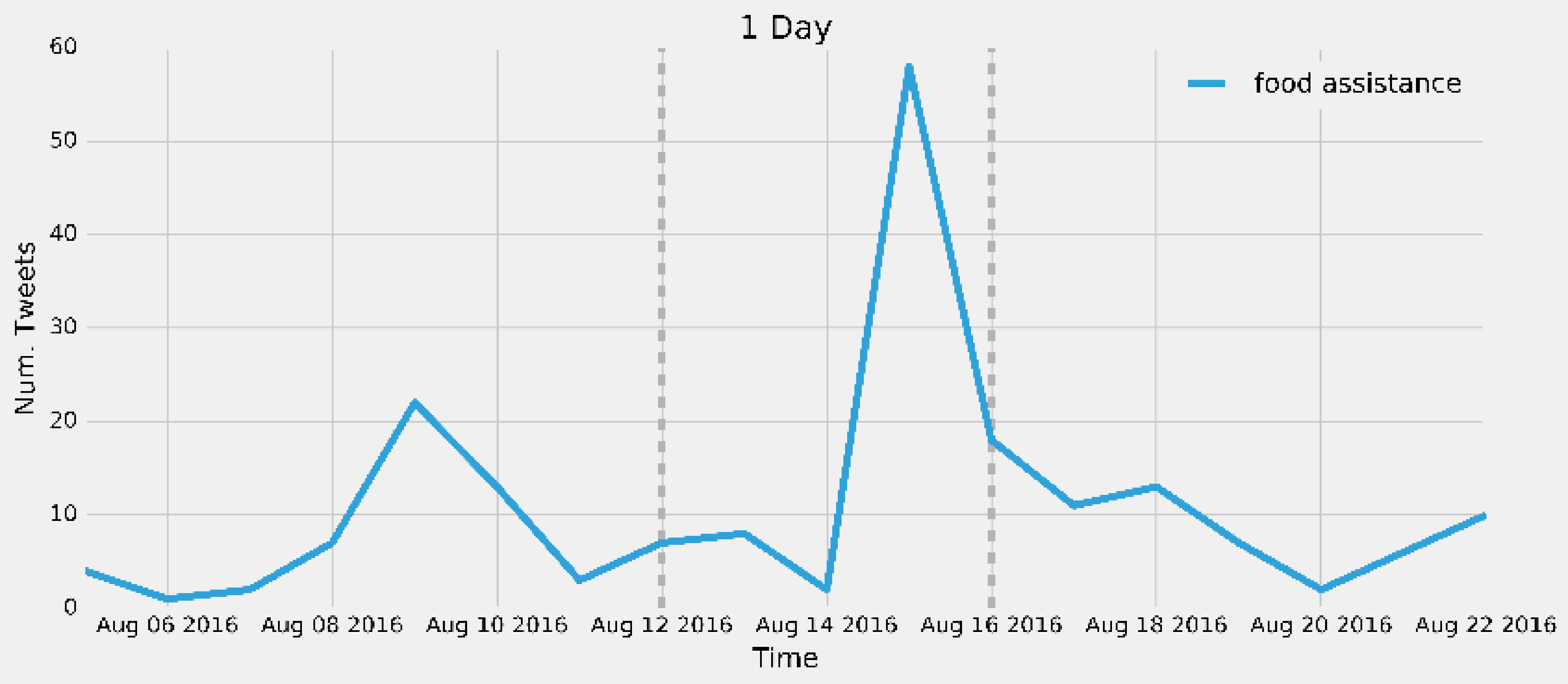
1 Day
food assistance
Num. Tweets
0
10
20
30
40
50
60
Aug 06 2016
Aug 08 2016
Aug 10 2016
Aug 12 2016
Aug 14 2016
Aug 16 2016
Aug 18 2016
Aug 20 2016
Aug 22 2016
Time

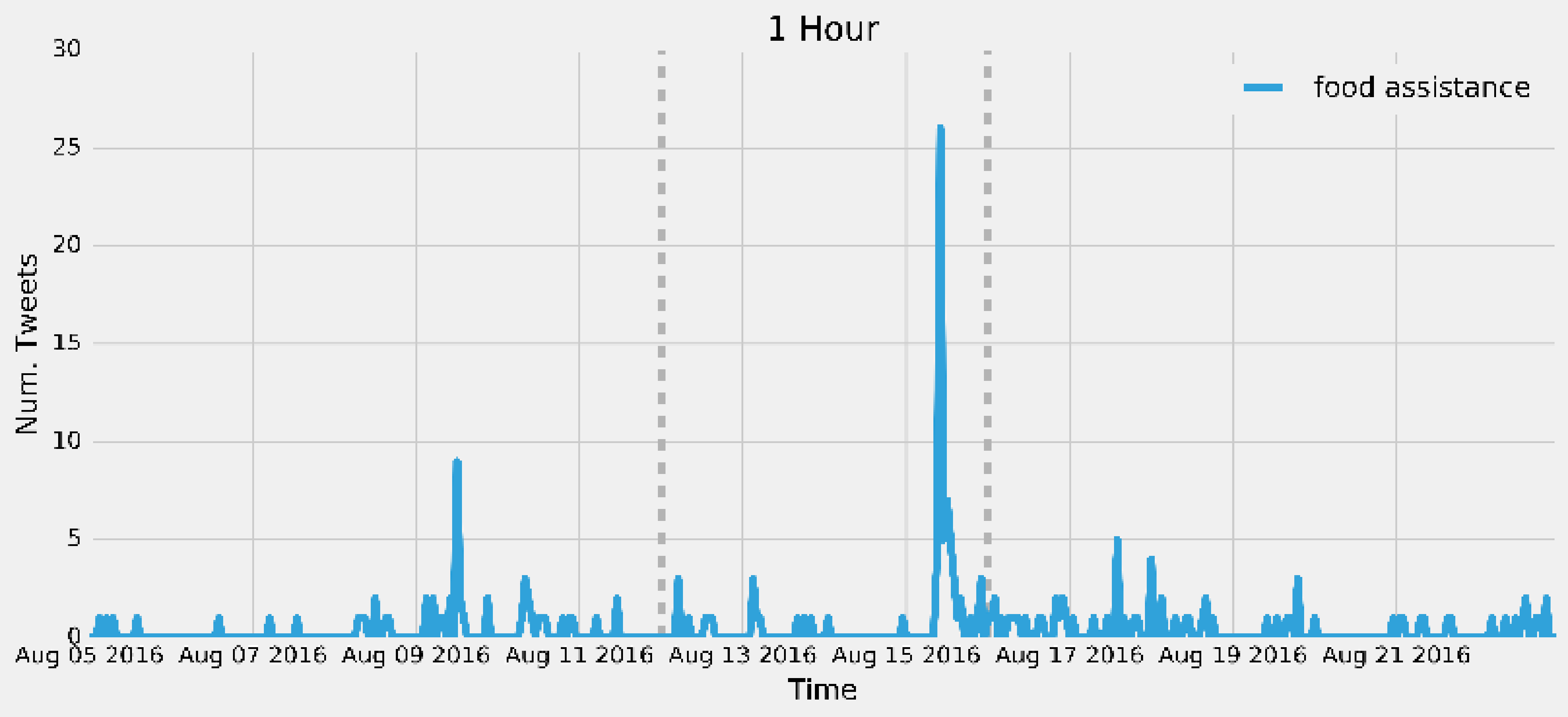
1 Hour
food assistance
Num. Tweets
Time
0
5
10
15
20
25
30
Aug 05 2016
Aug 07 2016
Aug 09 2016
Aug 11 2016
Aug 13 2016
Aug 15 2016
Aug 17 2016
Aug 19 2016
Aug 21 2016

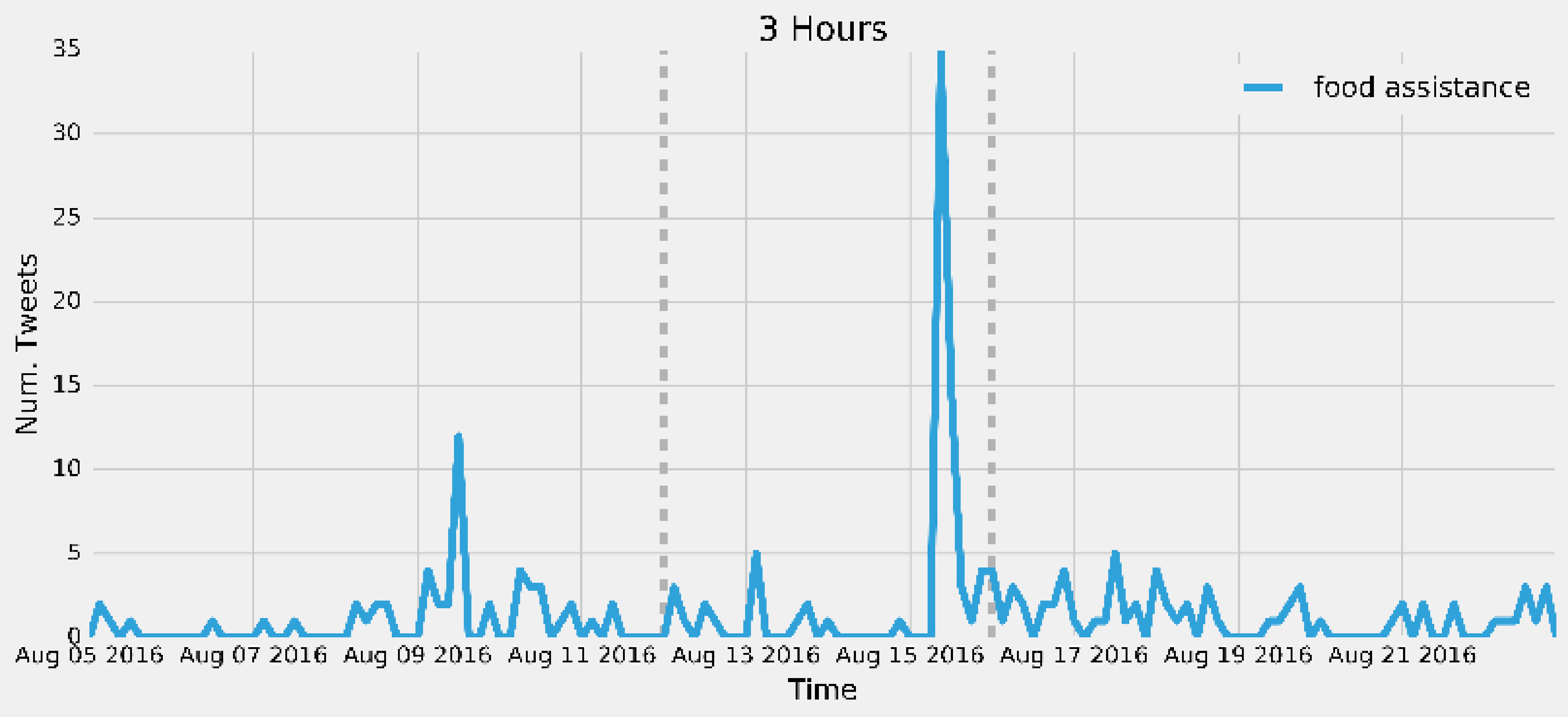
3 Hours
food assistance
Num. Tweets
0
5
10
15
20
25
30
35
Aug 05 2016
Aug 07 2016
Aug 09 2016
Aug 11 2016
Aug 13 2016
Aug 15 2016
Aug 17 2016
Aug 19 2016
Aug 21 2016
Time

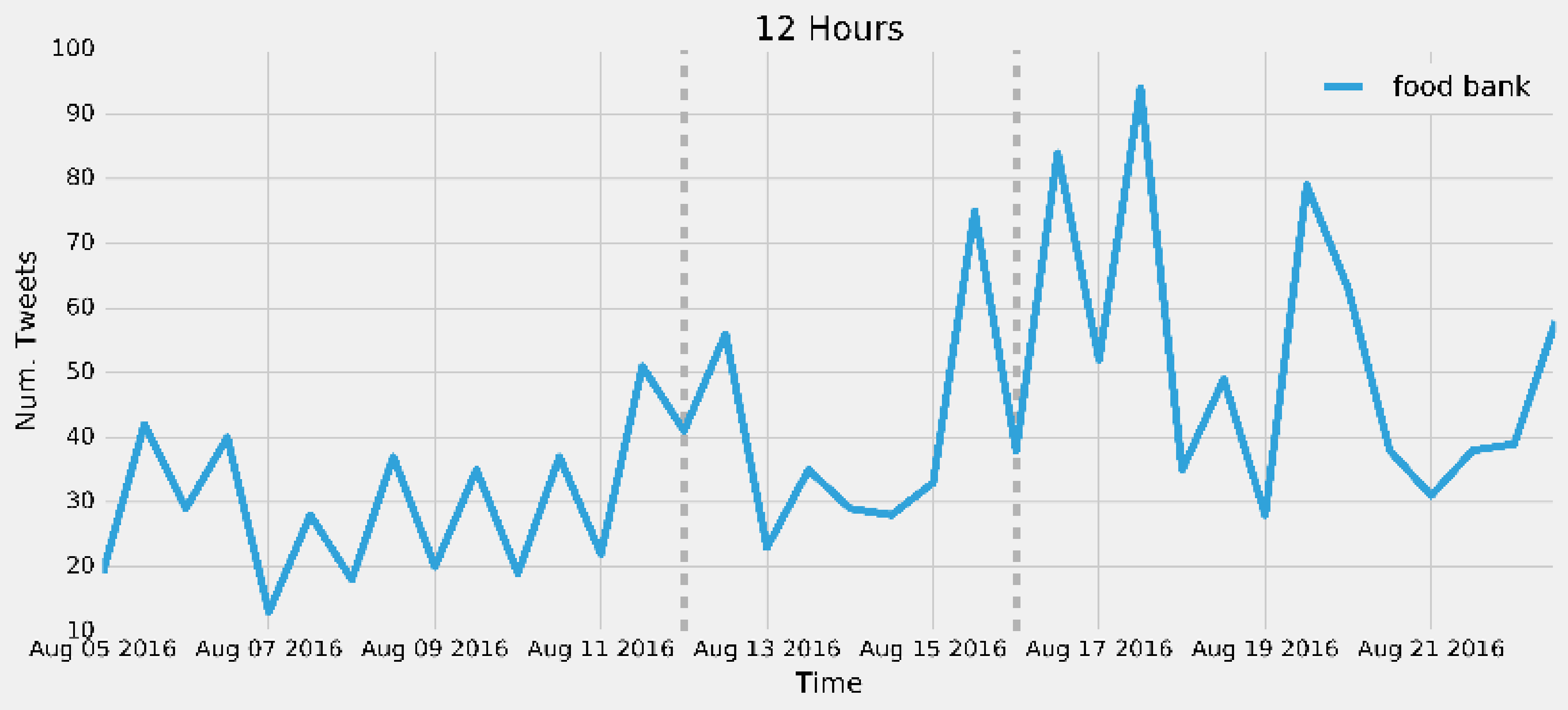
12 Hours
food bank
Num. Tweets
Time
100
90
80
70
60
50
40
30
20
10
Aug 05 2016
Aug 07 2016
Aug 09 2016
Aug 11 2016
Aug 13 2016
Aug 15 2016
Aug 17 2016
Aug 19 2016
Aug 21 2016

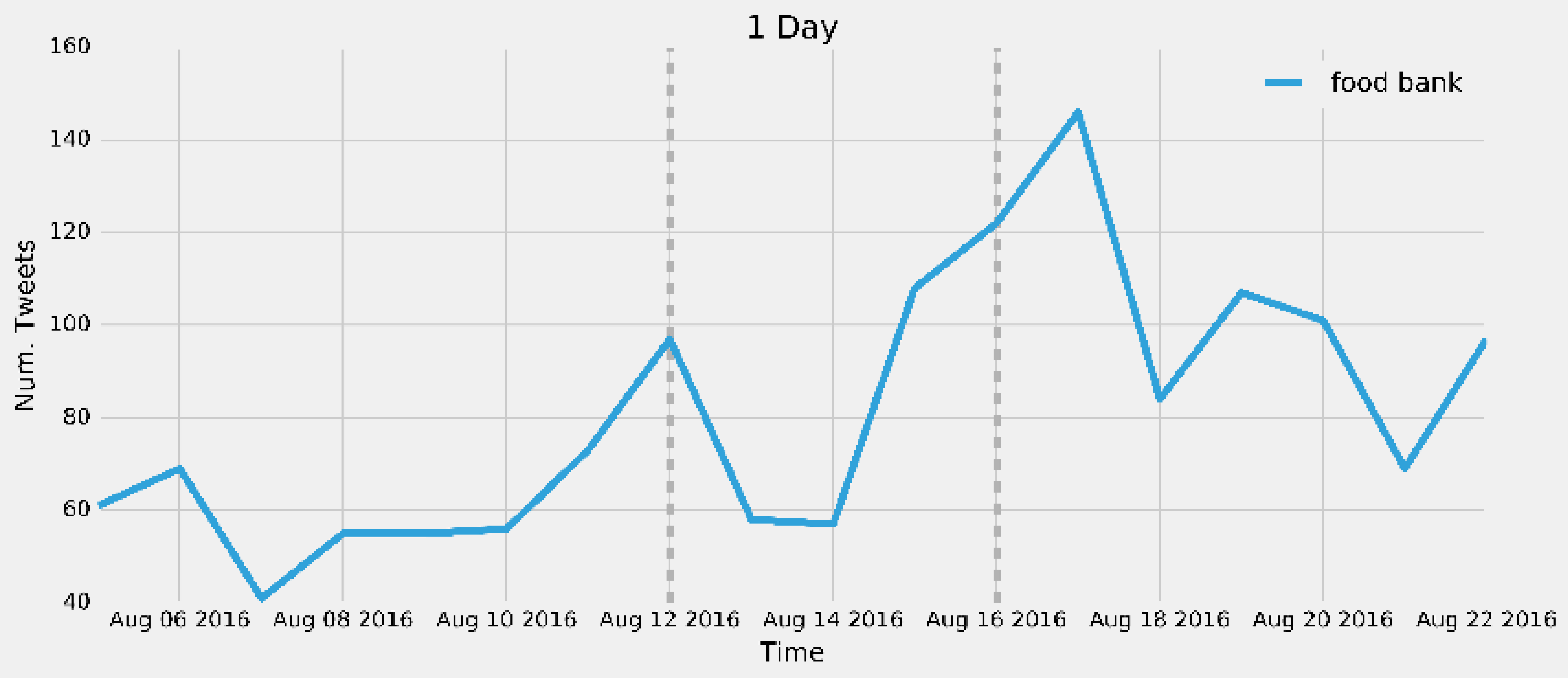
1 Day
food bank
Num. Tweets
160
140
120
100
80
60
40
Aug 06 2016
Aug 08 2016
Aug 10 2016
Aug 12 2016
Aug 14 2016
Aug 16 2016
Aug 18 2016
Aug 20 2016
Aug 22 2016
Time

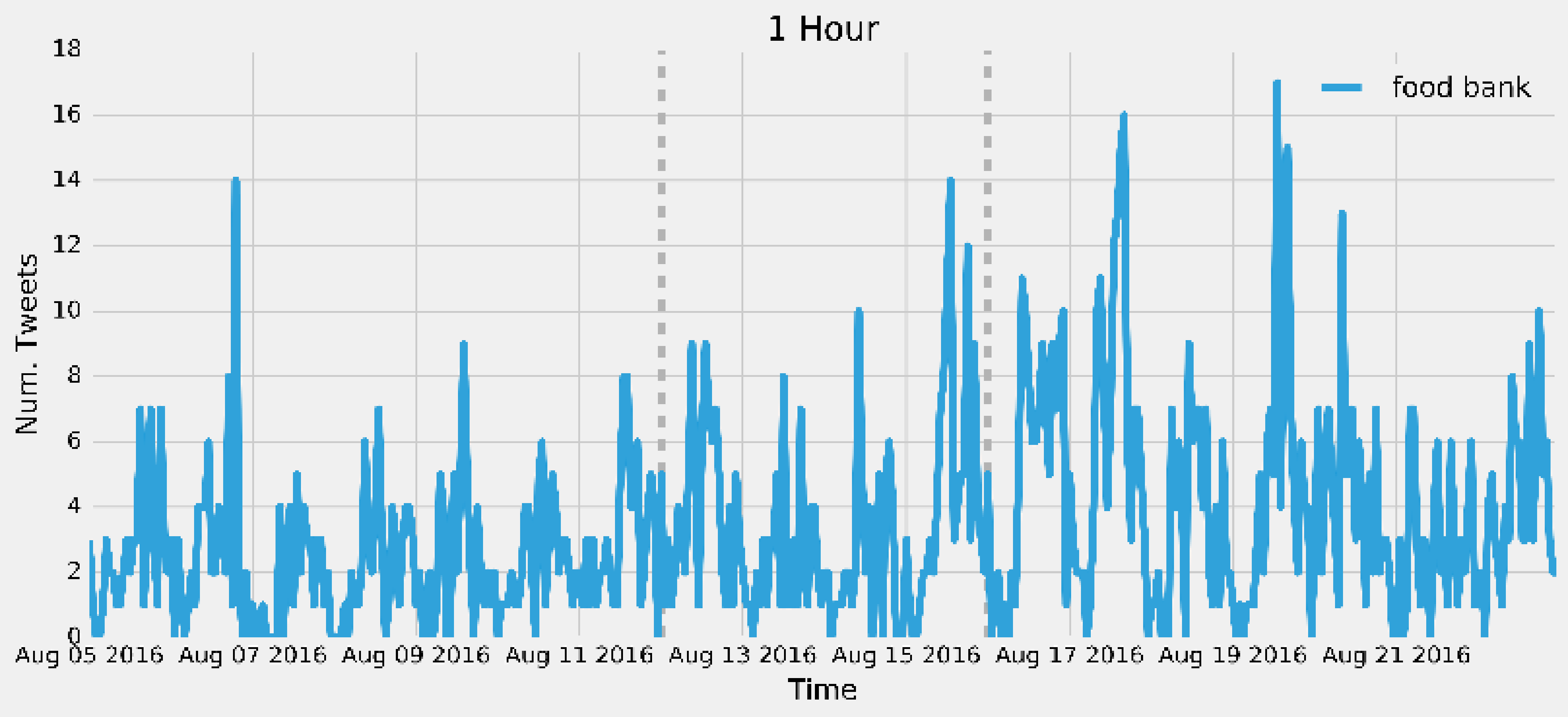

1 Hour
food bank
Num. Tweets
0
2
4
6
8
10
12
14
16
18
Aug 05 2016
Aug 07 2016
Aug 09 2016
Aug 11 2016
Aug 13 2016
Aug 15 2016
Aug 17 2016
Aug 19 2016
Aug 21 2016
Time

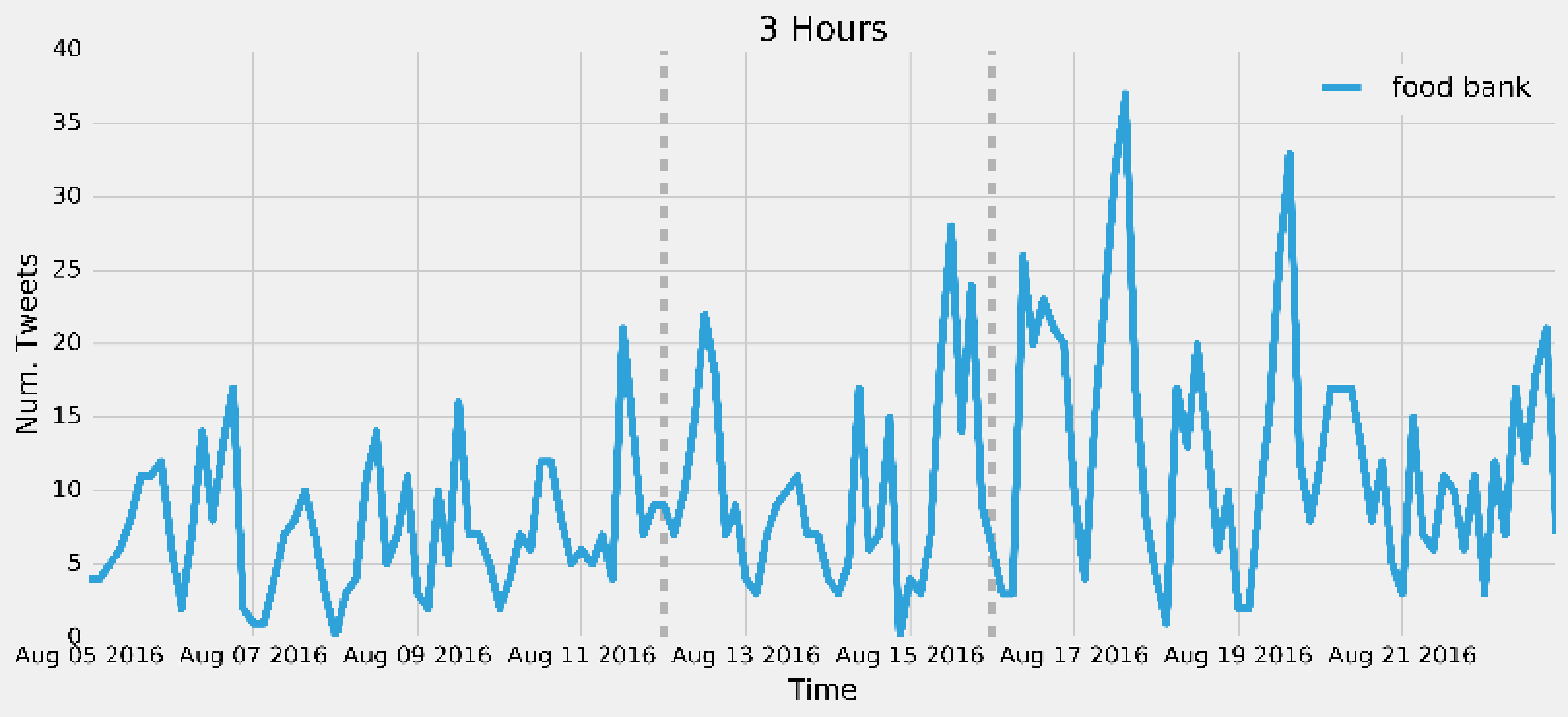
3 Hours
food bank
Num. Tweets
0
5
10
15
20
25
30
35
40
Aug 05 2016
Aug 07 2016
Aug 09 2016
Aug 11 2016
Aug 13 2016
Aug 15 2016
Aug 17 2016
Aug 19 2016
Aug 21 2016
Time

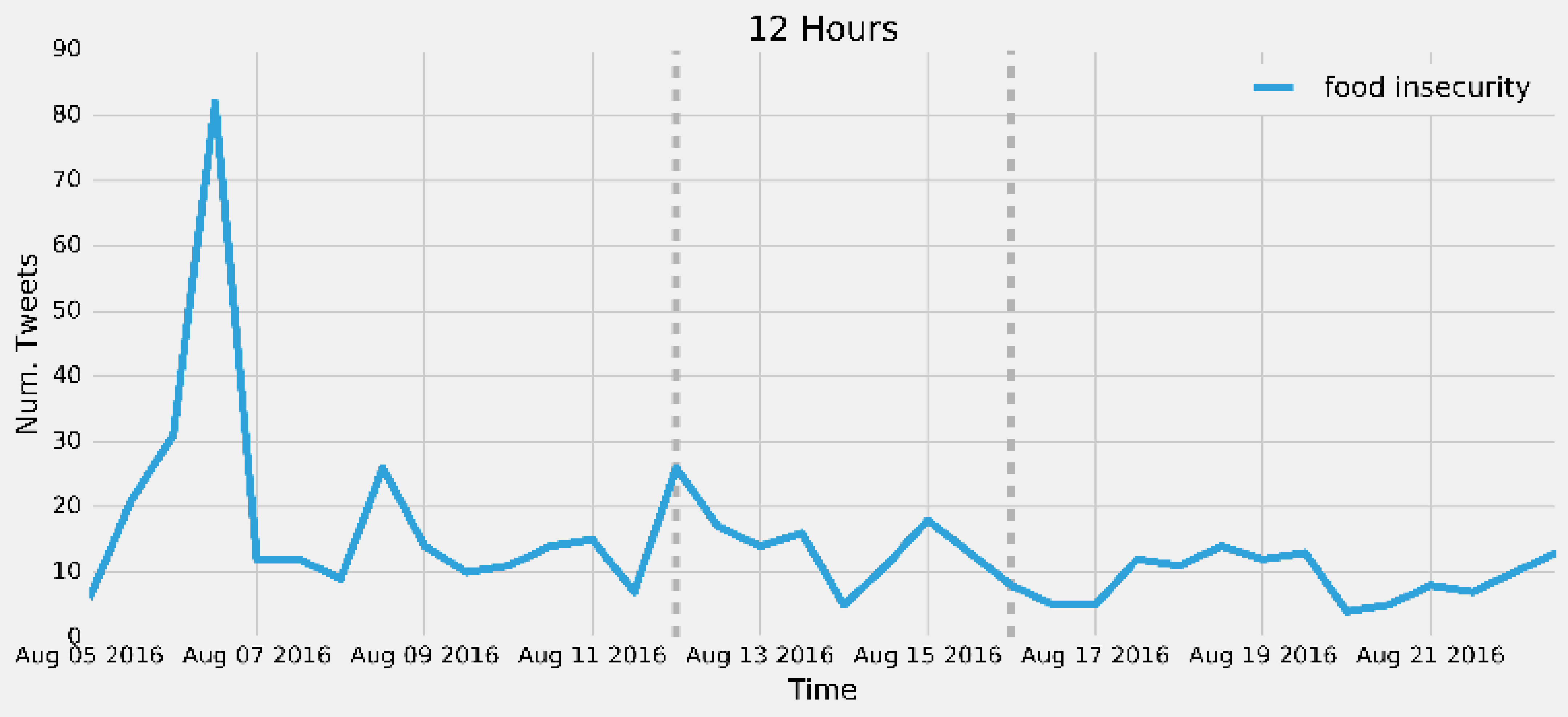
12 Hours
food insecurity
Num. Tweets
Time
0
10
20
30
40
50
60
70
80
90
Aug 05 2016
Aug 07 2016
Aug 09 2016
Aug 11 2016
Aug 13 2016
Aug 15 2016
Aug 17 2016
Aug 19 2016
Aug 21 2016

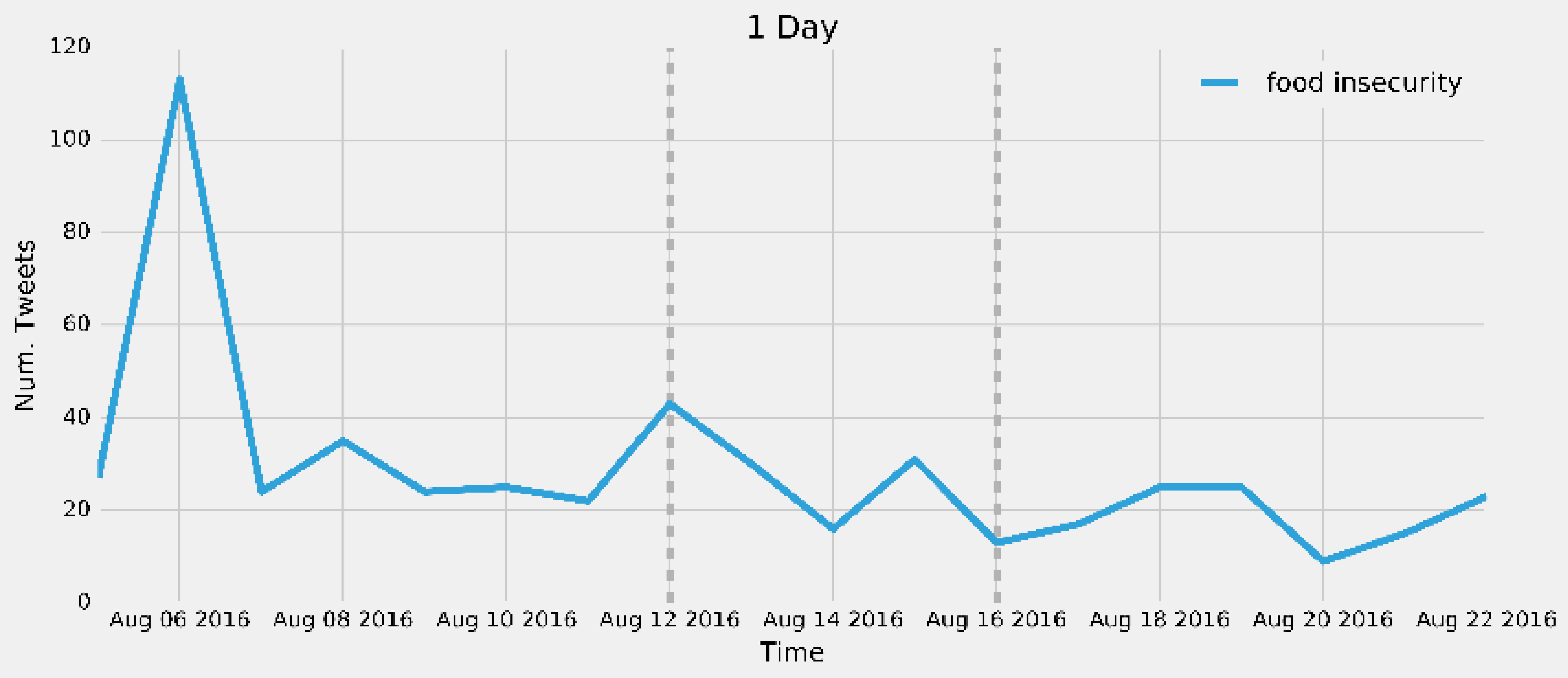

1 Day
food insecurity
Num. Tweets
0
20
40
60
80
100
120
Aug 06 2016
Aug 08 2016
Aug 10 2016
Aug 12 2016
Aug 14 2016
Aug 16 2016
Aug 18 2016
Aug 20 2016
Aug 22 2016
Time

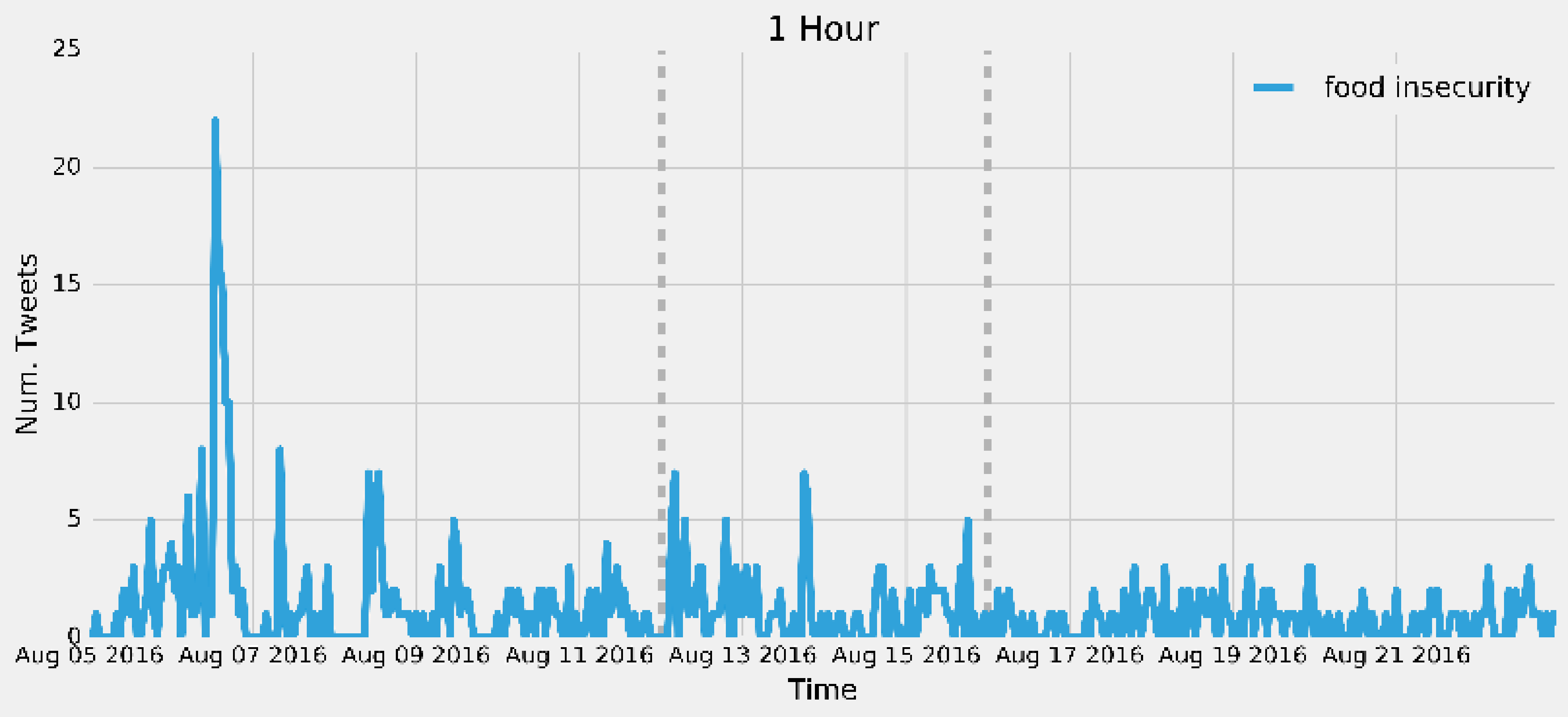
1 Hour
food insecurity
Num. Tweets
0
5
10
15
20
25
Aug 05 2016
Aug 07 2016
Aug 09 2016
Aug 11 2016
Aug 13 2016
Aug 15 2016
Aug 17 2016
Aug 19 2016
Aug 21 2016
Time

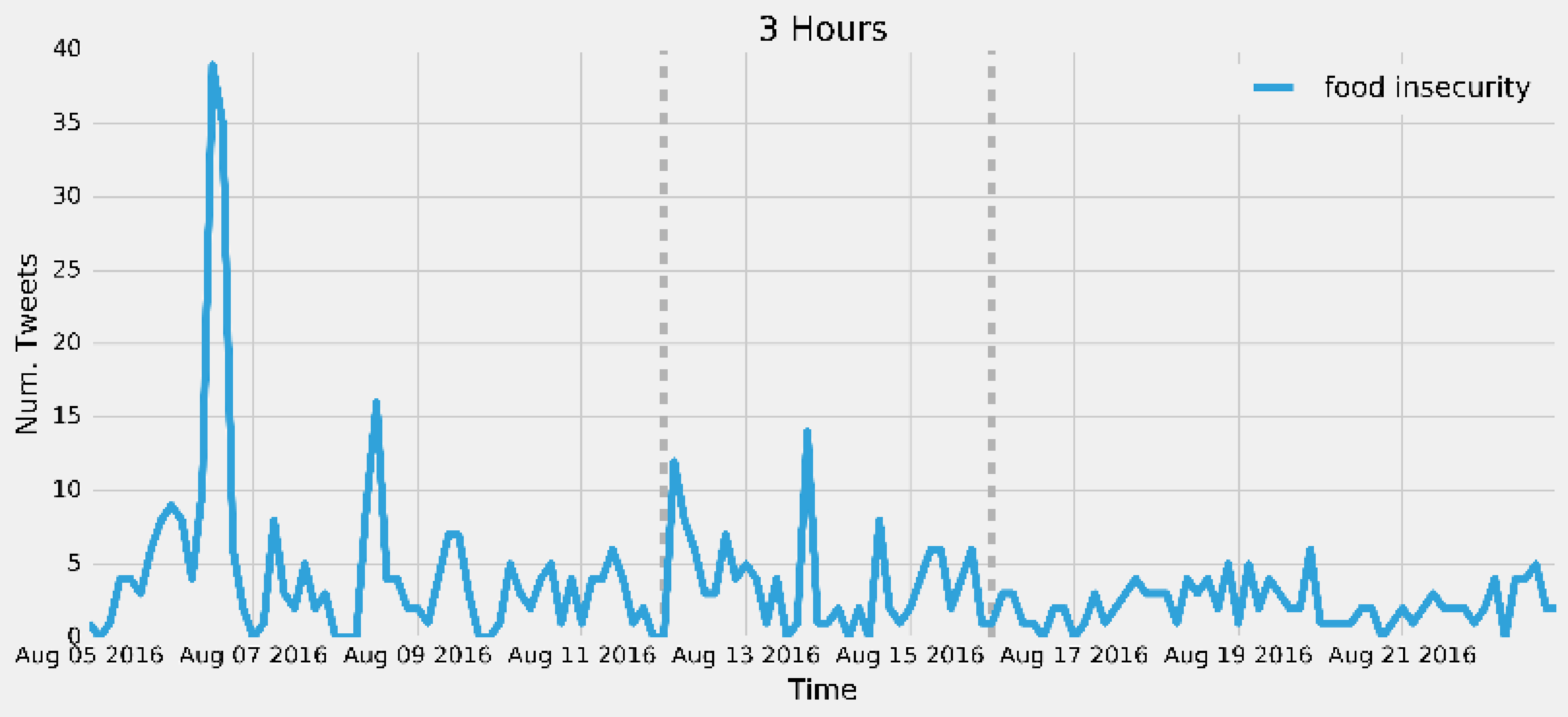
3 Hours
food insecurity
Num. Tweets
0
5
10
15
20
25
30
35
40
Aug 05 2016
Aug 07 2016
Aug 09 2016
Aug 11 2016
Aug 13 2016
Aug 15 2016
Aug 17 2016
Aug 19 2016
Aug 21 2016
Time

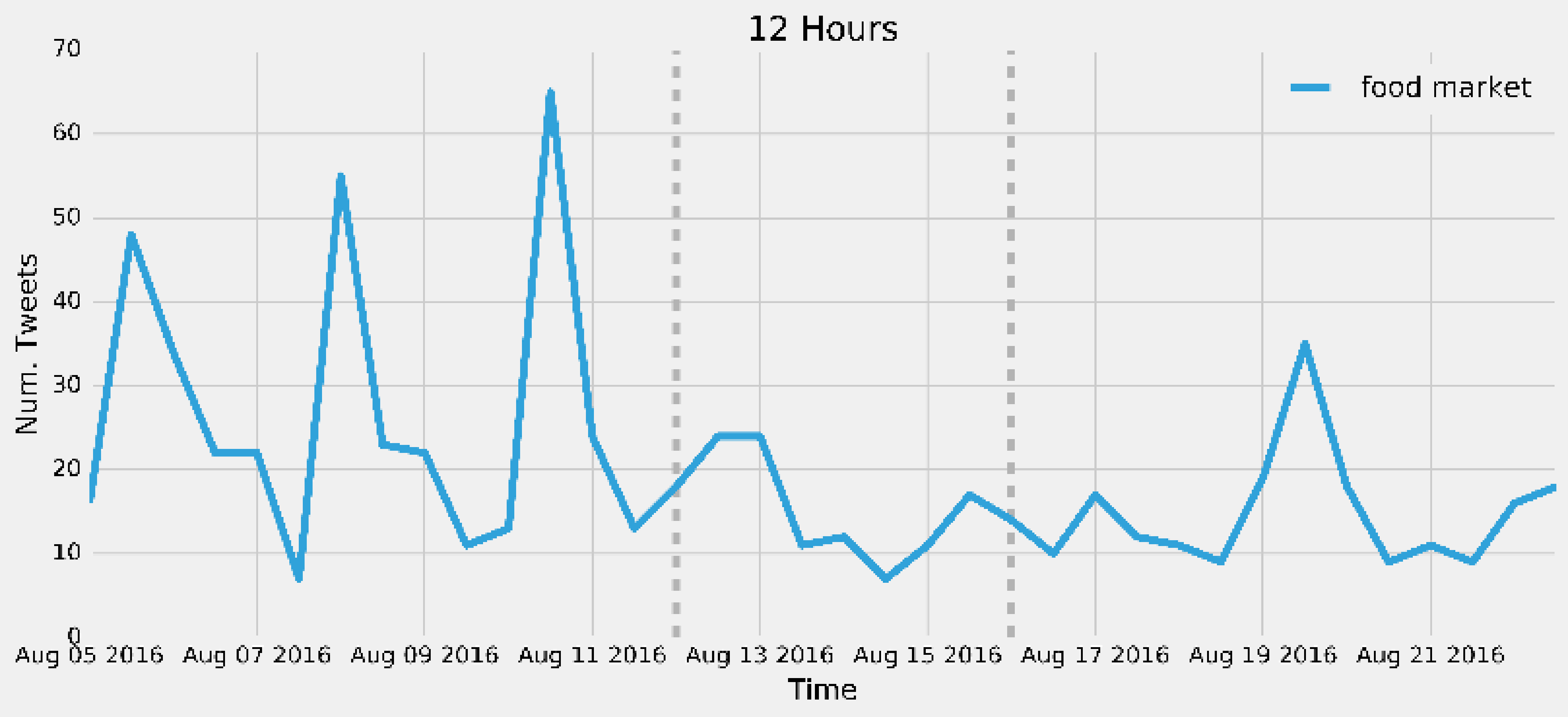
12 Hours
food market
Num. Tweets
0
10
20
30
40
50
60
70
Aug 05 2016
Aug 07 2016
Aug 09 2016
Aug 11 2016
Aug 13 2016
Aug 15 2016
Aug 17 2016
Aug 19 2016
Aug 21 2016
Time

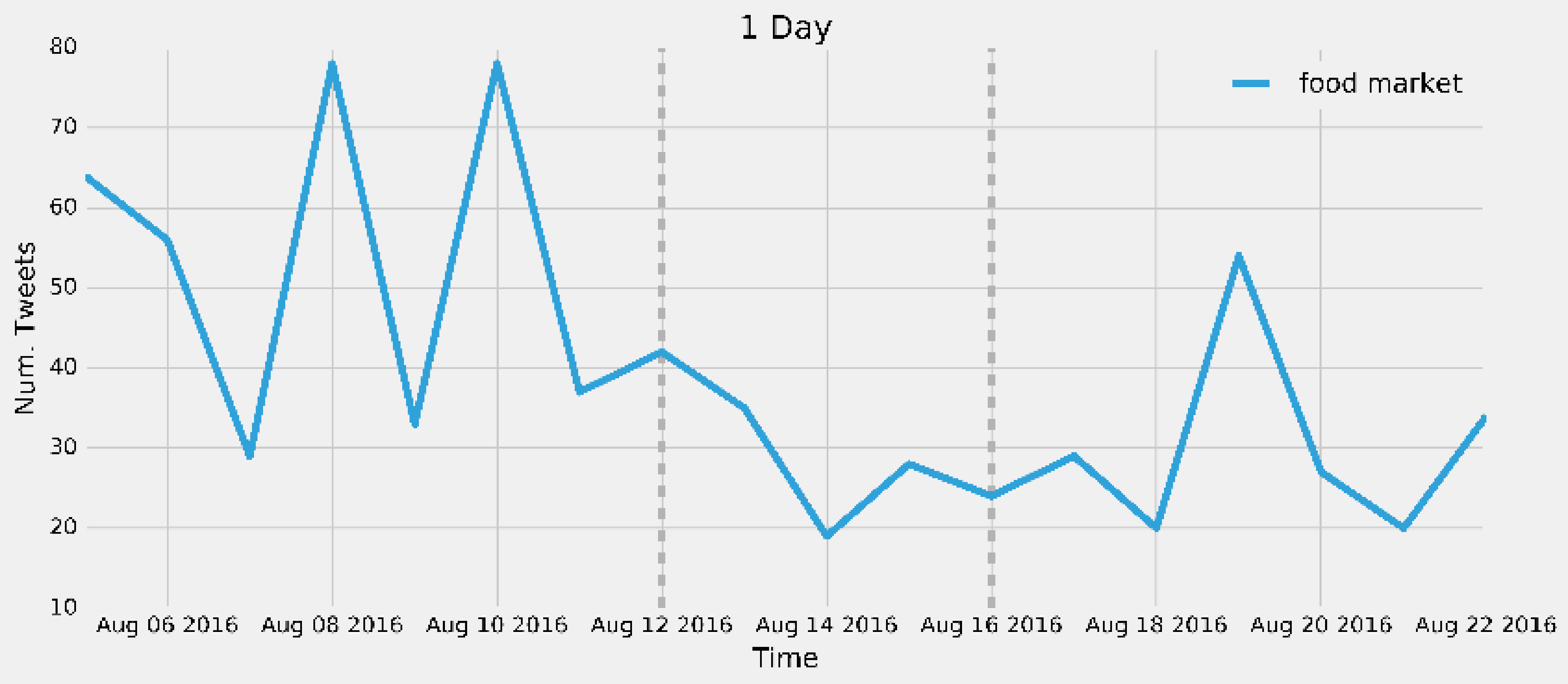

1 Day
food market
Num. Tweets
Time
80
70
60
50
40
30
20
10
Aug 06 2016
Aug 08 2016
Aug 10 2016
Aug 12 2016
Aug 14 2016
Aug 16 2016
Aug 18 2016
Aug 20 2016
Aug 22 2016

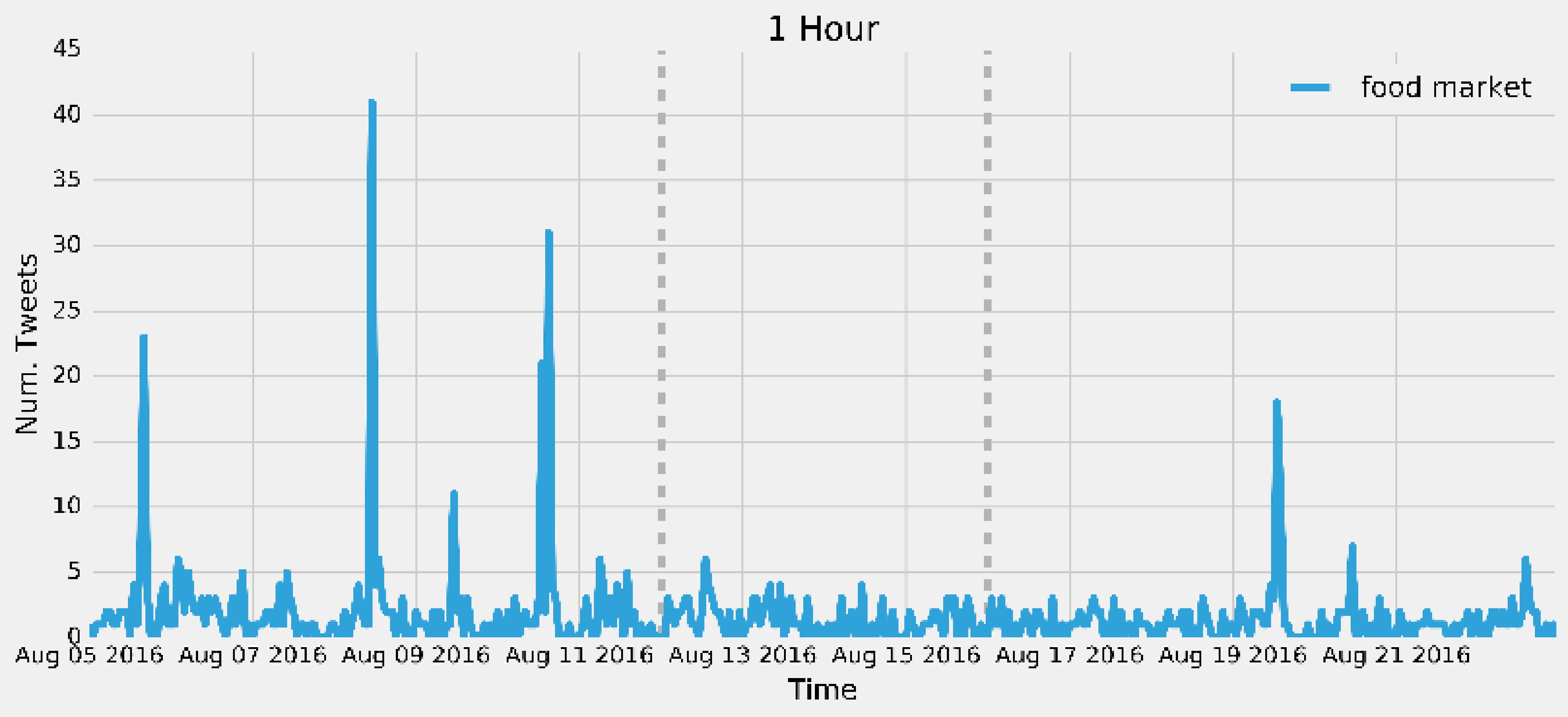

1 Hour
food market
Num. Tweets
0
5
10
15
20
25
30
35
40
45
Aug 05 2016
Aug 07 2016
Aug 09 2016
Aug 11 2016
Aug 13 2016
Aug 15 2016
Aug 17 2016
Aug 19 2016
Aug 21 2016
Time

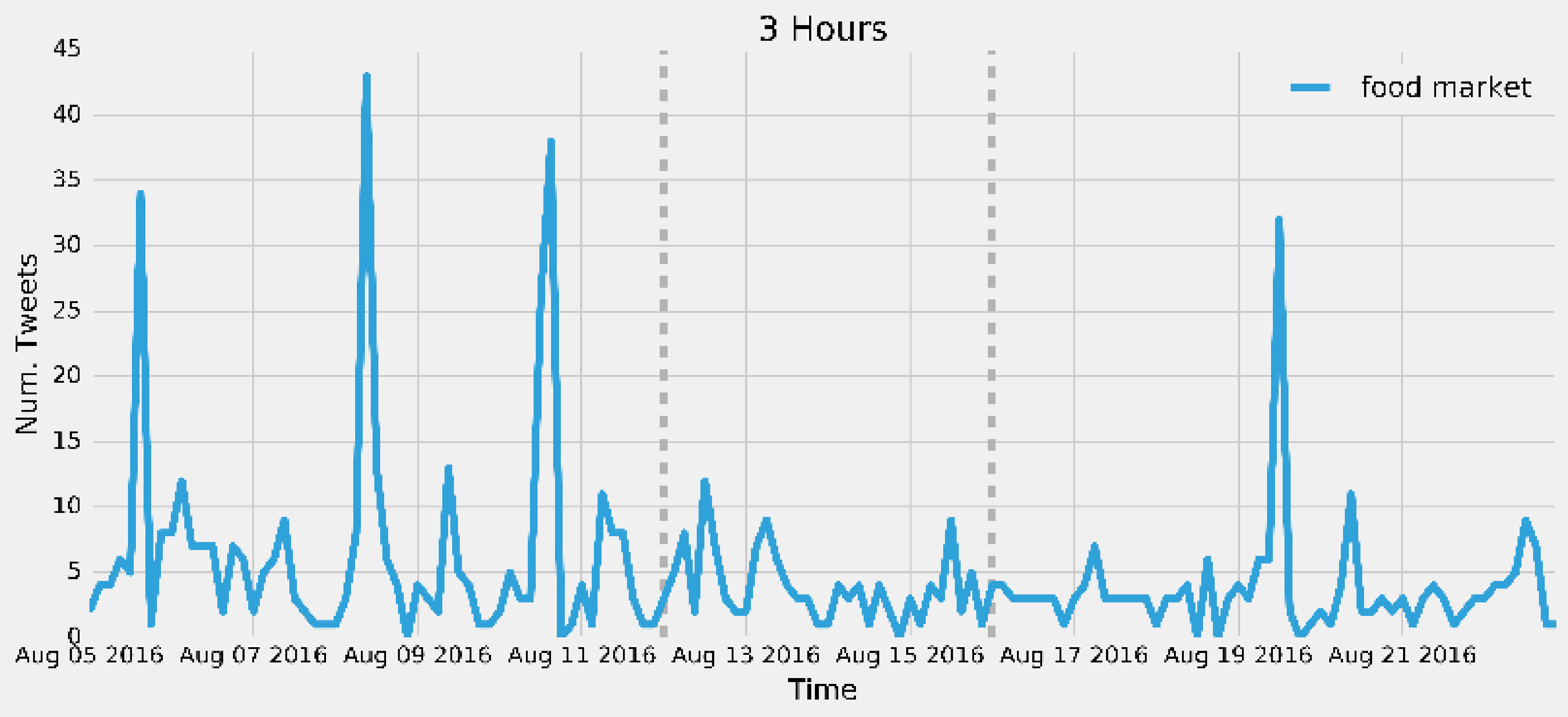
3 Hours
food market
Num. Tweets
0
5
10
15
20
25
30
35
40
45
Aug 05 2016
Aug 07 2016
Aug 09 2016
Aug 11 2016
Aug 13 2016
Aug 15 2016
Aug 17 2016
Aug 19 2016
Aug 21 2016
Time

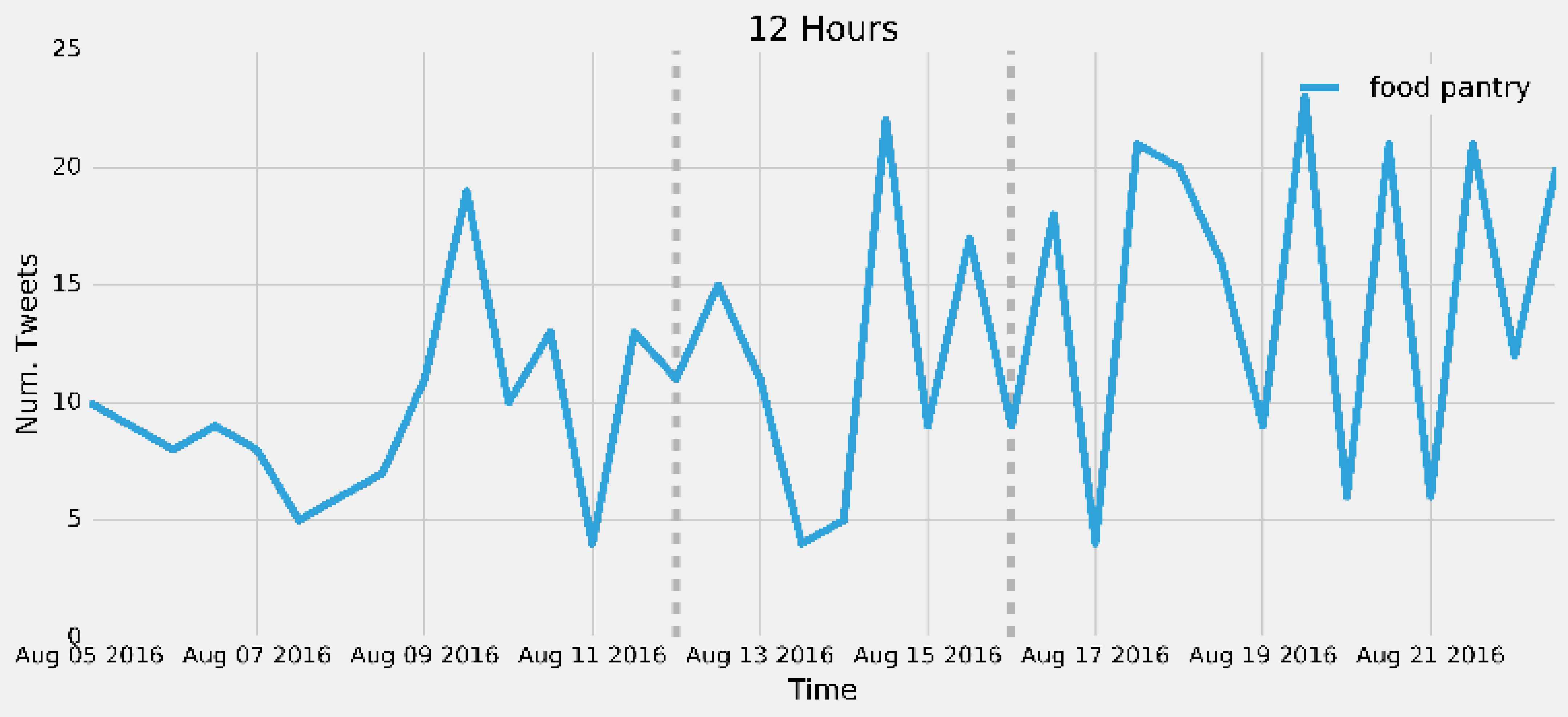
12 Hours
food pantry
Num. Tweets
0
5
10
15
20
25
Aug 05 2016
Aug 07 2016
Aug 09 2016
Aug 11 2016
Aug 13 2016
Aug 15 2016
Aug 17 2016
Aug 19 2016
Aug 21 2016
Time

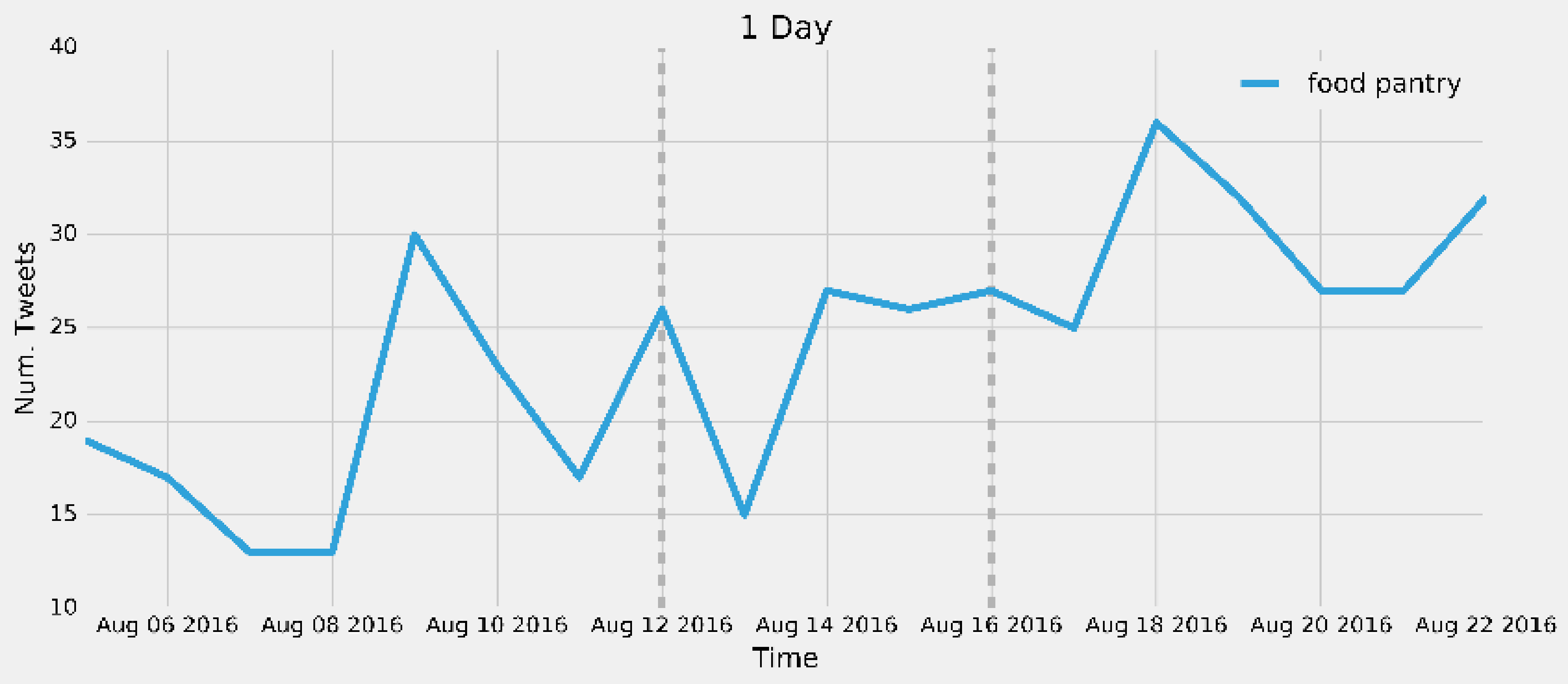
1 Day
food pantry
Num. Tweets
Time
10
15
20
25
30
35
40
Aug 06 2016
Aug 08 2016
Aug 10 2016
Aug 12 2016
Aug 14 2016
Aug 16 2016
Aug 18 2016
Aug 20 2016
Aug 22 2016

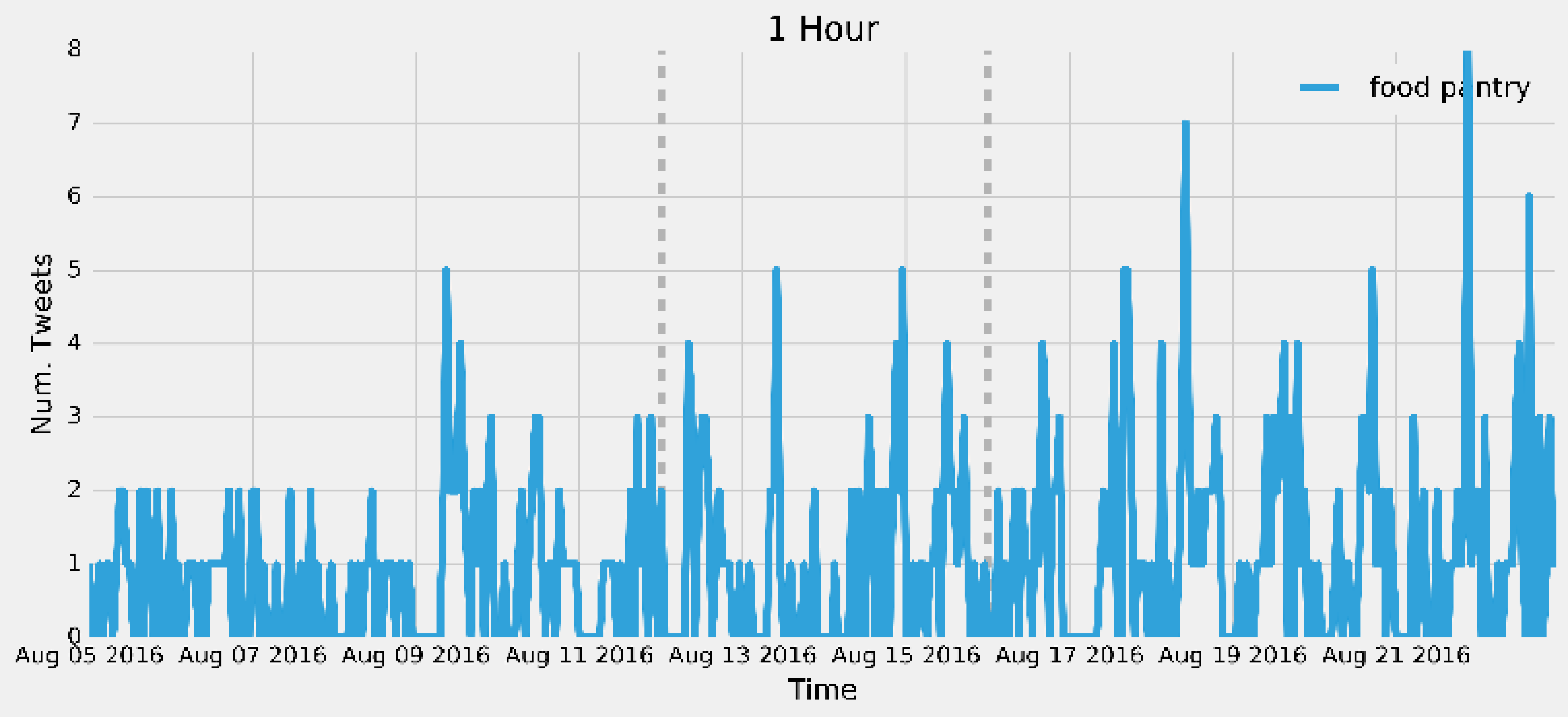
1 Hour
food pantry
Num. Tweets
Time
0
1
2
3
4
5
6
7
8
Aug 05 2016
Aug 07 2016
Aug 09 2016
Aug 11 2016
Aug 13 2016
Aug 15 2016
Aug 17 2016
Aug 19 2016
Aug 21 2016

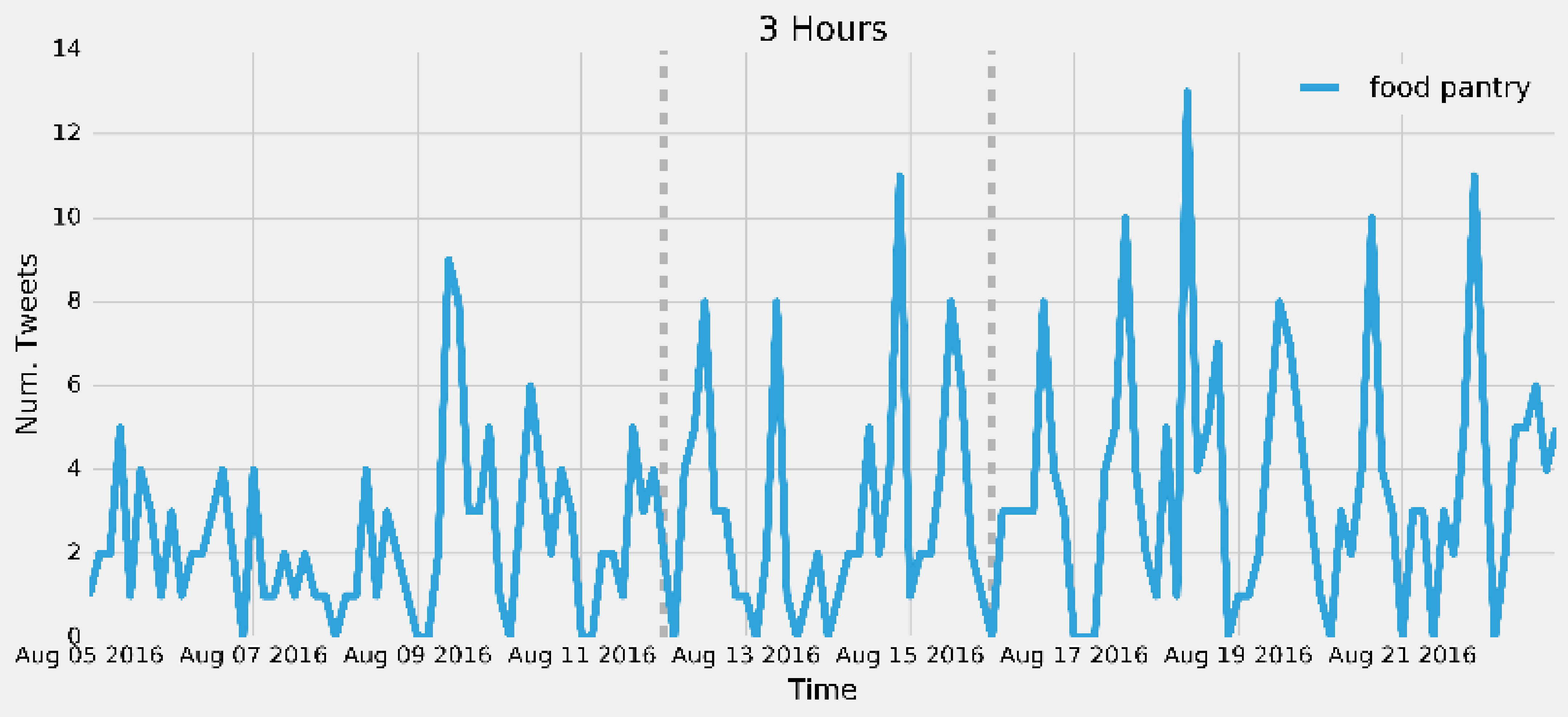
3 Hours
food pantry
Num. Tweets
Time
0
2
4
6
8
10
12
14
Aug 05 2016
Aug 07 2016
Aug 09 2016
Aug 11 2016
Aug 13 2016
Aug 15 2016
Aug 17 2016
Aug 19 2016
Aug 21 2016

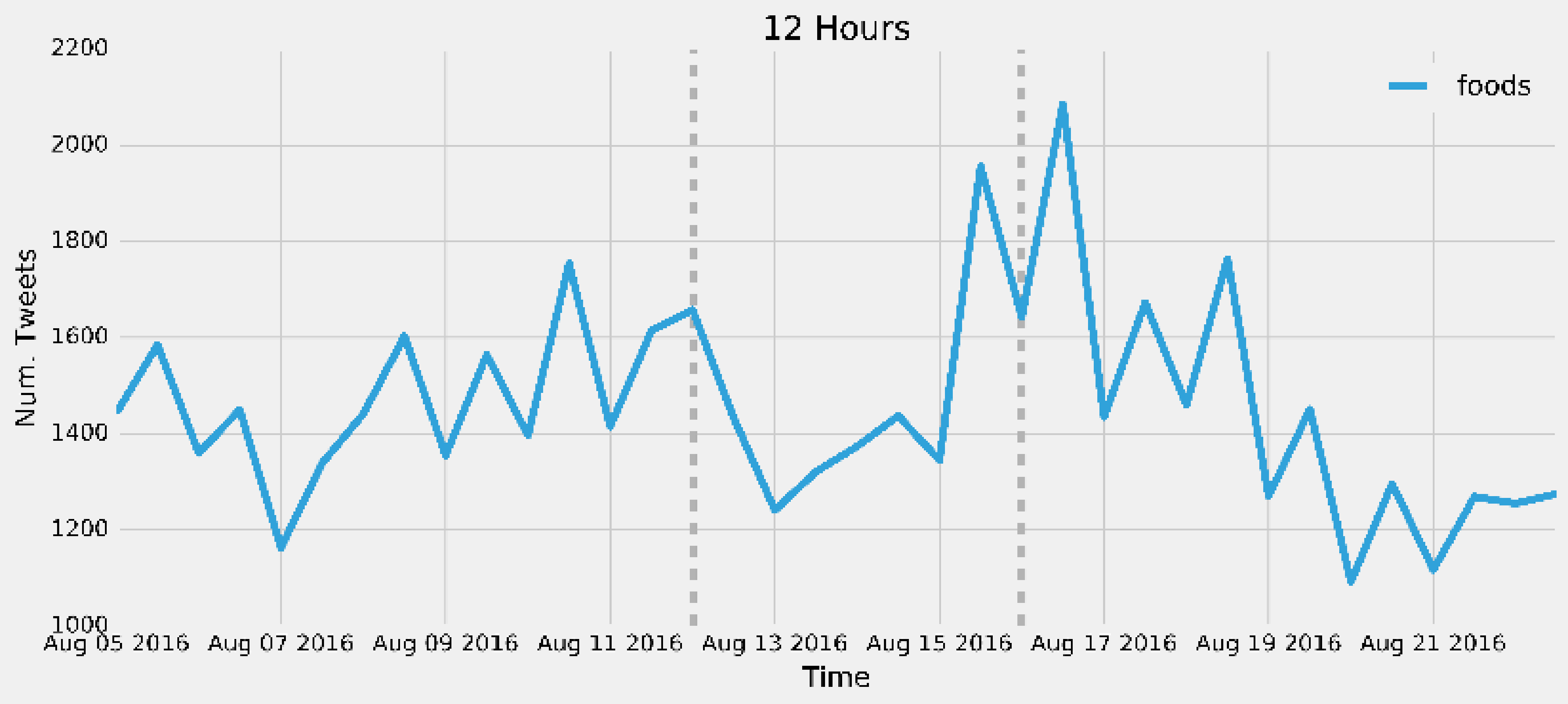

12 Hours
foods
Num. Tweets
Time
2200
2000
1800
1600
1400
1200
1000
Aug 05 2016
Aug 07 2016
Aug 09 2016
Aug 11 2016
Aug 13 2016
Aug 15 2016
Aug 17 2016
Aug 19 2016
Aug 21 2016

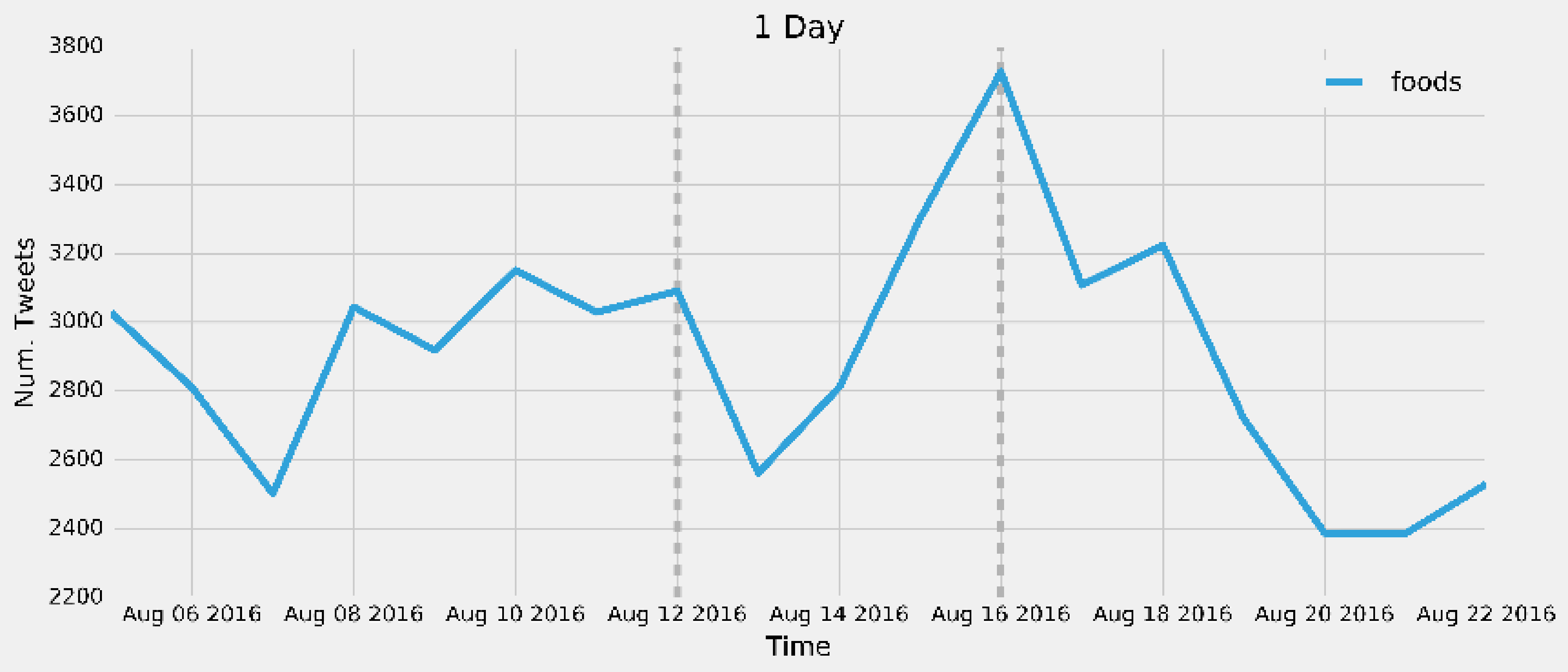
1 Day
foods
Num. Tweets
3800
3600
3400
3200
3000
2800
2600
2400
2200
Aug 06 2016
Aug 08 2016
Aug 10 2016
Aug 12 2016
Aug 14 2016
Aug 16 2016
Aug 18 2016
Aug 20 2016
Aug 22 2016
Time

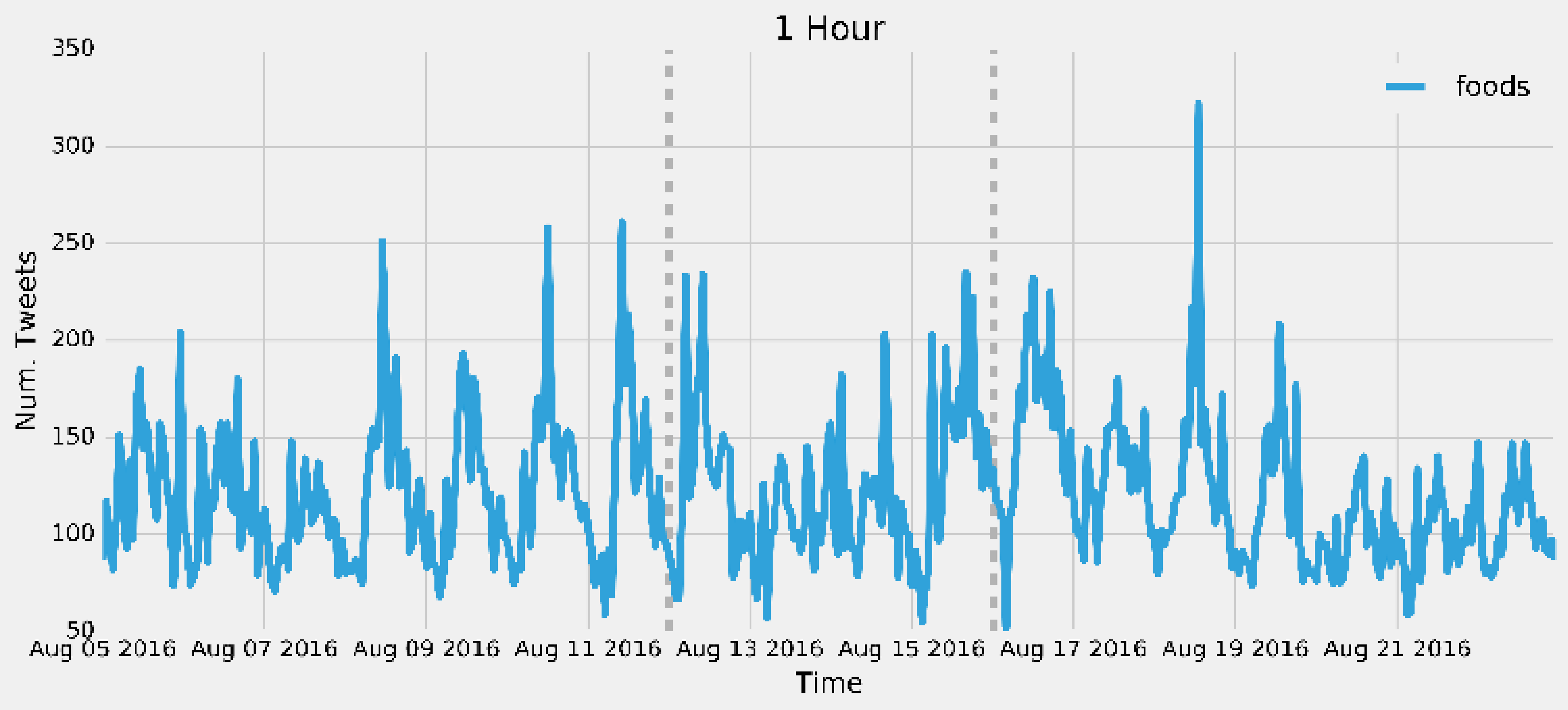
1 Hour
foods
Num. Tweets
350
300
250
200
150
100
50
Aug 05 2016
Aug 07 2016
Aug 09 2016
Aug 11 2016
Aug 13 2016
Aug 15 2016
Aug 17 2016
Aug 19 2016
Aug 21 2016
Time

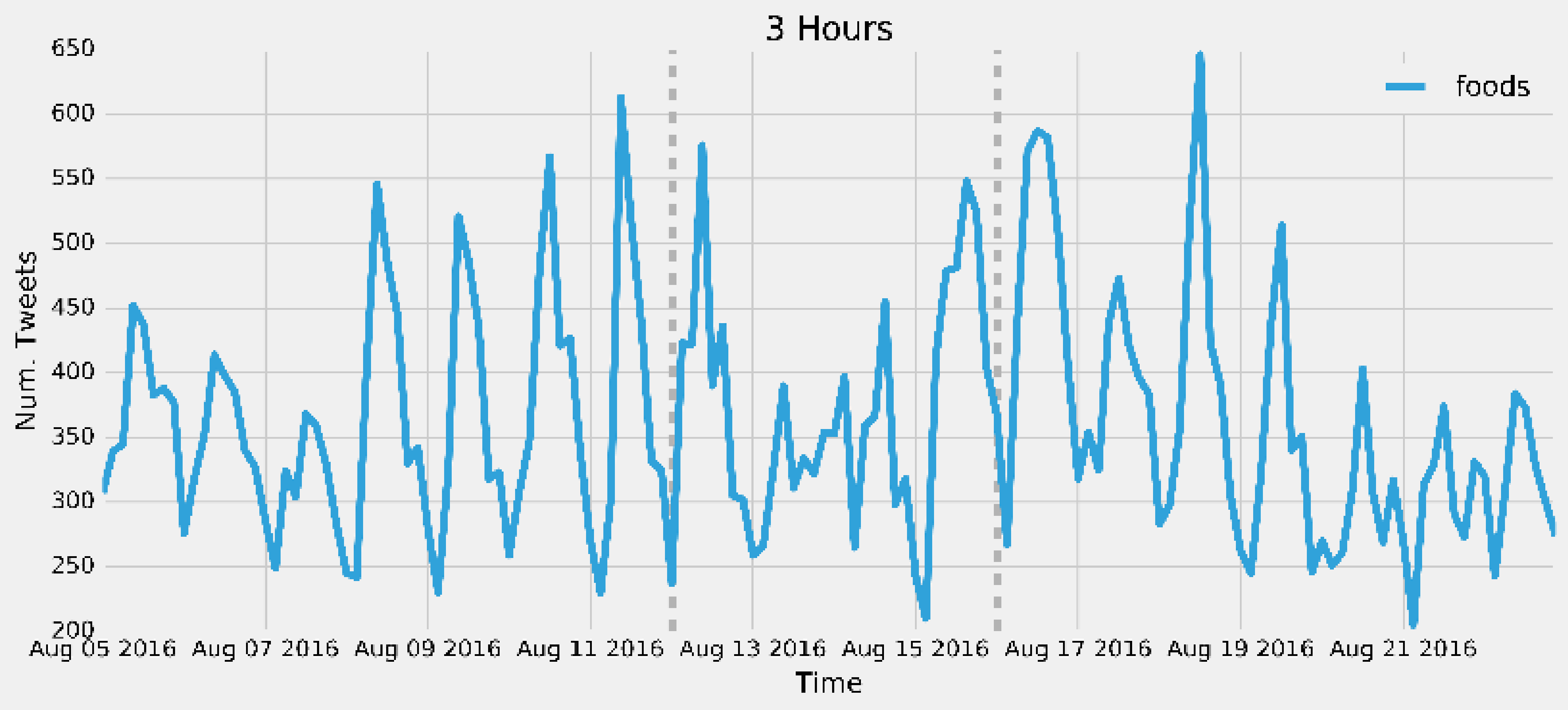

3 Hours
foods
Num. Tweets
650
600
550
500
450
400
350
300
250
200
Aug 05 2016
Aug 07 2016
Aug 09 2016
Aug 11 2016
Aug 13 2016
Aug 15 2016
Aug 17 2016
Aug 19 2016
Aug 21 2016
Time

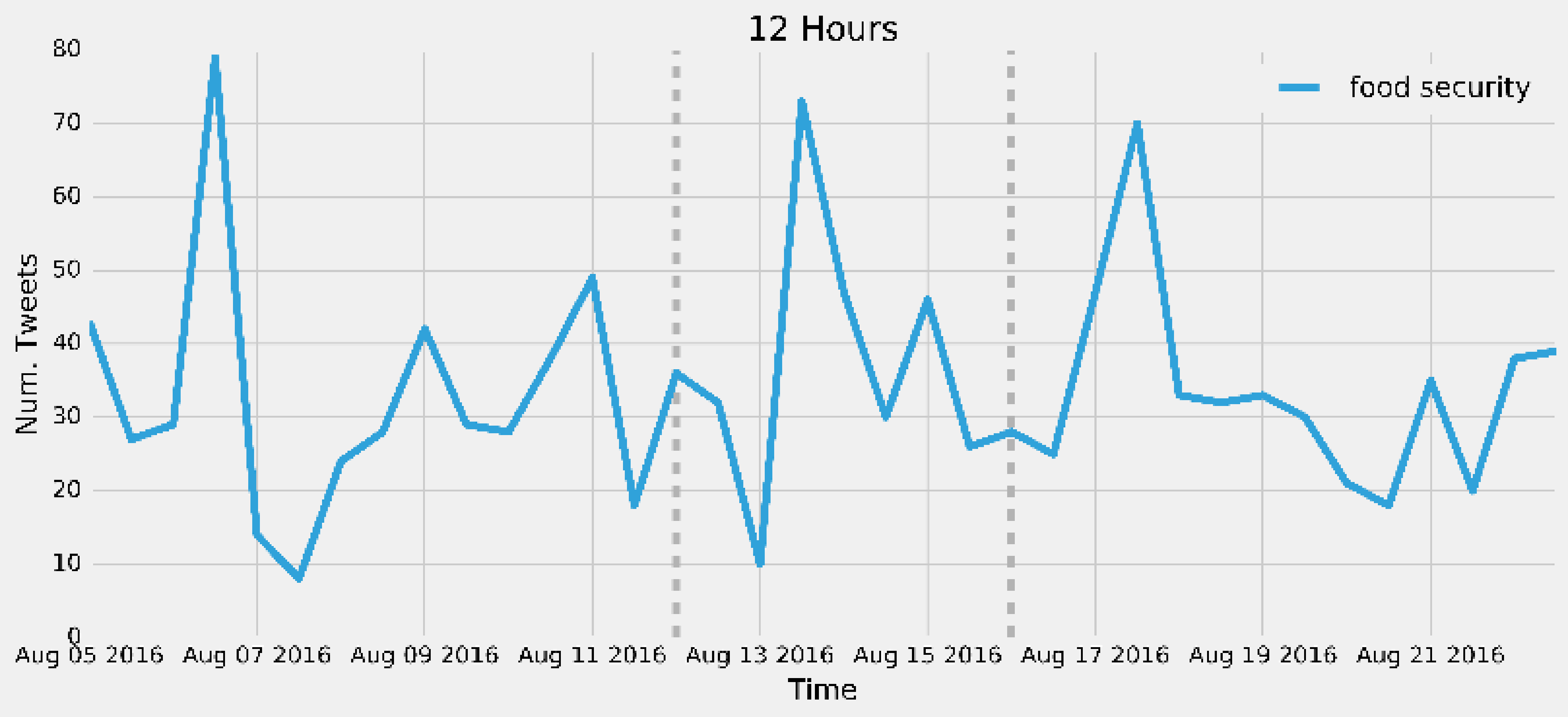
12 Hours
food security
Num. Tweets
0
10
20
30
40
50
60
70
80
Aug 05 2016
Aug 07 2016
Aug 09 2016
Aug 11 2016
Aug 13 2016
Aug 15 2016
Aug 17 2016
Aug 19 2016
Aug 21 2016
Time

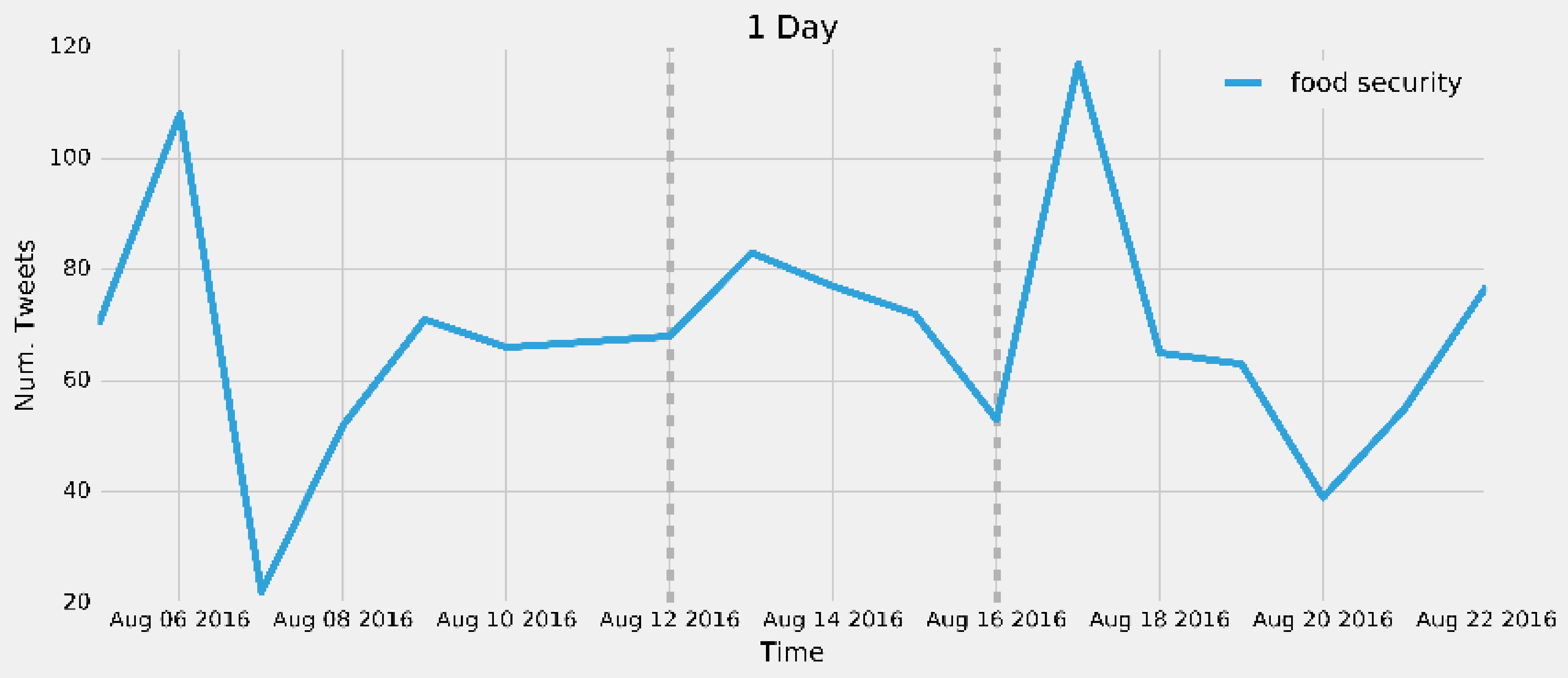
1 Day
food security
Num. Tweets
120
100
80
60
40
20
Aug 06 2016
Aug 08 2016
Aug 10 2016
Aug 12 2016
Aug 14 2016
Aug 16 2016
Aug 18 2016
Aug 20 2016
Aug 22 2016
Time

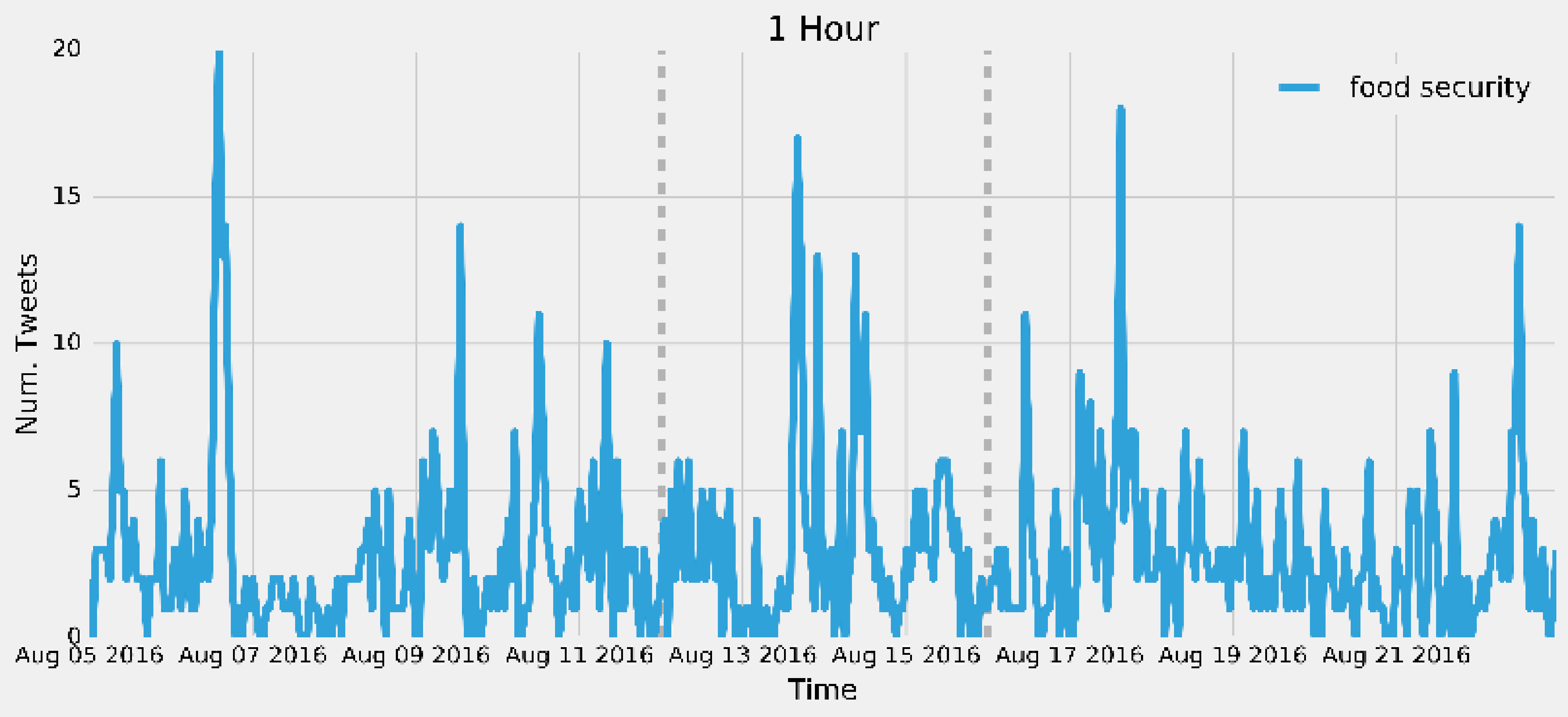
1 Hour
food security
Num. Tweets
20
15
10
5
0
Aug 05 2016
Aug 07 2016
Aug 09 2016
Aug 11 2016
Aug 13 2016
Aug 15 2016
Aug 17 2016
Aug 19 2016
Aug 21 2016
Time

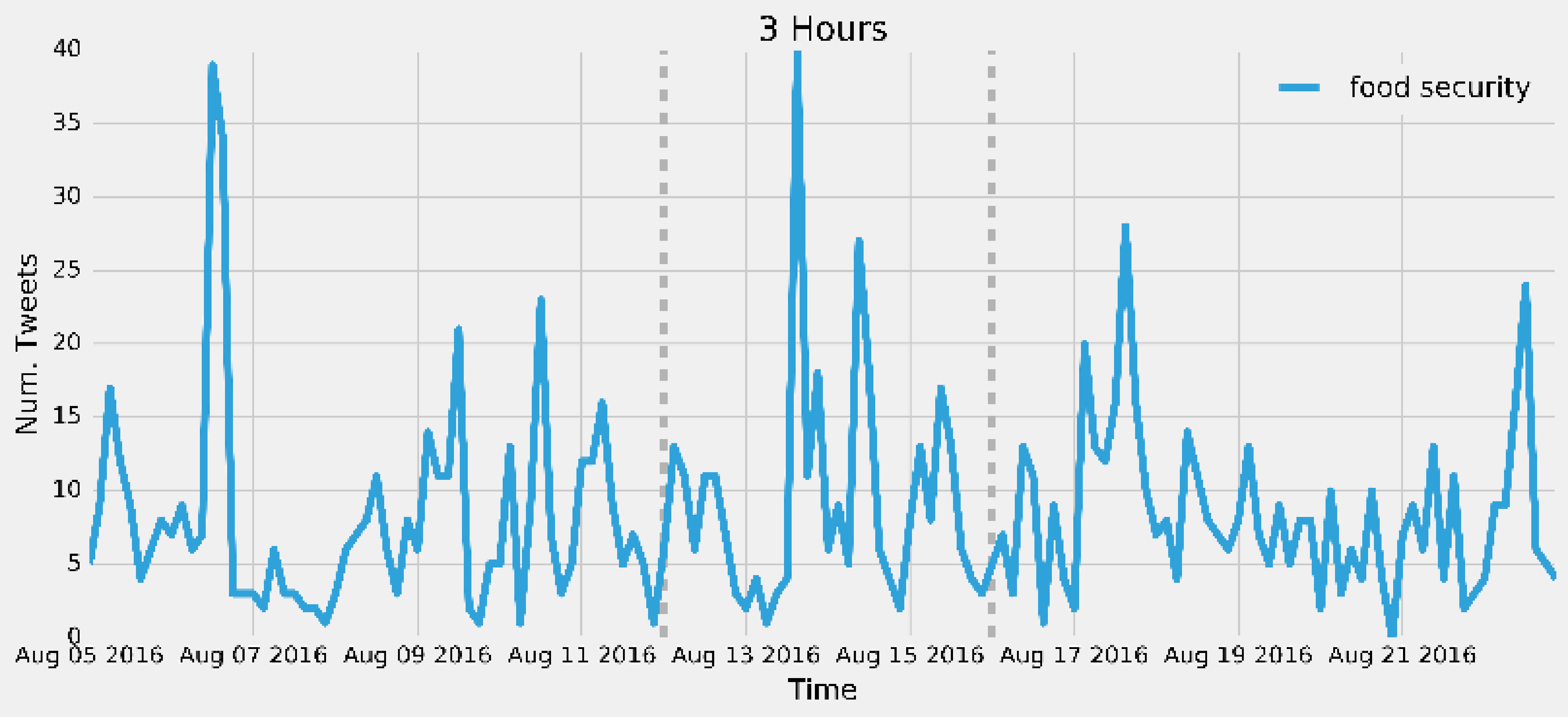

3 Hours
food security
Num. Tweets
Time
0
5
10
15
20
25
30
35
40
Aug 05 2016
Aug 07 2016
Aug 09 2016
Aug 11 2016
Aug 13 2016
Aug 15 2016
Aug 17 2016
Aug 19 2016
Aug 21 2016

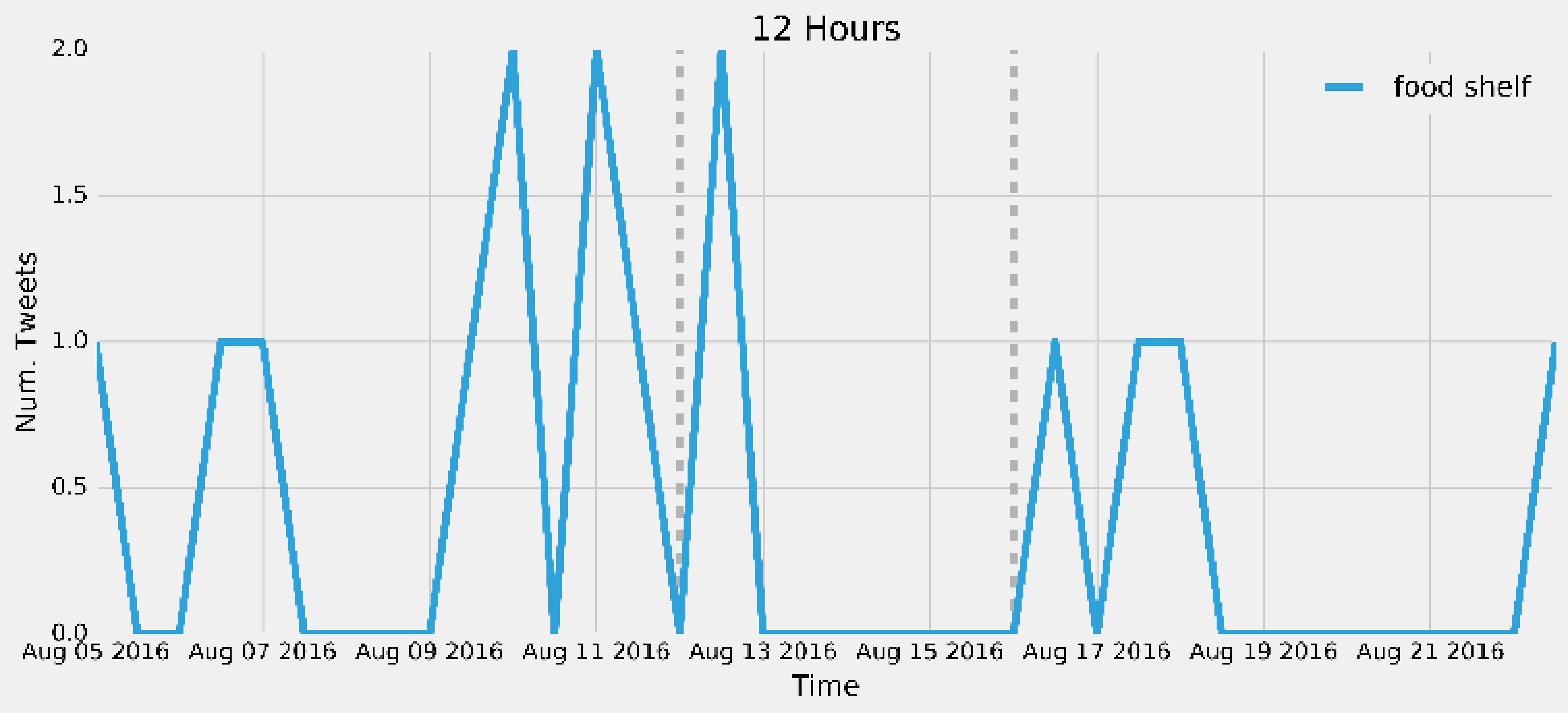
12 Hours
food shelf
Num. Tweets
2.0
1.5
1.0
0.5
0.0
Aug 05 2016
Aug 07 2016
Aug 09 2016
Aug 11 2016
Aug 13 2016
Aug 15 2016
Aug 17 2016
Aug 19 2016
Aug 21 2016
Time

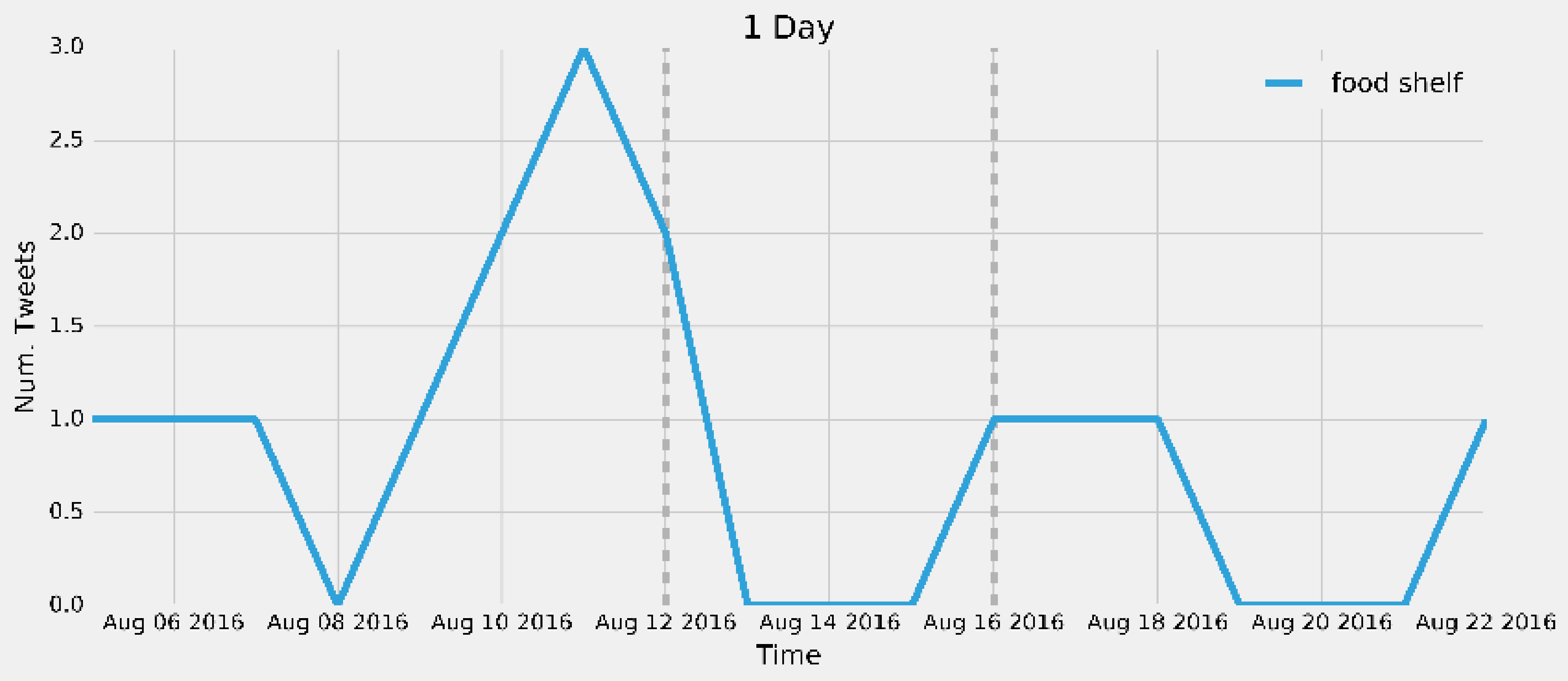

1 Day
food shelf
Num. Tweets
Time
3.0
2.5
2.0
1.5
1.0
0.5
0.0
Aug 06 2016
Aug 08 2016
Aug 10 2016
Aug 12 2016
Aug 14 2016
Aug 16 2016
Aug 18 2016
Aug 20 2016
Aug 22 2016

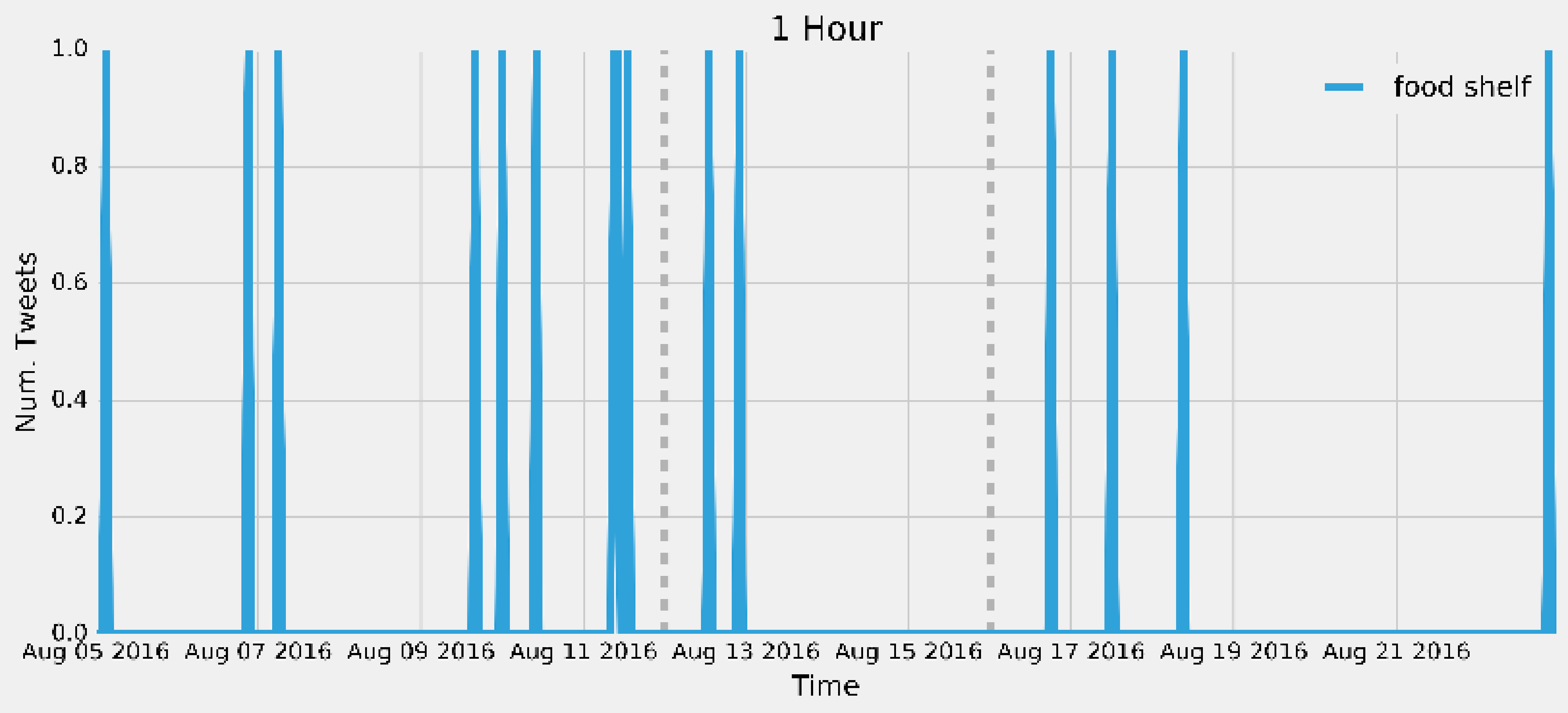
1 Hour
food shelf
Num. Tweets
1.0
0.8
0.6
0.4
0.2
0.0
Aug 05 2016
Aug 07 2016
Aug 09 2016
Aug 11 2016
Aug 13 2016
Aug 15 2016
Aug 17 2016
Aug 19 2016
Aug 21 2016
Time

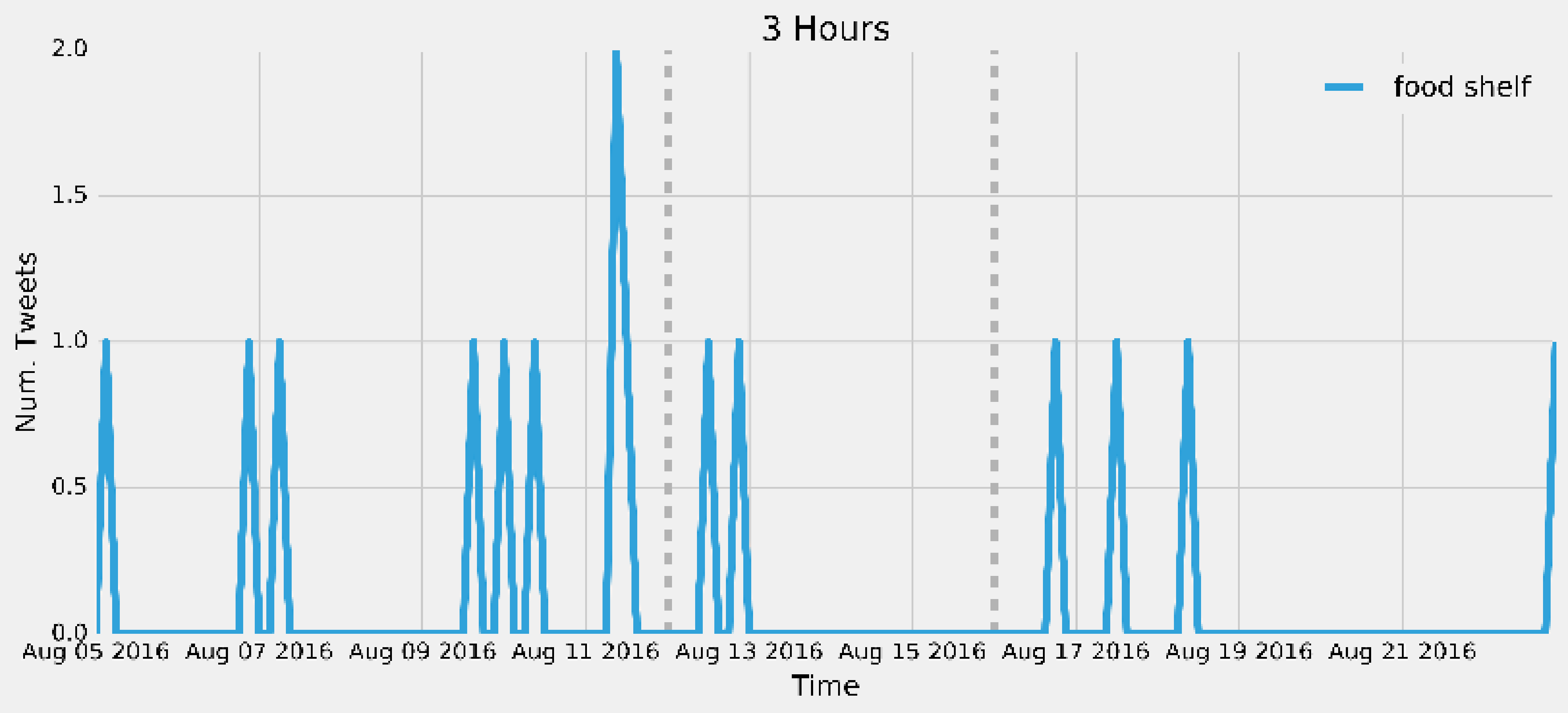
3 Hours
food shelf
Num. Tweets
2.0
1.5
1.0
0.5
0.0
Aug 05 2016
Aug 07 2016
Aug 09 2016
Aug 11 2016
Aug 13 2016
Aug 15 2016
Aug 17 2016
Aug 19 2016
Aug 21 2016
Time

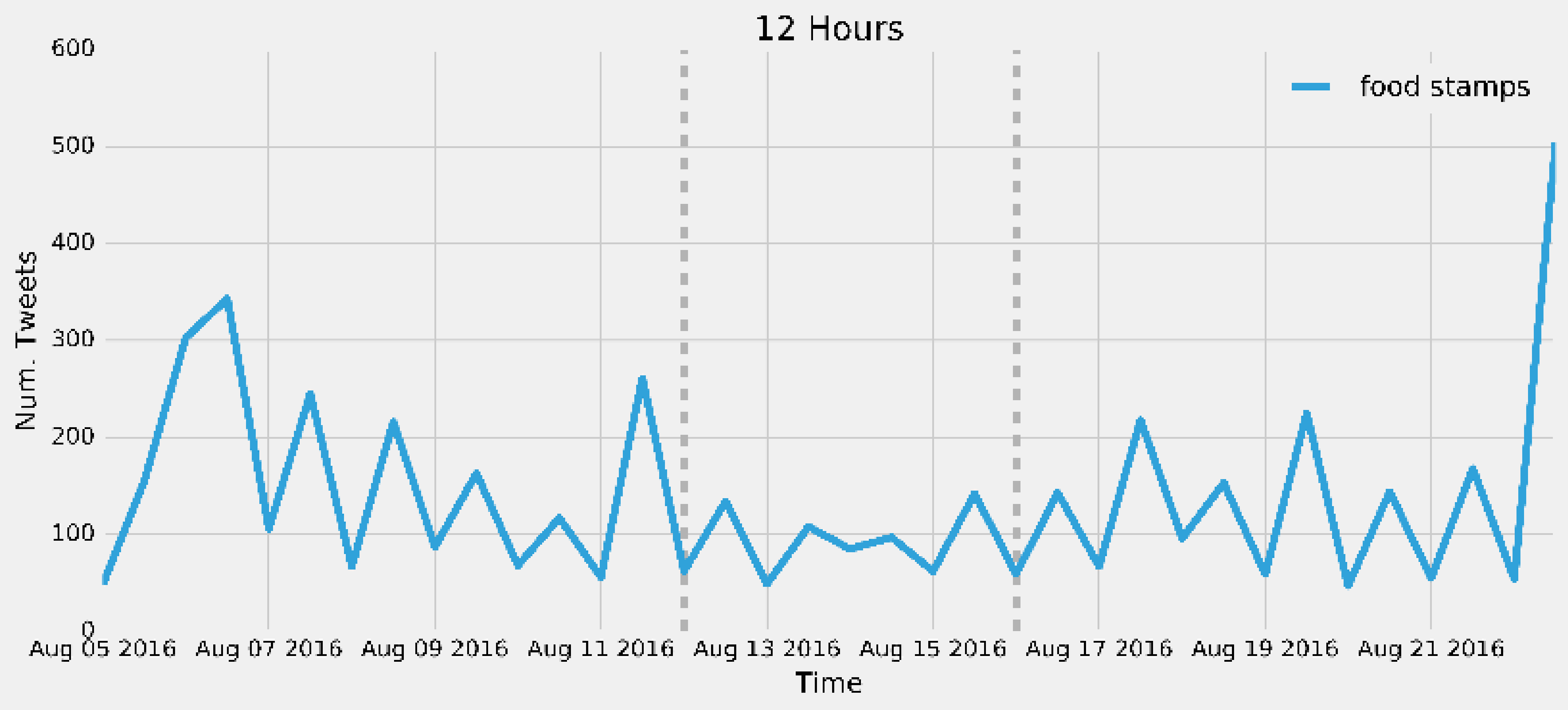

12 Hours
food stamps
Num. Tweets
Time
0
100
200
300
400
500
600
Aug 05 2016
Aug 07 2016
Aug 09 2016
Aug 11 2016
Aug 13 2016
Aug 15 2016
Aug 17 2016
Aug 19 2016
Aug 21 2016

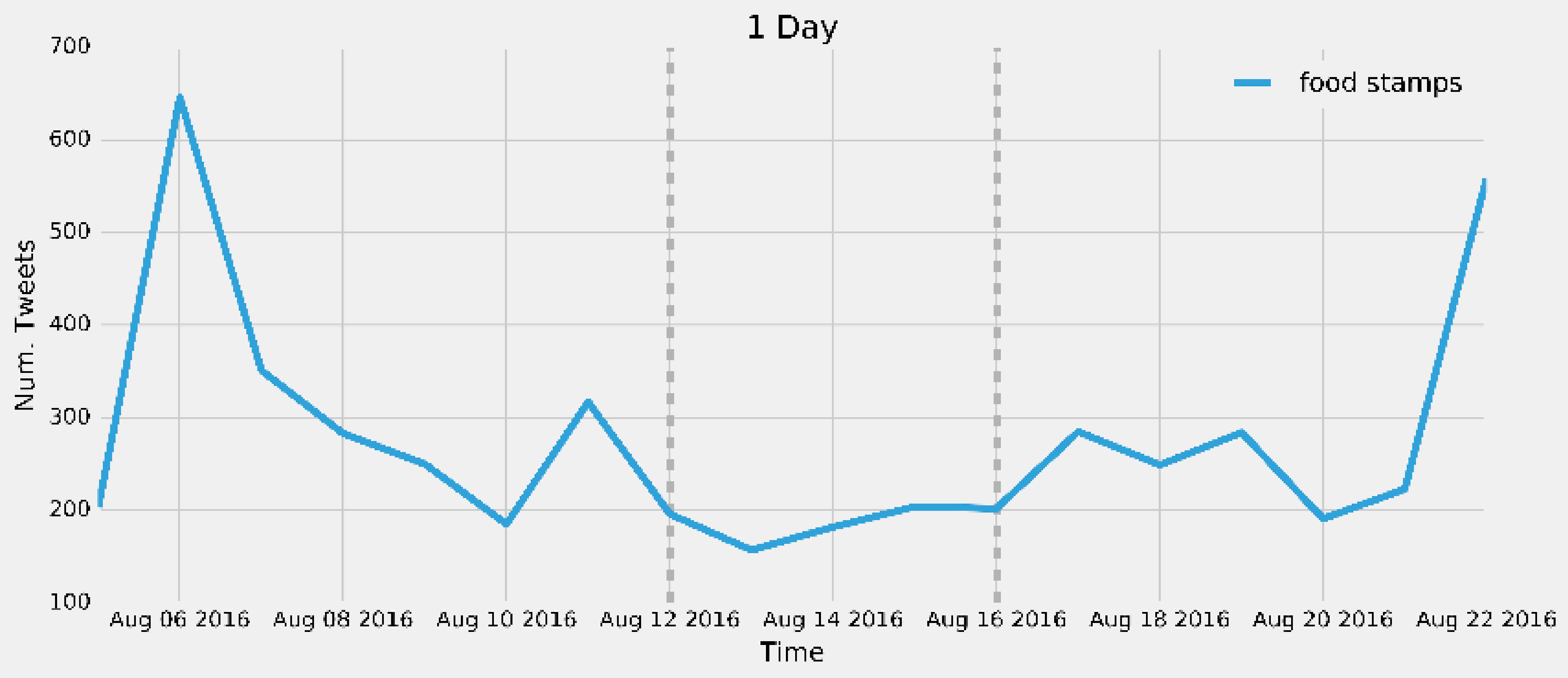
1 Day
food stamps
Num. Tweets
700
600
500
400
300
200
100
Aug 06 2016
Aug 08 2016
Aug 10 2016
Aug 12 2016
Aug 14 2016
Aug 16 2016
Aug 18 2016
Aug 20 2016
Aug 22 2016
Time

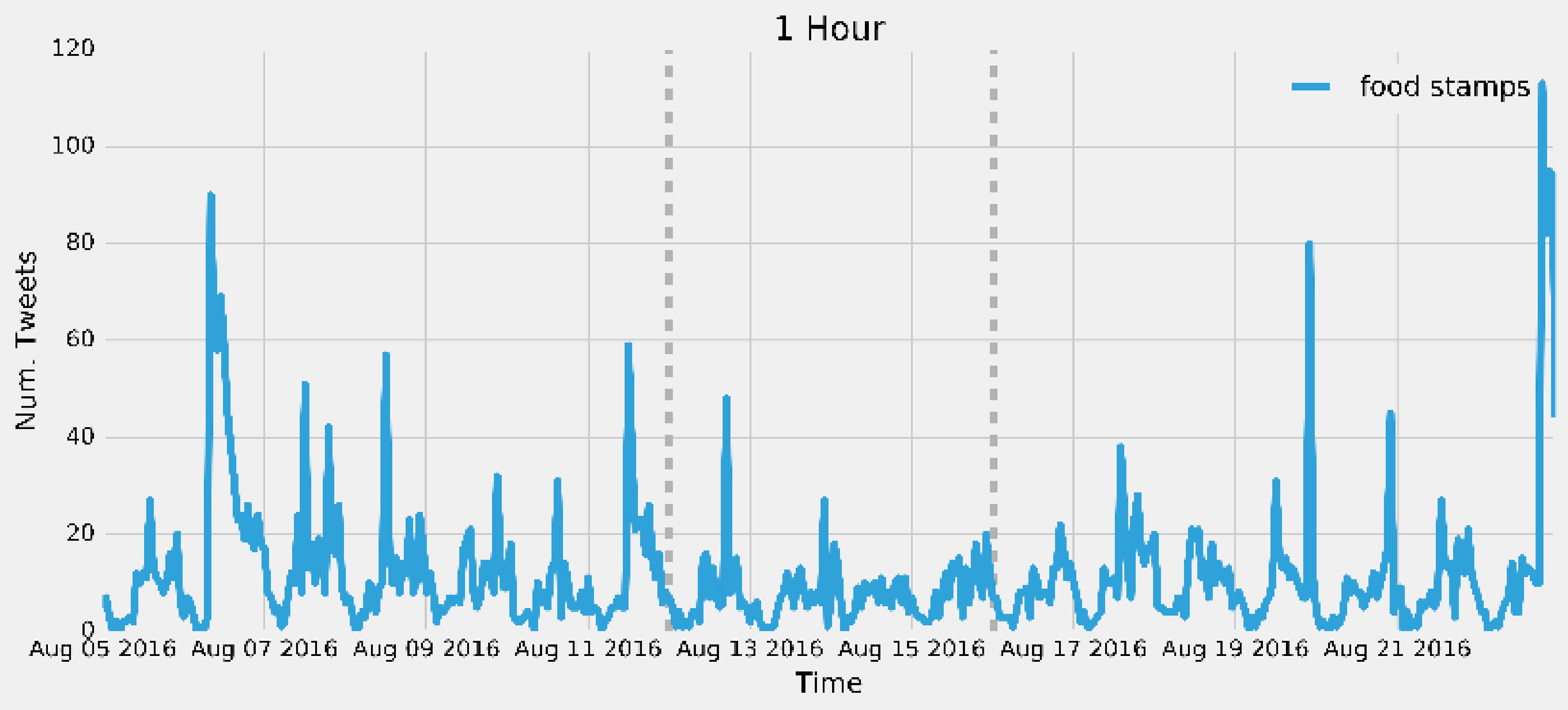

1 Hour
food stamps
Num. Tweets
0
20
40
60
80
100
120
Aug 05 2016
Aug 07 2016
Aug 09 2016
Aug 11 2016
Aug 13 2016
Aug 15 2016
Aug 17 2016
Aug 19 2016
Aug 21 2016
Time

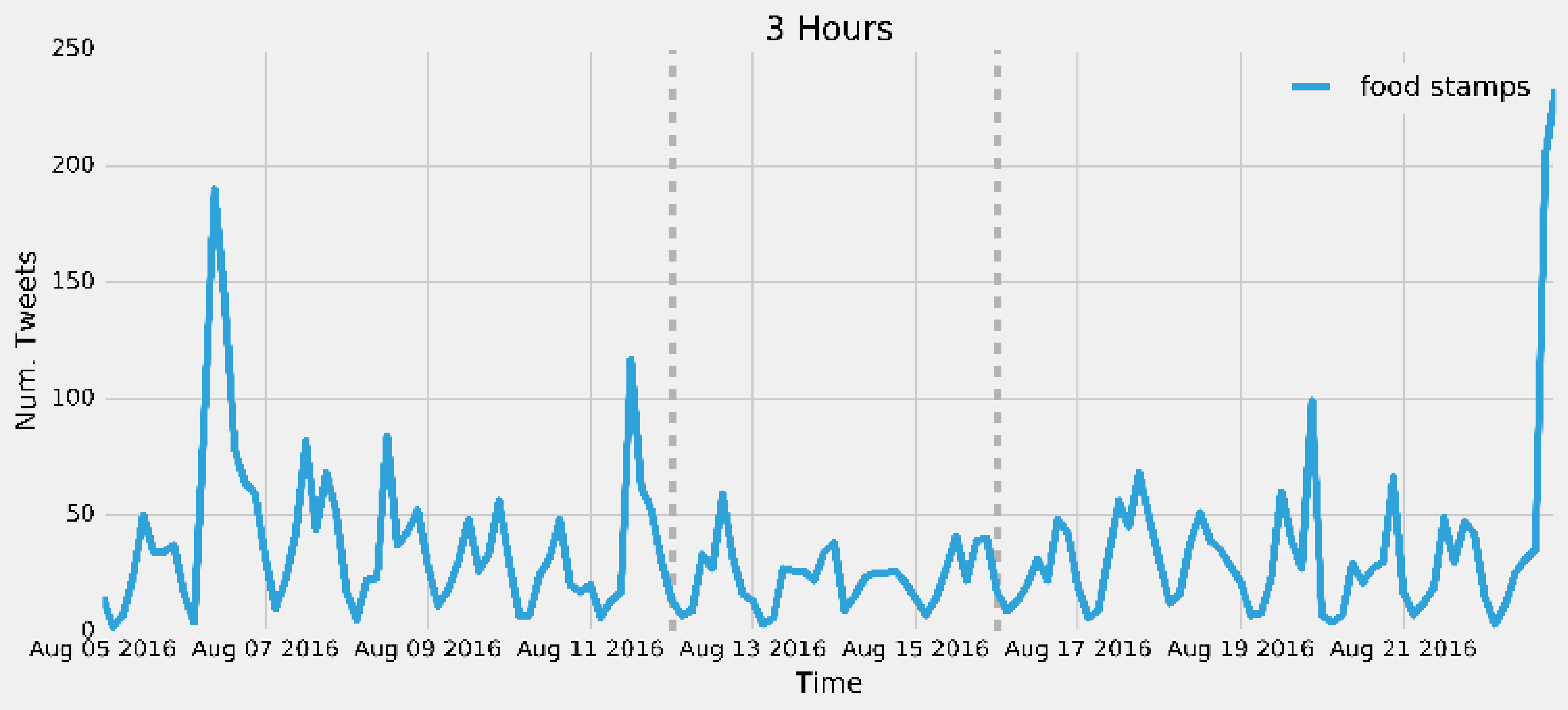

3 Hours
food stamps
Num. Tweets
0
50
100
150
200
250
Aug 05 2016
Aug 07 2016
Aug 09 2016
Aug 11 2016
Aug 13 2016
Aug 15 2016
Aug 17 2016
Aug 19 2016
Aug 21 2016
Time

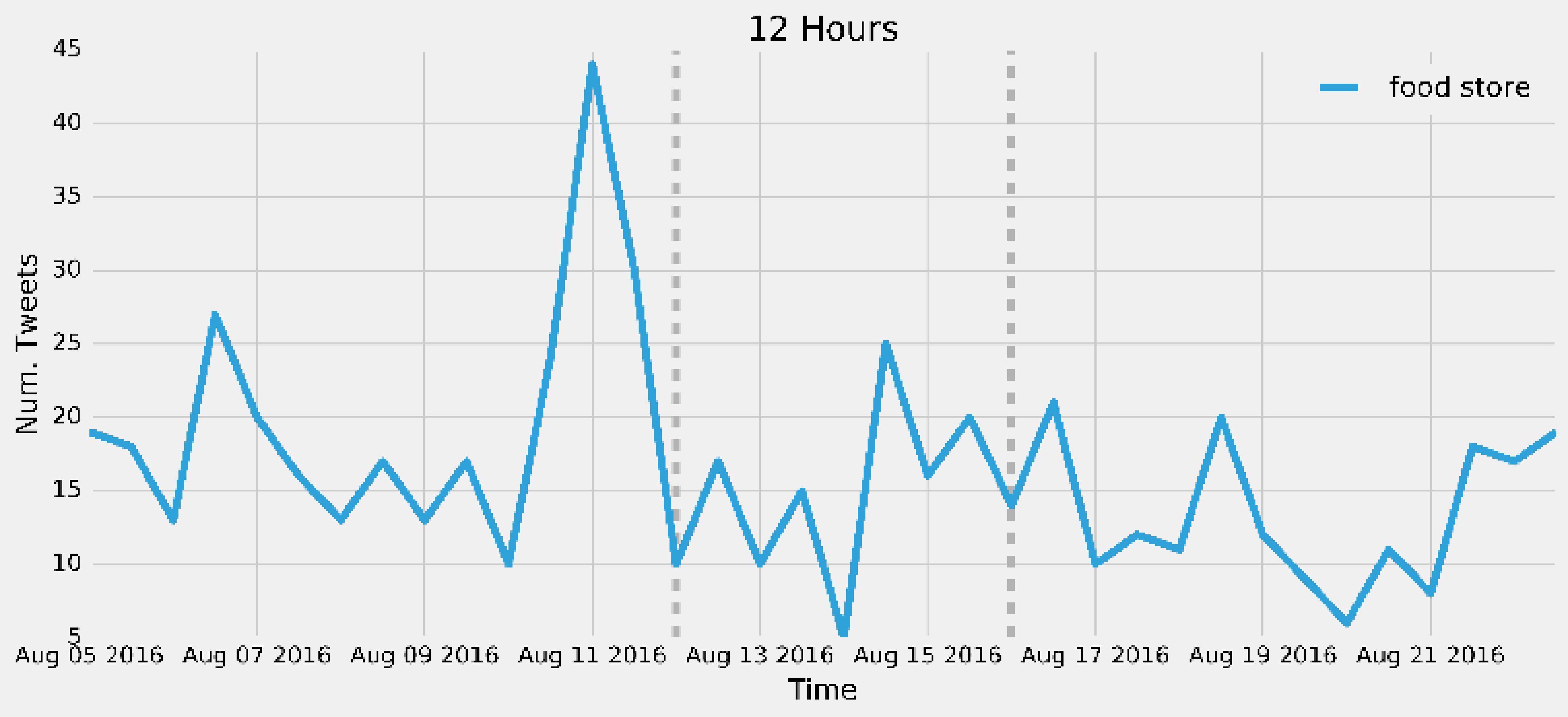

12 Hours
food store
Num. Tweets
Time
45
40
35
30
25
20
15
10
5
Aug 05 2016
Aug 07 2016
Aug 09 2016
Aug 11 2016
Aug 13 2016
Aug 15 2016
Aug 17 2016
Aug 19 2016
Aug 21 2016

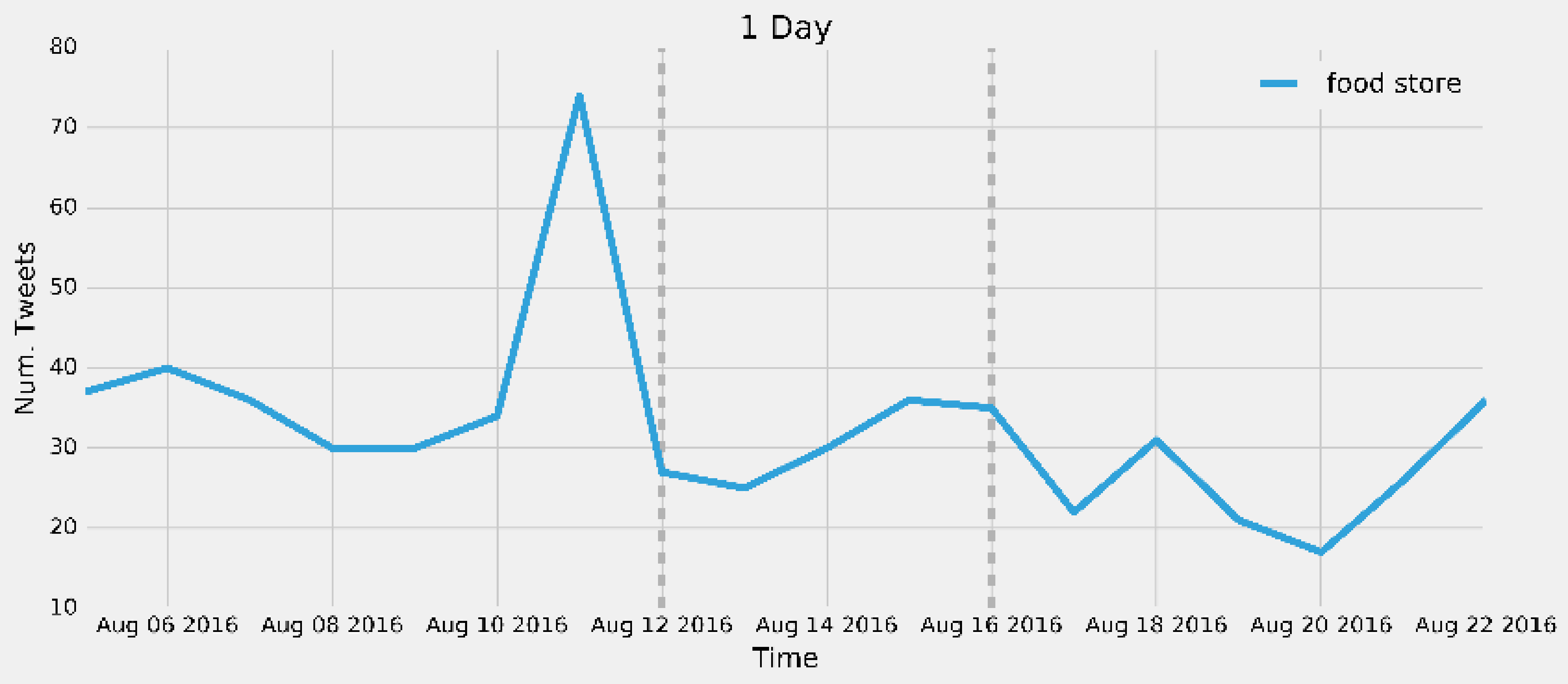

1 Day
food store
Num. Tweets
Time
80
70
60
50
40
30
20
10
Aug 06 2016
Aug 08 2016
Aug 10 2016
Aug 12 2016
Aug 14 2016
Aug 16 2016
Aug 18 2016
Aug 20 2016
Aug 22 2016

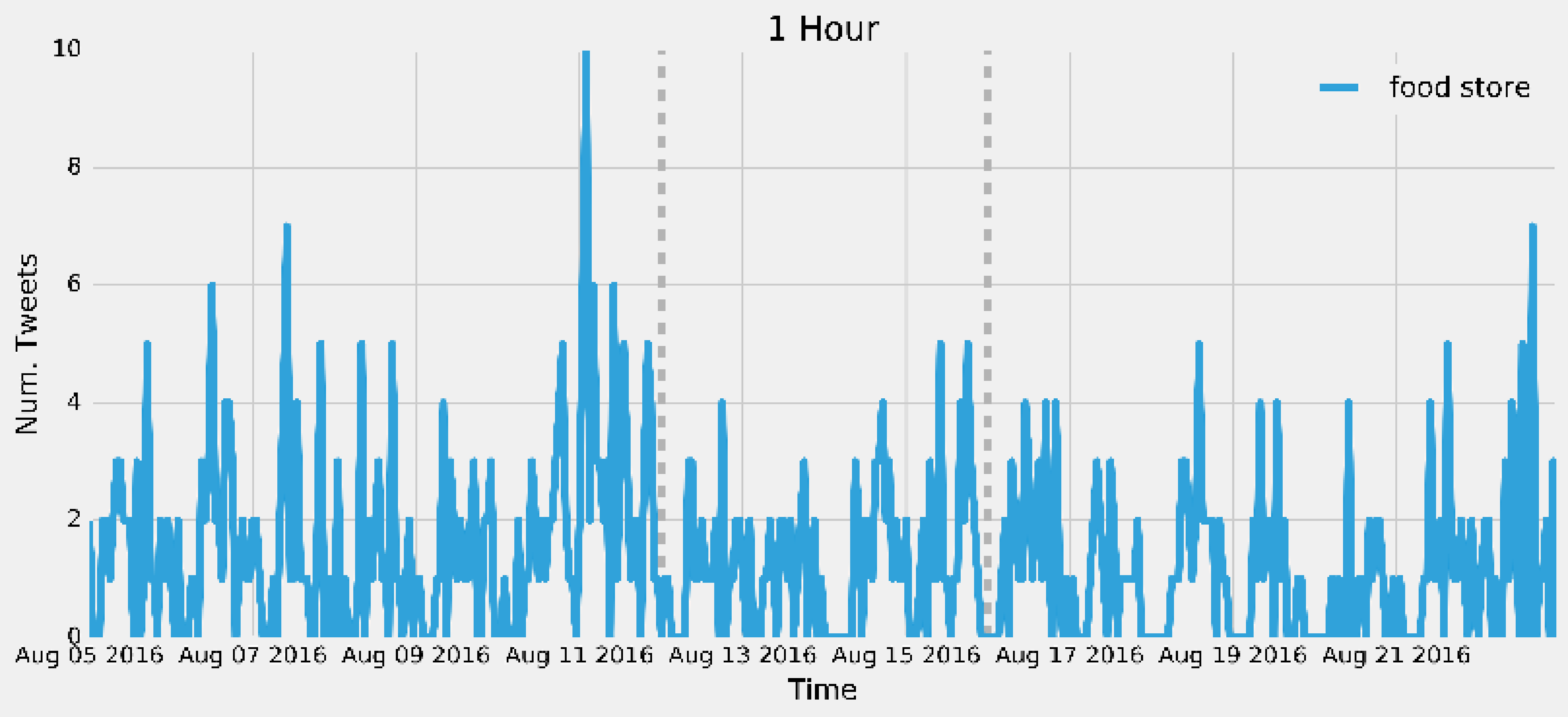
1 Hour
food store
Num. Tweets
0
2
4
6
8
10
Aug 05 2016
Aug 07 2016
Aug 09 2016
Aug 11 2016
Aug 13 2016
Aug 15 2016
Aug 17 2016
Aug 19 2016
Aug 21 2016
Time

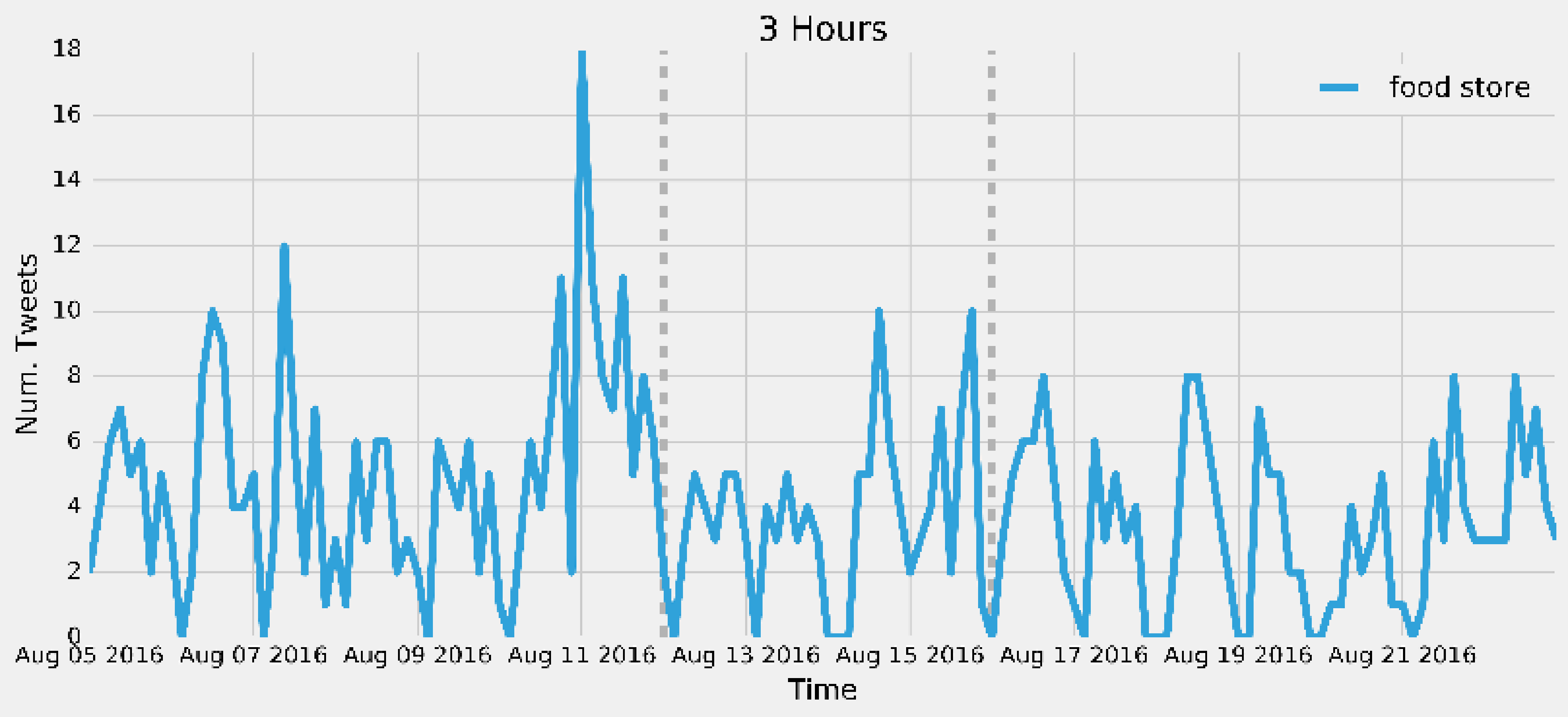
3 Hours
food store
Num. Tweets
0
2
4
6
8
10
12
14
16
18
Aug 05 2016
Aug 07 2016
Aug 09 2016
Aug 11 2016
Aug 13 2016
Aug 15 2016
Aug 17 2016
Aug 19 2016
Aug 21 2016
Time

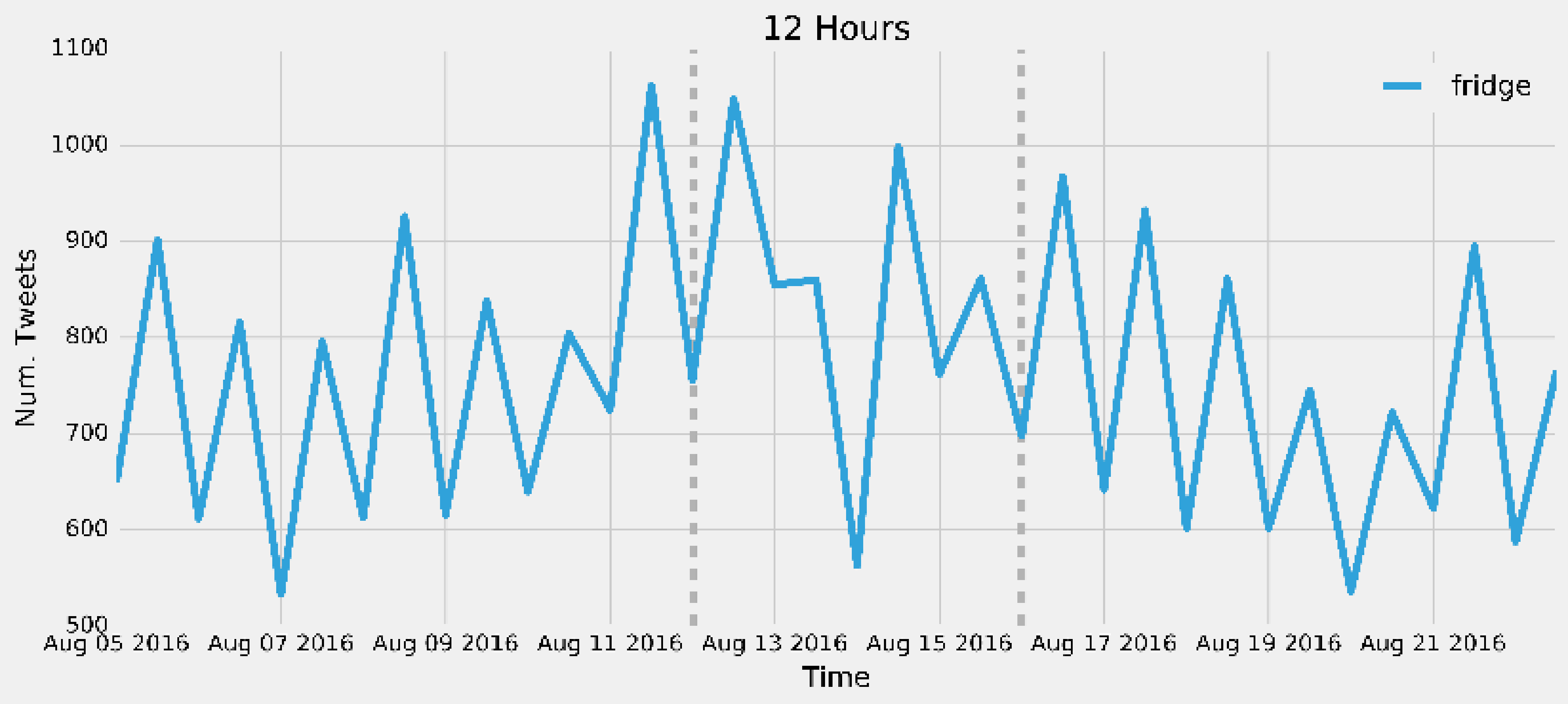

12 Hours
fridge
Num. Tweets
Time
1100
1000
900
800
700
600
500
Aug 05 2016
Aug 07 2016
Aug 09 2016
Aug 11 2016
Aug 13 2016
Aug 15 2016
Aug 17 2016
Aug 19 2016
Aug 21 2016

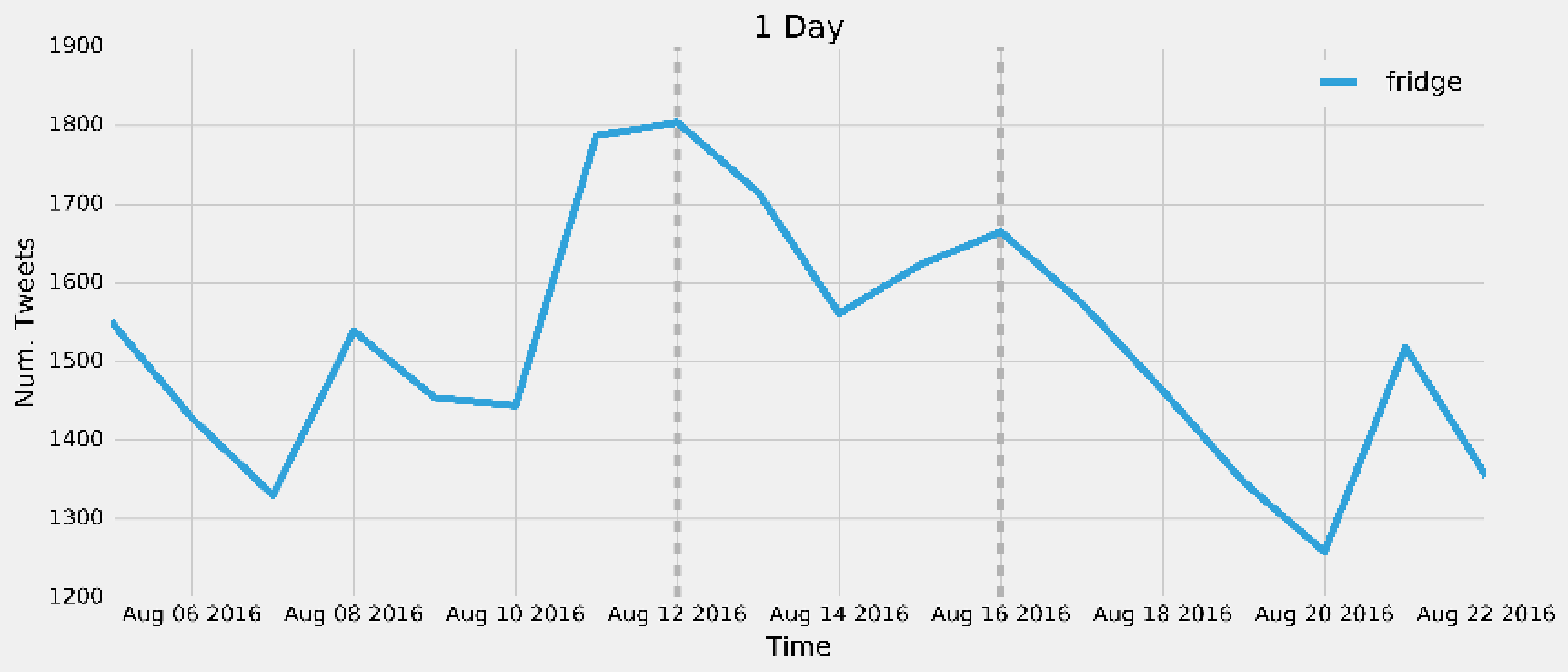

1 Day
fridge
Num. Tweets
1900
1800
1700
1600
1500
1400
1300
1200
Aug 06 2016
Aug 08 2016
Aug 10 2016
Aug 12 2016
Aug 14 2016
Aug 16 2016
Aug 18 2016
Aug 20 2016
Aug 22 2016
Time

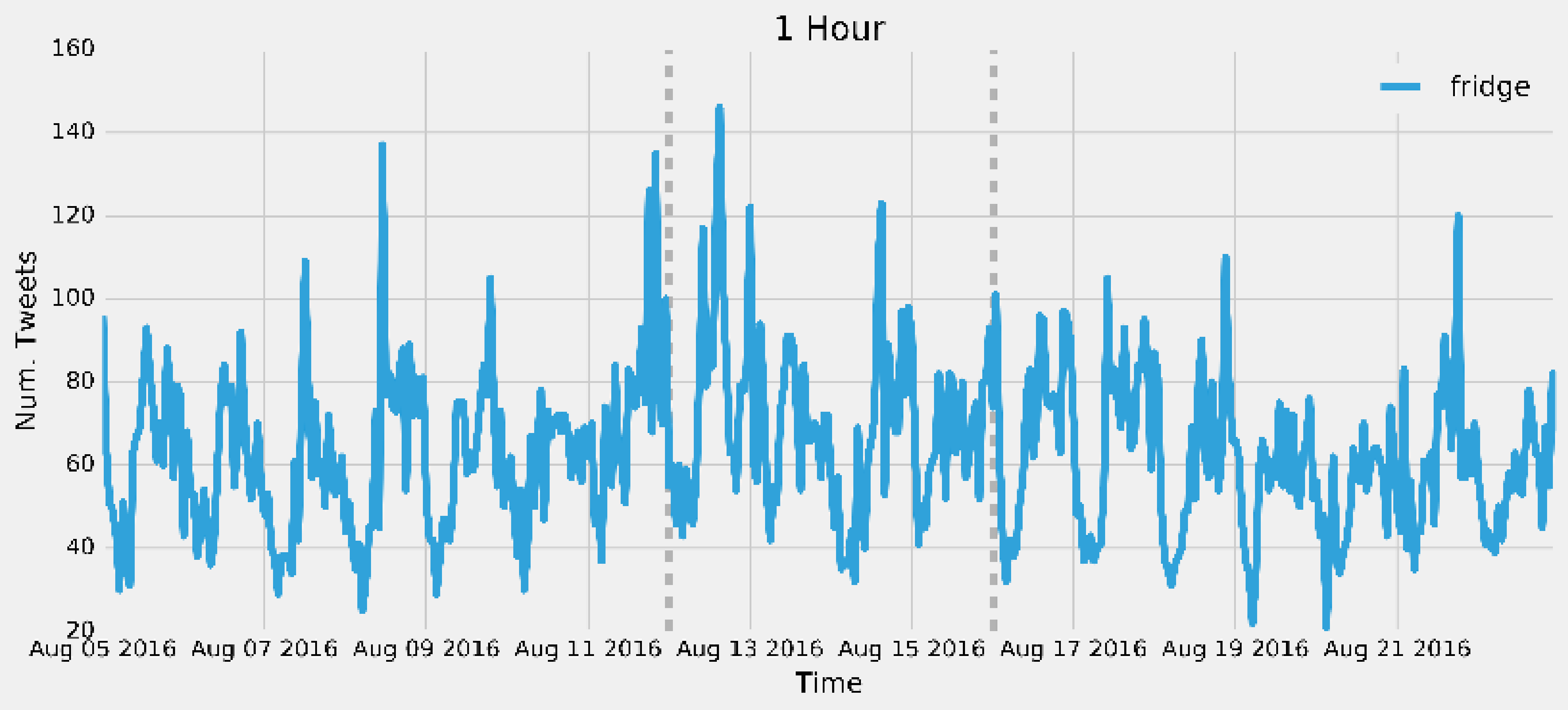

1 Hour
fridge
Num. Tweets
160
140
120
100
80
60
40
20
Aug 05 2016
Aug 07 2016
Aug 09 2016
Aug 11 2016
Aug 13 2016
Aug 15 2016
Aug 17 2016
Aug 19 2016
Aug 21 2016
Time

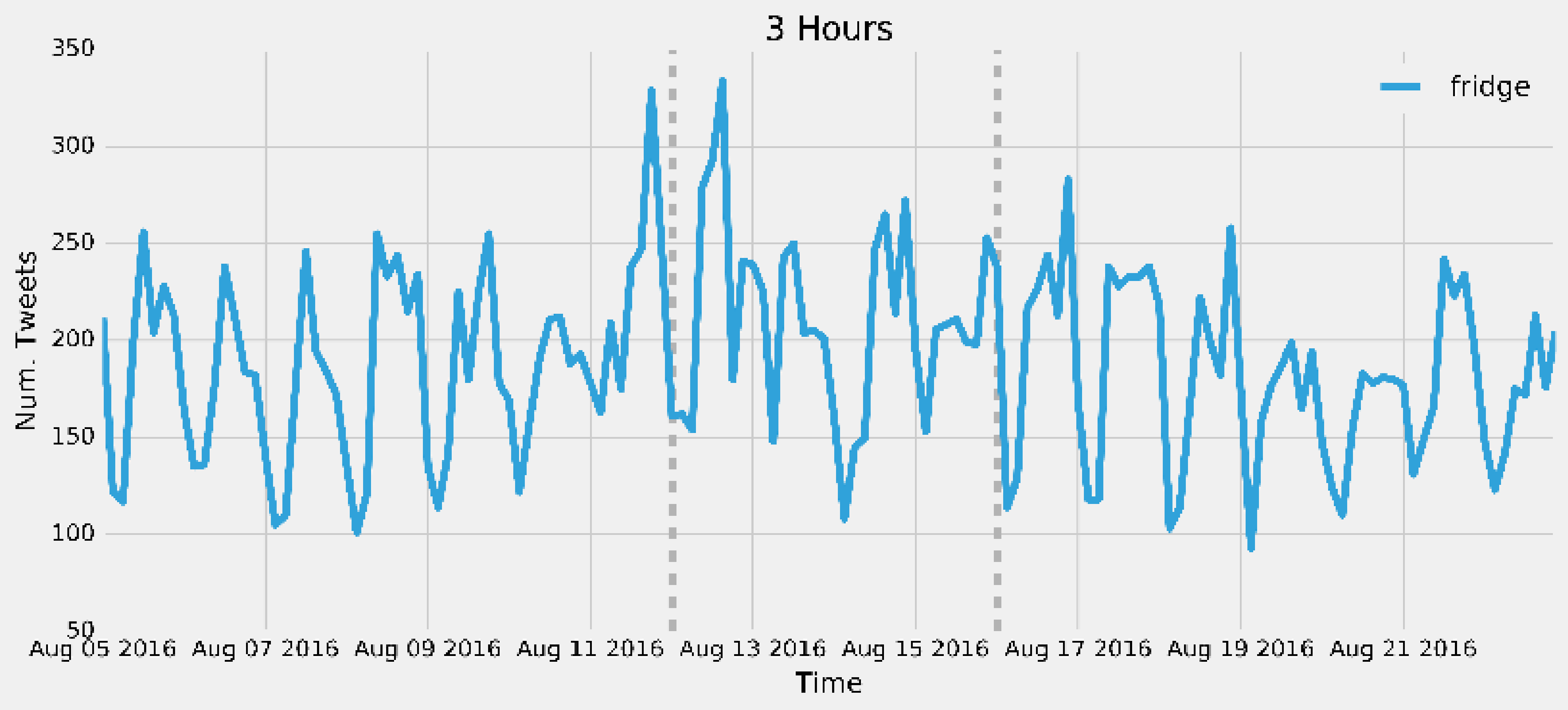
3 Hours
fridge
Num. Tweets
Time
350
300
250
200
150
100
50
Aug 05 2016
Aug 07 2016
Aug 09 2016
Aug 11 2016
Aug 13 2016
Aug 15 2016
Aug 17 2016
Aug 19 2016
Aug 21 2016

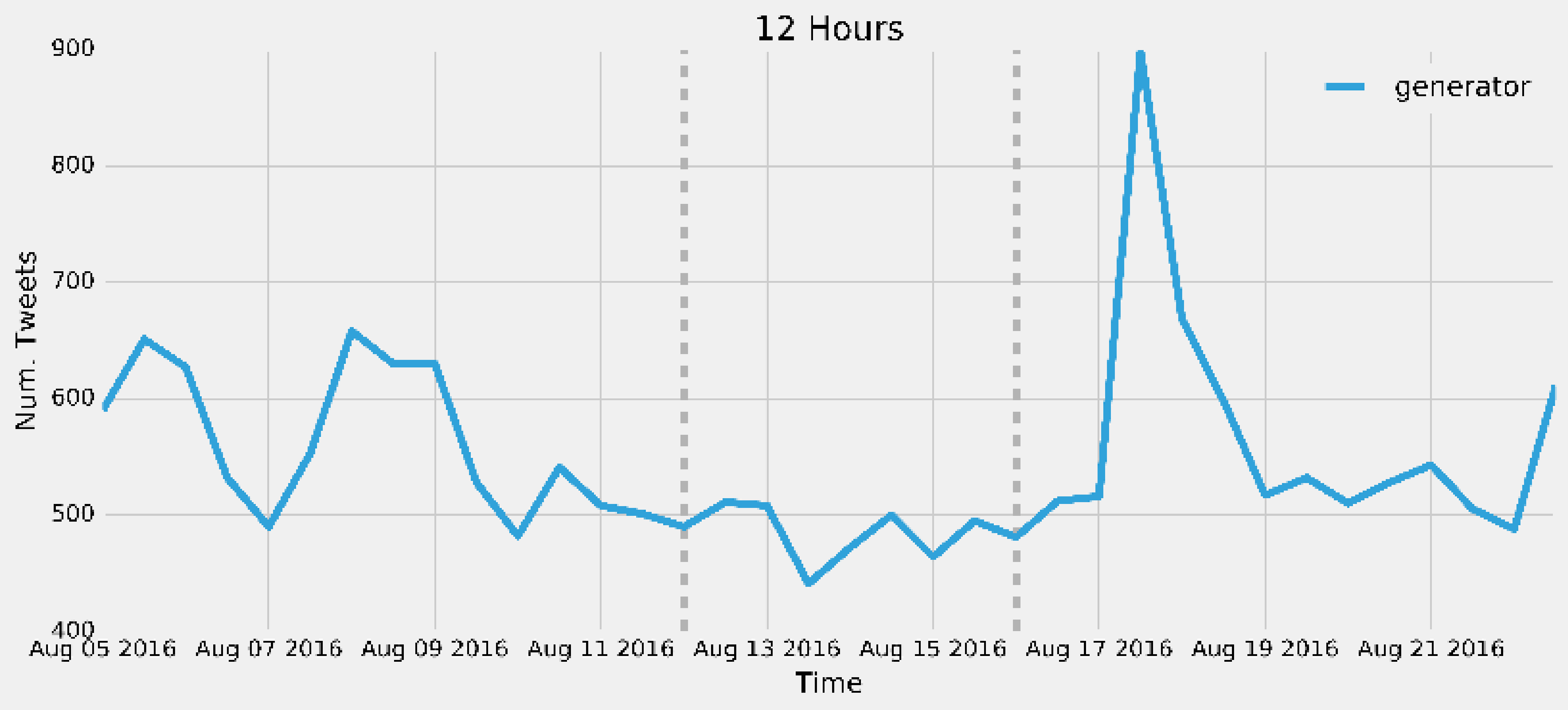

12 Hours
generator
Num. Tweets
900
800
700
600
500
400
Aug 05 2016
Aug 07 2016
Aug 09 2016
Aug 11 2016
Aug 13 2016
Aug 15 2016
Aug 17 2016
Aug 19 2016
Aug 21 2016
Time

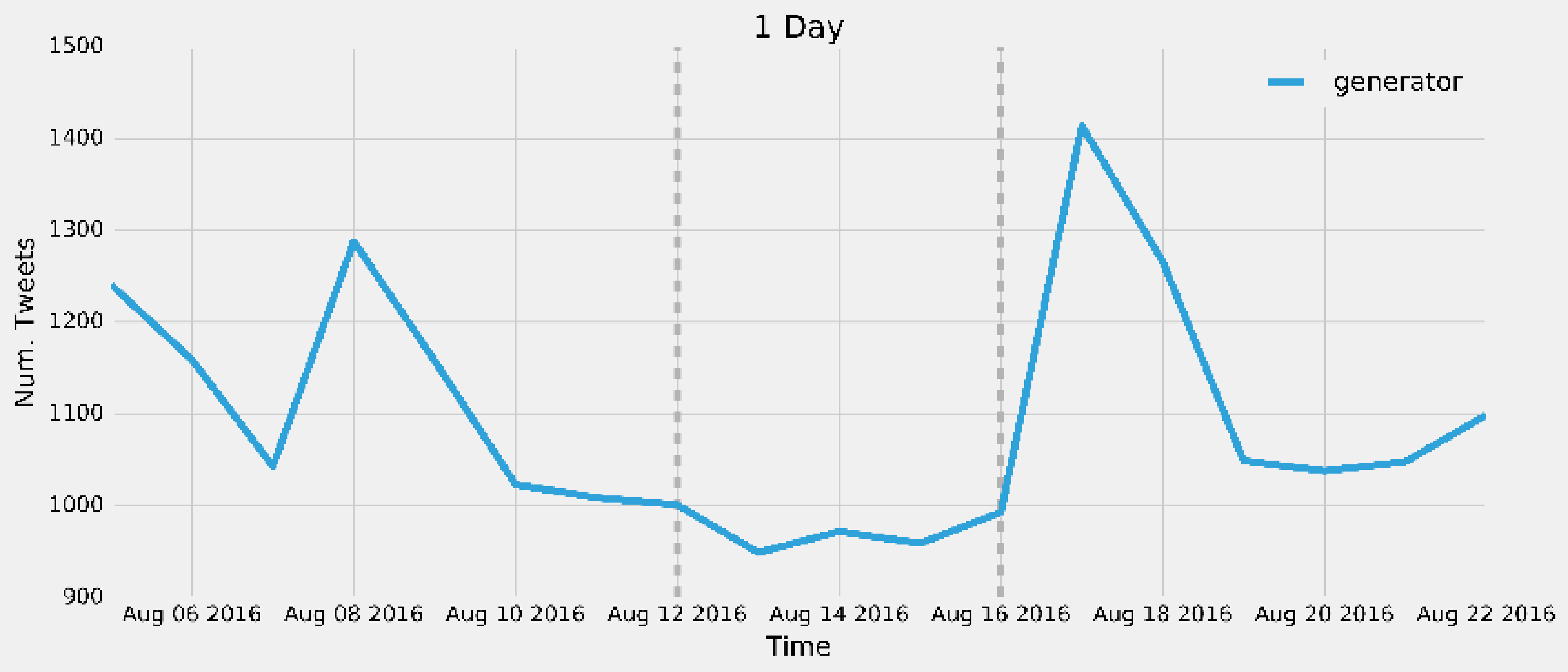
1 Day
1500
1400
1300
1200
1100
1000
900
Num. Tweets
generator
Aug 06 2016
Aug 08 2016
Aug 10 2016
Aug 12 2016
Aug 14 2016
Aug 16 2016
Aug 18 2016
Aug 20 2016
Aug 22 2016
Time

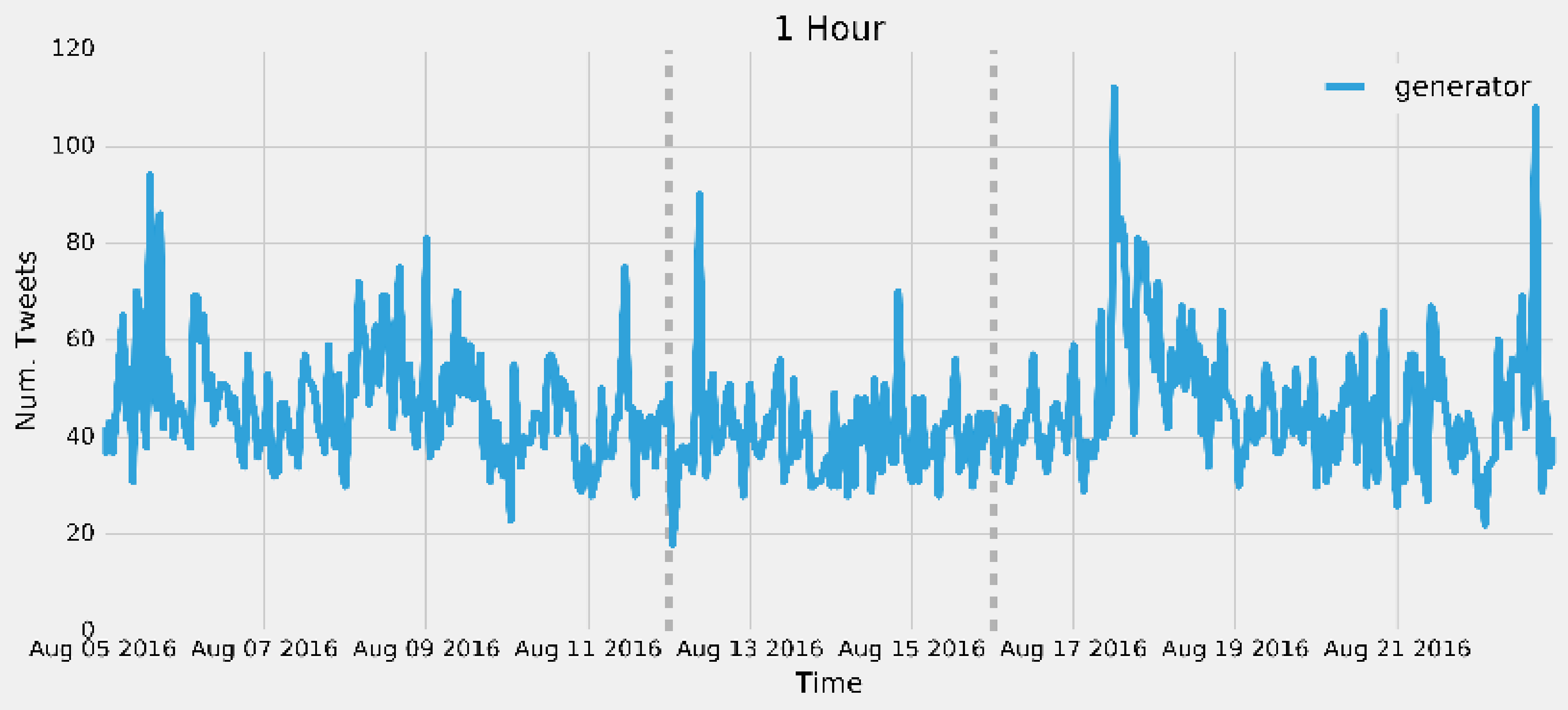
1 Hour
generator
Num. Tweets
Time
0
20
40
60
80
100
120
Aug 05 2016
Aug 07 2016
Aug 09 2016
Aug 11 2016
Aug 13 2016
Aug 15 2016
Aug 17 2016
Aug 19 2016
Aug 21 2016

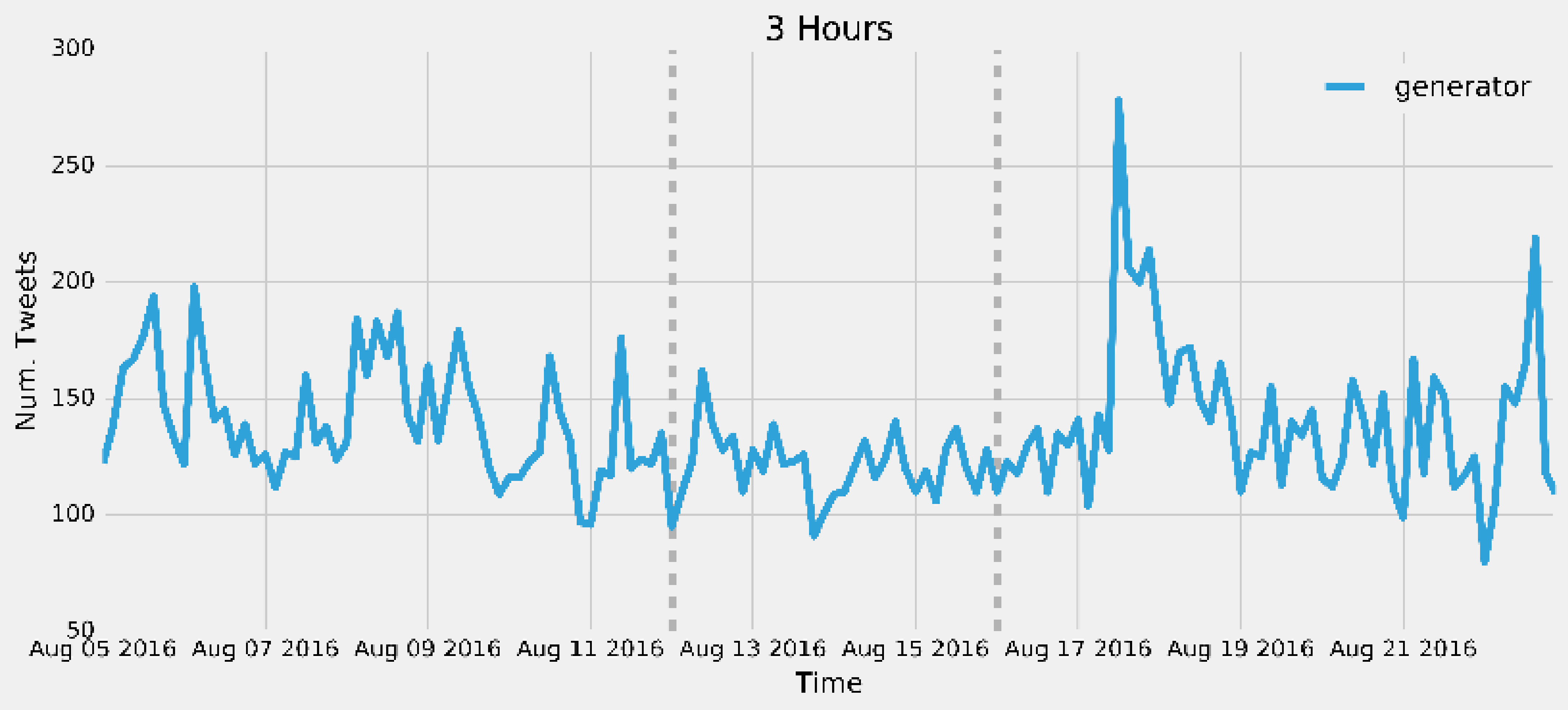
3 Hours
generator
Num. Tweets
300
250
200
150
100
50
Aug 05 2016
Aug 07 2016
Aug 09 2016
Aug 11 2016
Aug 13 2016
Aug 15 2016
Aug 17 2016
Aug 19 2016
Aug 21 2016
Time

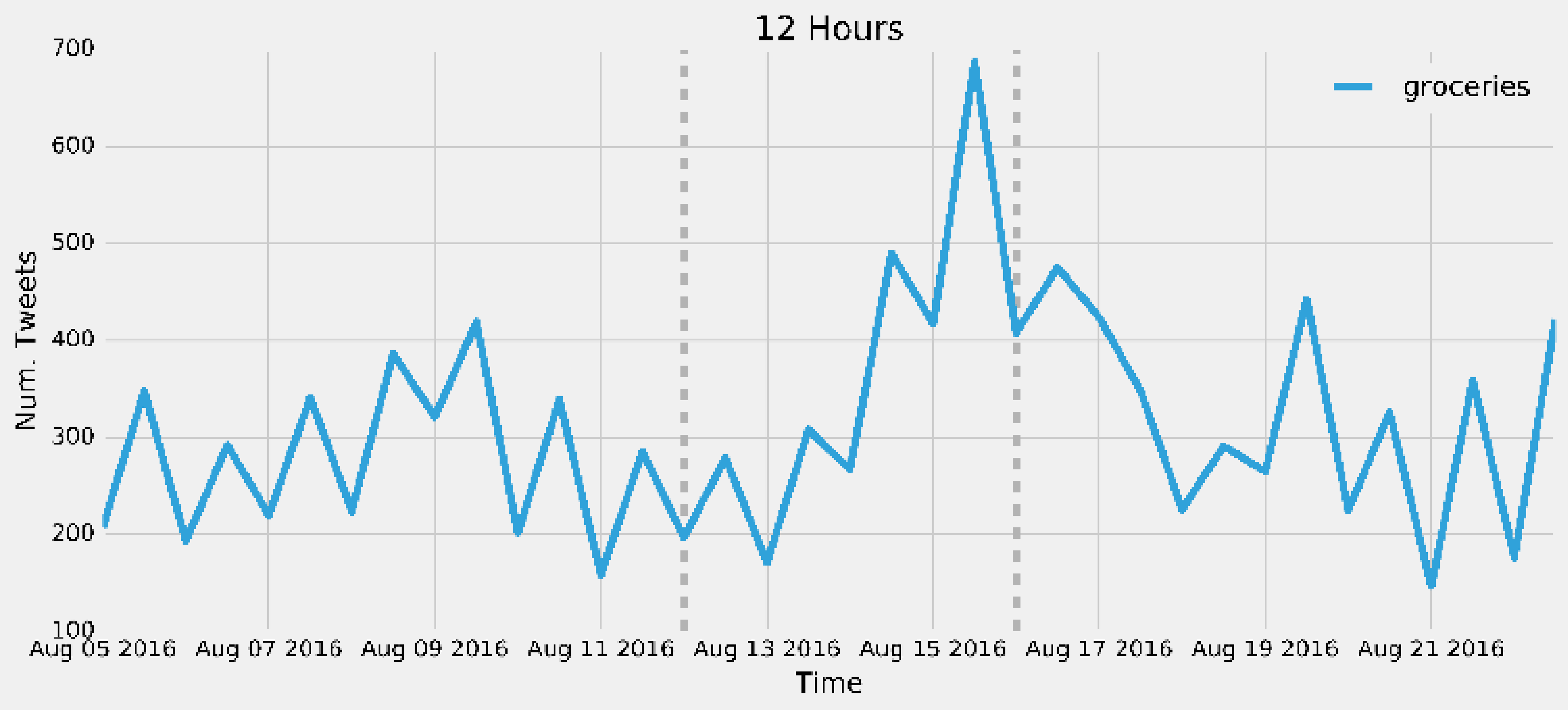

12 Hours
groceries
700
600
500
400
300
200
100
Num. Tweets
Aug 05 2016
Aug 07 2016
Aug 09 2016
Aug 11 2016
Aug 13 2016
Aug 15 2016
Aug 17 2016
Aug 19 2016
Aug 21 2016
Time

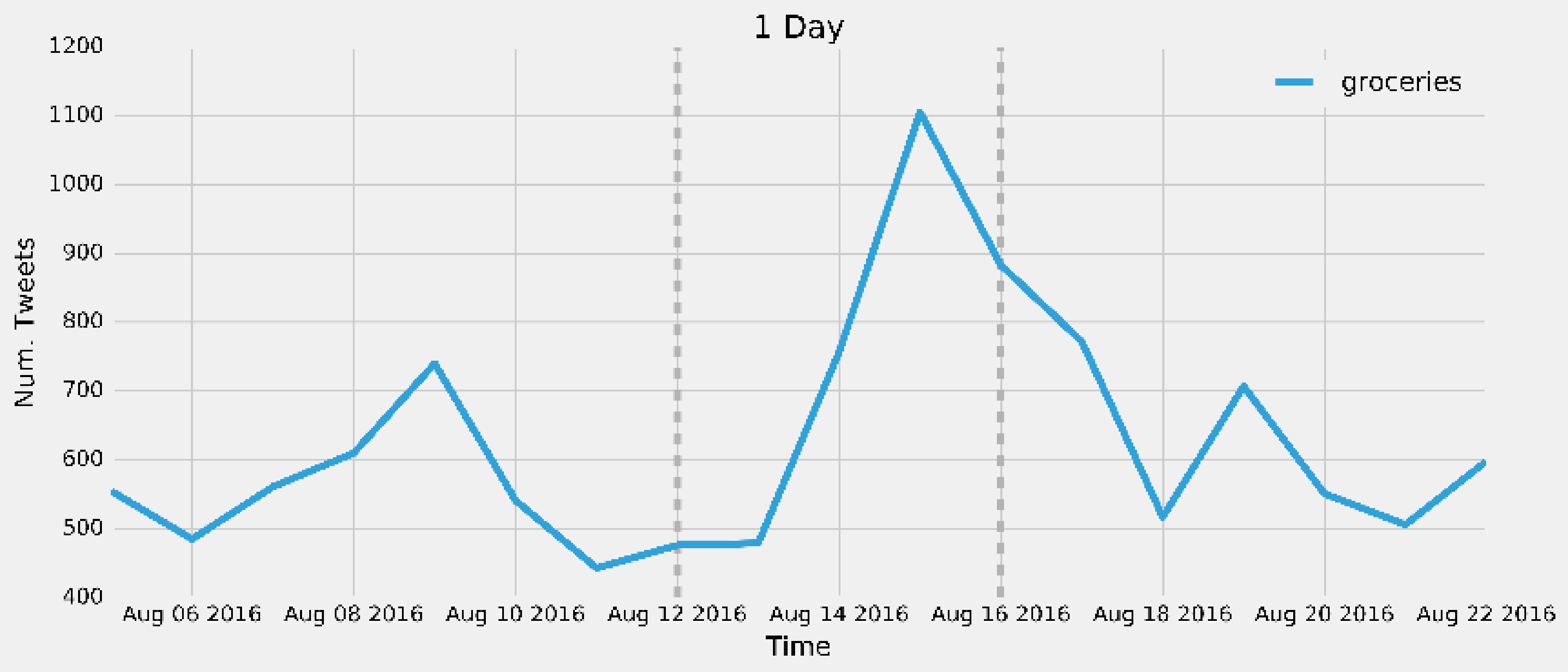
1 Day
groceries
Num. Tweets
1200
1100
1000
900
800
700
600
500
400
Aug 06 2016
Aug 08 2016
Aug 10 2016
Aug 12 2016
Aug 14 2016
Aug 16 2016
Aug 18 2016
Aug 20 2016
Aug 22 2016
Time

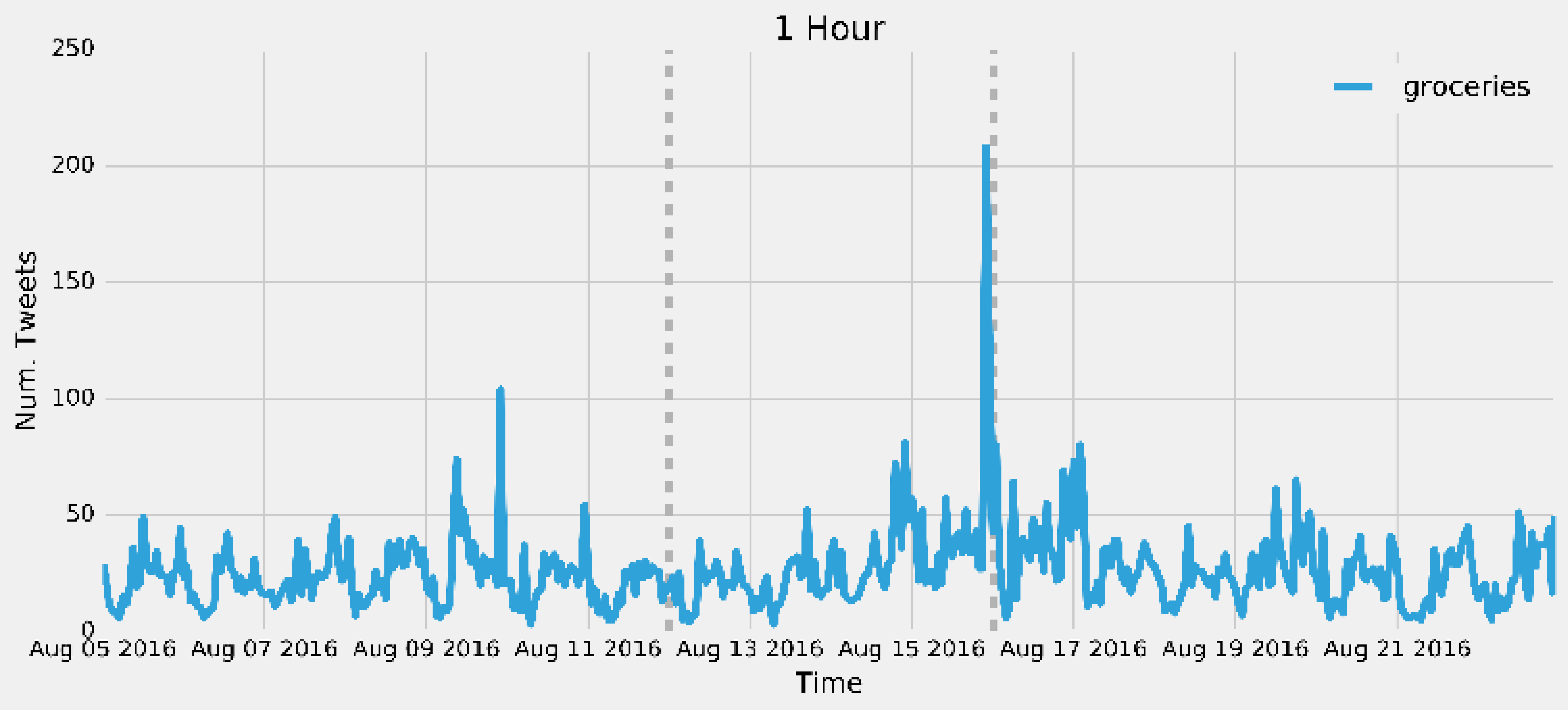
1 Hour
groceries
Num. Tweets
0
50
100
150
200
250
Aug 05 2016
Aug 07 2016
Aug 09 2016
Aug 11 2016
Aug 13 2016
Aug 15 2016
Aug 17 2016
Aug 19 2016
Aug 21 2016
Time

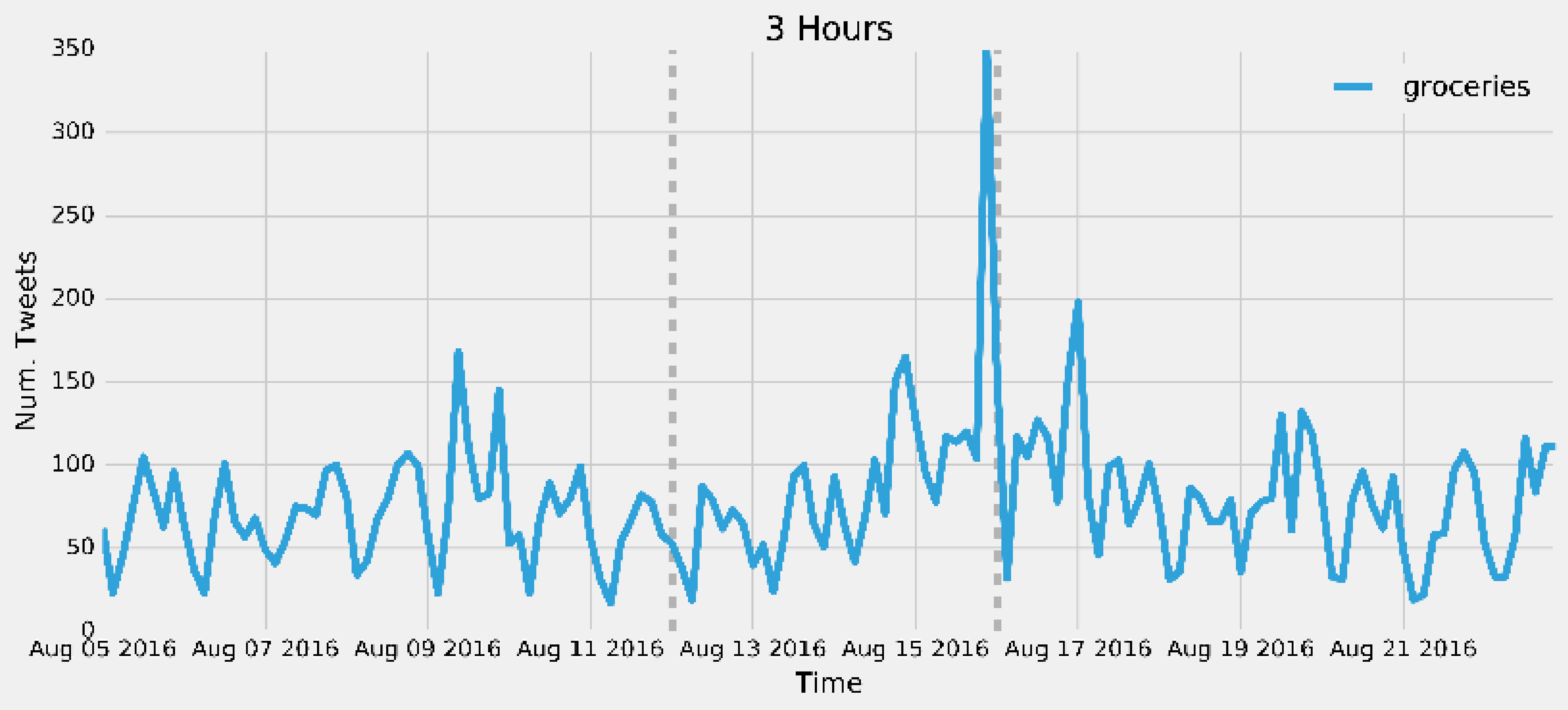

3 Hours
groceries
Num. Tweets
Time
0
50
100
150
200
250
300
350
Aug 05 2016
Aug 07 2016
Aug 09 2016
Aug 11 2016
Aug 13 2016
Aug 15 2016
Aug 17 2016
Aug 19 2016
Aug 21 2016

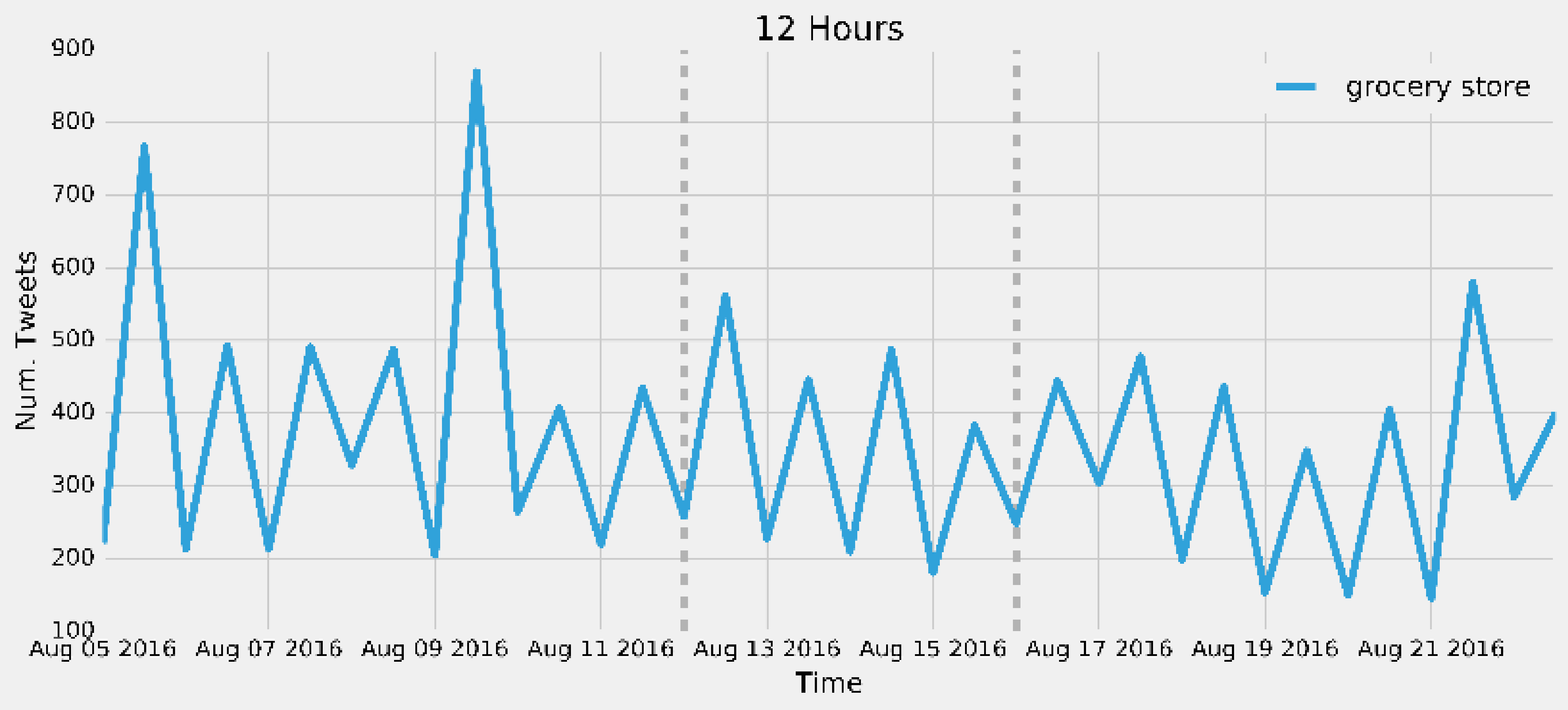
12 Hours
grocery store
Num. Tweets
900
800
700
600
500
400
300
200
100
Aug 05 2016
Aug 07 2016
Aug 09 2016
Aug 11 2016
Aug 13 2016
Aug 15 2016
Aug 17 2016
Aug 19 2016
Aug 21 2016
Time

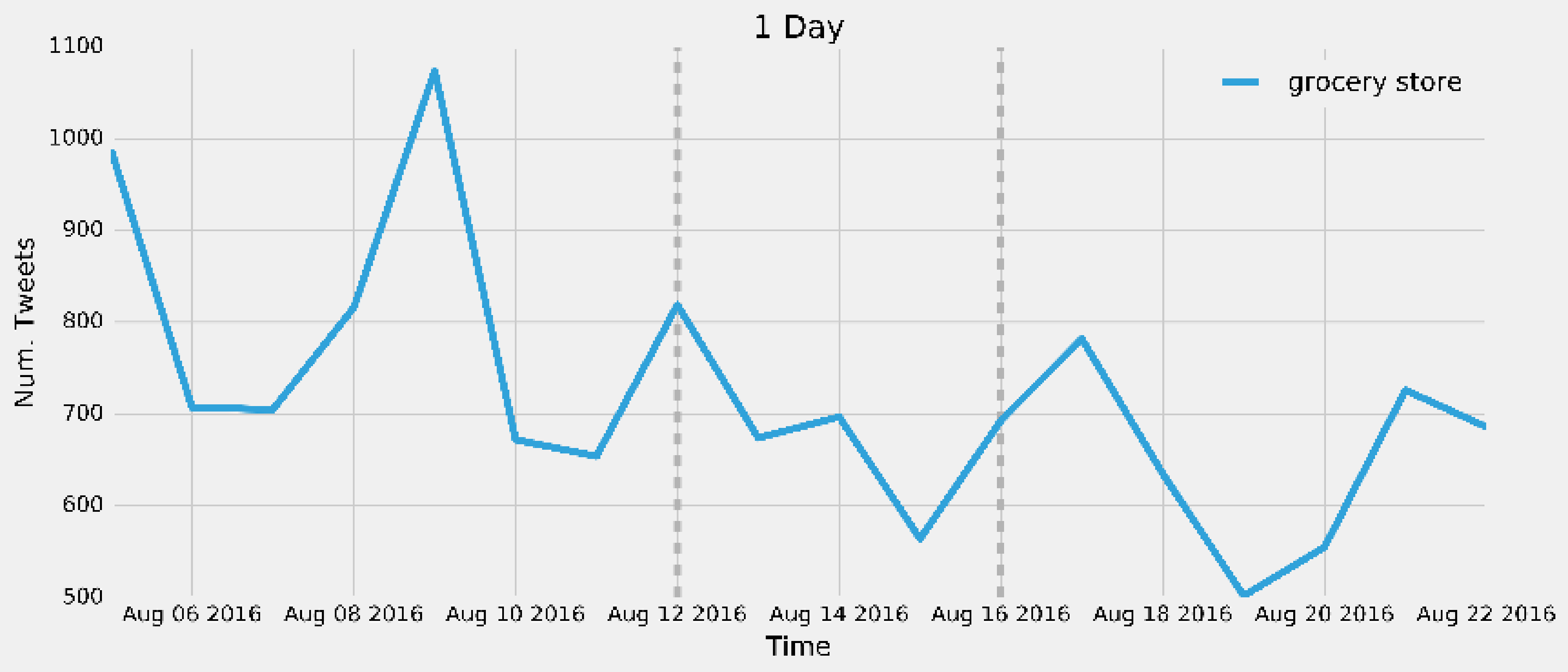

1 Day
grocery store
1100
1000
900
800
700
600
500
Num. Tweets
Aug 06 2016
Aug 08 2016
Aug 10 2016
Aug 12 2016
Aug 14 2016
Aug 16 2016
Aug 18 2016
Aug 20 2016
Aug 22 2016
Time

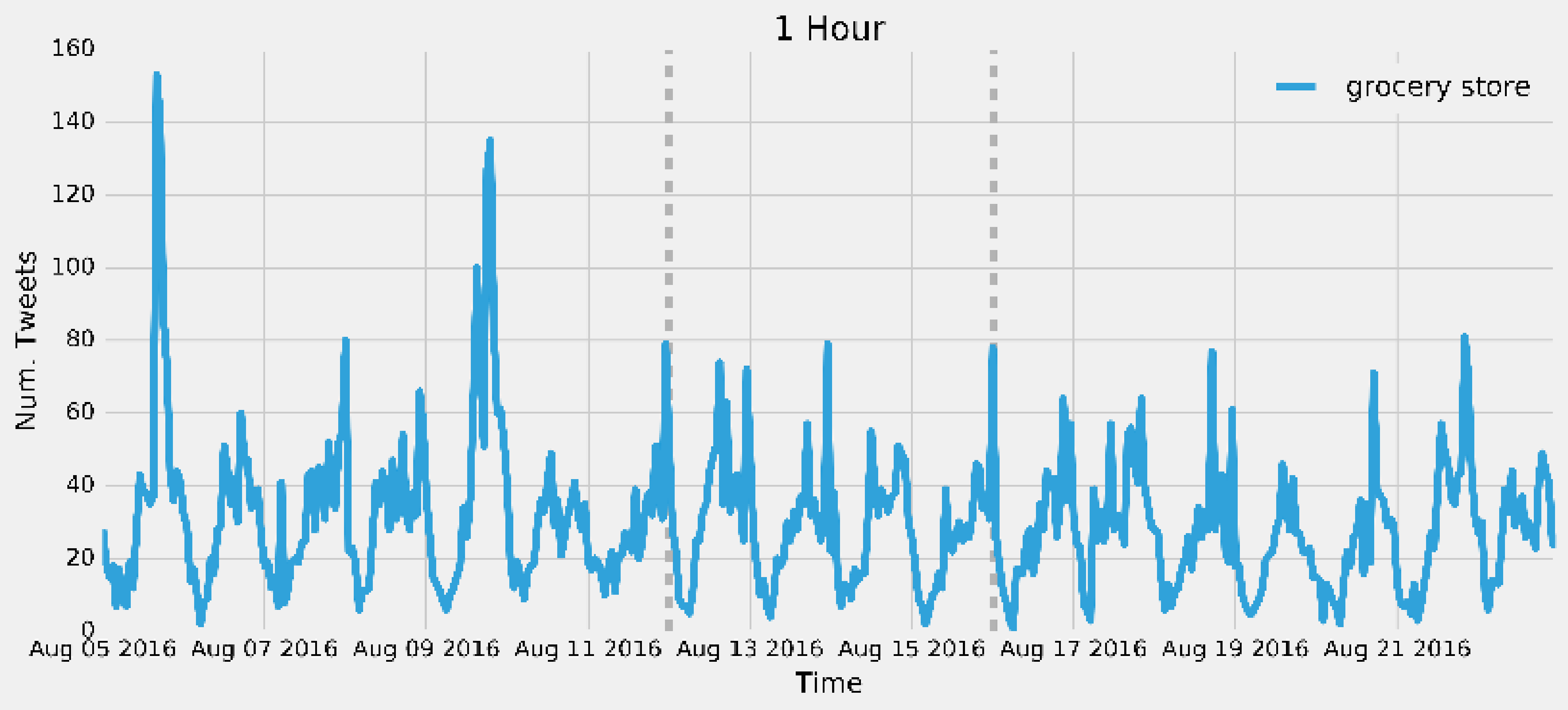
1 Hour
grocery store
Num. Tweets
Time
0
20
40
60
80
100
120
140
160
Aug 05 2016
Aug 07 2016
Aug 09 2016
Aug 11 2016
Aug 13 2016
Aug 15 2016
Aug 17 2016
Aug 19 2016
Aug 21 2016

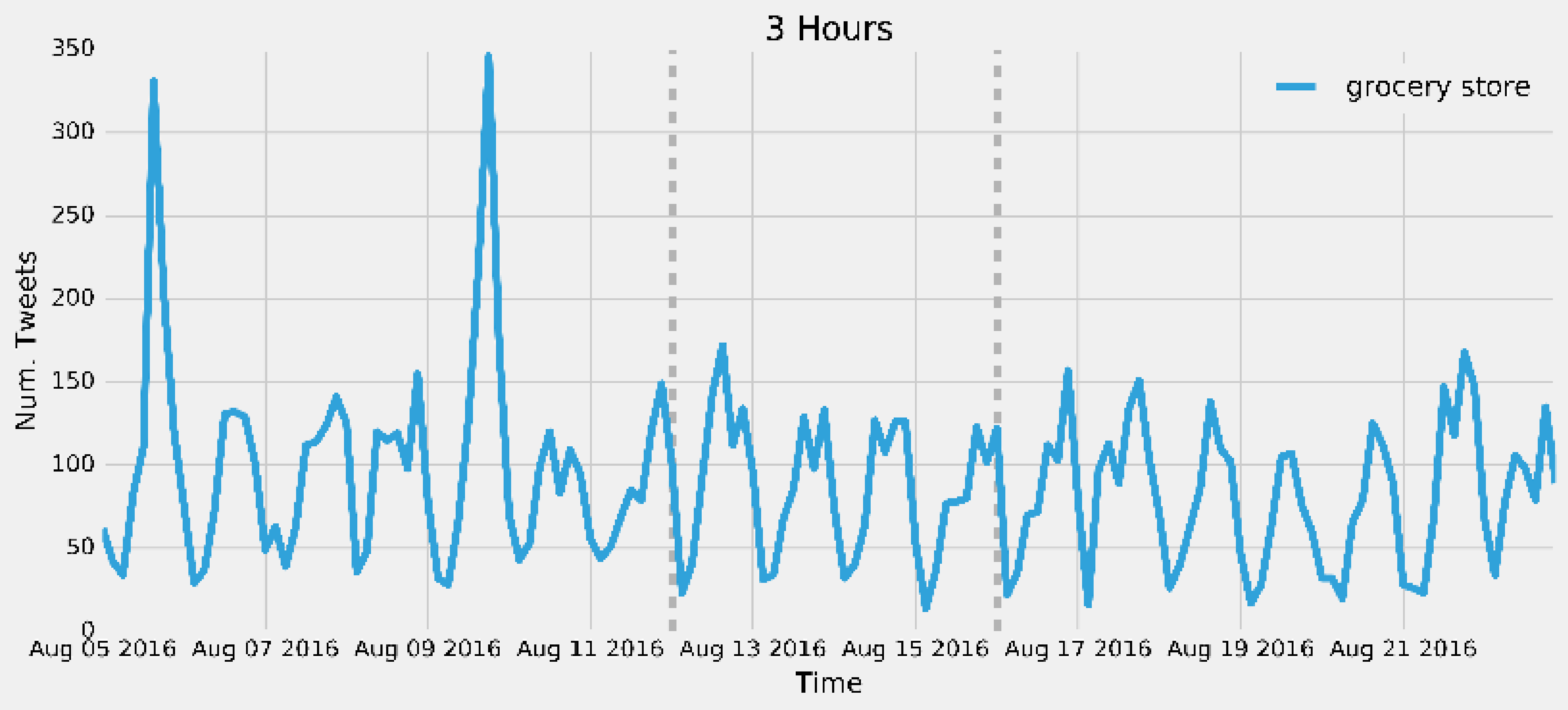
3 Hours
grocery store
Num. Tweets
0
50
100
150
200
250
300
350
Aug 05 2016
Aug 07 2016
Aug 09 2016
Aug 11 2016
Aug 13 2016
Aug 15 2016
Aug 17 2016
Aug 19 2016
Aug 21 2016
Time

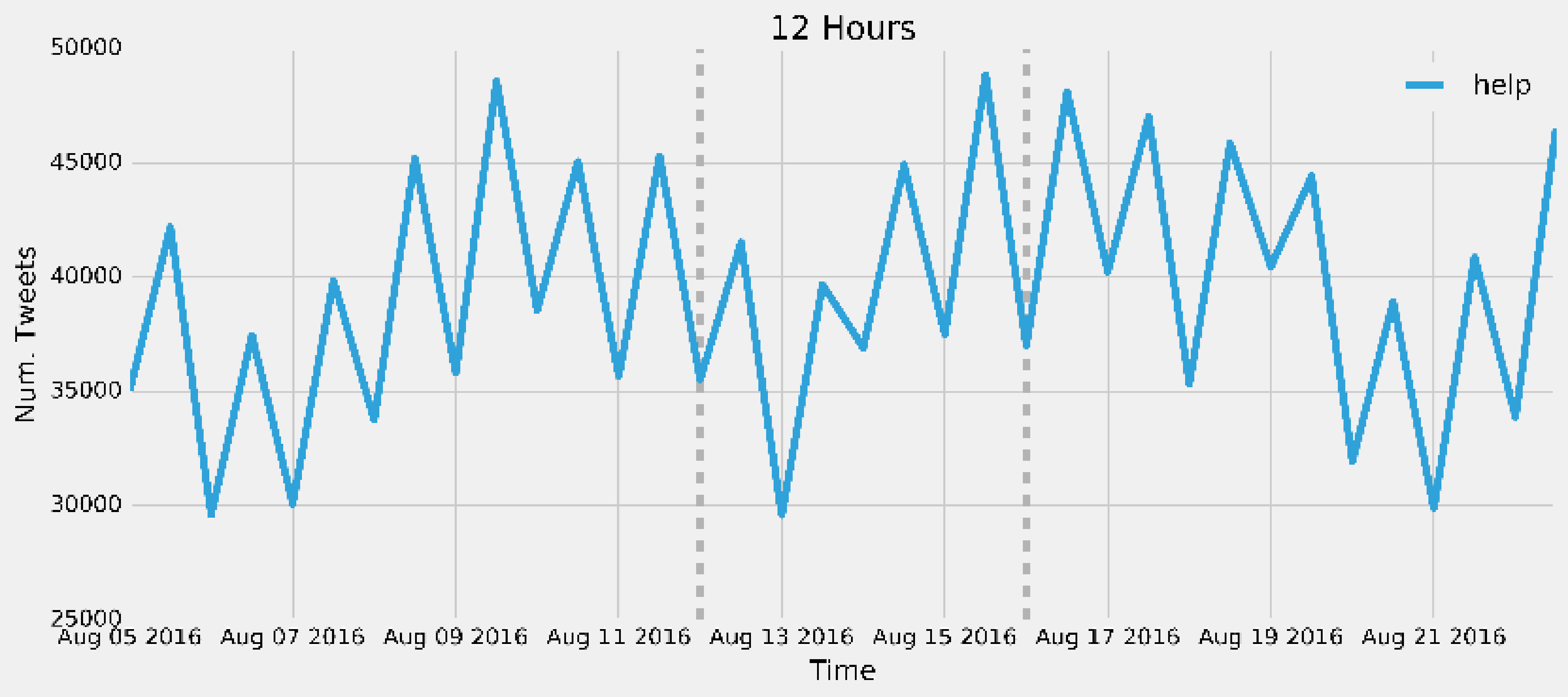

12 Hours
help
Num. Tweets
Time
50000
45000
40000
35000
30000
25000
Aug 05 2016
Aug 07 2016
Aug 09 2016
Aug 11 2016
Aug 13 2016
Aug 15 2016
Aug 17 2016
Aug 19 2016
Aug 21 2016

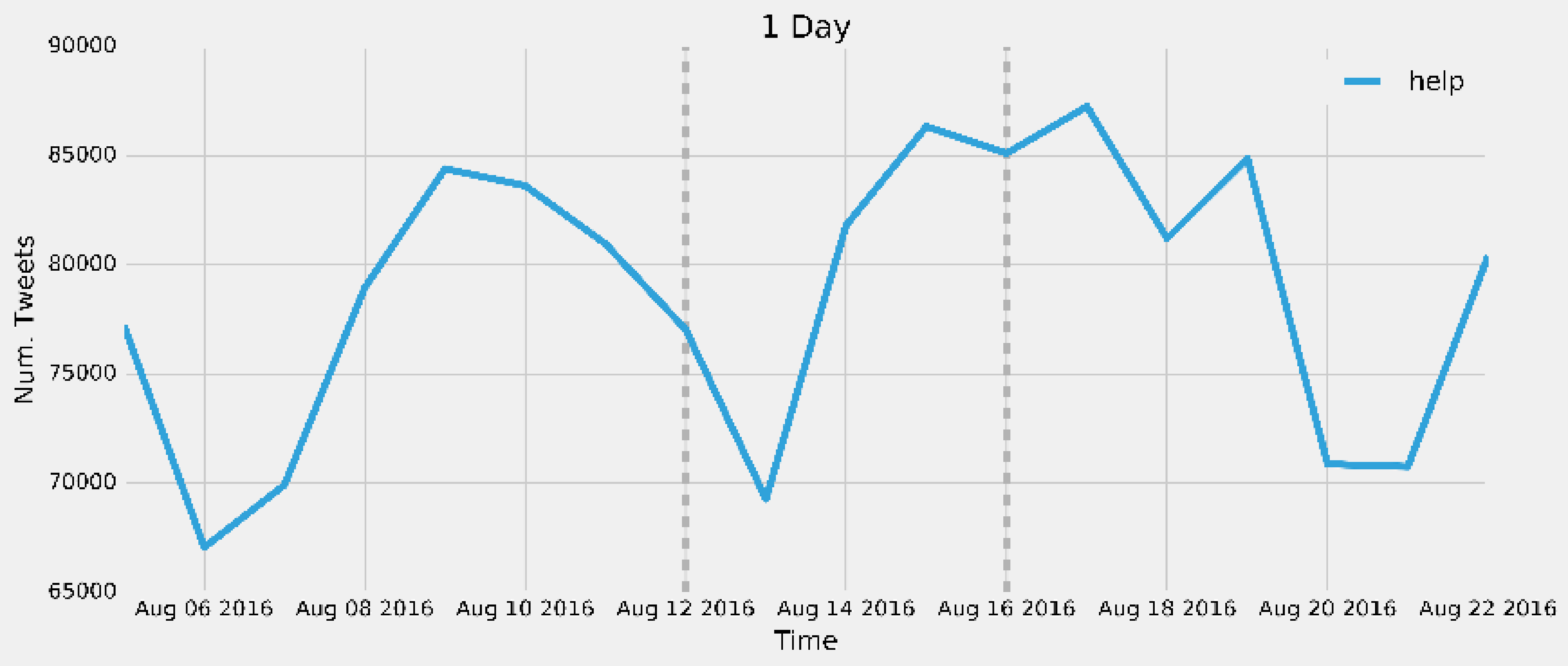
1 Day
help
Num. Tweets
90000
85000
80000
75000
70000
65000
Aug 06 2016
Aug 08 2016
Aug 10 2016
Aug 12 2016
Aug 14 2016
Aug 16 2016
Aug 18 2016
Aug 20 2016
Aug 22 2016
Time

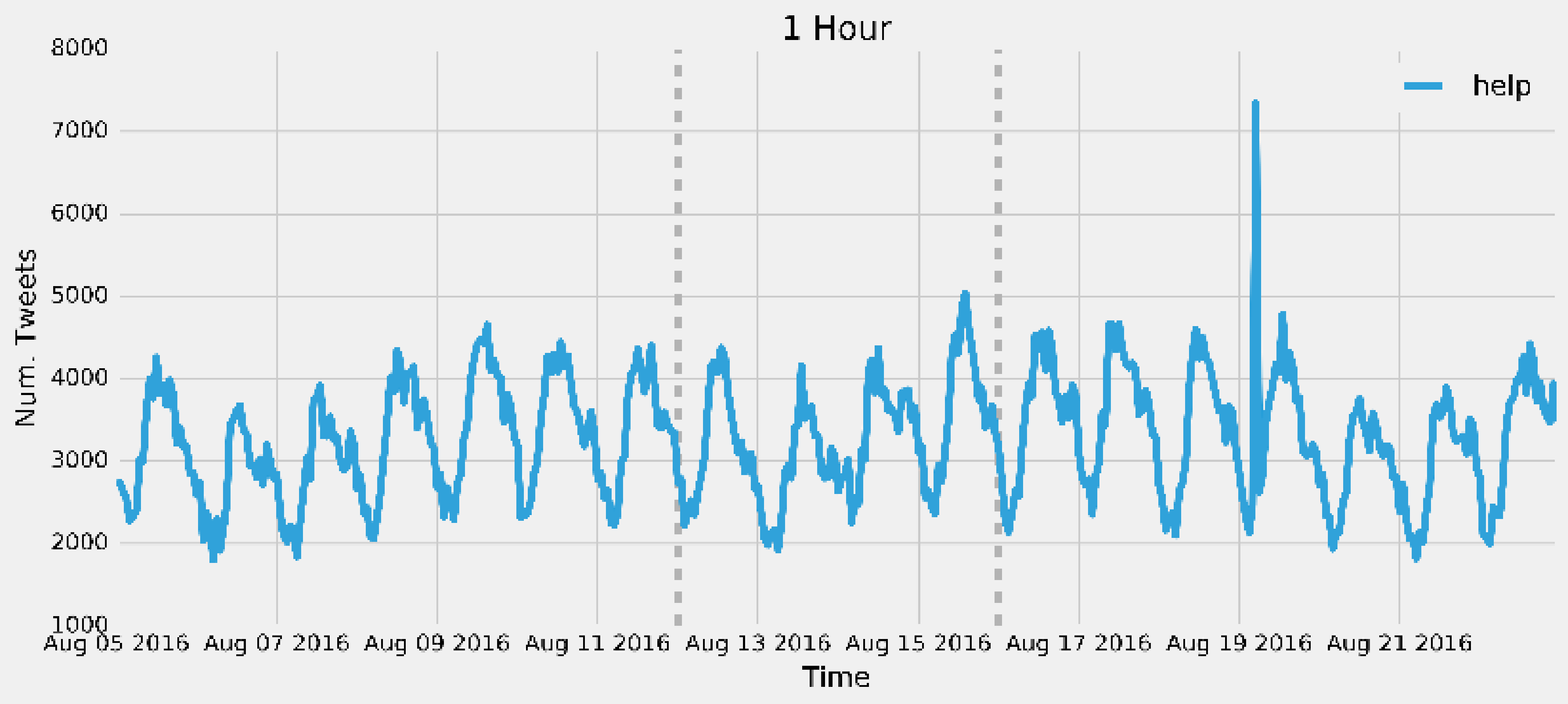

1 Hour
help
Num. Tweets
8000
7000
6000
5000
4000
3000
2000
1000
Aug 05 2016
Aug 07 2016
Aug 09 2016
Aug 11 2016
Aug 13 2016
Aug 15 2016
Aug 17 2016
Aug 19 2016
Aug 21 2016
Time

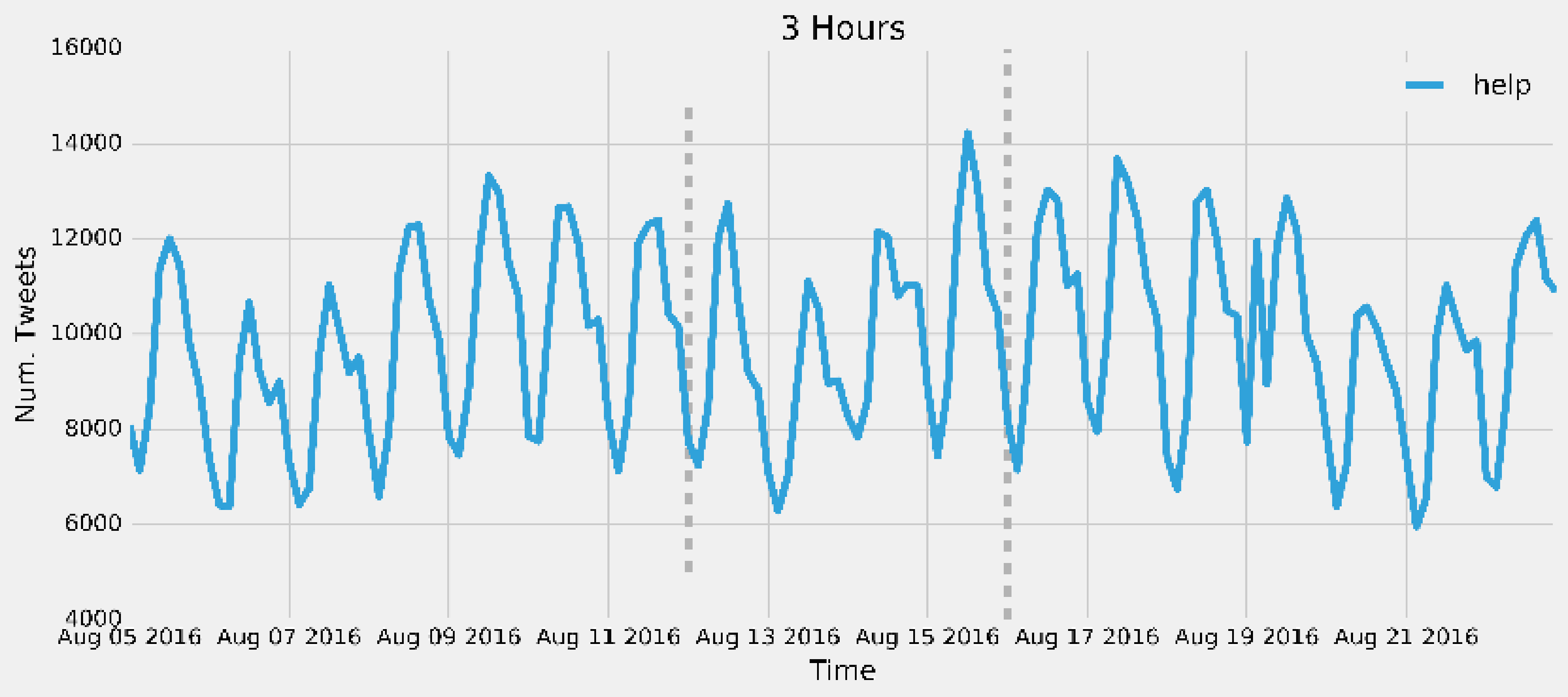

3 Hours
help
16000
14000
12000
10000
8000
6000
4000
Num. Tweets
Aug 05 2016
Aug 07 2016
Aug 09 2016
Aug 11 2016
Aug 13 2016
Aug 15 2016
Aug 17 2016
Aug 19 2016
Aug 21 2016
Time

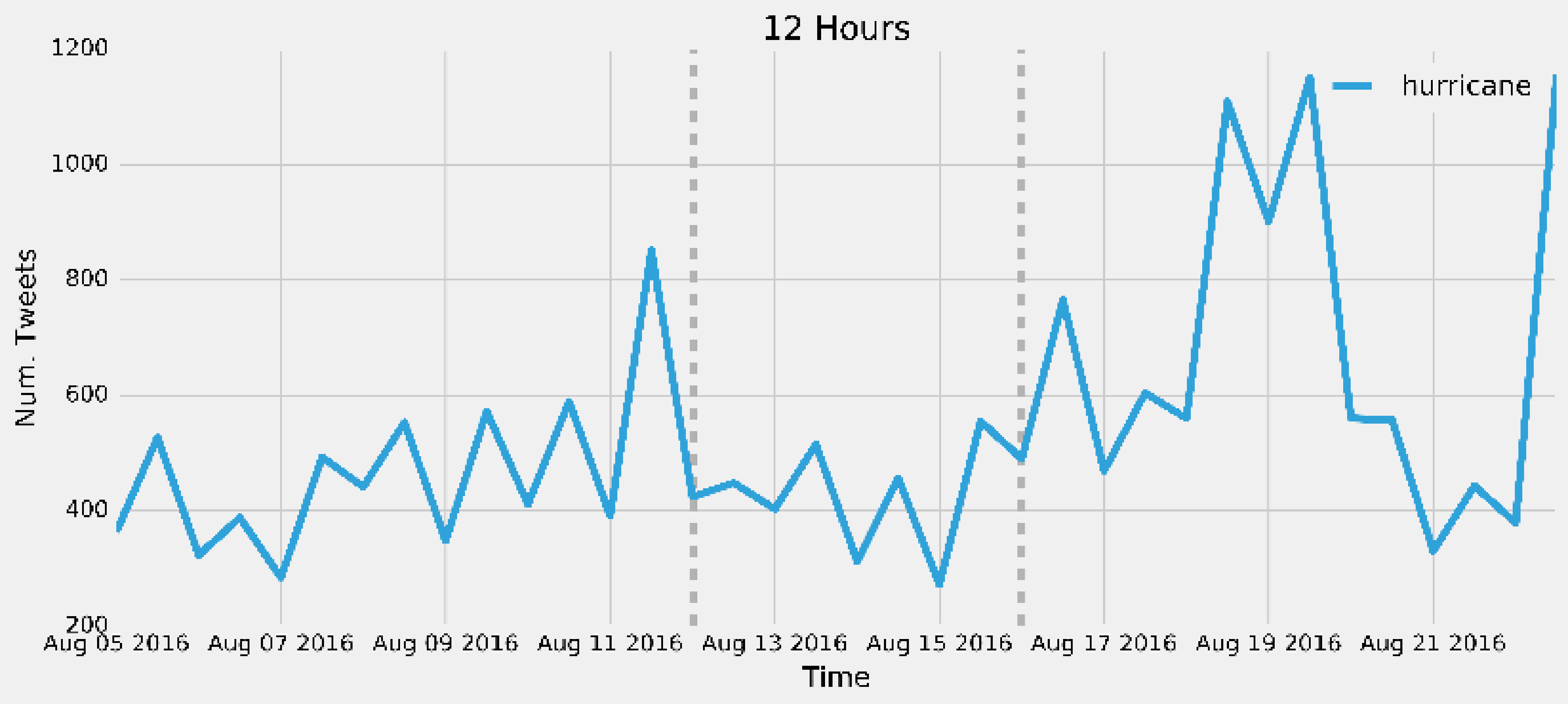

12 Hours
hurricane
Num. Tweets
1200
1000
800
600
400
200
Aug 05 2016
Aug 07 2016
Aug 09 2016
Aug 11 2016
Aug 13 2016
Aug 15 2016
Aug 17 2016
Aug 19 2016
Aug 21 2016
Time

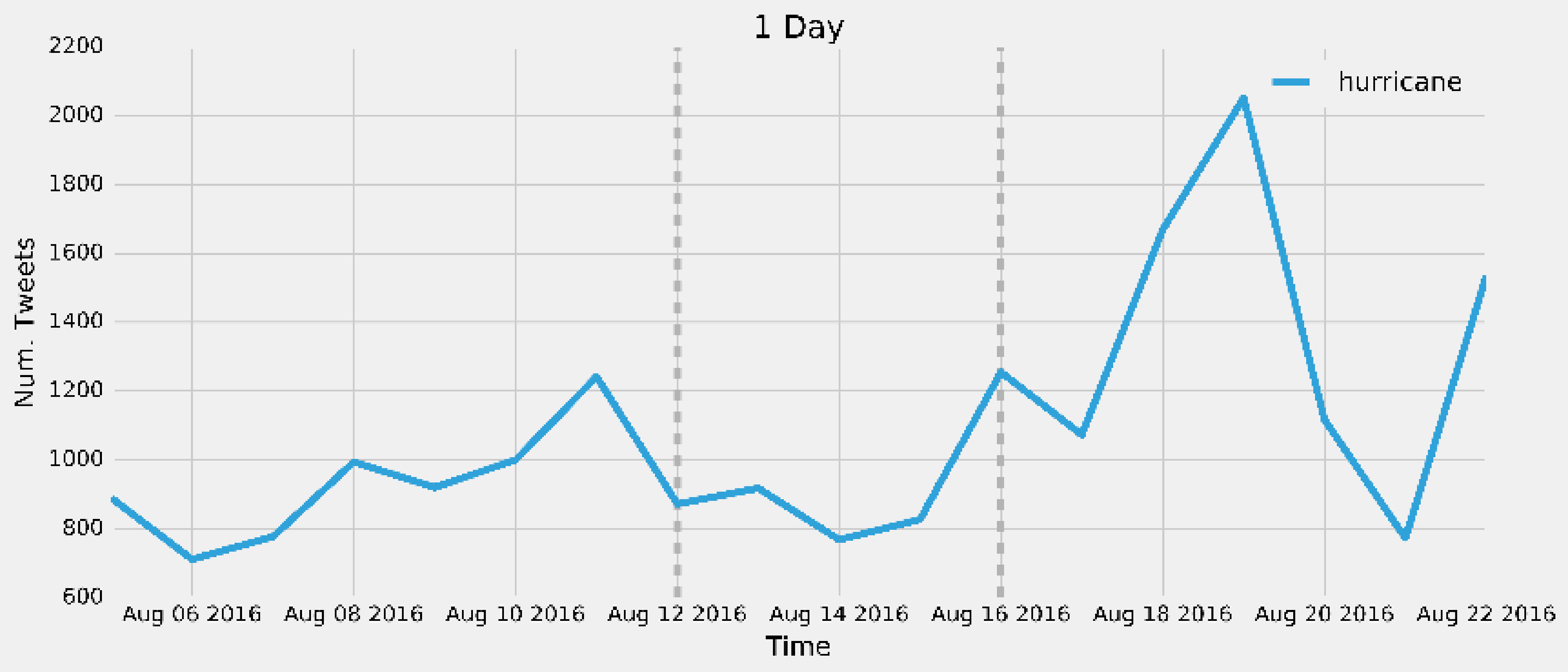

1 Day
hurricane
Num. Tweets
2200
2000
1800
1600
1400
1200
1000
800
600
Aug 06 2016
Aug 08 2016
Aug 10 2016
Aug 12 2016
Aug 14 2016
Aug 16 2016
Aug 18 2016
Aug 20 2016
Aug 22 2016
Time

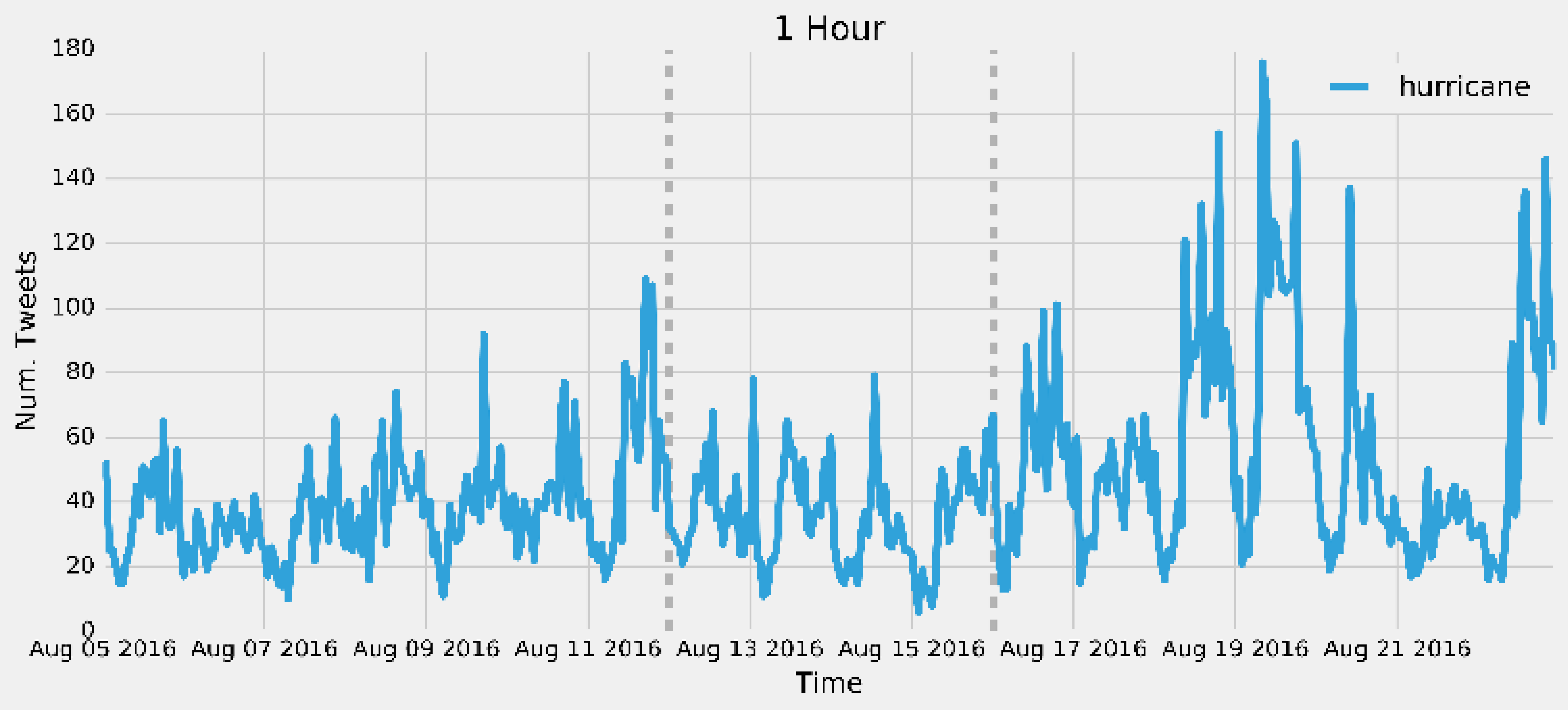
1 Hour
hurricane
Num. Tweets
180
160
140
120
100
80
60
40
20
0
Aug 05 2016
Aug 07 2016
Aug 09 2016
Aug 11 2016
Aug 13 2016
Aug 15 2016
Aug 17 2016
Aug 19 2016
Aug 21 2016
Time

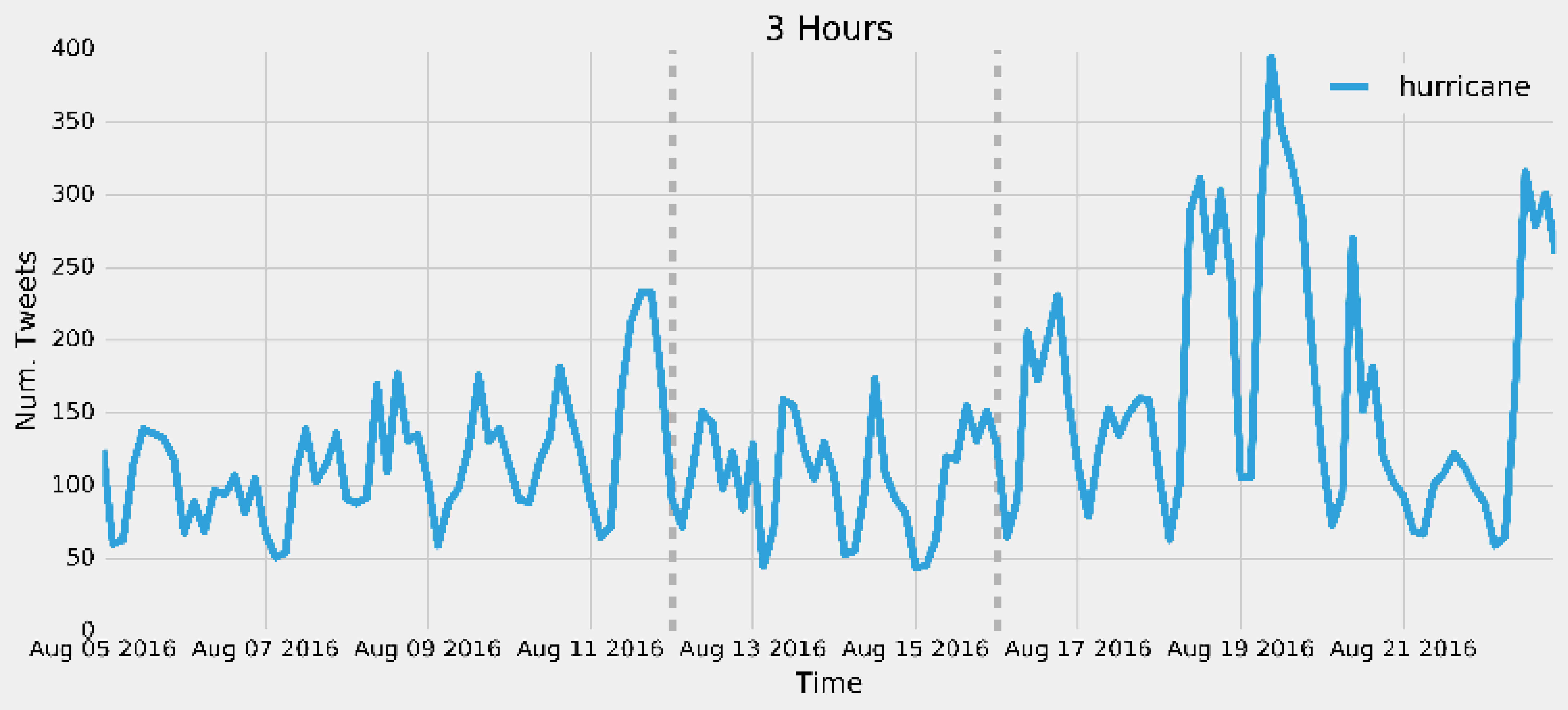

3 Hours
hurricane
Num. Tweets
Time
0
50
100
150
200
250
300
350
400
Aug 05 2016
Aug 07 2016
Aug 09 2016
Aug 11 2016
Aug 13 2016
Aug 15 2016
Aug 17 2016
Aug 19 2016
Aug 21 2016

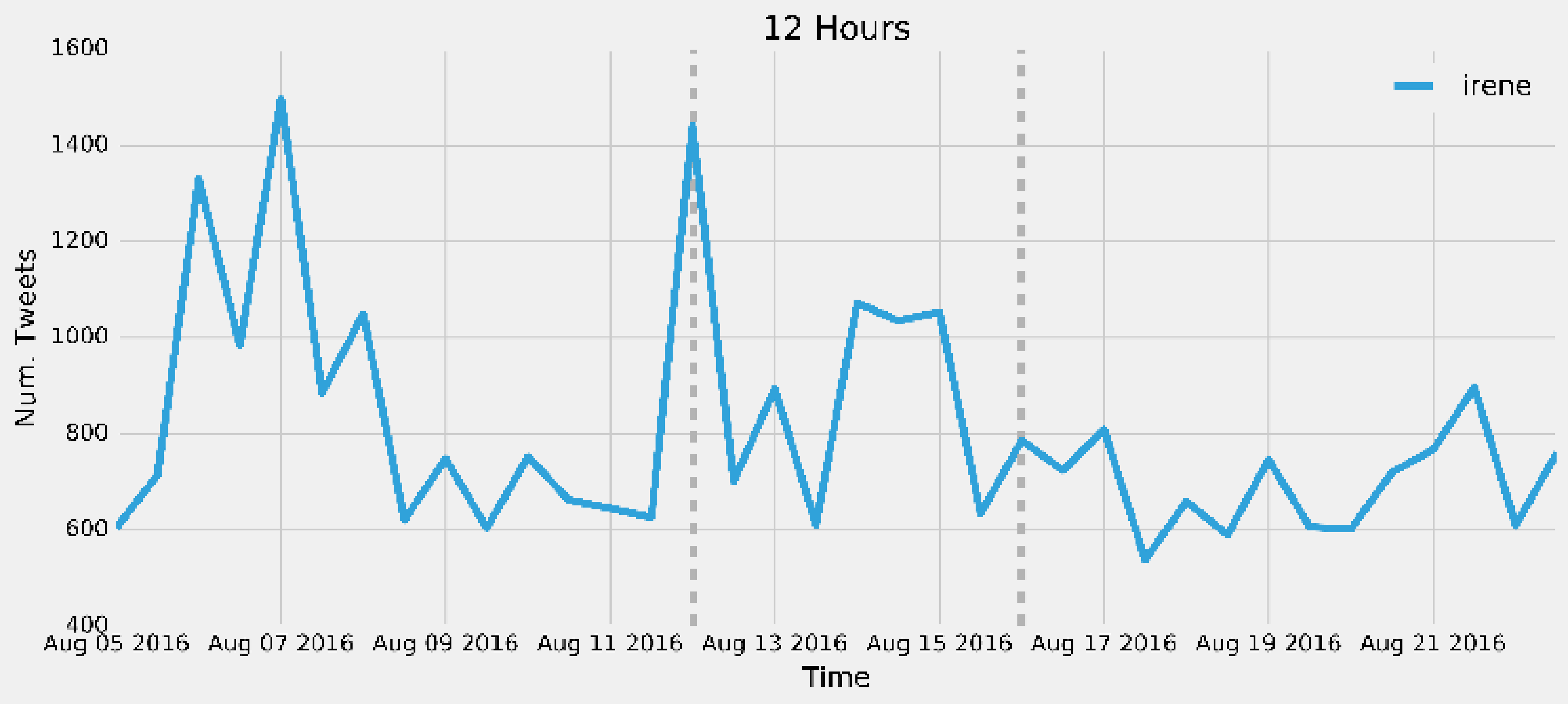
12 Hours
irene
Num. Tweets
1600
1400
1200
1000
800
600
400
Aug 05 2016
Aug 07 2016
Aug 09 2016
Aug 11 2016
Aug 13 2016
Aug 15 2016
Aug 17 2016
Aug 19 2016
Aug 21 2016
Time

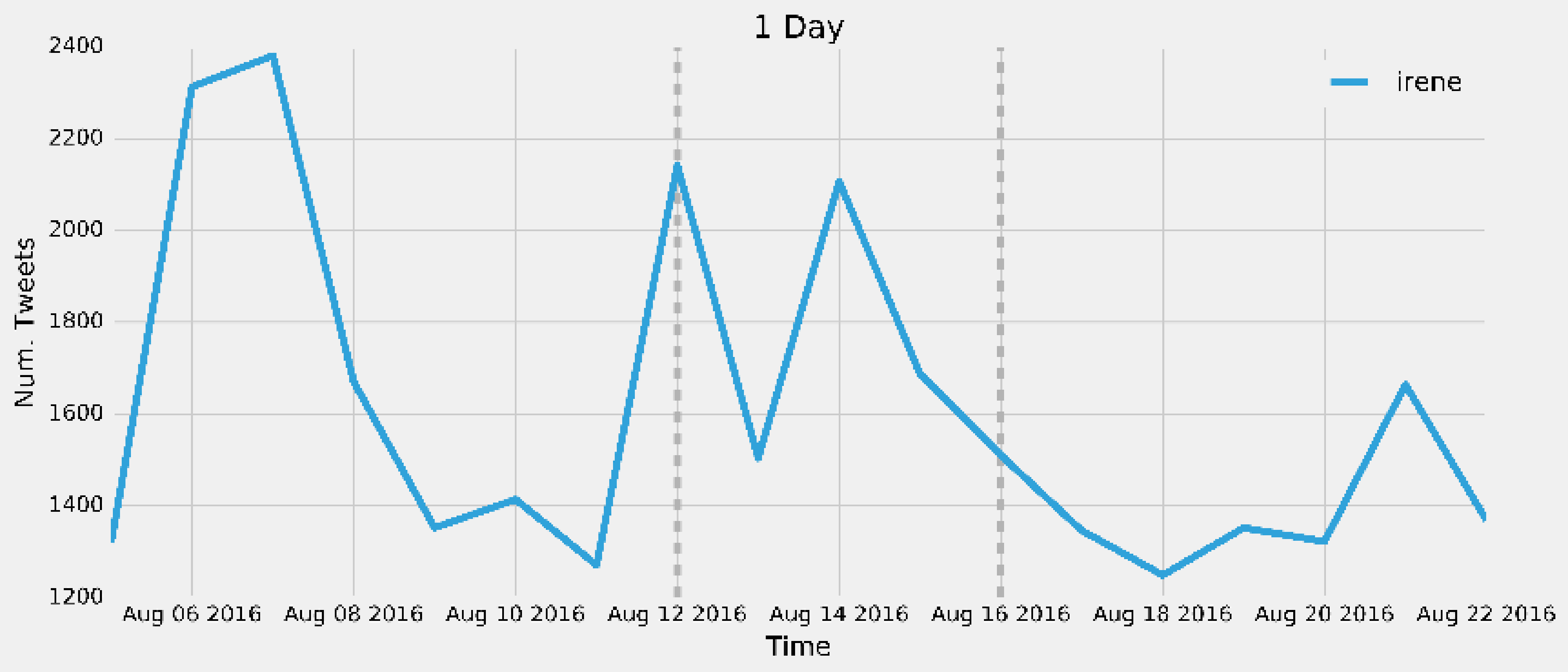
1 Day
2400
2200
2000
1800
1600
1400
1200
Num. Tweets
irene
Aug 06 2016
Aug 08 2016
Aug 10 2016
Aug 12 2016
Aug 14 2016
Aug 16 2016
Aug 18 2016
Aug 20 2016
Aug 22 2016
Time

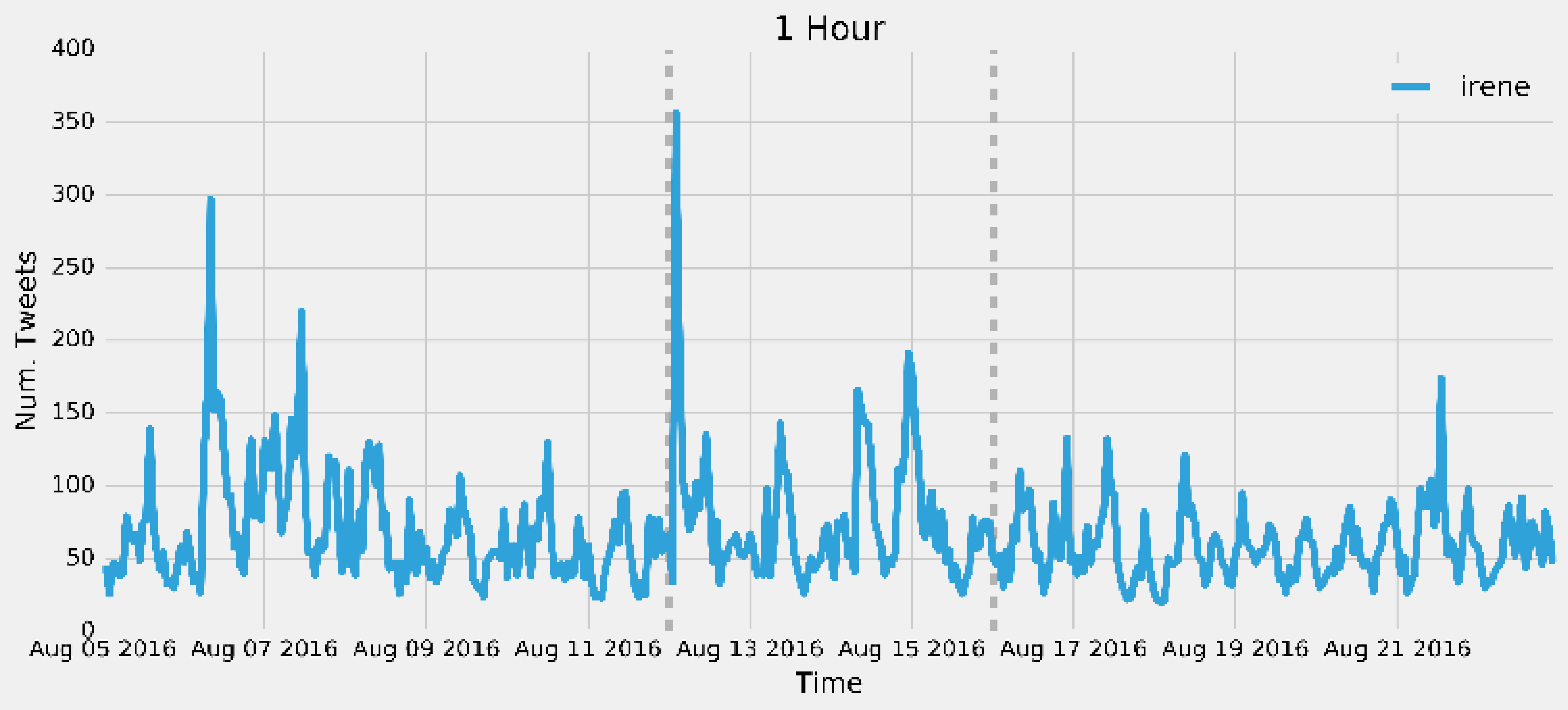
1 Hour
irene
Num. Tweets
0
50
100
150
200
250
300
350
400
Aug 05 2016
Aug 07 2016
Aug 09 2016
Aug 11 2016
Aug 13 2016
Aug 15 2016
Aug 17 2016
Aug 19 2016
Aug 21 2016
Time

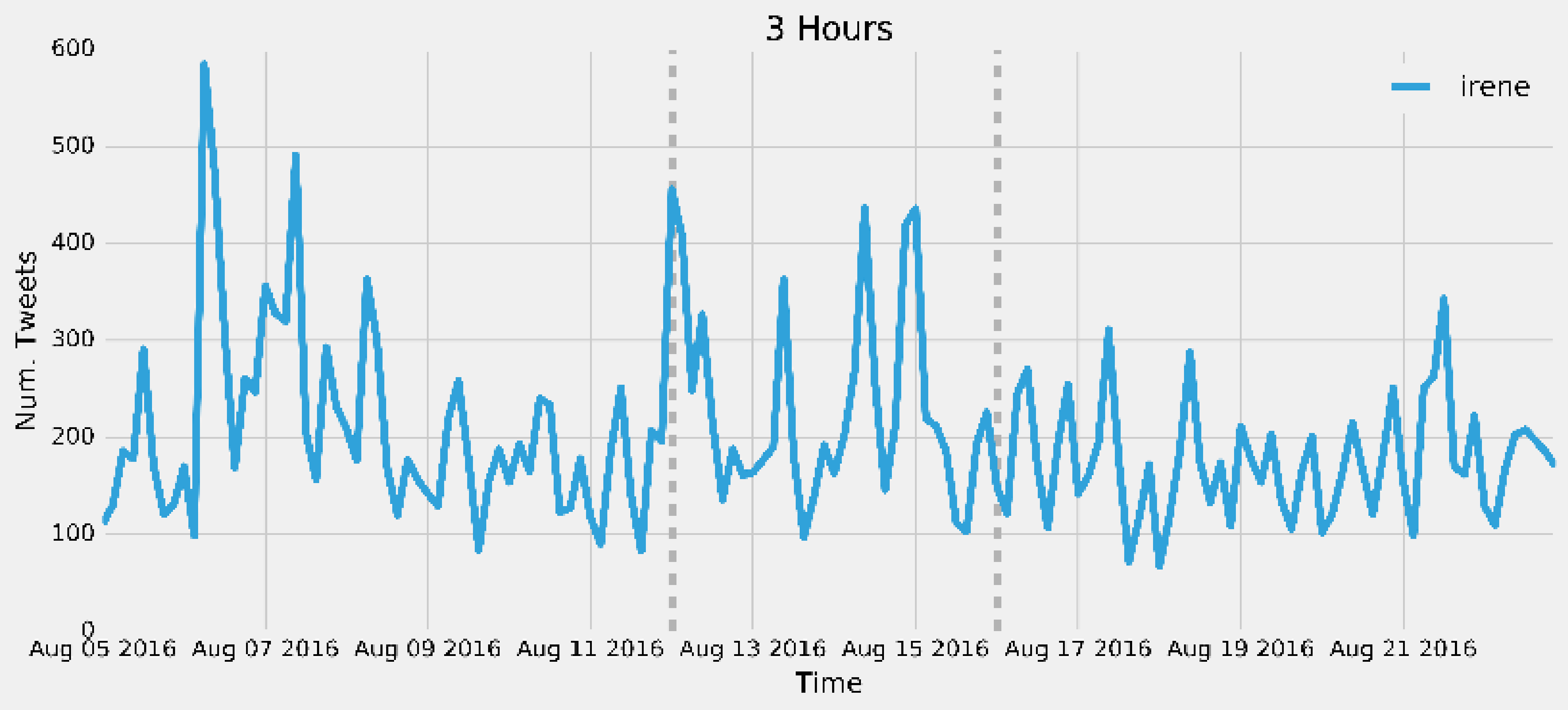
3 Hours
irene
Num. Tweets
Time
0
100
200
300
400
500
600
Aug 05 2016
Aug 07 2016
Aug 09 2016
Aug 11 2016
Aug 13 2016
Aug 15 2016
Aug 17 2016
Aug 19 2016
Aug 21 2016

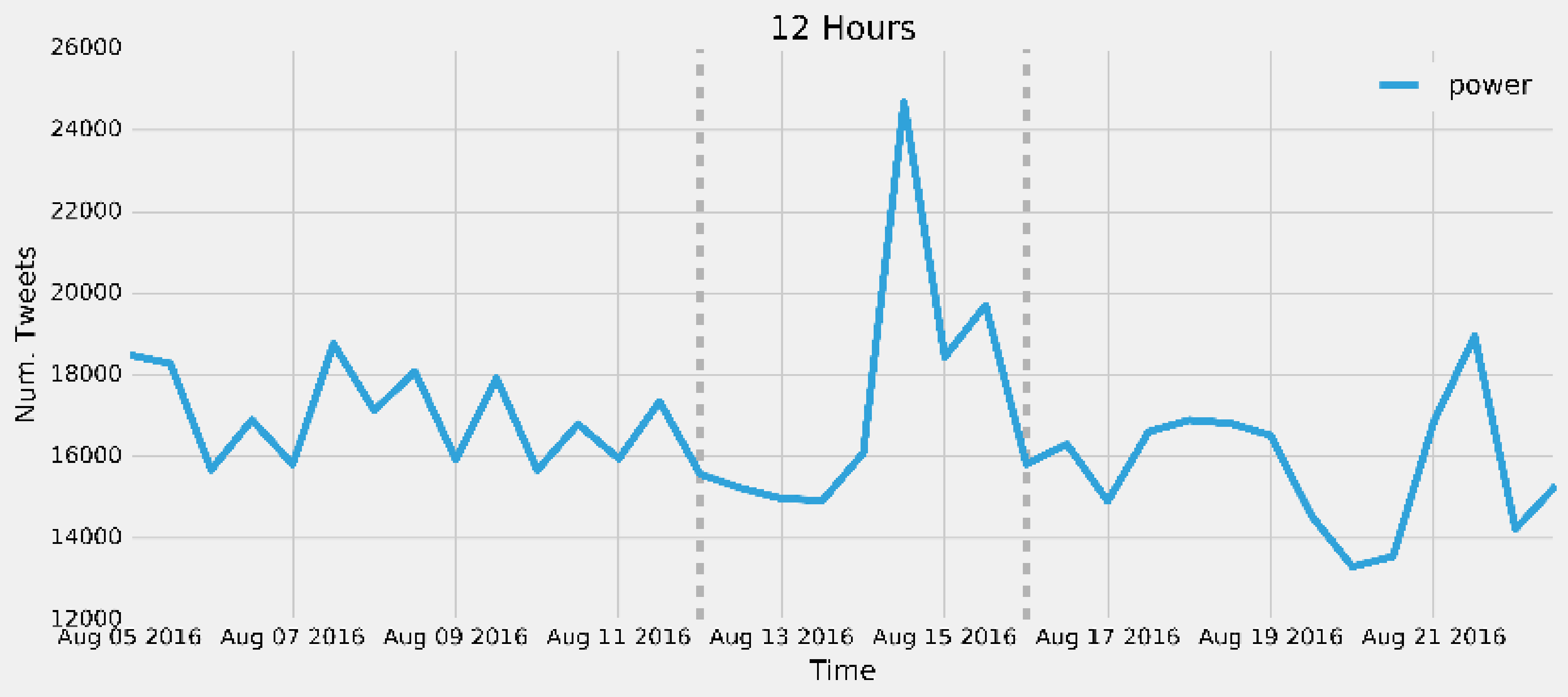

12 Hours
power
26000
24000
22000
20000
18000
16000
14000
12000
Num. Tweets
Aug 05 2016
Aug 07 2016
Aug 09 2016
Aug 11 2016
Aug 13 2016
Aug 15 2016
Aug 17 2016
Aug 19 2016
Aug 21 2016
Time

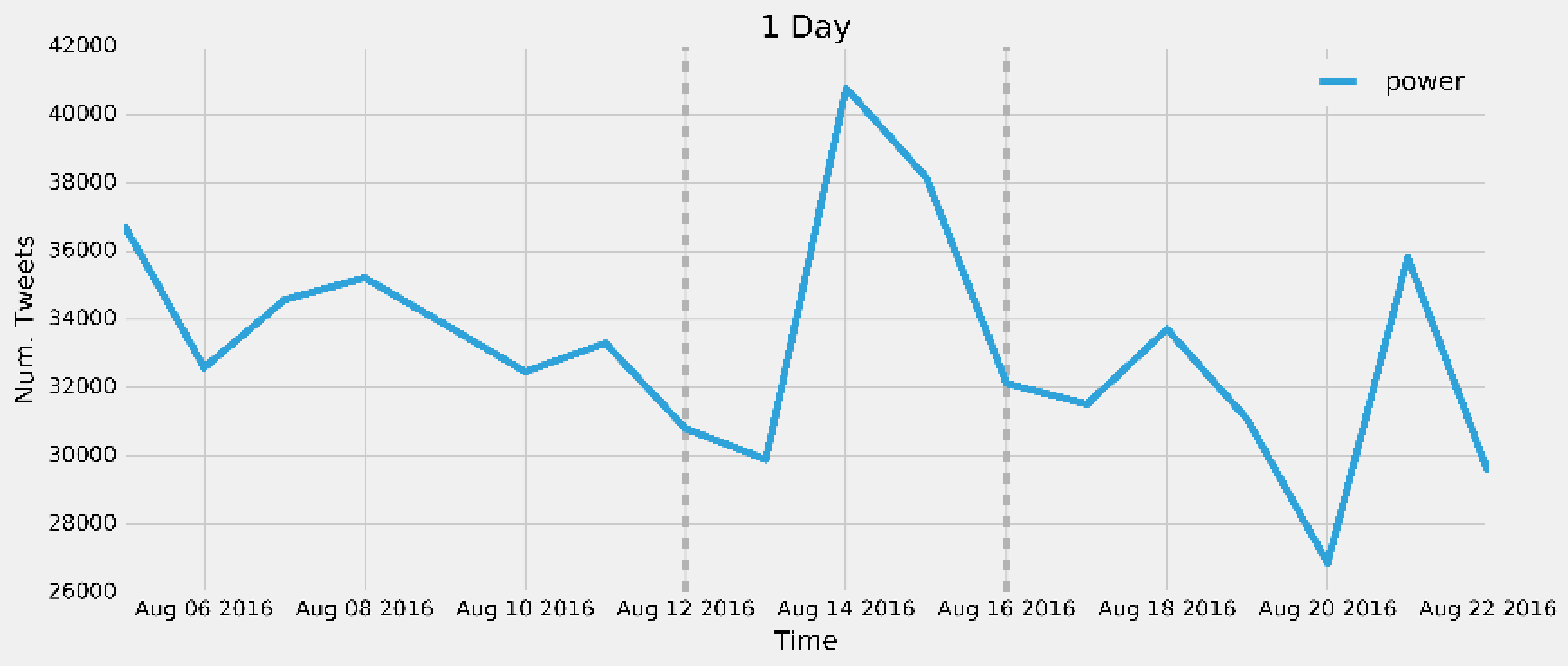
1 Day
power
Num. Tweets
42000
40000
38000
36000
34000
32000
30000
28000
26000
Aug 06 2016
Aug 08 2016
Aug 10 2016
Aug 12 2016
Aug 14 2016
Aug 16 2016
Aug 18 2016
Aug 20 2016
Aug 22 2016
Time

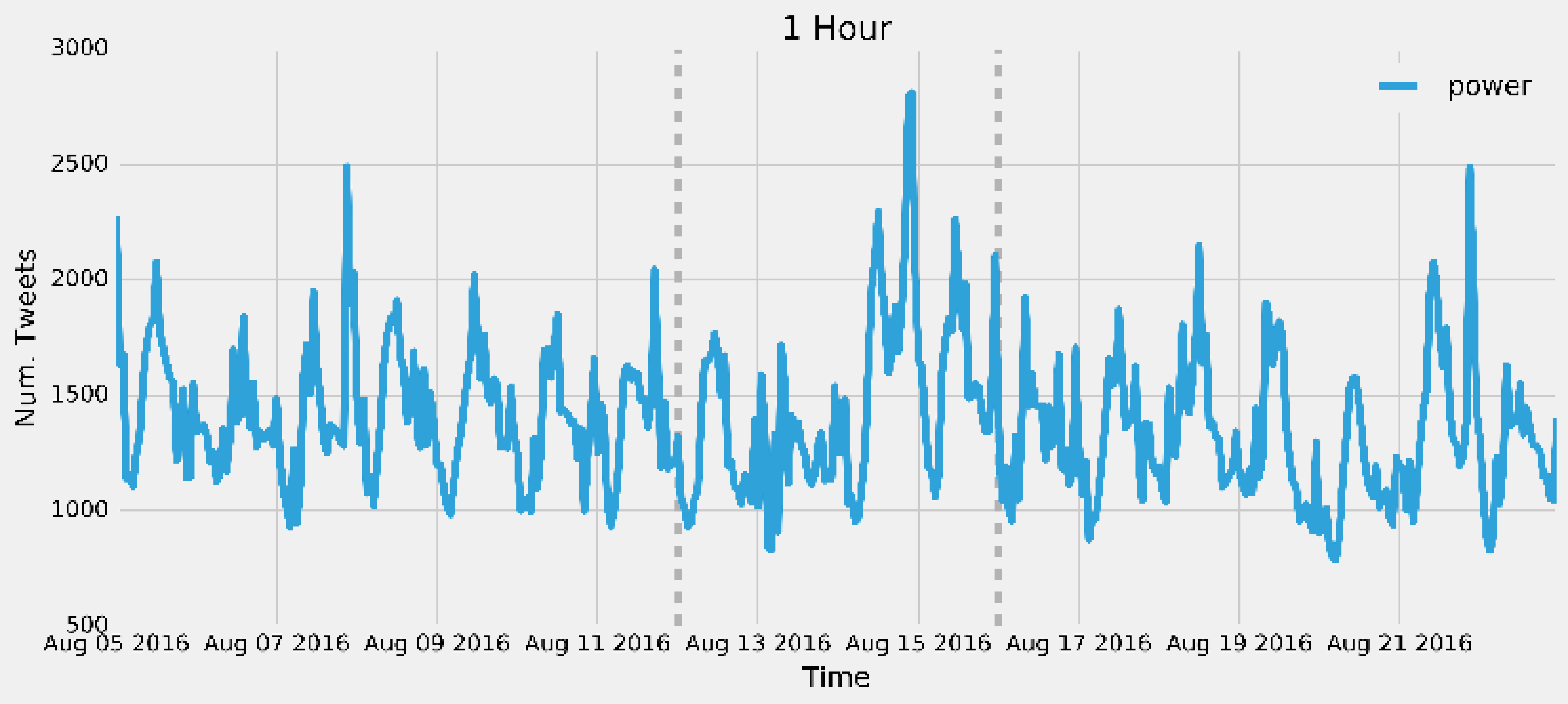

1 Hour
power
Num. Tweets
3000
2500
2000
1500
1000
500
Aug 05 2016
Aug 07 2016
Aug 09 2016
Aug 11 2016
Aug 13 2016
Aug 15 2016
Aug 17 2016
Aug 19 2016
Aug 21 2016
Time

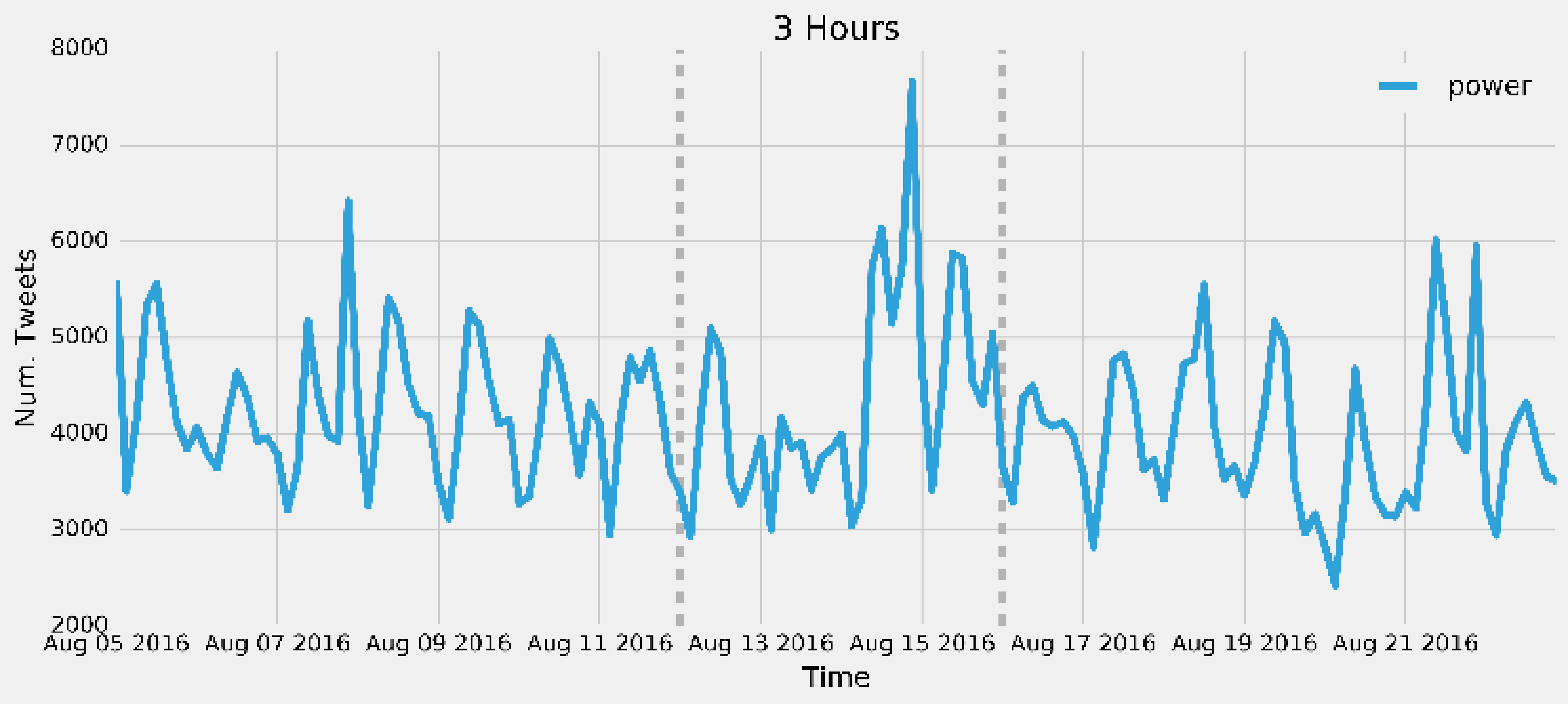

3 Hours
power
8000
7000
6000
5000
4000
3000
2000
Num. Tweets
Aug 05 2016
Aug 07 2016
Aug 09 2016
Aug 11 2016
Aug 13 2016
Aug 15 2016
Aug 17 2016
Aug 19 2016
Aug 21 2016
Time

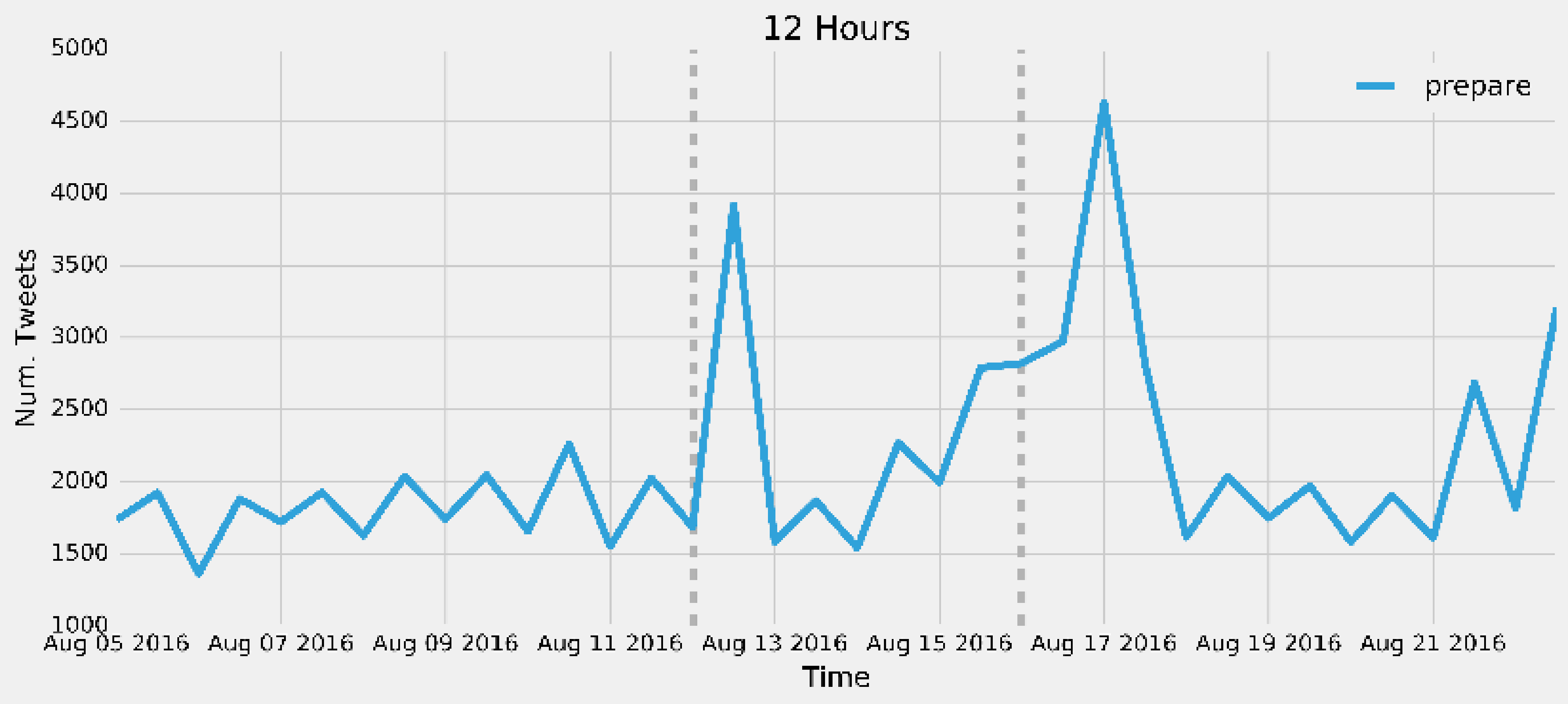

12 Hours
prepare
Num. Tweets
1000
1500
2000
2500
3000
3500
4000
4500
5000
Aug 05 2016
Aug 07 2016
Aug 09 2016
Aug 11 2016
Aug 13 2016
Aug 15 2016
Aug 17 2016
Aug 19 2016
Aug 21 2016
Time

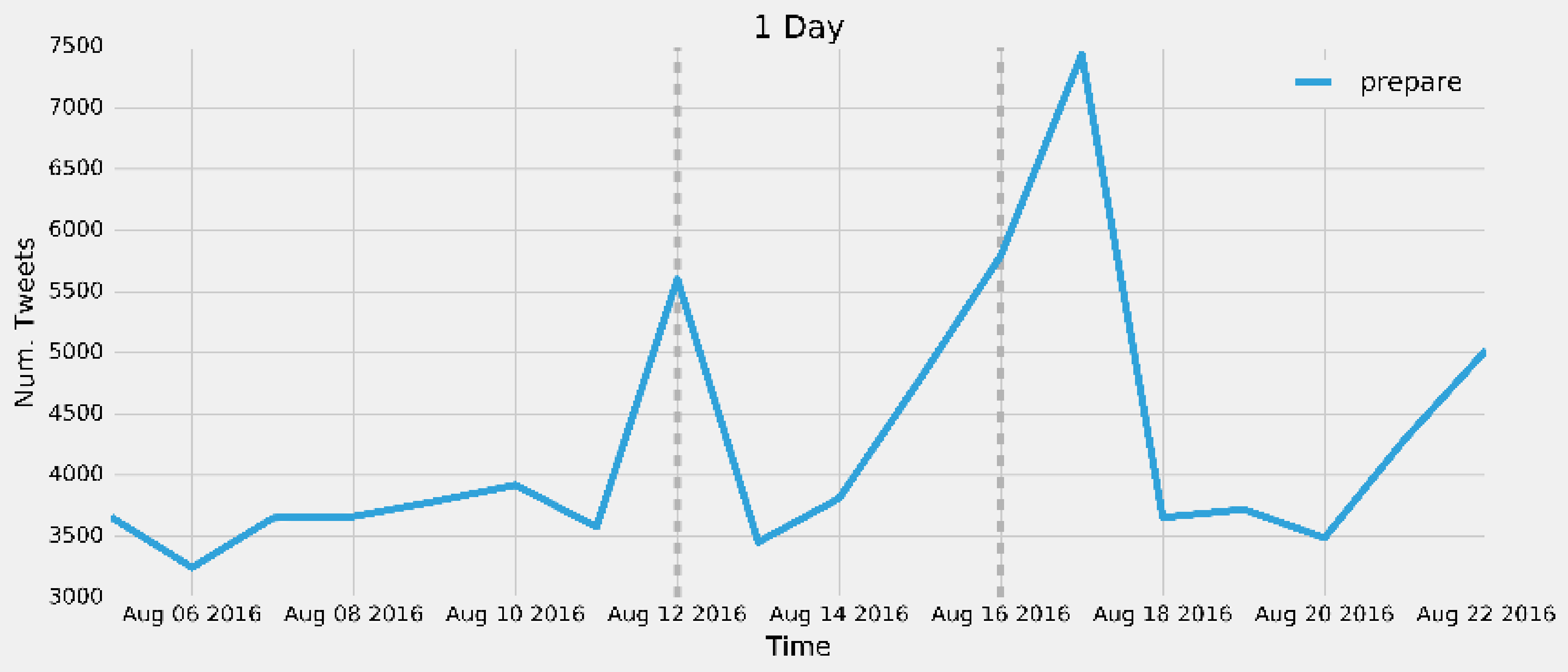
1 Day
prepare
Num. Tweets
7500
7000
6500
6000
5500
5000
4500
4000
3500
3000
Aug 06 2016
Aug 08 2016
Aug 10 2016
Aug 12 2016
Aug 14 2016
Aug 16 2016
Aug 18 2016
Aug 20 2016
Aug 22 2016
Time

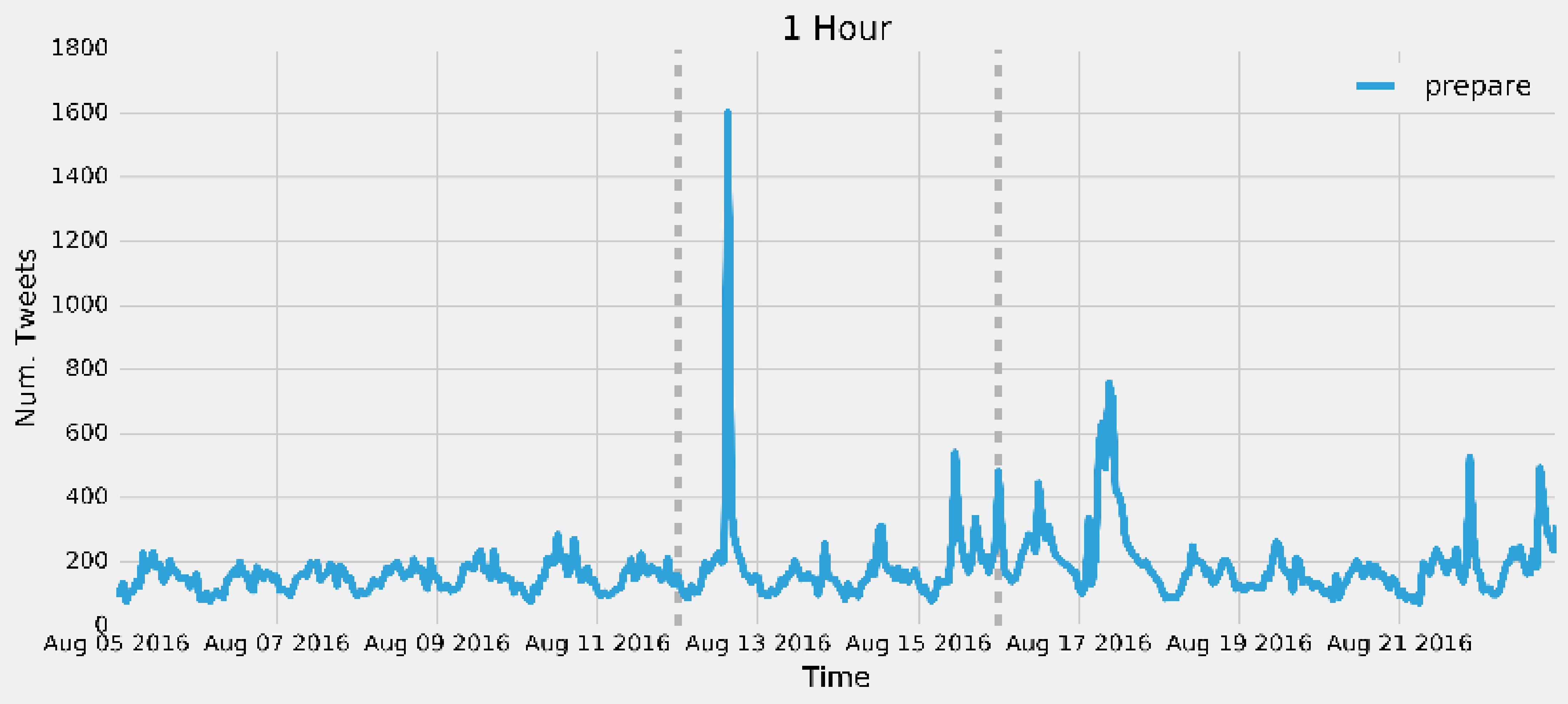
1 Hour
Num. Tweets
1800
1600
1400
1200
1000
800
600
400
200
0
Aug 05 2016
Aug 07 2016
Aug 09 2016
Aug 11 2016
Aug 13 2016
Aug 15 2016
Aug 17 2016
Aug 19 2016
Aug 21 2016
Time
prepare

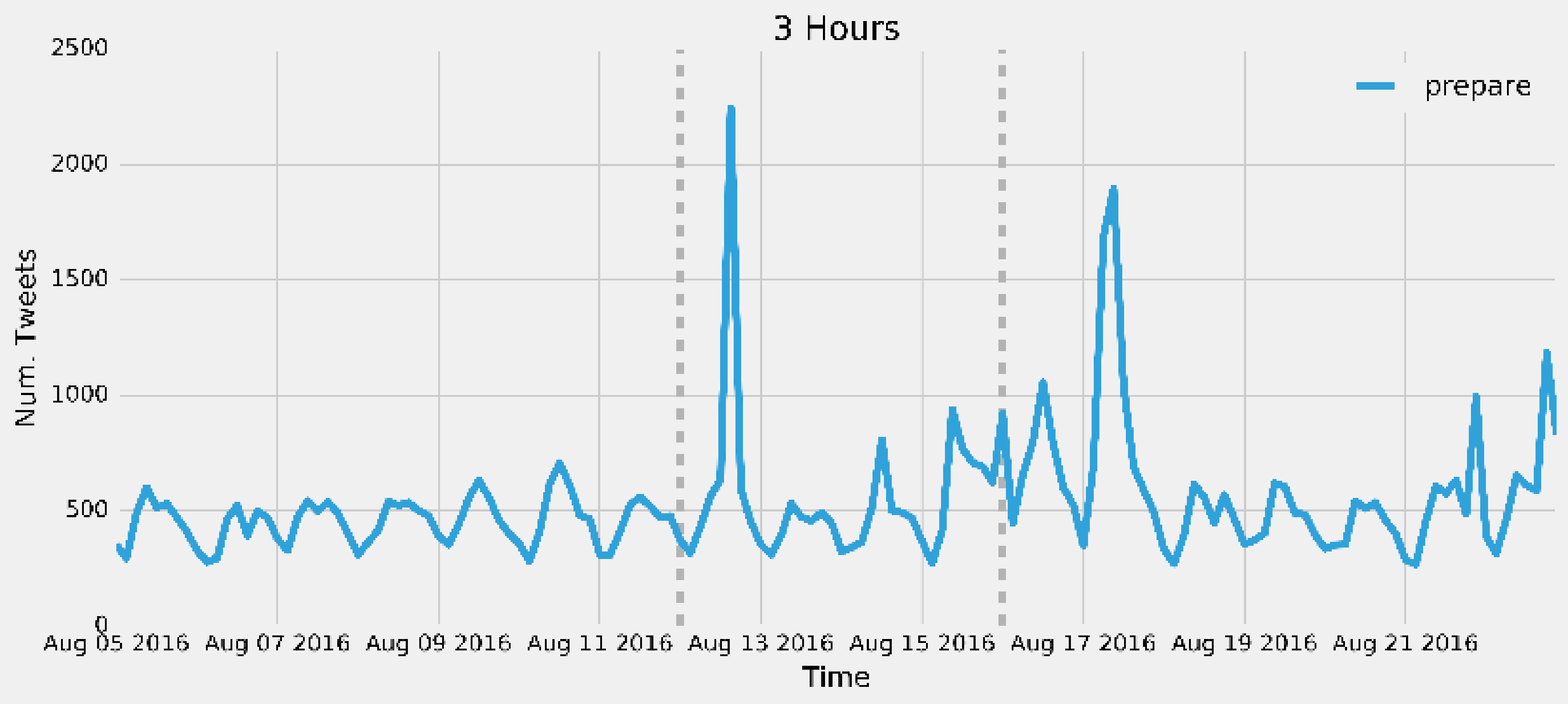
3 Hours
prepare
Num. Tweets
2500
2000
1500
1000
500
0
Aug 05 2016
Aug 07 2016
Aug 09 2016
Aug 11 2016
Aug 13 2016
Aug 15 2016
Aug 17 2016
Aug 19 2016
Aug 21 2016
Time

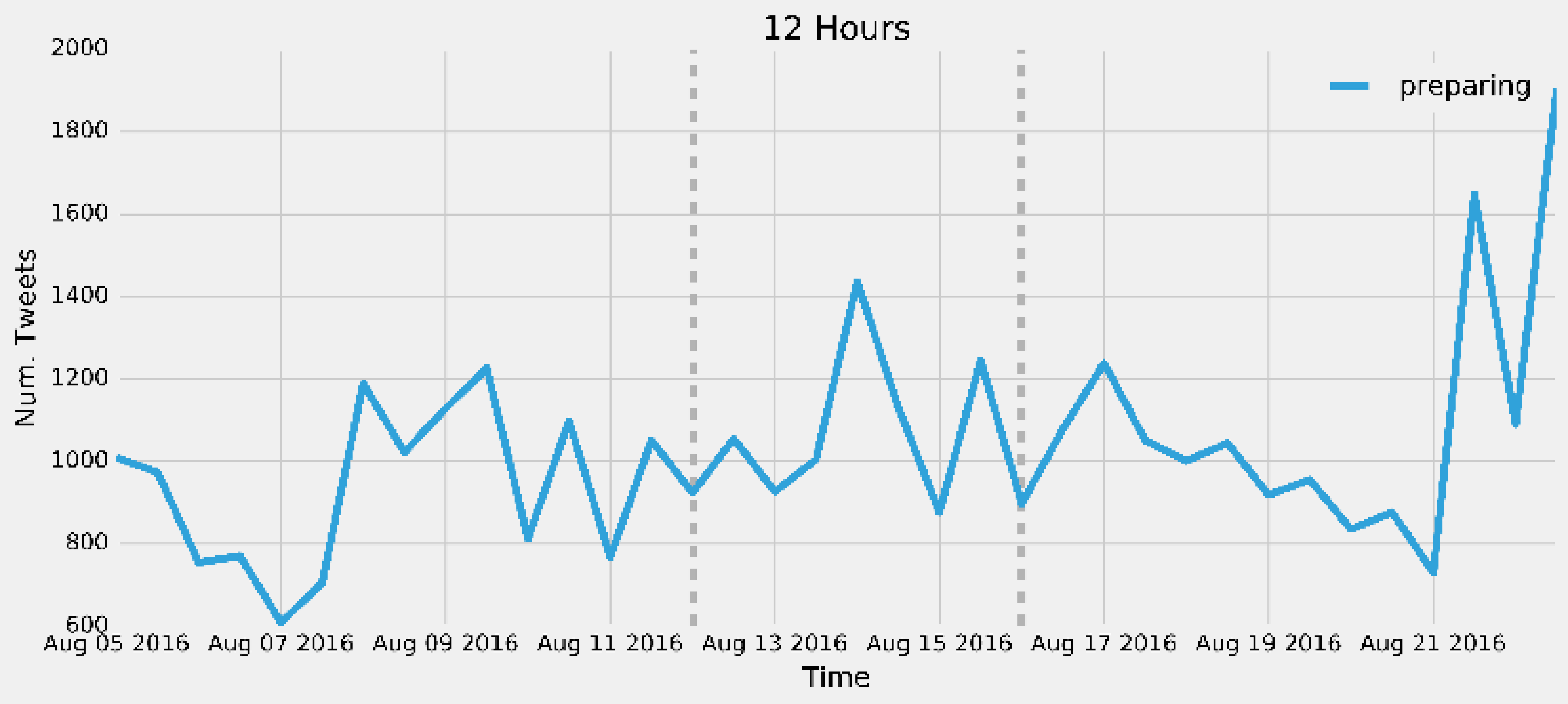

12 Hours
2000
1800
1600
1400
1200
1000
800
600
Num. Tweets
preparing
Aug 05 2016
Aug 07 2016
Aug 09 2016
Aug 11 2016
Aug 13 2016
Aug 15 2016
Aug 17 2016
Aug 19 2016
Aug 21 2016
Time

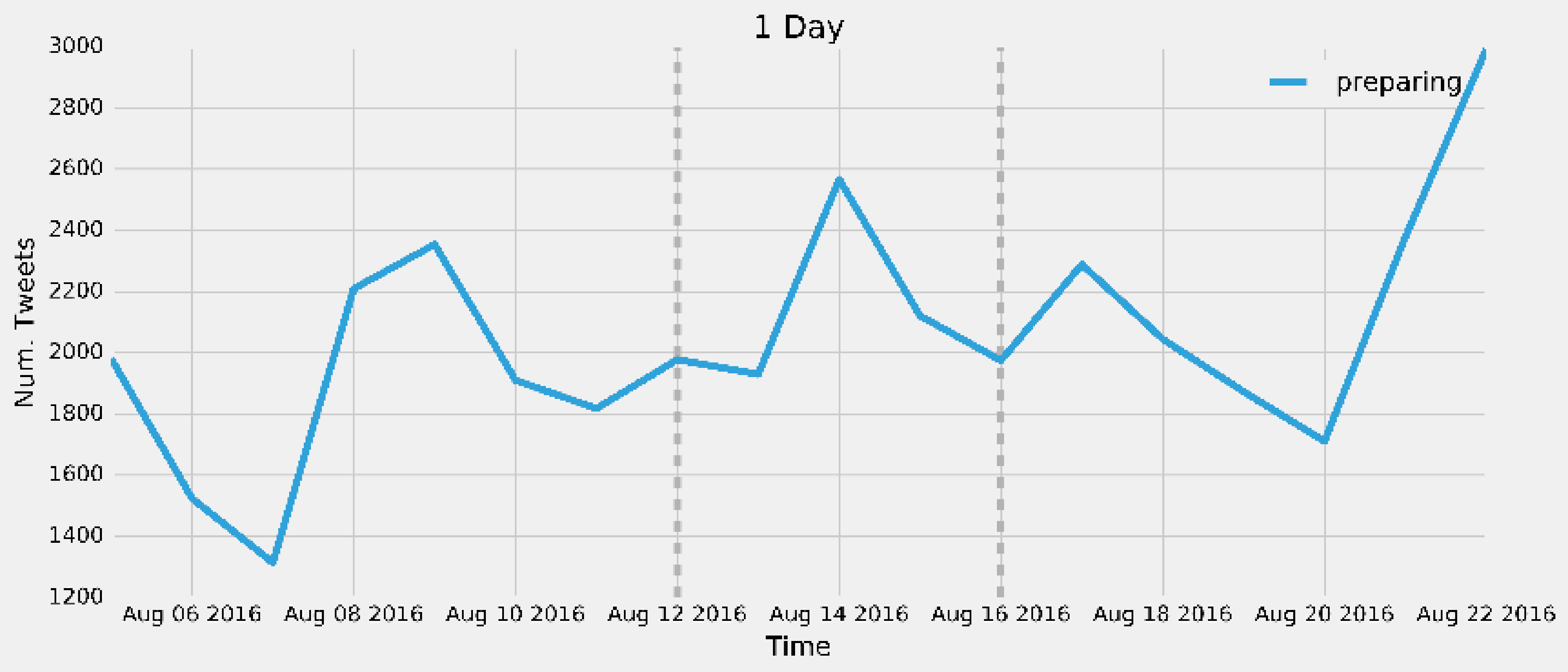

1 Day
preparing
Num. Tweets
3000
2800
2600
2400
2200
2000
1800
1600
1400
1200
Aug 06 2016
Aug 08 2016
Aug 10 2016
Aug 12 2016
Aug 14 2016
Aug 16 2016
Aug 18 2016
Aug 20 2016
Aug 22 2016
Time

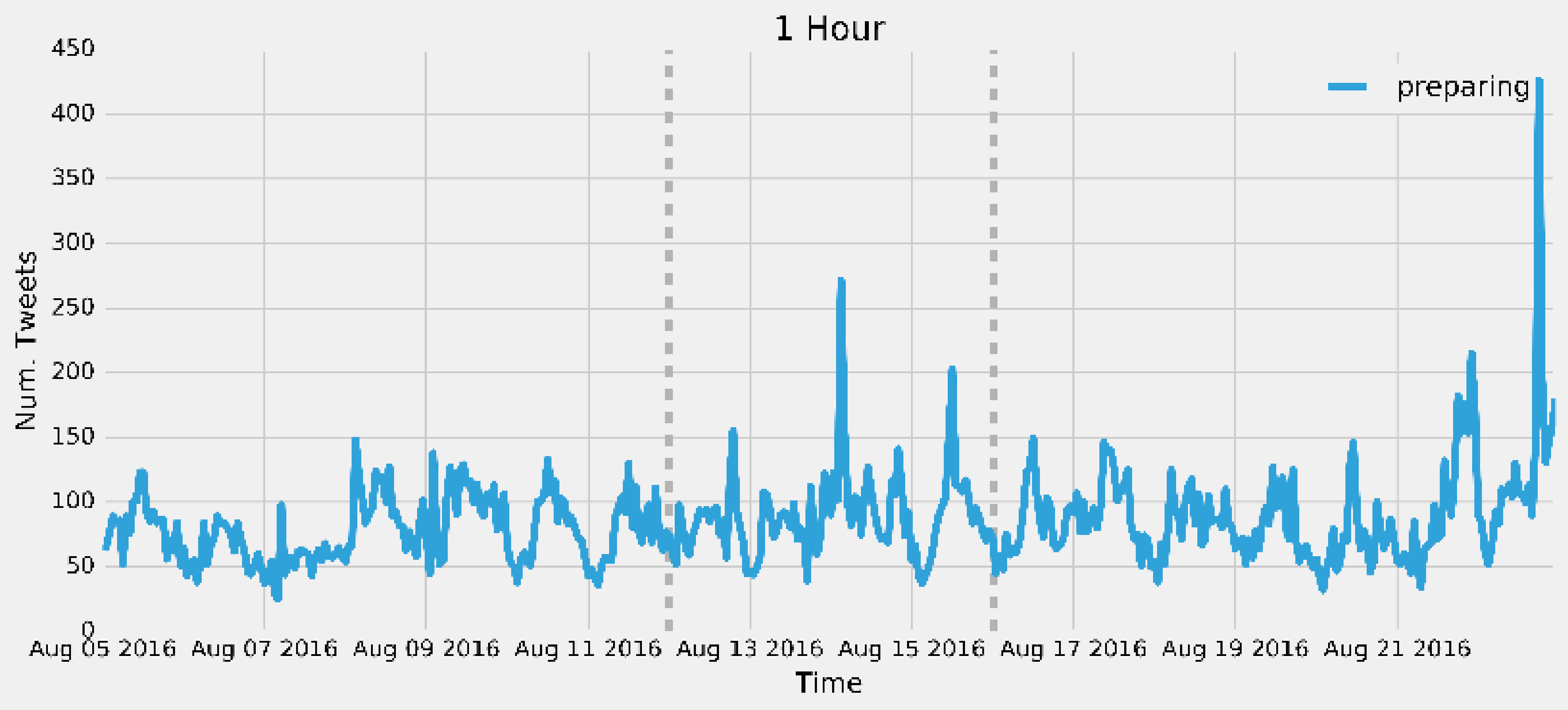

1 Hour
preparing
450
400
350
300
250
200
150
100
50
0
Num. Tweets
Aug 05 2016
Aug 07 2016
Aug 09 2016
Aug 11 2016
Aug 13 2016
Aug 15 2016
Aug 17 2016
Aug 19 2016
Aug 21 2016
Time

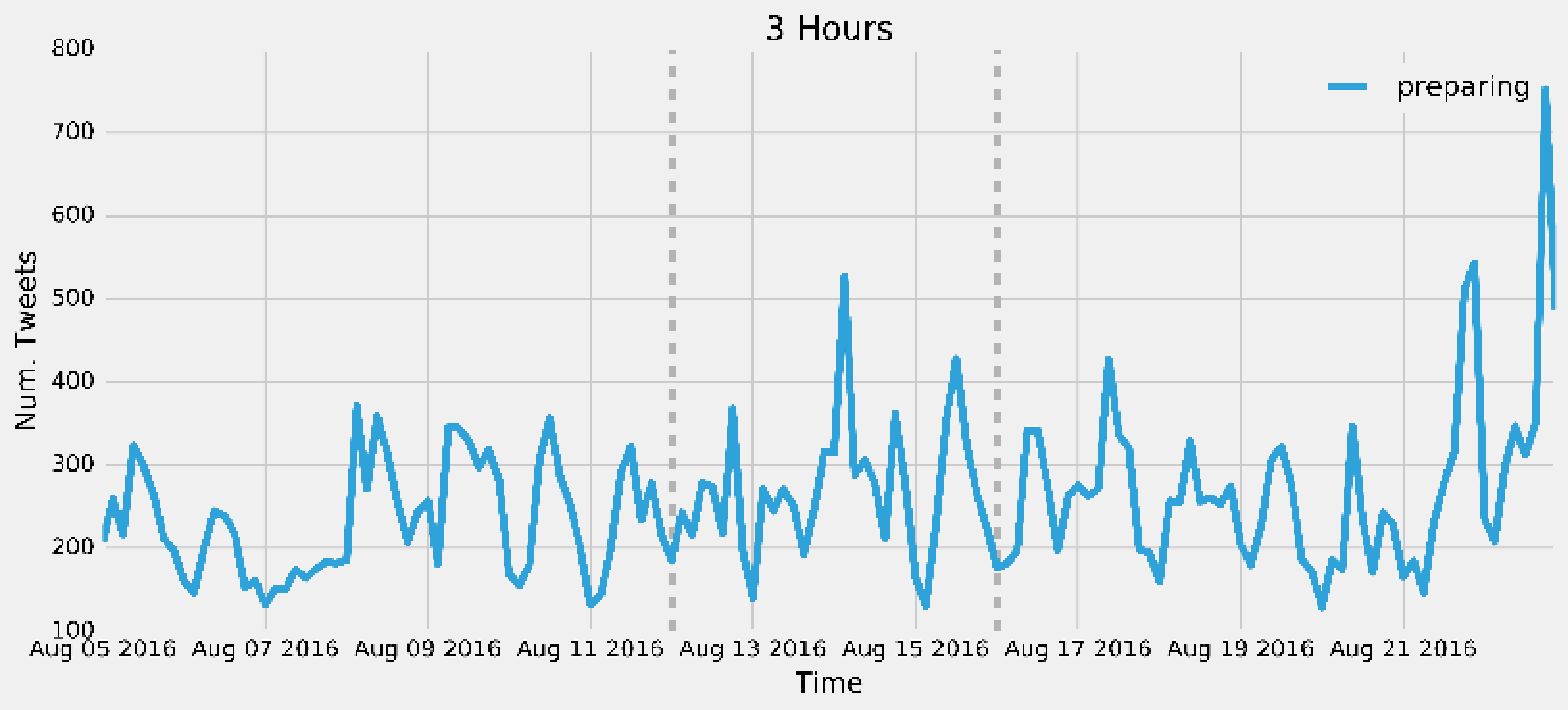

3 Hours
preparing
Num. Tweets
800
700
600
500
400
300
200
100
Aug 05 2016
Aug 07 2016
Aug 09 2016
Aug 11 2016
Aug 13 2016
Aug 15 2016
Aug 17 2016
Aug 19 2016
Aug 21 2016
Time

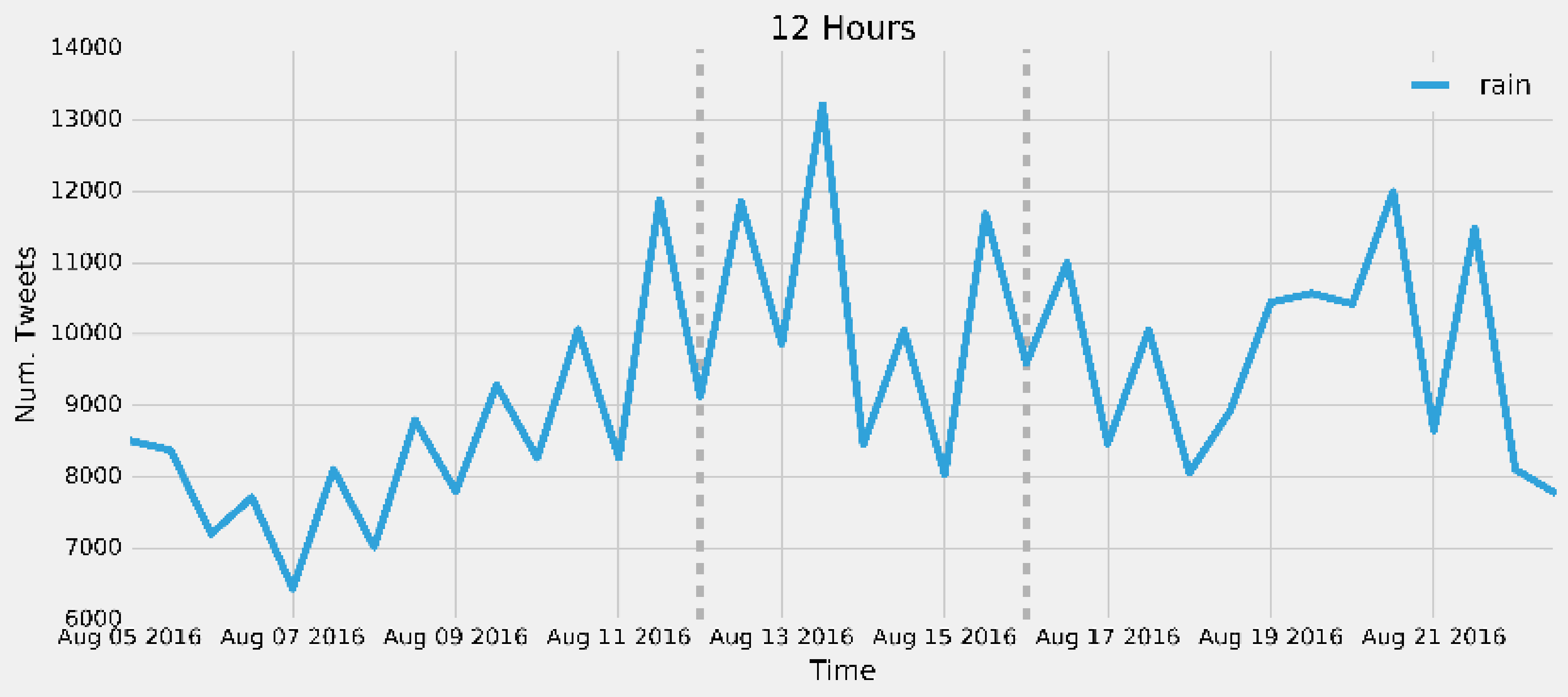
12 Hours
rain
Num. Tweets
14000
13000
12000
11000
10000
9000
8000
7000
6000
Aug 05 2016
Aug 07 2016
Aug 09 2016
Aug 11 2016
Aug 13 2016
Aug 15 2016
Aug 17 2016
Aug 19 2016
Aug 21 2016
Time

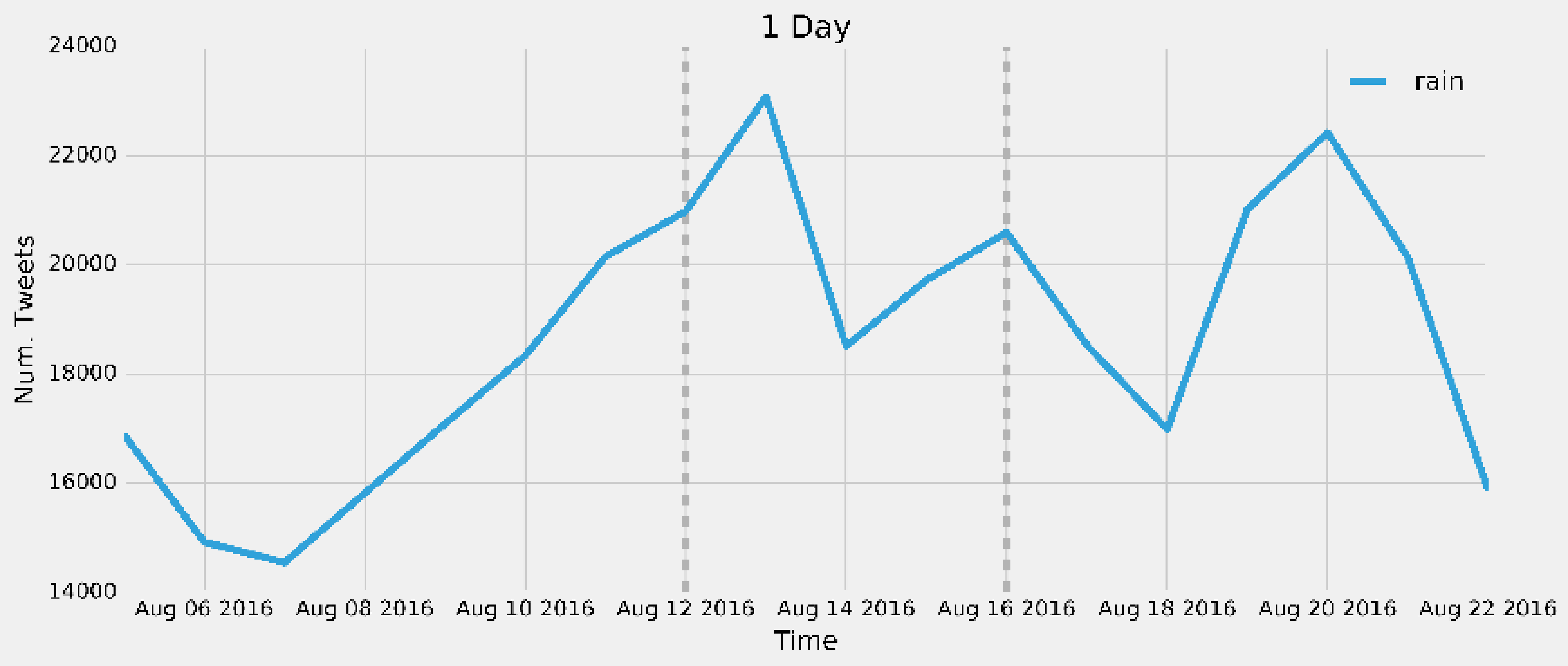
1 Day
rain
Num. Tweets
24000
22000
20000
18000
16000
14000
Aug 06 2016
Aug 08 2016
Aug 10 2016
Aug 12 2016
Aug 14 2016
Aug 16 2016
Aug 18 2016
Aug 20 2016
Aug 22 2016
Time

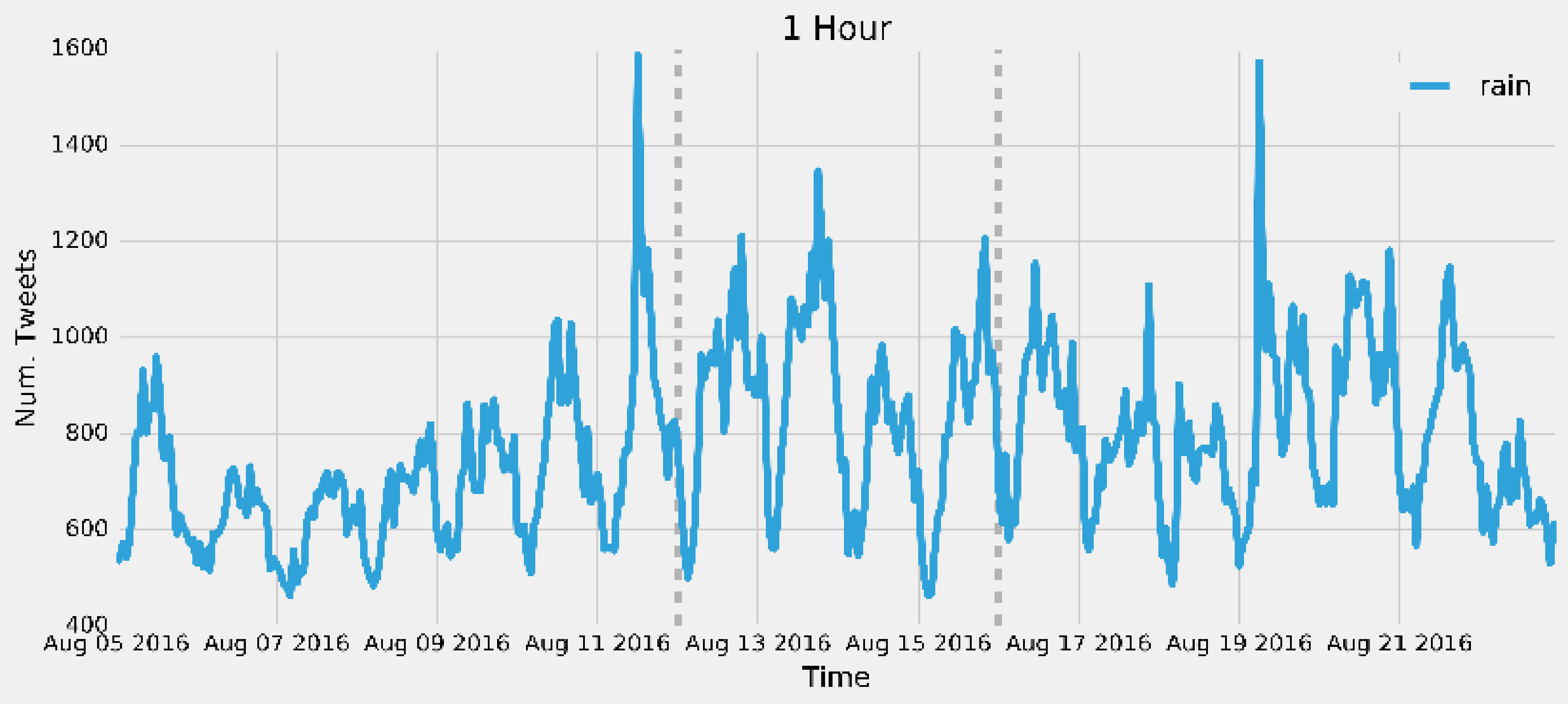
1 Hour
rain
Num. Tweets
Time
1600
1400
1200
1000
800
600
400
Aug 05 2016
Aug 07 2016
Aug 09 2016
Aug 11 2016
Aug 13 2016
Aug 15 2016
Aug 17 2016
Aug 19 2016
Aug 21 2016

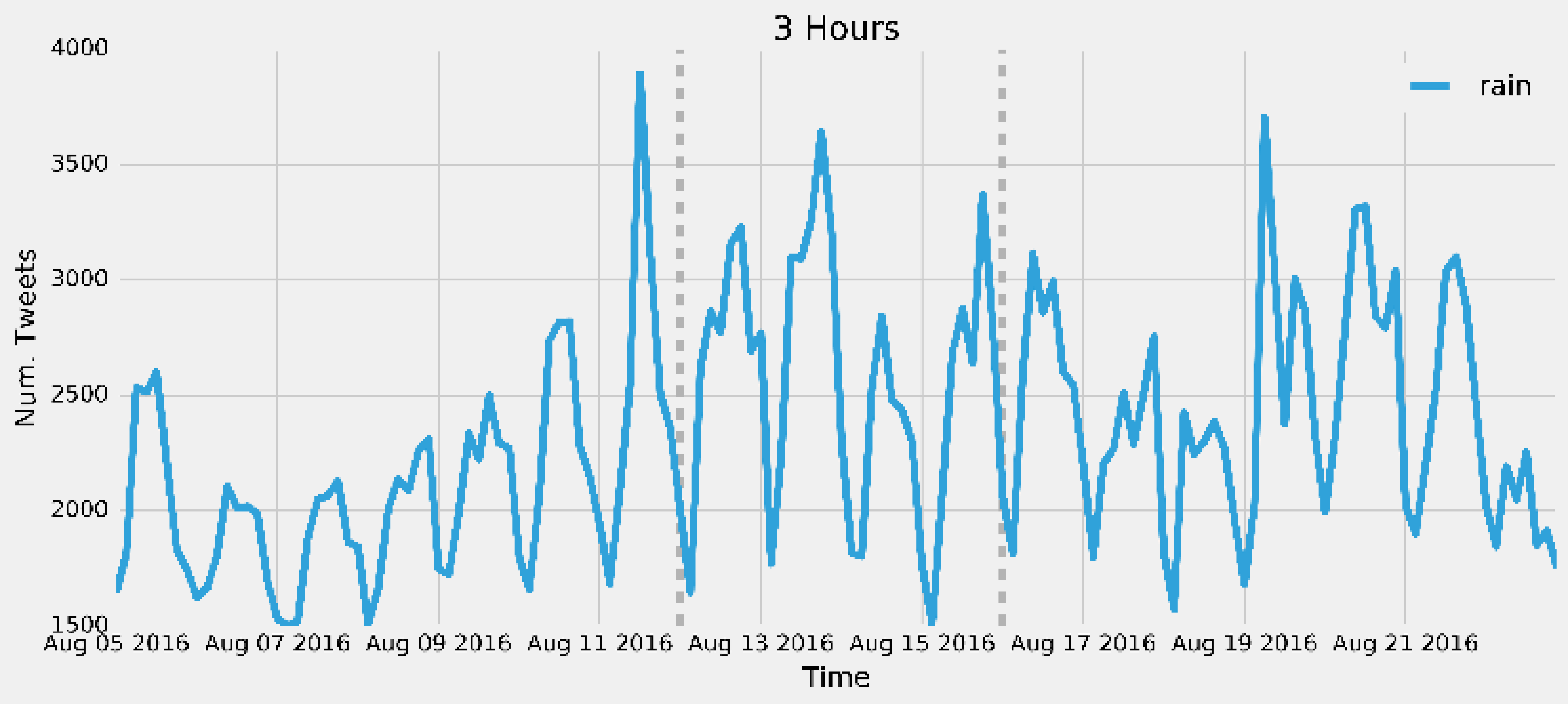
3 Hours
4000
3500
3000
2500
2000
1500
Num. Tweets
rain
Aug 05 2016
Aug 07 2016
Aug 09 2016
Aug 11 2016
Aug 13 2016
Aug 15 2016
Aug 17 2016
Aug 19 2016
Aug 21 2016
Time

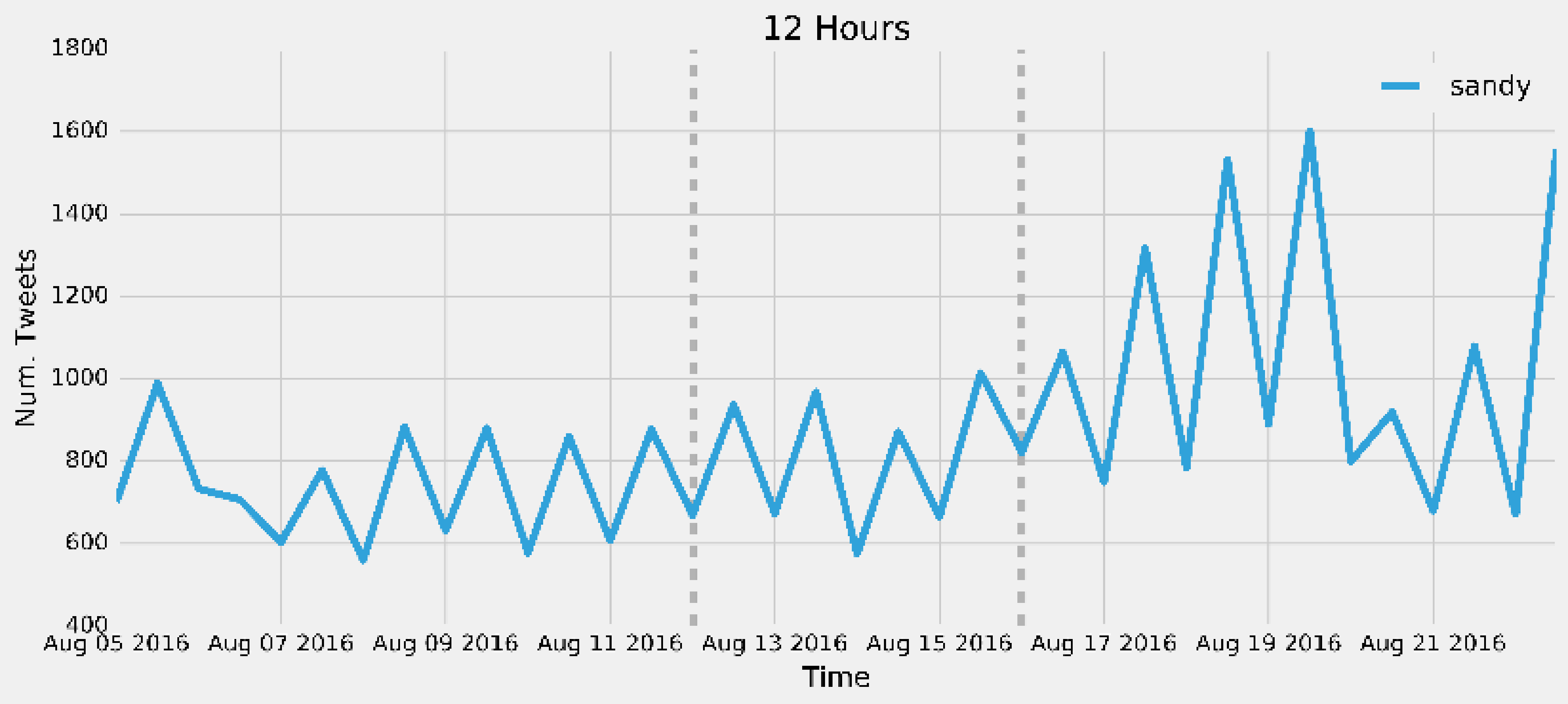
12 Hours
sandy
Num. Tweets
Time
1800
1600
1400
1200
1000
800
600
400
Aug 05 2016
Aug 07 2016
Aug 09 2016
Aug 11 2016
Aug 13 2016
Aug 15 2016
Aug 17 2016
Aug 19 2016
Aug 21 2016

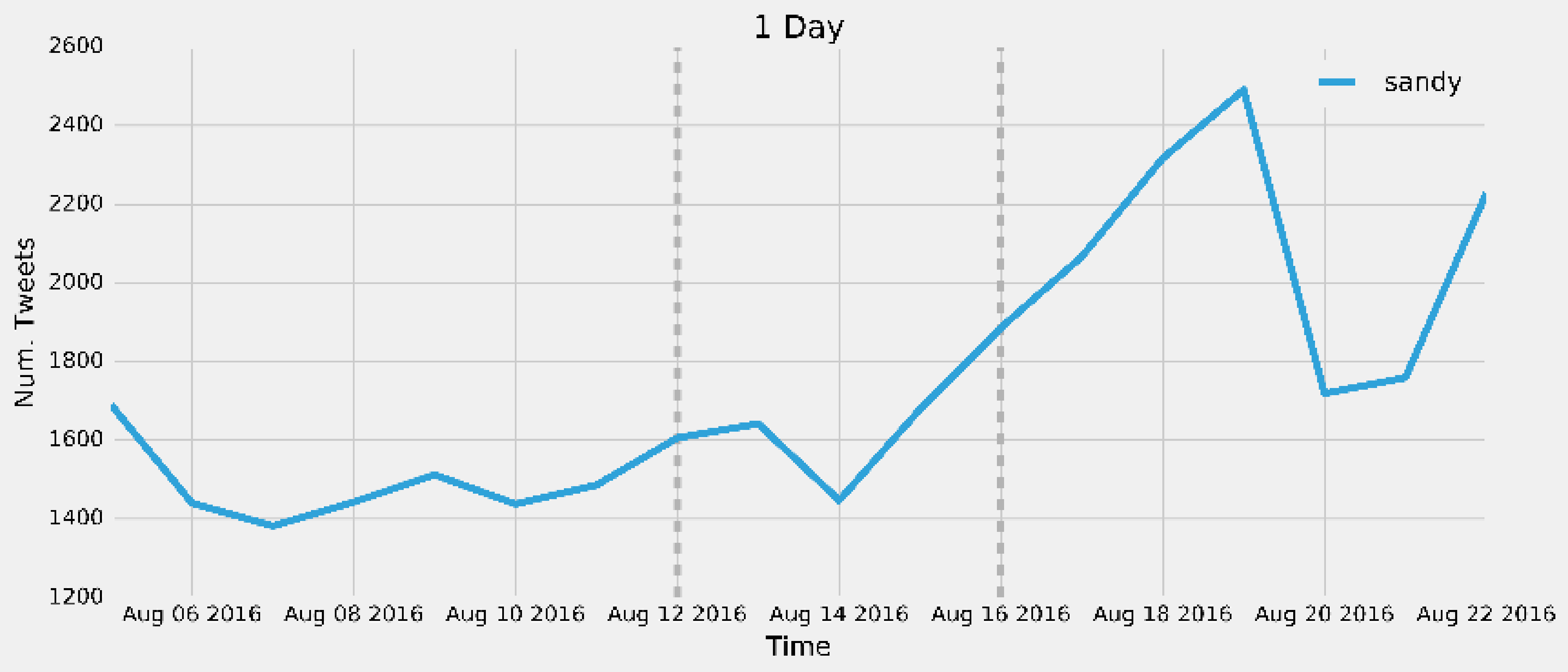
1 Day
sandy
Num. Tweets
Time
2600
2400
2200
2000
1800
1600
1400
1200
Aug 06 2016
Aug 08 2016
Aug 10 2016
Aug 12 2016
Aug 14 2016
Aug 16 2016
Aug 18 2016
Aug 20 2016
Aug 22 2016

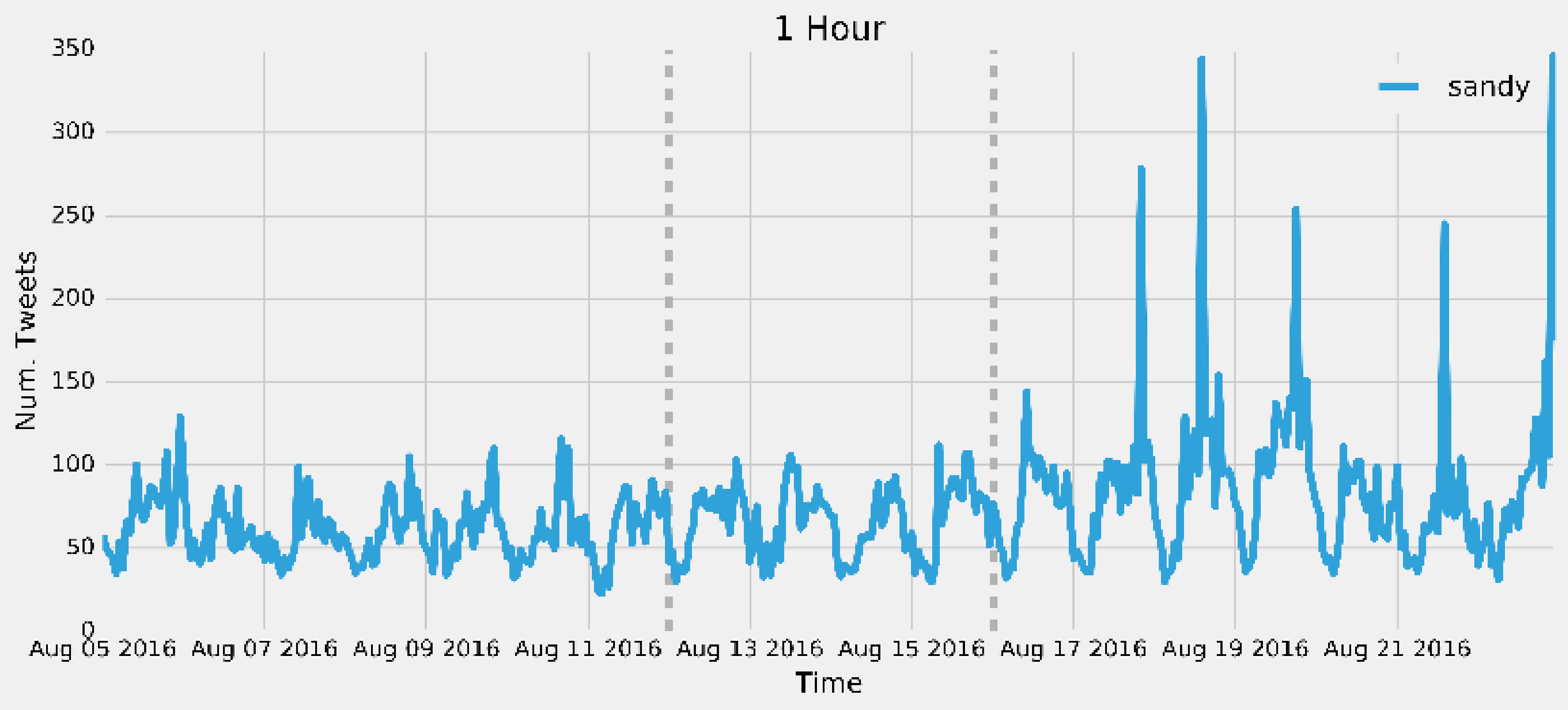
1 Hour
sandy
Num. Tweets
Time
0
50
100
150
200
250
300
350
Aug 05 2016
Aug 07 2016
Aug 09 2016
Aug 11 2016
Aug 13 2016
Aug 15 2016
Aug 17 2016
Aug 19 2016
Aug 21 2016

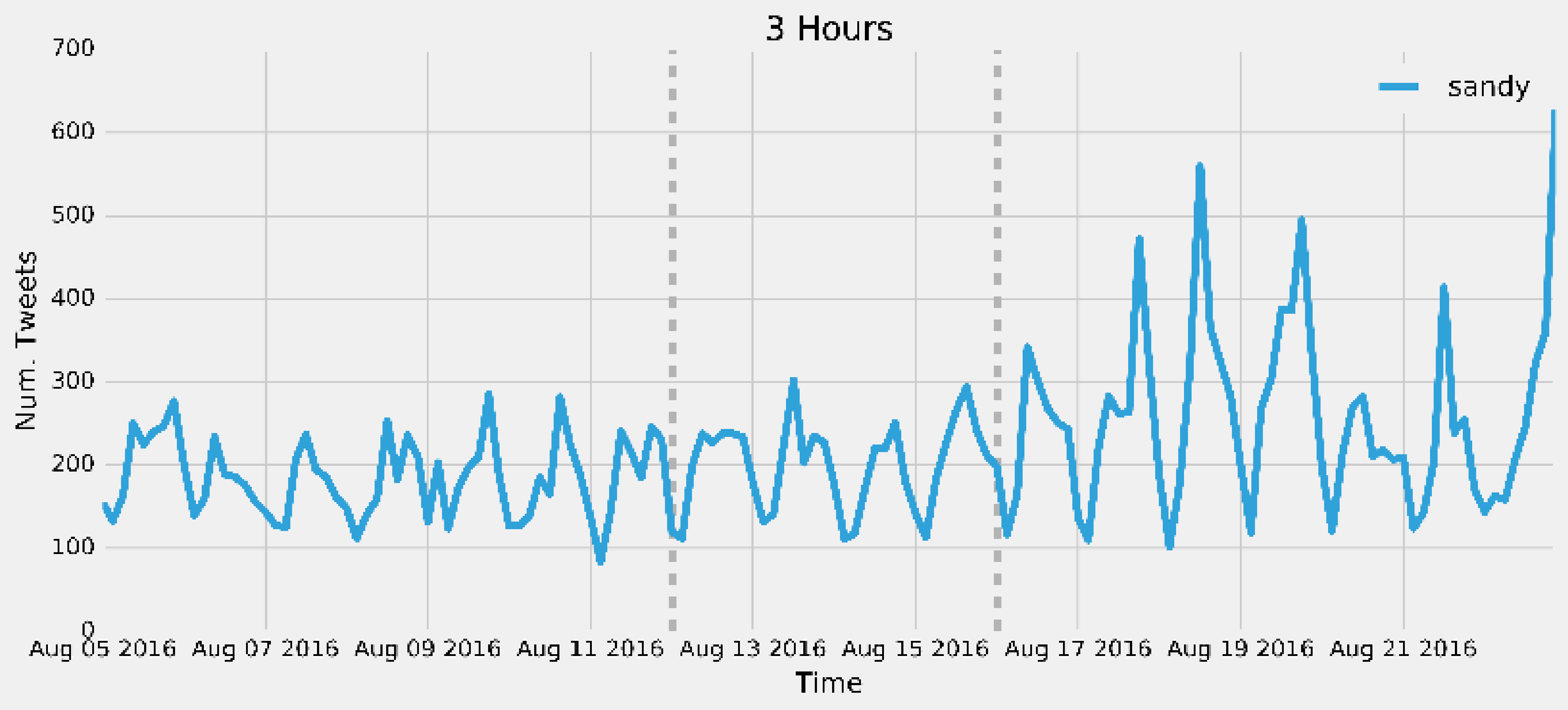

3 Hours
sandy
Num. Tweets
0
100
200
300
400
500
600
700
Aug 05 2016
Aug 07 2016
Aug 09 2016
Aug 11 2016
Aug 13 2016
Aug 15 2016
Aug 17 2016
Aug 19 2016
Aug 21 2016
Time

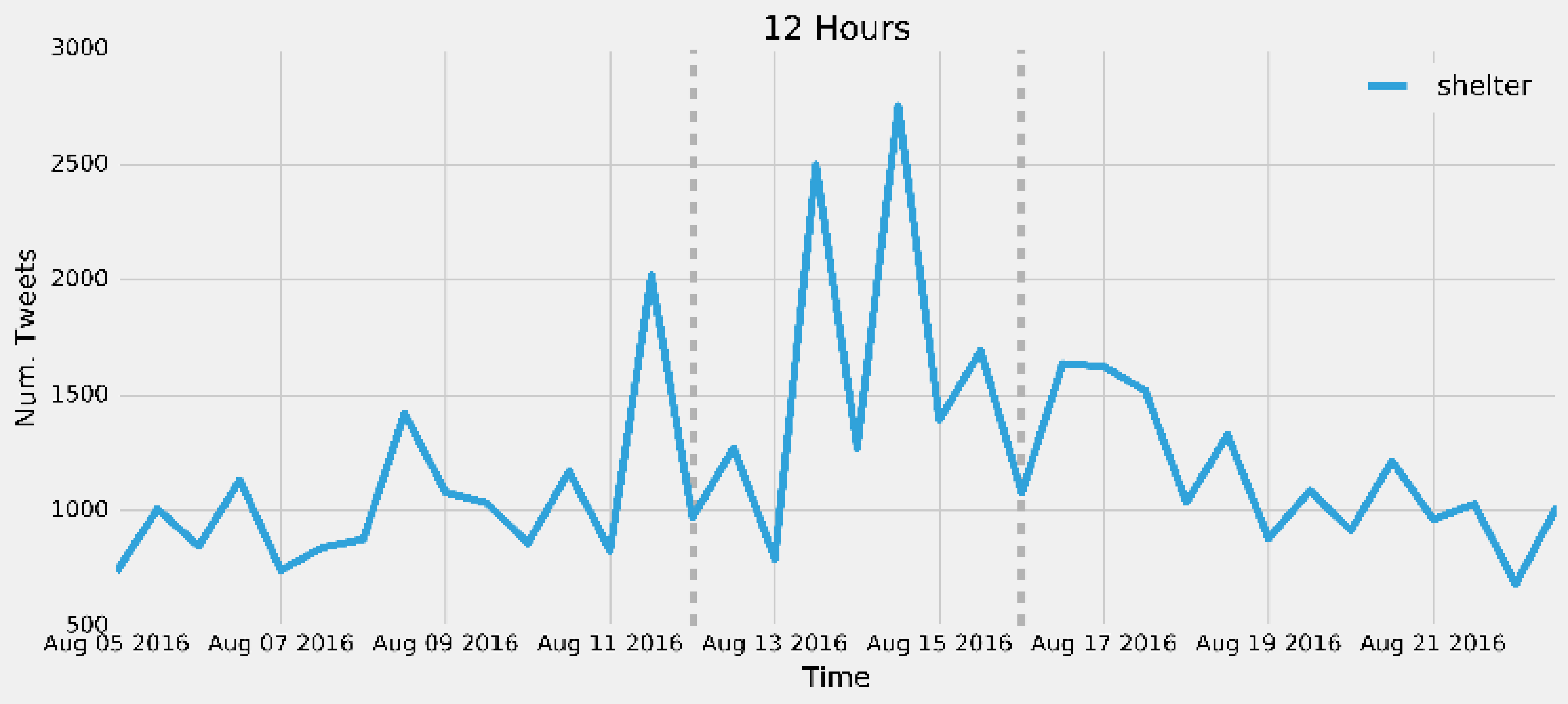
12 Hours
3000
2500
2000
1500
1000
500
Num. Tweets
shelter
Aug 05 2016
Aug 07 2016
Aug 09 2016
Aug 11 2016
Aug 13 2016
Aug 15 2016
Aug 17 2016
Aug 19 2016
Aug 21 2016
Time

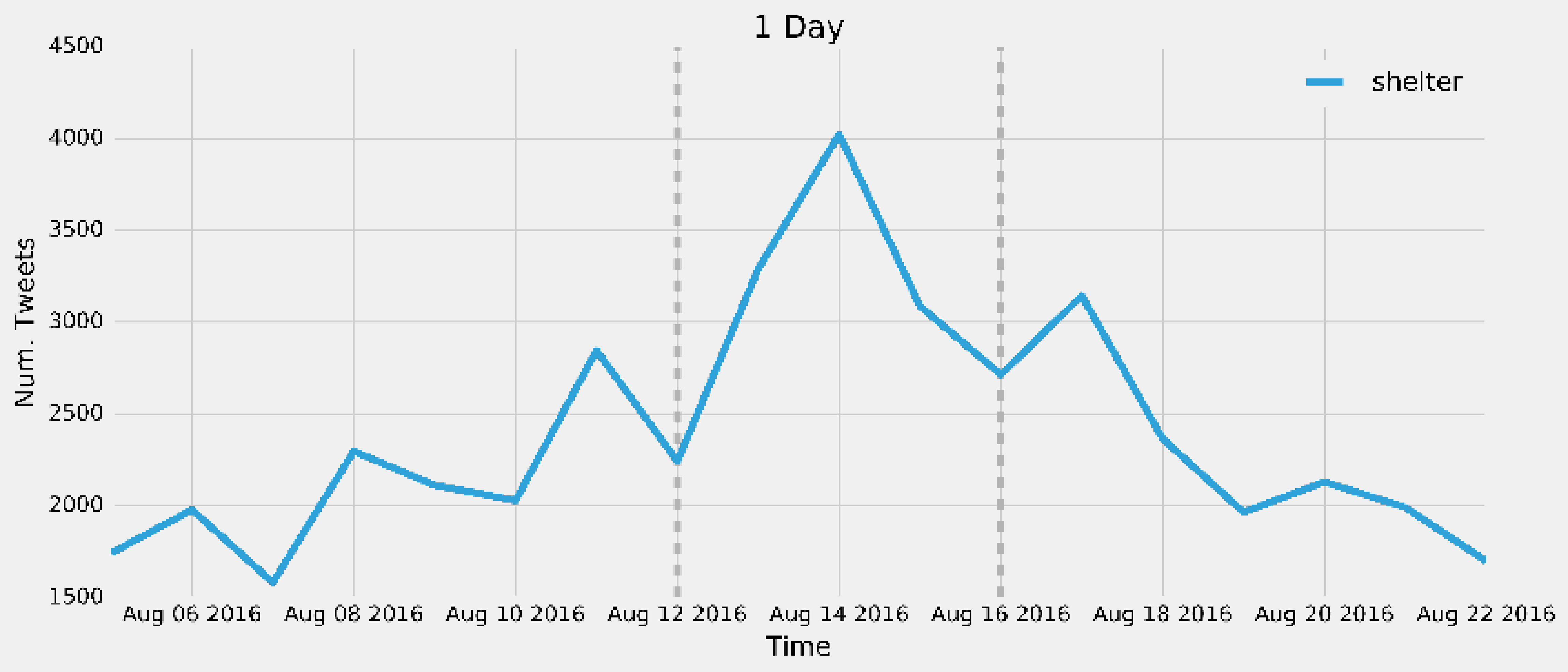
1 Day
shelter
Num. Tweets
4500
4000
3500
3000
2500
2000
1500
Aug 06 2016
Aug 08 2016
Aug 10 2016
Aug 12 2016
Aug 14 2016
Aug 16 2016
Aug 18 2016
Aug 20 2016
Aug 22 2016
Time

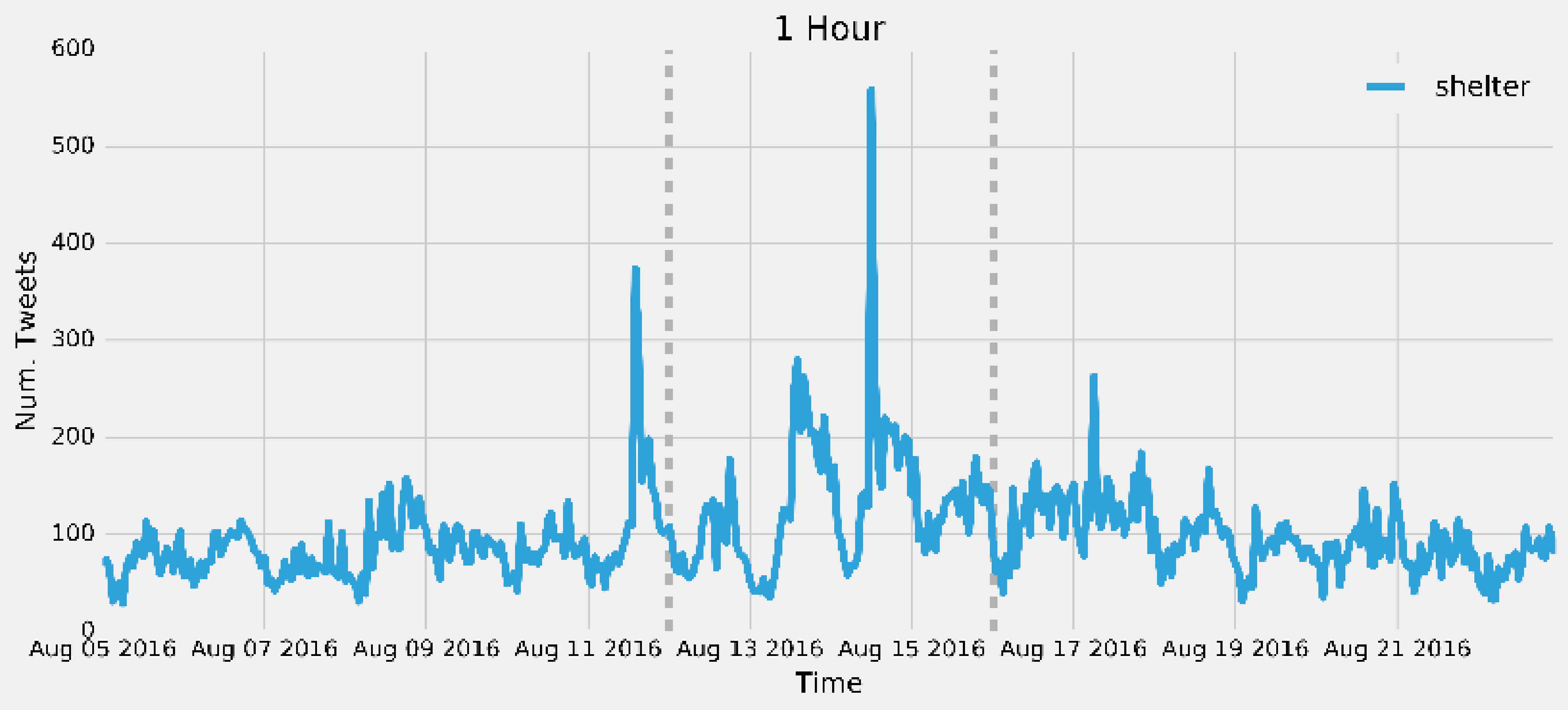
1 Hour
shelter
Num. Tweets
Time
0
100
200
300
400
500
600
Aug 05 2016
Aug 07 2016
Aug 09 2016
Aug 11 2016
Aug 13 2016
Aug 15 2016
Aug 17 2016
Aug 19 2016
Aug 21 2016

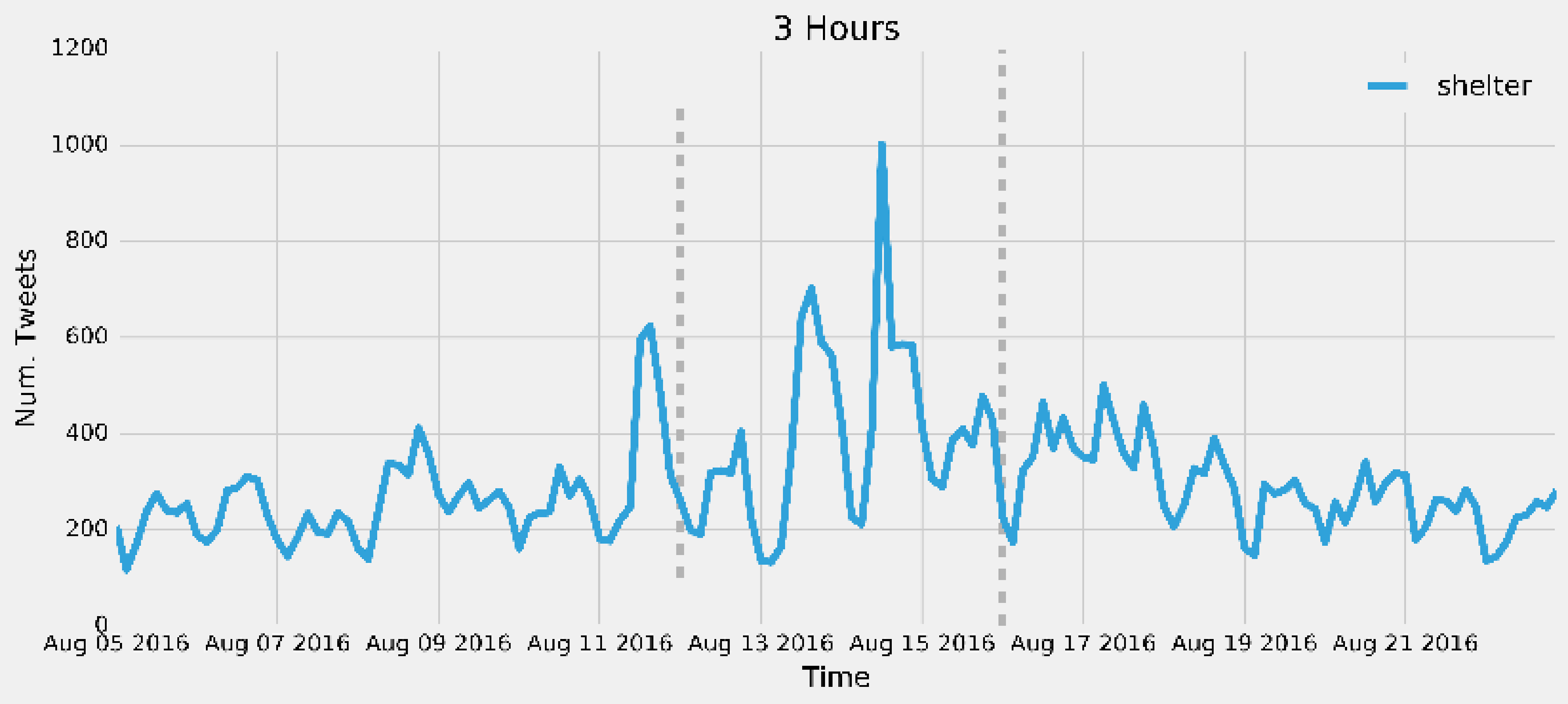

3 Hours
shelter
Num. Tweets
0
200
400
600
800
1000
1200
Aug 05 2016
Aug 07 2016
Aug 09 2016
Aug 11 2016
Aug 13 2016
Aug 15 2016
Aug 17 2016
Aug 19 2016
Aug 21 2016
Time

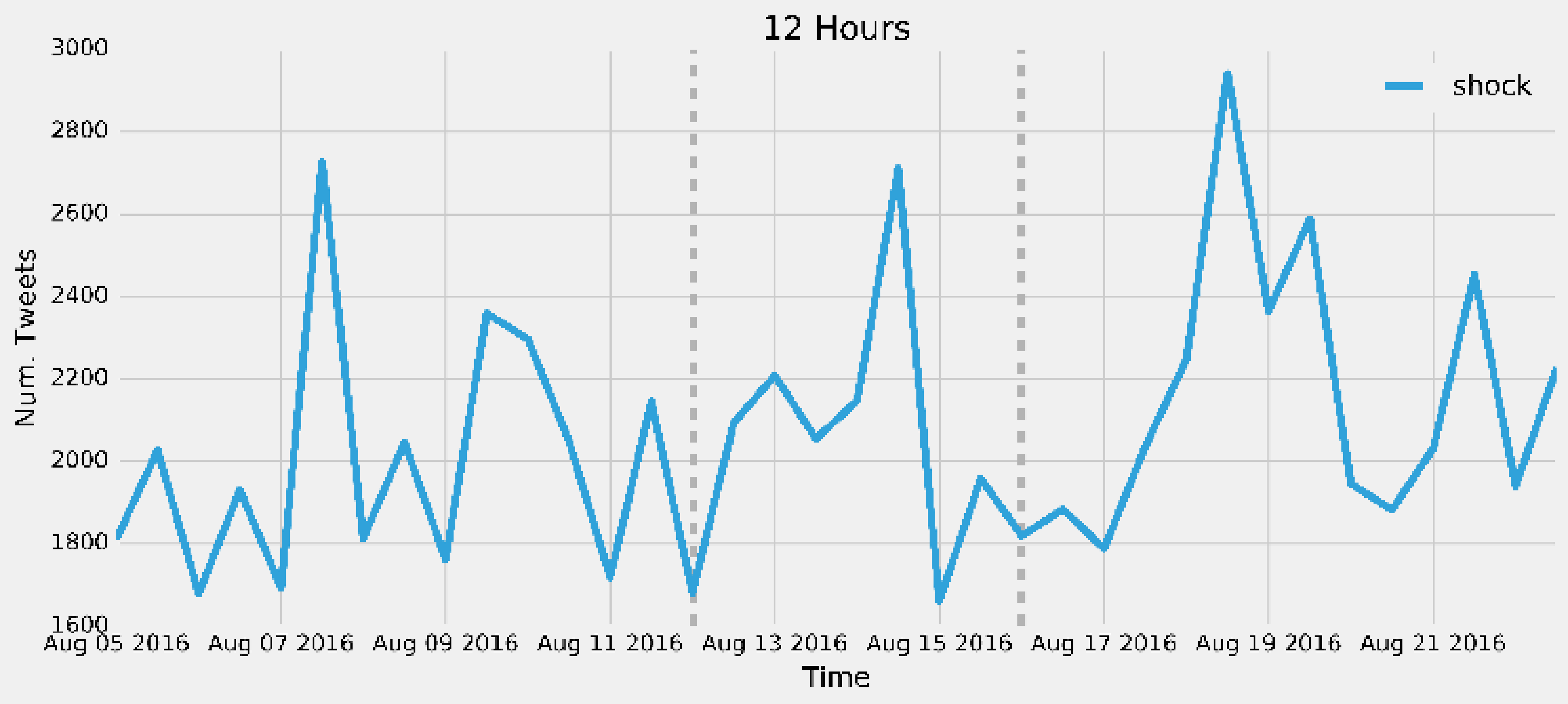

12 Hours
shock
Num. Tweets
3000
2800
2600
2400
2200
2000
1800
1600
Aug 05 2016
Aug 07 2016
Aug 09 2016
Aug 11 2016
Aug 13 2016
Aug 15 2016
Aug 17 2016
Aug 19 2016
Aug 21 2016
Time

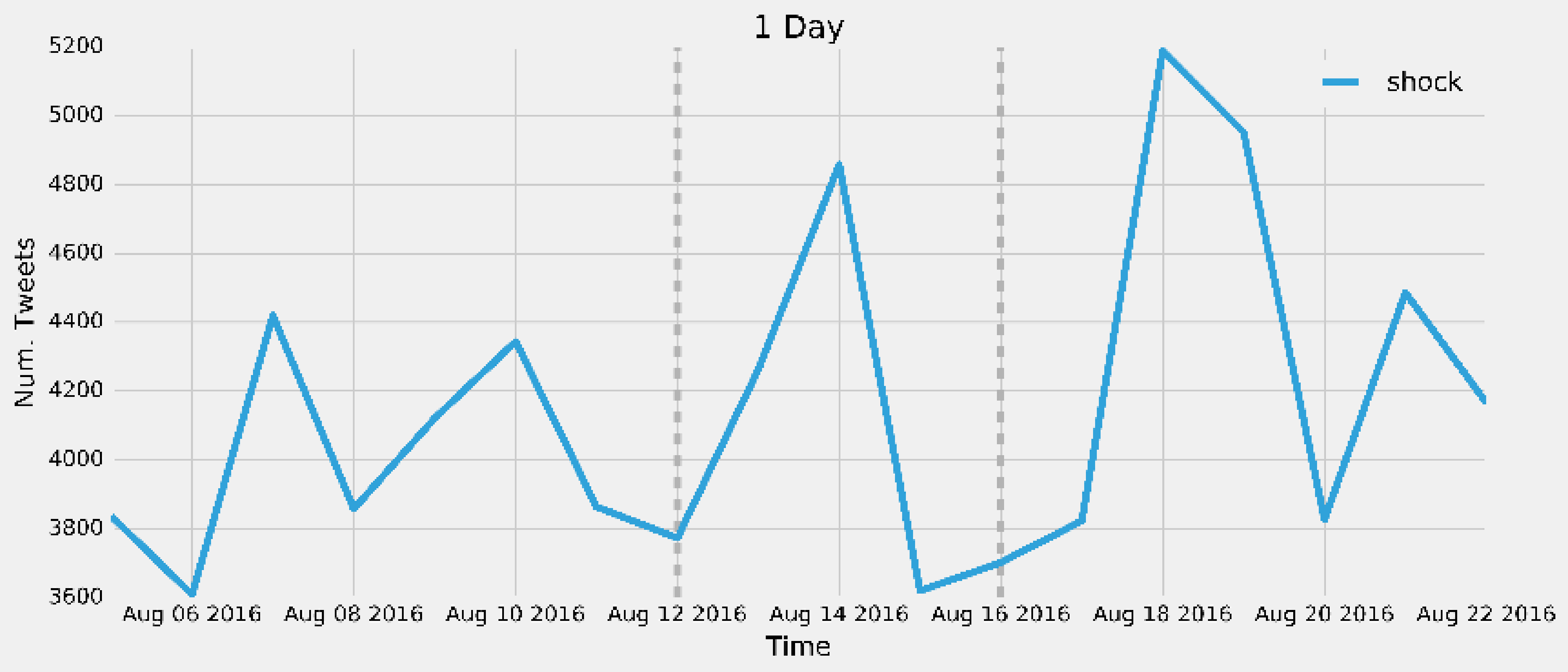
1 Day
shock
Num. Tweets
5200
5000
4800
4600
4400
4200
4000
3800
3600
Aug 06 2016
Aug 08 2016
Aug 10 2016
Aug 12 2016
Aug 14 2016
Aug 16 2016
Aug 18 2016
Aug 20 2016
Aug 22 2016
Time

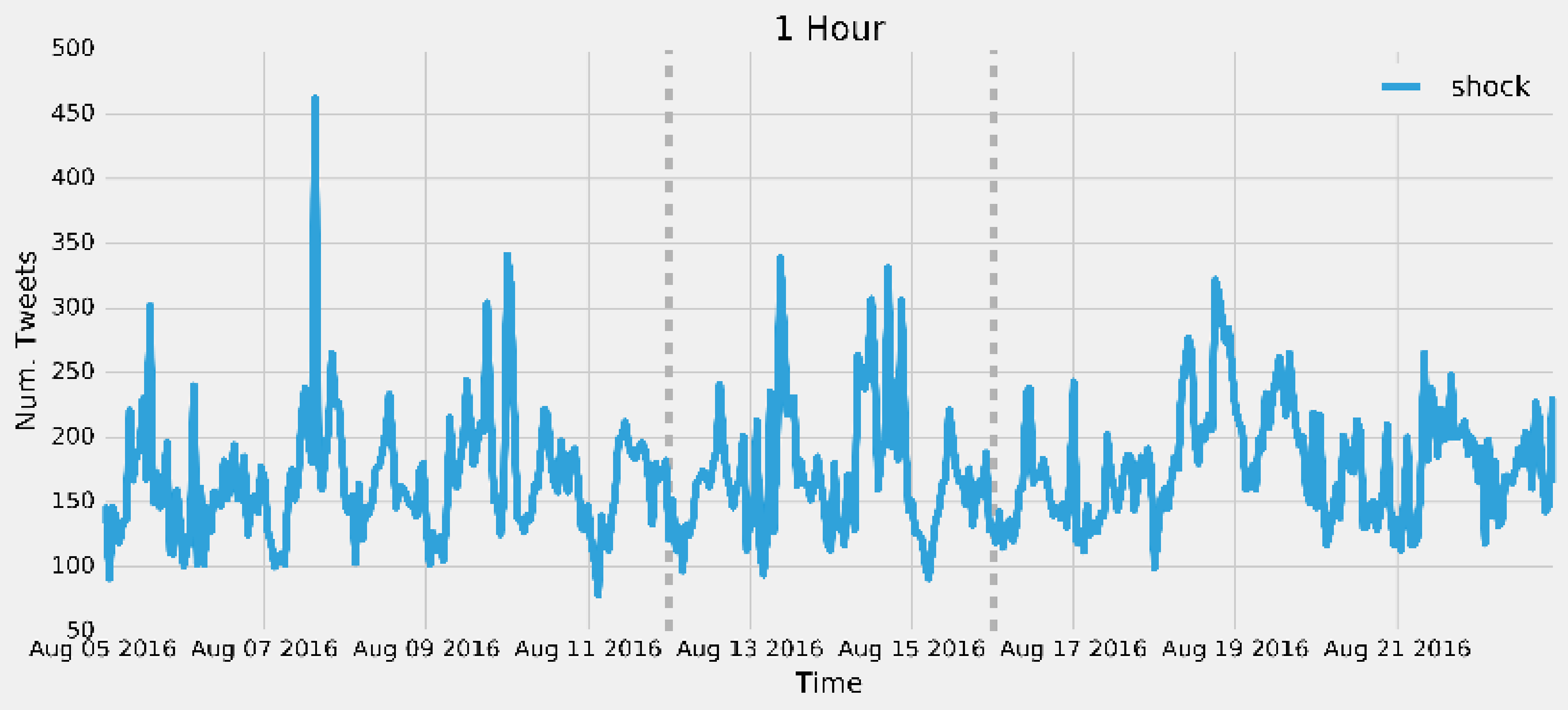
1 Hour
shock
Num. Tweets
500
450
400
350
300
250
200
150
100
50
Aug 05 2016
Aug 07 2016
Aug 09 2016
Aug 11 2016
Aug 13 2016
Aug 15 2016
Aug 17 2016
Aug 19 2016
Aug 21 2016
Time

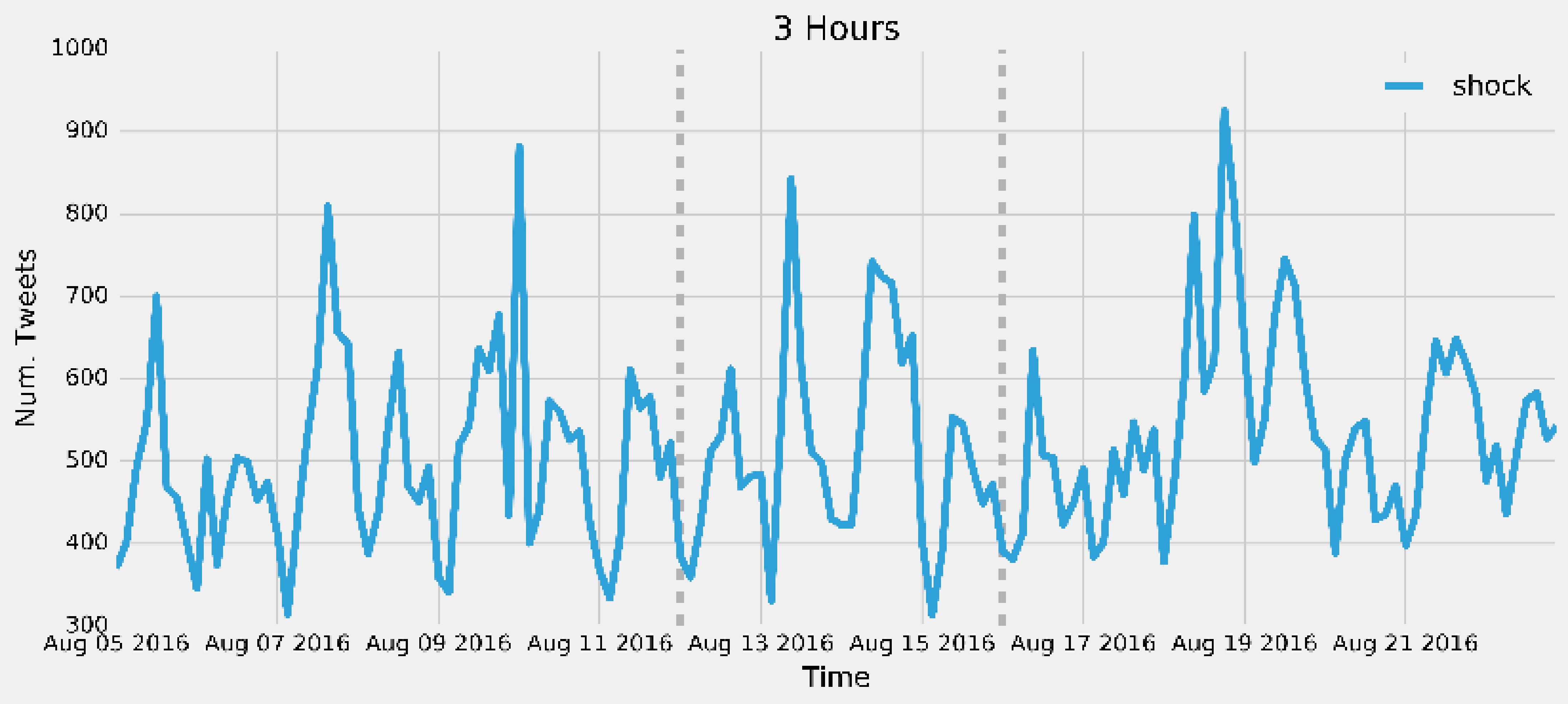

3 Hours
shock
Num. Tweets
1000
900
800
700
600
500
400
300
Aug 05 2016
Aug 07 2016
Aug 09 2016
Aug 11 2016
Aug 13 2016
Aug 15 2016
Aug 17 2016
Aug 19 2016
Aug 21 2016
Time

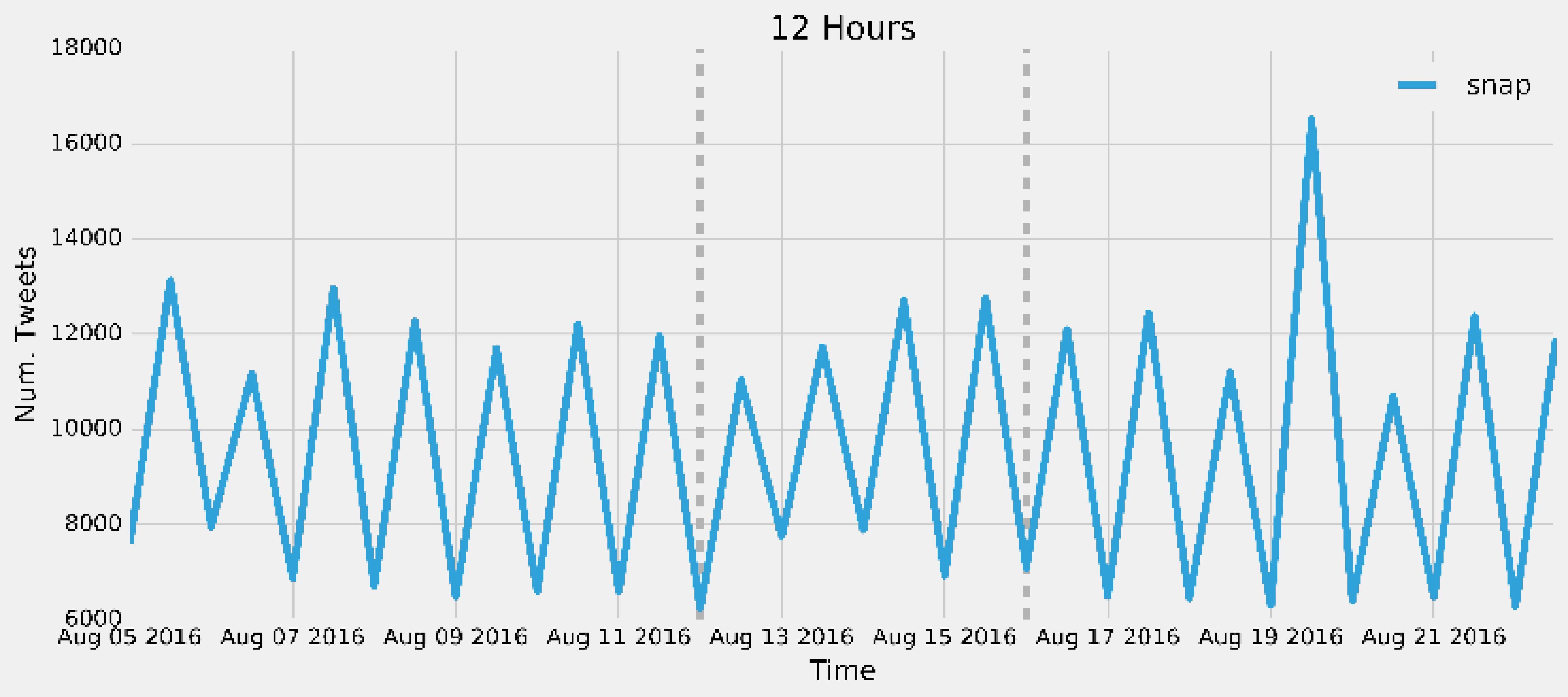

12 Hours
snap
Num. Tweets
18000
16000
14000
12000
10000
8000
6000
Aug 05 2016
Aug 07 2016
Aug 09 2016
Aug 11 2016
Aug 13 2016
Aug 15 2016
Aug 17 2016
Aug 19 2016
Aug 21 2016
Time

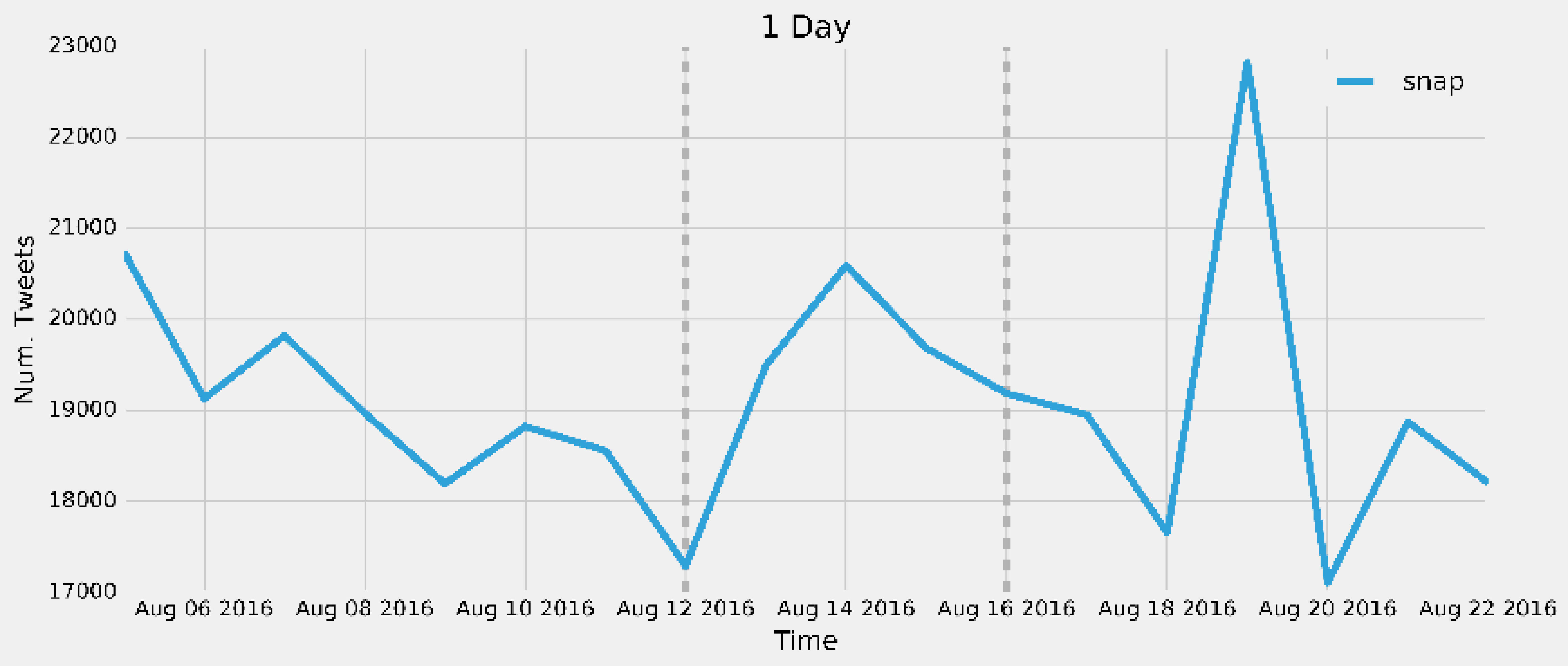

1 Day
snap
23000
22000
21000
20000
19000
18000
17000
Num. Tweets
Aug 06 2016
Aug 08 2016
Aug 10 2016
Aug 12 2016
Aug 14 2016
Aug 16 2016
Aug 18 2016
Aug 20 2016
Aug 22 2016
Time

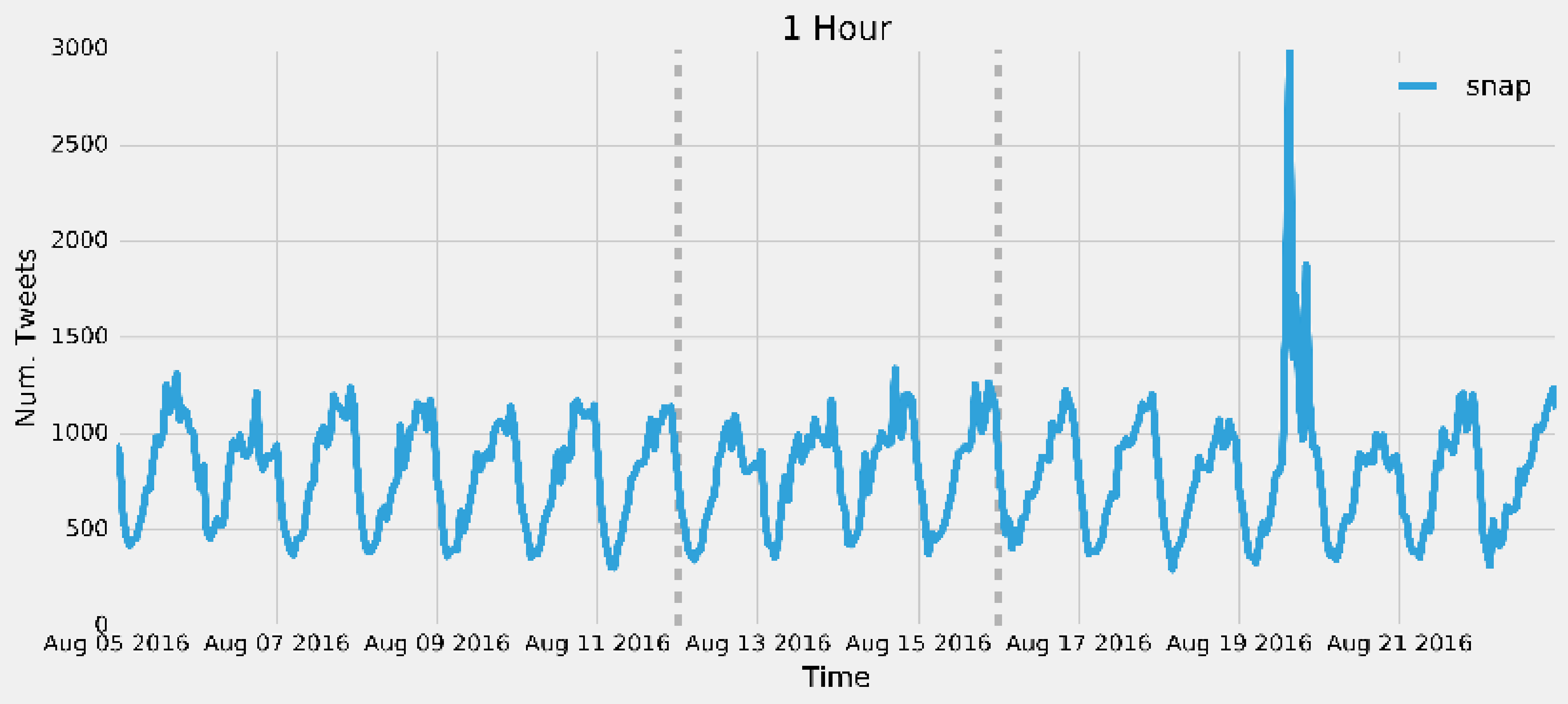
1 Hour
3000
2500
2000
1500
1000
500
0
Num. Tweets
snap
Aug 05 2016
Aug 07 2016
Aug 09 2016
Aug 11 2016
Aug 13 2016
Aug 15 2016
Aug 17 2016
Aug 19 2016
Aug 21 2016
Time

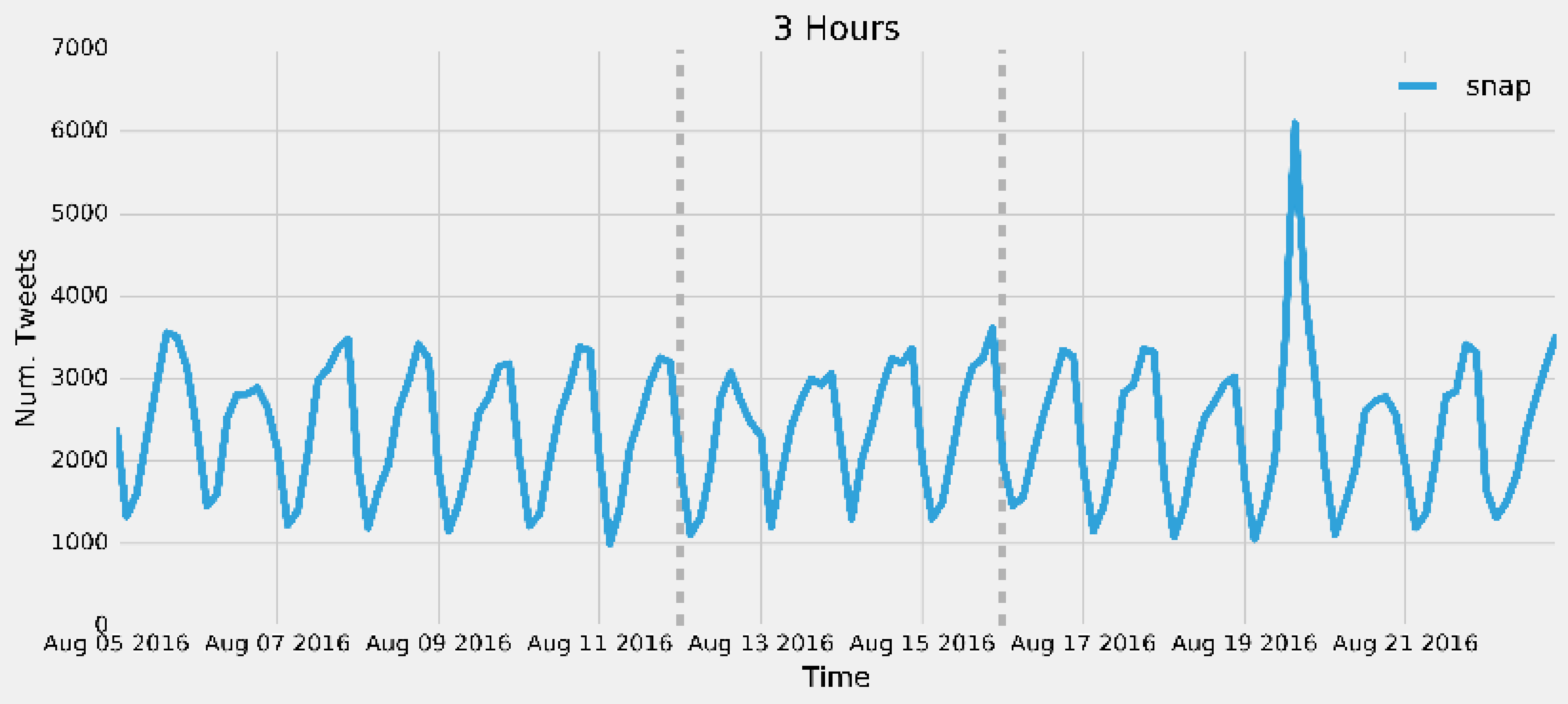
3 Hours
snap
Num. Tweets
0
1000
2000
3000
4000
5000
6000
7000
Aug 05 2016
Aug 07 2016
Aug 09 2016
Aug 11 2016
Aug 13 2016
Aug 15 2016
Aug 17 2016
Aug 19 2016
Aug 21 2016
Time

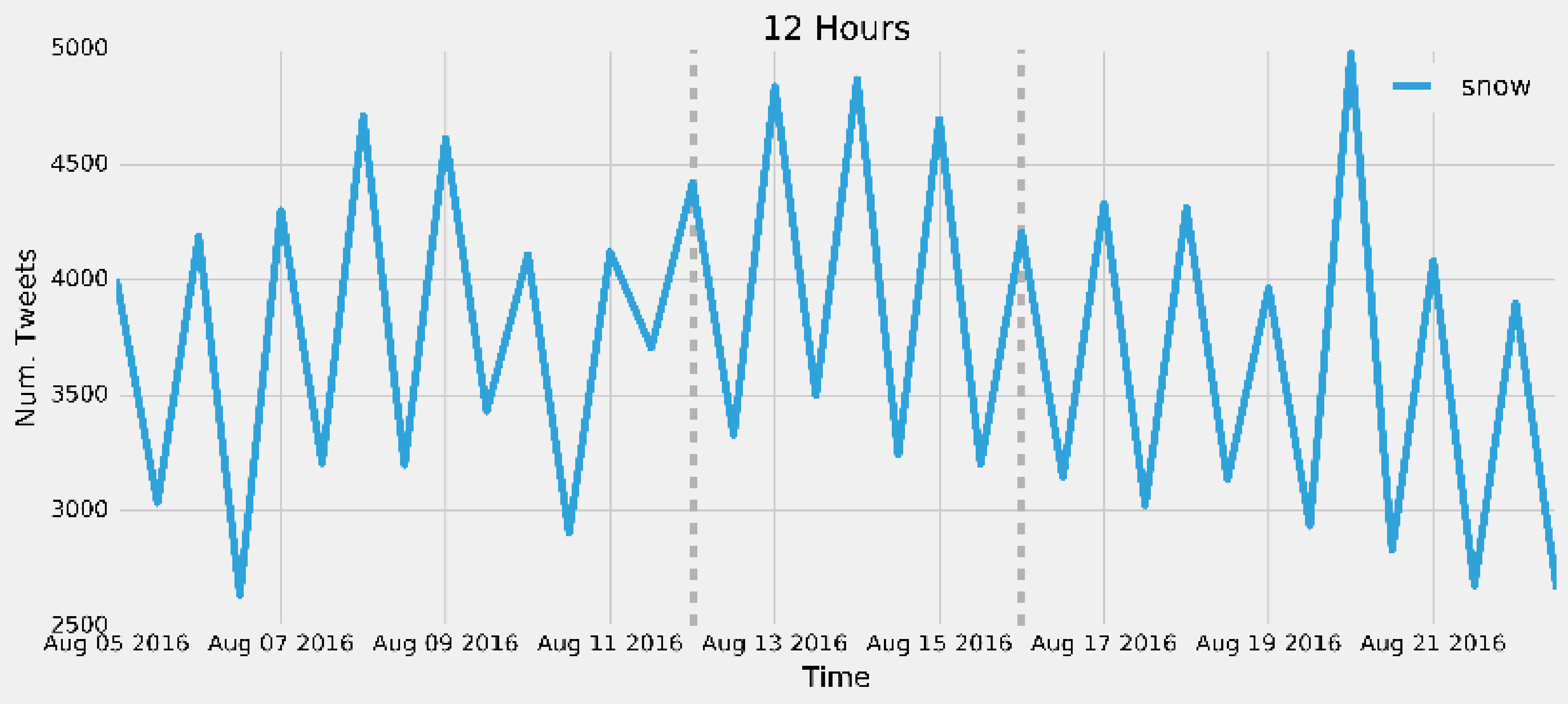

12 Hours
snow
Num. Tweets
5000
4500
4000
3500
3000
2500
Aug 05 2016
Aug 07 2016
Aug 09 2016
Aug 11 2016
Aug 13 2016
Aug 15 2016
Aug 17 2016
Aug 19 2016
Aug 21 2016
Time

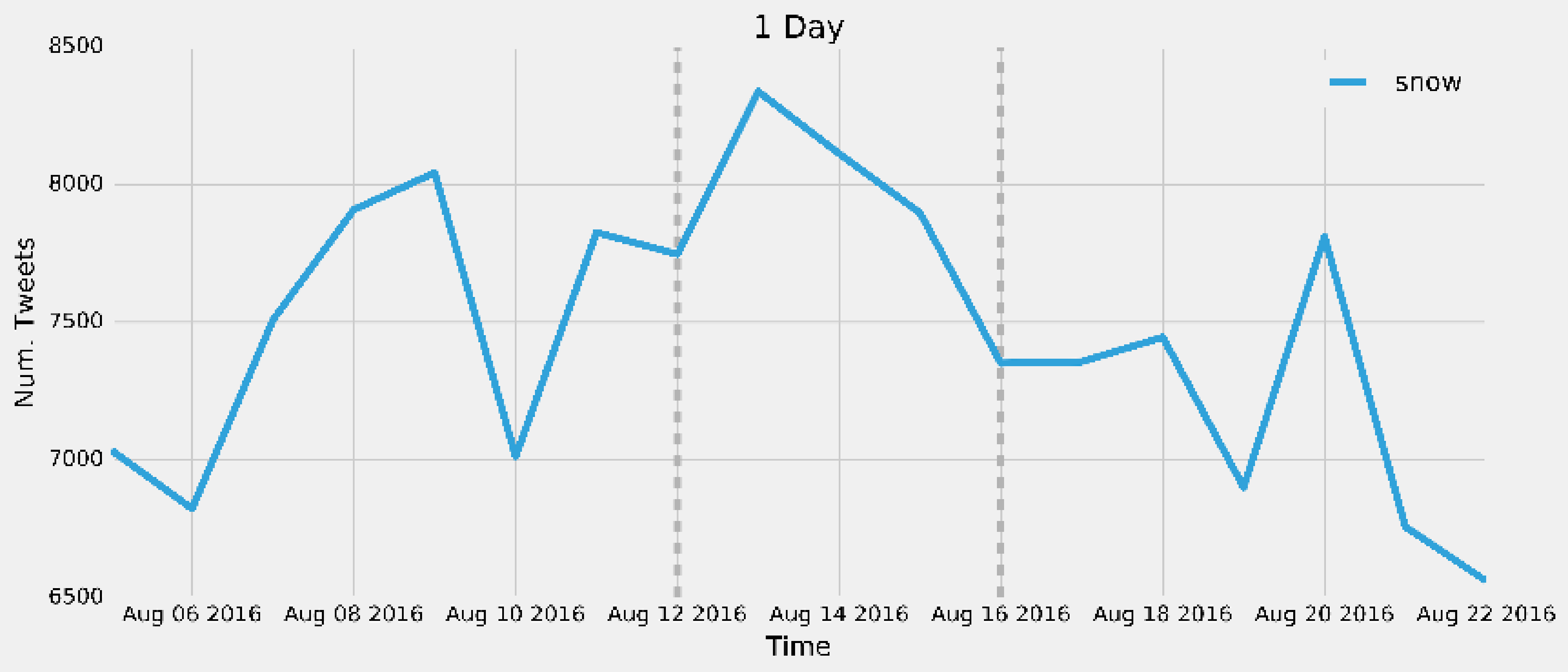
1 Day
8500
8000
7500
7000
6500
Num. Tweets
snow
Aug 06 2016
Aug 08 2016
Aug 10 2016
Aug 12 2016
Aug 14 2016
Aug 16 2016
Aug 18 2016
Aug 20 2016
Aug 22 2016
Time

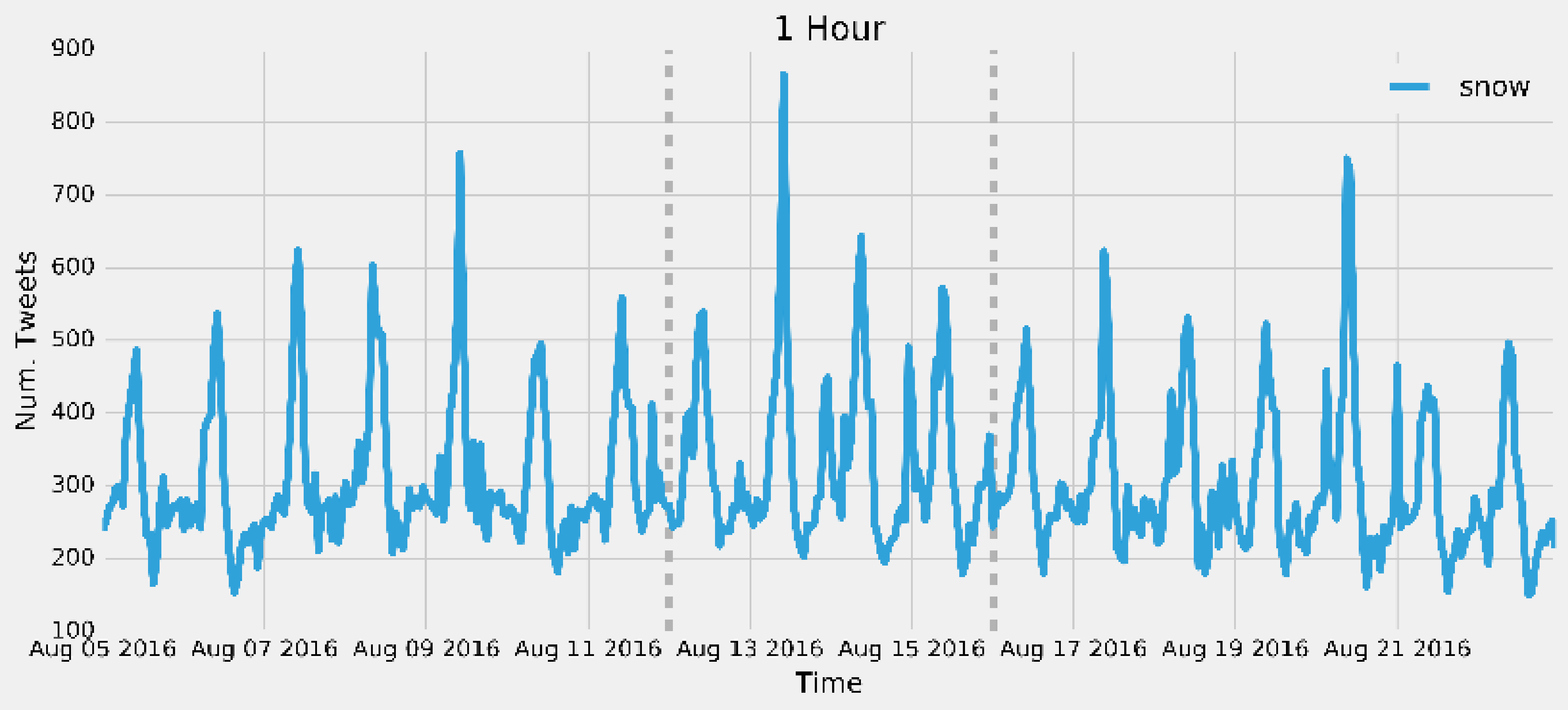

1 Hour
snow
Num. Tweets
900
800
700
600
500
400
300
200
100
Aug 05 2016
Aug 07 2016
Aug 09 2016
Aug 11 2016
Aug 13 2016
Aug 15 2016
Aug 17 2016
Aug 19 2016
Aug 21 2016
Time

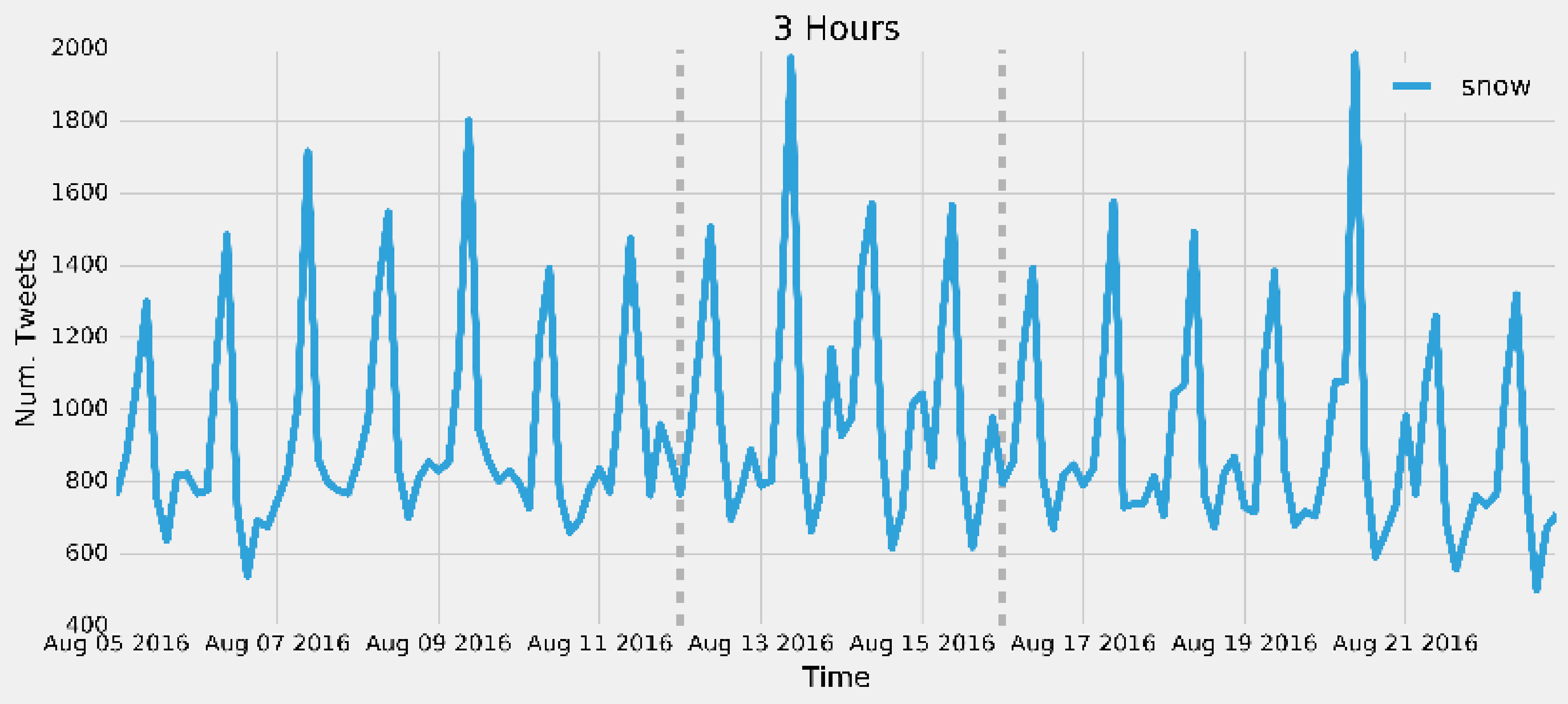
3 Hours
snow
Num. Tweets
Time
2000
1800
1600
1400
1200
1000
800
600
400
Aug 05 2016
Aug 07 2016
Aug 09 2016
Aug 11 2016
Aug 13 2016
Aug 15 2016
Aug 17 2016
Aug 19 2016
Aug 21 2016

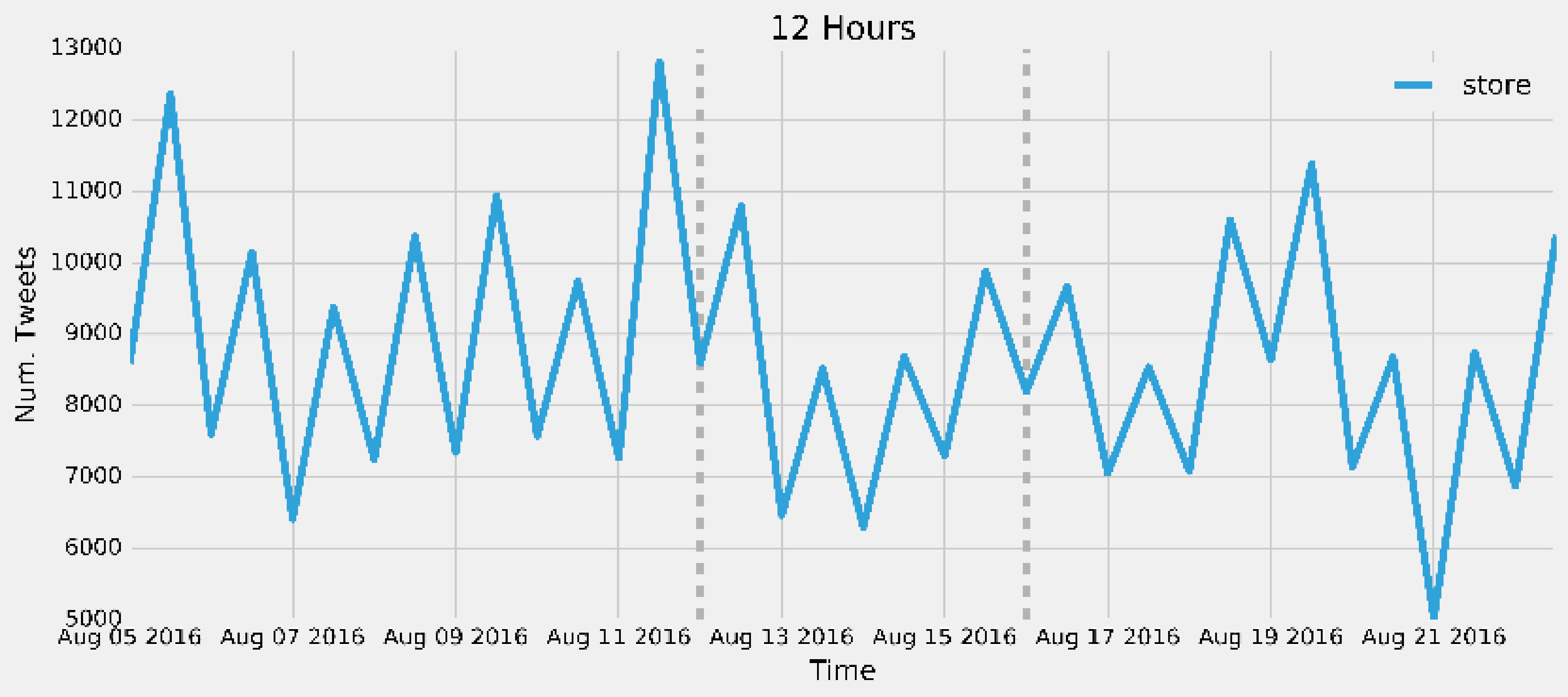
12 Hours
store
Num. Tweets
13000
12000
11000
10000
9000
8000
7000
6000
5000
Aug 05 2016
Aug 07 2016
Aug 09 2016
Aug 11 2016
Aug 13 2016
Aug 15 2016
Aug 17 2016
Aug 19 2016
Aug 21 2016
Time

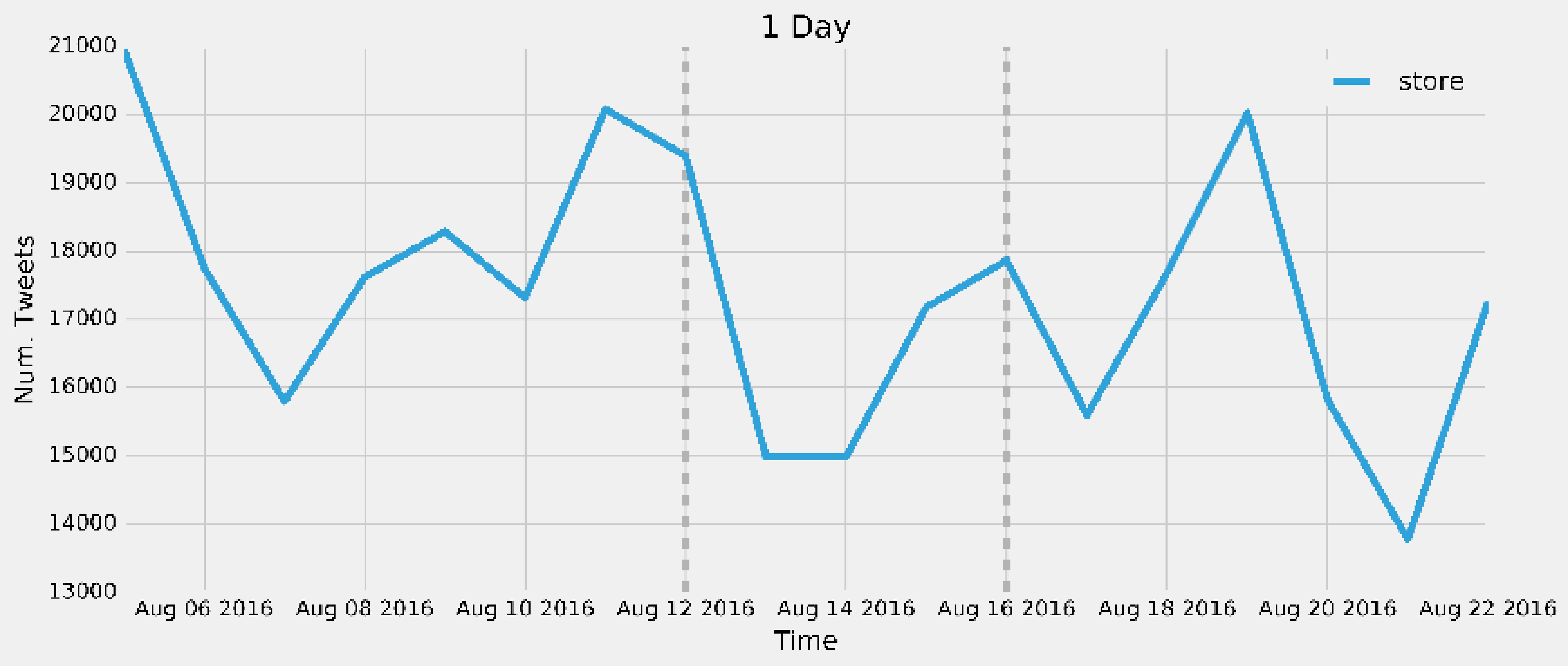

1 Day
store
21000
20000
19000
18000
17000
16000
15000
14000
13000
Num. Tweets
Aug 06 2016
Aug 08 2016
Aug 10 2016
Aug 12 2016
Aug 14 2016
Aug 16 2016
Aug 18 2016
Aug 20 2016
Aug 22 2016
Time

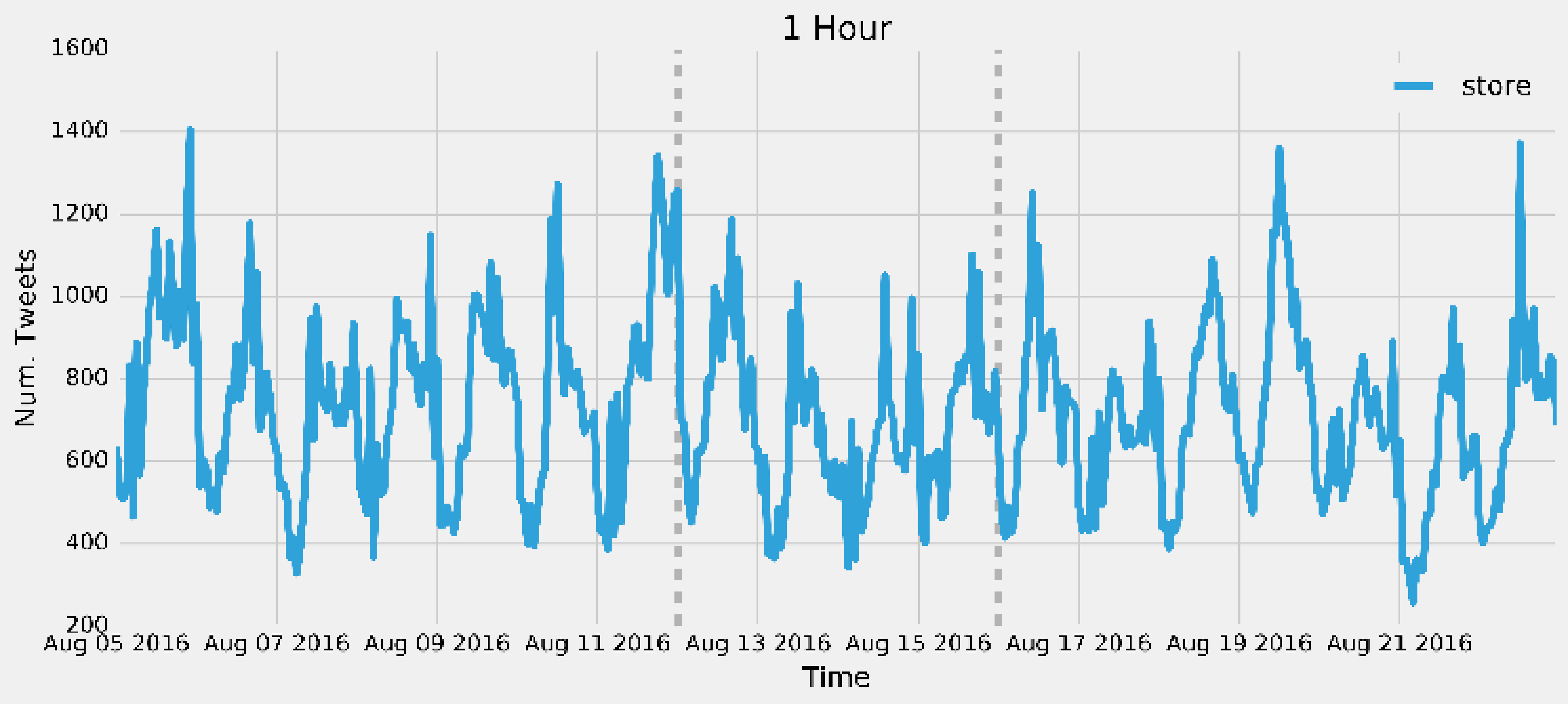
1 Hour
store
Num. Tweets
1600
1400
1200
1000
800
600
400
200
Aug 05 2016
Aug 07 2016
Aug 09 2016
Aug 11 2016
Aug 13 2016
Aug 15 2016
Aug 17 2016
Aug 19 2016
Aug 21 2016
Time

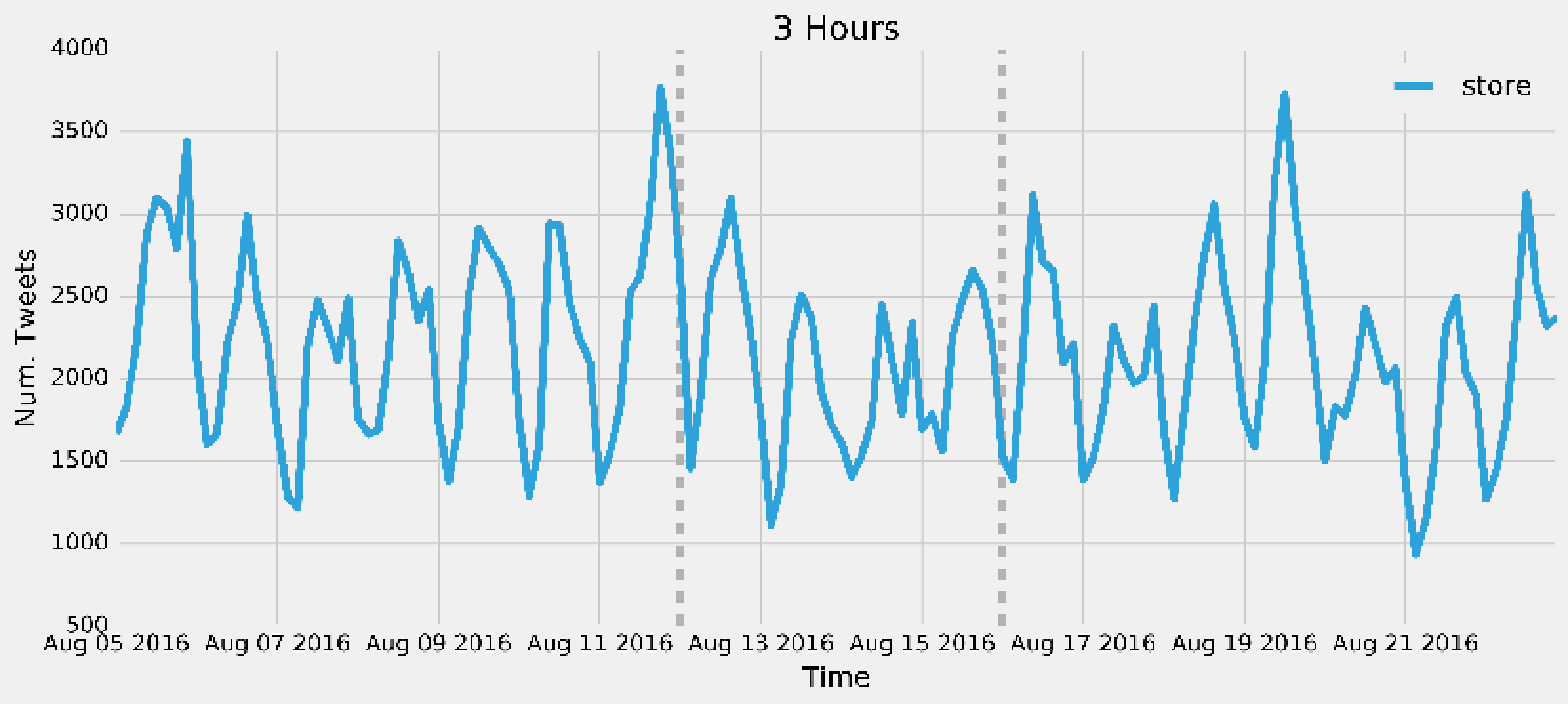
3 Hours
store
Num. Tweets
4000
3500
3000
2500
2000
1500
1000
500
Aug 05 2016
Aug 07 2016
Aug 09 2016
Aug 11 2016
Aug 13 2016
Aug 15 2016
Aug 17 2016
Aug 19 2016
Aug 21 2016
Time

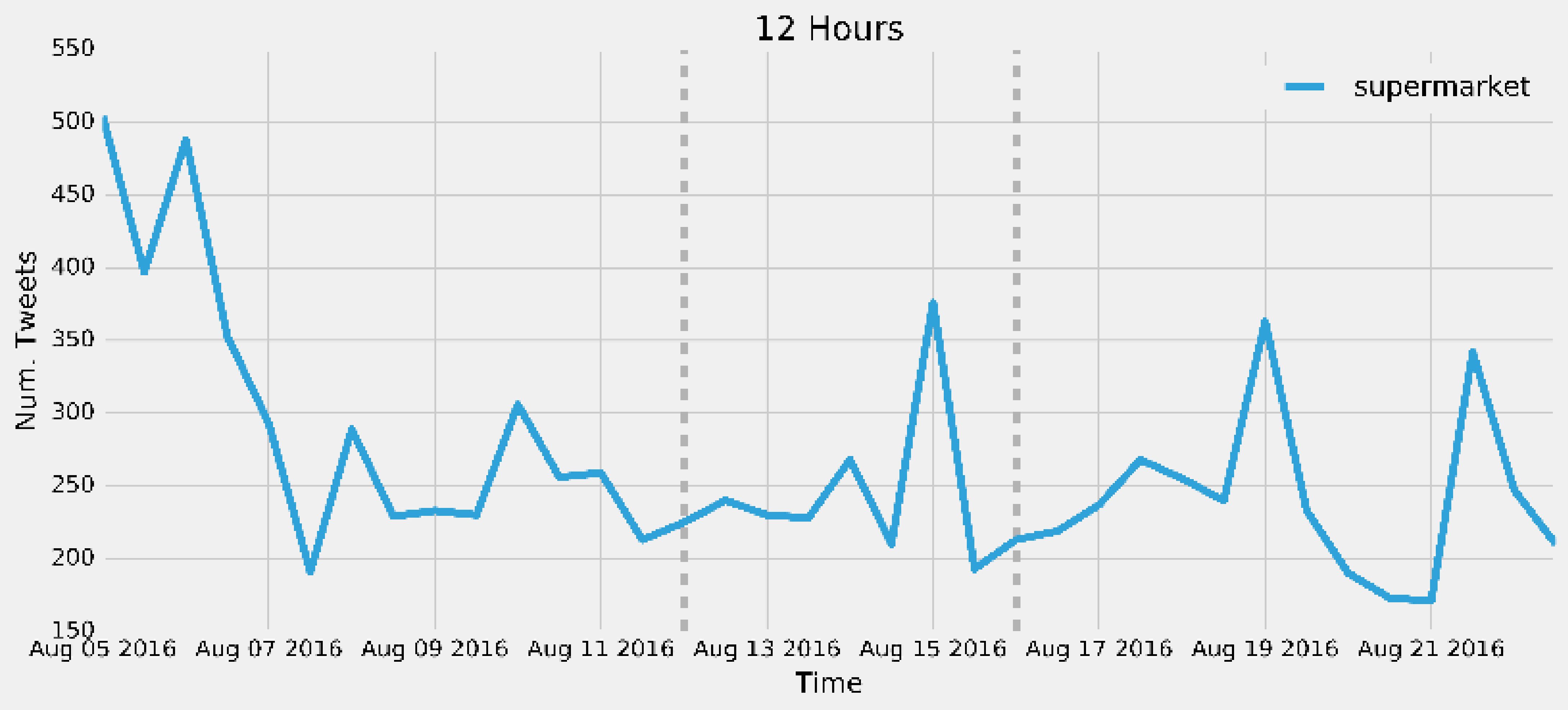

12 Hours
supermarket
Num. Tweets
550
500
450
400
350
300
250
200
150
Aug 05 2016
Aug 07 2016
Aug 09 2016
Aug 11 2016
Aug 13 2016
Aug 15 2016
Aug 17 2016
Aug 19 2016
Aug 21 2016
Time

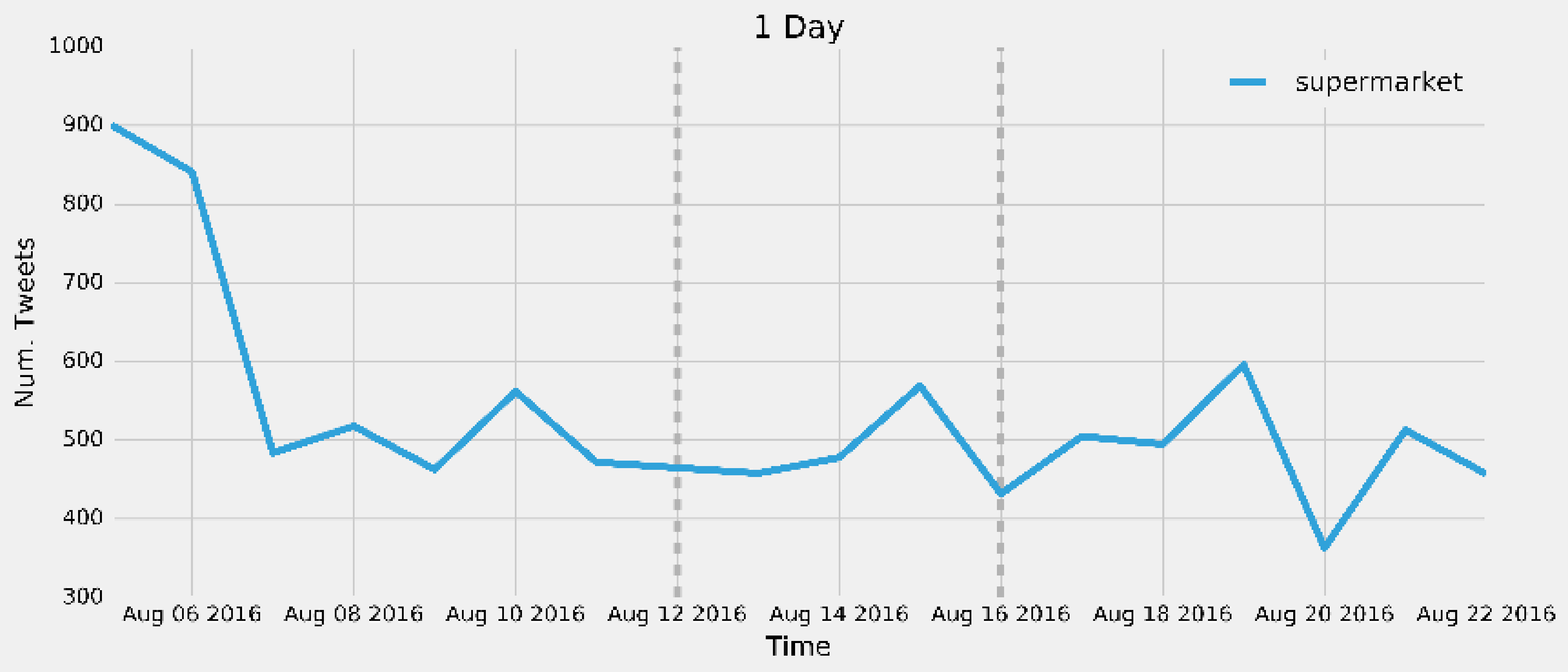
1 Day
1000
900
800
700
600
500
400
300
Num. Tweets
supermarket
Aug 06 2016
Aug 08 2016
Aug 10 2016
Aug 12 2016
Aug 14 2016
Aug 16 2016
Aug 18 2016
Aug 20 2016
Aug 22 2016
Time

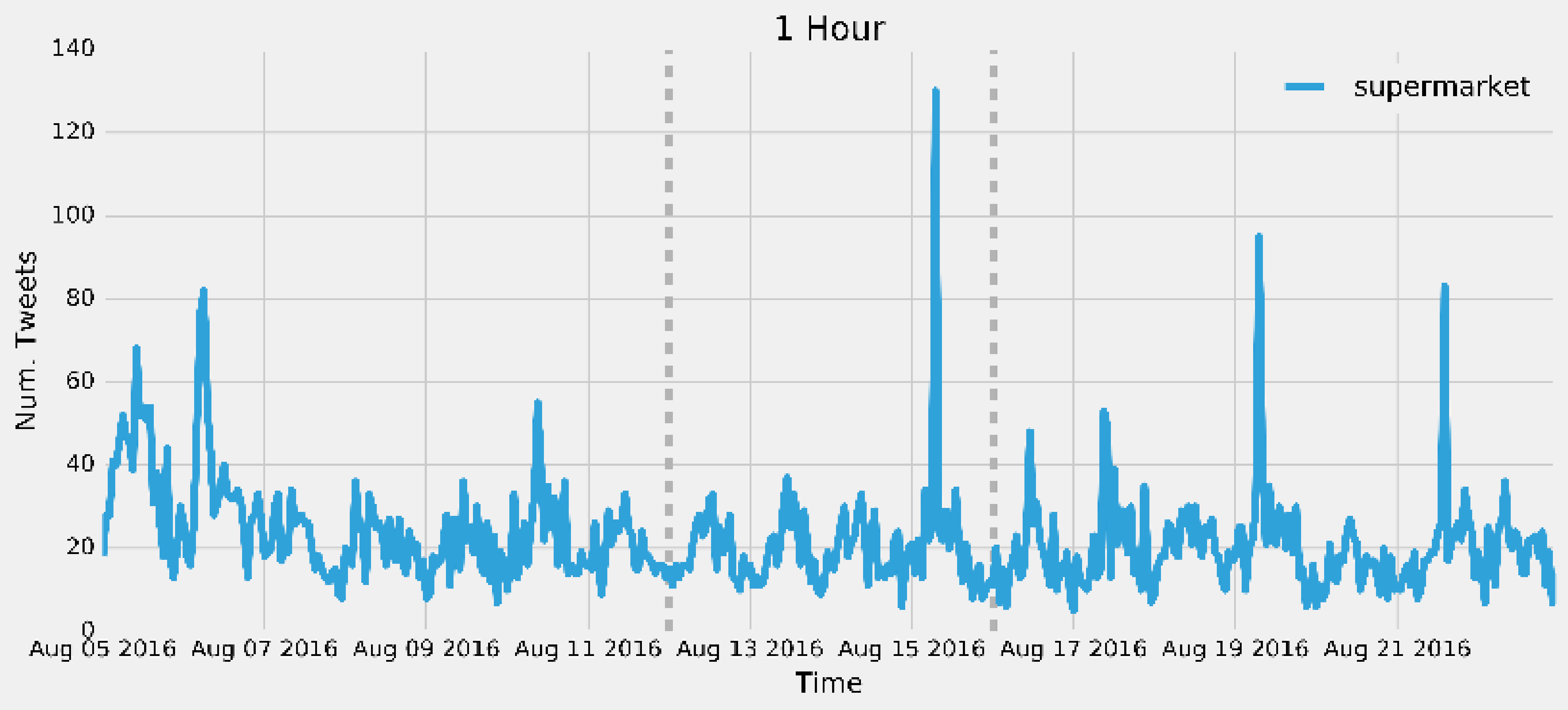
1 Hour
supermarket
Num. Tweets
0
20
40
60
80
100
120
140
Aug 05 2016
Aug 07 2016
Aug 09 2016
Aug 11 2016
Aug 13 2016
Aug 15 2016
Aug 17 2016
Aug 19 2016
Aug 21 2016
Time

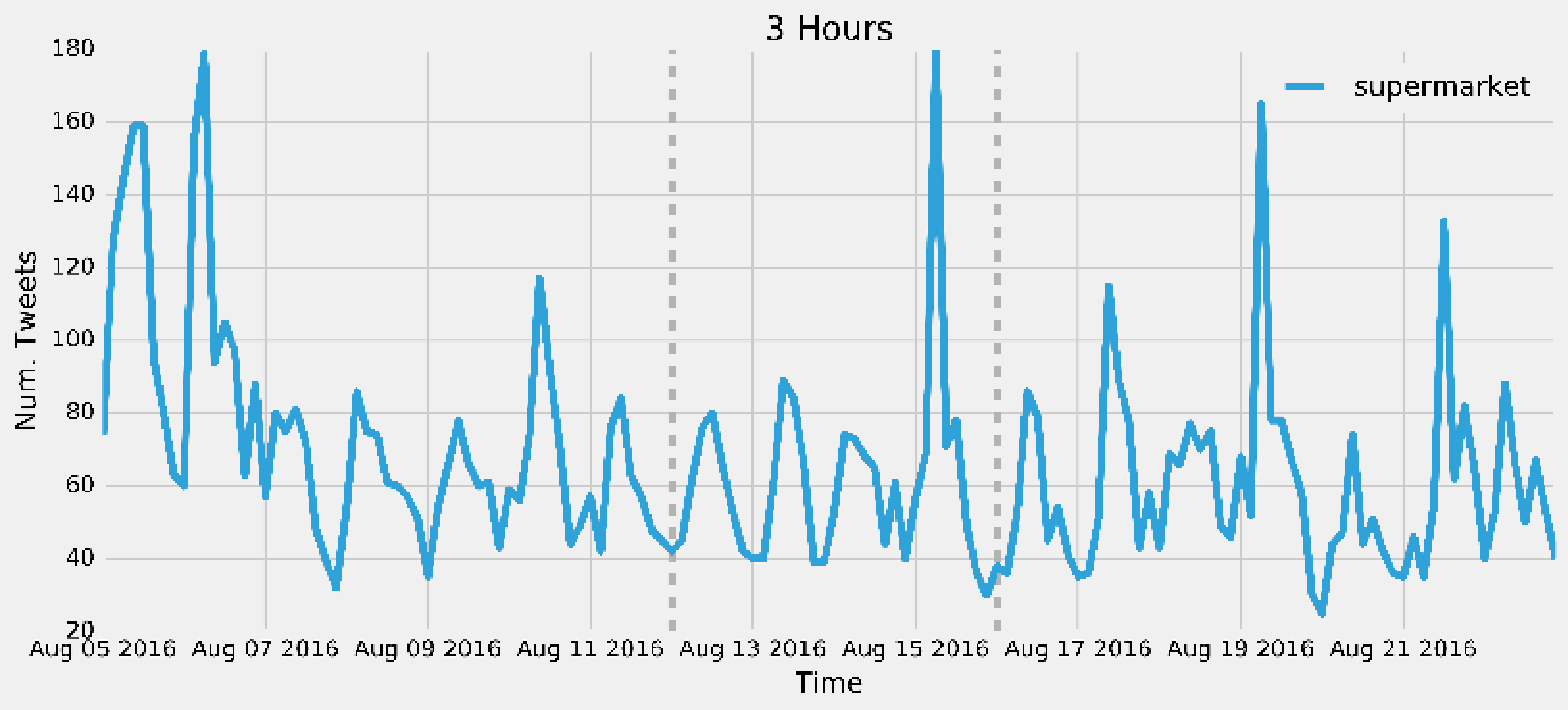
3 Hours
supermarket
Num. Tweets
180
160
140
120
100
80
60
40
20
Aug 05 2016
Aug 07 2016
Aug 09 2016
Aug 11 2016
Aug 13 2016
Aug 15 2016
Aug 17 2016
Aug 19 2016
Aug 21 2016
Time

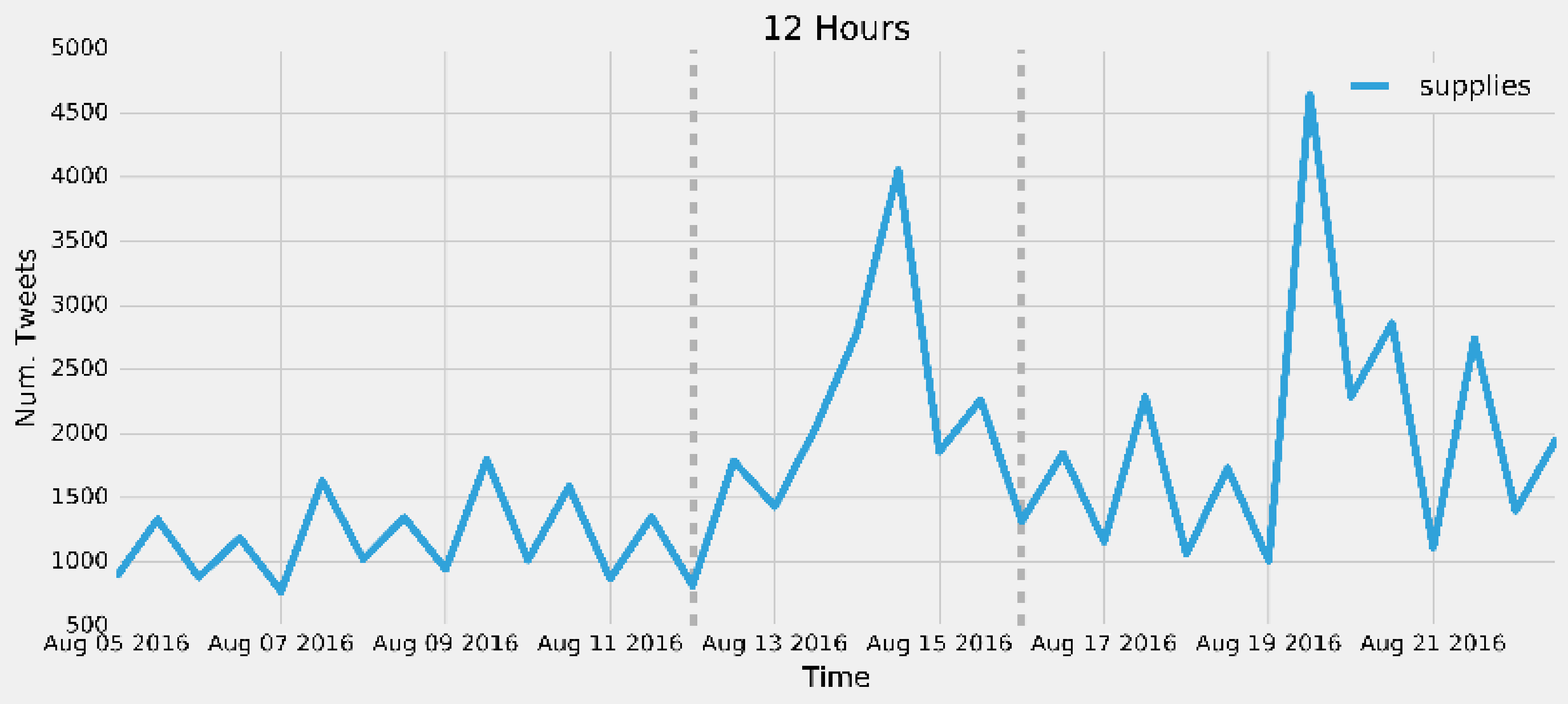

12 Hours
Num. Tweets
5000
4500
4000
3500
3000
2500
2000
1500
1000
500
Aug 05 2016
Aug 07 2016
Aug 09 2016
Aug 11 2016
Aug 13 2016
Aug 15 2016
Aug 17 2016
Aug 19 2016
Aug 21 2016
Time
supplies

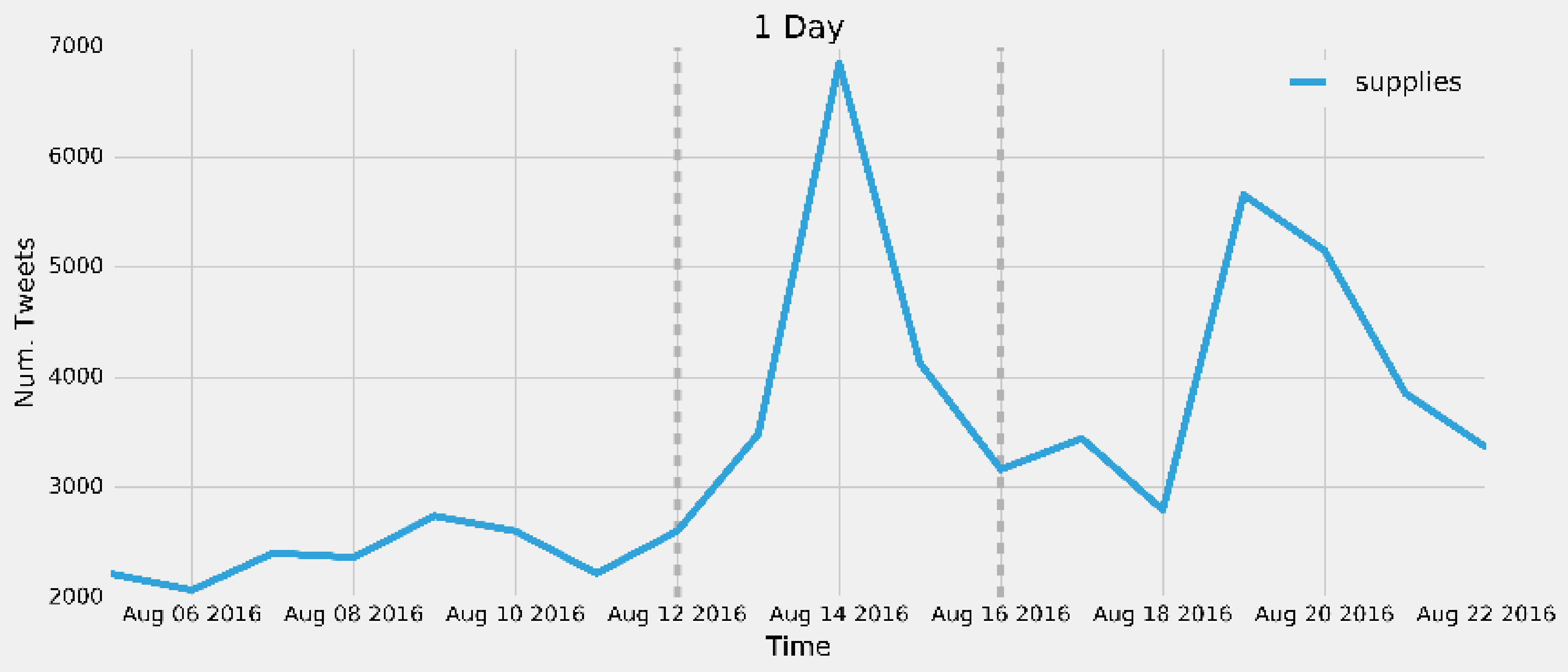

1 Day
supplies
Num. Tweets
7000
6000
5000
4000
3000
2000
Aug 06 2016
Aug 08 2016
Aug 10 2016
Aug 12 2016
Aug 14 2016
Aug 16 2016
Aug 18 2016
Aug 20 2016
Aug 22 2016
Time

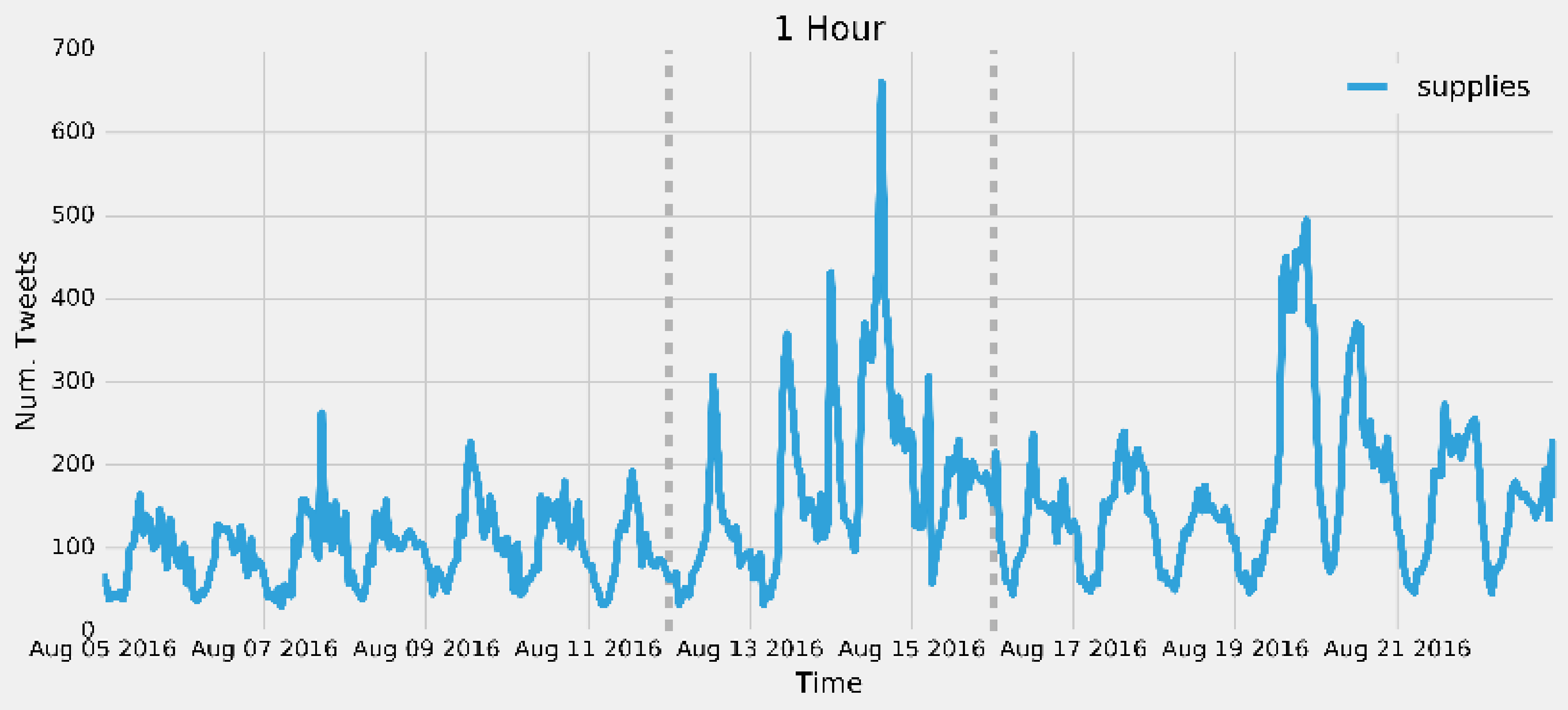
1 Hour
supplies
Num. Tweets
0
100
200
300
400
500
600
700
Aug 05 2016
Aug 07 2016
Aug 09 2016
Aug 11 2016
Aug 13 2016
Aug 15 2016
Aug 17 2016
Aug 19 2016
Aug 21 2016
Time

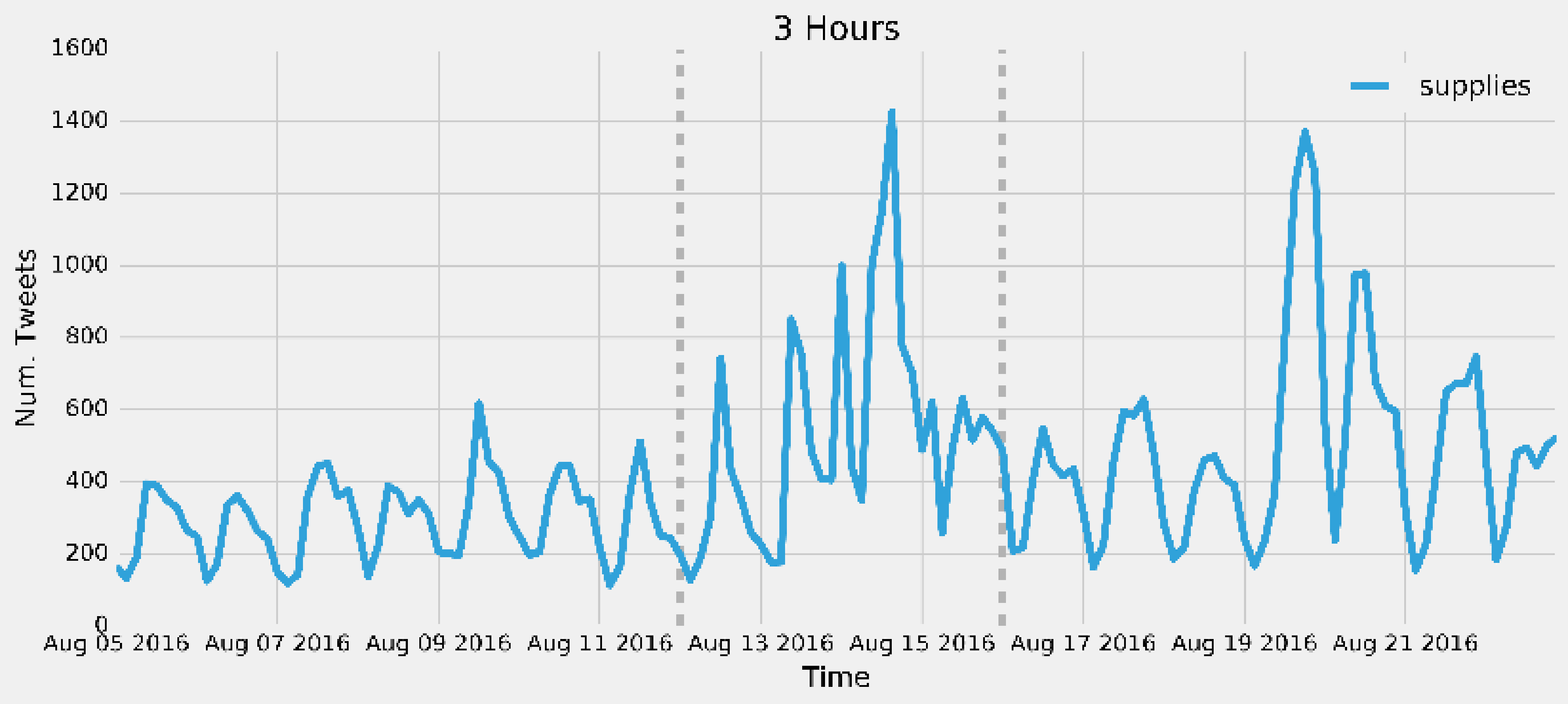
3 Hours
supplies
Num. Tweets
0
200
400
600
800
1000
1200
1400
1600
Aug 05 2016
Aug 07 2016
Aug 09 2016
Aug 11 2016
Aug 13 2016
Aug 15 2016
Aug 17 2016
Aug 19 2016
Aug 21 2016
Time

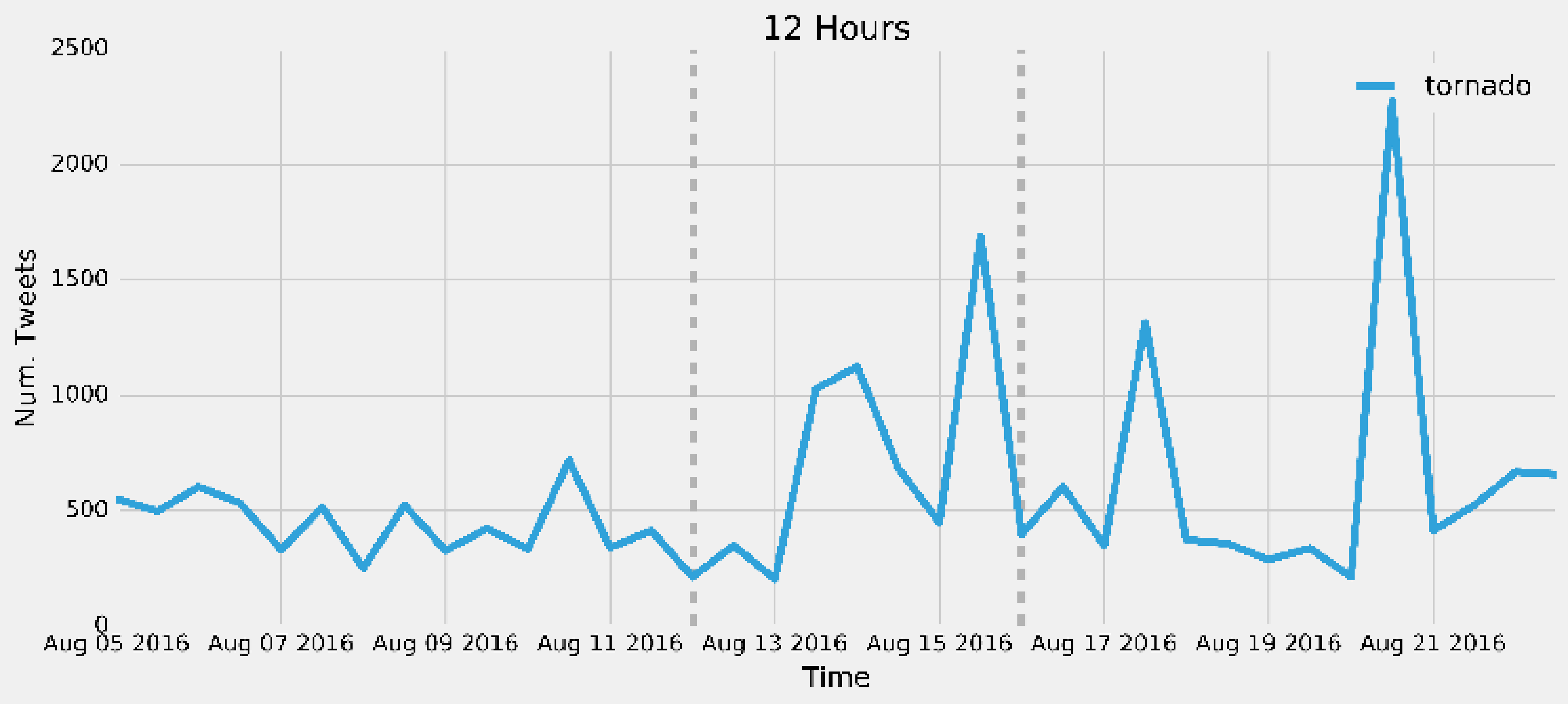
12 Hours
tornado
Num. Tweets
Time
0
500
1000
1500
2000
2500
Aug 05 2016
Aug 07 2016
Aug 09 2016
Aug 11 2016
Aug 13 2016
Aug 15 2016
Aug 17 2016
Aug 19 2016
Aug 21 2016

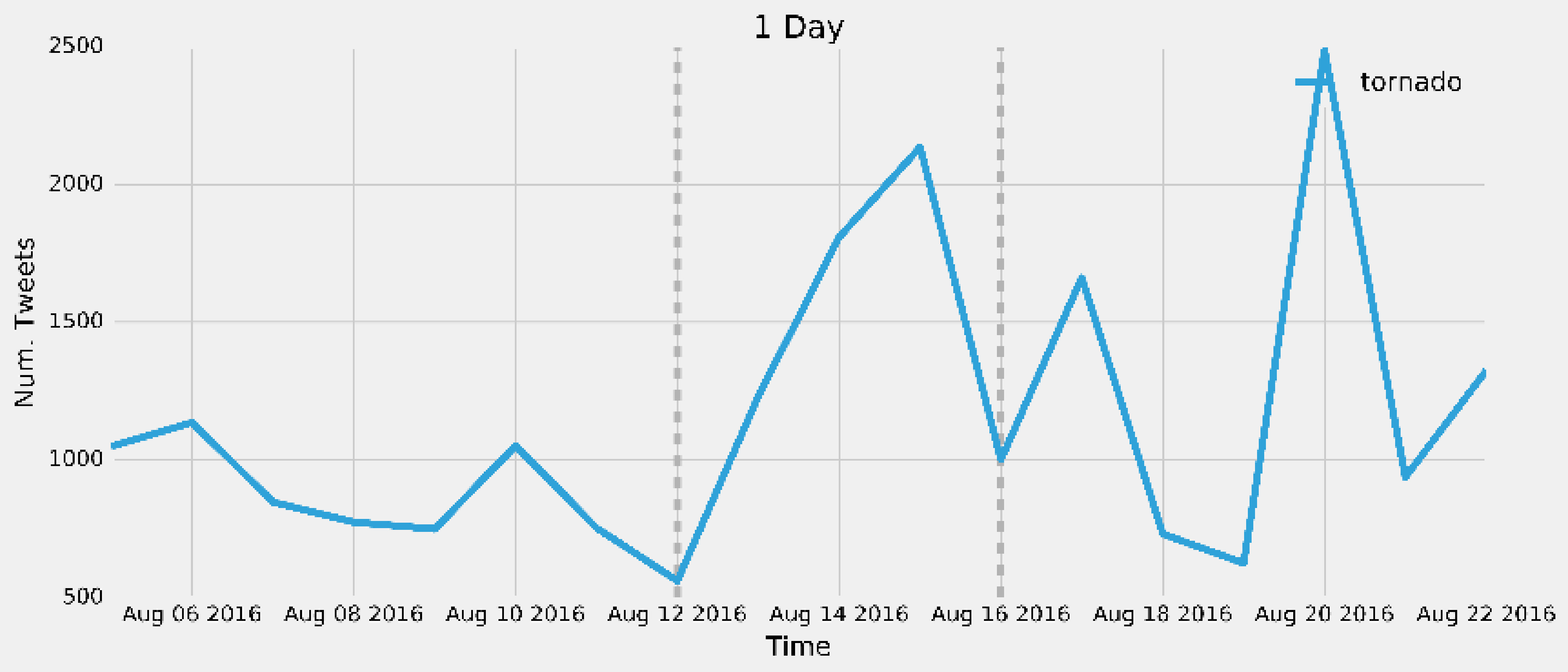

1 Day
tornado
Num. Tweets
2500
2000
1500
1000
500
Aug 06 2016
Aug 08 2016
Aug 10 2016
Aug 12 2016
Aug 14 2016
Aug 16 2016
Aug 18 2016
Aug 20 2016
Aug 22 2016
Time

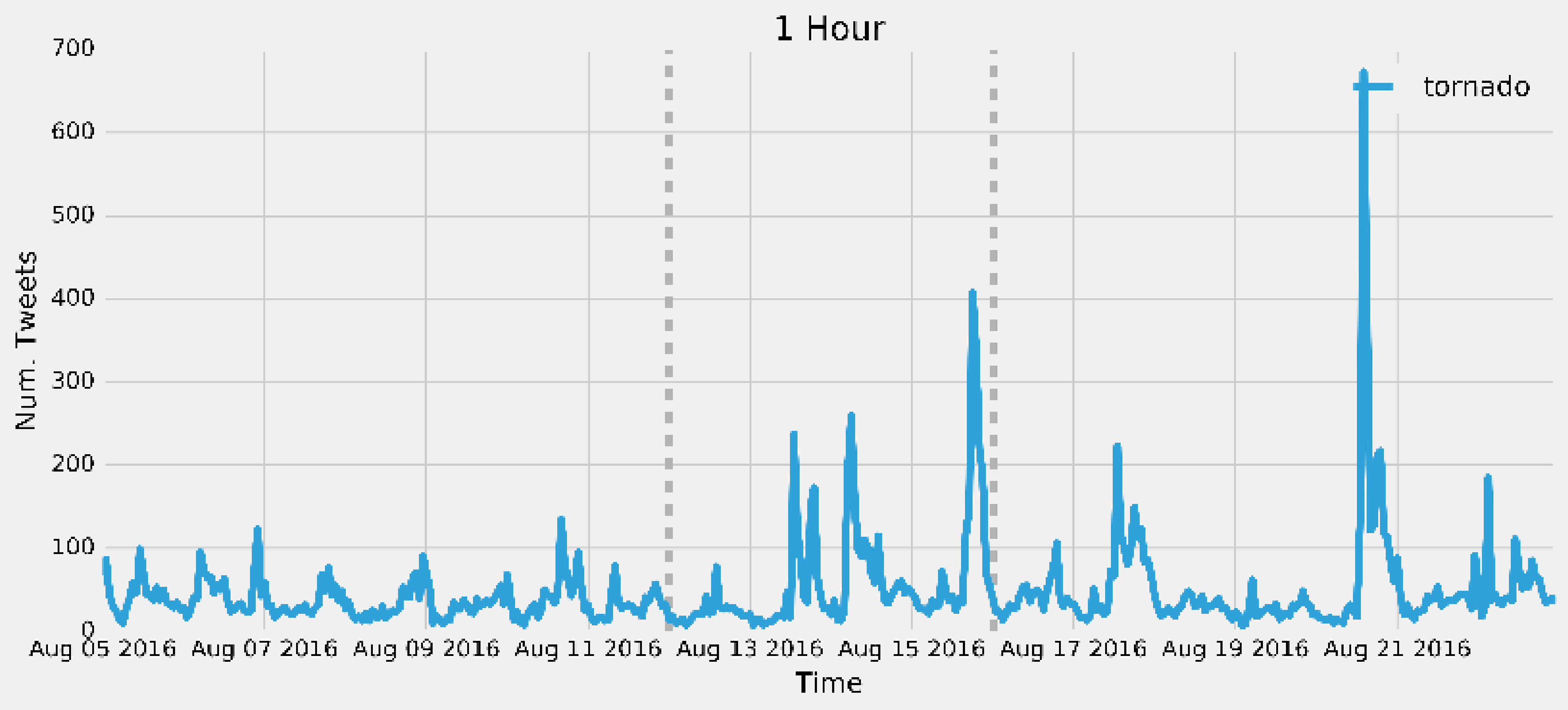

1 Hour
tornado
Num. Tweets
Time
0
100
200
300
400
500
600
700
Aug 05 2016
Aug 07 2016
Aug 09 2016
Aug 11 2016
Aug 13 2016
Aug 15 2016
Aug 17 2016
Aug 19 2016
Aug 21 2016

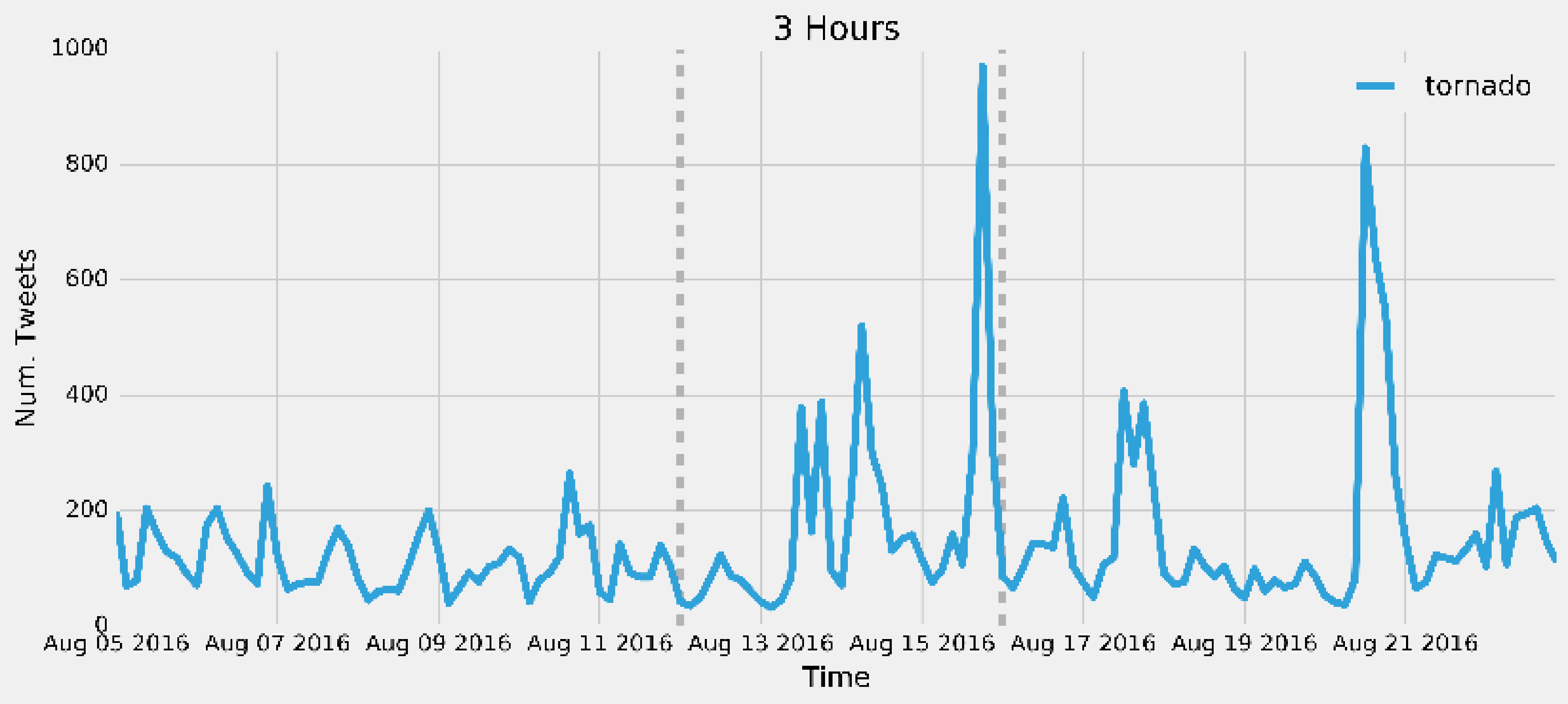

3 Hours
tornado
Num. Tweets
1000
800
600
400
200
0
Aug 05 2016
Aug 07 2016
Aug 09 2016
Aug 11 2016
Aug 13 2016
Aug 15 2016
Aug 17 2016
Aug 19 2016
Aug 21 2016
Time

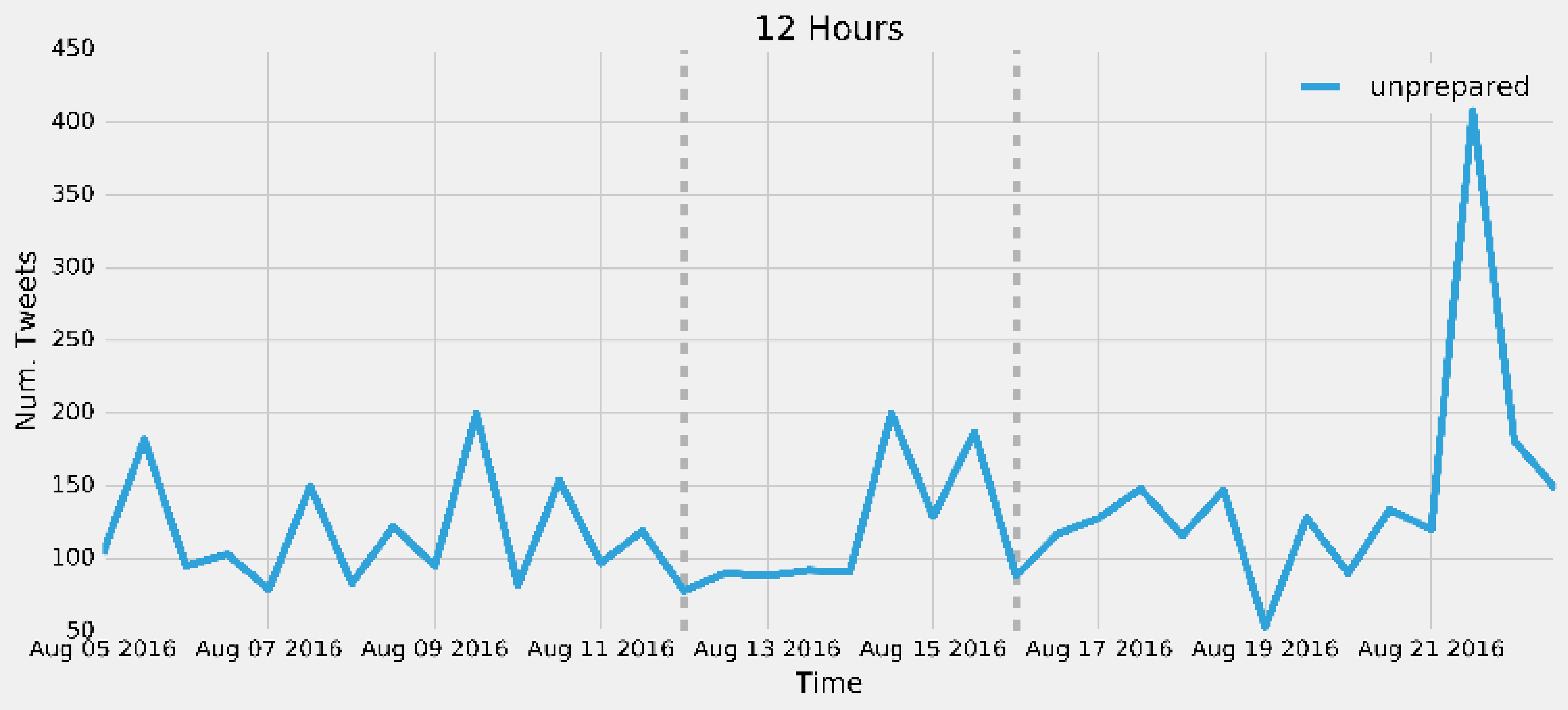
12 Hours
unprepared
Num. Tweets
450
400
350
300
250
200
150
100
50
Aug 05 2016
Aug 07 2016
Aug 09 2016
Aug 11 2016
Aug 13 2016
Aug 15 2016
Aug 17 2016
Aug 19 2016
Aug 21 2016
Time

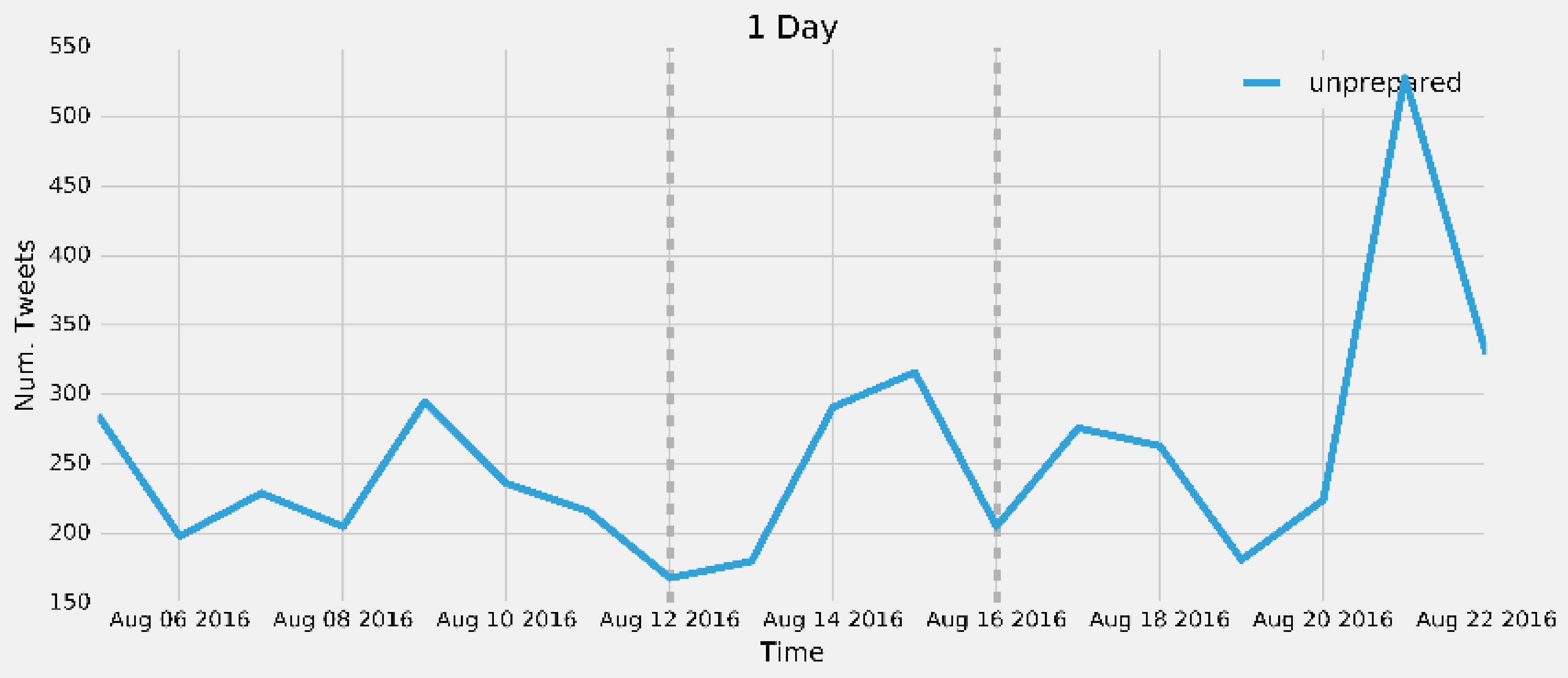
1 Day
unprepared
Num. Tweets
550
500
450
400
350
300
250
200
150
Aug 06 2016
Aug 08 2016
Aug 10 2016
Aug 12 2016
Aug 14 2016
Aug 16 2016
Aug 18 2016
Aug 20 2016
Aug 22 2016
Time

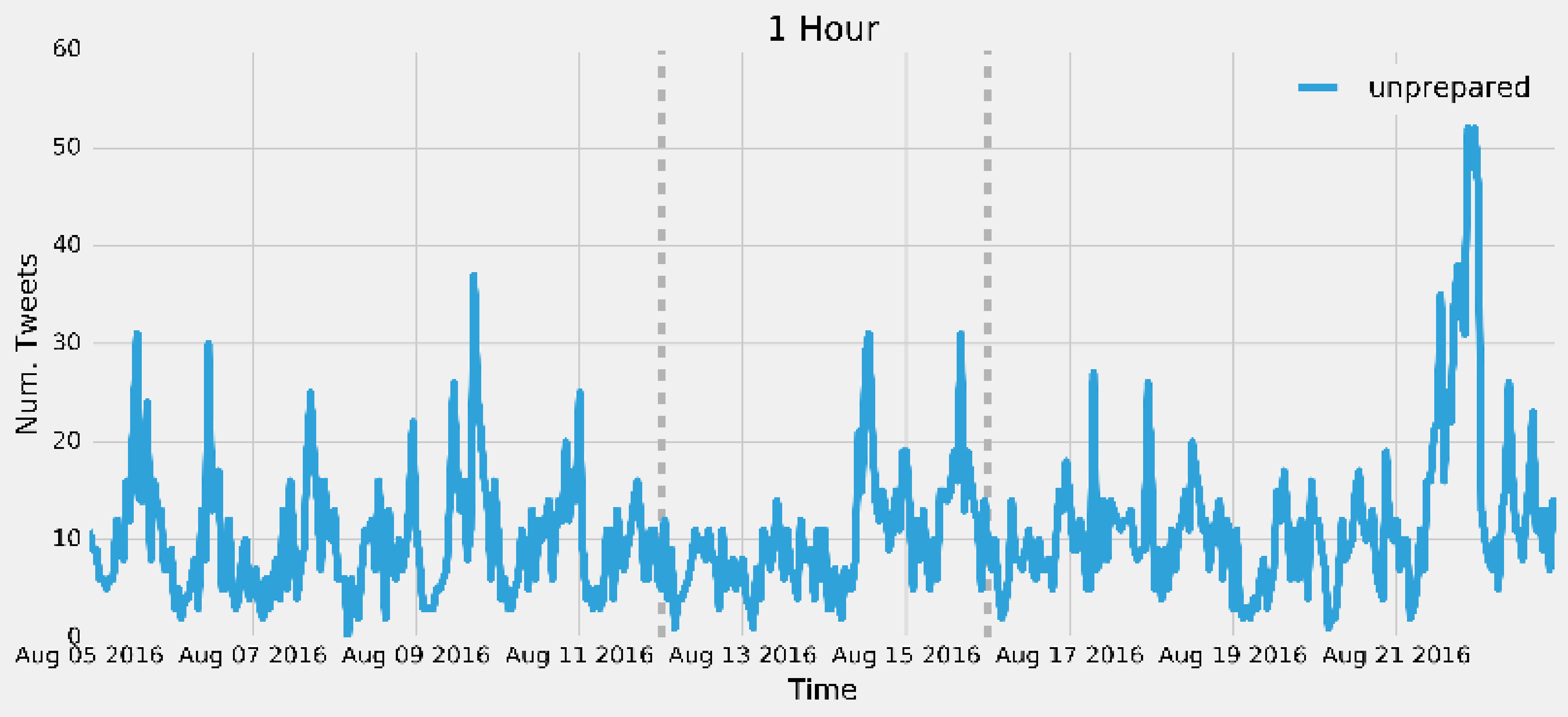
1 Hour
unprepared
Num. Tweets
Time
0
10
20
30
40
50
60
Aug 05 2016
Aug 07 2016
Aug 09 2016
Aug 11 2016
Aug 13 2016
Aug 15 2016
Aug 17 2016
Aug 19 2016
Aug 21 2016

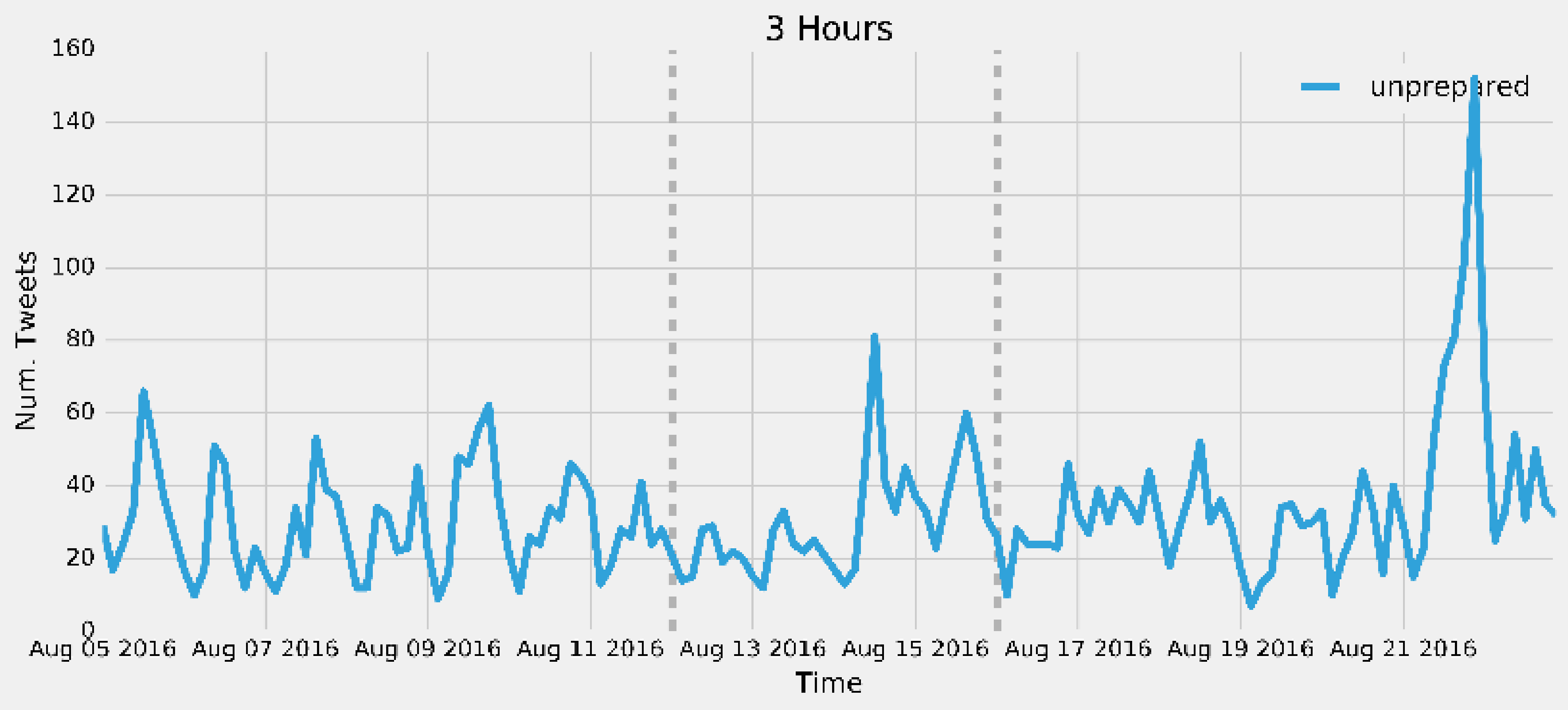
3 Hours
unprepared
Num. Tweets
0
20
40
60
80
100
120
140
160
Aug 05 2016
Aug 07 2016
Aug 09 2016
Aug 11 2016
Aug 13 2016
Aug 15 2016
Aug 17 2016
Aug 19 2016
Aug 21 2016
Time

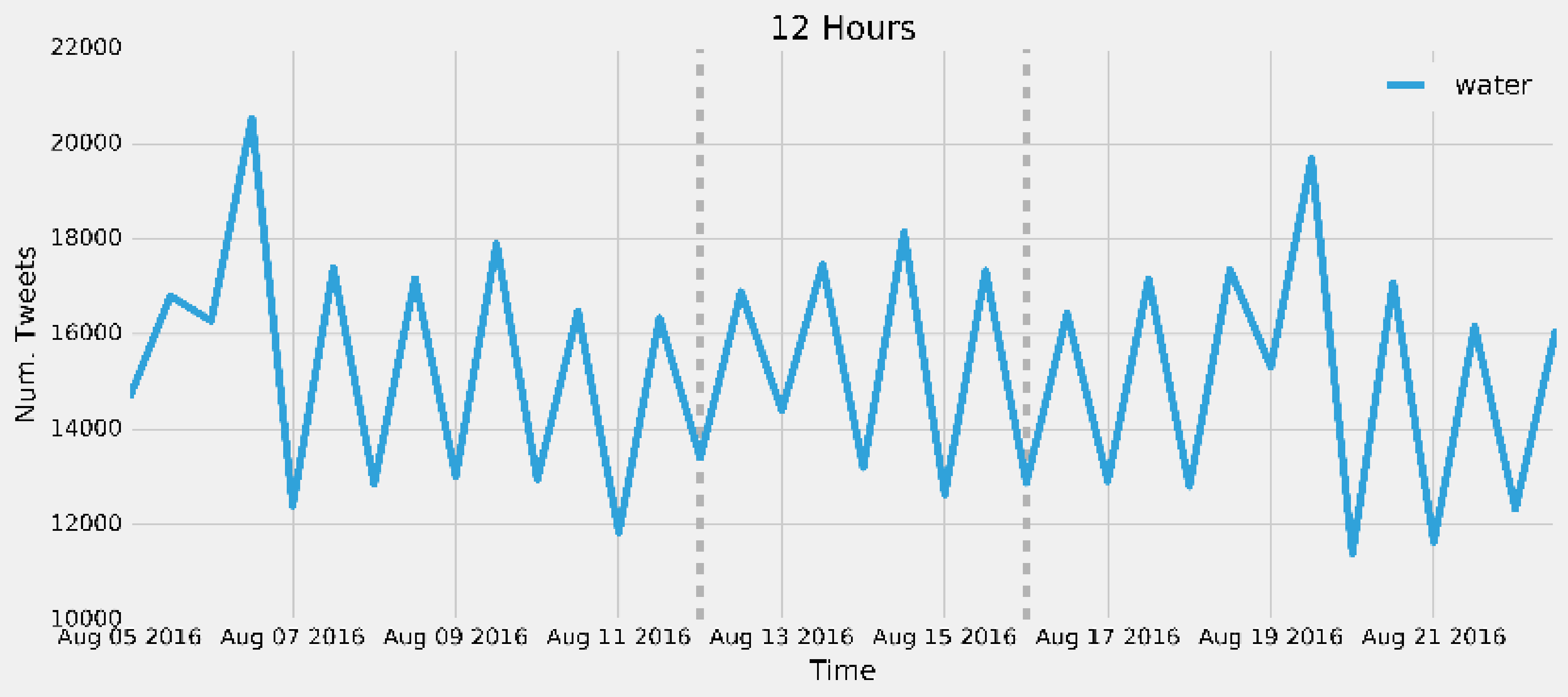

12 Hours
water
22000
20000
18000
16000
14000
12000
10000
Num. Tweets
Aug 05 2016
Aug 07 2016
Aug 09 2016
Aug 11 2016
Aug 13 2016
Aug 15 2016
Aug 17 2016
Aug 19 2016
Aug 21 2016
Time

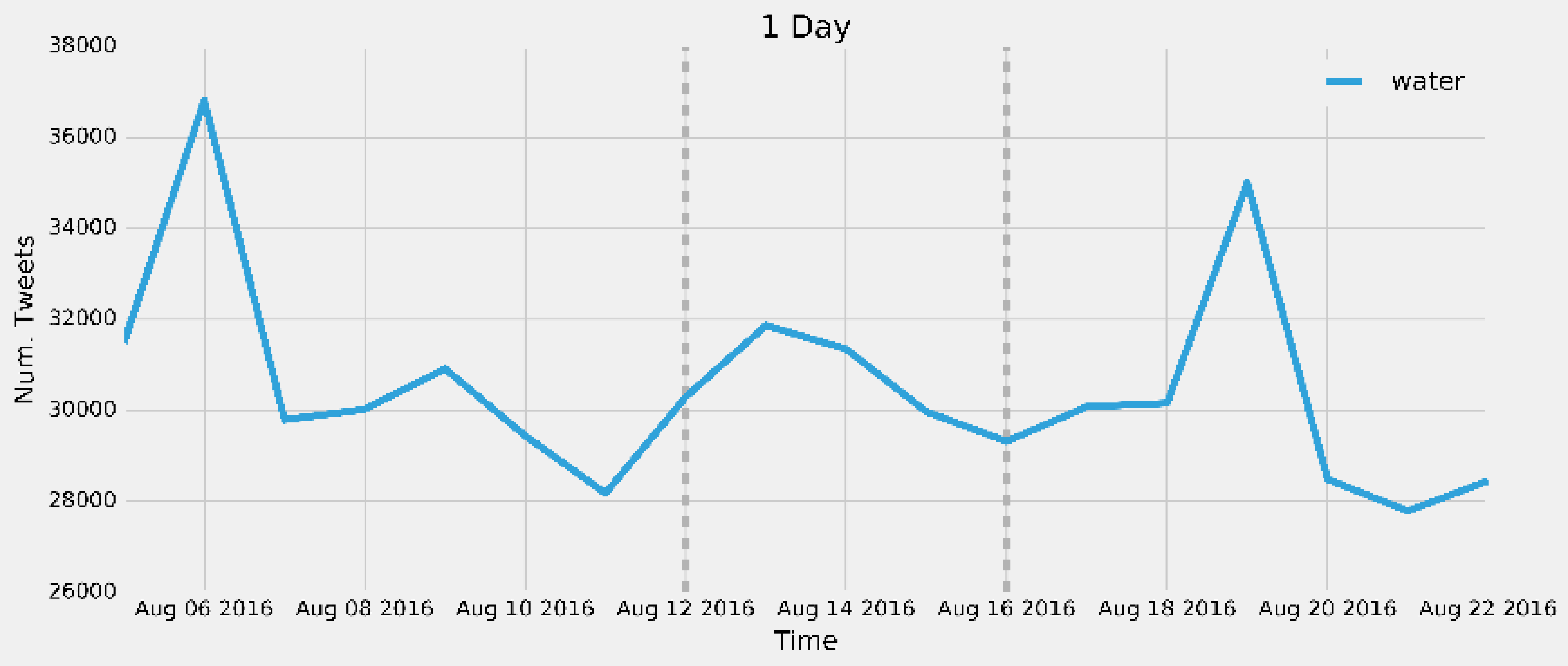
1 Day
water
Num. Tweets
Time
38000
36000
34000
32000
30000
28000
26000
Aug 06 2016
Aug 08 2016
Aug 10 2016
Aug 12 2016
Aug 14 2016
Aug 16 2016
Aug 18 2016
Aug 20 2016
Aug 22 2016

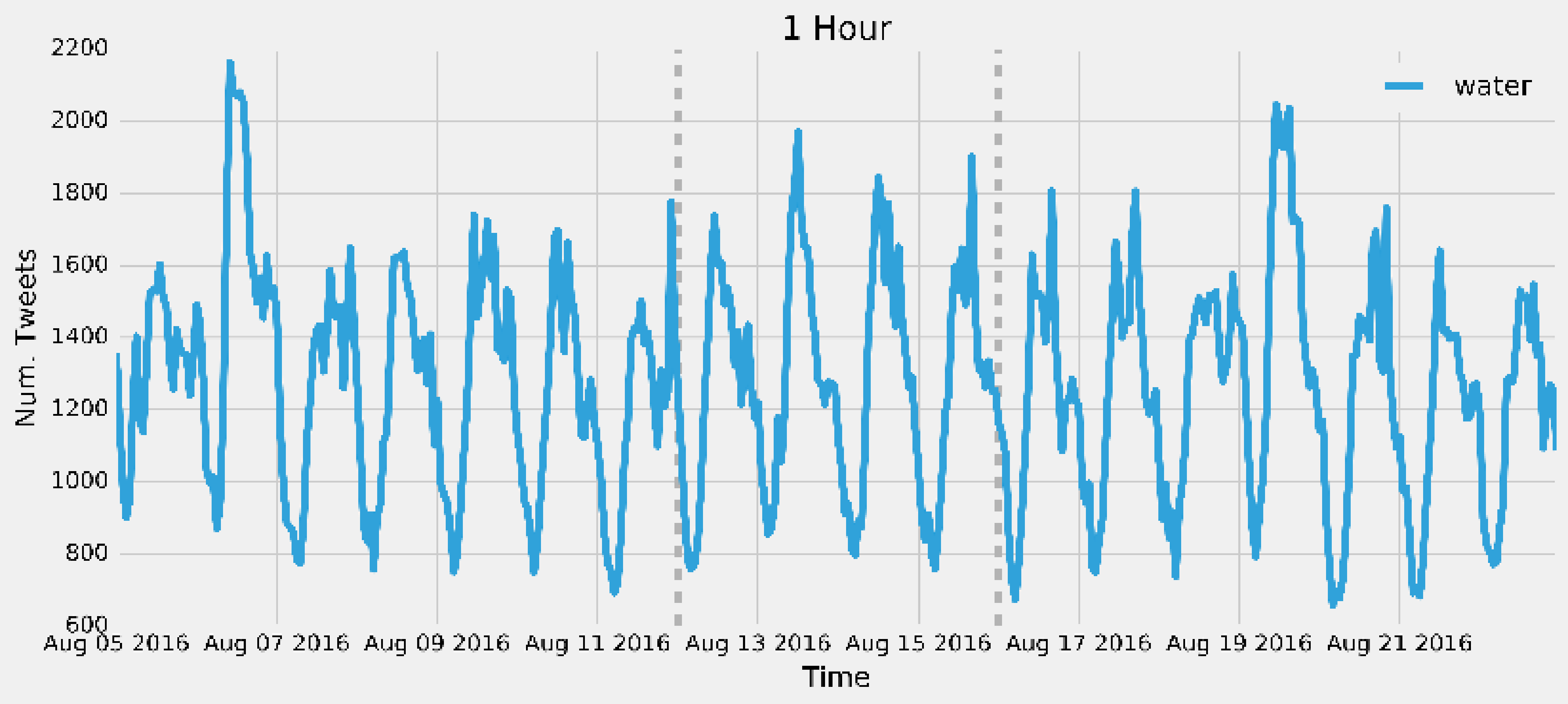

1 Hour
water
2200
2000
1800
1600
1400
1200
1000
800
600
Num. Tweets
Aug 05 2016
Aug 07 2016
Aug 09 2016
Aug 11 2016
Aug 13 2016
Aug 15 2016
Aug 17 2016
Aug 19 2016
Aug 21 2016
Time

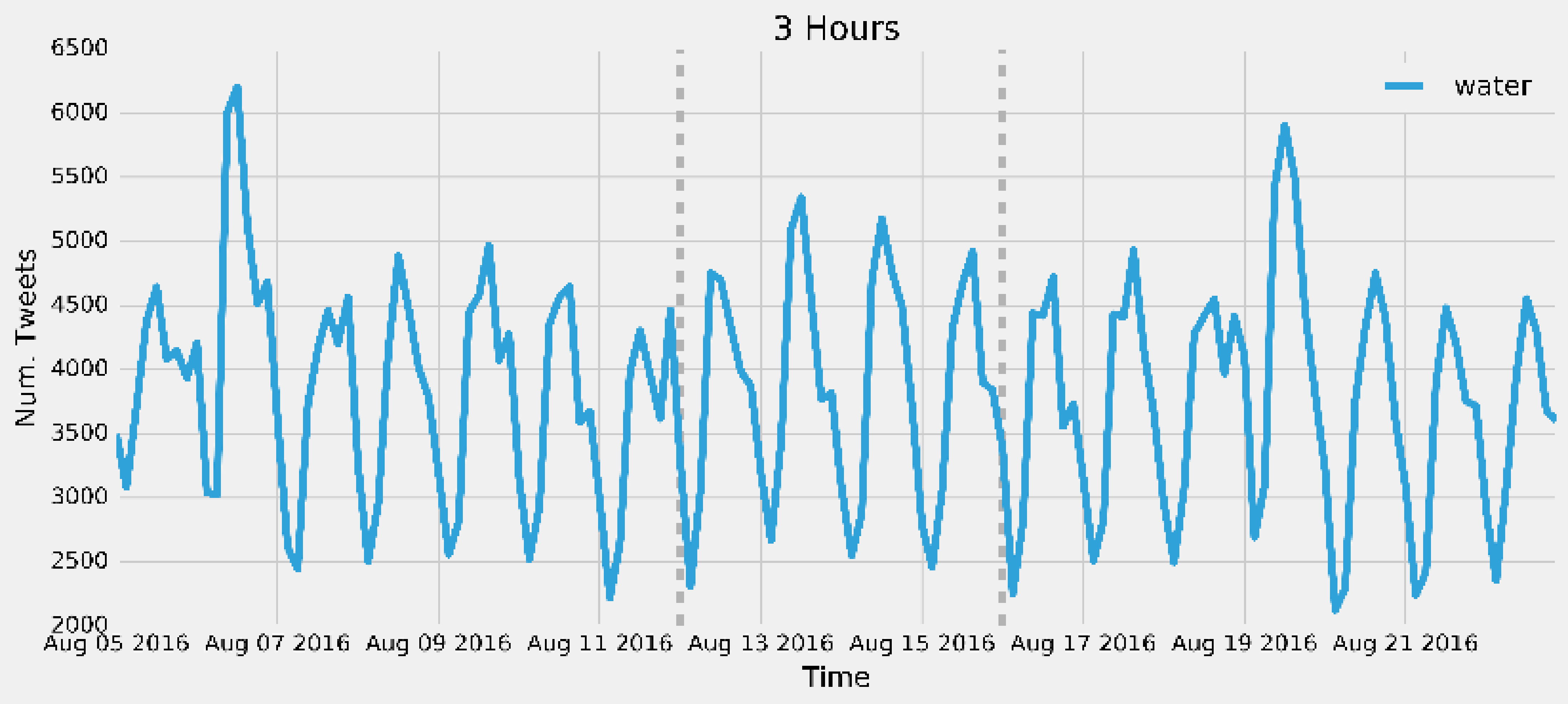

3 Hours
water
Num. Tweets
6500
6000
5500
5000
4500
4000
3500
3000
2500
2000
Aug 05 2016
Aug 07 2016
Aug 09 2016
Aug 11 2016
Aug 13 2016
Aug 15 2016
Aug 17 2016
Aug 19 2016
Aug 21 2016
Time

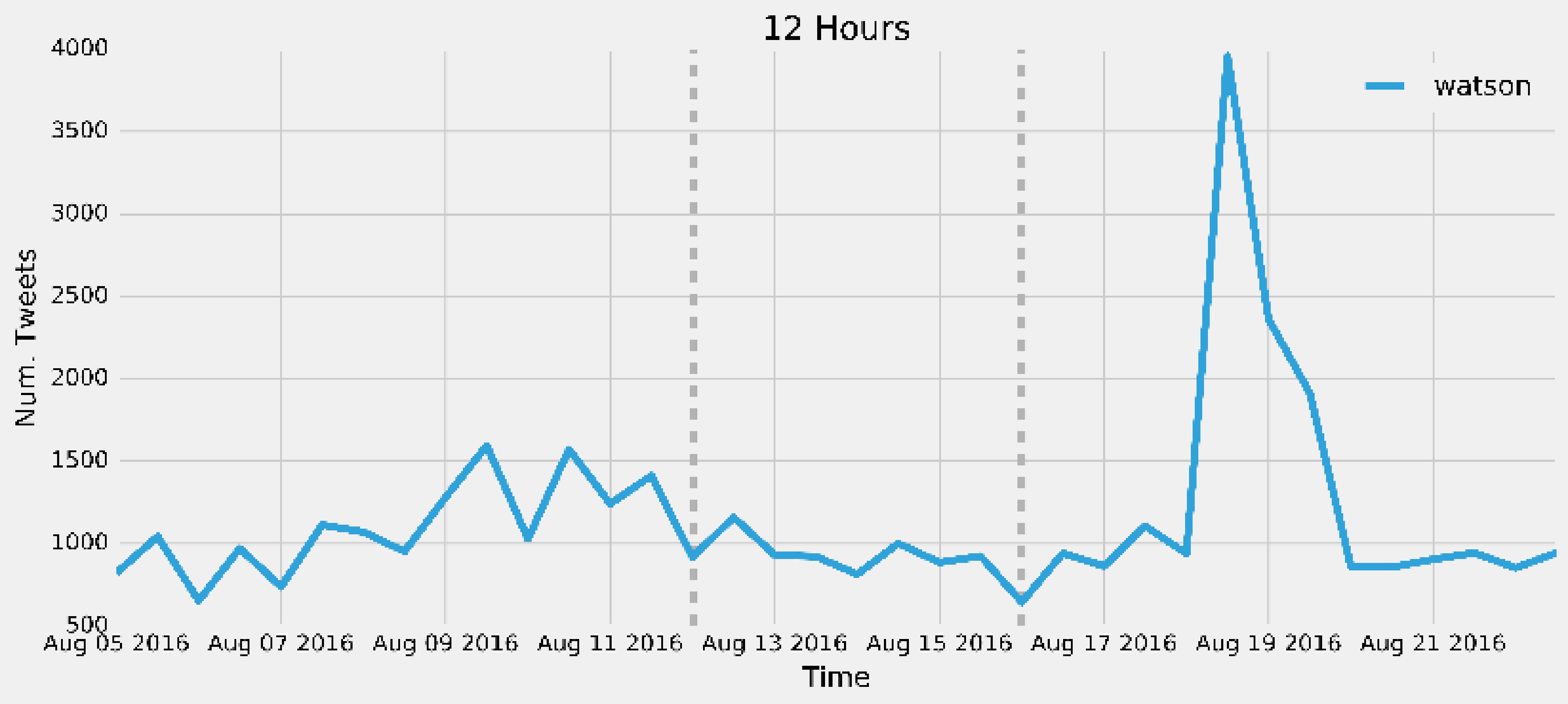
12 Hours
watson
Num. Tweets
4000
3500
3000
2500
2000
1500
1000
500
Aug 05 2016
Aug 07 2016
Aug 09 2016
Aug 11 2016
Aug 13 2016
Aug 15 2016
Aug 17 2016
Aug 19 2016
Aug 21 2016
Time

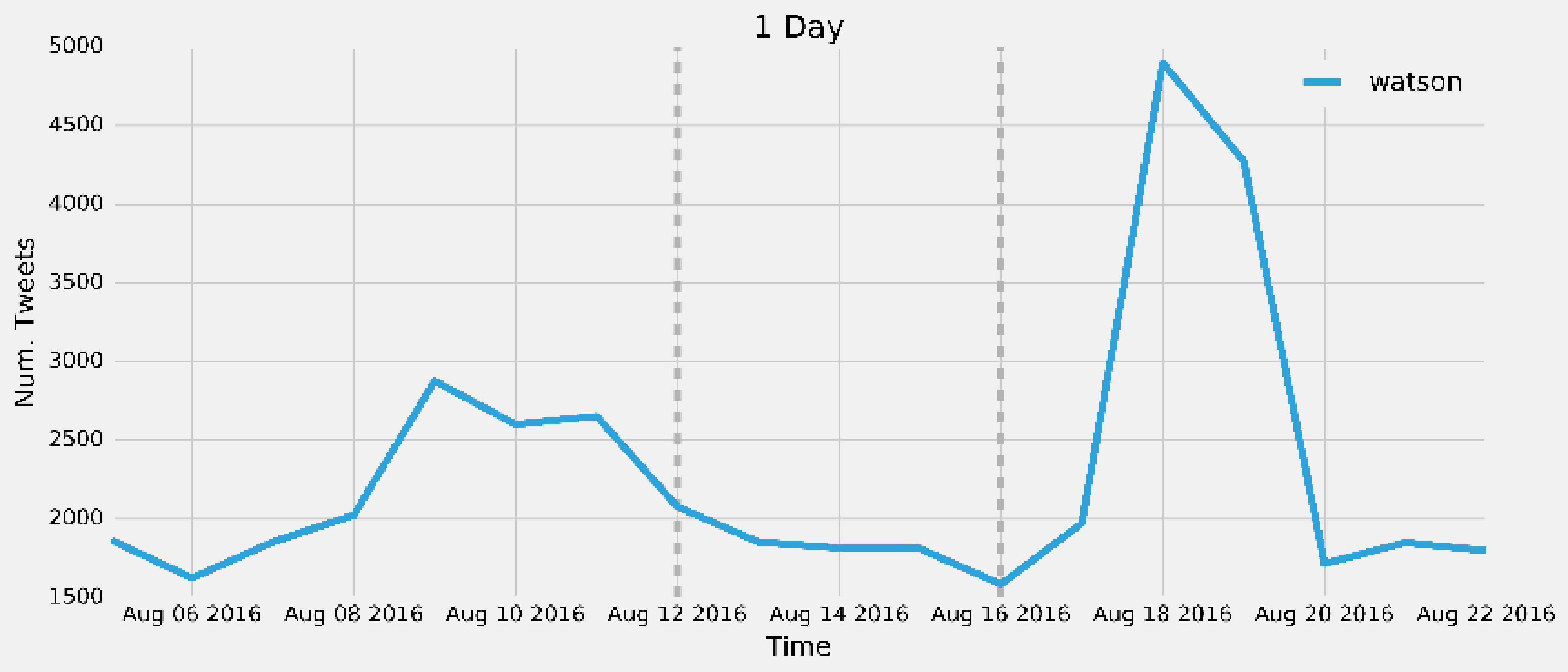

1 Day
5000
4500
4000
3500
3000
2500
2000
1500
Num. Tweets
watson
Aug 06 2016
Aug 08 2016
Aug 10 2016
Aug 12 2016
Aug 14 2016
Aug 16 2016
Aug 18 2016
Aug 20 2016
Aug 22 2016
Time

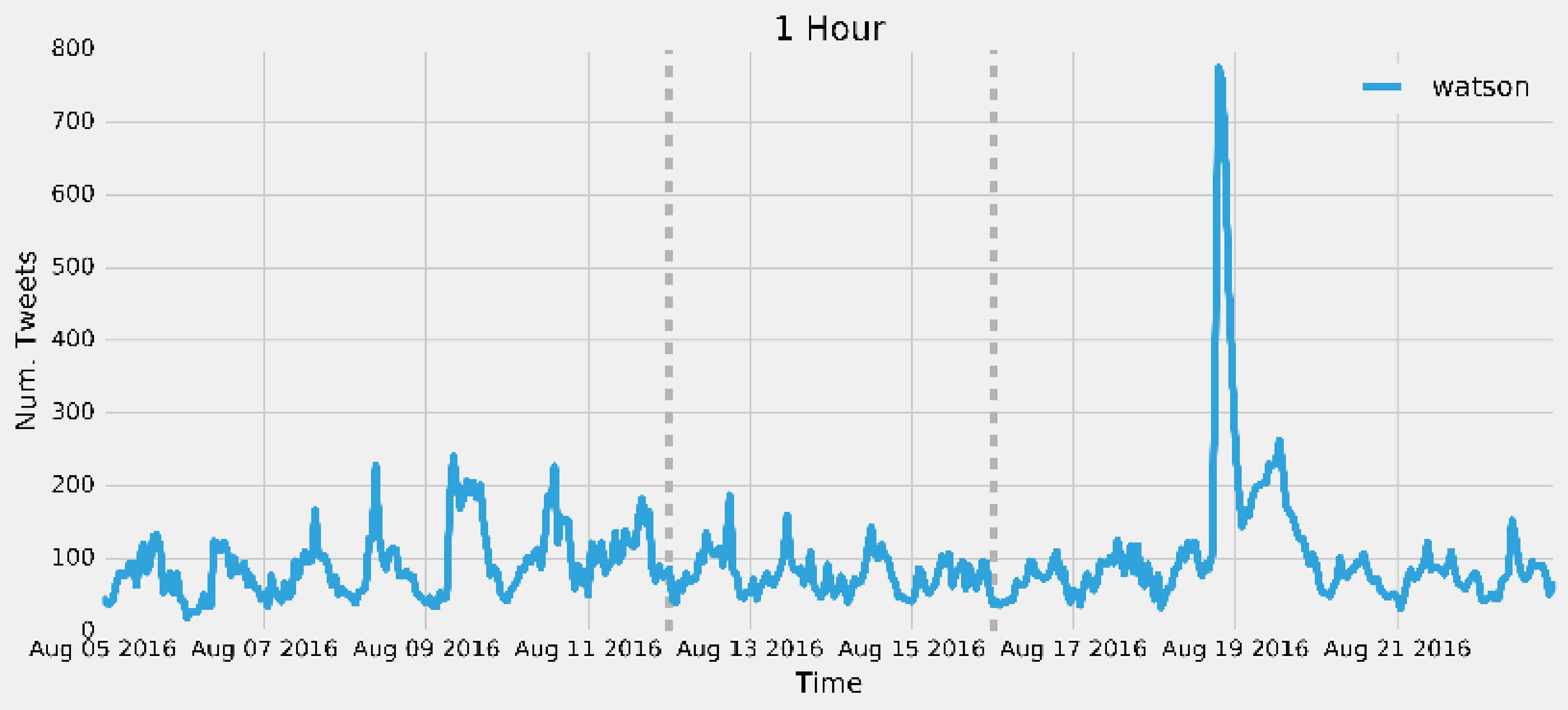

1 Hour
watson
Num. Tweets
0
100
200
300
400
500
600
700
800
Aug 05 2016
Aug 07 2016
Aug 09 2016
Aug 11 2016
Aug 13 2016
Aug 15 2016
Aug 17 2016
Aug 19 2016
Aug 21 2016
Time

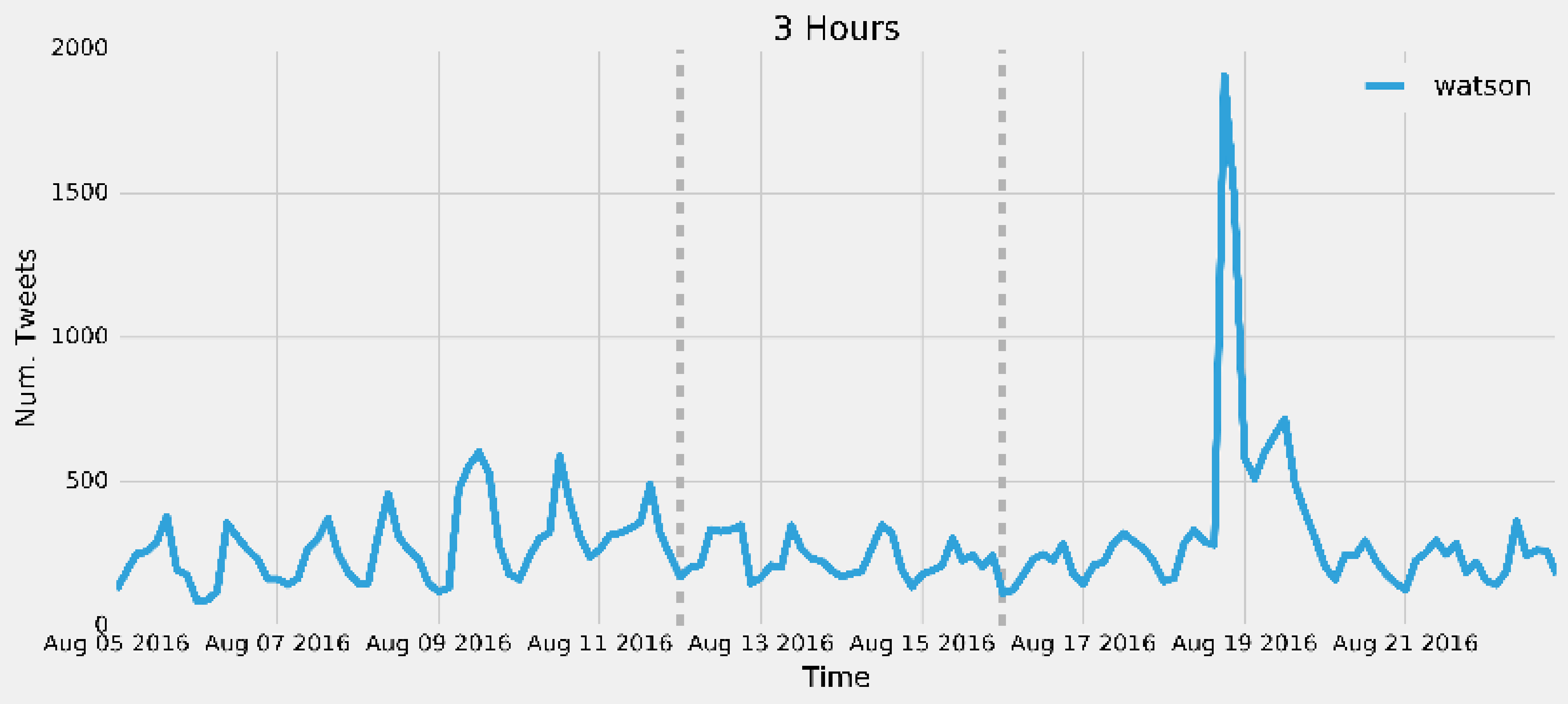

3 Hours
watson
Num. Tweets
2000
1500
1000
500
0
Aug 05 2016
Aug 07 2016
Aug 09 2016
Aug 11 2016
Aug 13 2016
Aug 15 2016
Aug 17 2016
Aug 19 2016
Aug 21 2016
Time

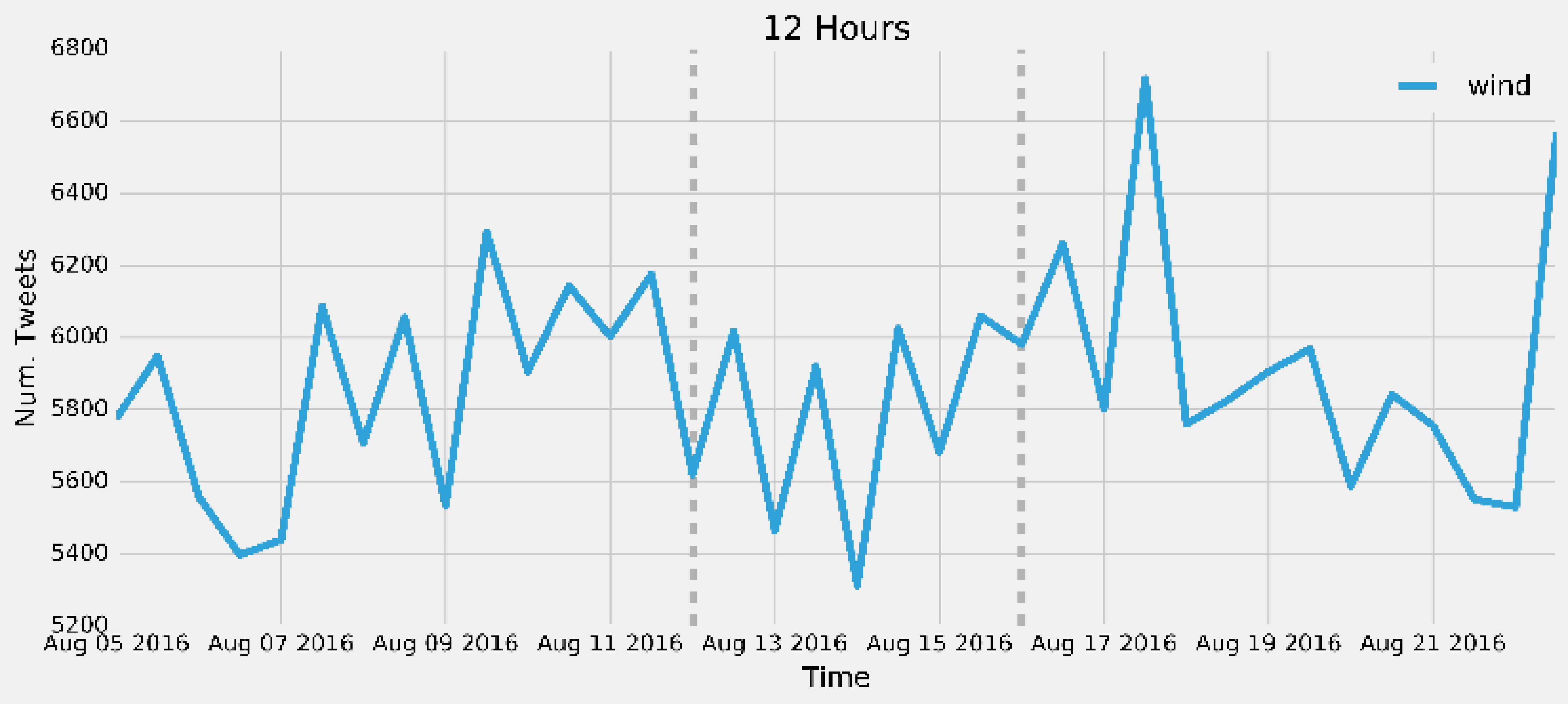

12 Hours
wind
Num. Tweets
6800
6600
6400
6200
6000
5800
5600
5400
5200
Aug 05 2016
Aug 07 2016
Aug 09 2016
Aug 11 2016
Aug 13 2016
Aug 15 2016
Aug 17 2016
Aug 19 2016
Aug 21 2016
Time

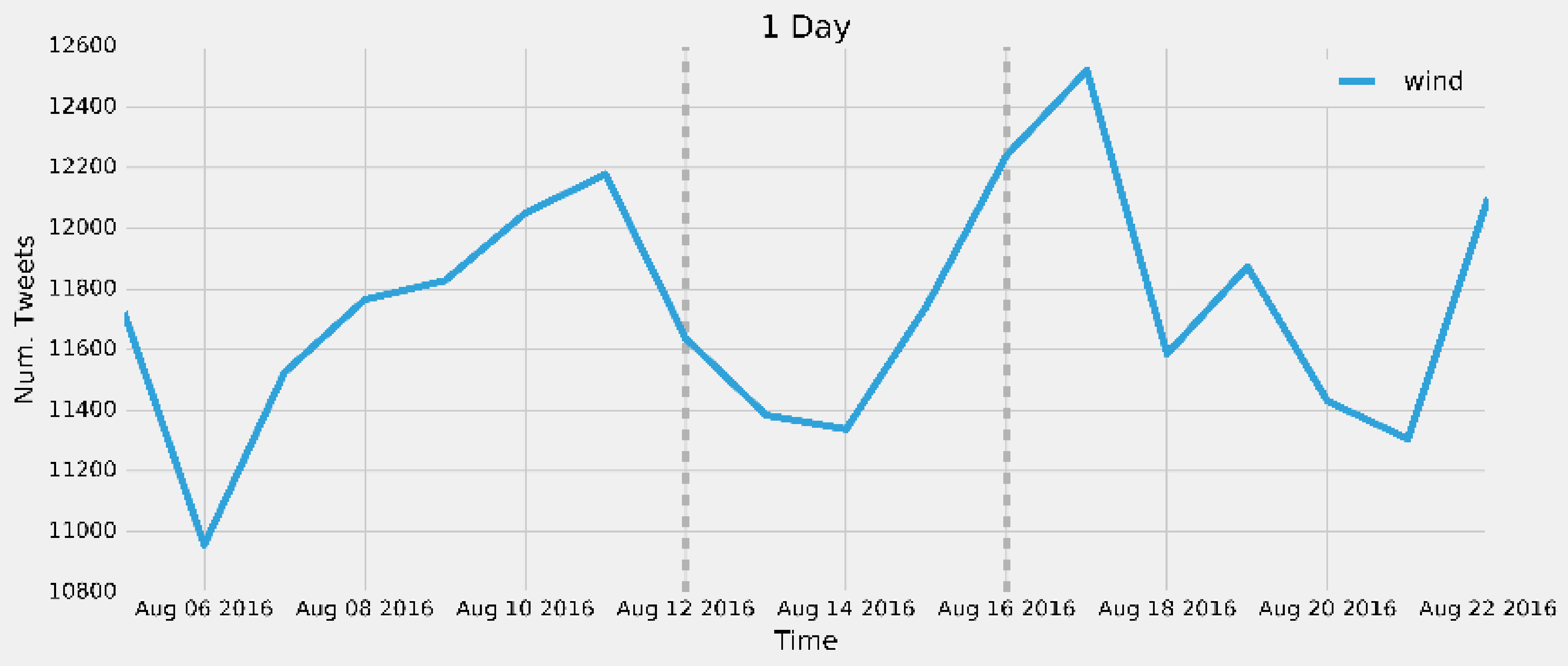
1 Day
wind
Num. Tweets
12600
12400
12200
12000
11800
11600
11400
11200
11000
10800
Aug 06 2016
Aug 08 2016
Aug 10 2016
Aug 12 2016
Aug 14 2016
Aug 16 2016
Aug 18 2016
Aug 20 2016
Aug 22 2016
Time

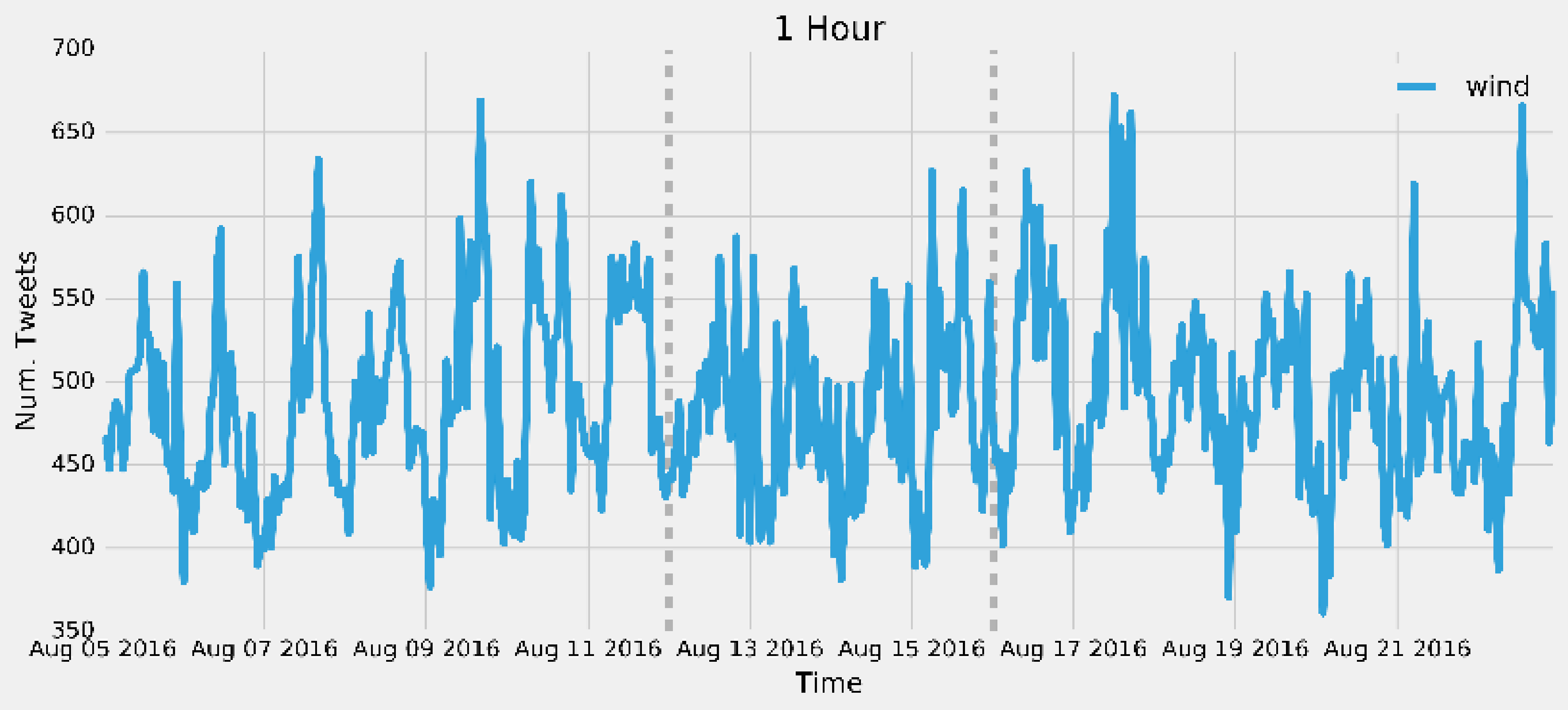

1 Hour
wind
Num. Tweets
700
650
600
550
500
450
400
350
Aug 05 2016
Aug 07 2016
Aug 09 2016
Aug 11 2016
Aug 13 2016
Aug 15 2016
Aug 17 2016
Aug 19 2016
Aug 21 2016
Time

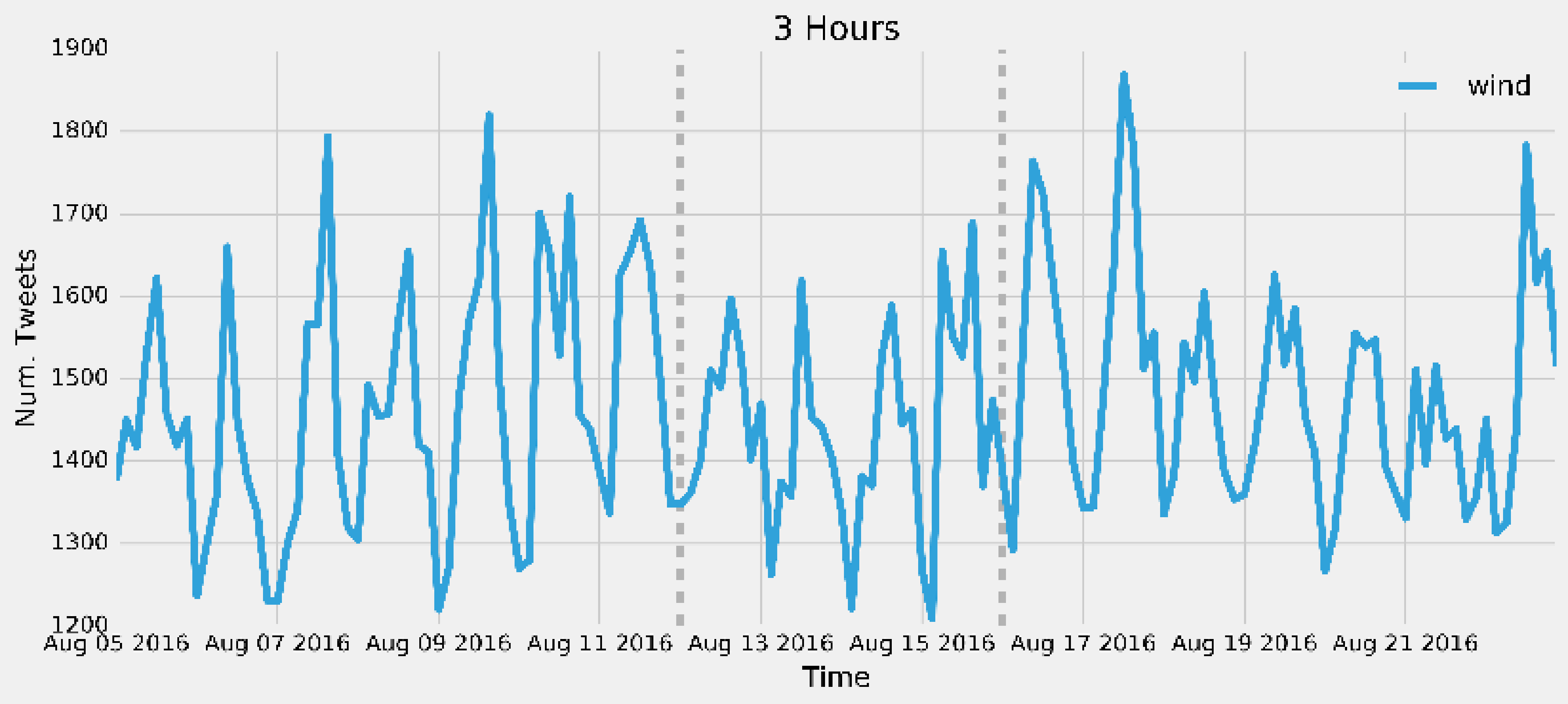

3 Hours
1900
1800
1700
1600
1500
1400
1300
1200
Num. Tweets
wind
Aug 05 2016
Aug 07 2016
Aug 09 2016
Aug 11 2016
Aug 13 2016
Aug 15 2016
Aug 17 2016
Aug 19 2016
Aug 21 2016
Time
