# Supplementary material for: Social media usage patterns during natural hazards
Source: PLoS One. 2019 Feb 13;14(2):e0210484. doi: 10.1371/journal.pone.0210484 (PMC6374021; doi:10.1371/journal.pone.0210484)
Supplement: S4 Fig — (PDF) [file pone.0210484.s004.pdf]

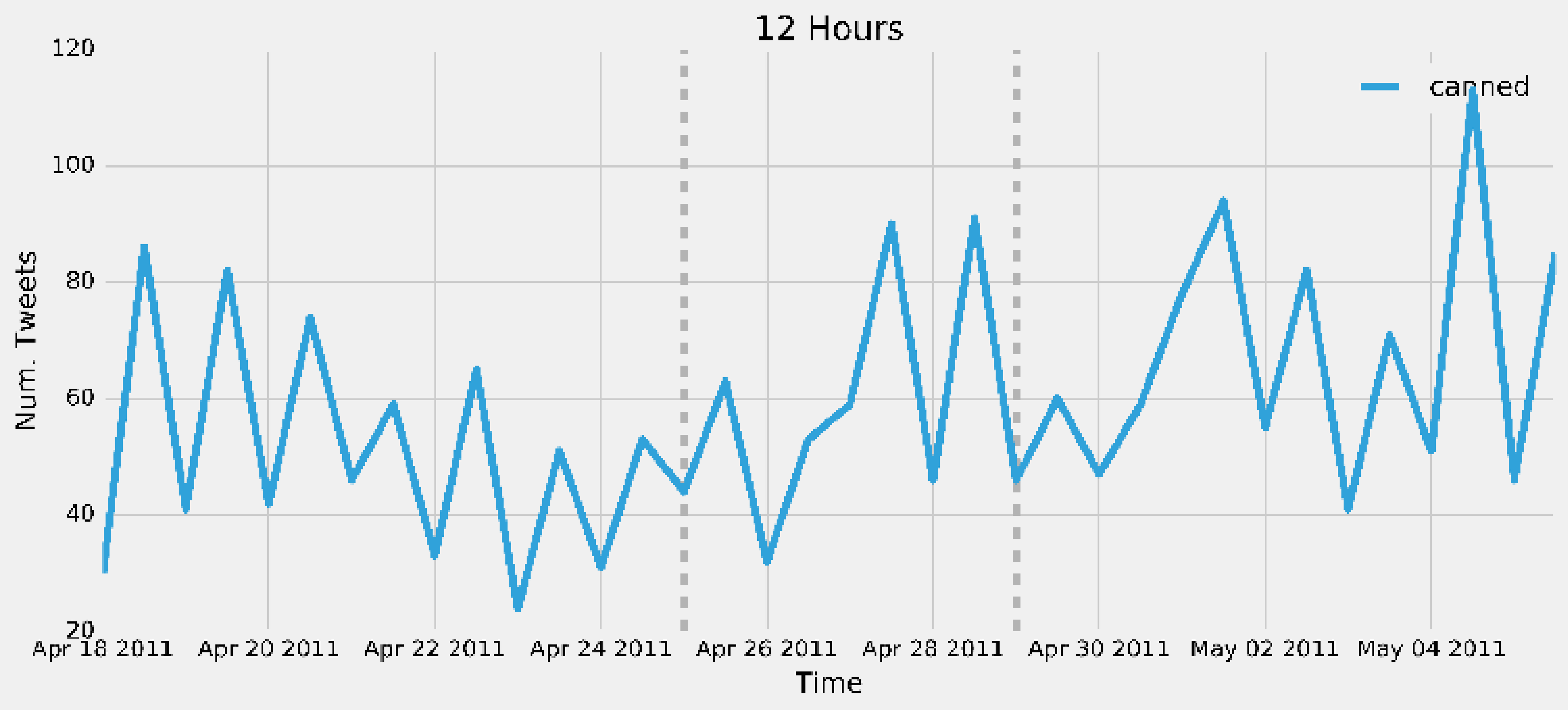

12 Hours
canned
Num. Tweets
120
100
80
60
40
20
Apr 18 2011
Apr 20 2011
Apr 22 2011
Apr 24 2011
Apr 26 2011
Apr 28 2011
Apr 30 2011
May 02 2011
May 04 2011
Time

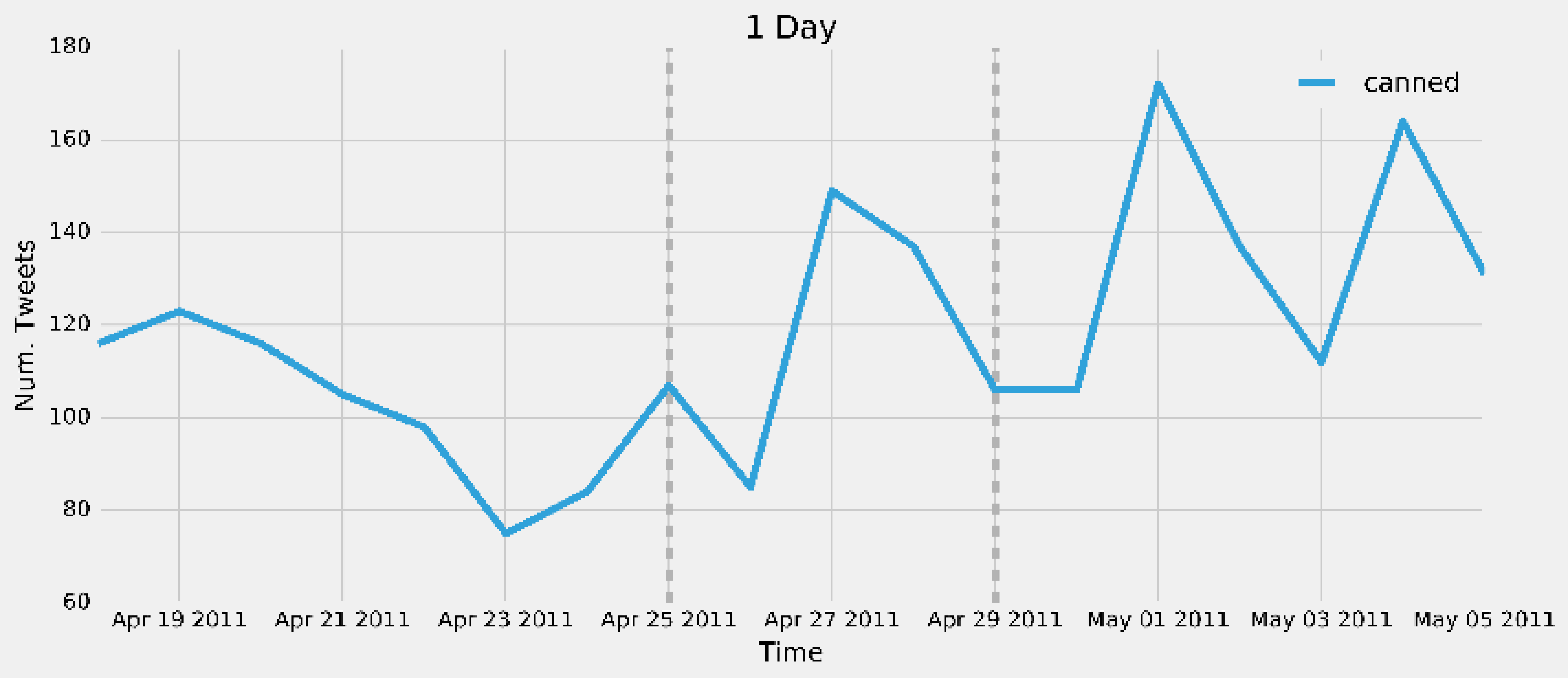
1 Day
canned
Num. Tweets
180
160
140
120
100
80
60
Apr 19 2011
Apr 21 2011
Apr 23 2011
Apr 25 2011
Apr 27 2011
Apr 29 2011
May 01 2011
May 03 2011
May 05 2011
Time

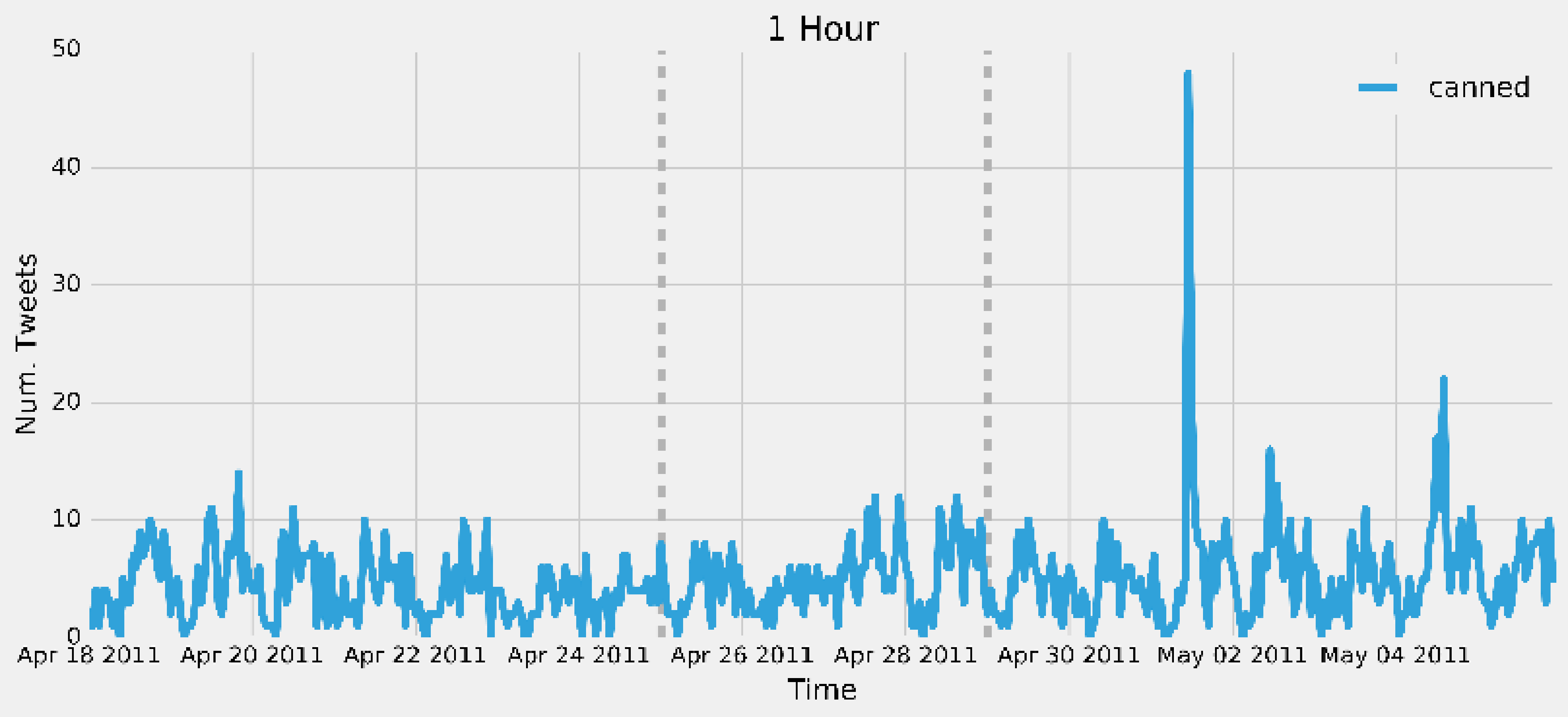
1 Hour
canned
Num. Tweets
0
10
20
30
40
50
Apr 18 2011
Apr 20 2011
Apr 22 2011
Apr 24 2011
Apr 26 2011
Apr 28 2011
Apr 30 2011
May 02 2011
May 04 2011
Time

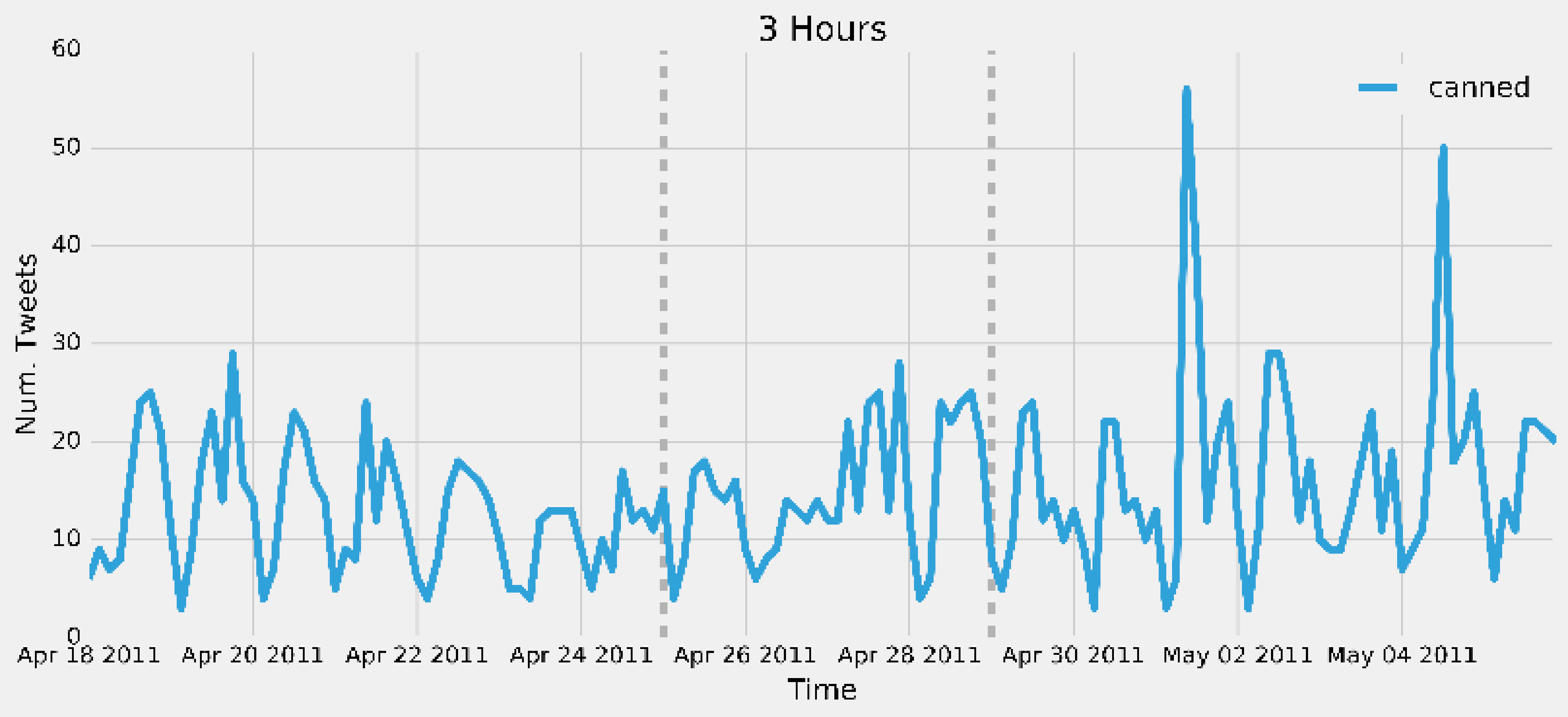
3 Hours
canned
Num. Tweets
0
10
20
30
40
50
60
Apr 18 2011
Apr 20 2011
Apr 22 2011
Apr 24 2011
Apr 26 2011
Apr 28 2011
Apr 30 2011
May 02 2011
May 04 2011
Time

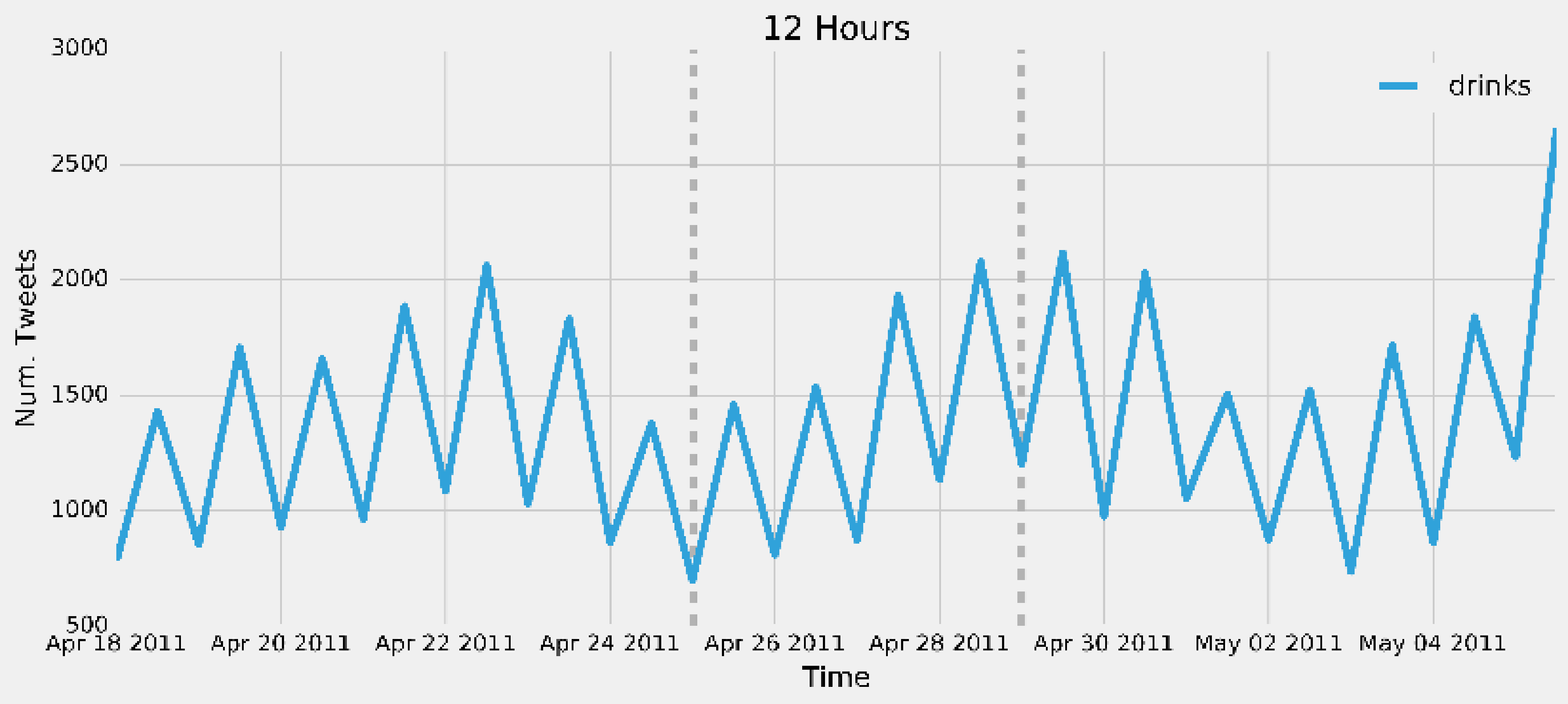
12 Hours
drinks
Num. Tweets
3000
2500
2000
1500
1000
500
Apr 18 2011
Apr 20 2011
Apr 22 2011
Apr 24 2011
Apr 26 2011
Apr 28 2011
Apr 30 2011
May 02 2011
May 04 2011
Time

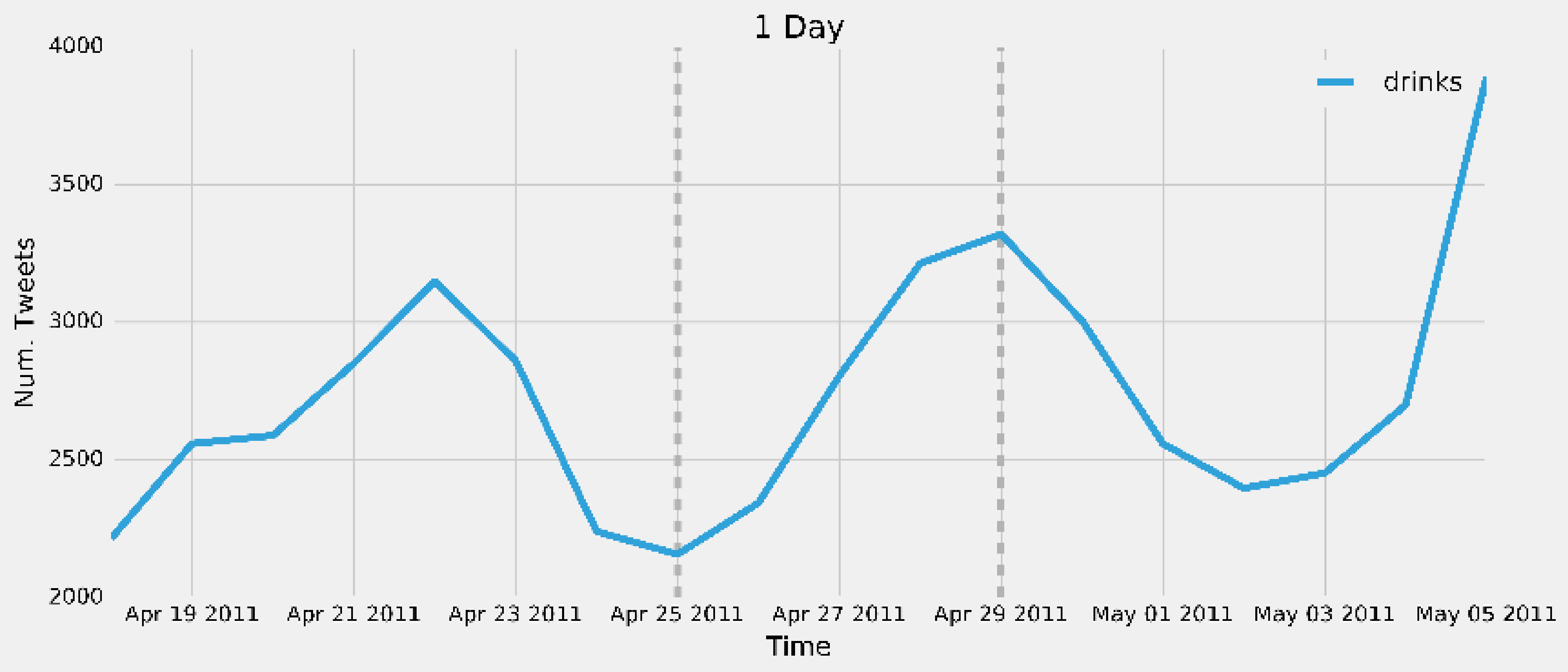

1 Day
drinks
Num. Tweets
Time
2000
2500
3000
3500
4000
Apr 19 2011
Apr 21 2011
Apr 23 2011
Apr 25 2011
Apr 27 2011
Apr 29 2011
May 01 2011
May 03 2011
May 05 2011

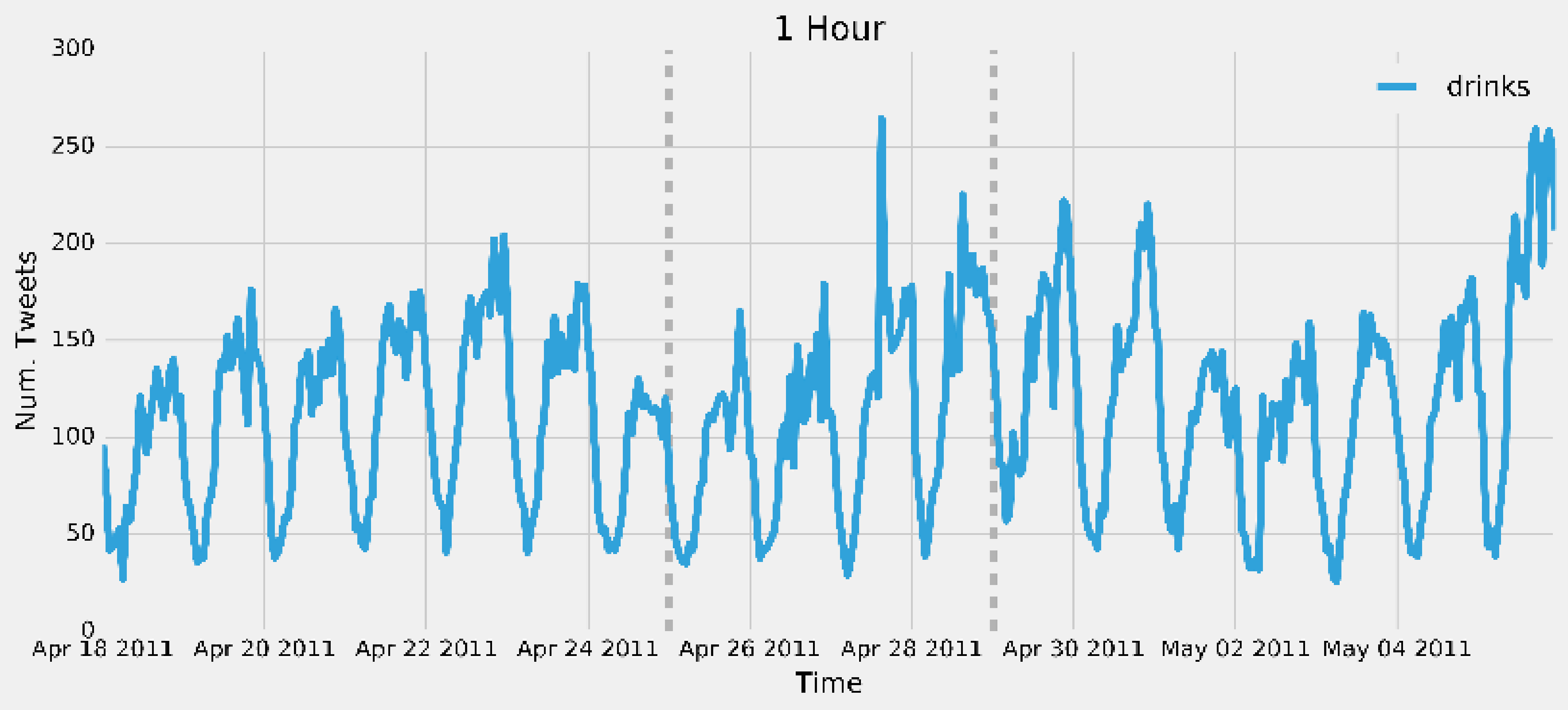

1 Hour
drinks
Num. Tweets
0
50
100
150
200
250
300
Apr 18 2011
Apr 20 2011
Apr 22 2011
Apr 24 2011
Apr 26 2011
Apr 28 2011
Apr 30 2011
May 02 2011
May 04 2011
Time

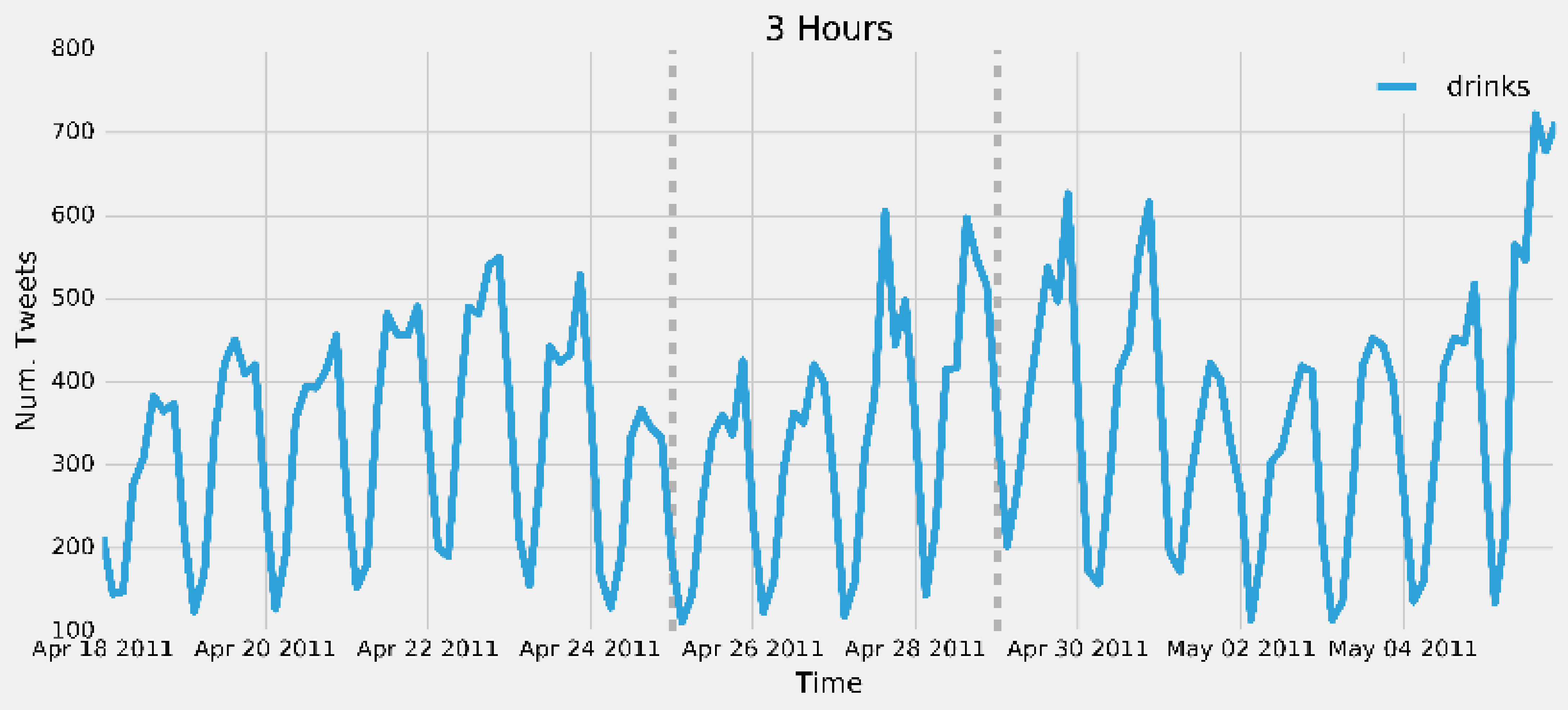

3 Hours
drinks
Num. Tweets
100
200
300
400
500
600
700
800
Apr 18 2011
Apr 20 2011
Apr 22 2011
Apr 24 2011
Apr 26 2011
Apr 28 2011
Apr 30 2011
May 02 2011
May 04 2011
Time

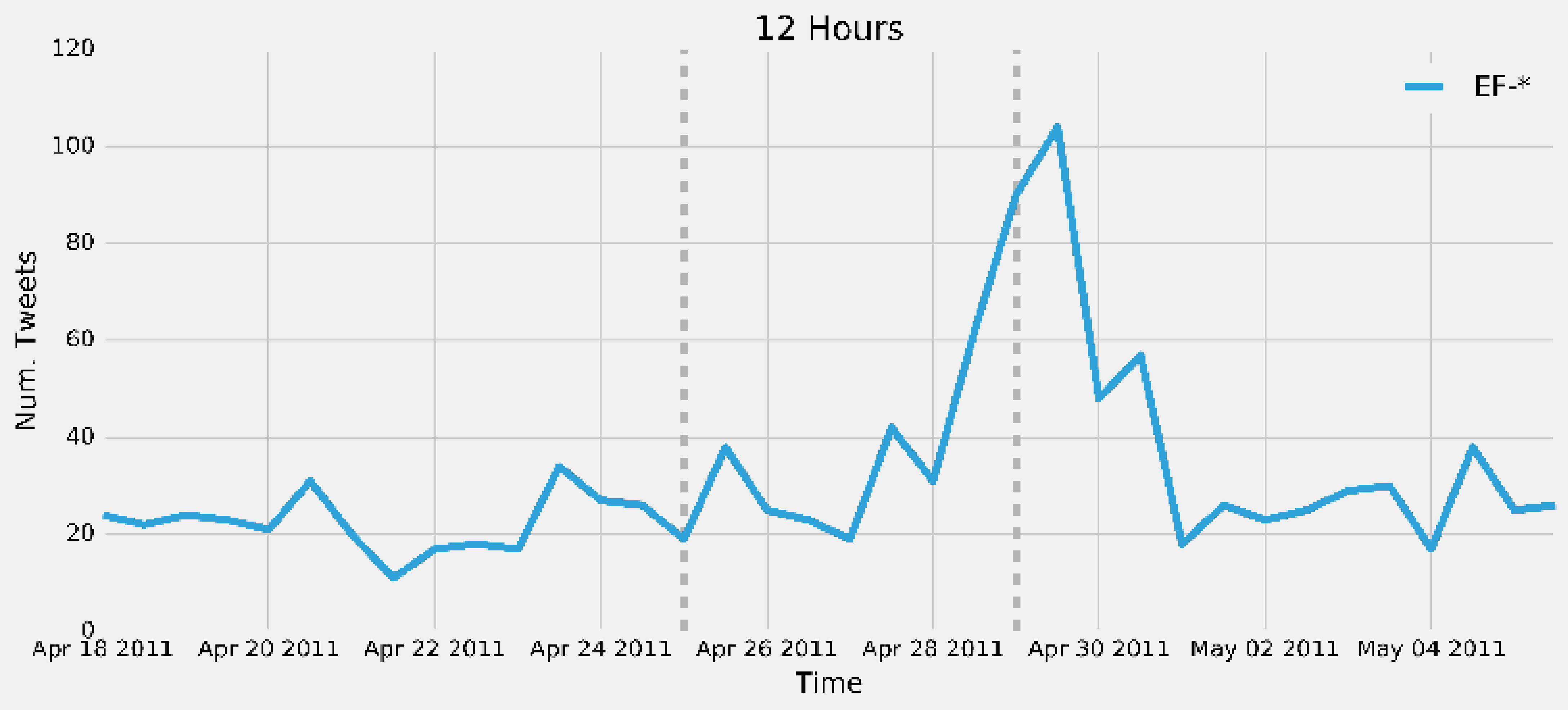
12 Hours
EF-*
Num. Tweets
0
20
40
60
80
100
120
Apr 18 2011
Apr 20 2011
Apr 22 2011
Apr 24 2011
Apr 26 2011
Apr 28 2011
Apr 30 2011
May 02 2011
May 04 2011
Time

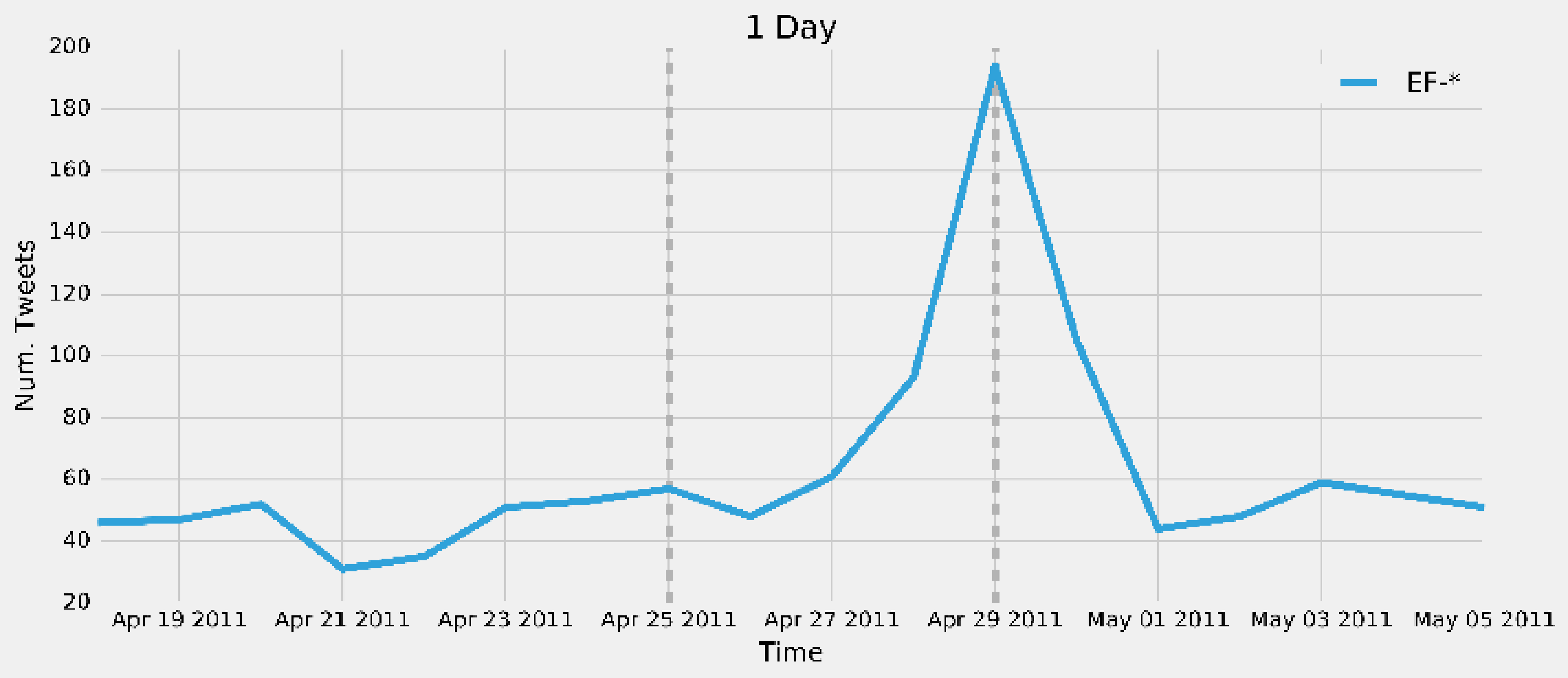
1 Day
EF-*
Num. Tweets
Time
200
180
160
140
120
100
80
60
40
20
Apr 19 2011
Apr 21 2011
Apr 23 2011
Apr 25 2011
Apr 27 2011
Apr 29 2011
May 01 2011
May 03 2011
May 05 2011

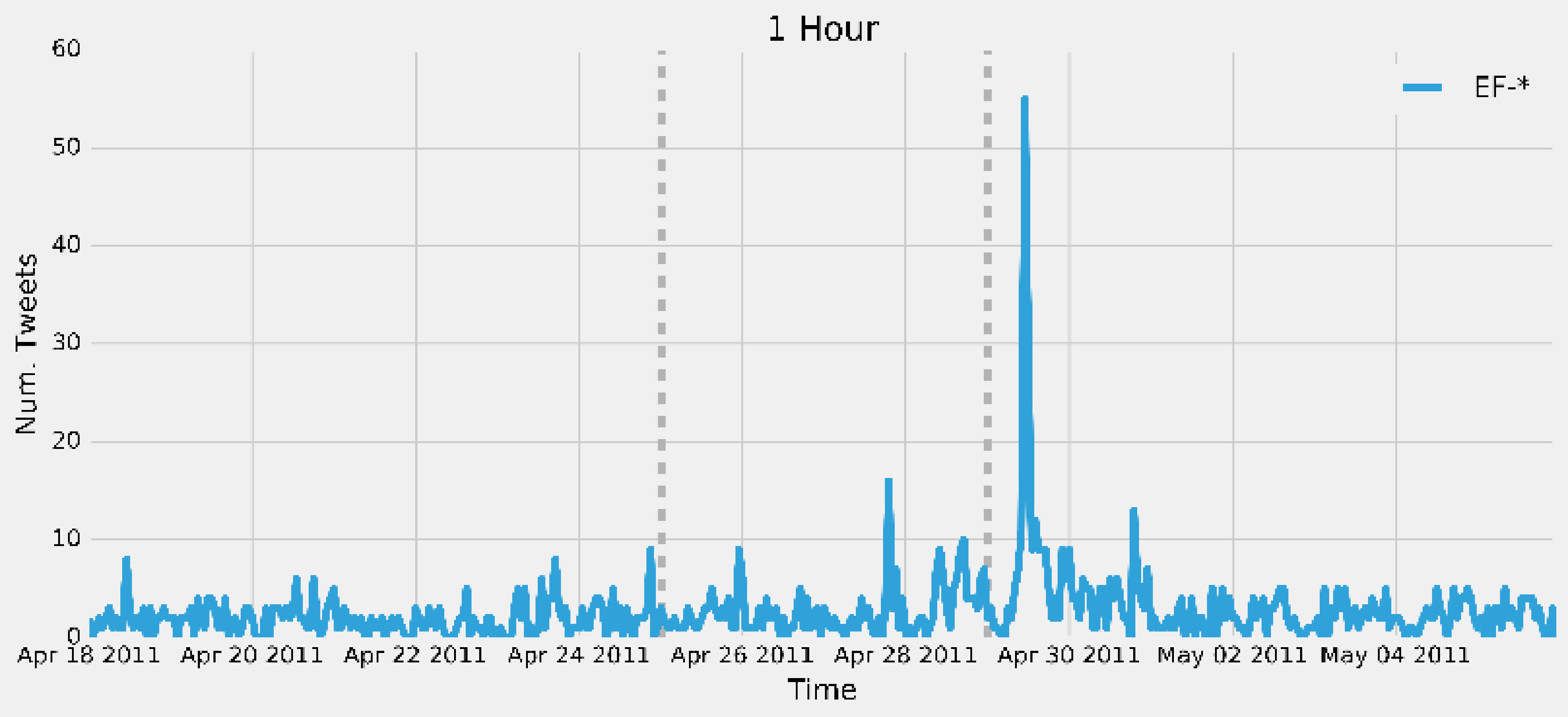
1 Hour
EF-*
60
50
40
30
20
10
0
Num. Tweets
Apr 18 2011
Apr 20 2011
Apr 22 2011
Apr 24 2011
Apr 26 2011
Apr 28 2011
Apr 30 2011
May 02 2011
May 04 2011
Time

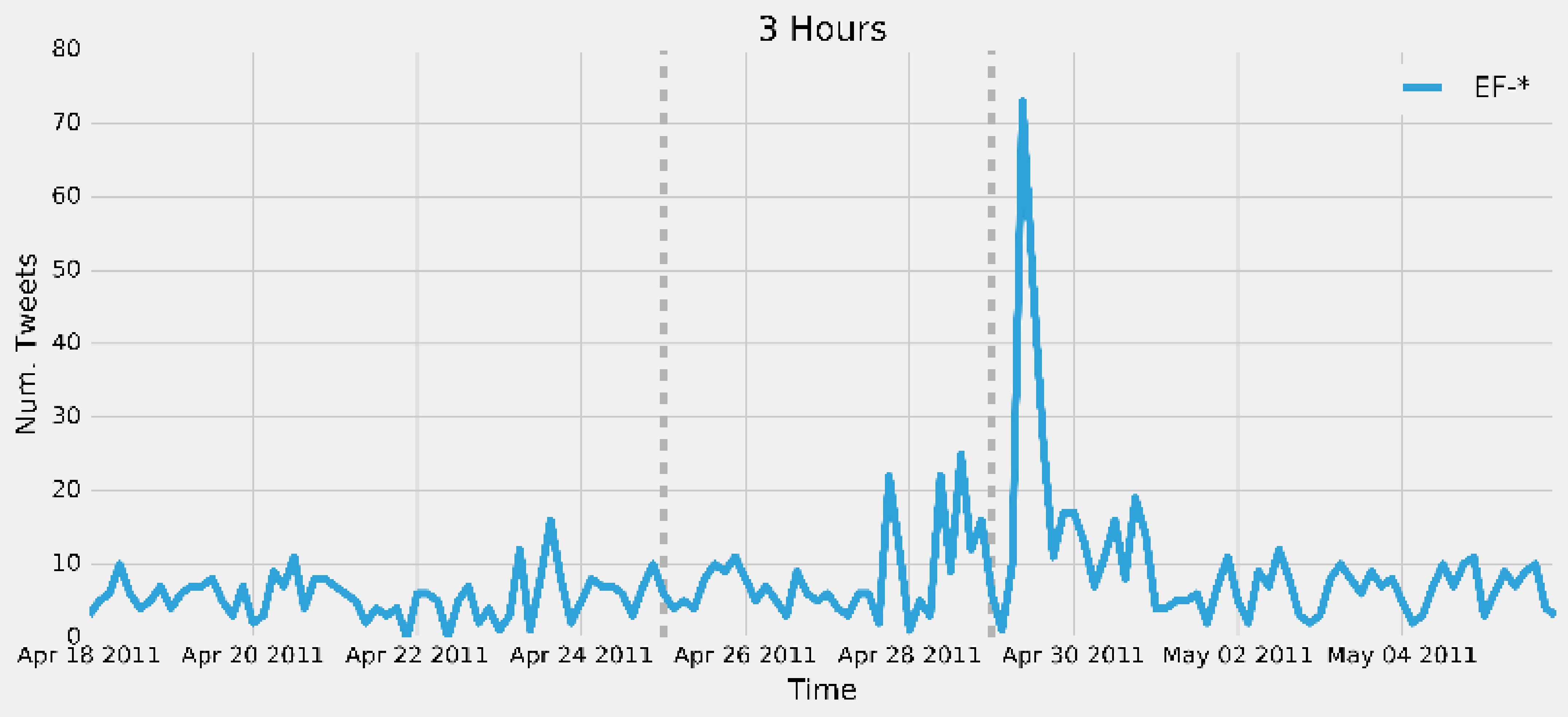

3 Hours
EF-*
Num. Tweets
0
10
20
30
40
50
60
70
80
Apr 18 2011
Apr 20 2011
Apr 22 2011
Apr 24 2011
Apr 26 2011
Apr 28 2011
Apr 30 2011
May 02 2011
May 04 2011
Time

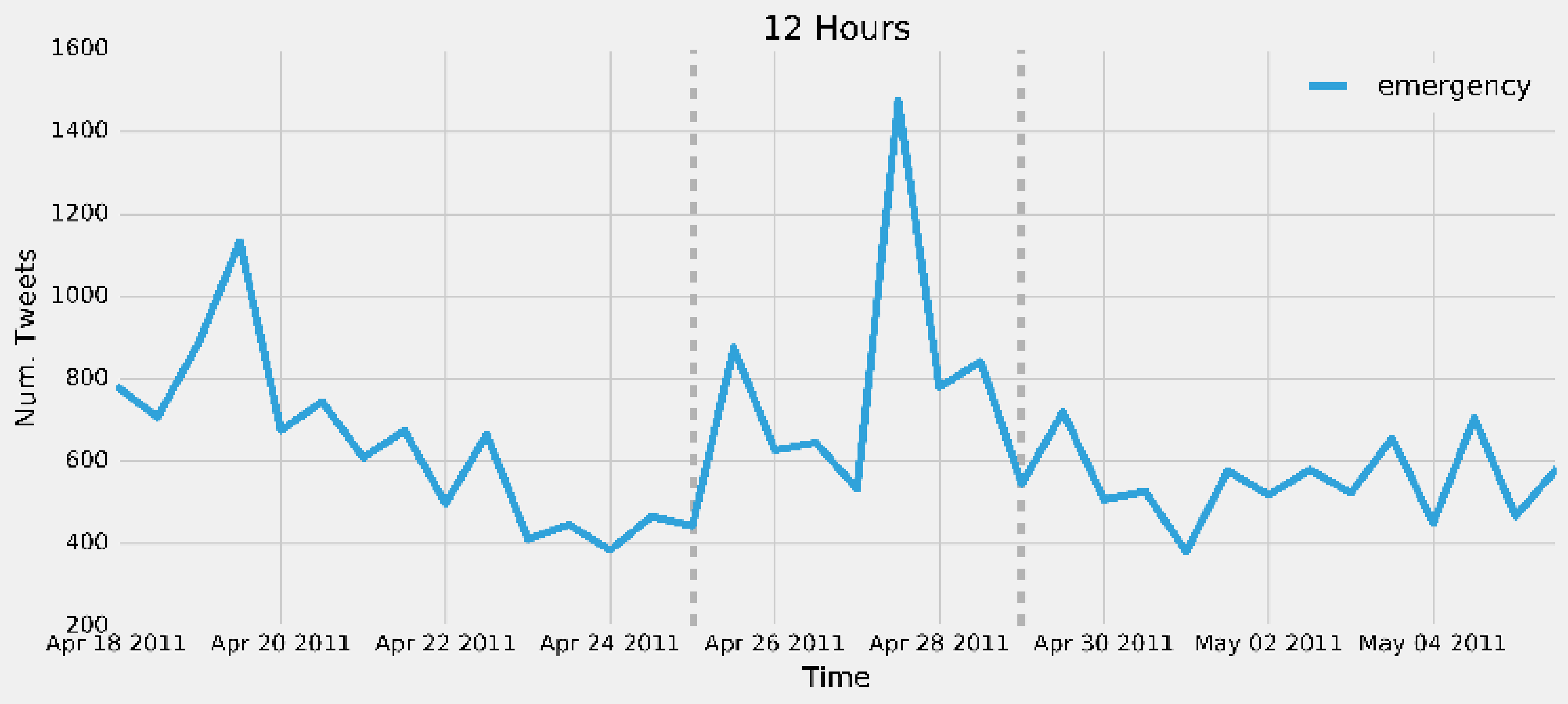

12 Hours
emergency
Num. Tweets
1600
1400
1200
1000
800
600
400
200
Apr 18 2011
Apr 20 2011
Apr 22 2011
Apr 24 2011
Apr 26 2011
Apr 28 2011
Apr 30 2011
May 02 2011
May 04 2011
Time

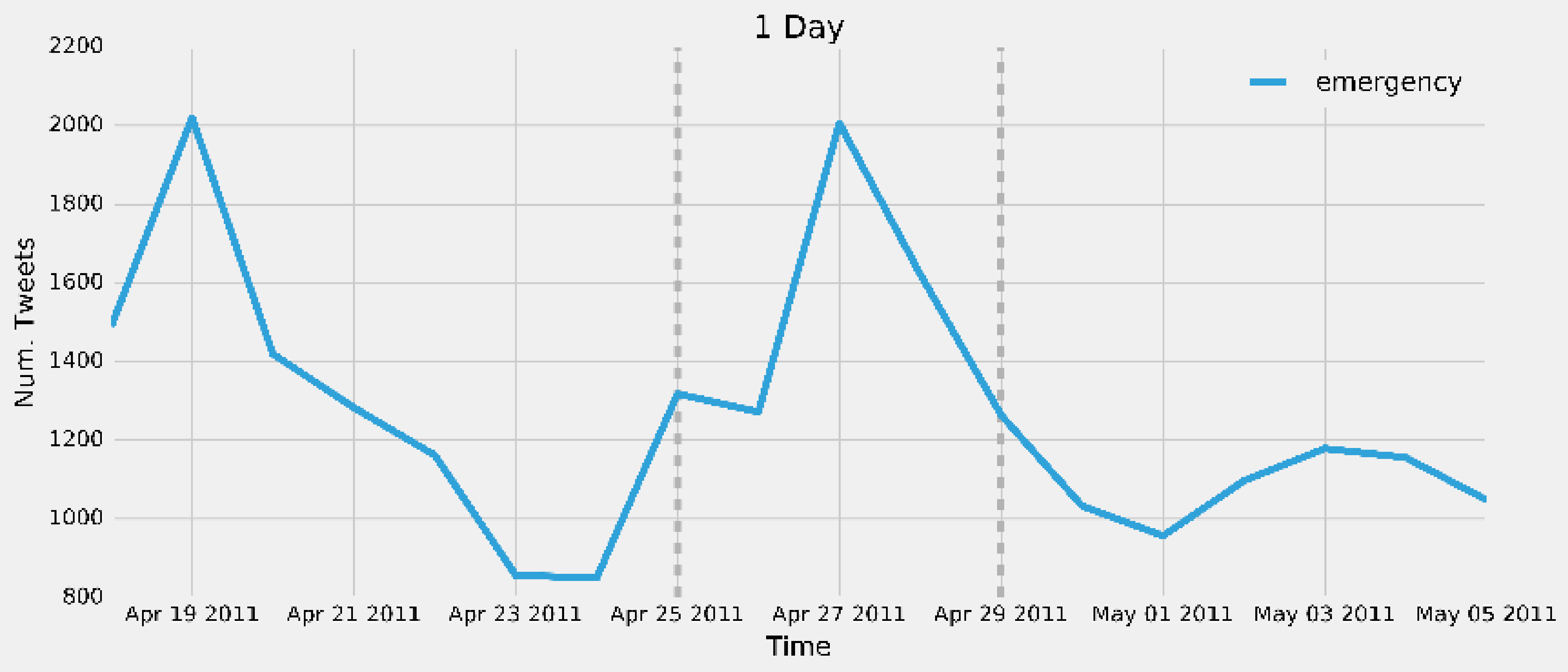
1 Day
emergency
2200
2000
1800
1600
1400
1200
1000
800
Num. Tweets
Apr 19 2011
Apr 21 2011
Apr 23 2011
Apr 25 2011
Apr 27 2011
Apr 29 2011
May 01 2011
May 03 2011
May 05 2011
Time

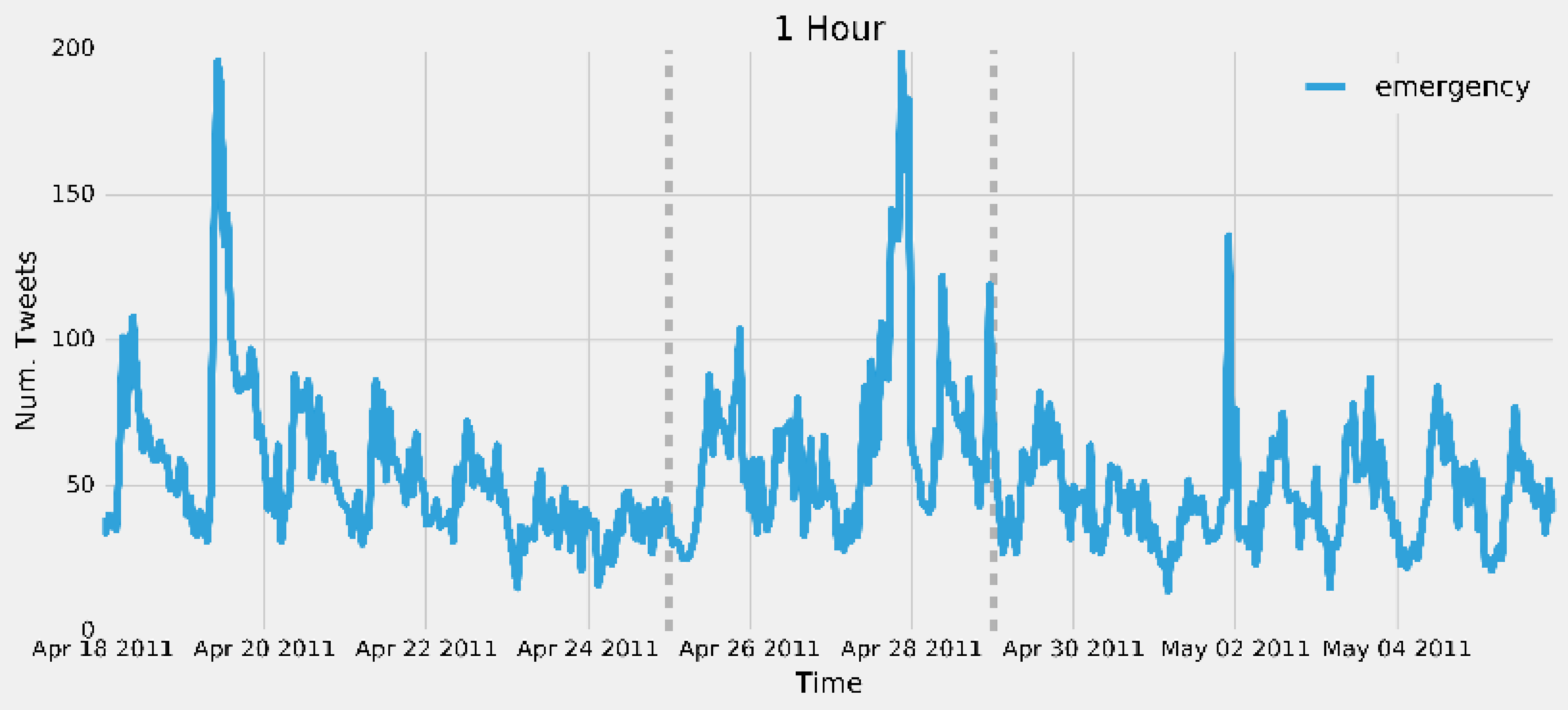

1 Hour
emergency
Num. Tweets
200
150
100
50
0
Apr 18 2011
Apr 20 2011
Apr 22 2011
Apr 24 2011
Apr 26 2011
Apr 28 2011
Apr 30 2011
May 02 2011
May 04 2011
Time

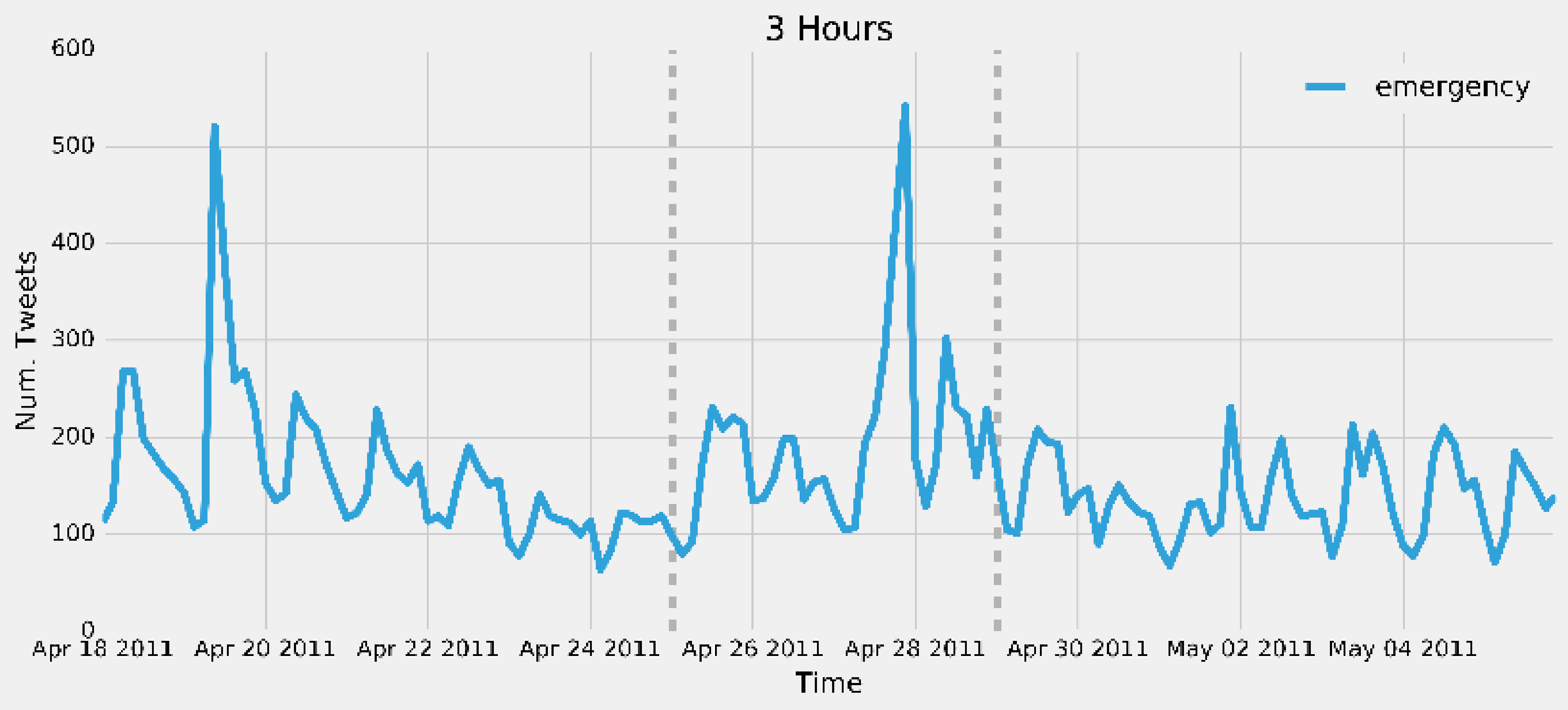
3 Hours
emergency
Num. Tweets
0
100
200
300
400
500
600
Apr 18 2011
Apr 20 2011
Apr 22 2011
Apr 24 2011
Apr 26 2011
Apr 28 2011
Apr 30 2011
May 02 2011
May 04 2011
Time

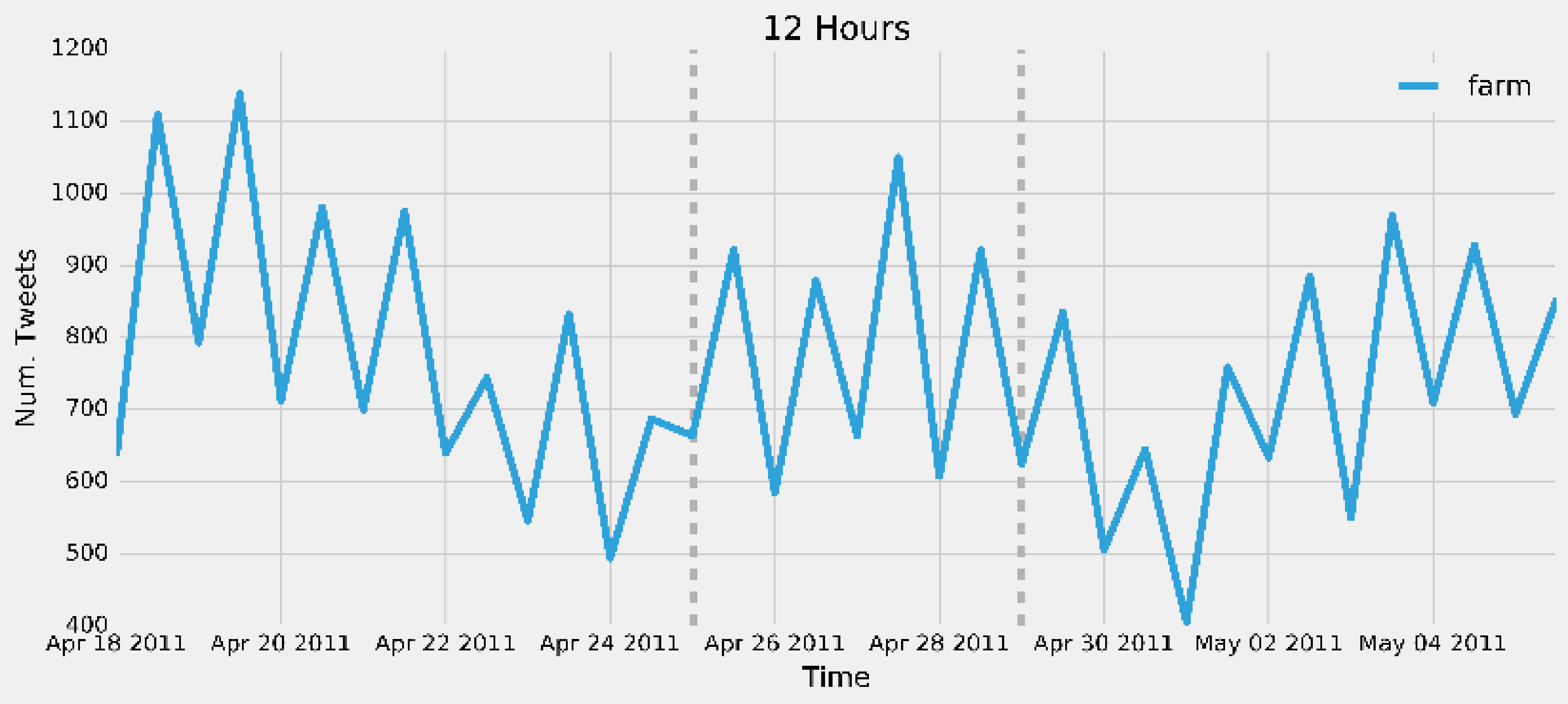

12 Hours
farm
1200
1100
1000
900
800
700
600
500
400
Num. Tweets
Apr 18 2011
Apr 20 2011
Apr 22 2011
Apr 24 2011
Apr 26 2011
Apr 28 2011
Apr 30 2011
May 02 2011
May 04 2011
Time

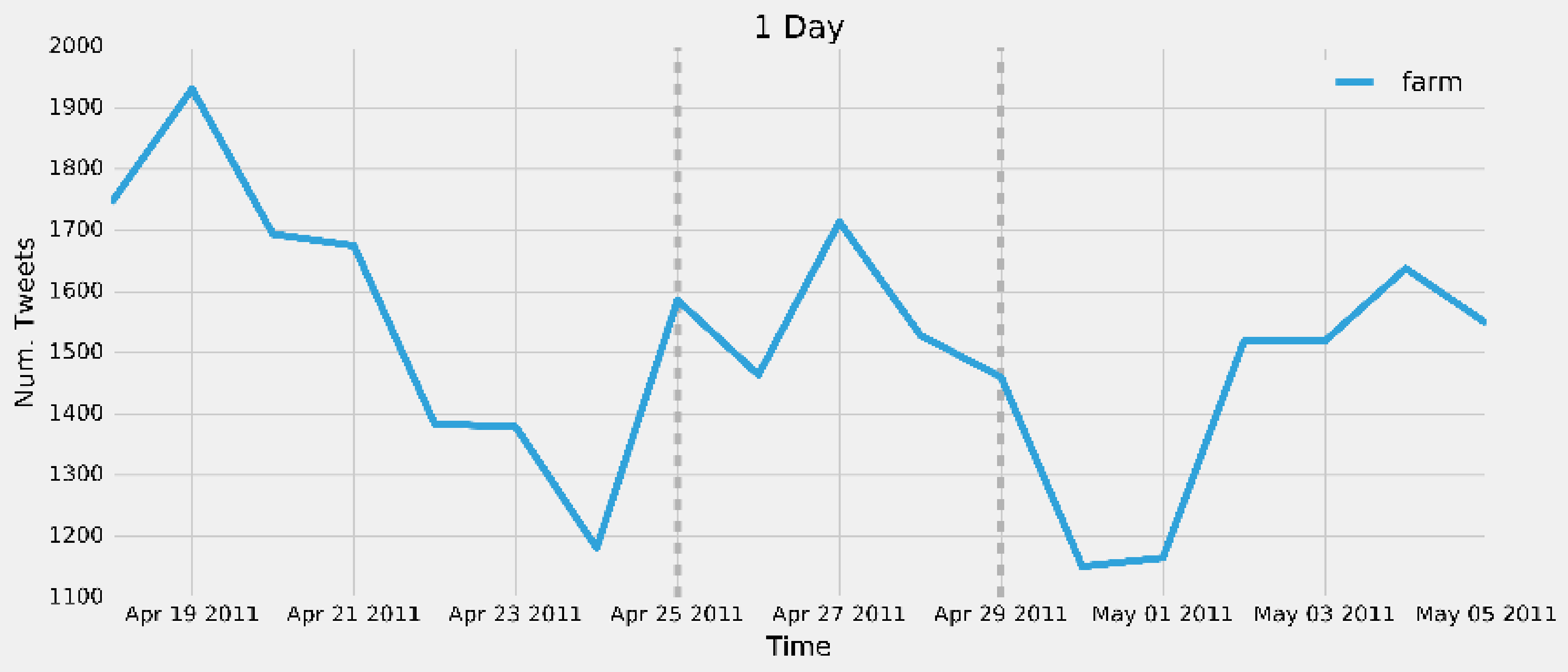
1 Day
farm
Num. Tweets
2000
1900
1800
1700
1600
1500
1400
1300
1200
1100
Apr 19 2011
Apr 21 2011
Apr 23 2011
Apr 25 2011
Apr 27 2011
Apr 29 2011
May 01 2011
May 03 2011
May 05 2011
Time

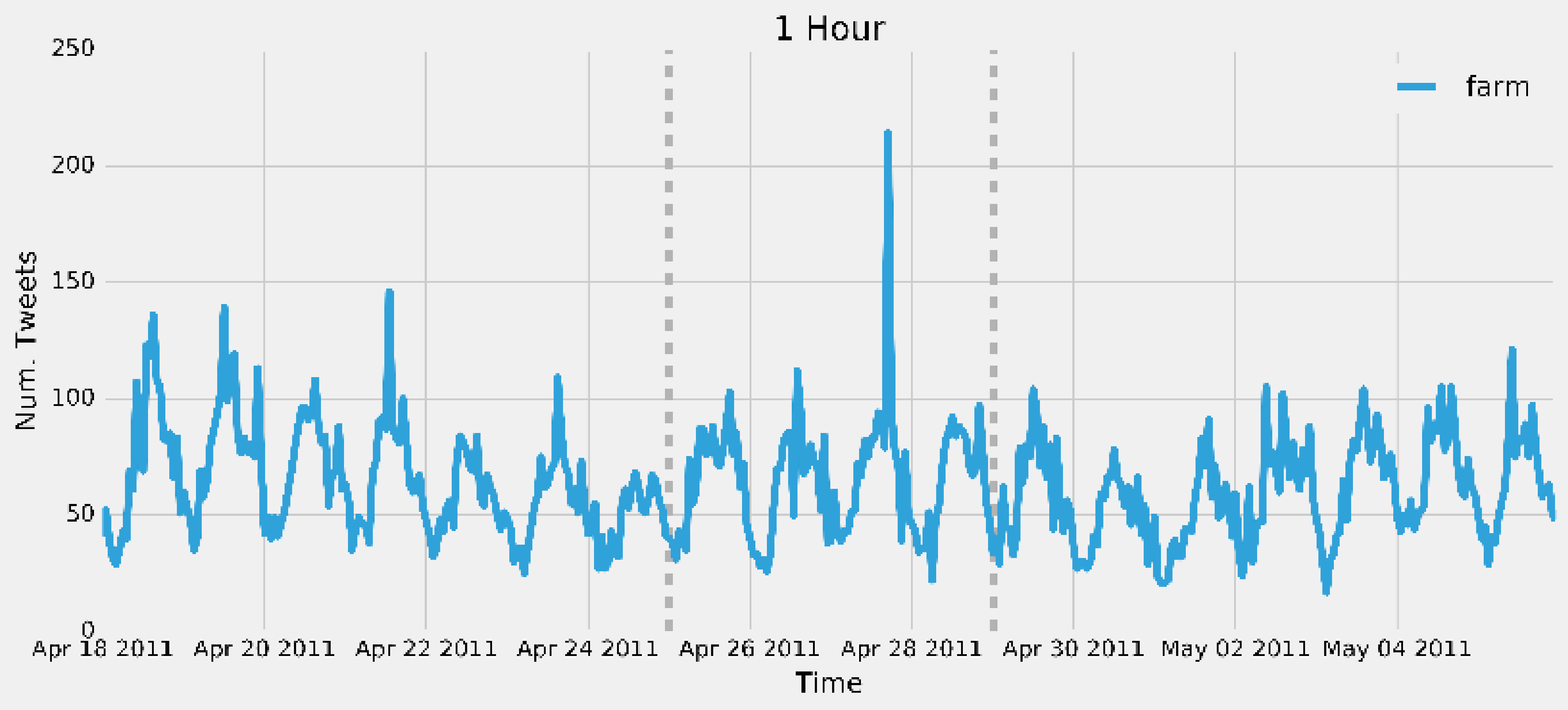

1 Hour
farm
Num. Tweets
Time
250
200
150
100
50
0
Apr 18 2011
Apr 20 2011
Apr 22 2011
Apr 24 2011
Apr 26 2011
Apr 28 2011
Apr 30 2011
May 02 2011
May 04 2011

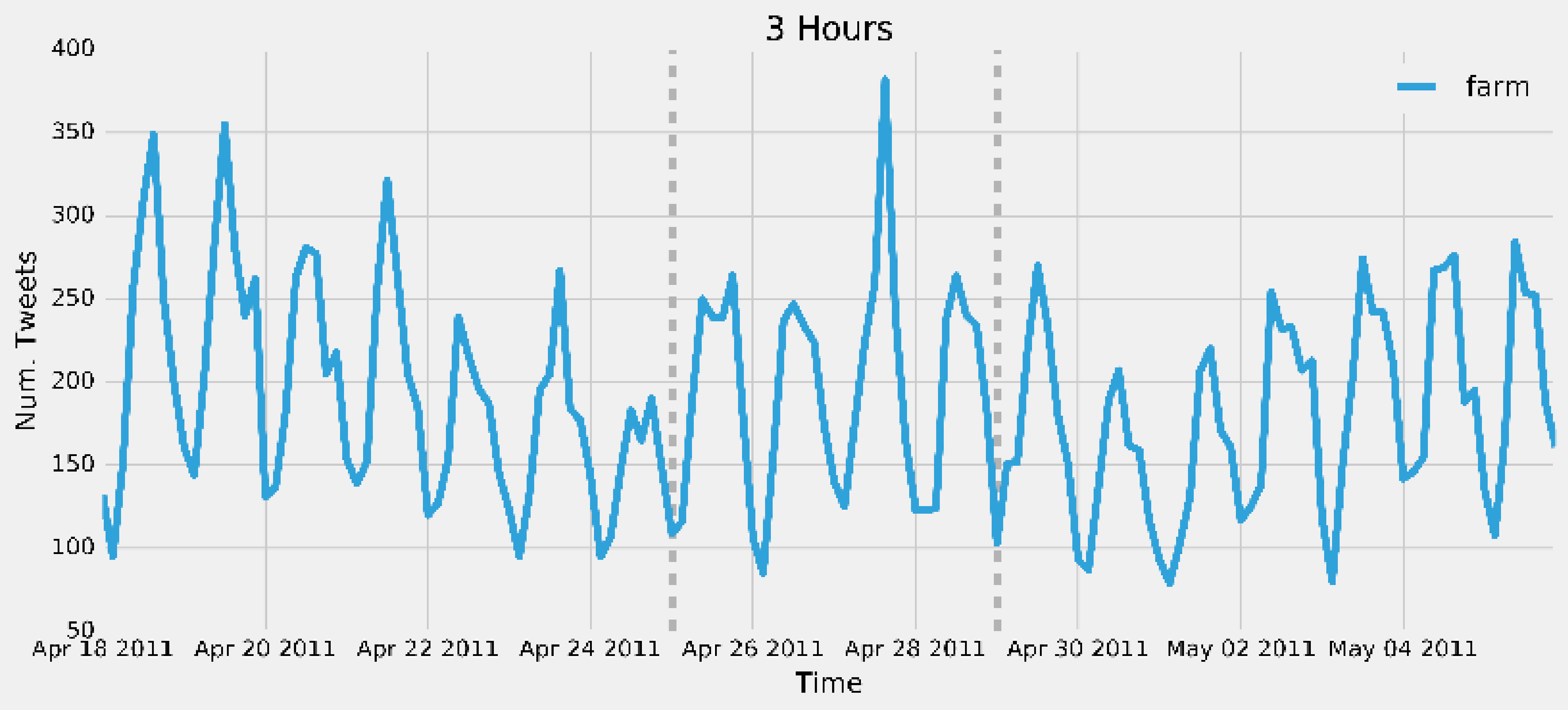

3 Hours
farm
Num. Tweets
400
350
300
250
200
150
100
50
Apr 18 2011
Apr 20 2011
Apr 22 2011
Apr 24 2011
Apr 26 2011
Apr 28 2011
Apr 30 2011
May 02 2011
May 04 2011
Time

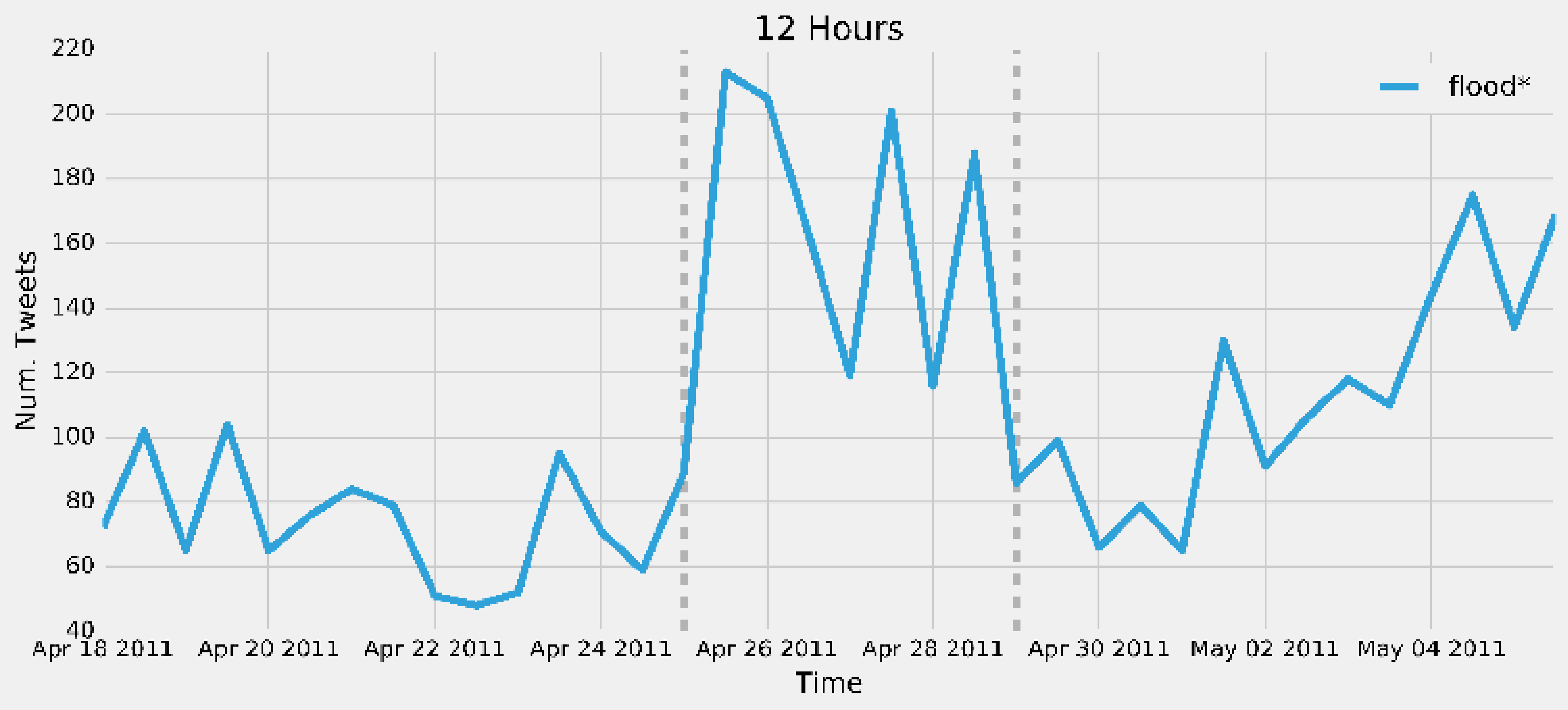

12 Hours
flood*
Num. Tweets
Time
220
200
180
160
140
120
100
80
60
40
Apr 18 2011
Apr 20 2011
Apr 22 2011
Apr 24 2011
Apr 26 2011
Apr 28 2011
Apr 30 2011
May 02 2011
May 04 2011

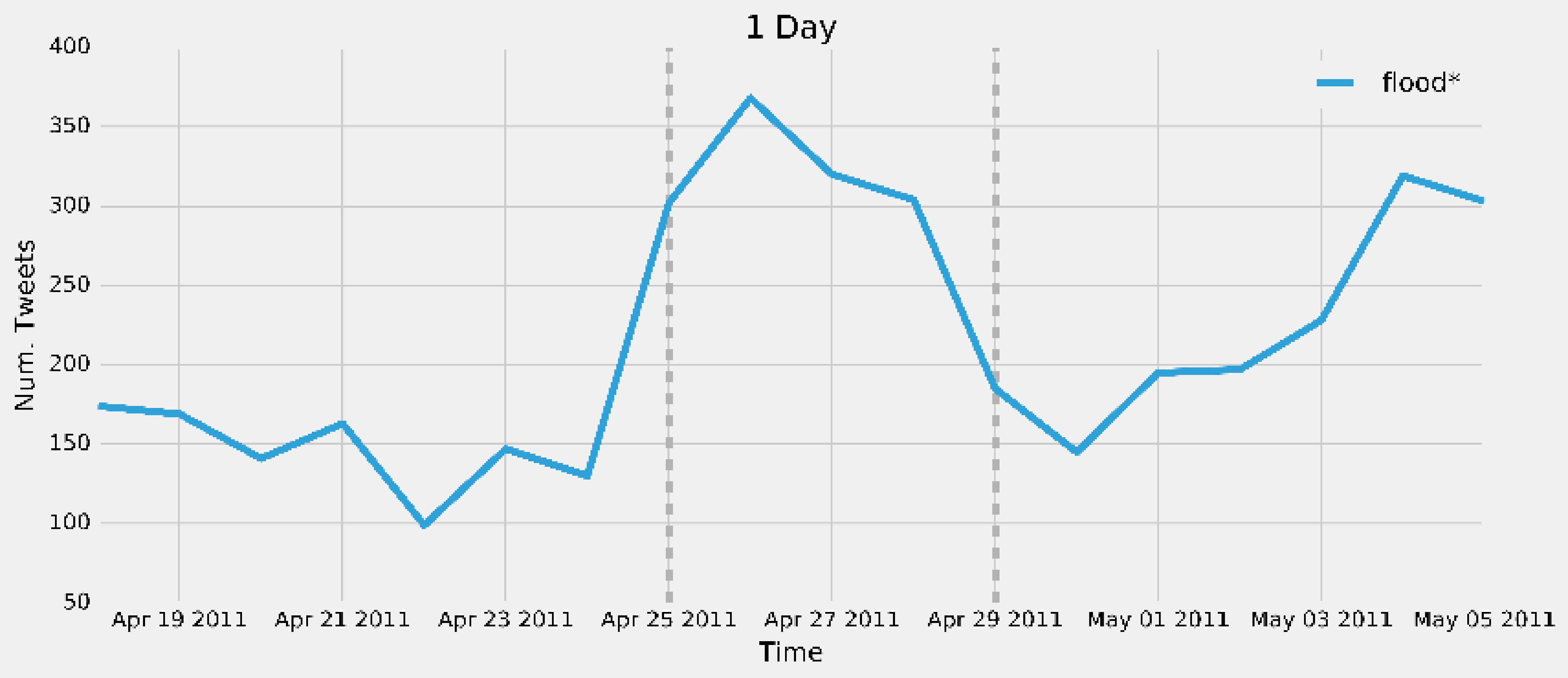
1 Day
flood*
Num. Tweets
Time
400
350
300
250
200
150
100
50
Apr 19 2011
Apr 21 2011
Apr 23 2011
Apr 25 2011
Apr 27 2011
Apr 29 2011
May 01 2011
May 03 2011
May 05 2011

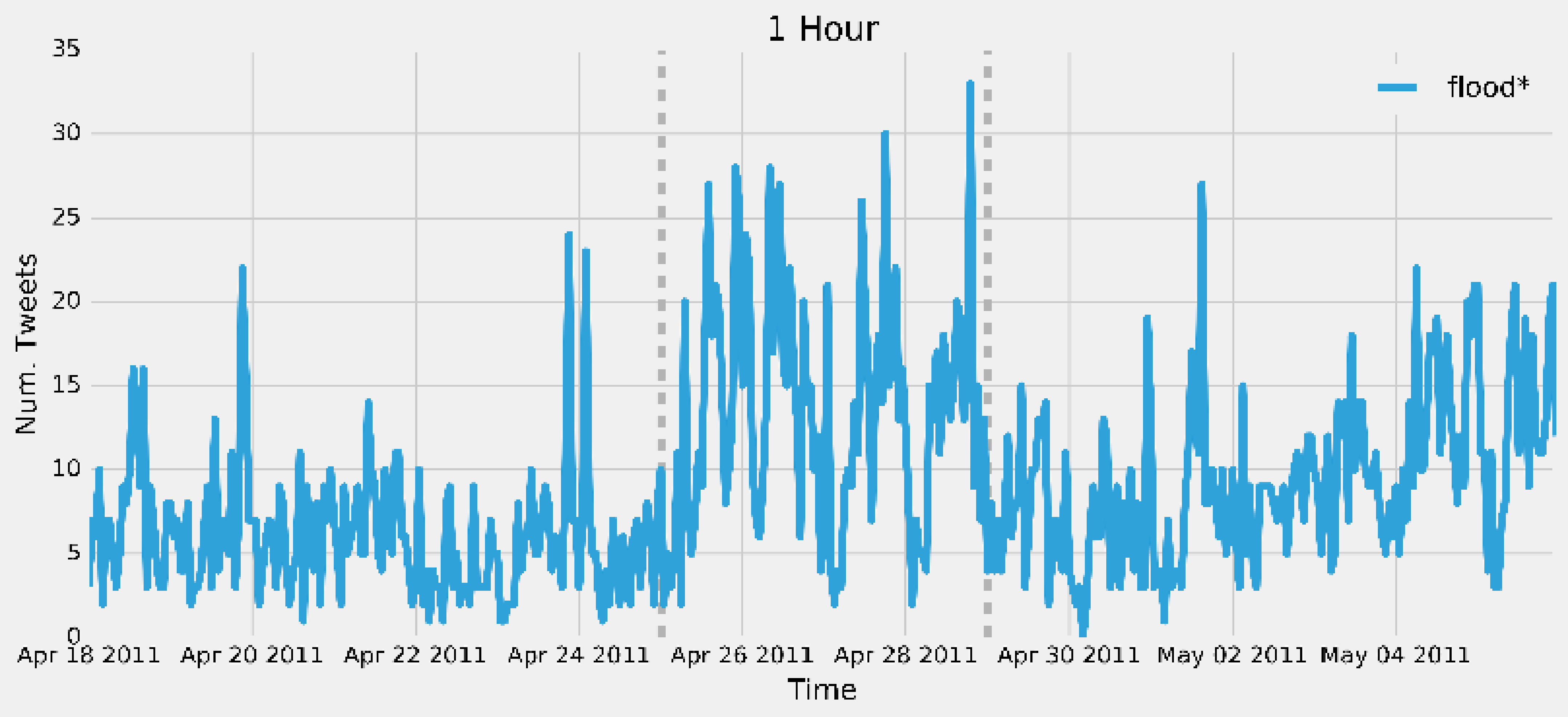
1 Hour
flood*
Num. Tweets
35
30
25
20
15
10
5
0
Apr 18 2011
Apr 20 2011
Apr 22 2011
Apr 24 2011
Apr 26 2011
Apr 28 2011
Apr 30 2011
May 02 2011
May 04 2011
Time

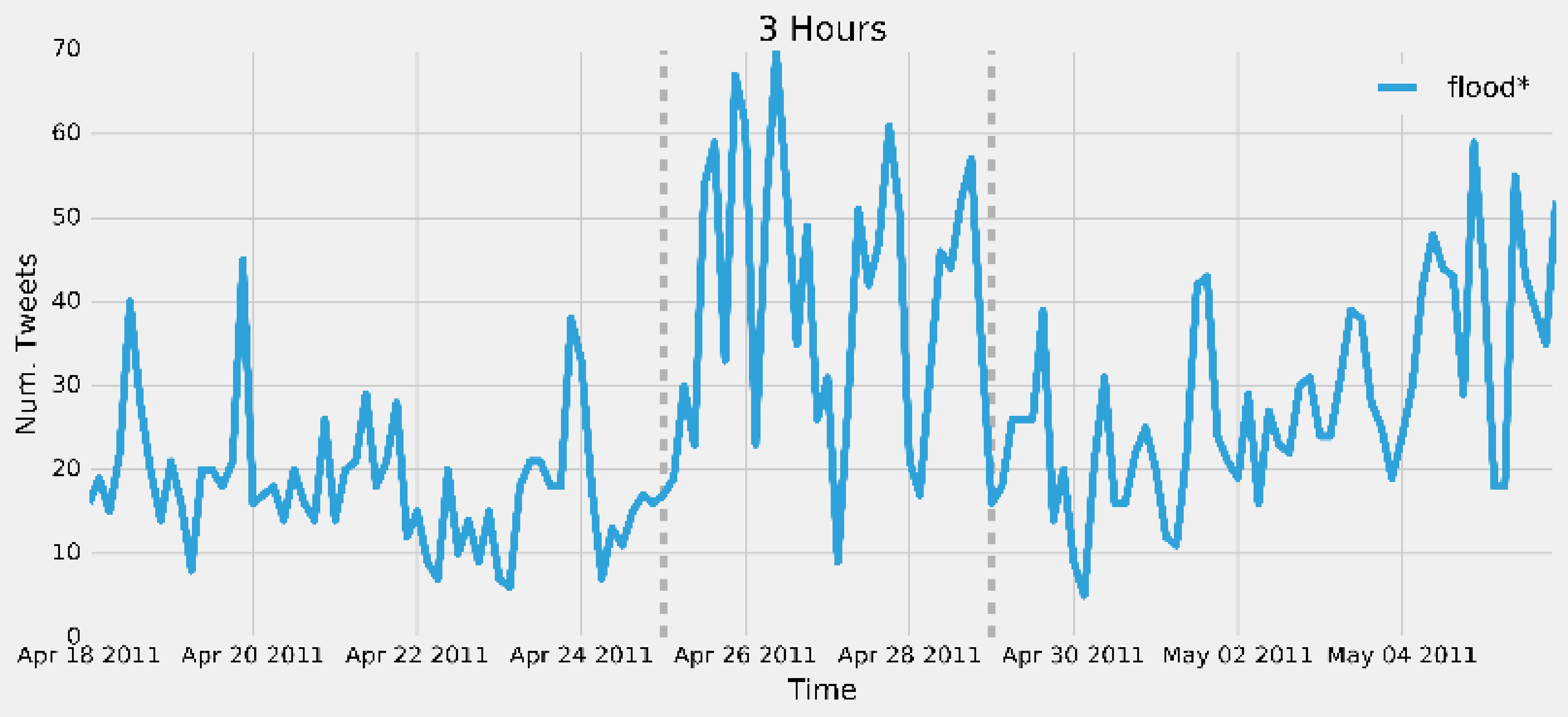
3 Hours
flood*
Num. Tweets
Time
0
10
20
30
40
50
60
70
Apr 18 2011
Apr 20 2011
Apr 22 2011
Apr 24 2011
Apr 26 2011
Apr 28 2011
Apr 30 2011
May 02 2011
May 04 2011

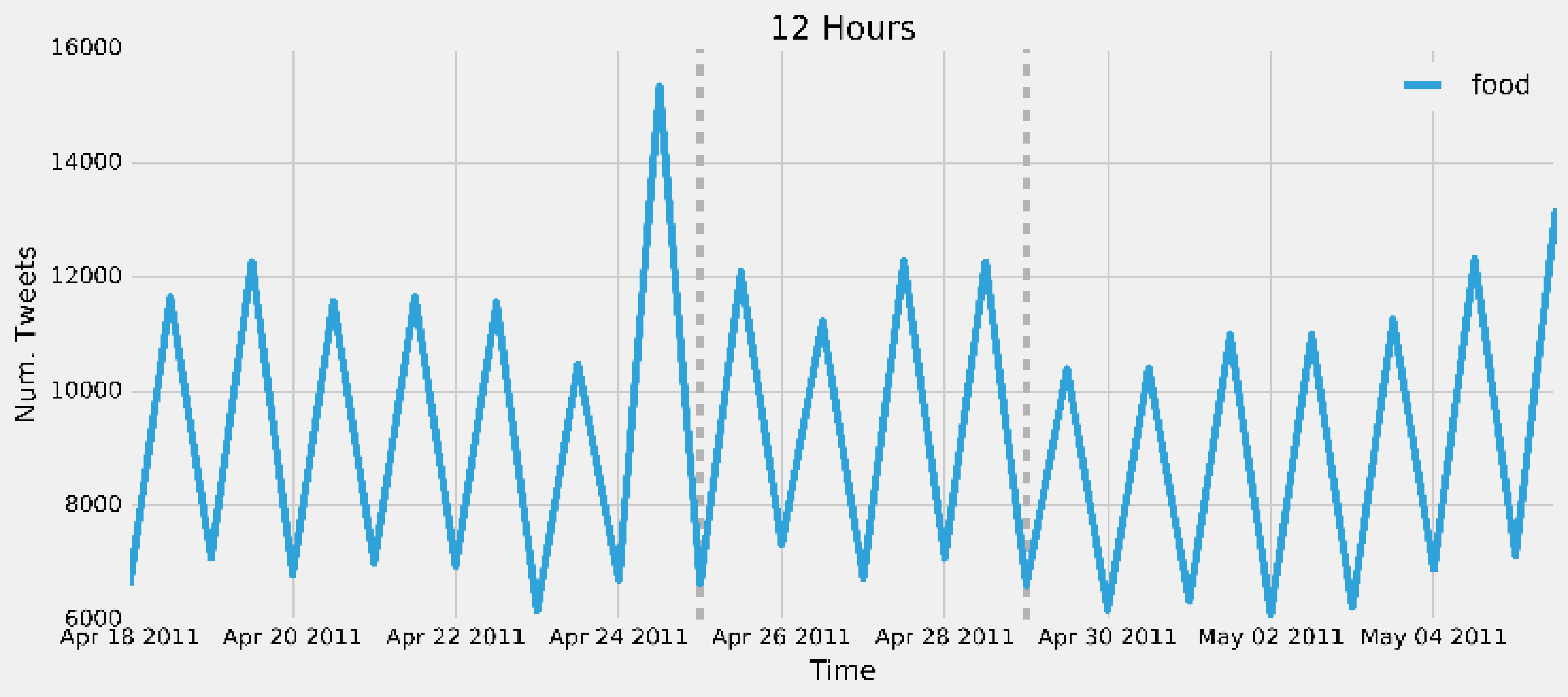
12 Hours
food
Num. Tweets
16000
14000
12000
10000
8000
6000
Apr 18 2011
Apr 20 2011
Apr 22 2011
Apr 24 2011
Apr 26 2011
Apr 28 2011
Apr 30 2011
May 02 2011
May 04 2011
Time

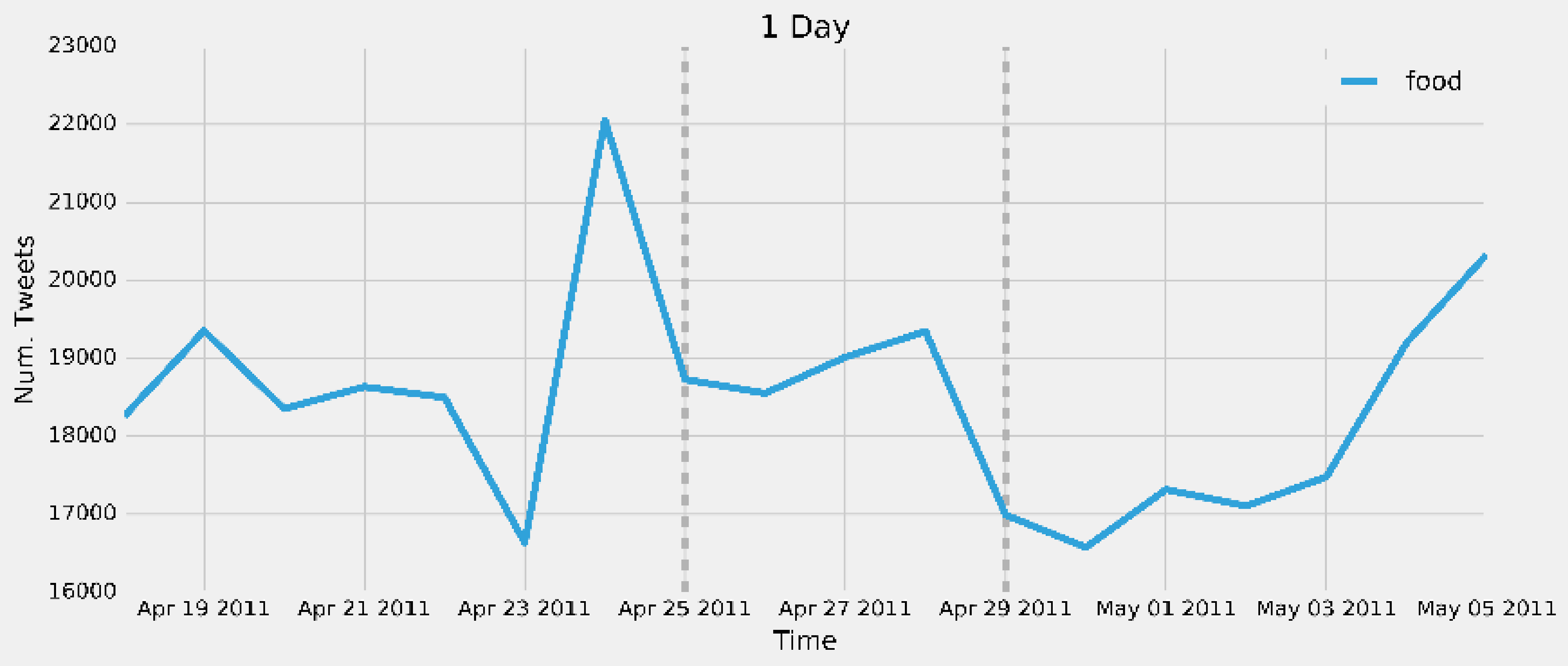
1 Day
food
Num. Tweets
23000
22000
21000
20000
19000
18000
17000
16000
Apr 19 2011
Apr 21 2011
Apr 23 2011
Apr 25 2011
Apr 27 2011
Apr 29 2011
May 01 2011
May 03 2011
May 05 2011
Time

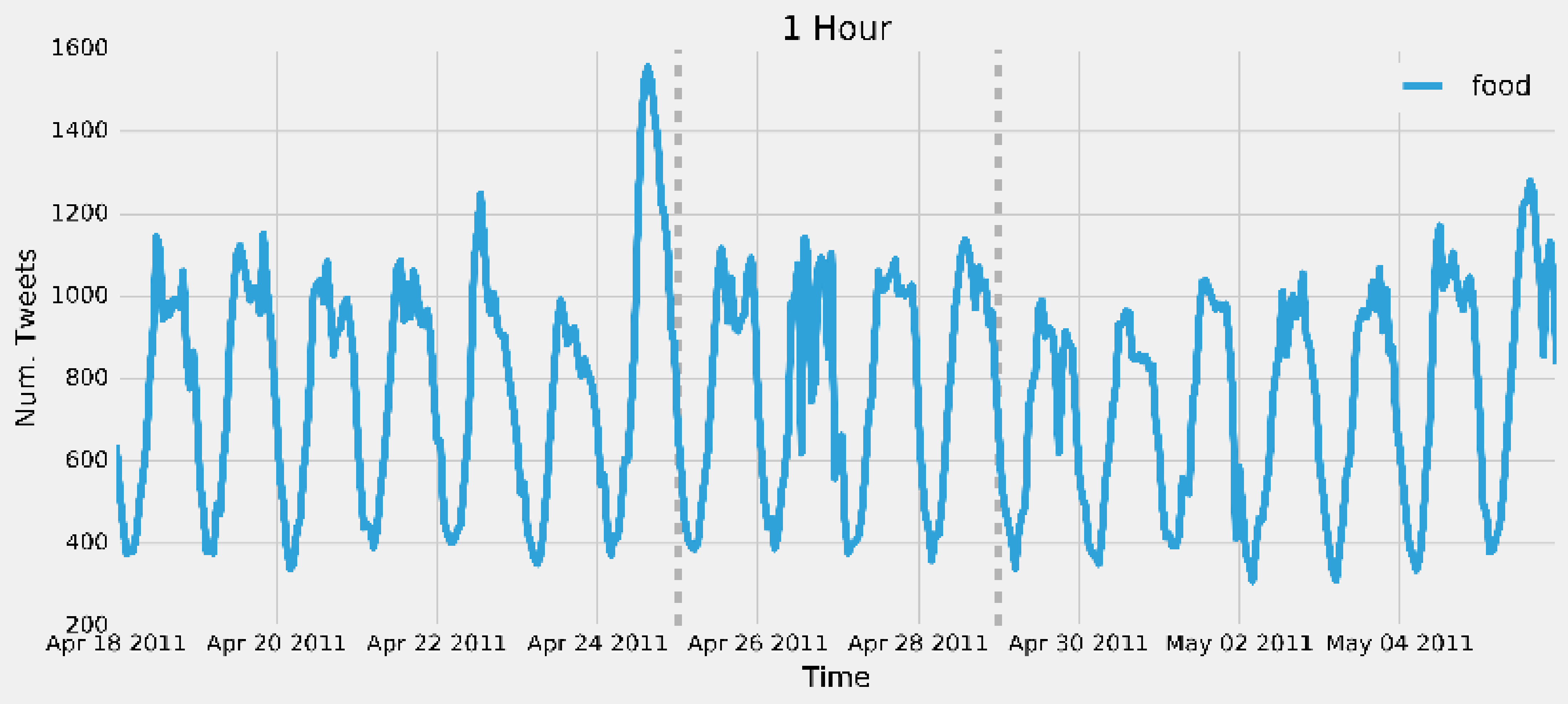
1 Hour
food
Num. Tweets
1600
1400
1200
1000
800
600
400
200
Apr 18 2011
Apr 20 2011
Apr 22 2011
Apr 24 2011
Apr 26 2011
Apr 28 2011
Apr 30 2011
May 02 2011
May 04 2011
Time

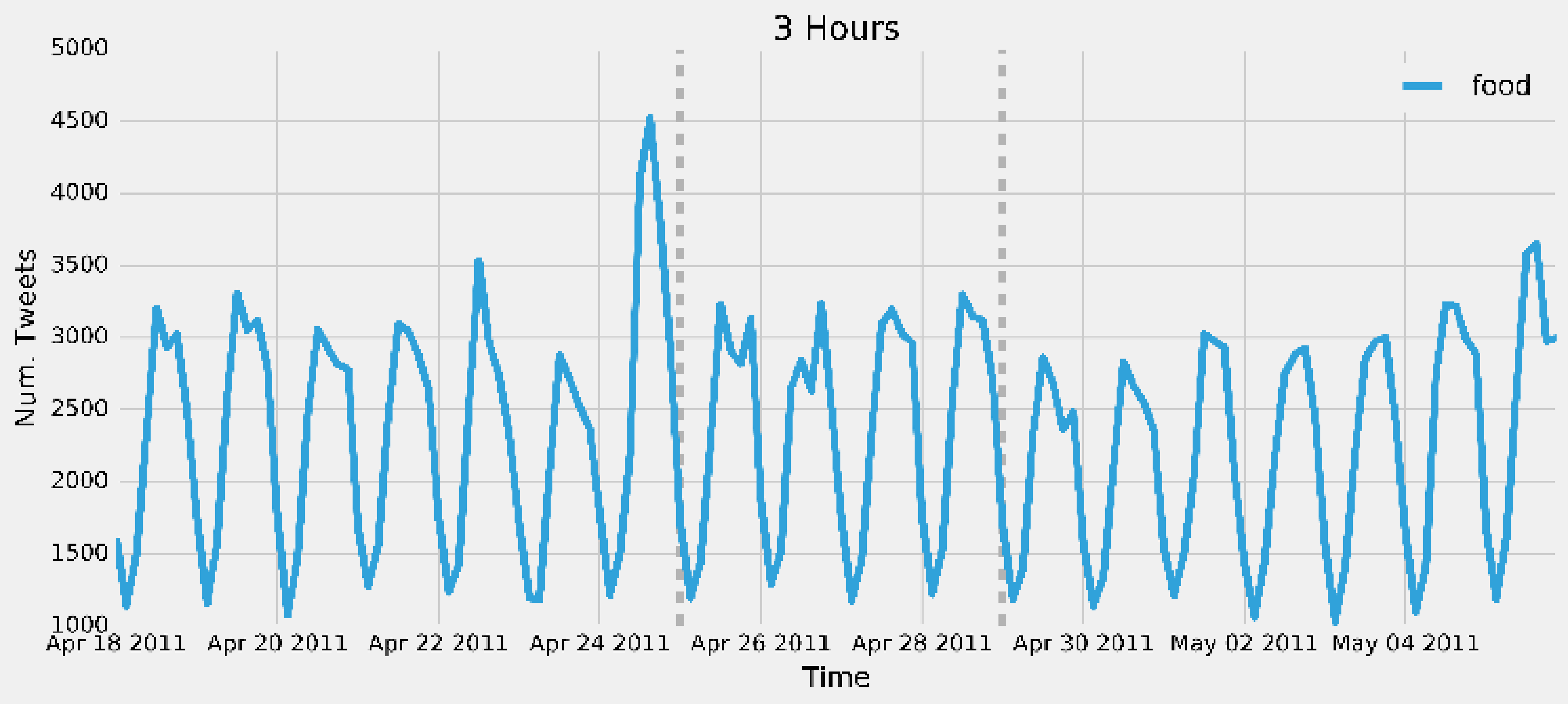
3 Hours
food
Num. Tweets
1000
1500
2000
2500
3000
3500
4000
4500
5000
Apr 18 2011
Apr 20 2011
Apr 22 2011
Apr 24 2011
Apr 26 2011
Apr 28 2011
Apr 30 2011
May 02 2011
May 04 2011
Time

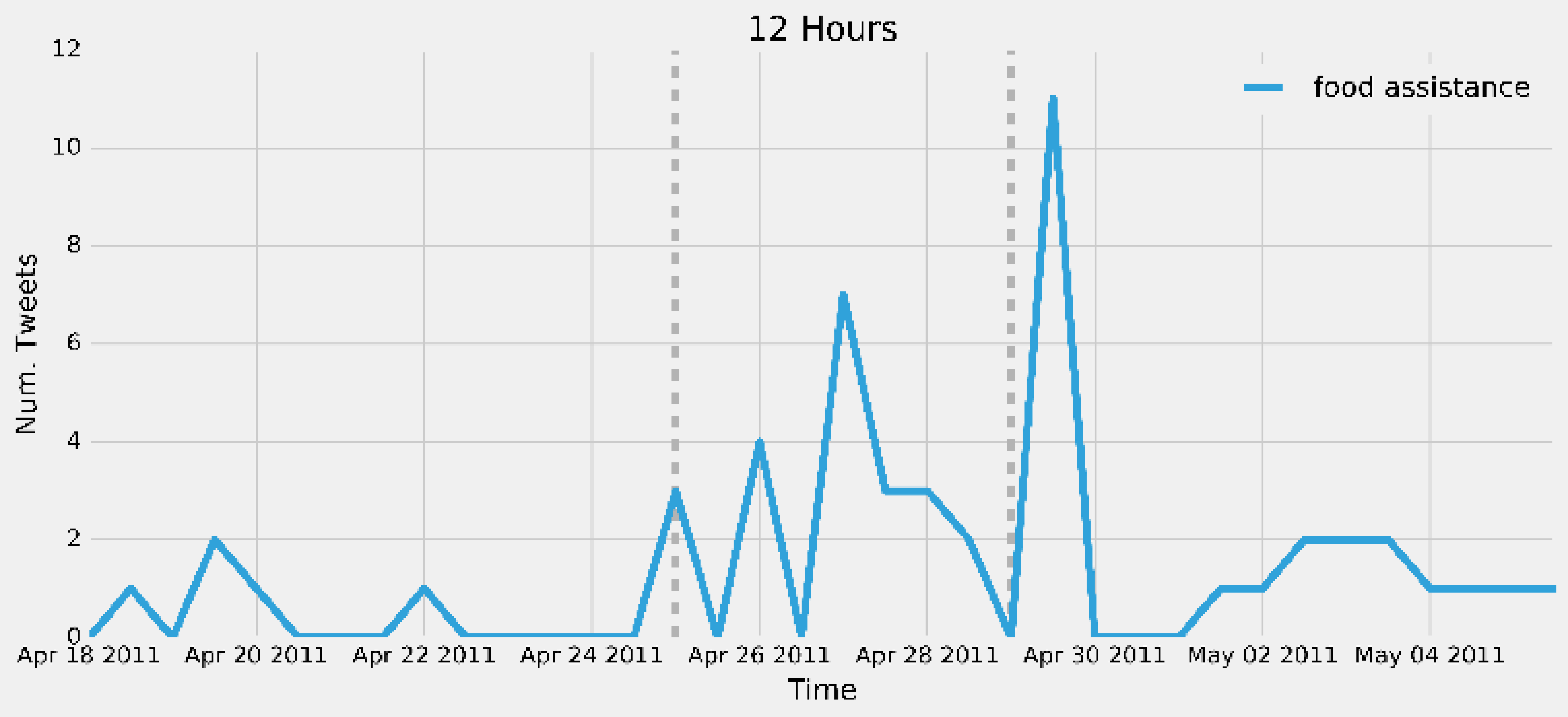

12 Hours
food assistance
Num. Tweets
0
2
4
6
8
10
12
Apr 18 2011
Apr 20 2011
Apr 22 2011
Apr 24 2011
Apr 26 2011
Apr 28 2011
Apr 30 2011
May 02 2011
May 04 2011
Time

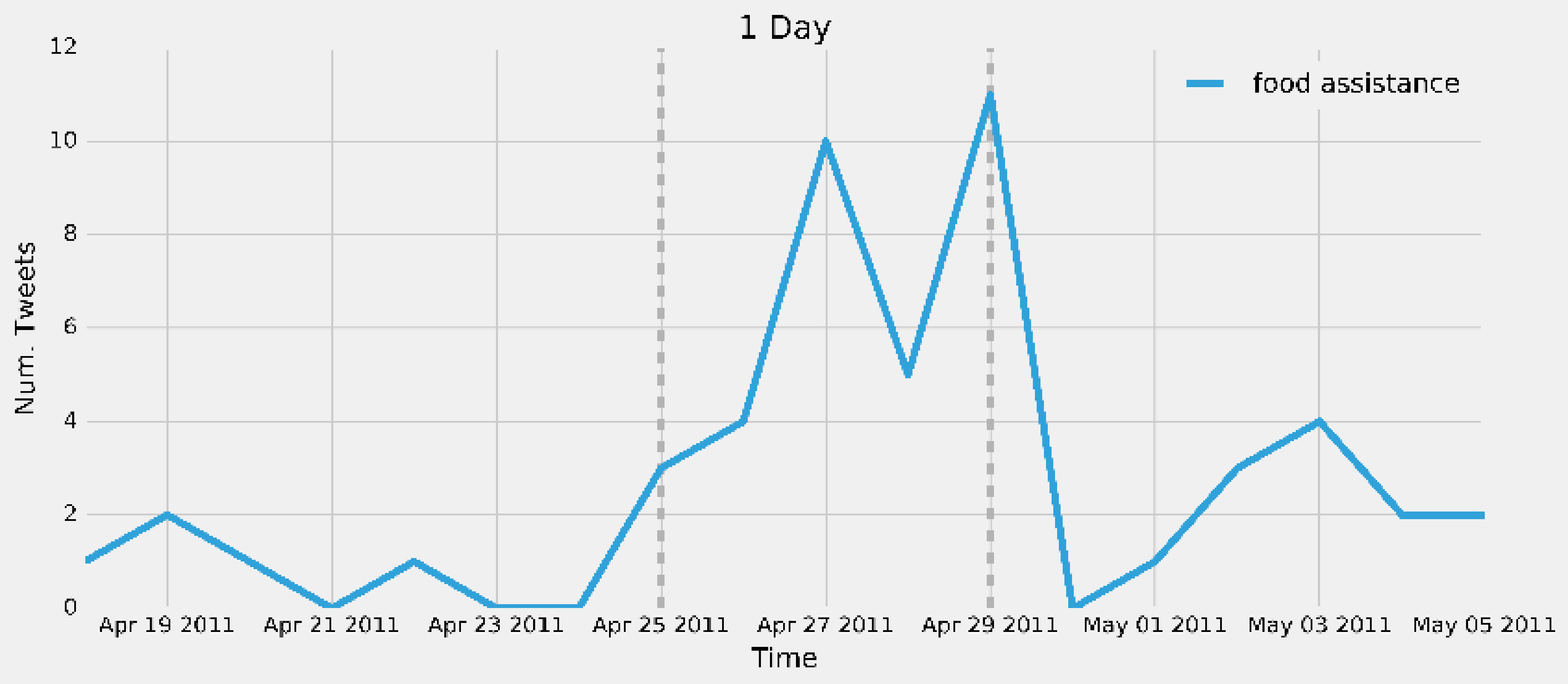
1 Day
food assistance
Num. Tweets
0
2
4
6
8
10
12
Apr 19 2011
Apr 21 2011
Apr 23 2011
Apr 25 2011
Apr 27 2011
Apr 29 2011
May 01 2011
May 03 2011
May 05 2011
Time

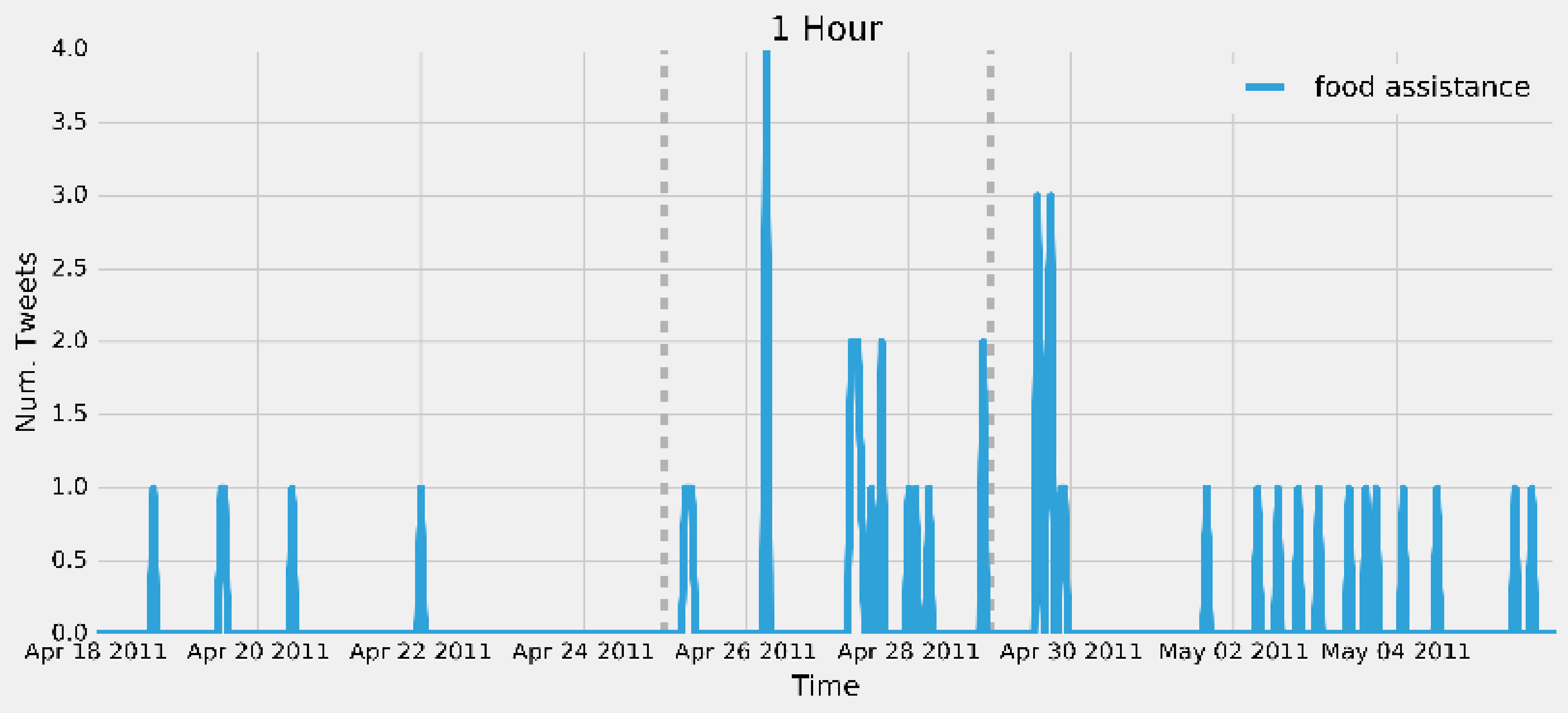
1 Hour
food assistance
Num. Tweets
0.0
0.5
1.0
1.5
2.0
2.5
3.0
3.5
4.0
Apr 18 2011
Apr 20 2011
Apr 22 2011
Apr 24 2011
Apr 26 2011
Apr 28 2011
Apr 30 2011
May 02 2011
May 04 2011
Time

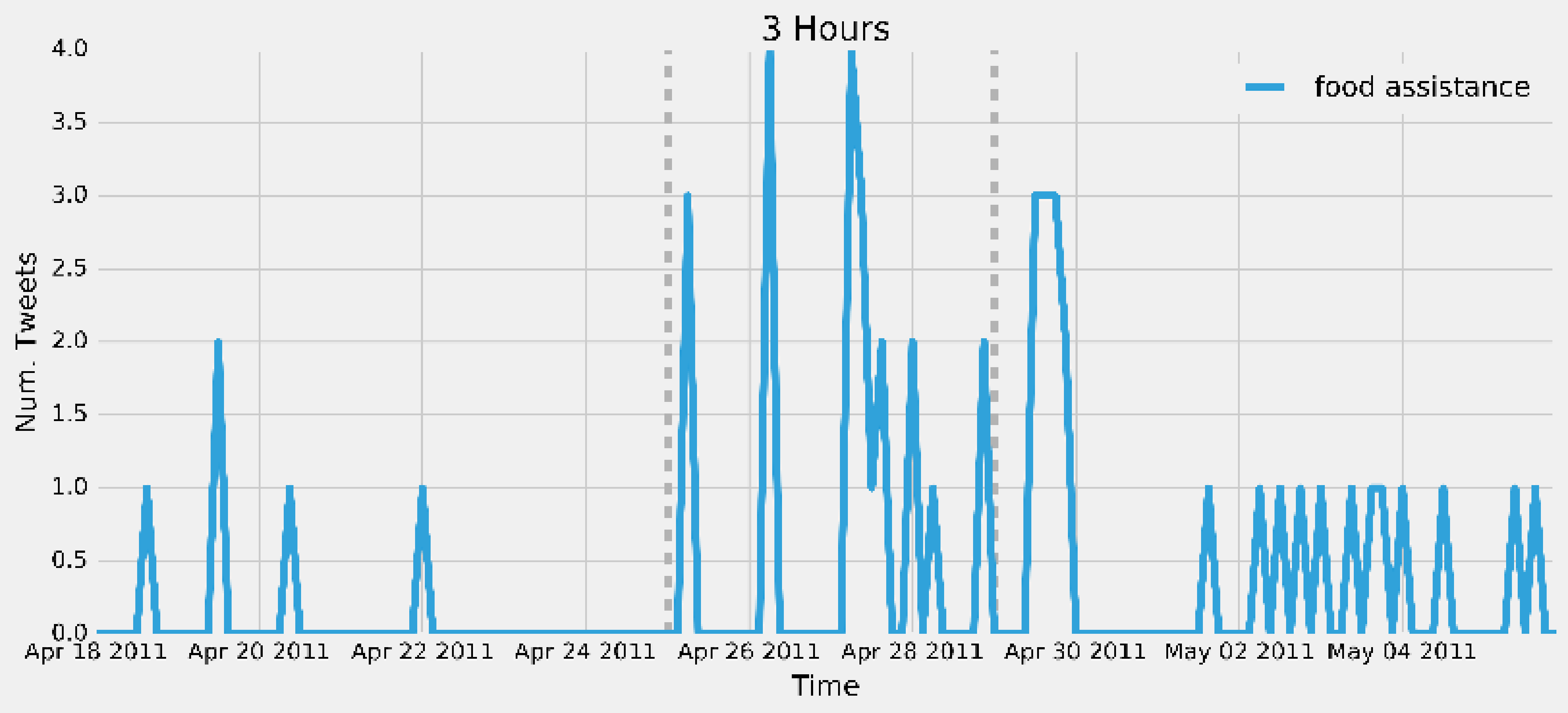

3 Hours
food assistance
Num. Tweets
4.0
3.5
3.0
2.5
2.0
1.5
1.0
0.5
0.0
Apr 18 2011
Apr 20 2011
Apr 22 2011
Apr 24 2011
Apr 26 2011
Apr 28 2011
Apr 30 2011
May 02 2011
May 04 2011
Time

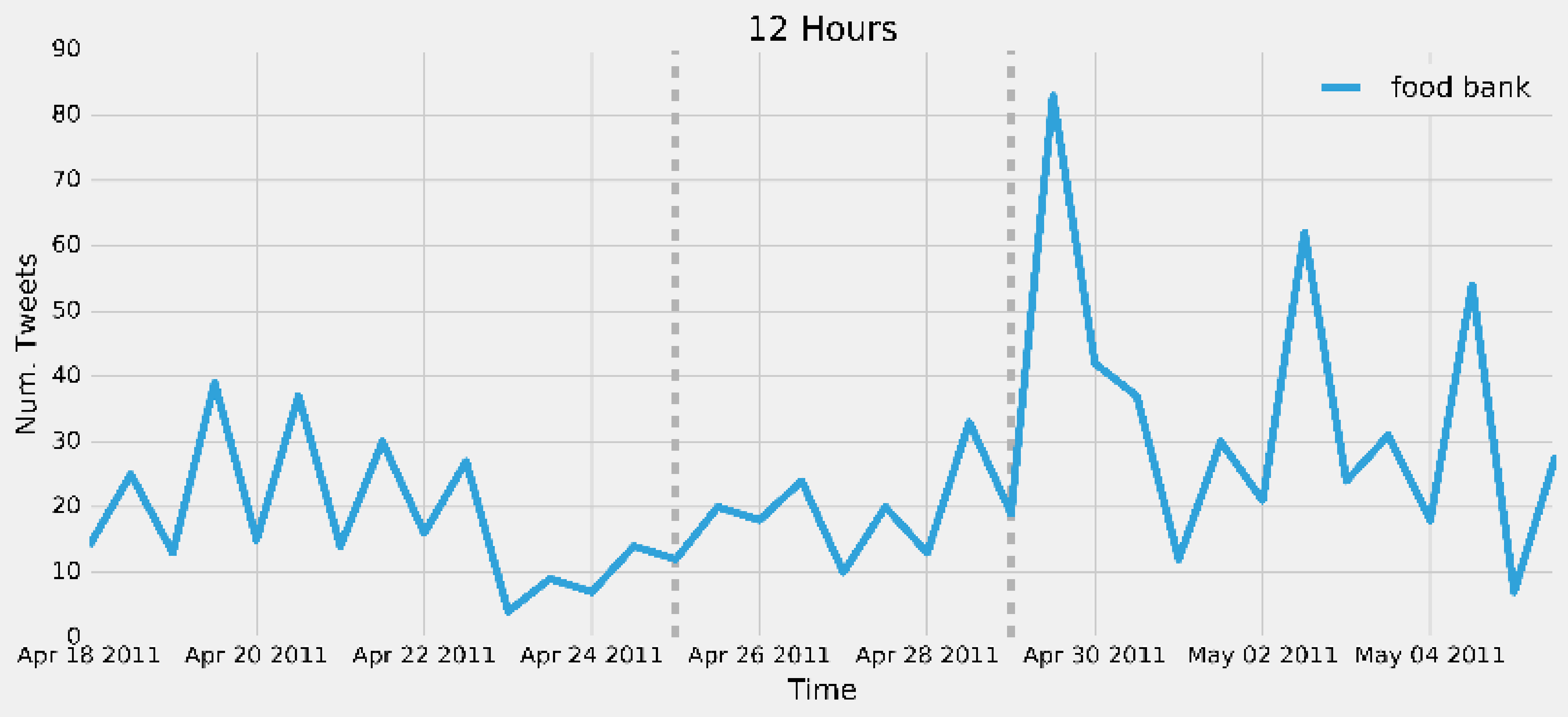
12 Hours
food bank
Num. Tweets
Time
0
10
20
30
40
50
60
70
80
90
Apr 18 2011
Apr 20 2011
Apr 22 2011
Apr 24 2011
Apr 26 2011
Apr 28 2011
Apr 30 2011
May 02 2011
May 04 2011

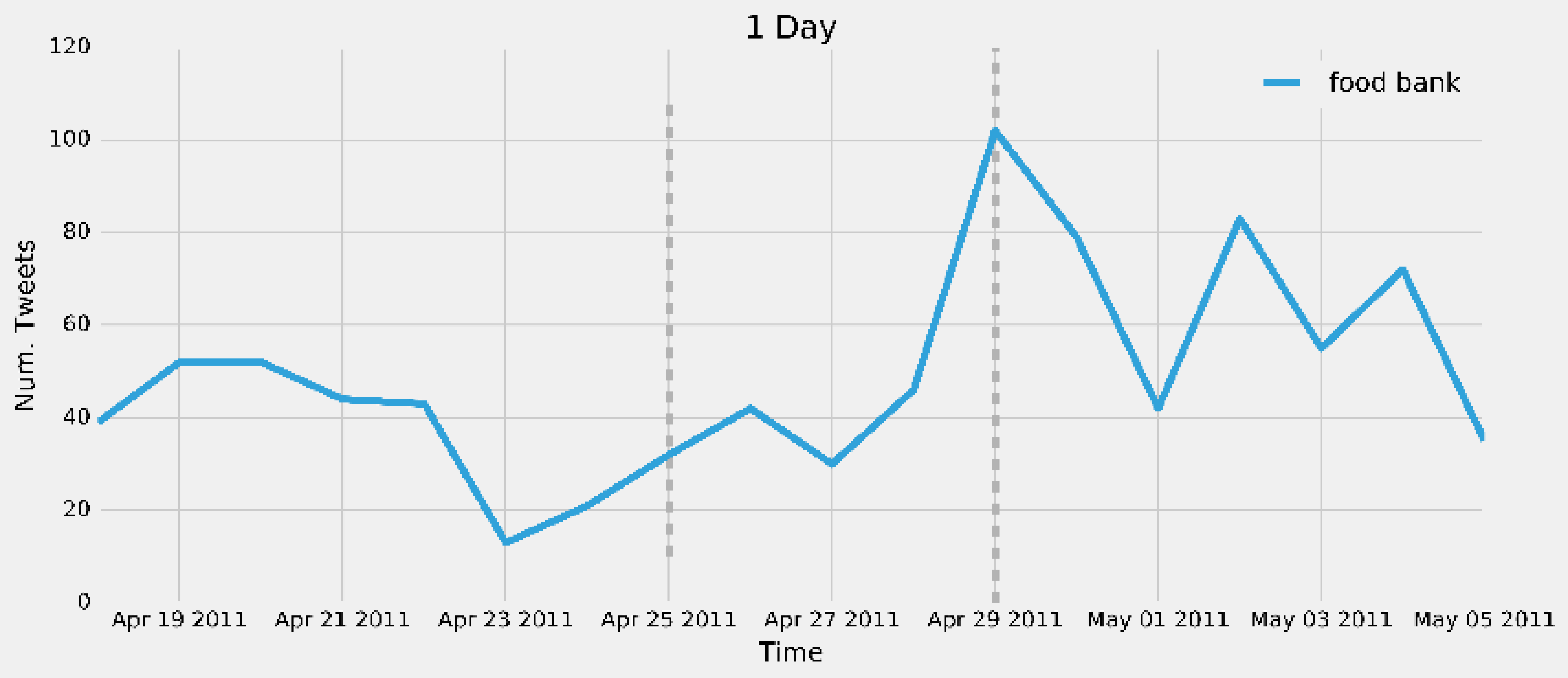

1 Day
food bank
Num. Tweets
Time
0
20
40
60
80
100
120
Apr 19 2011
Apr 21 2011
Apr 23 2011
Apr 25 2011
Apr 27 2011
Apr 29 2011
May 01 2011
May 03 2011
May 05 2011

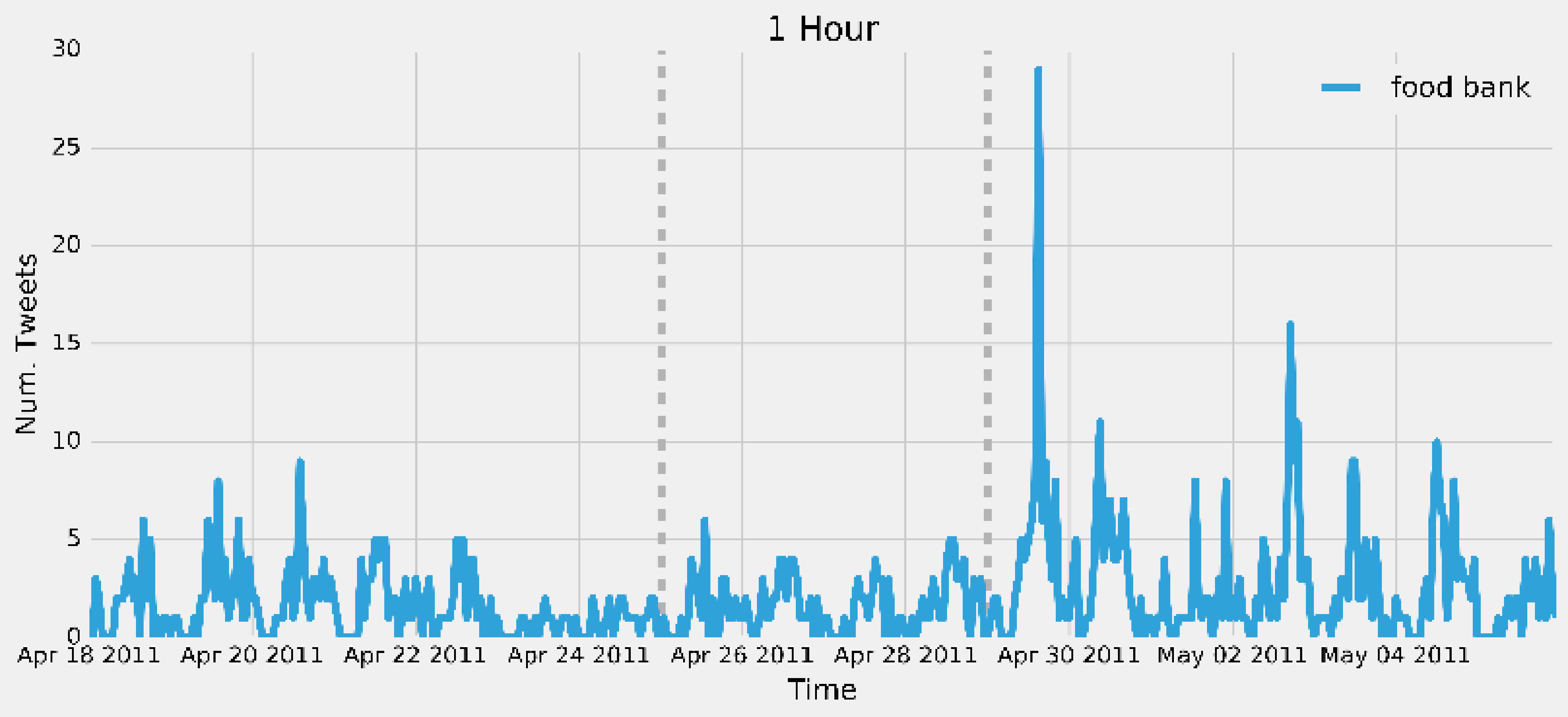
1 Hour
food bank
Num. Tweets
Time
30
25
20
15
10
5
0
Apr 18 2011
Apr 20 2011
Apr 22 2011
Apr 24 2011
Apr 26 2011
Apr 28 2011
Apr 30 2011
May 02 2011
May 04 2011

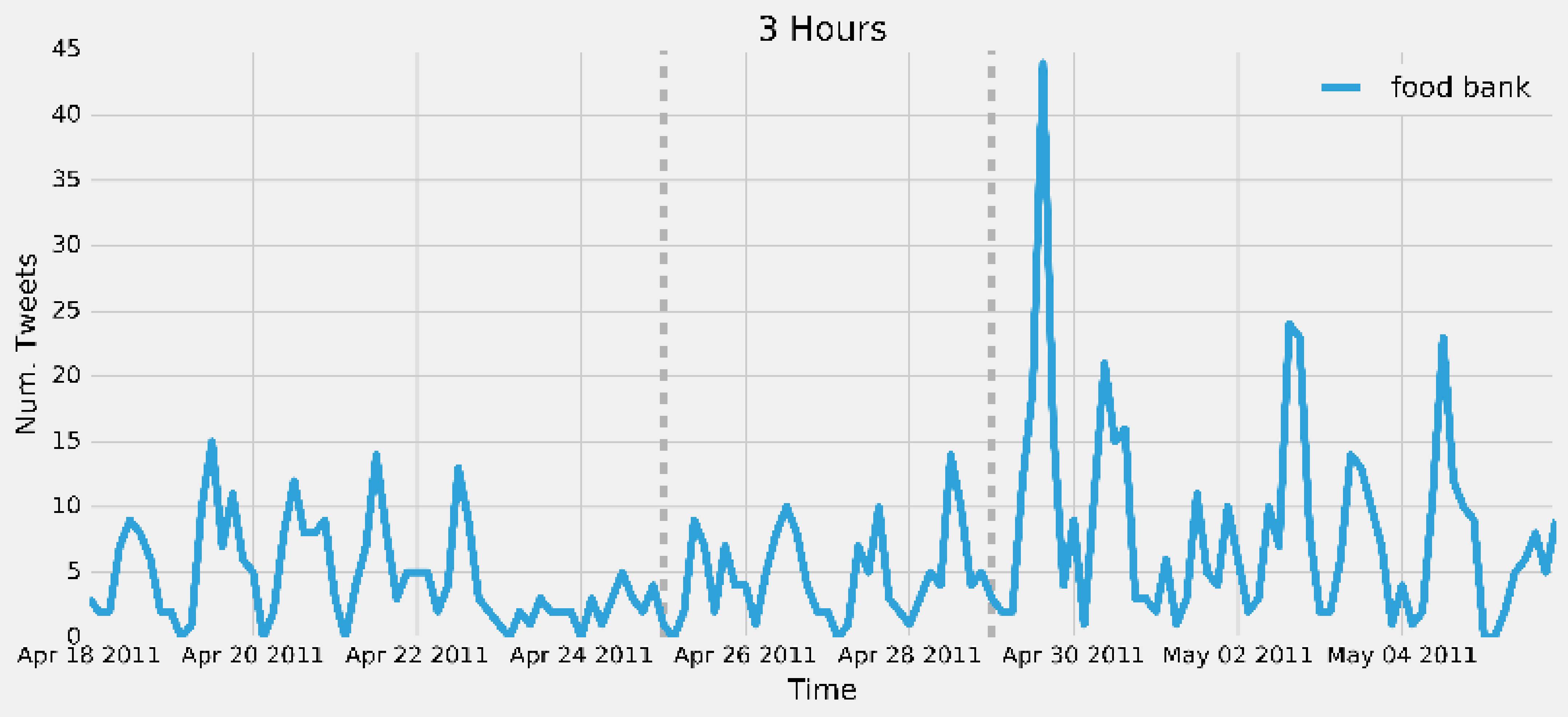
3 Hours
food bank
Num. Tweets
Time
0
5
10
15
20
25
30
35
40
45
Apr 18 2011
Apr 20 2011
Apr 22 2011
Apr 24 2011
Apr 26 2011
Apr 28 2011
Apr 30 2011
May 02 2011
May 04 2011

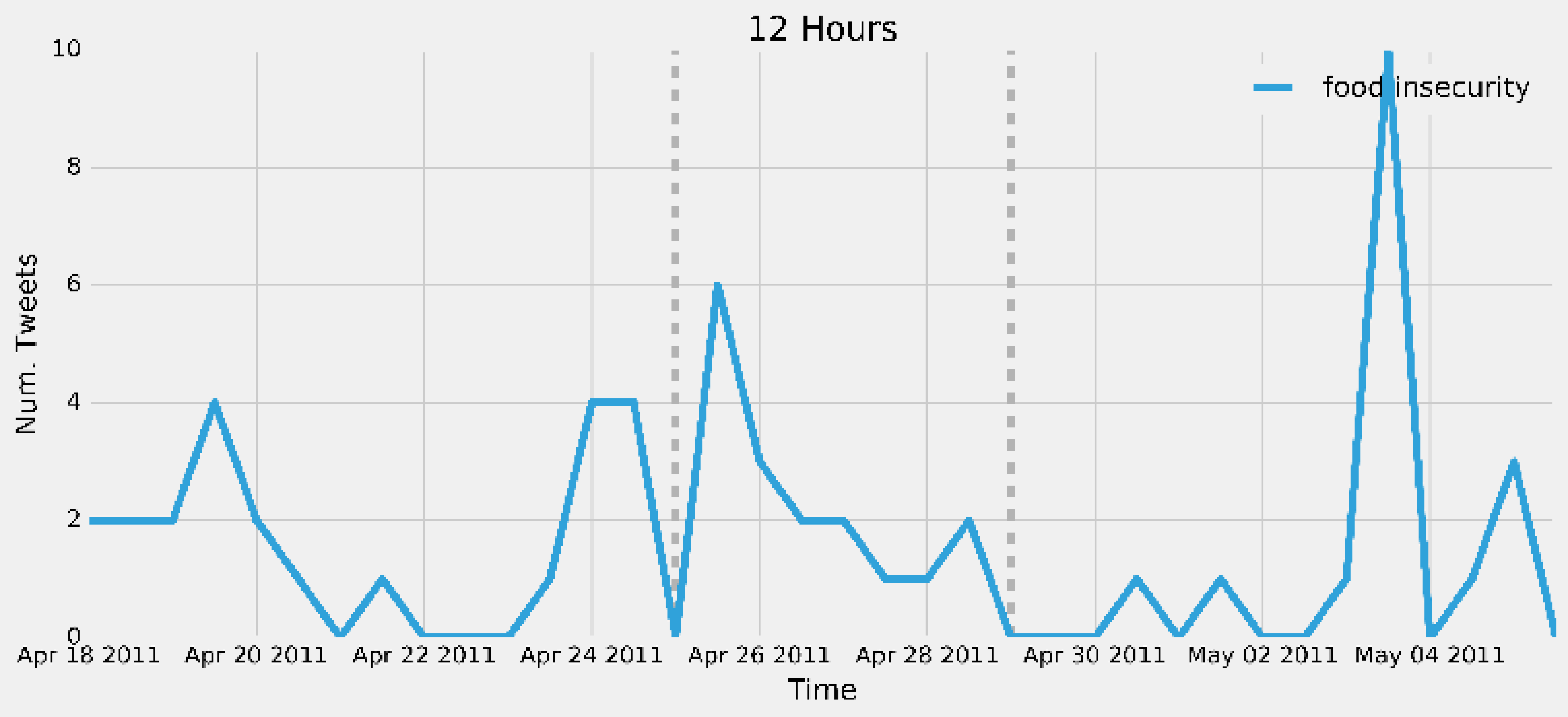

12 Hours
food insecurity
Num. Tweets
Time
0
2
4
6
8
10
Apr 18 2011
Apr 20 2011
Apr 22 2011
Apr 24 2011
Apr 26 2011
Apr 28 2011
Apr 30 2011
May 02 2011
May 04 2011

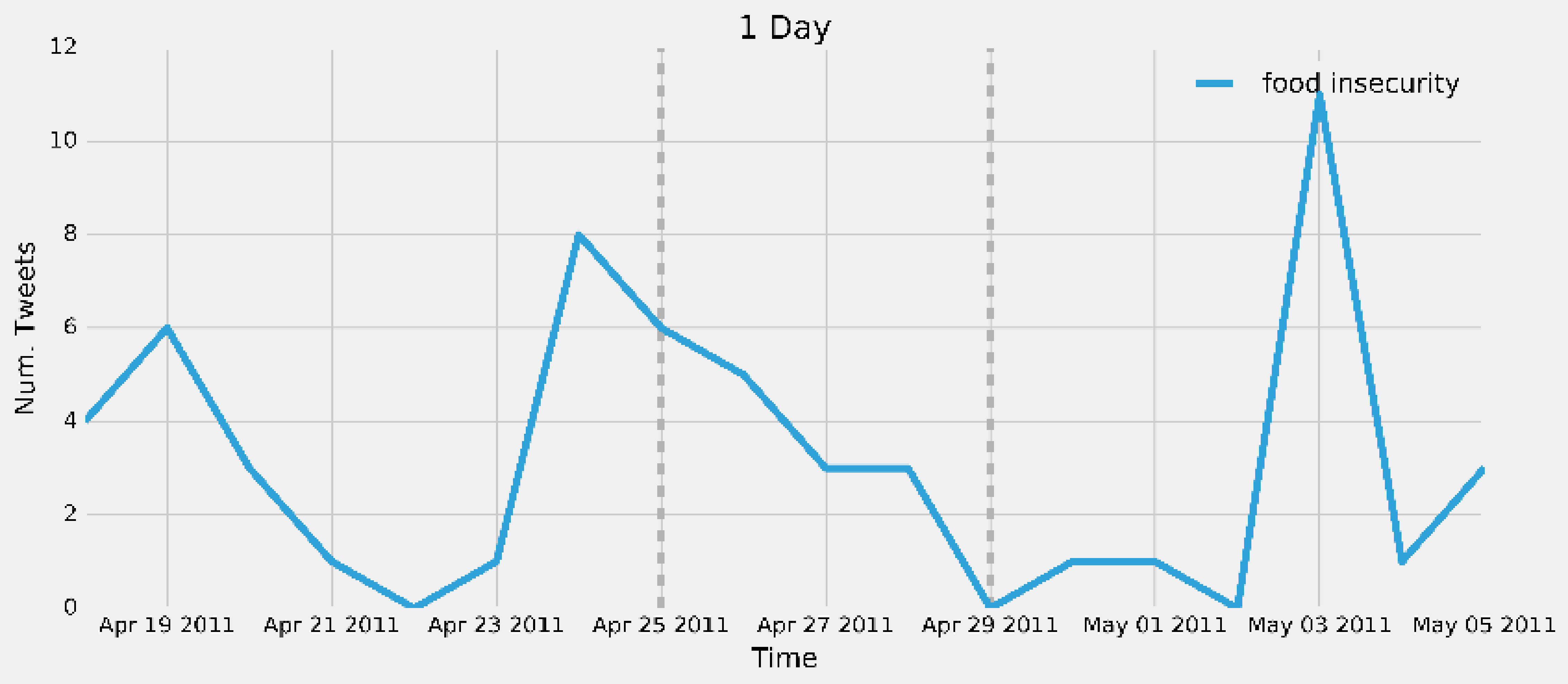
1 Day
food insecurity
Num. Tweets
0
2
4
6
8
10
12
Apr 19 2011
Apr 21 2011
Apr 23 2011
Apr 25 2011
Apr 27 2011
Apr 29 2011
May 01 2011
May 03 2011
May 05 2011
Time

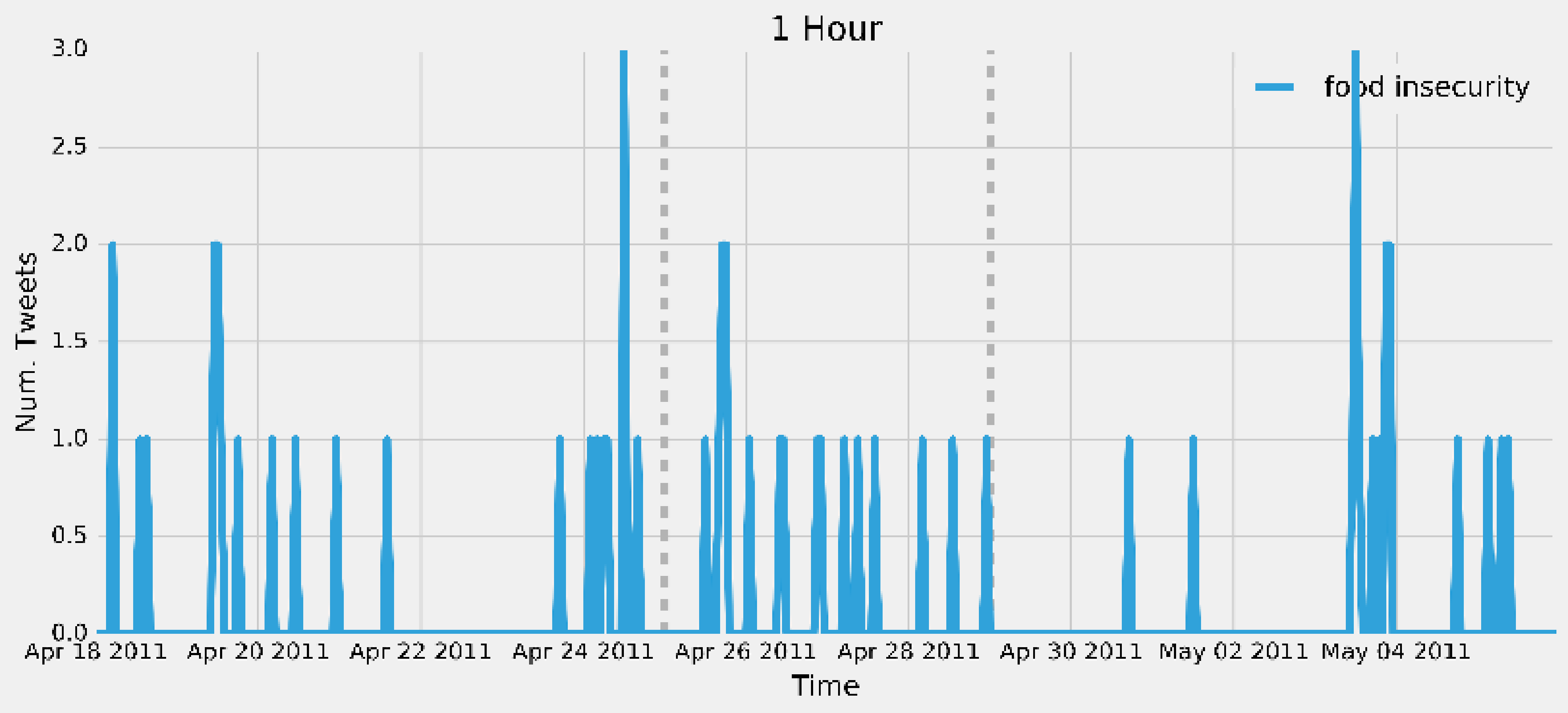
1 Hour
food insecurity
Num. Tweets
0.0
0.5
1.0
1.5
2.0
2.5
3.0
Apr 18 2011
Apr 20 2011
Apr 22 2011
Apr 24 2011
Apr 26 2011
Apr 28 2011
Apr 30 2011
May 02 2011
May 04 2011
Time

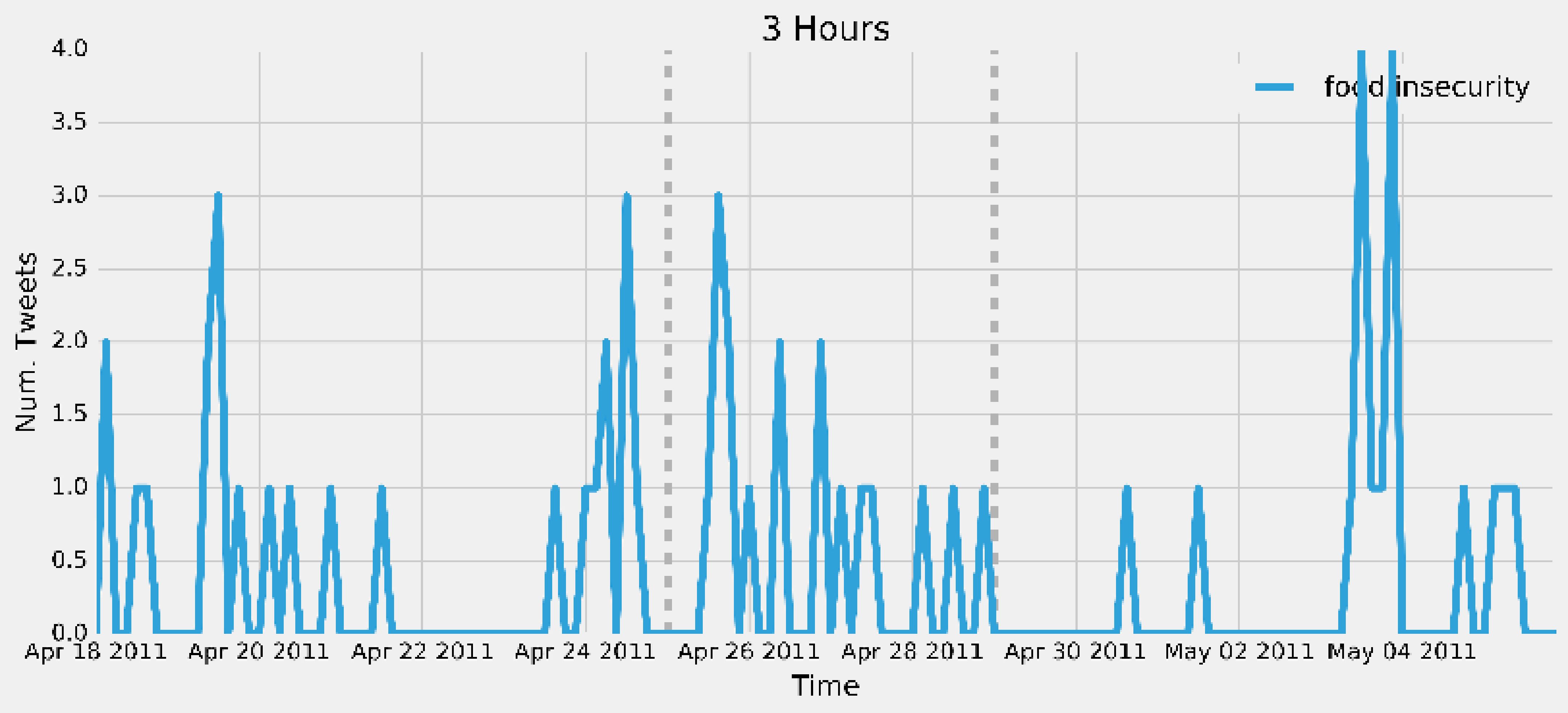

3 Hours
food insecurity
Num. Tweets
4.0
3.5
3.0
2.5
2.0
1.5
1.0
0.5
0.0
Apr 18 2011
Apr 20 2011
Apr 22 2011
Apr 24 2011
Apr 26 2011
Apr 28 2011
Apr 30 2011
May 02 2011
May 04 2011
Time

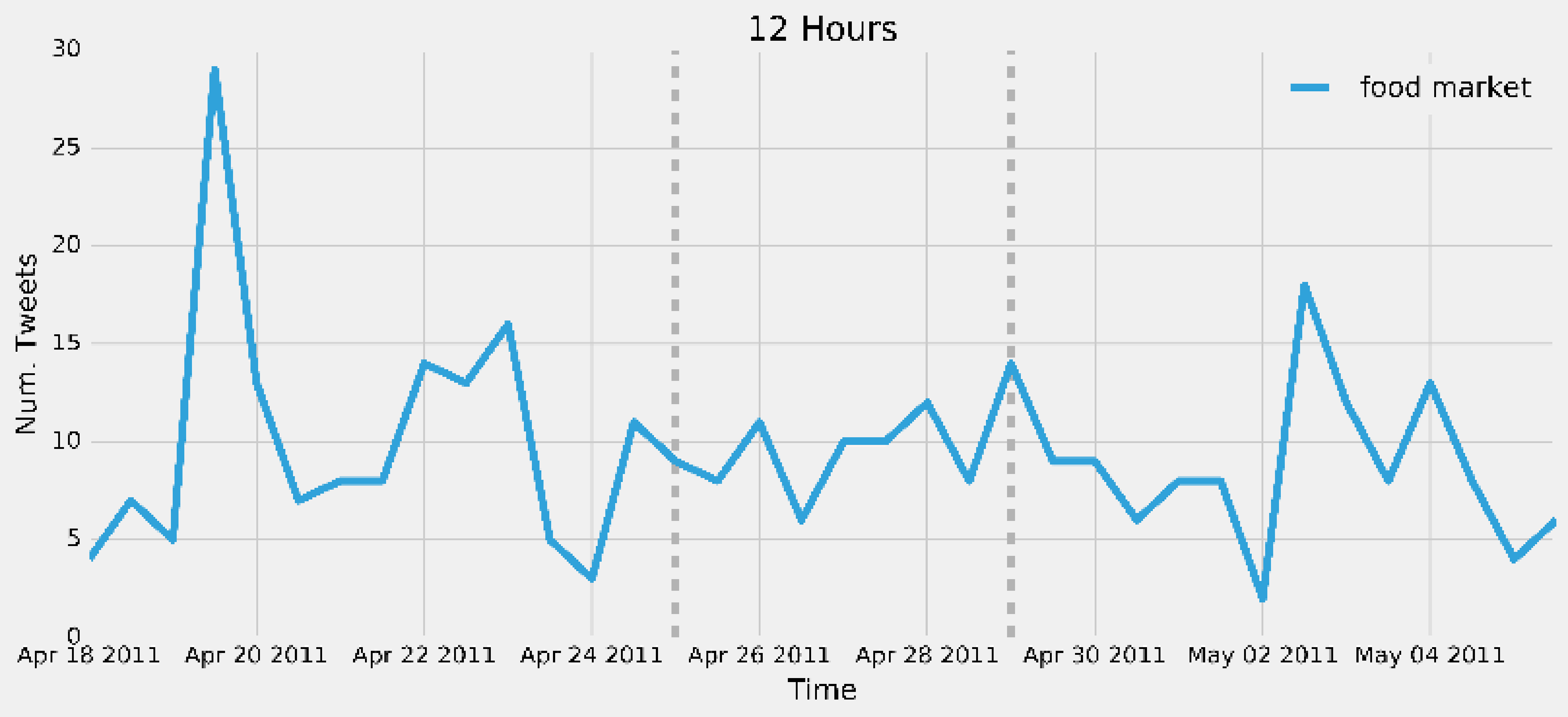
12 Hours
food market
Num. Tweets
Time
0
5
10
15
20
25
30
Apr 18 2011
Apr 20 2011
Apr 22 2011
Apr 24 2011
Apr 26 2011
Apr 28 2011
Apr 30 2011
May 02 2011
May 04 2011

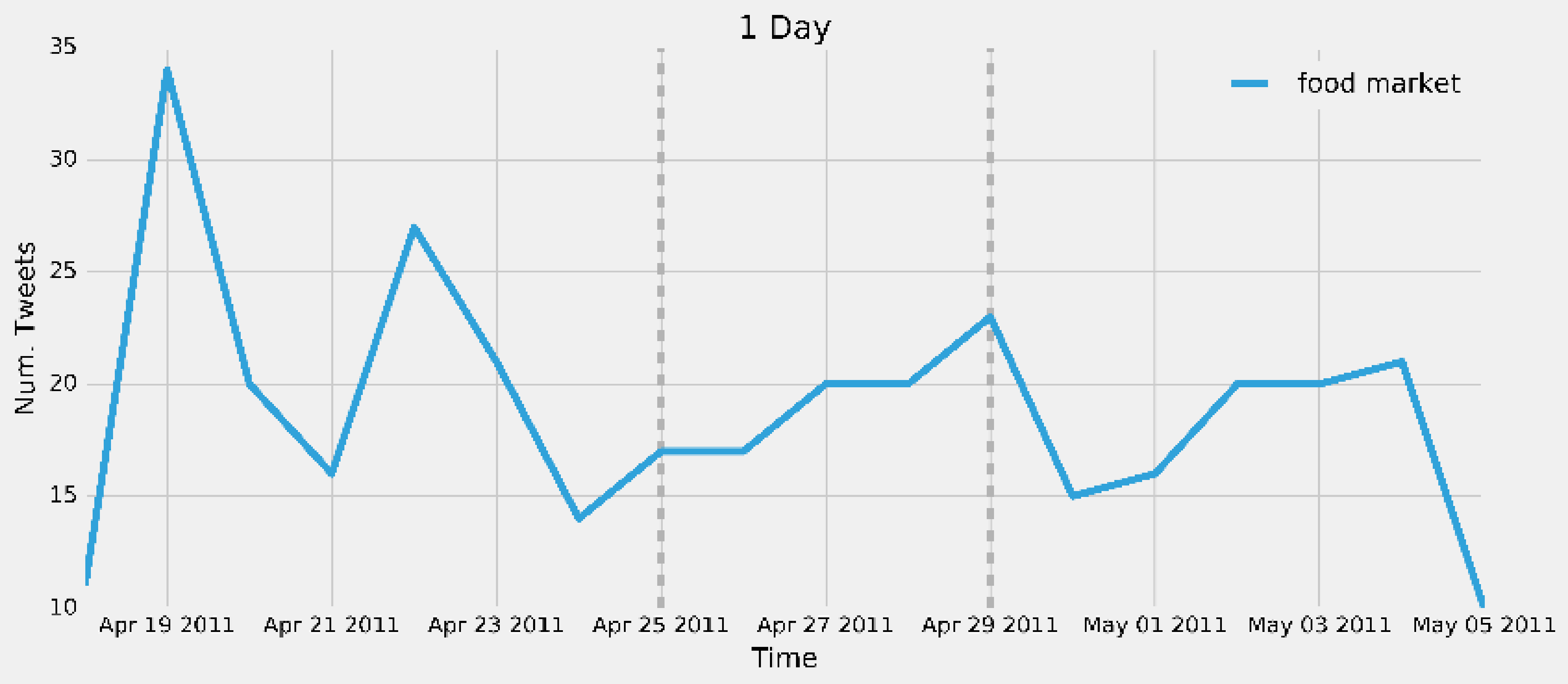
1 Day
35
30
25
20
15
10
Num. Tweets
food market
Apr 19 2011
Apr 21 2011
Apr 23 2011
Apr 25 2011
Apr 27 2011
Apr 29 2011
May 01 2011
May 03 2011
May 05 2011
Time

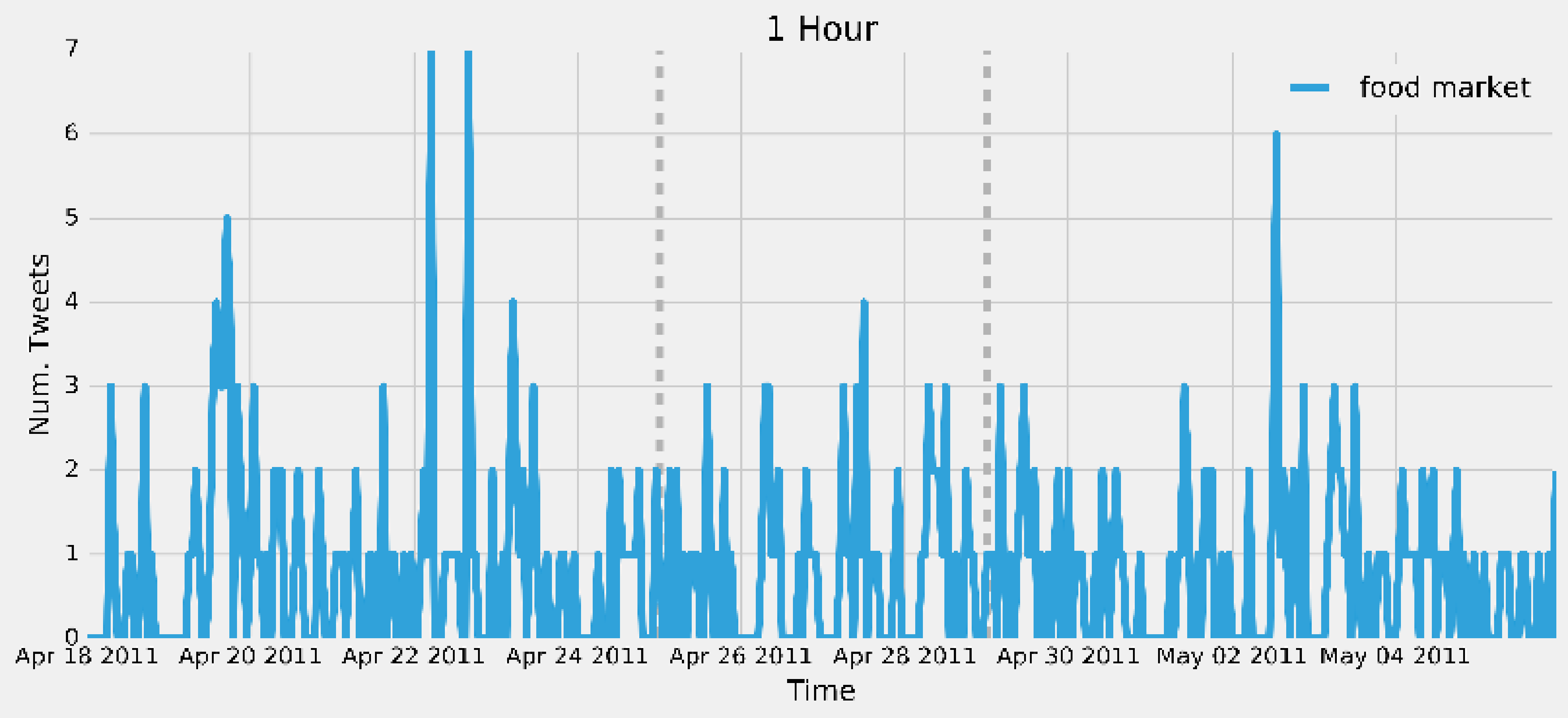
1 Hour
food market
Num. Tweets
0
1
2
3
4
5
6
7
Apr 18 2011
Apr 20 2011
Apr 22 2011
Apr 24 2011
Apr 26 2011
Apr 28 2011
Apr 30 2011
May 02 2011
May 04 2011
Time

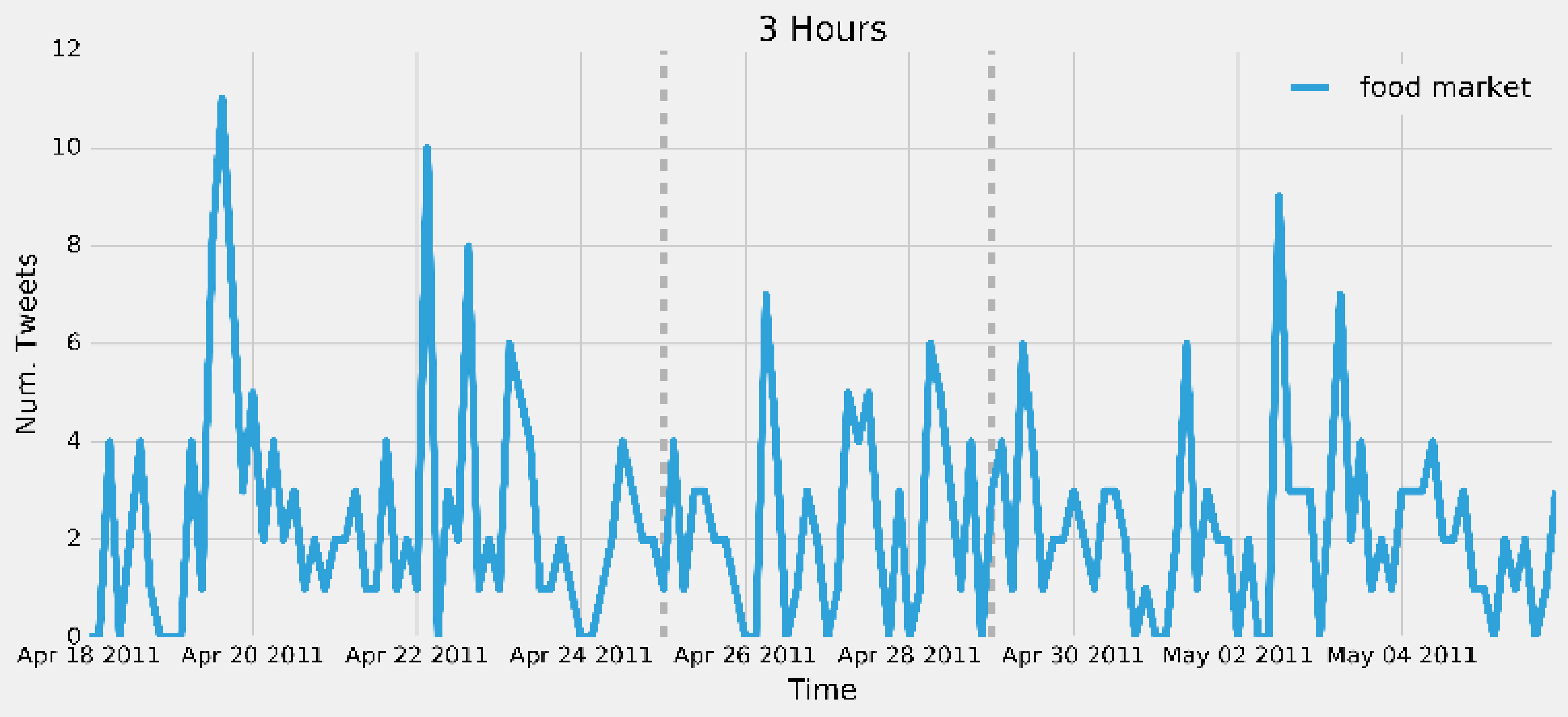

3 Hours
food market
Num. Tweets
Time
0
2
4
6
8
10
12
Apr 18 2011
Apr 20 2011
Apr 22 2011
Apr 24 2011
Apr 26 2011
Apr 28 2011
Apr 30 2011
May 02 2011
May 04 2011

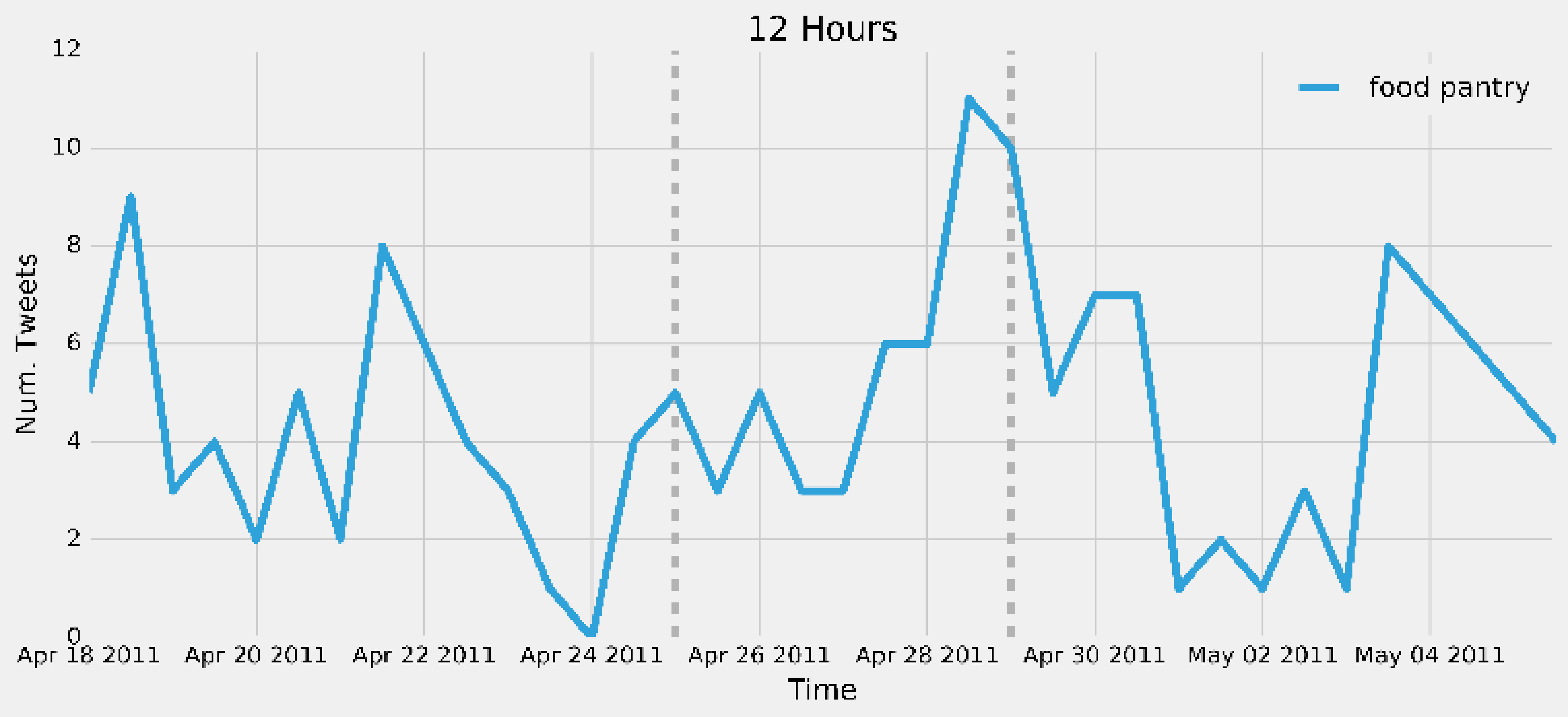
12 Hours
food pantry
Num. Tweets
0
2
4
6
8
10
12
Apr 18 2011
Apr 20 2011
Apr 22 2011
Apr 24 2011
Apr 26 2011
Apr 28 2011
Apr 30 2011
May 02 2011
May 04 2011
Time

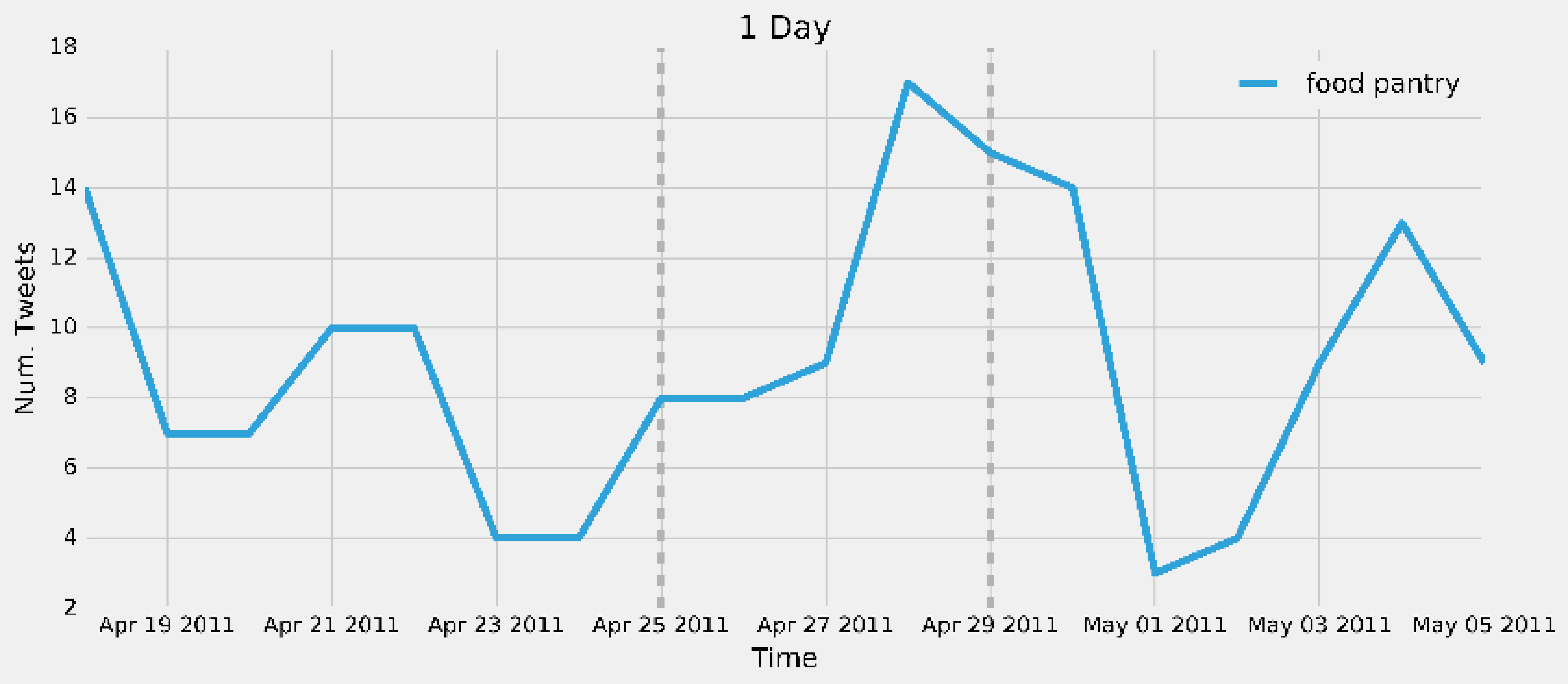
1 Day
food pantry
Num. Tweets
Time
18
16
14
12
10
8
6
4
2
Apr 19 2011
Apr 21 2011
Apr 23 2011
Apr 25 2011
Apr 27 2011
Apr 29 2011
May 01 2011
May 03 2011
May 05 2011

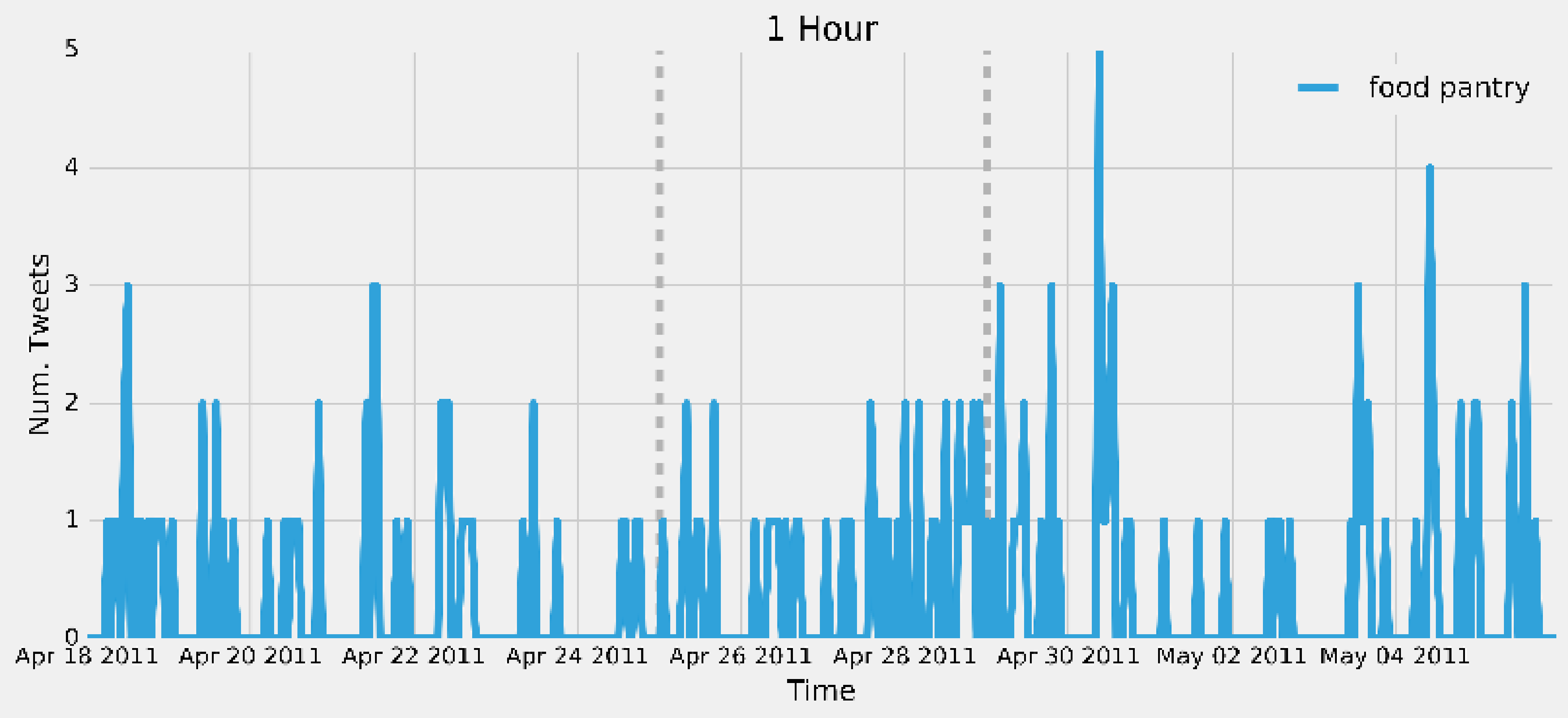
1 Hour
food pantry
Num. Tweets
0
1
2
3
4
5
Apr 18 2011
Apr 20 2011
Apr 22 2011
Apr 24 2011
Apr 26 2011
Apr 28 2011
Apr 30 2011
May 02 2011
May 04 2011
Time

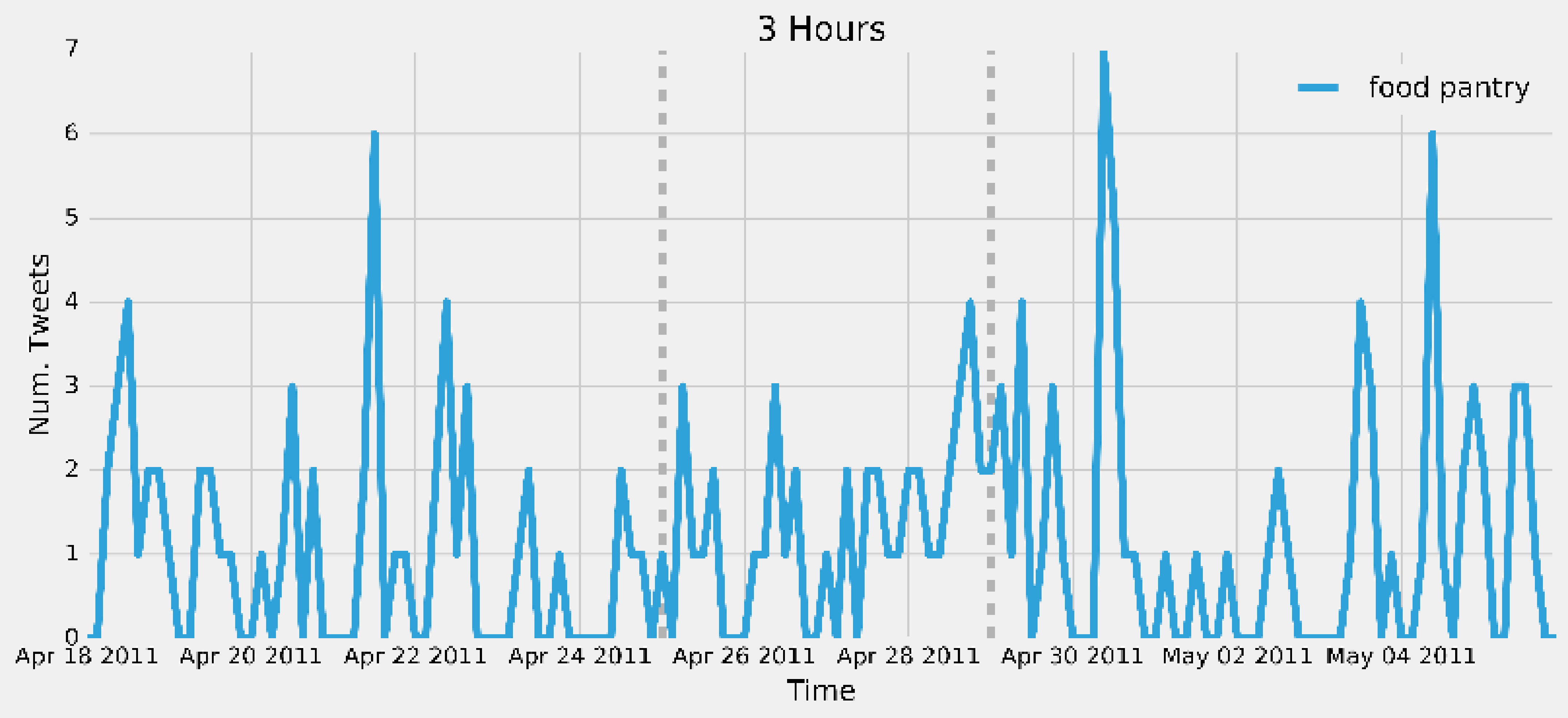
3 Hours
food pantry
Num. Tweets
Time
0
1
2
3
4
5
6
7
Apr 18 2011
Apr 20 2011
Apr 22 2011
Apr 24 2011
Apr 26 2011
Apr 28 2011
Apr 30 2011
May 02 2011
May 04 2011

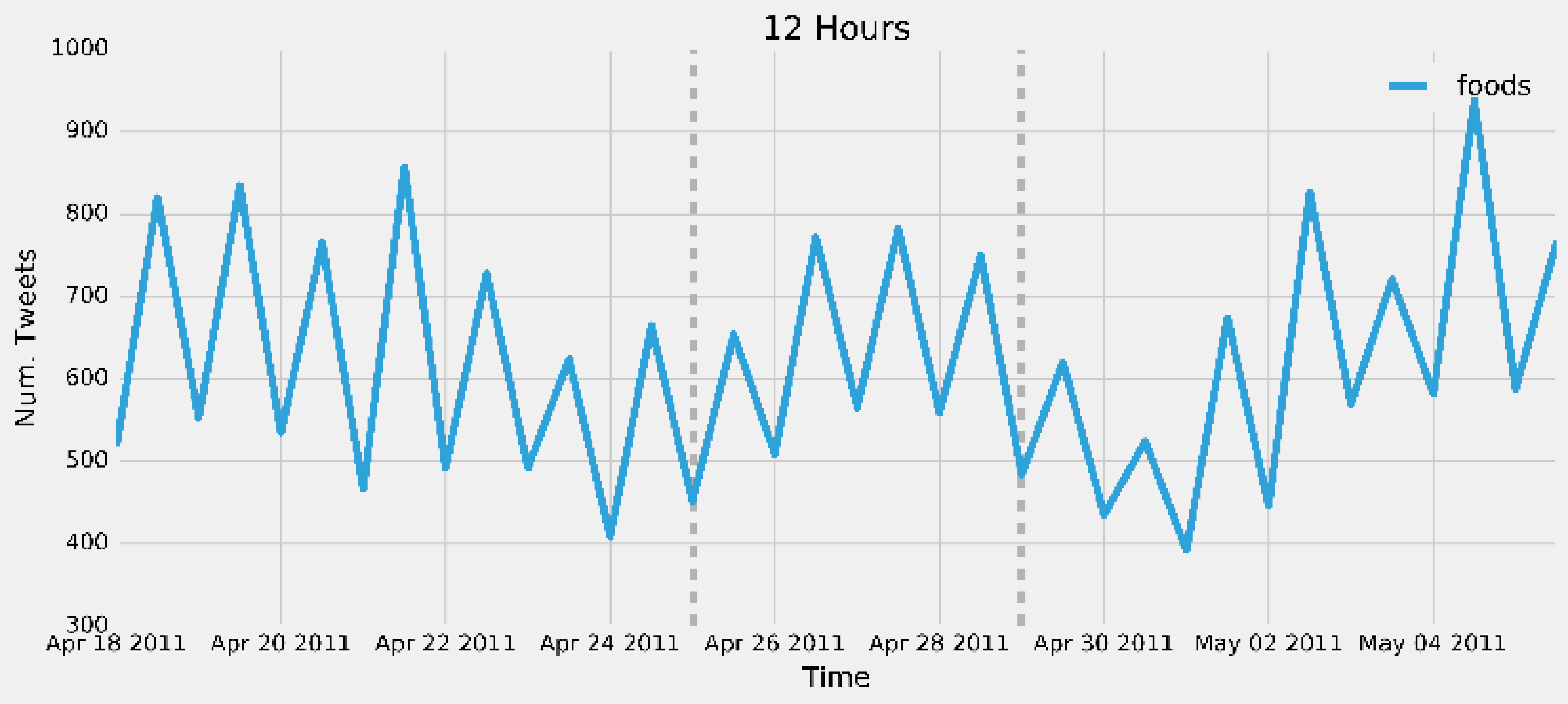

12 Hours
foods
Num. Tweets
1000
900
800
700
600
500
400
300
Apr 18 2011
Apr 20 2011
Apr 22 2011
Apr 24 2011
Apr 26 2011
Apr 28 2011
Apr 30 2011
May 02 2011
May 04 2011
Time

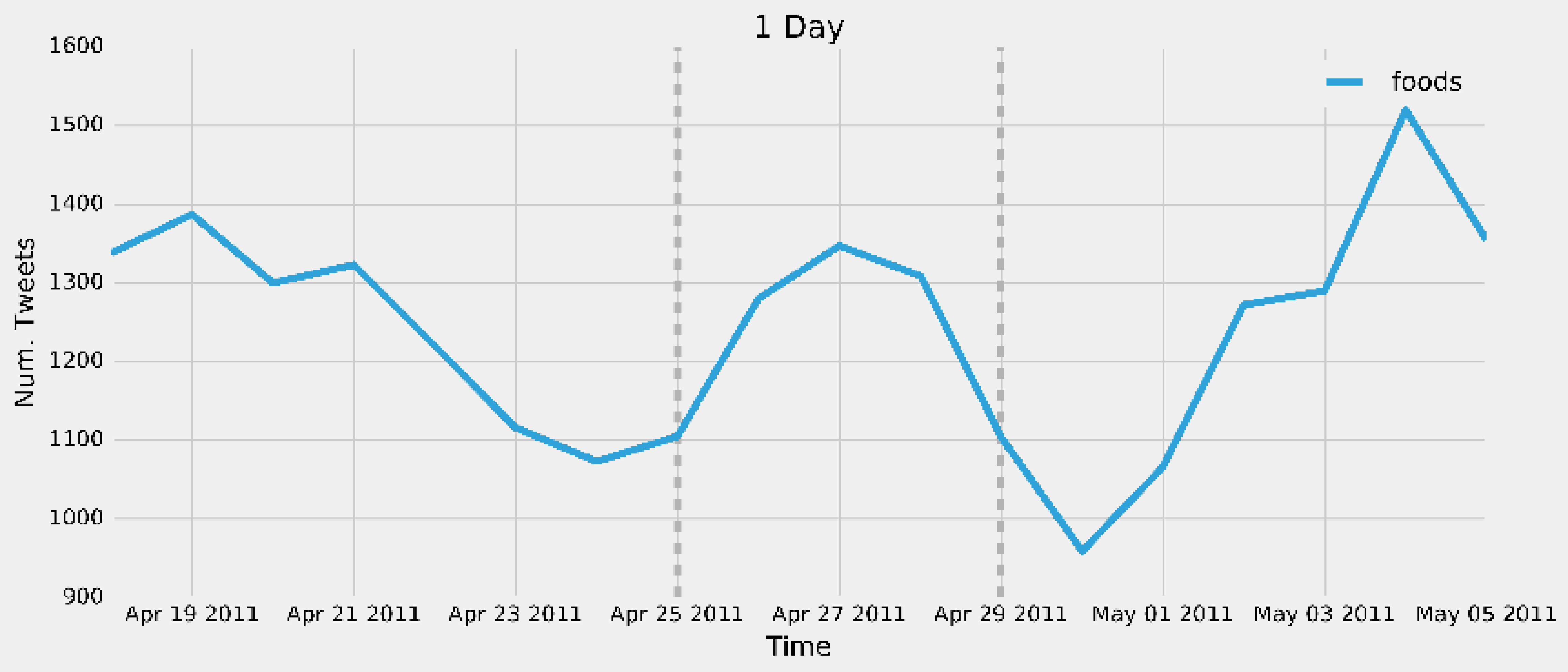

1 Day
foods
Num. Tweets
Time
1600
1500
1400
1300
1200
1100
1000
900
Apr 19 2011
Apr 21 2011
Apr 23 2011
Apr 25 2011
Apr 27 2011
Apr 29 2011
May 01 2011
May 03 2011
May 05 2011

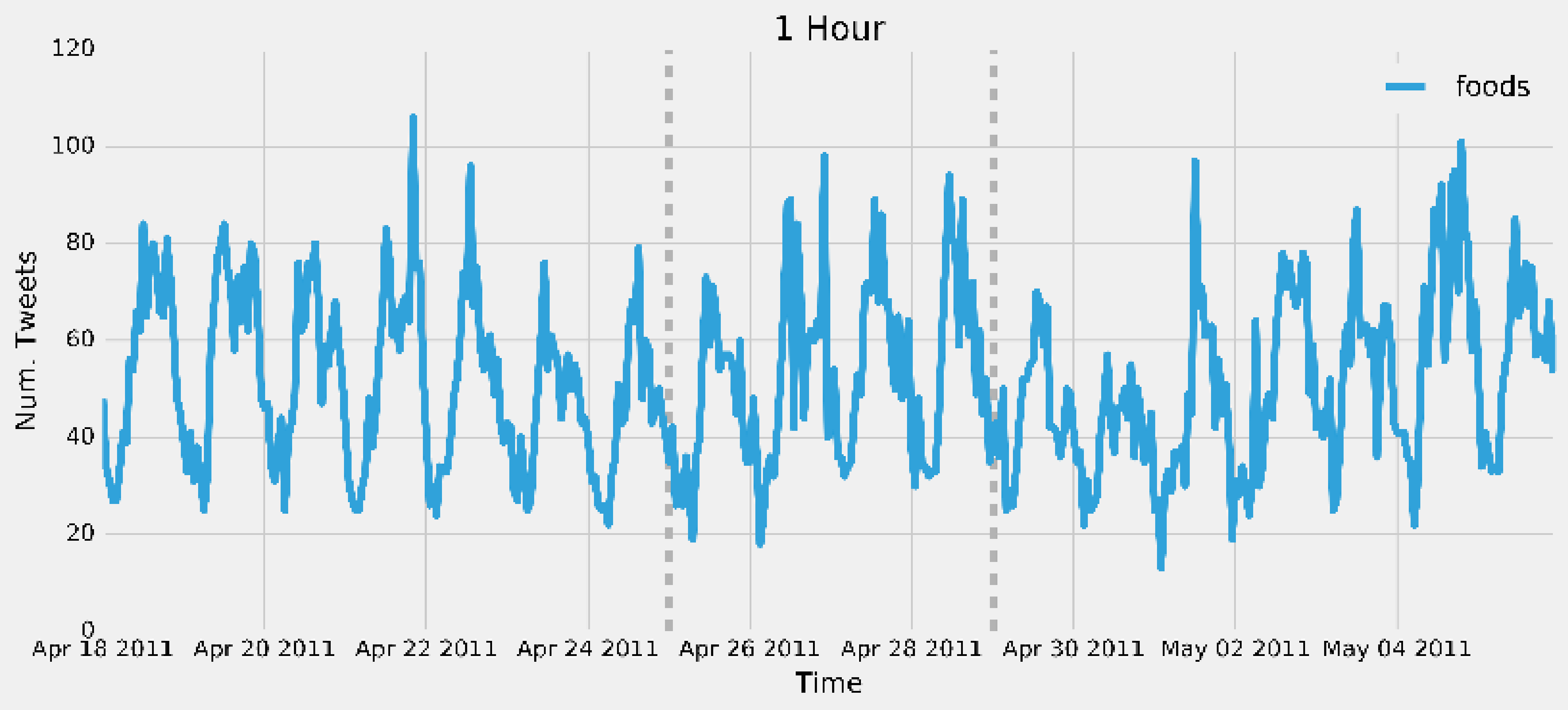
1 Hour
foods
Num. Tweets
0
20
40
60
80
100
120
Apr 18 2011
Apr 20 2011
Apr 22 2011
Apr 24 2011
Apr 26 2011
Apr 28 2011
Apr 30 2011
May 02 2011
May 04 2011
Time

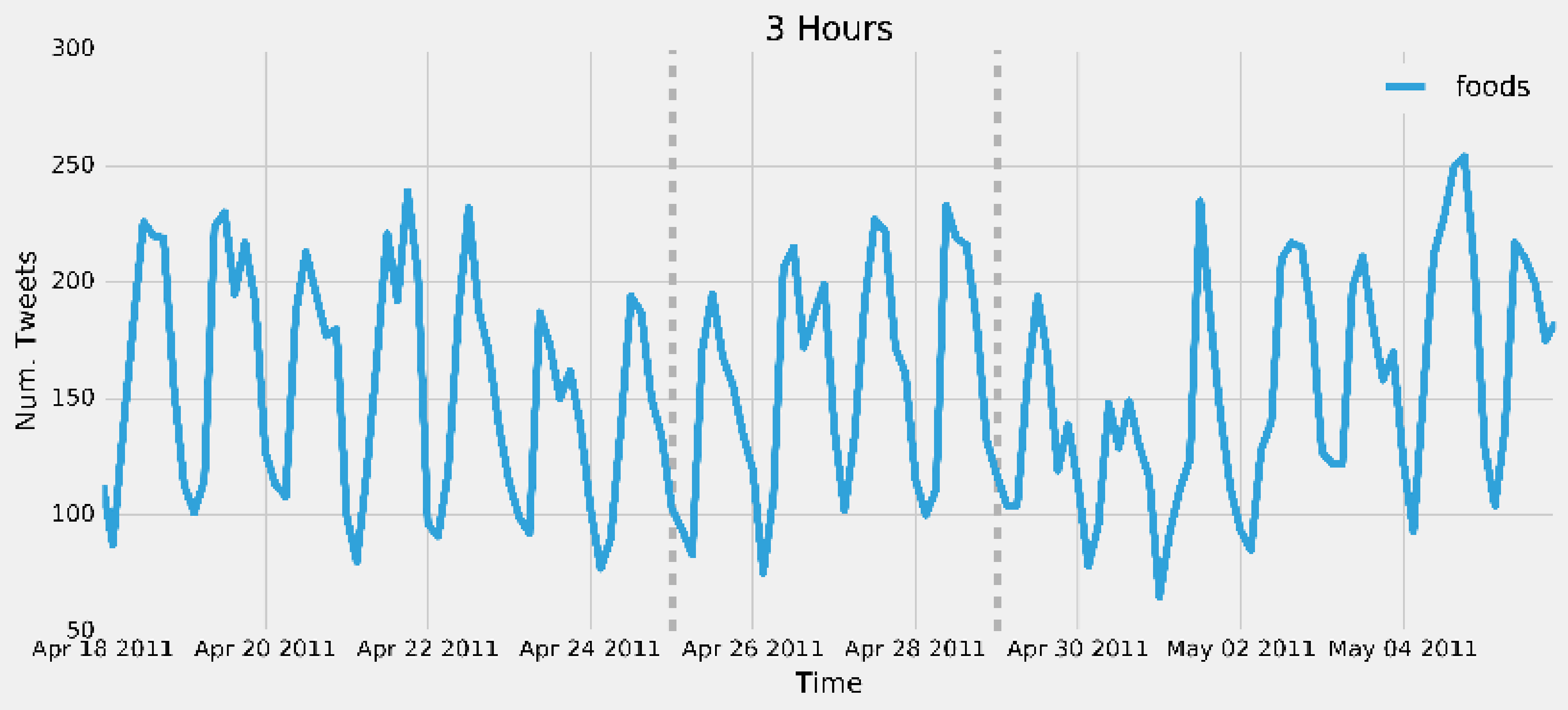
3 Hours
foods
Num. Tweets
300
250
200
150
100
50
Apr 18 2011
Apr 20 2011
Apr 22 2011
Apr 24 2011
Apr 26 2011
Apr 28 2011
Apr 30 2011
May 02 2011
May 04 2011
Time

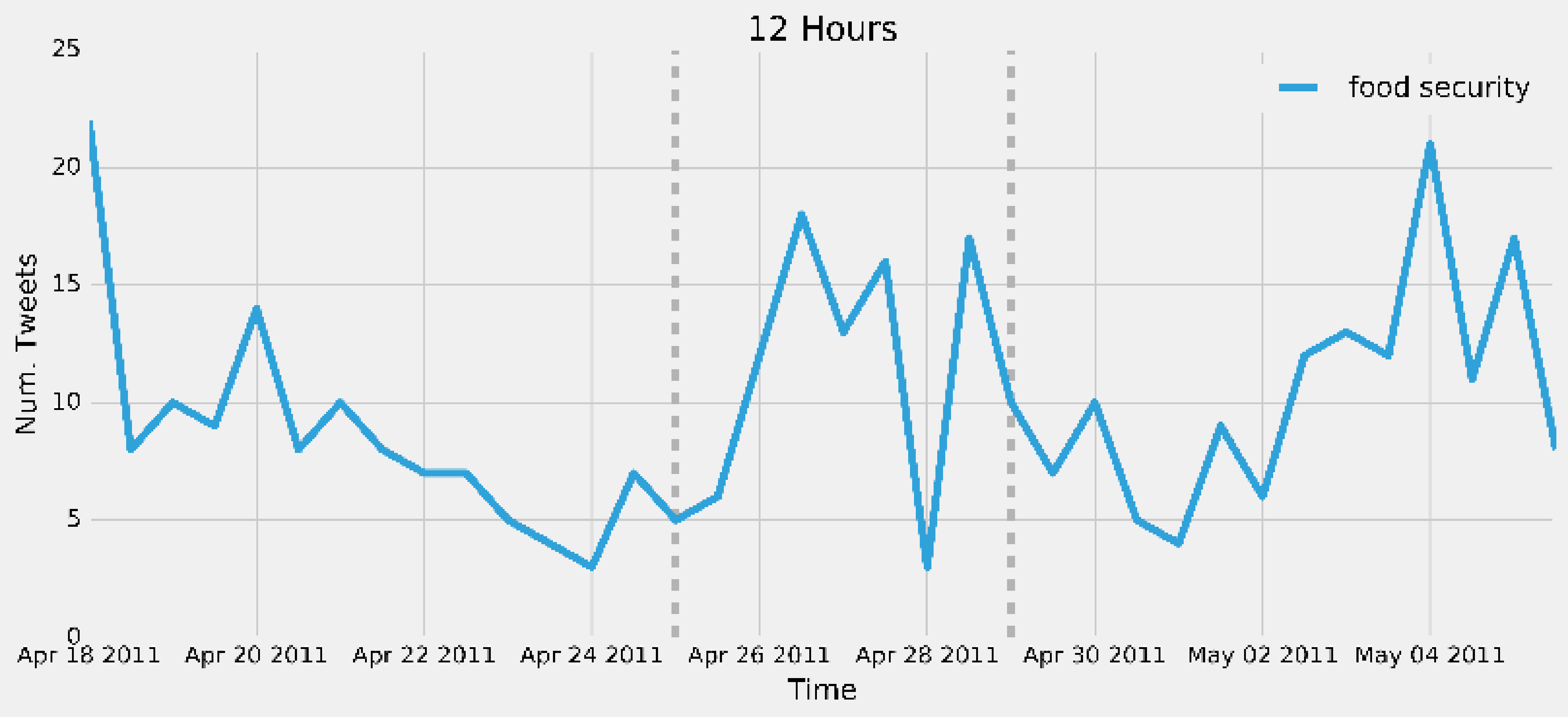

12 Hours
food security
Num. Tweets
0
5
10
15
20
25
Apr 18 2011
Apr 20 2011
Apr 22 2011
Apr 24 2011
Apr 26 2011
Apr 28 2011
Apr 30 2011
May 02 2011
May 04 2011
Time

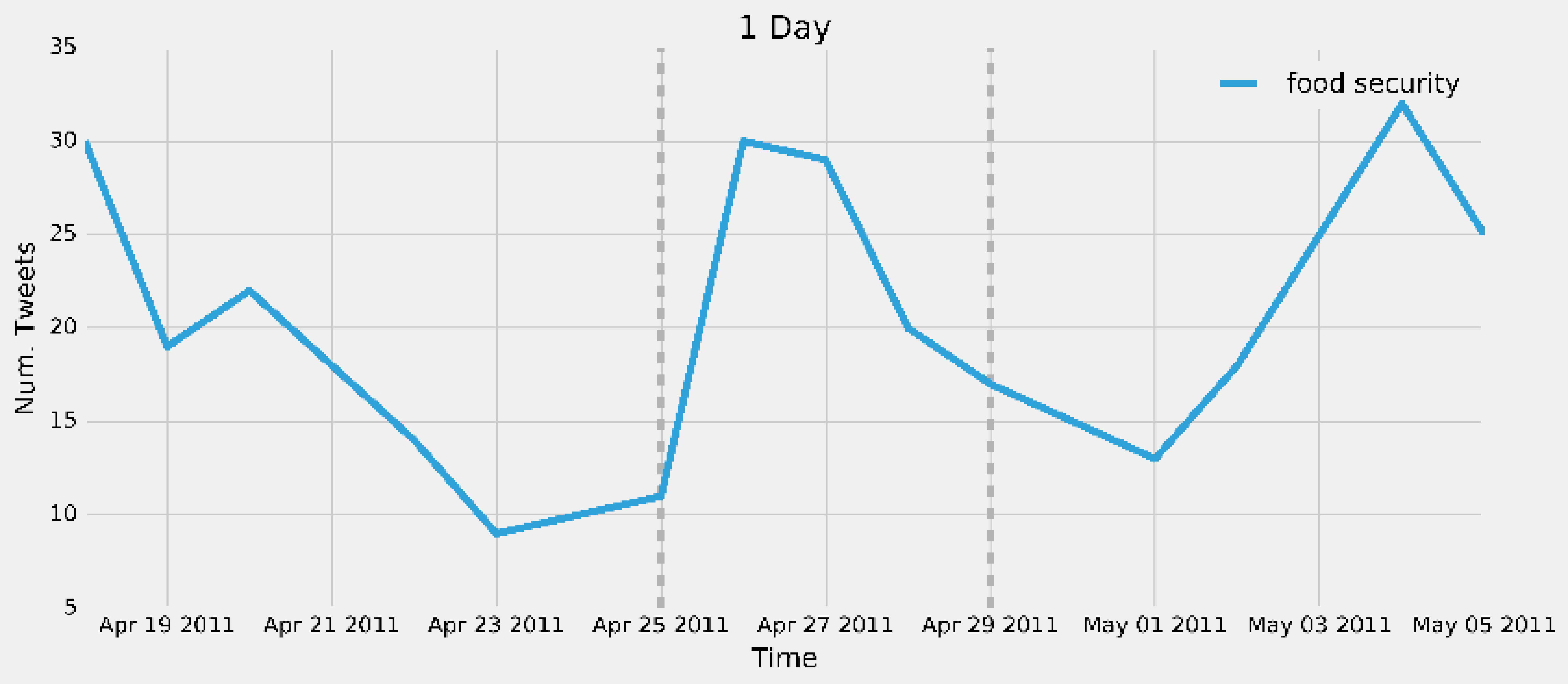
1 Day
food security
Num. Tweets
Time
35
30
25
20
15
10
5
Apr 19 2011
Apr 21 2011
Apr 23 2011
Apr 25 2011
Apr 27 2011
Apr 29 2011
May 01 2011
May 03 2011
May 05 2011

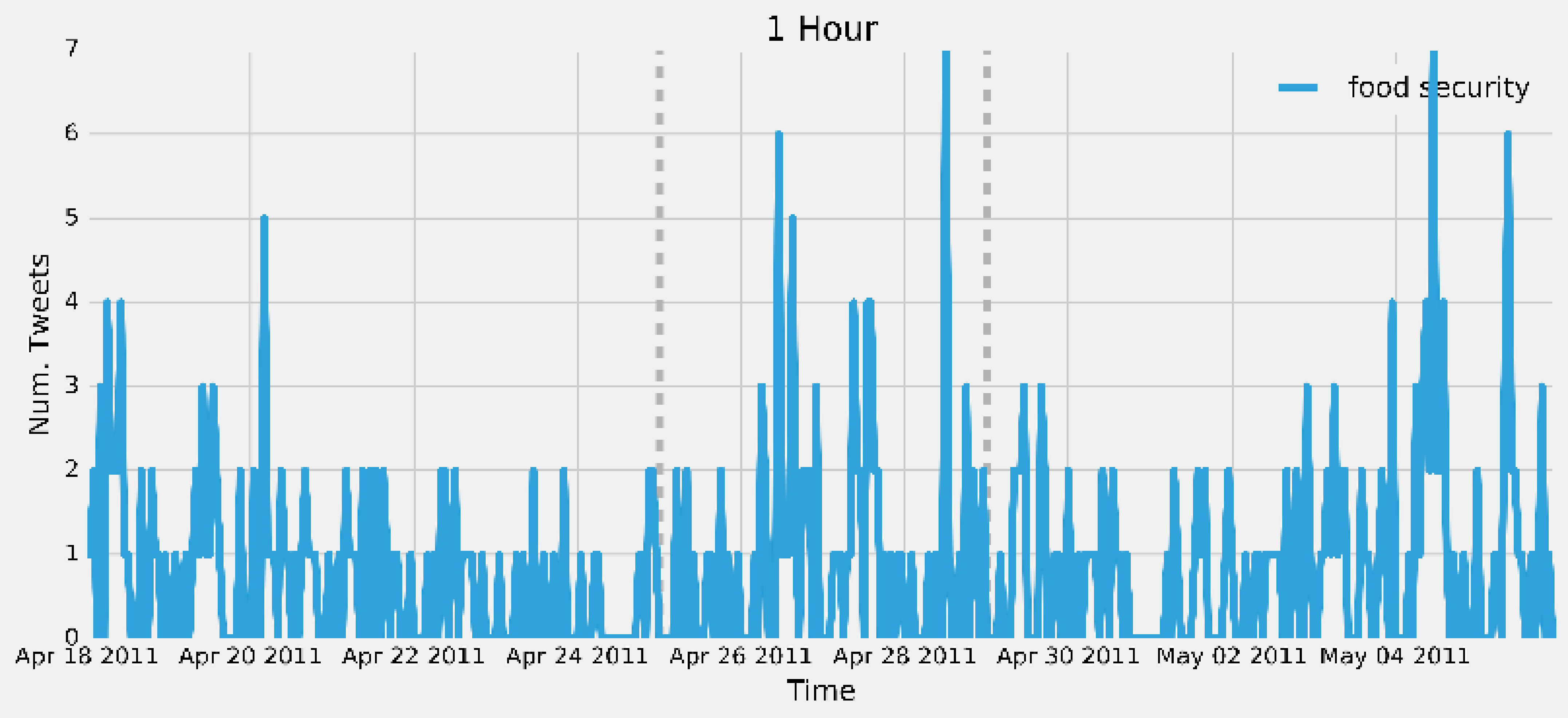

1 Hour
food security
Num. Tweets
0
1
2
3
4
5
6
7
Apr 18 2011
Apr 20 2011
Apr 22 2011
Apr 24 2011
Apr 26 2011
Apr 28 2011
Apr 30 2011
May 02 2011
May 04 2011
Time

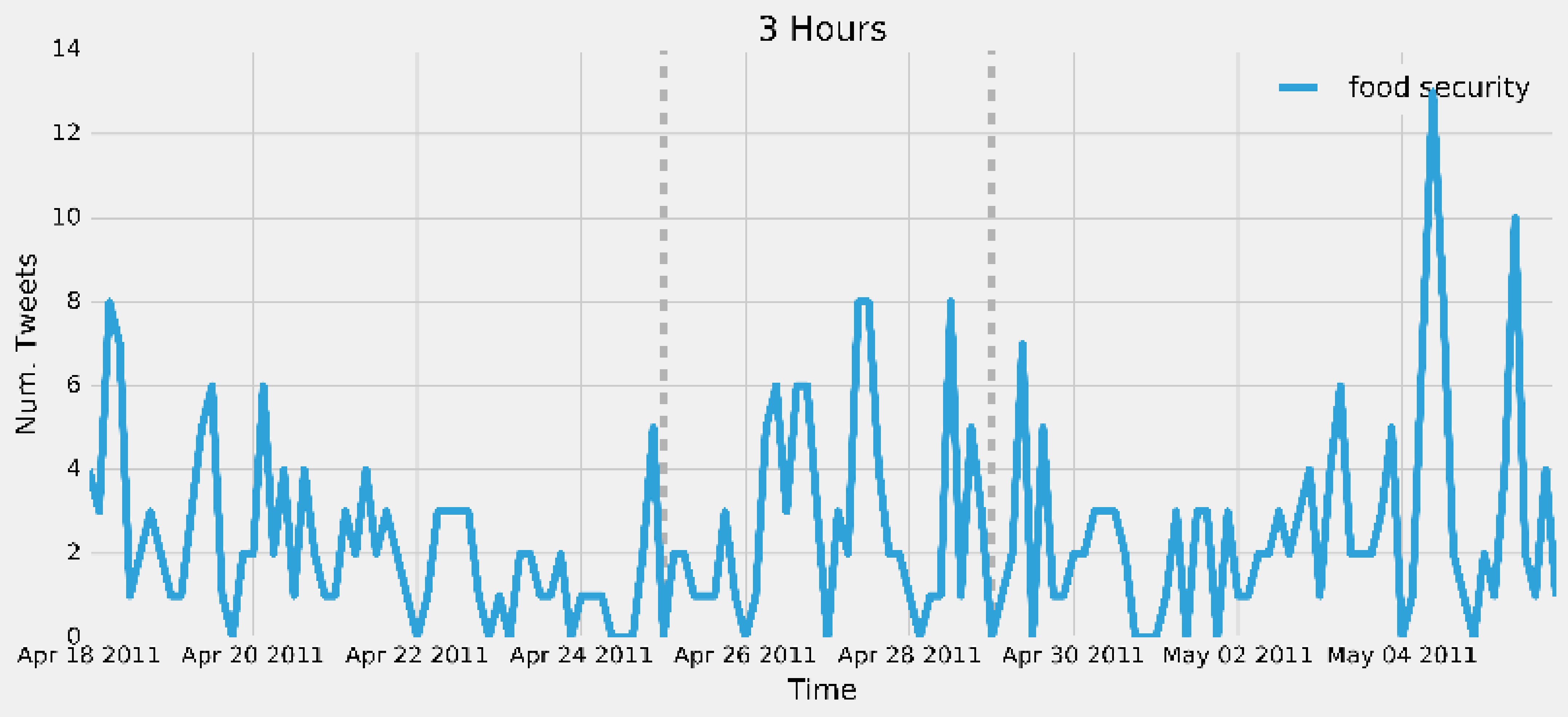

3 Hours
food security
Num. Tweets
Time
0
2
4
6
8
10
12
14
Apr 18 2011
Apr 20 2011
Apr 22 2011
Apr 24 2011
Apr 26 2011
Apr 28 2011
Apr 30 2011
May 02 2011
May 04 2011

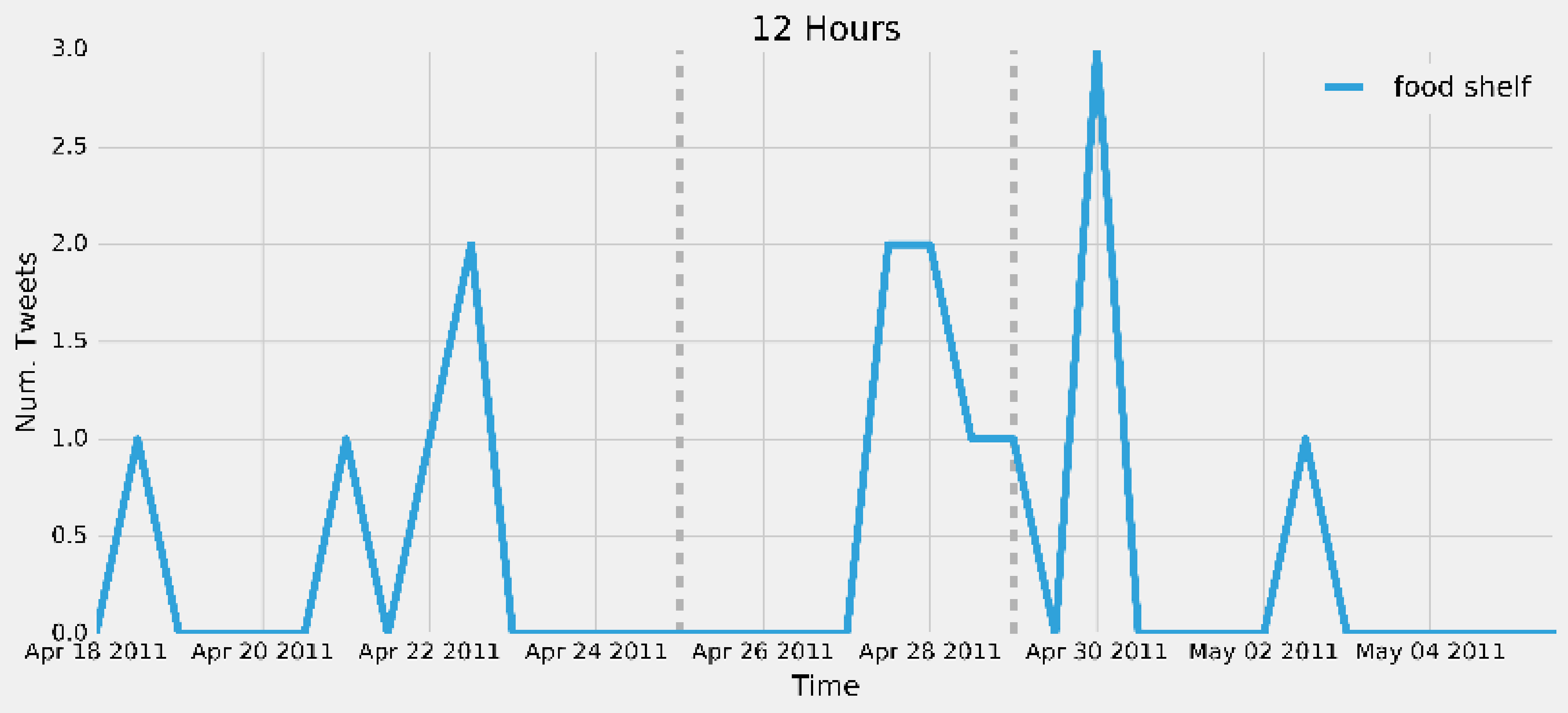
12 Hours
food shelf
Num. Tweets
Time
3.0
2.5
2.0
1.5
1.0
0.5
0.0
Apr 18 2011
Apr 20 2011
Apr 22 2011
Apr 24 2011
Apr 26 2011
Apr 28 2011
Apr 30 2011
May 02 2011
May 04 2011

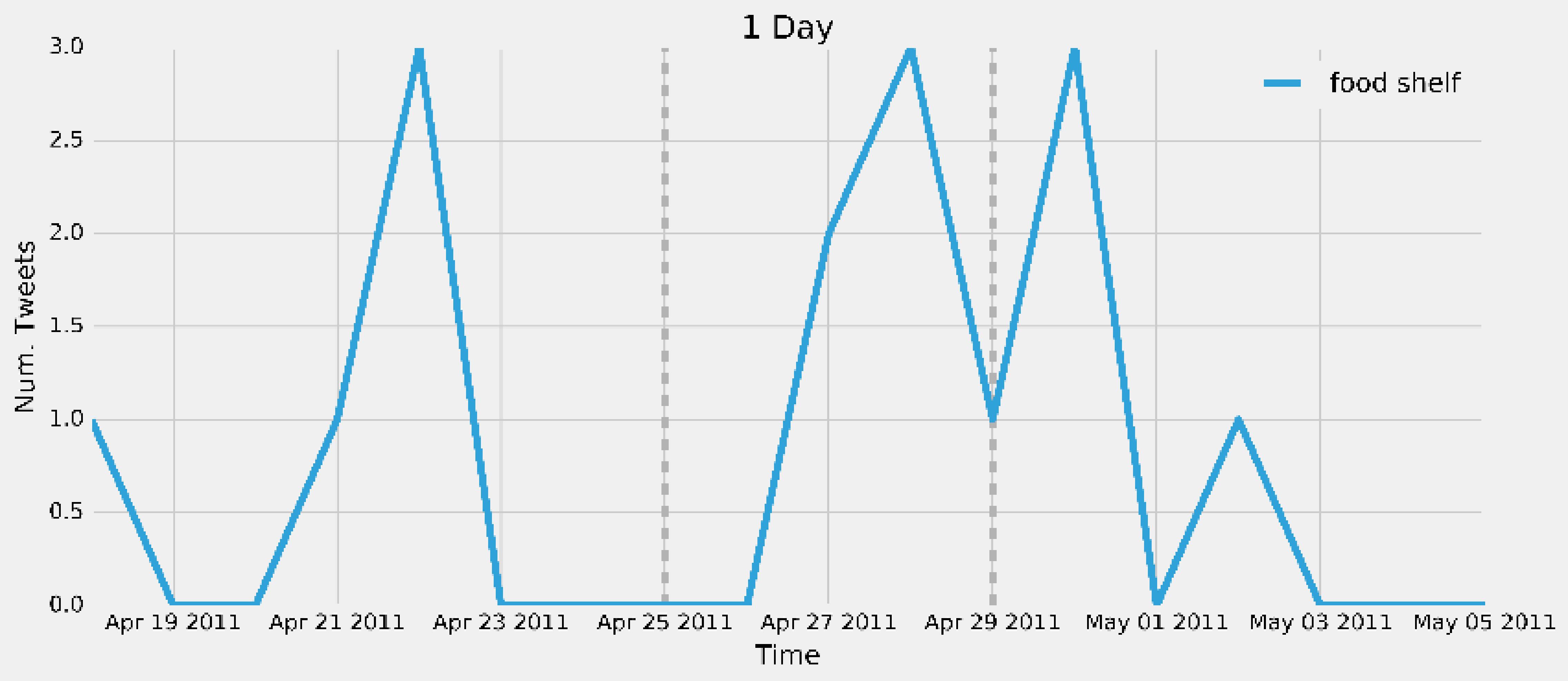

1 Day
3.0
2.5
2.0
1.5
1.0
0.5
0.0
Num. Tweets
food shelf
Apr 19 2011
Apr 21 2011
Apr 23 2011
Apr 25 2011
Apr 27 2011
Apr 29 2011
May 01 2011
May 03 2011
May 05 2011
Time

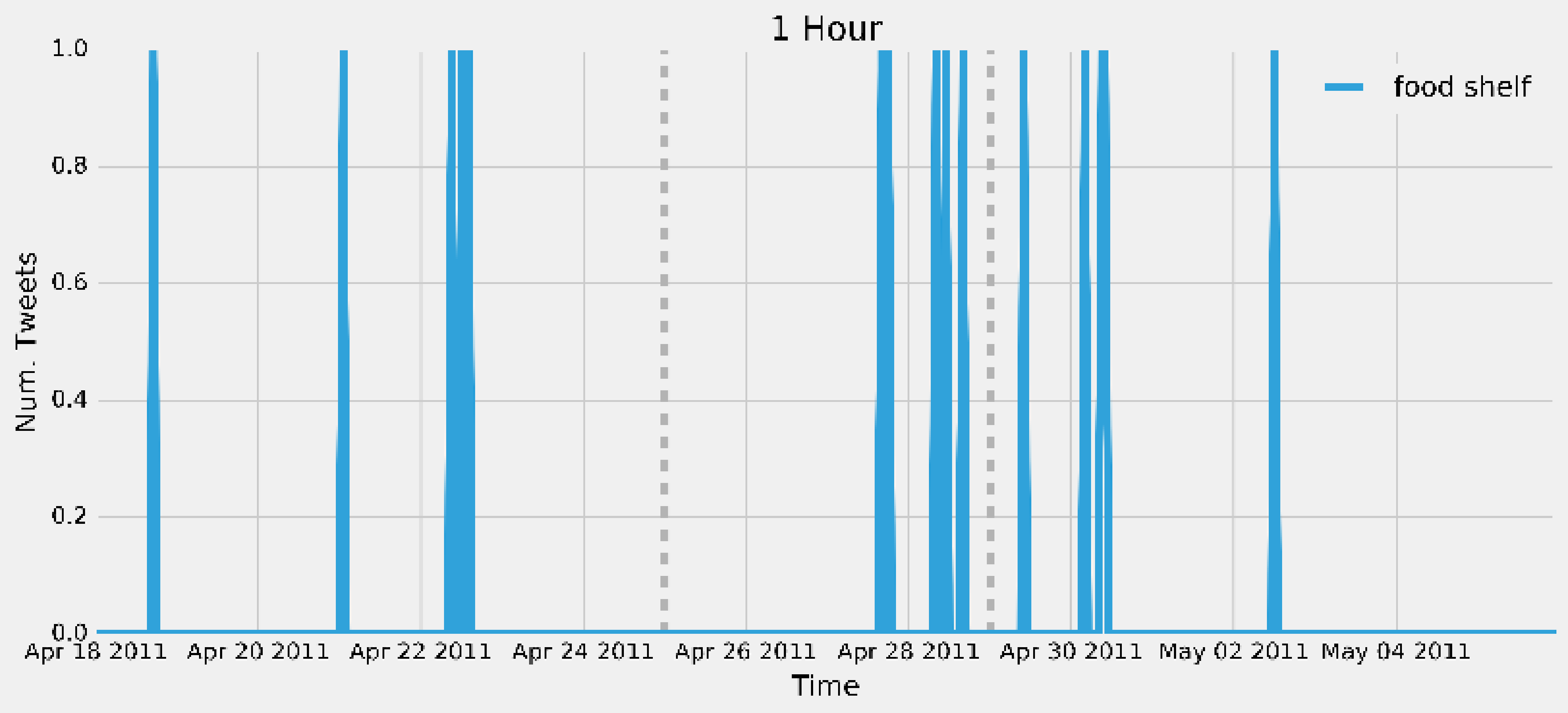
1 Hour
food shelf
Num. Tweets
1.0
0.8
0.6
0.4
0.2
0.0
Apr 18 2011
Apr 20 2011
Apr 22 2011
Apr 24 2011
Apr 26 2011
Apr 28 2011
Apr 30 2011
May 02 2011
May 04 2011
Time

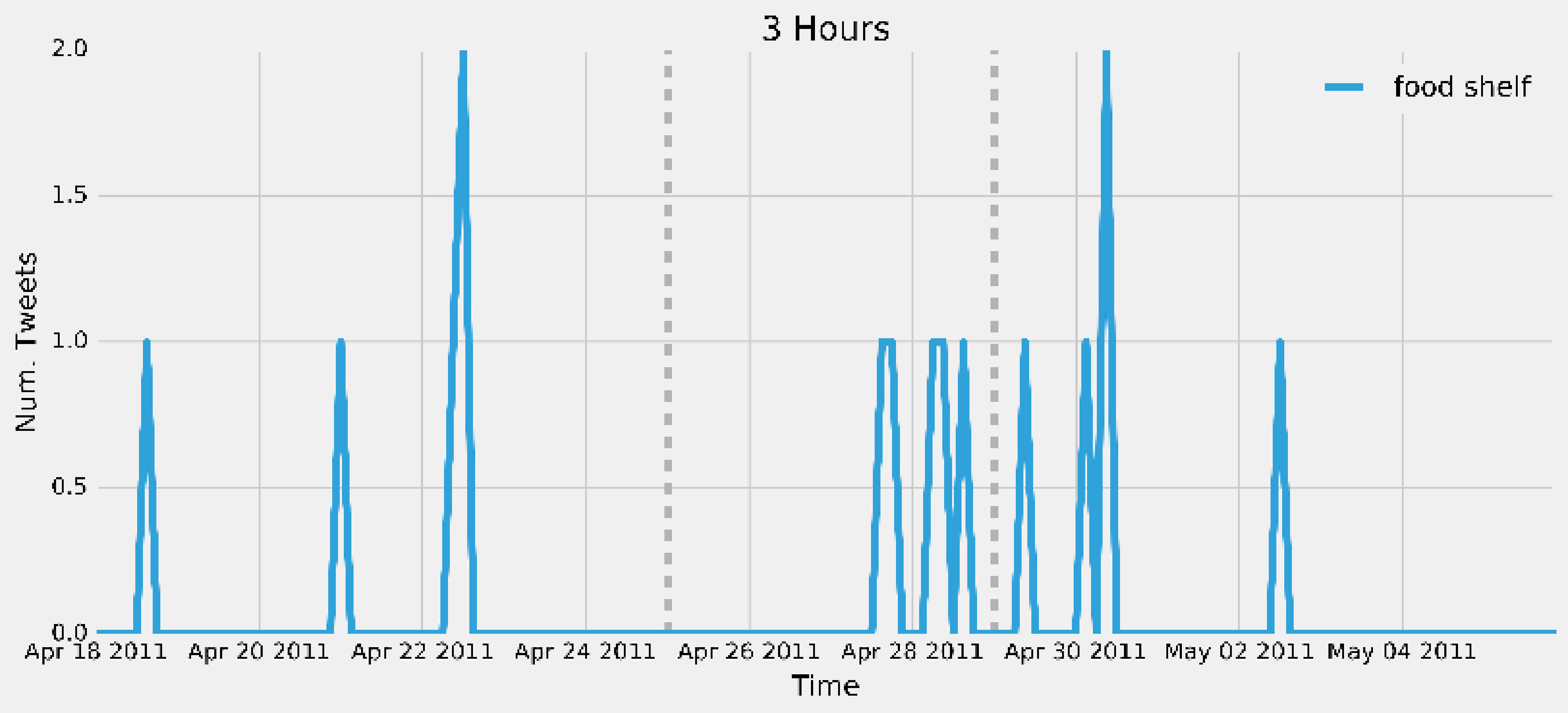
3 Hours
food shelf
Num. Tweets
0.0
0.5
1.0
1.5
2.0
Apr 18 2011
Apr 20 2011
Apr 22 2011
Apr 24 2011
Apr 26 2011
Apr 28 2011
Apr 30 2011
May 02 2011
May 04 2011
Time

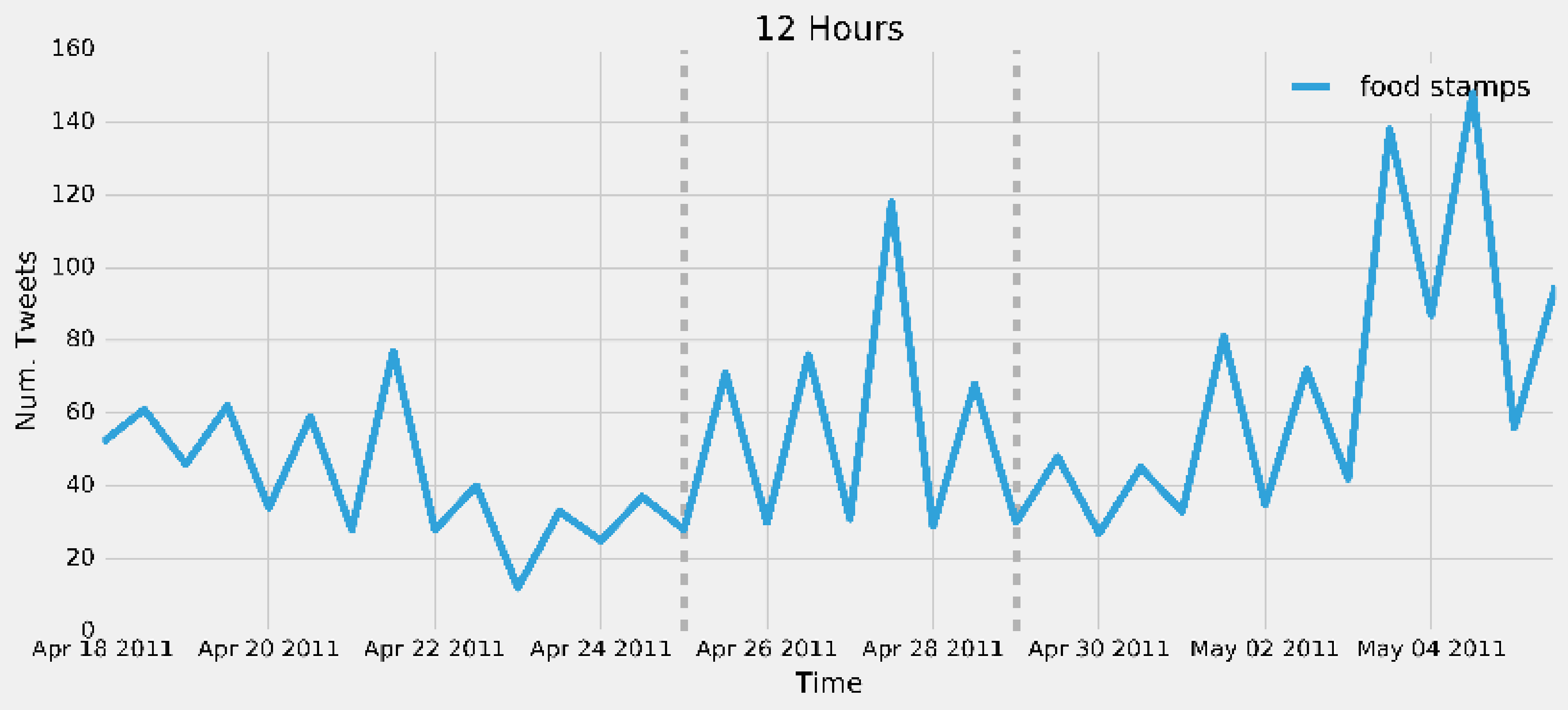
12 Hours
food stamps
Num. Tweets
0
20
40
60
80
100
120
140
160
Apr 18 2011
Apr 20 2011
Apr 22 2011
Apr 24 2011
Apr 26 2011
Apr 28 2011
Apr 30 2011
May 02 2011
May 04 2011
Time

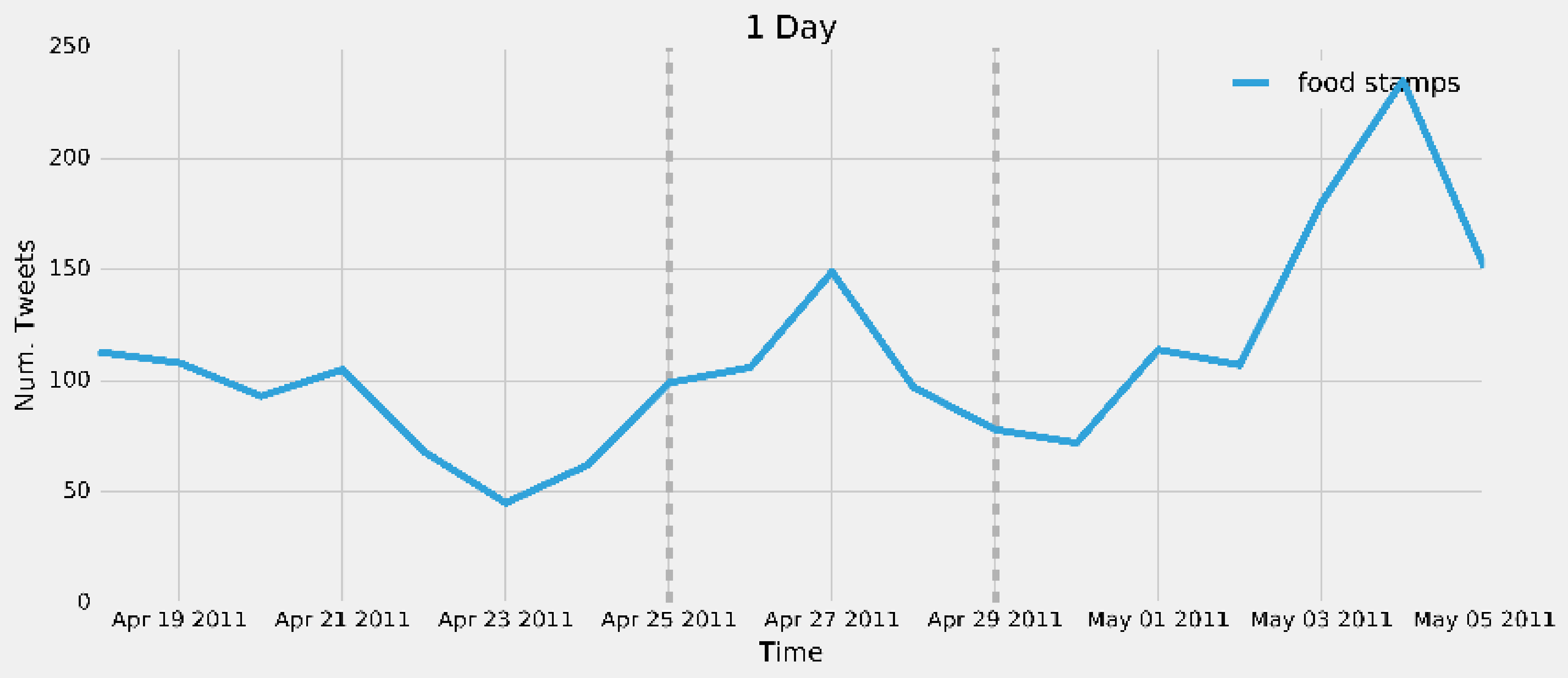

1 Day
food stamps
Num. Tweets
Time
0
50
100
150
200
250
Apr 19 2011
Apr 21 2011
Apr 23 2011
Apr 25 2011
Apr 27 2011
Apr 29 2011
May 01 2011
May 03 2011
May 05 2011

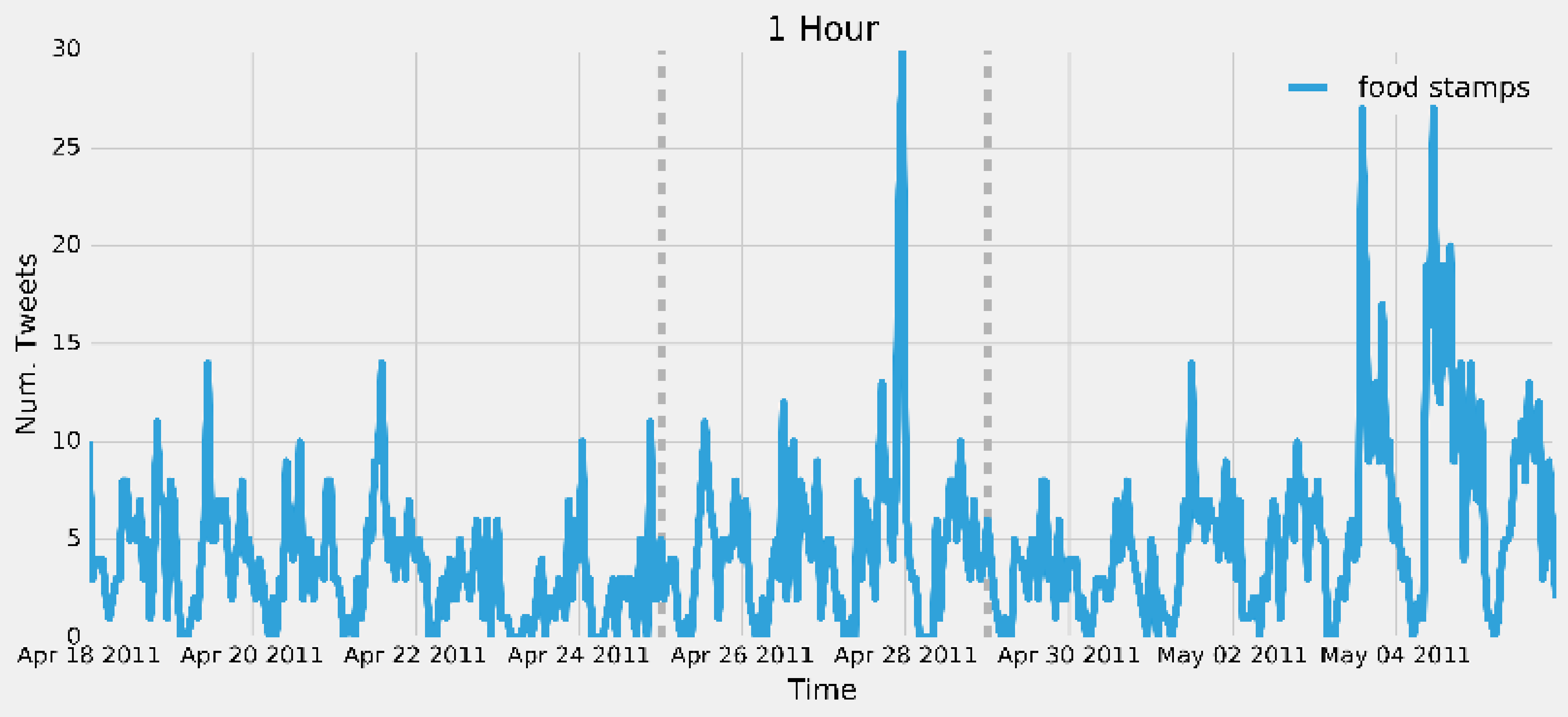

1 Hour
food stamps
Num. Tweets
0
5
10
15
20
25
30
Apr 18 2011
Apr 20 2011
Apr 22 2011
Apr 24 2011
Apr 26 2011
Apr 28 2011
Apr 30 2011
May 02 2011
May 04 2011
Time

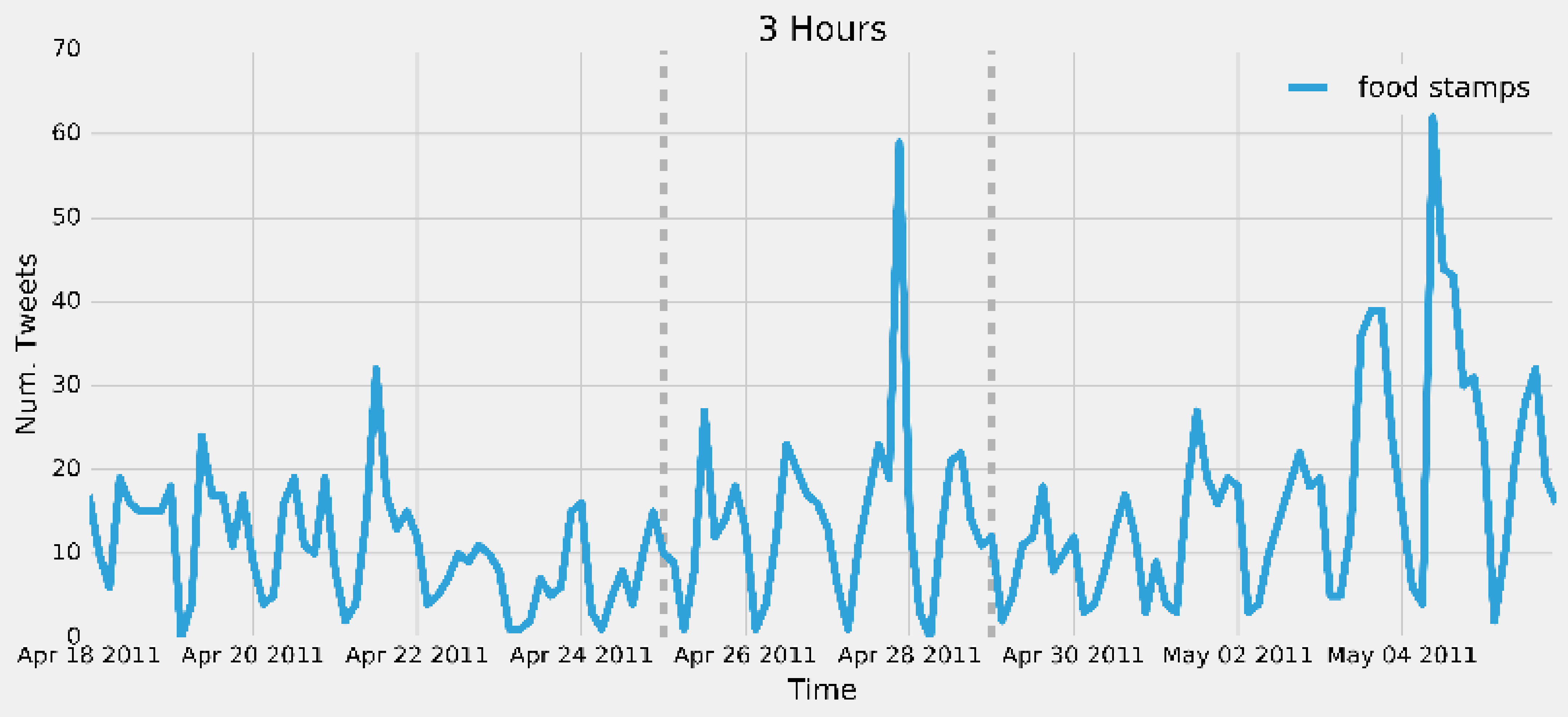
3 Hours
food stamps
Num. Tweets
0
10
20
30
40
50
60
70
Apr 18 2011
Apr 20 2011
Apr 22 2011
Apr 24 2011
Apr 26 2011
Apr 28 2011
Apr 30 2011
May 02 2011
May 04 2011
Time

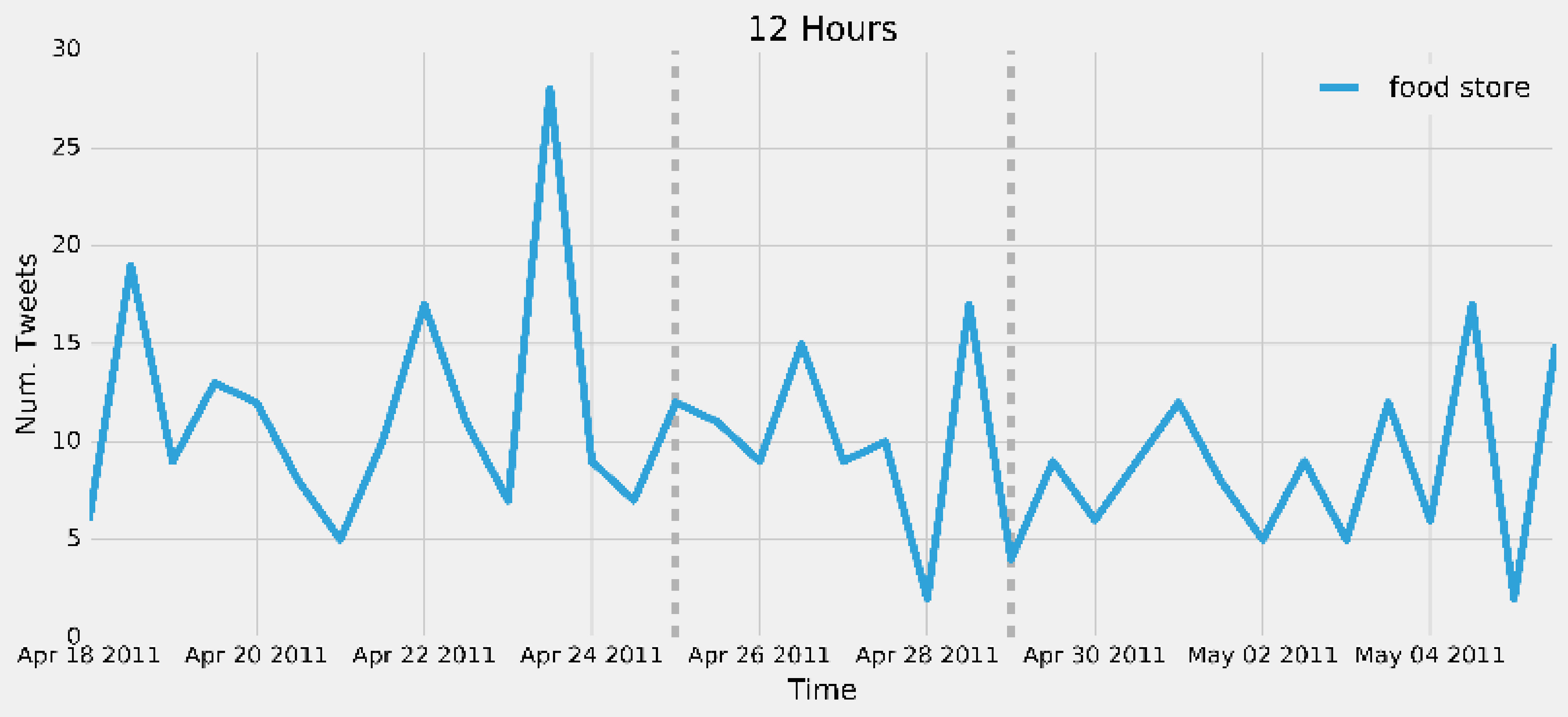
12 Hours
food store
Num. Tweets
0
5
10
15
20
25
30
Apr 18 2011
Apr 20 2011
Apr 22 2011
Apr 24 2011
Apr 26 2011
Apr 28 2011
Apr 30 2011
May 02 2011
May 04 2011
Time

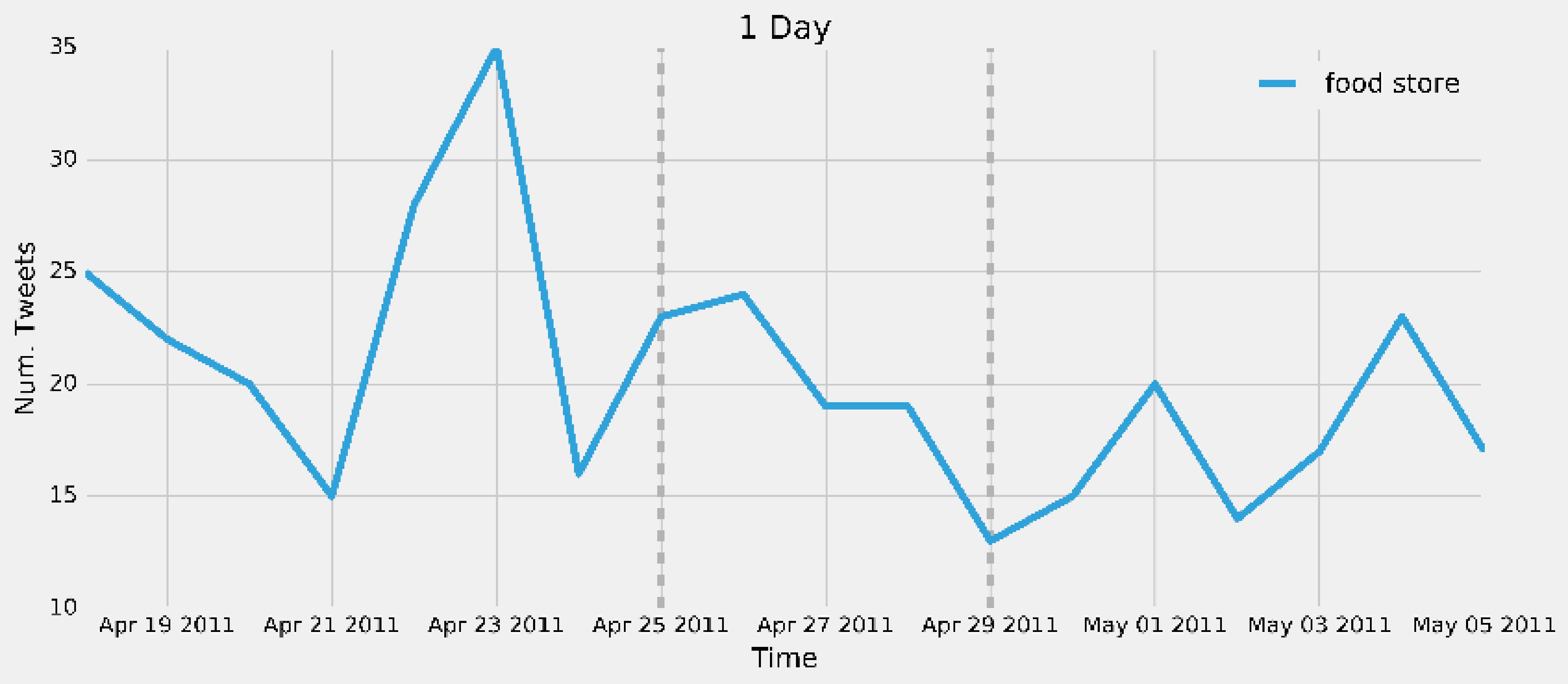
1 Day
food store
Num. Tweets
35
30
25
20
15
10
Apr 19 2011
Apr 21 2011
Apr 23 2011
Apr 25 2011
Apr 27 2011
Apr 29 2011
May 01 2011
May 03 2011
May 05 2011
Time

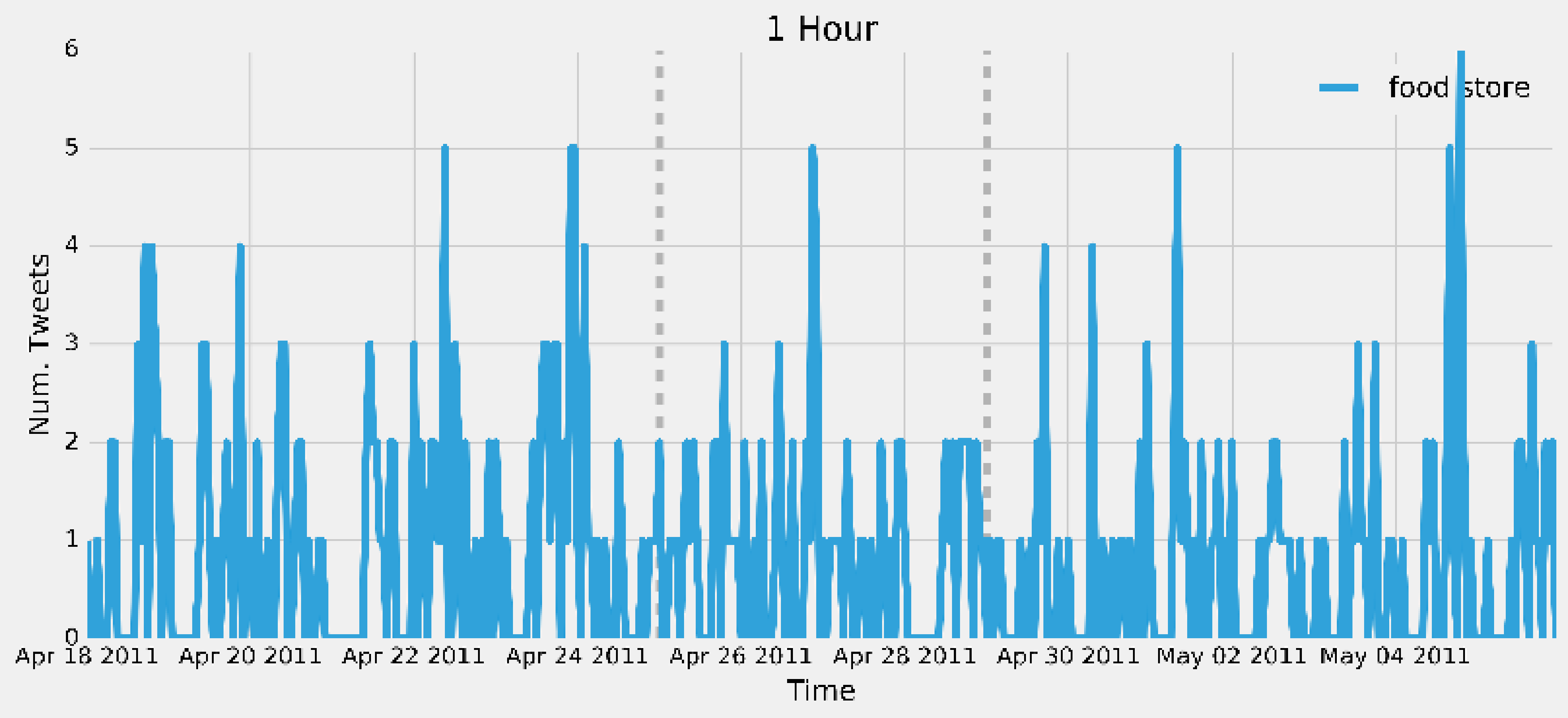
1 Hour
food store
Num. Tweets
0
1
2
3
4
5
6
Apr 18 2011
Apr 20 2011
Apr 22 2011
Apr 24 2011
Apr 26 2011
Apr 28 2011
Apr 30 2011
May 02 2011
May 04 2011
Time

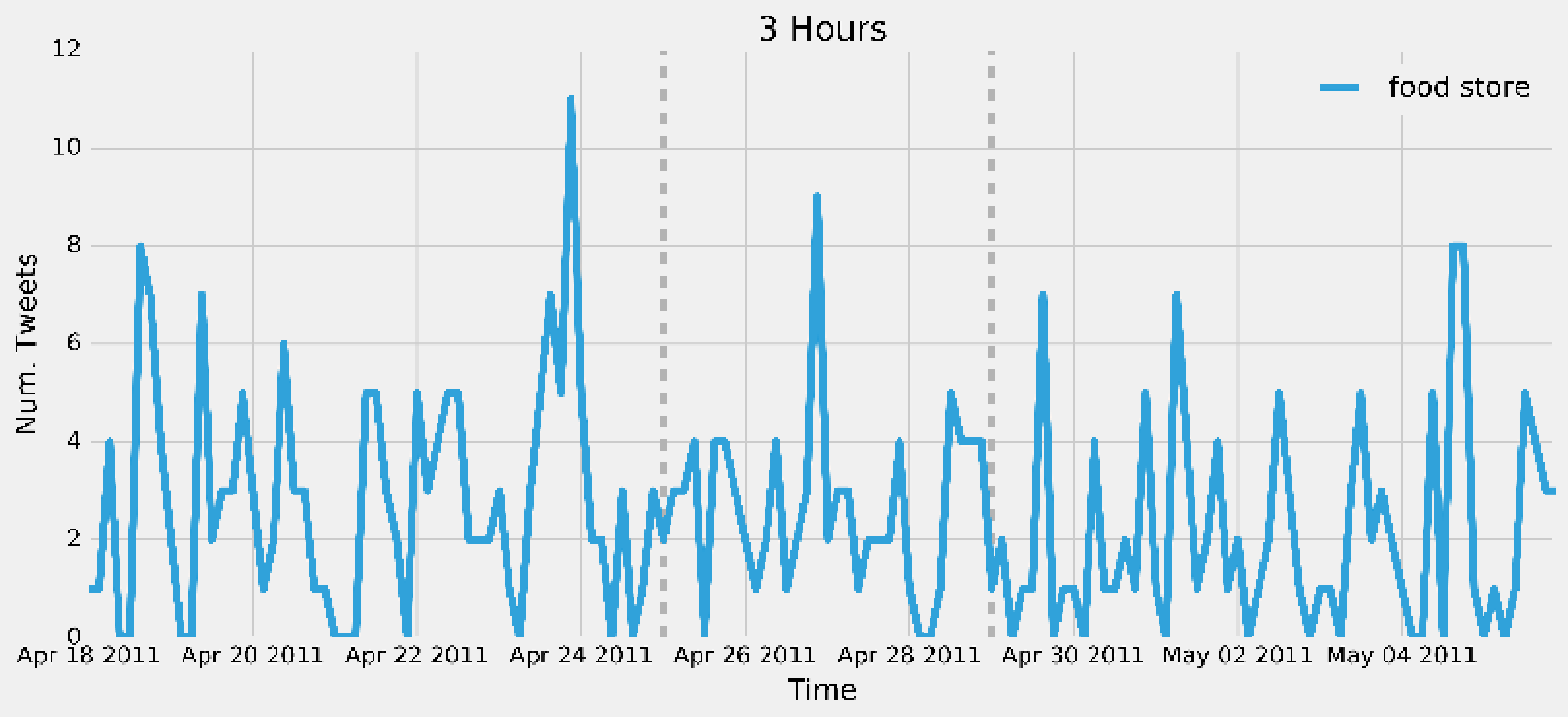
3 Hours
food store
Num. Tweets
0
2
4
6
8
10
12
Apr 18 2011
Apr 20 2011
Apr 22 2011
Apr 24 2011
Apr 26 2011
Apr 28 2011
Apr 30 2011
May 02 2011
May 04 2011
Time

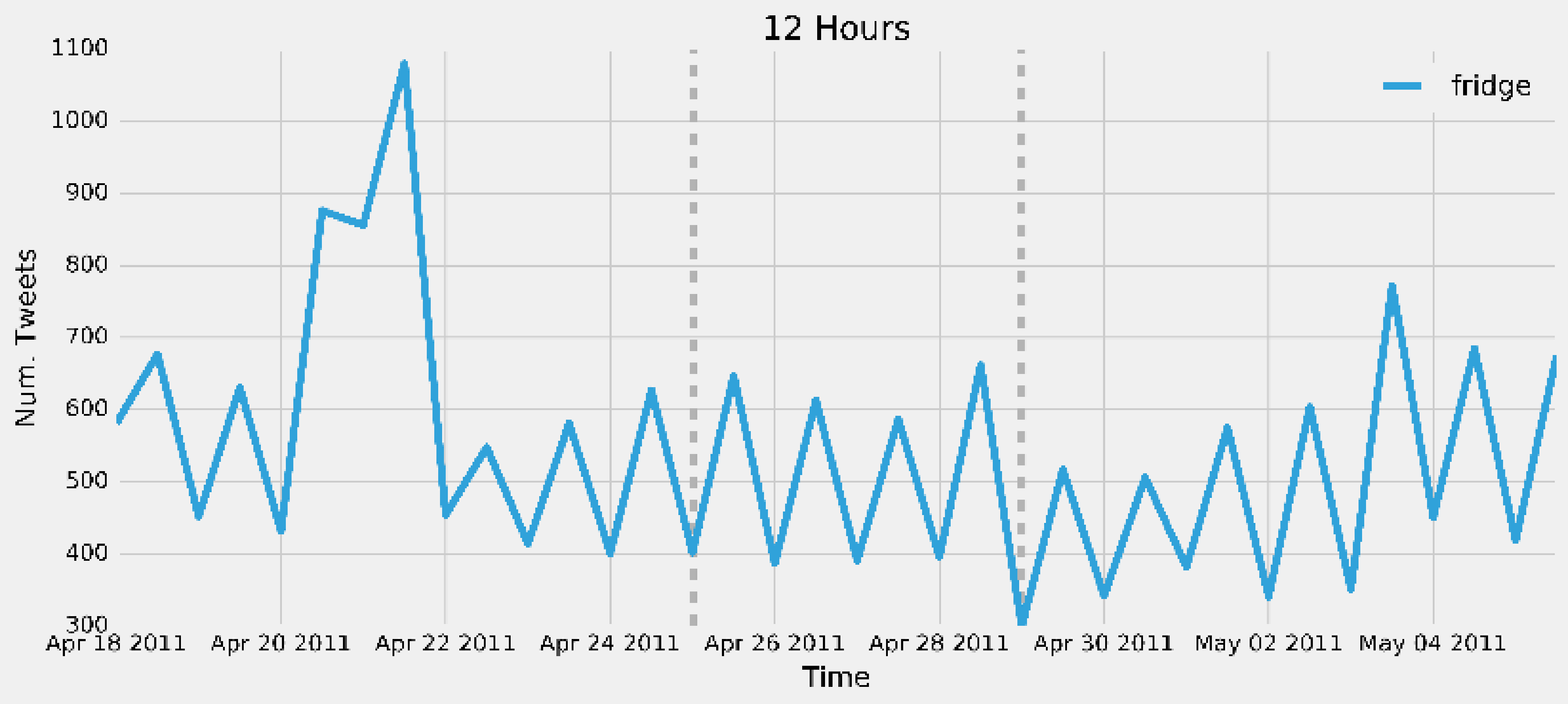
12 Hours
fridge
Num. Tweets
1100
1000
900
800
700
600
500
400
300
Apr 18 2011
Apr 20 2011
Apr 22 2011
Apr 24 2011
Apr 26 2011
Apr 28 2011
Apr 30 2011
May 02 2011
May 04 2011
Time

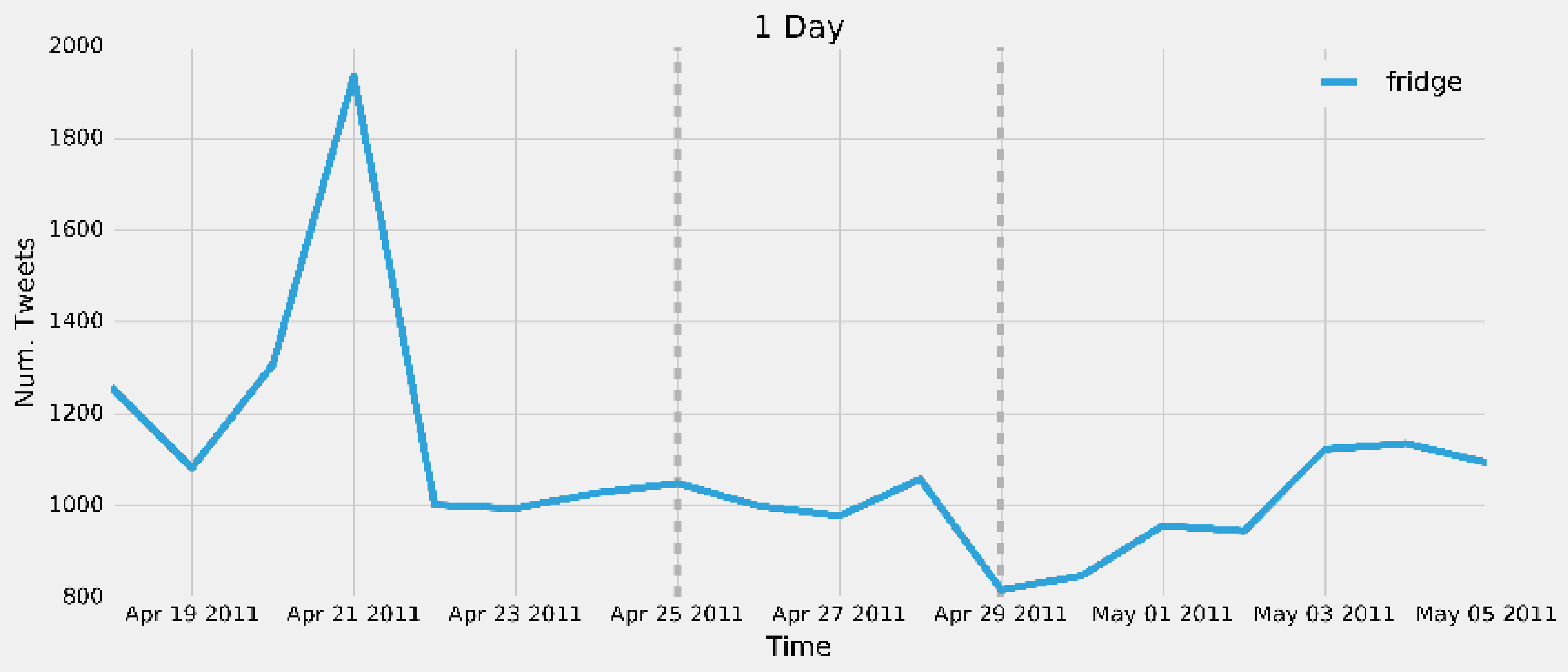

1 Day
fridge
Num. Tweets
2000
1800
1600
1400
1200
1000
800
Apr 19 2011
Apr 21 2011
Apr 23 2011
Apr 25 2011
Apr 27 2011
Apr 29 2011
May 01 2011
May 03 2011
May 05 2011
Time

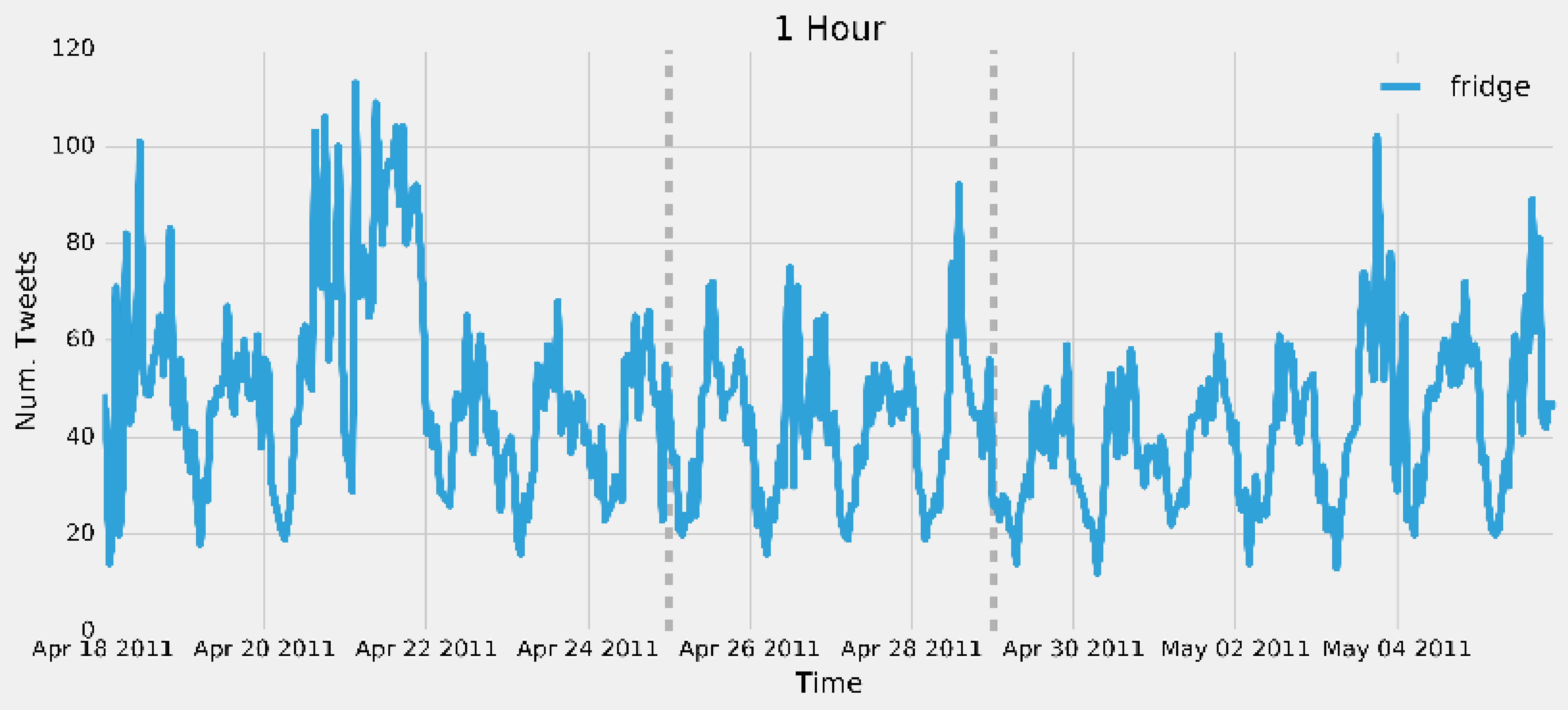

1 Hour
fridge
Num. Tweets
0
20
40
60
80
100
120
Apr 18 2011
Apr 20 2011
Apr 22 2011
Apr 24 2011
Apr 26 2011
Apr 28 2011
Apr 30 2011
May 02 2011
May 04 2011
Time

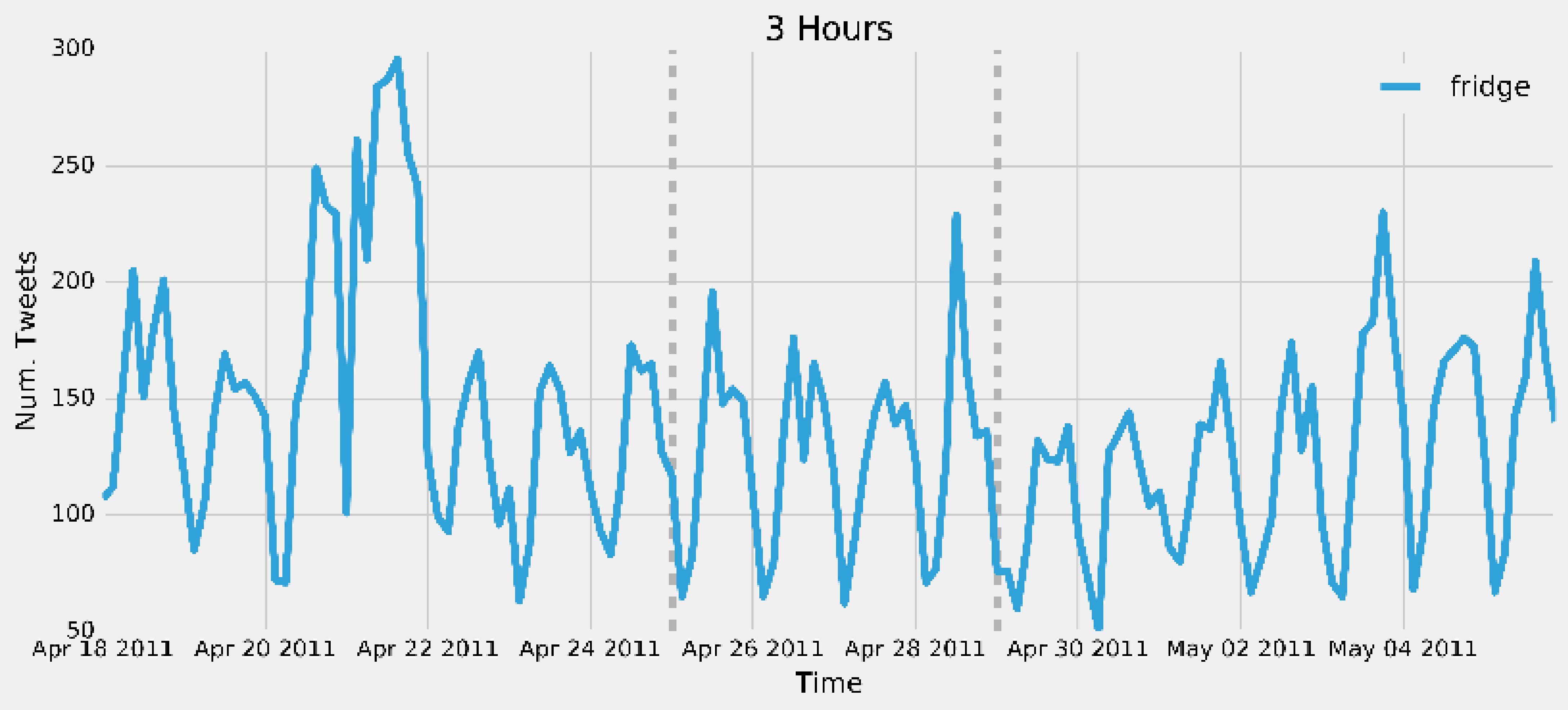
3 Hours
fridge
Num. Tweets
300
250
200
150
100
50
Apr 18 2011
Apr 20 2011
Apr 22 2011
Apr 24 2011
Apr 26 2011
Apr 28 2011
Apr 30 2011
May 02 2011
May 04 2011
Time

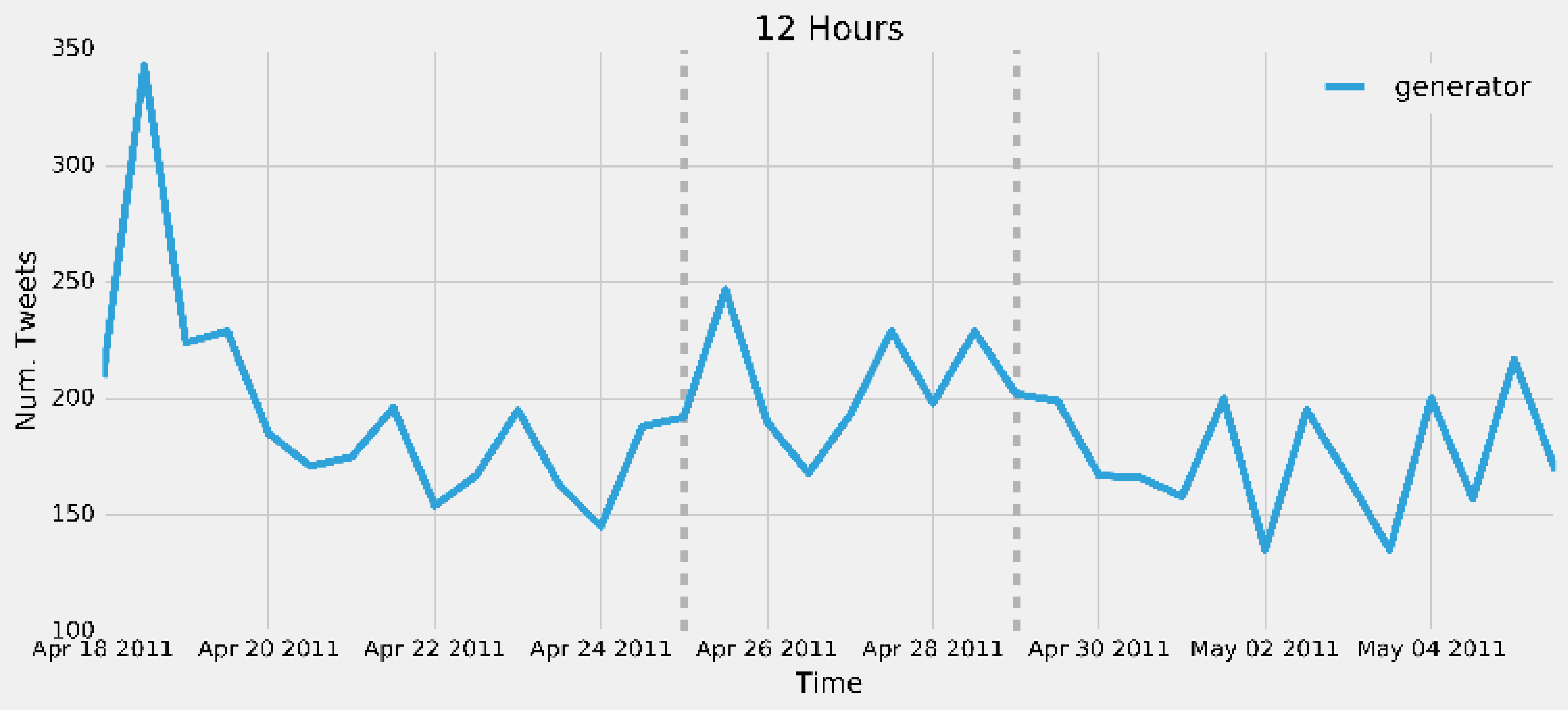

12 Hours
generator
Num. Tweets
350
300
250
200
150
100
Apr 18 2011
Apr 20 2011
Apr 22 2011
Apr 24 2011
Apr 26 2011
Apr 28 2011
Apr 30 2011
May 02 2011
May 04 2011
Time

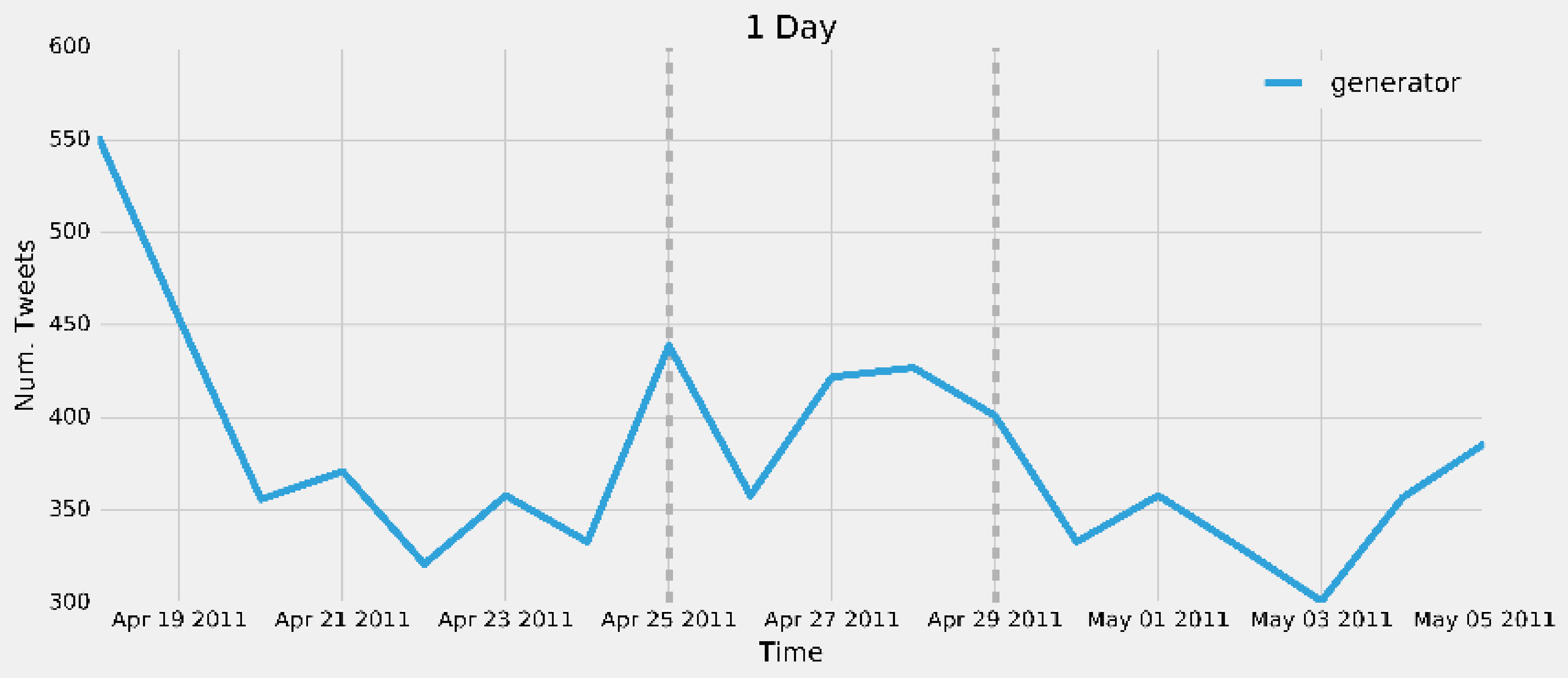
1 Day
generator
Num. Tweets
600
550
500
450
400
350
300
Apr 19 2011
Apr 21 2011
Apr 23 2011
Apr 25 2011
Apr 27 2011
Apr 29 2011
May 01 2011
May 03 2011
May 05 2011
Time

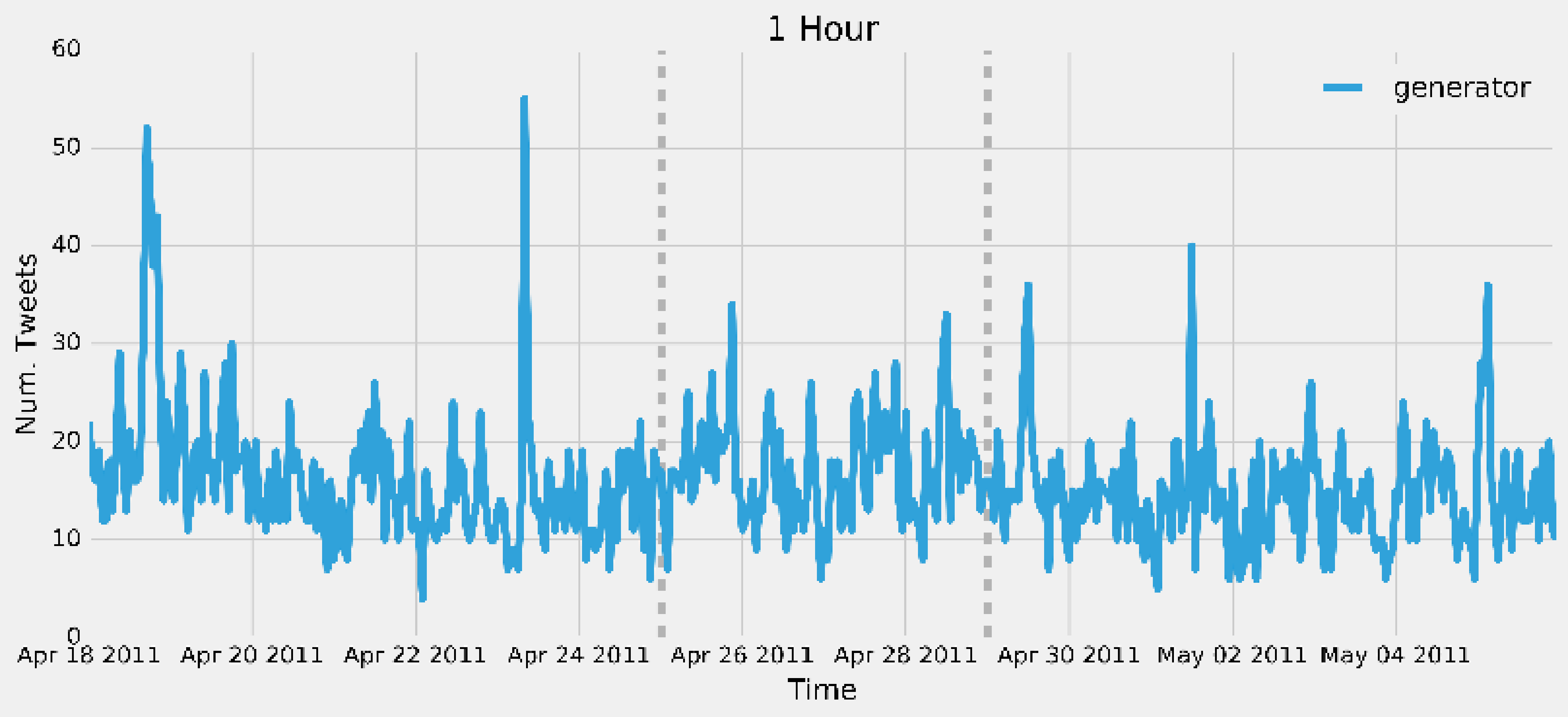
1 Hour
generator
Num. Tweets
0
10
20
30
40
50
60
Apr 18 2011
Apr 20 2011
Apr 22 2011
Apr 24 2011
Apr 26 2011
Apr 28 2011
Apr 30 2011
May 02 2011
May 04 2011
Time

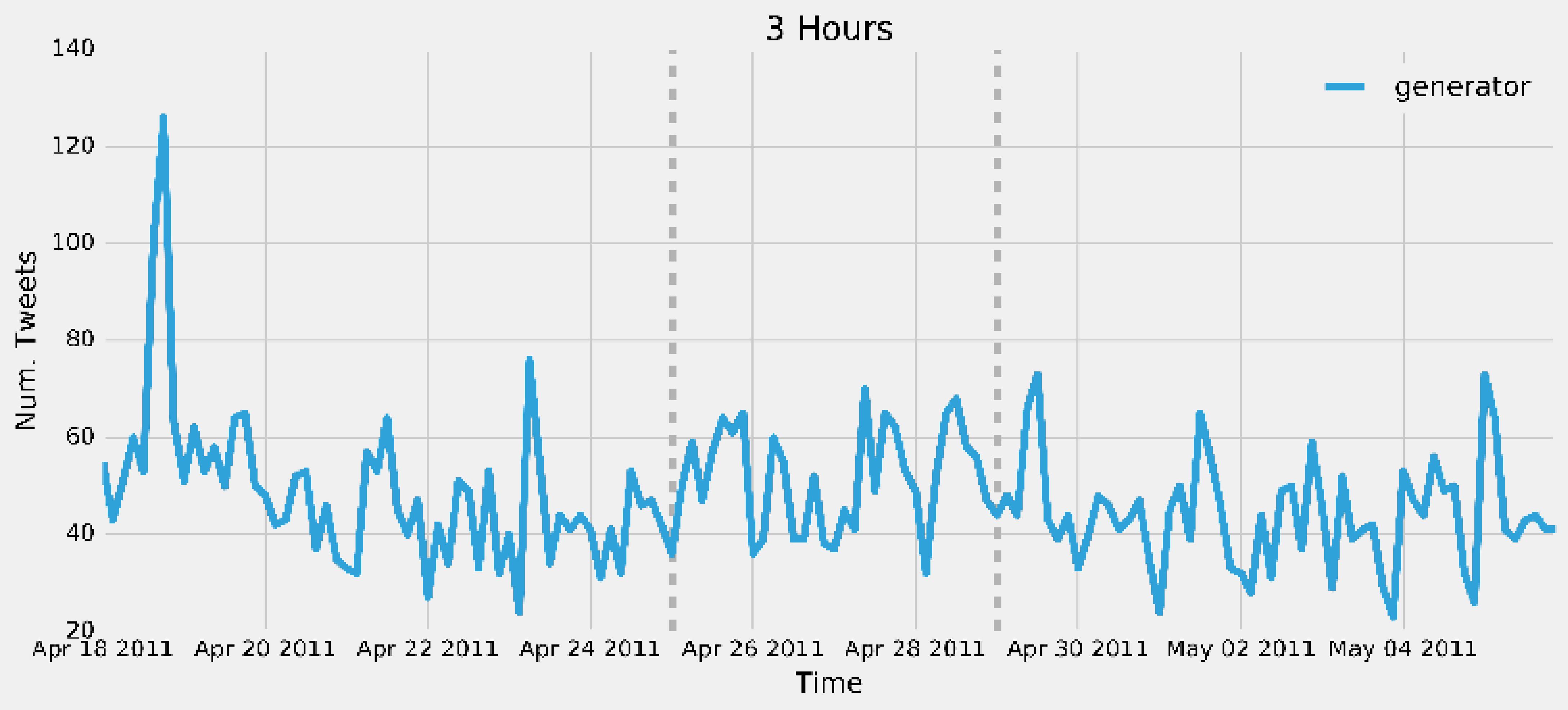
3 Hours
140
120
100
80
60
40
20
Num. Tweets
generator
Apr 18 2011
Apr 20 2011
Apr 22 2011
Apr 24 2011
Apr 26 2011
Apr 28 2011
Apr 30 2011
May 02 2011
May 04 2011
Time

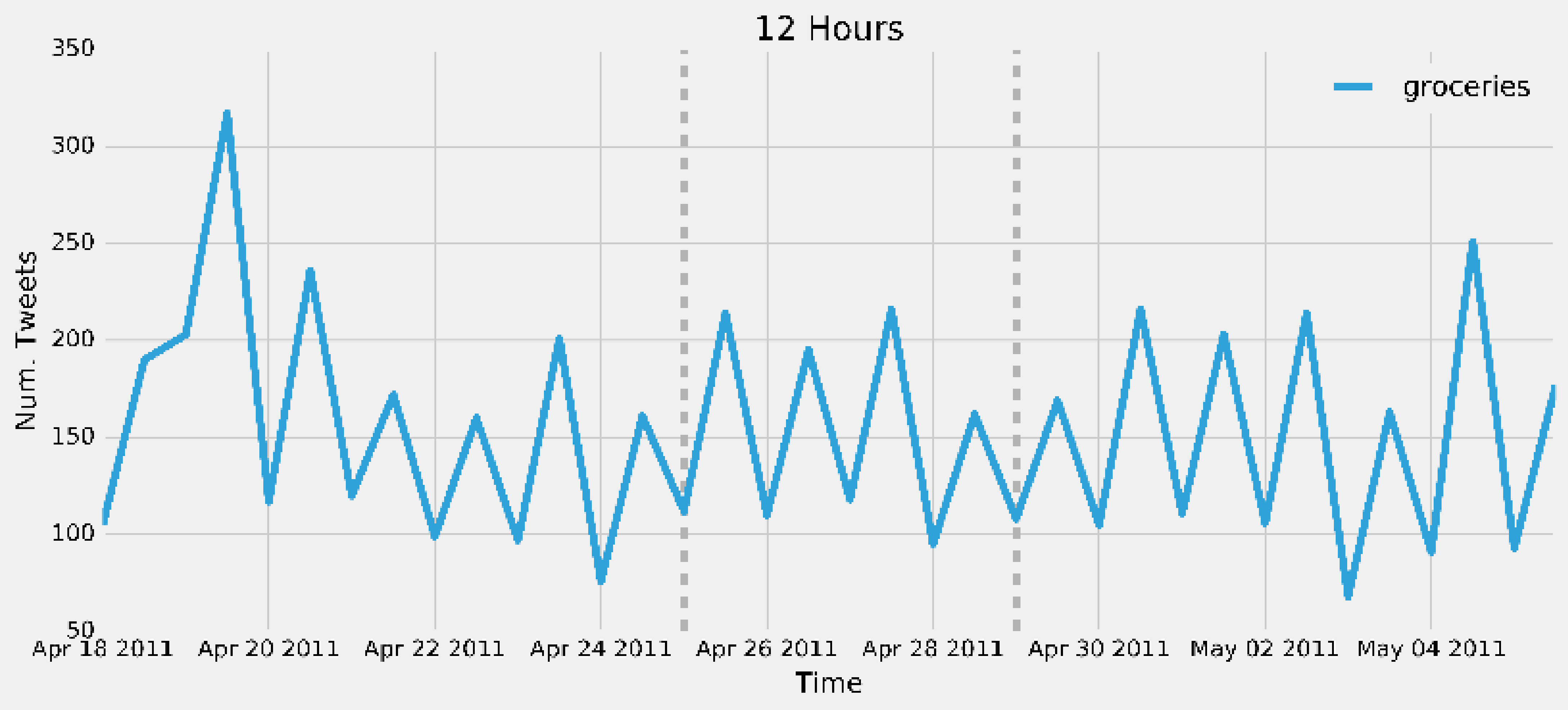
12 Hours
groceries
Num. Tweets
350
300
250
200
150
100
50
Apr 18 2011
Apr 20 2011
Apr 22 2011
Apr 24 2011
Apr 26 2011
Apr 28 2011
Apr 30 2011
May 02 2011
May 04 2011
Time

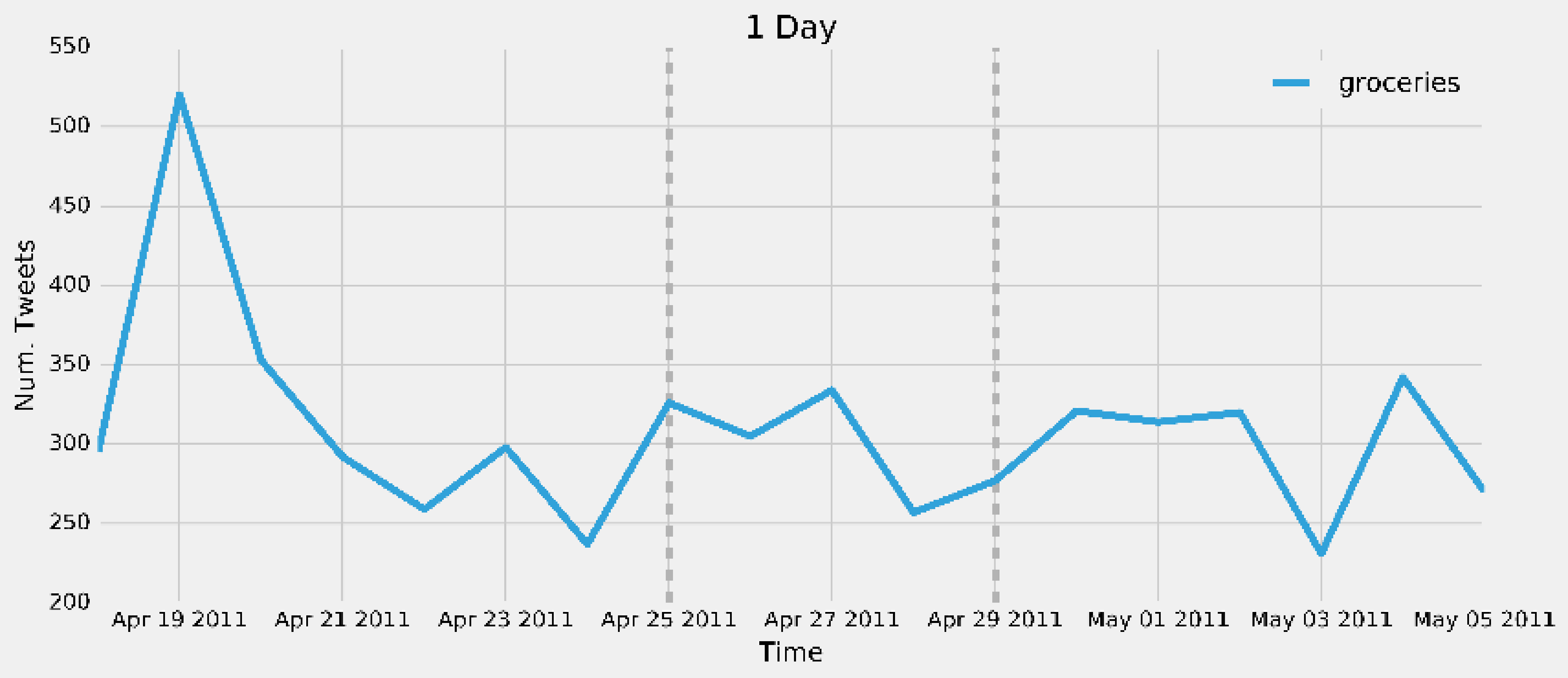

1 Day
groceries
Num. Tweets
Time
550
500
450
400
350
300
250
200
Apr 19 2011
Apr 21 2011
Apr 23 2011
Apr 25 2011
Apr 27 2011
Apr 29 2011
May 01 2011
May 03 2011
May 05 2011

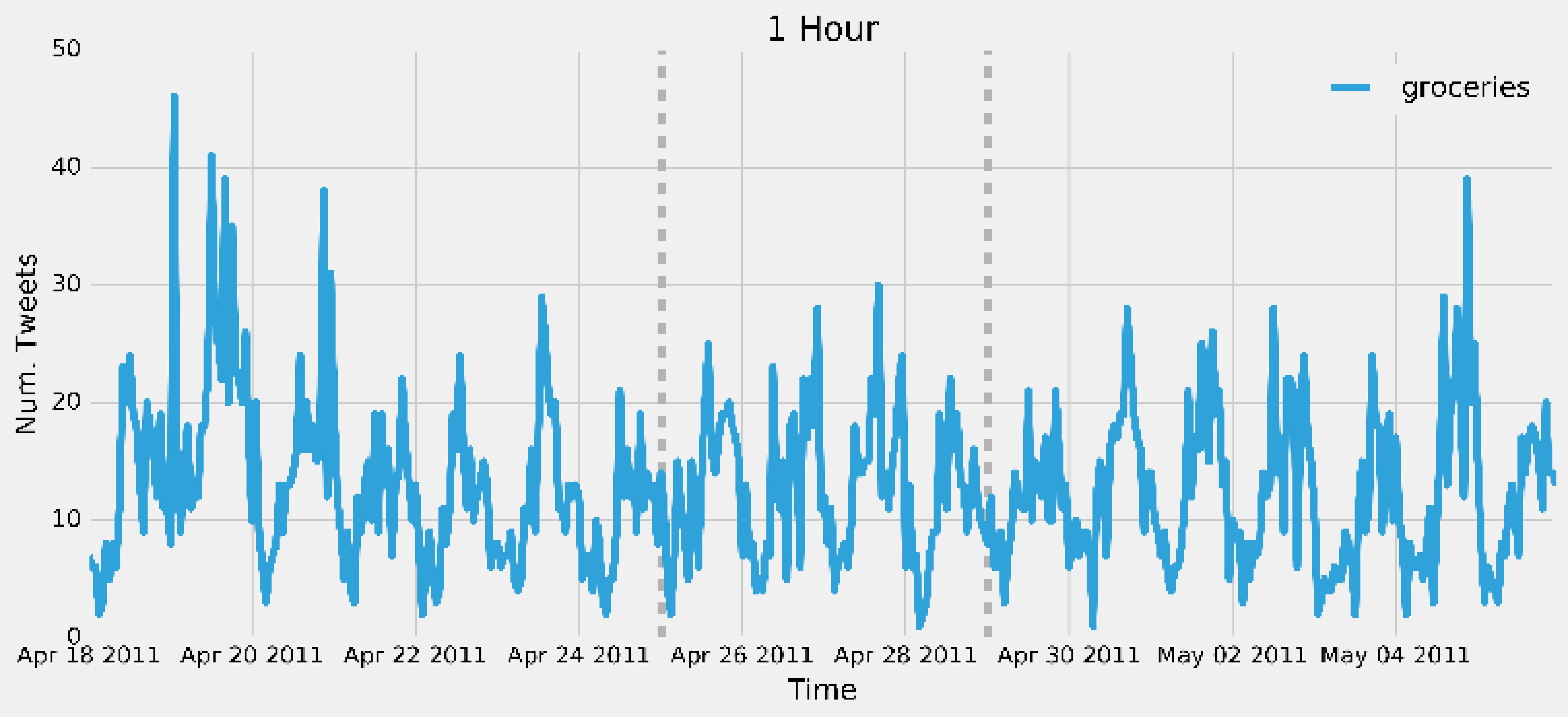
1 Hour
groceries
Num. Tweets
0
10
20
30
40
50
Apr 18 2011
Apr 20 2011
Apr 22 2011
Apr 24 2011
Apr 26 2011
Apr 28 2011
Apr 30 2011
May 02 2011
May 04 2011
Time

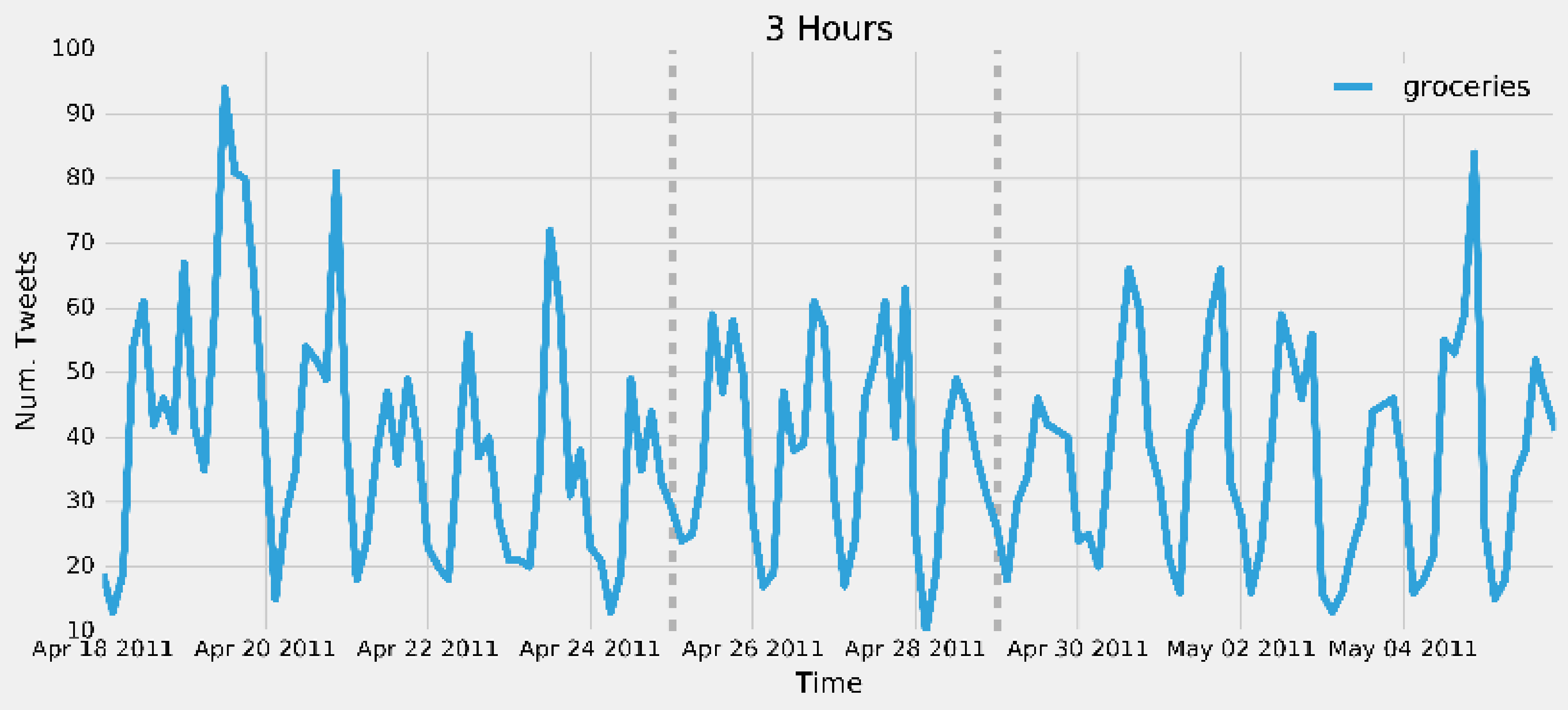
3 Hours
groceries
100
90
80
70
60
50
40
30
20
10
Num. Tweets
Apr 18 2011
Apr 20 2011
Apr 22 2011
Apr 24 2011
Apr 26 2011
Apr 28 2011
Apr 30 2011
May 02 2011
May 04 2011
Time

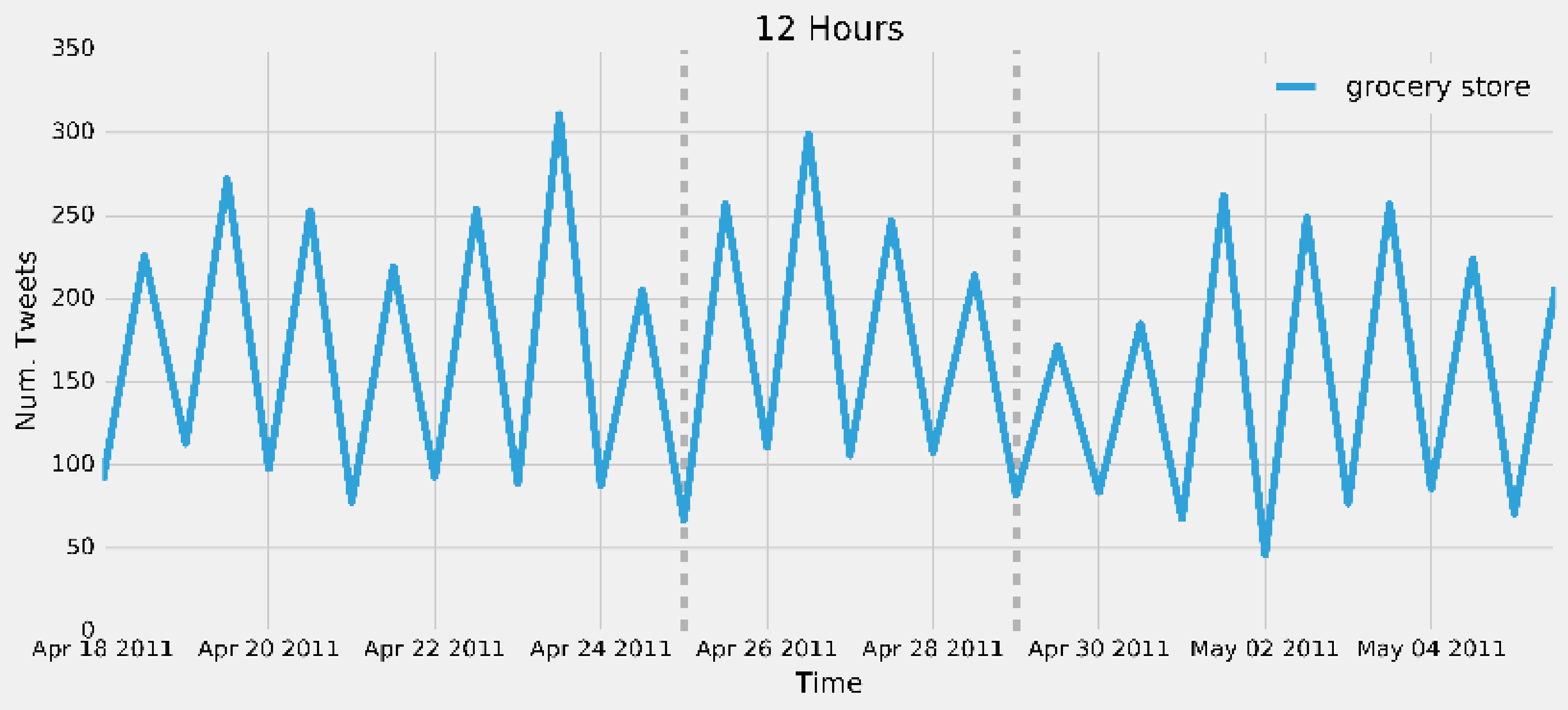

12 Hours
grocery store
Num. Tweets
0
50
100
150
200
250
300
350
Apr 18 2011
Apr 20 2011
Apr 22 2011
Apr 24 2011
Apr 26 2011
Apr 28 2011
Apr 30 2011
May 02 2011
May 04 2011
Time

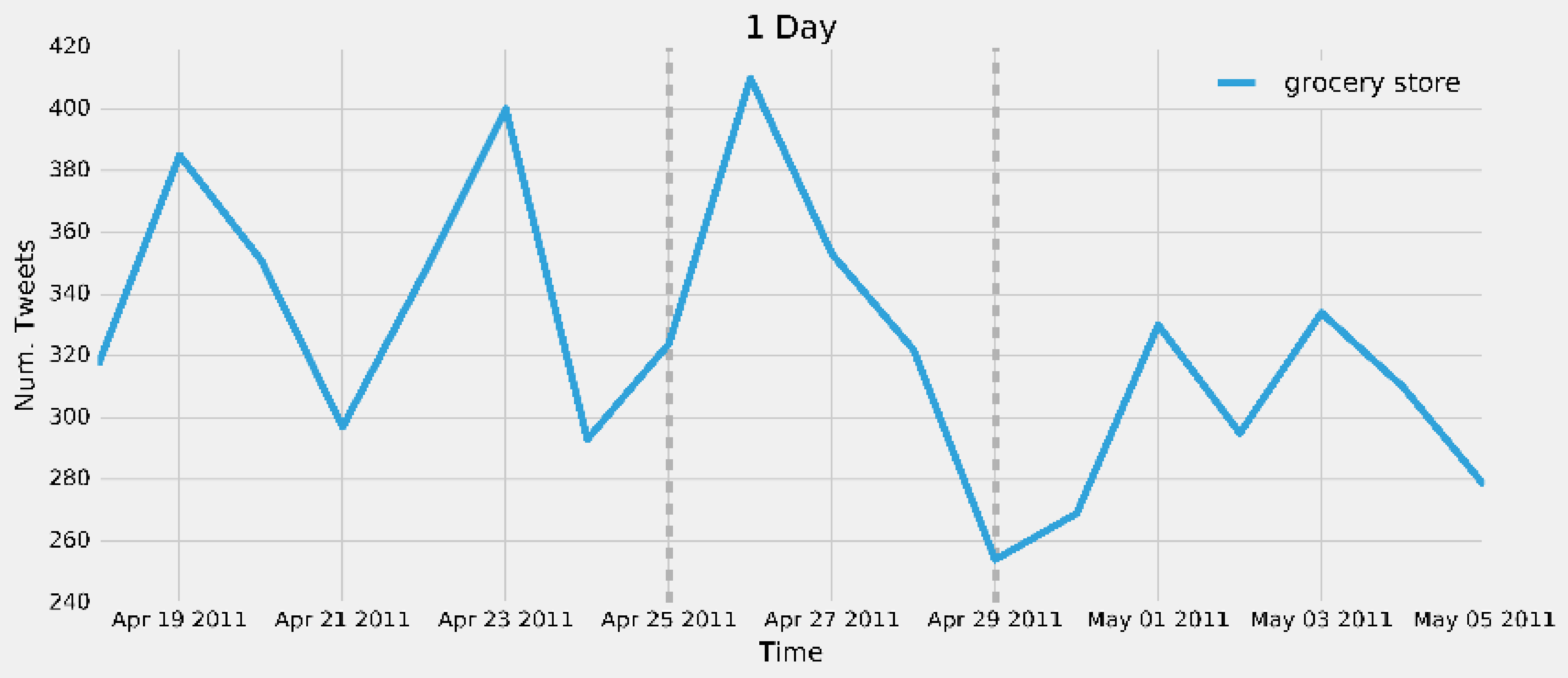
1 Day
grocery store
Num. Tweets
Time
420
400
380
360
340
320
300
280
260
240
Apr 19 2011
Apr 21 2011
Apr 23 2011
Apr 25 2011
Apr 27 2011
Apr 29 2011
May 01 2011
May 03 2011
May 05 2011

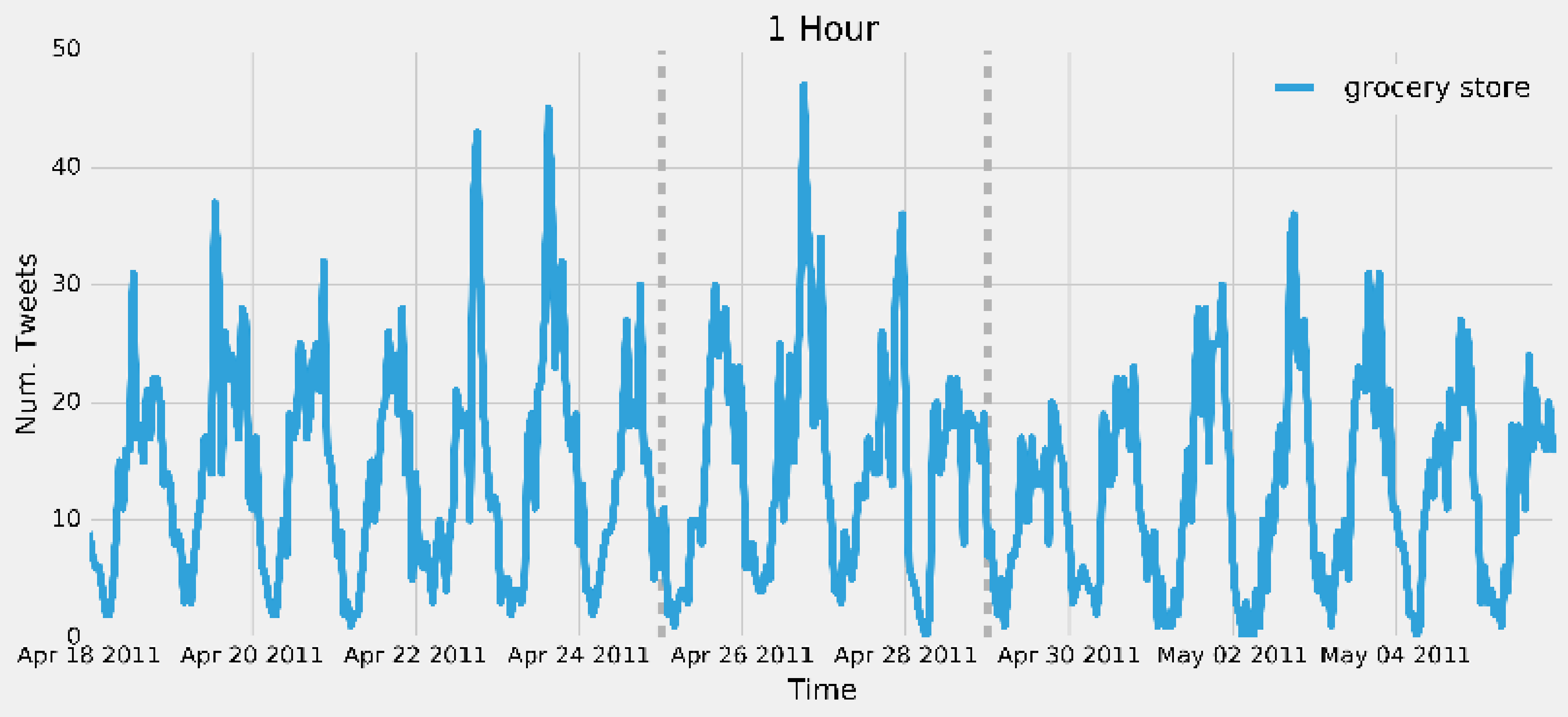
1 Hour
grocery store
Num. Tweets
0
10
20
30
40
50
Apr 18 2011
Apr 20 2011
Apr 22 2011
Apr 24 2011
Apr 26 2011
Apr 28 2011
Apr 30 2011
May 02 2011
May 04 2011
Time

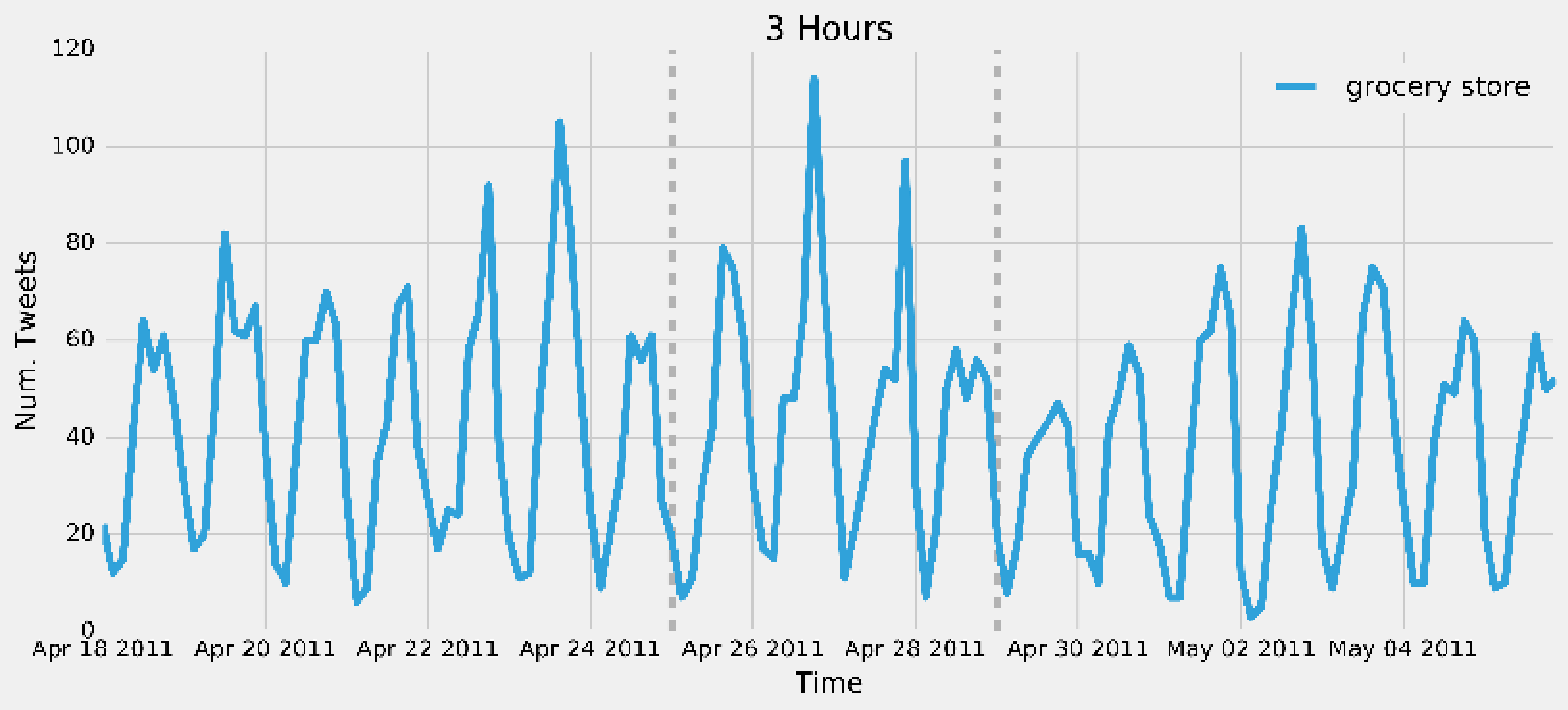
3 Hours
grocery store
Num. Tweets
0
20
40
60
80
100
120
Apr 18 2011
Apr 20 2011
Apr 22 2011
Apr 24 2011
Apr 26 2011
Apr 28 2011
Apr 30 2011
May 02 2011
May 04 2011
Time

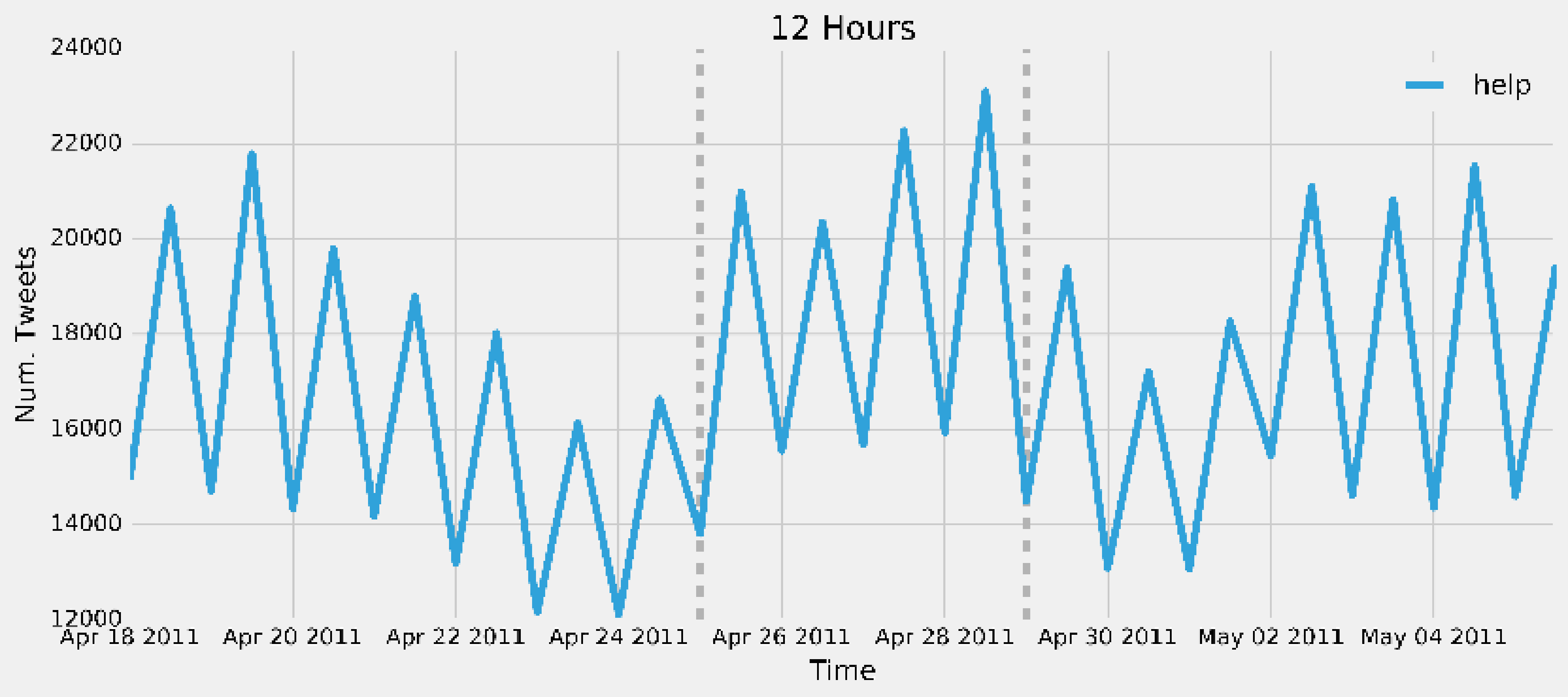
12 Hours
help
Num. Tweets
24000
22000
20000
18000
16000
14000
12000
Apr 18 2011
Apr 20 2011
Apr 22 2011
Apr 24 2011
Apr 26 2011
Apr 28 2011
Apr 30 2011
May 02 2011
May 04 2011
Time

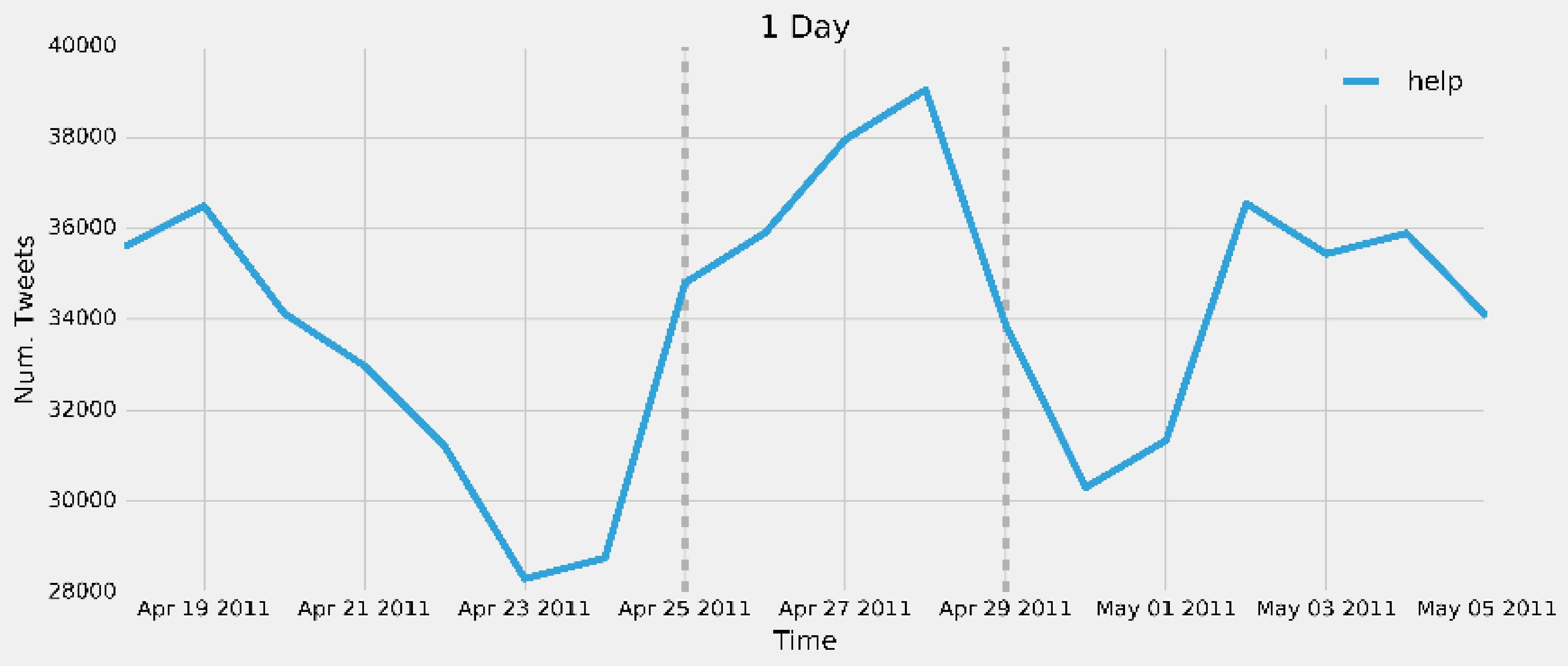

1 Day
help
Num. Tweets
40000
38000
36000
34000
32000
30000
28000
Apr 19 2011
Apr 21 2011
Apr 23 2011
Apr 25 2011
Apr 27 2011
Apr 29 2011
May 01 2011
May 03 2011
May 05 2011
Time

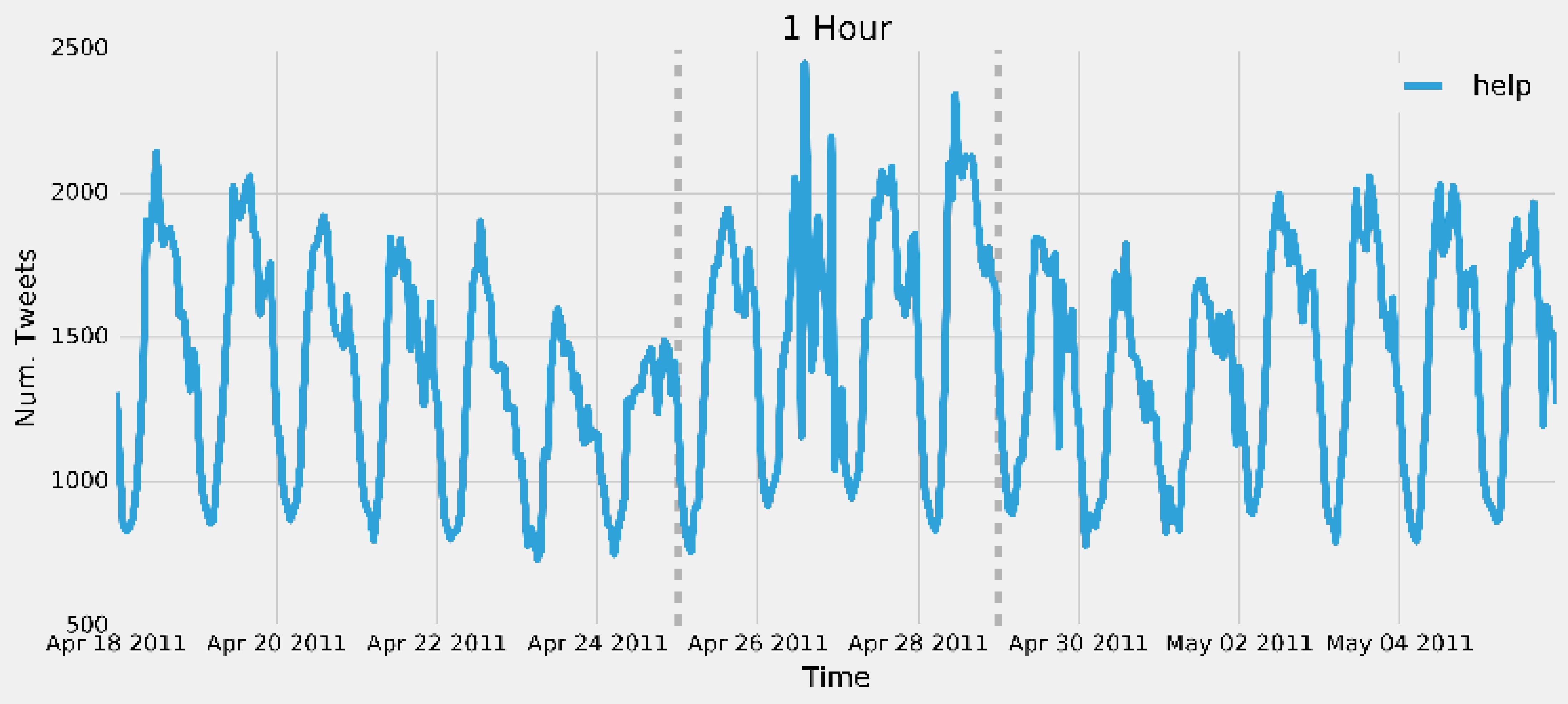

1 Hour
help
Num. Tweets
2500
2000
1500
1000
500
Apr 18 2011
Apr 20 2011
Apr 22 2011
Apr 24 2011
Apr 26 2011
Apr 28 2011
Apr 30 2011
May 02 2011
May 04 2011
Time

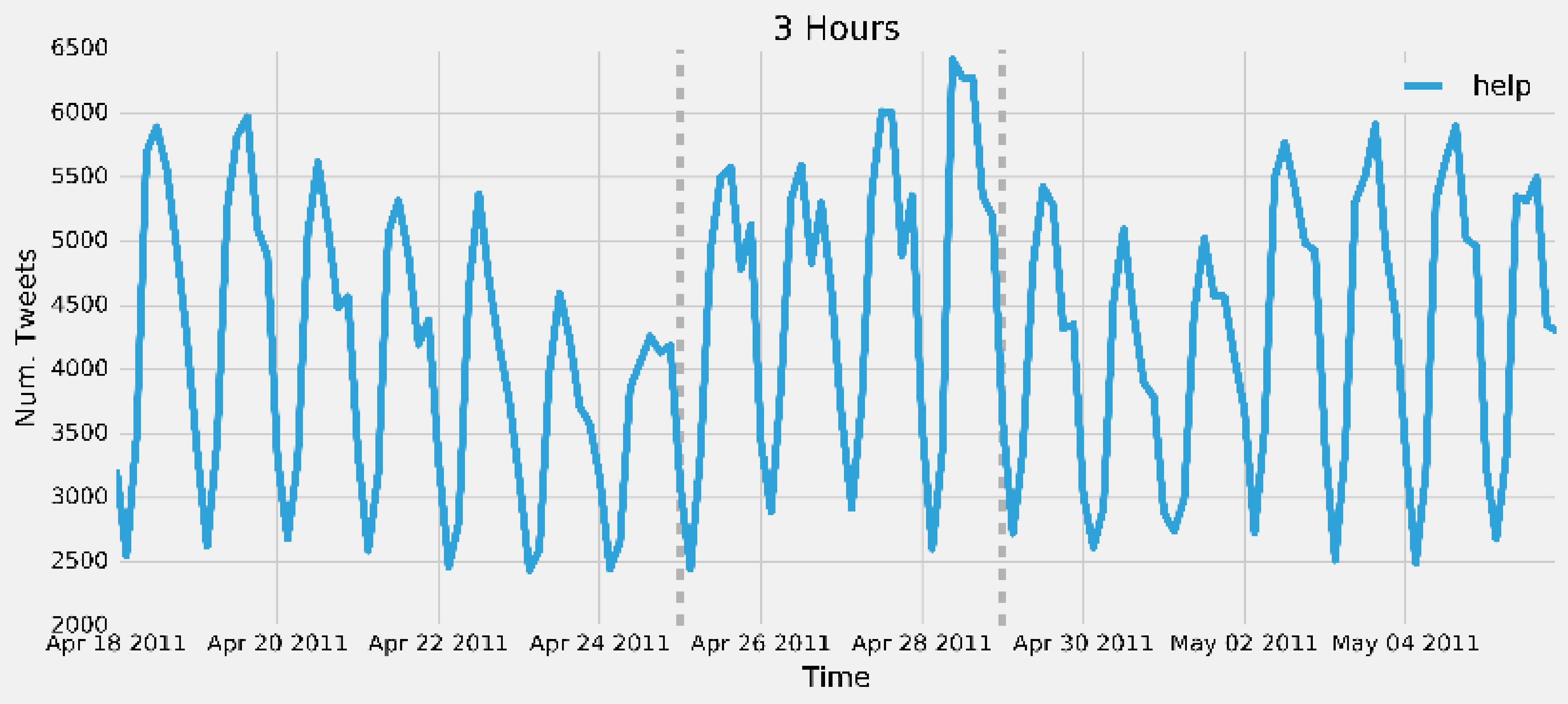

3 Hours
help
Num. Tweets
6500
6000
5500
5000
4500
4000
3500
3000
2500
2000
Apr 18 2011
Apr 20 2011
Apr 22 2011
Apr 24 2011
Apr 26 2011
Apr 28 2011
Apr 30 2011
May 02 2011
May 04 2011
Time

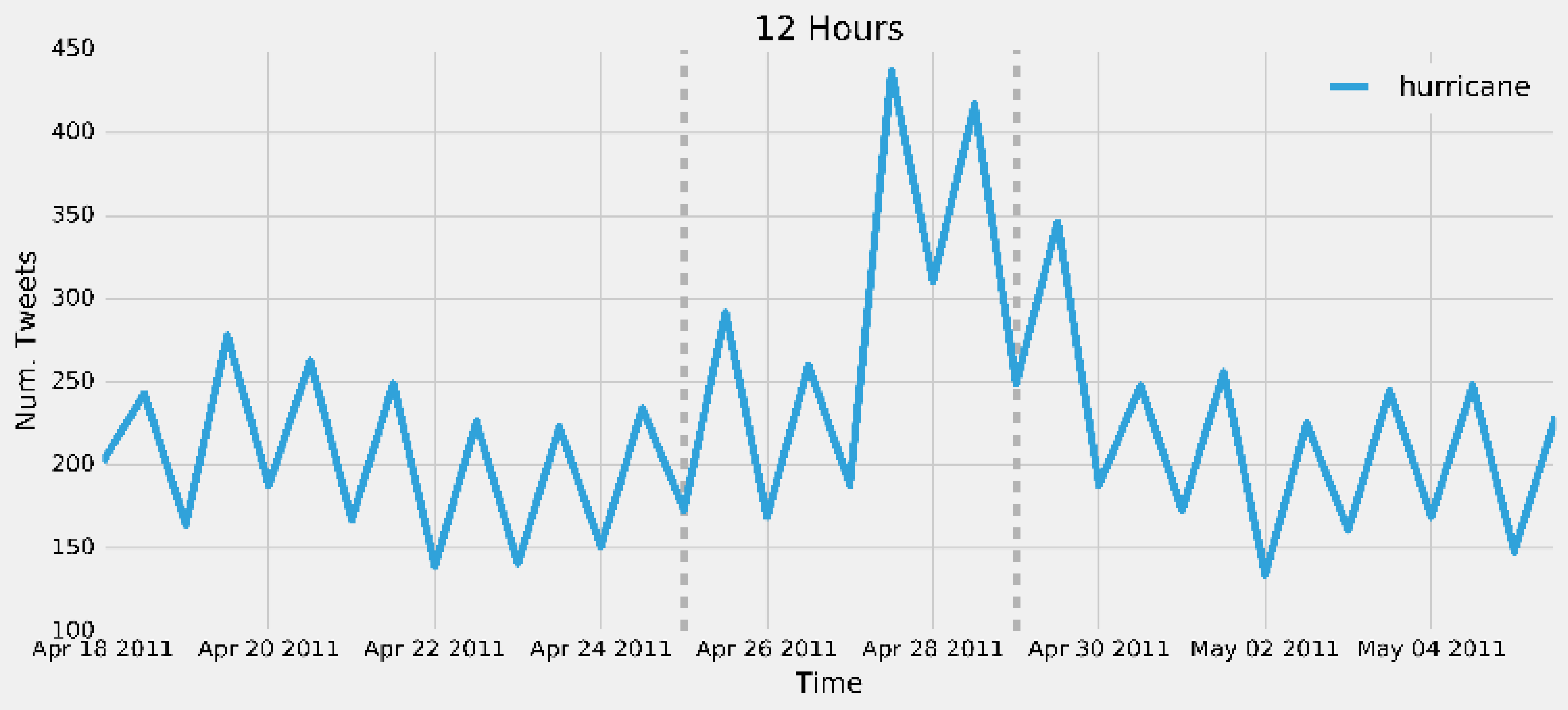
12 Hours
450
400
350
300
250
200
150
100
Num. Tweets
hurricane
Apr 18 2011
Apr 20 2011
Apr 22 2011
Apr 24 2011
Apr 26 2011
Apr 28 2011
Apr 30 2011
May 02 2011
May 04 2011
Time

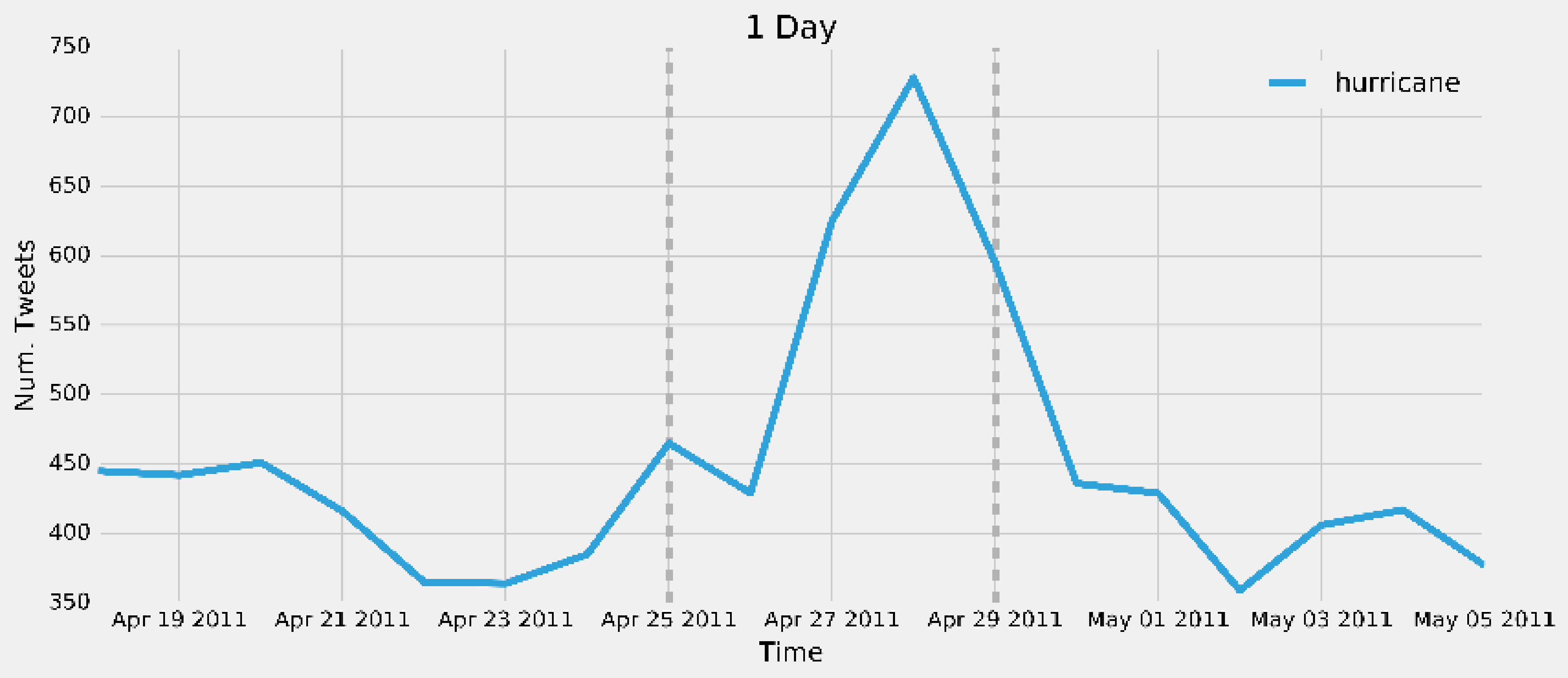

1 Day
hurricane
Num. Tweets
Time
750
700
650
600
550
500
450
400
350
Apr 19 2011
Apr 21 2011
Apr 23 2011
Apr 25 2011
Apr 27 2011
Apr 29 2011
May 01 2011
May 03 2011
May 05 2011

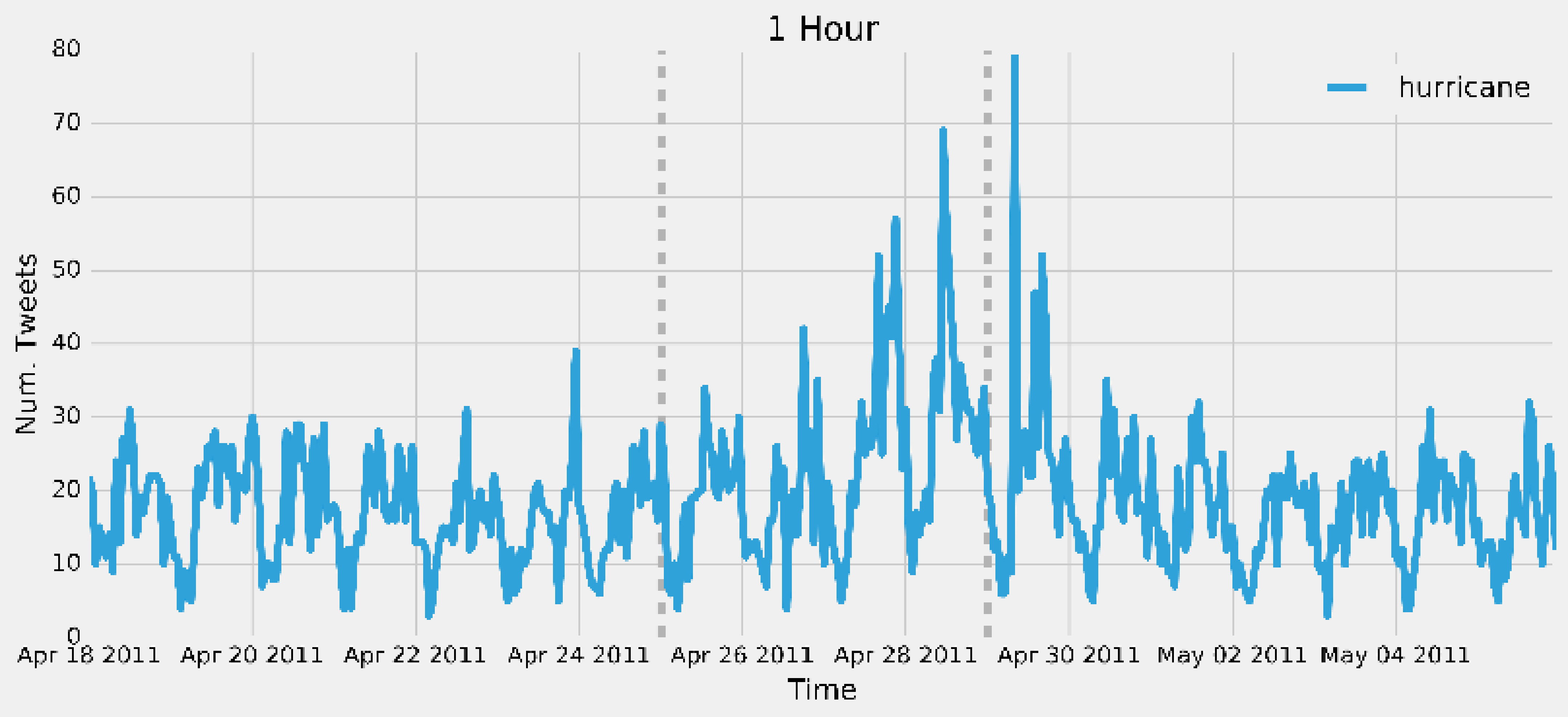

1 Hour
hurricane
Num. Tweets
0
10
20
30
40
50
60
70
80
Apr 18 2011
Apr 20 2011
Apr 22 2011
Apr 24 2011
Apr 26 2011
Apr 28 2011
Apr 30 2011
May 02 2011
May 04 2011
Time

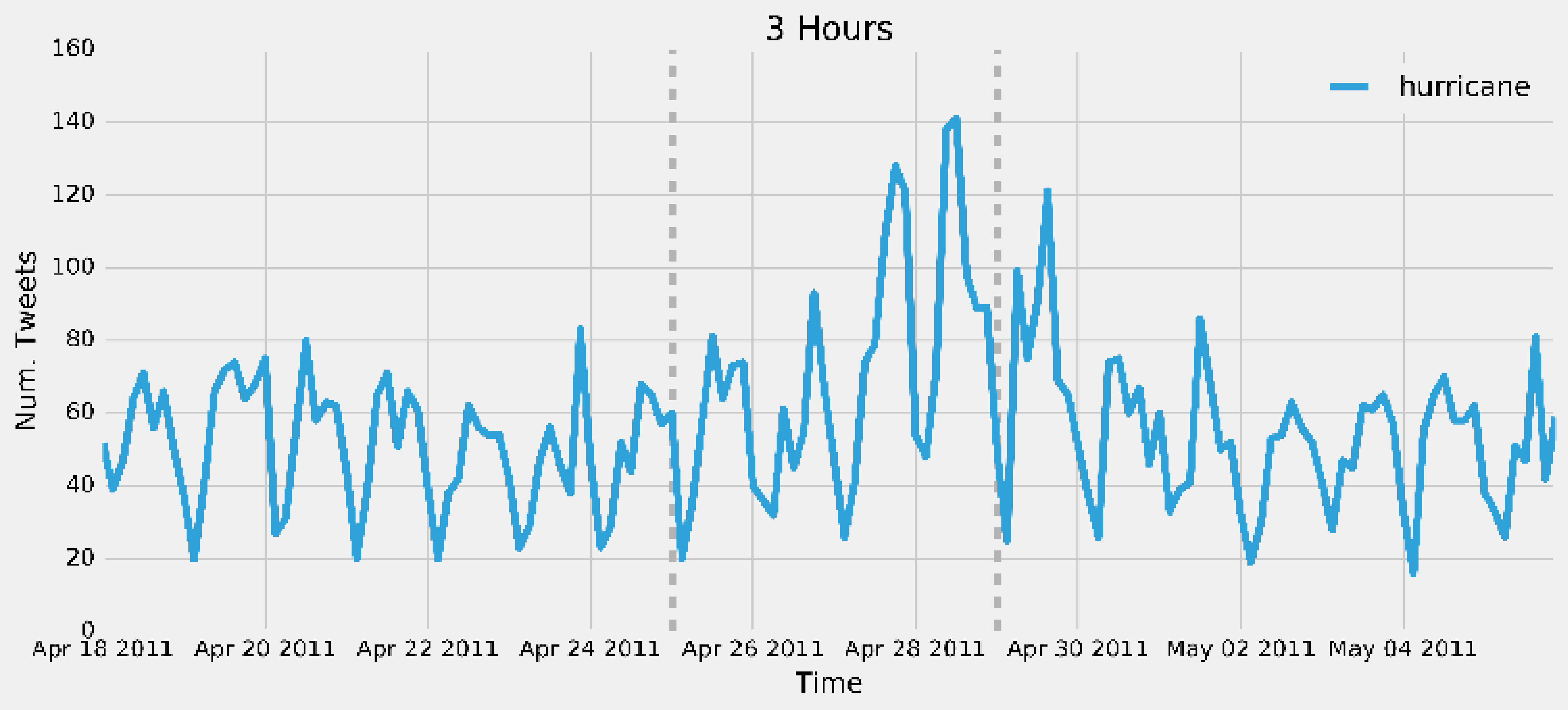

3 Hours
hurricane
Num. Tweets
0
20
40
60
80
100
120
140
160
Apr 18 2011
Apr 20 2011
Apr 22 2011
Apr 24 2011
Apr 26 2011
Apr 28 2011
Apr 30 2011
May 02 2011
May 04 2011
Time

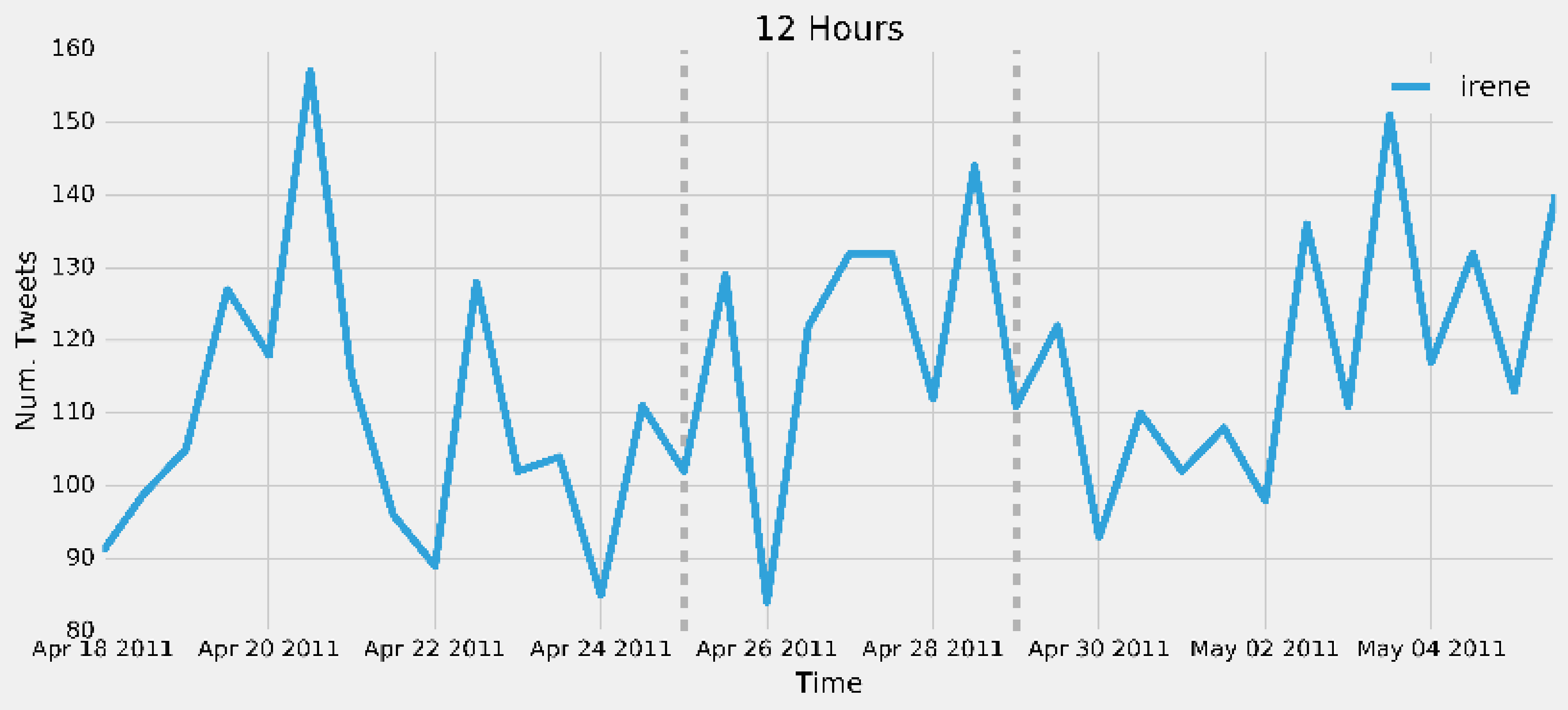

12 Hours
160
150
140
130
120
110
100
90
80
Num. Tweets
irene
Apr 18 2011
Apr 20 2011
Apr 22 2011
Apr 24 2011
Apr 26 2011
Apr 28 2011
Apr 30 2011
May 02 2011
May 04 2011
Time

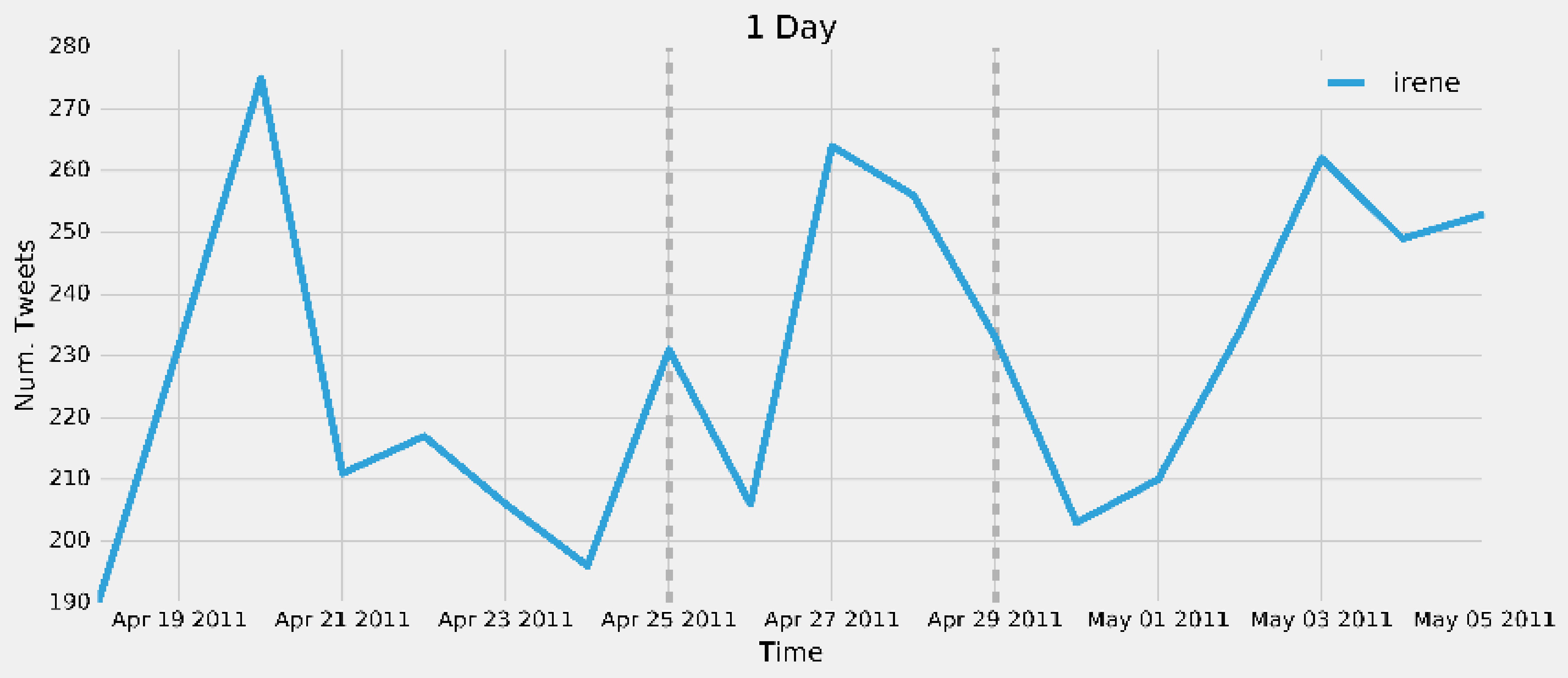
1 Day
irene
Num. Tweets
280
270
260
250
240
230
220
210
200
190
Apr 19 2011
Apr 21 2011
Apr 23 2011
Apr 25 2011
Apr 27 2011
Apr 29 2011
May 01 2011
May 03 2011
May 05 2011
Time

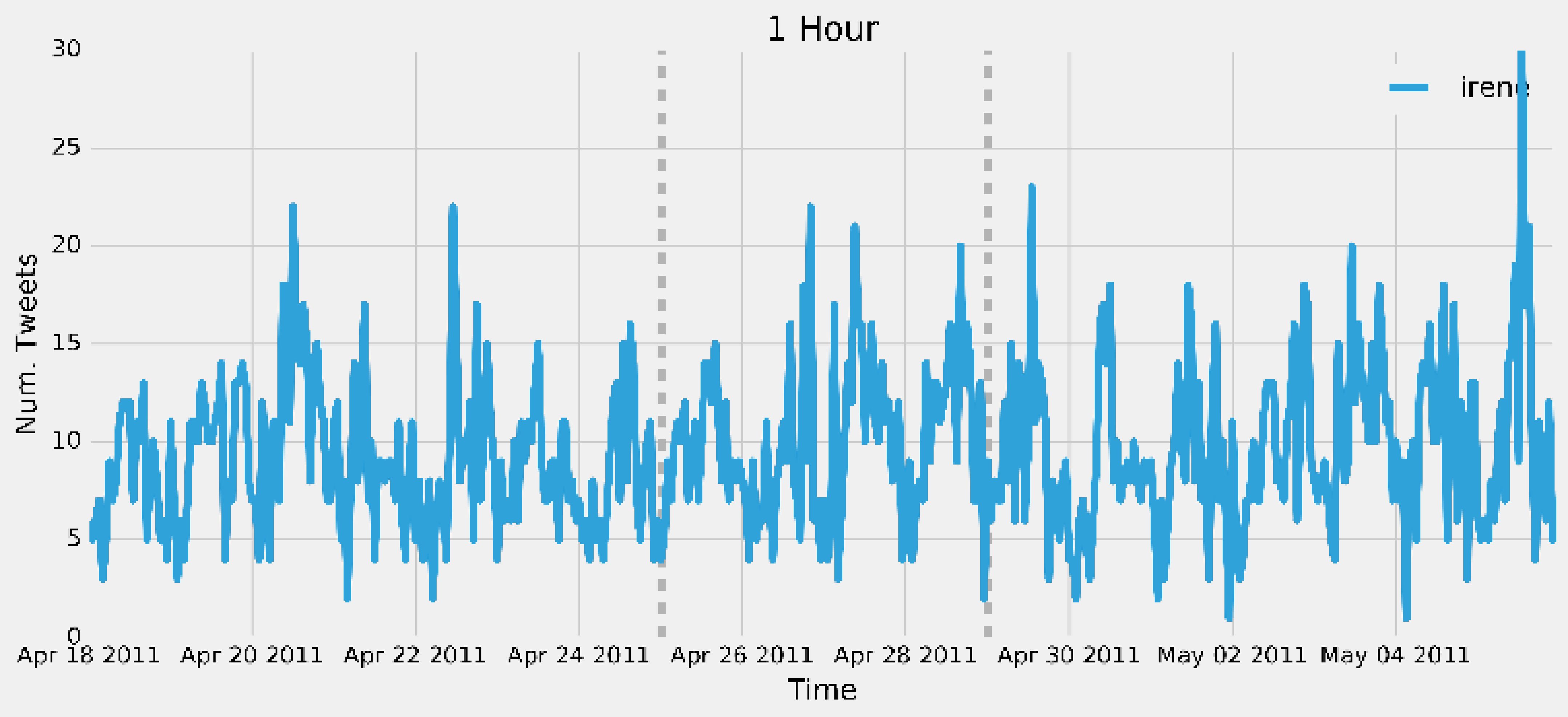
1 Hour
irene
Num. Tweets
Time
0
5
10
15
20
25
30
Apr 18 2011
Apr 20 2011
Apr 22 2011
Apr 24 2011
Apr 26 2011
Apr 28 2011
Apr 30 2011
May 02 2011
May 04 2011

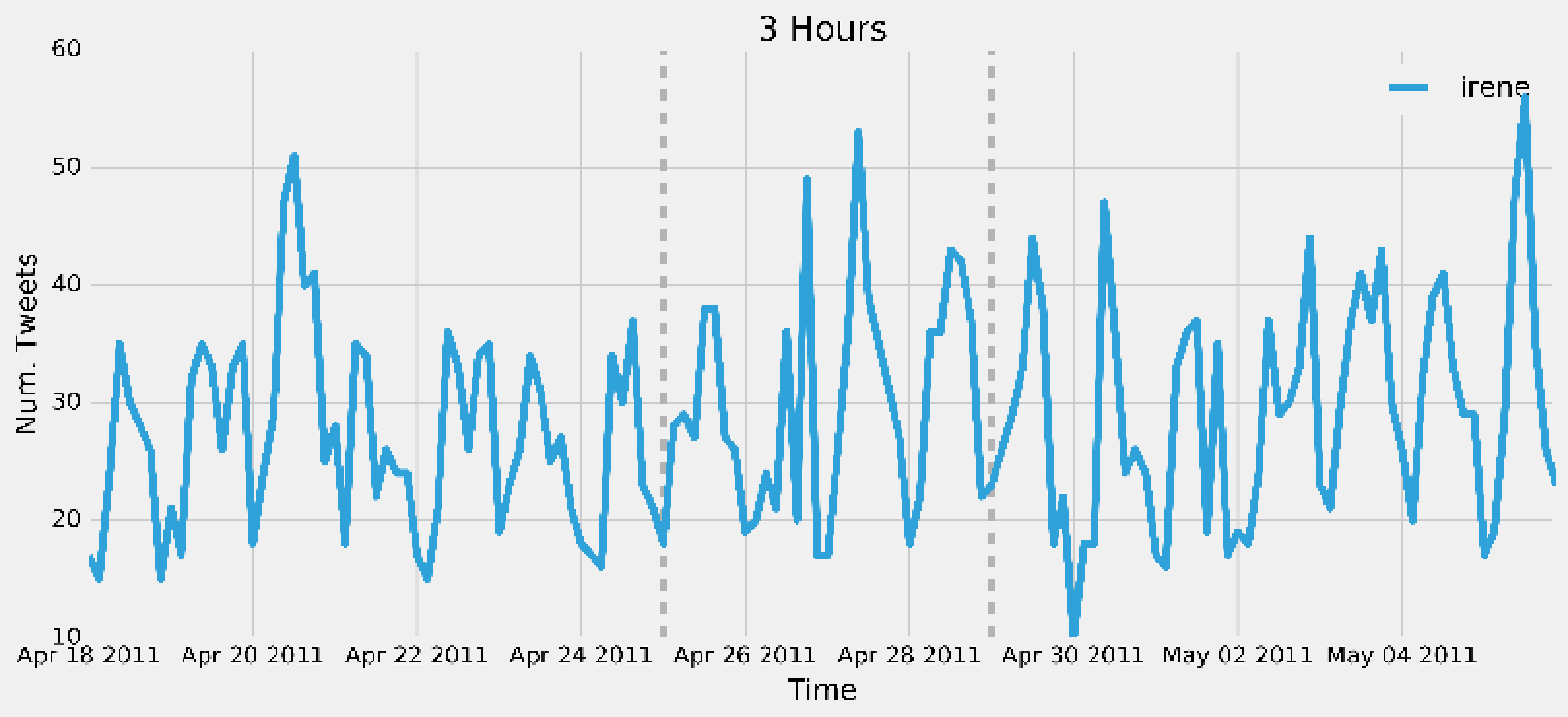
3 Hours
irene
Num. Tweets
60
50
40
30
20
10
Apr 18 2011
Apr 20 2011
Apr 22 2011
Apr 24 2011
Apr 26 2011
Apr 28 2011
Apr 30 2011
May 02 2011
May 04 2011
Time

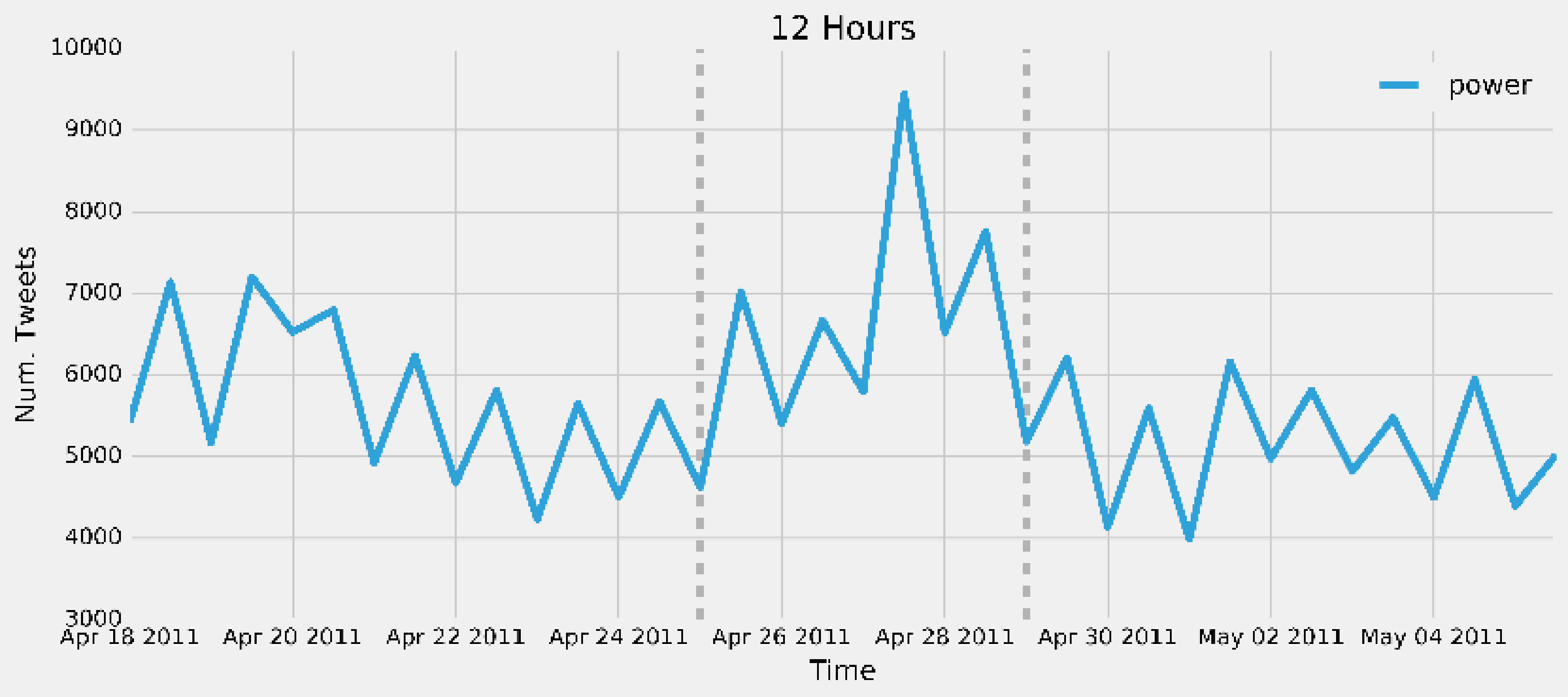
12 Hours
power
Num. Tweets
Time
10000
9000
8000
7000
6000
5000
4000
3000
Apr 18 2011
Apr 20 2011
Apr 22 2011
Apr 24 2011
Apr 26 2011
Apr 28 2011
Apr 30 2011
May 02 2011
May 04 2011

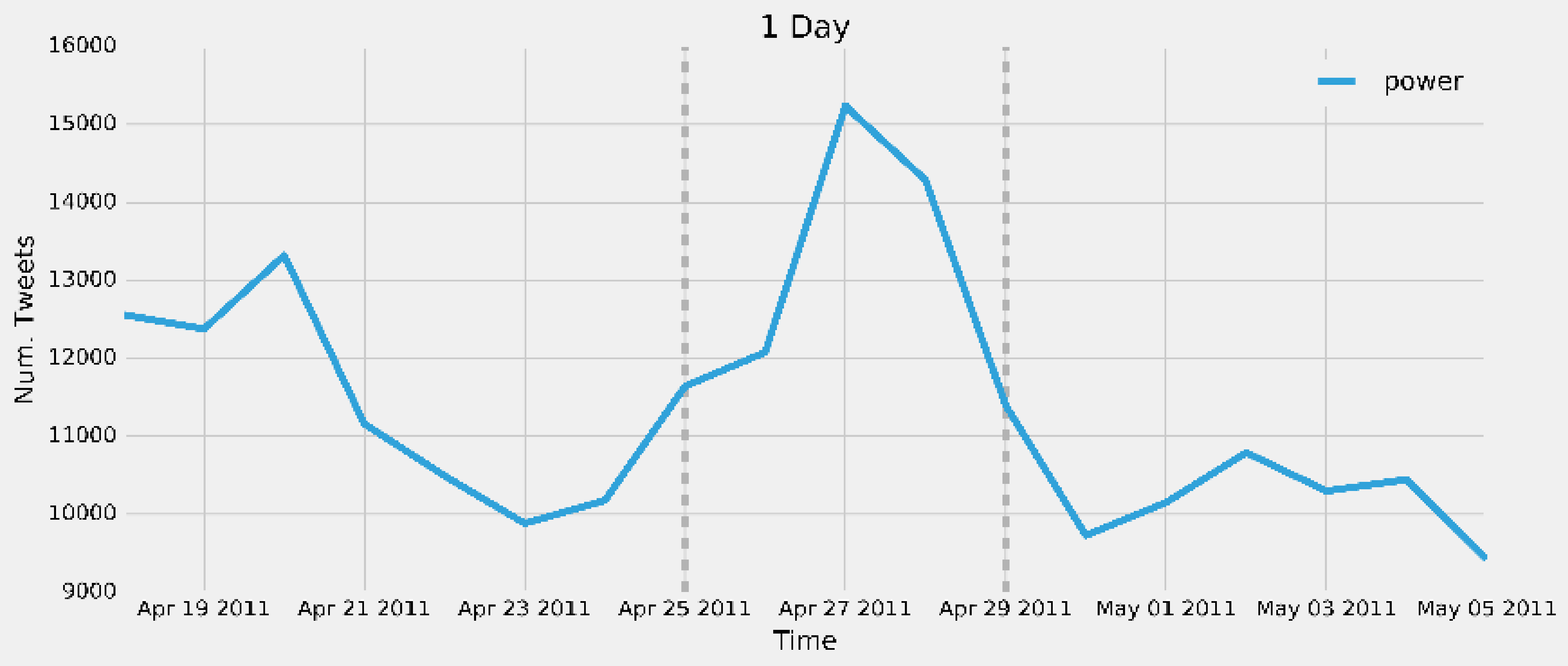

1 Day
power
Num. Tweets
16000
15000
14000
13000
12000
11000
10000
9000
Apr 19 2011
Apr 21 2011
Apr 23 2011
Apr 25 2011
Apr 27 2011
Apr 29 2011
May 01 2011
May 03 2011
May 05 2011
Time

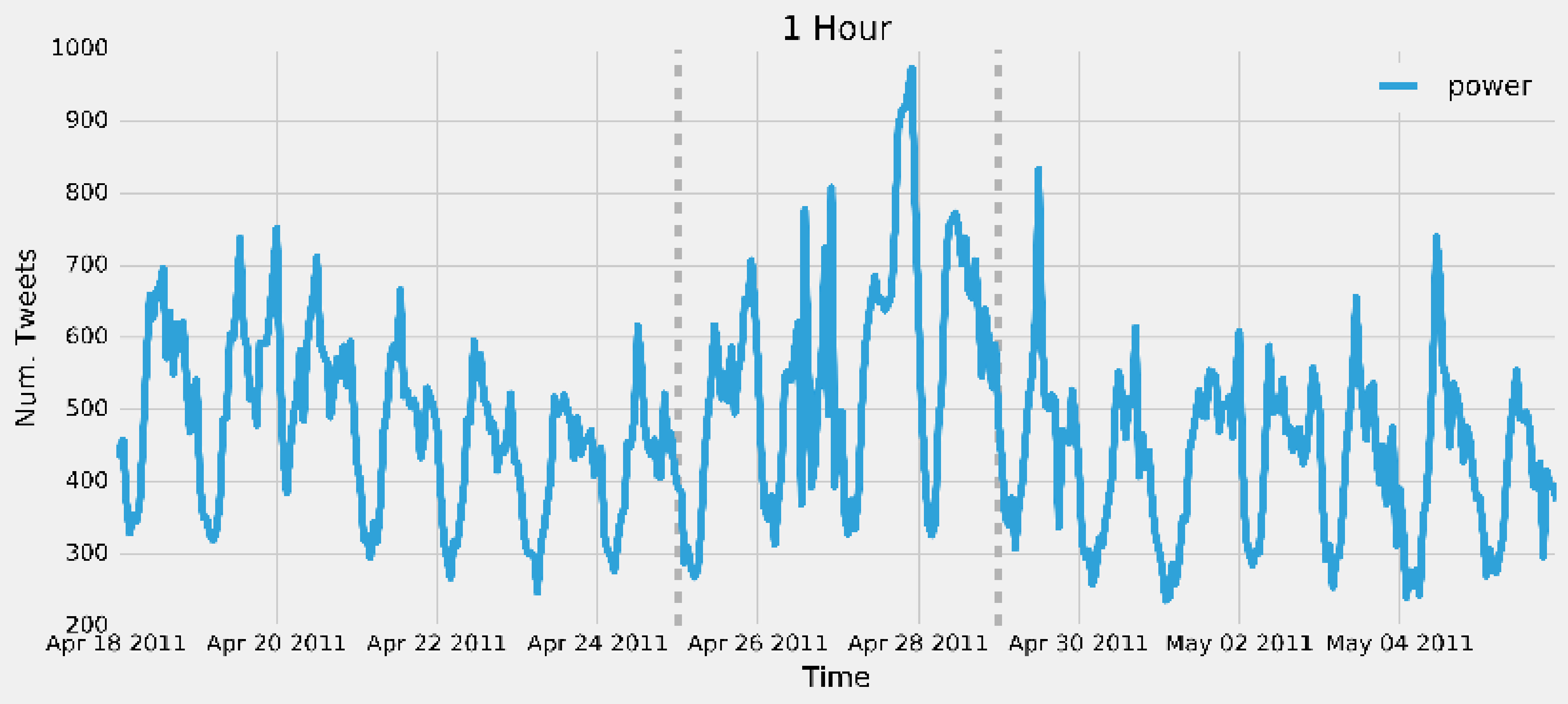
1 Hour
power
Num. Tweets
1000
900
800
700
600
500
400
300
200
Apr 18 2011
Apr 20 2011
Apr 22 2011
Apr 24 2011
Apr 26 2011
Apr 28 2011
Apr 30 2011
May 02 2011
May 04 2011
Time

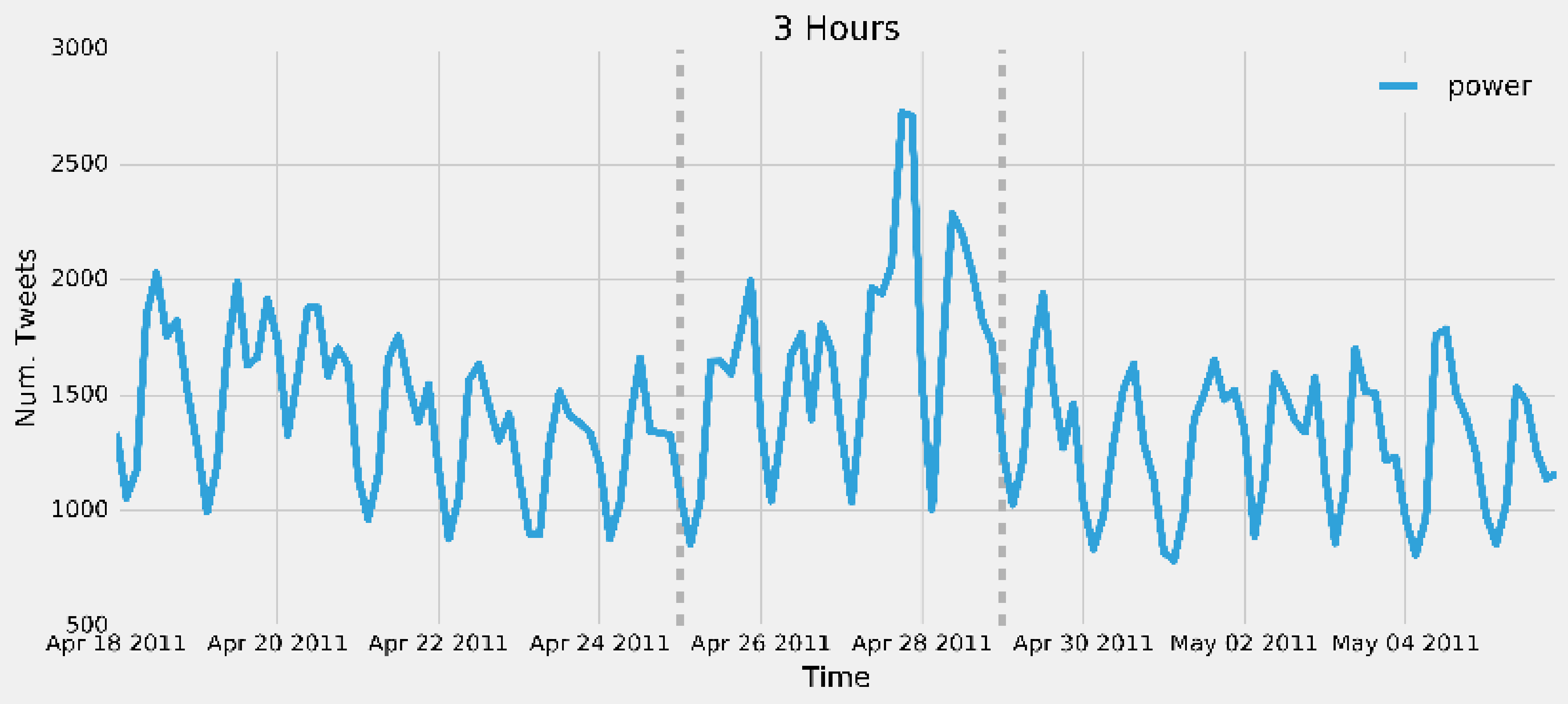

3 Hours
power
Num. Tweets
3000
2500
2000
1500
1000
500
Apr 18 2011
Apr 20 2011
Apr 22 2011
Apr 24 2011
Apr 26 2011
Apr 28 2011
Apr 30 2011
May 02 2011
May 04 2011
Time

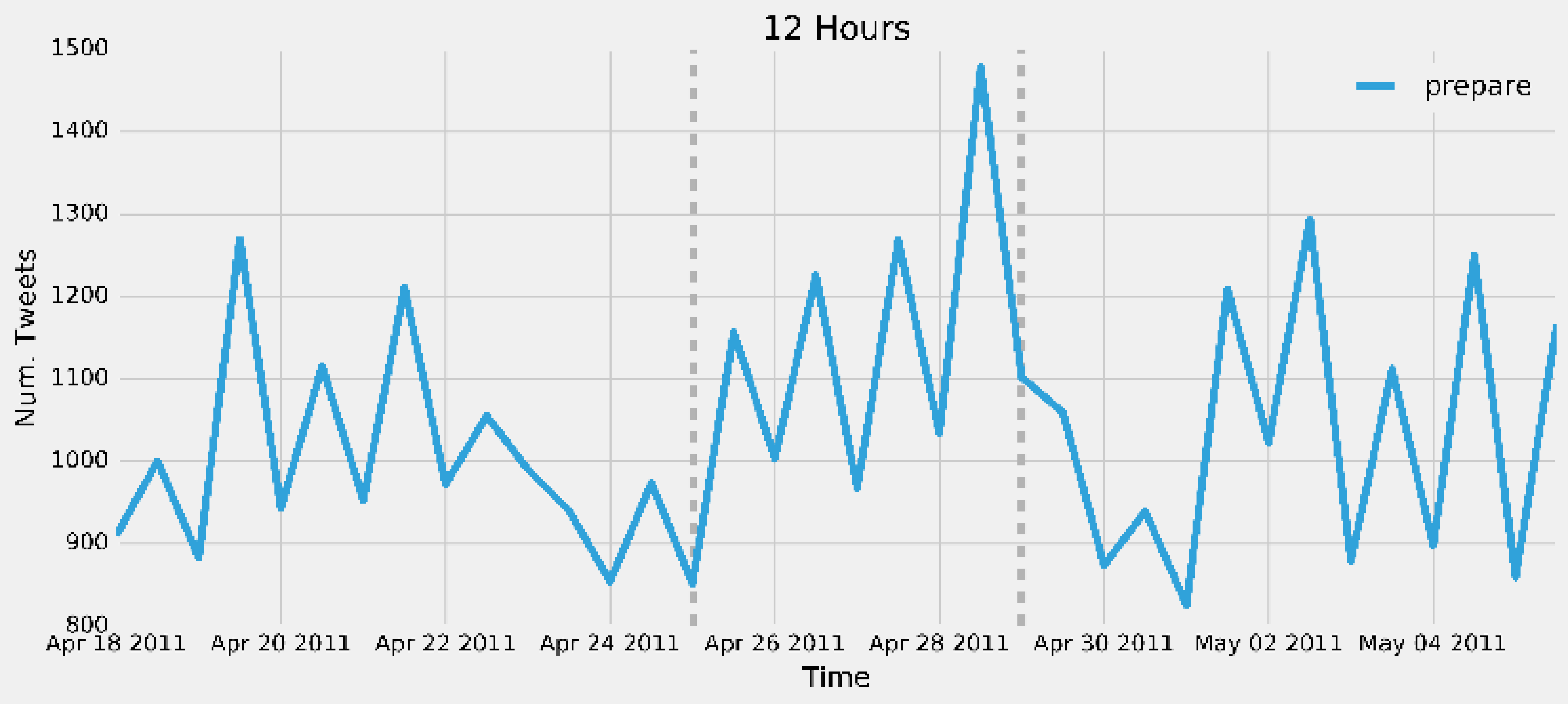
12 Hours
prepare
Num. Tweets
1500
1400
1300
1200
1100
1000
900
800
Apr 18 2011
Apr 20 2011
Apr 22 2011
Apr 24 2011
Apr 26 2011
Apr 28 2011
Apr 30 2011
May 02 2011
May 04 2011
Time

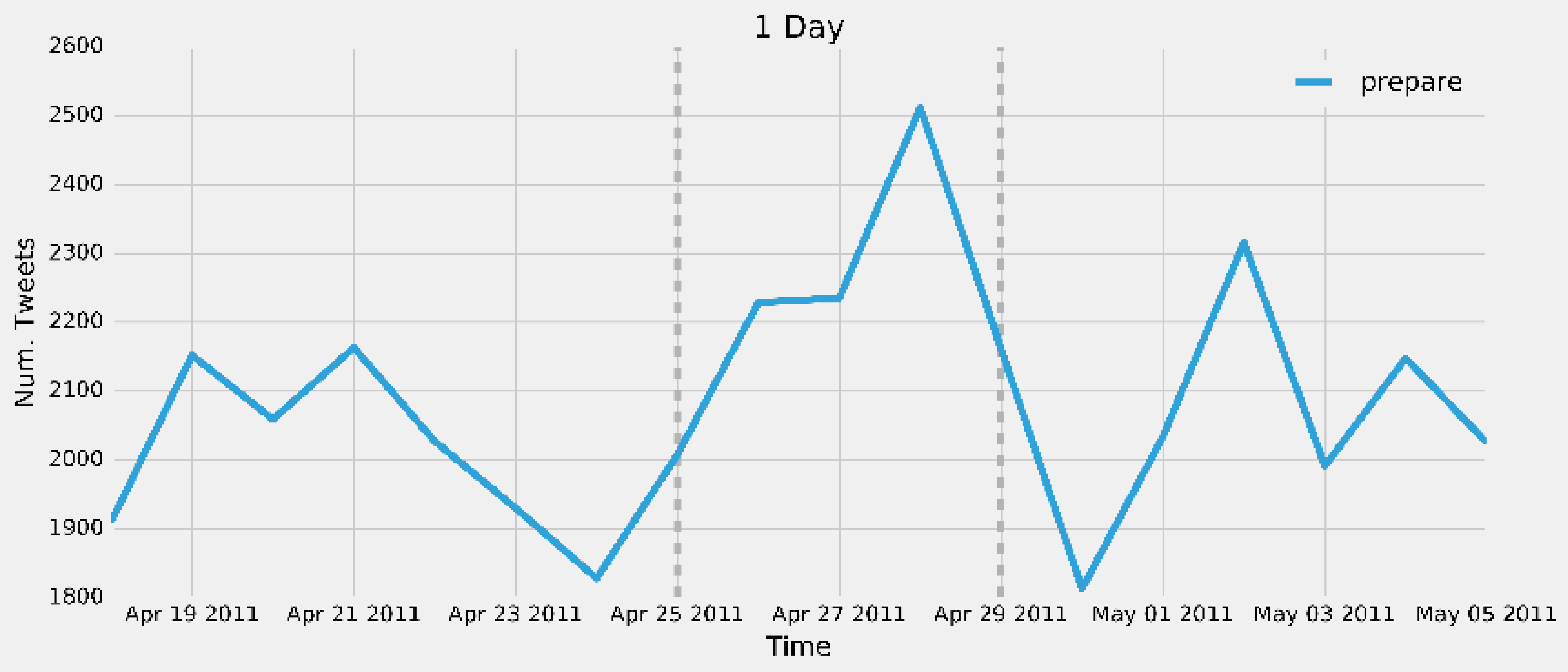
1 Day
prepare
Num. Tweets
2600
2500
2400
2300
2200
2100
2000
1900
1800
Apr 19 2011
Apr 21 2011
Apr 23 2011
Apr 25 2011
Apr 27 2011
Apr 29 2011
May 01 2011
May 03 2011
May 05 2011
Time

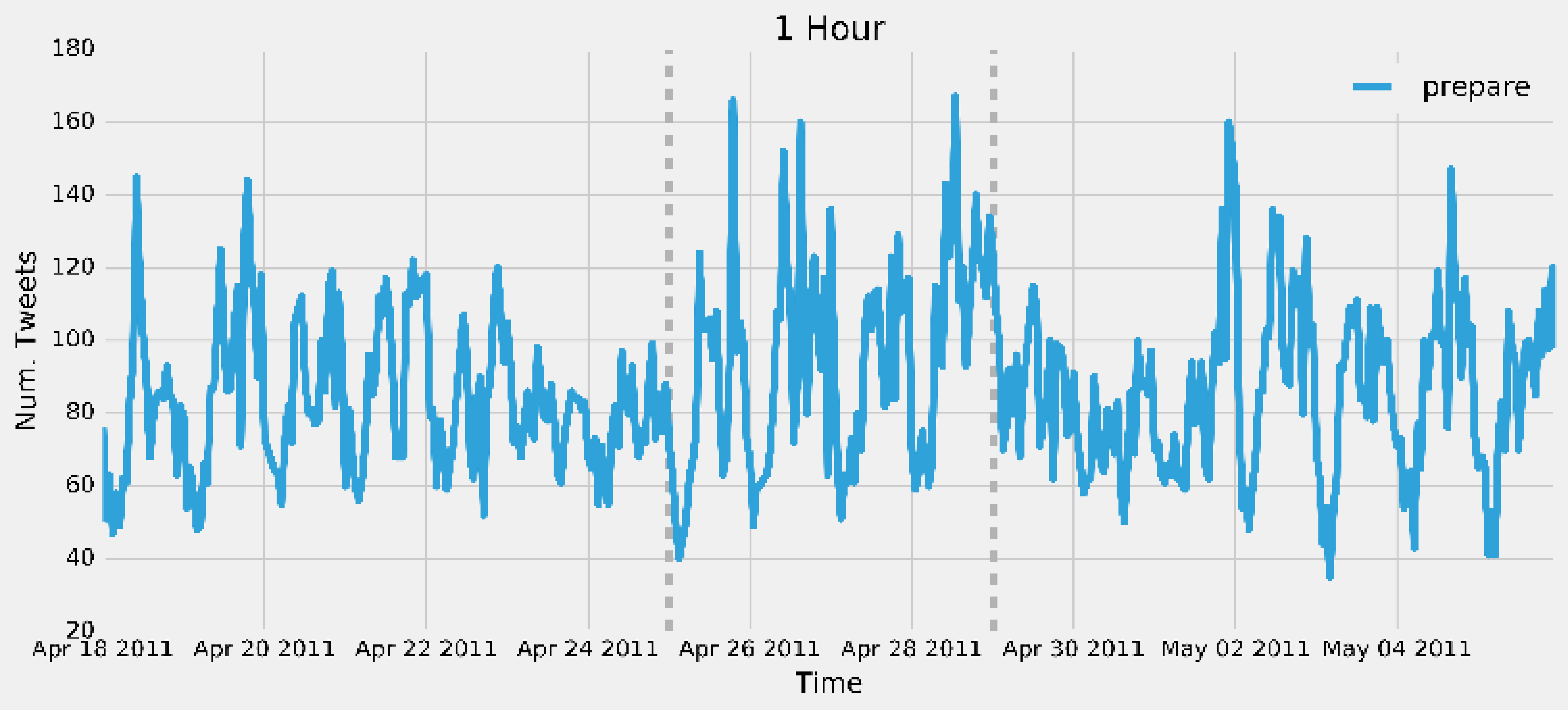
1 Hour
prepare
Num. Tweets
180
160
140
120
100
80
60
40
20
Apr 18 2011
Apr 20 2011
Apr 22 2011
Apr 24 2011
Apr 26 2011
Apr 28 2011
Apr 30 2011
May 02 2011
May 04 2011
Time

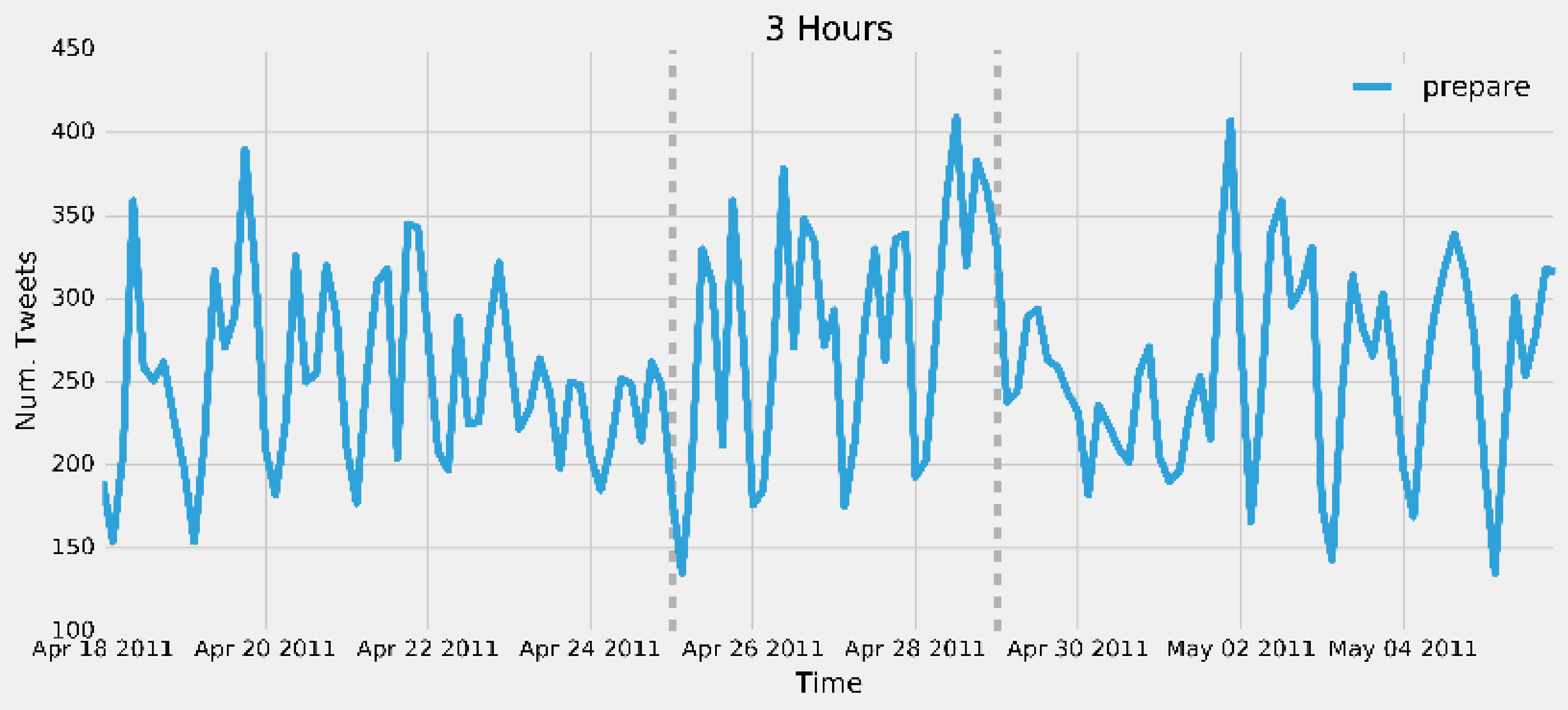

3 Hours
prepare
Num. Tweets
450
400
350
300
250
200
150
100
Apr 18 2011
Apr 20 2011
Apr 22 2011
Apr 24 2011
Apr 26 2011
Apr 28 2011
Apr 30 2011
May 02 2011
May 04 2011
Time

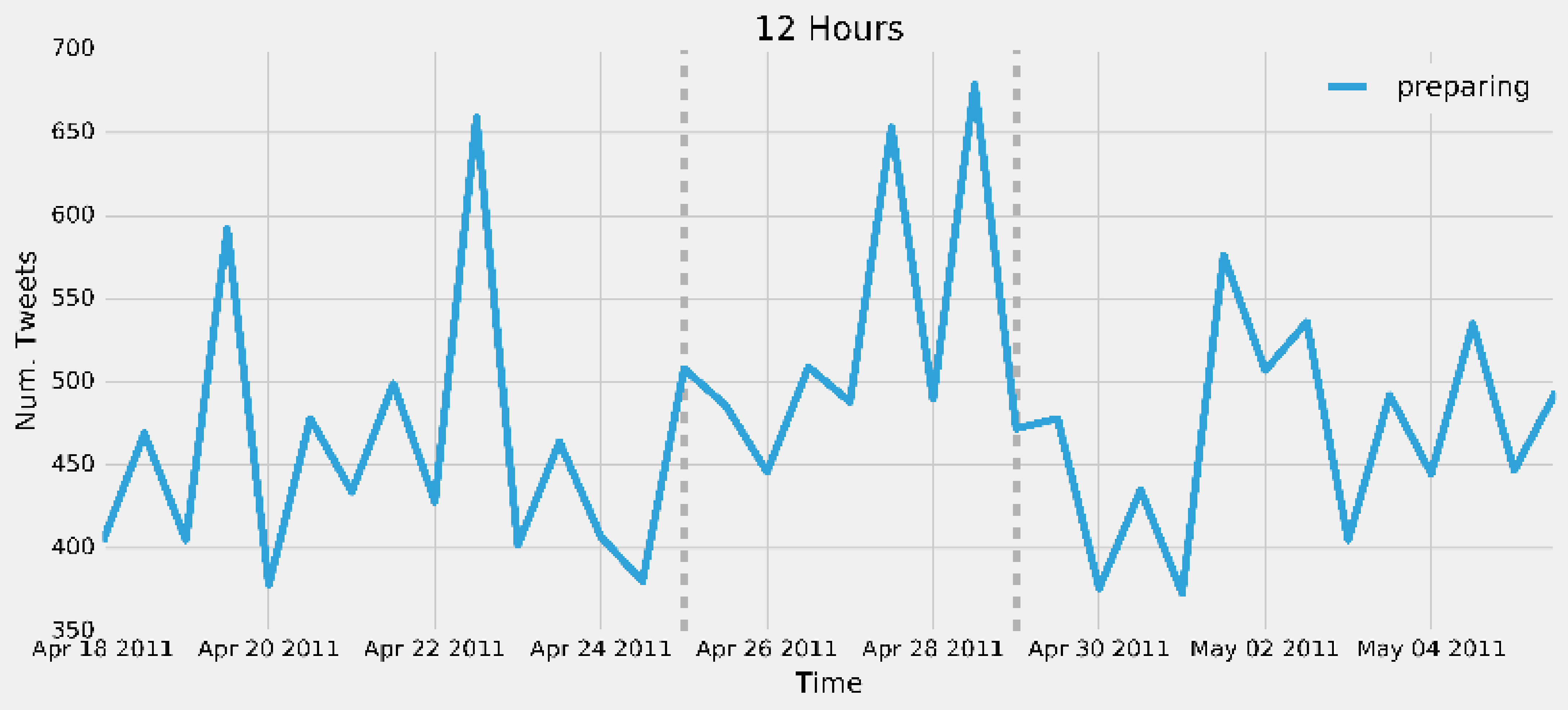

12 Hours
preparing
700
650
600
550
500
450
400
350
Num. Tweets
Apr 18 2011
Apr 20 2011
Apr 22 2011
Apr 24 2011
Apr 26 2011
Apr 28 2011
Apr 30 2011
May 02 2011
May 04 2011
Time

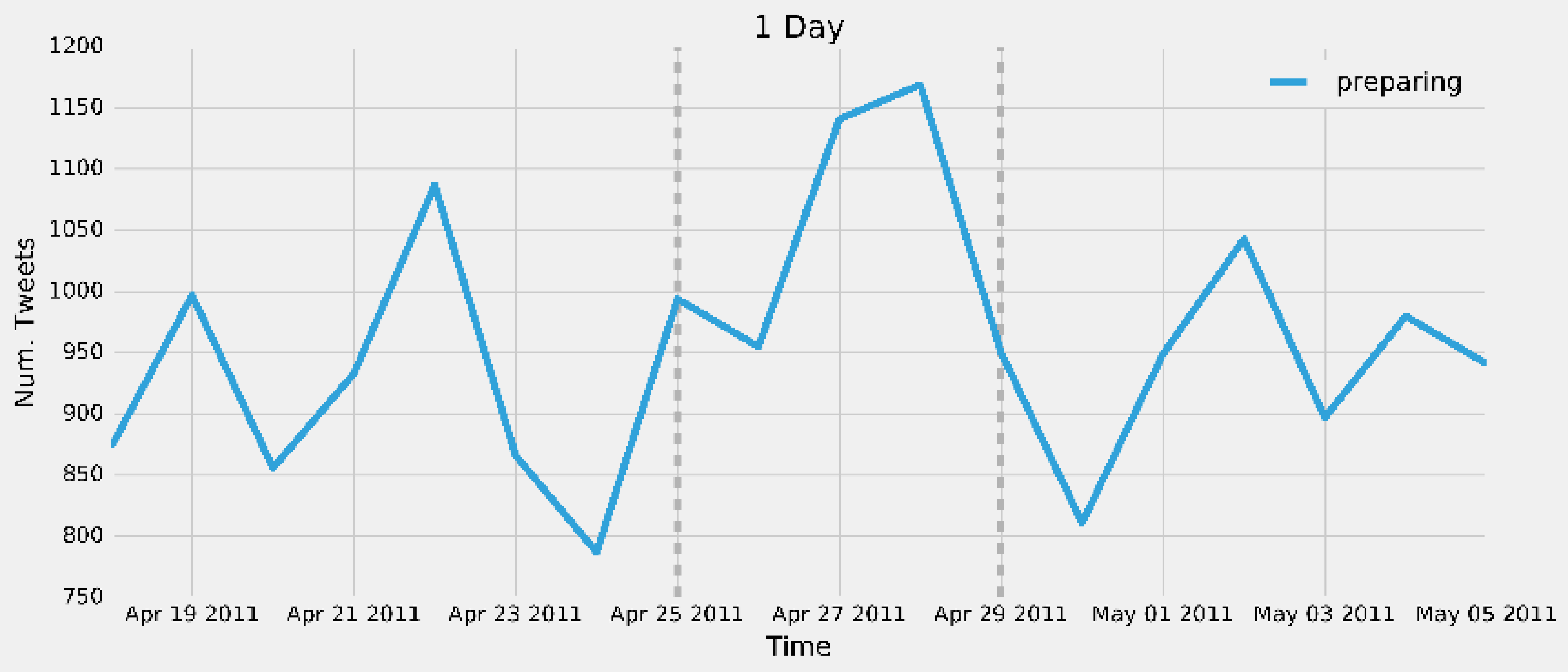
1 Day
preparing
Num. Tweets
1200
1150
1100
1050
1000
950
900
850
800
750
Apr 19 2011
Apr 21 2011
Apr 23 2011
Apr 25 2011
Apr 27 2011
Apr 29 2011
May 01 2011
May 03 2011
May 05 2011
Time

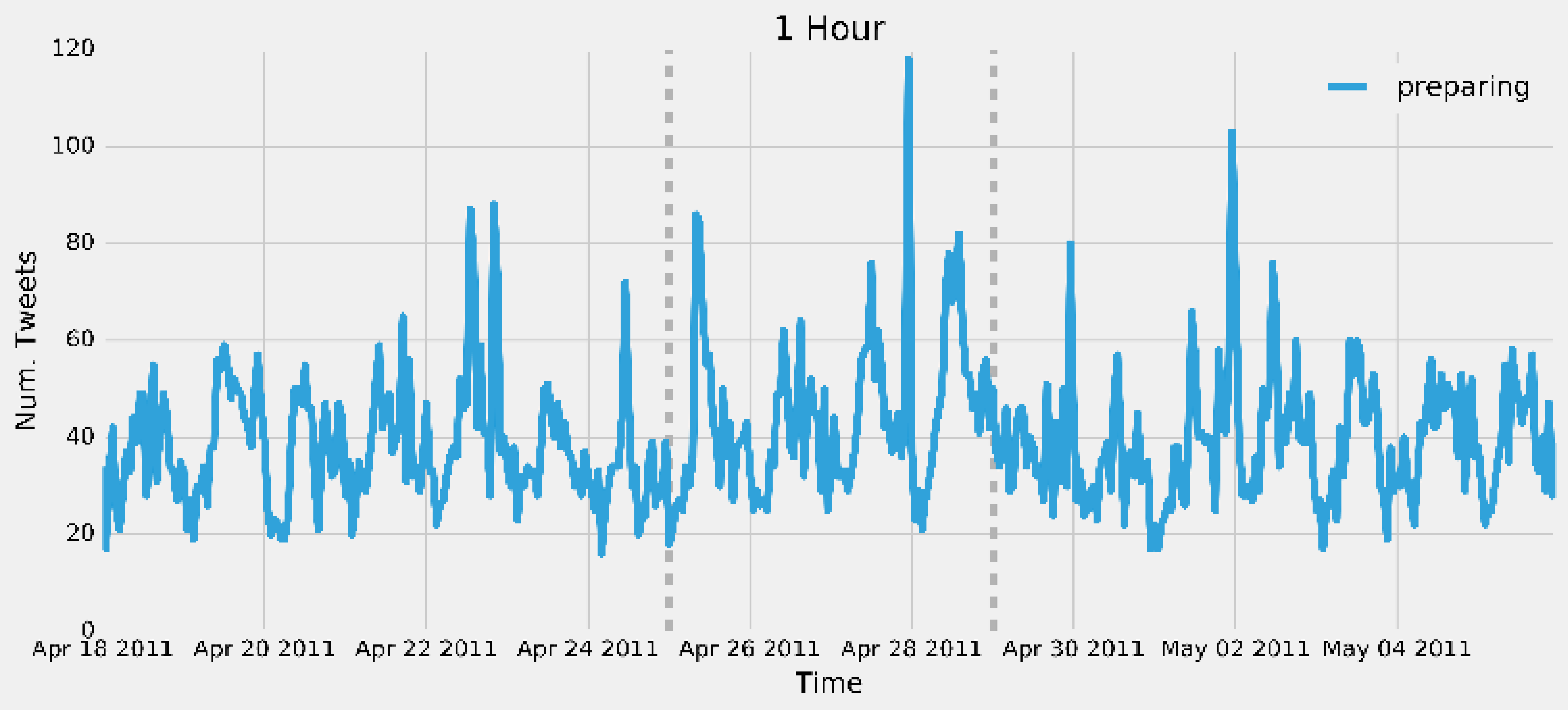
1 Hour
preparing
Num. Tweets
Time
0
20
40
60
80
100
120
Apr 18 2011
Apr 20 2011
Apr 22 2011
Apr 24 2011
Apr 26 2011
Apr 28 2011
Apr 30 2011
May 02 2011
May 04 2011

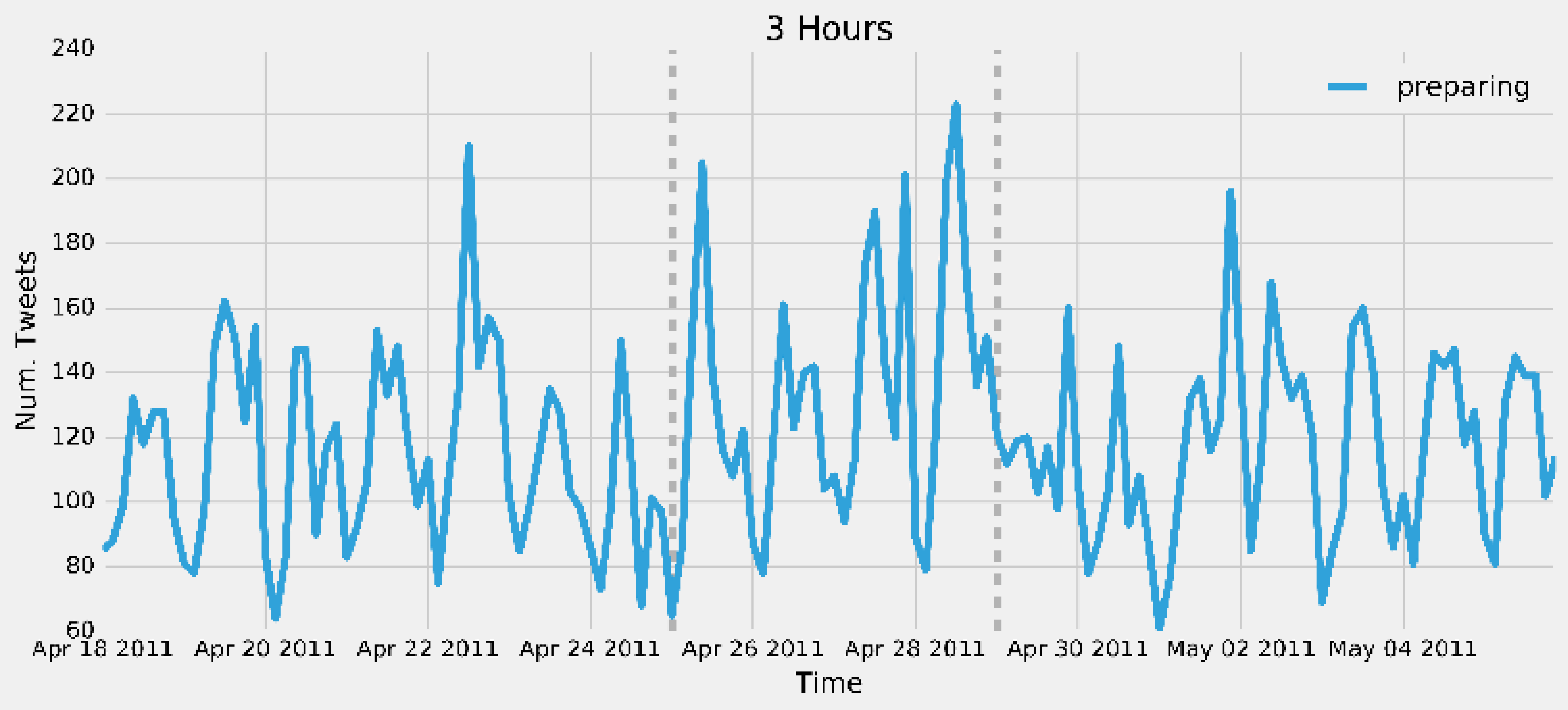
3 Hours
preparing
Num. Tweets
240
220
200
180
160
140
120
100
80
60
Apr 18 2011
Apr 20 2011
Apr 22 2011
Apr 24 2011
Apr 26 2011
Apr 28 2011
Apr 30 2011
May 02 2011
May 04 2011
Time

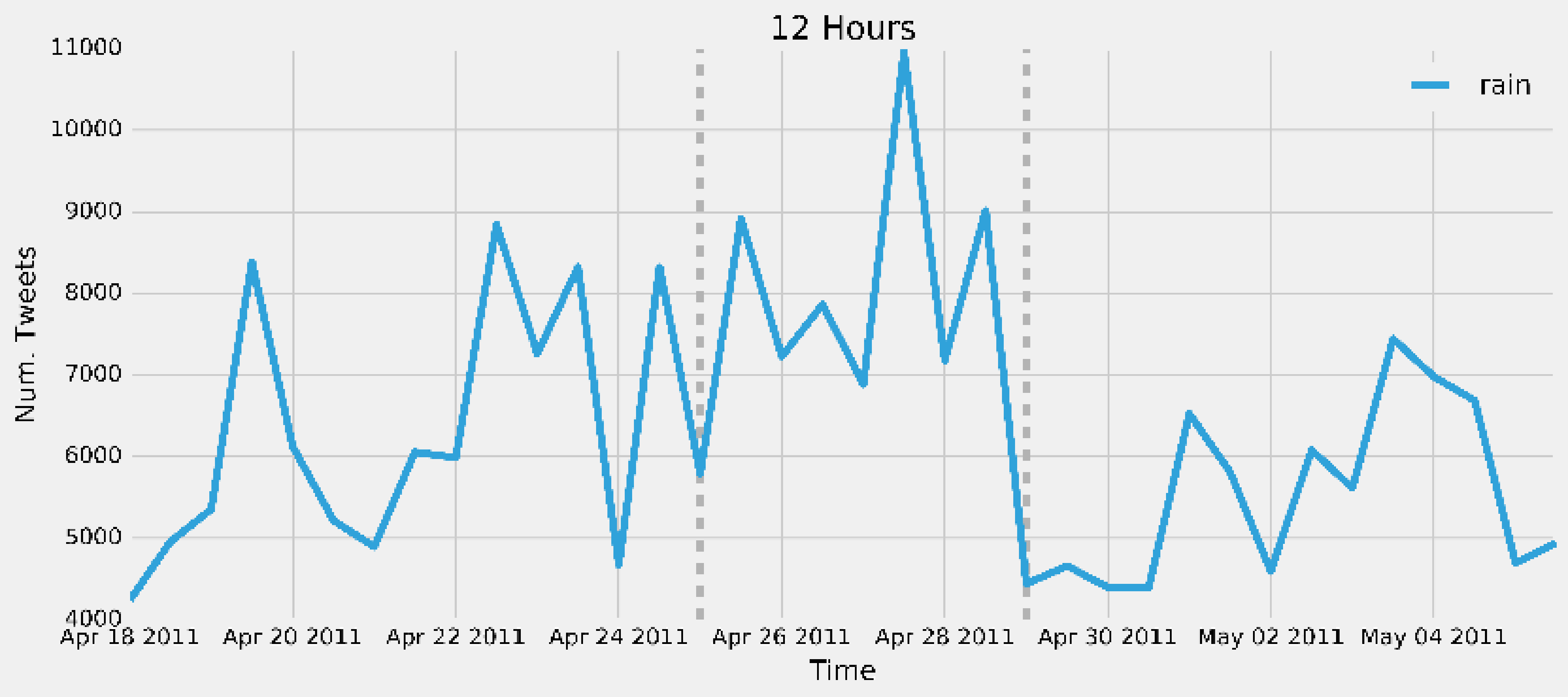

12 Hours
rain
Num. Tweets
Time
11000
10000
9000
8000
7000
6000
5000
4000
Apr 18 2011
Apr 20 2011
Apr 22 2011
Apr 24 2011
Apr 26 2011
Apr 28 2011
Apr 30 2011
May 02 2011
May 04 2011

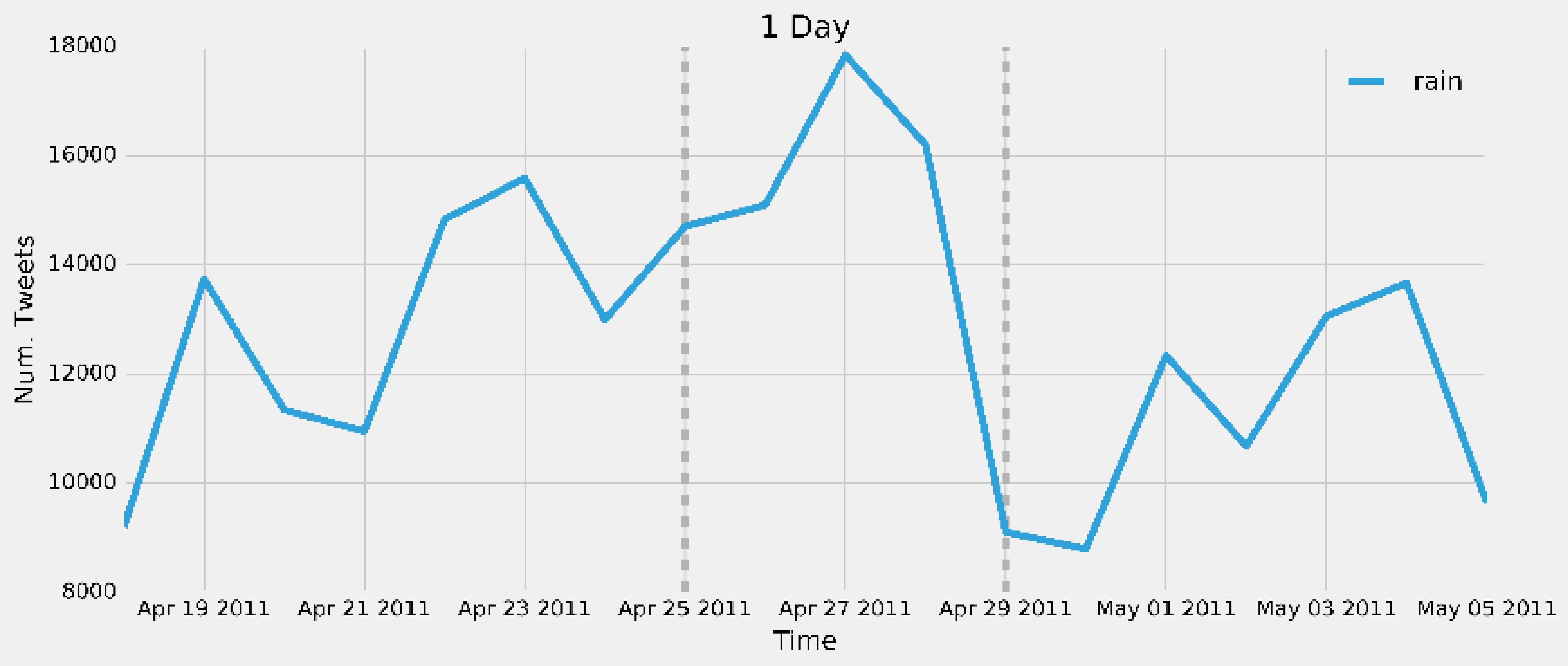

1 Day
rain
18000
16000
14000
12000
10000
8000
Num. Tweets
Apr 19 2011
Apr 21 2011
Apr 23 2011
Apr 25 2011
Apr 27 2011
Apr 29 2011
May 01 2011
May 03 2011
May 05 2011
Time

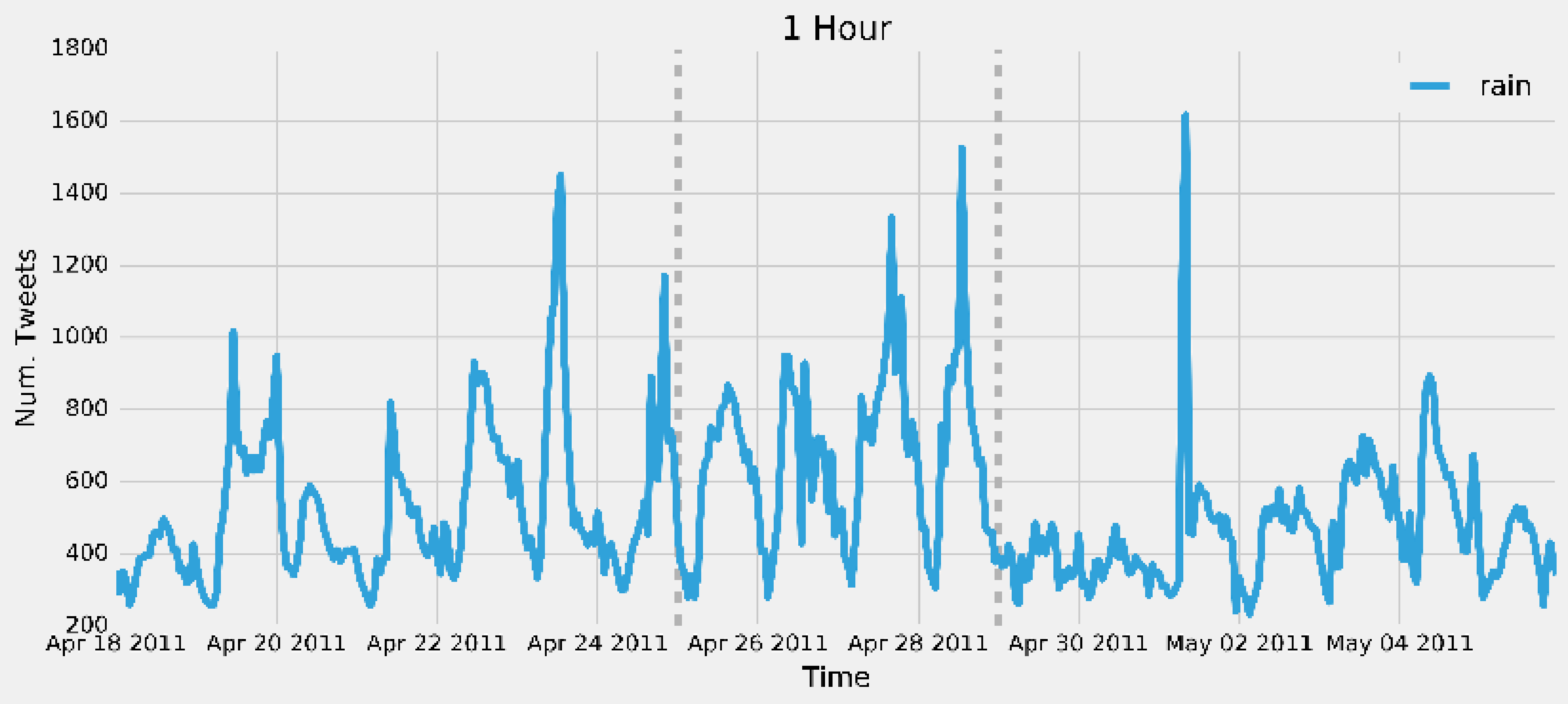

1 Hour
rain
Num. Tweets
200
400
600
800
1000
1200
1400
1600
1800
Apr 18 2011
Apr 20 2011
Apr 22 2011
Apr 24 2011
Apr 26 2011
Apr 28 2011
Apr 30 2011
May 02 2011
May 04 2011
Time

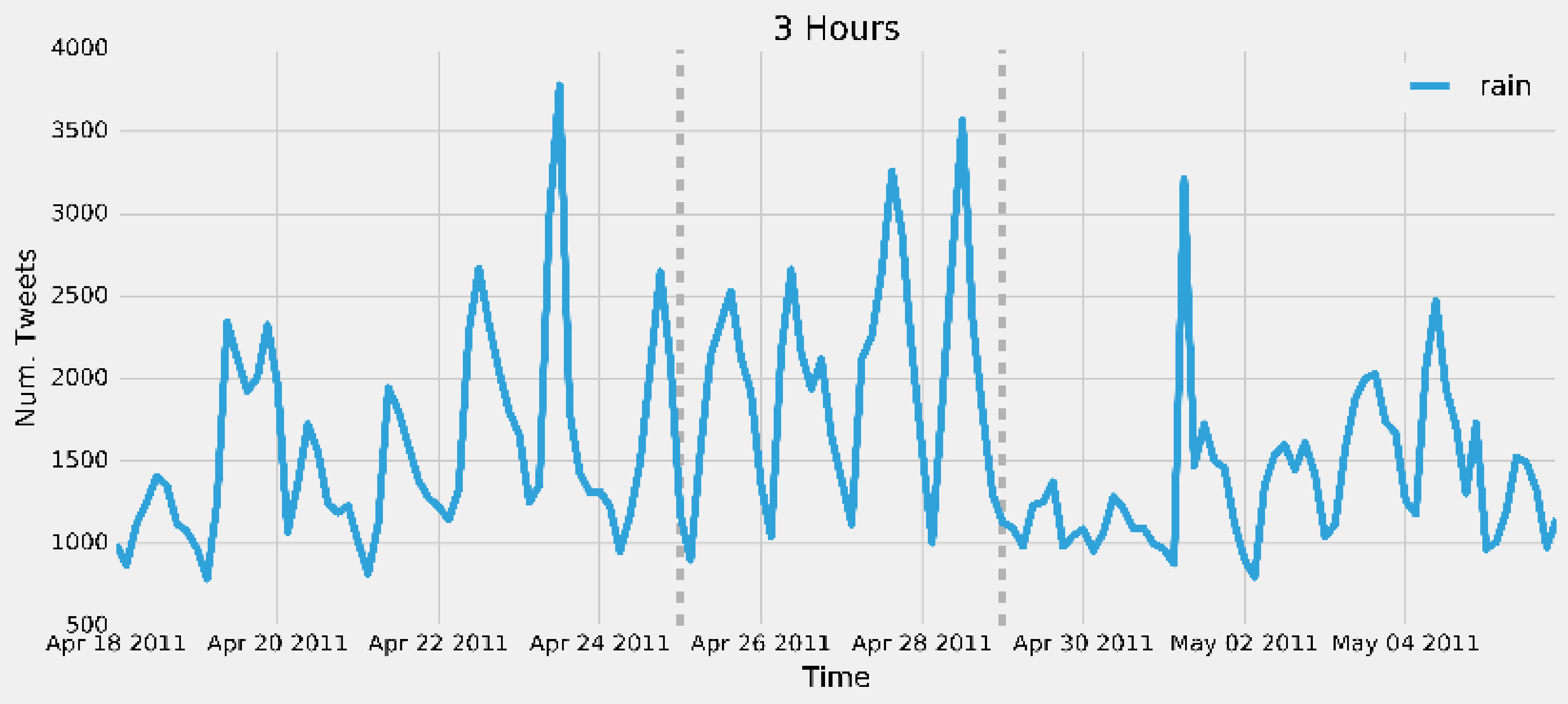

3 Hours
rain
Num. Tweets
4000
3500
3000
2500
2000
1500
1000
500
Apr 18 2011
Apr 20 2011
Apr 22 2011
Apr 24 2011
Apr 26 2011
Apr 28 2011
Apr 30 2011
May 02 2011
May 04 2011
Time

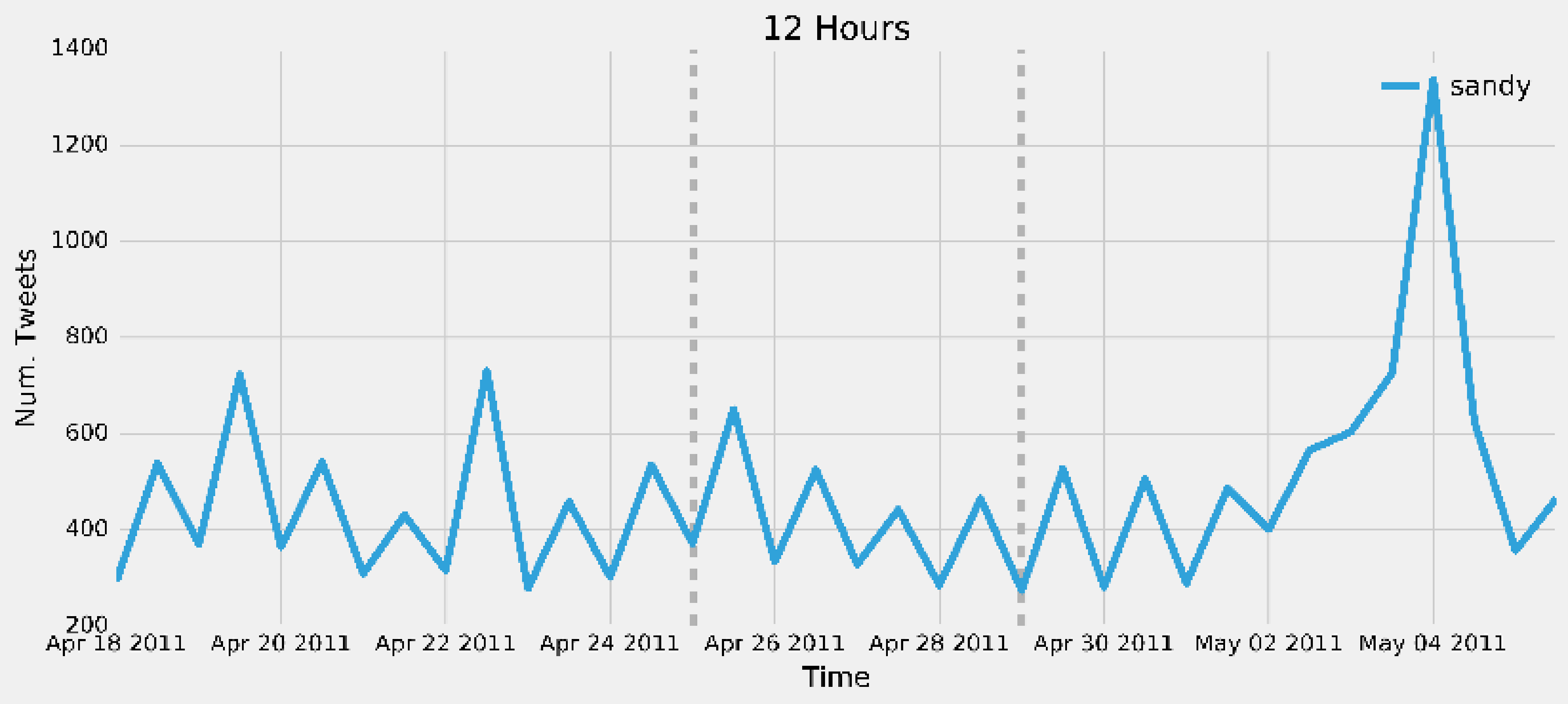

12 Hours
sandy
1400
1200
1000
800
600
400
200
Num. Tweets
Apr 18 2011
Apr 20 2011
Apr 22 2011
Apr 24 2011
Apr 26 2011
Apr 28 2011
Apr 30 2011
May 02 2011
May 04 2011
Time

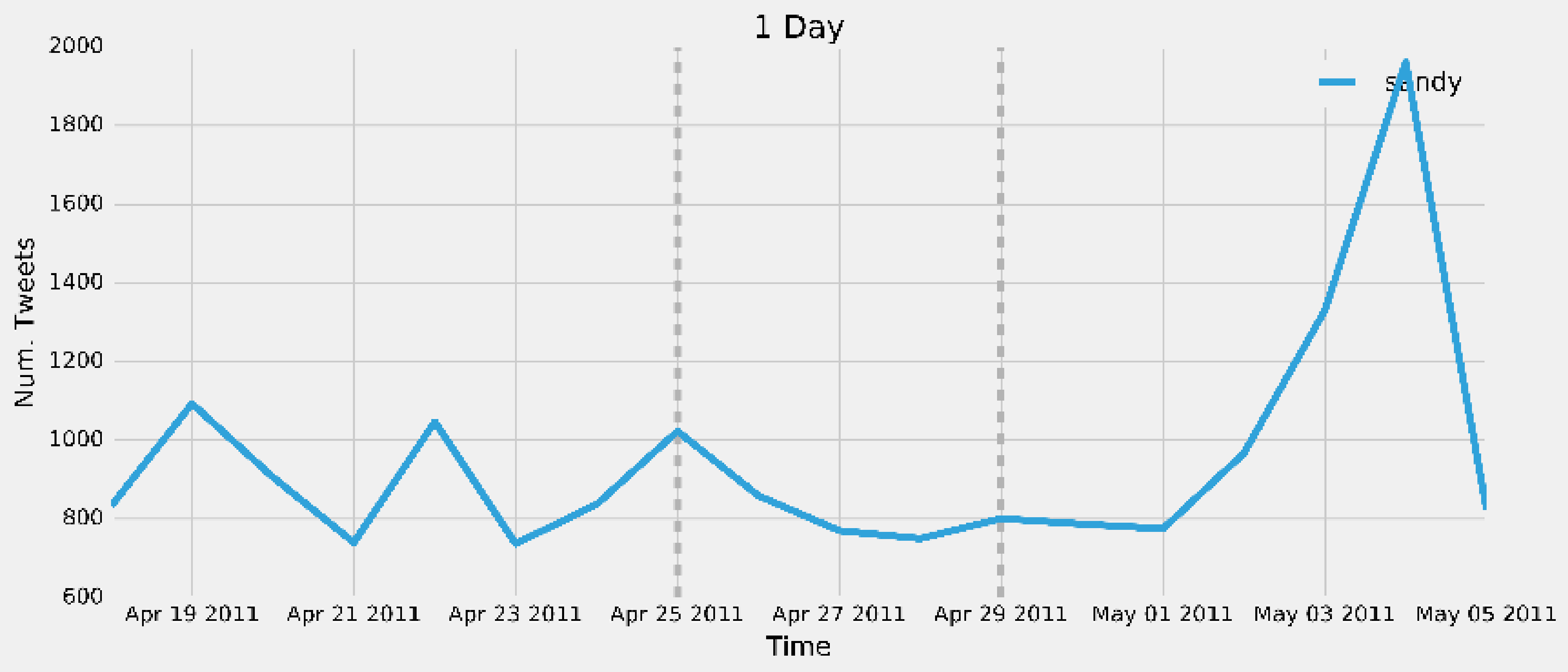

1 Day
sandy
2000
1800
1600
1400
1200
1000
800
600
Num. Tweets
Apr 19 2011
Apr 21 2011
Apr 23 2011
Apr 25 2011
Apr 27 2011
Apr 29 2011
May 01 2011
May 03 2011
May 05 2011
Time

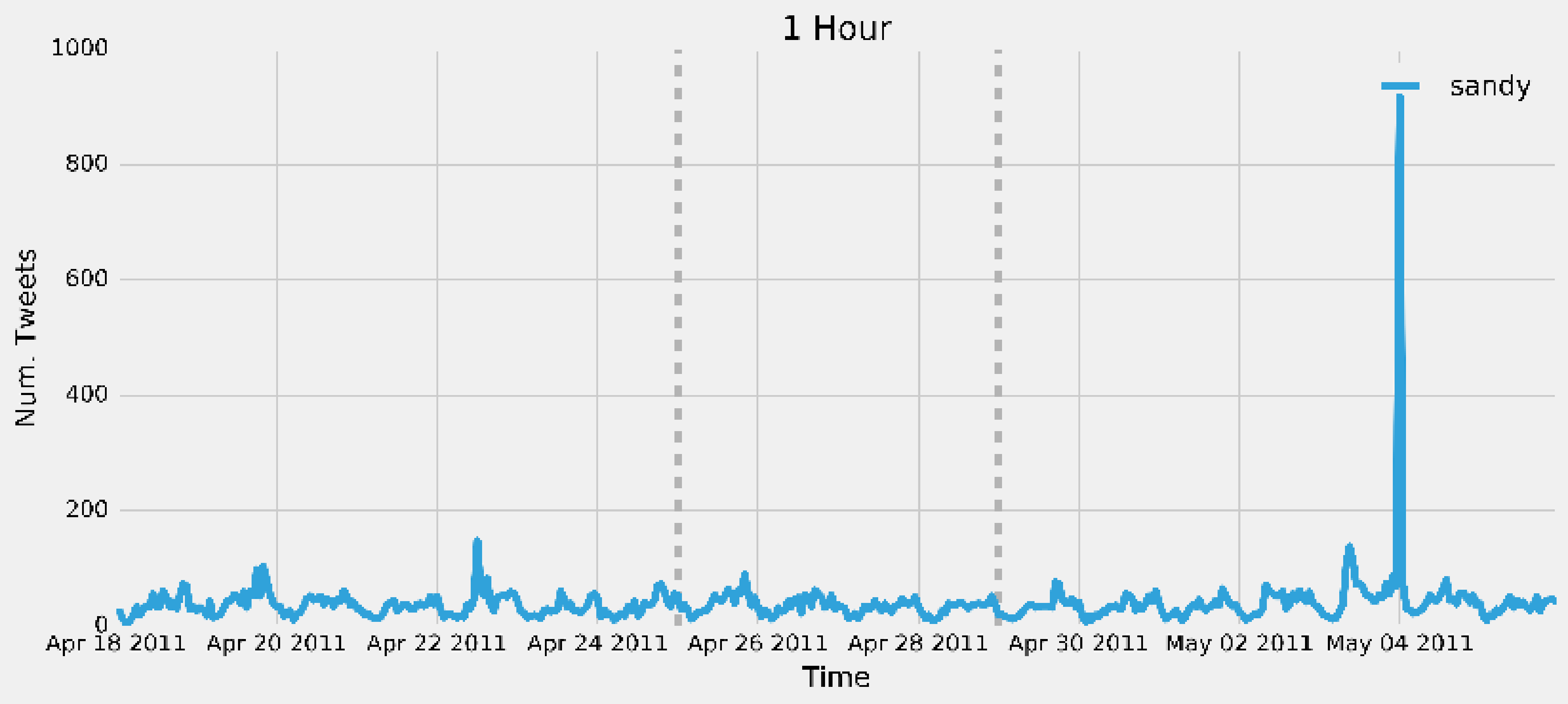

1 Hour
Num. Tweets
Time
sandy
0
200
400
600
800
1000
Apr 18 2011
Apr 20 2011
Apr 22 2011
Apr 24 2011
Apr 26 2011
Apr 28 2011
Apr 30 2011
May 02 2011
May 04 2011

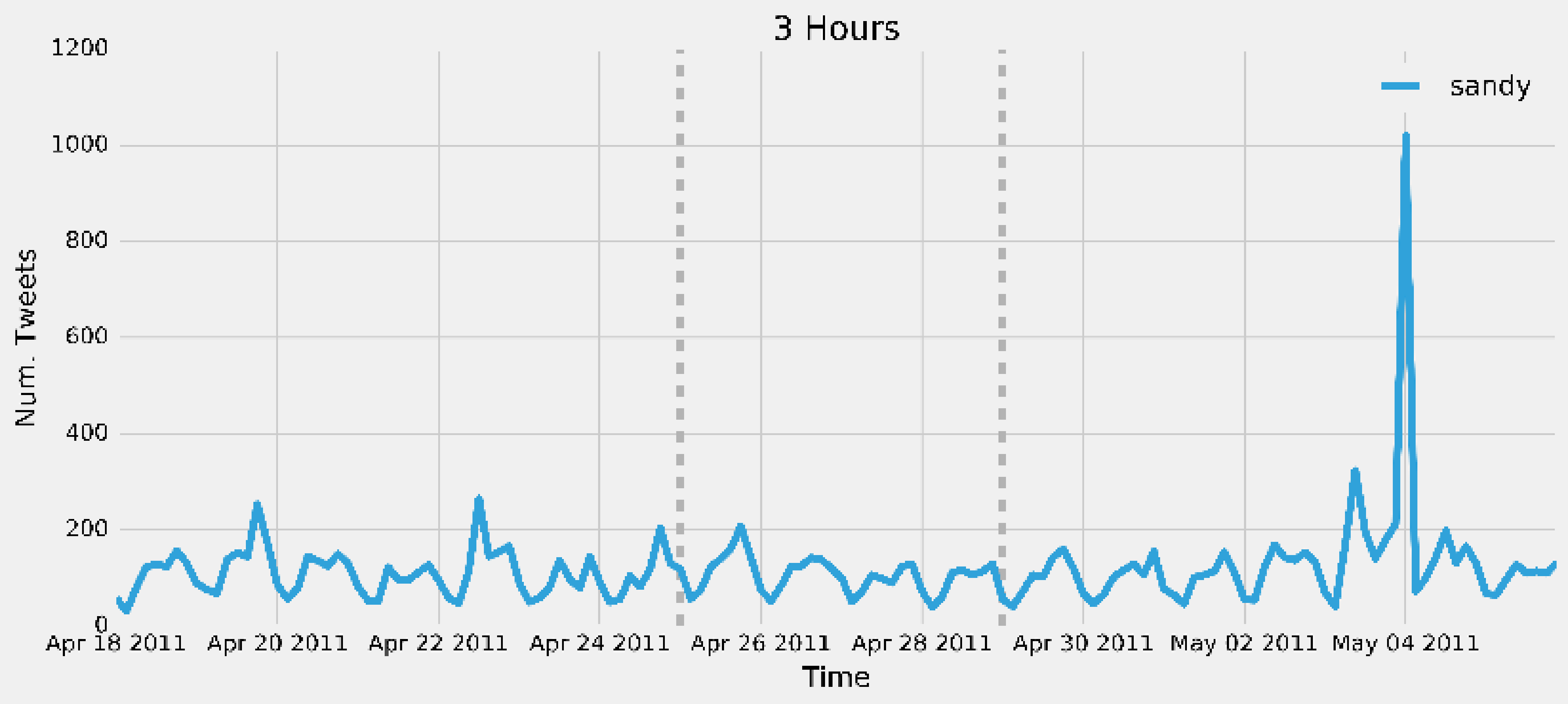
3 Hours
sandy
Num. Tweets
Time
0
200
400
600
800
1000
1200
Apr 18 2011
Apr 20 2011
Apr 22 2011
Apr 24 2011
Apr 26 2011
Apr 28 2011
Apr 30 2011
May 02 2011
May 04 2011

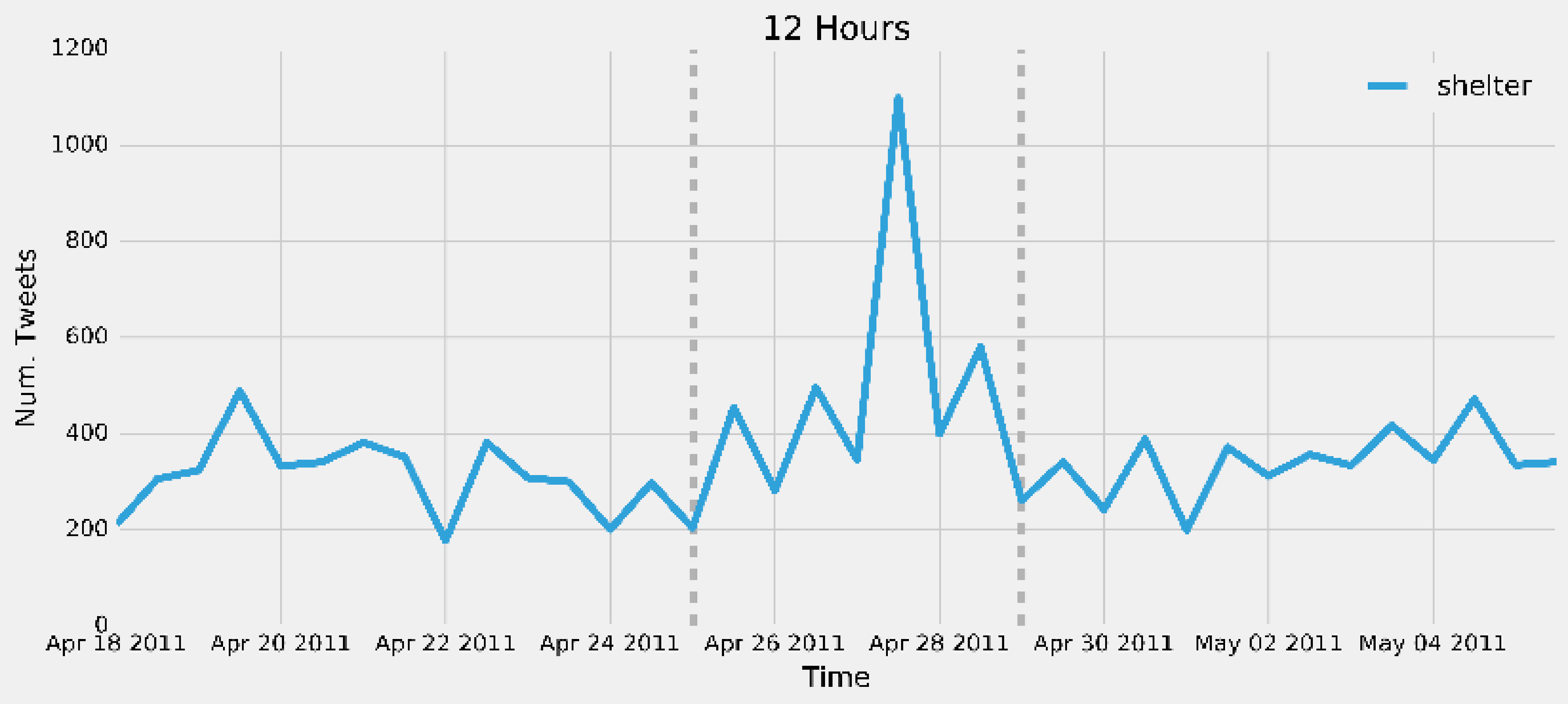
12 Hours
shelter
Num. Tweets
0
200
400
600
800
1000
1200
Apr 18 2011
Apr 20 2011
Apr 22 2011
Apr 24 2011
Apr 26 2011
Apr 28 2011
Apr 30 2011
May 02 2011
May 04 2011
Time

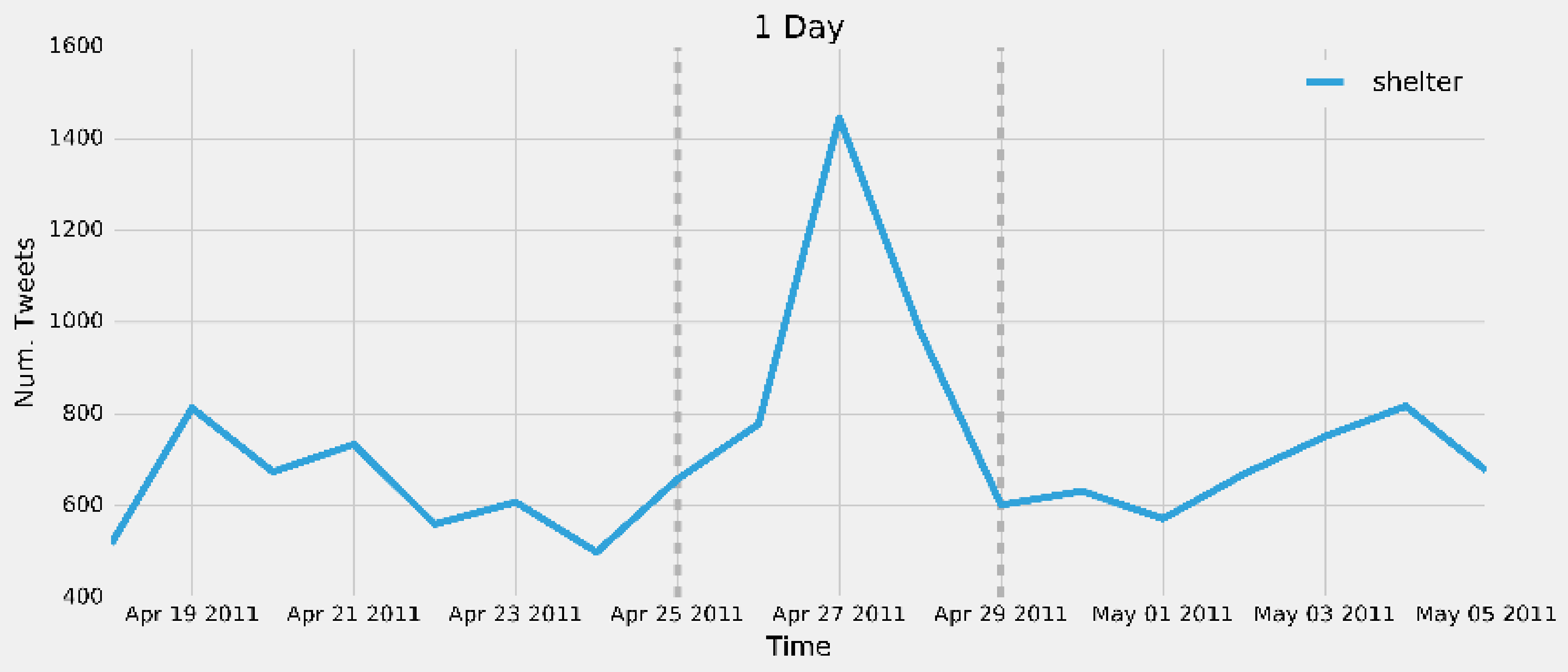

1 Day
shelter
Num. Tweets
400
600
800
1000
1200
1400
1600
Apr 19 2011
Apr 21 2011
Apr 23 2011
Apr 25 2011
Apr 27 2011
Apr 29 2011
May 01 2011
May 03 2011
May 05 2011
Time

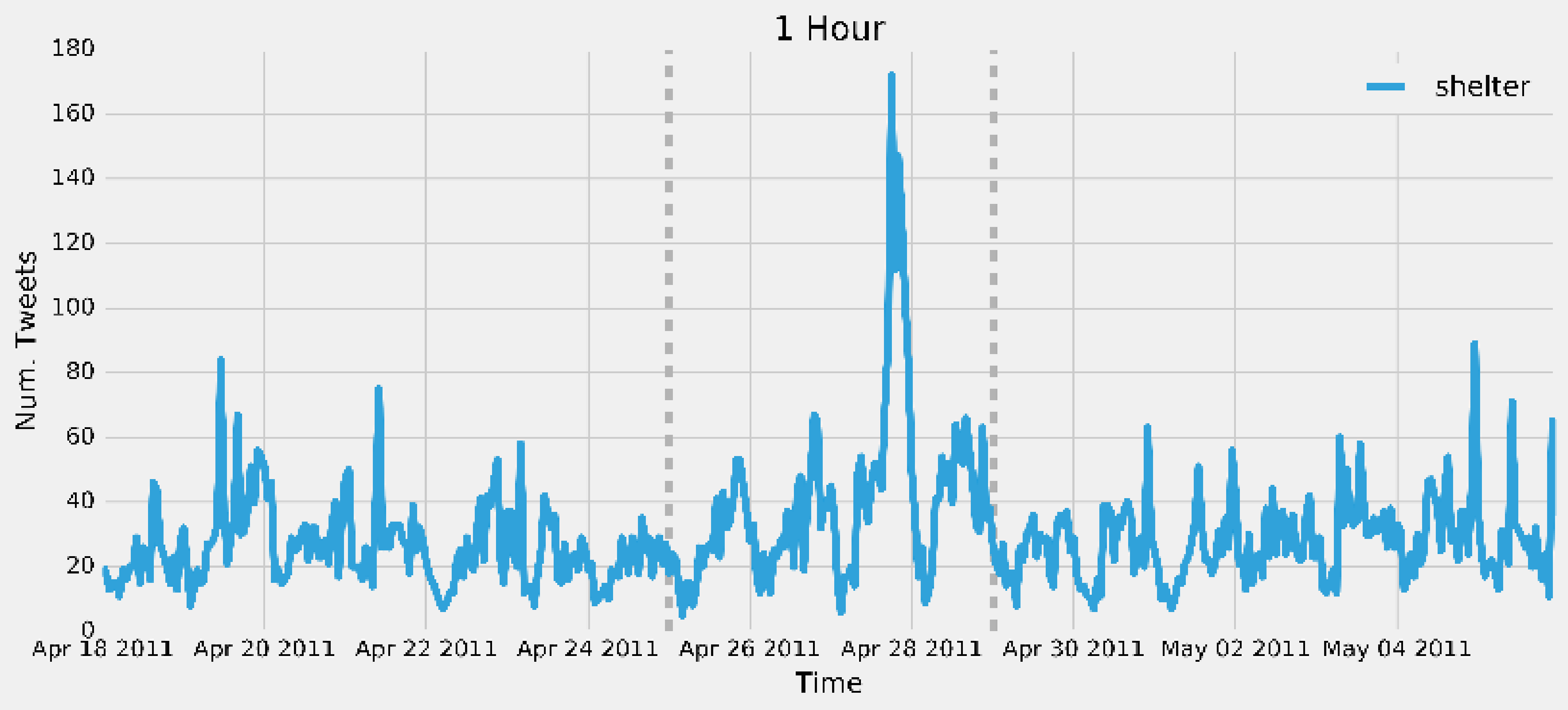

1 Hour
Num. Tweets
0
20
40
60
80
100
120
140
160
180
shelter
Apr 18 2011
Apr 20 2011
Apr 22 2011
Apr 24 2011
Apr 26 2011
Apr 28 2011
Apr 30 2011
May 02 2011
May 04 2011
Time

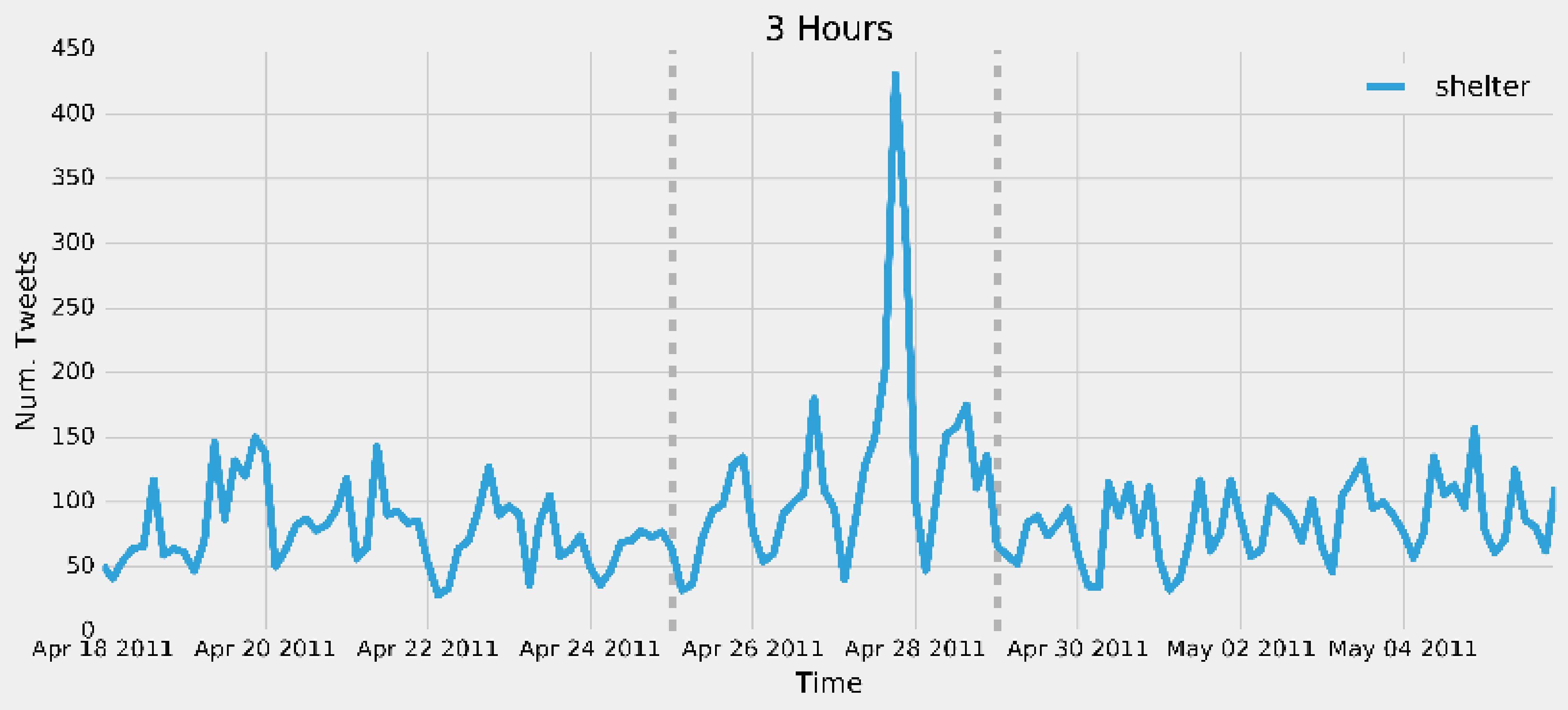

3 Hours
shelter
Num. Tweets
0
50
100
150
200
250
300
350
400
450
Apr 18 2011
Apr 20 2011
Apr 22 2011
Apr 24 2011
Apr 26 2011
Apr 28 2011
Apr 30 2011
May 02 2011
May 04 2011
Time

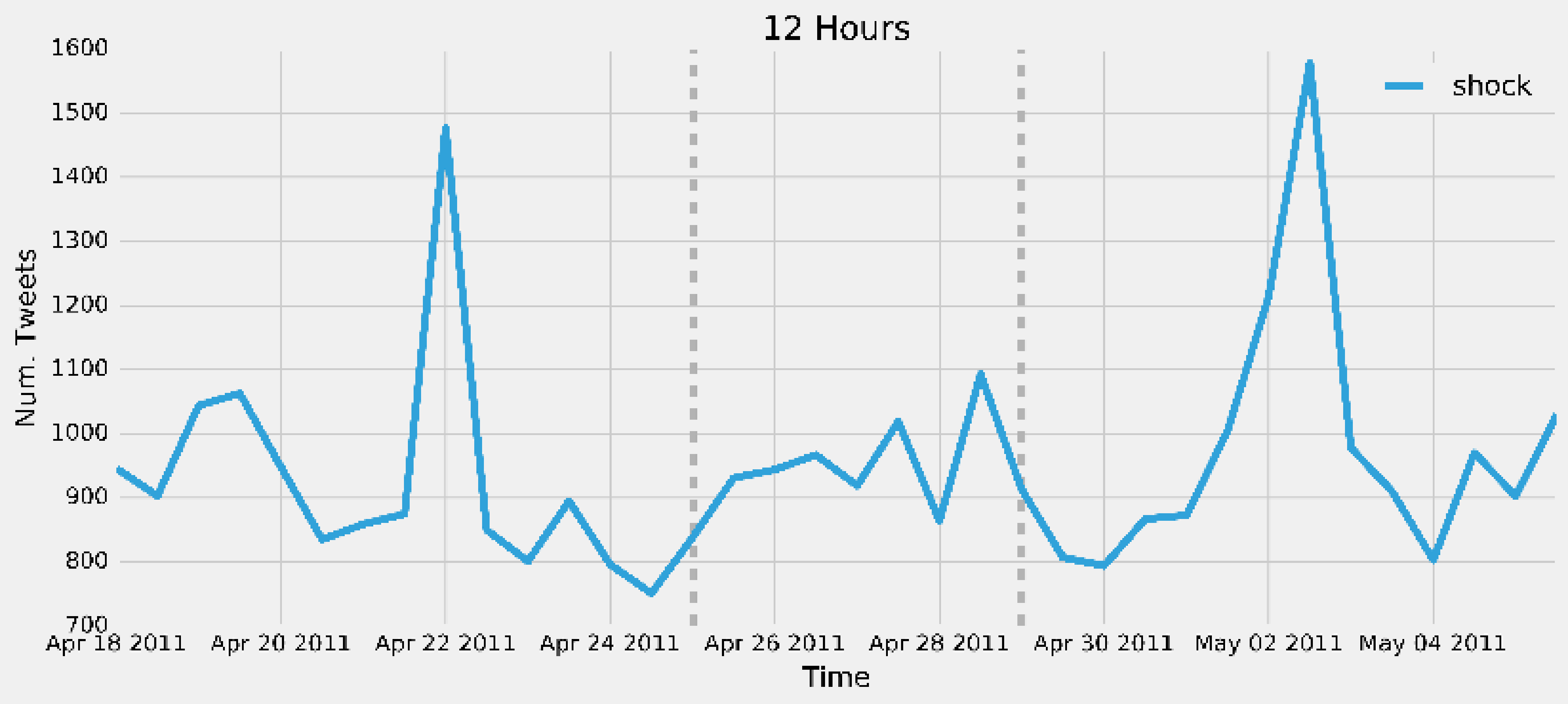

12 Hours
shock
Num. Tweets
1600
1500
1400
1300
1200
1100
1000
900
800
700
Apr 18 2011
Apr 20 2011
Apr 22 2011
Apr 24 2011
Apr 26 2011
Apr 28 2011
Apr 30 2011
May 02 2011
May 04 2011
Time

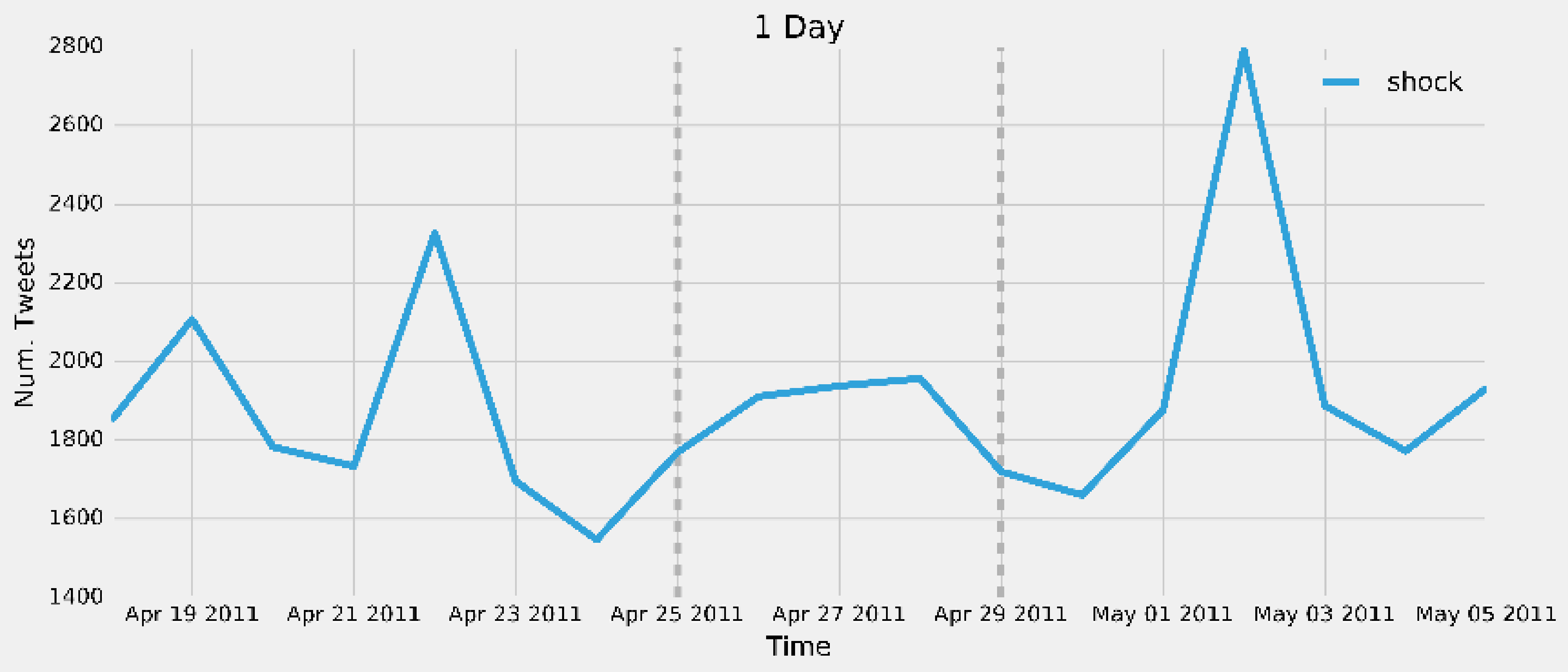

1 Day
shock
Num. Tweets
Time
2800
2600
2400
2200
2000
1800
1600
1400
Apr 19 2011
Apr 21 2011
Apr 23 2011
Apr 25 2011
Apr 27 2011
Apr 29 2011
May 01 2011
May 03 2011
May 05 2011

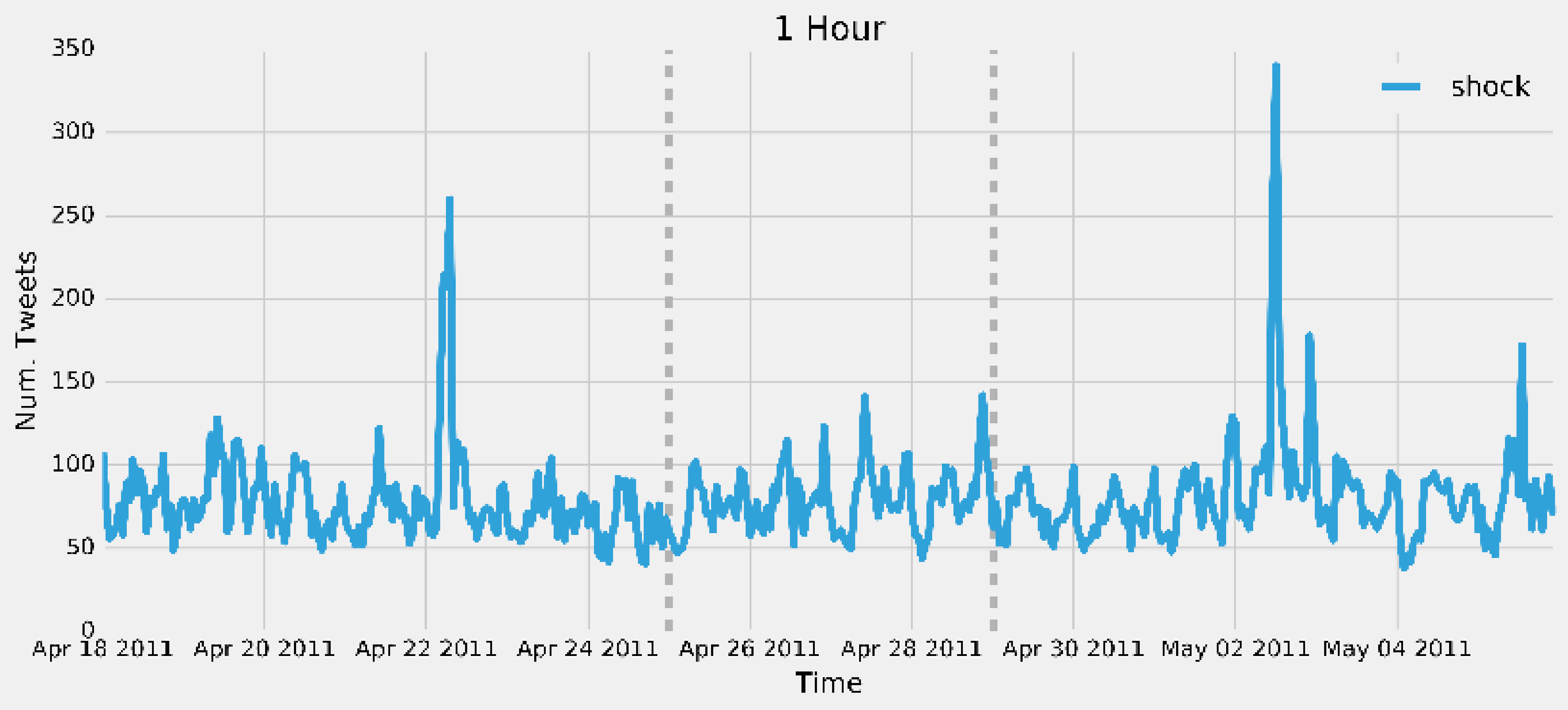

1 Hour
Num. Tweets
Time
shock
0
50
100
150
200
250
300
350
Apr 18 2011
Apr 20 2011
Apr 22 2011
Apr 24 2011
Apr 26 2011
Apr 28 2011
Apr 30 2011
May 02 2011
May 04 2011

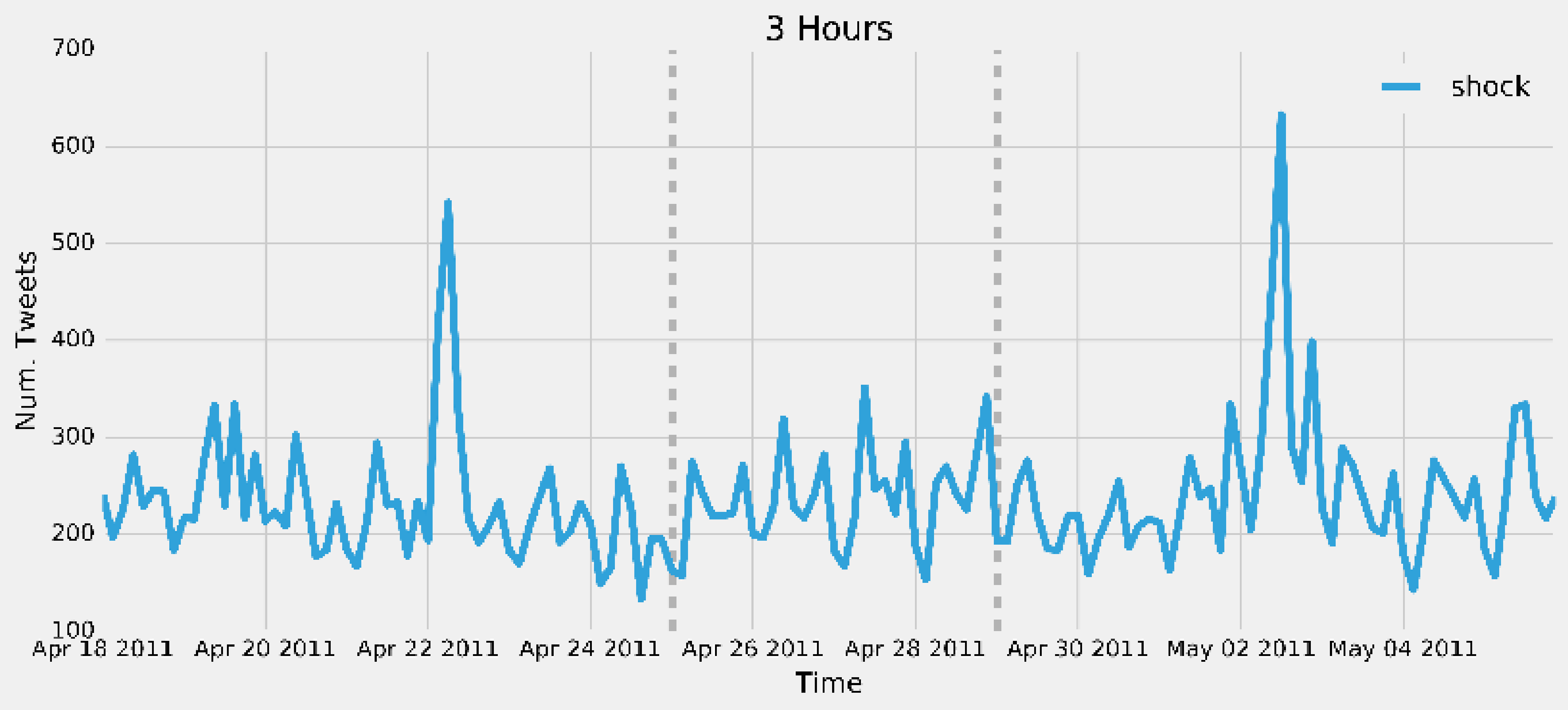

3 Hours
shock
Num. Tweets
100
200
300
400
500
600
700
Apr 18 2011
Apr 20 2011
Apr 22 2011
Apr 24 2011
Apr 26 2011
Apr 28 2011
Apr 30 2011
May 02 2011
May 04 2011
Time

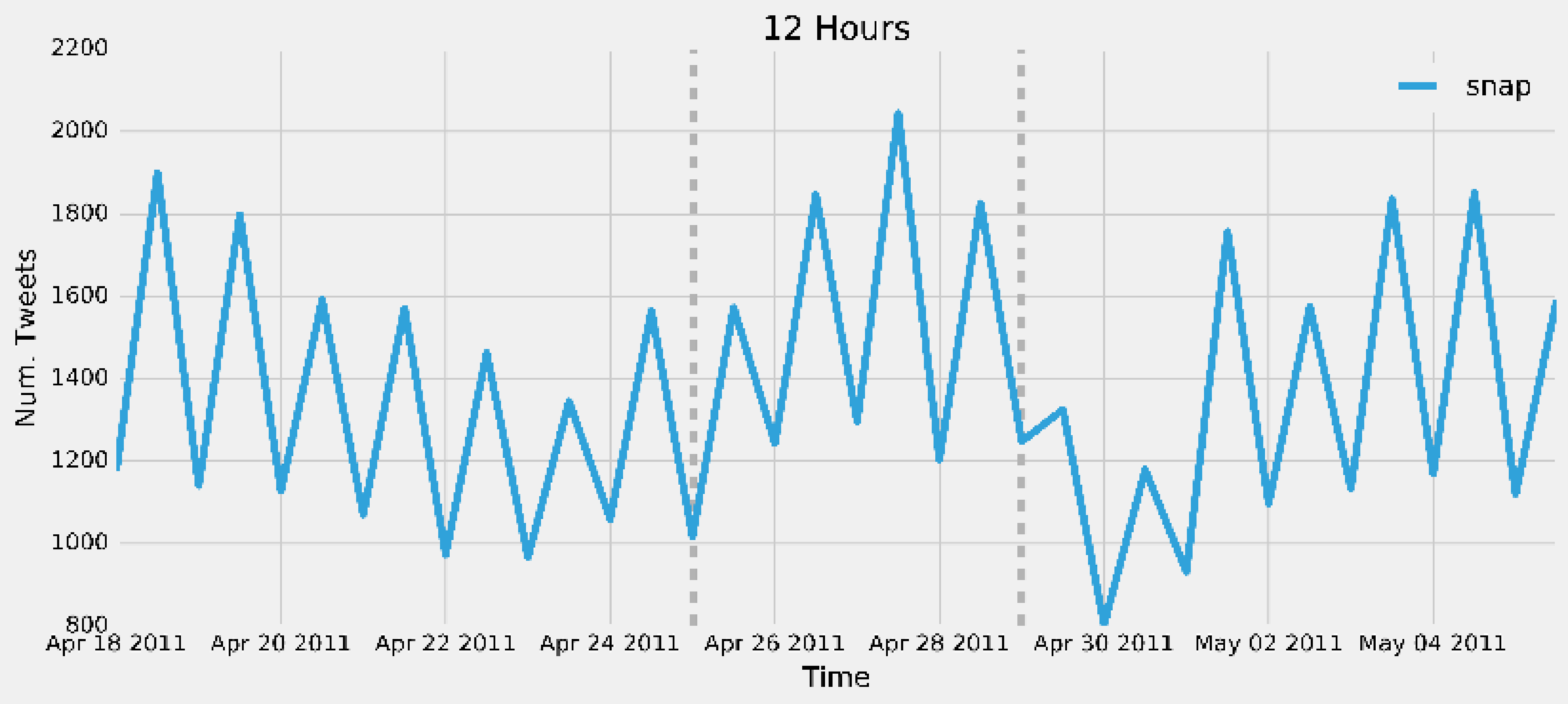
12 Hours
snap
2200
2000
1800
1600
1400
1200
1000
800
Num. Tweets
Apr 18 2011
Apr 20 2011
Apr 22 2011
Apr 24 2011
Apr 26 2011
Apr 28 2011
Apr 30 2011
May 02 2011
May 04 2011
Time

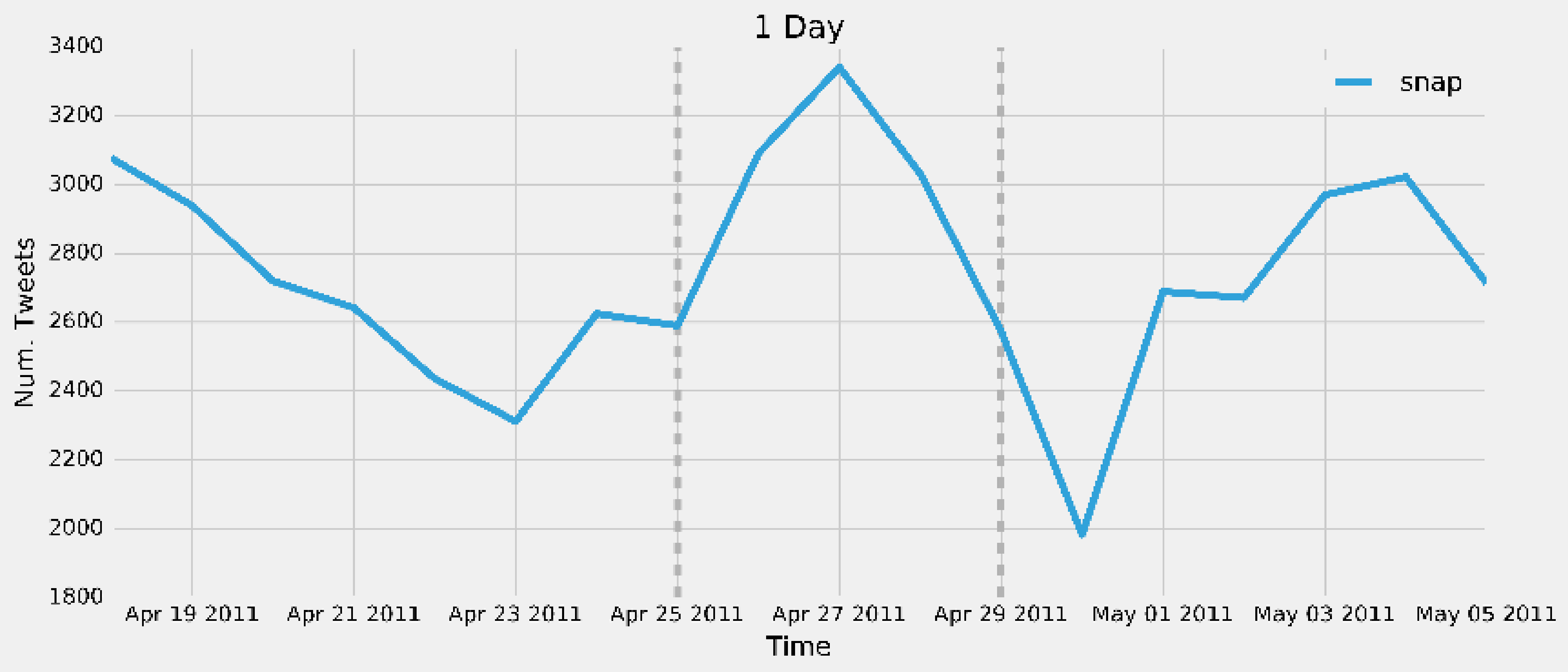

1 Day
snap
Num. Tweets
3400
3200
3000
2800
2600
2400
2200
2000
1800
Apr 19 2011
Apr 21 2011
Apr 23 2011
Apr 25 2011
Apr 27 2011
Apr 29 2011
May 01 2011
May 03 2011
May 05 2011
Time

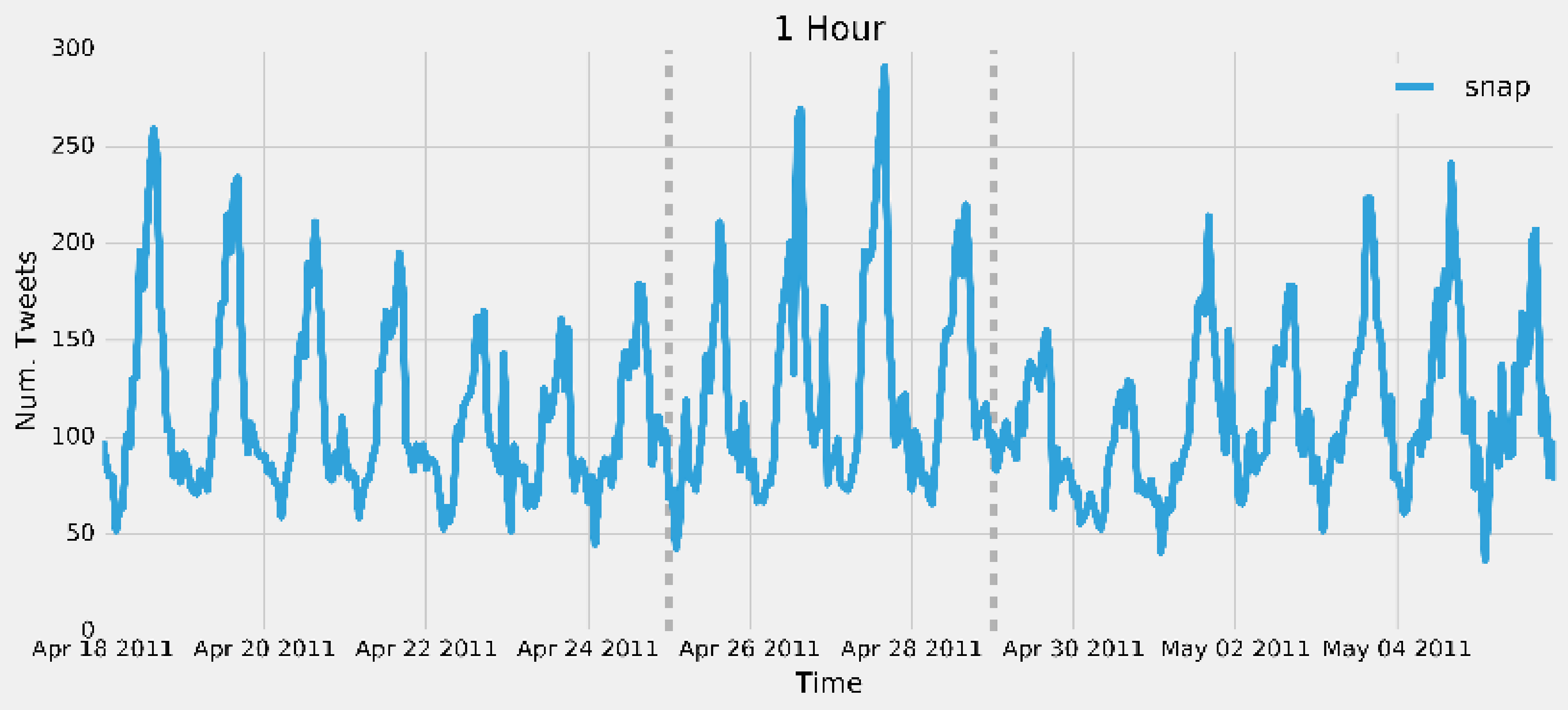
1 Hour
snap
Num. Tweets
Time
0
50
100
150
200
250
300
Apr 18 2011
Apr 20 2011
Apr 22 2011
Apr 24 2011
Apr 26 2011
Apr 28 2011
Apr 30 2011
May 02 2011
May 04 2011

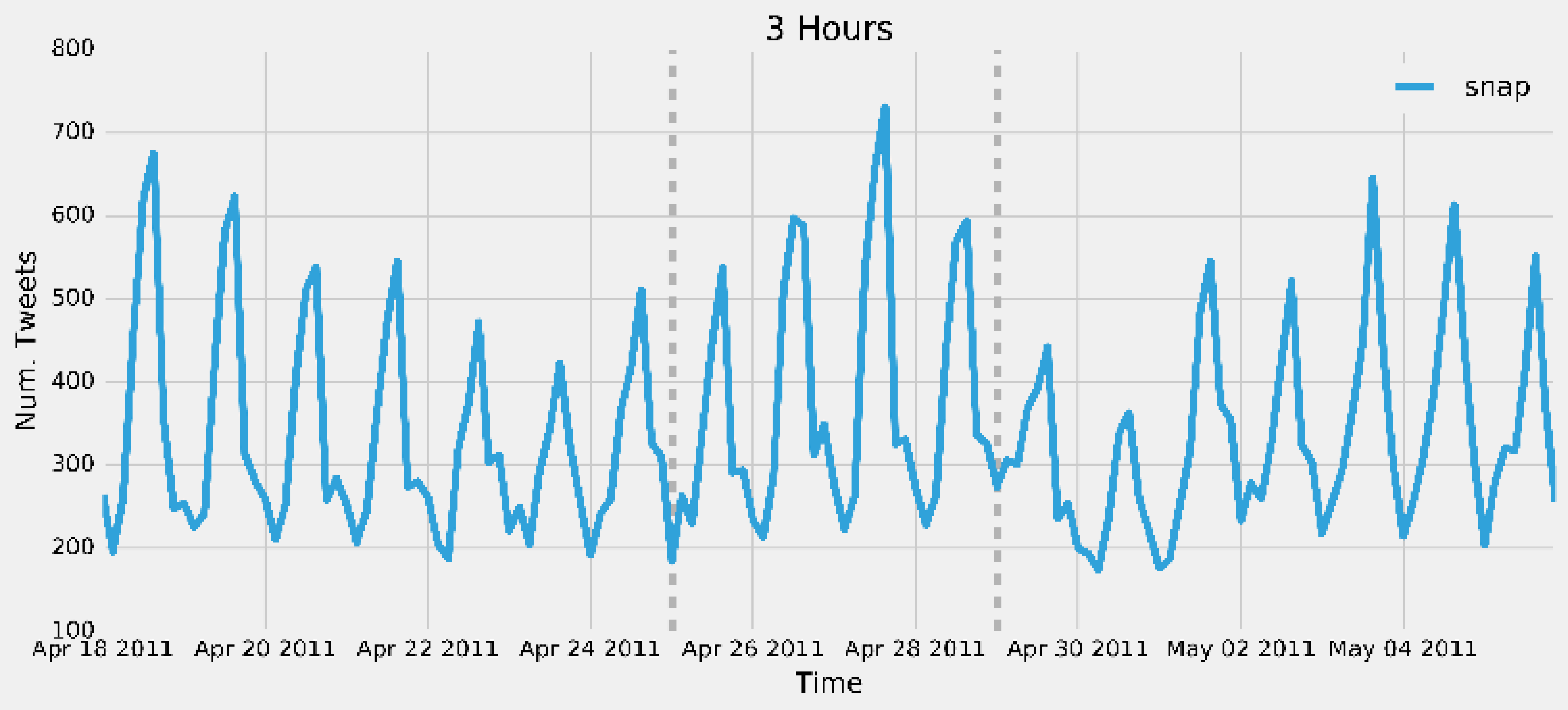

3 Hours
snap
Num. Tweets
100
200
300
400
500
600
700
800
Apr 18 2011
Apr 20 2011
Apr 22 2011
Apr 24 2011
Apr 26 2011
Apr 28 2011
Apr 30 2011
May 02 2011
May 04 2011
Time

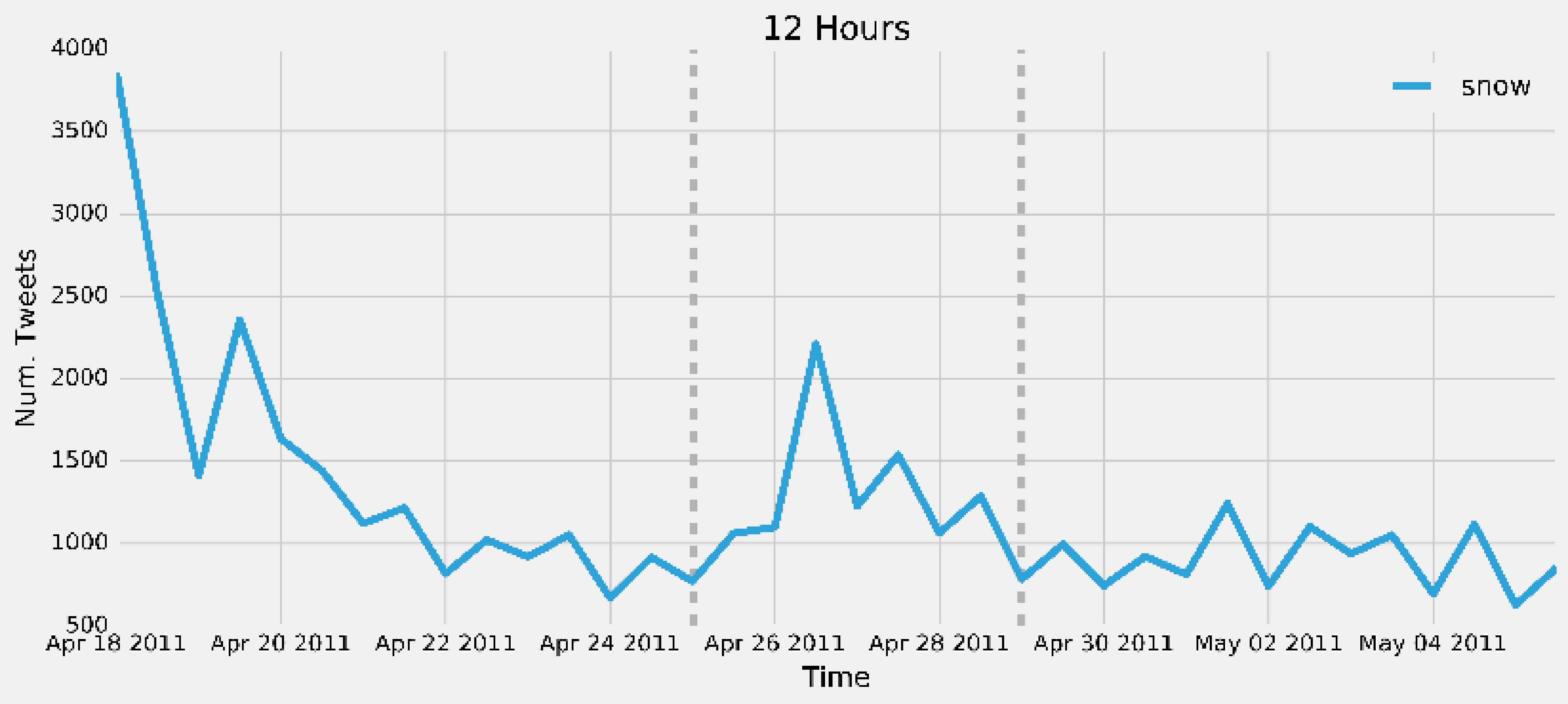

12 Hours
snow
4000
3500
3000
2500
2000
1500
1000
500
Num. Tweets
Apr 18 2011
Apr 20 2011
Apr 22 2011
Apr 24 2011
Apr 26 2011
Apr 28 2011
Apr 30 2011
May 02 2011
May 04 2011
Time

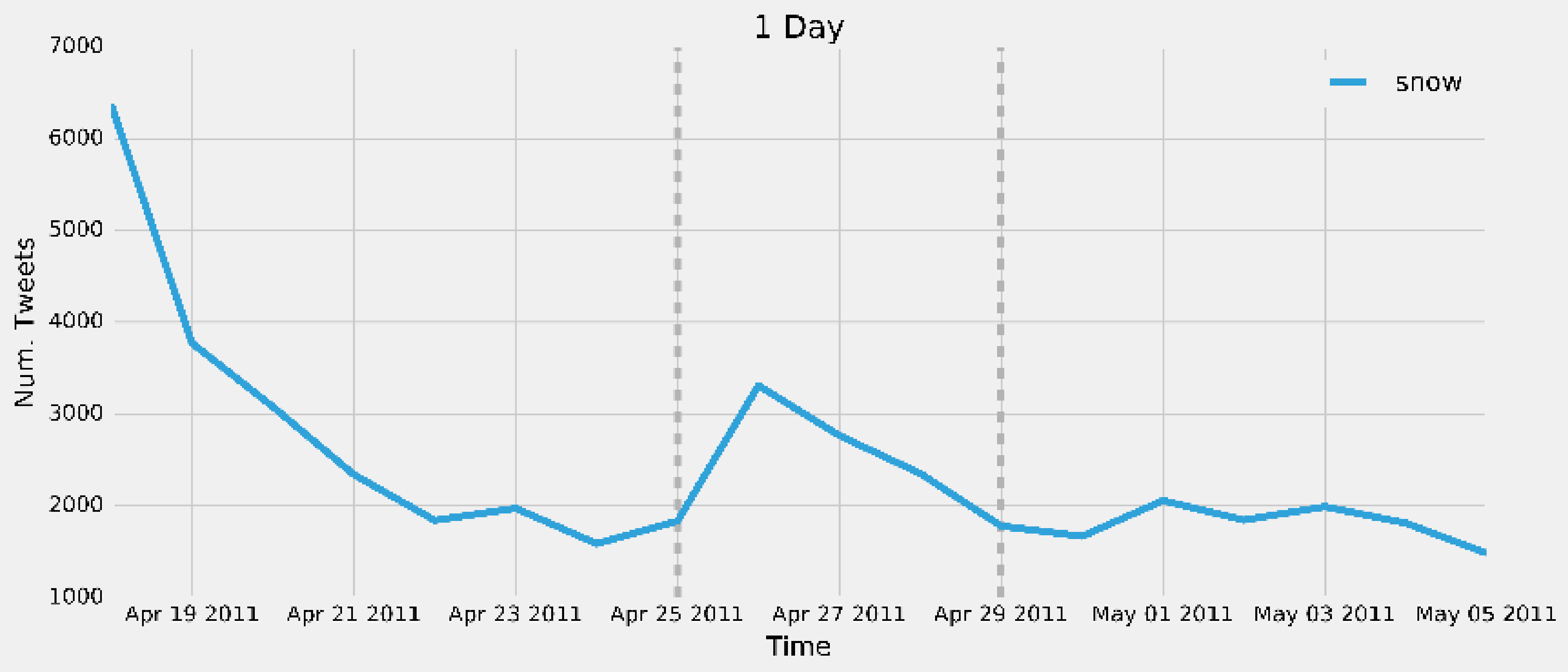
1 Day
snow
Num. Tweets
7000
6000
5000
4000
3000
2000
1000
Apr 19 2011
Apr 21 2011
Apr 23 2011
Apr 25 2011
Apr 27 2011
Apr 29 2011
May 01 2011
May 03 2011
May 05 2011
Time

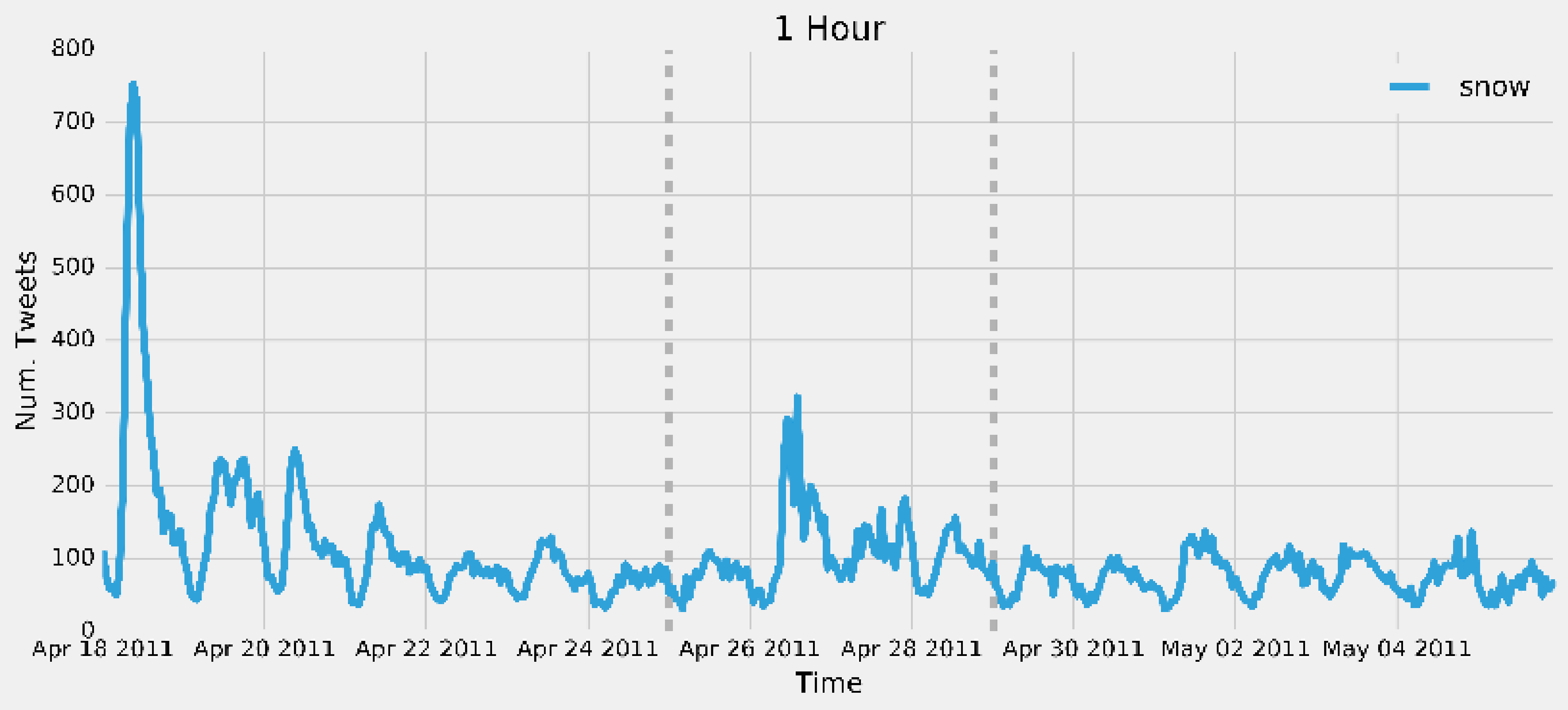

1 Hour
Num. Tweets
0
100
200
300
400
500
600
700
800
snow
Apr 18 2011
Apr 20 2011
Apr 22 2011
Apr 24 2011
Apr 26 2011
Apr 28 2011
Apr 30 2011
May 02 2011
May 04 2011
Time

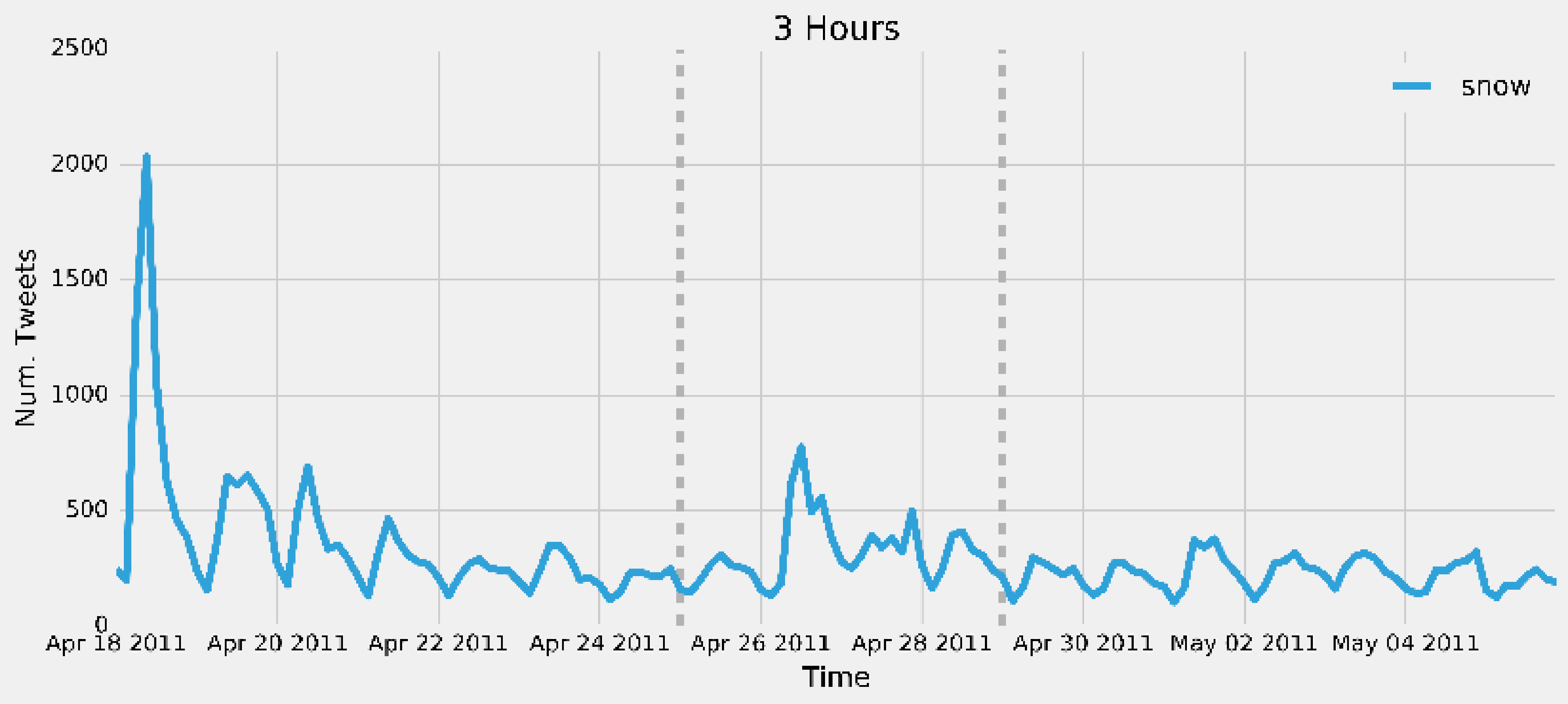

3 Hours
snow
Num. Tweets
0
500
1000
1500
2000
2500
Apr 18 2011
Apr 20 2011
Apr 22 2011
Apr 24 2011
Apr 26 2011
Apr 28 2011
Apr 30 2011
May 02 2011
May 04 2011
Time

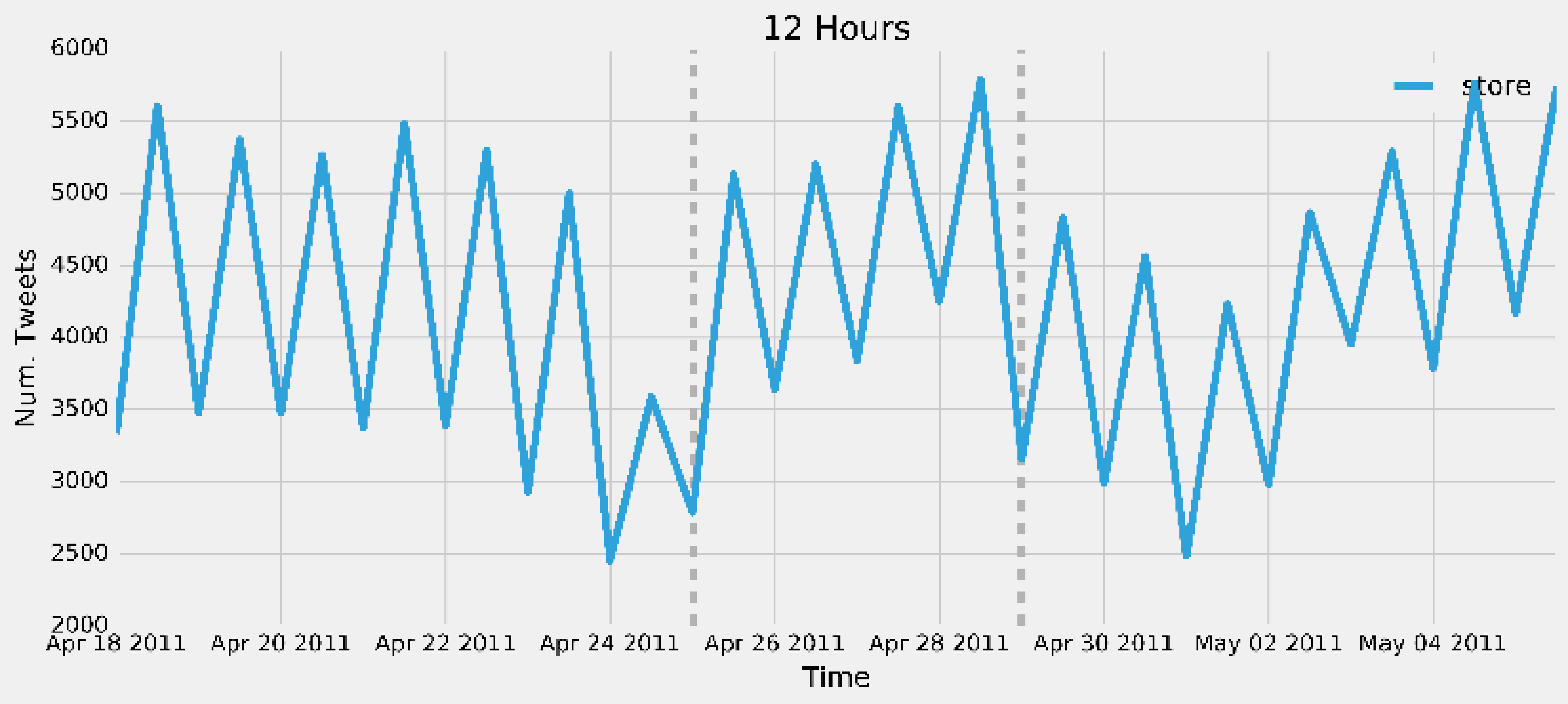
12 Hours
store
Num. Tweets
6000
5500
5000
4500
4000
3500
3000
2500
2000
Apr 18 2011
Apr 20 2011
Apr 22 2011
Apr 24 2011
Apr 26 2011
Apr 28 2011
Apr 30 2011
May 02 2011
May 04 2011
Time

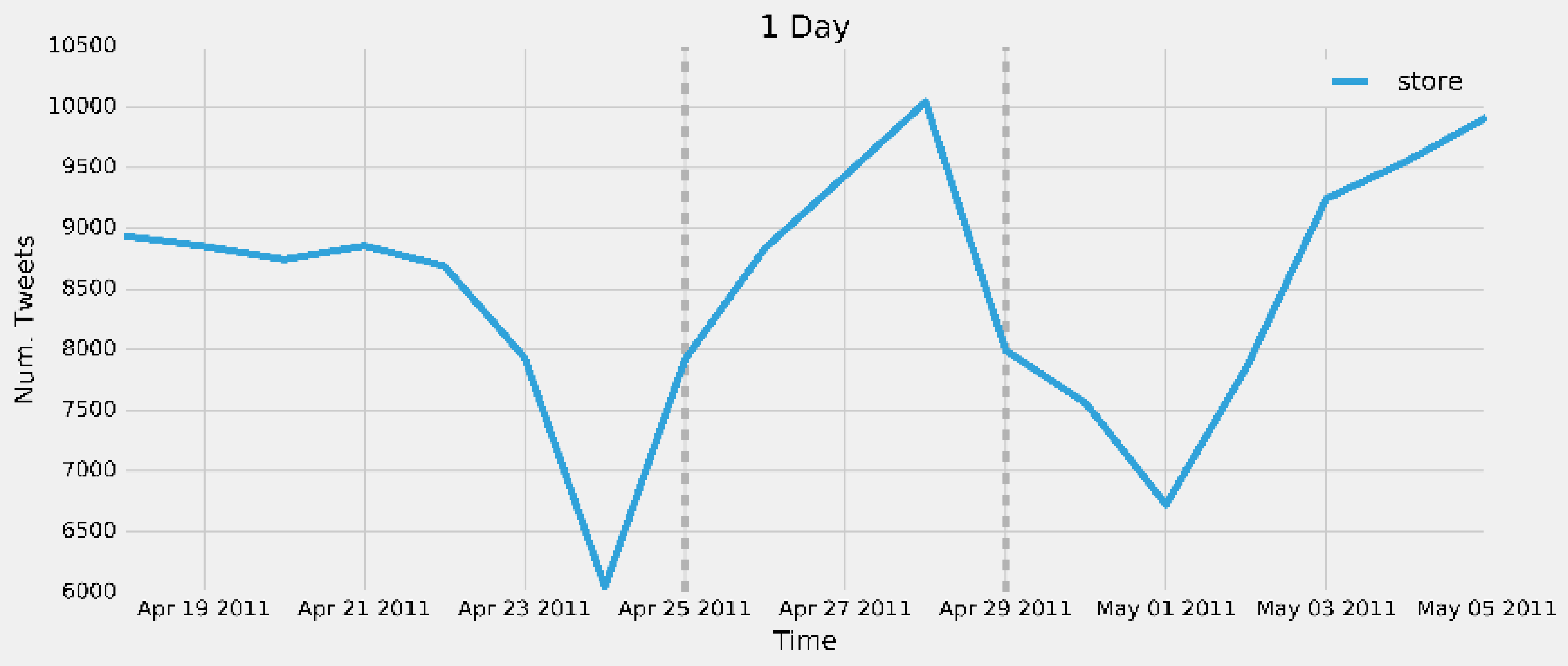

1 Day
store
Num. Tweets
10500
10000
9500
9000
8500
8000
7500
7000
6500
6000
Apr 19 2011
Apr 21 2011
Apr 23 2011
Apr 25 2011
Apr 27 2011
Apr 29 2011
May 01 2011
May 03 2011
May 05 2011
Time

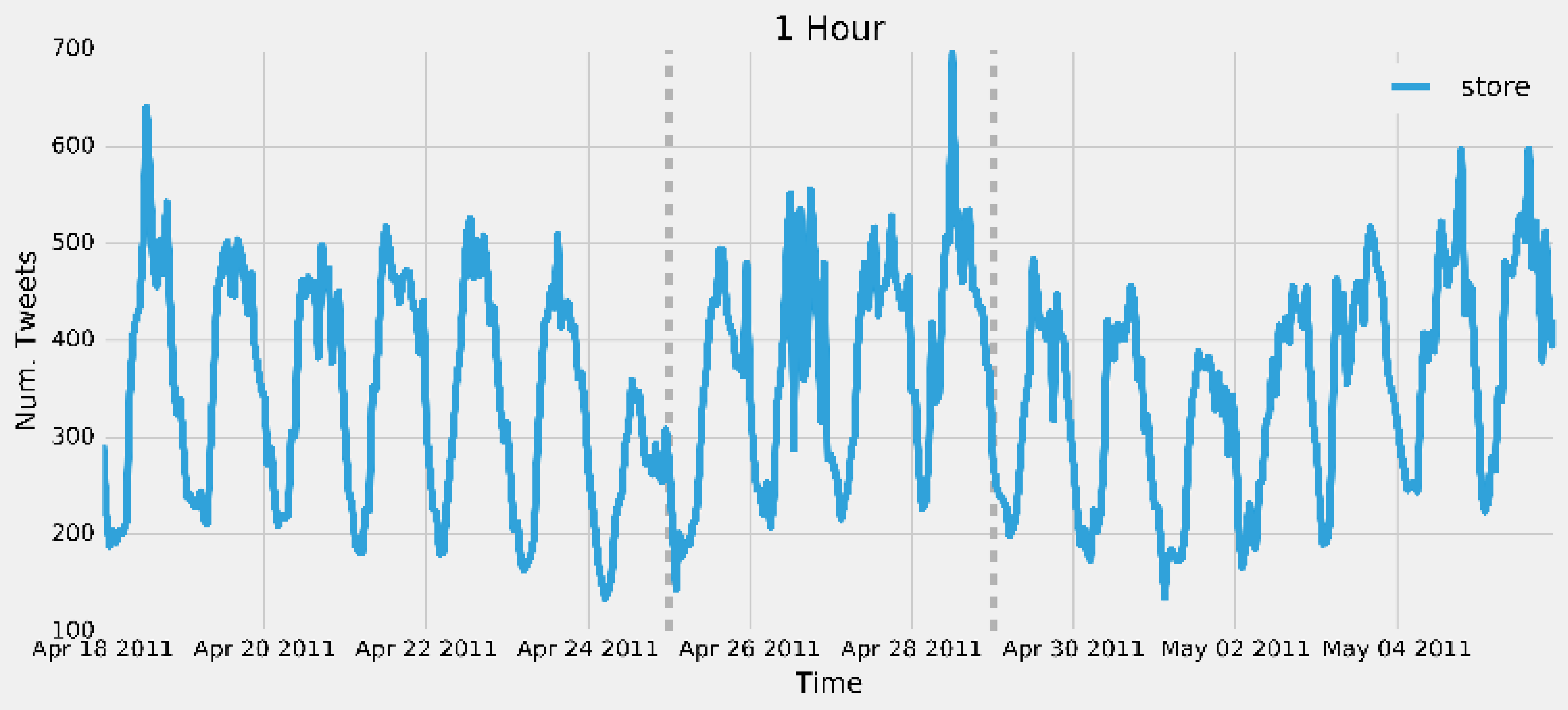

1 Hour
store
Num. Tweets
700
600
500
400
300
200
100
Apr 18 2011
Apr 20 2011
Apr 22 2011
Apr 24 2011
Apr 26 2011
Apr 28 2011
Apr 30 2011
May 02 2011
May 04 2011
Time

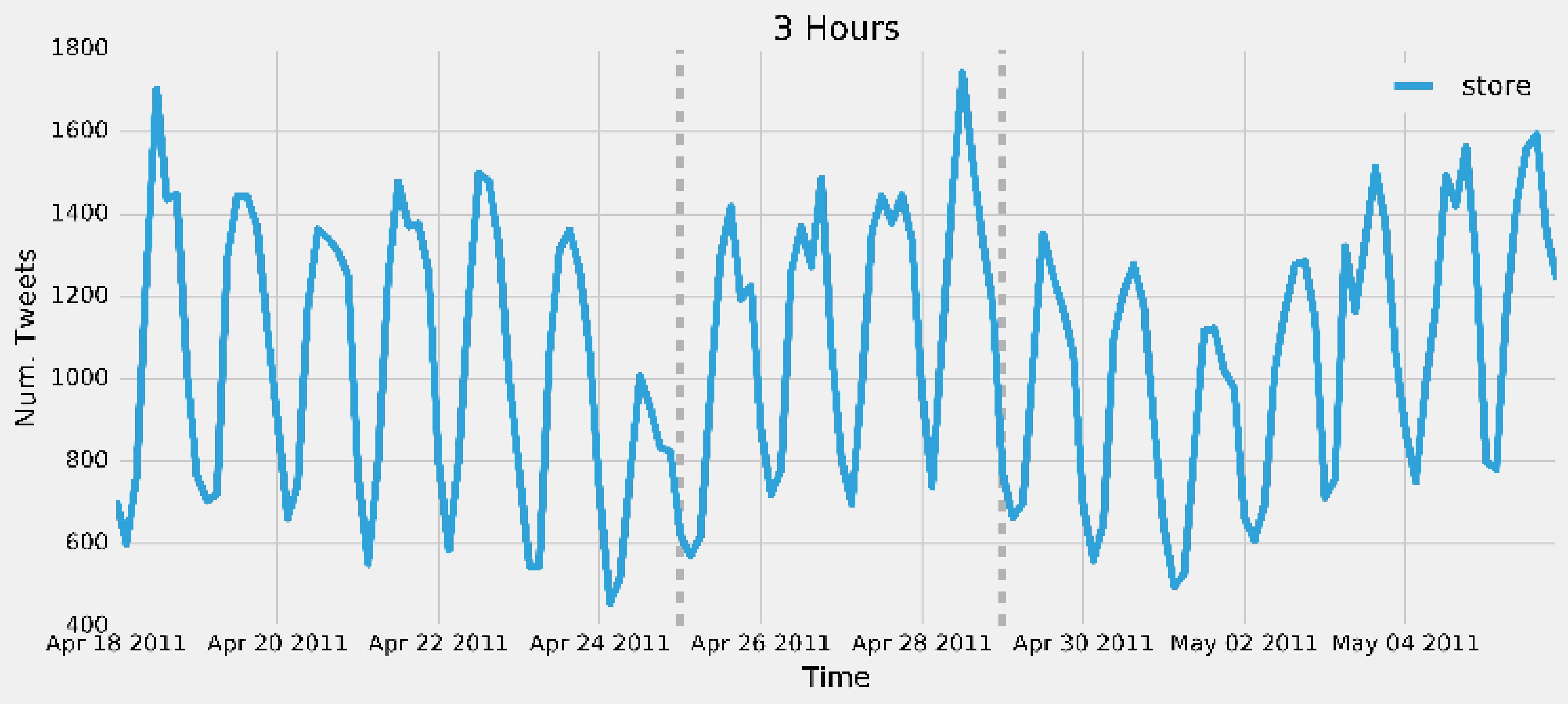
3 Hours
store
Num. Tweets
1800
1600
1400
1200
1000
800
600
400
Apr 18 2011
Apr 20 2011
Apr 22 2011
Apr 24 2011
Apr 26 2011
Apr 28 2011
Apr 30 2011
May 02 2011
May 04 2011
Time

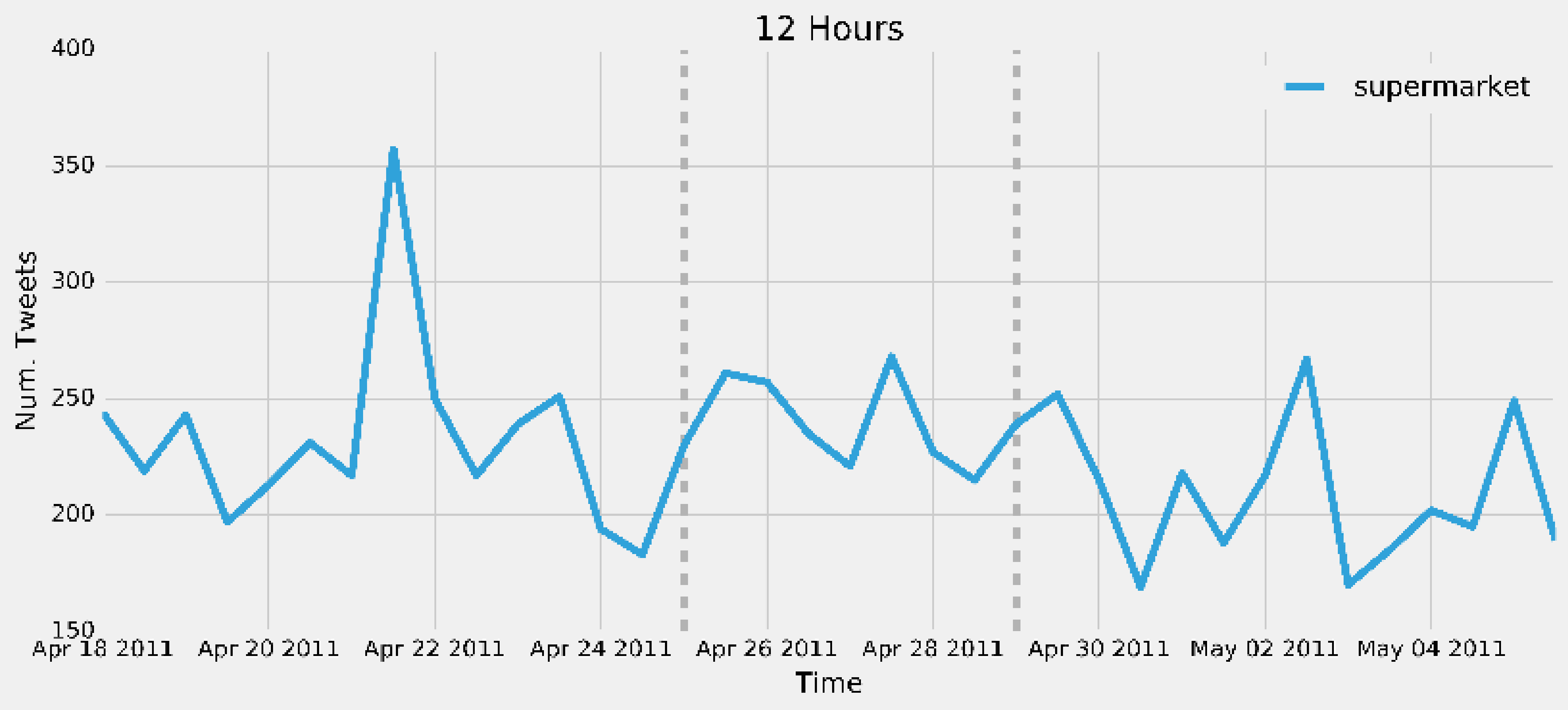
12 Hours
supermarket
Num. Tweets
Time
400
350
300
250
200
150
Apr 18 2011
Apr 20 2011
Apr 22 2011
Apr 24 2011
Apr 26 2011
Apr 28 2011
Apr 30 2011
May 02 2011
May 04 2011

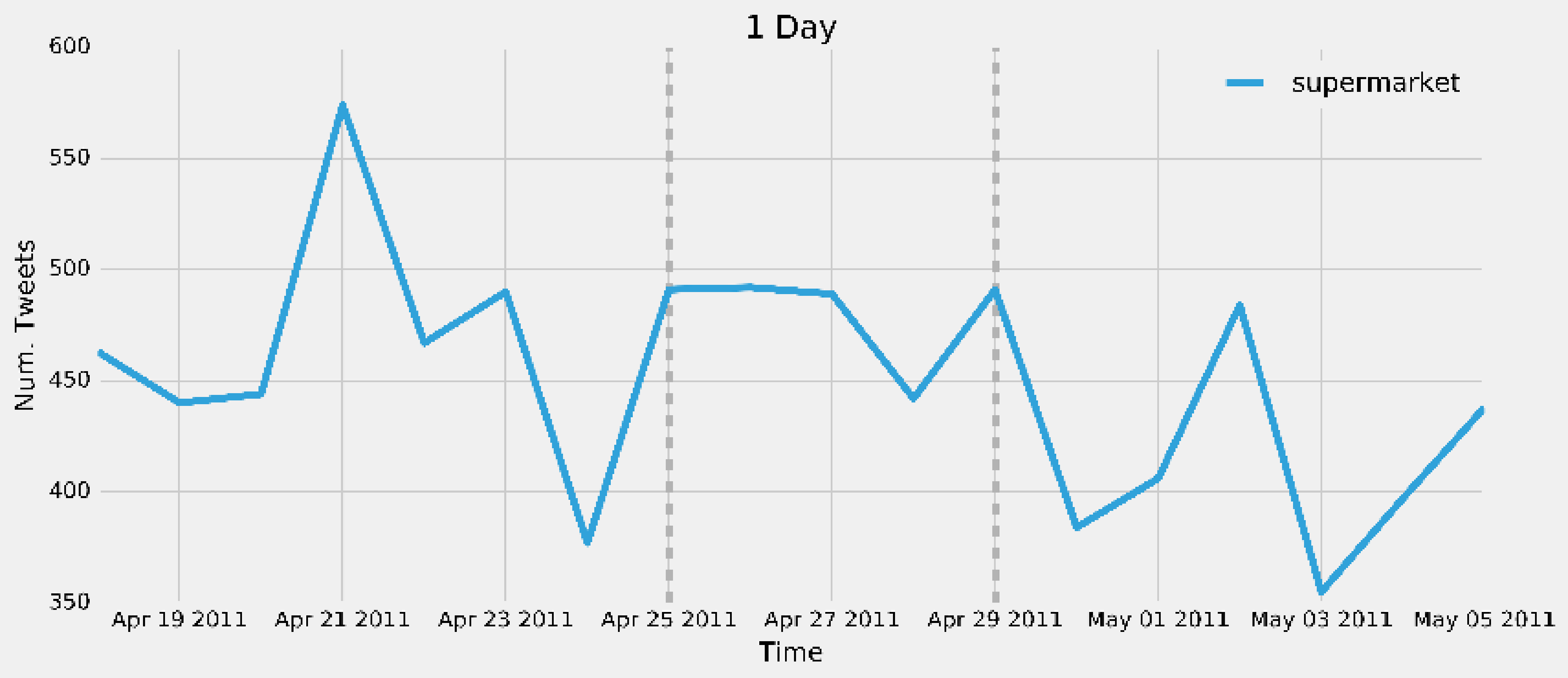
1 Day
supermarket
Num. Tweets
600
550
500
450
400
350
Apr 19 2011
Apr 21 2011
Apr 23 2011
Apr 25 2011
Apr 27 2011
Apr 29 2011
May 01 2011
May 03 2011
May 05 2011
Time

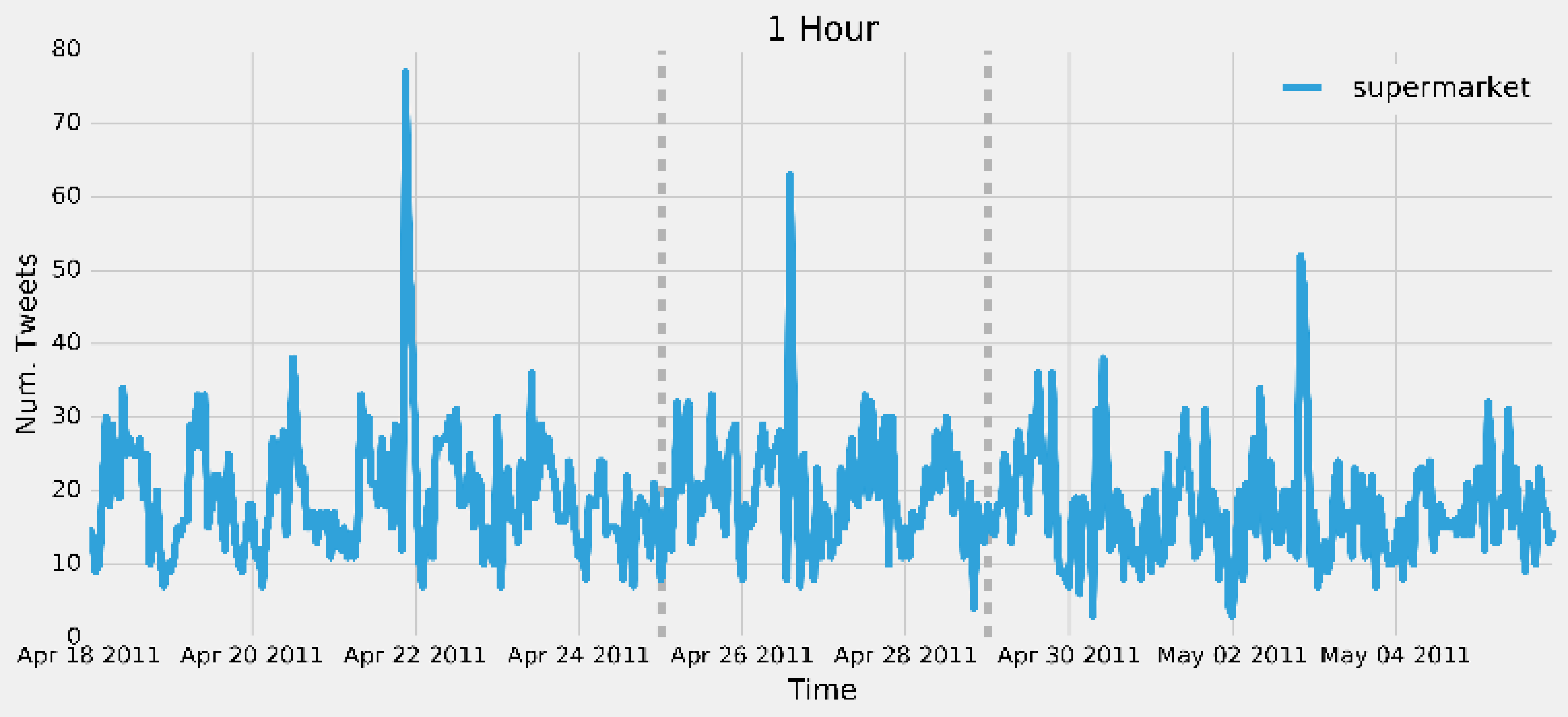
1 Hour
supermarket
Num. Tweets
0
10
20
30
40
50
60
70
80
Apr 18 2011
Apr 20 2011
Apr 22 2011
Apr 24 2011
Apr 26 2011
Apr 28 2011
Apr 30 2011
May 02 2011
May 04 2011
Time

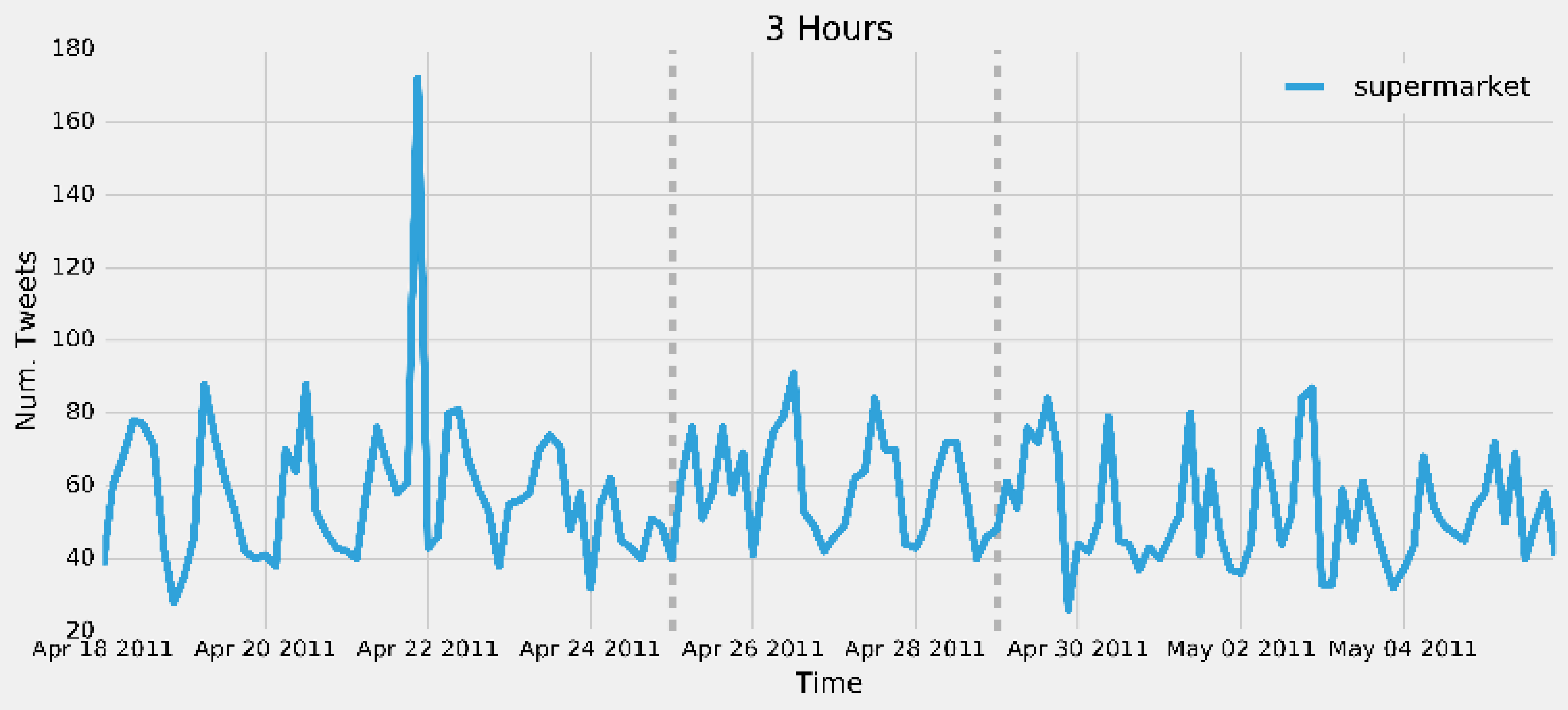

3 Hours
supermarket
Num. Tweets
180
160
140
120
100
80
60
40
20
Apr 18 2011
Apr 20 2011
Apr 22 2011
Apr 24 2011
Apr 26 2011
Apr 28 2011
Apr 30 2011
May 02 2011
May 04 2011
Time

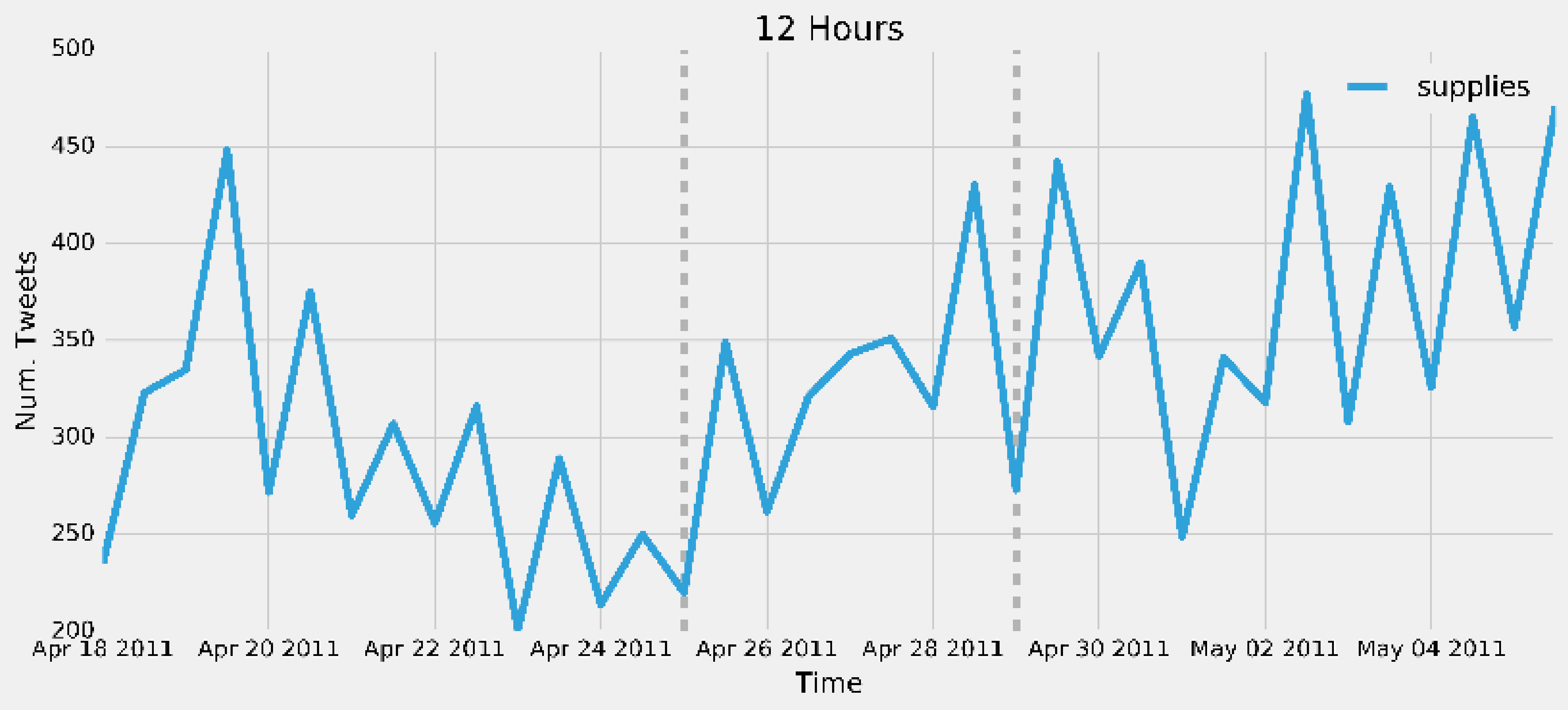
12 Hours
supplies
Num. Tweets
500
450
400
350
300
250
200
Apr 18 2011
Apr 20 2011
Apr 22 2011
Apr 24 2011
Apr 26 2011
Apr 28 2011
Apr 30 2011
May 02 2011
May 04 2011
Time

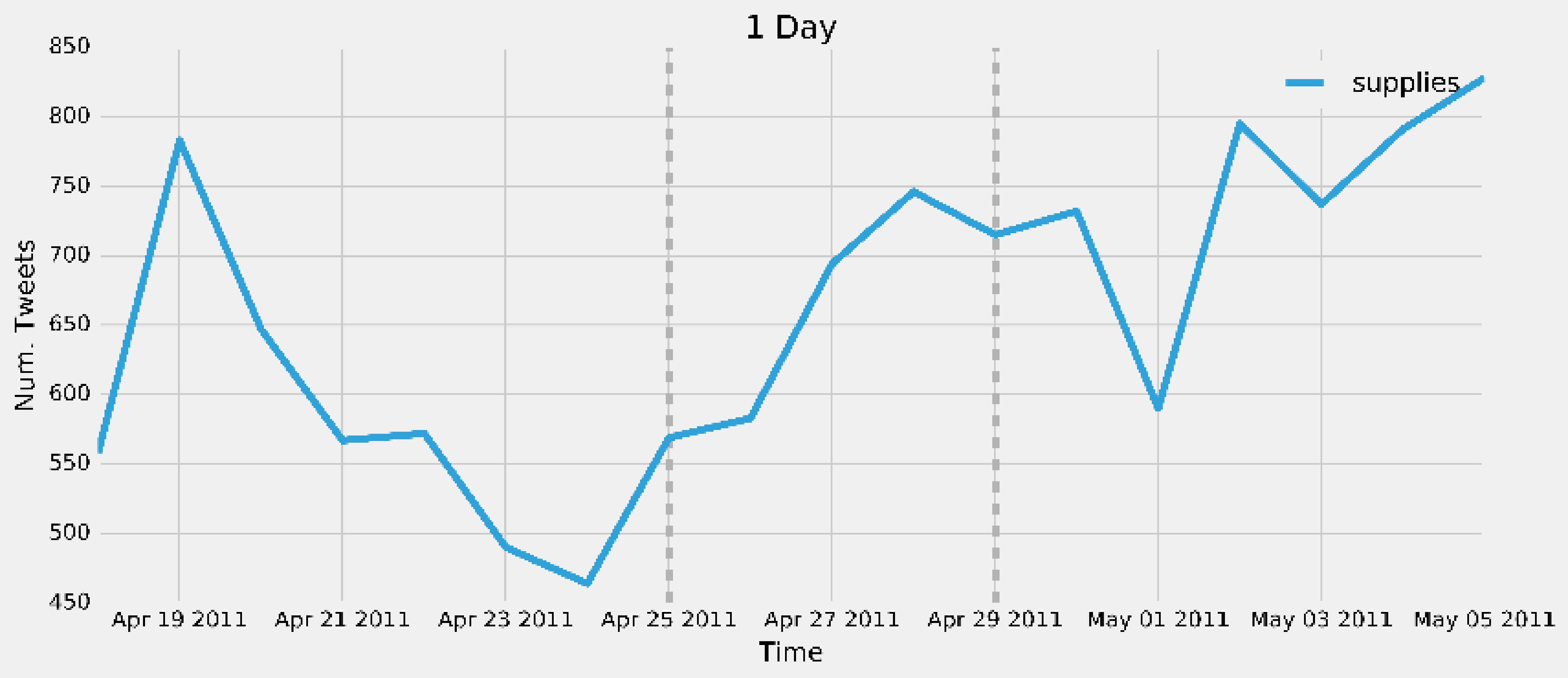
1 Day
850
800
750
700
650
600
550
500
450
Num. Tweets
Apr 19 2011
Apr 21 2011
Apr 23 2011
Apr 25 2011
Apr 27 2011
Apr 29 2011
May 01 2011
May 03 2011
May 05 2011
Time
supplies

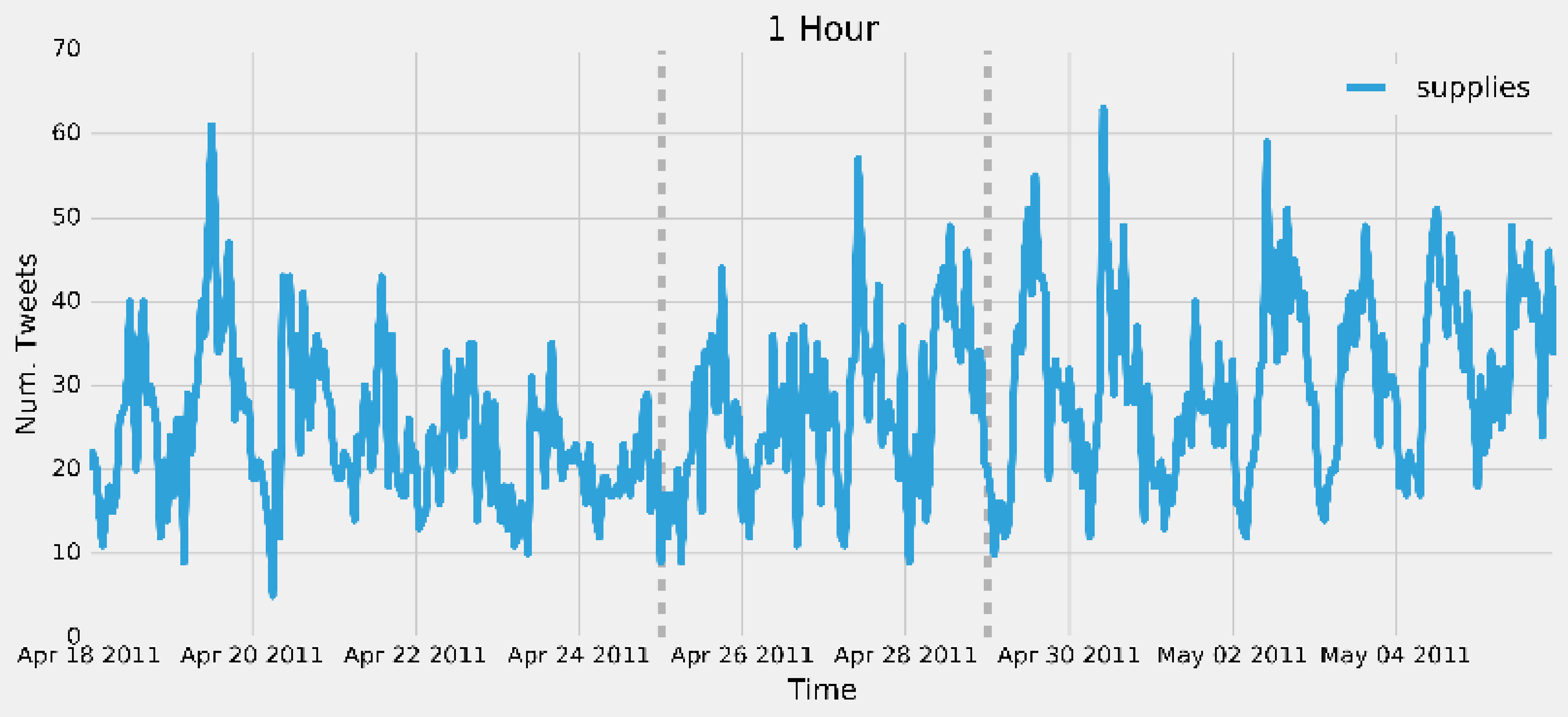
1 Hour
supplies
Num. Tweets
0
10
20
30
40
50
60
70
Apr 18 2011
Apr 20 2011
Apr 22 2011
Apr 24 2011
Apr 26 2011
Apr 28 2011
Apr 30 2011
May 02 2011
May 04 2011
Time

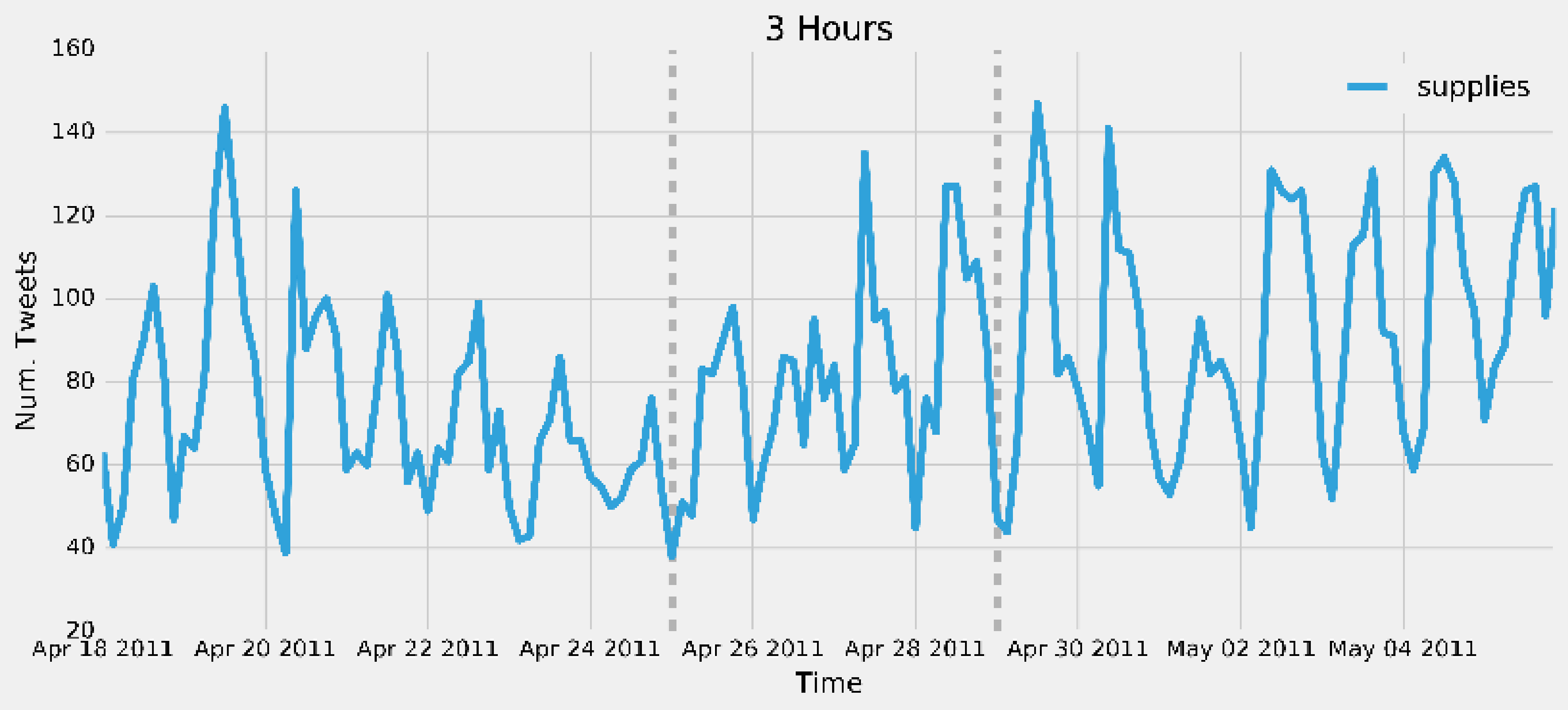

3 Hours
supplies
Num. Tweets
Time
160
140
120
100
80
60
40
20
Apr 18 2011
Apr 20 2011
Apr 22 2011
Apr 24 2011
Apr 26 2011
Apr 28 2011
Apr 30 2011
May 02 2011
May 04 2011

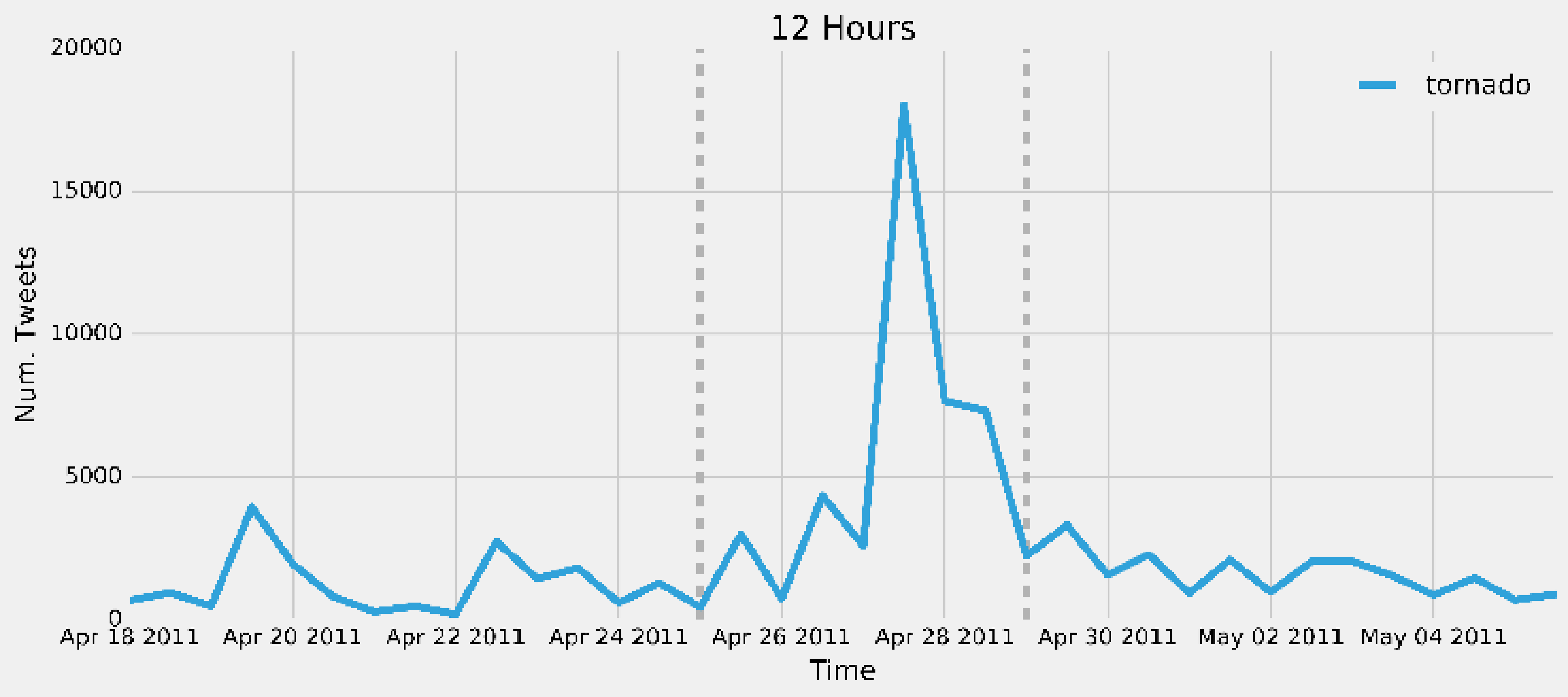
12 Hours
tornado
Num. Tweets
20000
15000
10000
5000
0
Apr 18 2011
Apr 20 2011
Apr 22 2011
Apr 24 2011
Apr 26 2011
Apr 28 2011
Apr 30 2011
May 02 2011
May 04 2011
Time

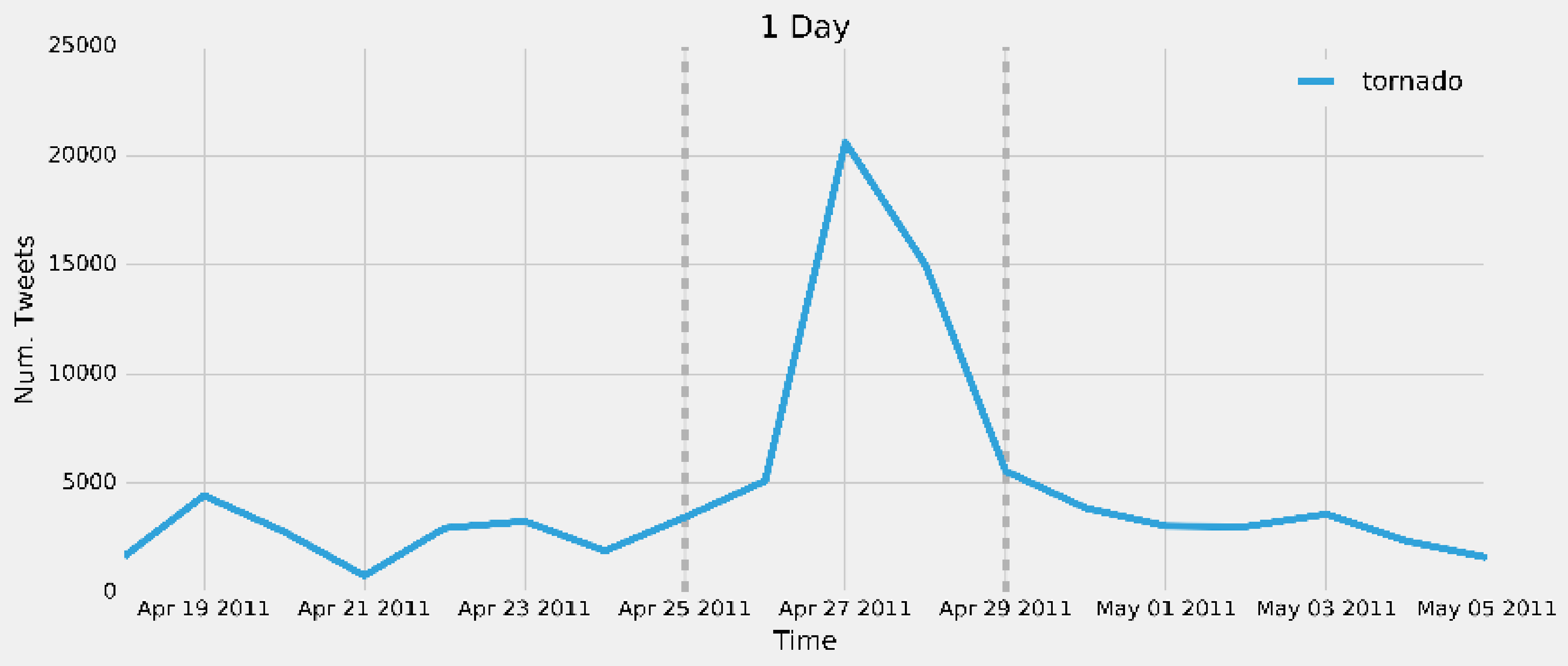
1 Day
tornado
Num. Tweets
0
5000
10000
15000
20000
25000
Apr 19 2011
Apr 21 2011
Apr 23 2011
Apr 25 2011
Apr 27 2011
Apr 29 2011
May 01 2011
May 03 2011
May 05 2011
Time

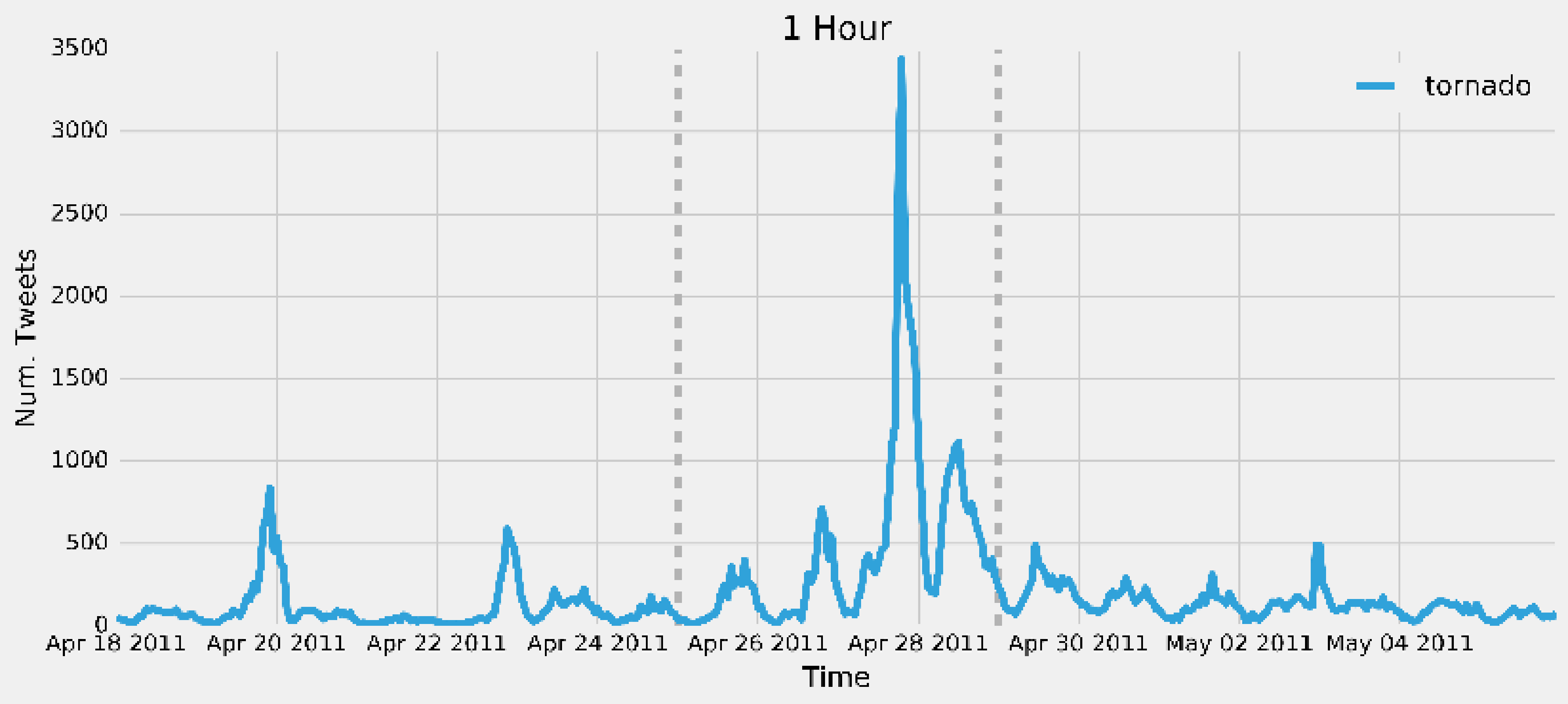
1 Hour
tornado
Num. Tweets
Time
0
500
1000
1500
2000
2500
3000
3500
Apr 18 2011
Apr 20 2011
Apr 22 2011
Apr 24 2011
Apr 26 2011
Apr 28 2011
Apr 30 2011
May 02 2011
May 04 2011

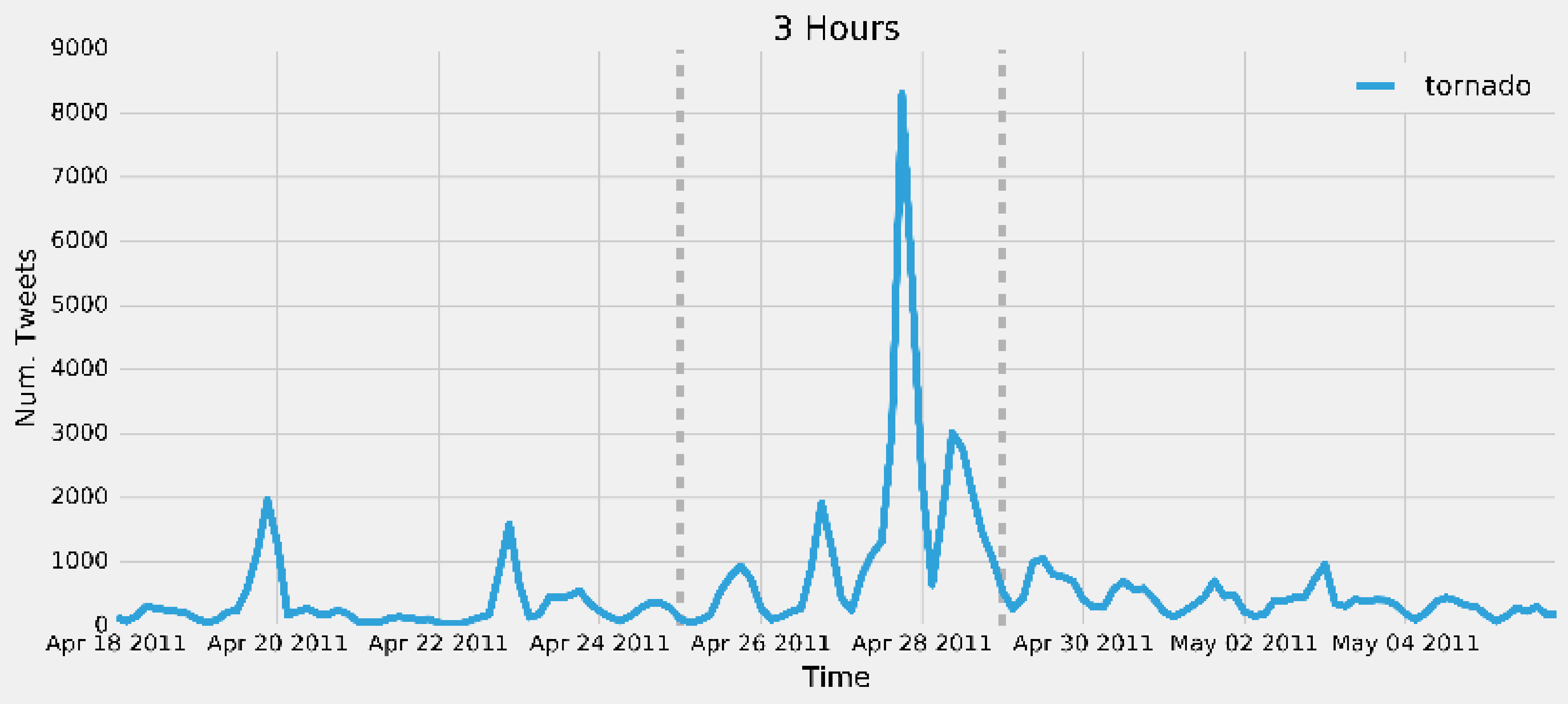
3 Hours
tornado
Num. Tweets
9000
8000
7000
6000
5000
4000
3000
2000
1000
0
Apr 18 2011
Apr 20 2011
Apr 22 2011
Apr 24 2011
Apr 26 2011
Apr 28 2011
Apr 30 2011
May 02 2011
May 04 2011
Time

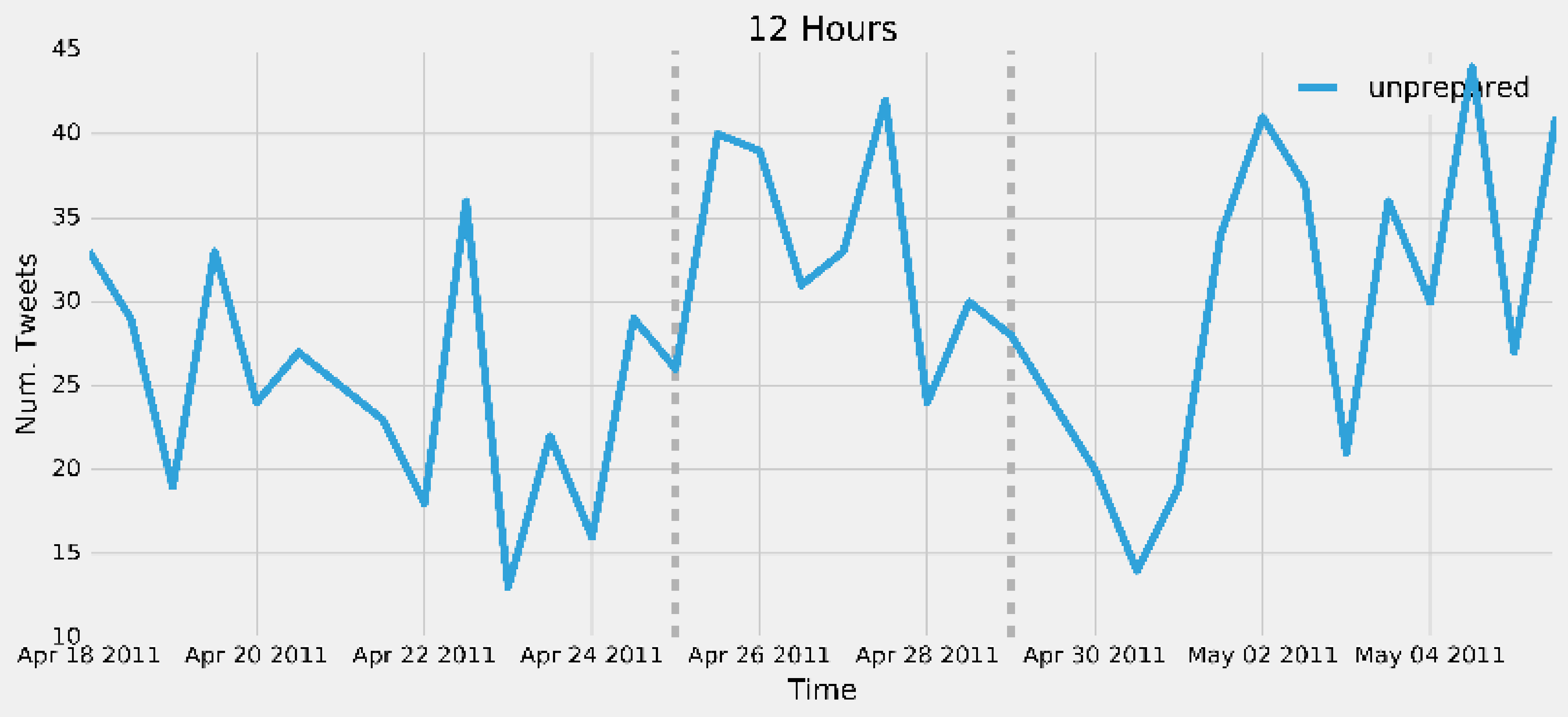
12 Hours
unprepared
Num. Tweets
Time
45
40
35
30
25
20
15
10
Apr 18 2011
Apr 20 2011
Apr 22 2011
Apr 24 2011
Apr 26 2011
Apr 28 2011
Apr 30 2011
May 02 2011
May 04 2011

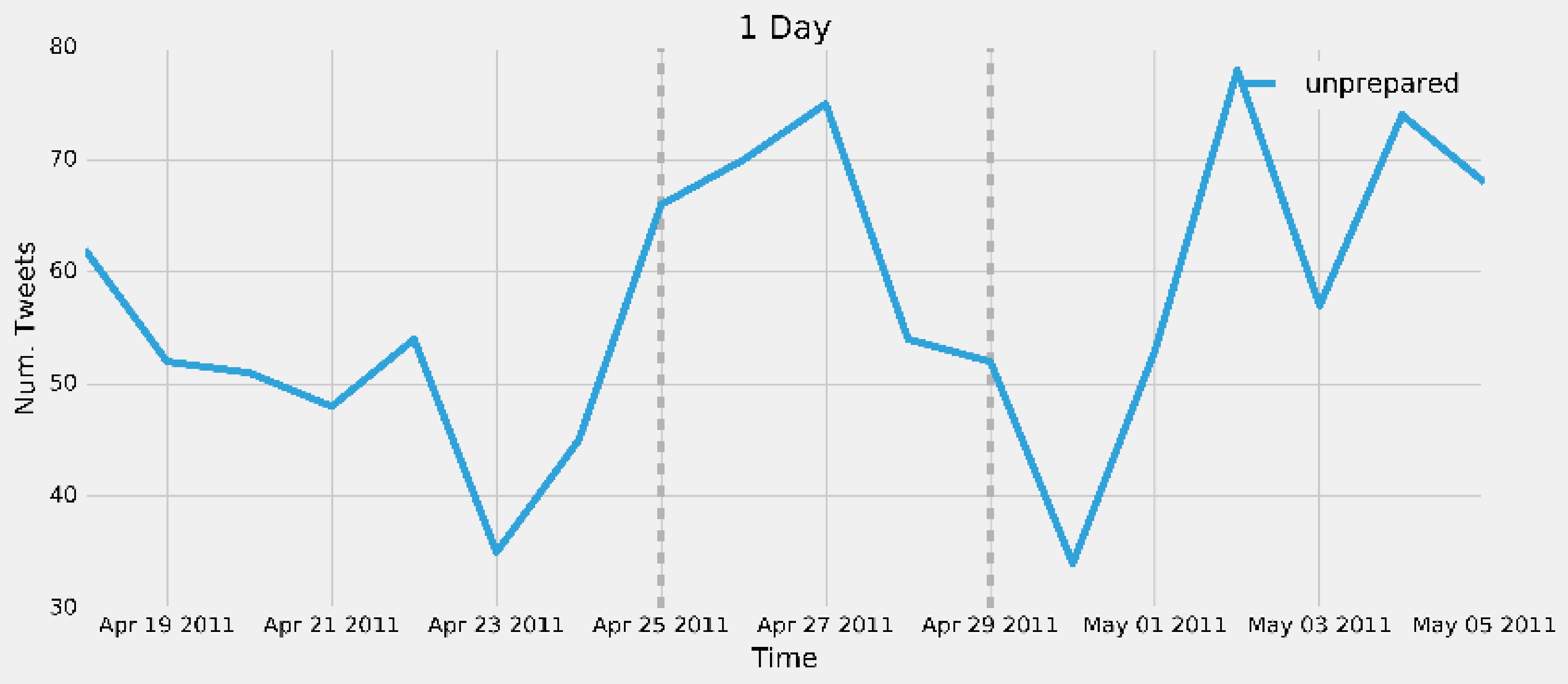

1 Day
unprepared
Num. Tweets
80
70
60
50
40
30
Apr 19 2011
Apr 21 2011
Apr 23 2011
Apr 25 2011
Apr 27 2011
Apr 29 2011
May 01 2011
May 03 2011
May 05 2011
Time

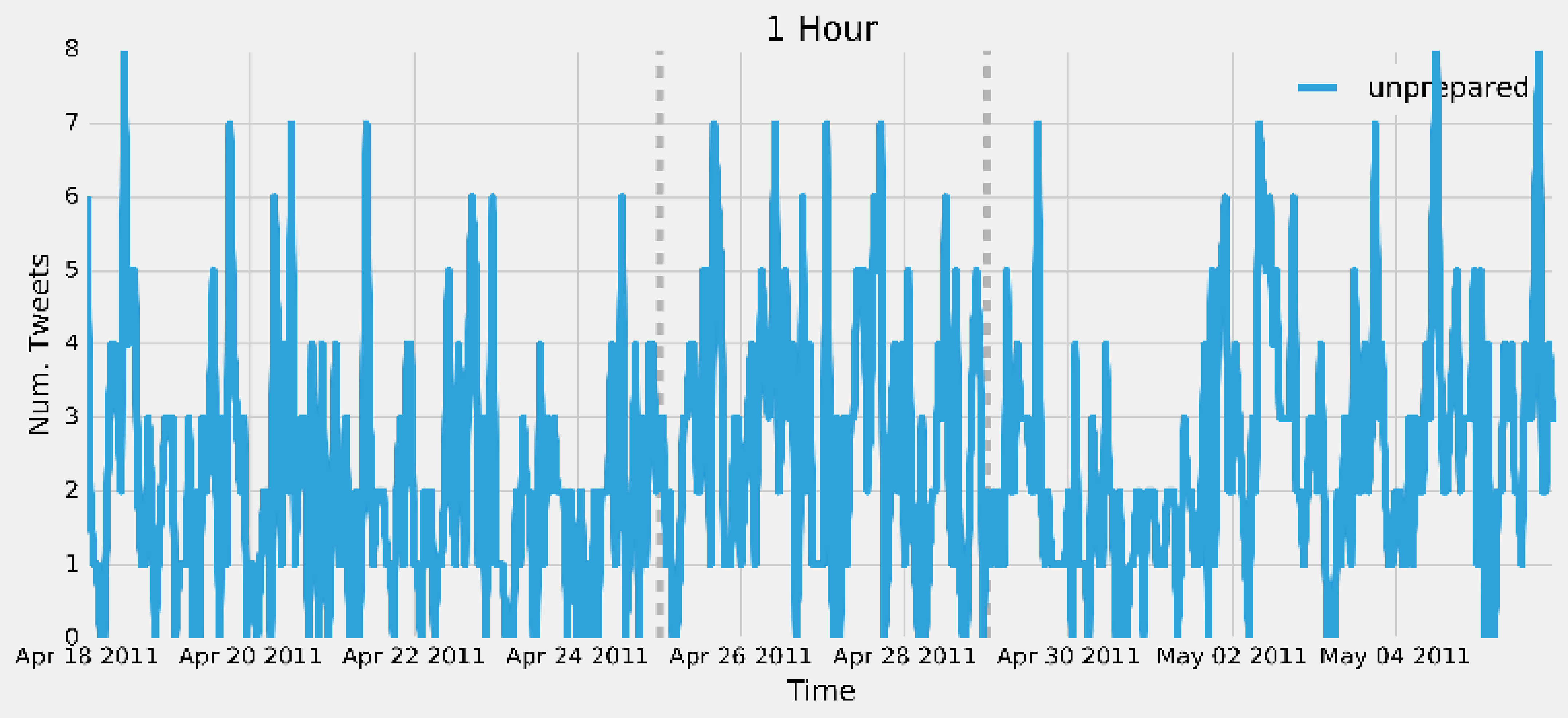

1 Hour
unprepared
Num. Tweets
Time
0
1
2
3
4
5
6
7
8
Apr 18 2011
Apr 20 2011
Apr 22 2011
Apr 24 2011
Apr 26 2011
Apr 28 2011
Apr 30 2011
May 02 2011
May 04 2011

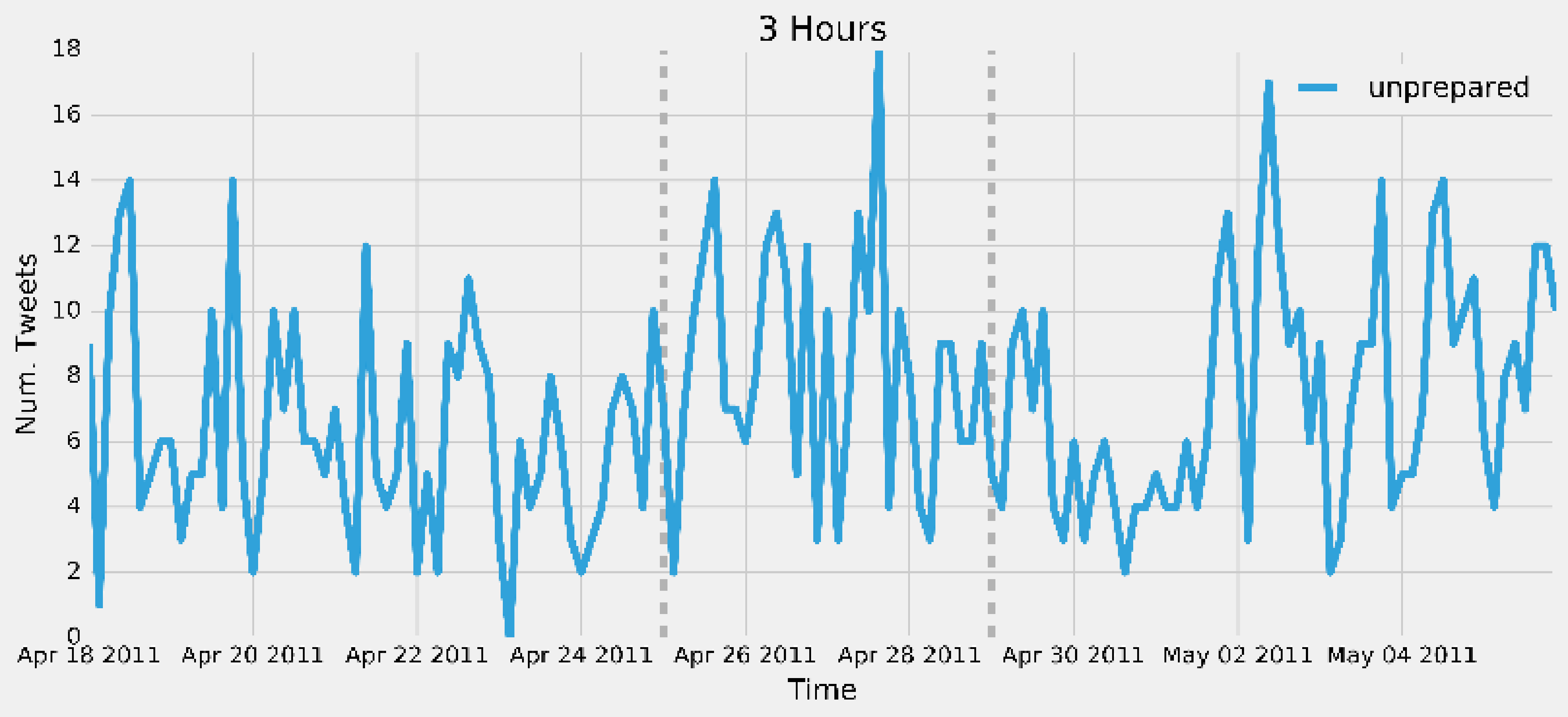
3 Hours
unprepared
Num. Tweets
Time
0
2
4
6
8
10
12
14
16
18
Apr 18 2011
Apr 20 2011
Apr 22 2011
Apr 24 2011
Apr 26 2011
Apr 28 2011
Apr 30 2011
May 02 2011
May 04 2011

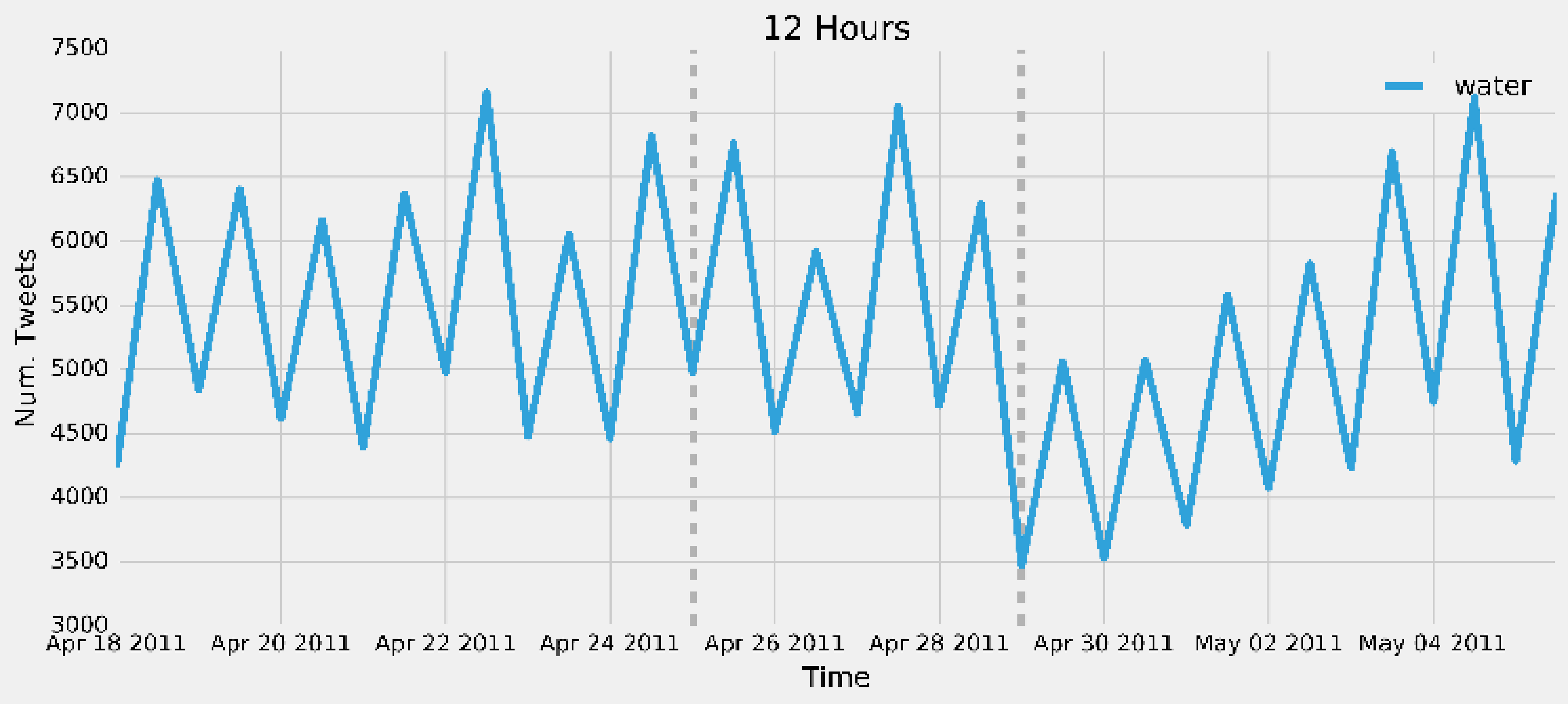

12 Hours
water
7500
7000
6500
6000
5500
5000
4500
4000
3500
3000
Num. Tweets
Apr 18 2011
Apr 20 2011
Apr 22 2011
Apr 24 2011
Apr 26 2011
Apr 28 2011
Apr 30 2011
May 02 2011
May 04 2011
Time

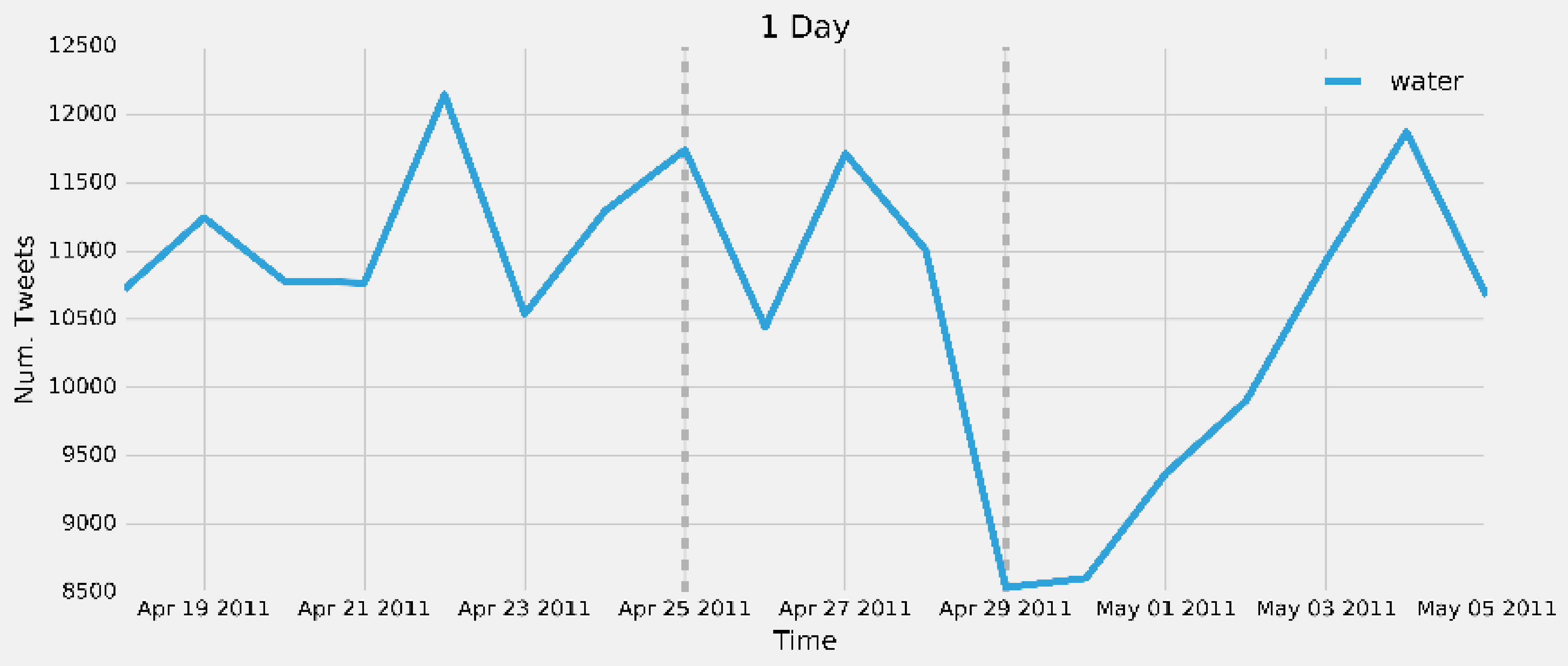

1 Day
water
12500
12000
11500
11000
10500
10000
9500
9000
8500
Num. Tweets
Apr 19 2011
Apr 21 2011
Apr 23 2011
Apr 25 2011
Apr 27 2011
Apr 29 2011
May 01 2011
May 03 2011
May 05 2011
Time

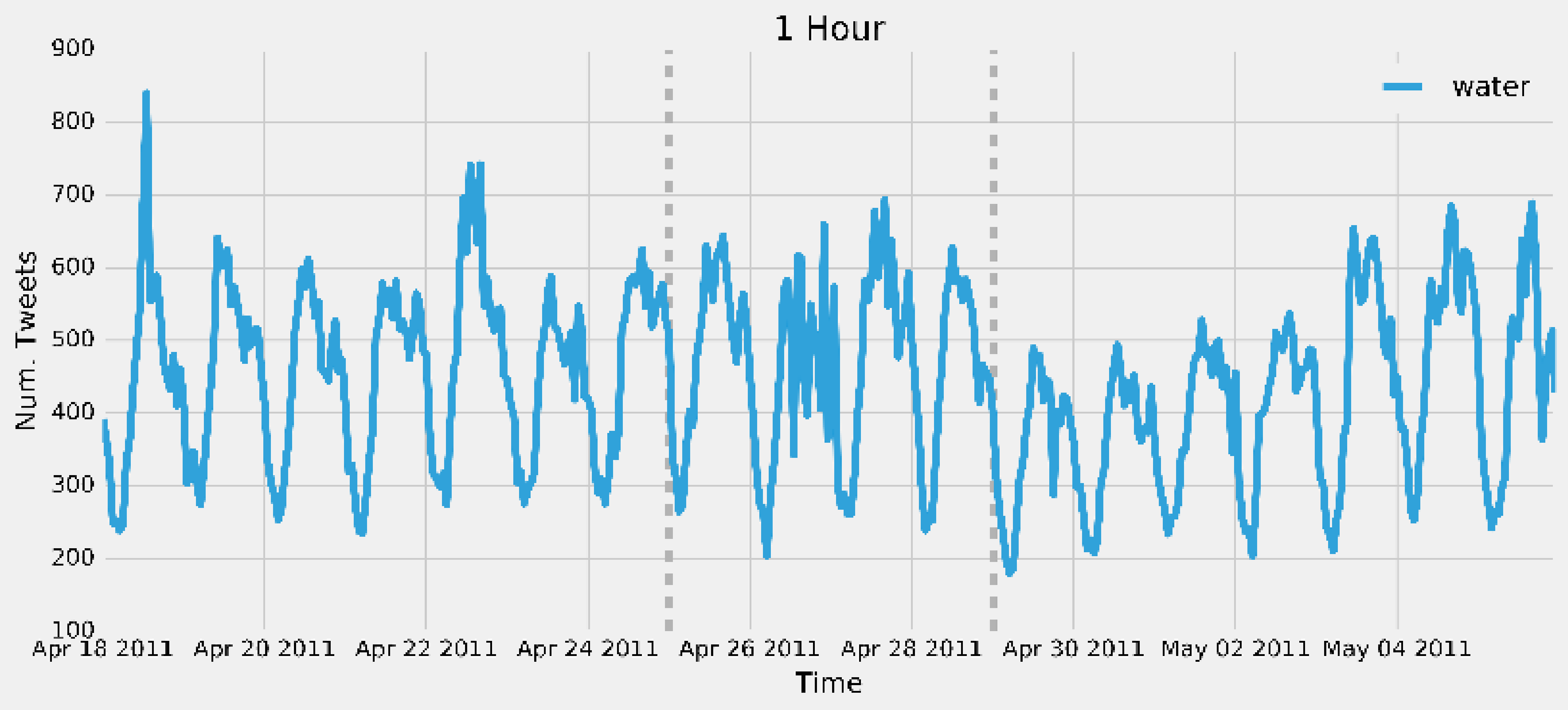
1 Hour
water
Num. Tweets
900
800
700
600
500
400
300
200
100
Apr 18 2011
Apr 20 2011
Apr 22 2011
Apr 24 2011
Apr 26 2011
Apr 28 2011
Apr 30 2011
May 02 2011
May 04 2011
Time

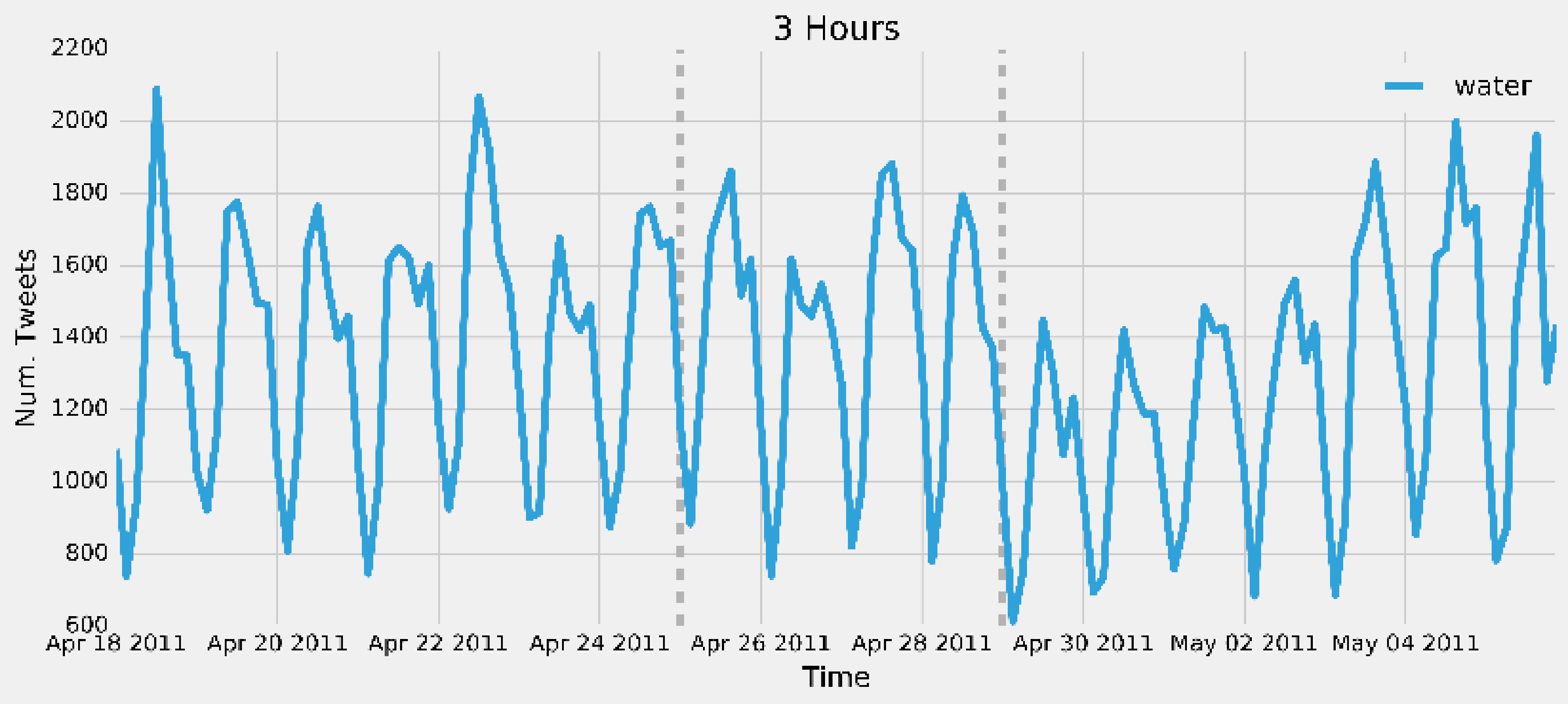
3 Hours
water
Num. Tweets
2200
2000
1800
1600
1400
1200
1000
800
600
Apr 18 2011
Apr 20 2011
Apr 22 2011
Apr 24 2011
Apr 26 2011
Apr 28 2011
Apr 30 2011
May 02 2011
May 04 2011
Time

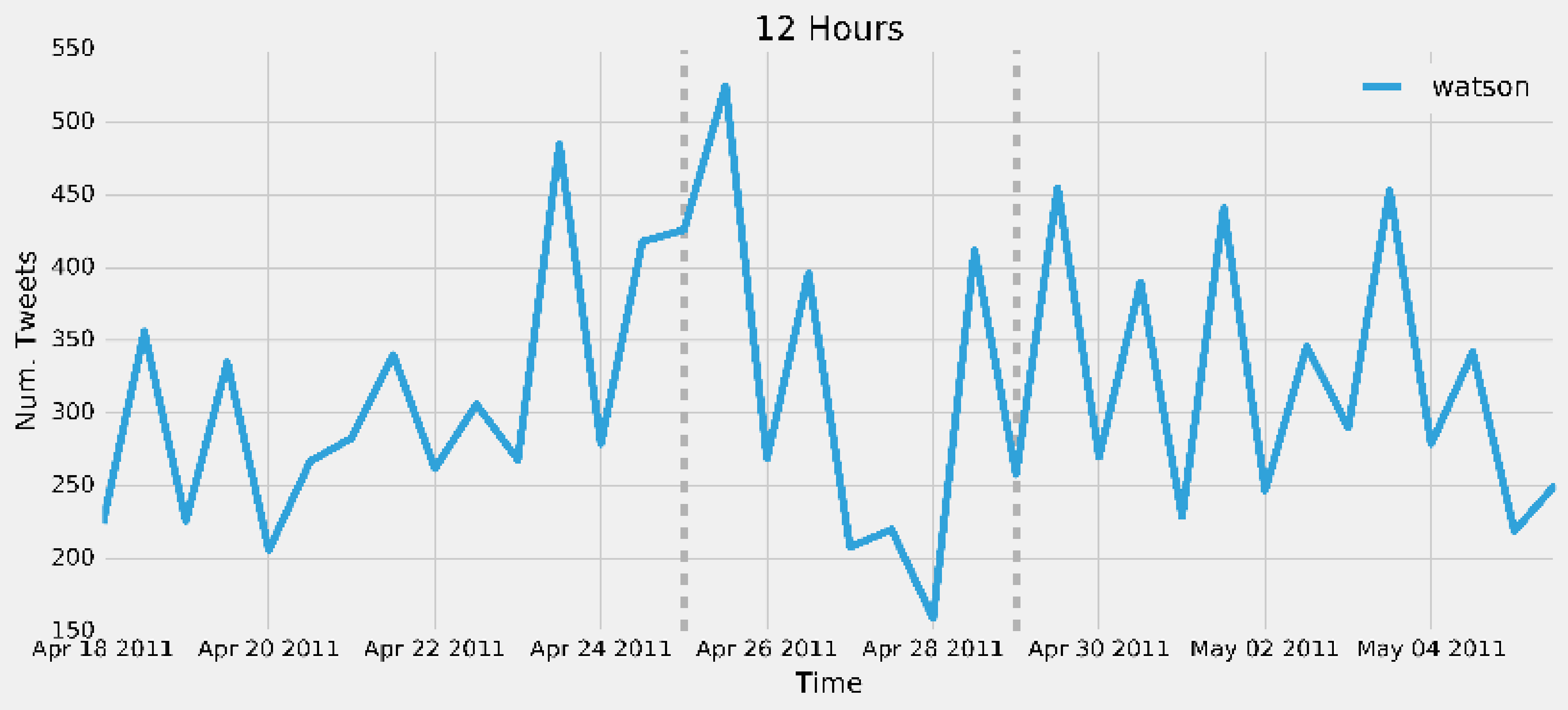
12 Hours
watson
Num. Tweets
550
500
450
400
350
300
250
200
150
Apr 18 2011
Apr 20 2011
Apr 22 2011
Apr 24 2011
Apr 26 2011
Apr 28 2011
Apr 30 2011
May 02 2011
May 04 2011
Time

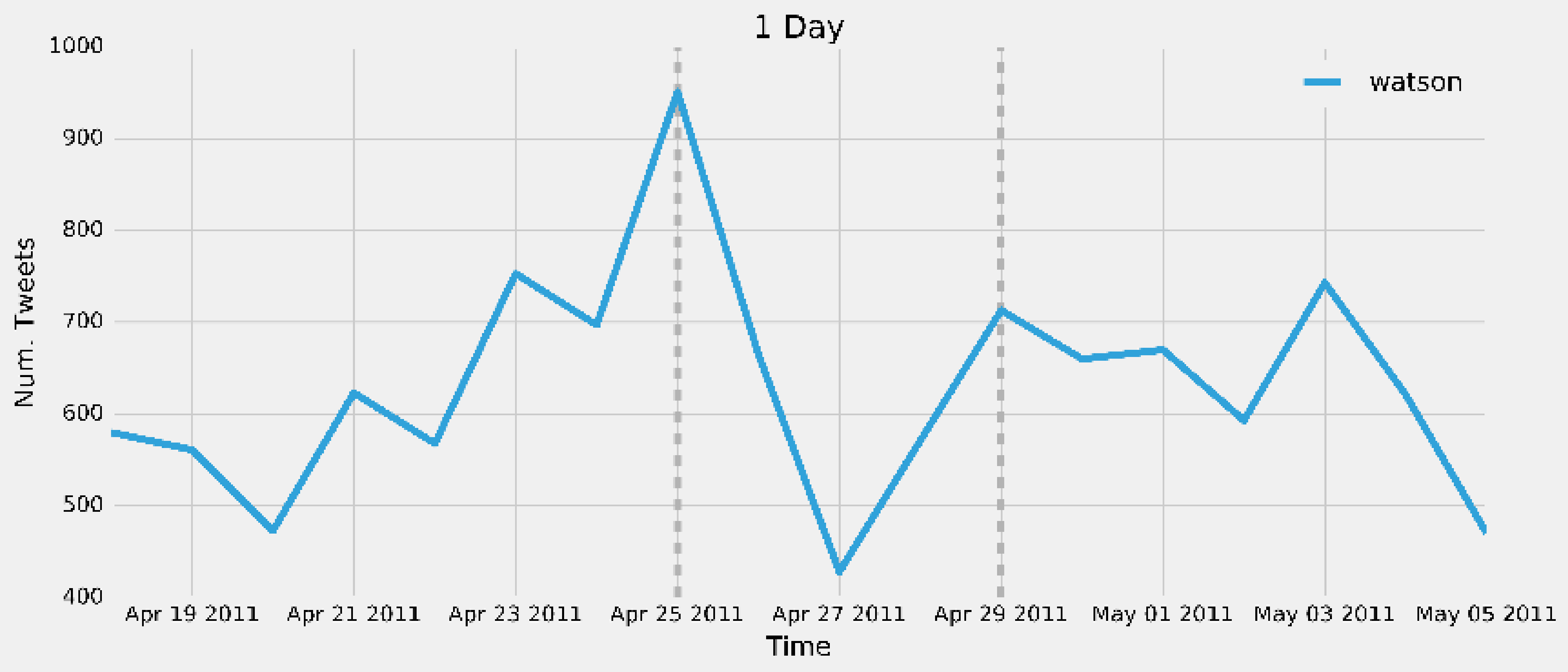

1 Day
watson
Num. Tweets
1000
900
800
700
600
500
400
Apr 19 2011
Apr 21 2011
Apr 23 2011
Apr 25 2011
Apr 27 2011
Apr 29 2011
May 01 2011
May 03 2011
May 05 2011
Time

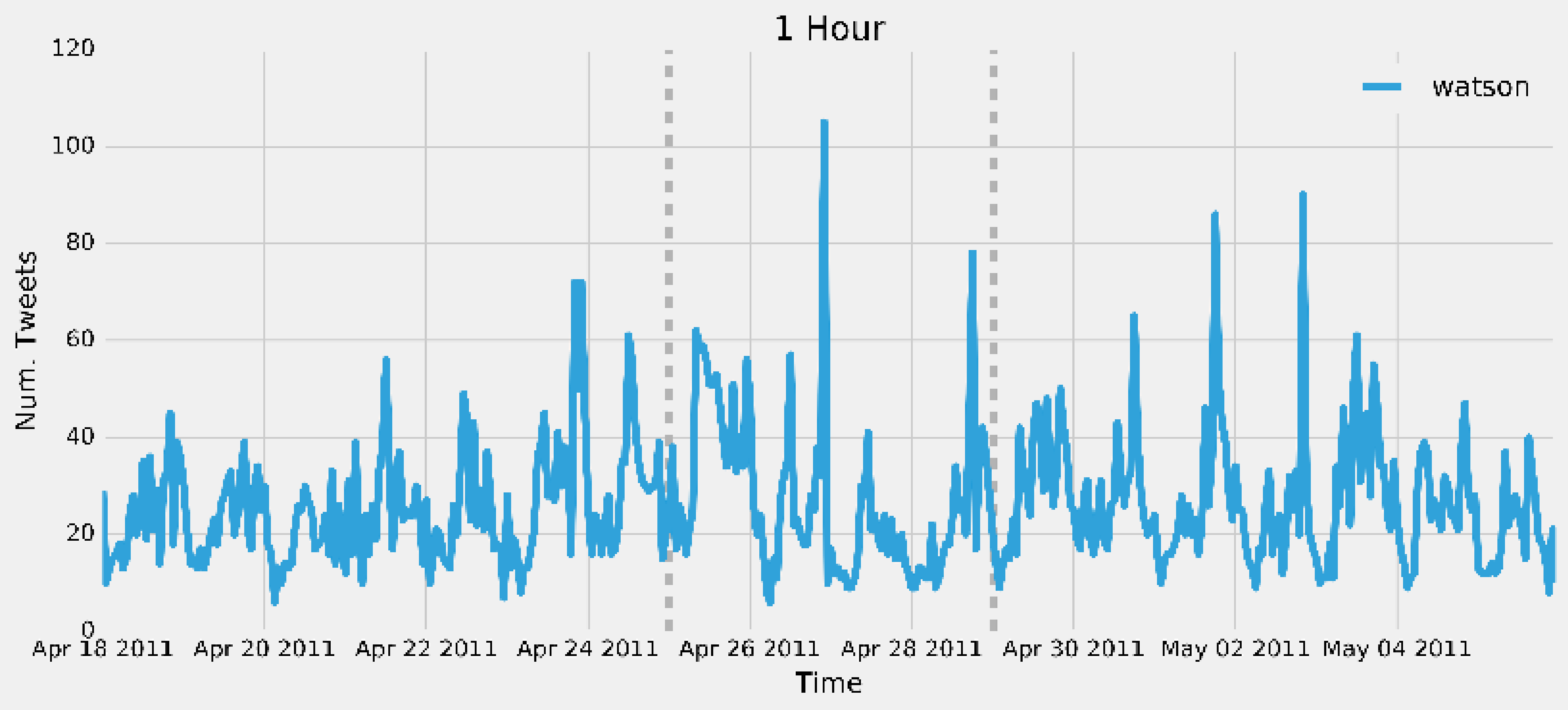

1 Hour
watson
Num. Tweets
Time
0
20
40
60
80
100
120
Apr 18 2011
Apr 20 2011
Apr 22 2011
Apr 24 2011
Apr 26 2011
Apr 28 2011
Apr 30 2011
May 02 2011
May 04 2011

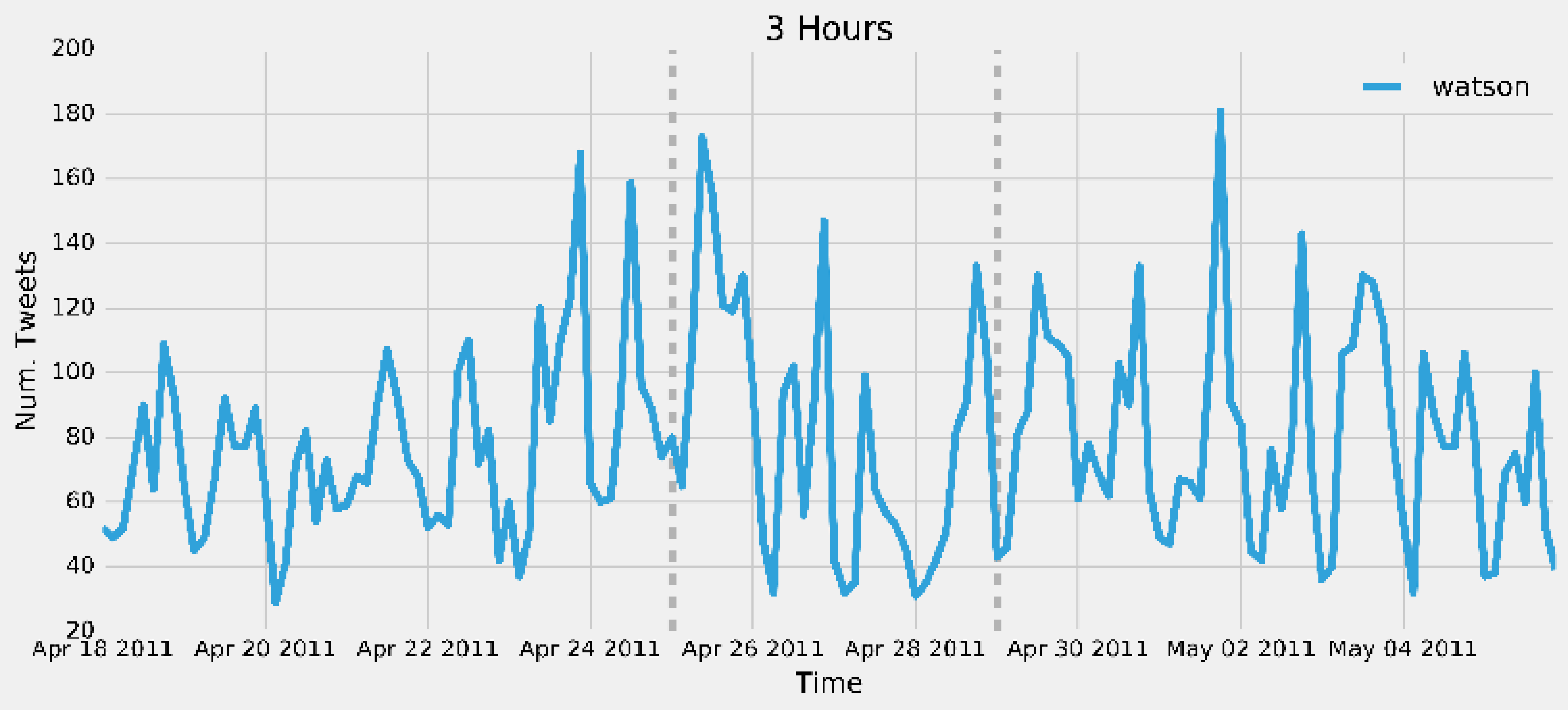

3 Hours
watson
Num. Tweets
200
180
160
140
120
100
80
60
40
20
Apr 18 2011
Apr 20 2011
Apr 22 2011
Apr 24 2011
Apr 26 2011
Apr 28 2011
Apr 30 2011
May 02 2011
May 04 2011
Time

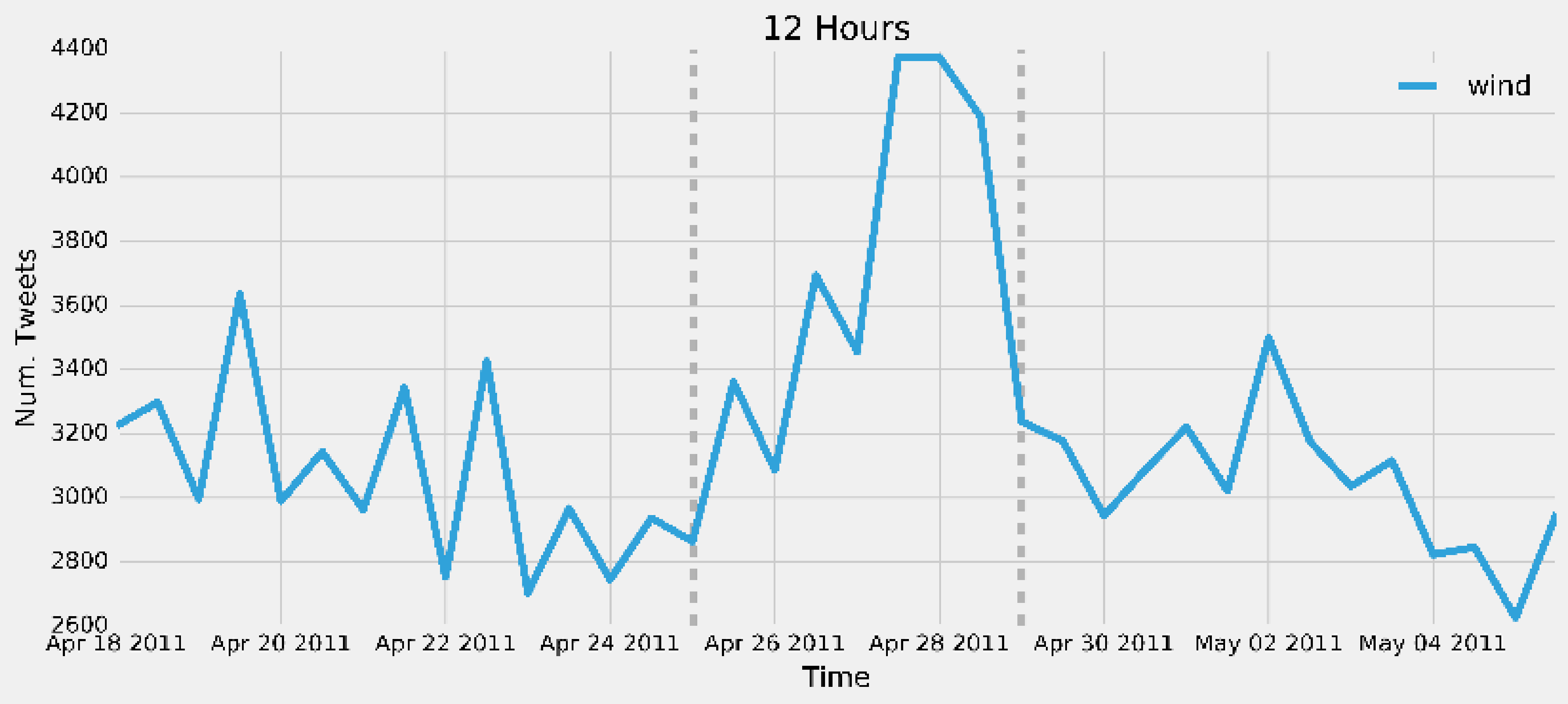
12 Hours
wind
Num. Tweets
Time
4400
4200
4000
3800
3600
3400
3200
3000
2800
2600
Apr 18 2011
Apr 20 2011
Apr 22 2011
Apr 24 2011
Apr 26 2011
Apr 28 2011
Apr 30 2011
May 02 2011
May 04 2011

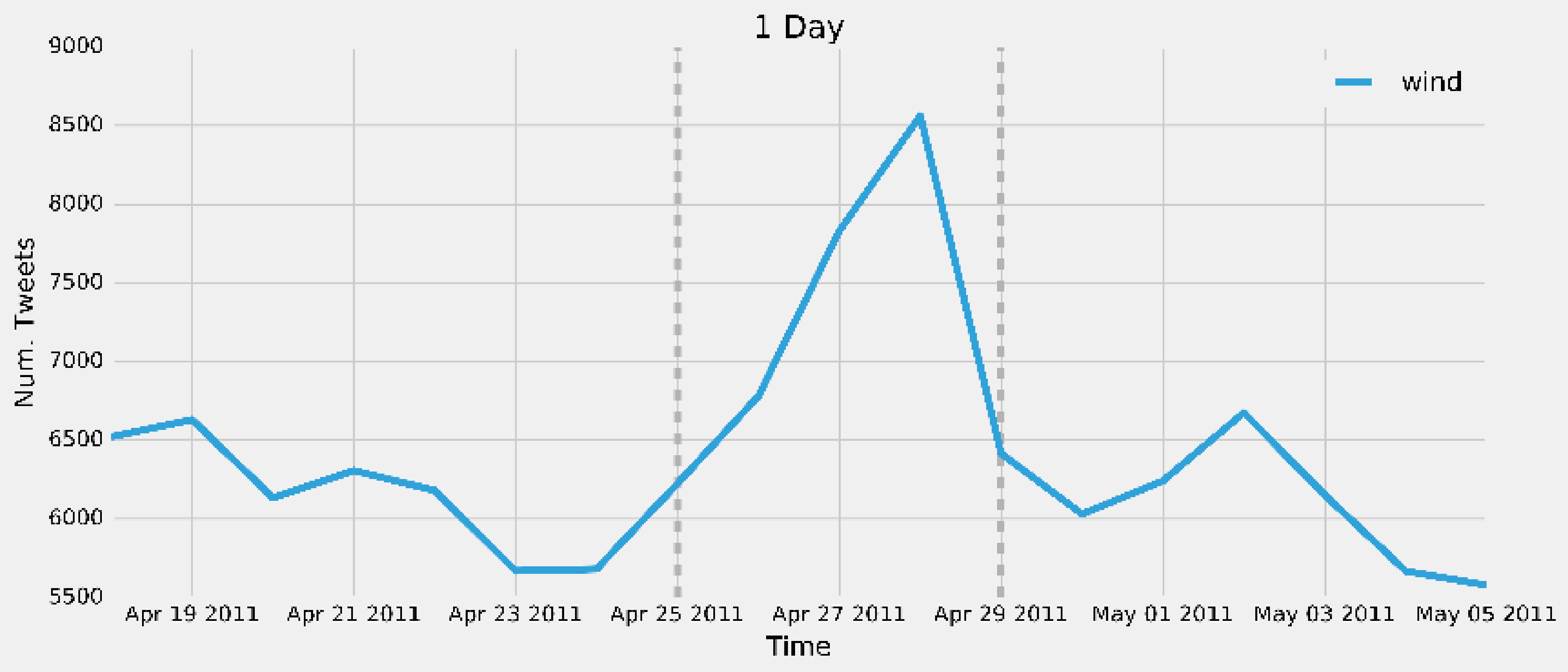
1 Day
wind
Num. Tweets
Time
9000
8500
8000
7500
7000
6500
6000
5500
Apr 19 2011
Apr 21 2011
Apr 23 2011
Apr 25 2011
Apr 27 2011
Apr 29 2011
May 01 2011
May 03 2011
May 05 2011

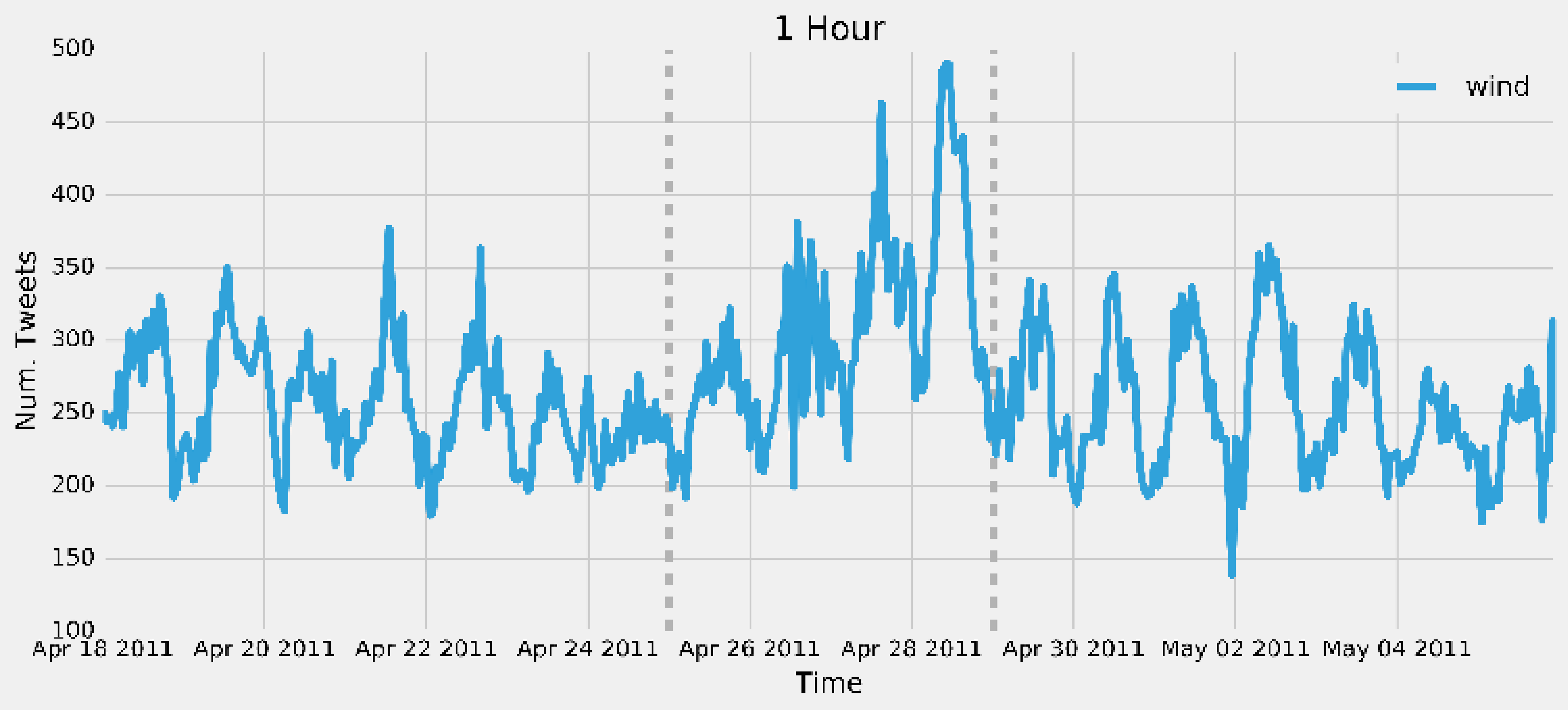

1 Hour
wind
Num. Tweets
Time
500
450
400
350
300
250
200
150
100
Apr 18 2011
Apr 20 2011
Apr 22 2011
Apr 24 2011
Apr 26 2011
Apr 28 2011
Apr 30 2011
May 02 2011
May 04 2011

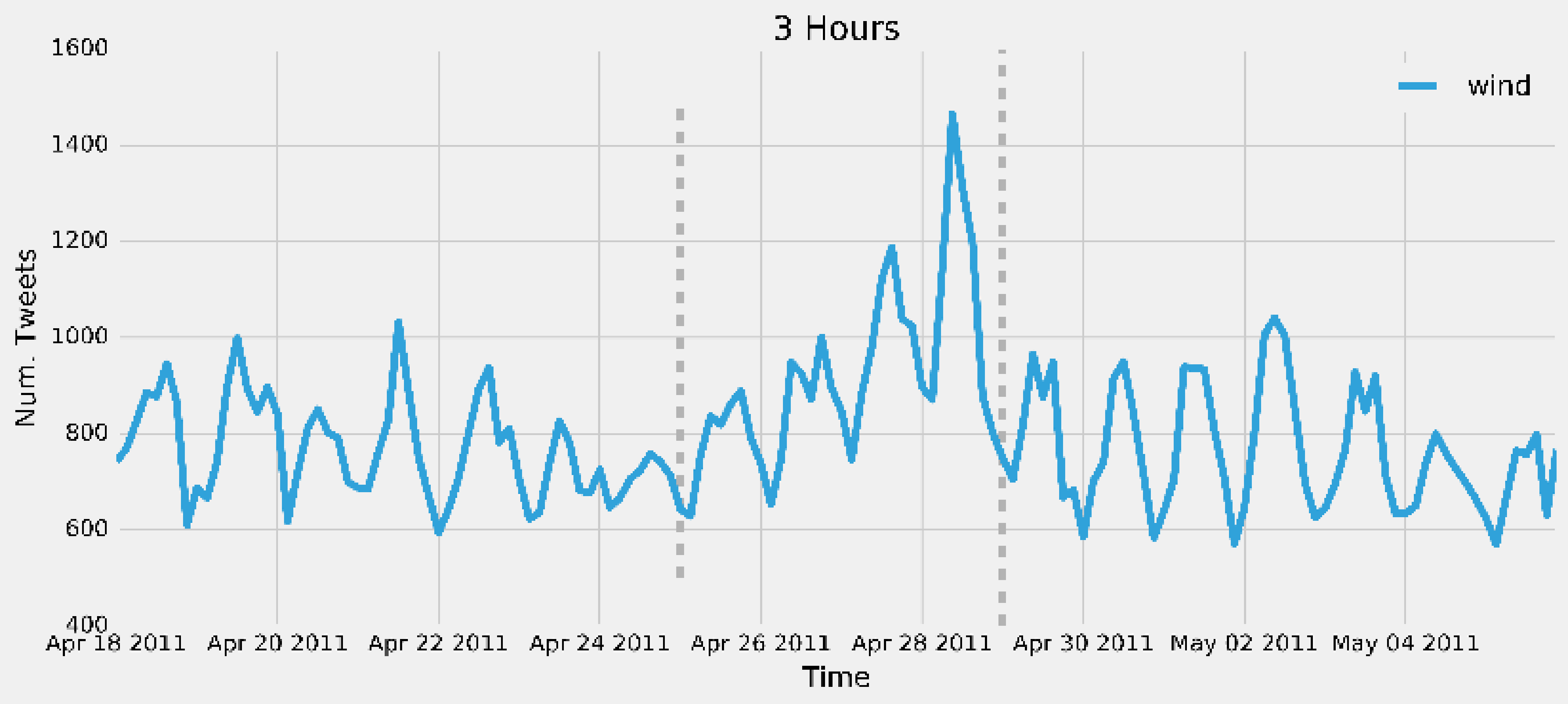
3 Hours
wind
Num. Tweets
1600
1400
1200
1000
800
600
400
Apr 18 2011
Apr 20 2011
Apr 22 2011
Apr 24 2011
Apr 26 2011
Apr 28 2011
Apr 30 2011
May 02 2011
May 04 2011
Time
