# Supplementary material for: Social media usage patterns during natural hazards
Source: PLoS One. 2019 Feb 13;14(2):e0210484. doi: 10.1371/journal.pone.0210484 (PMC6374021; doi:10.1371/journal.pone.0210484)
Supplement: S5 Fig — (PDF) [file pone.0210484.s005.pdf]

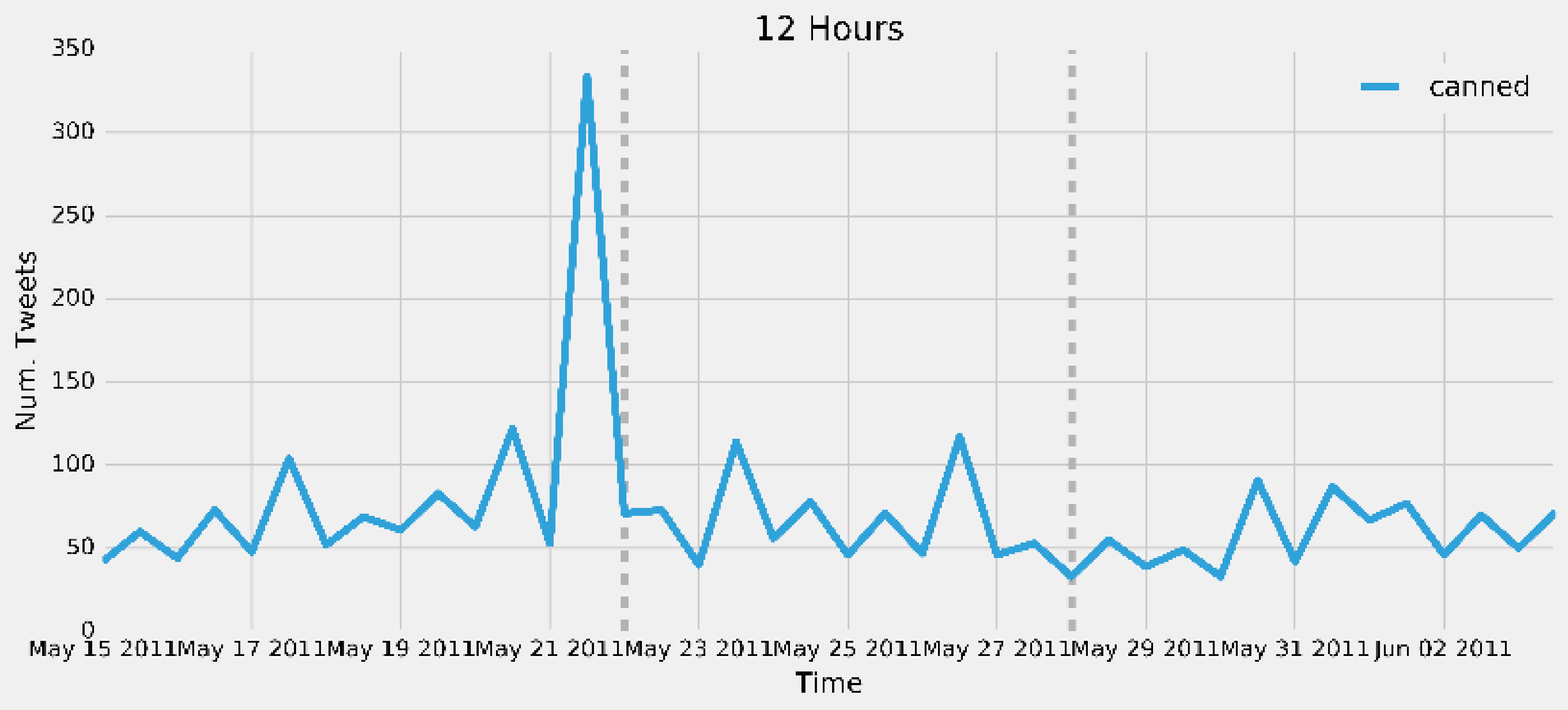
12 Hours
canned
Num. Tweets
0
50
100
150
200
250
300
350
May 15 2011
May 17 2011
May 19 2011
May 21 2011
May 23 2011
May 25 2011
May 27 2011
May 29 2011
May 31 2011
Jun 02 2011
Time

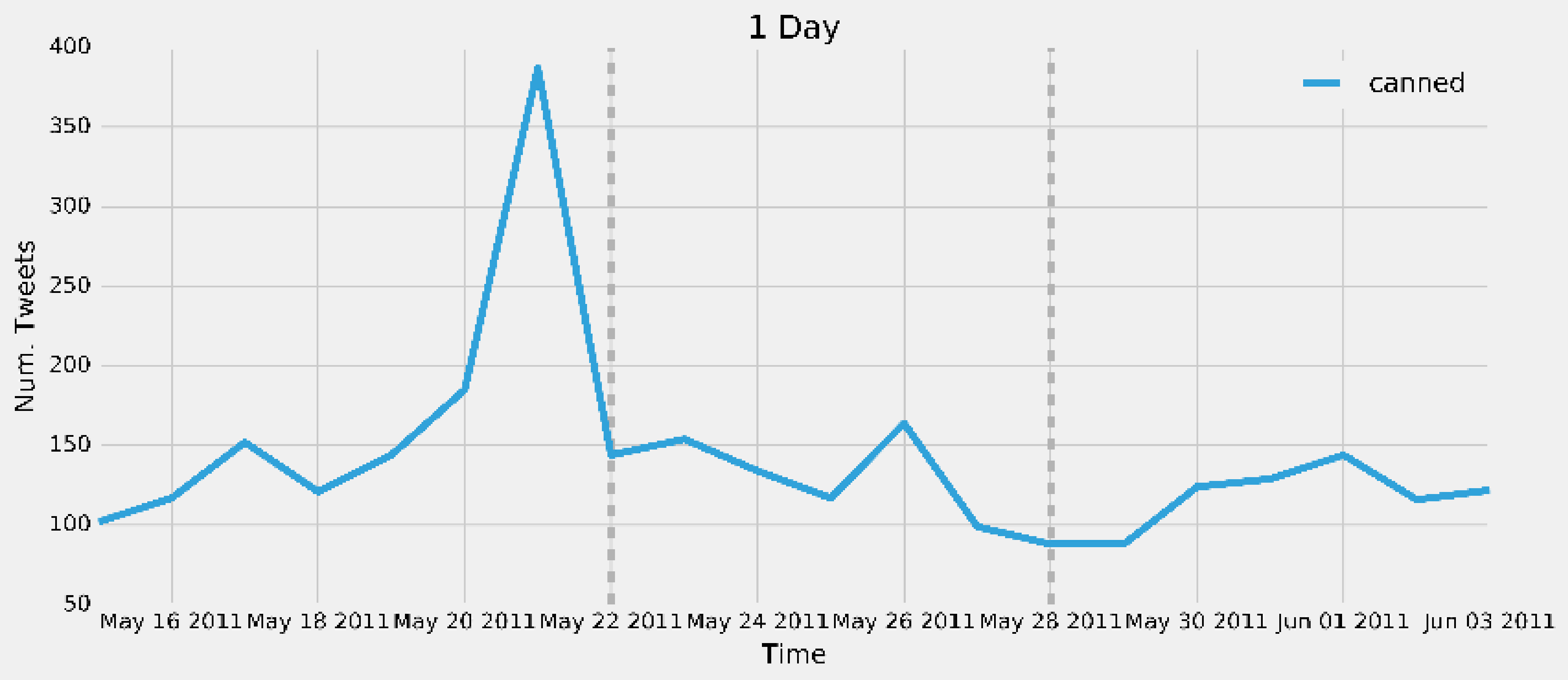
1 Day
canned
Num. Tweets
400
350
300
250
200
150
100
50
May 16 2011
May 18 2011
May 20 2011
May 22 2011
May 24 2011
May 26 2011
May 28 2011
May 30 2011
Jun 01 2011
Jun 03 2011
Time

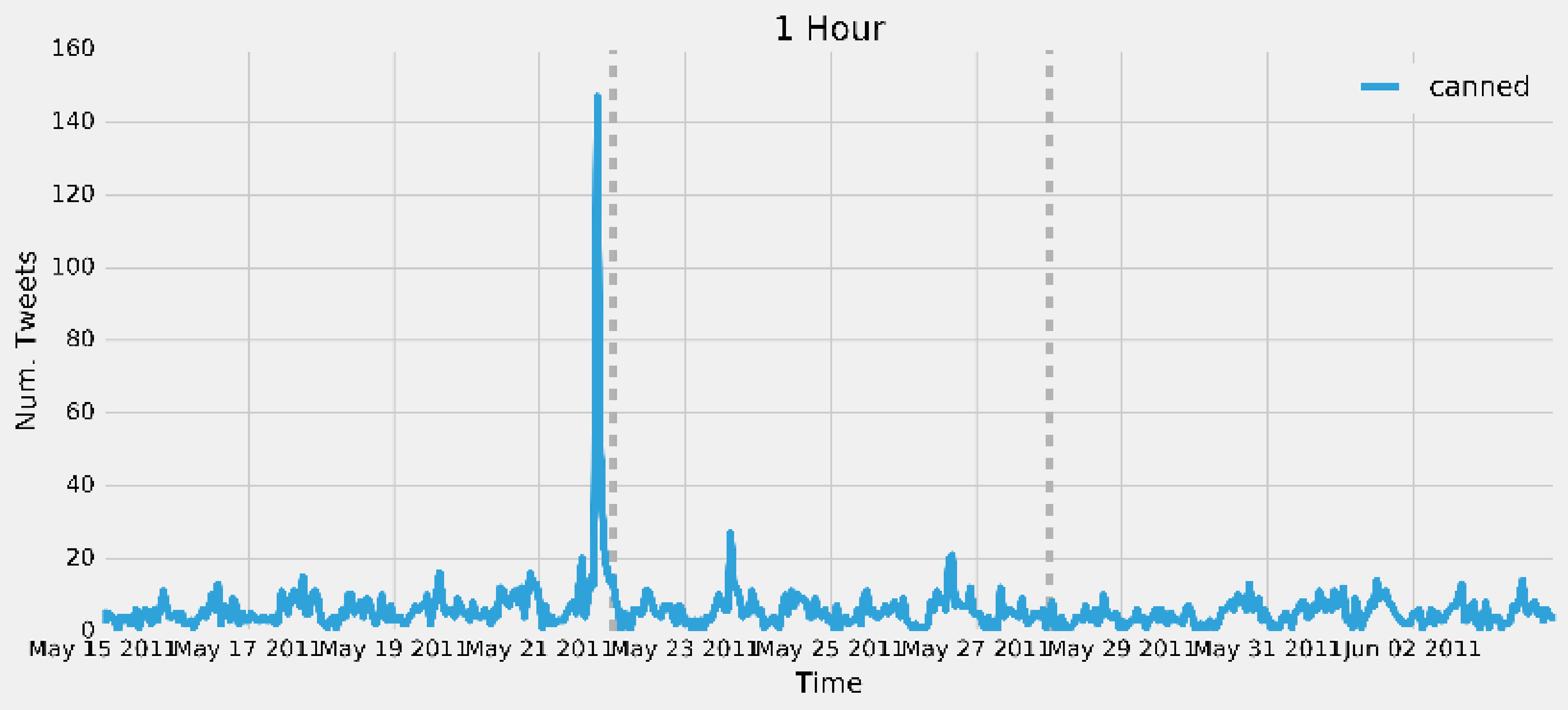
1 Hour
canned
Num. Tweets
Time
160
140
120
100
80
60
40
20
0
May 15 2011
May 17 2011
May 19 2011
May 21 2011
May 23 2011
May 25 2011
May 27 2011
May 29 2011
May 31 2011
Jun 02 2011

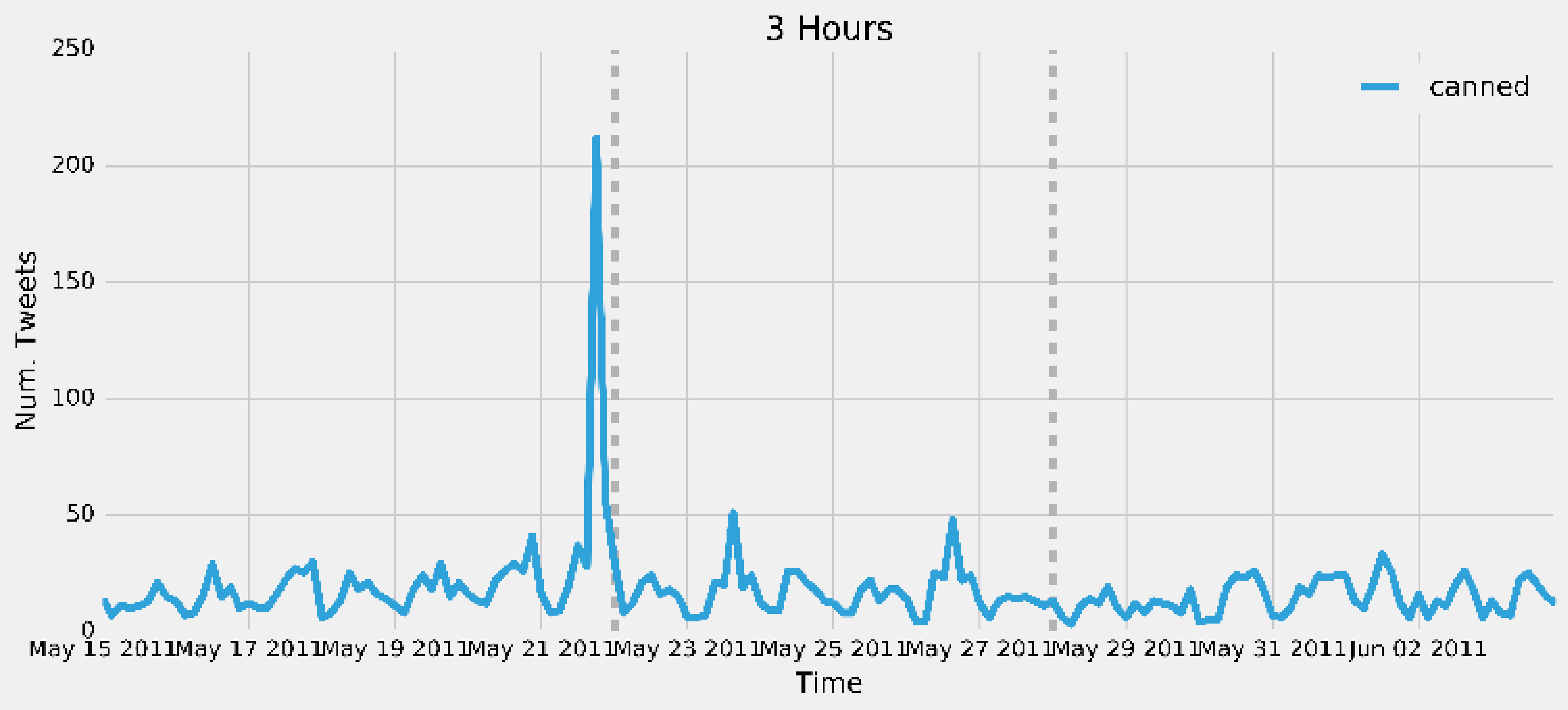

3 Hours
canned
Num. Tweets
0
50
100
150
200
250
May 15 2011
May 17 2011
May 19 2011
May 21 2011
May 23 2011
May 25 2011
May 27 2011
May 29 2011
May 31 2011
Jun 02 2011
Time

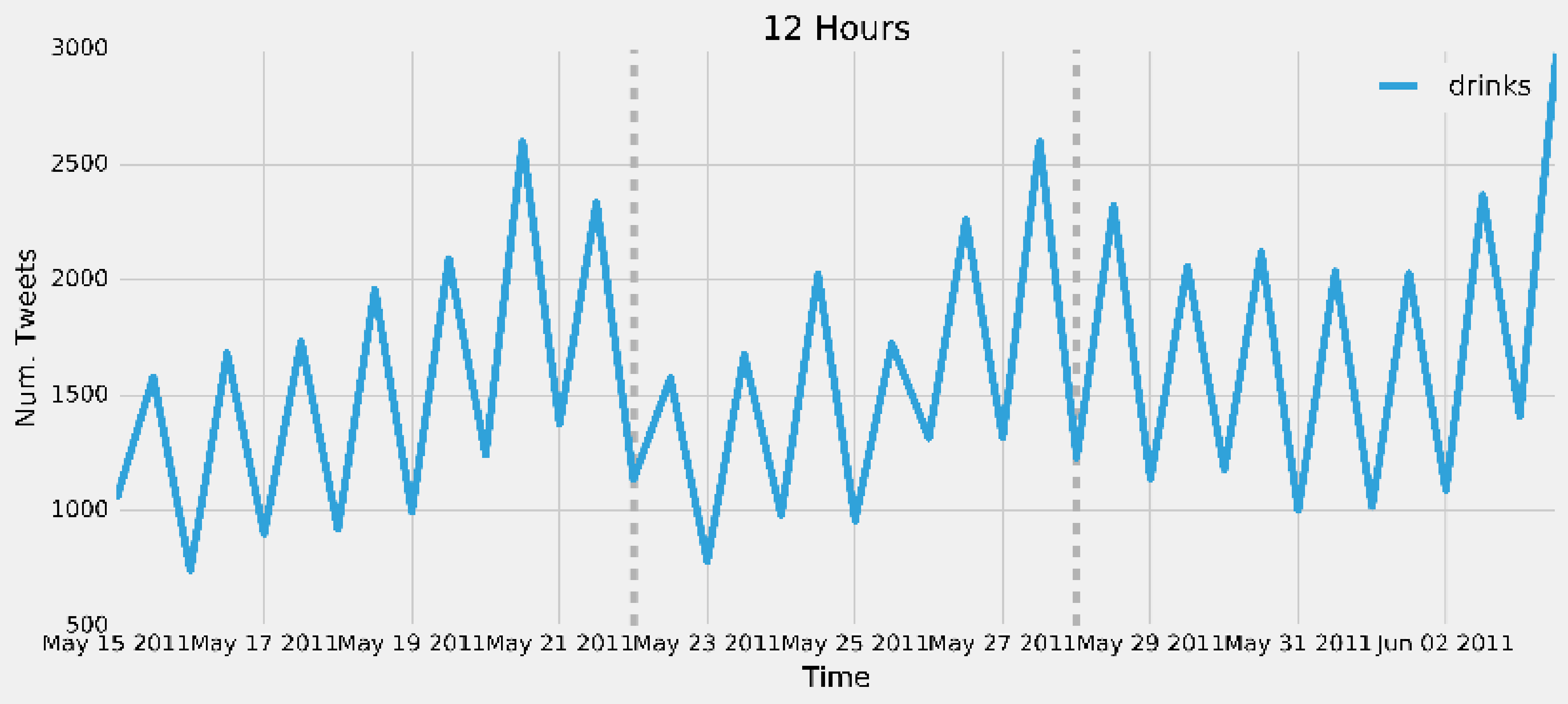
12 Hours
drinks
3000
2500
2000
1500
1000
500
Num. Tweets
May 15 2011
May 17 2011
May 19 2011
May 21 2011
May 23 2011
May 25 2011
May 27 2011
May 29 2011
May 31 2011
Jun 02 2011
Time

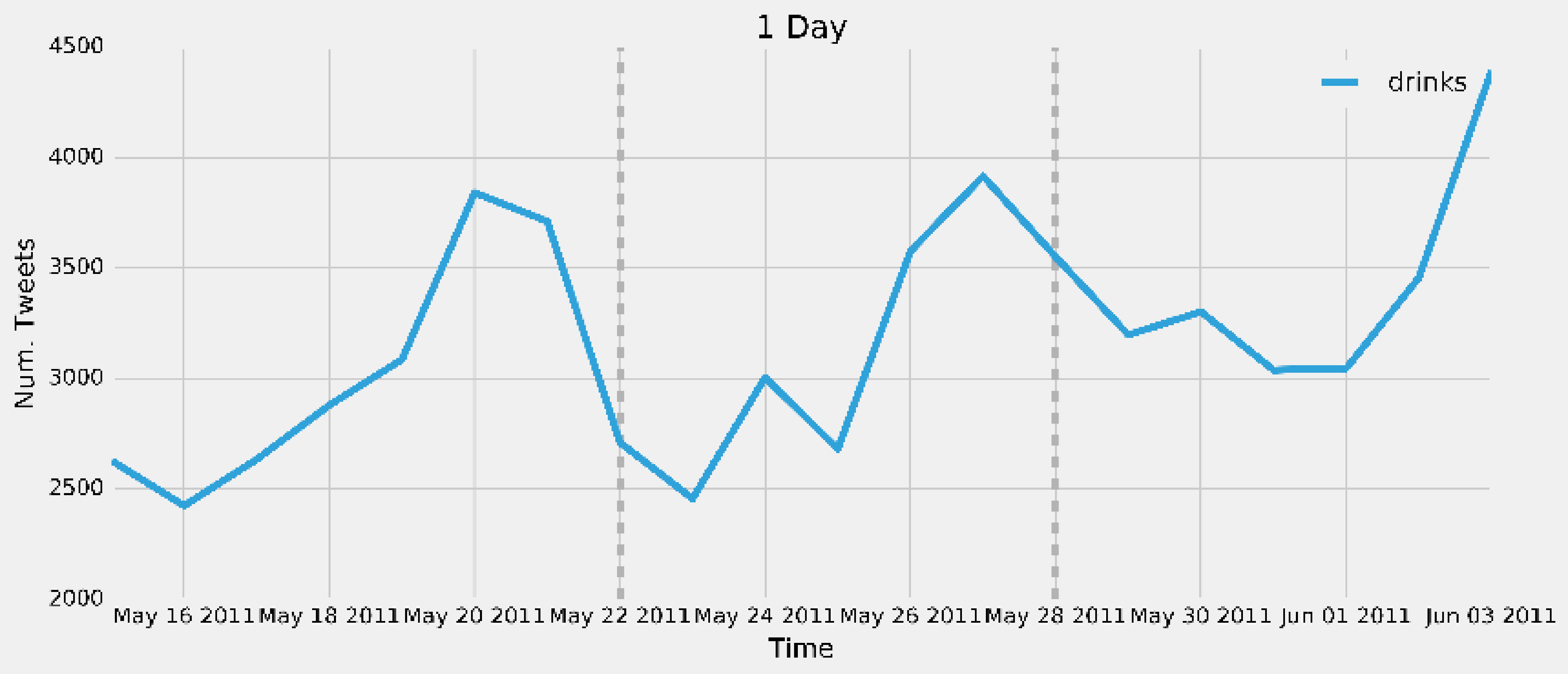
1 Day
drinks
Num. Tweets
4500
4000
3500
3000
2500
2000
May 16 2011
May 18 2011
May 20 2011
May 22 2011
May 24 2011
May 26 2011
May 28 2011
May 30 2011
Jun 01 2011
Jun 03 2011
Time

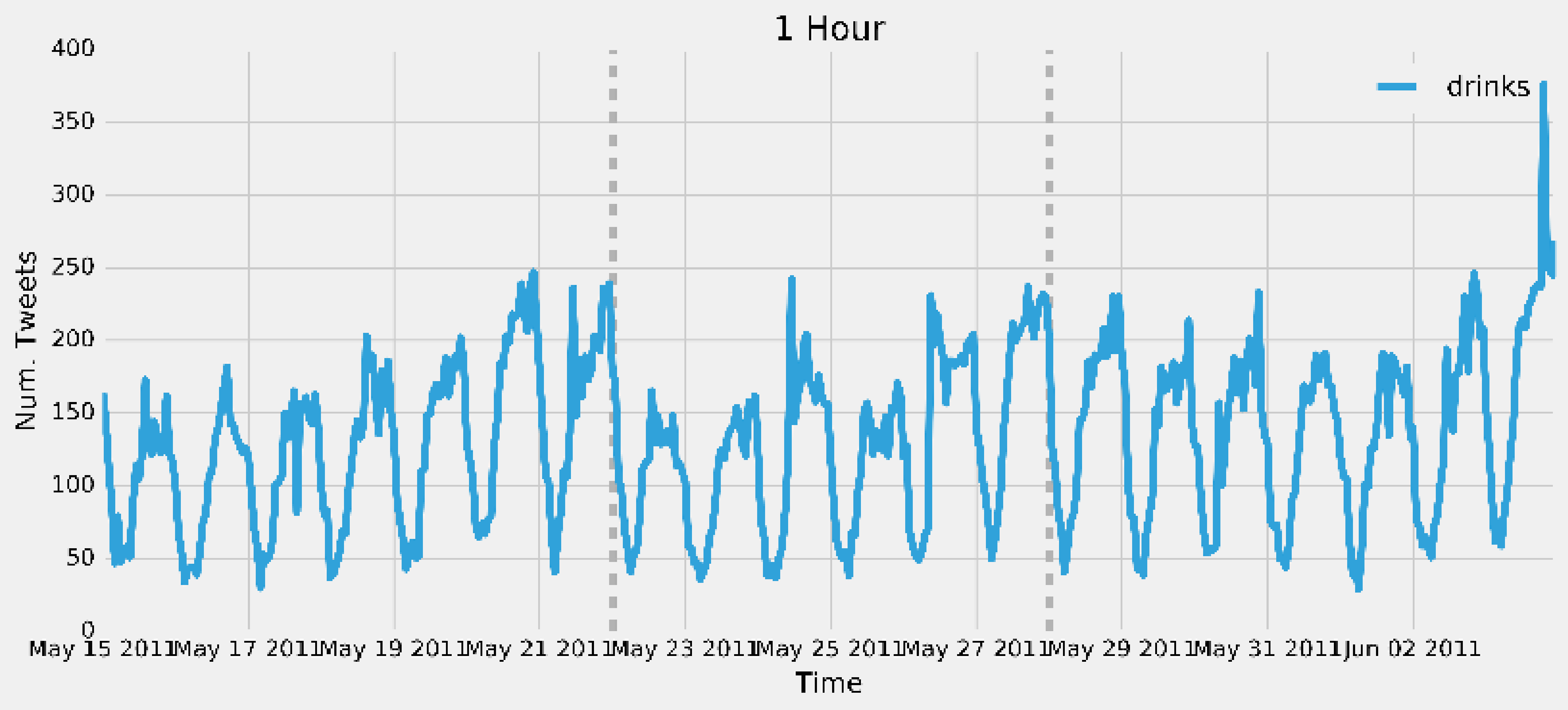
1 Hour
drinks
Num. Tweets
400
350
300
250
200
150
100
50
0
May 15 2011
May 17 2011
May 19 2011
May 21 2011
May 23 2011
May 25 2011
May 27 2011
May 29 2011
May 31 2011
Jun 02 2011
Time

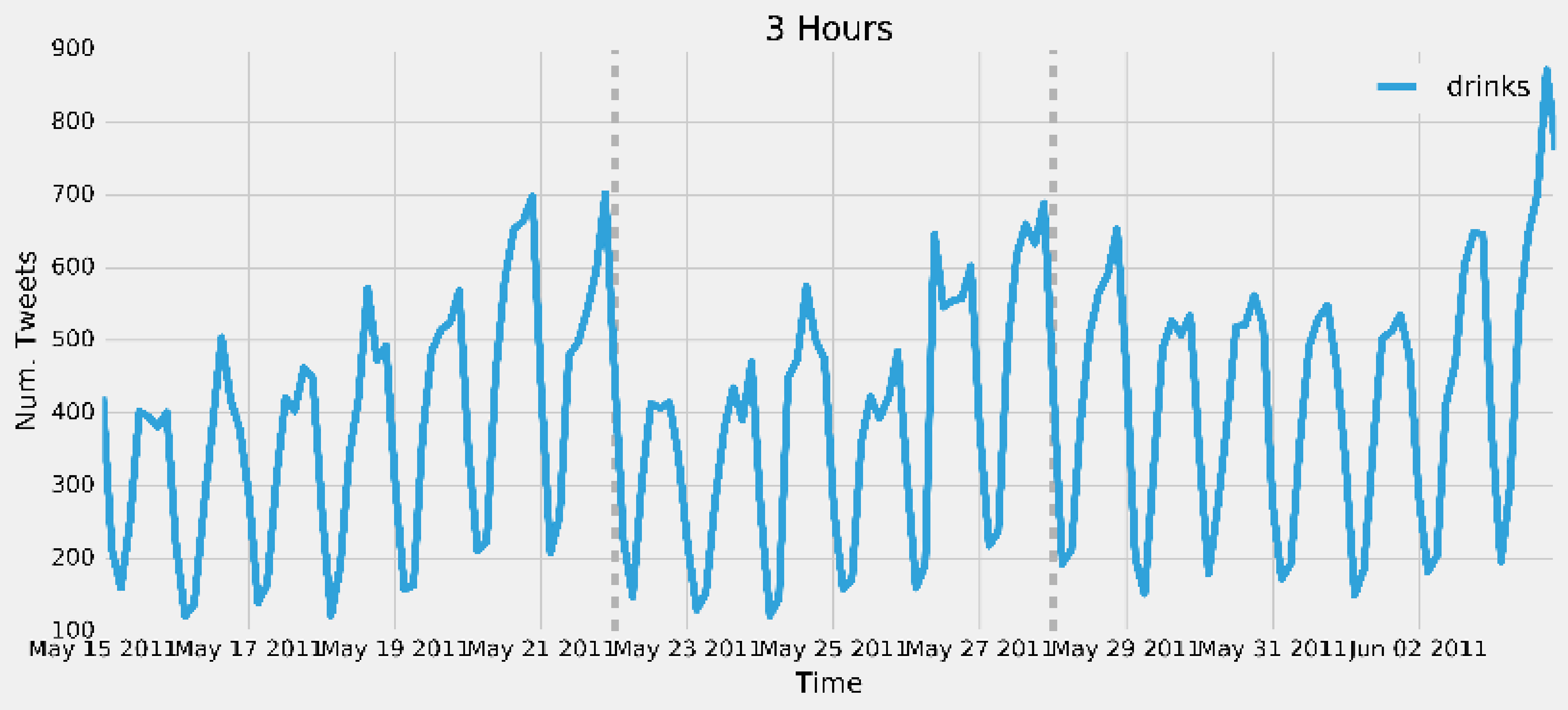
3 Hours
drinks
900
800
700
600
500
400
300
200
100
Num. Tweets
May 15 2011
May 17 2011
May 19 2011
May 21 2011
May 23 2011
May 25 2011
May 27 2011
May 29 2011
May 31 2011
Jun 02 2011
Time

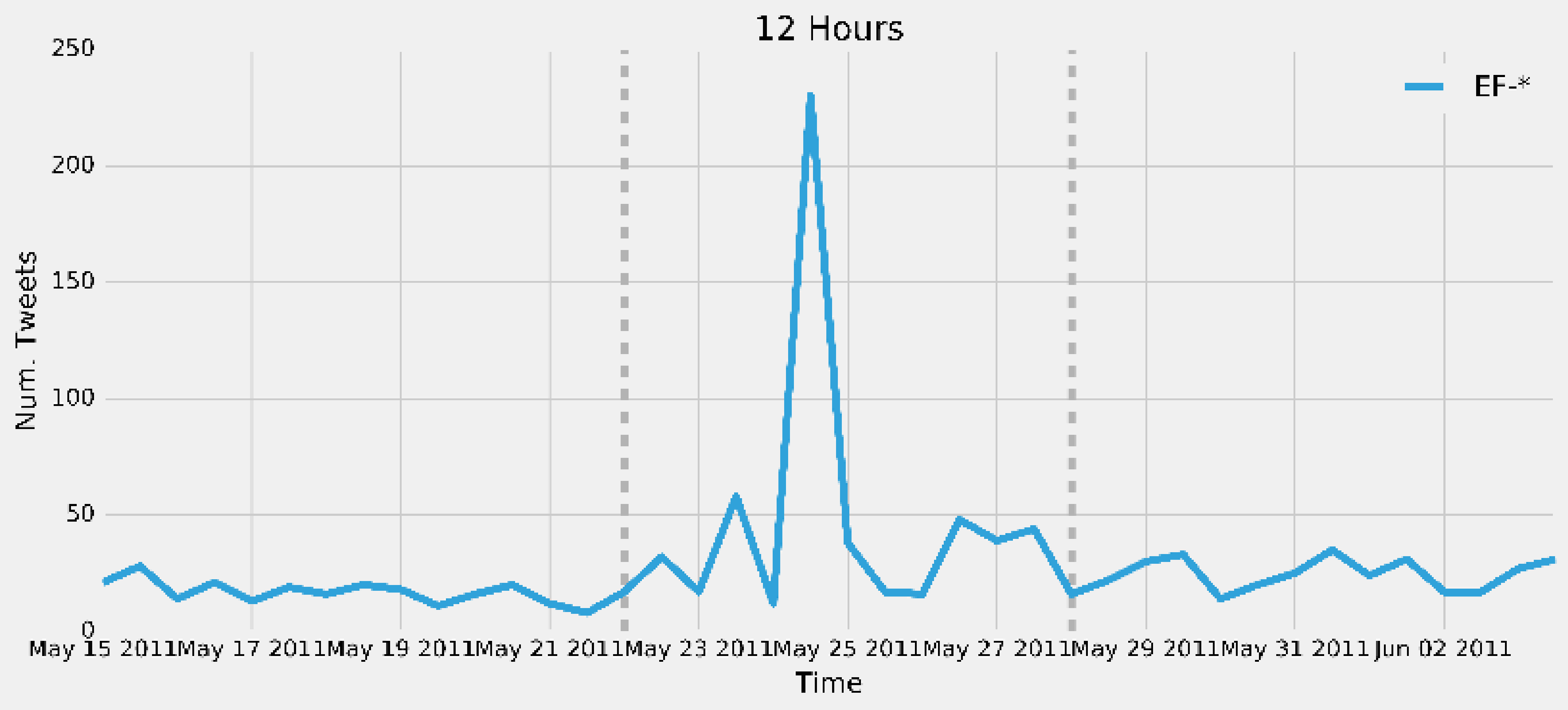

12 Hours
EF-*
Num. Tweets
250
200
150
100
50
0
May 15 2011
May 17 2011
May 19 2011
May 21 2011
May 23 2011
May 25 2011
May 27 2011
May 29 2011
May 31 2011
Jun 02 2011
Time

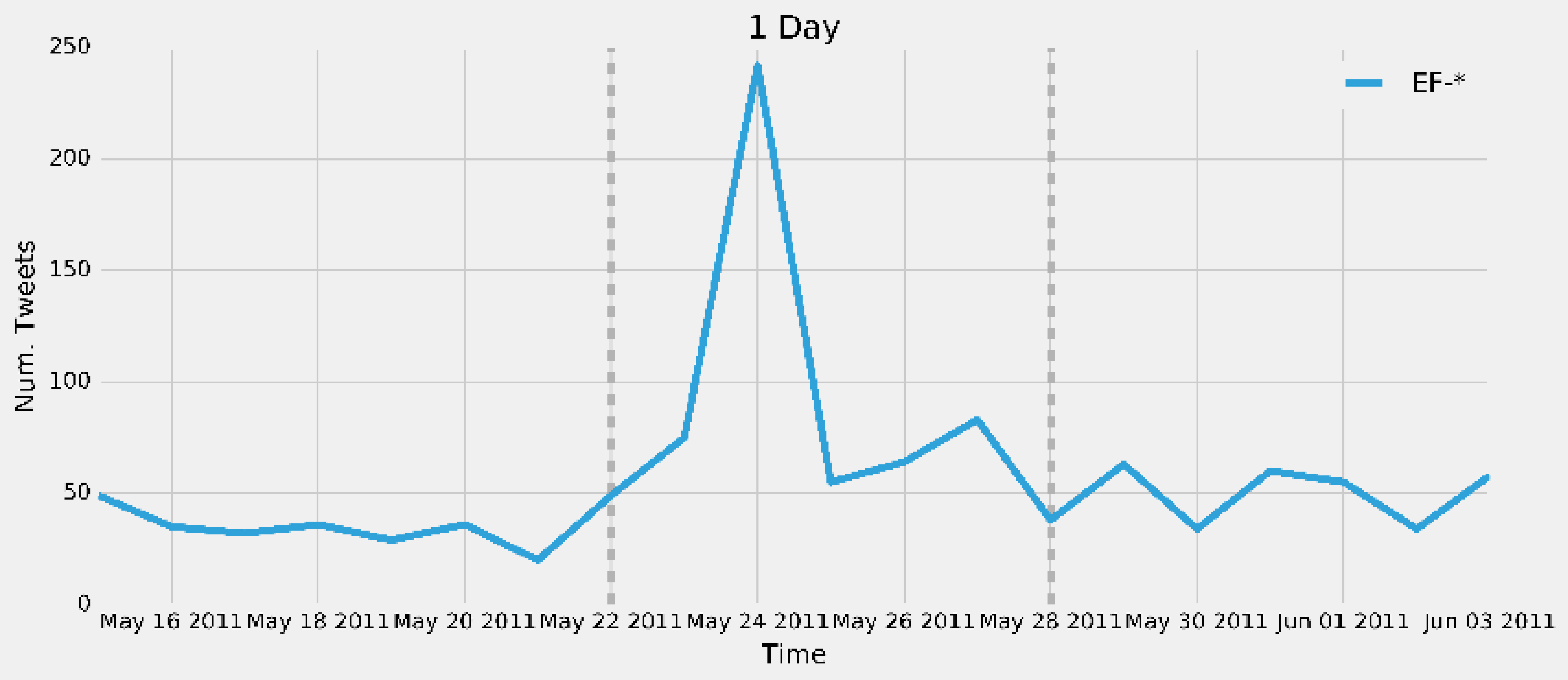
1 Day
EF-*
Num. Tweets
Time
0
50
100
150
200
250
May 16 2011
May 18 2011
May 20 2011
May 22 2011
May 24 2011
May 26 2011
May 28 2011
May 30 2011
Jun 01 2011
Jun 03 2011

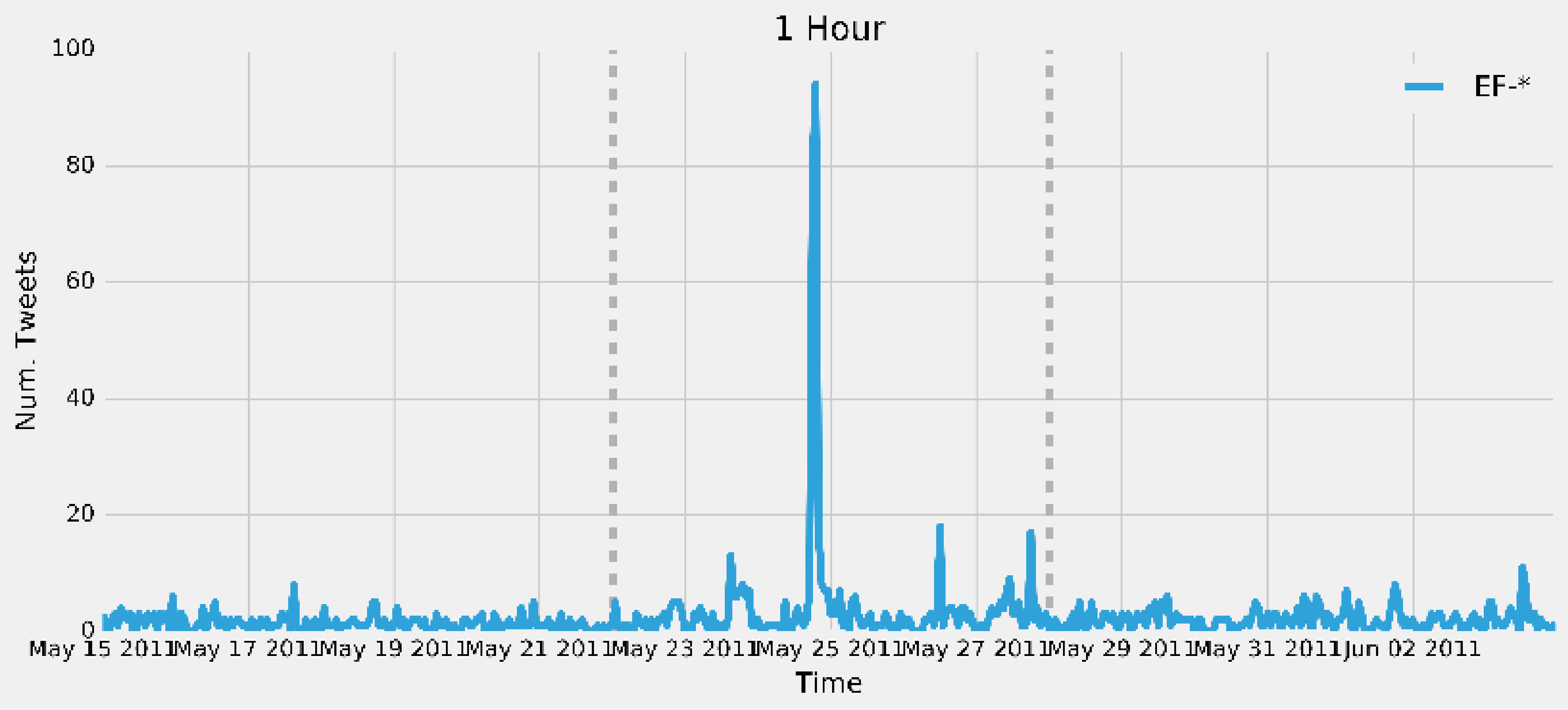
1 Hour
EF-*
Num. Tweets
100
80
60
40
20
0
May 15 2011
May 17 2011
May 19 2011
May 21 2011
May 23 2011
May 25 2011
May 27 2011
May 29 2011
May 31 2011
Jun 02 2011
Time

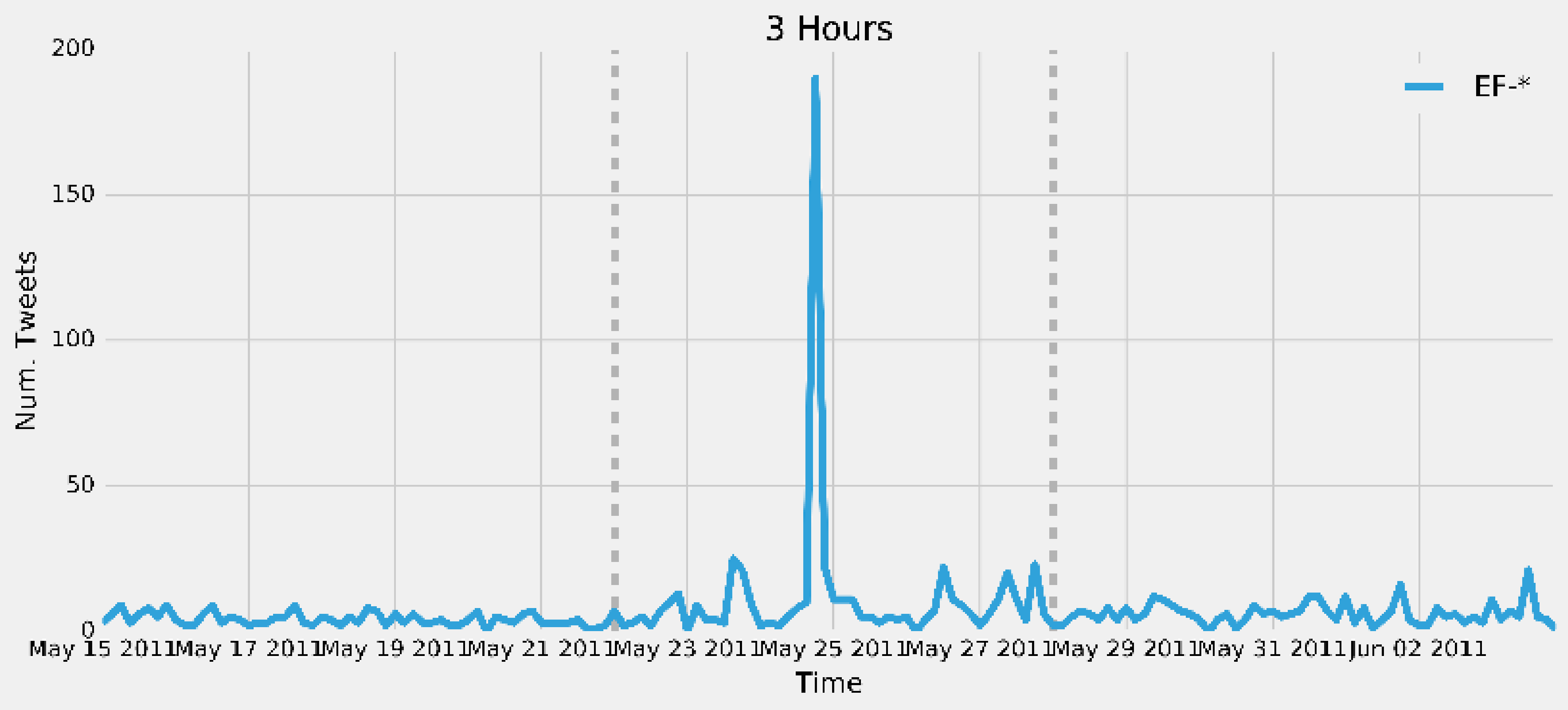

3 Hours
200
150
100
50
0
Num. Tweets
EF-*
May 15 2011
May 17 2011
May 19 2011
May 21 2011
May 23 2011
May 25 2011
May 27 2011
May 29 2011
May 31 2011
Jun 02 2011
Time

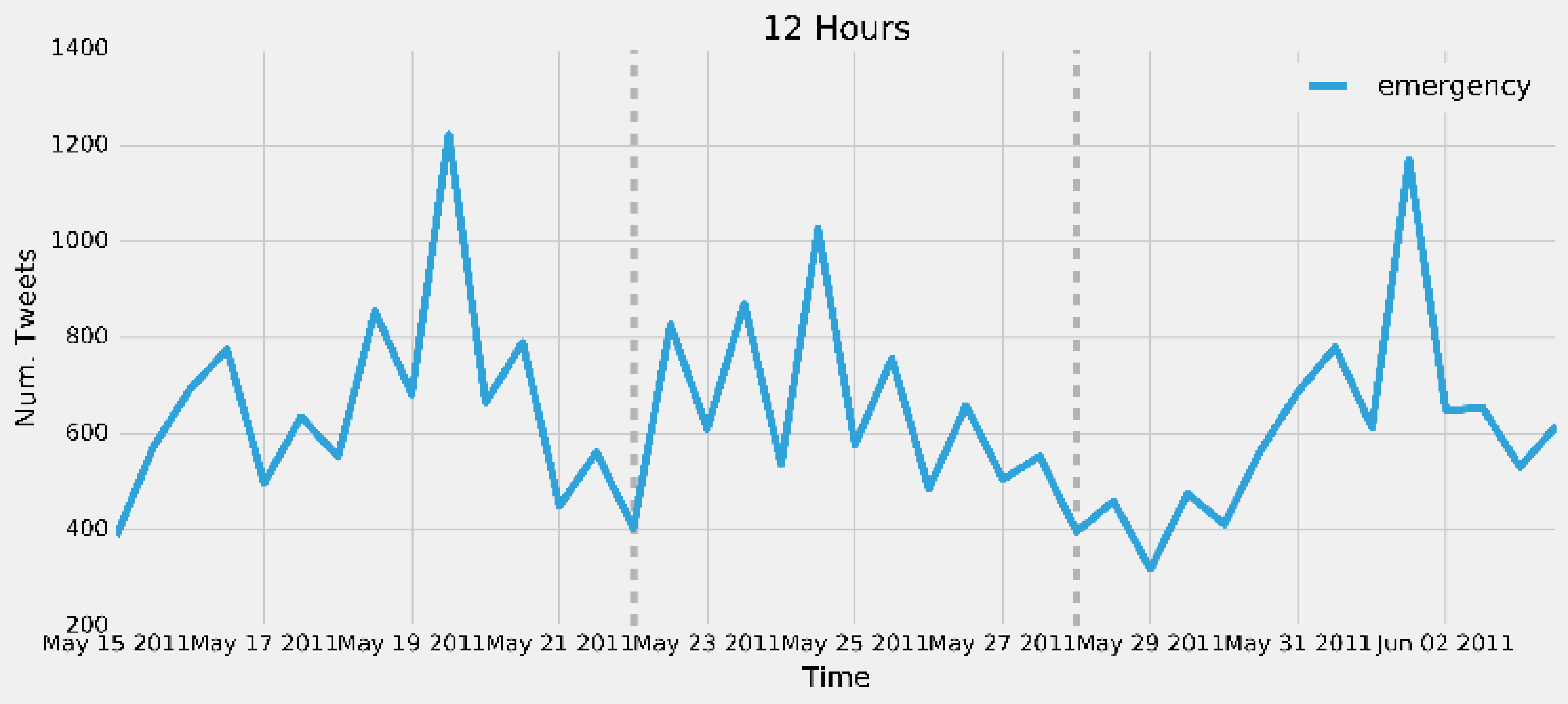
12 Hours
emergency
Num. Tweets
Time
1400
1200
1000
800
600
400
200
May 15 2011
May 17 2011
May 19 2011
May 21 2011
May 23 2011
May 25 2011
May 27 2011
May 29 2011
May 31 2011
Jun 02 2011

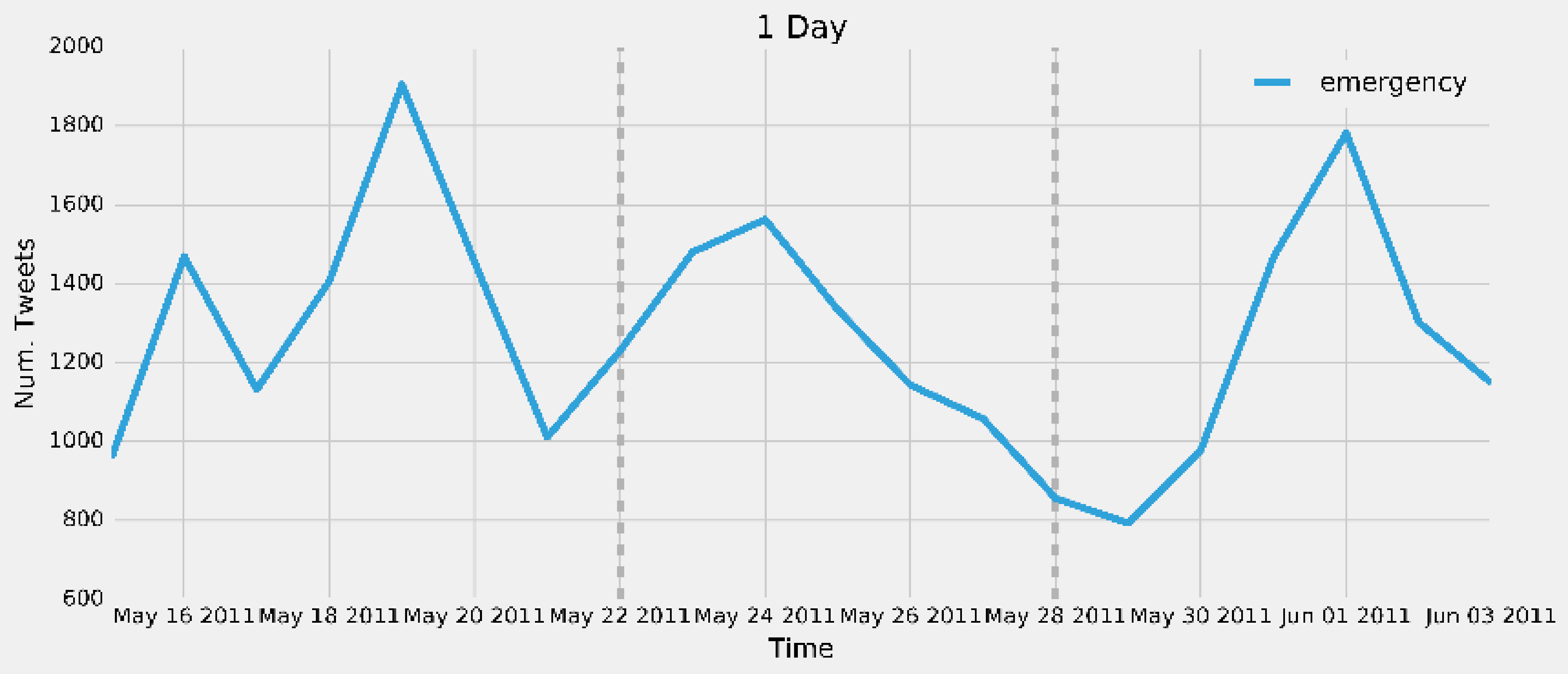
1 Day
emergency
Num. Tweets
Time
2000
1800
1600
1400
1200
1000
800
600
May 16 2011
May 18 2011
May 20 2011
May 22 2011
May 24 2011
May 26 2011
May 28 2011
May 30 2011
Jun 01 2011
Jun 03 2011

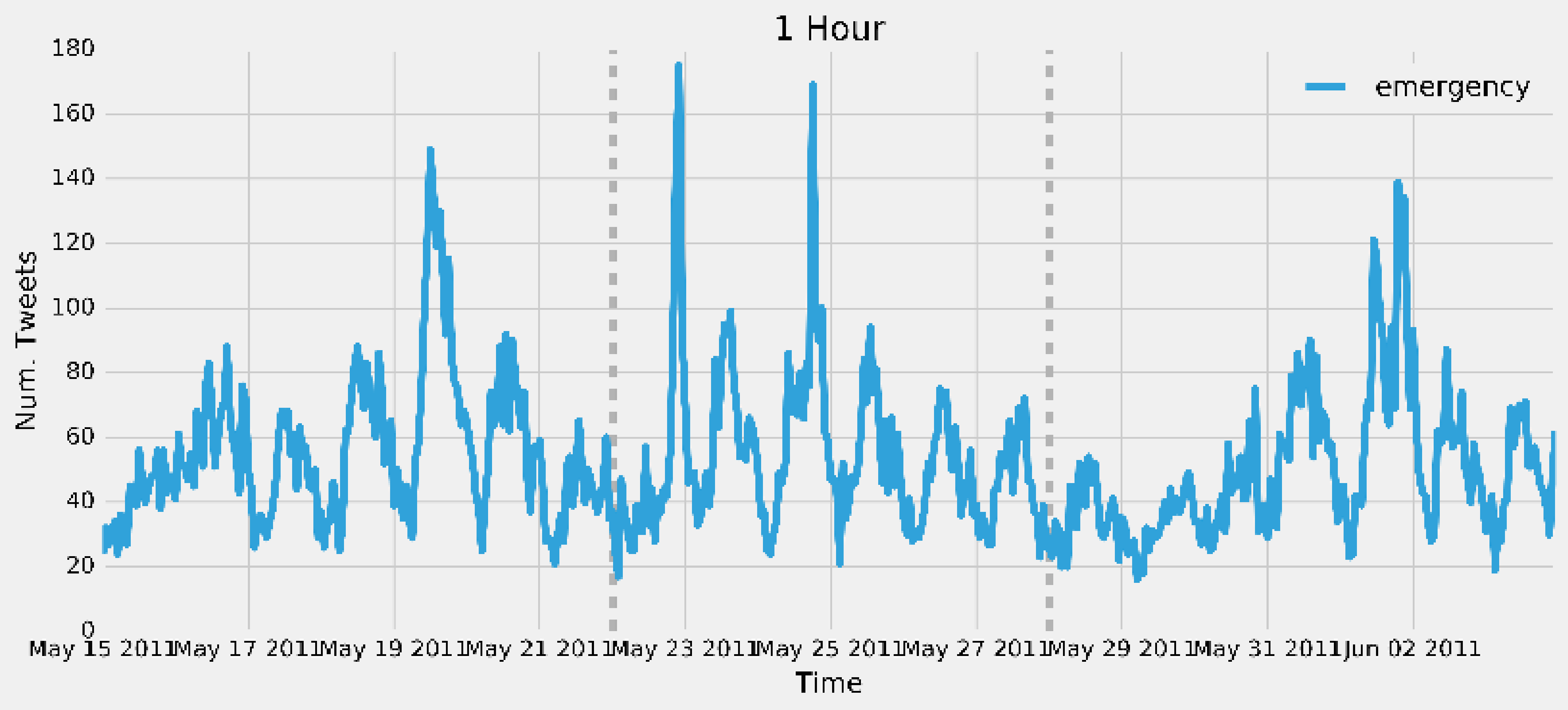
1 Hour
emergency
Num. Tweets
0
20
40
60
80
100
120
140
160
180
May 15 2011
May 17 2011
May 19 2011
May 21 2011
May 23 2011
May 25 2011
May 27 2011
May 29 2011
May 31 2011
Jun 02 2011
Time

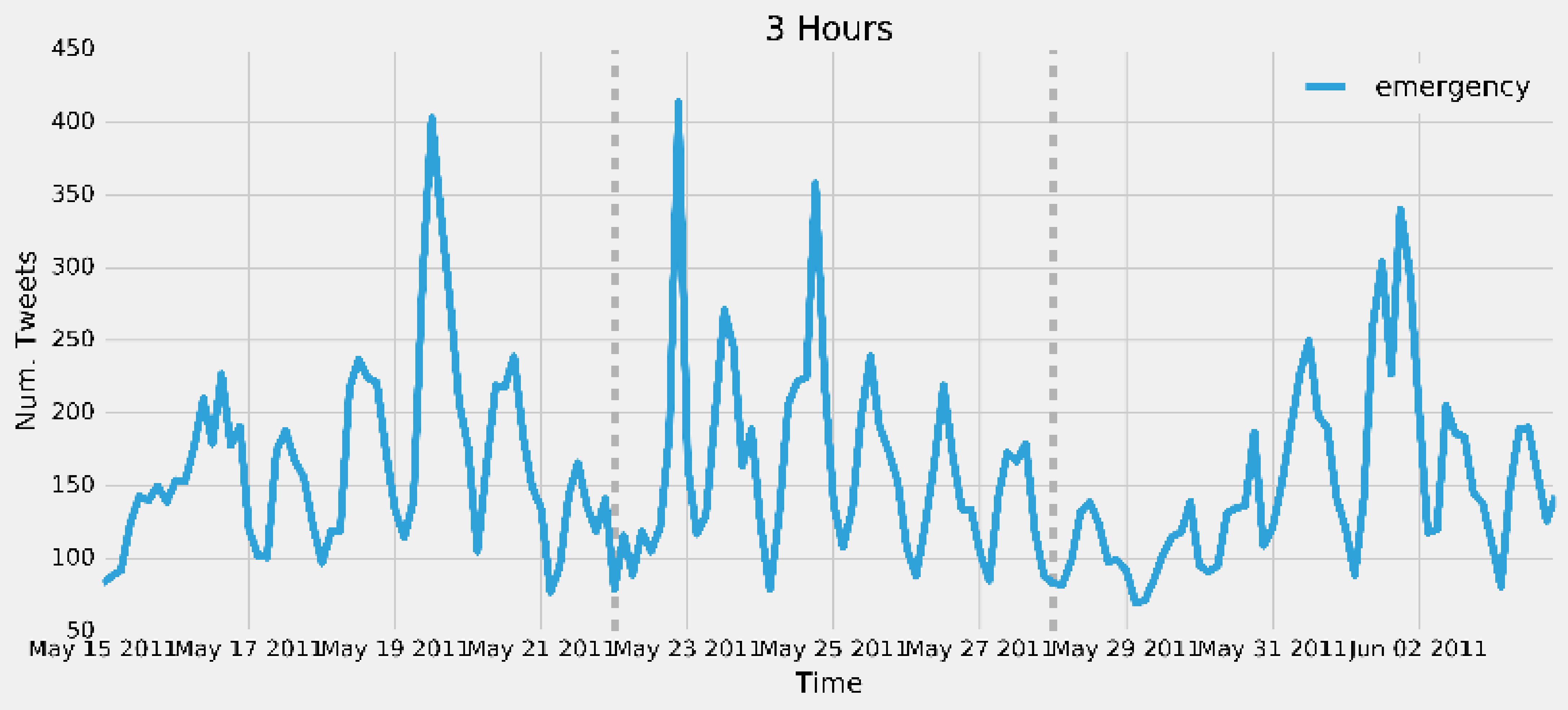

3 Hours
emergency
Num. Tweets
Time
450
400
350
300
250
200
150
100
50
May 15 2011
May 17 2011
May 19 2011
May 21 2011
May 23 2011
May 25 2011
May 27 2011
May 29 2011
May 31 2011
Jun 02 2011

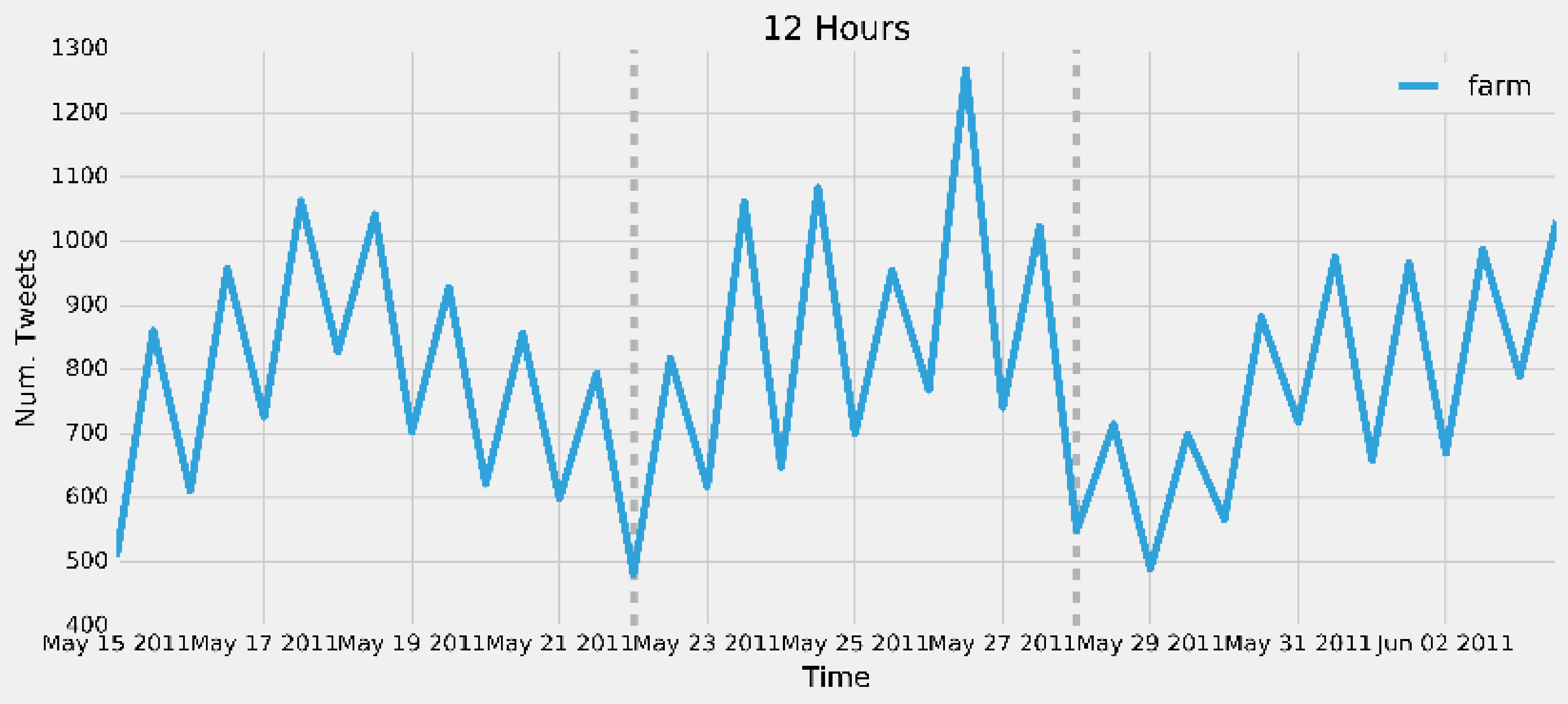

12 Hours
farm
Num. Tweets
1300
1200
1100
1000
900
800
700
600
500
400
May 15 2011
May 17 2011
May 19 2011
May 21 2011
May 23 2011
May 25 2011
May 27 2011
May 29 2011
May 31 2011
Jun 02 2011
Time

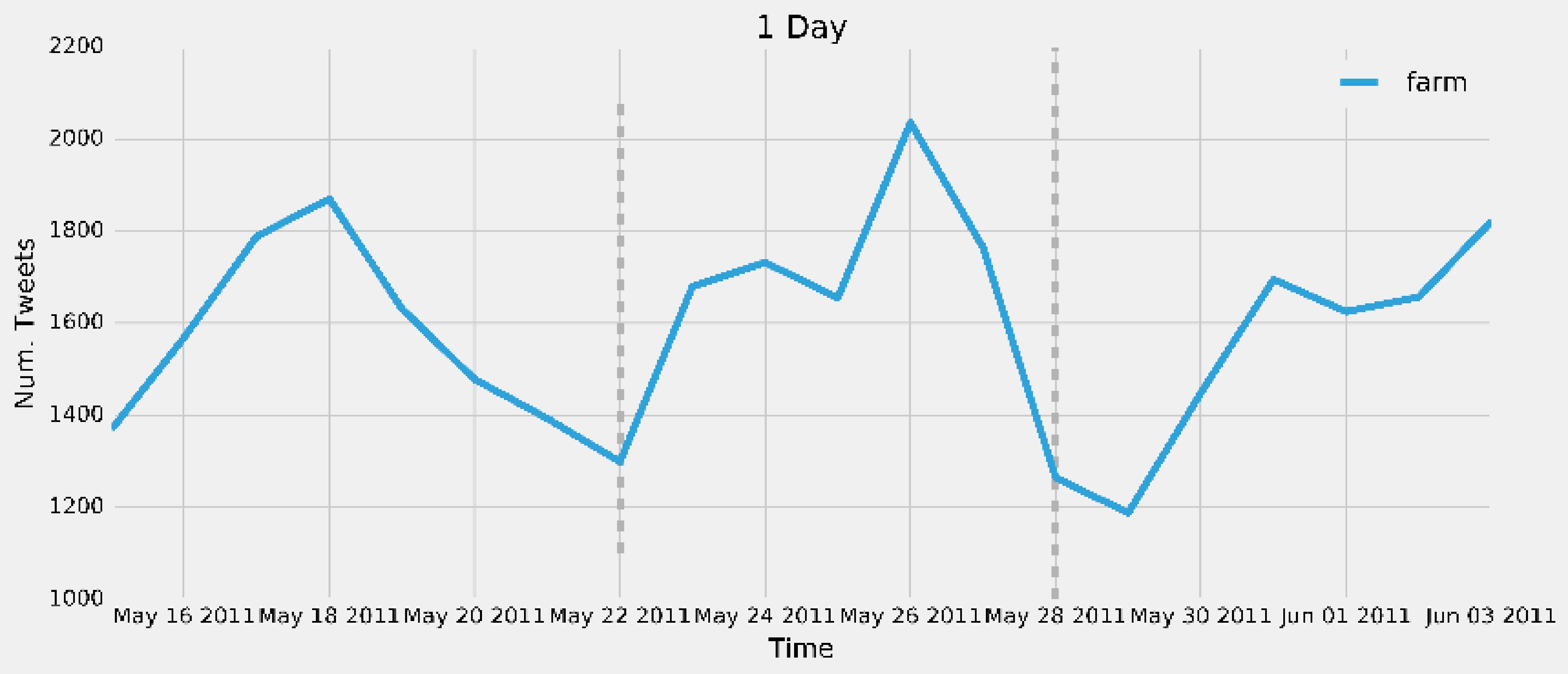

1 Day
farm
Num. Tweets
2200
2000
1800
1600
1400
1200
1000
May 16 2011
May 18 2011
May 20 2011
May 22 2011
May 24 2011
May 26 2011
May 28 2011
May 30 2011
Jun 01 2011
Jun 03 2011
Time

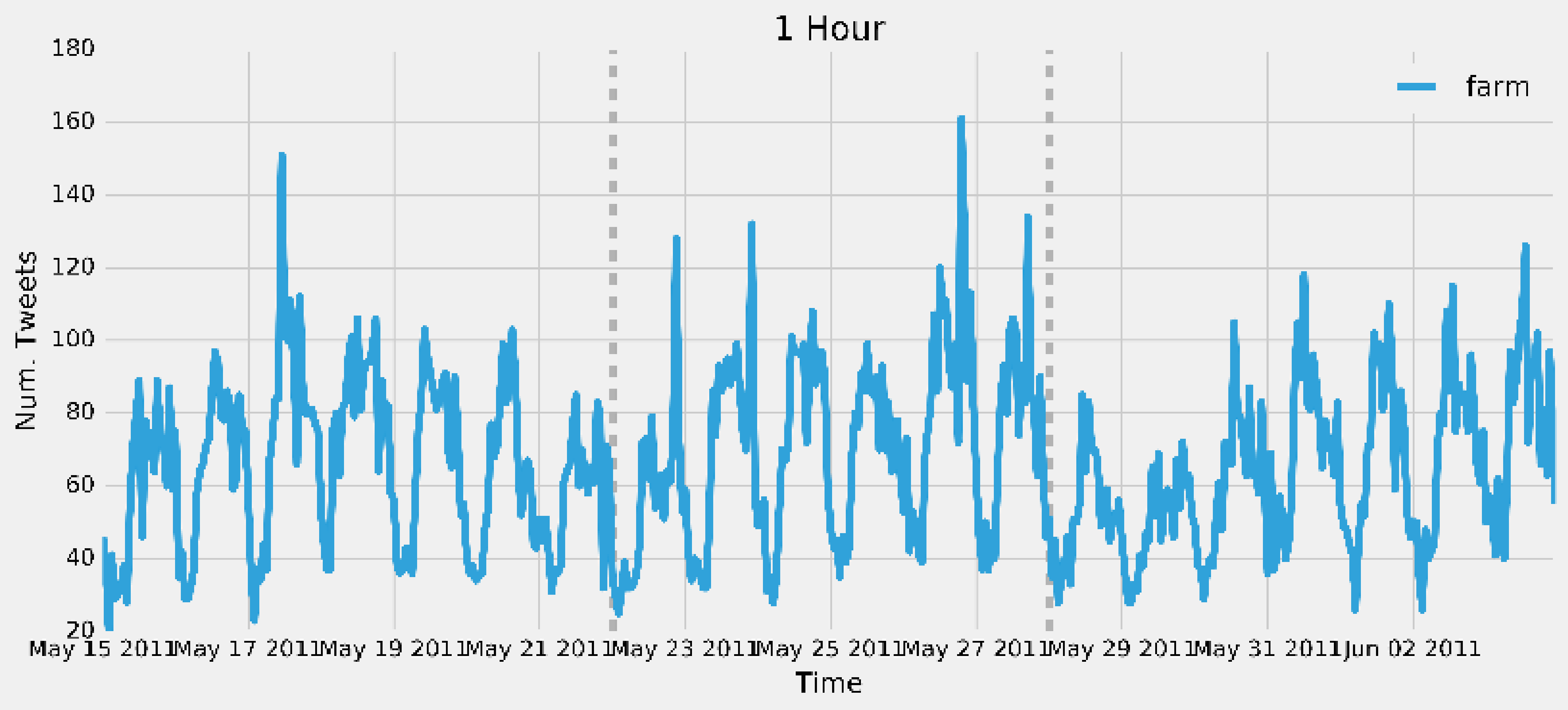
1 Hour
farm
Num. Tweets
180
160
140
120
100
80
60
40
20
May 15 2011
May 17 2011
May 19 2011
May 21 2011
May 23 2011
May 25 2011
May 27 2011
May 29 2011
May 31 2011
Jun 02 2011
Time

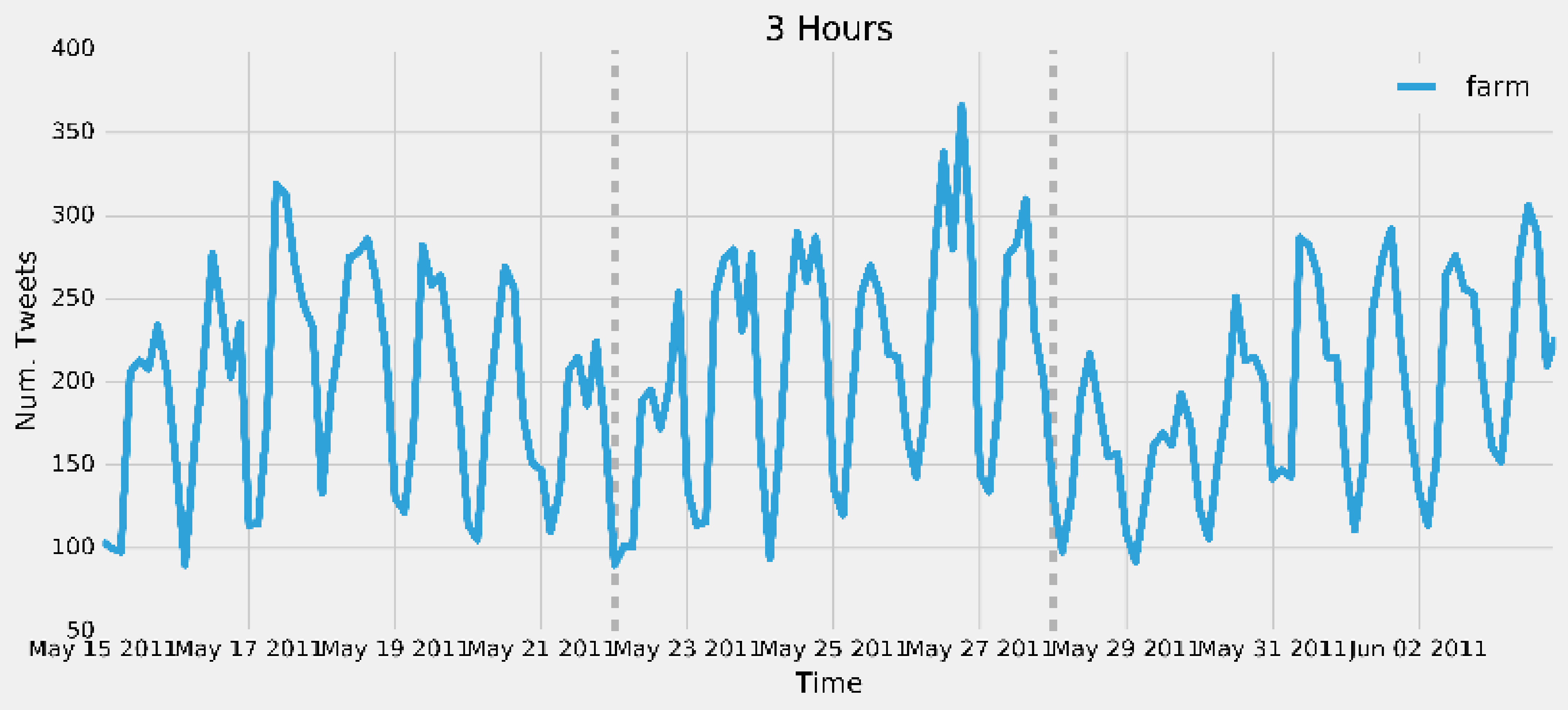

3 Hours
farm
Num. Tweets
400
350
300
250
200
150
100
50
May 15 2011
May 17 2011
May 19 2011
May 21 2011
May 23 2011
May 25 2011
May 27 2011
May 29 2011
May 31 2011
Jun 02 2011
Time

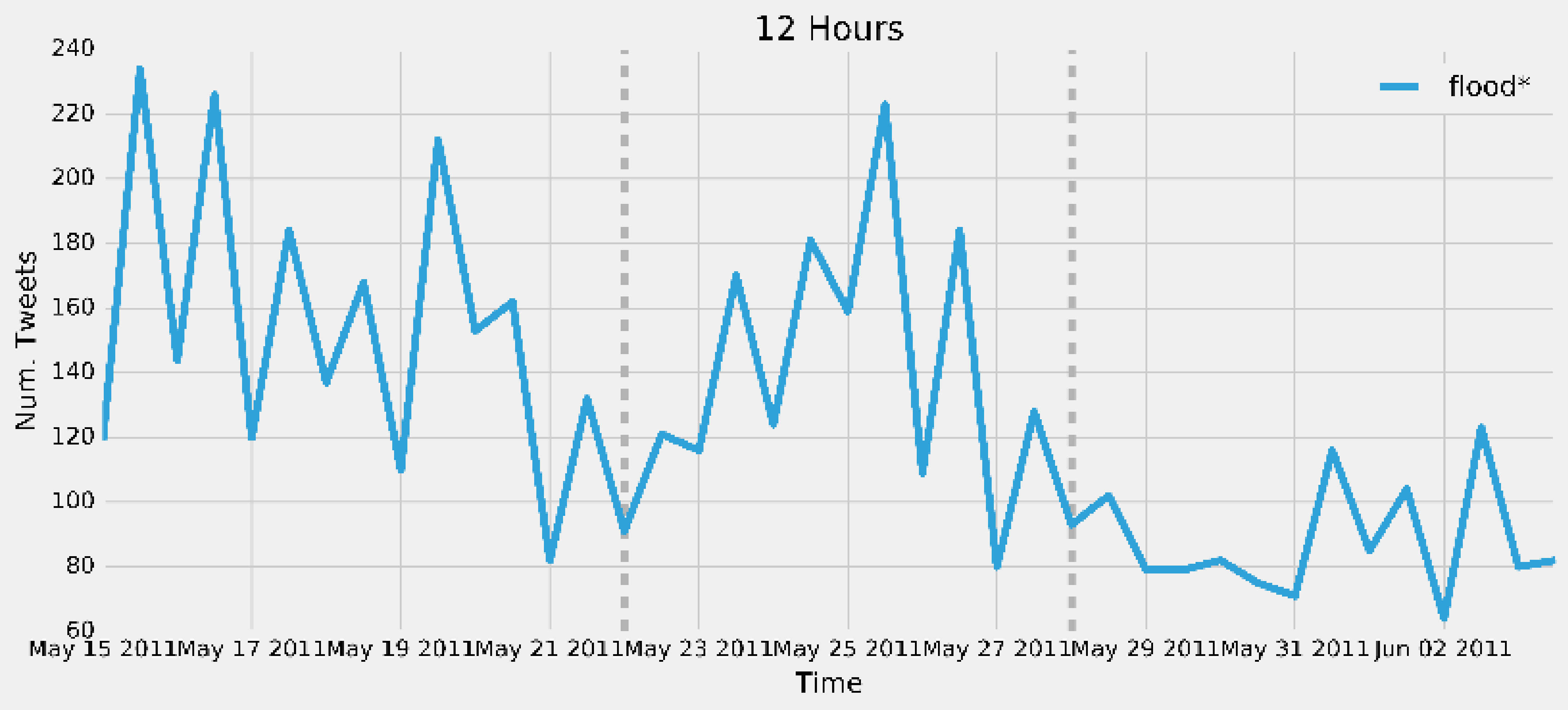
12 Hours
flood*
Num. Tweets
240
220
200
180
160
140
120
100
80
60
May 15 2011
May 17 2011
May 19 2011
May 21 2011
May 23 2011
May 25 2011
May 27 2011
May 29 2011
May 31 2011
Jun 02 2011
Time

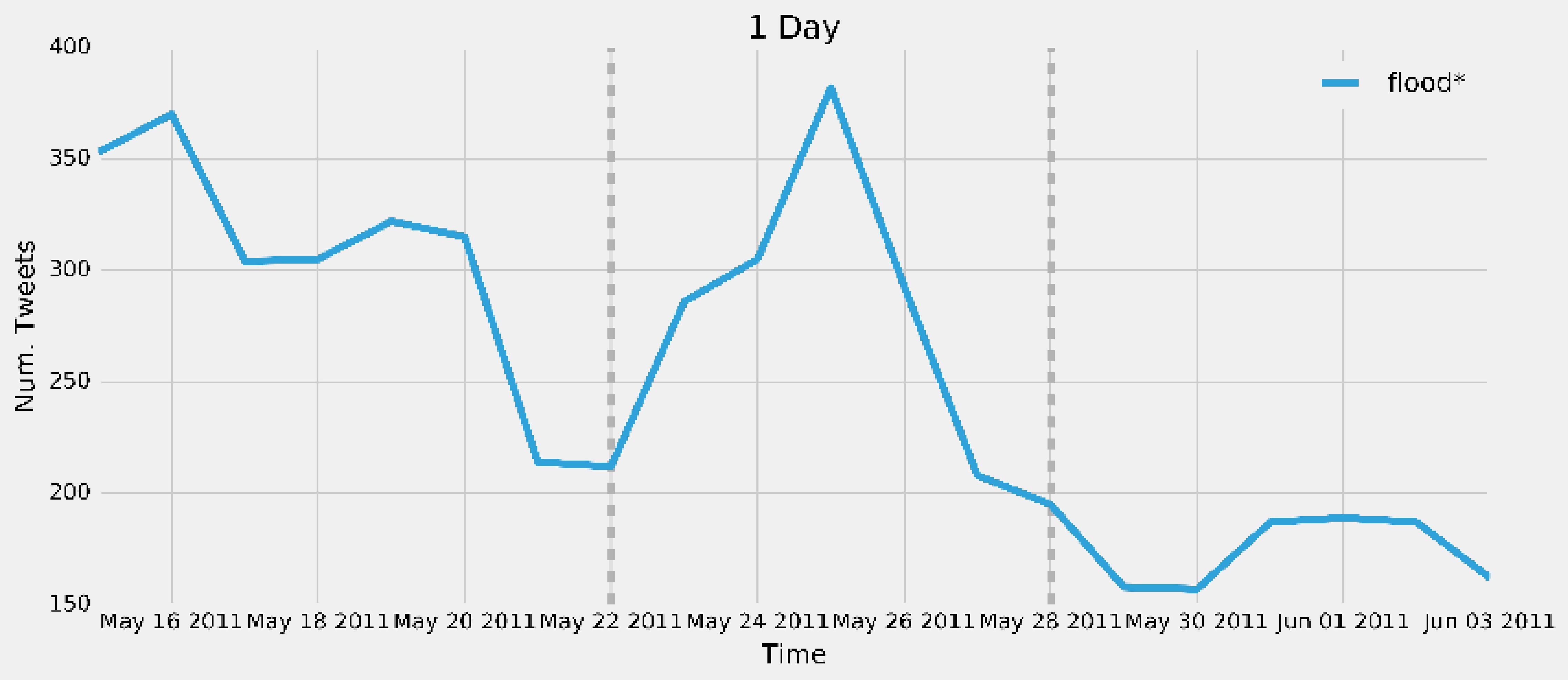

1 Day
flood*
Num. Tweets
400
350
300
250
200
150
May 16 2011
May 18 2011
May 20 2011
May 22 2011
May 24 2011
May 26 2011
May 28 2011
May 30 2011
Jun 01 2011
Jun 03 2011
Time

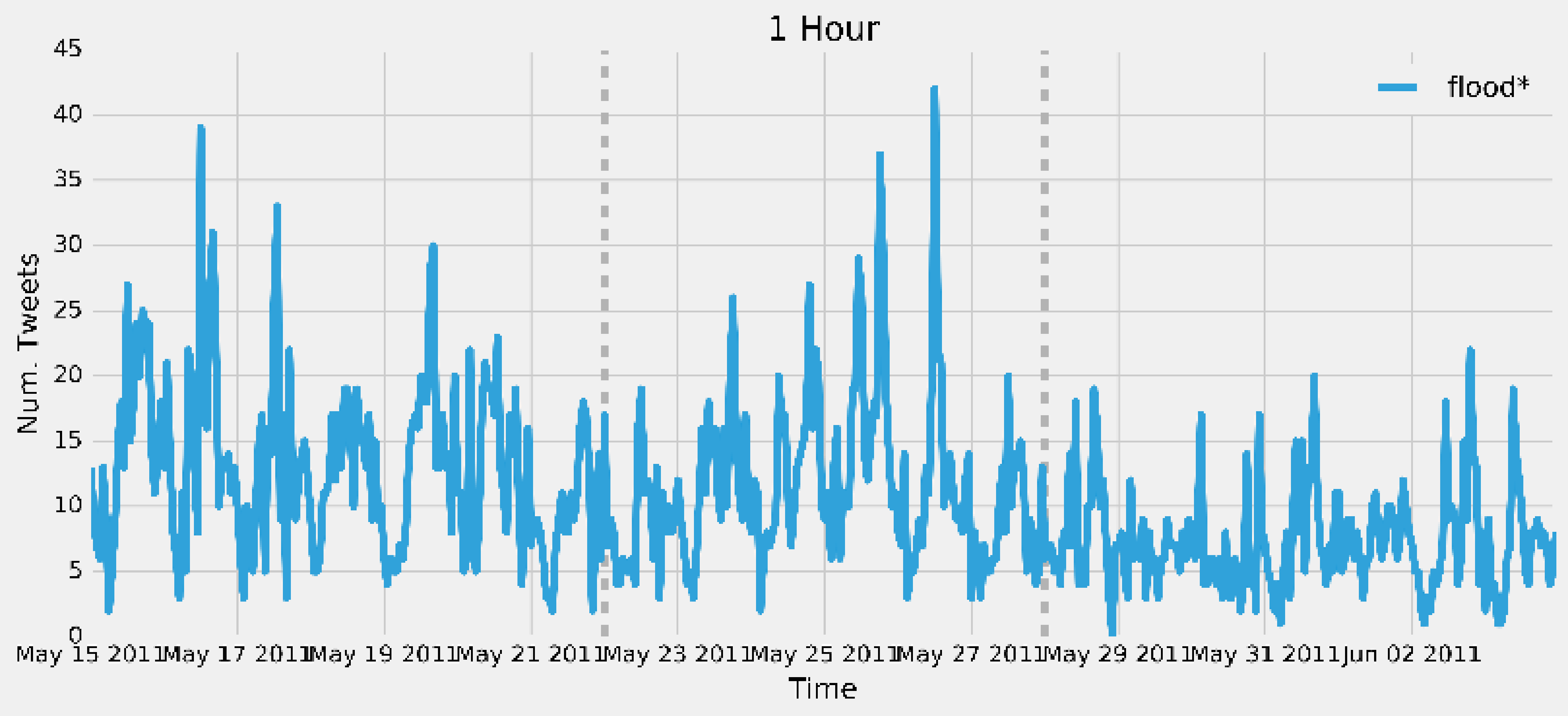
1 Hour
flood*
Num. Tweets
Time
0
5
10
15
20
25
30
35
40
45
May 15 2011
May 17 2011
May 19 2011
May 21 2011
May 23 2011
May 25 2011
May 27 2011
May 29 2011
May 31 2011
Jun 02 2011

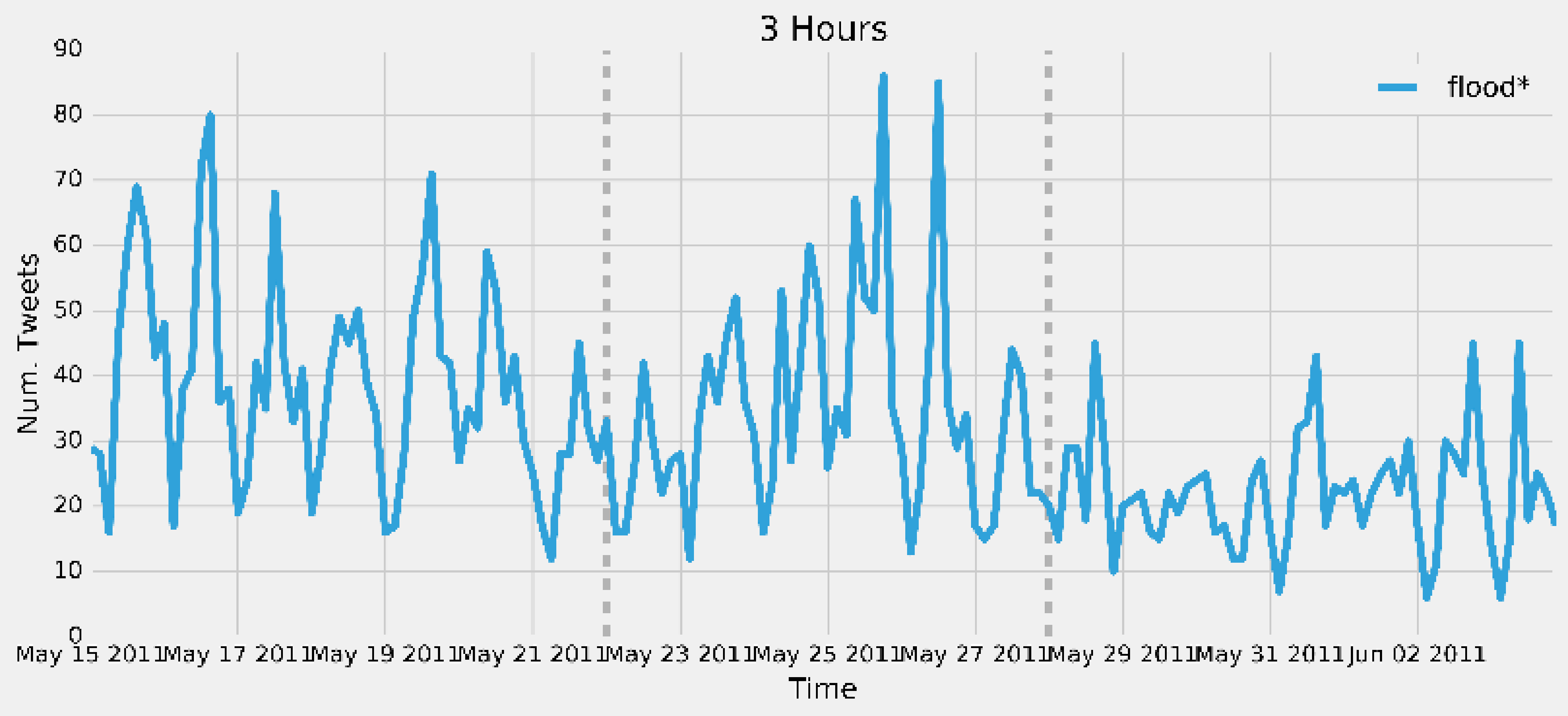
3 Hours
flood*
Num. Tweets
0
10
20
30
40
50
60
70
80
90
May 15 2011
May 17 2011
May 19 2011
May 21 2011
May 23 2011
May 25 2011
May 27 2011
May 29 2011
May 31 2011
Jun 02 2011
Time

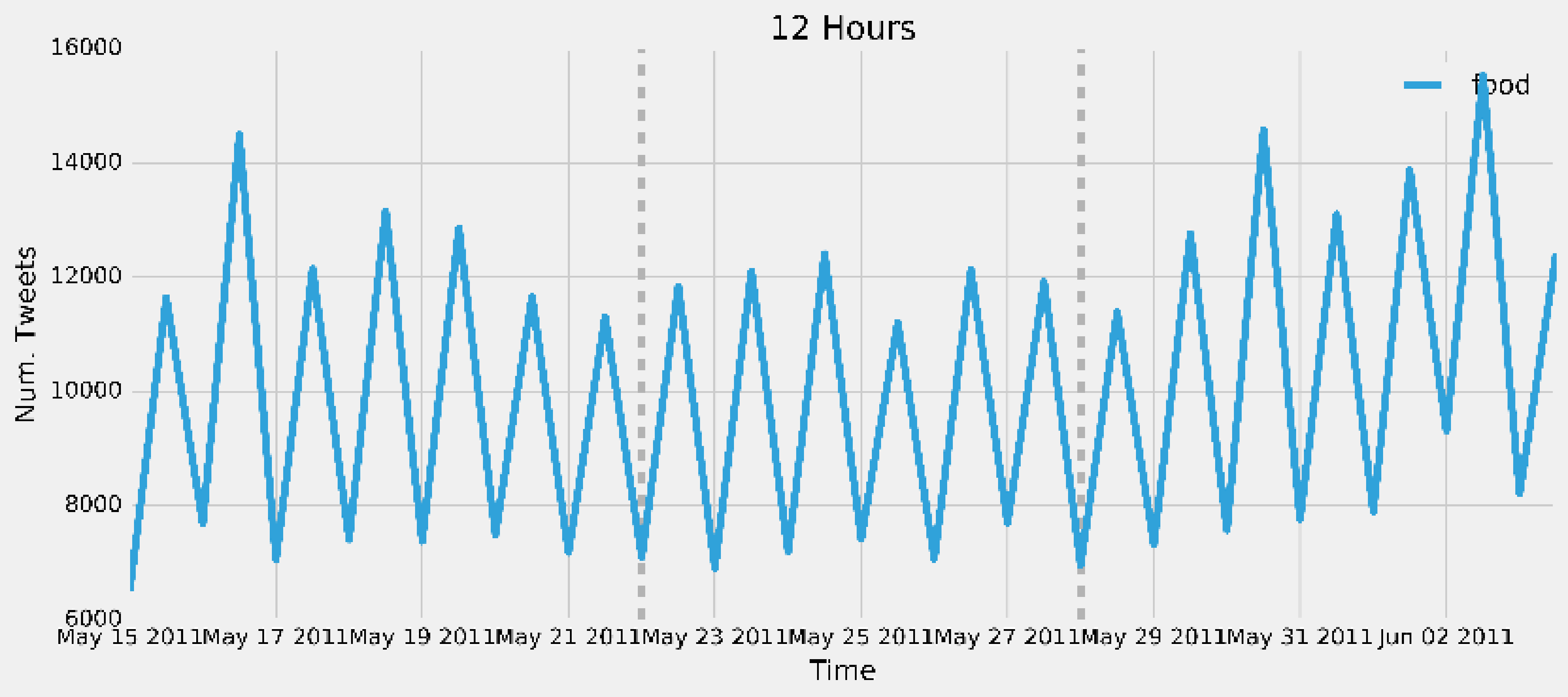

12 Hours
food
Num. Tweets
16000
14000
12000
10000
8000
6000
May 15 2011
May 17 2011
May 19 2011
May 21 2011
May 23 2011
May 25 2011
May 27 2011
May 29 2011
May 31 2011
Jun 02 2011
Time

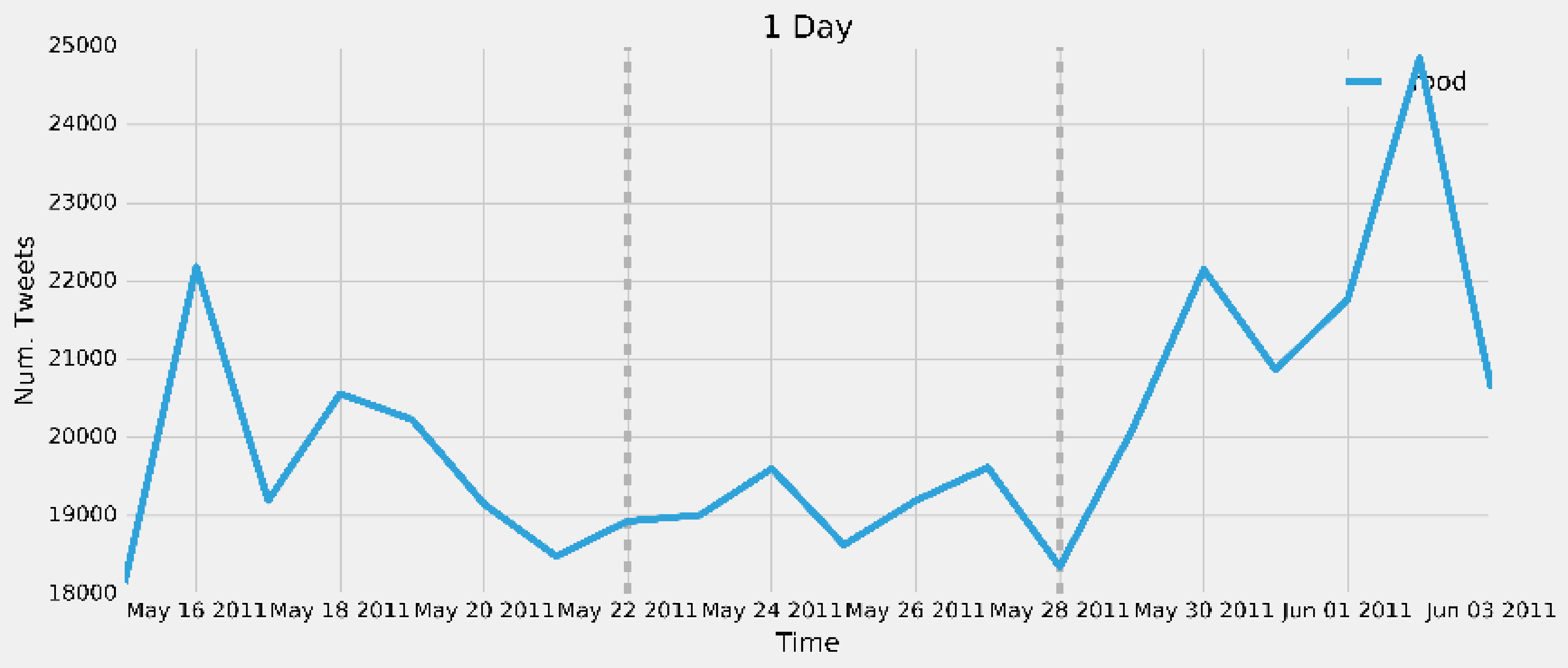
1 Day
Num. Tweets
25000
24000
23000
22000
21000
20000
19000
18000
May 16 2011
May 18 2011
May 20 2011
May 22 2011
May 24 2011
May 26 2011
May 28 2011
May 30 2011
Jun 01 2011
Jun 03 2011
Time

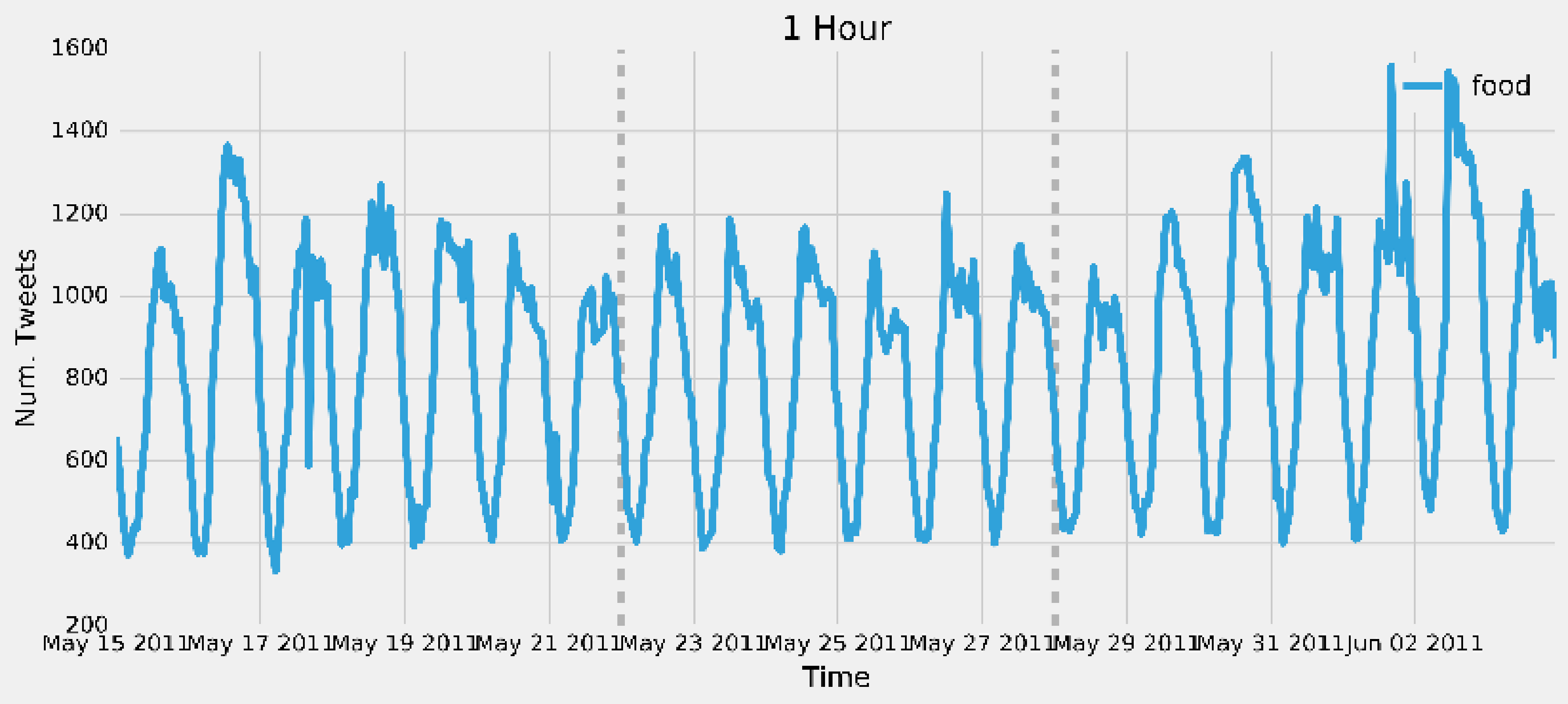

1 Hour
food
Num. Tweets
1600
1400
1200
1000
800
600
400
200
May 15 2011
May 17 2011
May 19 2011
May 21 2011
May 23 2011
May 25 2011
May 27 2011
May 29 2011
May 31 2011
Jun 02 2011
Time

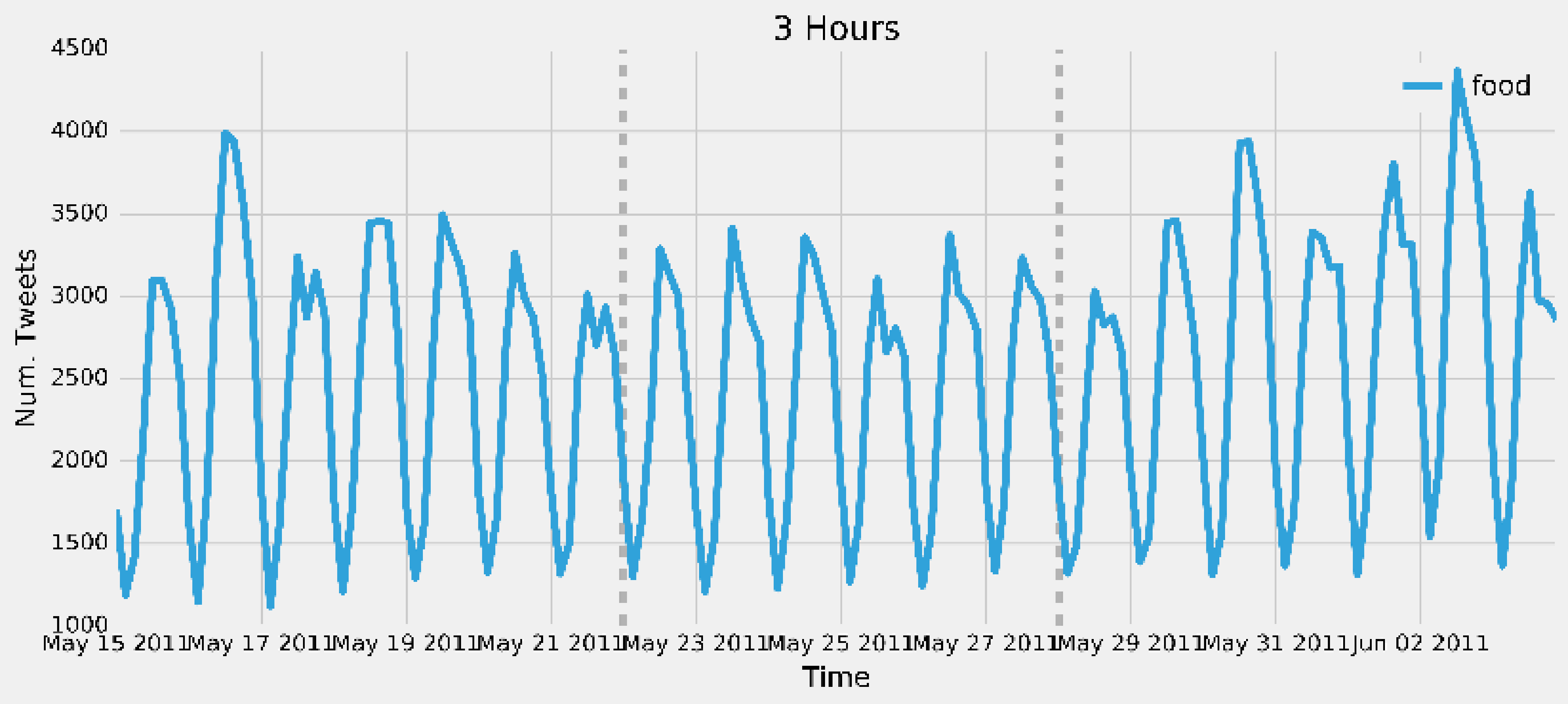

3 Hours
food
4500
4000
3500
3000
2500
2000
1500
1000
Num. Tweets
May 15 2011
May 17 2011
May 19 2011
May 21 2011
May 23 2011
May 25 2011
May 27 2011
May 29 2011
May 31 2011
Jun 02 2011
Time

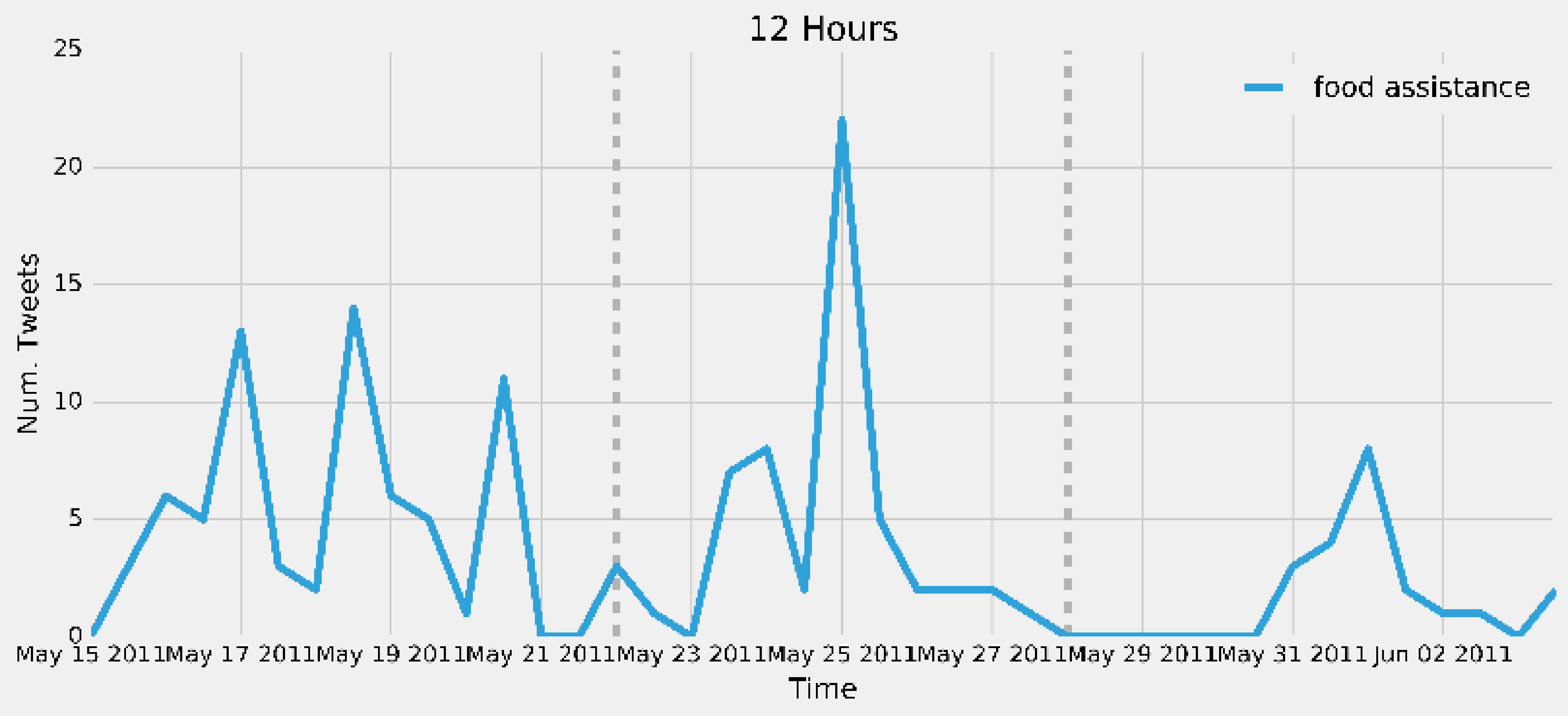
12 Hours
food assistance
Num. Tweets
0
5
10
15
20
25
May 15 2011
May 17 2011
May 19 2011
May 21 2011
May 23 2011
May 25 2011
May 27 2011
May 29 2011
May 31 2011
Jun 02 2011
Time

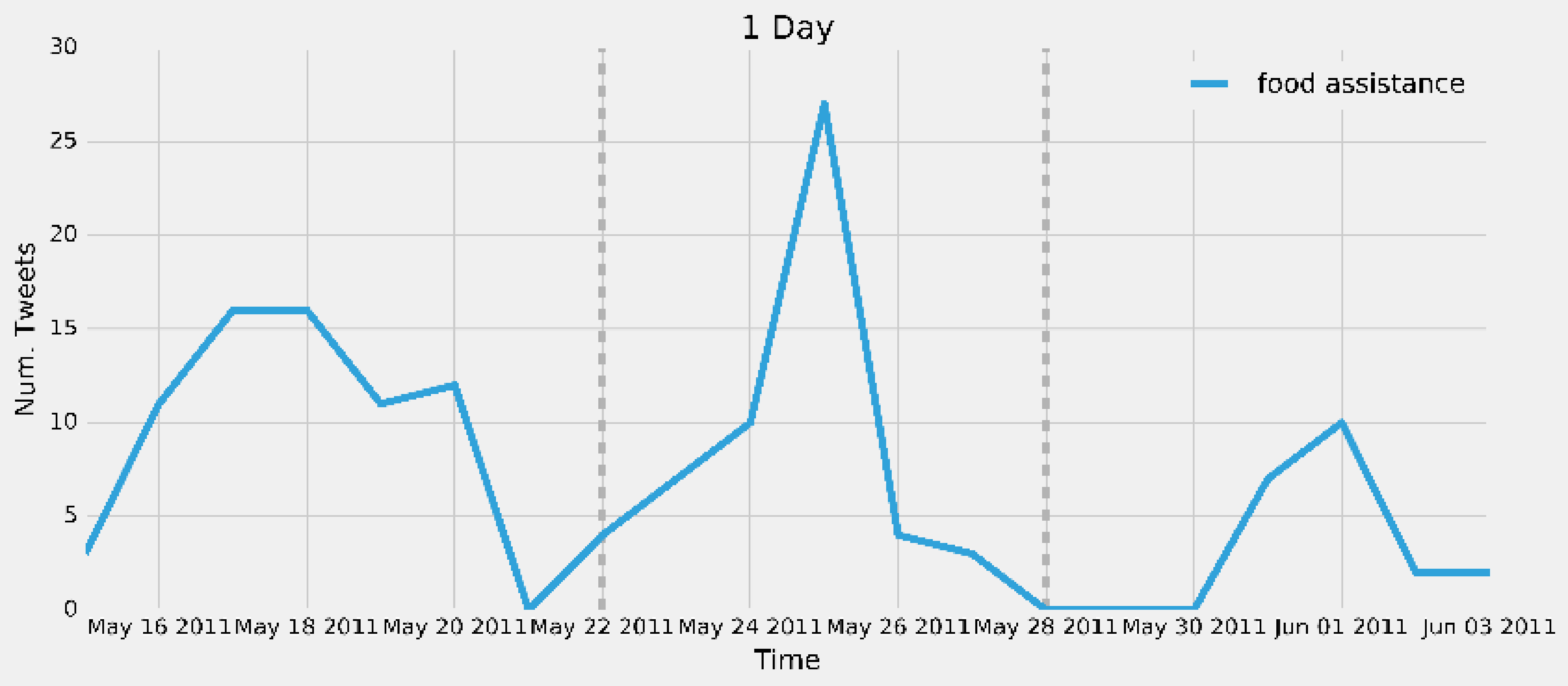

1 Day
food assistance
Num. Tweets
Time
0
5
10
15
20
25
30
May 16 2011
May 18 2011
May 20 2011
May 22 2011
May 24 2011
May 26 2011
May 28 2011
May 30 2011
Jun 01 2011
Jun 03 2011

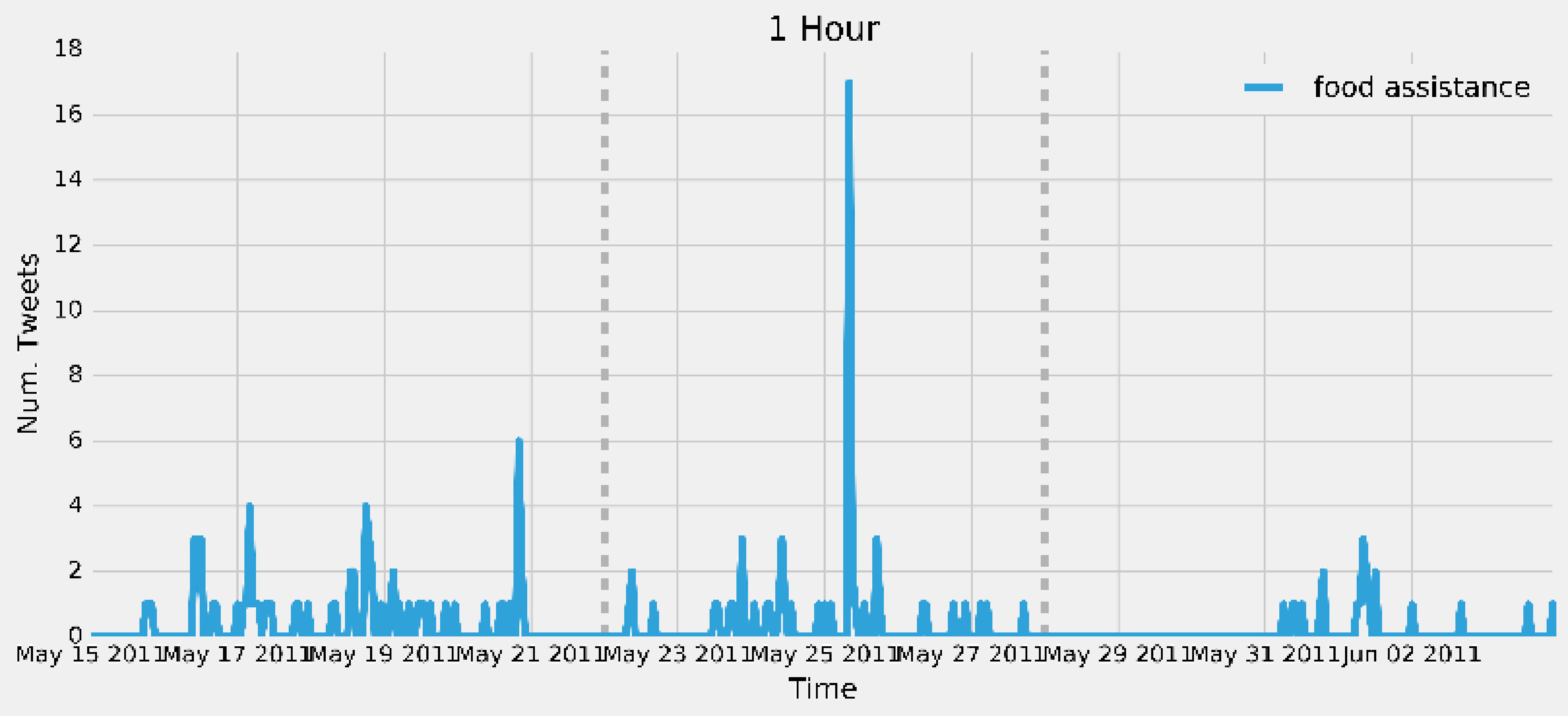

1 Hour
food assistance
Num. Tweets
Time
0
2
4
6
8
10
12
14
16
18
May 15 2011
May 17 2011
May 19 2011
May 21 2011
May 23 2011
May 25 2011
May 27 2011
May 29 2011
May 31 2011
Jun 02 2011

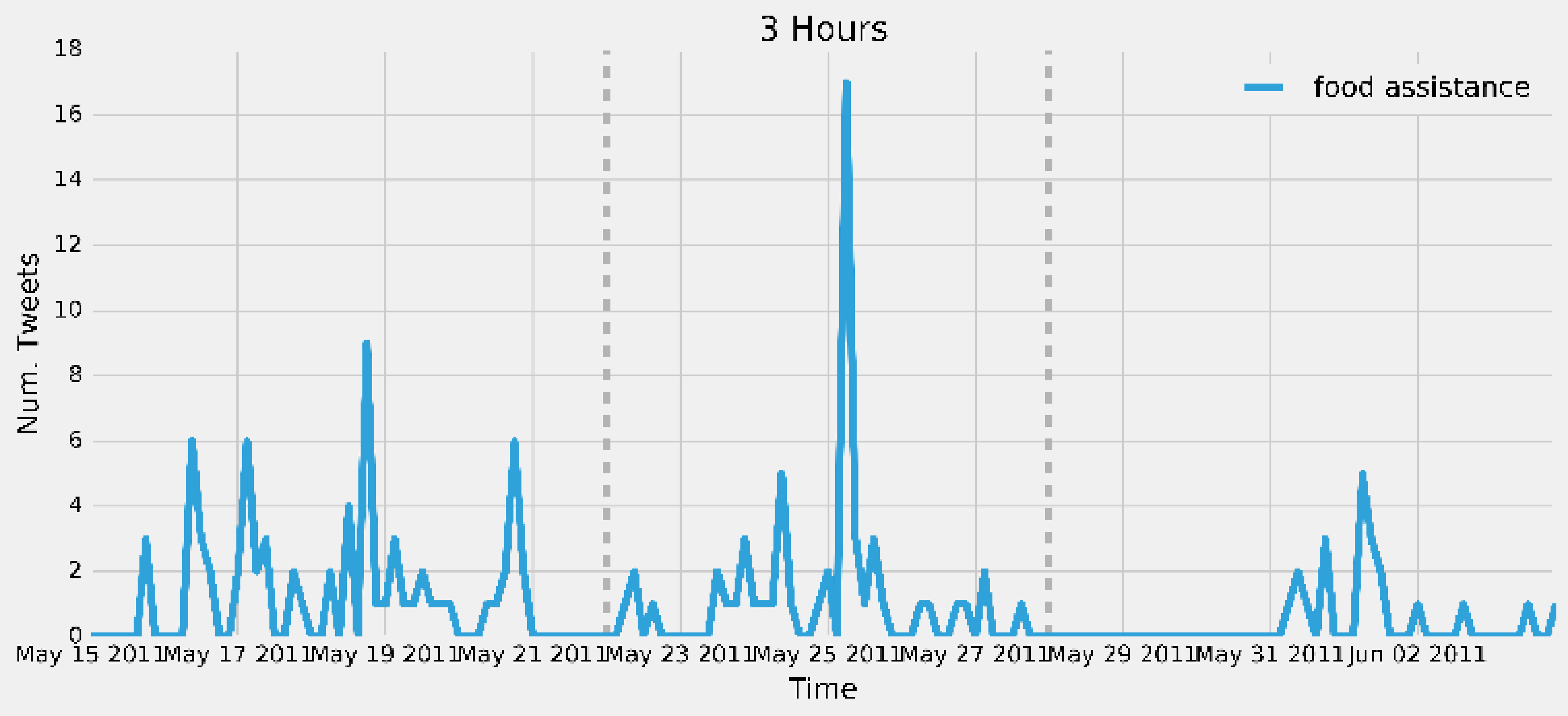

3 Hours
food assistance
Num. Tweets
0
2
4
6
8
10
12
14
16
18
May 15 2011
May 17 2011
May 19 2011
May 21 2011
May 23 2011
May 25 2011
May 27 2011
May 29 2011
May 31 2011
Jun 02 2011
Time

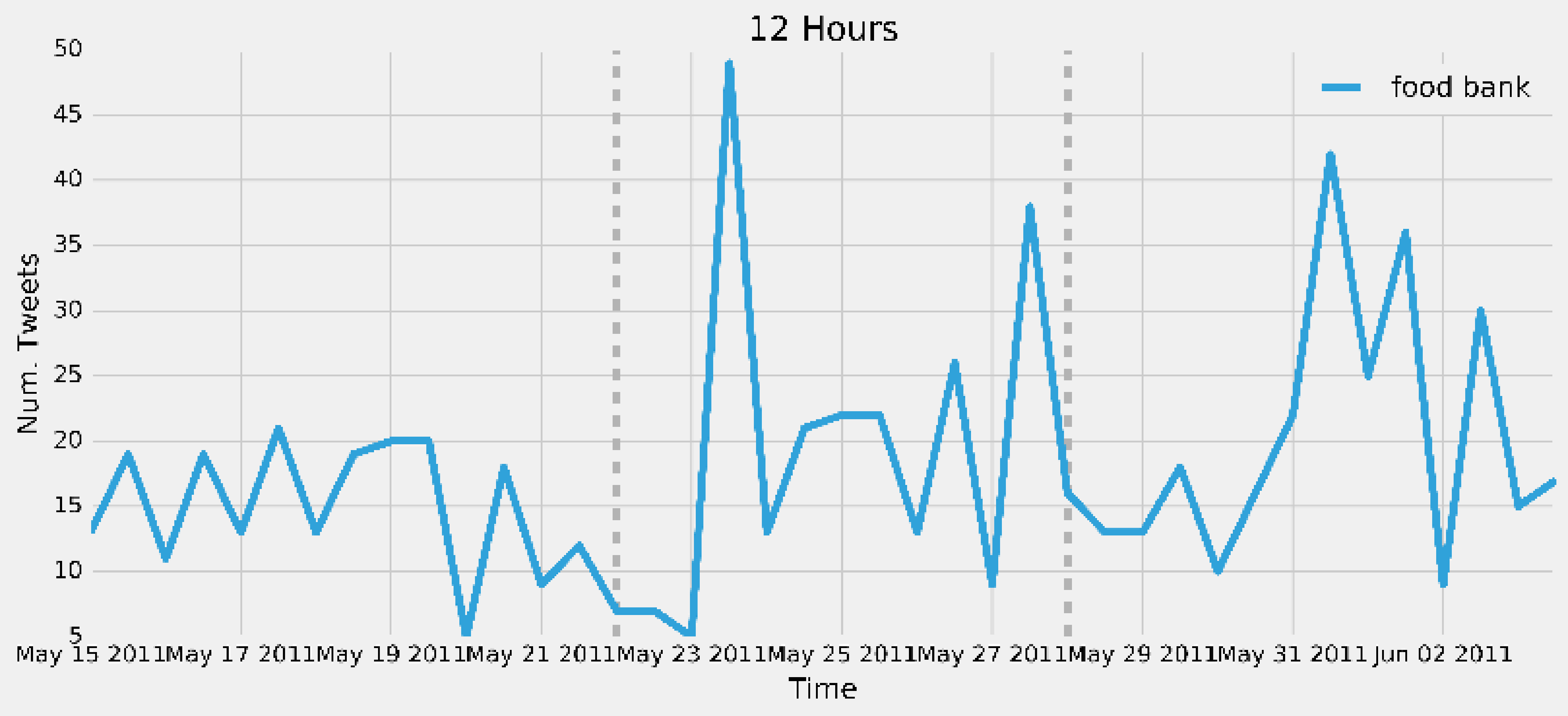

12 Hours
food bank
Num. Tweets
50
45
40
35
30
25
20
15
10
5
May 15 2011
May 17 2011
May 19 2011
May 21 2011
May 23 2011
May 25 2011
May 27 2011
May 29 2011
May 31 2011
Jun 02 2011
Time

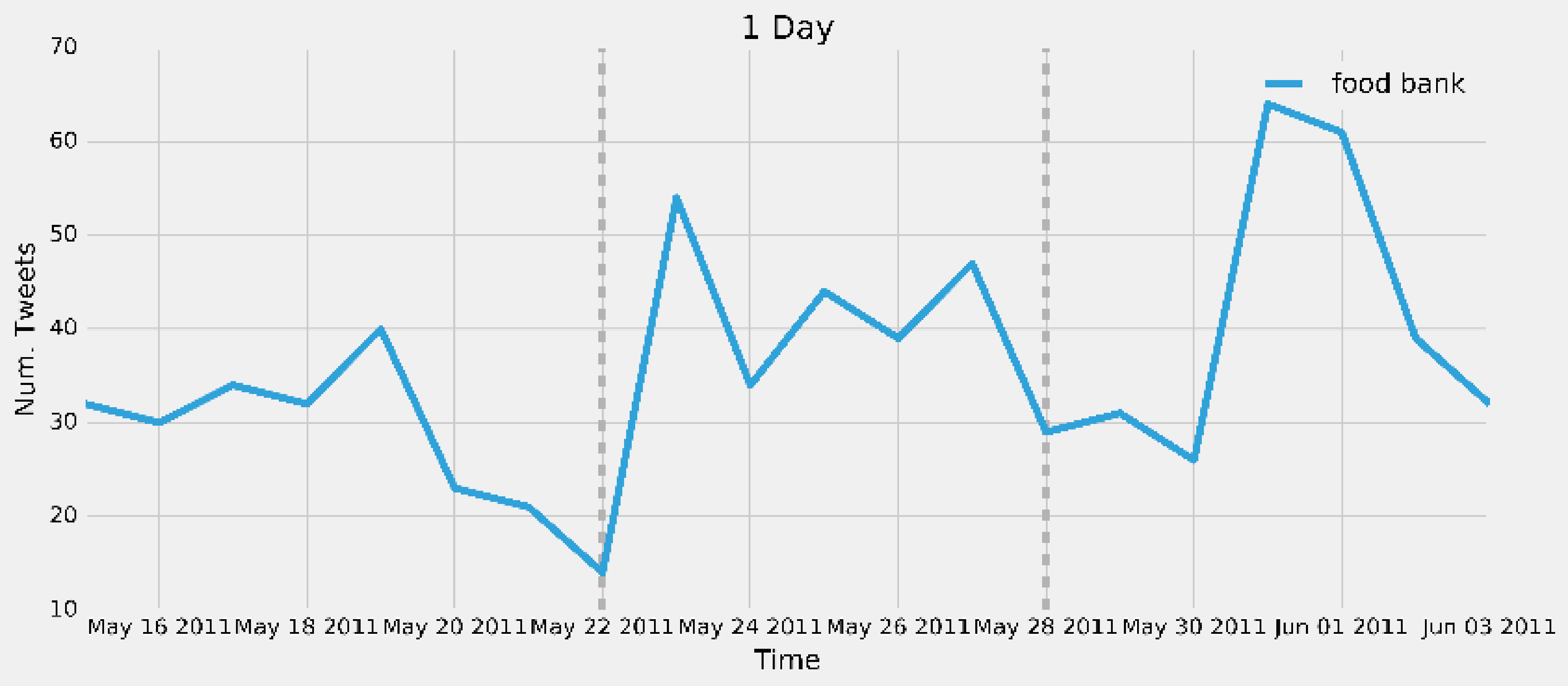

1 Day
food bank
Num. Tweets
70
60
50
40
30
20
10
May 16 2011
May 18 2011
May 20 2011
May 22 2011
May 24 2011
May 26 2011
May 28 2011
May 30 2011
Jun 01 2011
Jun 03 2011
Time

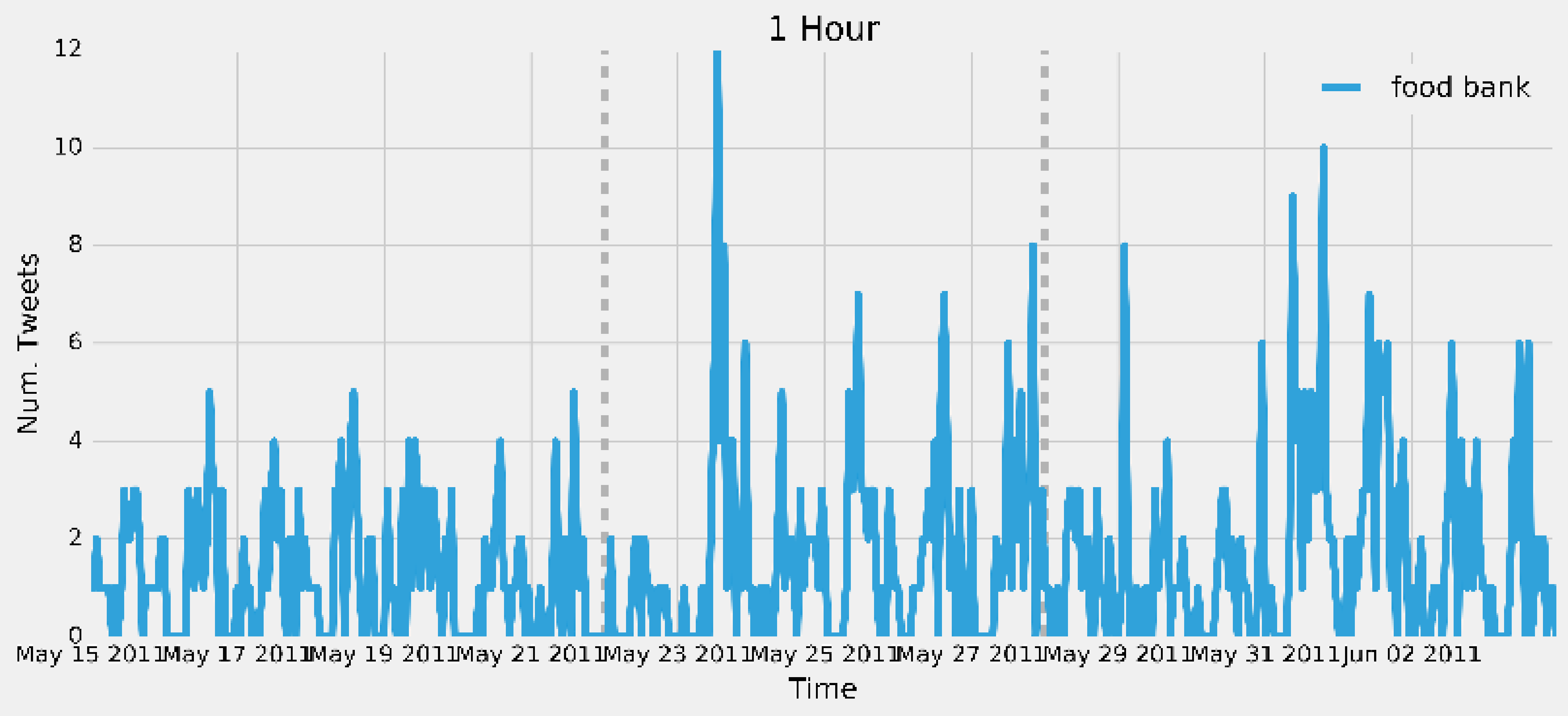
1 Hour
food bank
Num. Tweets
Time
0
2
4
6
8
10
12
May 15 2011
May 17 2011
May 19 2011
May 21 2011
May 23 2011
May 25 2011
May 27 2011
May 29 2011
May 31 2011
Jun 02 2011

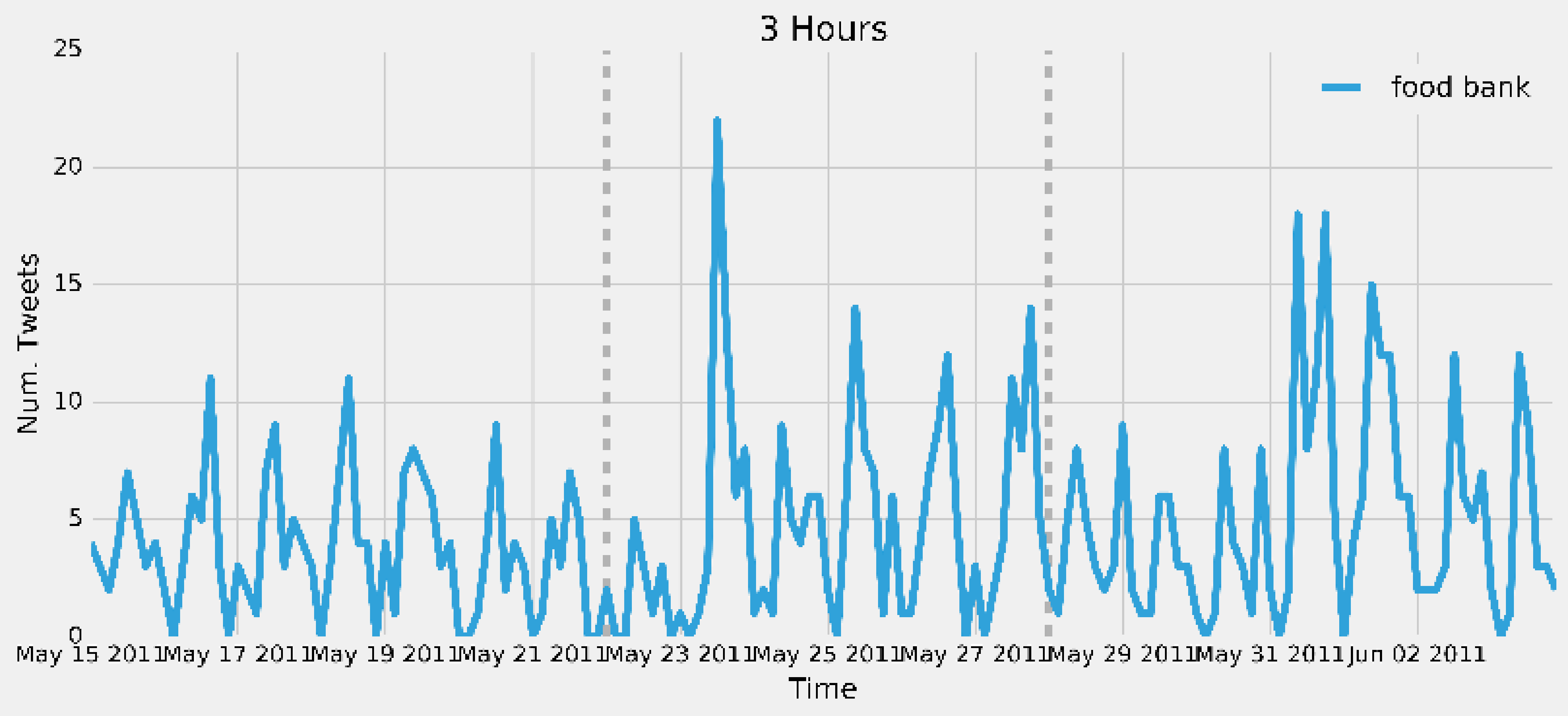

3 Hours
food bank
Num. Tweets
0
5
10
15
20
25
May 15 2011
May 17 2011
May 19 2011
May 21 2011
May 23 2011
May 25 2011
May 27 2011
May 29 2011
May 31 2011
Jun 02 2011
Time

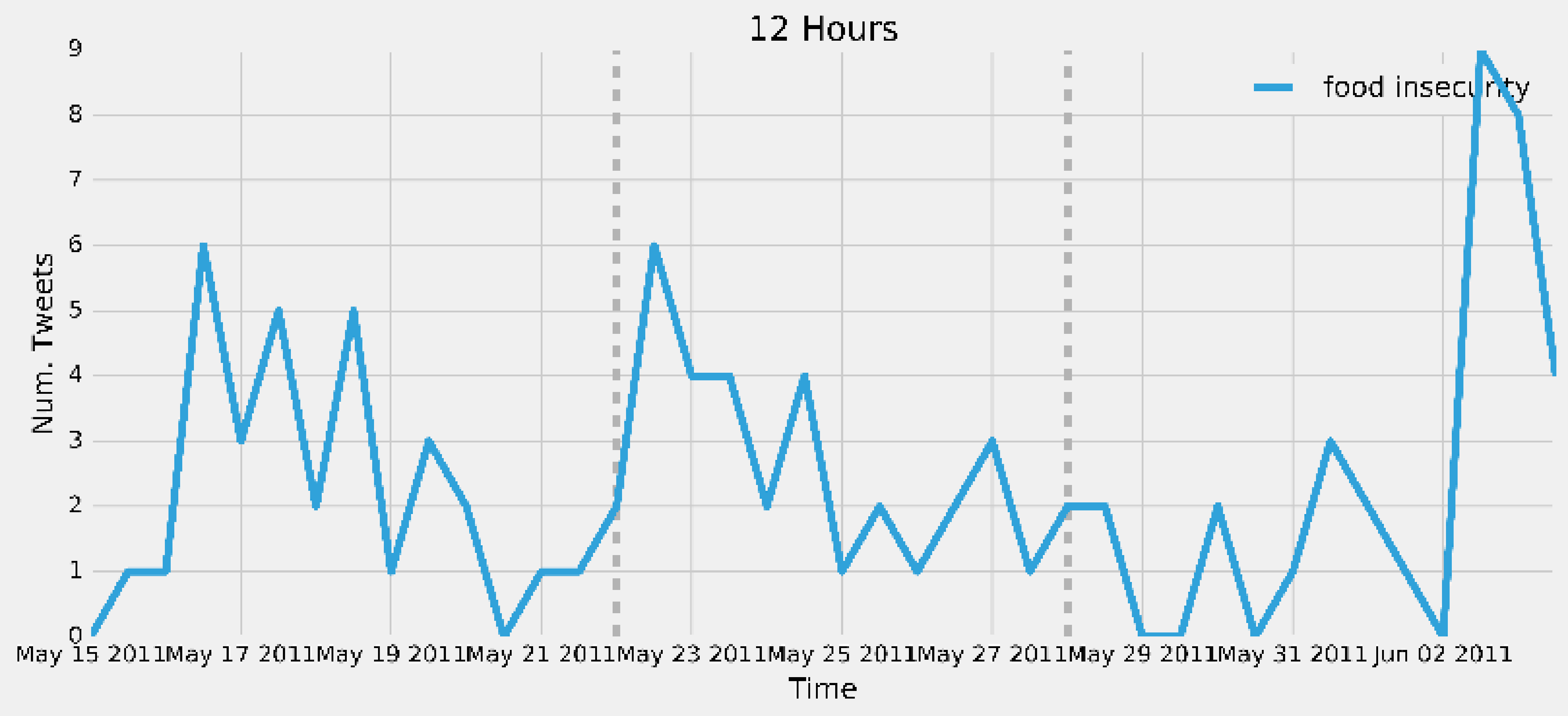
12 Hours
food insecurity
Num. Tweets
0
1
2
3
4
5
6
7
8
9
May 15 2011
May 17 2011
May 19 2011
May 21 2011
May 23 2011
May 25 2011
May 27 2011
May 29 2011
May 31 2011
Jun 02 2011
Time

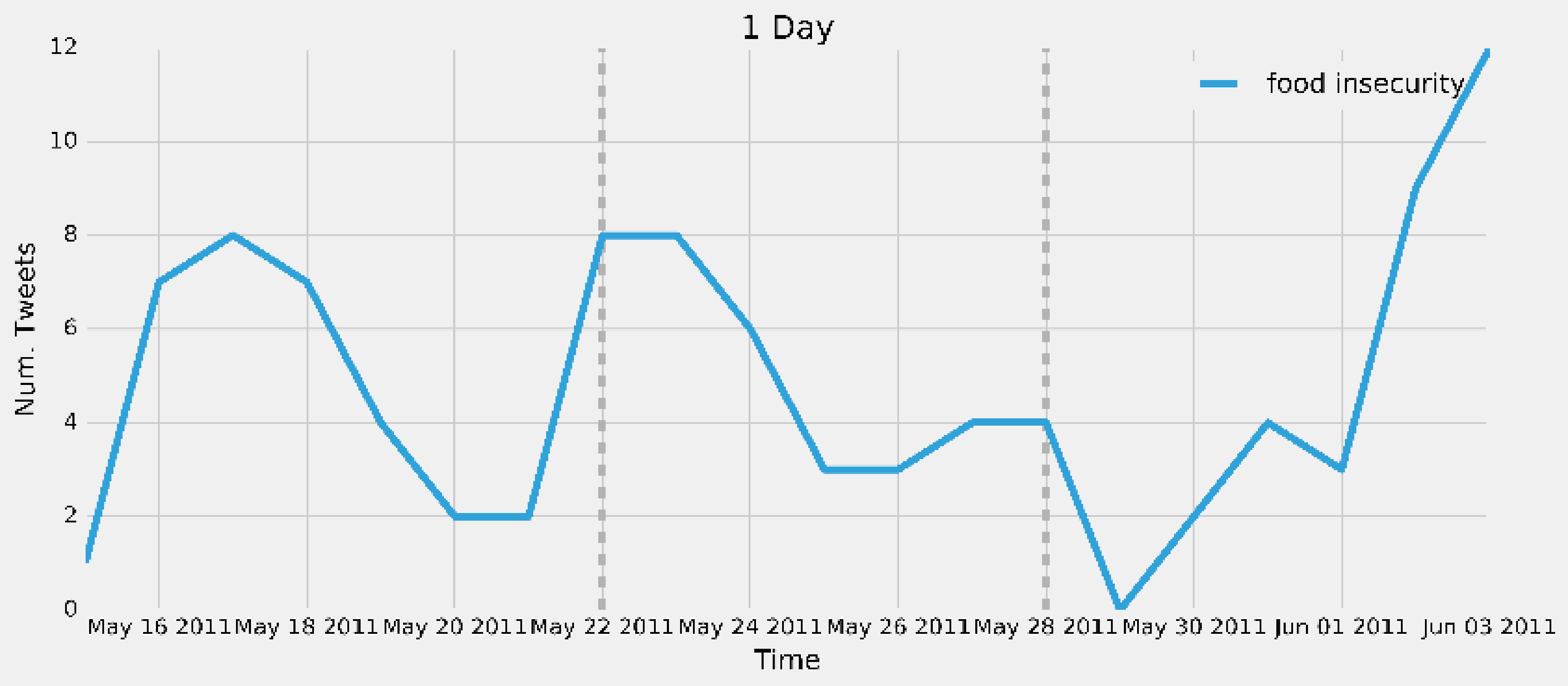
1 Day
food insecurity
Num. Tweets
0
2
4
6
8
10
12
May 16 2011
May 18 2011
May 20 2011
May 22 2011
May 24 2011
May 26 2011
May 28 2011
May 30 2011
Jun 01 2011
Jun 03 2011
Time

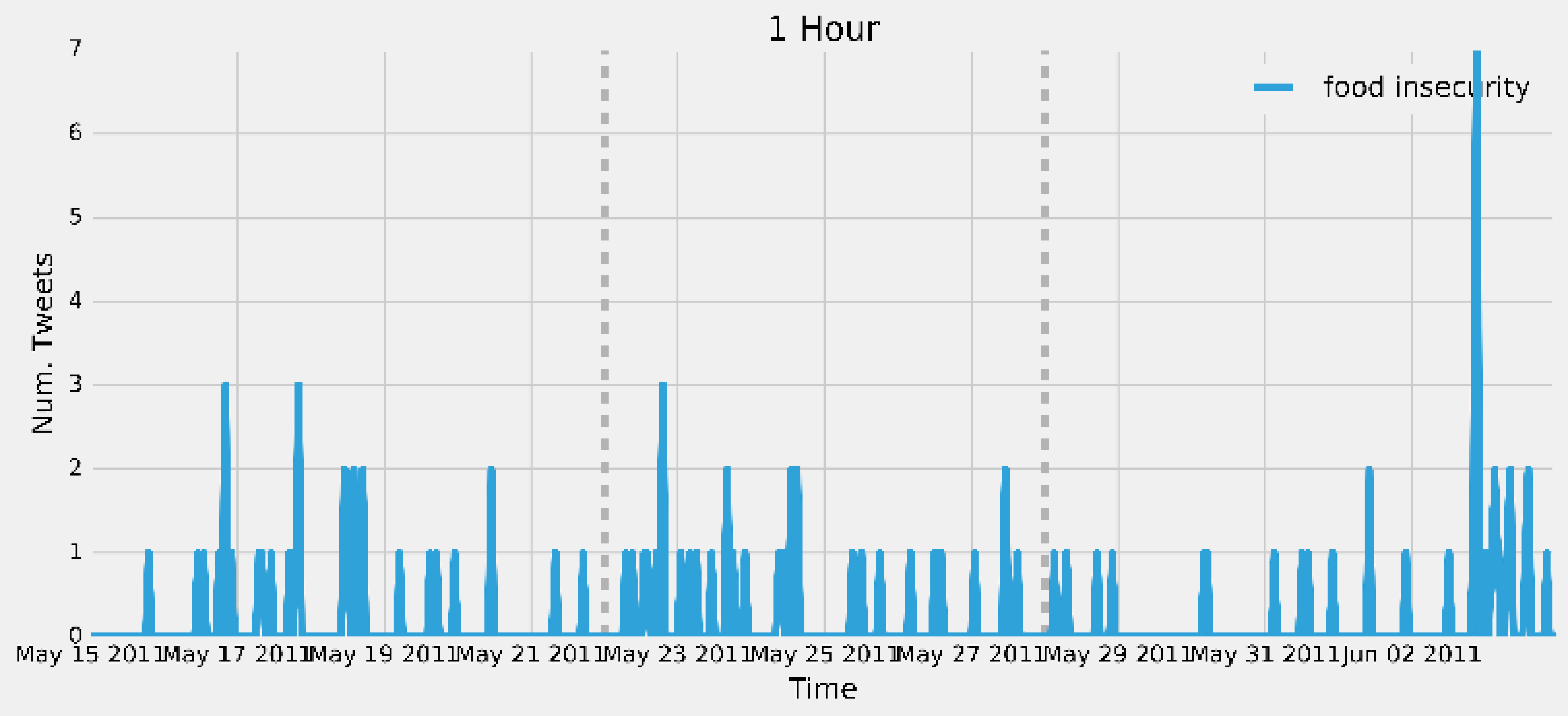
1 Hour
food insecurity
Num. Tweets
0
1
2
3
4
5
6
7
May 15 2011
May 17 2011
May 19 2011
May 21 2011
May 23 2011
May 25 2011
May 27 2011
May 29 2011
May 31 2011
Jun 02 2011
Time

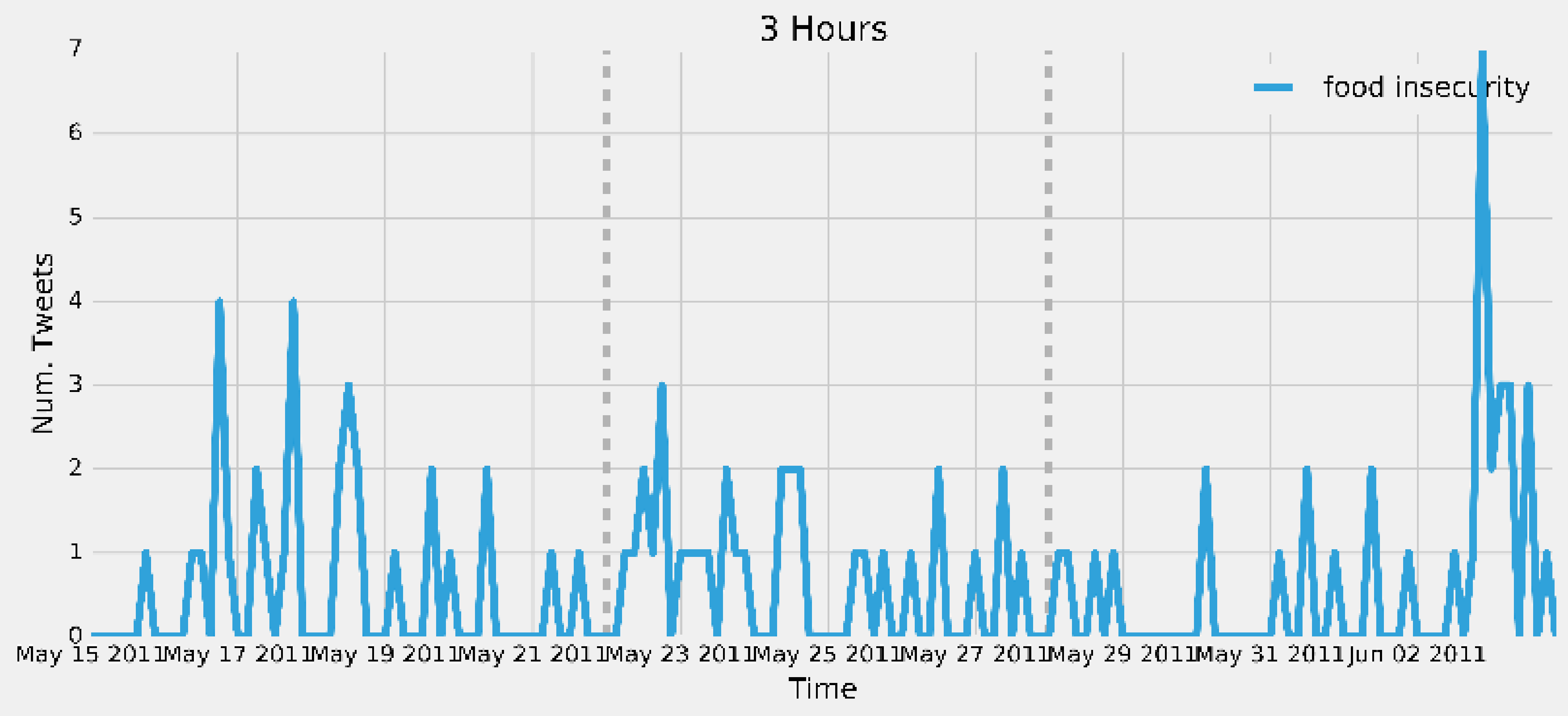

3 Hours
food insecurity
Num. Tweets
Time
0
1
2
3
4
5
6
7
May 15 2011
May 17 2011
May 19 2011
May 21 2011
May 23 2011
May 25 2011
May 27 2011
May 29 2011
May 31 2011
Jun 02 2011

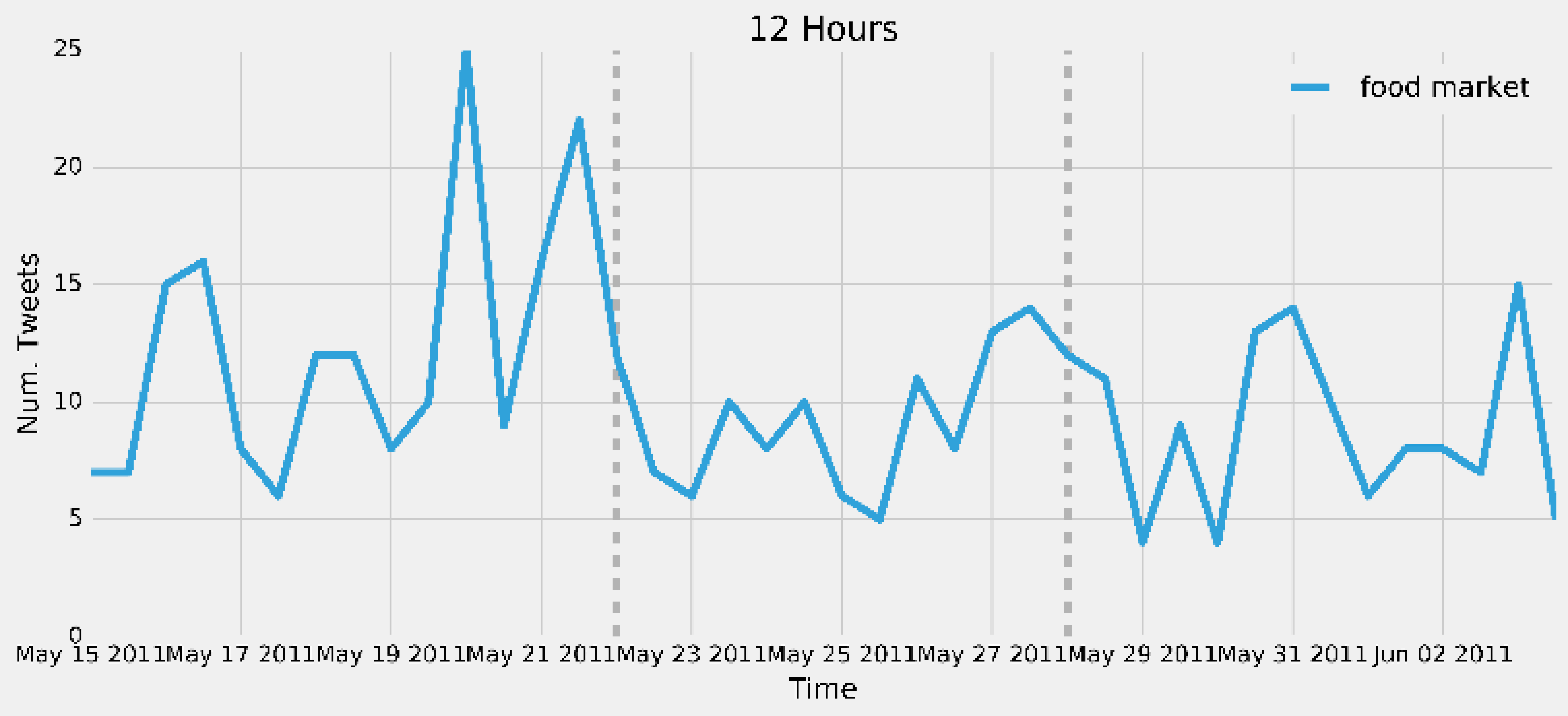
12 Hours
food market
Num. Tweets
0
5
10
15
20
25
May 15 2011
May 17 2011
May 19 2011
May 21 2011
May 23 2011
May 25 2011
May 27 2011
May 29 2011
May 31 2011
Jun 02 2011
Time

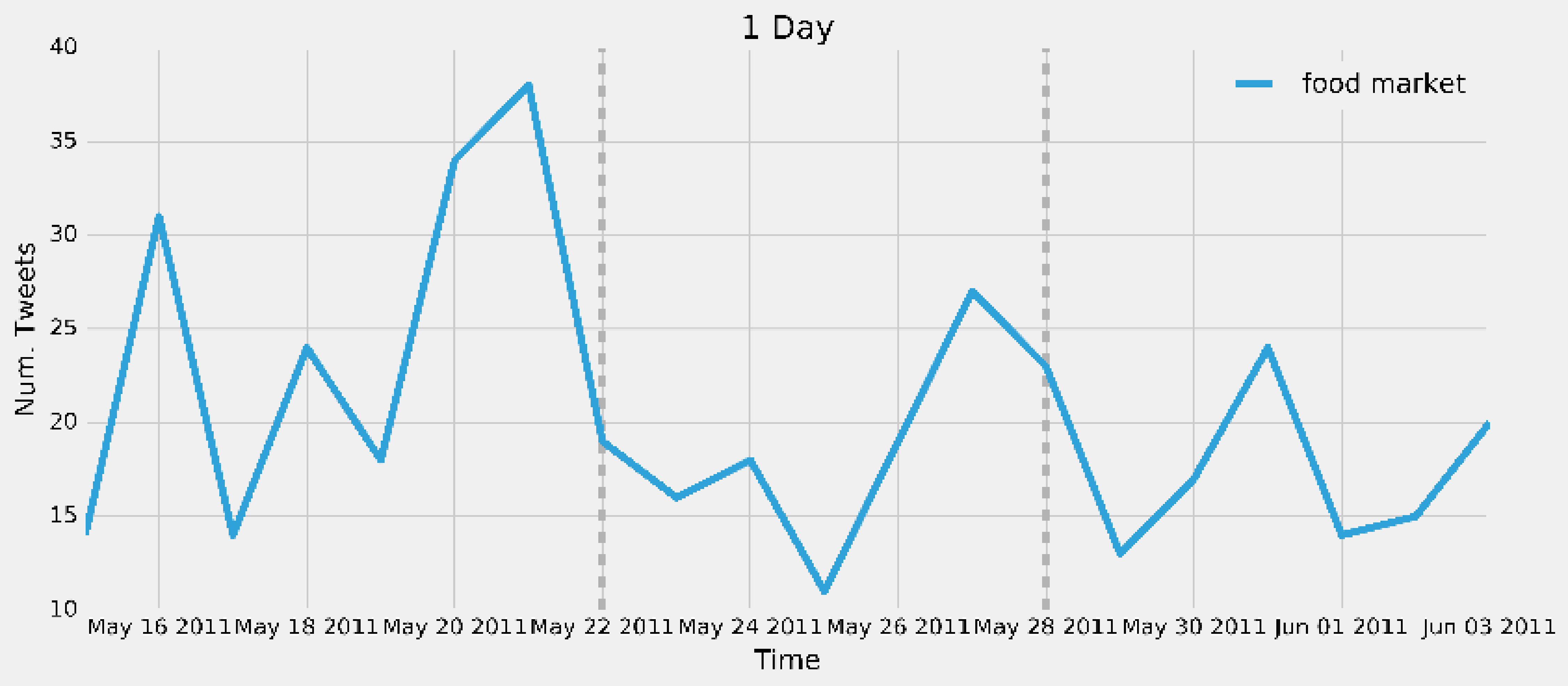
1 Day
food market
Num. Tweets
10
15
20
25
30
35
40
May 16 2011
May 18 2011
May 20 2011
May 22 2011
May 24 2011
May 26 2011
May 28 2011
May 30 2011
Jun 01 2011
Jun 03 2011
Time

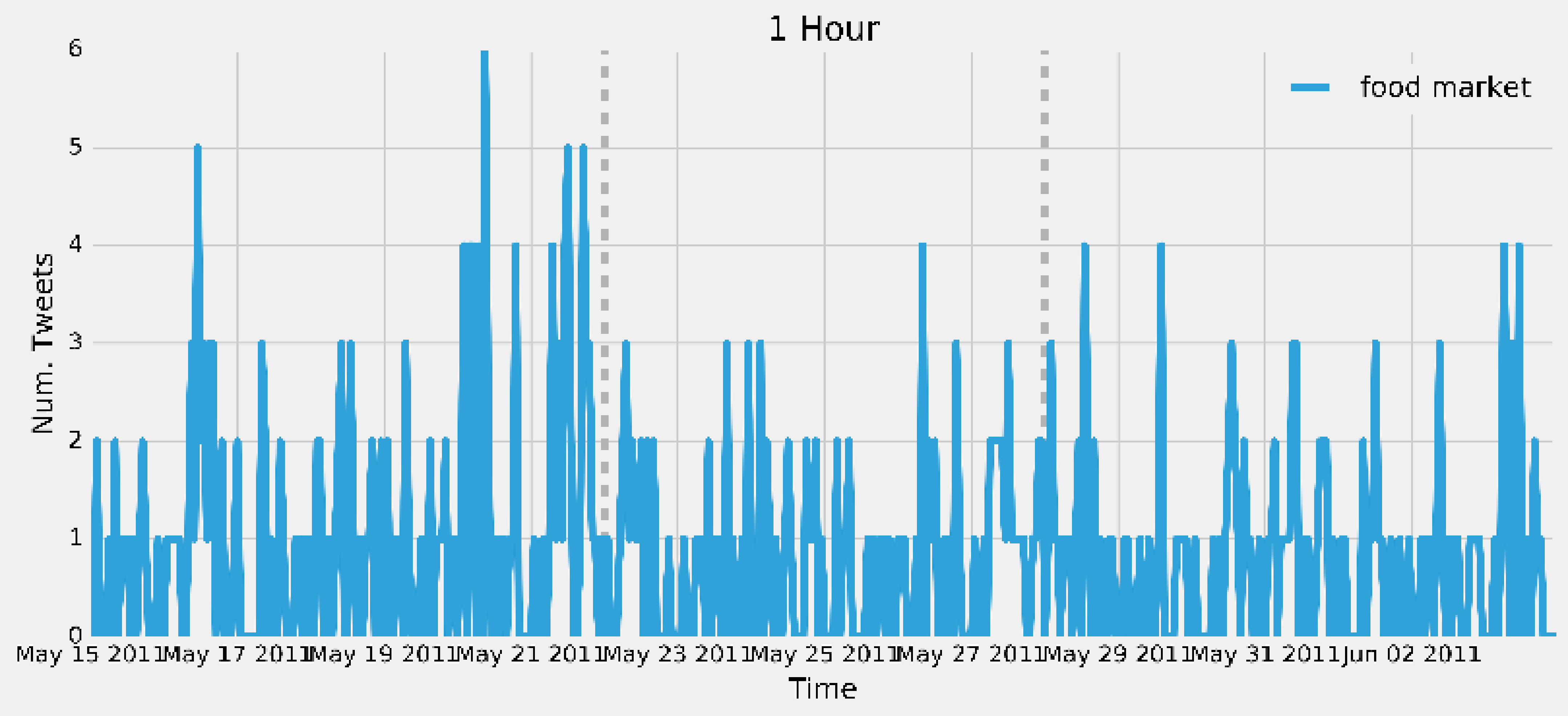
1 Hour
food market
Num. Tweets
0
1
2
3
4
5
6
May 15 2011
May 17 2011
May 19 2011
May 21 2011
May 23 2011
May 25 2011
May 27 2011
May 29 2011
May 31 2011
Jun 02 2011
Time

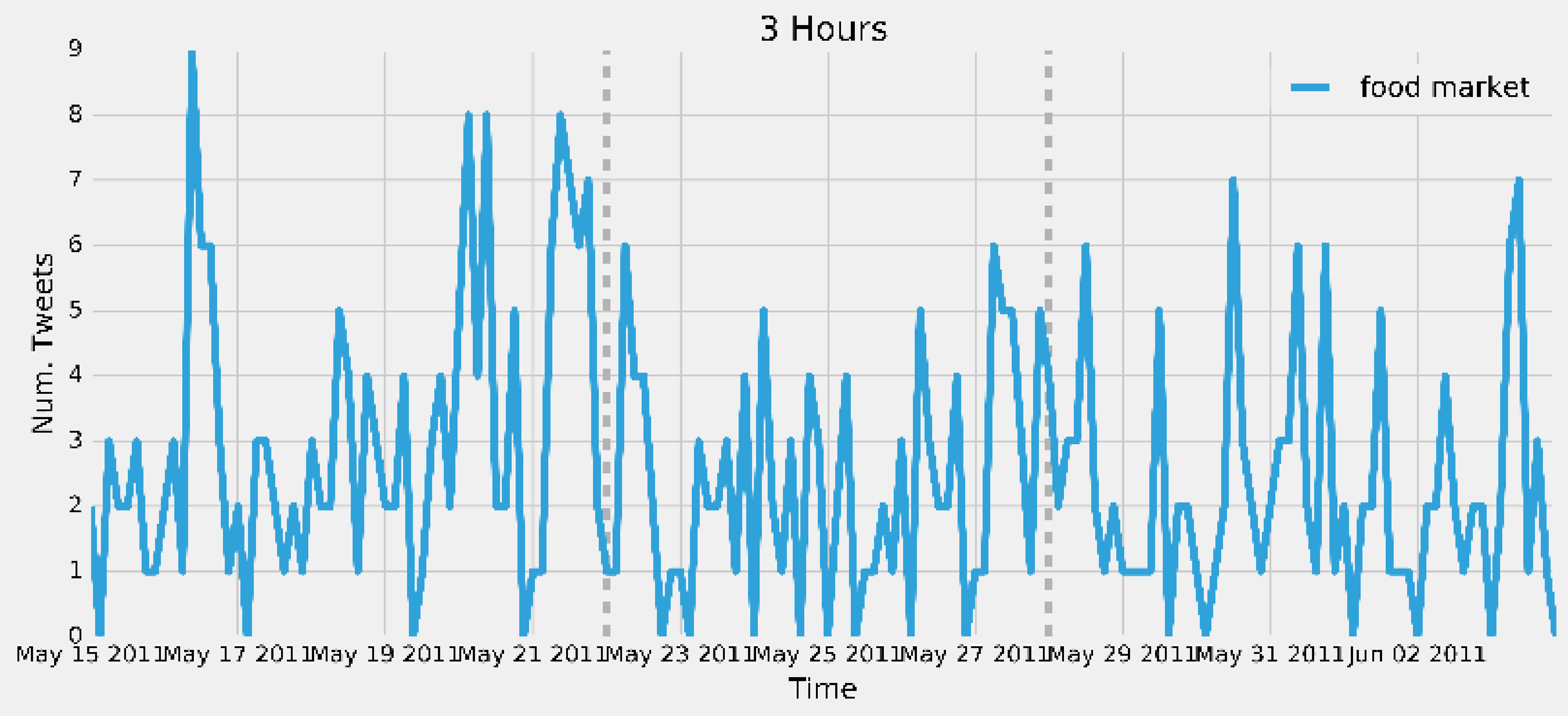

3 Hours
food market
Num. Tweets
0
1
2
3
4
5
6
7
8
9
May 15 2011
May 17 2011
May 19 2011
May 21 2011
May 23 2011
May 25 2011
May 27 2011
May 29 2011
May 31 2011
Jun 02 2011
Time

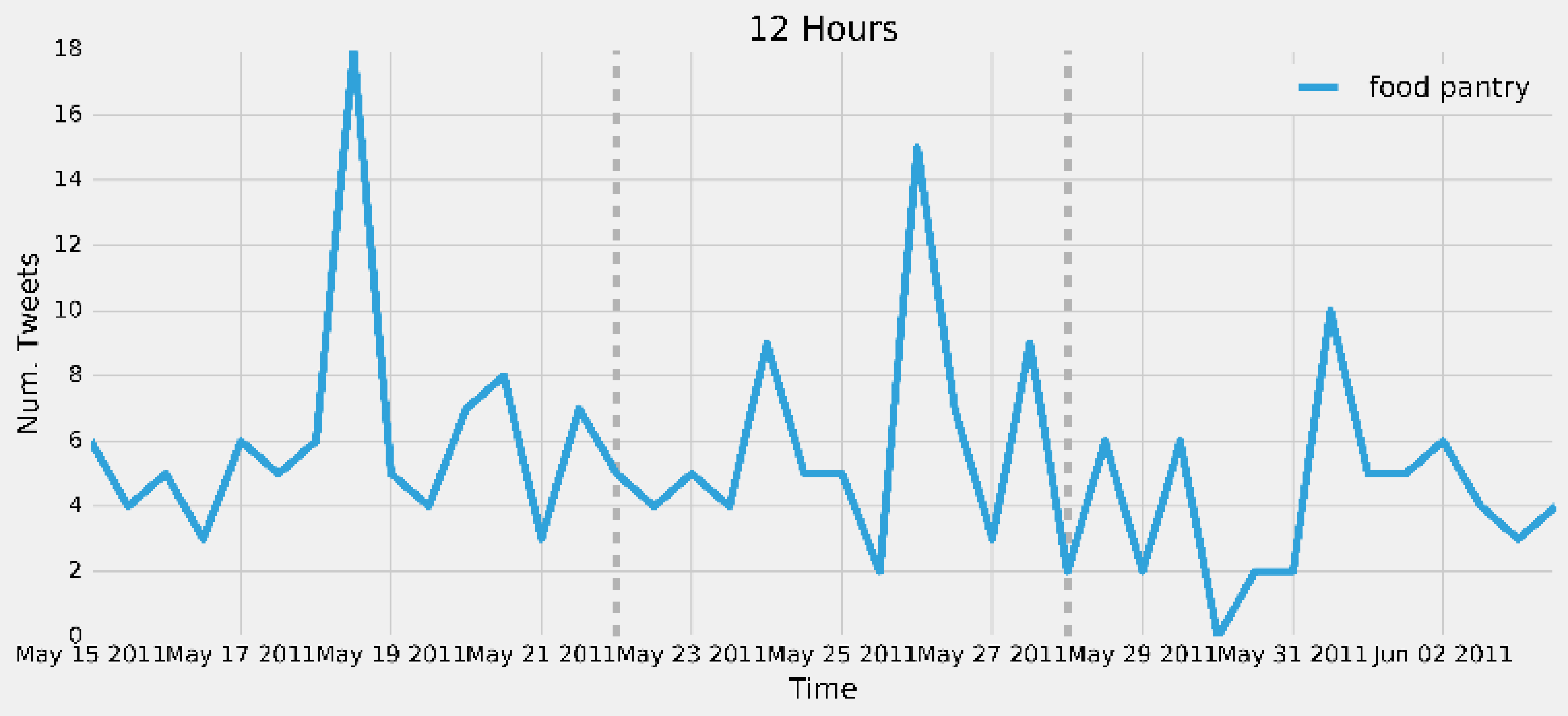

12 Hours
food pantry
Num. Tweets
Time
0
2
4
6
8
10
12
14
16
18
May 15 2011
May 17 2011
May 19 2011
May 21 2011
May 23 2011
May 25 2011
May 27 2011
May 29 2011
May 31 2011
Jun 02 2011

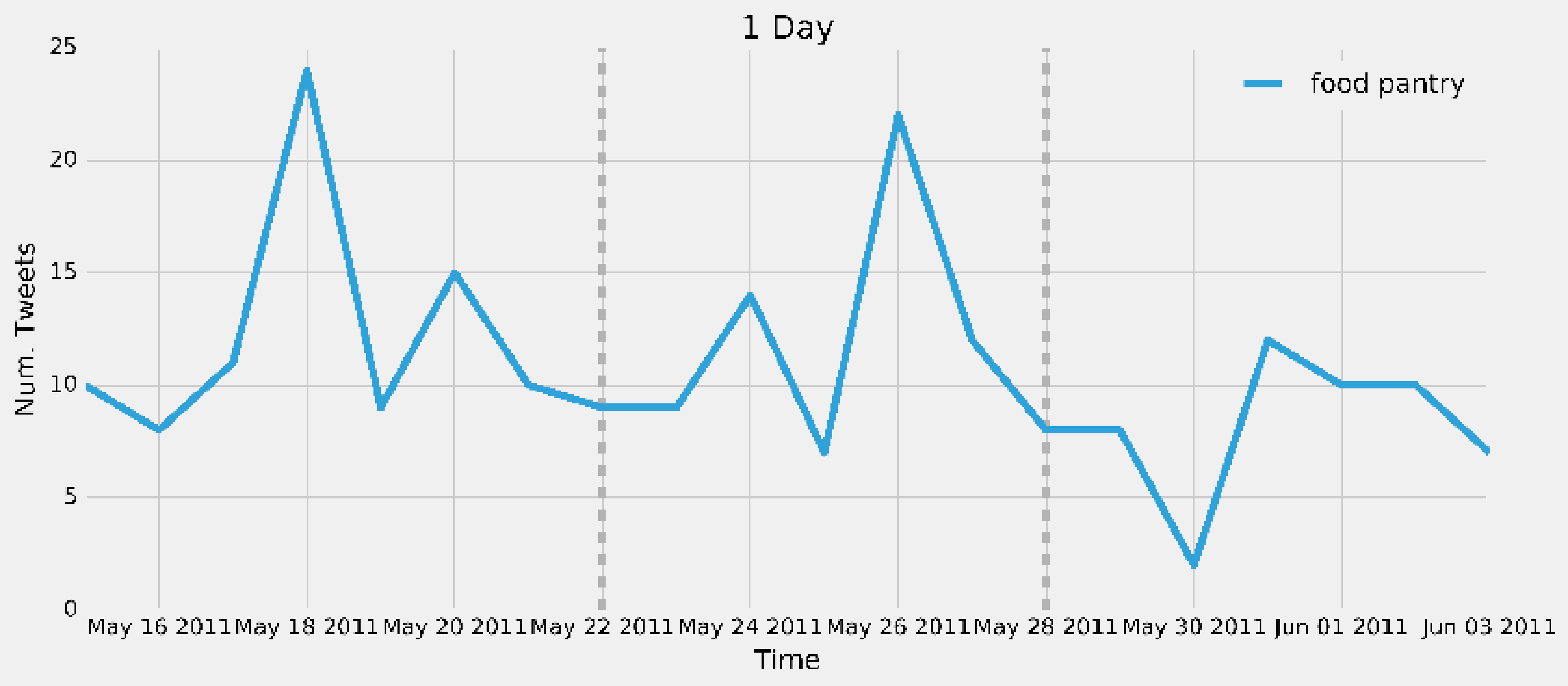
1 Day
food pantry
Num. Tweets
0
5
10
15
20
25
May 16 2011
May 18 2011
May 20 2011
May 22 2011
May 24 2011
May 26 2011
May 28 2011
May 30 2011
Jun 01 2011
Jun 03 2011
Time

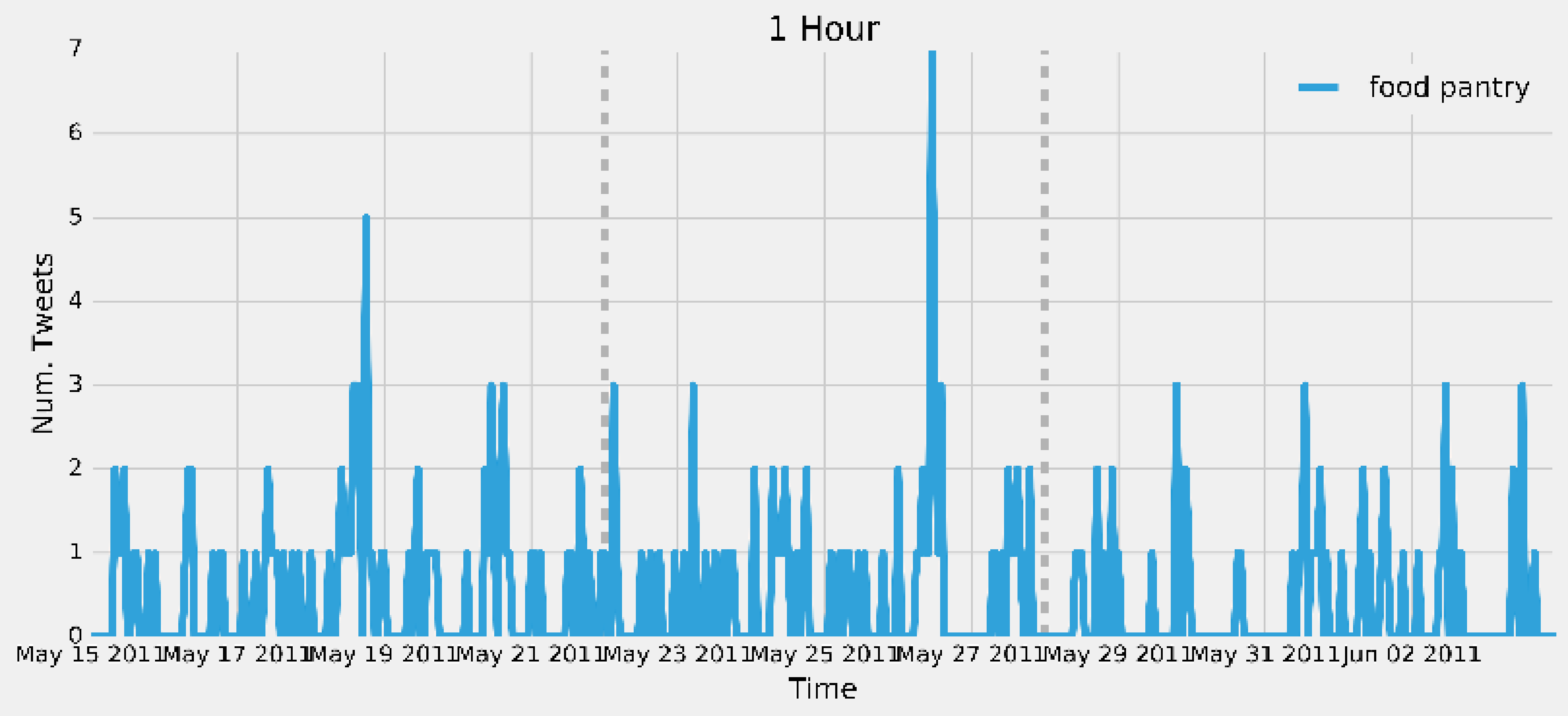
1 Hour
food pantry
Num. Tweets
0
1
2
3
4
5
6
7
May 15 2011
May 17 2011
May 19 2011
May 21 2011
May 23 2011
May 25 2011
May 27 2011
May 29 2011
May 31 2011
Jun 02 2011
Time

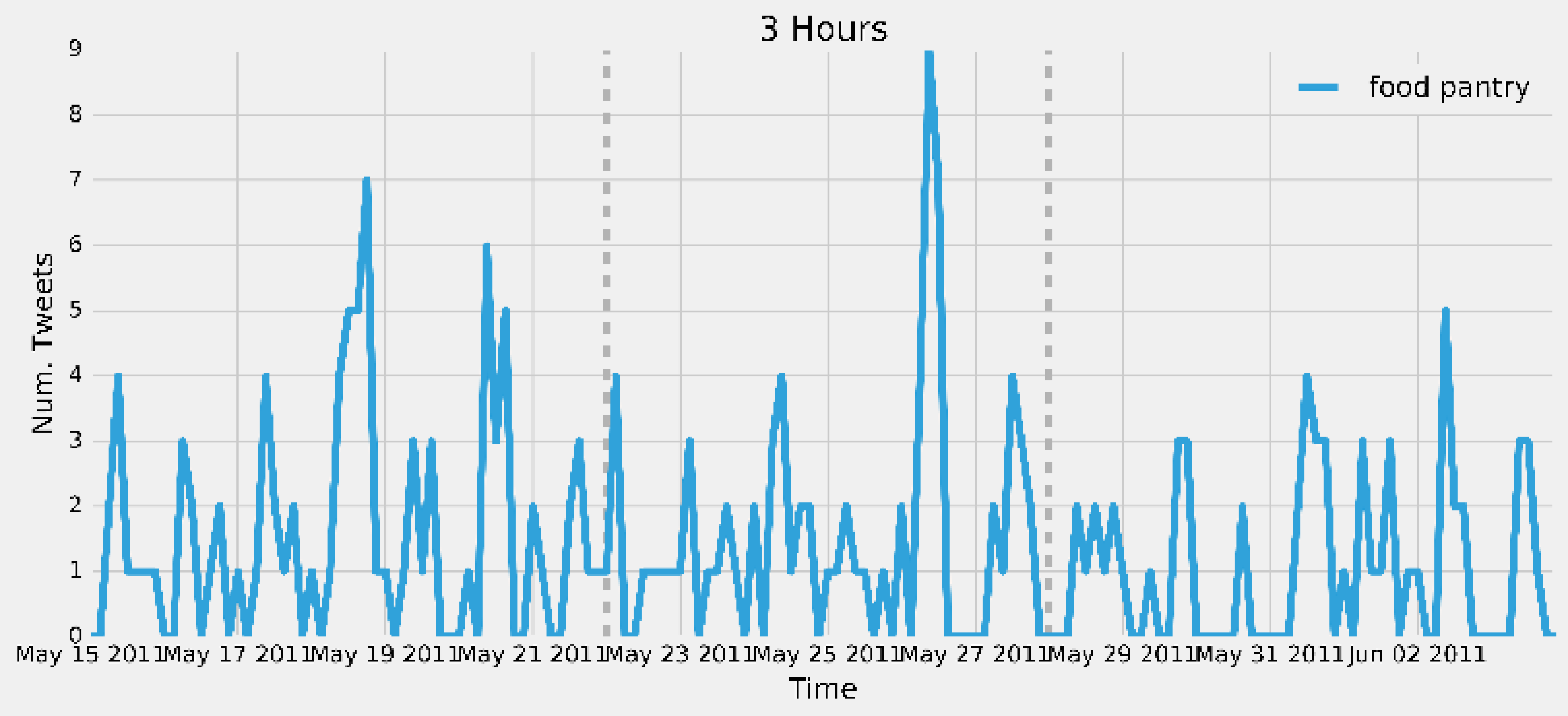

3 Hours
food pantry
Num. Tweets
0
1
2
3
4
5
6
7
8
9
May 15 2011
May 17 2011
May 19 2011
May 21 2011
May 23 2011
May 25 2011
May 27 2011
May 29 2011
May 31 2011
Jun 02 2011
Time

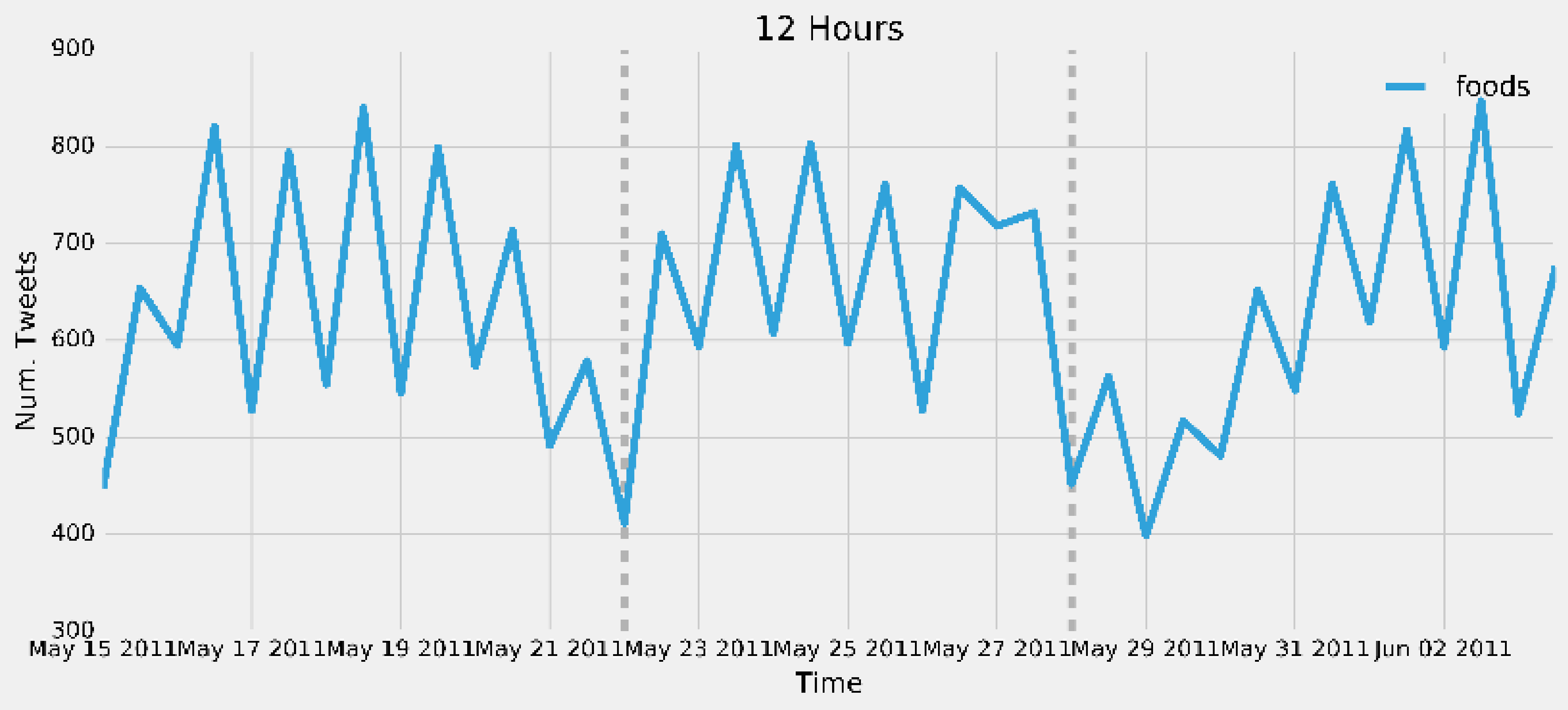
12 Hours
foods
Num. Tweets
900
800
700
600
500
400
300
May 15 2011
May 17 2011
May 19 2011
May 21 2011
May 23 2011
May 25 2011
May 27 2011
May 29 2011
May 31 2011
Jun 02 2011
Time

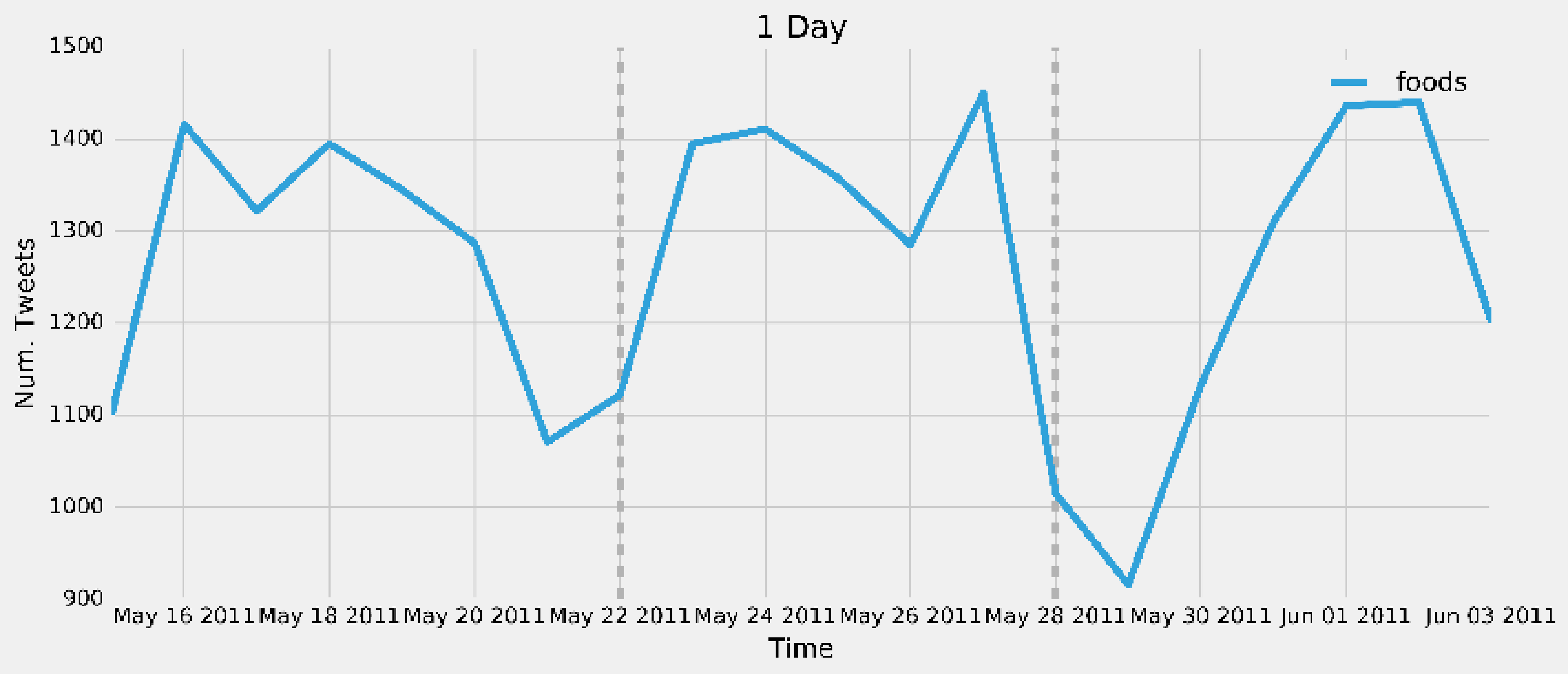
1 Day
foods
Num. Tweets
Time
1500
1400
1300
1200
1100
1000
900
May 16 2011
May 18 2011
May 20 2011
May 22 2011
May 24 2011
May 26 2011
May 28 2011
May 30 2011
Jun 01 2011
Jun 03 2011

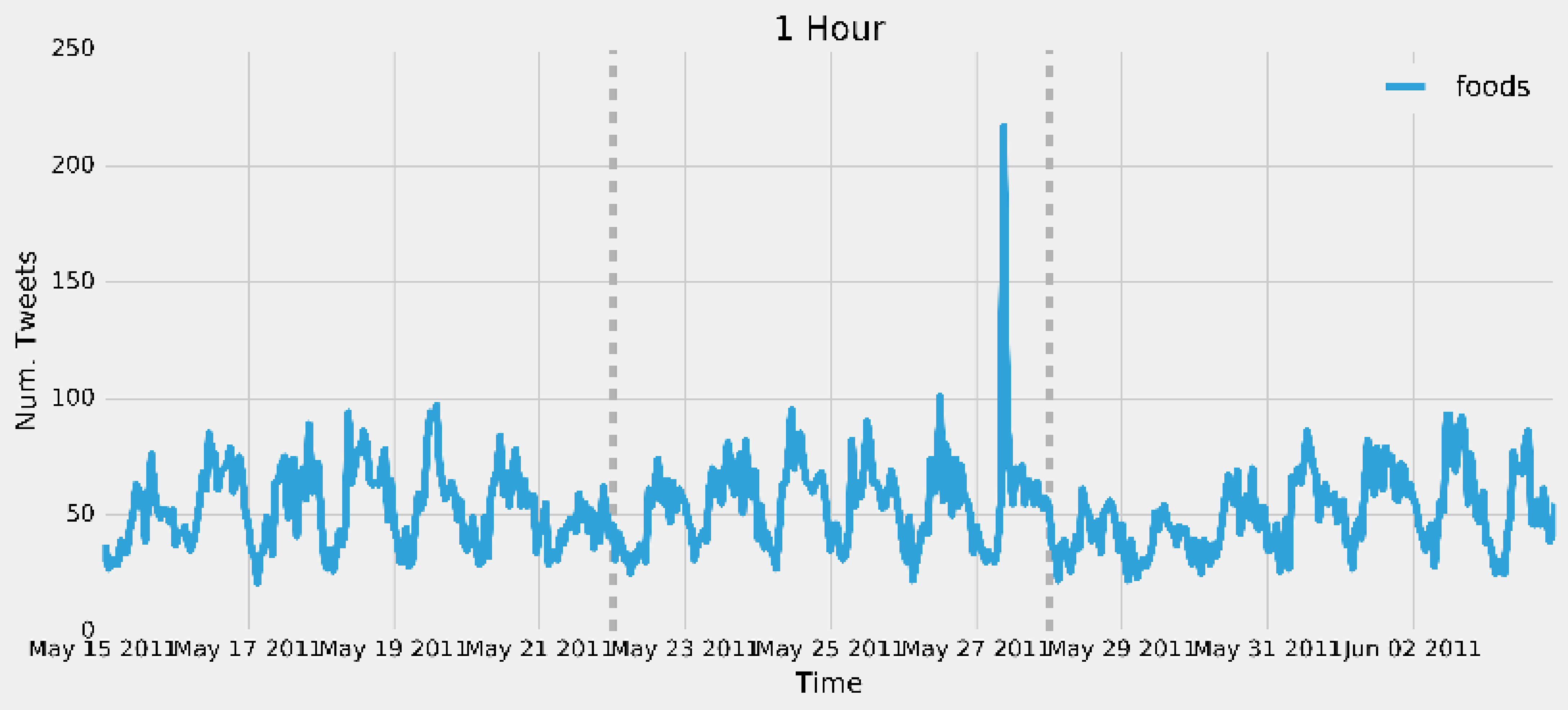
1 Hour
foods
Num. Tweets
0
50
100
150
200
250
May 15 2011
May 17 2011
May 19 2011
May 21 2011
May 23 2011
May 25 2011
May 27 2011
May 29 2011
May 31 2011
Jun 02 2011
Time

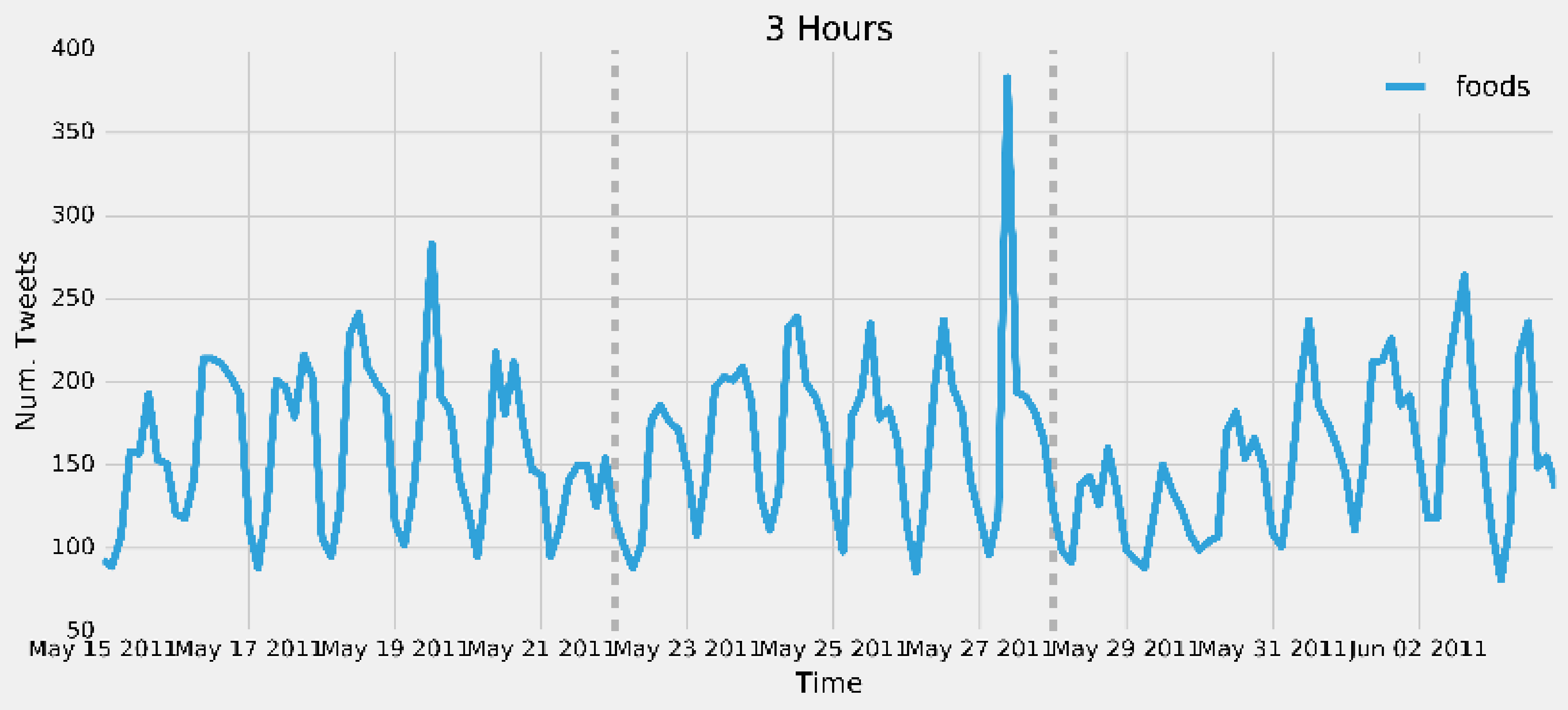

3 Hours
foods
Num. Tweets
400
350
300
250
200
150
100
50
May 15 2011
May 17 2011
May 19 2011
May 21 2011
May 23 2011
May 25 2011
May 27 2011
May 29 2011
May 31 2011
Jun 02 2011
Time

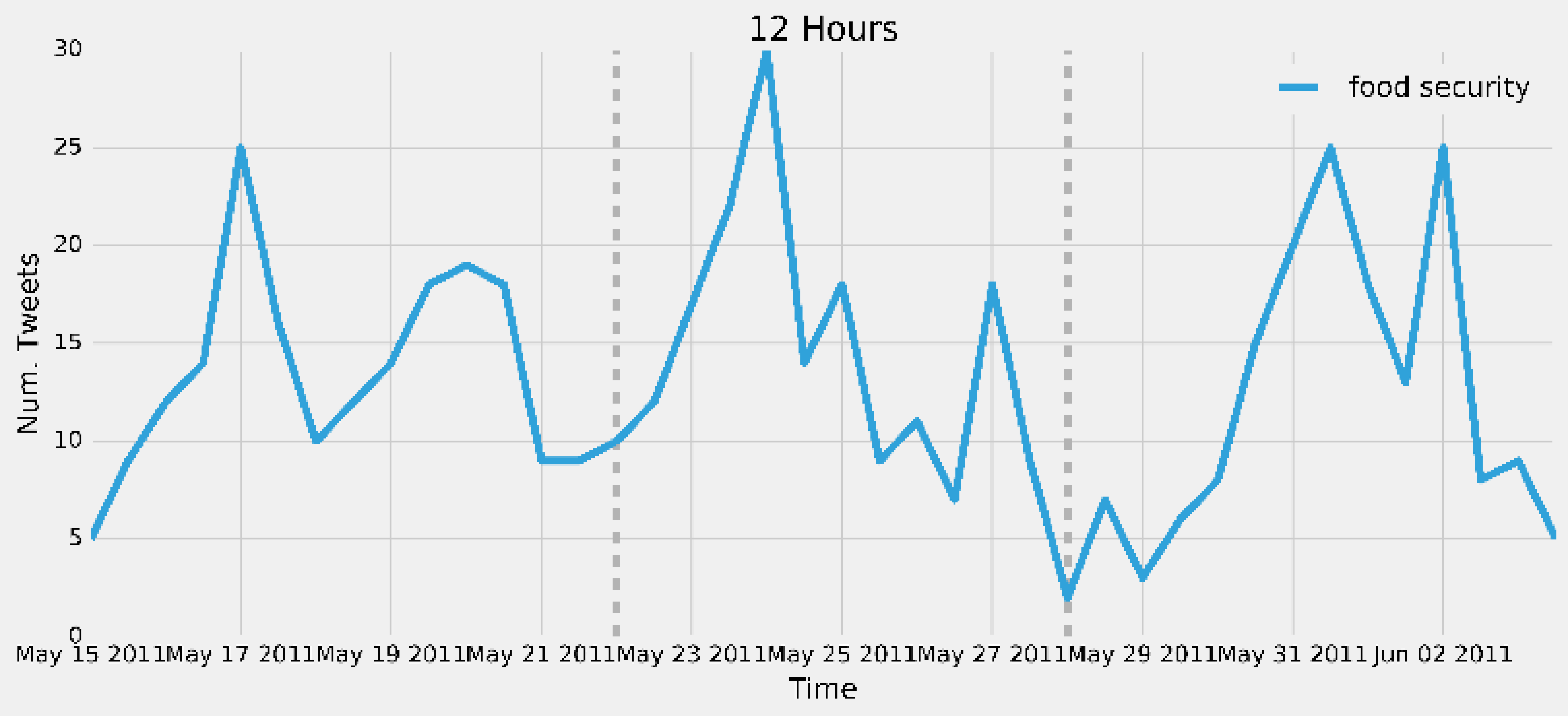

12 Hours
food security
Num. Tweets
Time
0
5
10
15
20
25
30
May 15 2011
May 17 2011
May 19 2011
May 21 2011
May 23 2011
May 25 2011
May 27 2011
May 29 2011
May 31 2011
Jun 02 2011

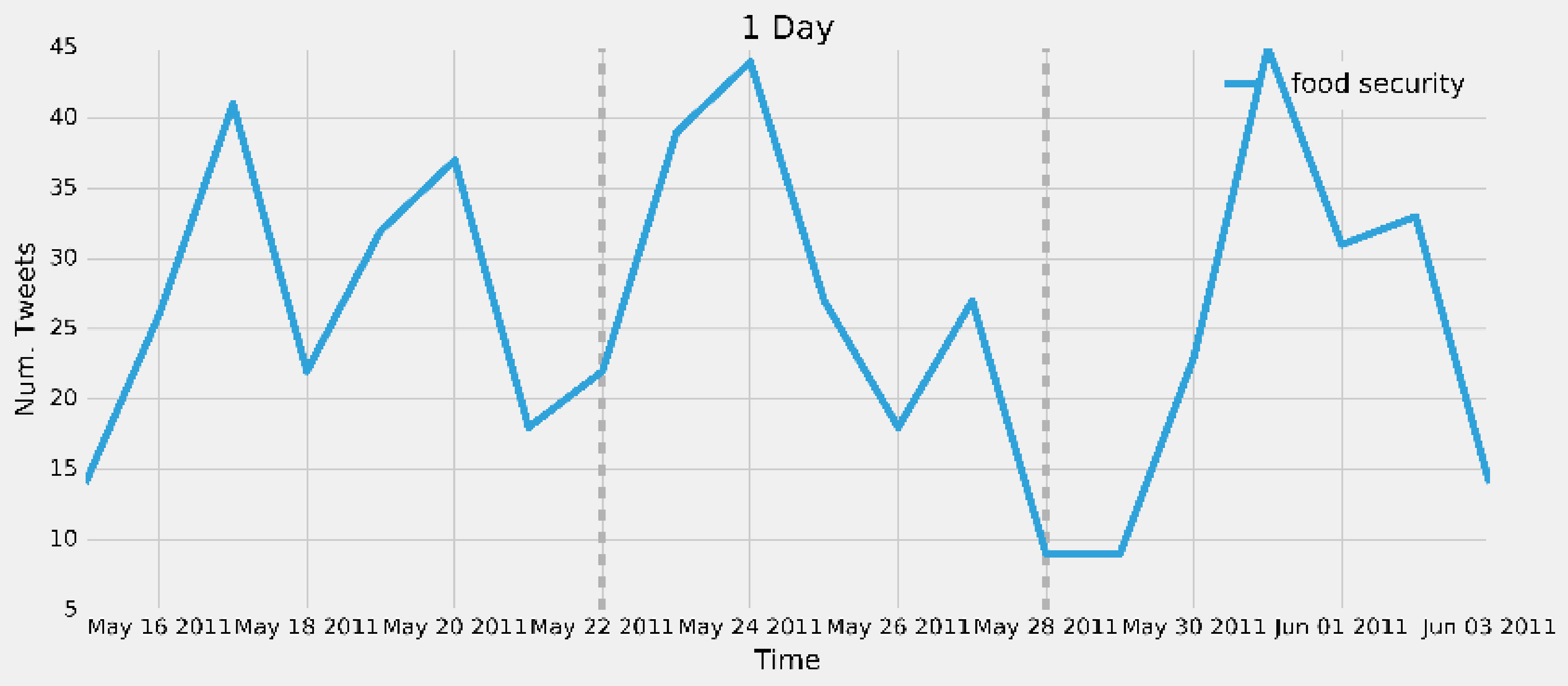
1 Day
food security
Num. Tweets
Time
45
40
35
30
25
20
15
10
5
May 16 2011
May 18 2011
May 20 2011
May 22 2011
May 24 2011
May 26 2011
May 28 2011
May 30 2011
Jun 01 2011
Jun 03 2011

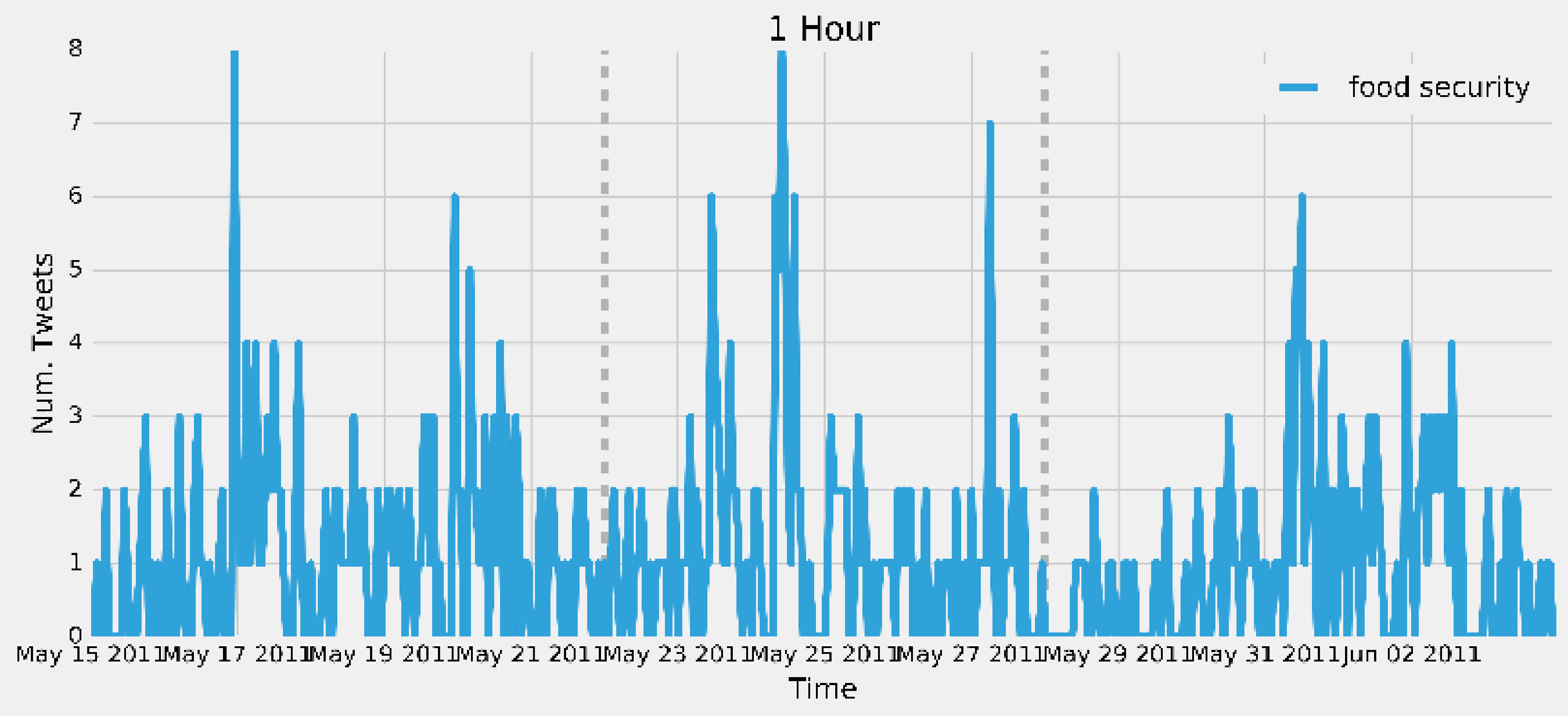
1 Hour
food security
Num. Tweets
0
1
2
3
4
5
6
7
8
May 15 2011
May 17 2011
May 19 2011
May 21 2011
May 23 2011
May 25 2011
May 27 2011
May 29 2011
May 31 2011
Jun 02 2011
Time

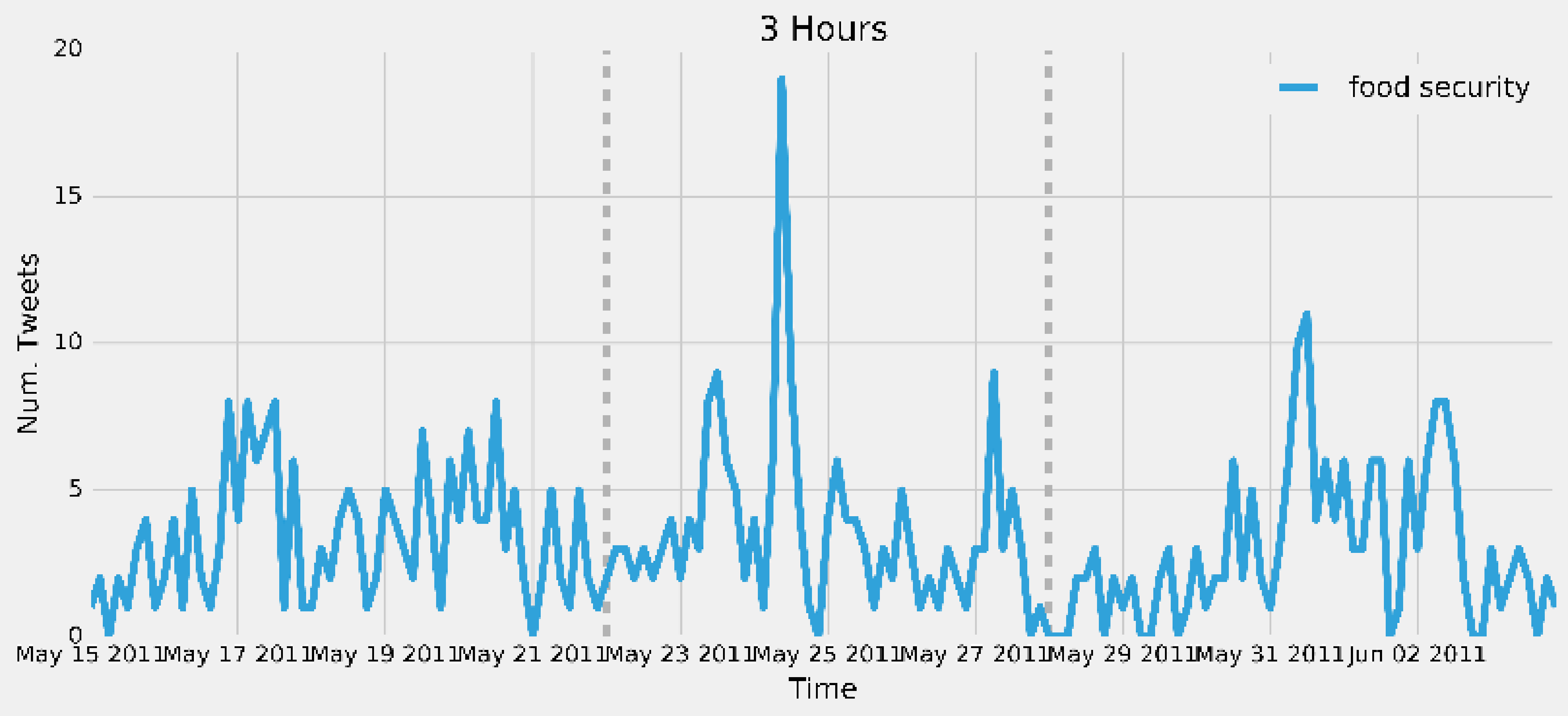

3 Hours
food security
Num. Tweets
Time
0
5
10
15
20
May 15 2011
May 17 2011
May 19 2011
May 21 2011
May 23 2011
May 25 2011
May 27 2011
May 29 2011
May 31 2011
Jun 02 2011

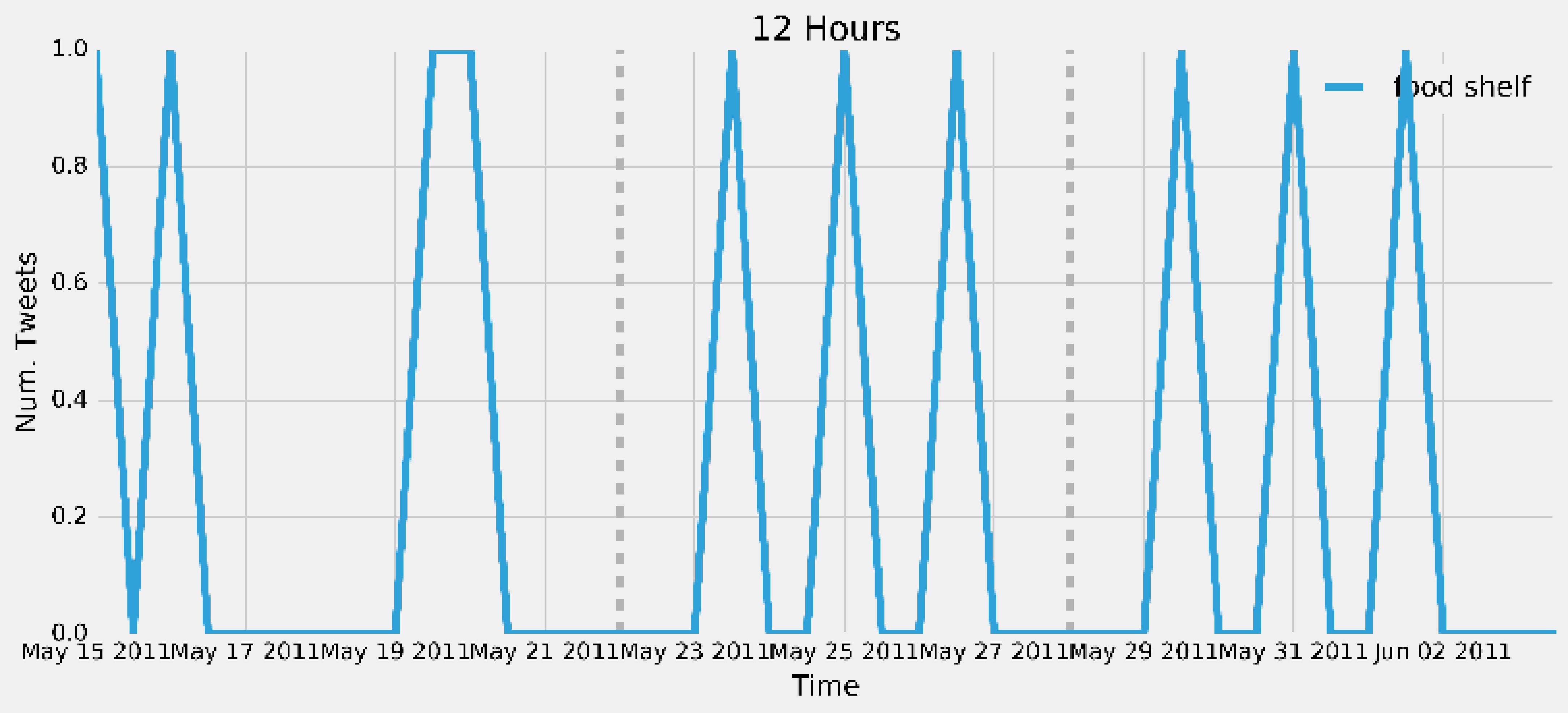

12 Hours
food shelf
Num. Tweets
1.0
0.8
0.6
0.4
0.2
0.0
May 15 2011
May 17 2011
May 19 2011
May 21 2011
May 23 2011
May 25 2011
May 27 2011
May 29 2011
May 31 2011
Jun 02 2011
Time

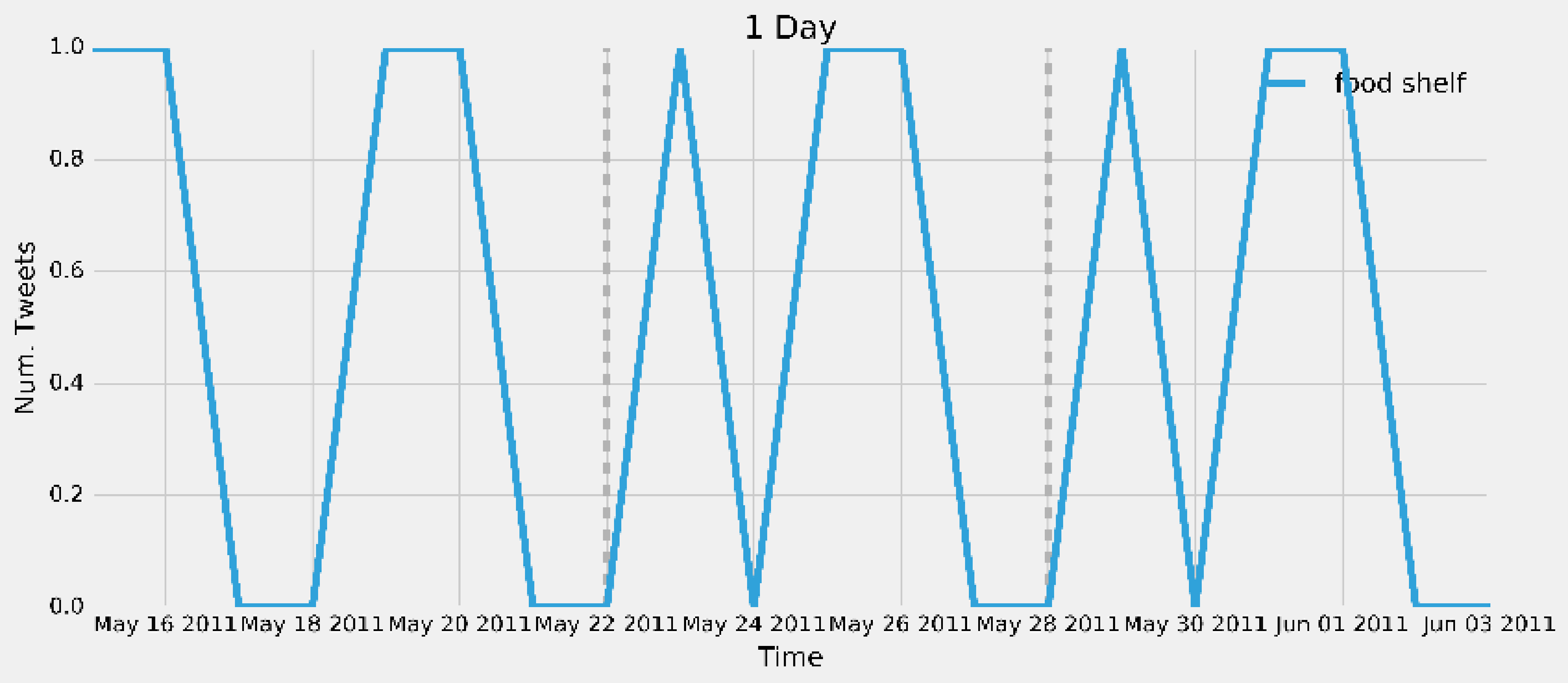

1 Day
Num. Tweets
Time
food shelf
1.0
0.8
0.6
0.4
0.2
0.0
May 16 2011
May 18 2011
May 20 2011
May 22 2011
May 24 2011
May 26 2011
May 28 2011
May 30 2011
Jun 01 2011
Jun 03 2011

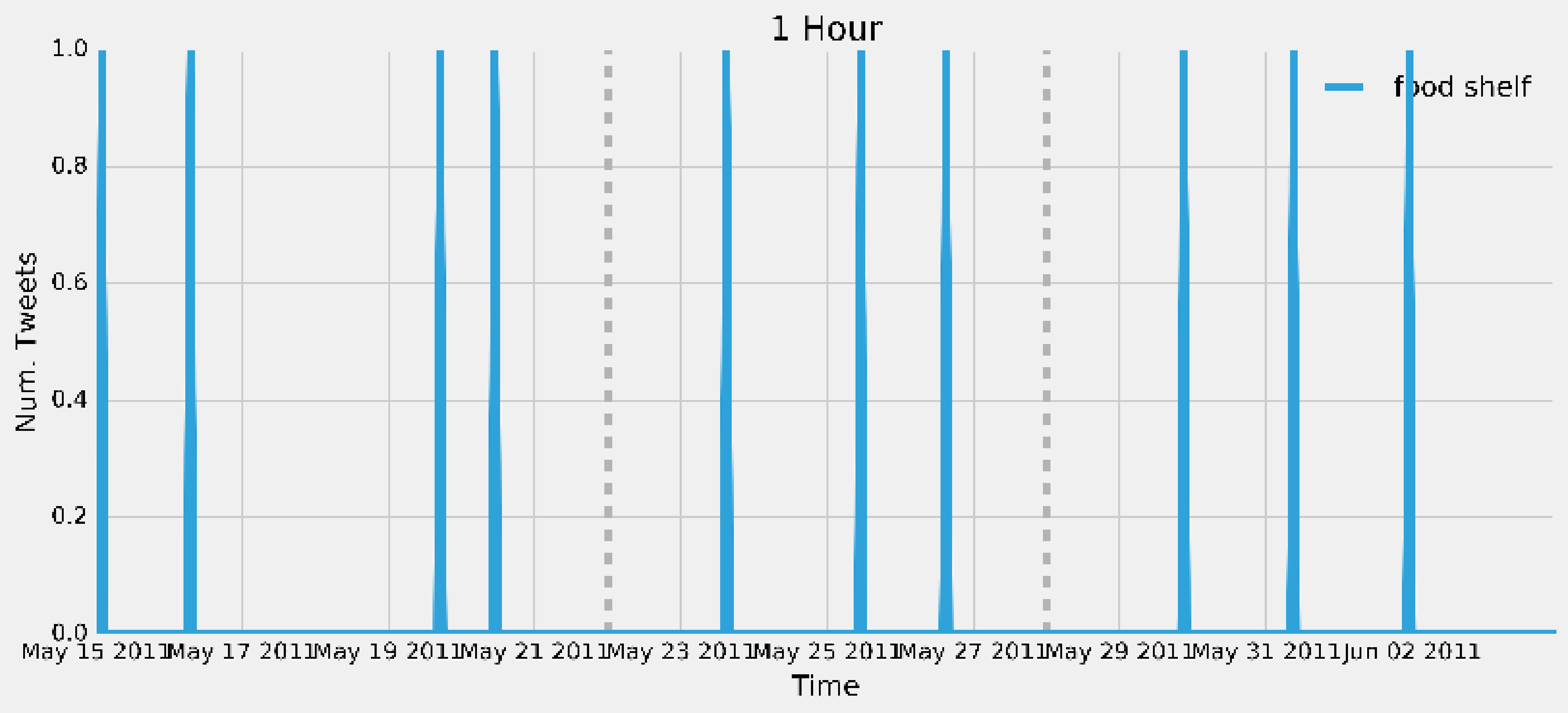
1 Hour
food shelf
Num. Tweets
1.0
0.8
0.6
0.4
0.2
0.0
May 15 2011
May 17 2011
May 19 2011
May 21 2011
May 23 2011
May 25 2011
May 27 2011
May 29 2011
May 31 2011
Jun 02 2011
Time

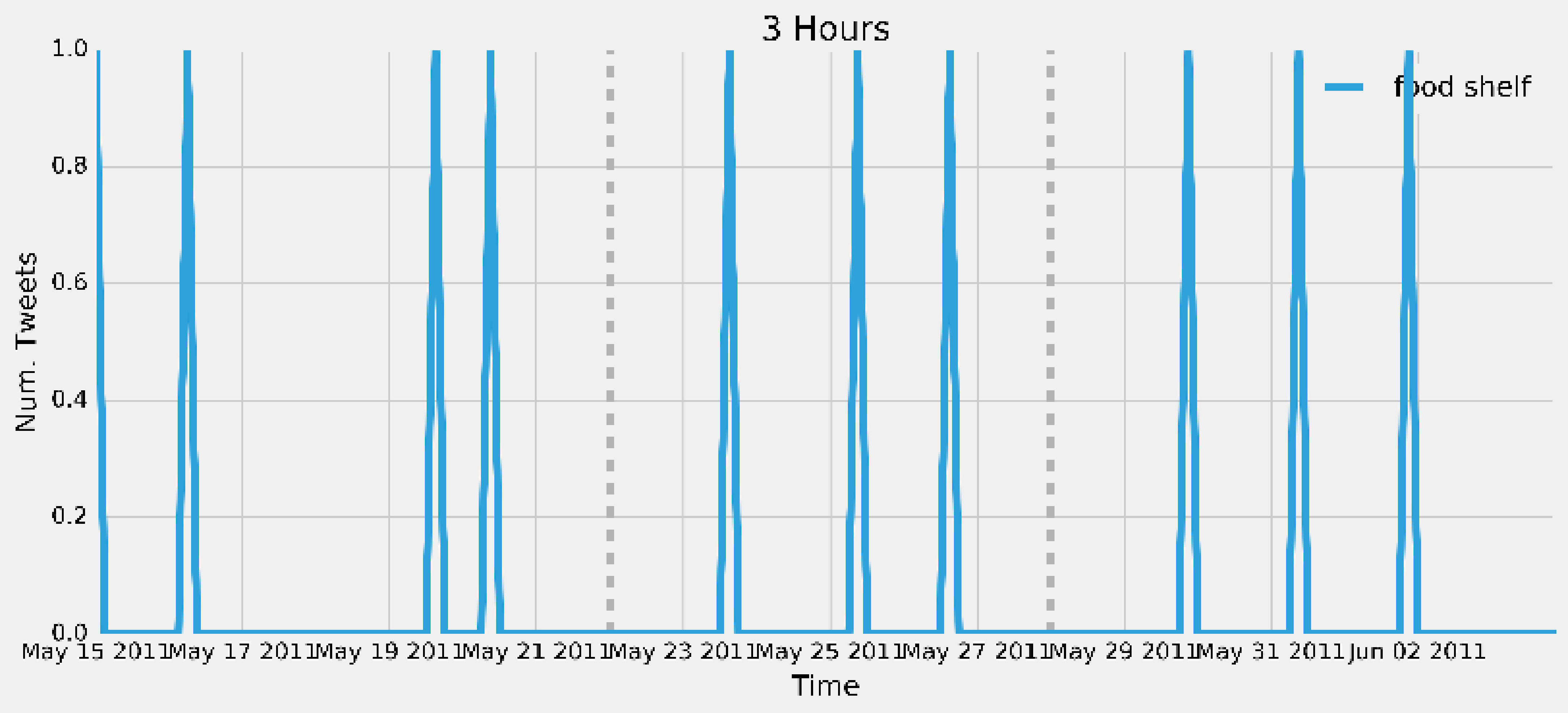
3 Hours
food shelf
Num. Tweets
1.0
0.8
0.6
0.4
0.2
0.0
May 15 2011
May 17 2011
May 19 2011
May 21 2011
May 23 2011
May 25 2011
May 27 2011
May 29 2011
May 31 2011
Jun 02 2011
Time

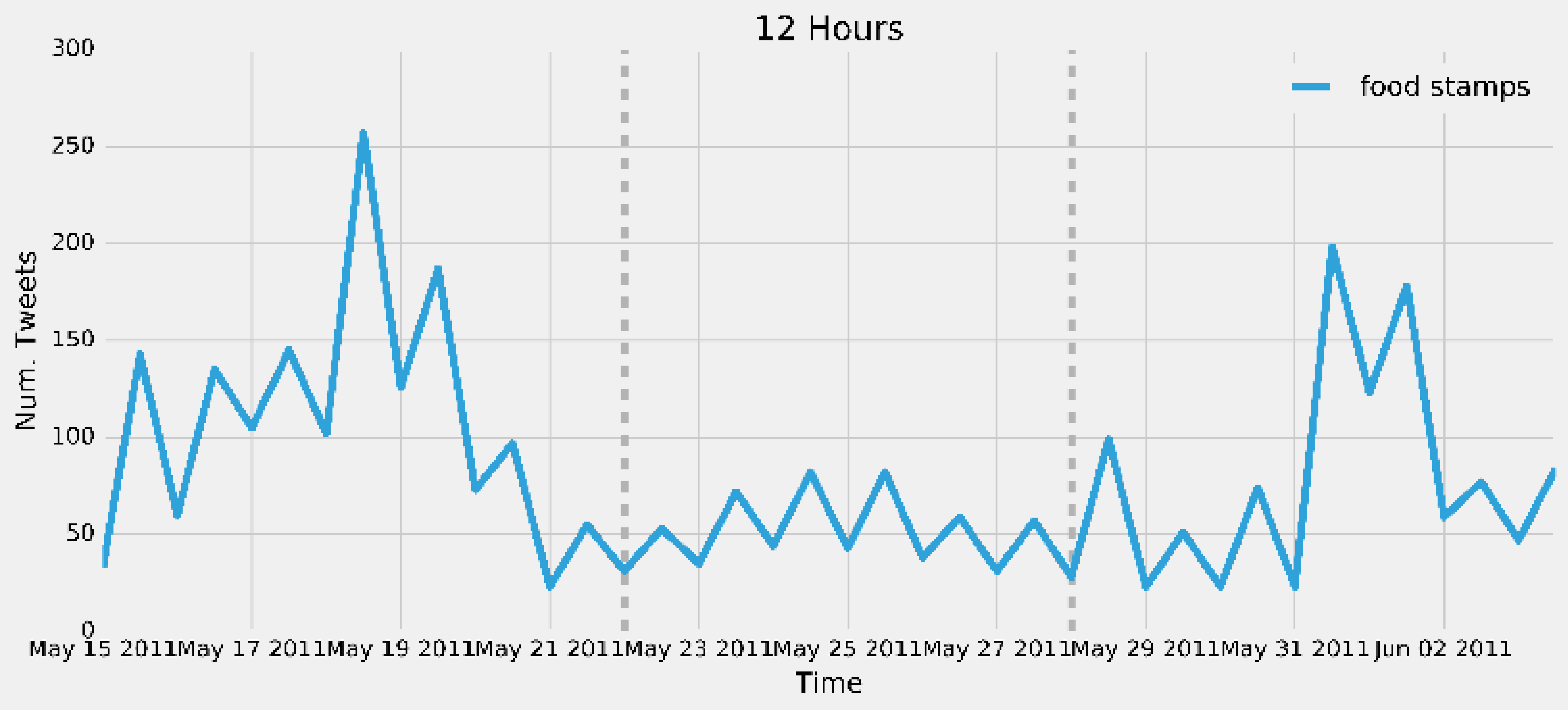
12 Hours
food stamps
Num. Tweets
0
50
100
150
200
250
300
May 15 2011
May 17 2011
May 19 2011
May 21 2011
May 23 2011
May 25 2011
May 27 2011
May 29 2011
May 31 2011
Jun 02 2011
Time

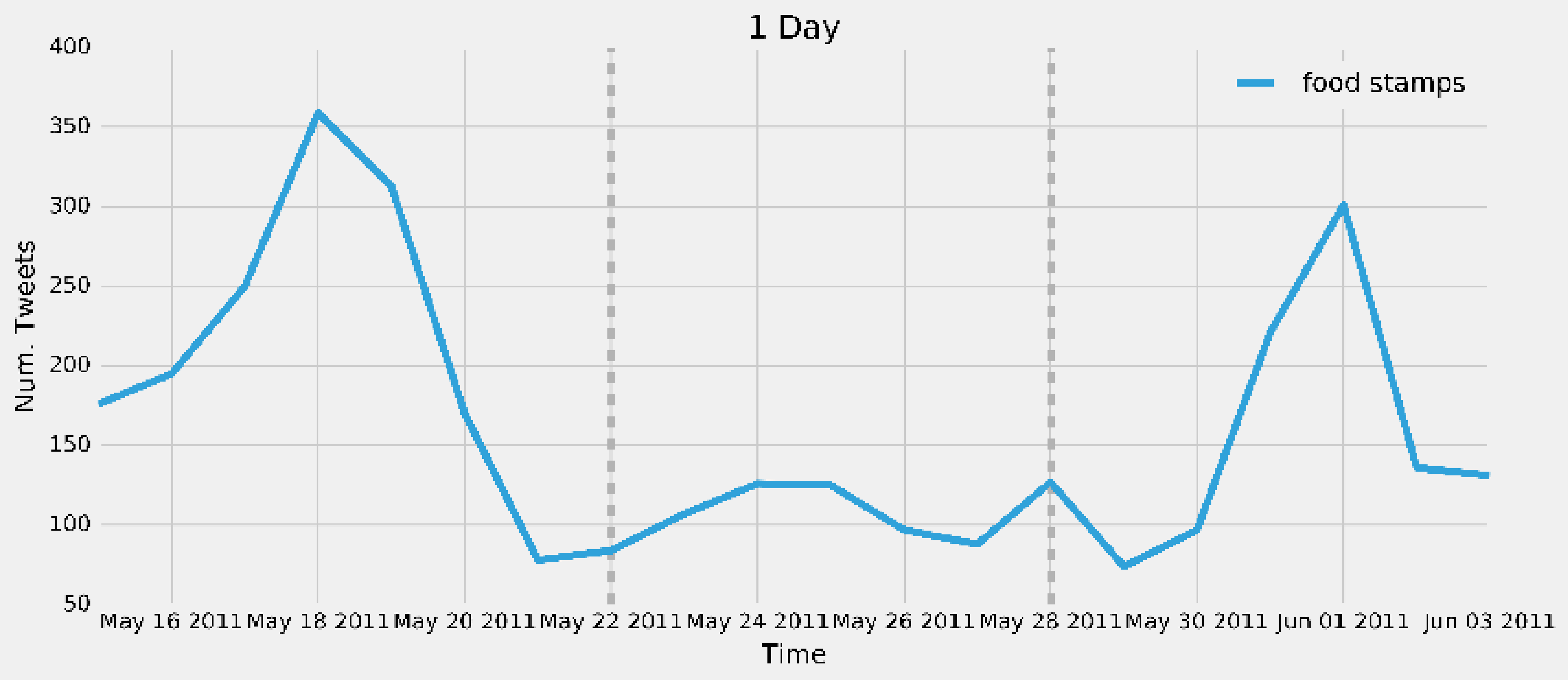

1 Day
food stamps
Num. Tweets
400
350
300
250
200
150
100
50
May 16 2011
May 18 2011
May 20 2011
May 22 2011
May 24 2011
May 26 2011
May 28 2011
May 30 2011
Jun 01 2011
Jun 03 2011
Time

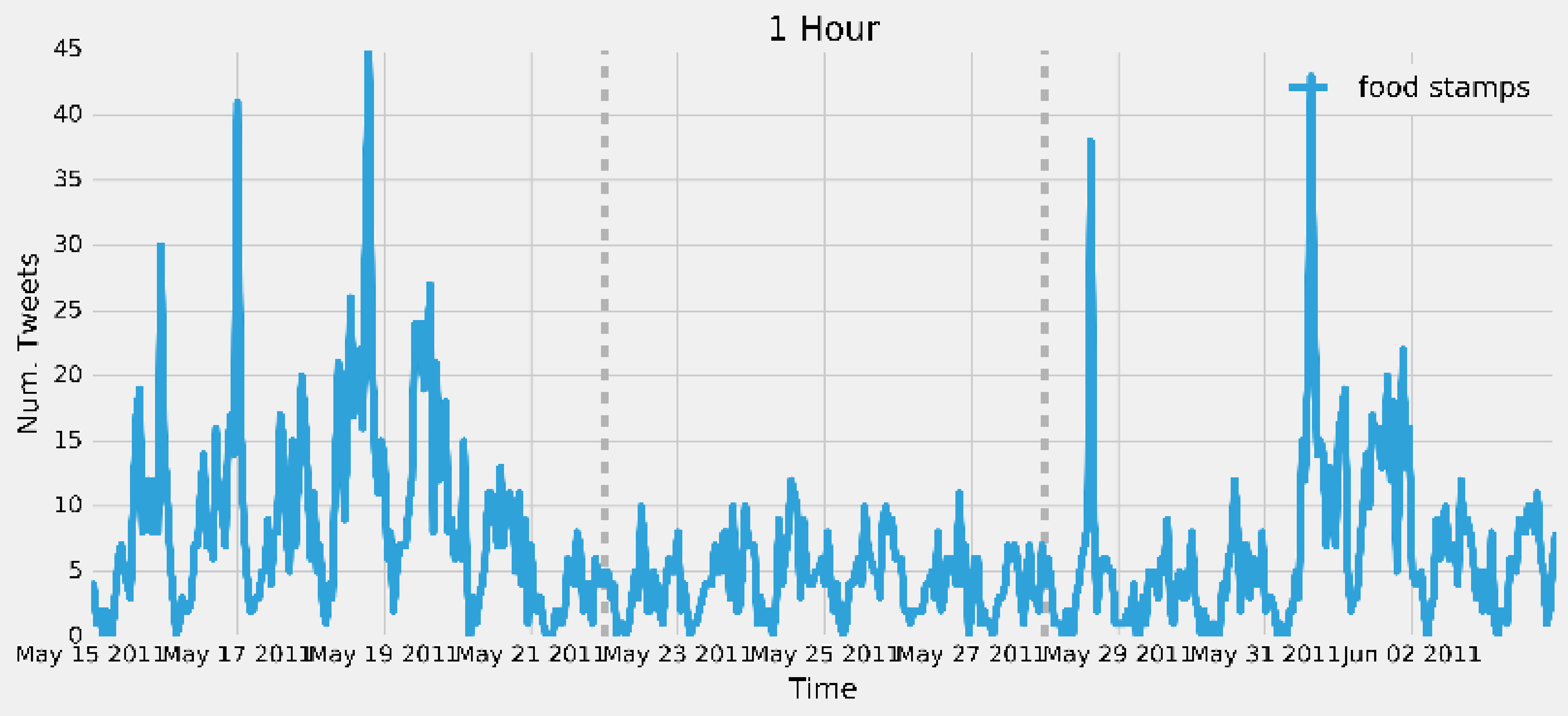
1 Hour
food stamps
Num. Tweets
0
5
10
15
20
25
30
35
40
45
May 15 2011
May 17 2011
May 19 2011
May 21 2011
May 23 2011
May 25 2011
May 27 2011
May 29 2011
May 31 2011
Jun 02 2011
Time

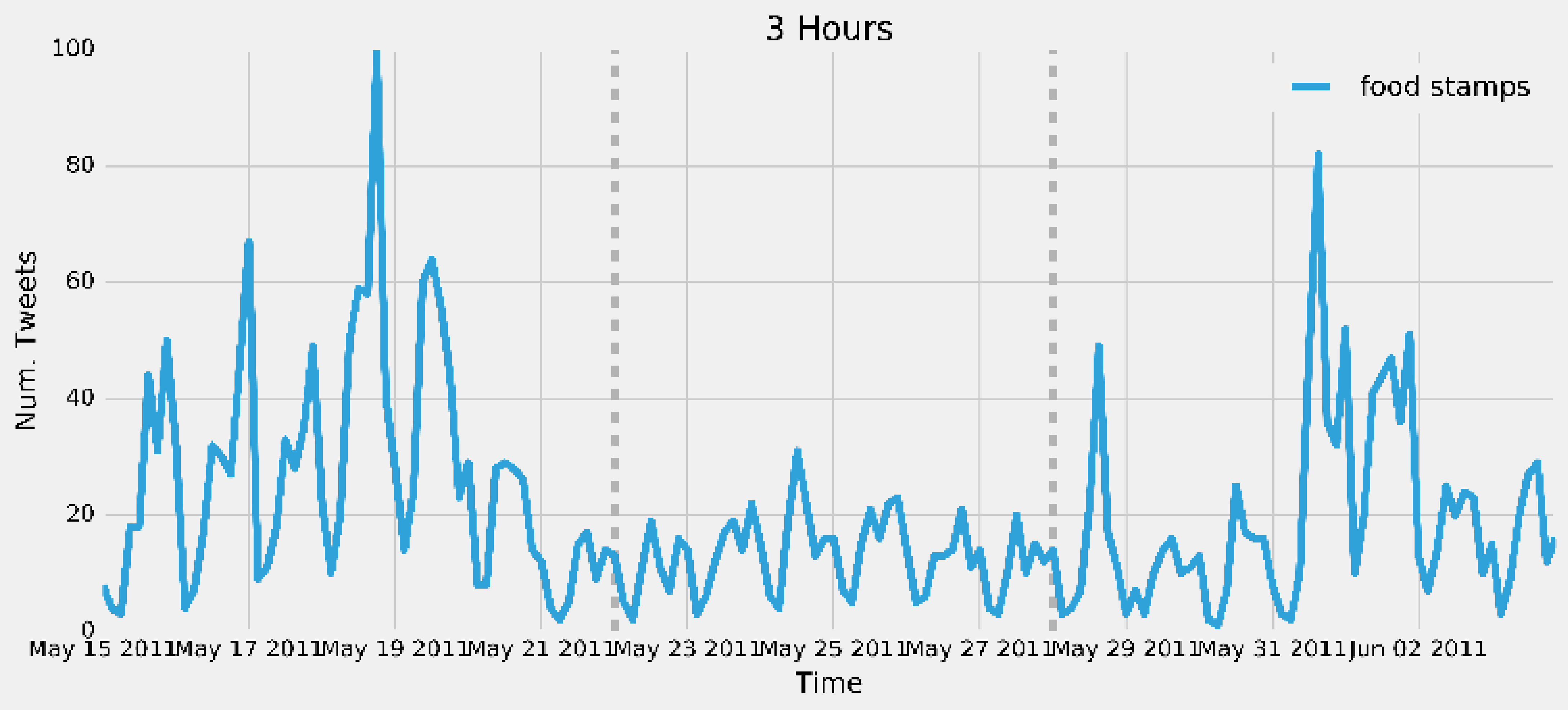
3 Hours
food stamps
Num. Tweets
0
20
40
60
80
100
May 15 2011
May 17 2011
May 19 2011
May 21 2011
May 23 2011
May 25 2011
May 27 2011
May 29 2011
May 31 2011
Jun 02 2011
Time

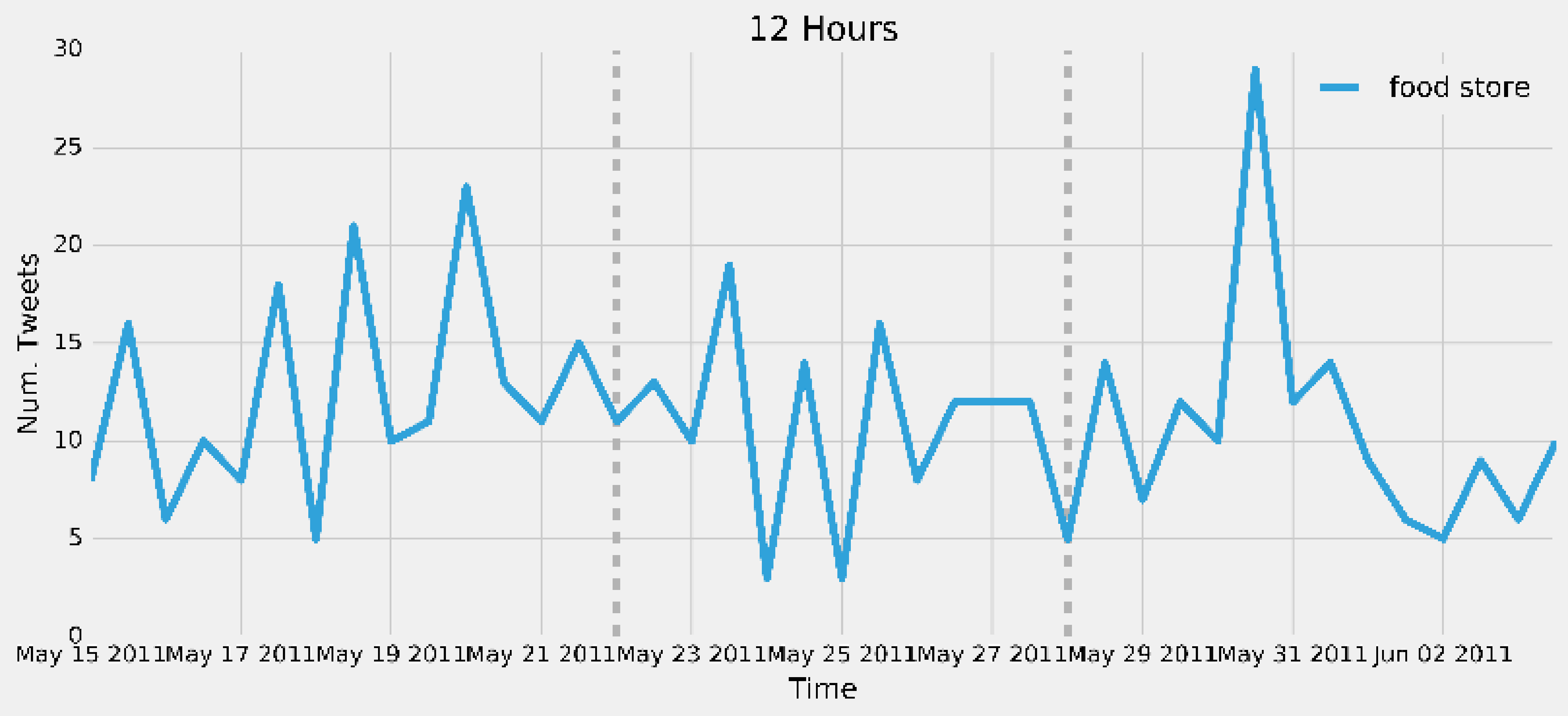

12 Hours
food store
Num. Tweets
30
25
20
15
10
5
0
May 15 2011
May 17 2011
May 19 2011
May 21 2011
May 23 2011
May 25 2011
May 27 2011
May 29 2011
May 31 2011
Jun 02 2011
Time

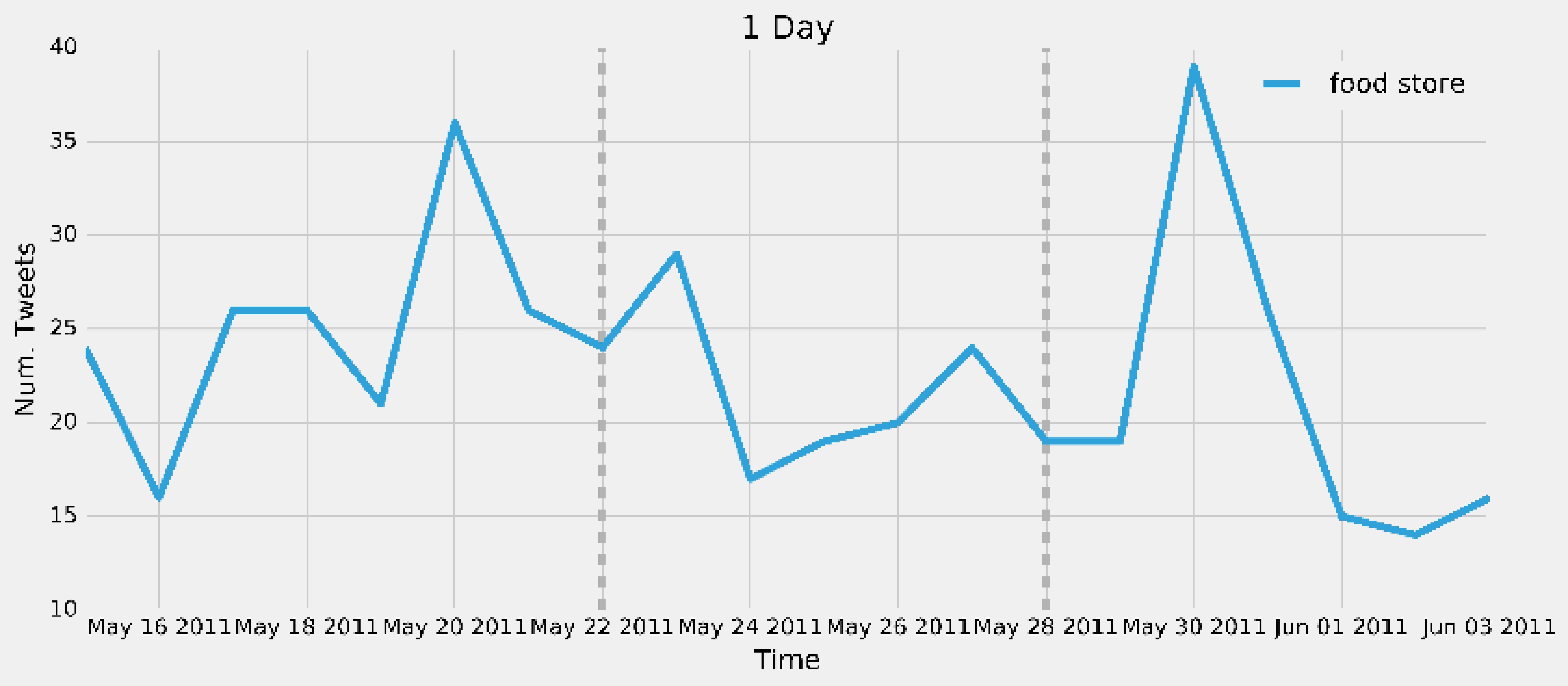
1 Day
food store
Num. Tweets
40
35
30
25
20
15
10
May 16 2011
May 18 2011
May 20 2011
May 22 2011
May 24 2011
May 26 2011
May 28 2011
May 30 2011
Jun 01 2011
Jun 03 2011
Time

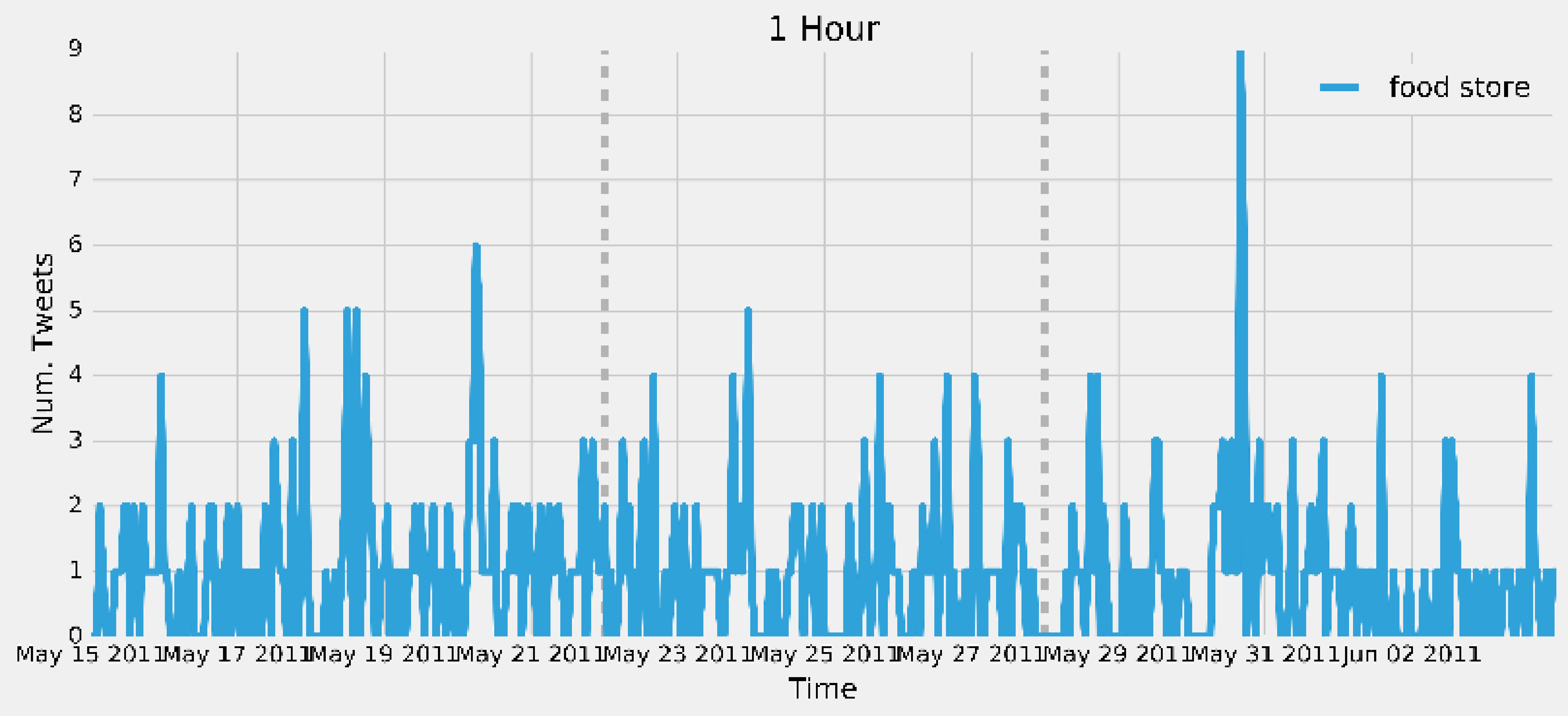
1 Hour
food store
Num. Tweets
9
8
7
6
5
4
3
2
1
0
May 15 2011
May 17 2011
May 19 2011
May 21 2011
May 23 2011
May 25 2011
May 27 2011
May 29 2011
May 31 2011
Jun 02 2011
Time

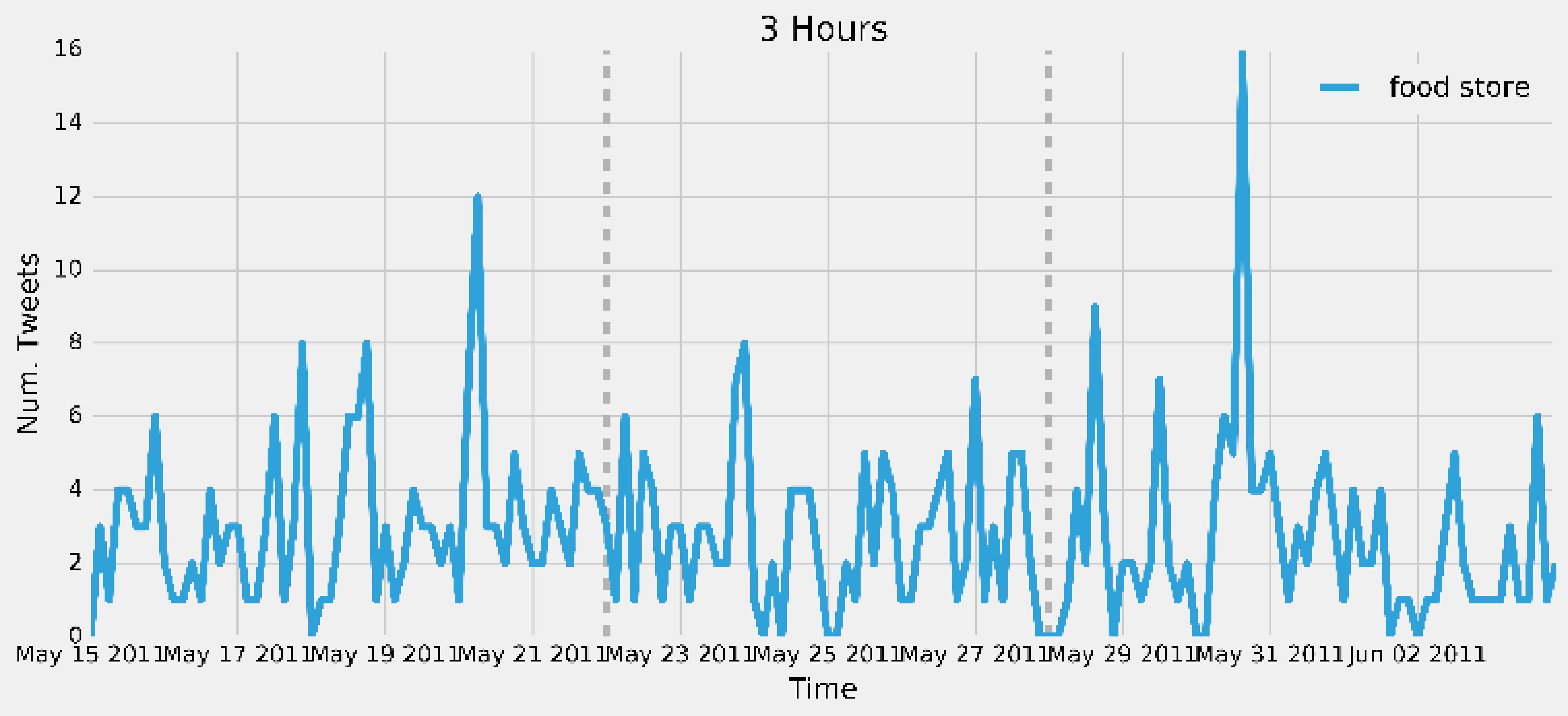
3 Hours
food store
Num. Tweets
Time
0
2
4
6
8
10
12
14
16
May 15 2011
May 17 2011
May 19 2011
May 21 2011
May 23 2011
May 25 2011
May 27 2011
May 29 2011
May 31 2011
Jun 02 2011

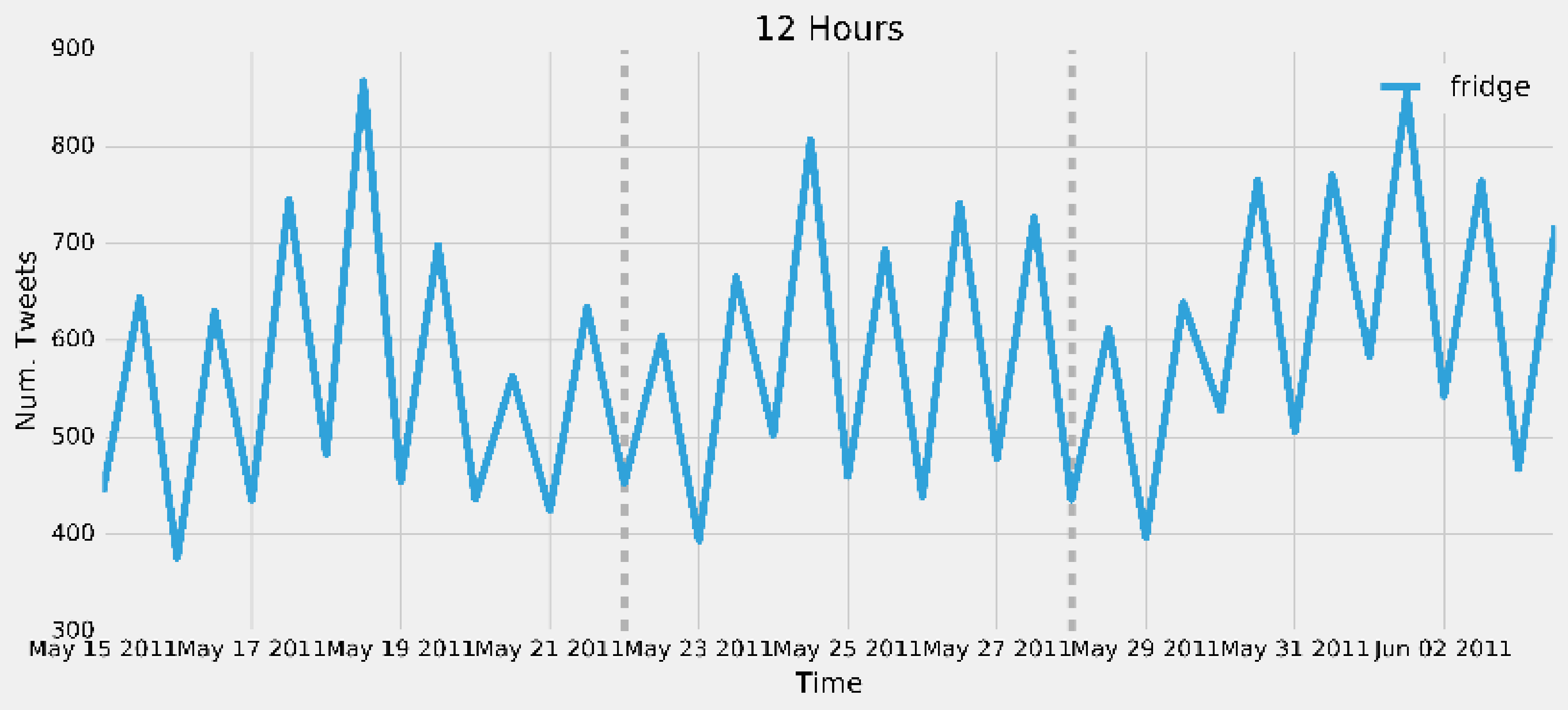
12 Hours
fridge
Num. Tweets
900
800
700
600
500
400
300
May 15 2011
May 17 2011
May 19 2011
May 21 2011
May 23 2011
May 25 2011
May 27 2011
May 29 2011
May 31 2011
Jun 02 2011
Time

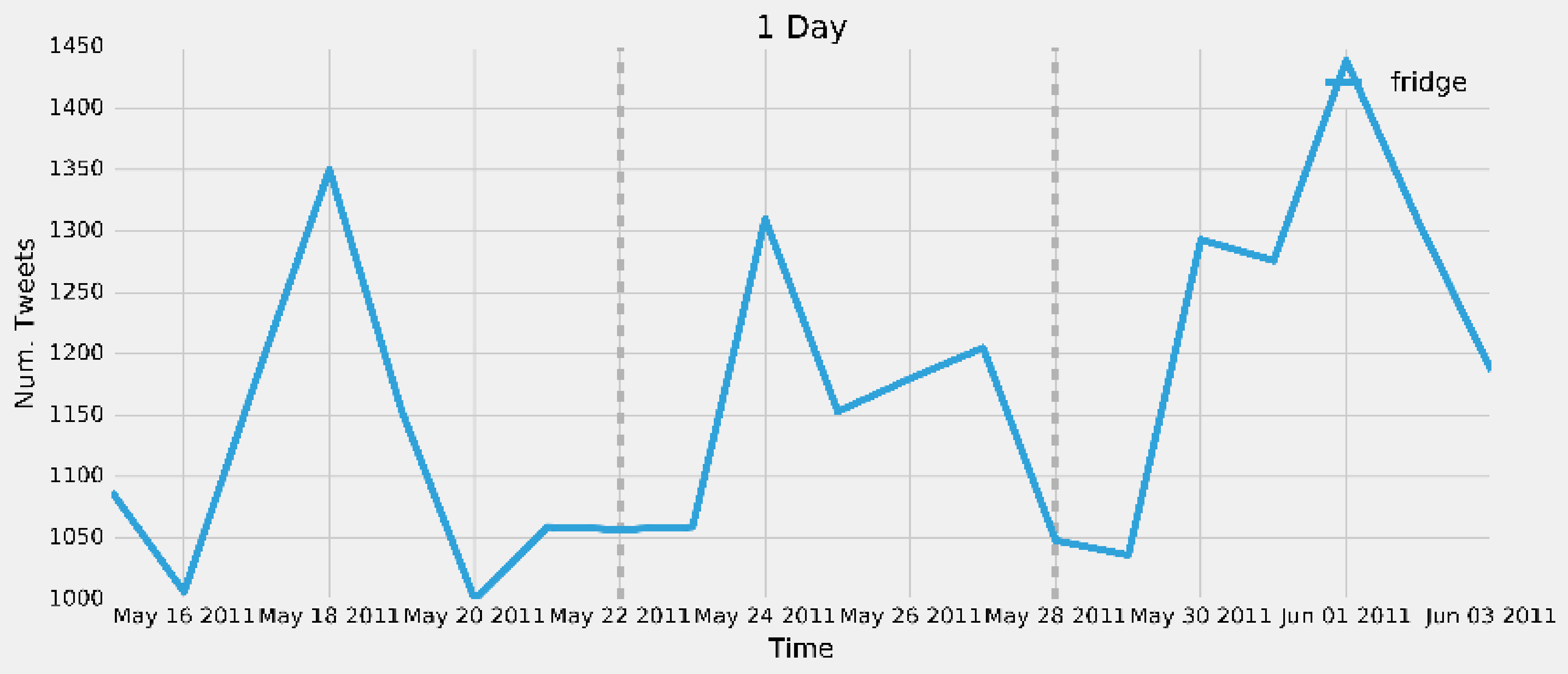

1 Day
fridge
Num. Tweets
1450
1400
1350
1300
1250
1200
1150
1100
1050
1000
May 16 2011
May 18 2011
May 20 2011
May 22 2011
May 24 2011
May 26 2011
May 28 2011
May 30 2011
Jun 01 2011
Jun 03 2011
Time

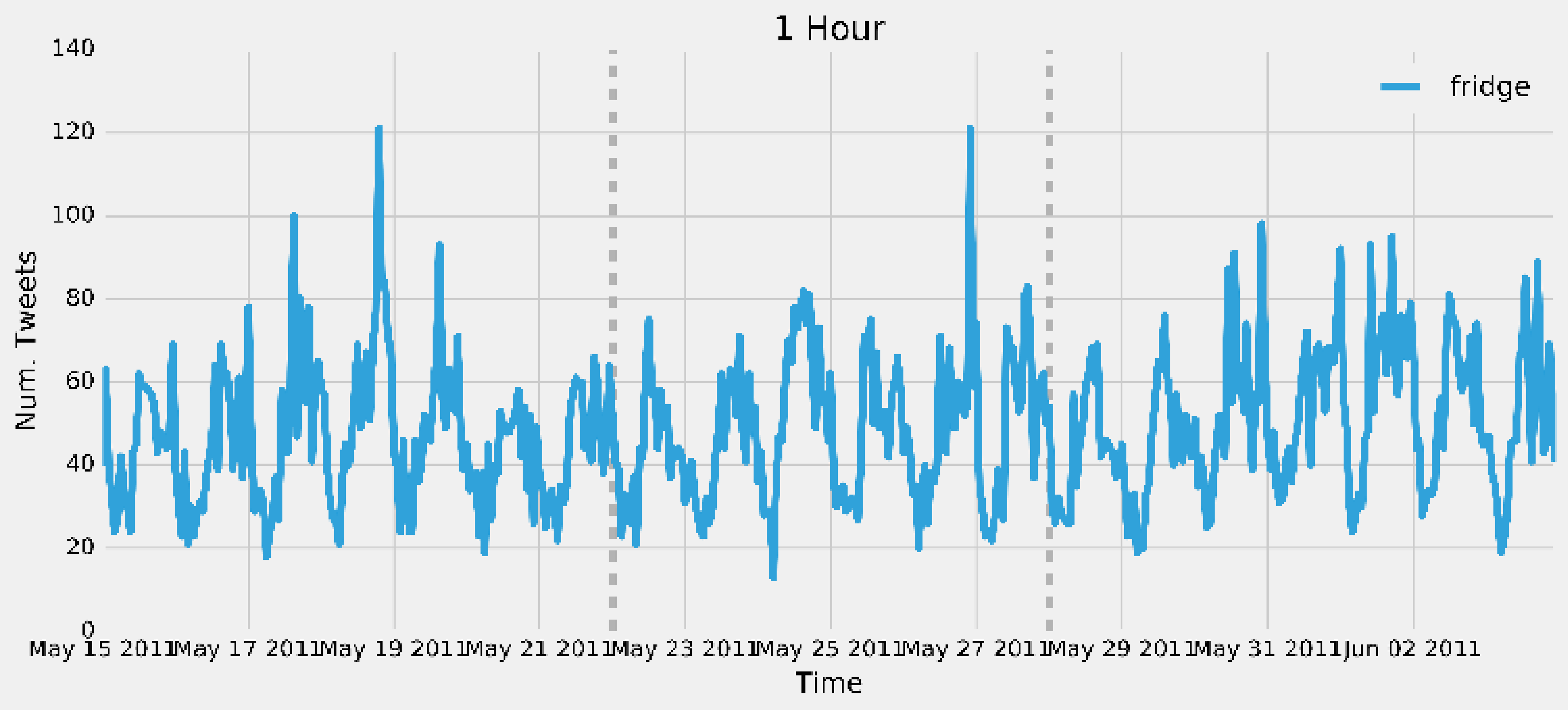

1 Hour
fridge
140
120
100
80
60
40
20
0
Num. Tweets
May 15 2011
May 17 2011
May 19 2011
May 21 2011
May 23 2011
May 25 2011
May 27 2011
May 29 2011
May 31 2011
Jun 02 2011
Time

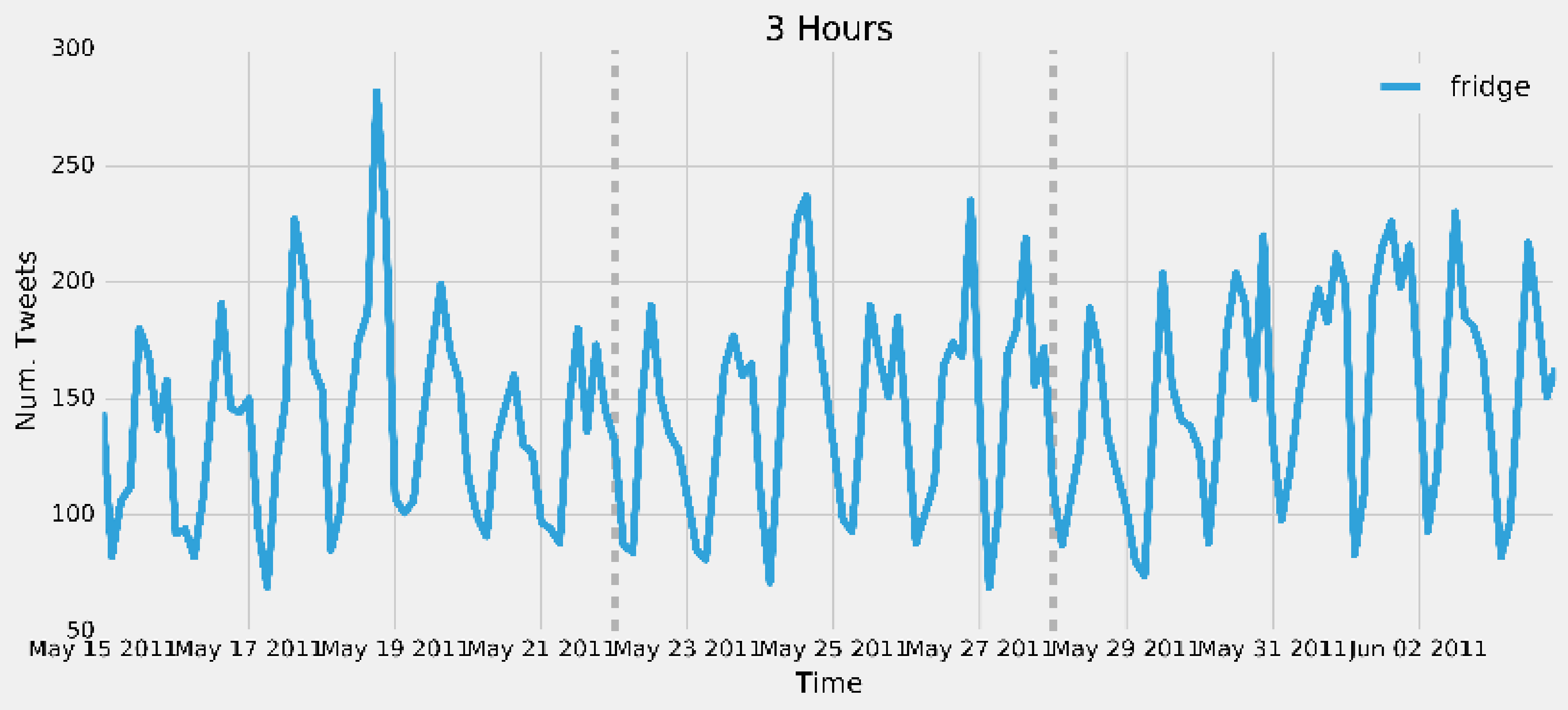
3 Hours
fridge
Num. Tweets
300
250
200
150
100
50
May 15 2011
May 17 2011
May 19 2011
May 21 2011
May 23 2011
May 25 2011
May 27 2011
May 29 2011
May 31 2011
Jun 02 2011
Time

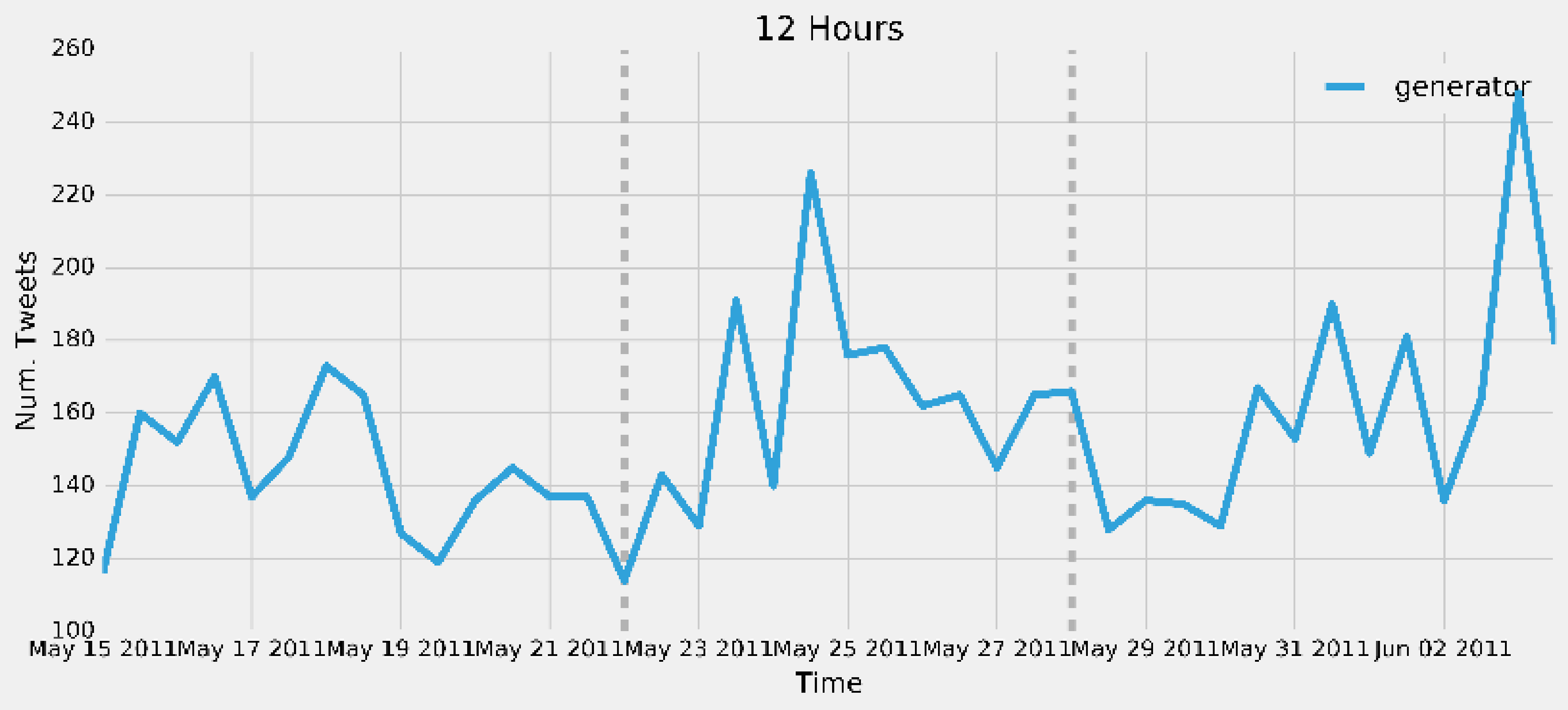
12 Hours
generator
Num. Tweets
Time
260
240
220
200
180
160
140
120
100
May 15 2011
May 17 2011
May 19 2011
May 21 2011
May 23 2011
May 25 2011
May 27 2011
May 29 2011
May 31 2011
Jun 02 2011

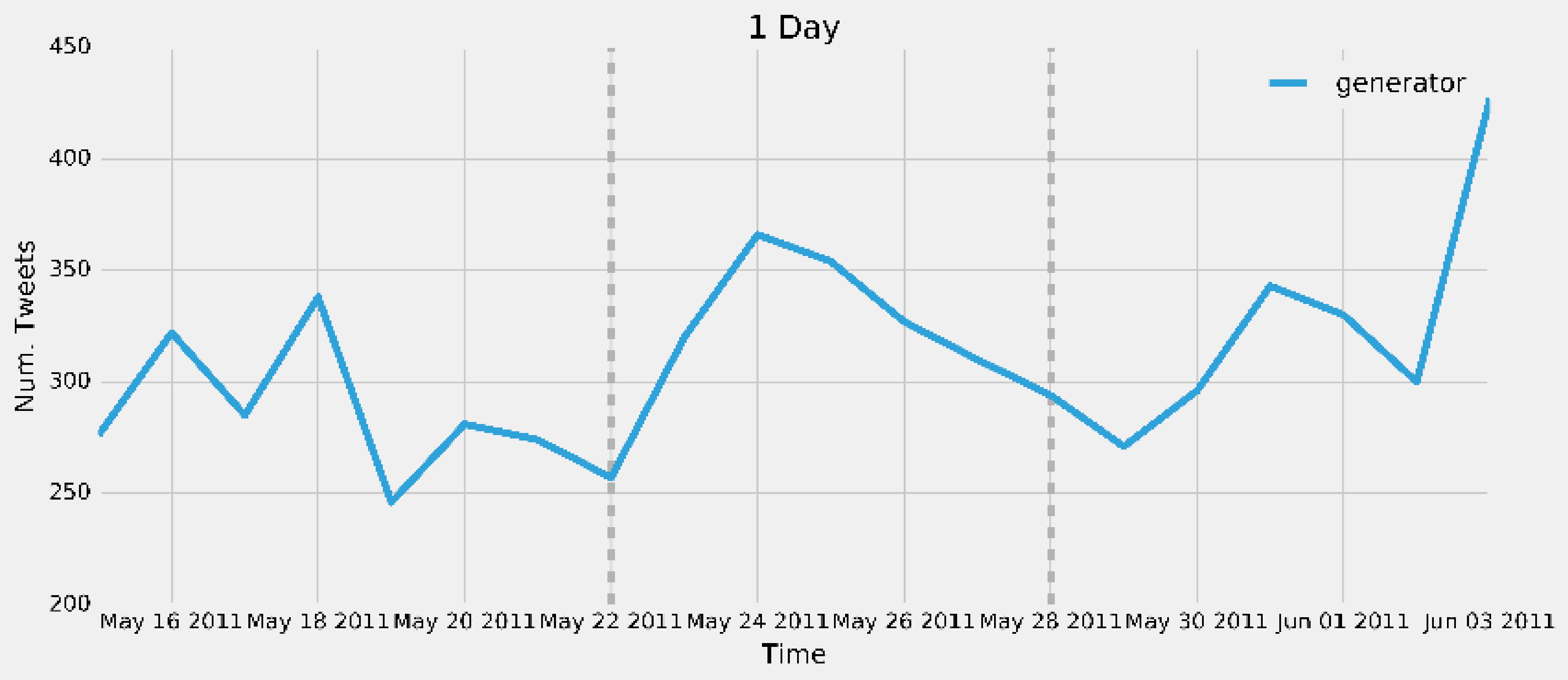
1 Day
generator
Num. Tweets
450
400
350
300
250
200
May 16 2011
May 18 2011
May 20 2011
May 22 2011
May 24 2011
May 26 2011
May 28 2011
May 30 2011
Jun 01 2011
Jun 03 2011
Time

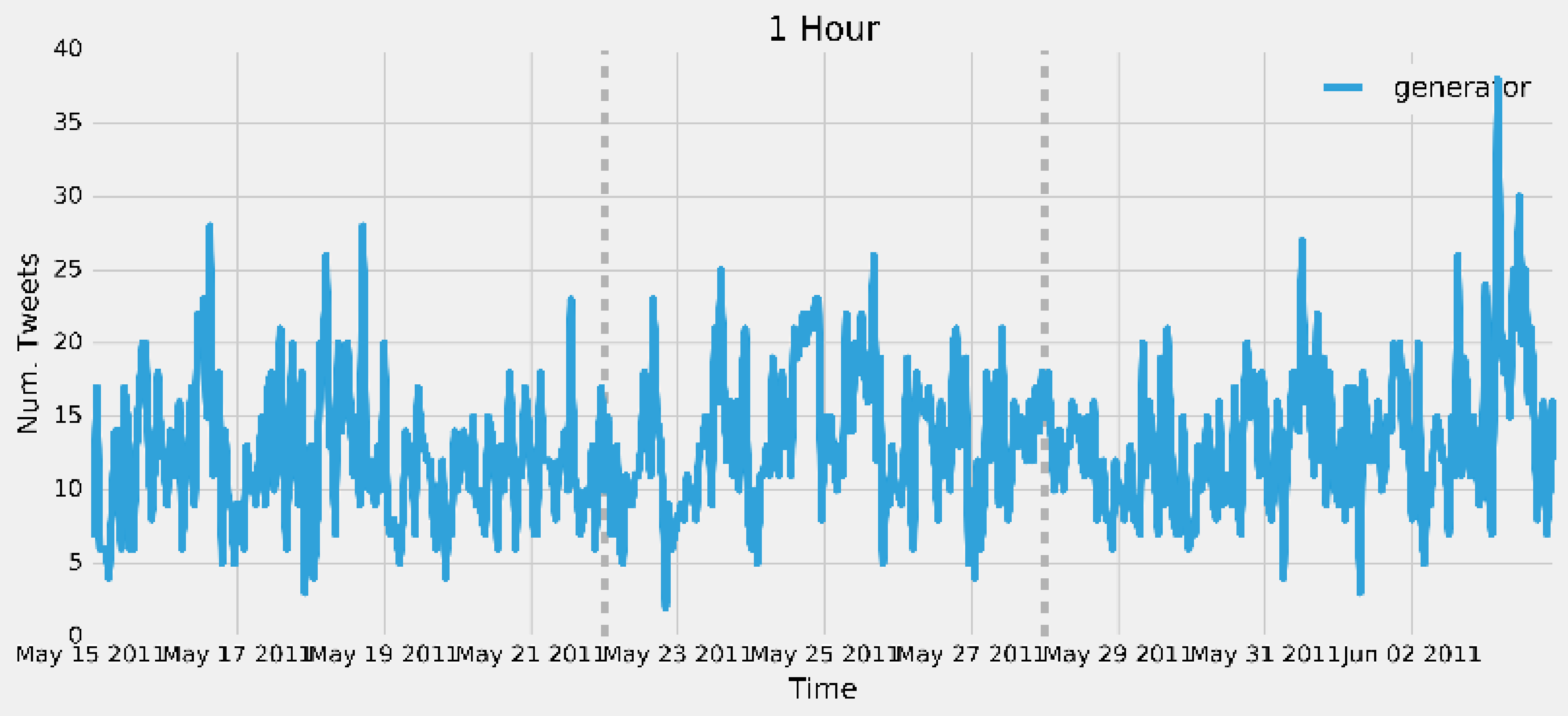

1 Hour
generator
Num. Tweets
0
5
10
15
20
25
30
35
40
May 15 2011
May 17 2011
May 19 2011
May 21 2011
May 23 2011
May 25 2011
May 27 2011
May 29 2011
May 31 2011
Jun 02 2011
Time

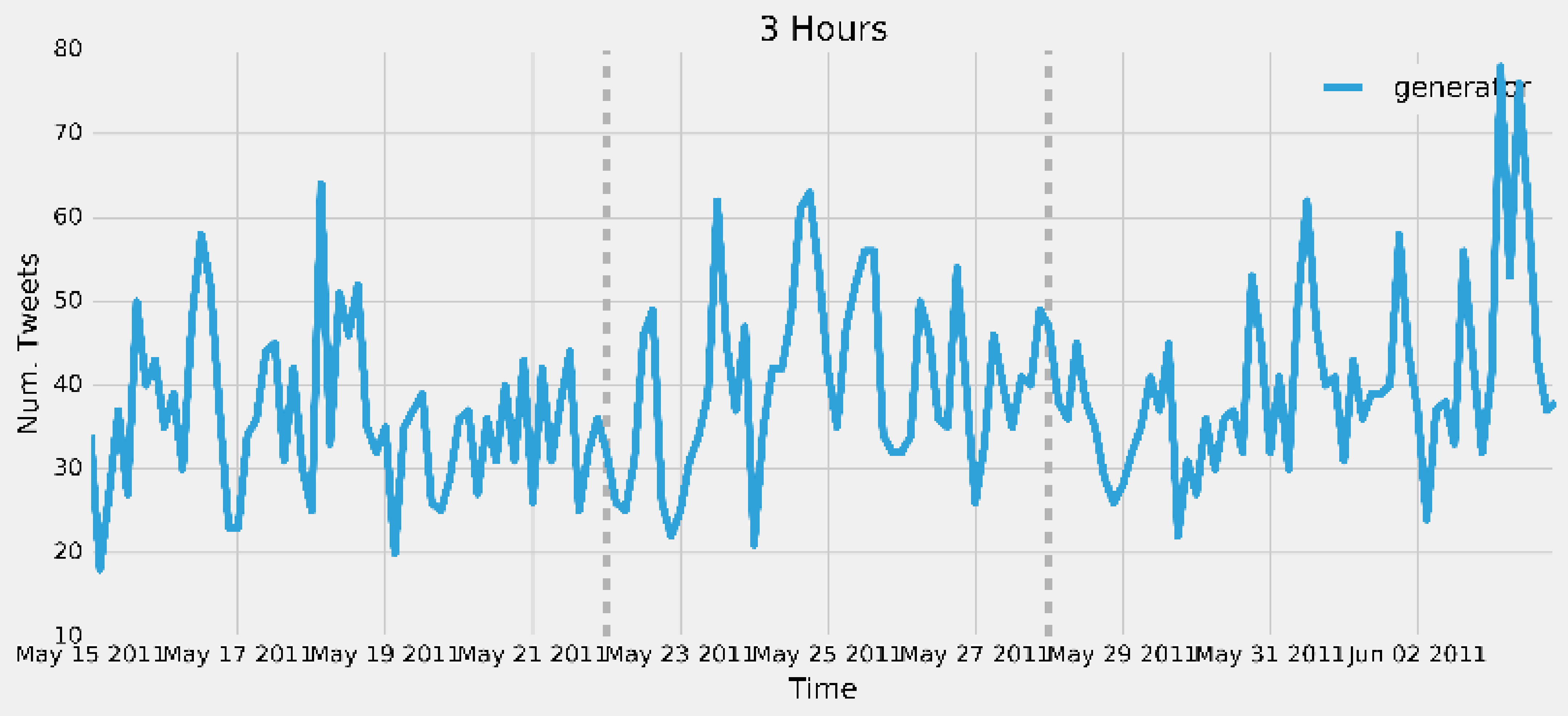
3 Hours
generator
Num. Tweets
80
70
60
50
40
30
20
10
May 15 2011
May 17 2011
May 19 2011
May 21 2011
May 23 2011
May 25 2011
May 27 2011
May 29 2011
May 31 2011
Jun 02 2011
Time

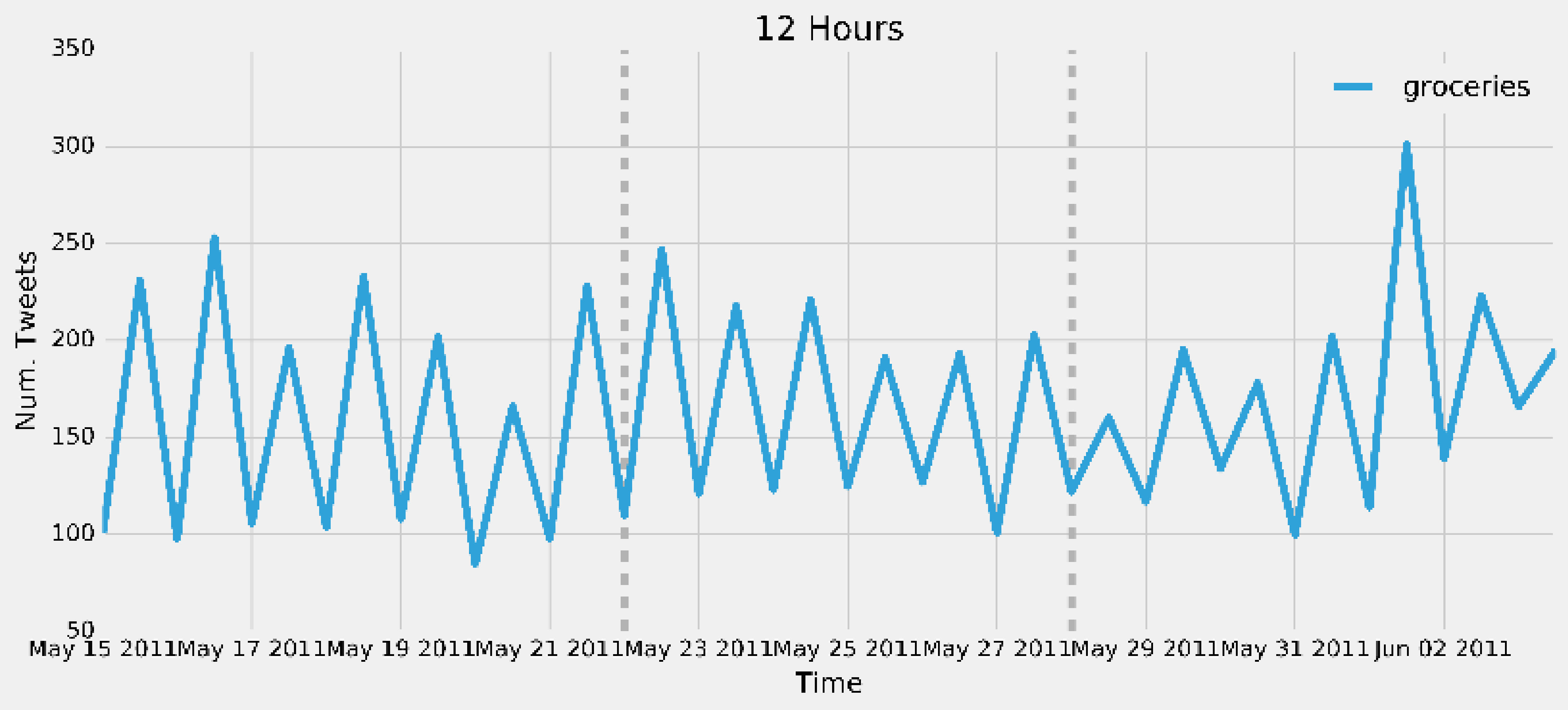

12 Hours
groceries
350
300
250
200
150
100
50
Num. Tweets
May 15 2011
May 17 2011
May 19 2011
May 21 2011
May 23 2011
May 25 2011
May 27 2011
May 29 2011
May 31 2011
Jun 02 2011
Time

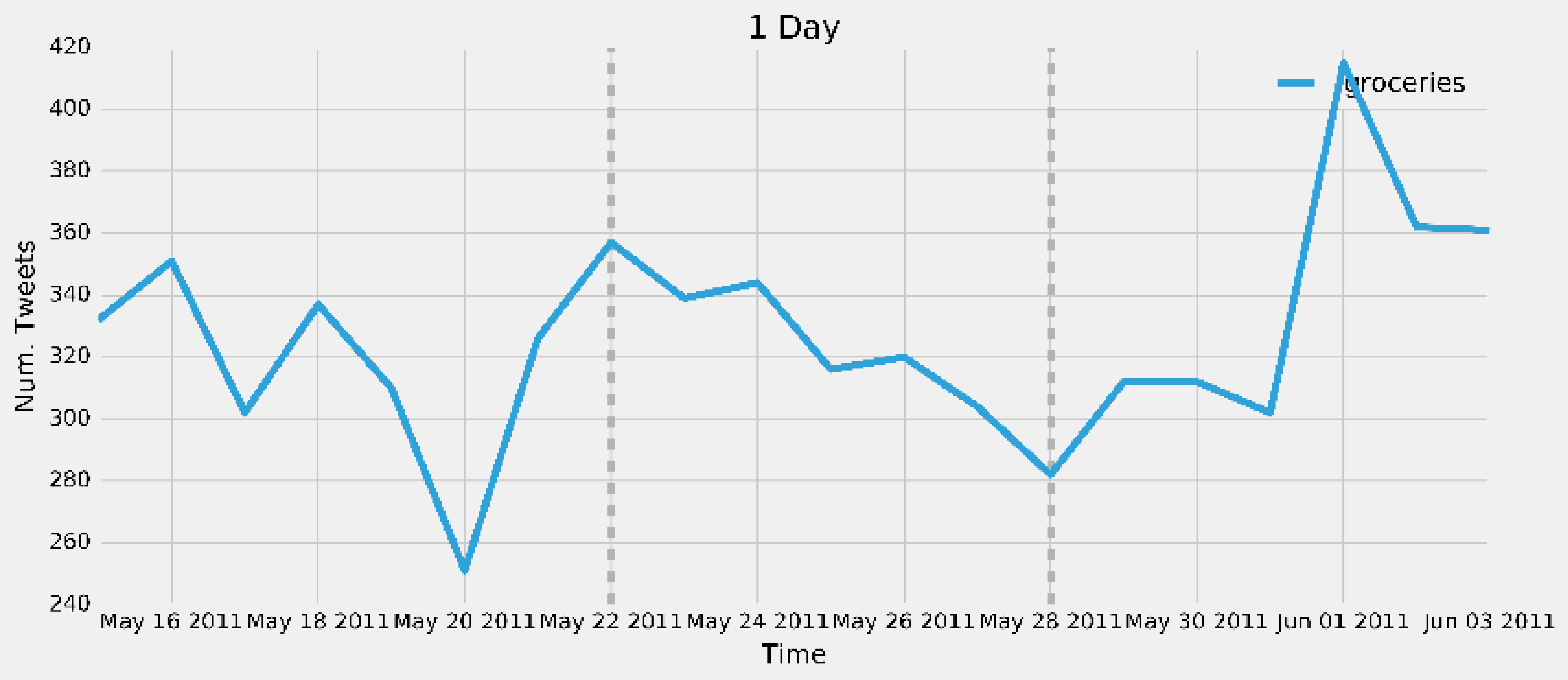
1 Day
groceries
420
400
380
360
340
320
300
280
260
240
Num. Tweets
May 16 2011
May 18 2011
May 20 2011
May 22 2011
May 24 2011
May 26 2011
May 28 2011
May 30 2011
Jun 01 2011
Jun 03 2011
Time

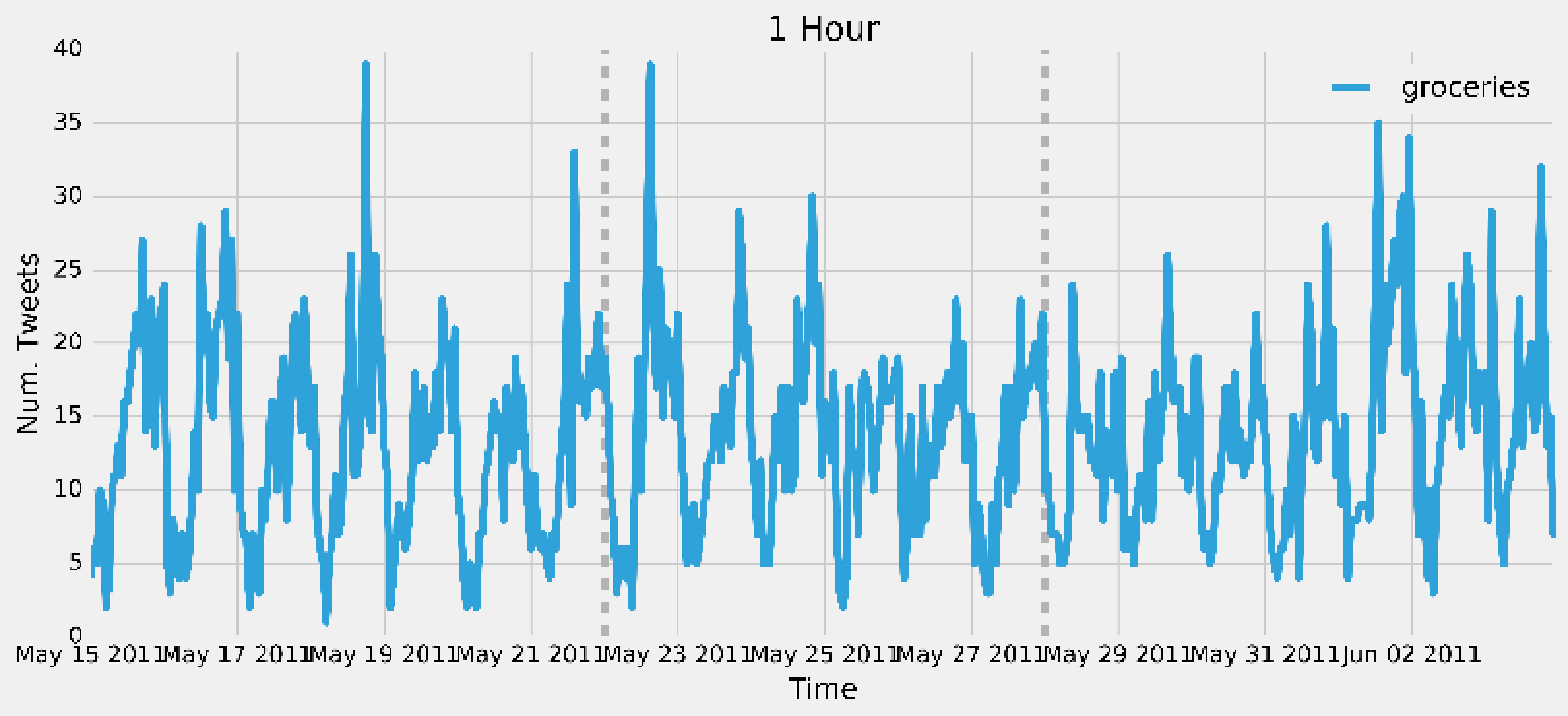

1 Hour
groceries
Num. Tweets
0
5
10
15
20
25
30
35
40
May 15 2011
May 17 2011
May 19 2011
May 21 2011
May 23 2011
May 25 2011
May 27 2011
May 29 2011
May 31 2011
Jun 02 2011
Time

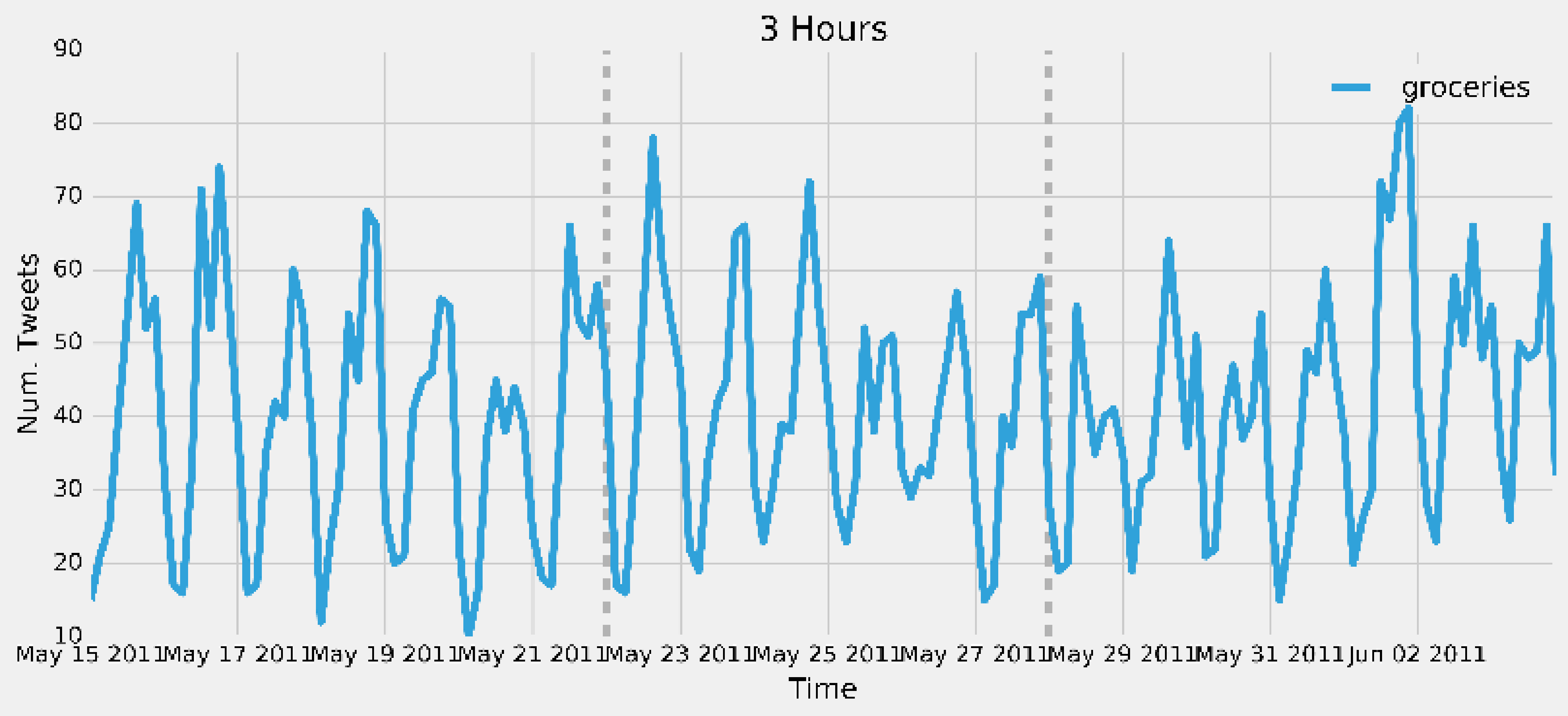

3 Hours
groceries
Num. Tweets
90
80
70
60
50
40
30
20
10
May 15 2011
May 17 2011
May 19 2011
May 21 2011
May 23 2011
May 25 2011
May 27 2011
May 29 2011
May 31 2011
Jun 02 2011
Time

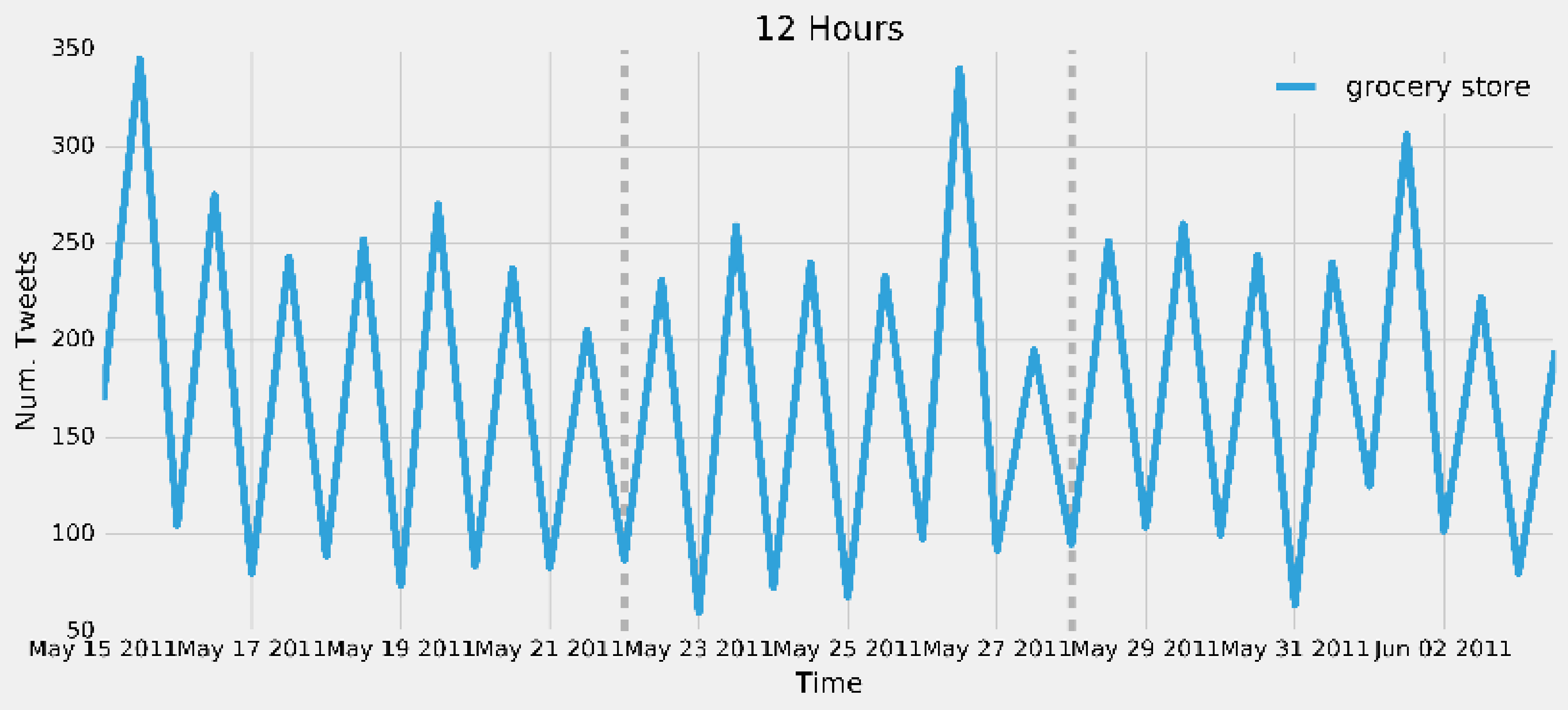
12 Hours
grocery store
Num. Tweets
350
300
250
200
150
100
50
May 15 2011
May 17 2011
May 19 2011
May 21 2011
May 23 2011
May 25 2011
May 27 2011
May 29 2011
May 31 2011
Jun 02 2011
Time

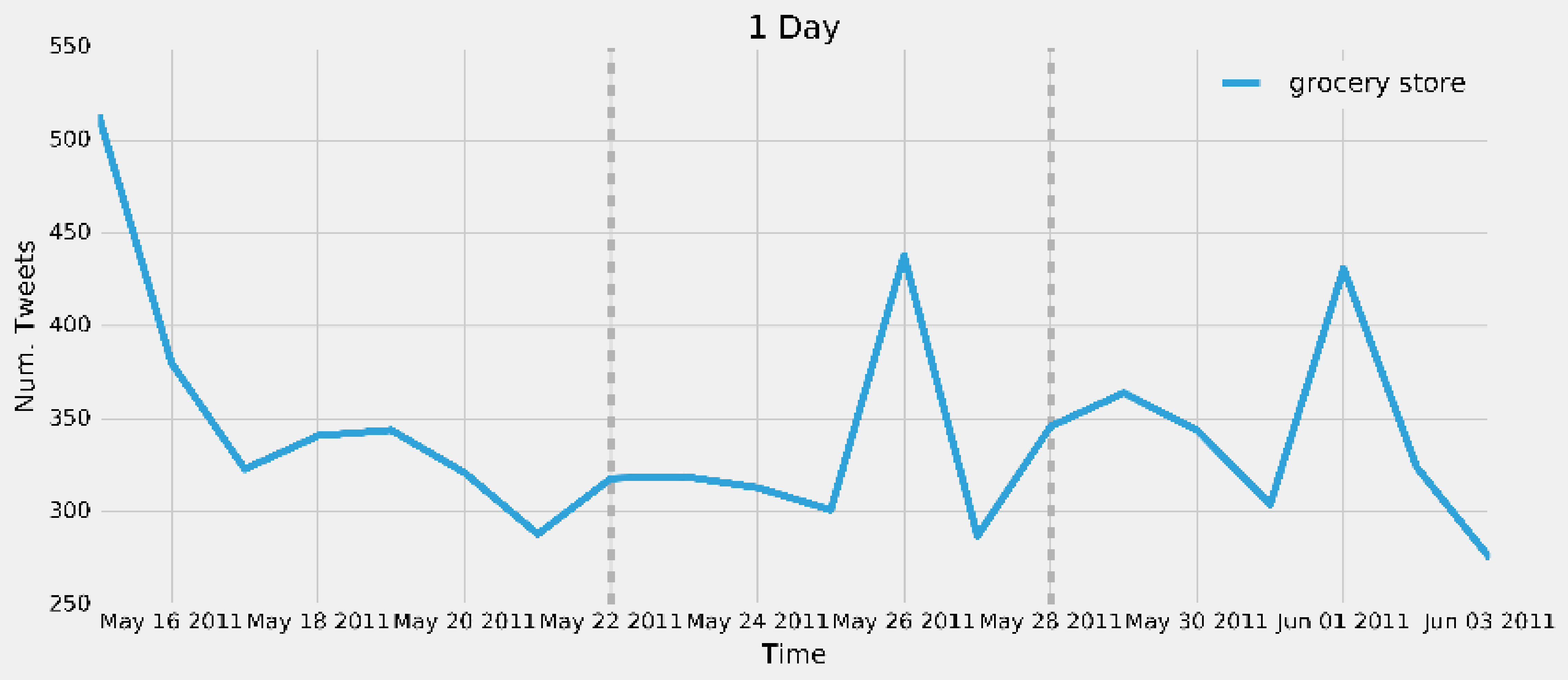
1 Day
grocery store
Num. Tweets
Time
550
500
450
400
350
300
250
May 16 2011
May 18 2011
May 20 2011
May 22 2011
May 24 2011
May 26 2011
May 28 2011
May 30 2011
Jun 01 2011
Jun 03 2011

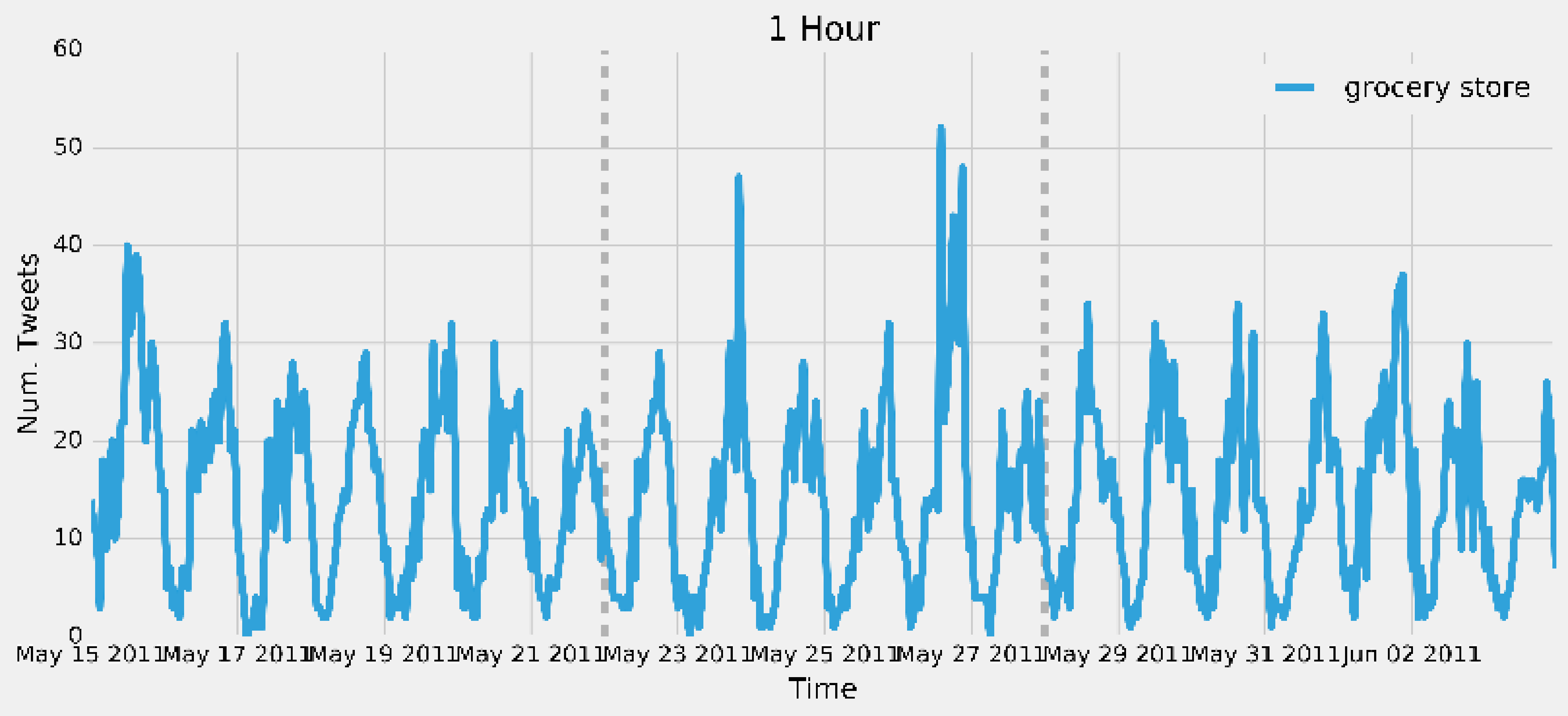

1 Hour
grocery store
Num. Tweets
60
50
40
30
20
10
0
May 15 2011
May 17 2011
May 19 2011
May 21 2011
May 23 2011
May 25 2011
May 27 2011
May 29 2011
May 31 2011
Jun 02 2011
Time

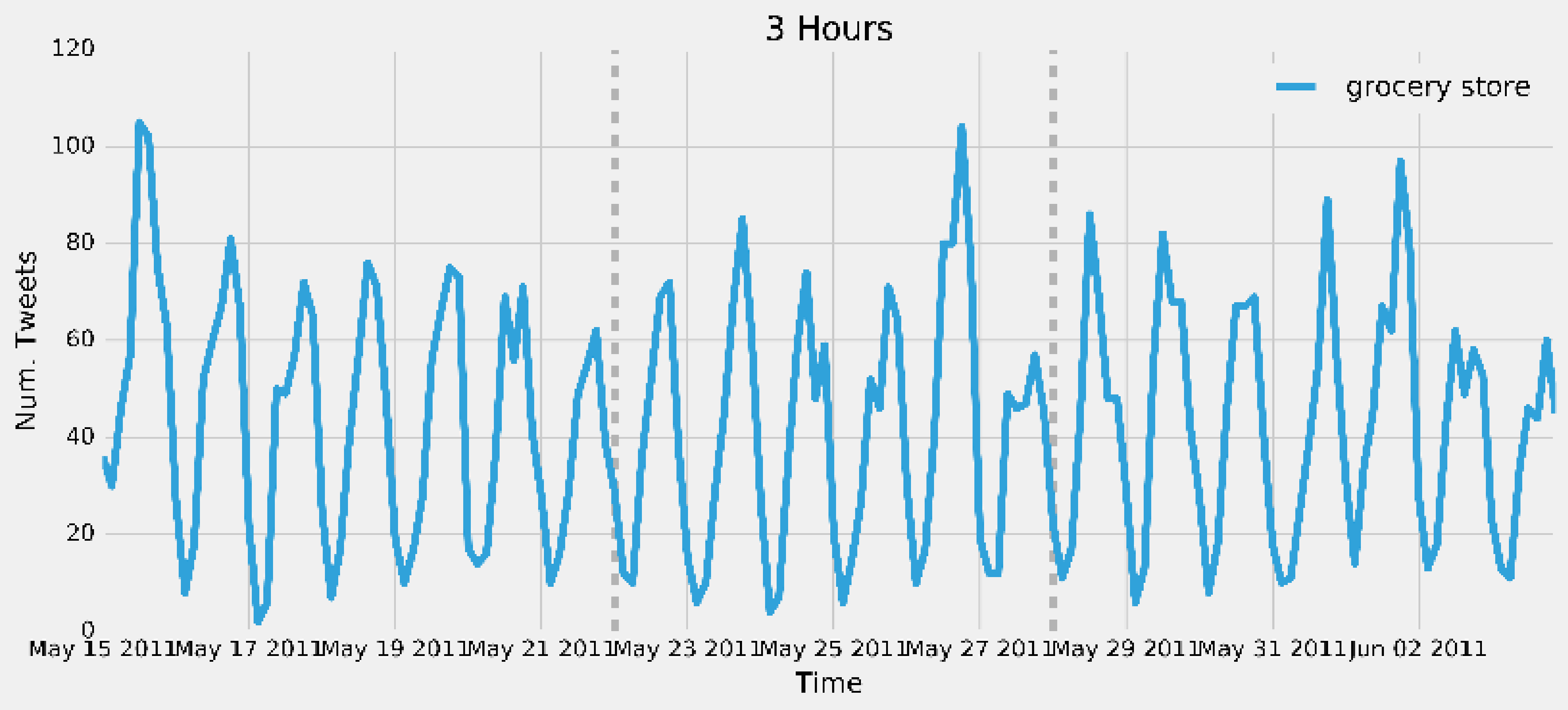

3 Hours
grocery store
Num. Tweets
120
100
80
60
40
20
0
May 15 2011
May 17 2011
May 19 2011
May 21 2011
May 23 2011
May 25 2011
May 27 2011
May 29 2011
May 31 2011
Jun 02 2011
Time

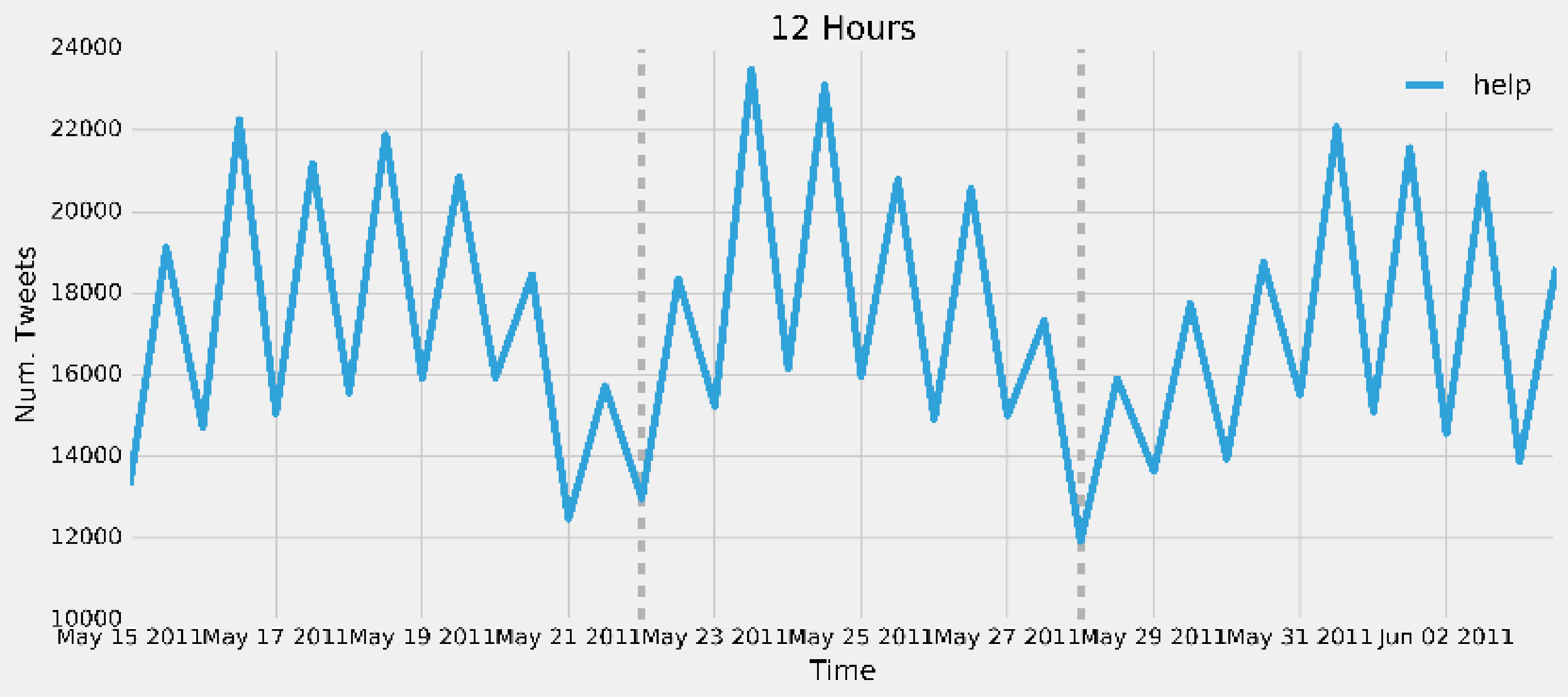

12 Hours
help
24000
22000
20000
18000
16000
14000
12000
10000
Num. Tweets
May 15 2011
May 17 2011
May 19 2011
May 21 2011
May 23 2011
May 25 2011
May 27 2011
May 29 2011
May 31 2011
Jun 02 2011
Time

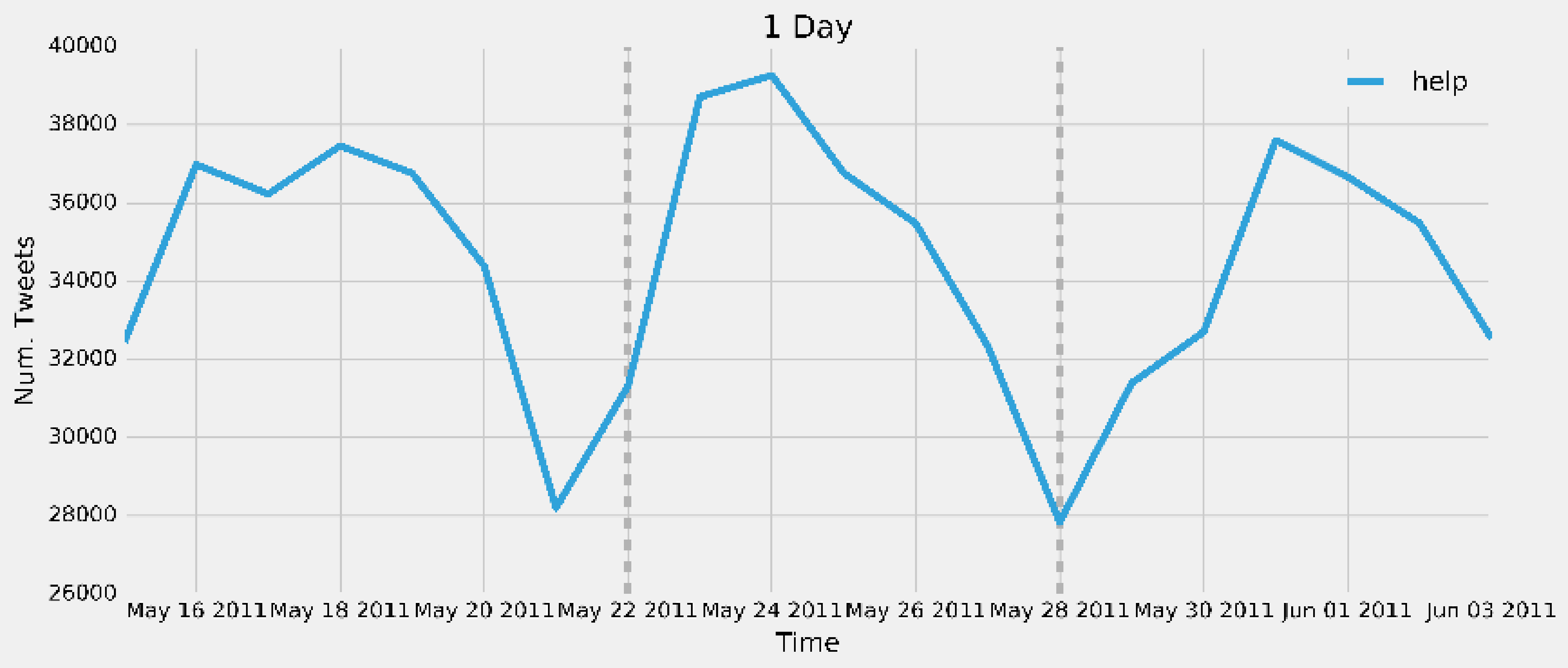

1 Day
help
Num. Tweets
40000
38000
36000
34000
32000
30000
28000
26000
May 16 2011
May 18 2011
May 20 2011
May 22 2011
May 24 2011
May 26 2011
May 28 2011
May 30 2011
Jun 01 2011
Jun 03 2011
Time

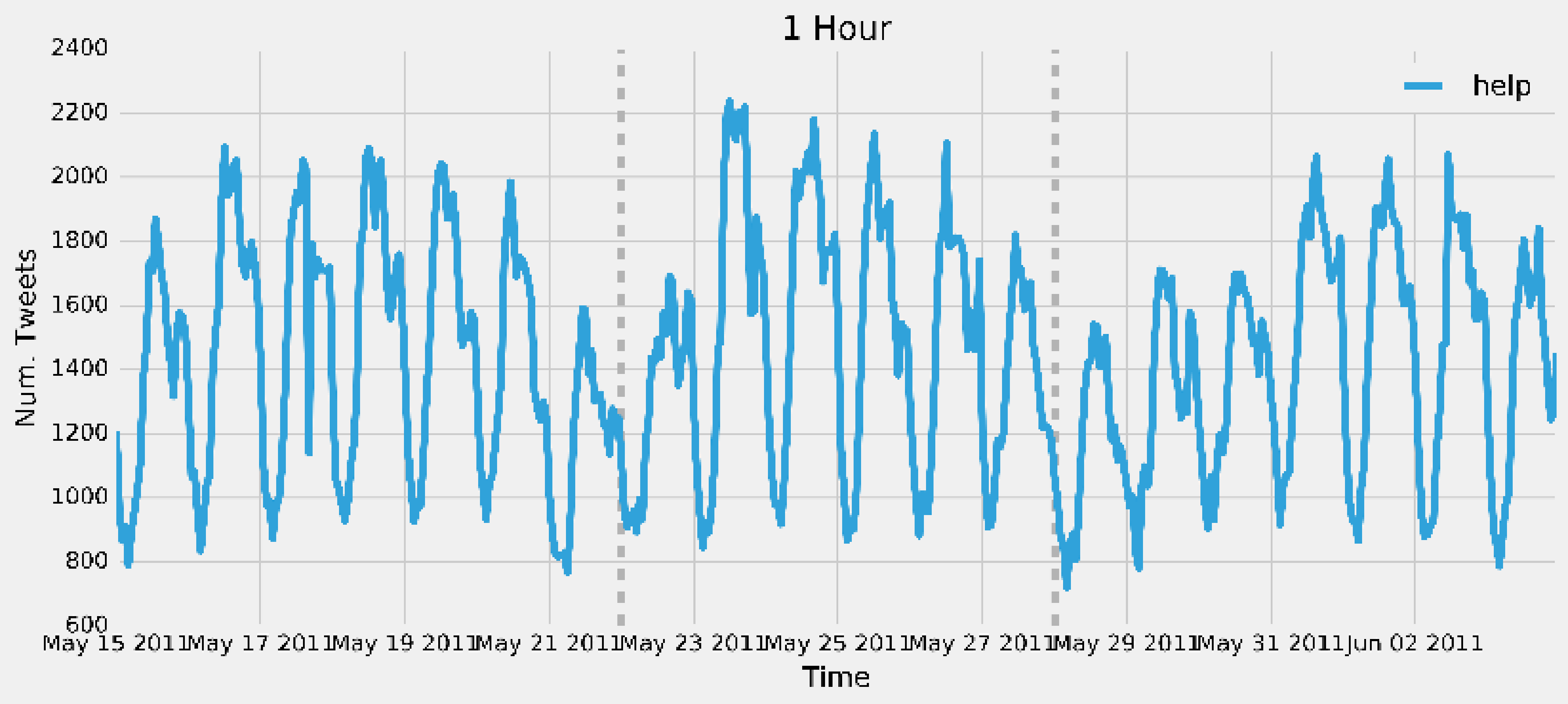
1 Hour
help
Num. Tweets
2400
2200
2000
1800
1600
1400
1200
1000
800
600
May 15 2011
May 17 2011
May 19 2011
May 21 2011
May 23 2011
May 25 2011
May 27 2011
May 29 2011
May 31 2011
Jun 02 2011
Time

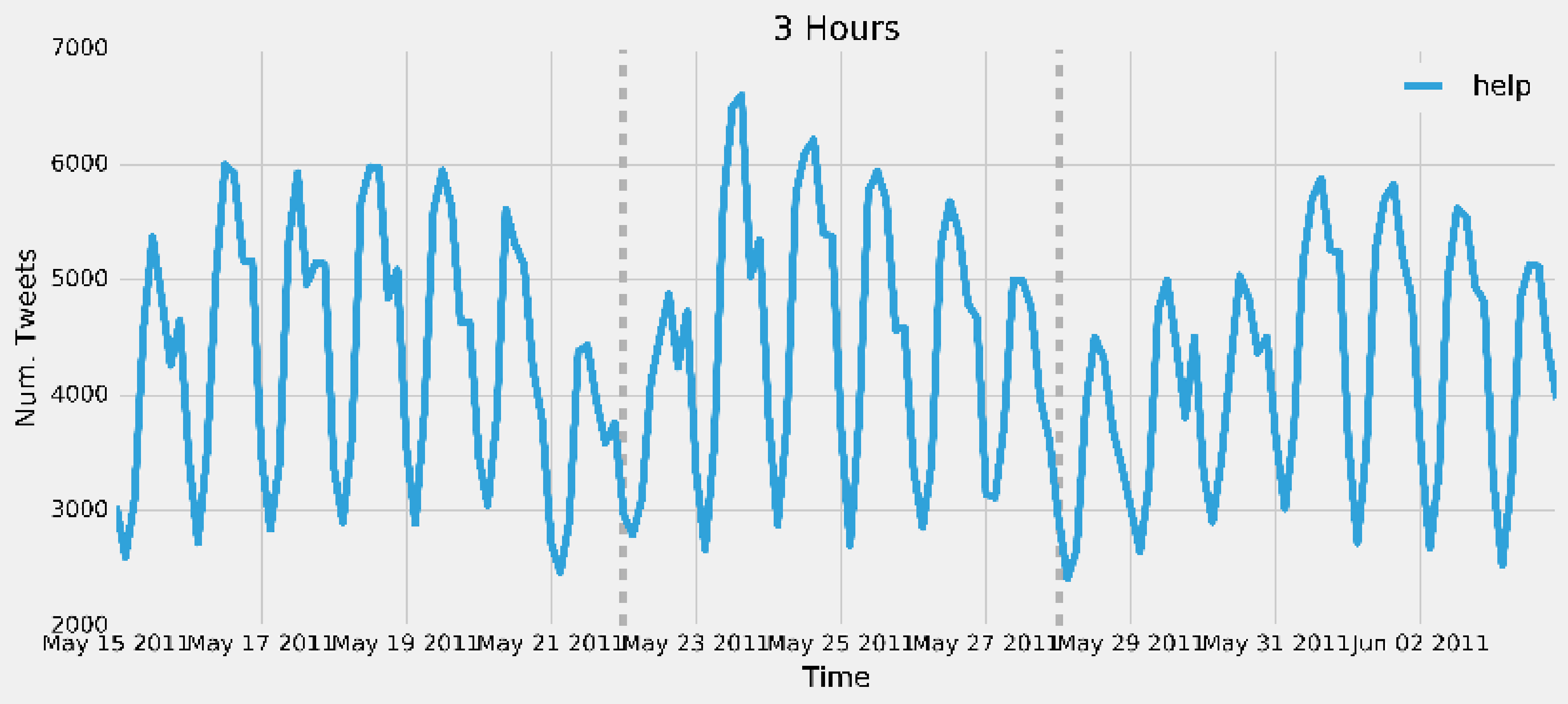

3 Hours
help
Num. Tweets
7000
6000
5000
4000
3000
2000
May 15 2011
May 17 2011
May 19 2011
May 21 2011
May 23 2011
May 25 2011
May 27 2011
May 29 2011
May 31 2011
Jun 02 2011
Time

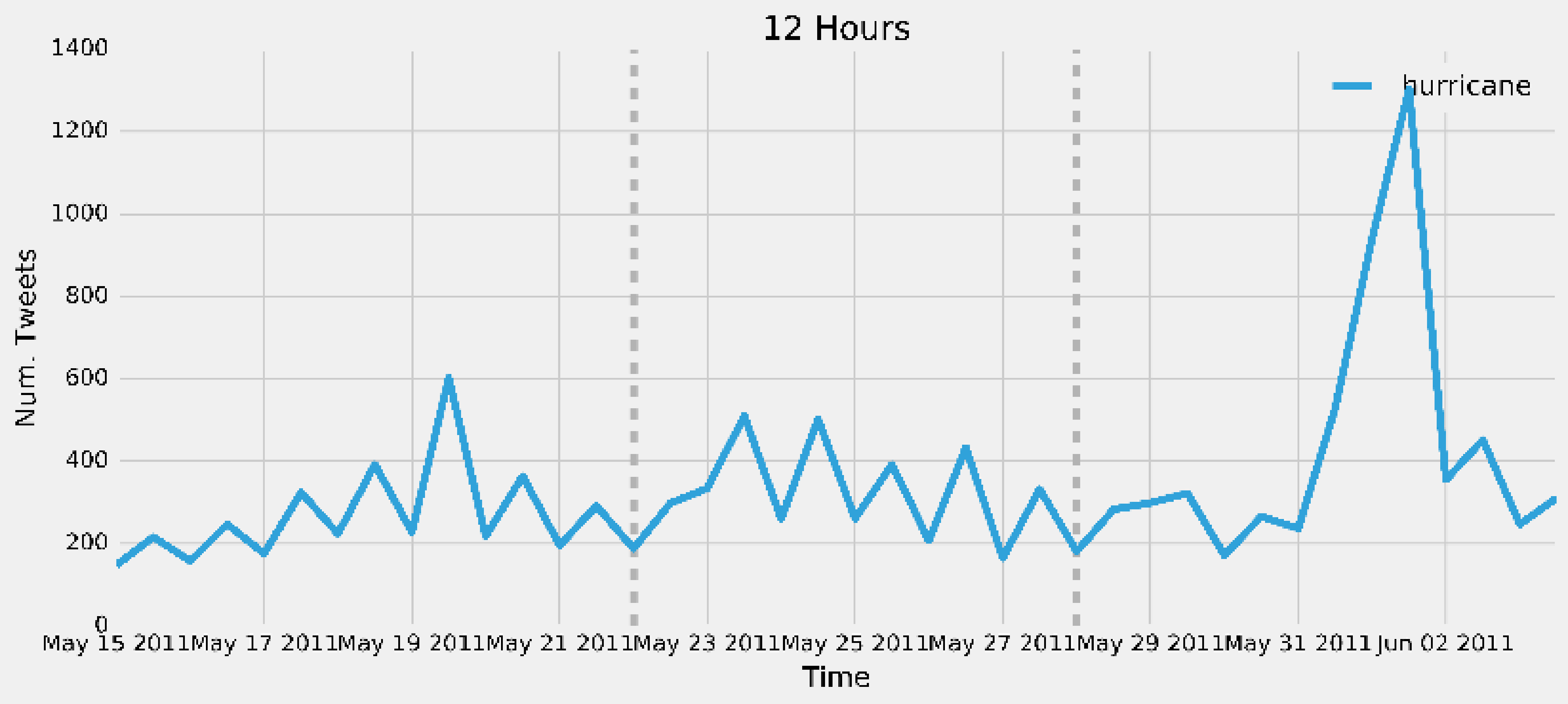
12 Hours
hurricane
Num. Tweets
0
200
400
600
800
1000
1200
1400
May 15 2011
May 17 2011
May 19 2011
May 21 2011
May 23 2011
May 25 2011
May 27 2011
May 29 2011
May 31 2011
Jun 02 2011
Time

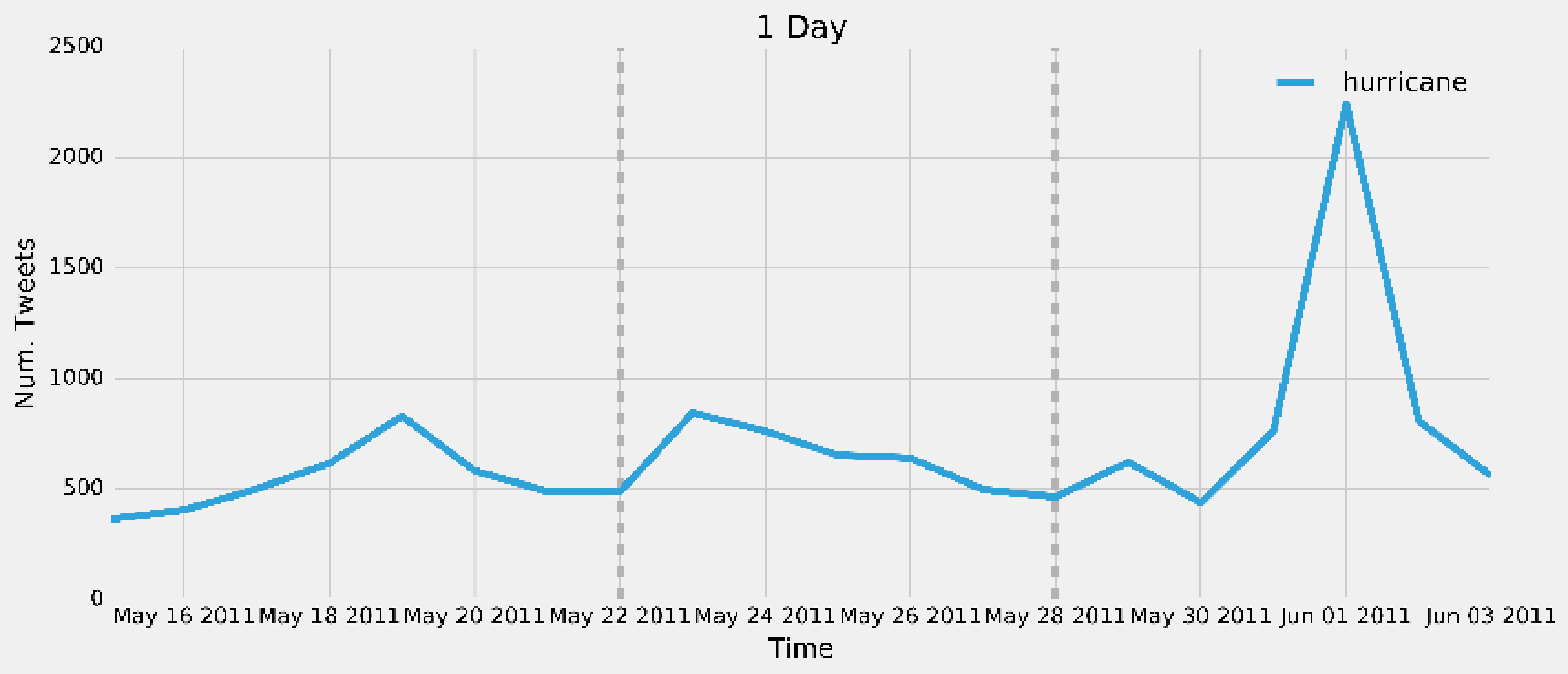

1 Day
hurricane
Num. Tweets
Time
0
500
1000
1500
2000
2500
May 16 2011
May 18 2011
May 20 2011
May 22 2011
May 24 2011
May 26 2011
May 28 2011
May 30 2011
Jun 01 2011
Jun 03 2011

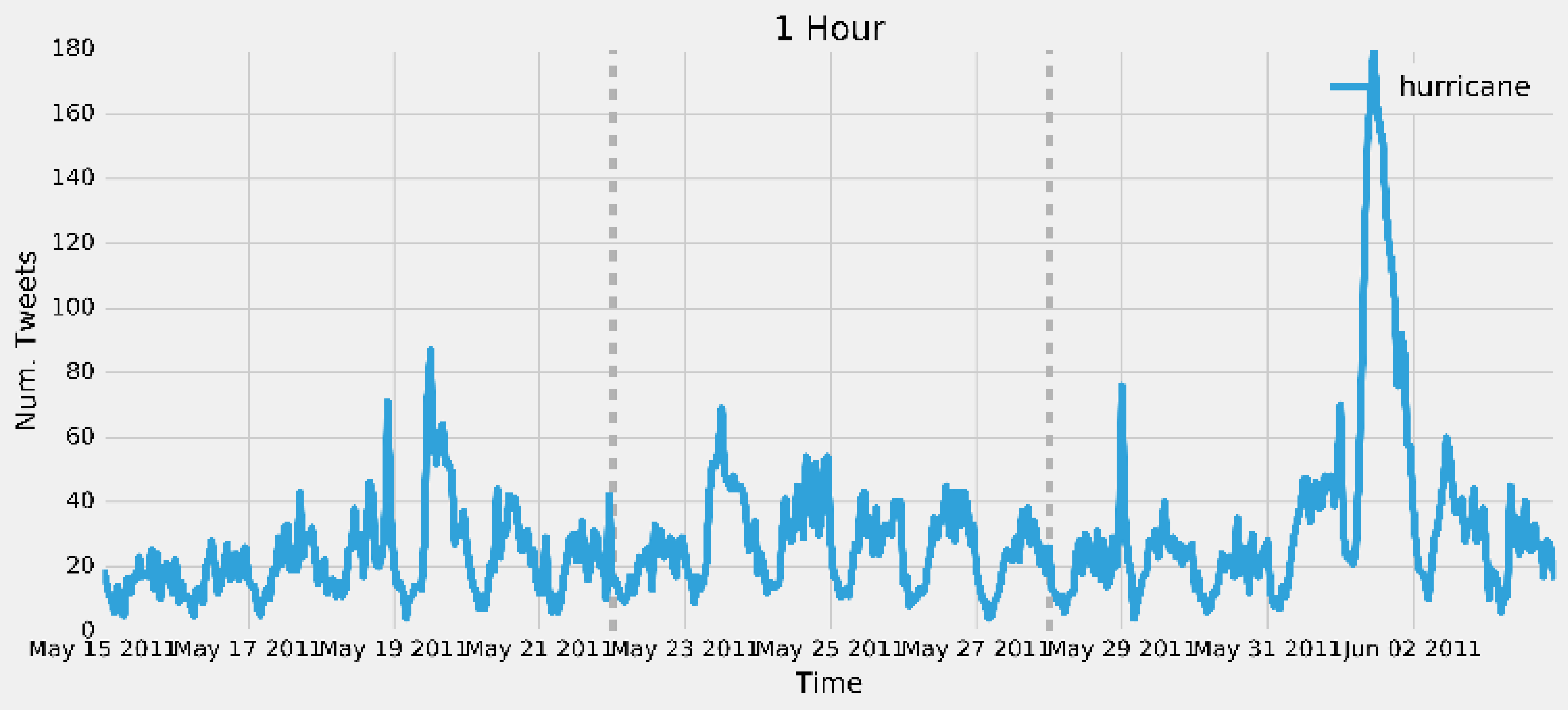

1 Hour
hurricane
Num. Tweets
Time
0
20
40
60
80
100
120
140
160
180
May 15 2011
May 17 2011
May 19 2011
May 21 2011
May 23 2011
May 25 2011
May 27 2011
May 29 2011
May 31 2011
Jun 02 2011

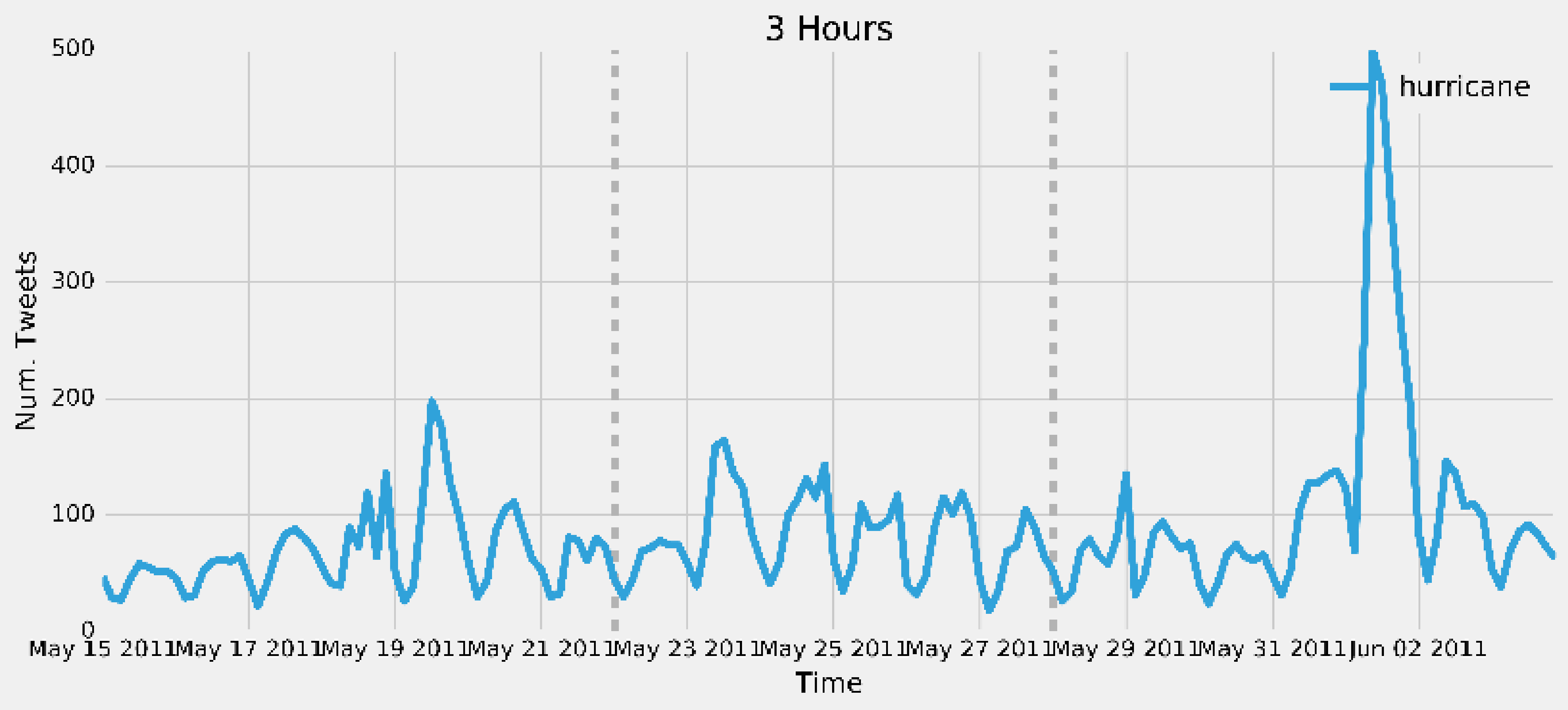
3 Hours
hurricane
Num. Tweets
0
100
200
300
400
500
May 15 2011
May 17 2011
May 19 2011
May 21 2011
May 23 2011
May 25 2011
May 27 2011
May 29 2011
May 31 2011
Jun 02 2011
Time

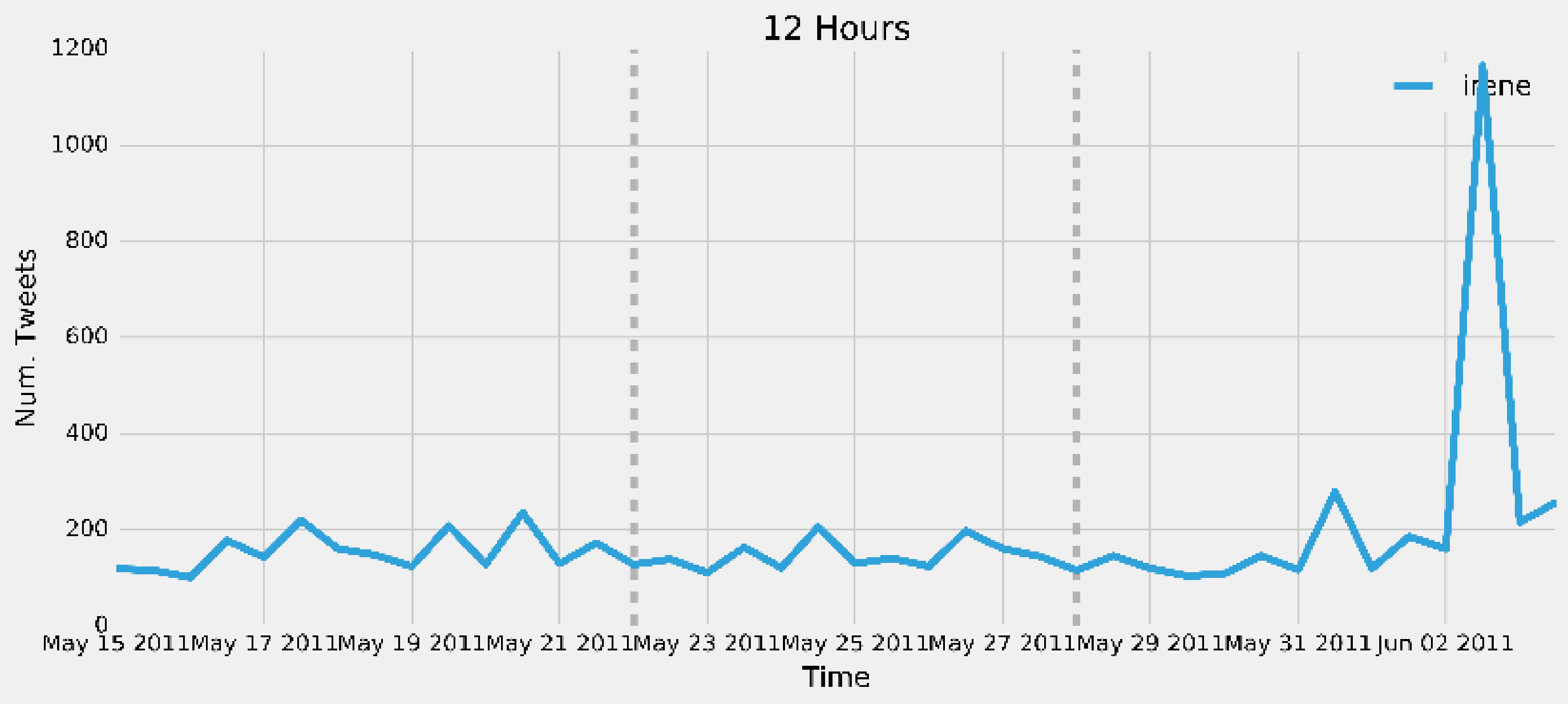
12 Hours
Num. Tweets
Time
irene
0
200
400
600
800
1000
1200
May 15 2011
May 17 2011
May 19 2011
May 21 2011
May 23 2011
May 25 2011
May 27 2011
May 29 2011
May 31 2011
Jun 02 2011

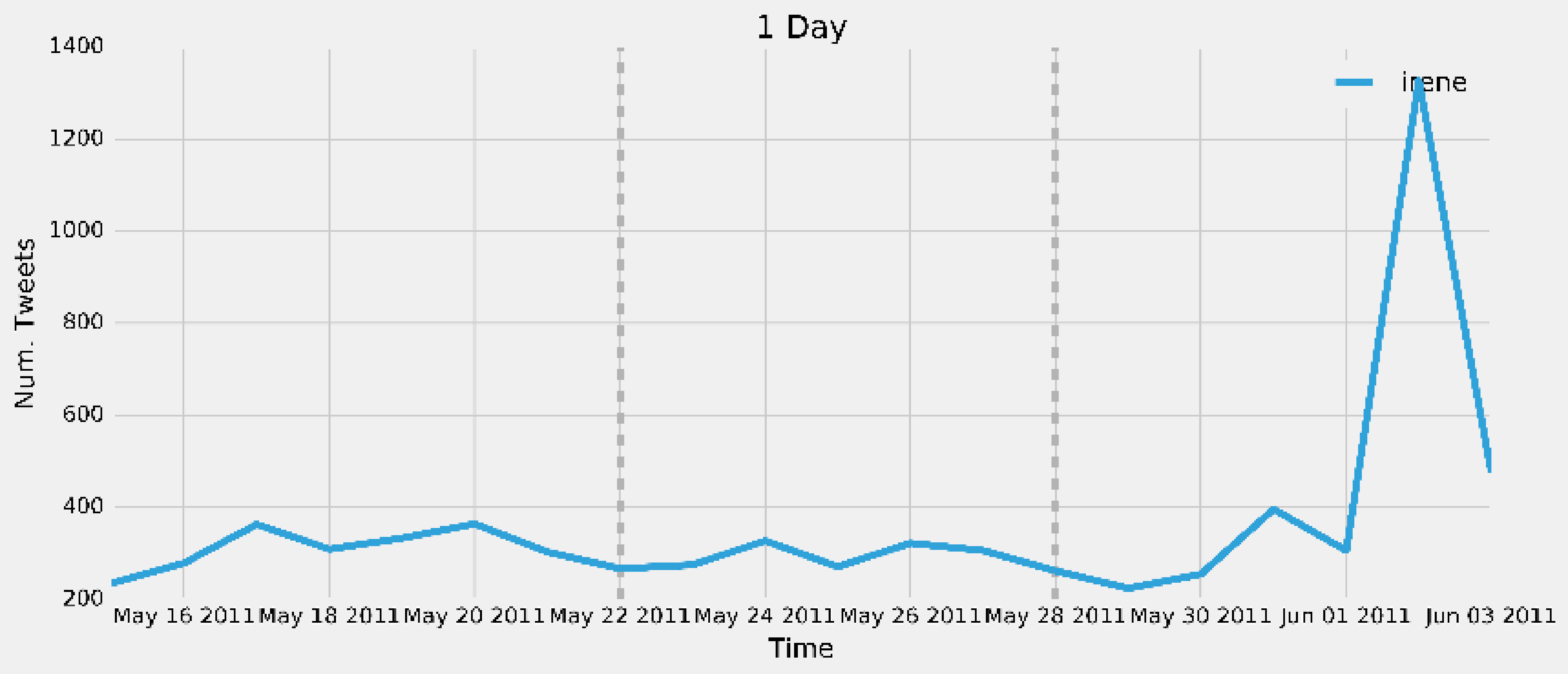
1 Day
Num. Tweets
Time
irene
1400
1200
1000
800
600
400
200
May 16 2011
May 18 2011
May 20 2011
May 22 2011
May 24 2011
May 26 2011
May 28 2011
May 30 2011
Jun 01 2011
Jun 03 2011

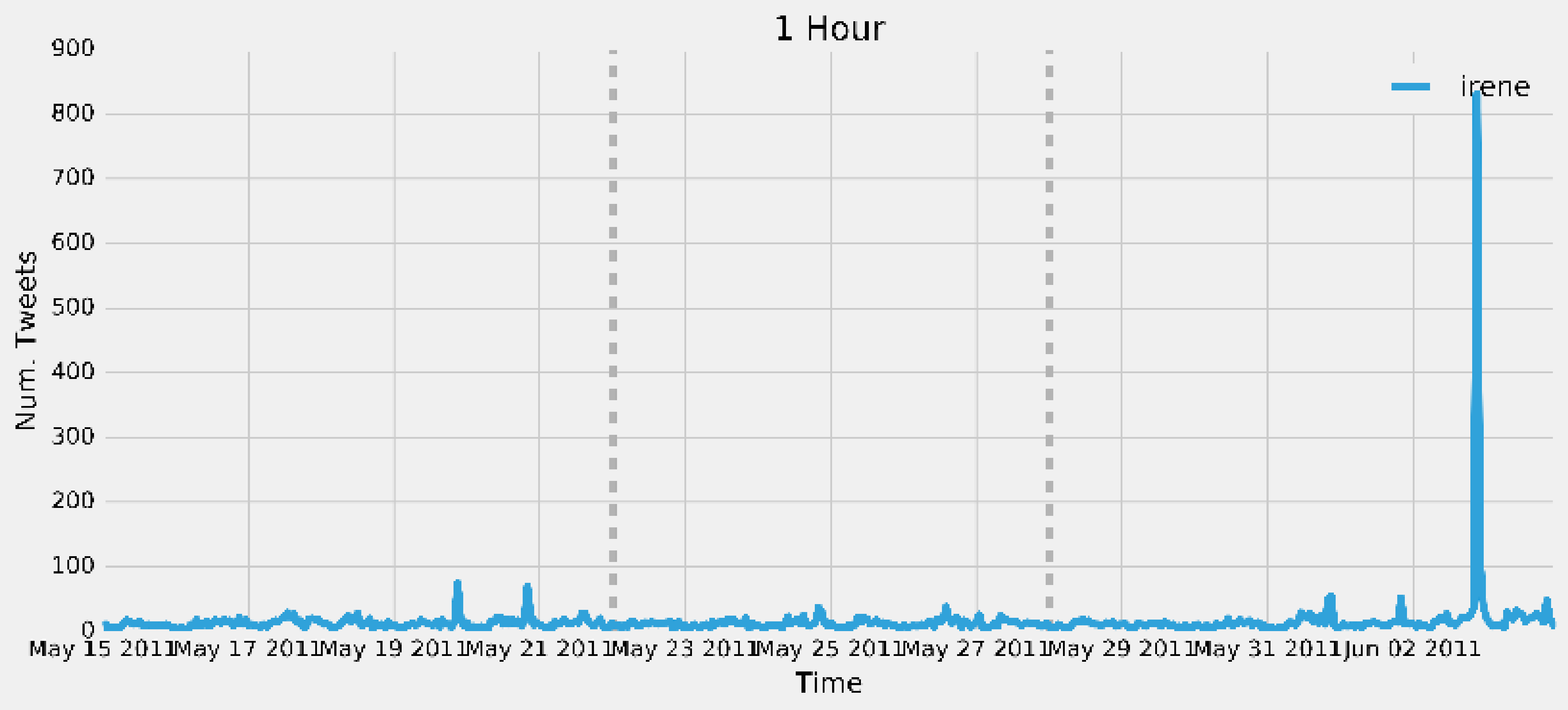

1 Hour
irene
Num. Tweets
0
100
200
300
400
500
600
700
800
900
May 15 2011
May 17 2011
May 19 2011
May 21 2011
May 23 2011
May 25 2011
May 27 2011
May 29 2011
May 31 2011
Jun 02 2011
Time

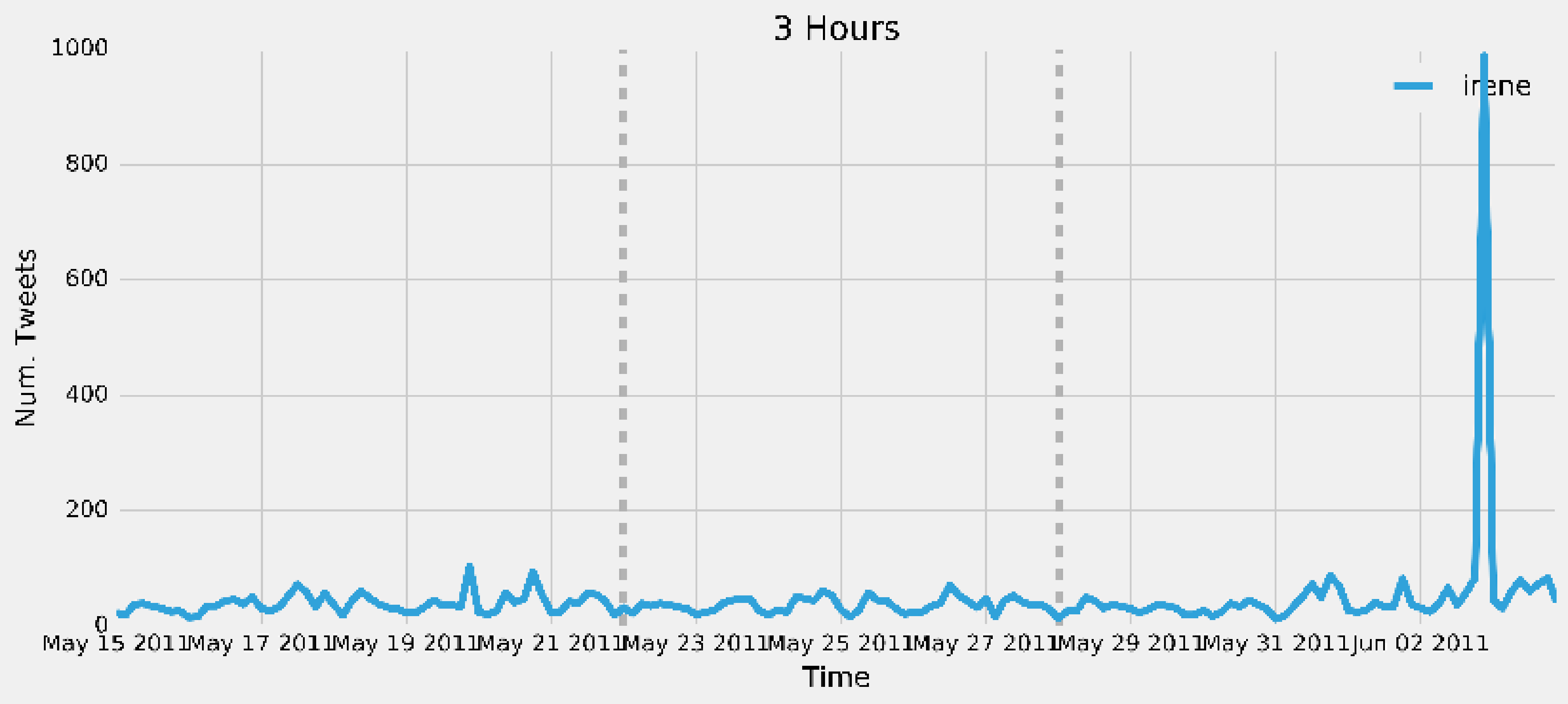
3 Hours
irene
Num. Tweets
Time
0
200
400
600
800
1000
May 15 2011
May 17 2011
May 19 2011
May 21 2011
May 23 2011
May 25 2011
May 27 2011
May 29 2011
May 31 2011
Jun 02 2011

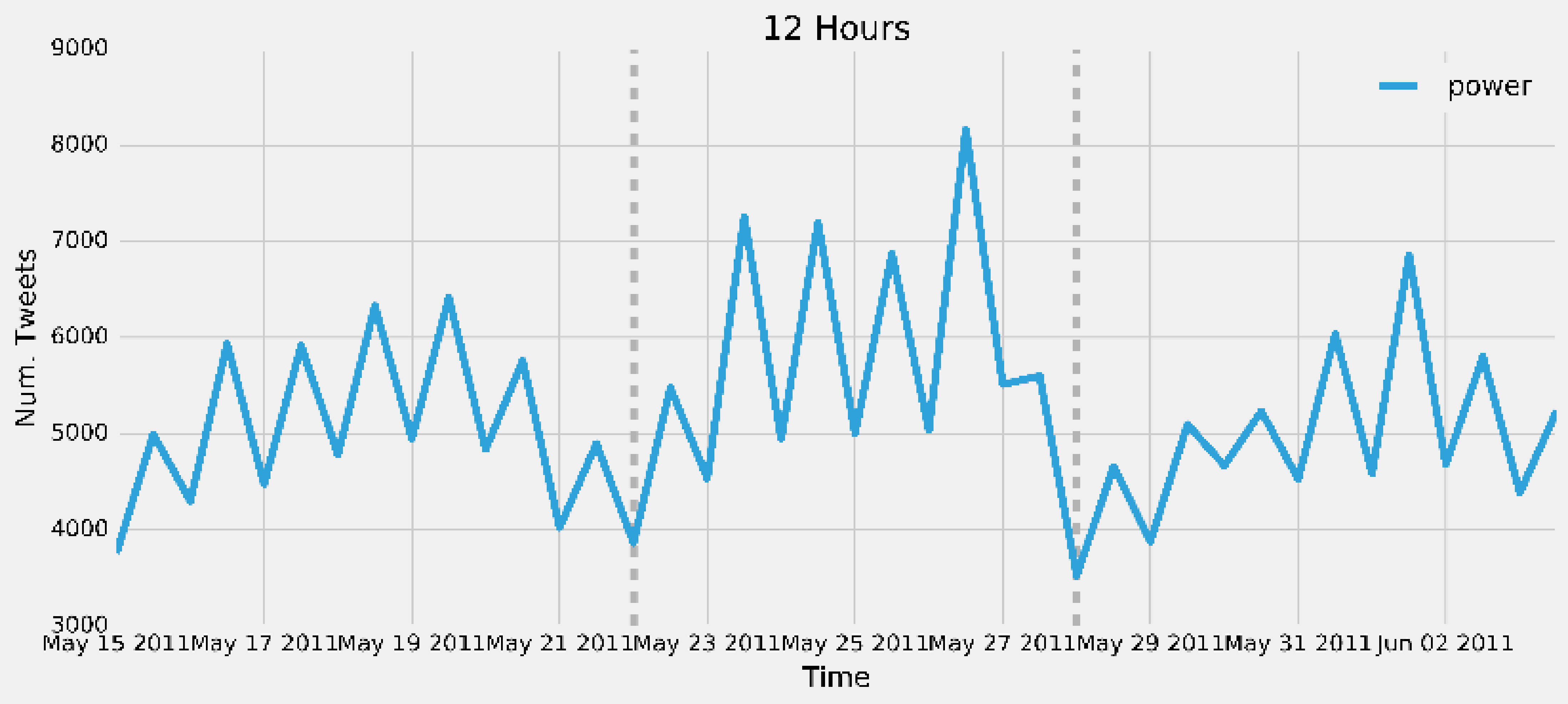
12 Hours
power
Num. Tweets
9000
8000
7000
6000
5000
4000
3000
May 15 2011
May 17 2011
May 19 2011
May 21 2011
May 23 2011
May 25 2011
May 27 2011
May 29 2011
May 31 2011
Jun 02 2011
Time

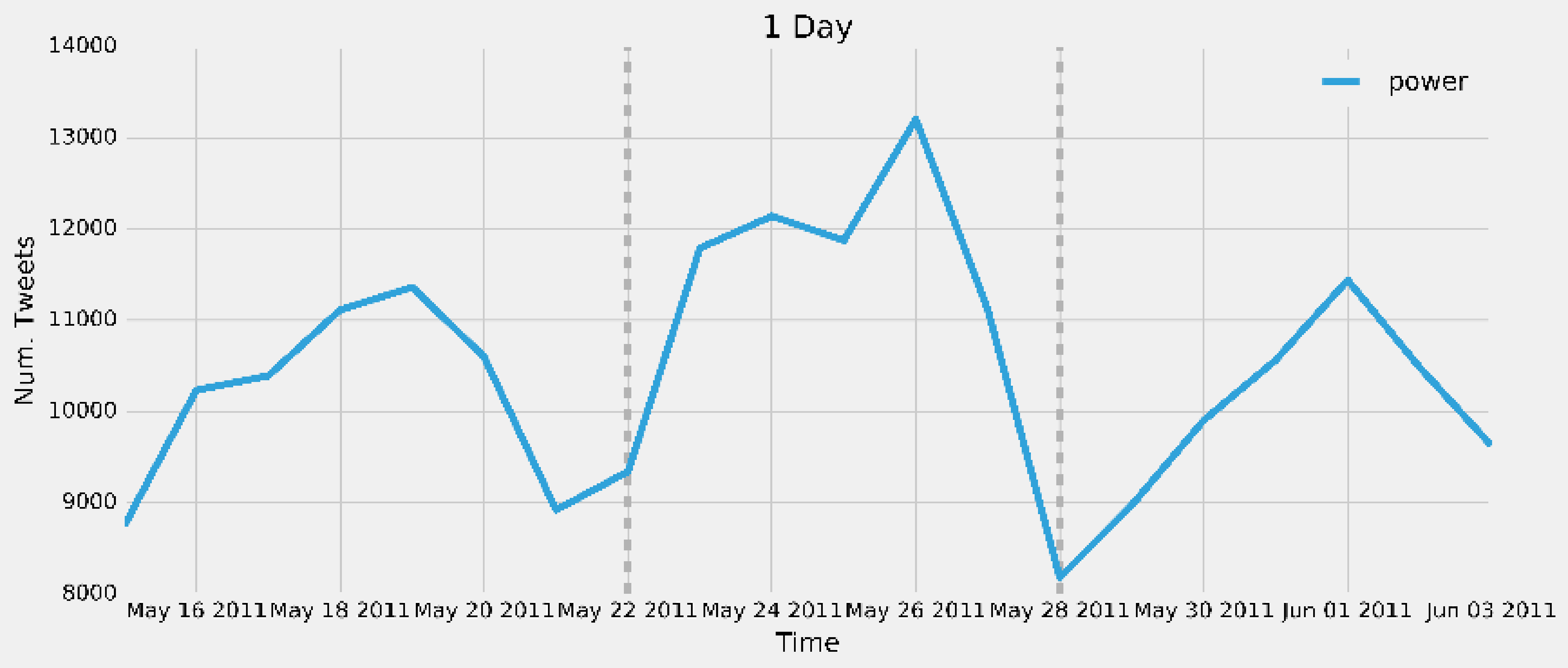

1 Day
power
Num. Tweets
14000
13000
12000
11000
10000
9000
8000
May 16 2011
May 18 2011
May 20 2011
May 22 2011
May 24 2011
May 26 2011
May 28 2011
May 30 2011
Jun 01 2011
Jun 03 2011
Time

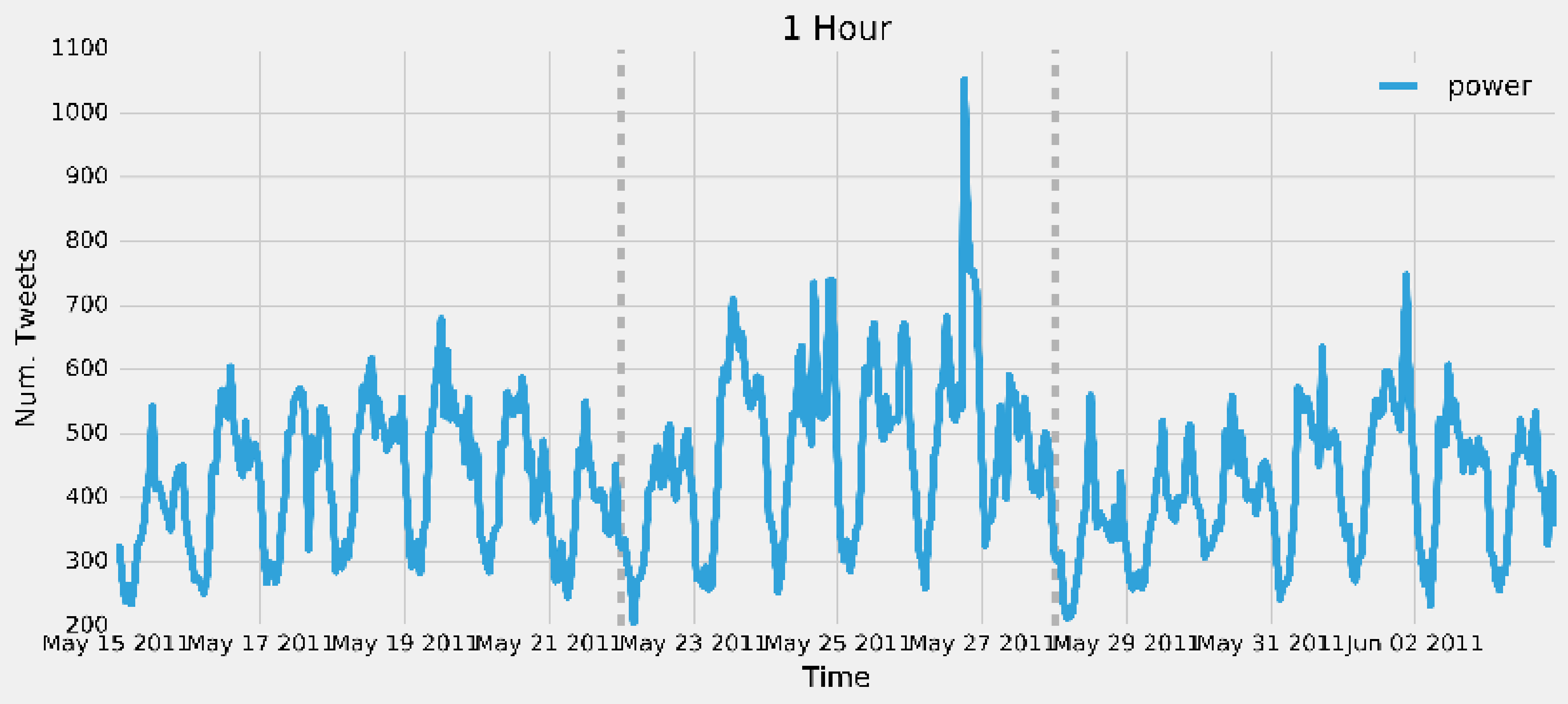

1 Hour
power
Num. Tweets
Time
1100
1000
900
800
700
600
500
400
300
200
May 15 2011
May 17 2011
May 19 2011
May 21 2011
May 23 2011
May 25 2011
May 27 2011
May 29 2011
May 31 2011
Jun 02 2011

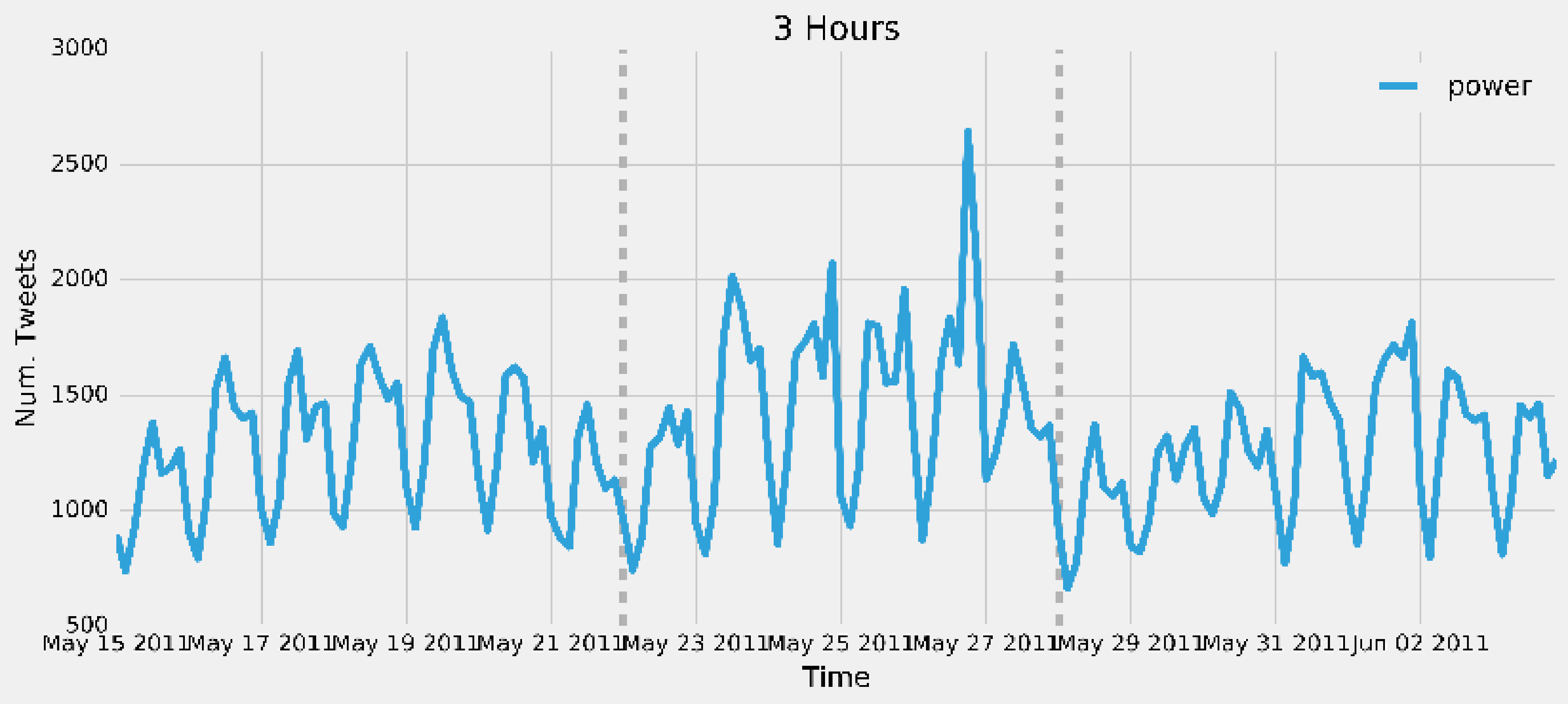
3 Hours
power
Num. Tweets
3000
2500
2000
1500
1000
500
May 15 2011
May 17 2011
May 19 2011
May 21 2011
May 23 2011
May 25 2011
May 27 2011
May 29 2011
May 31 2011
Jun 02 2011
Time

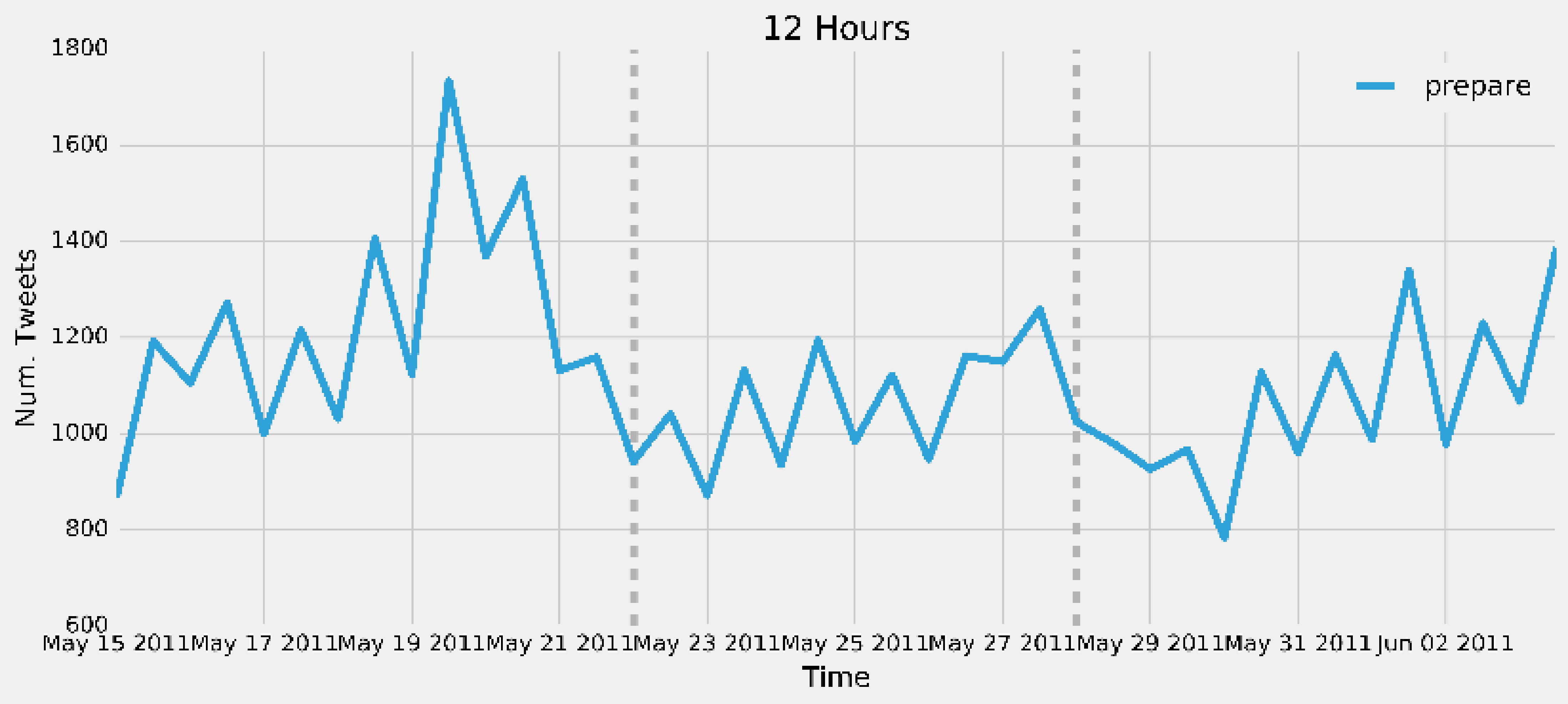
12 Hours
prepare
Num. Tweets
1800
1600
1400
1200
1000
800
600
May 15 2011
May 17 2011
May 19 2011
May 21 2011
May 23 2011
May 25 2011
May 27 2011
May 29 2011
May 31 2011
Jun 02 2011
Time

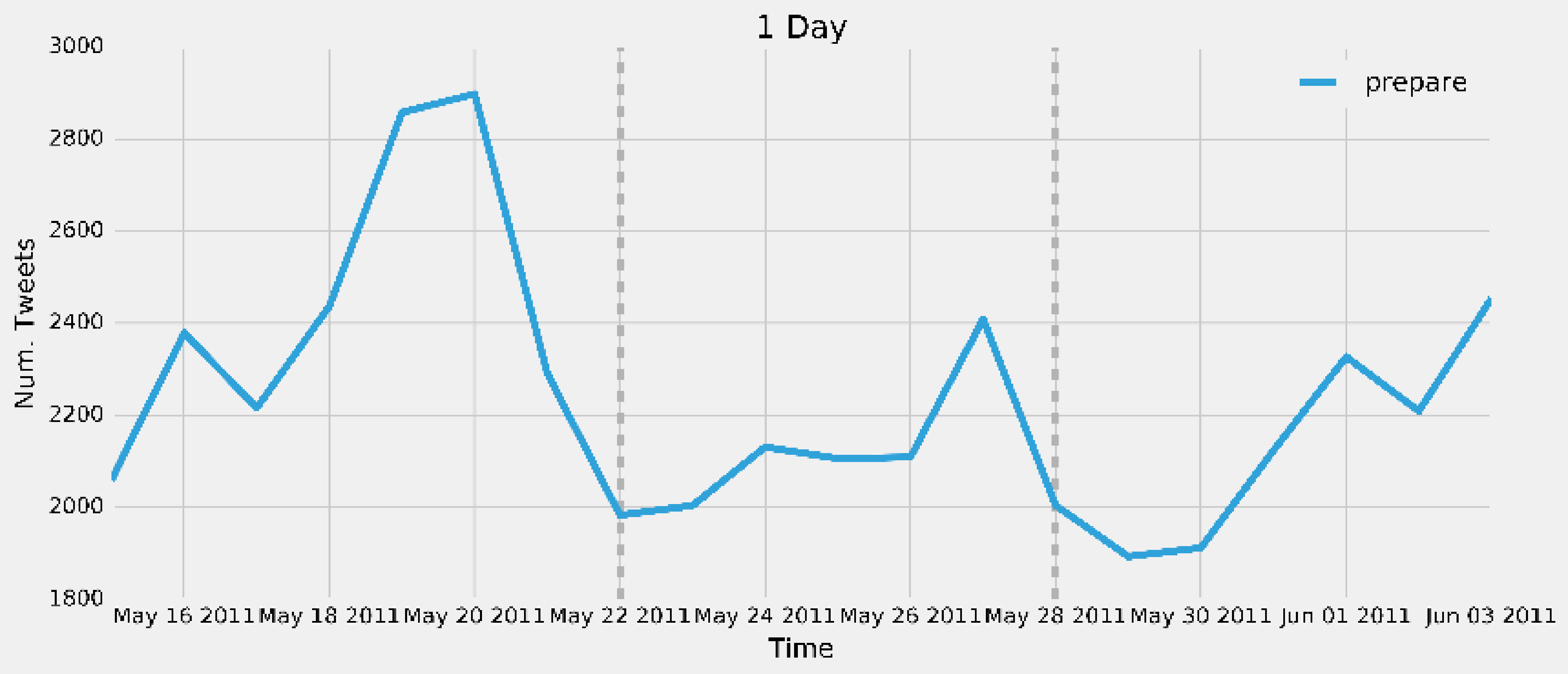
1 Day
prepare
Num. Tweets
3000
2800
2600
2400
2200
2000
1800
May 16 2011
May 18 2011
May 20 2011
May 22 2011
May 24 2011
May 26 2011
May 28 2011
May 30 2011
Jun 01 2011
Jun 03 2011
Time

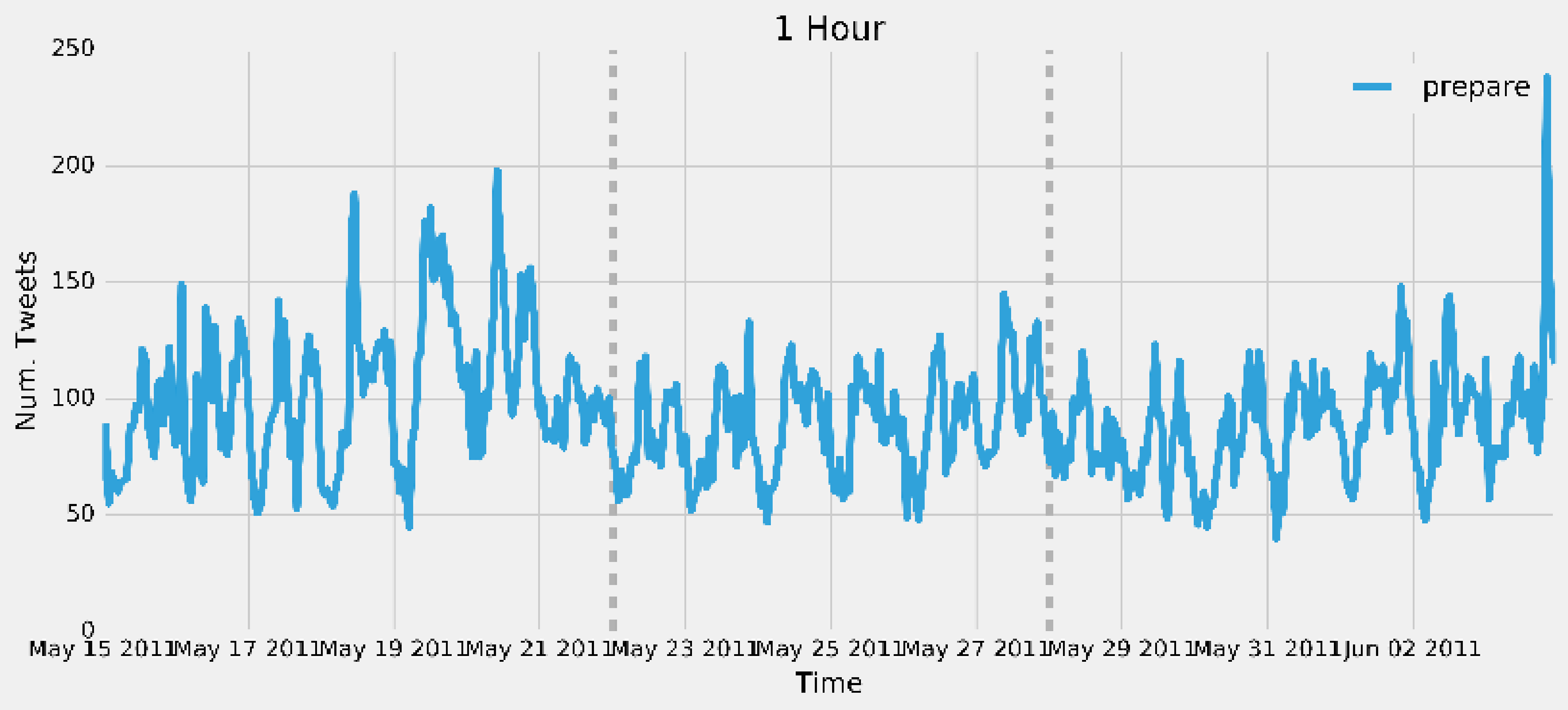

1 Hour
prepare
Num. Tweets
250
200
150
100
50
0
May 15 2011
May 17 2011
May 19 2011
May 21 2011
May 23 2011
May 25 2011
May 27 2011
May 29 2011
May 31 2011
Jun 02 2011
Time

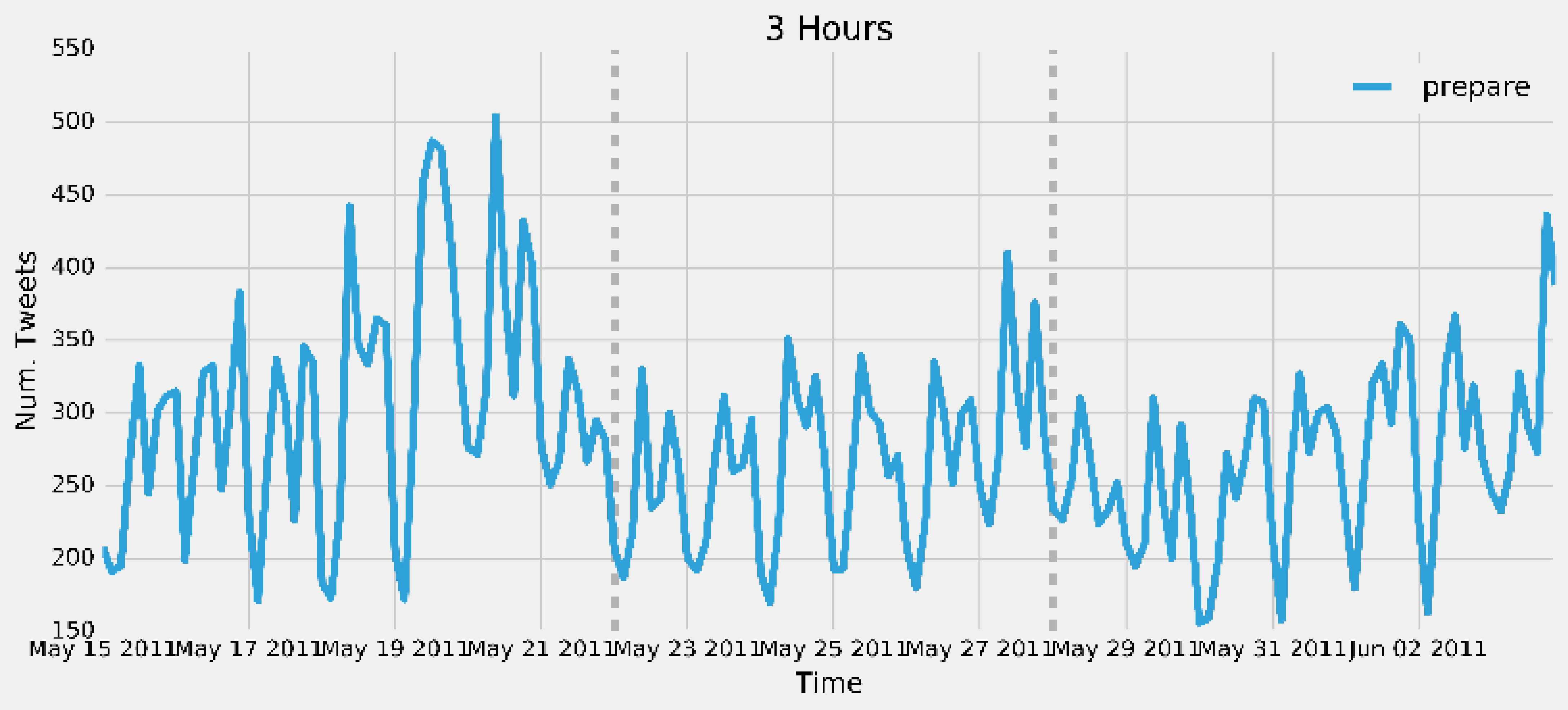

3 Hours
prepare
Num. Tweets
550
500
450
400
350
300
250
200
150
May 15 2011
May 17 2011
May 19 2011
May 21 2011
May 23 2011
May 25 2011
May 27 2011
May 29 2011
May 31 2011
Jun 02 2011
Time

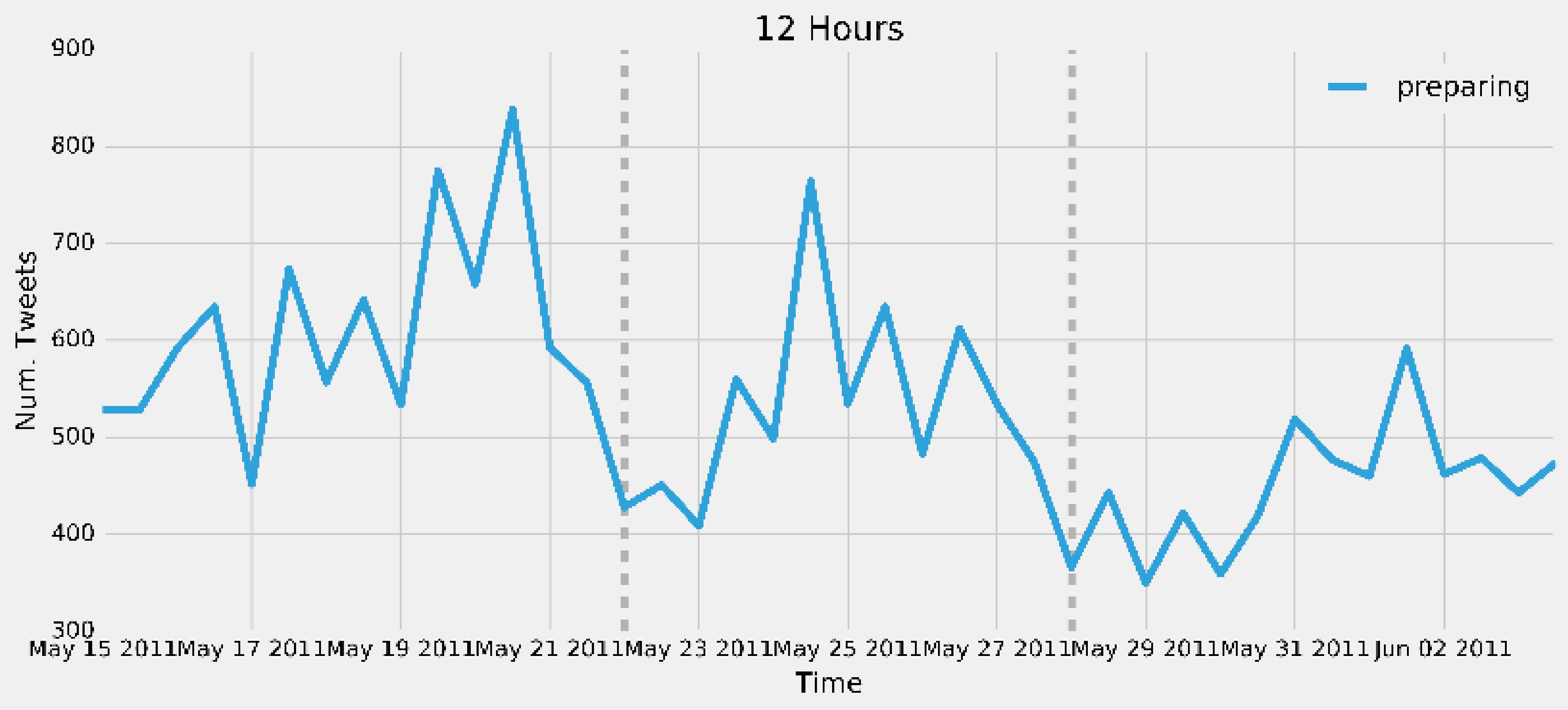

12 Hours
preparing
Num. Tweets
900
800
700
600
500
400
300
May 15 2011
May 17 2011
May 19 2011
May 21 2011
May 23 2011
May 25 2011
May 27 2011
May 29 2011
May 31 2011
Jun 02 2011
Time

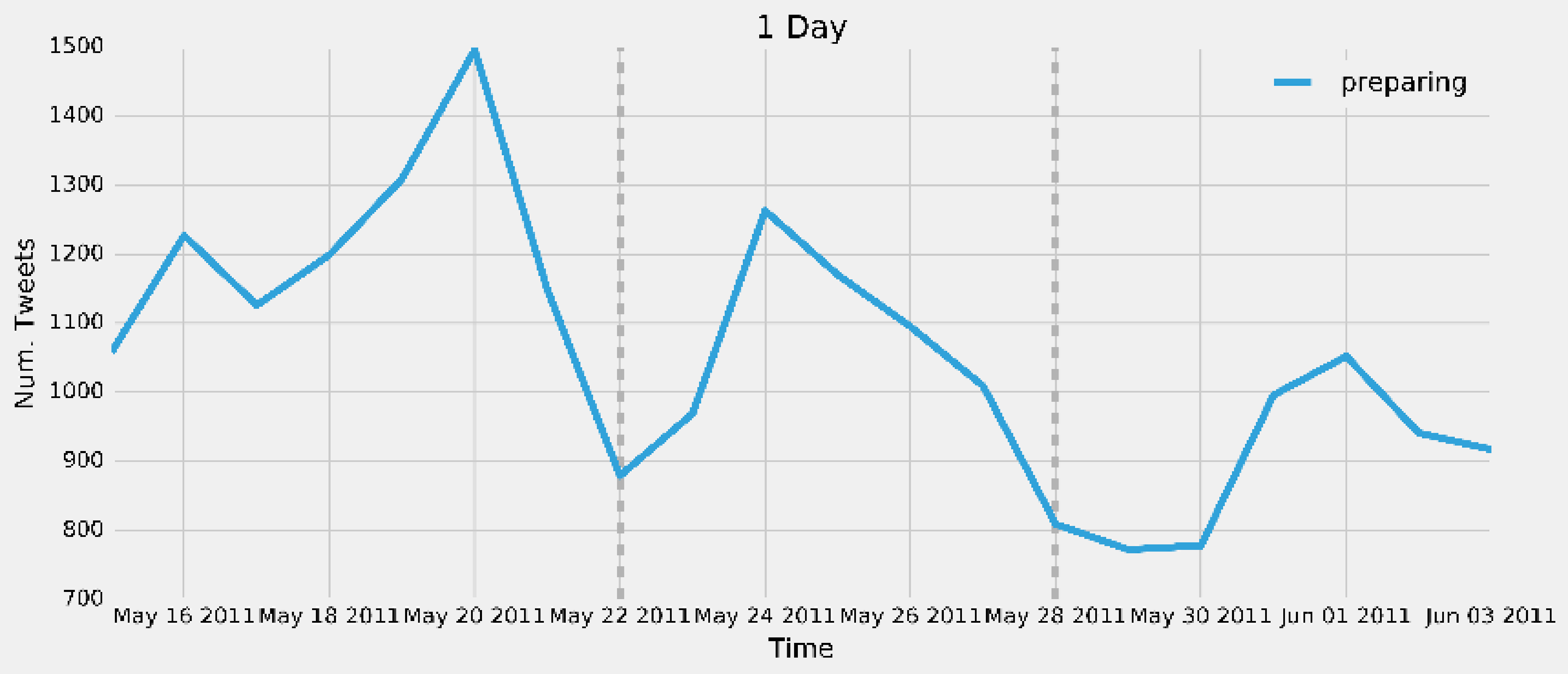

1 Day
preparing
Num. Tweets
1500
1400
1300
1200
1100
1000
900
800
700
May 16 2011
May 18 2011
May 20 2011
May 22 2011
May 24 2011
May 26 2011
May 28 2011
May 30 2011
Jun 01 2011
Jun 03 2011
Time

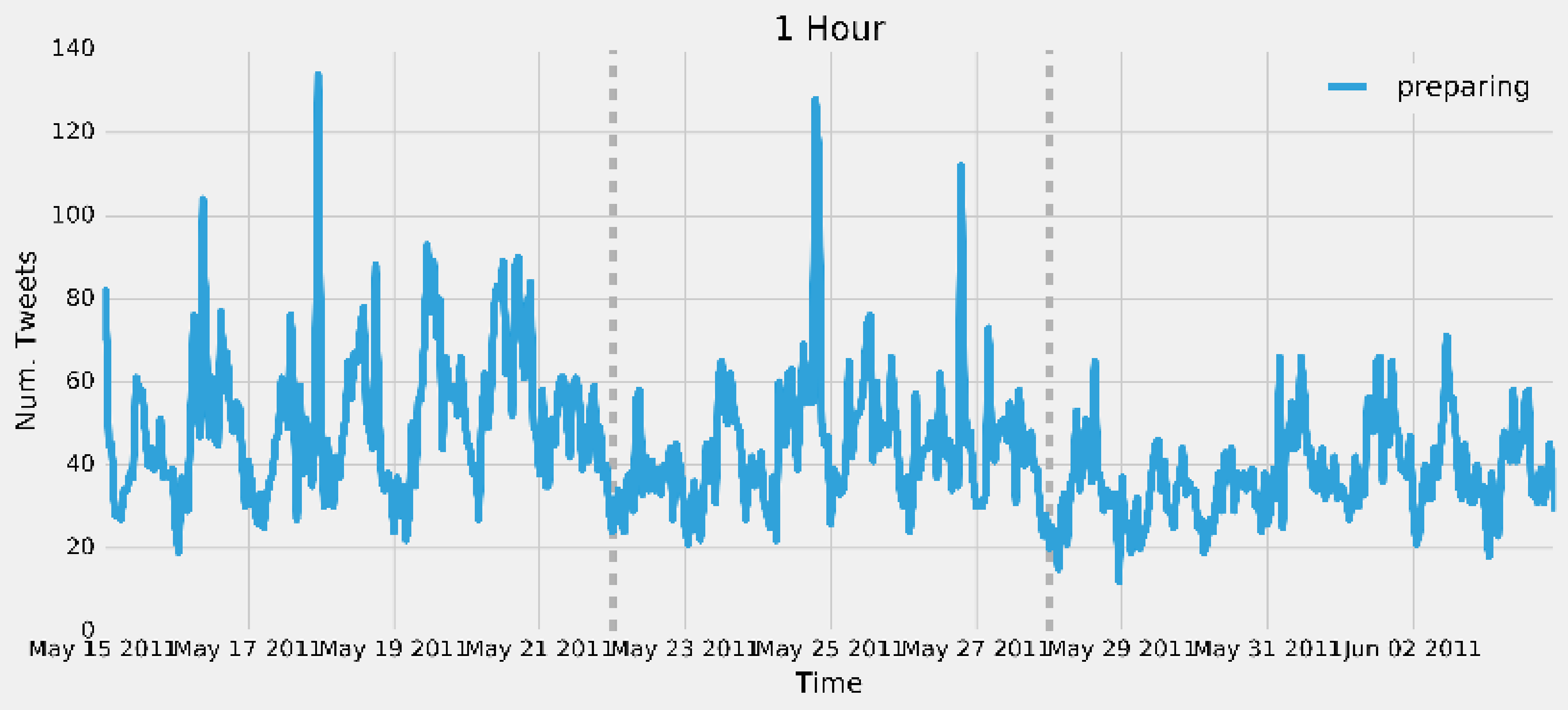

1 Hour
preparing
Num. Tweets
140
120
100
80
60
40
20
0
May 15 2011
May 17 2011
May 19 2011
May 21 2011
May 23 2011
May 25 2011
May 27 2011
May 29 2011
May 31 2011
Jun 02 2011
Time

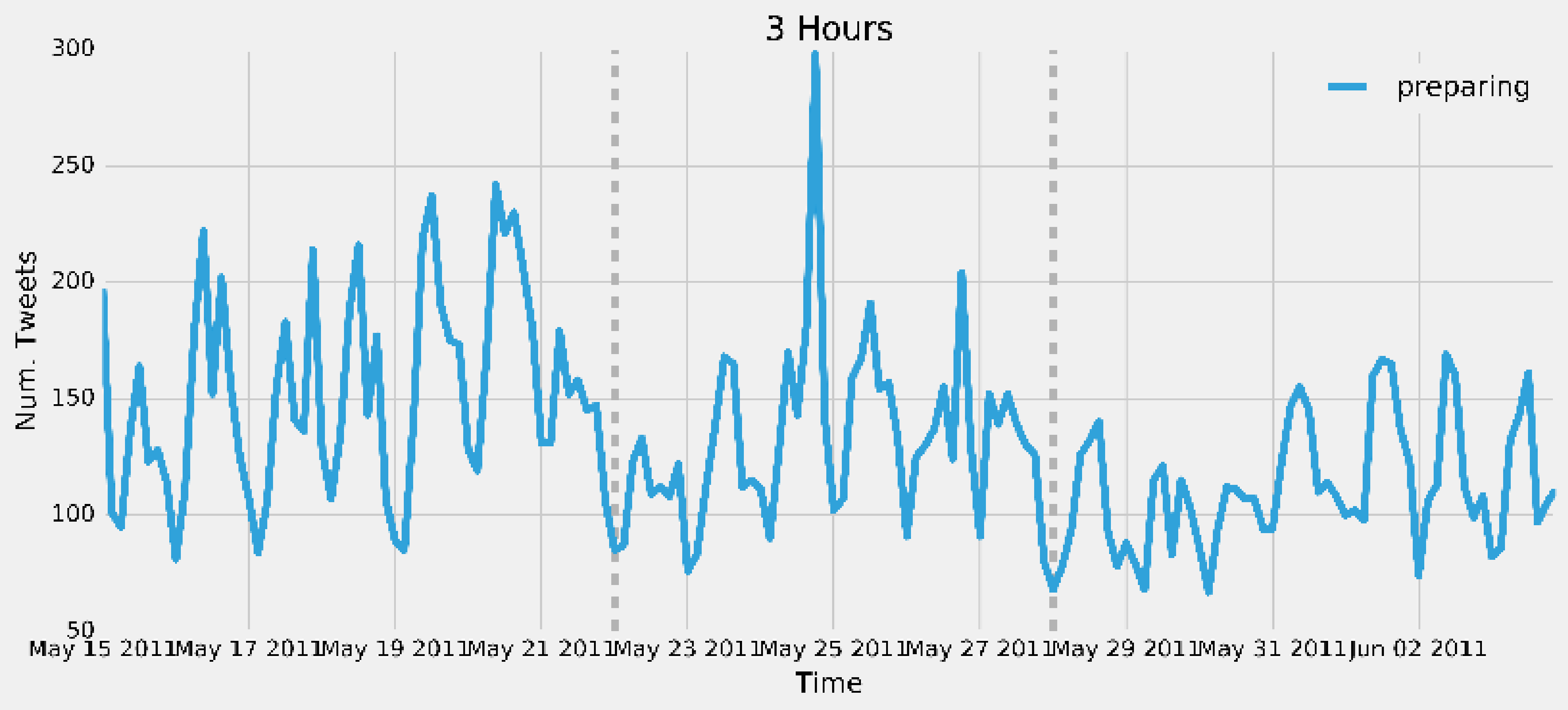
3 Hours
preparing
Num. Tweets
300
250
200
150
100
50
May 15 2011
May 17 2011
May 19 2011
May 21 2011
May 23 2011
May 25 2011
May 27 2011
May 29 2011
May 31 2011
Jun 02 2011
Time

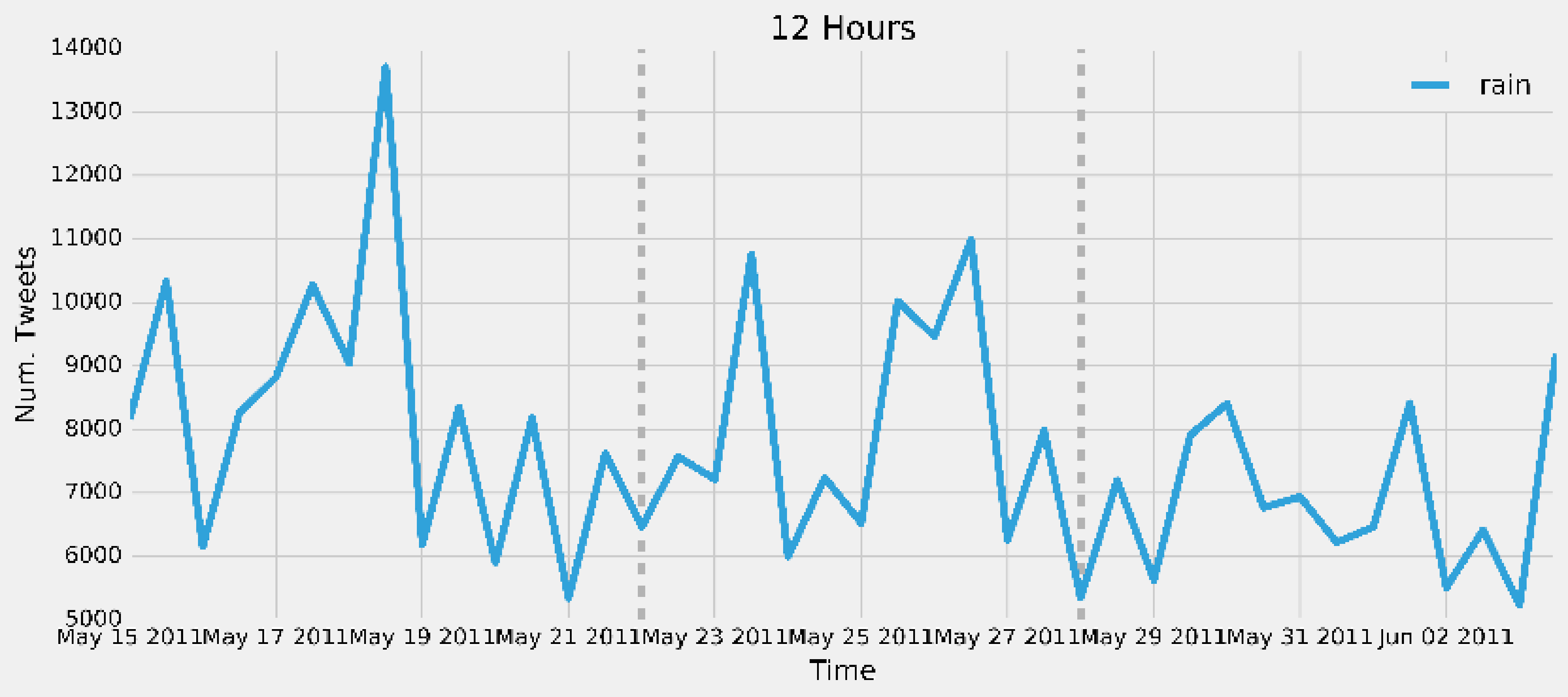
12 Hours
rain
Num. Tweets
Time
14000
13000
12000
11000
10000
9000
8000
7000
6000
5000
May 15 2011
May 17 2011
May 19 2011
May 21 2011
May 23 2011
May 25 2011
May 27 2011
May 29 2011
May 31 2011
Jun 02 2011

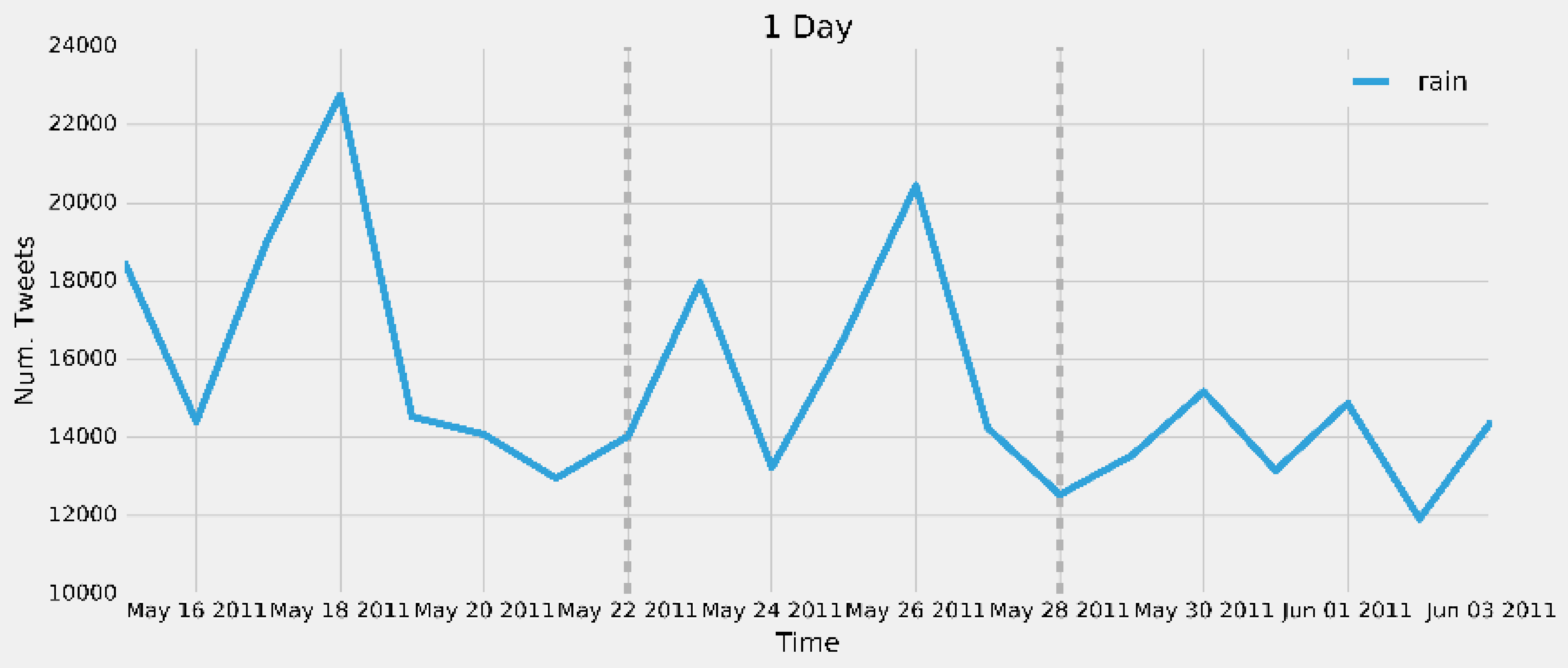
1 Day
rain
Num. Tweets
24000
22000
20000
18000
16000
14000
12000
10000
May 16 2011
May 18 2011
May 20 2011
May 22 2011
May 24 2011
May 26 2011
May 28 2011
May 30 2011
Jun 01 2011
Jun 03 2011
Time

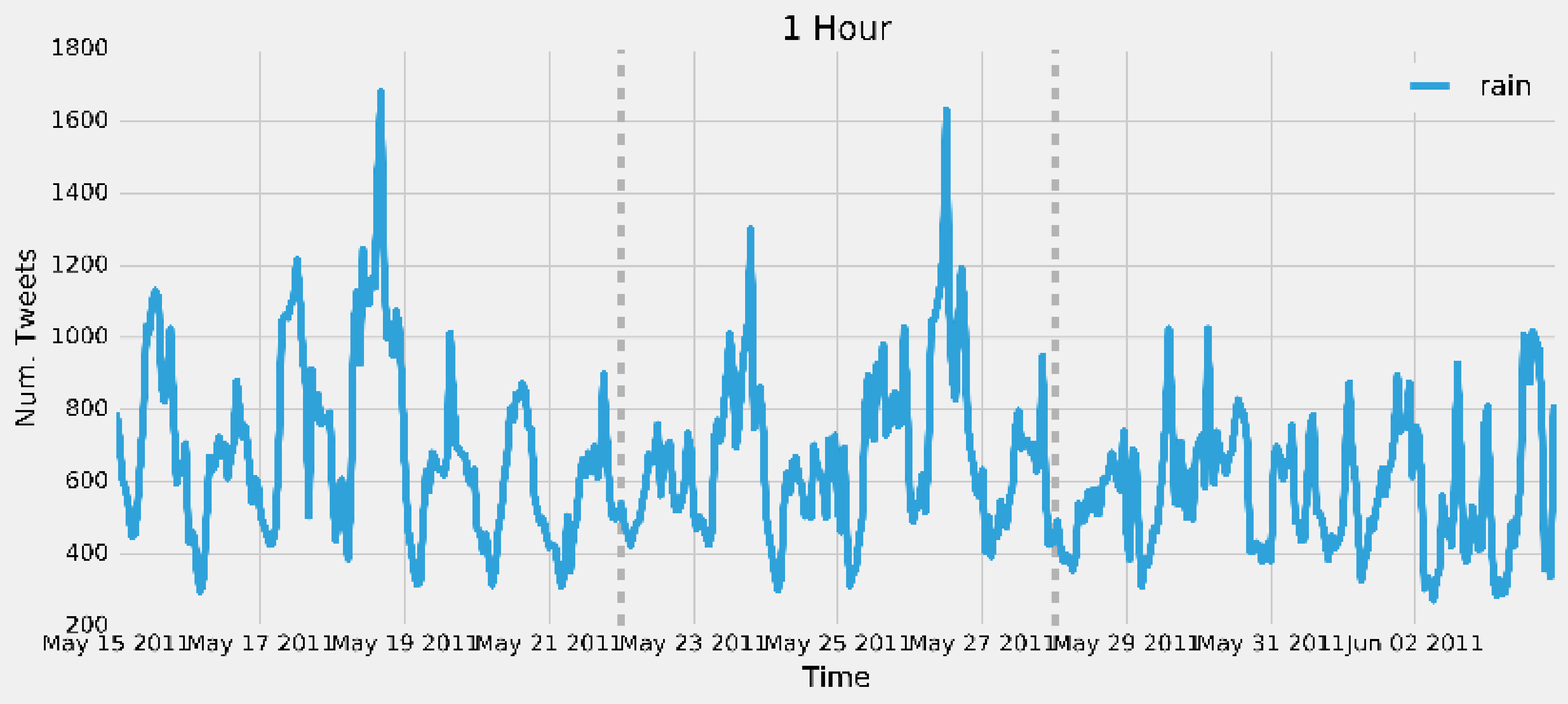

1 Hour
rain
Num. Tweets
1800
1600
1400
1200
1000
800
600
400
200
May 15 2011
May 17 2011
May 19 2011
May 21 2011
May 23 2011
May 25 2011
May 27 2011
May 29 2011
May 31 2011
Jun 02 2011
Time

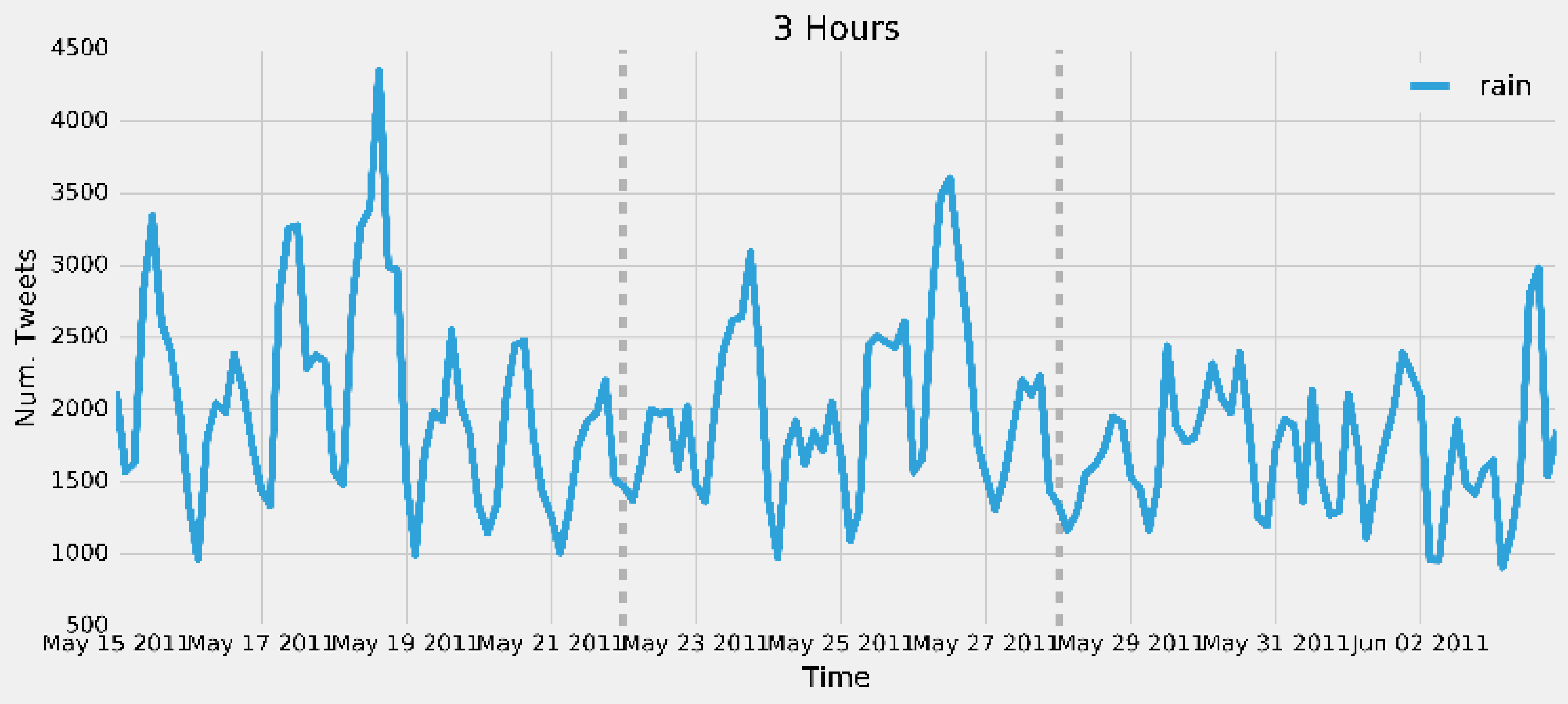

3 Hours
4500
4000
3500
3000
2500
2000
1500
1000
500
Num. Tweets
rain
May 15 2011
May 17 2011
May 19 2011
May 21 2011
May 23 2011
May 25 2011
May 27 2011
May 29 2011
May 31 2011
Jun 02 2011
Time

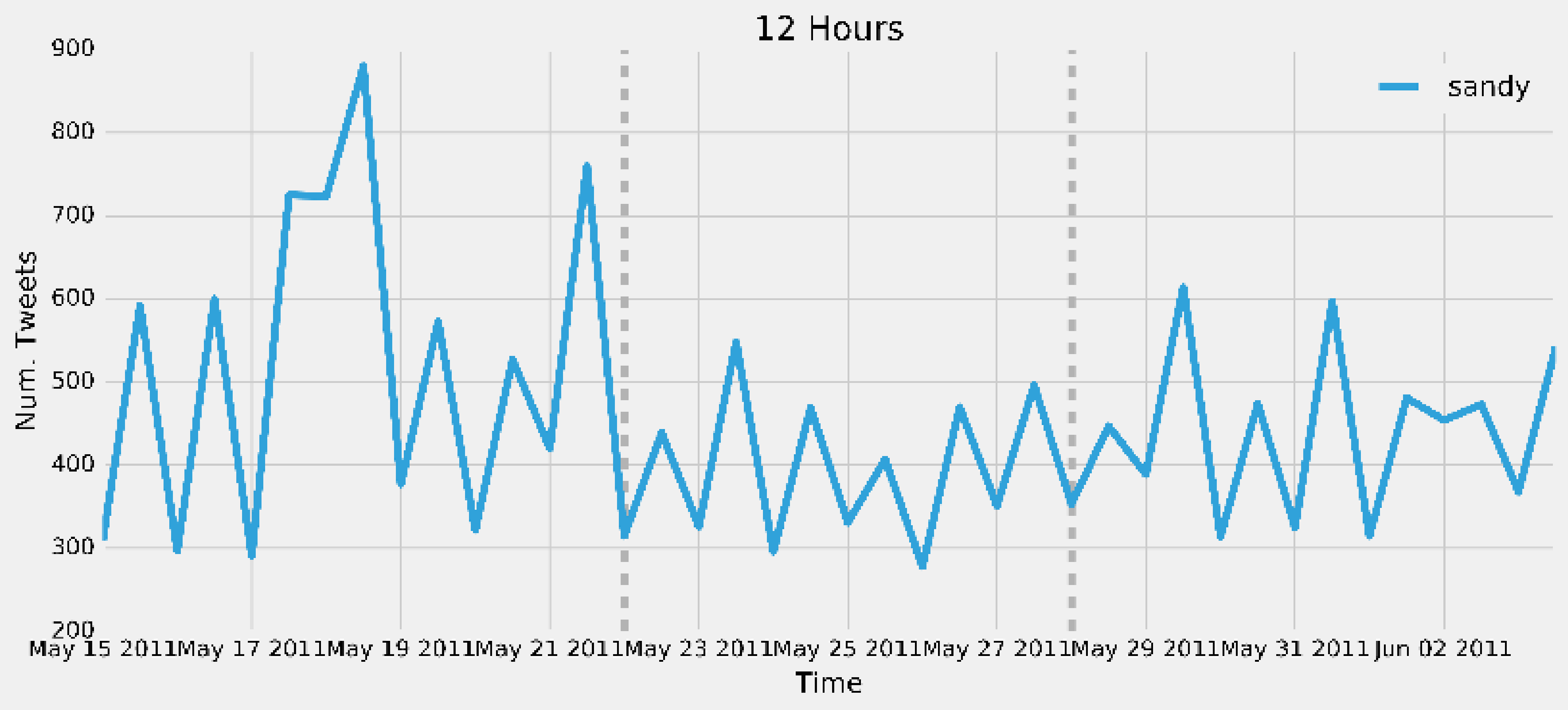
12 Hours
sandy
Num. Tweets
900
800
700
600
500
400
300
200
May 15 2011
May 17 2011
May 19 2011
May 21 2011
May 23 2011
May 25 2011
May 27 2011
May 29 2011
May 31 2011
Jun 02 2011
Time

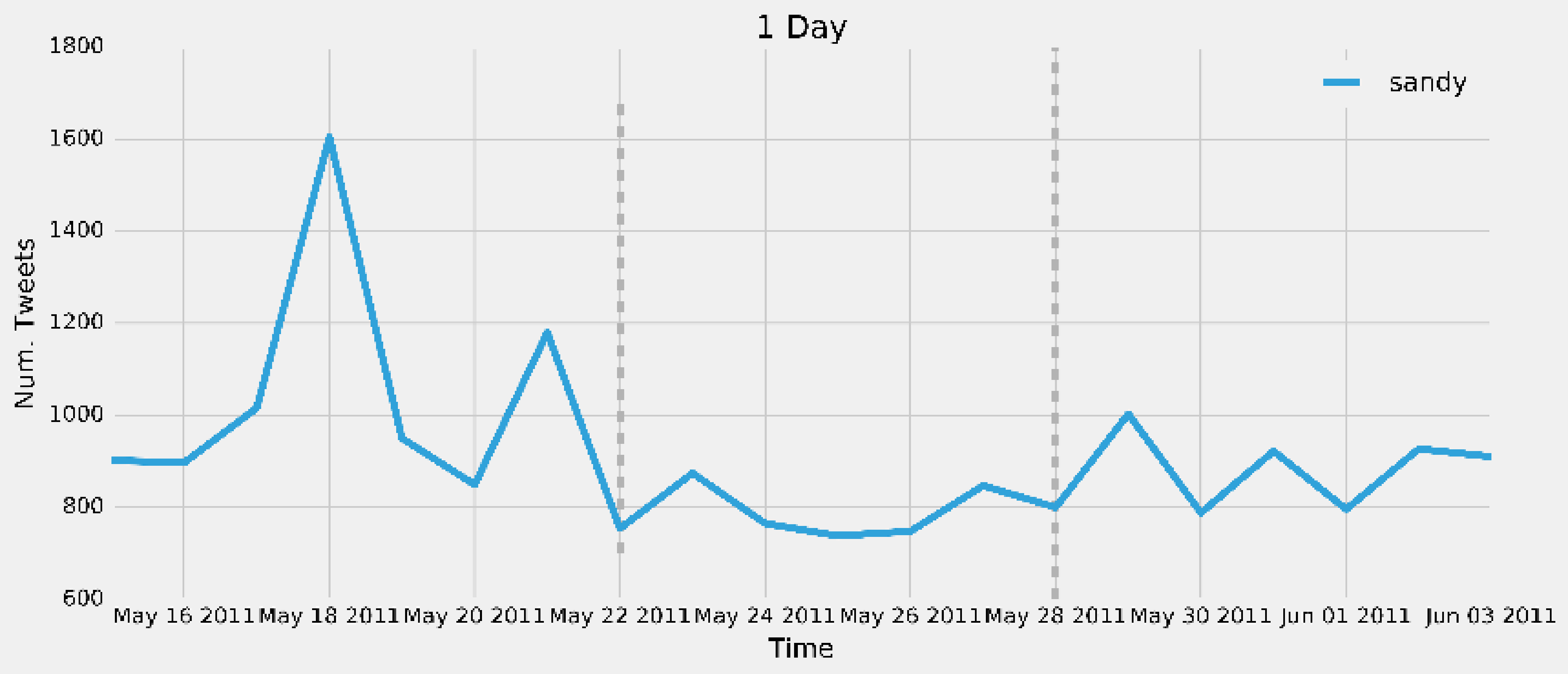
1 Day
sandy
Num. Tweets
Time
1800
1600
1400
1200
1000
800
600
May 16 2011
May 18 2011
May 20 2011
May 22 2011
May 24 2011
May 26 2011
May 28 2011
May 30 2011
Jun 01 2011
Jun 03 2011

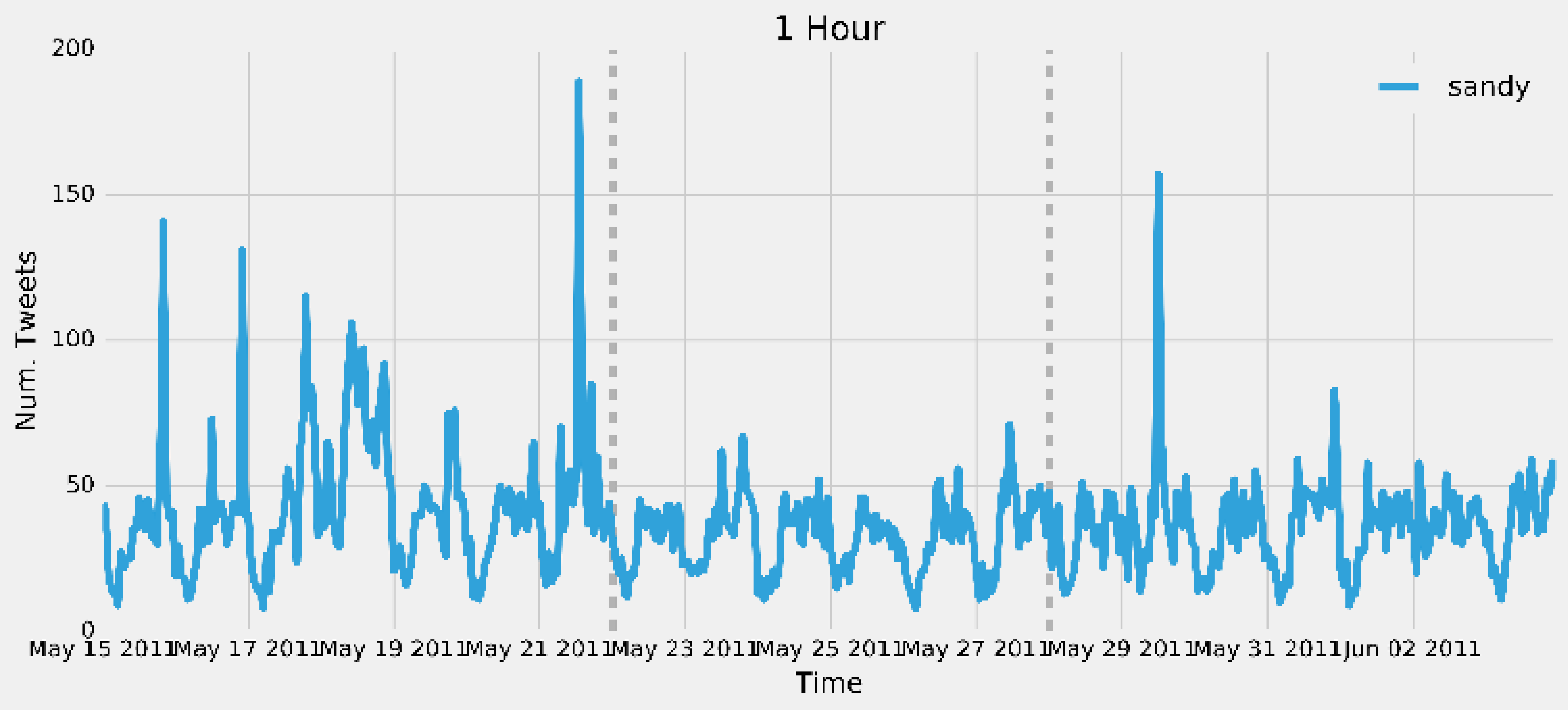

1 Hour
sandy
Num. Tweets
Time
0
50
100
150
200
May 15 2011
May 17 2011
May 19 2011
May 21 2011
May 23 2011
May 25 2011
May 27 2011
May 29 2011
May 31 2011
Jun 02 2011

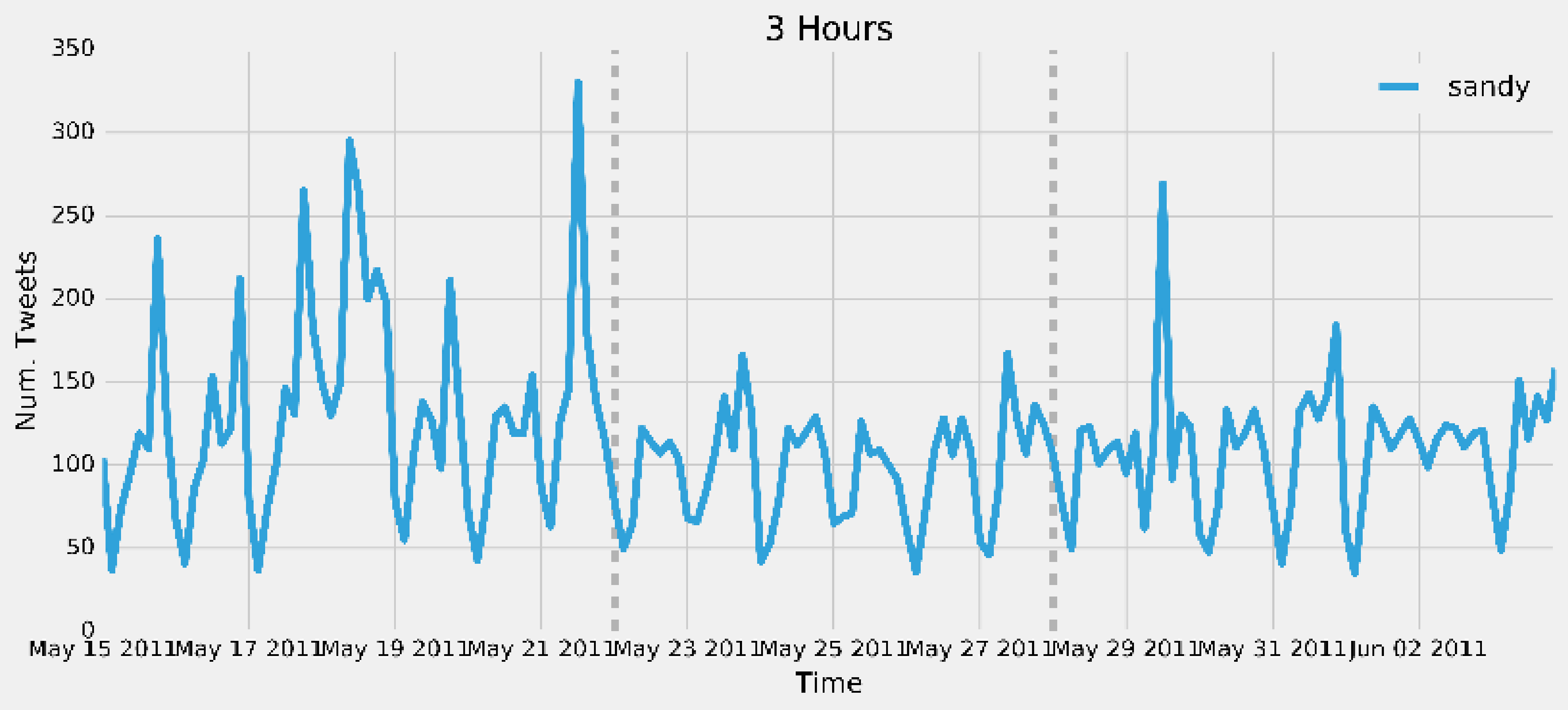

3 Hours
Num. Tweets
350
300
250
200
150
100
50
0
May 15 2011
May 17 2011
May 19 2011
May 21 2011
May 23 2011
May 25 2011
May 27 2011
May 29 2011
May 31 2011
Jun 02 2011
Time
sandy

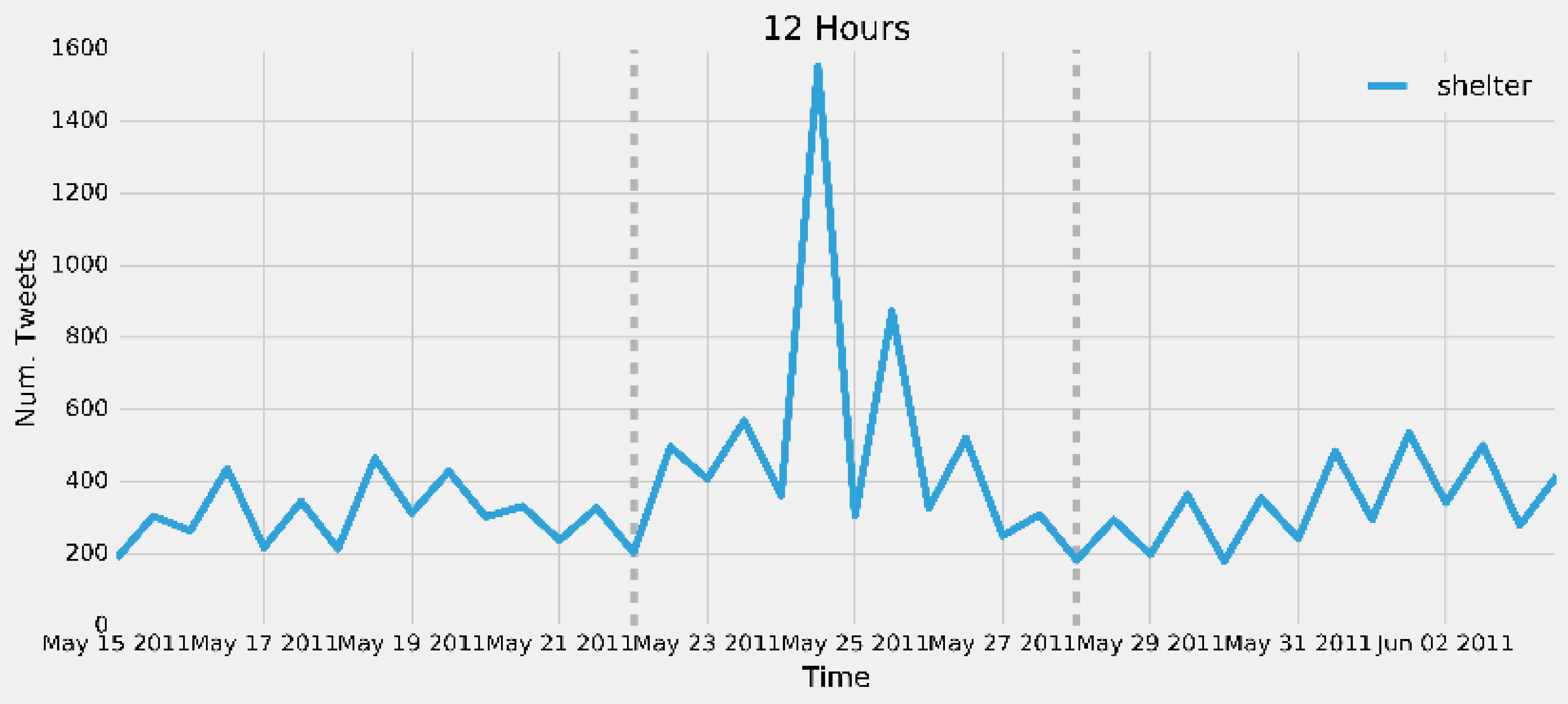
12 Hours
shelter
Num. Tweets
0
200
400
600
800
1000
1200
1400
1600
May 15 2011
May 17 2011
May 19 2011
May 21 2011
May 23 2011
May 25 2011
May 27 2011
May 29 2011
May 31 2011
Jun 02 2011
Time

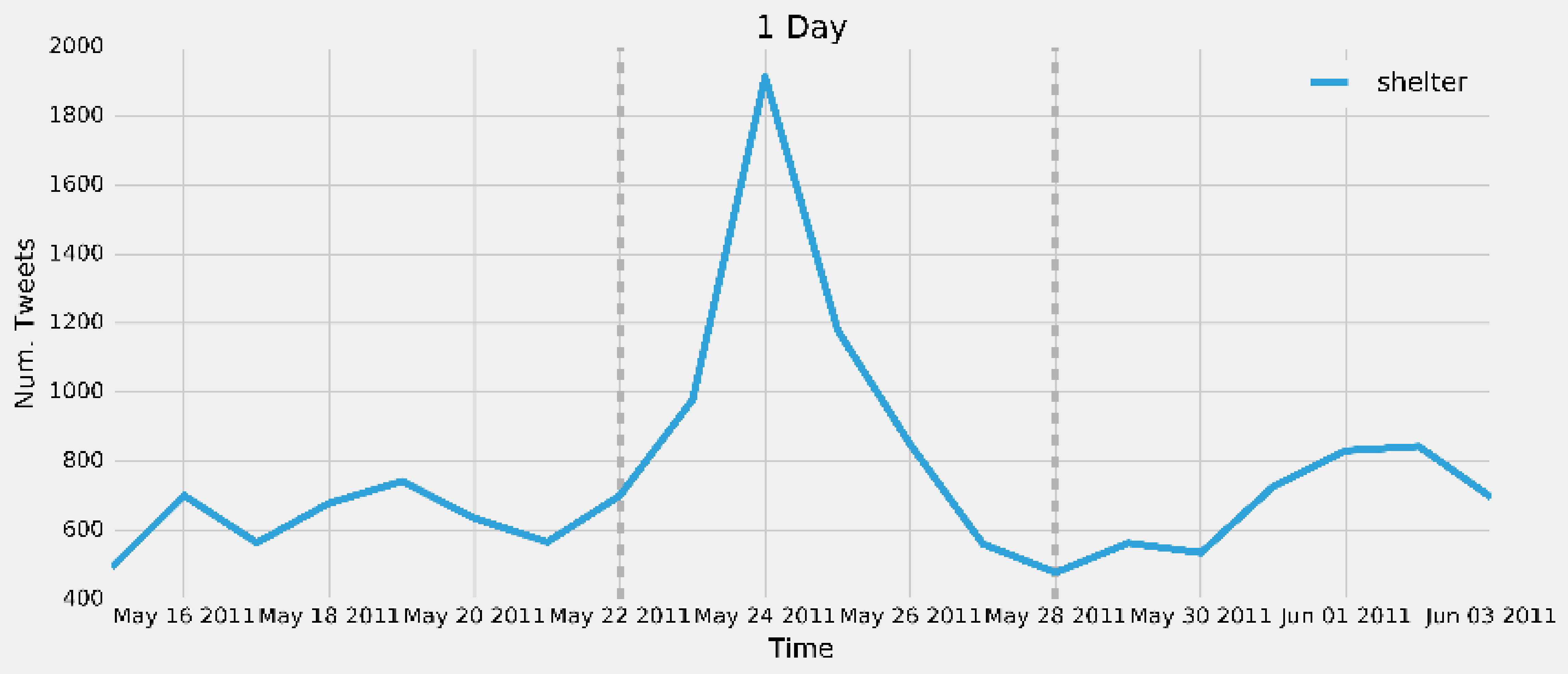

1 Day
shelter
Num. Tweets
2000
1800
1600
1400
1200
1000
800
600
400
May 16 2011
May 18 2011
May 20 2011
May 22 2011
May 24 2011
May 26 2011
May 28 2011
May 30 2011
Jun 01 2011
Jun 03 2011
Time

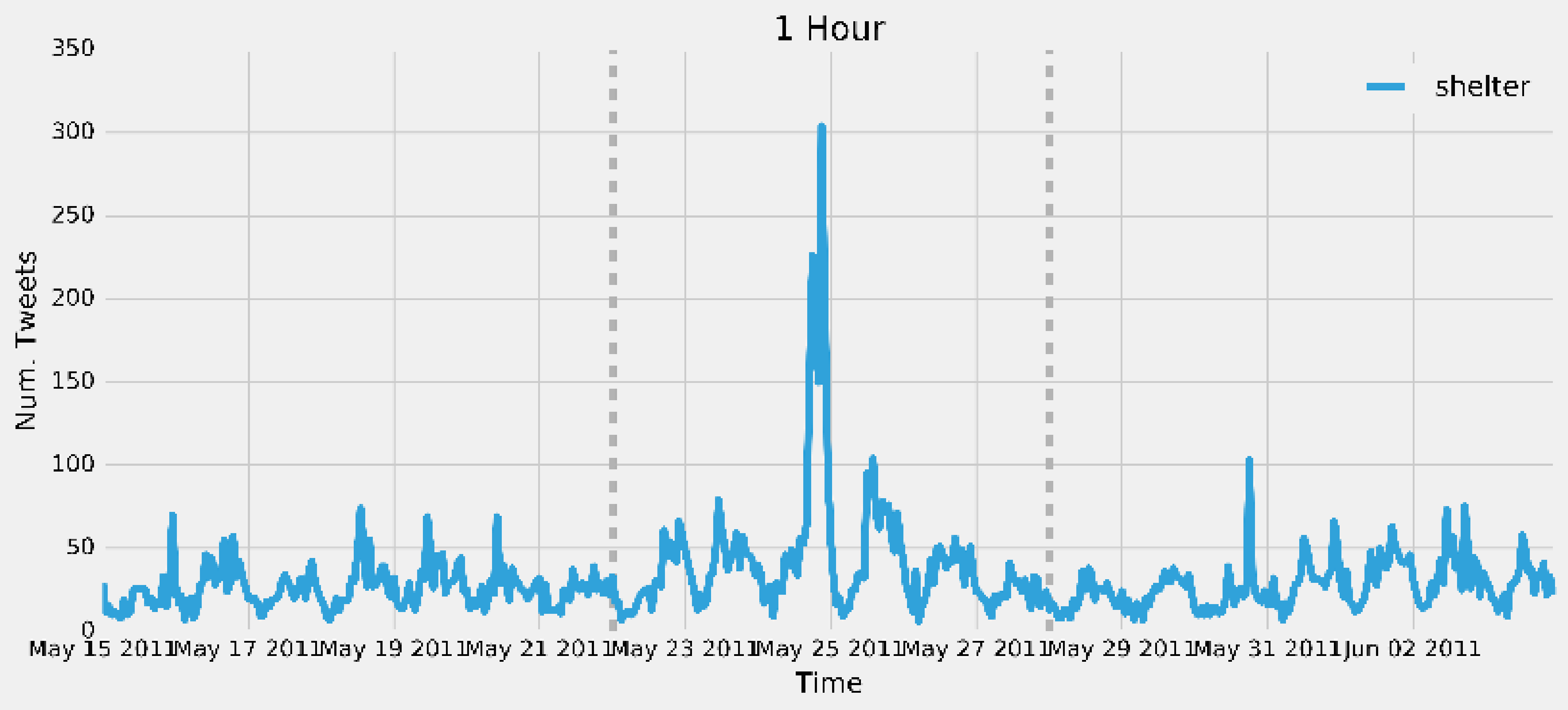
1 Hour
shelter
350
300
250
200
150
100
50
0
Num. Tweets
May 15 2011
May 17 2011
May 19 2011
May 21 2011
May 23 2011
May 25 2011
May 27 2011
May 29 2011
May 31 2011
Jun 02 2011
Time

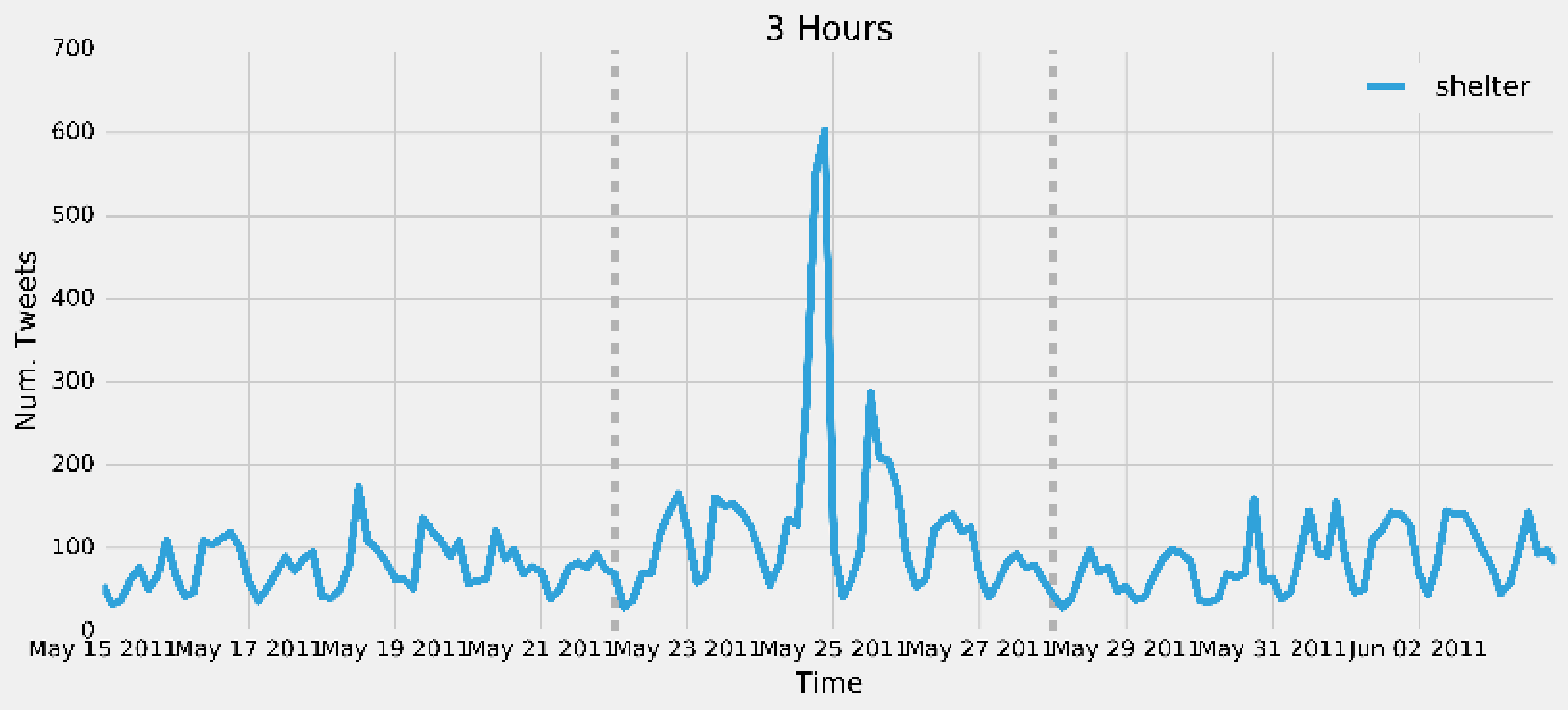

3 Hours
shelter
Num. Tweets
0
100
200
300
400
500
600
700
May 15 2011
May 17 2011
May 19 2011
May 21 2011
May 23 2011
May 25 2011
May 27 2011
May 29 2011
May 31 2011
Jun 02 2011
Time

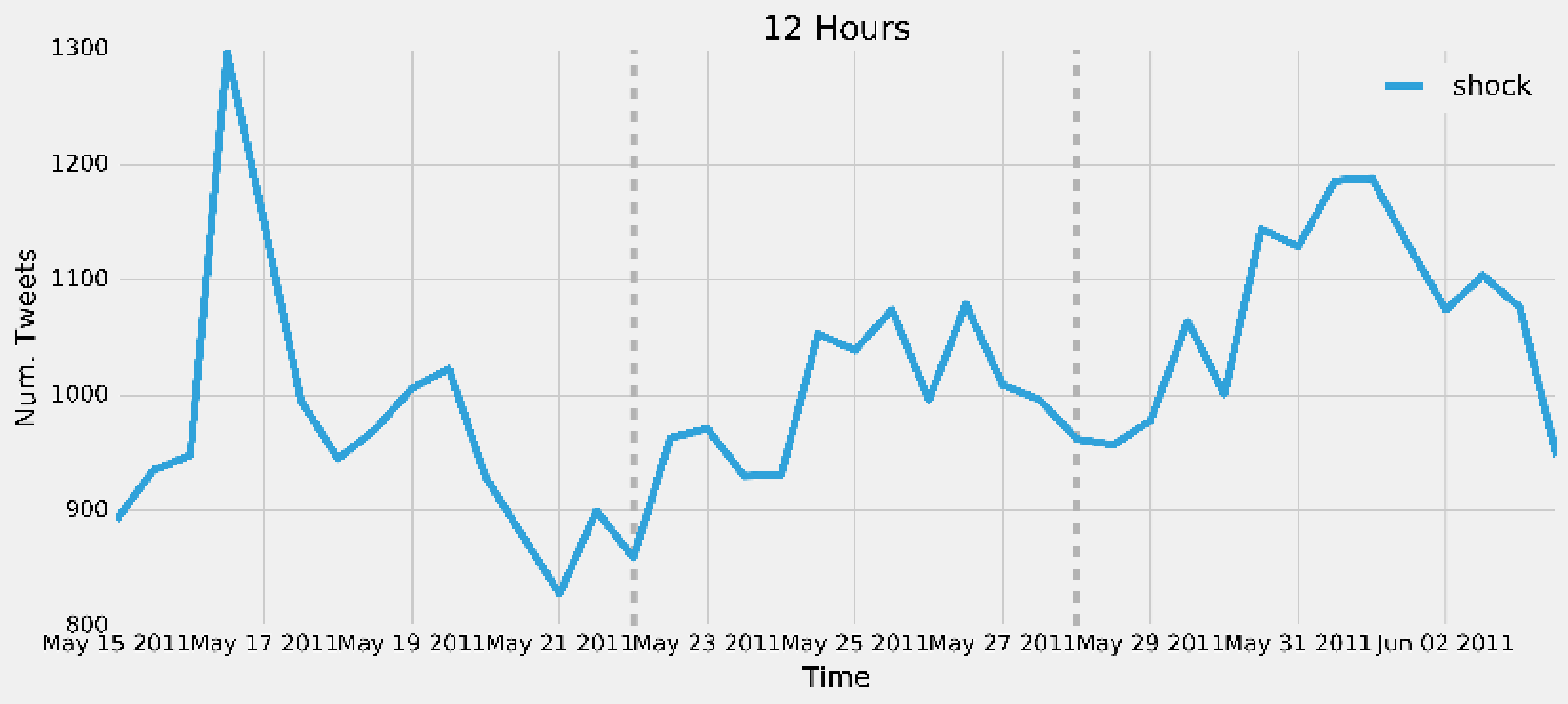
12 Hours
shock
Num. Tweets
Time
1300
1200
1100
1000
900
800
May 15 2011
May 17 2011
May 19 2011
May 21 2011
May 23 2011
May 25 2011
May 27 2011
May 29 2011
May 31 2011
Jun 02 2011

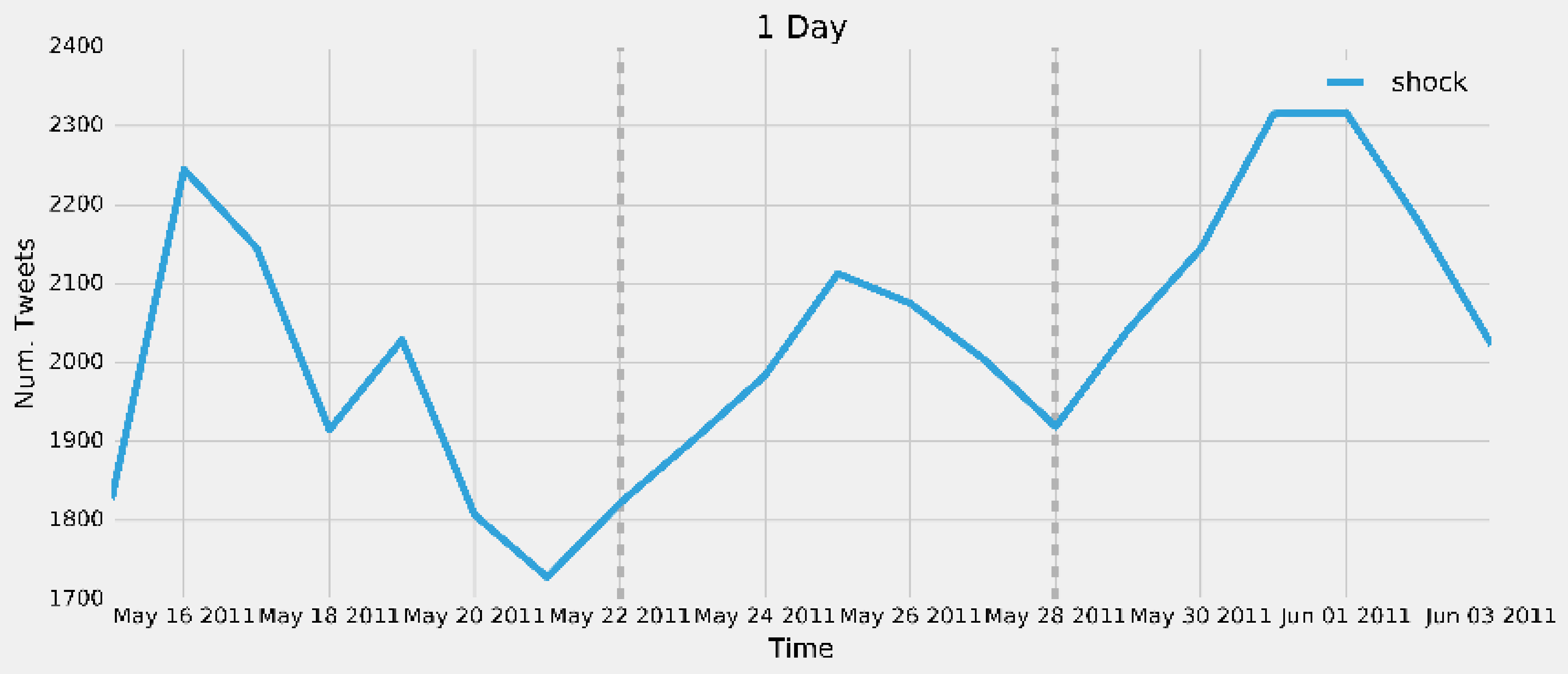
1 Day
shock
Num. Tweets
Time
2400
2300
2200
2100
2000
1900
1800
1700
May 16 2011
May 18 2011
May 20 2011
May 22 2011
May 24 2011
May 26 2011
May 28 2011
May 30 2011
Jun 01 2011
Jun 03 2011

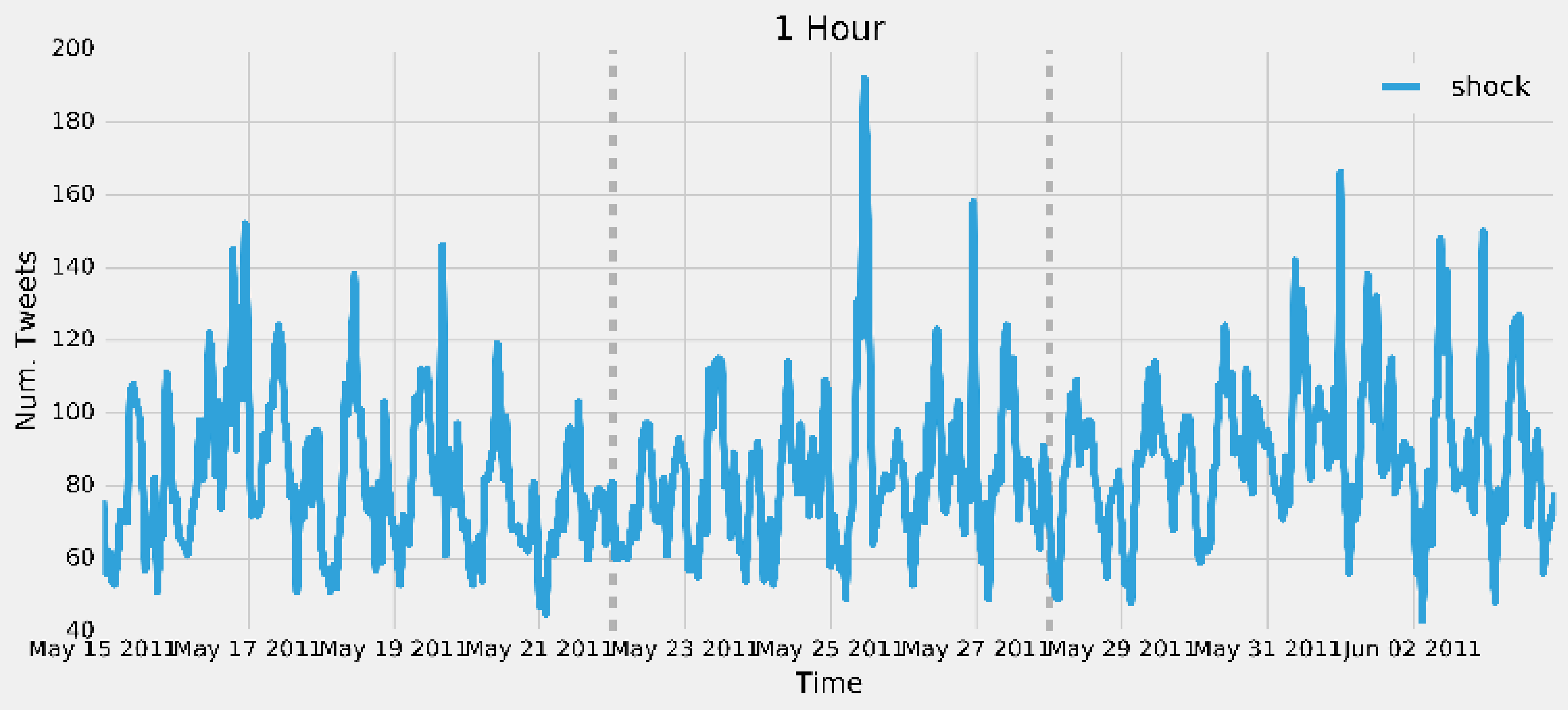

1 Hour
shock
Num. Tweets
200
180
160
140
120
100
80
60
40
May 15 2011
May 17 2011
May 19 2011
May 21 2011
May 23 2011
May 25 2011
May 27 2011
May 29 2011
May 31 2011
Jun 02 2011
Time

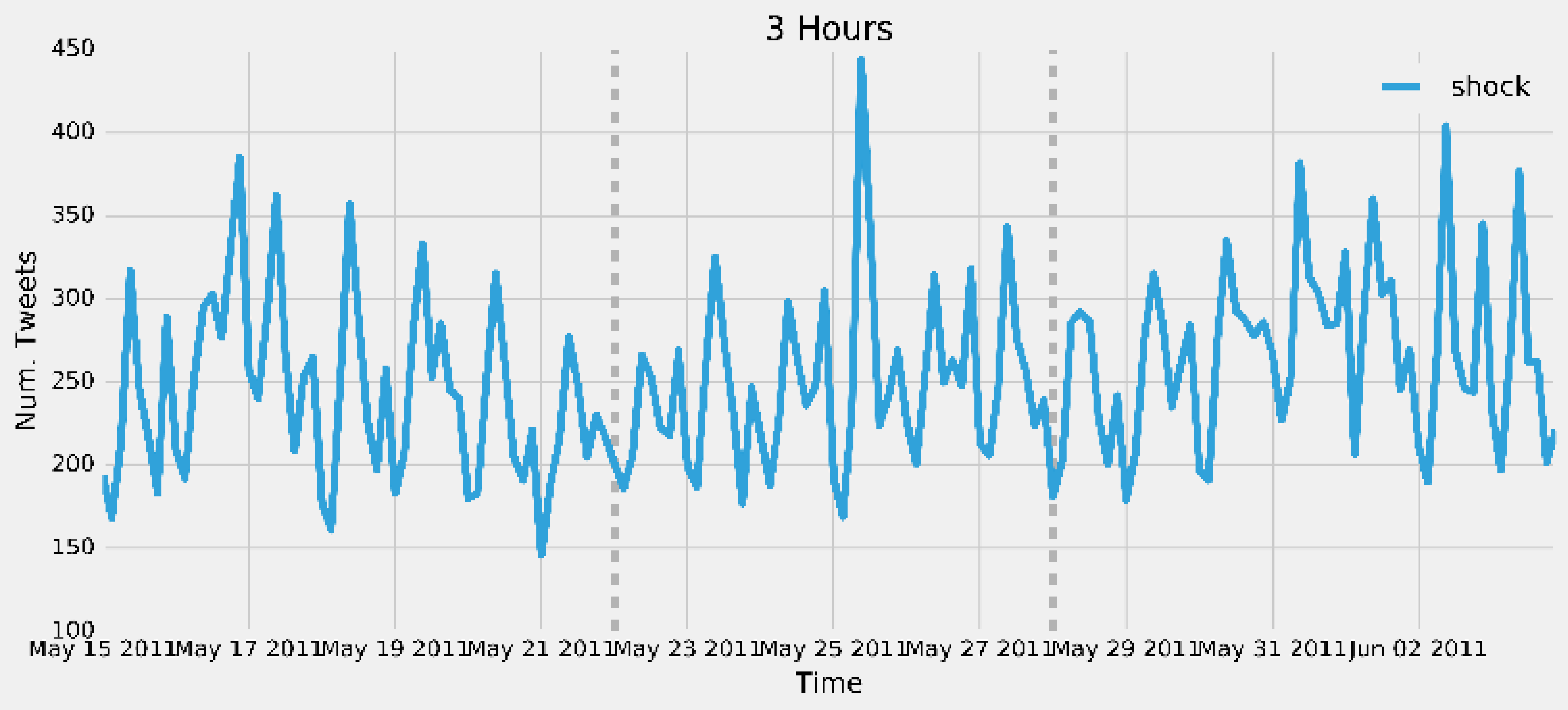

3 Hours
shock
Num. Tweets
450
400
350
300
250
200
150
100
May 15 2011
May 17 2011
May 19 2011
May 21 2011
May 23 2011
May 25 2011
May 27 2011
May 29 2011
May 31 2011
Jun 02 2011
Time

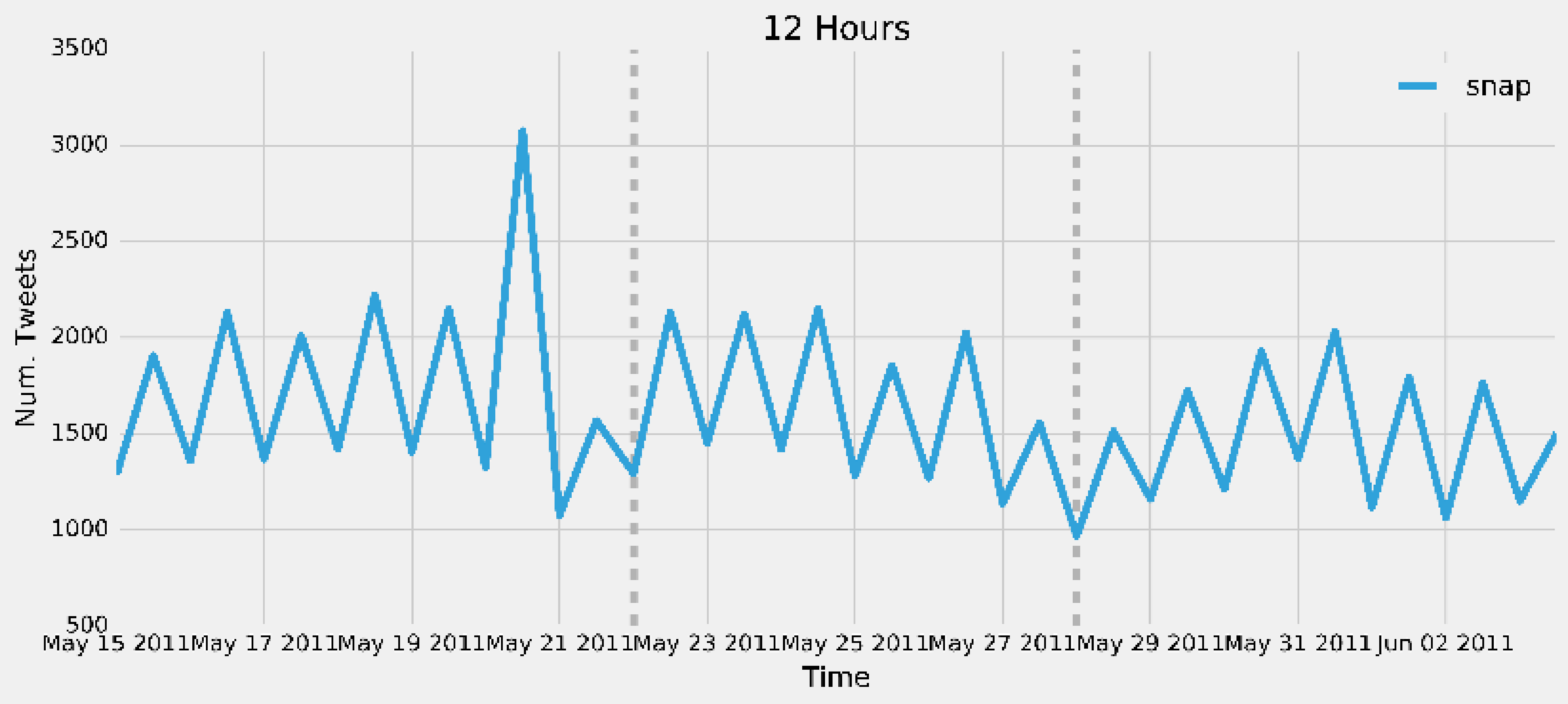
12 Hours
snap
Num. Tweets
3500
3000
2500
2000
1500
1000
500
May 15 2011
May 17 2011
May 19 2011
May 21 2011
May 23 2011
May 25 2011
May 27 2011
May 29 2011
May 31 2011
Jun 02 2011
Time

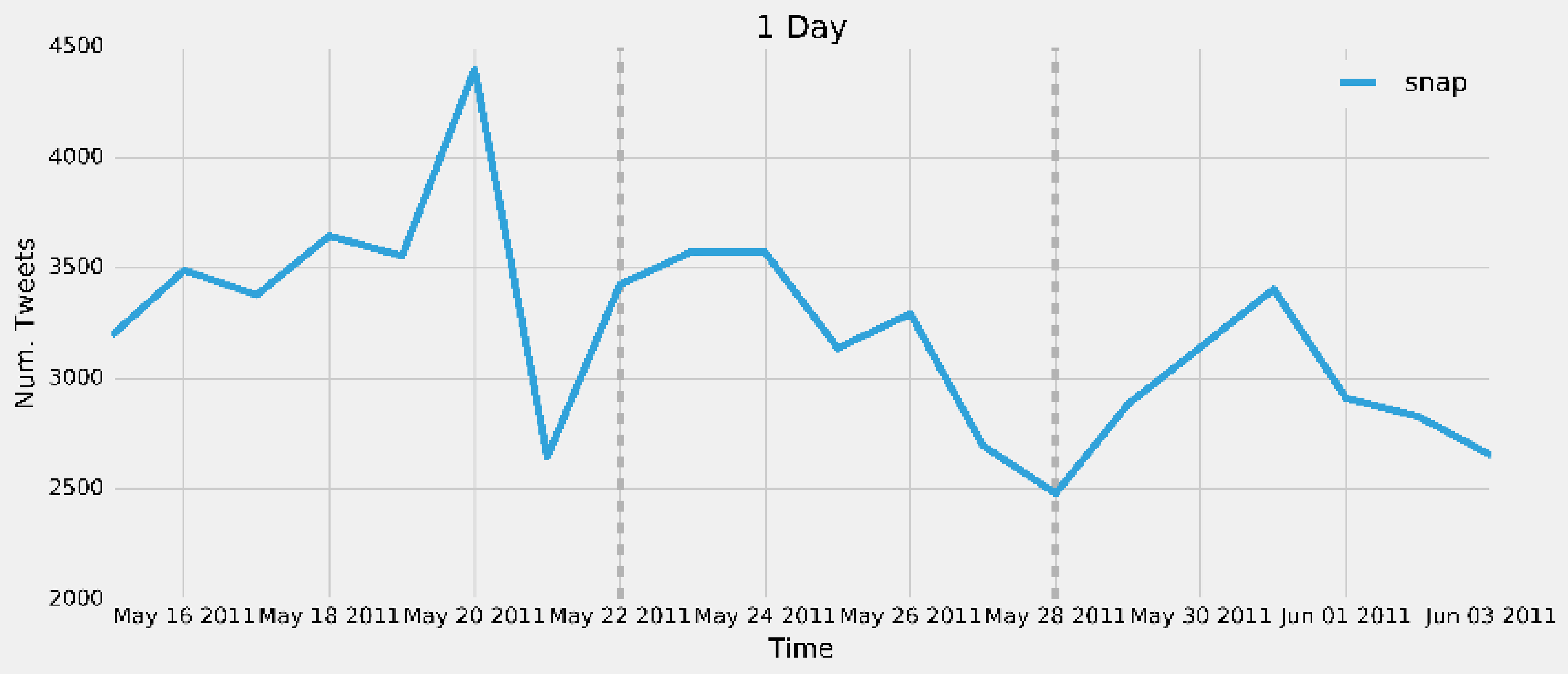

1 Day
snap
4500
4000
3500
3000
2500
2000
Num. Tweets
May 16 2011
May 18 2011
May 20 2011
May 22 2011
May 24 2011
May 26 2011
May 28 2011
May 30 2011
Jun 01 2011
Jun 03 2011
Time

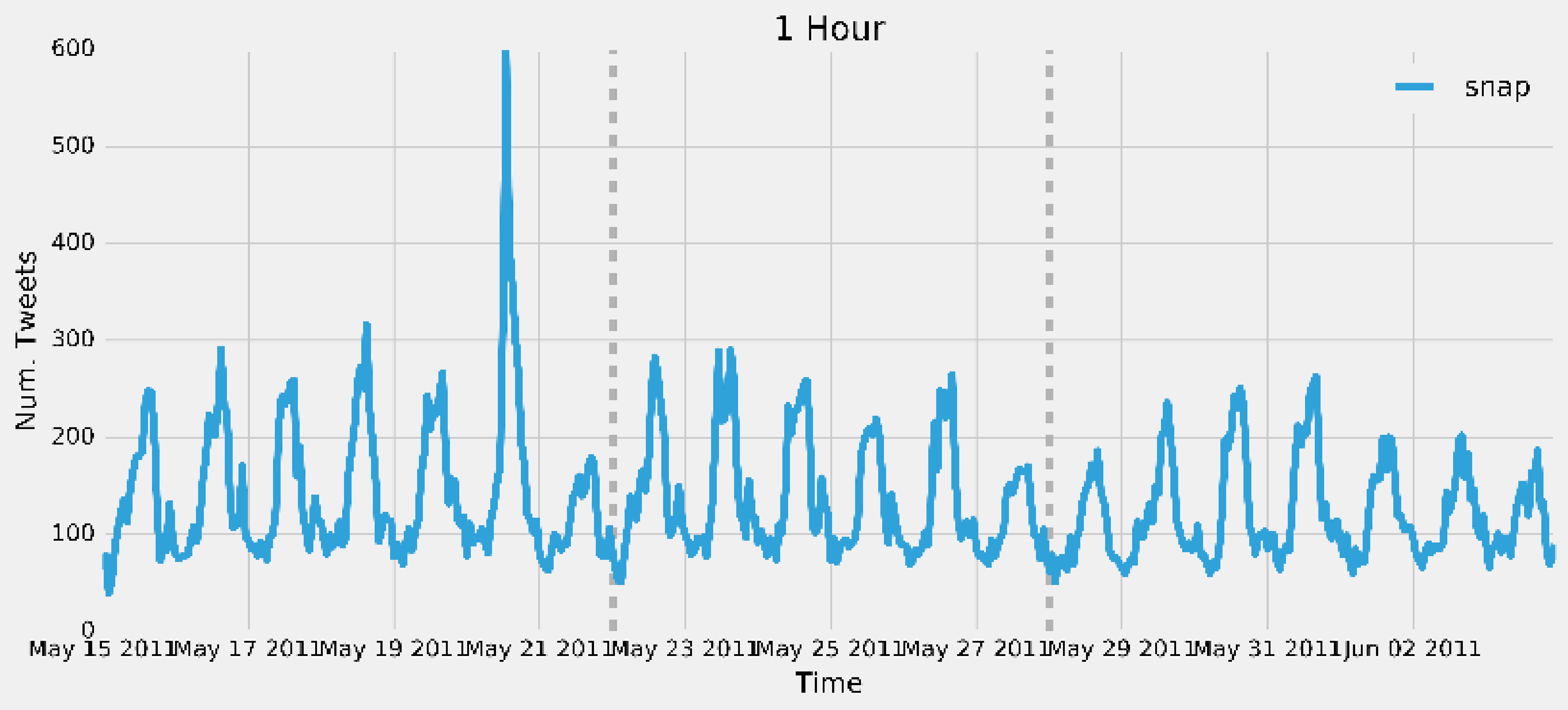

1 Hour
snap
Num. Tweets
0
100
200
300
400
500
600
May 15 2011
May 17 2011
May 19 2011
May 21 2011
May 23 2011
May 25 2011
May 27 2011
May 29 2011
May 31 2011
Jun 02 2011
Time

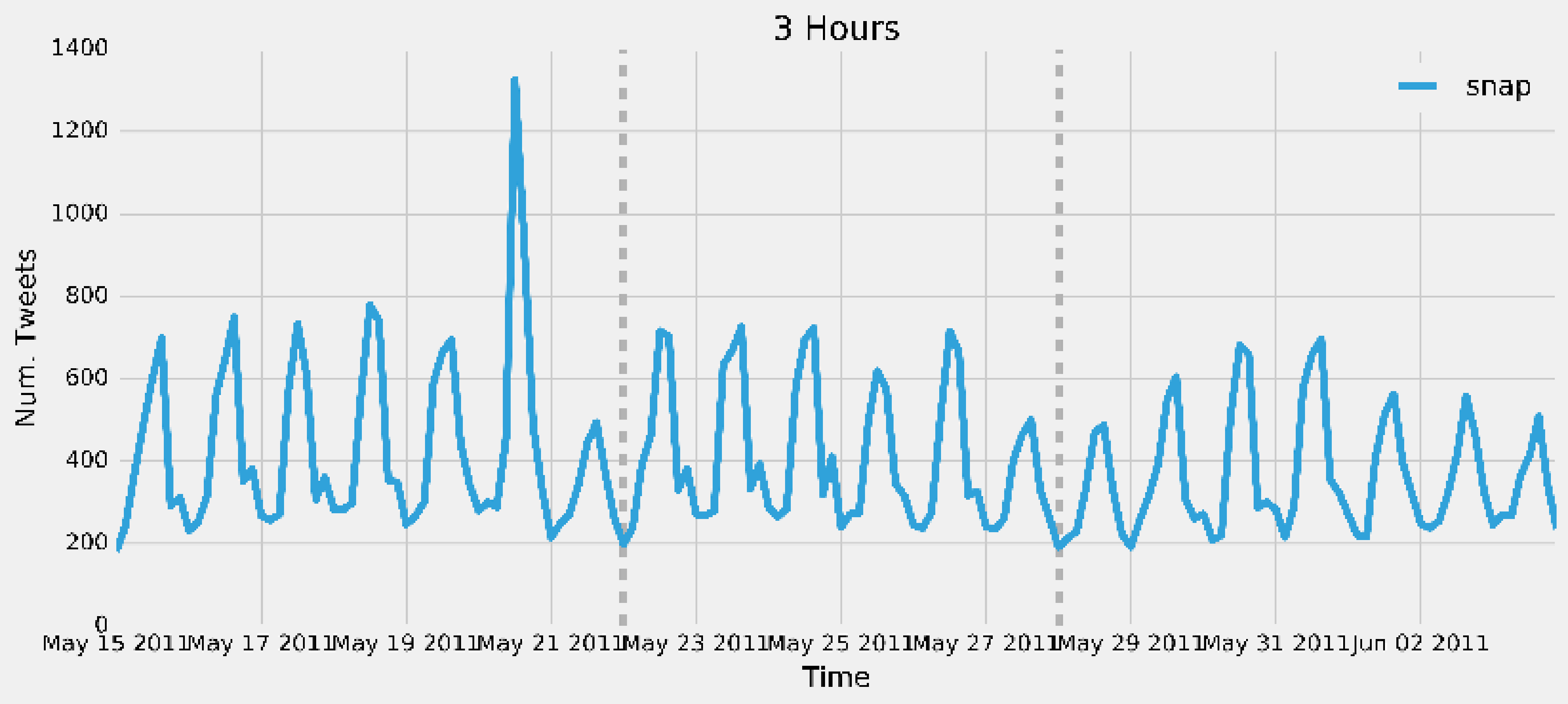
3 Hours
snap
Num. Tweets
0
200
400
600
800
1000
1200
1400
May 15 2011
May 17 2011
May 19 2011
May 21 2011
May 23 2011
May 25 2011
May 27 2011
May 29 2011
May 31 2011
Jun 02 2011
Time

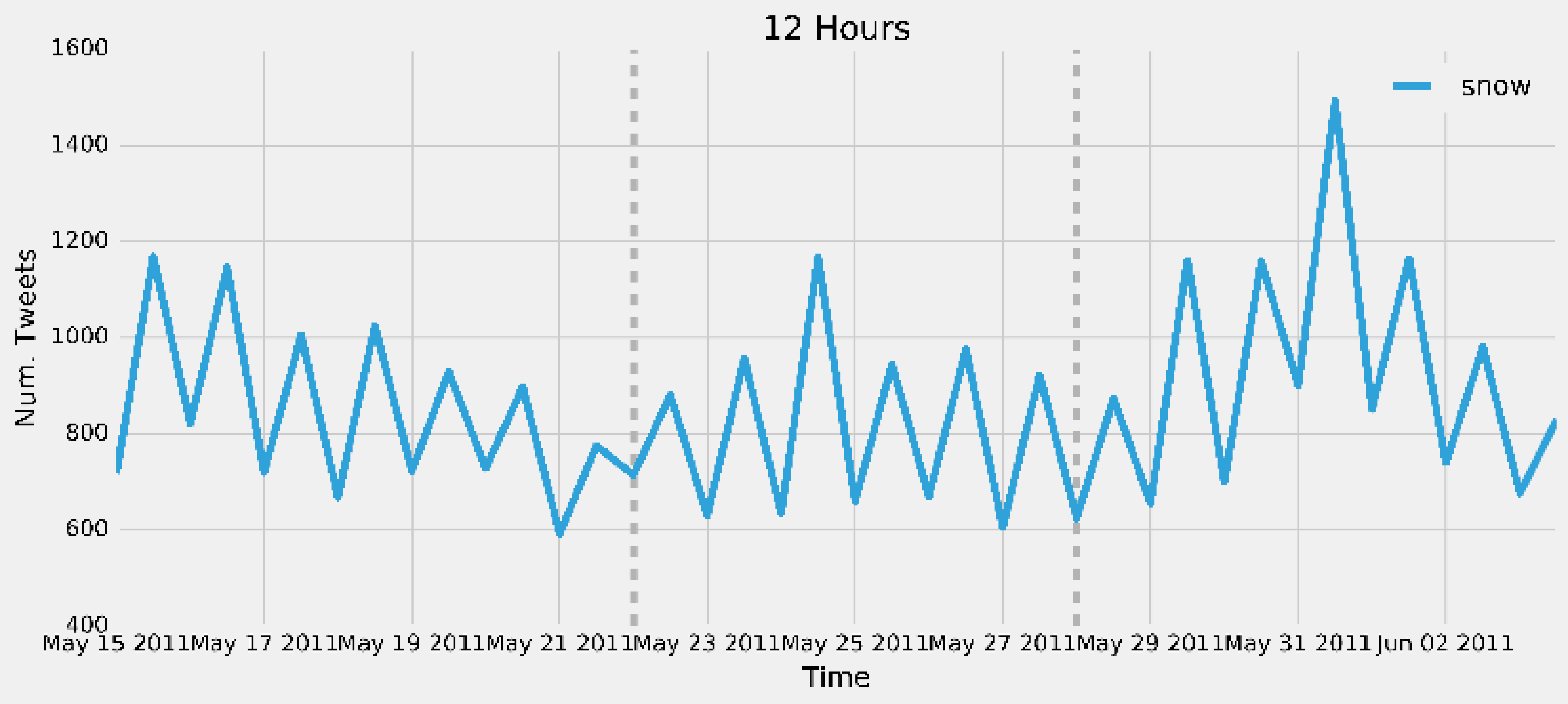

12 Hours
snow
1600
1400
1200
1000
800
600
400
Num. Tweets
May 15 2011
May 17 2011
May 19 2011
May 21 2011
May 23 2011
May 25 2011
May 27 2011
May 29 2011
May 31 2011
Jun 02 2011
Time

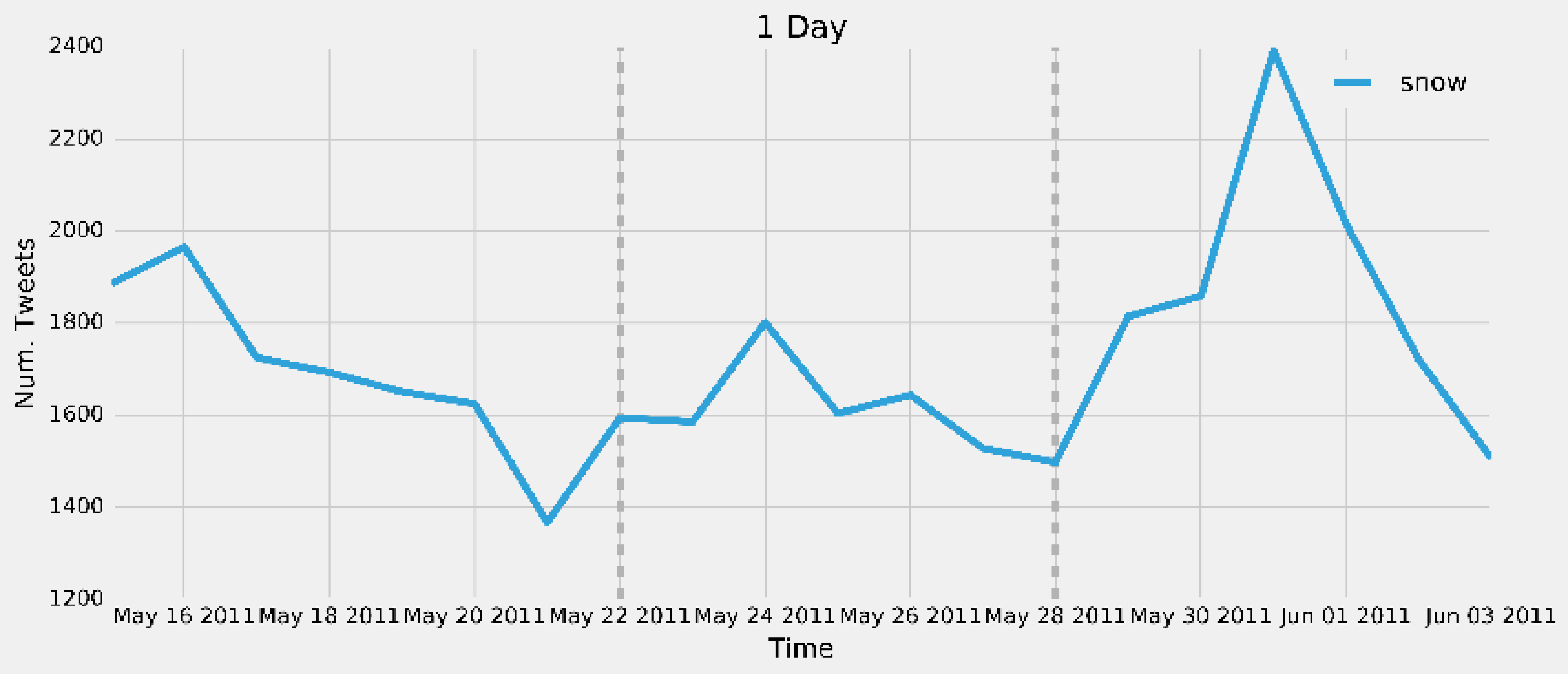
1 Day
snow
Num. Tweets
2400
2200
2000
1800
1600
1400
1200
May 16 2011
May 18 2011
May 20 2011
May 22 2011
May 24 2011
May 26 2011
May 28 2011
May 30 2011
Jun 01 2011
Jun 03 2011
Time

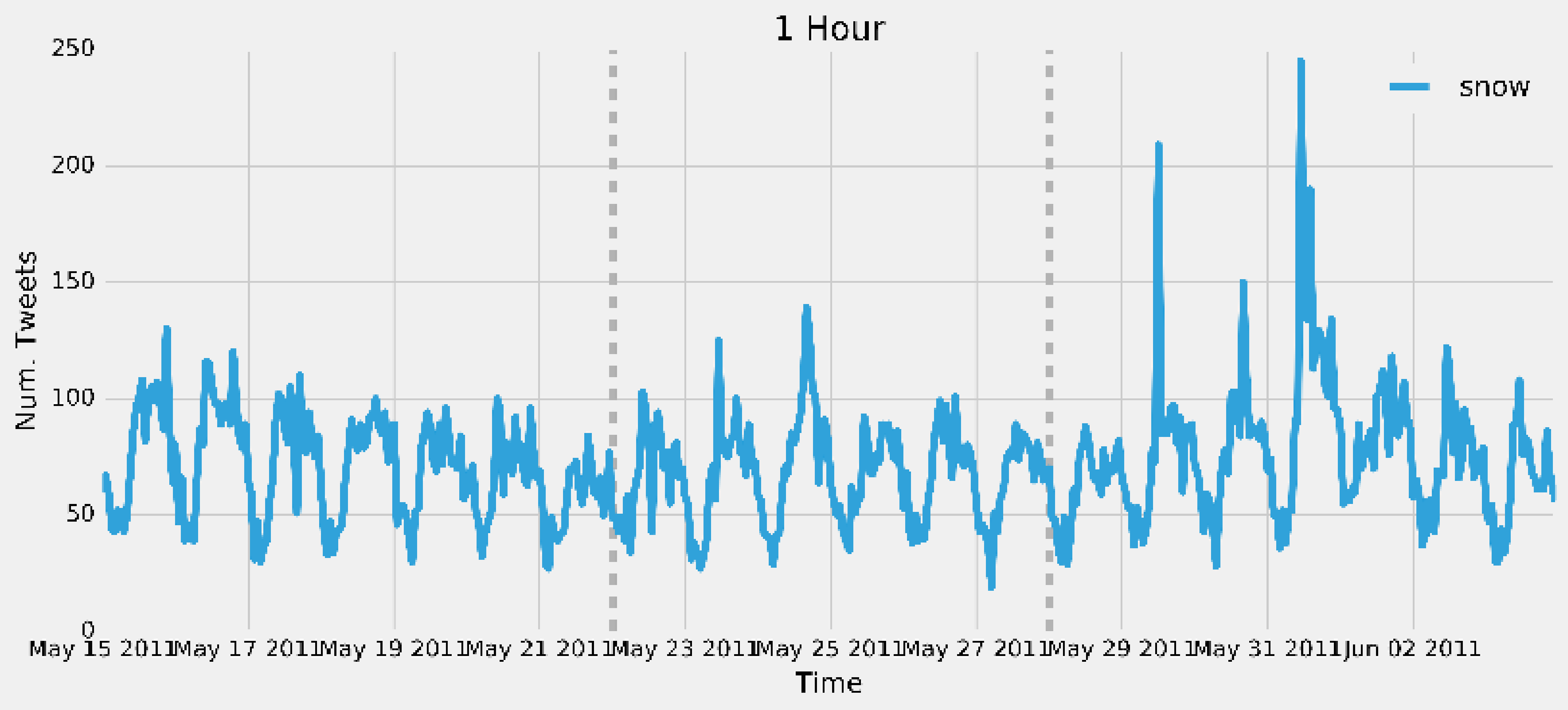
1 Hour
snow
Num. Tweets
250
200
150
100
50
0
May 15 2011
May 17 2011
May 19 2011
May 21 2011
May 23 2011
May 25 2011
May 27 2011
May 29 2011
May 31 2011
Jun 02 2011
Time

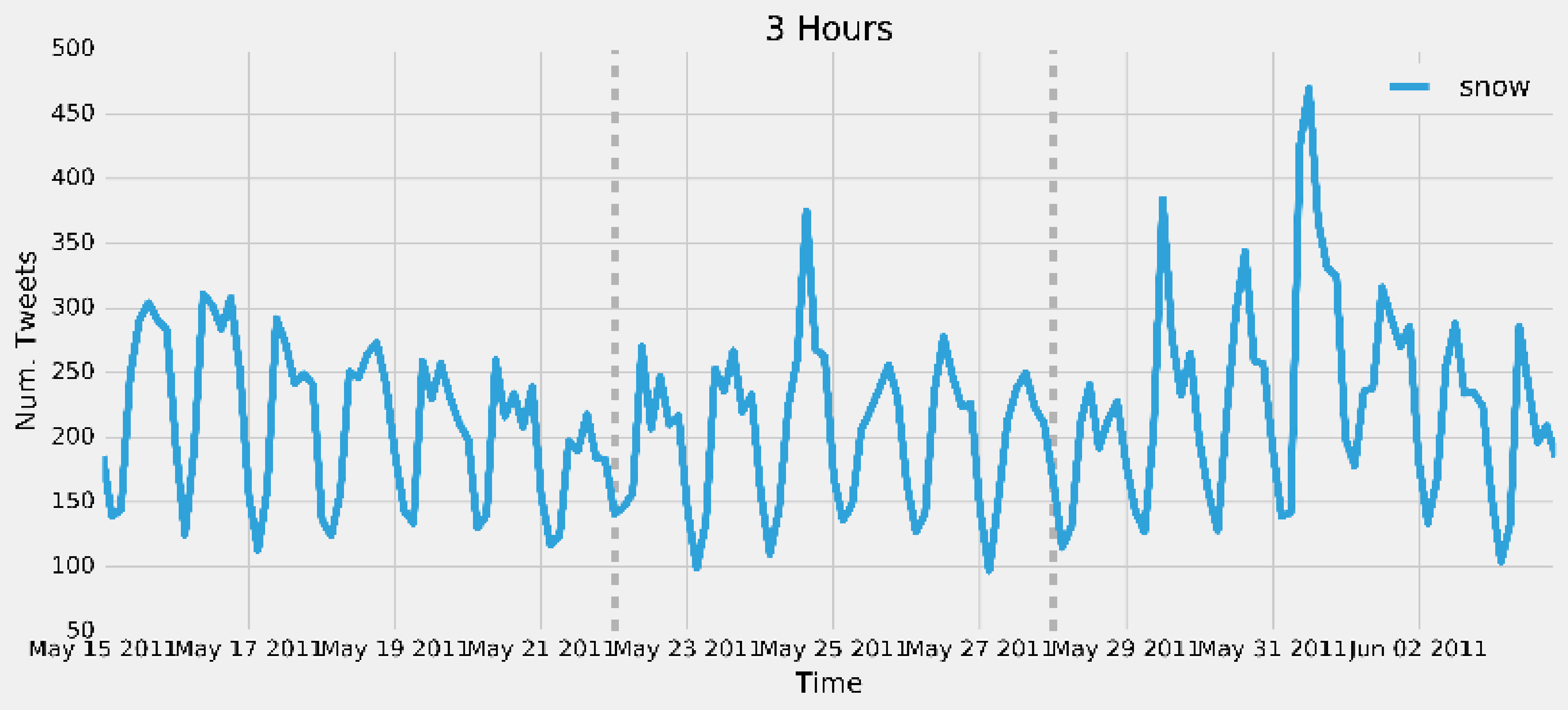
3 Hours
500
450
400
350
300
250
200
150
100
50
Num. Tweets
May 15 2011
May 17 2011
May 19 2011
May 21 2011
May 23 2011
May 25 2011
May 27 2011
May 29 2011
May 31 2011
Jun 02 2011
Time
snow

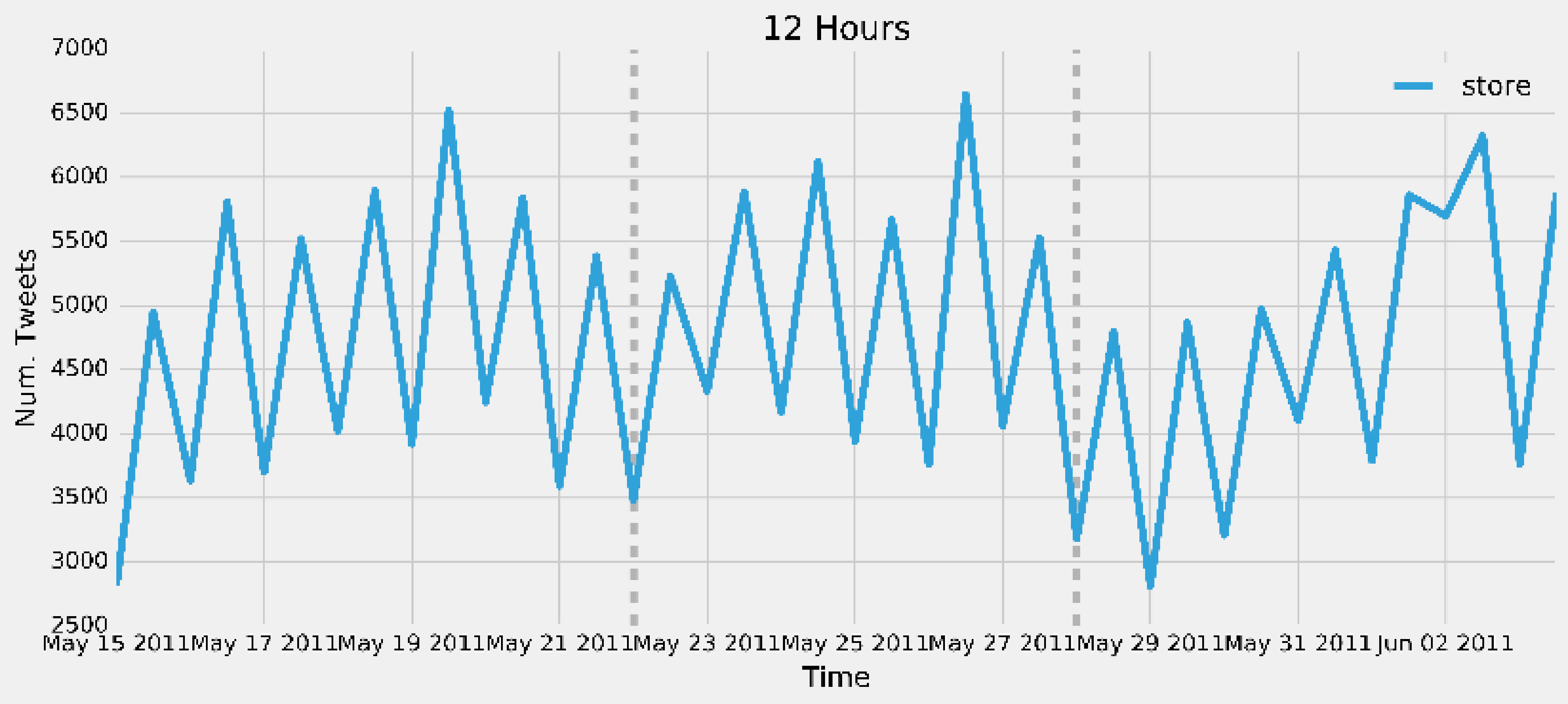
12 Hours
store
Num. Tweets
Time
7000
6500
6000
5500
5000
4500
4000
3500
3000
2500
May 15 2011
May 17 2011
May 19 2011
May 21 2011
May 23 2011
May 25 2011
May 27 2011
May 29 2011
May 31 2011
Jun 02 2011

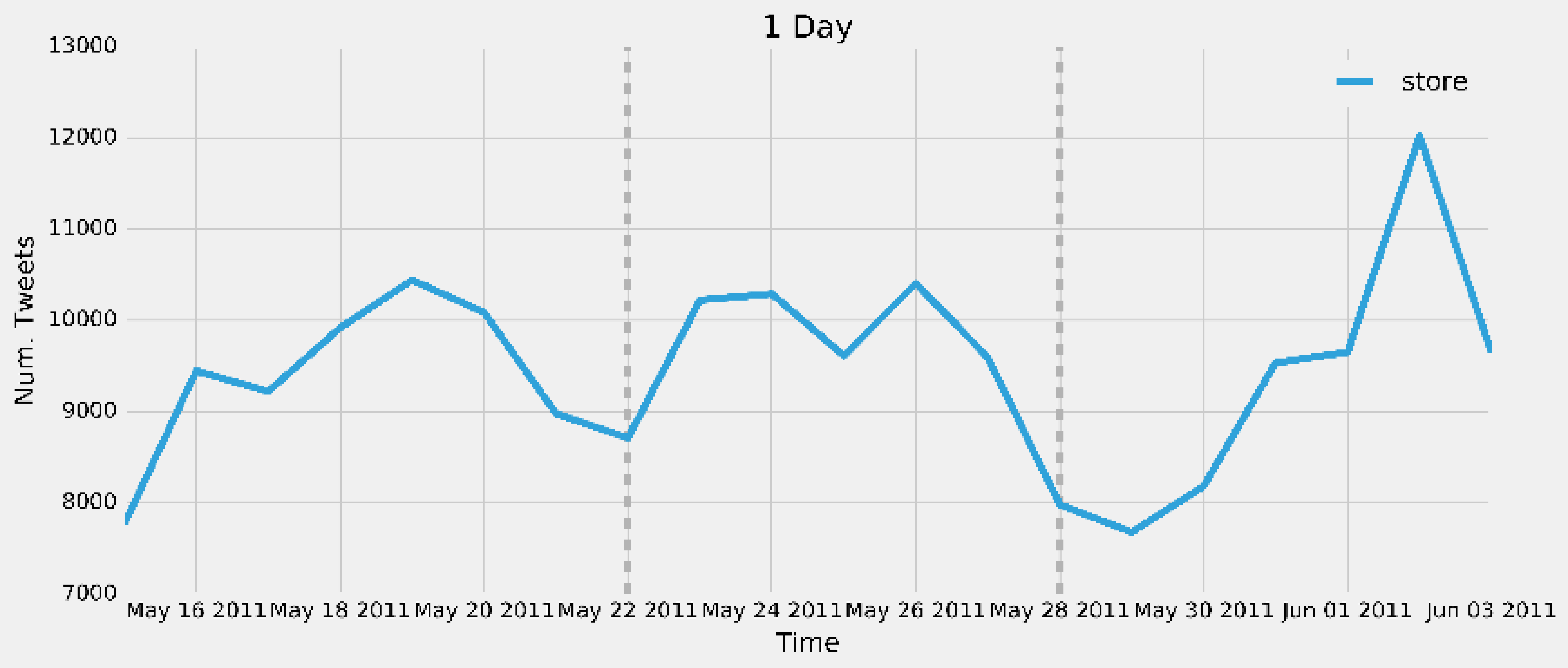

1 Day
store
Num. Tweets
Time
13000
12000
11000
10000
9000
8000
7000
May 16 2011
May 18 2011
May 20 2011
May 22 2011
May 24 2011
May 26 2011
May 28 2011
May 30 2011
Jun 01 2011
Jun 03 2011

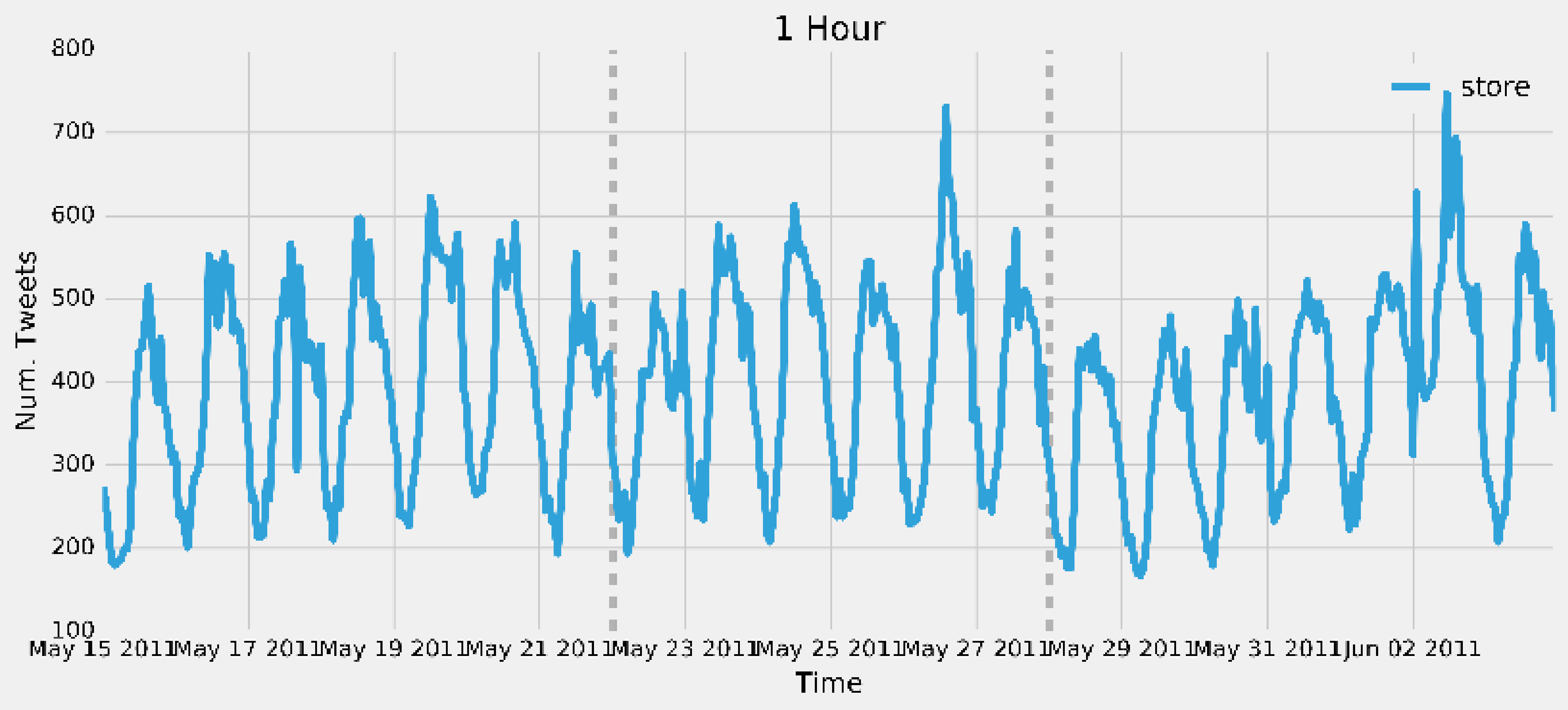

1 Hour
store
800
700
600
500
400
300
200
100
Num. Tweets
May 15 2011
May 17 2011
May 19 2011
May 21 2011
May 23 2011
May 25 2011
May 27 2011
May 29 2011
May 31 2011
Jun 02 2011
Time

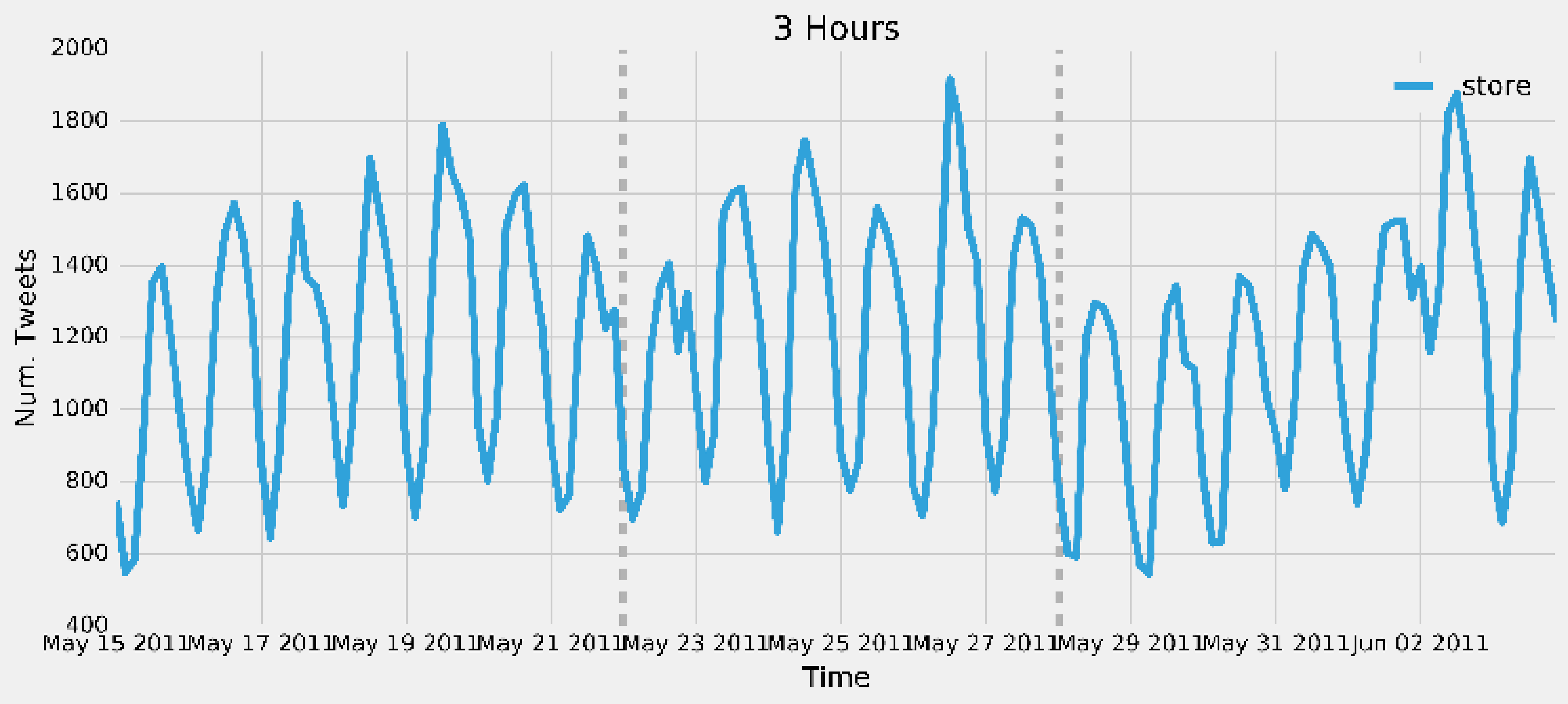
3 Hours
store
Num. Tweets
2000
1800
1600
1400
1200
1000
800
600
400
May 15 2011
May 17 2011
May 19 2011
May 21 2011
May 23 2011
May 25 2011
May 27 2011
May 29 2011
May 31 2011
Jun 02 2011
Time

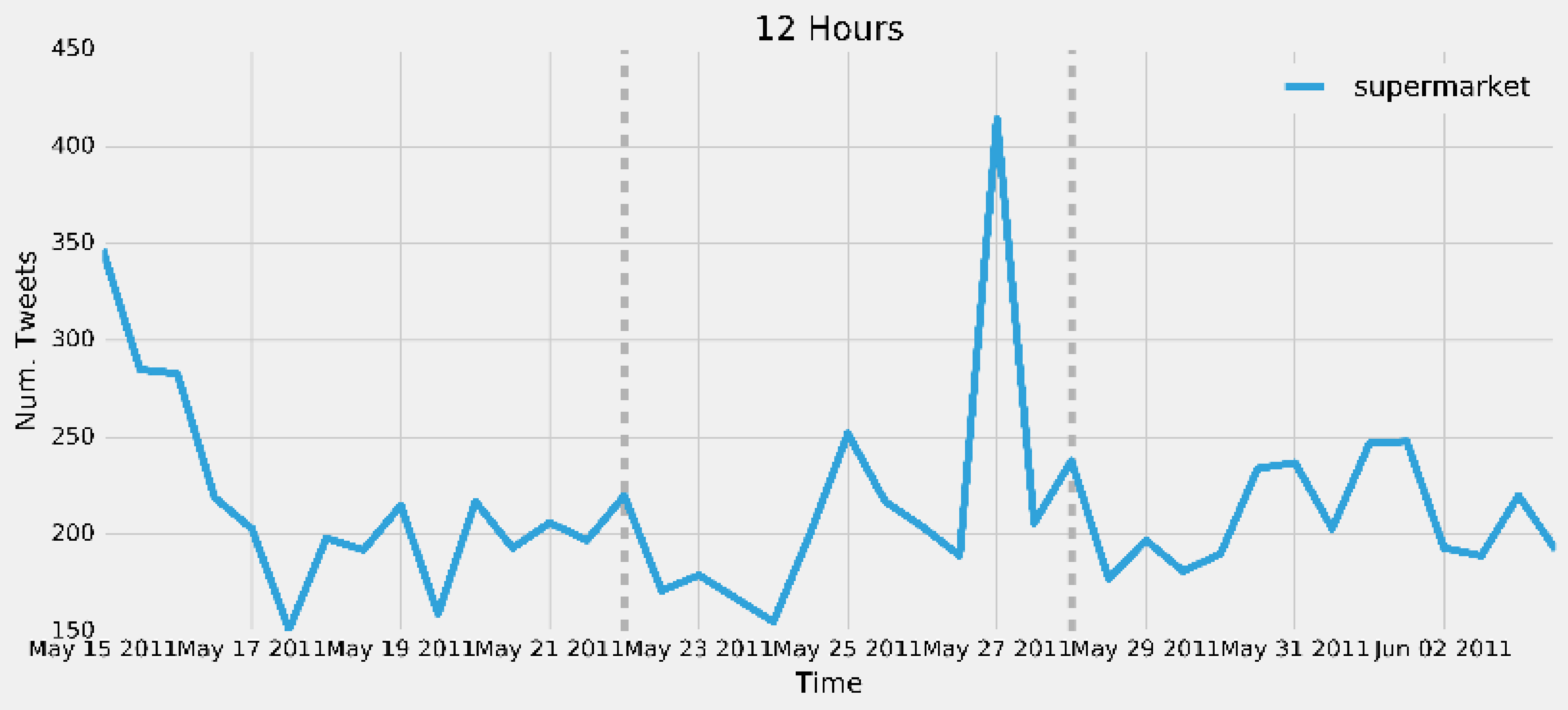
12 Hours
supermarket
450
400
350
300
250
200
150
Num. Tweets
May 15 2011
May 17 2011
May 19 2011
May 21 2011
May 23 2011
May 25 2011
May 27 2011
May 29 2011
May 31 2011
Jun 02 2011
Time

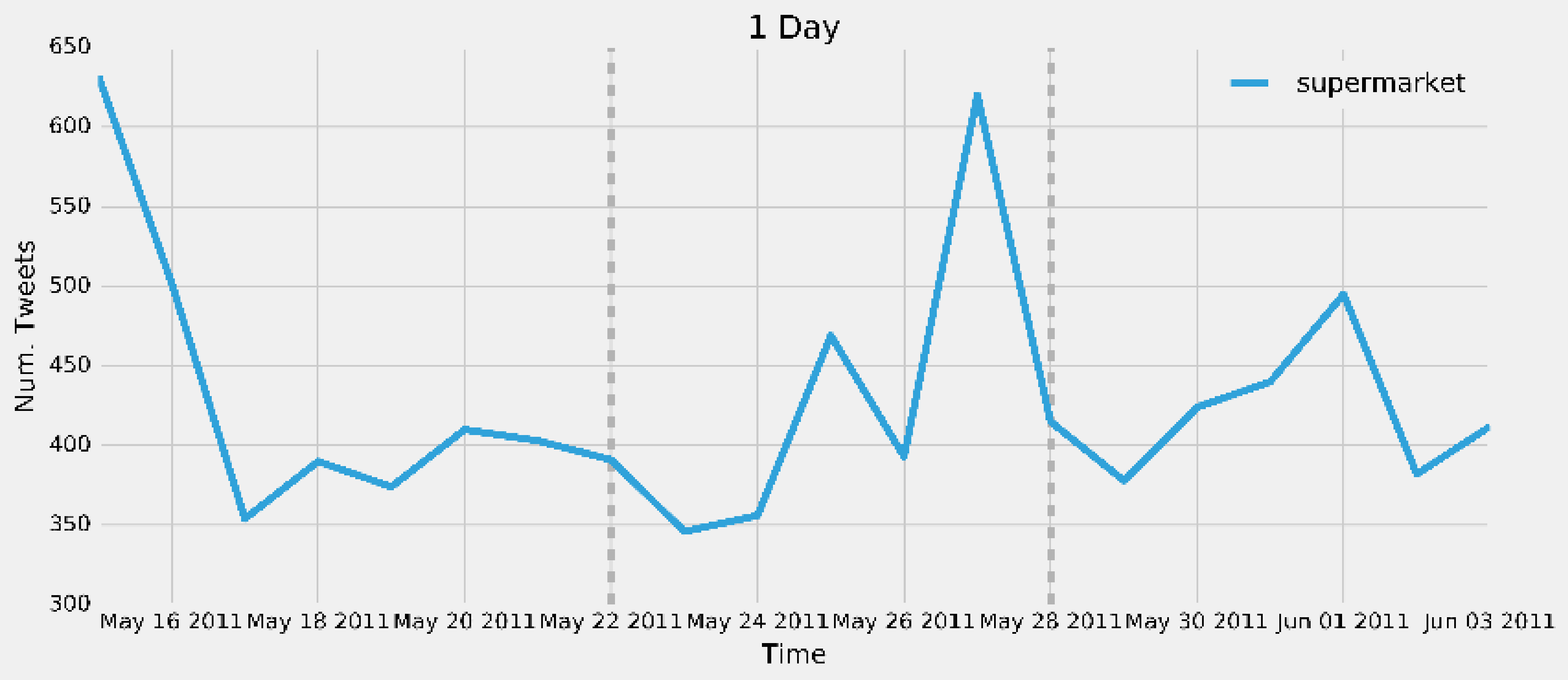

1 Day
supermarket
Num. Tweets
650
600
550
500
450
400
350
300
May 16 2011
May 18 2011
May 20 2011
May 22 2011
May 24 2011
May 26 2011
May 28 2011
May 30 2011
Jun 01 2011
Jun 03 2011
Time

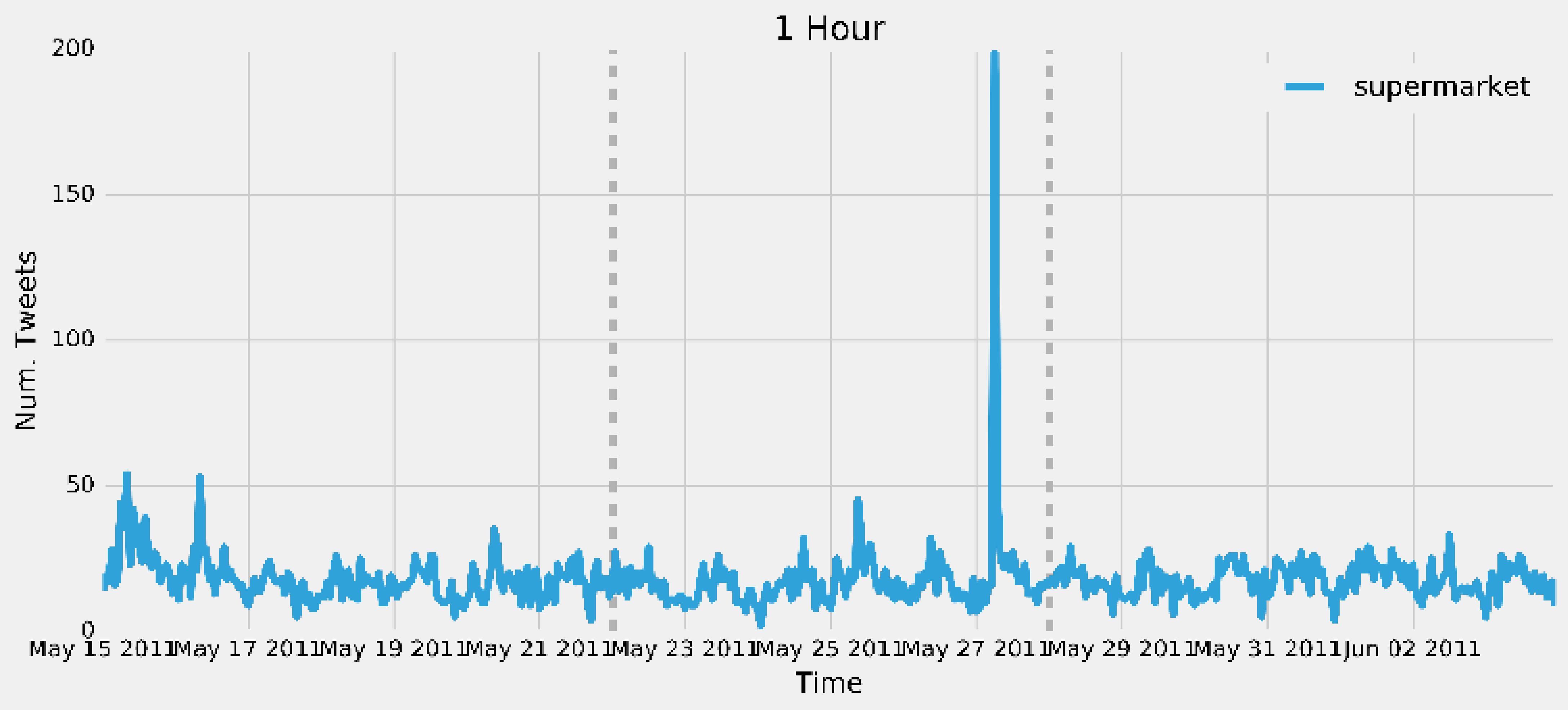
1 Hour
supermarket
Num. Tweets
0
50
100
150
200
May 15 2011
May 17 2011
May 19 2011
May 21 2011
May 23 2011
May 25 2011
May 27 2011
May 29 2011
May 31 2011
Jun 02 2011
Time

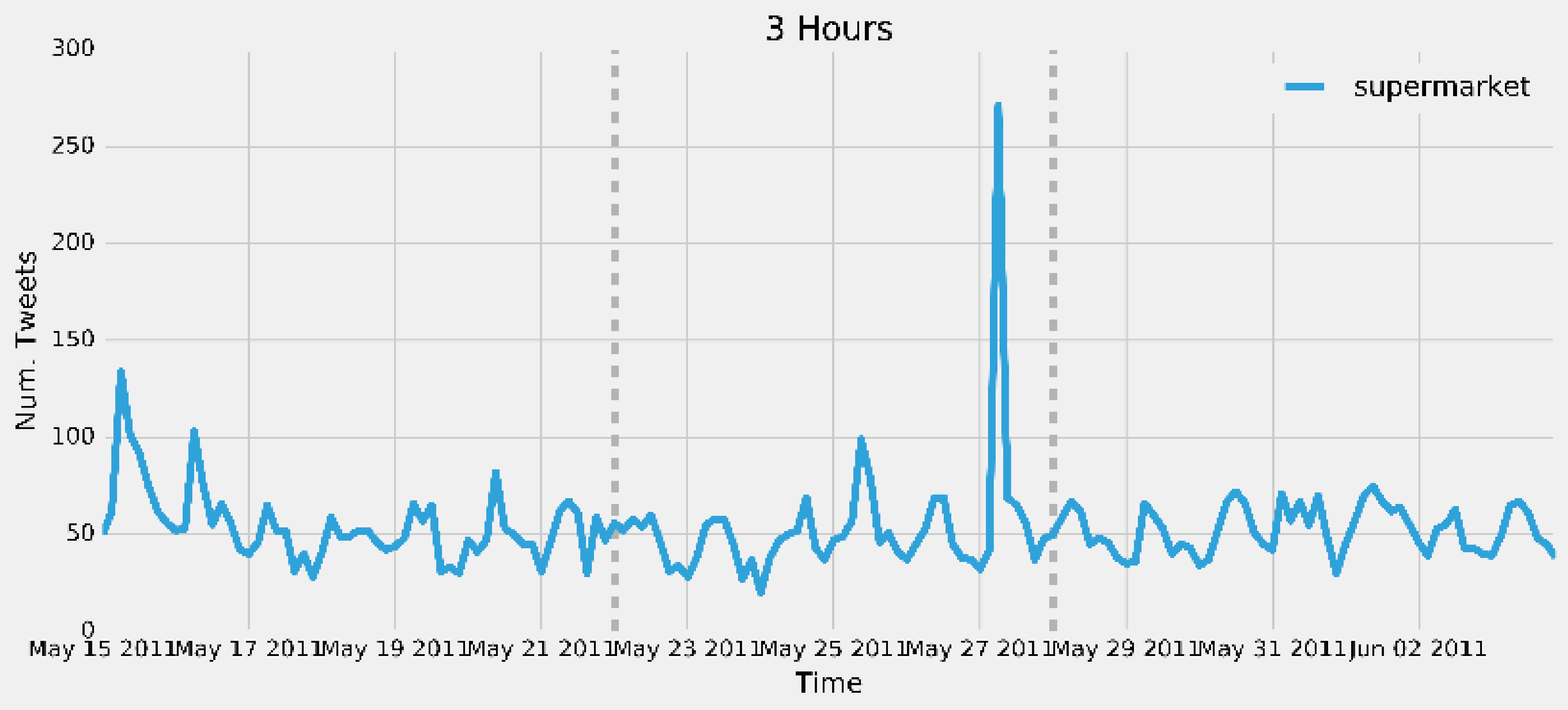
3 Hours
supermarket
Num. Tweets
300
250
200
150
100
50
0
May 15 2011
May 17 2011
May 19 2011
May 21 2011
May 23 2011
May 25 2011
May 27 2011
May 29 2011
May 31 2011
Jun 02 2011
Time

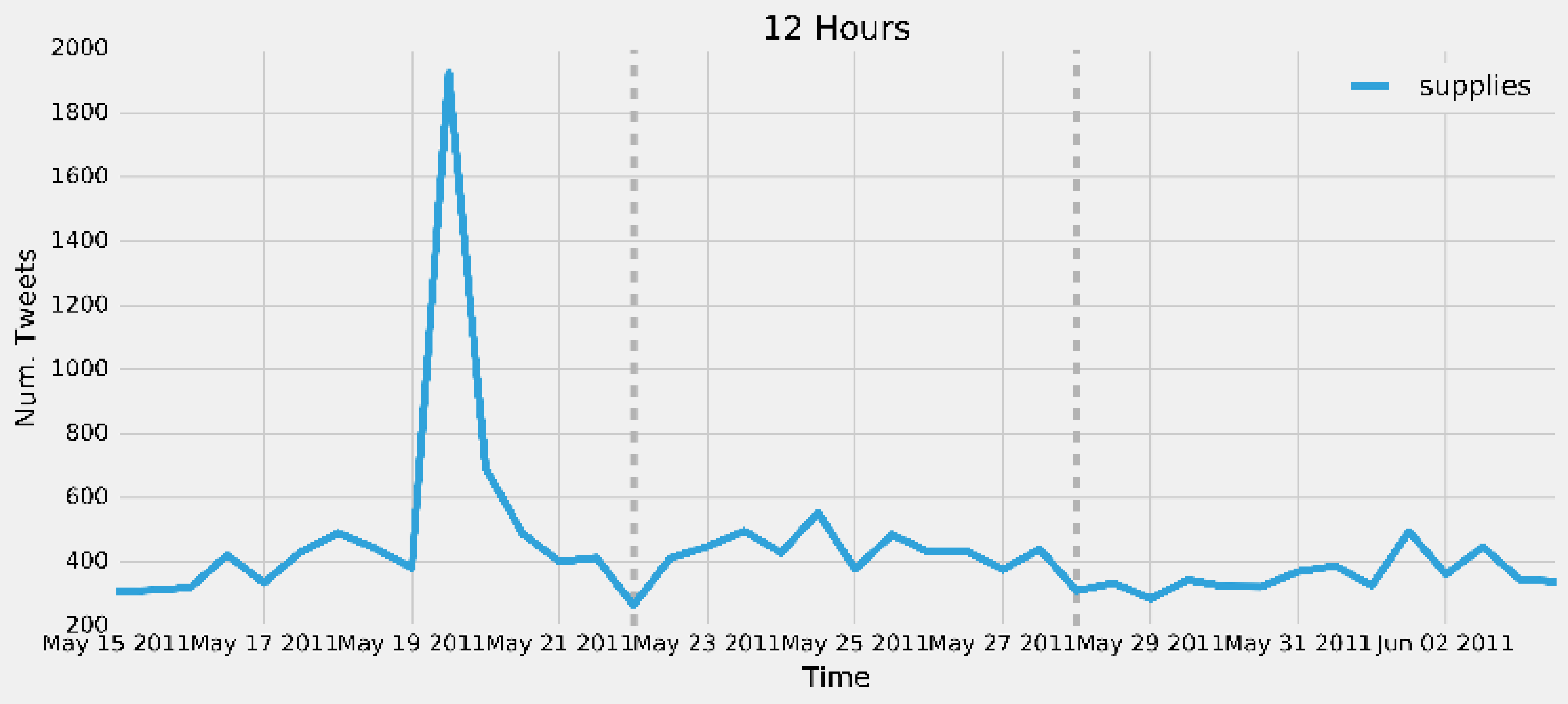
12 Hours
supplies
Num. Tweets
2000
1800
1600
1400
1200
1000
800
600
400
200
May 15 2011
May 17 2011
May 19 2011
May 21 2011
May 23 2011
May 25 2011
May 27 2011
May 29 2011
May 31 2011
Jun 02 2011
Time

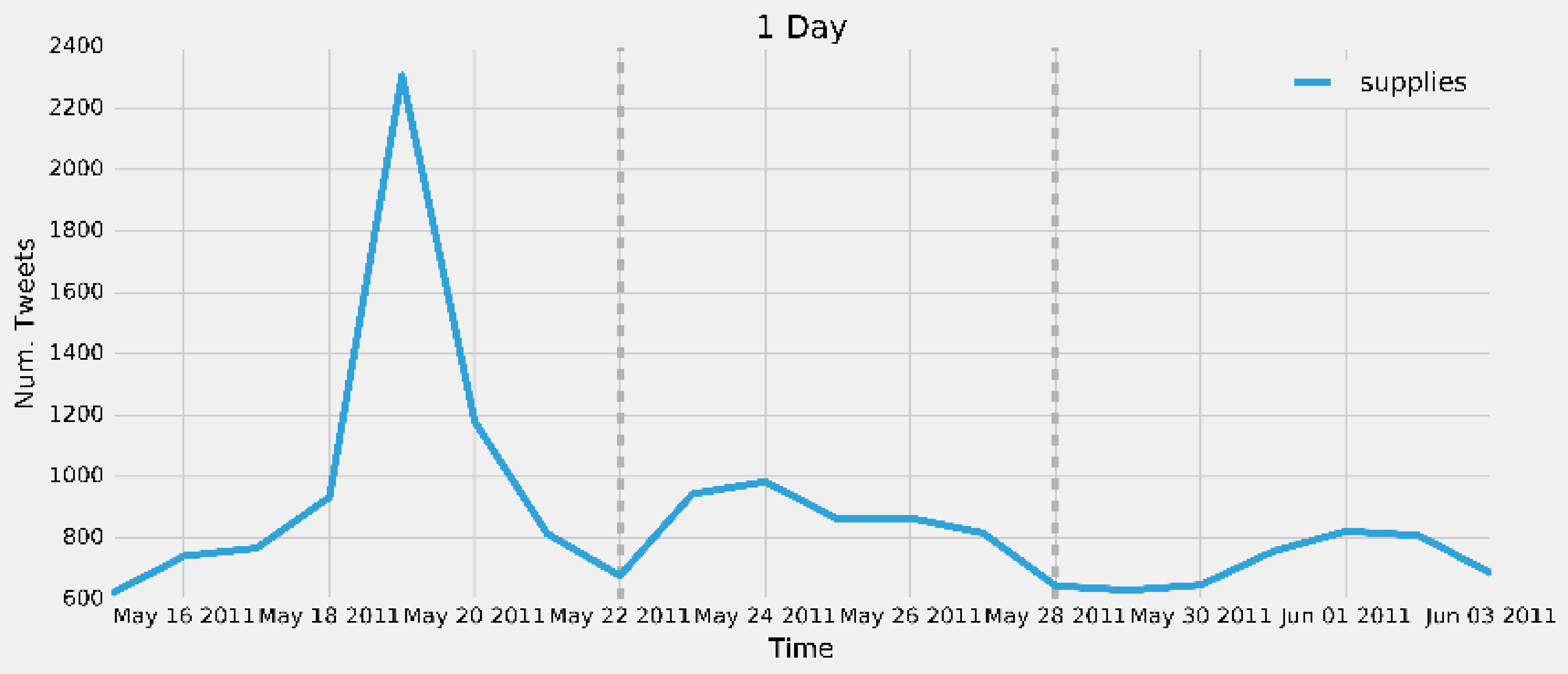

1 Day
supplies
Num. Tweets
2400
2200
2000
1800
1600
1400
1200
1000
800
600
May 16 2011
May 18 2011
May 20 2011
May 22 2011
May 24 2011
May 26 2011
May 28 2011
May 30 2011
Jun 01 2011
Jun 03 2011
Time

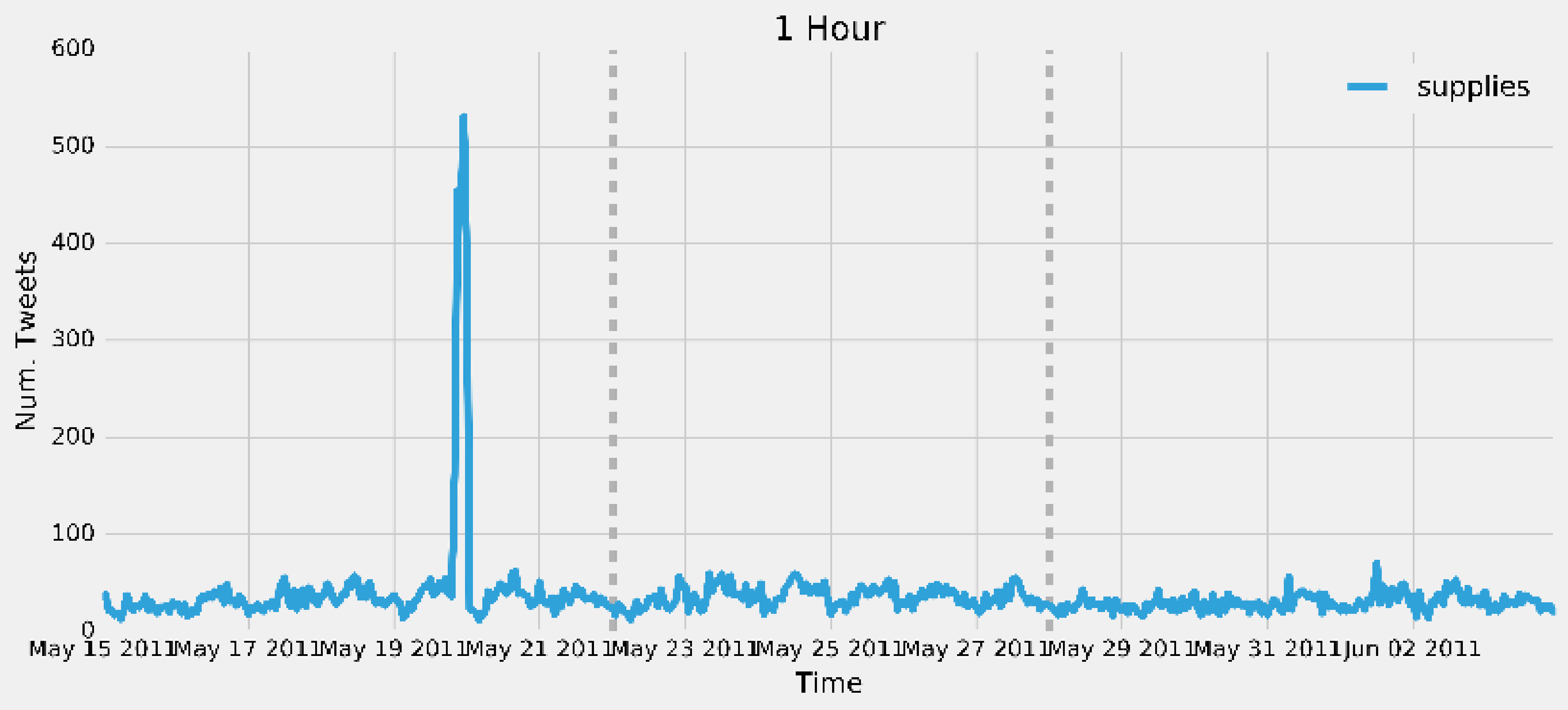

1 Hour
Num. Tweets
0
100
200
300
400
500
600
supplies
May 15 2011
May 17 2011
May 19 2011
May 21 2011
May 23 2011
May 25 2011
May 27 2011
May 29 2011
May 31 2011
Jun 02 2011
Time

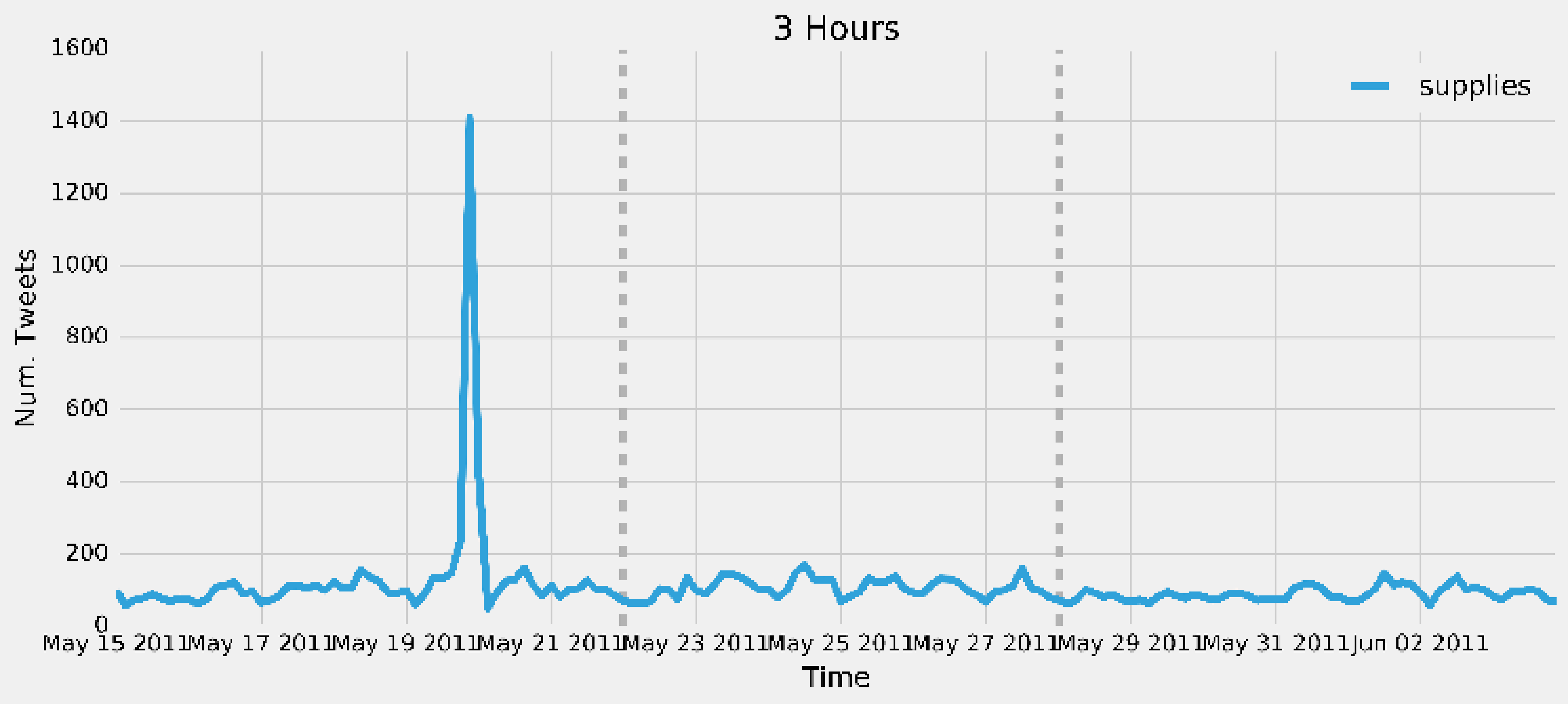

3 Hours
supplies
Num. Tweets
0
200
400
600
800
1000
1200
1400
1600
May 15 2011
May 17 2011
May 19 2011
May 21 2011
May 23 2011
May 25 2011
May 27 2011
May 29 2011
May 31 2011
Jun 02 2011
Time

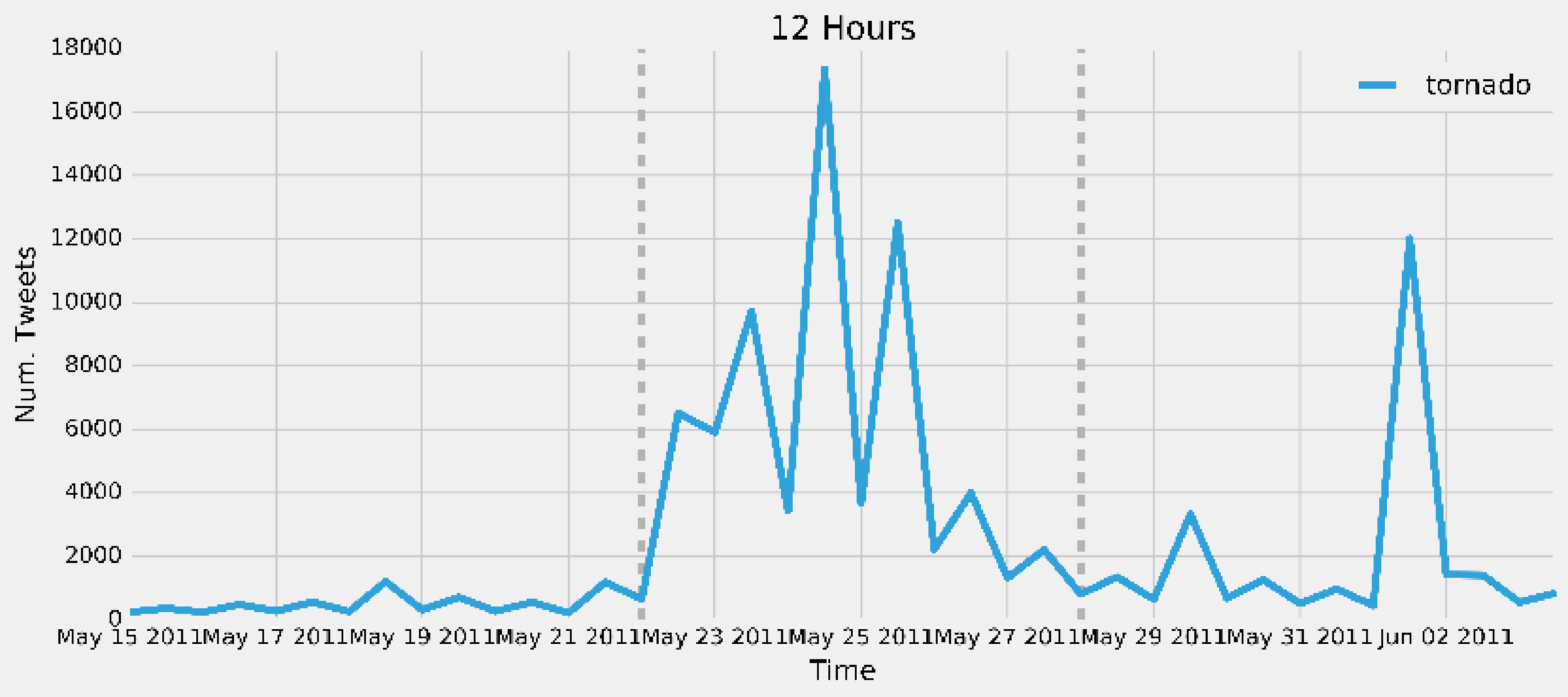
12 Hours
tornado
Num. Tweets
Time
0
2000
4000
6000
8000
10000
12000
14000
16000
18000
May 15 2011
May 17 2011
May 19 2011
May 21 2011
May 23 2011
May 25 2011
May 27 2011
May 29 2011
May 31 2011
Jun 02 2011

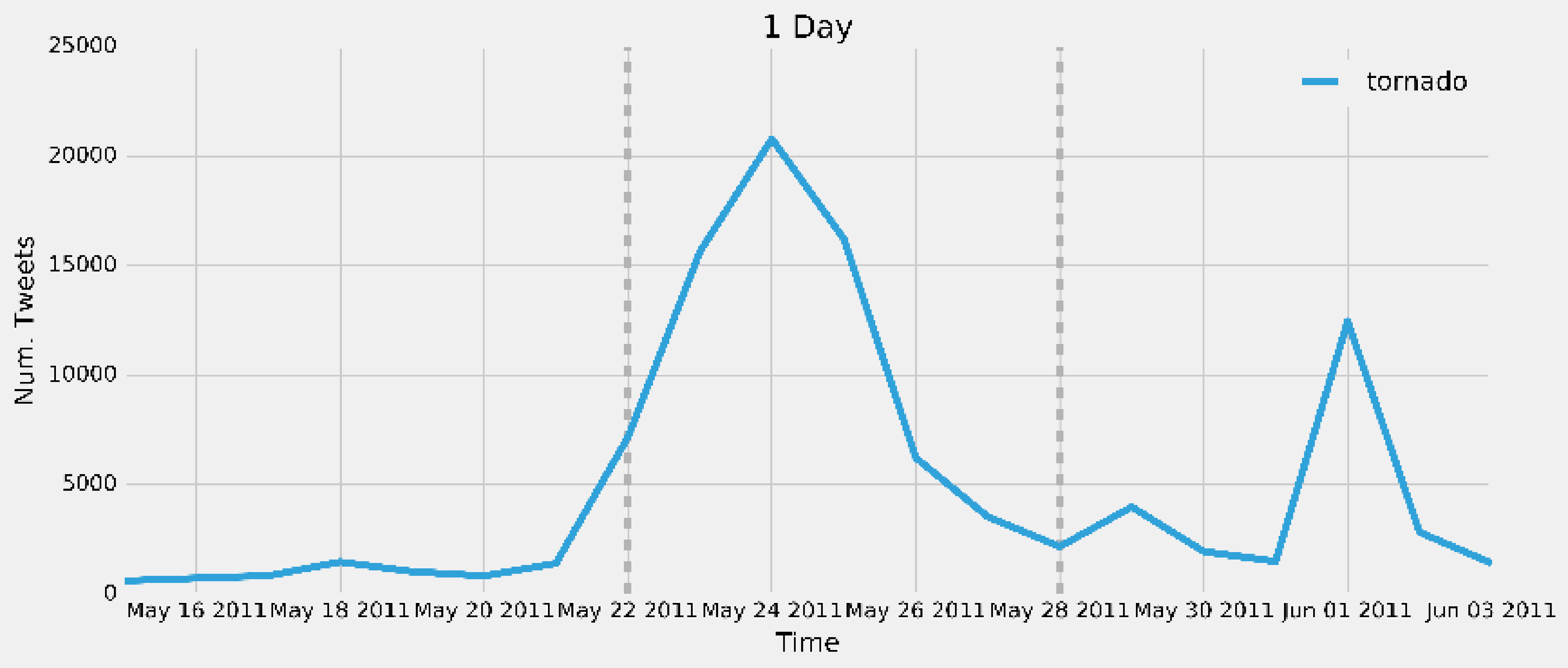
1 Day
25000
20000
15000
10000
5000
0
Num. Tweets
tornado
May 16 2011
May 18 2011
May 20 2011
May 22 2011
May 24 2011
May 26 2011
May 28 2011
May 30 2011
Jun 01 2011
Jun 03 2011
Time

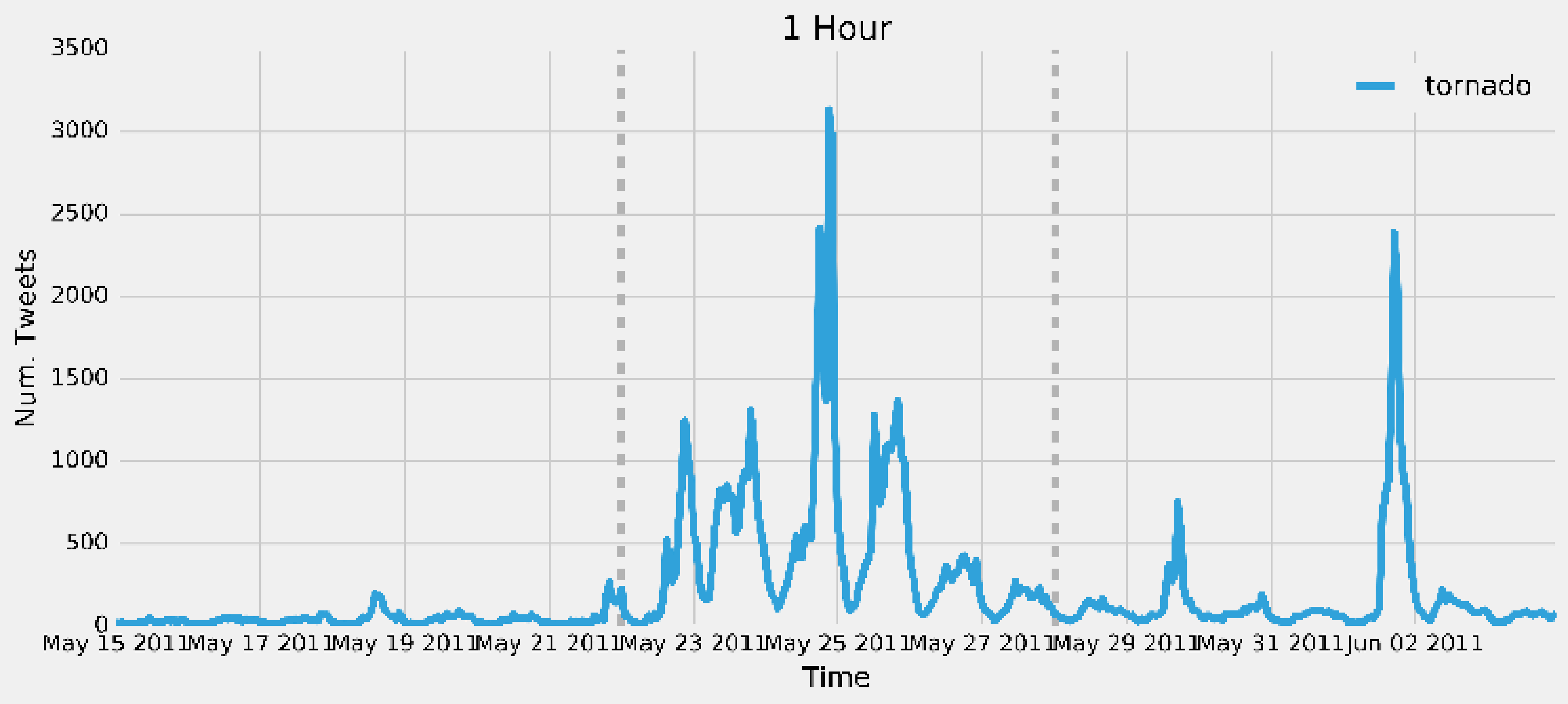
1 Hour
tornado
Num. Tweets
Time
0
500
1000
1500
2000
2500
3000
3500
May 15 2011
May 17 2011
May 19 2011
May 21 2011
May 23 2011
May 25 2011
May 27 2011
May 29 2011
May 31 2011
Jun 02 2011

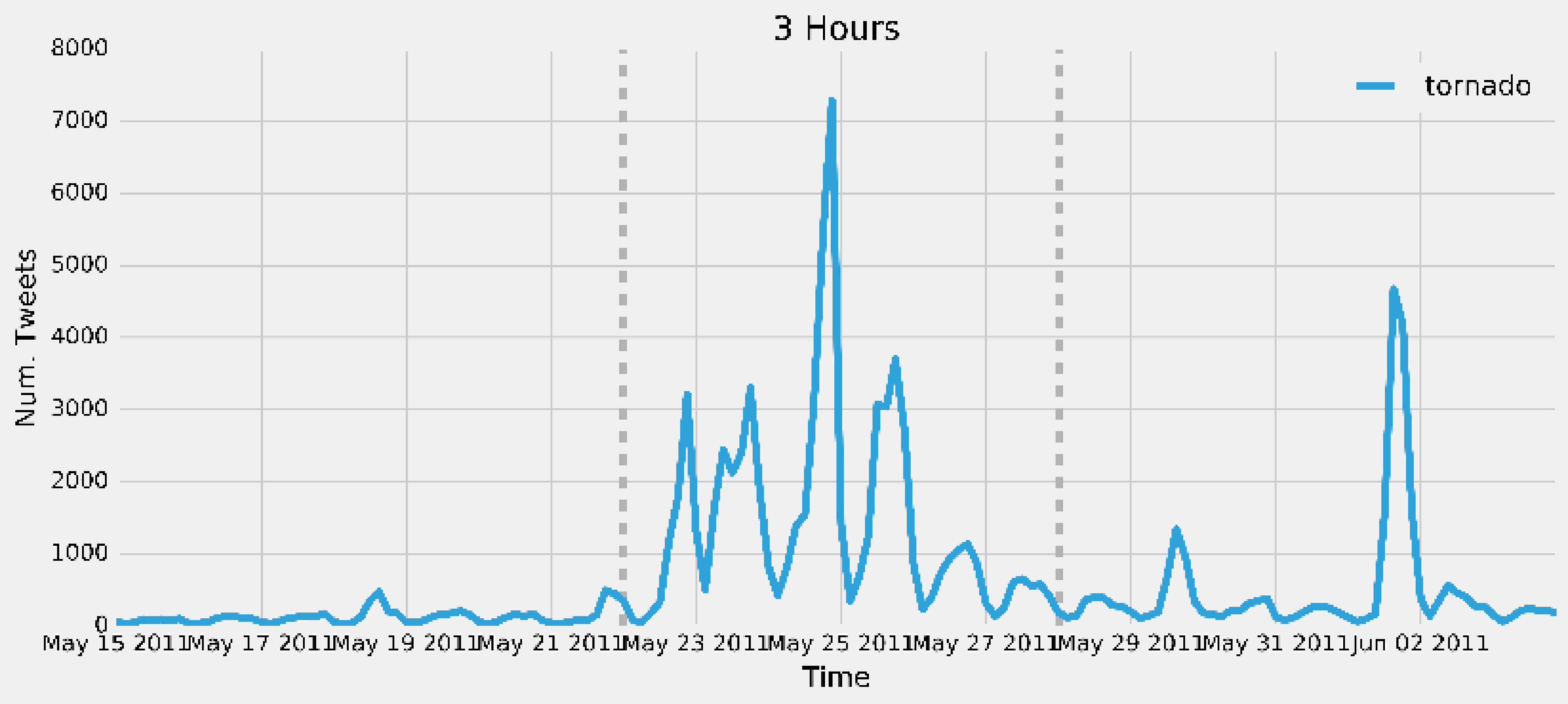

3 Hours
tornado
Num. Tweets
Time
0
1000
2000
3000
4000
5000
6000
7000
8000
May 15 2011
May 17 2011
May 19 2011
May 21 2011
May 23 2011
May 25 2011
May 27 2011
May 29 2011
May 31 2011
Jun 02 2011

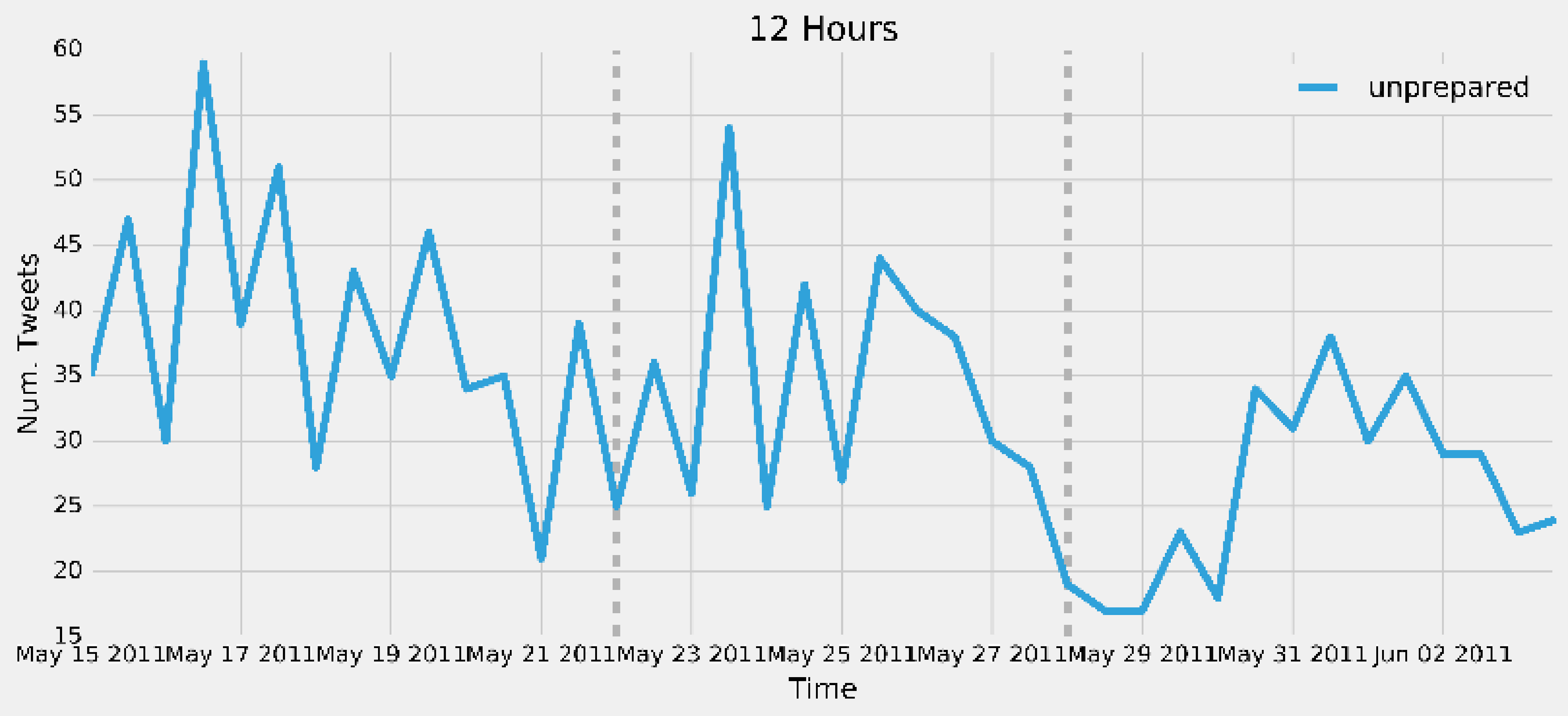
12 Hours
unprepared
Num. Tweets
60
55
50
45
40
35
30
25
20
15
May 15 2011
May 17 2011
May 19 2011
May 21 2011
May 23 2011
May 25 2011
May 27 2011
May 29 2011
May 31 2011
Jun 02 2011
Time

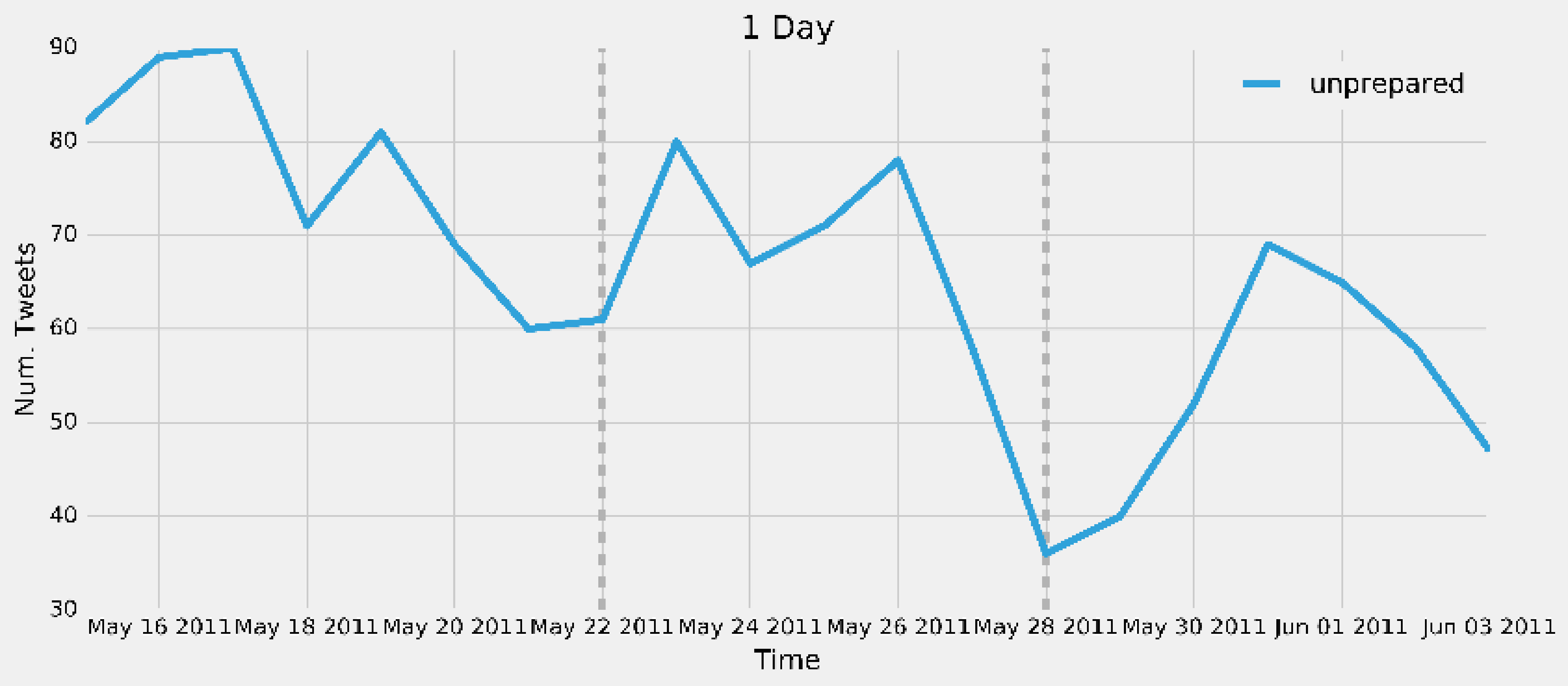
1 Day
unprepared
Num. Tweets
90
80
70
60
50
40
30
May 16 2011
May 18 2011
May 20 2011
May 22 2011
May 24 2011
May 26 2011
May 28 2011
May 30 2011
Jun 01 2011
Jun 03 2011
Time

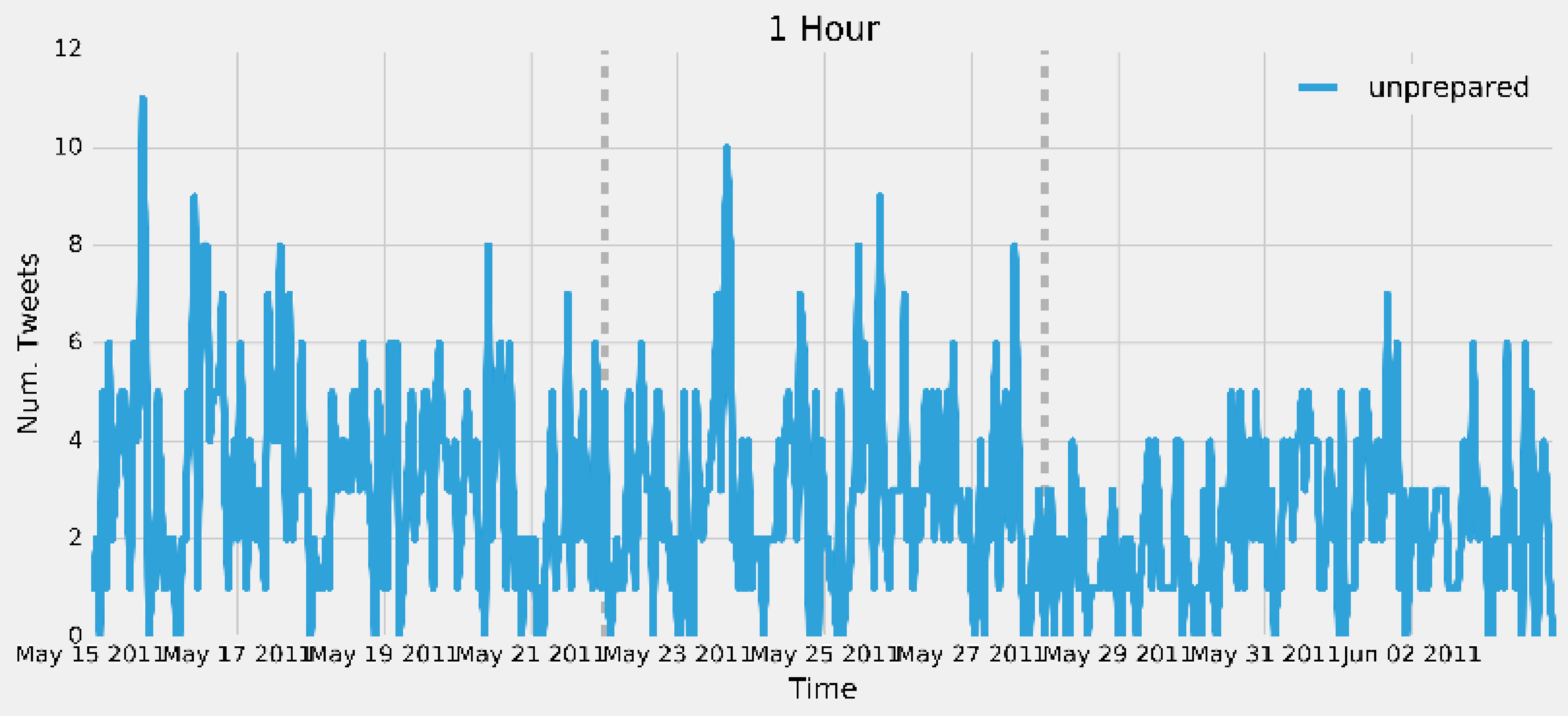
1 Hour
unprepared
Num. Tweets
Time
0
2
4
6
8
10
12
May 15 2011
May 17 2011
May 19 2011
May 21 2011
May 23 2011
May 25 2011
May 27 2011
May 29 2011
May 31 2011
Jun 02 2011

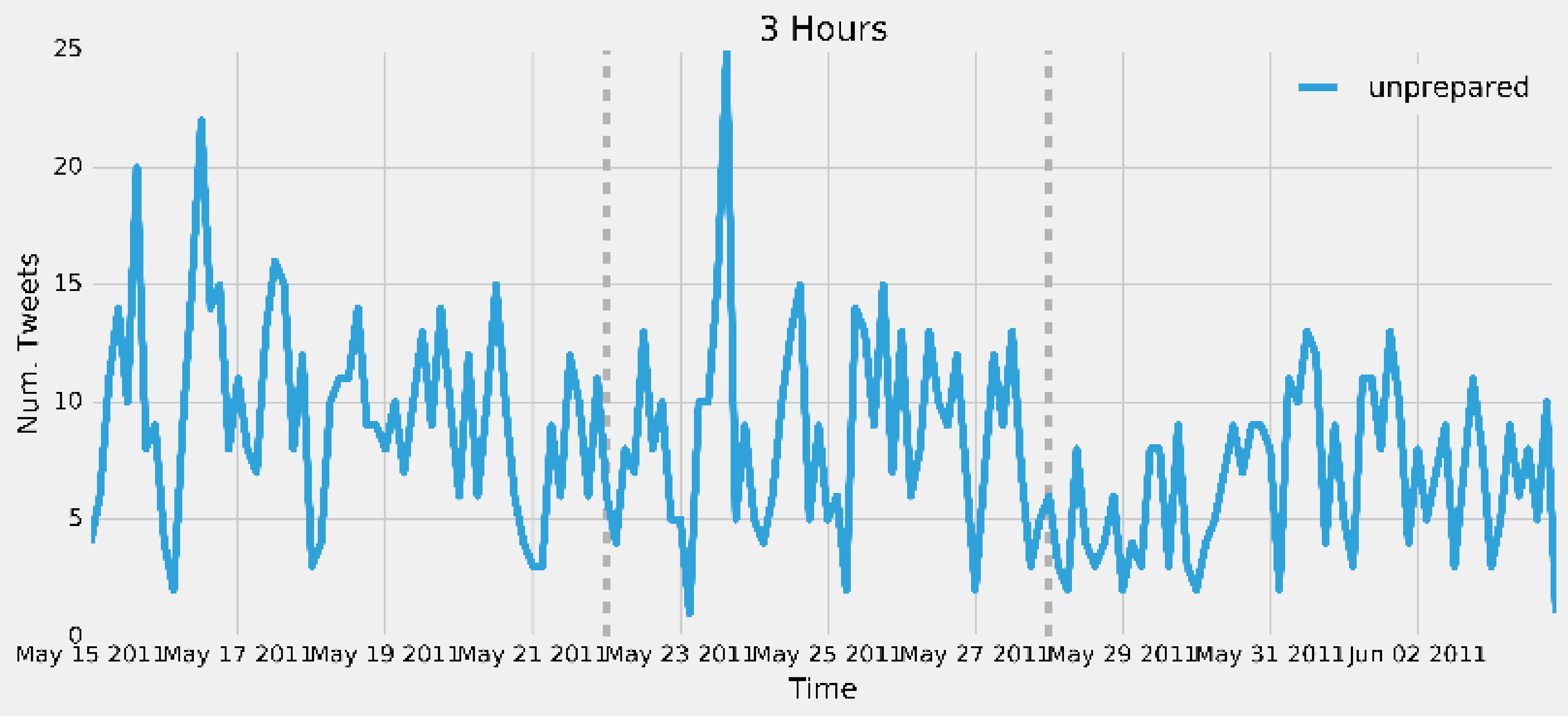
3 Hours
unprepared
Num. Tweets
0
5
10
15
20
25
May 15 2011
May 17 2011
May 19 2011
May 21 2011
May 23 2011
May 25 2011
May 27 2011
May 29 2011
May 31 2011
Jun 02 2011
Time

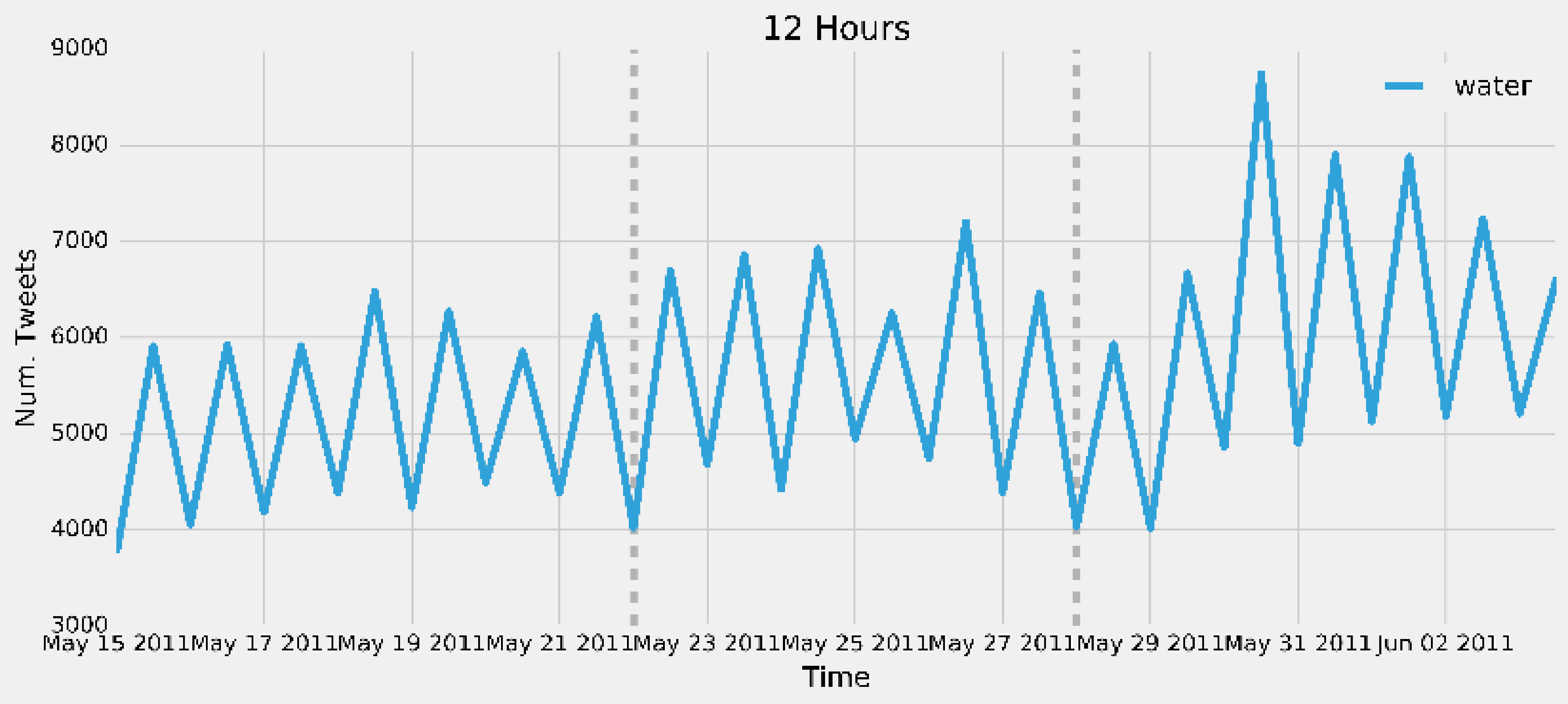
12 Hours
water
Num. Tweets
9000
8000
7000
6000
5000
4000
3000
May 15 2011
May 17 2011
May 19 2011
May 21 2011
May 23 2011
May 25 2011
May 27 2011
May 29 2011
May 31 2011
Jun 02 2011
Time

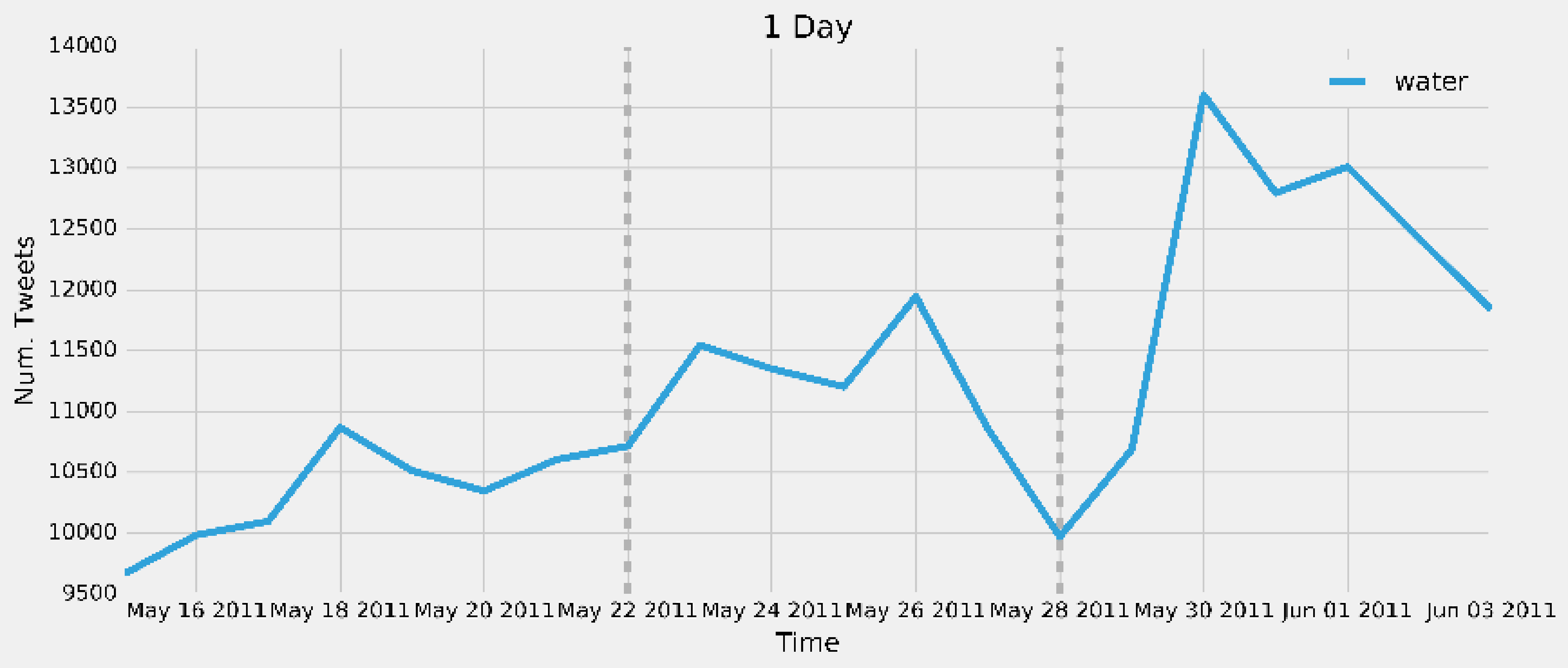
1 Day
water
Num. Tweets
9500
10000
10500
11000
11500
12000
12500
13000
13500
14000
May 16 2011
May 18 2011
May 20 2011
May 22 2011
May 24 2011
May 26 2011
May 28 2011
May 30 2011
Jun 01 2011
Jun 03 2011
Time

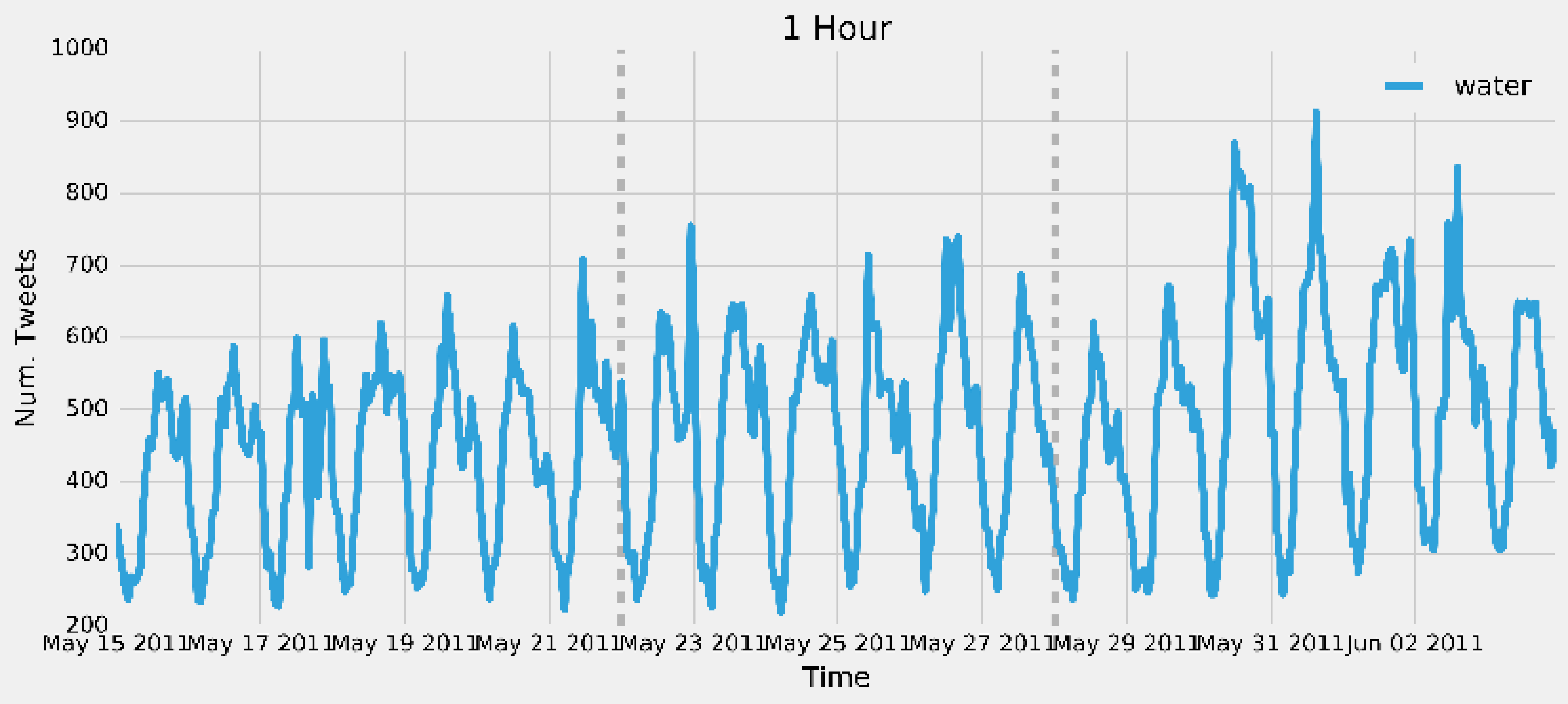

1 Hour
1000
900
800
700
600
500
400
300
200
Num. Tweets
water
May 15 2011
May 17 2011
May 19 2011
May 21 2011
May 23 2011
May 25 2011
May 27 2011
May 29 2011
May 31 2011
Jun 02 2011
Time

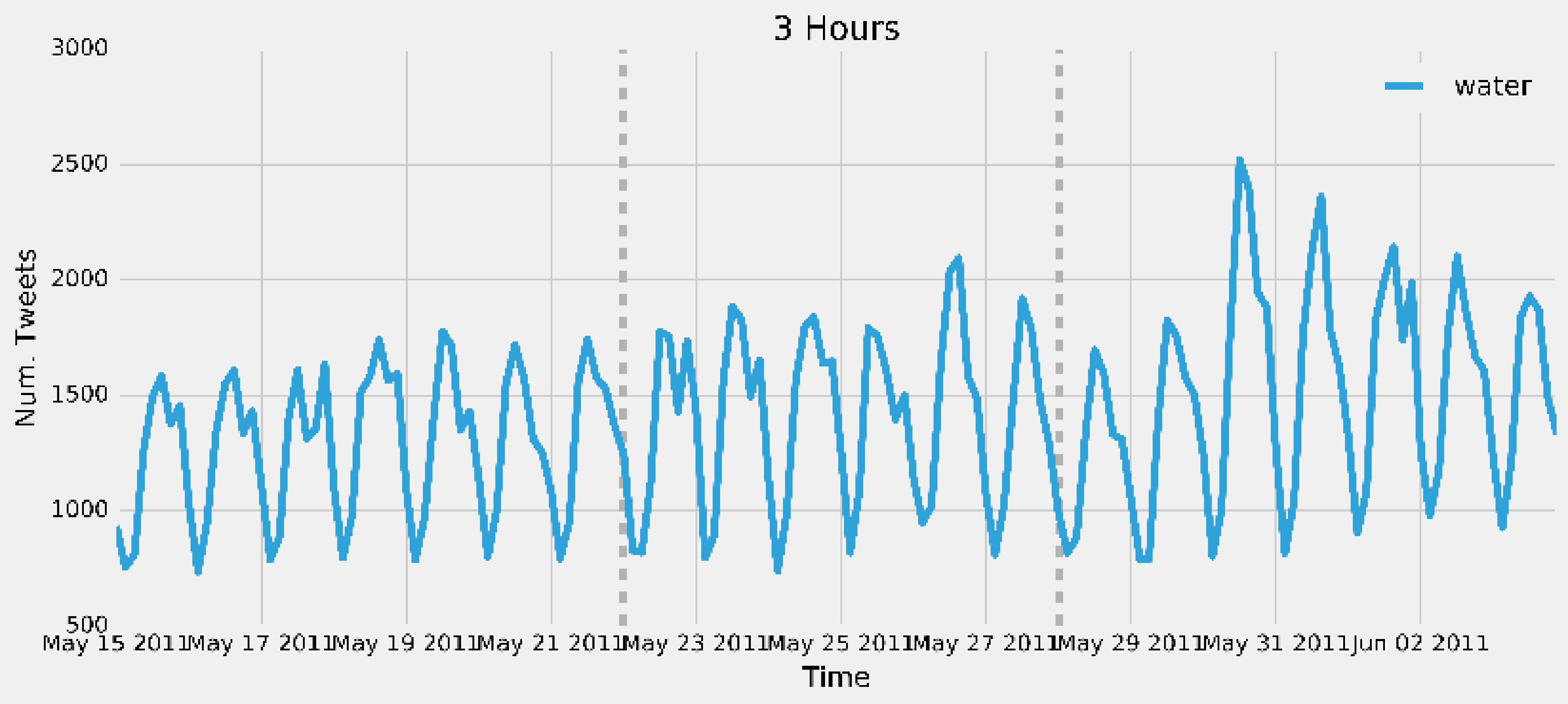

3 Hours
water
3000
2500
2000
1500
1000
500
Num. Tweets
May 15 2011
May 17 2011
May 19 2011
May 21 2011
May 23 2011
May 25 2011
May 27 2011
May 29 2011
May 31 2011
Jun 02 2011
Time

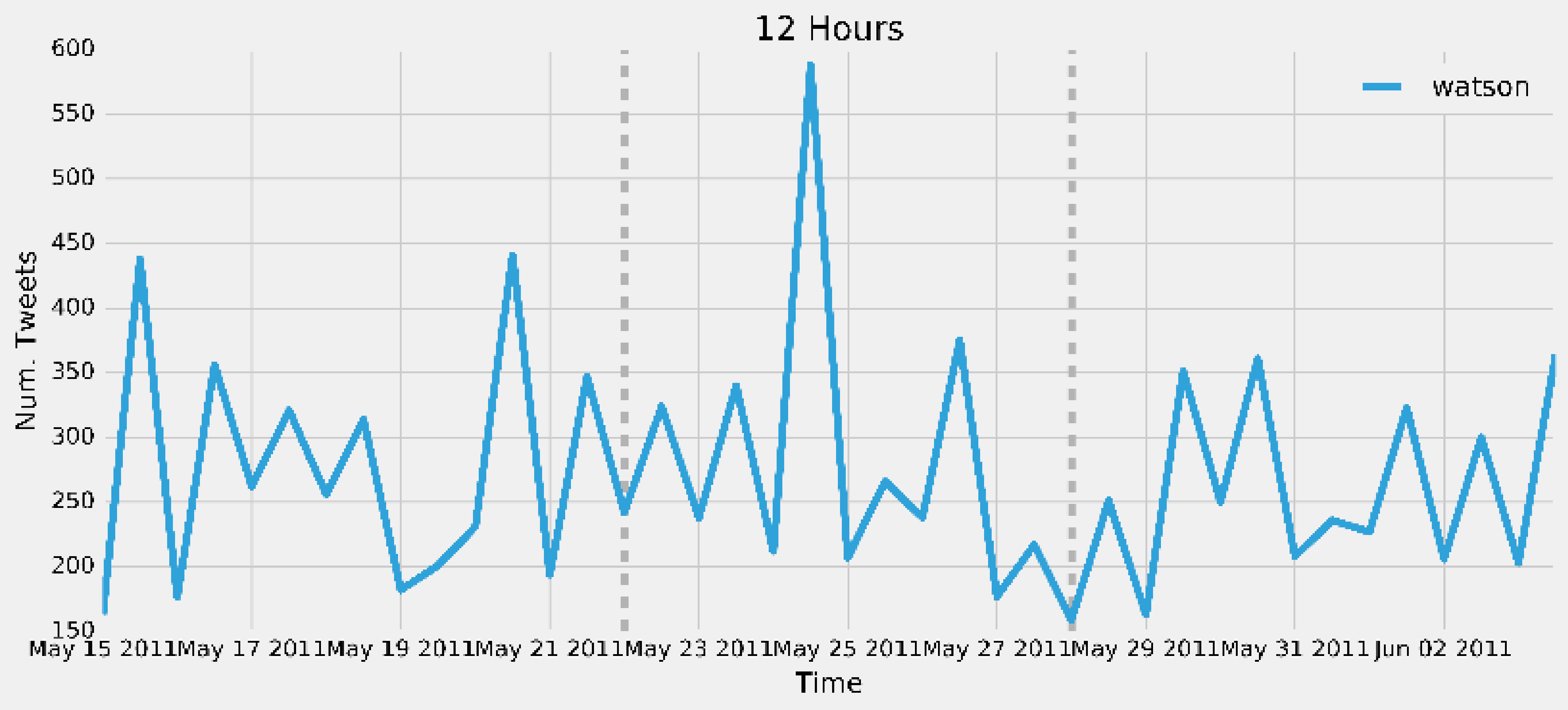
12 Hours
watson
Num. Tweets
600
550
500
450
400
350
300
250
200
150
May 15 2011
May 17 2011
May 19 2011
May 21 2011
May 23 2011
May 25 2011
May 27 2011
May 29 2011
May 31 2011
Jun 02 2011
Time

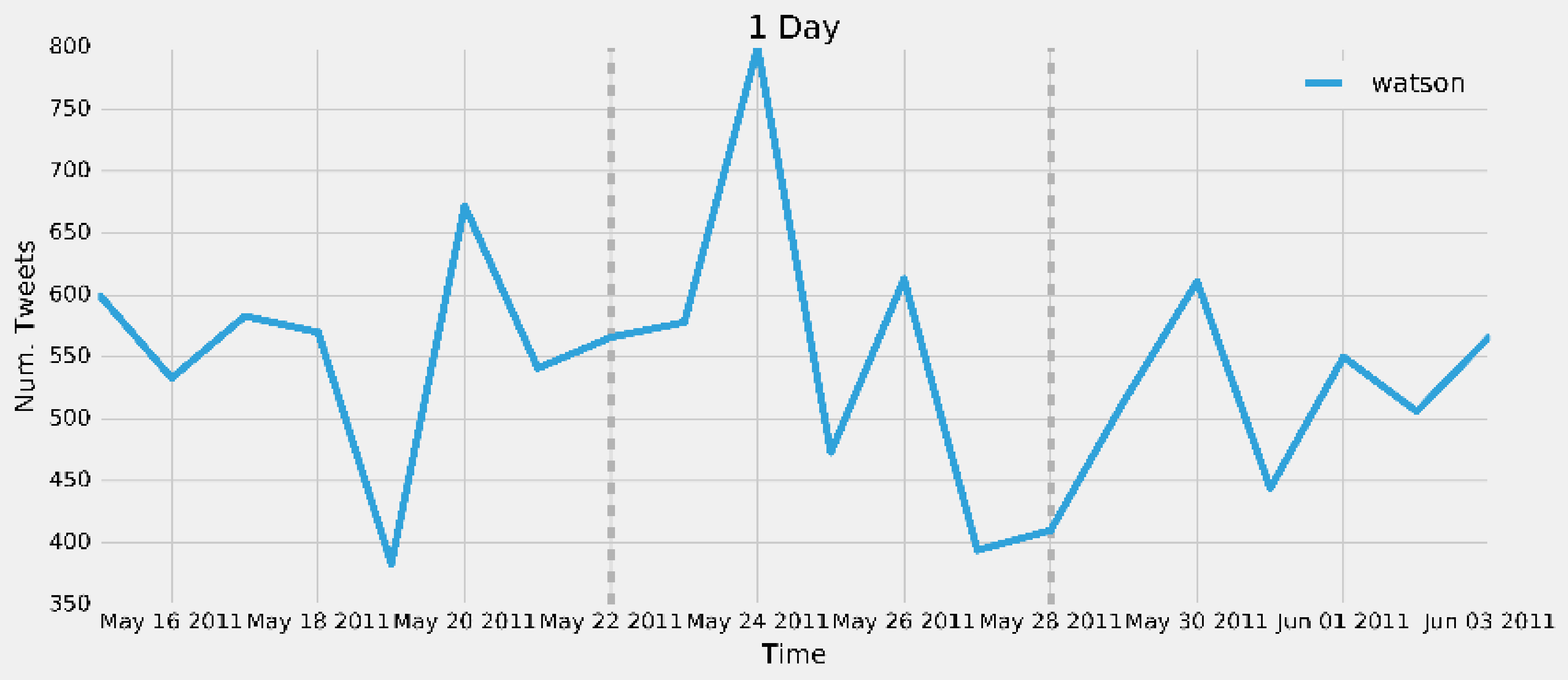
1 Day
watson
800
750
700
650
600
550
500
450
400
350
Num. Tweets
May 16 2011
May 18 2011
May 20 2011
May 22 2011
May 24 2011
May 26 2011
May 28 2011
May 30 2011
Jun 01 2011
Jun 03 2011
Time

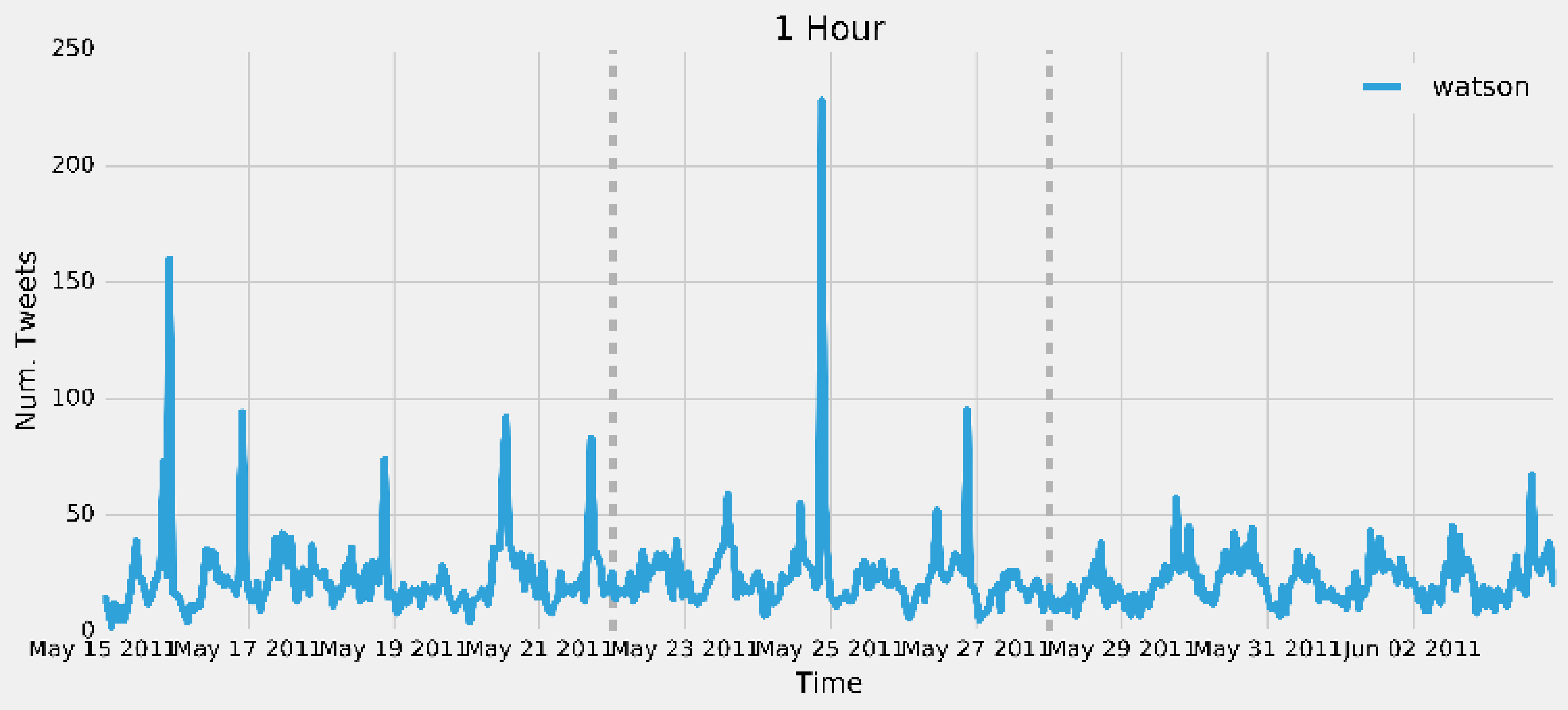
1 Hour
watson
Num. Tweets
Time
0
50
100
150
200
250
May 15 2011
May 17 2011
May 19 2011
May 21 2011
May 23 2011
May 25 2011
May 27 2011
May 29 2011
May 31 2011
Jun 02 2011

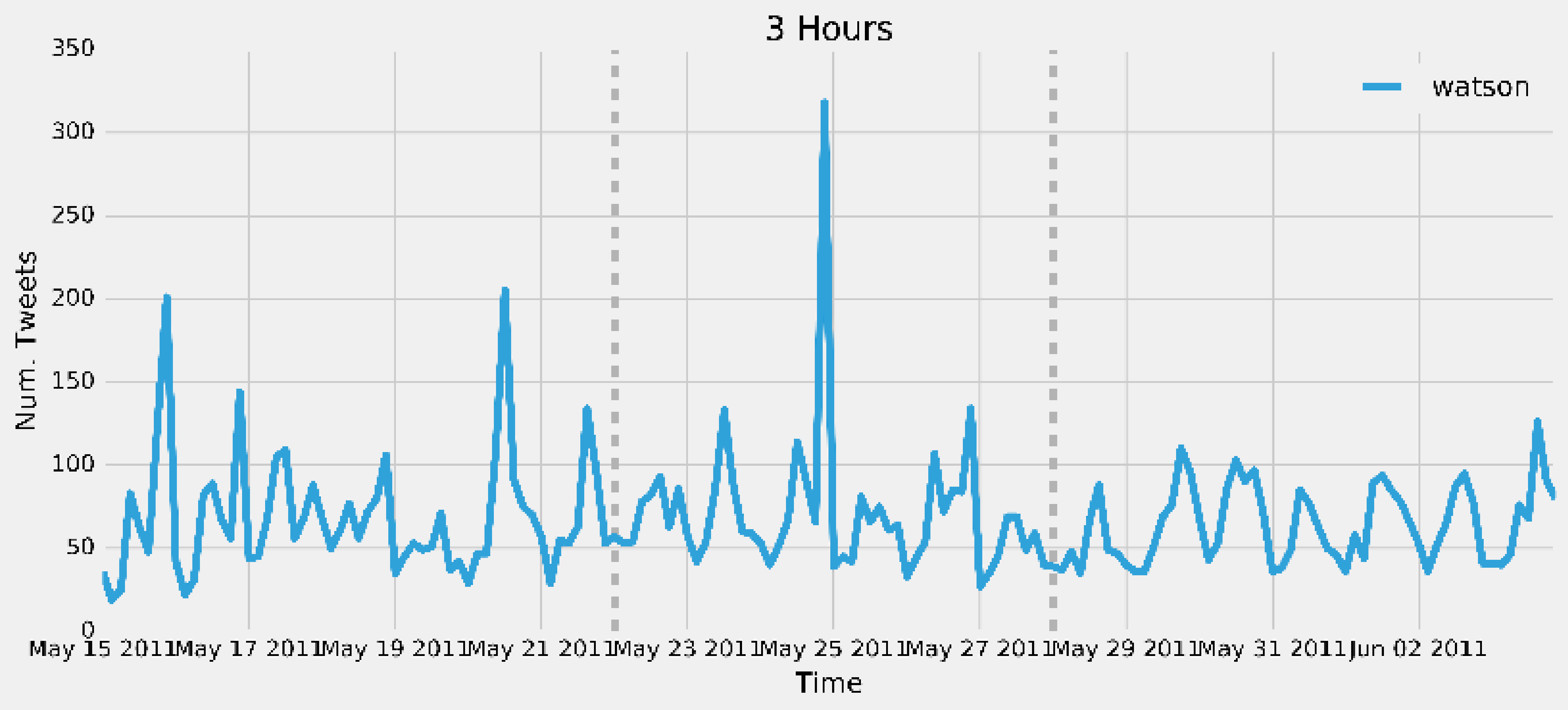
3 Hours
watson
Num. Tweets
0
50
100
150
200
250
300
350
May 15 2011
May 17 2011
May 19 2011
May 21 2011
May 23 2011
May 25 2011
May 27 2011
May 29 2011
May 31 2011
Jun 02 2011
Time

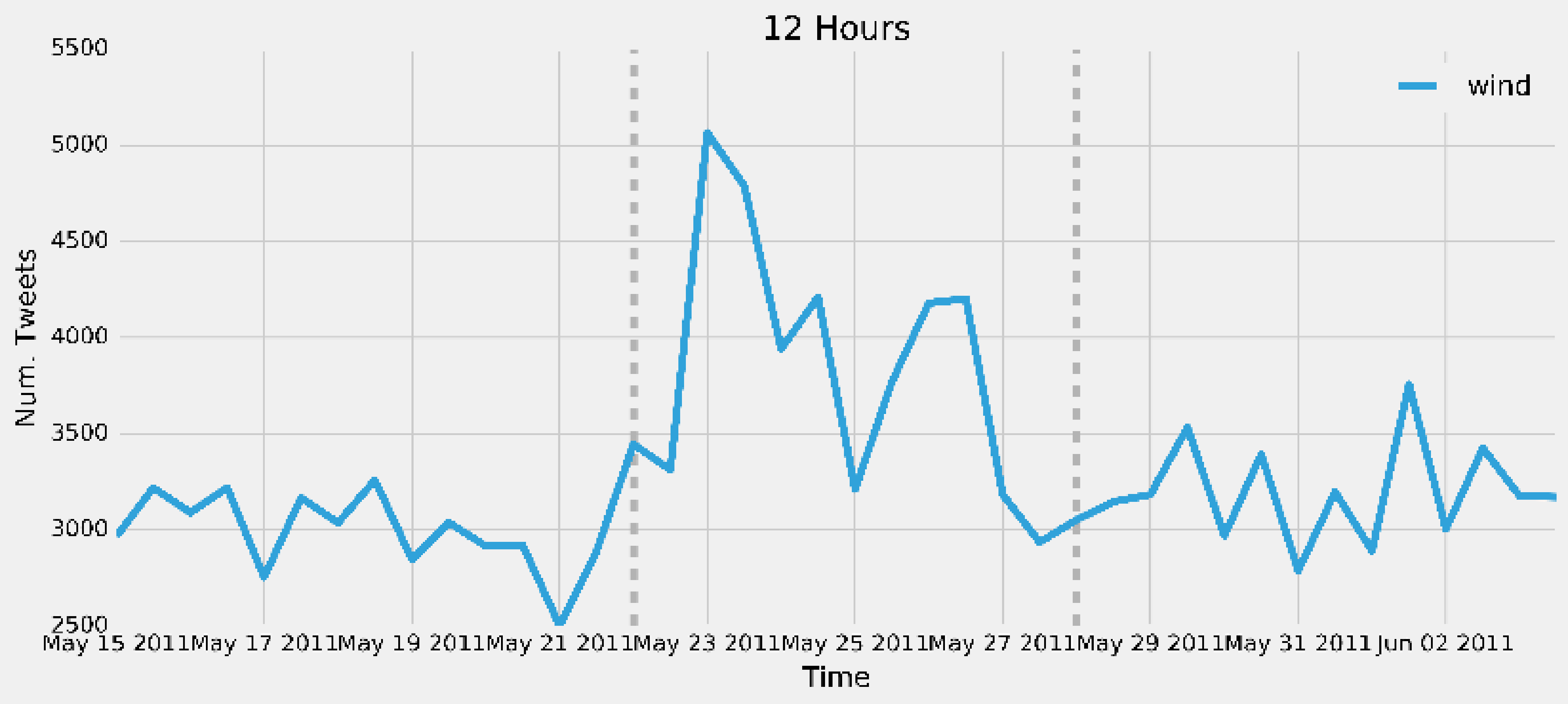
12 Hours
wind
Num. Tweets
5500
5000
4500
4000
3500
3000
2500
May 15 2011
May 17 2011
May 19 2011
May 21 2011
May 23 2011
May 25 2011
May 27 2011
May 29 2011
May 31 2011
Jun 02 2011
Time

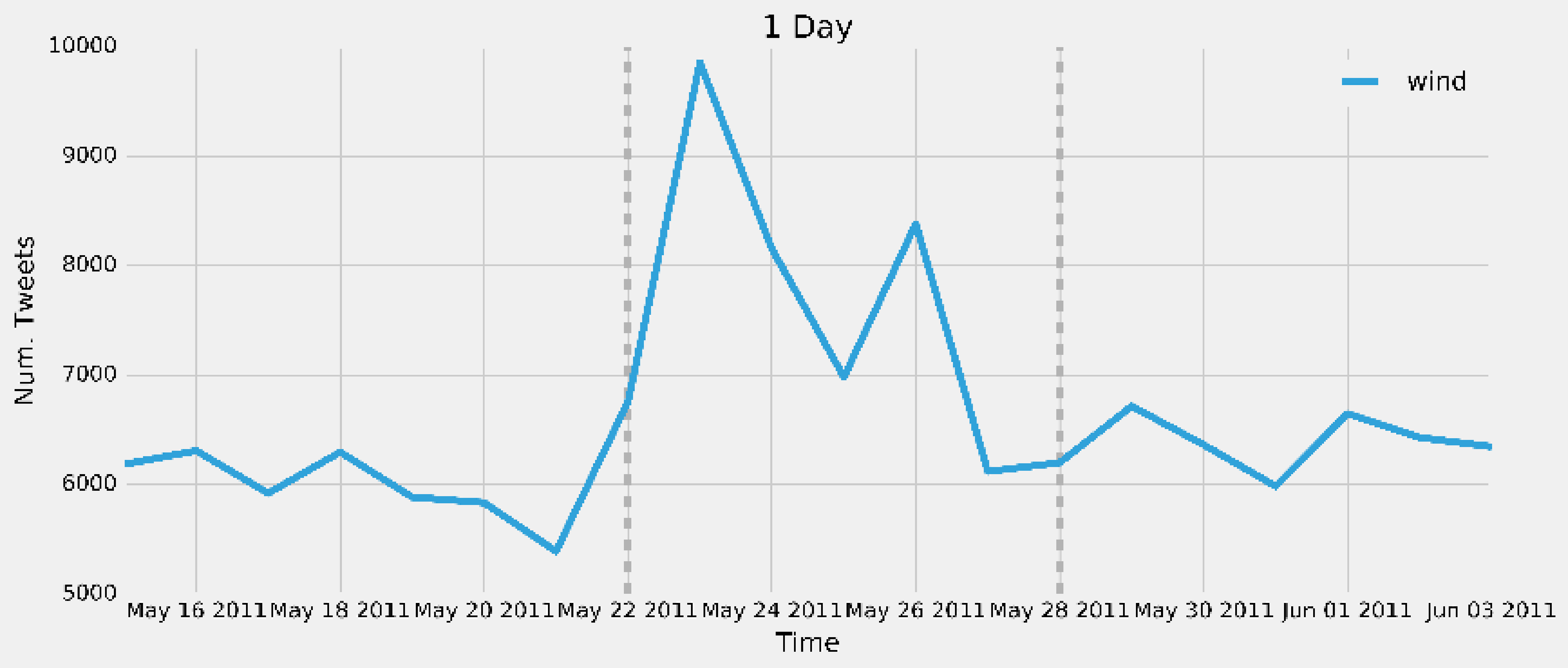
1 Day
10000
9000
8000
7000
6000
5000
Num. Tweets
wind
May 16 2011
May 18 2011
May 20 2011
May 22 2011
May 24 2011
May 26 2011
May 28 2011
May 30 2011
Jun 01 2011
Jun 03 2011
Time

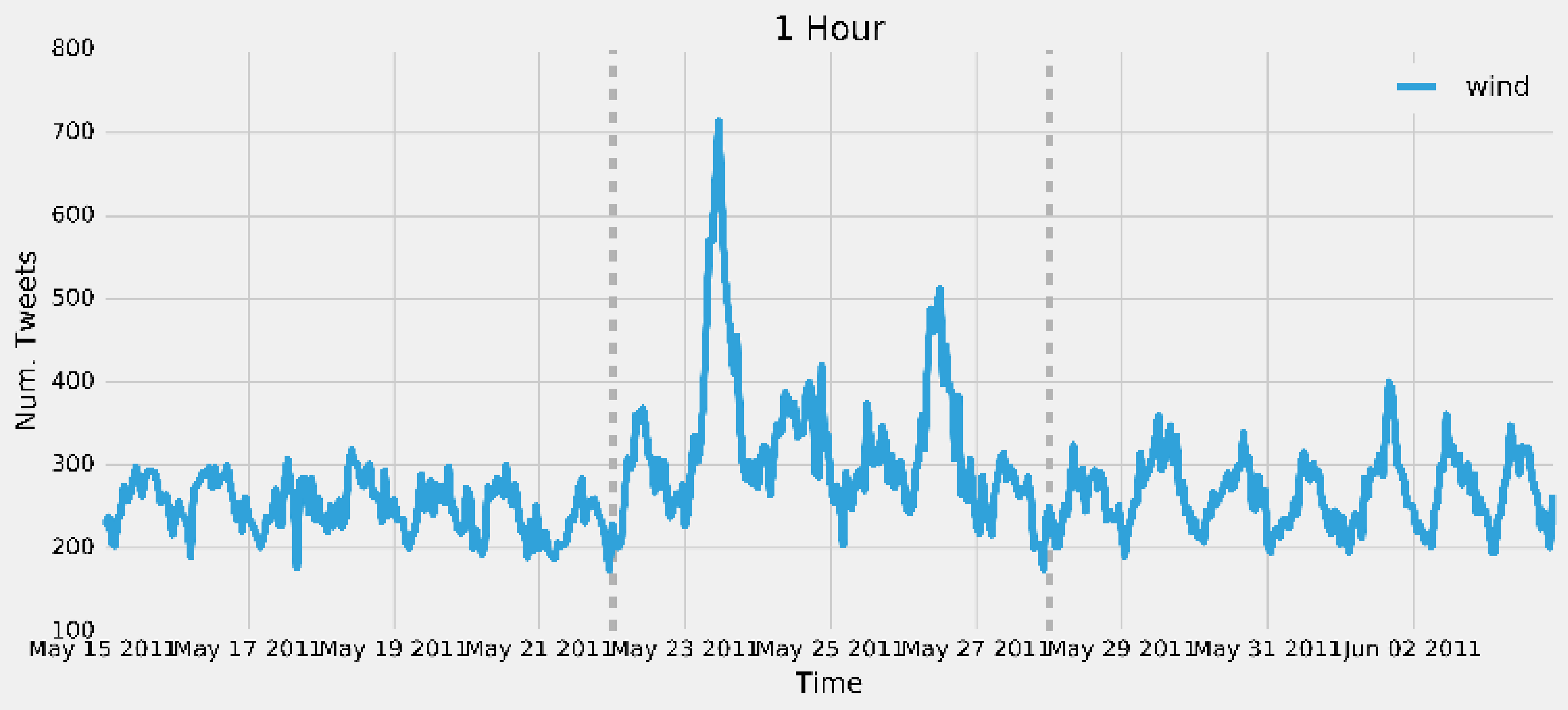

1 Hour
wind
Num. Tweets
100
200
300
400
500
600
700
800
May 15 2011
May 17 2011
May 19 2011
May 21 2011
May 23 2011
May 25 2011
May 27 2011
May 29 2011
May 31 2011
Jun 02 2011
Time

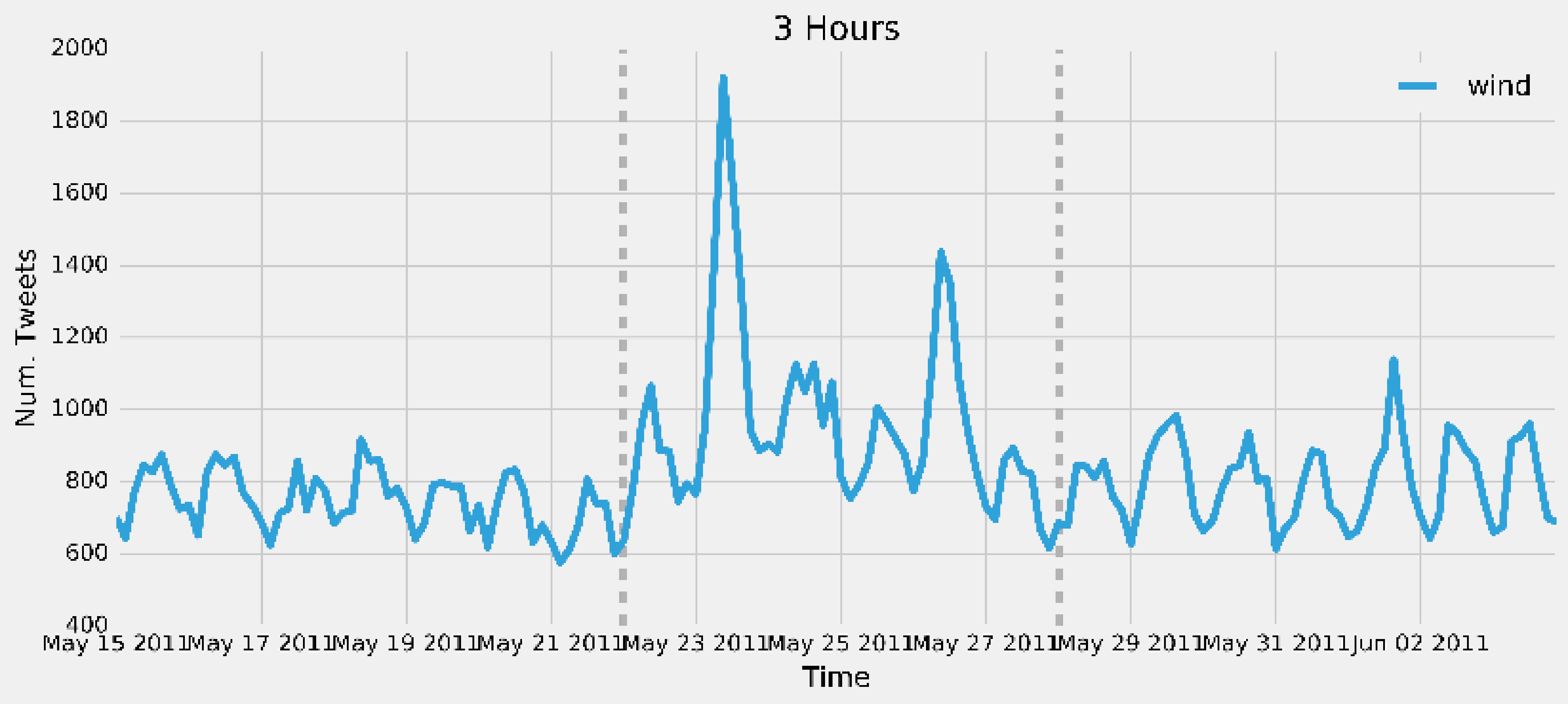

3 Hours
wind
Num. Tweets
2000
1800
1600
1400
1200
1000
800
600
400
May 15 2011
May 17 2011
May 19 2011
May 21 2011
May 23 2011
May 25 2011
May 27 2011
May 29 2011
May 31 2011
Jun 02 2011
Time
