# Supplementary material for: Social media usage patterns during natural hazards
Source: PLoS One. 2019 Feb 13;14(2):e0210484. doi: 10.1371/journal.pone.0210484 (PMC6374021; doi:10.1371/journal.pone.0210484)
Supplement: S1 Table — (DOCX) [file pone.0210484.s006.docx]

|  | | | |
| --- | --- | --- | --- |
|  | **Order of magnitude** | **Lower Bound** | **Upper Bound** |
| Sandy | 10­^0^ | 0.1365 | 0.2255 |
|  | 10^1^ | 0.1738 | 0.1876 |
|  | 10^2^ | 0.1086 | 0.1143 |
|  | 10^3^ | 0.0947 | 0.1080 |
|  | 10^4^ | 0.0628 | 0.0968 |
|  | 10^5^ | 0.0315 | 0.1205 |
| Null | 10­^0^ | 0.0337 | 0.1114 |
|  | 10^1^ | 0.0458 | 0.0543 |
|  | 10^2^ | 0.0513 | 0.0542 |
|  | 10^3^ | 0.0308 | 0.0365 |
|  | 10^4^ | 0.0169 | 0.0314 |
|  | 10^5^ | -0.0028 | 0.0317 |
